# Enantioselective Copper-Catalyzed sp^2^/sp^3^ Diborylation of 1-Chloro-1-Trifluoromethylalkenes

**DOI:** 10.1021/acscentsci.2c00339

**Published:** 2022-07-20

**Authors:** Zhenwei Fan, Mingxing Ye, Yahao Wang, Jian Qiu, Wangyang Li, Xingxing Ma, Kai Yang, Qiuling Song

**Affiliations:** † Key Laboratory of Molecule Synthesis and Function Discovery, Fujian Province University, College of Chemistry at Fuzhou University, Fuzhou, Fujian 350108, China; ‡ Institute of Next Generation Matter Transformation, College of Material Sciences Engineering, Huaqiao University, Xiamen, Fujian 361021, China; § School of Chemistry and Chemical Engineering, Henan Normal University, Xinxiang, Henan 453007, China

## Abstract

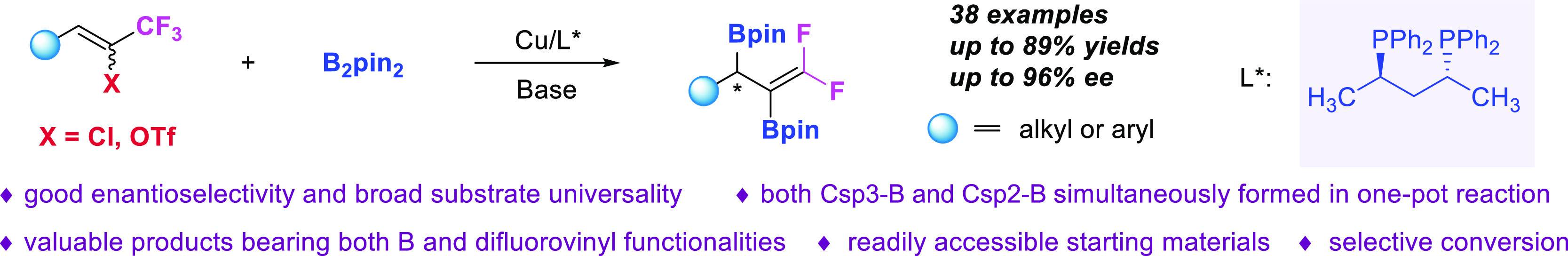

Fluorine-containing organoboron compounds have emerged as novel building blocks in chemical synthesis; among them, fluorinated sp^2^/sp^3^ diborylated compounds are particularly appealing, since they might undergo chemoselective and diversified transformations of different C–B bonds with fluorinated functionality, thus bringing versatility and complexity to the eventual products. However, expedient, synthetic strategies for the construction of such fluorinated diborylative compounds are very sparse. Herein, we disclose enantioselective Cu-catalyzed sp^2^/sp^3^ diborylations of 1-chloro-1-trifluoromethylalkenes, leading to diborylated compounds bearing a *gem*-difluoroalkenyl moiety; most intriguingly, the new formed C–B bonds include one stereoselective and optically pure Csp^3^–B bond. Further transformations on the eventual products demonstrated the values of our presented strategy.

## Abstract 2

Enantioselective Cu-catalyzed sp^2^/sp^3^ diborylation of 1-chloro-1-trifluoromethylalkenes leads to diborylated compounds with optically pure Csp^3^−B and Csp^3^−B with a *gem*-difluoroalkenyl moiety.

## Introduction

**Figure 1:**
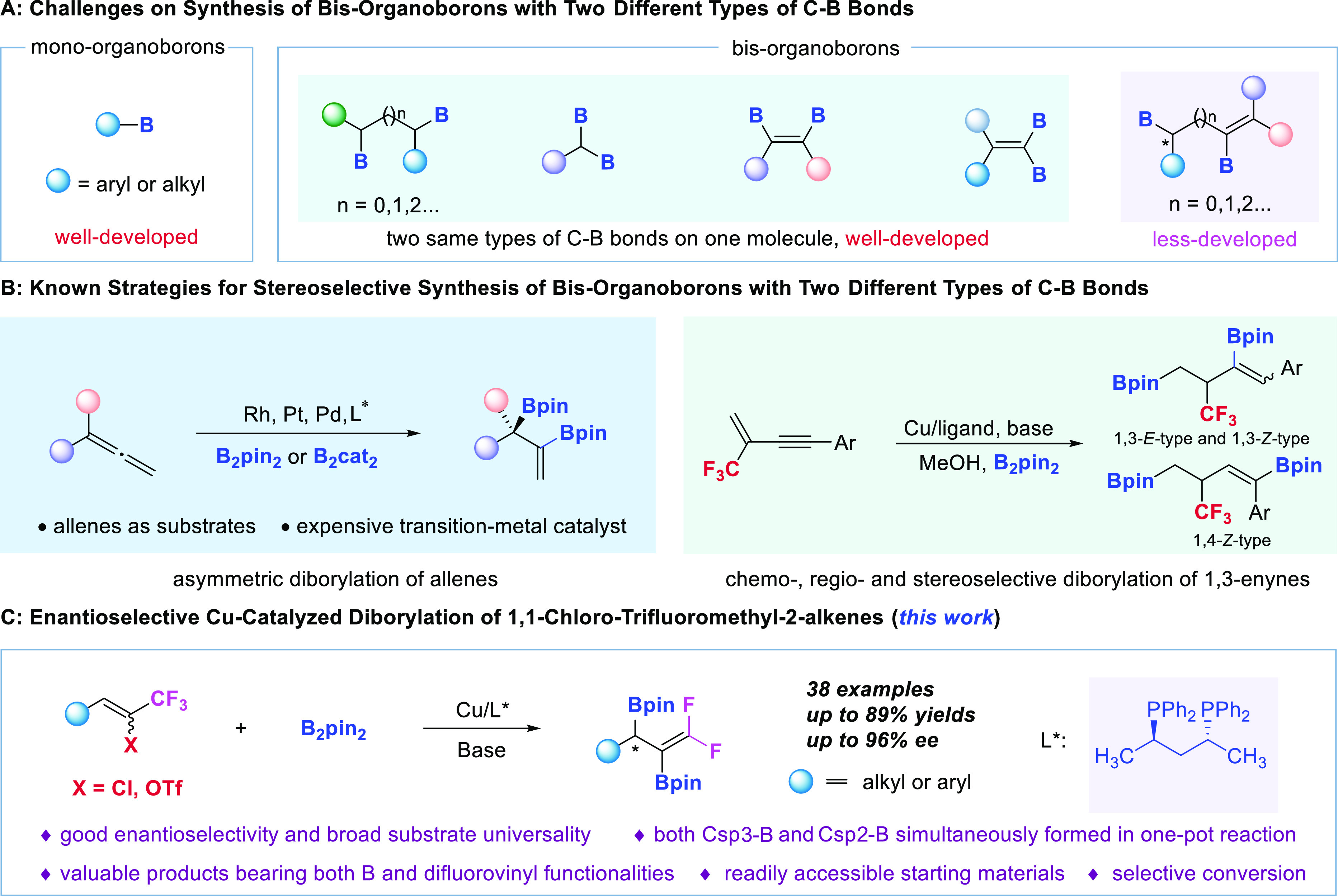
Challenges in constructing diborons with two different types of C–B bonds and our strategy.

Organoboron compounds, as one of the most important building blocks, have been widely used in organic synthesis,^1−7^ and numerous methods have been developed to construct them.^8−13^ Among different types of organoboron compounds, mono-organoborons are the most well-studied^14−23^ (Figure 1A, left). Compared with mono-organoboron compounds, diborons are especially fascinating synthons since the two boron moieties could bring more complex and diversified structural elaborations.^24−27^ There are several types of diborons that have appeared in the literature;^28,29^ however, the two C–B bonds of the same type show up on the same structure.^30−36^ In sharp contrast, molecules that contains two different types of C–B bonds, especially with one enantioenriched Csp^3^–B bond, are less-developed (Figure 1A, right).^37−42^

Fluorine-containing organoboron compounds have emerged as novel synthons in chemical synthesis due to the existence of two important functionalities—fluorine and boron on one molecule scaffold, which brings versatility and complexity to the eventual products.^43−53^ Among them, the fluorinated ones bearing multiple types of C–B bonds are particularly appealing, since they might undergo chemoselective and diversified transformations on different C–B bonds, leading to well-controlled and multifunctionalized complex targeted molecules.^54^ However, successful examples of constructions of multiborylated compounds with distinct C–B bonds are very rare, particularly for enantioselective synthesis, and most of them are focused on the transformations of allenes. For instance, Morken and co-workers^38−40^ reported a Pd-catalyzed protocol for asymmetric diborations from allenes. Recently, Tang and Ding^37^ et al. developed a similar strategy to realize asymmetric diboration once again from allenes in which a chiral tertiary boronic ester was constructed (Figure 1B, left). Of note, the two aforementioned synthetic methods could not lead to fluorine-containing multiborylated molecules. In 2020,^54^ our group disclosed novel Cu-catalyzed regio- and stereodivergent chemoselective diborylations of CF_3_-containing 1,3-enynes, rendering three different sp^2^/sp^3^ diborylated products bearing CF_3_ functionality (Figure 1B, right). As we all know, fluorinated compounds have unique and fascinating physicochemical and biological properties, and the fluorine atom is a bioisostere replacement of the hydrogen atom;^55−58^ specifically, the *gem*-difluoroalkenyl moiety has been considered as isosteric and isopolar synthons to carbonyl groups, which play a critical role in pharmaceuticals and drug designs, and is thus one of the most attractive fluorine-containing structural motifs.^59−63^ Given the importance of fluorinated sp^2^/sp^3^ diborylated compounds and the paucity of their efficient construction, as well as our long-term interests in both organofluorine^64−71^ and organoboron^72−79^ chemistry, herein, we report the first expedient Cu-catalyzed enantioselective sp^2^/sp^3^ diborylations of 1-chloro-1-trifluoromethylalkenes, in which one Csp^2^–B bond and one Csp^3^–B bond are constructed simultaneously in a one-vessel strategy along with the formation of a *gem*-difluoroalkenyl functionality (Figure 1C). The reaction features readily accessible starting materials, high enantioselectivity, and broad substrate scope with excellent functionality tolerance. Most intriguingly, two different types of C–B bonds are chemo- and stereoselectively constructed together with the formation of a *gem*-difluoroalkenyl moiety, thus leading to one new type of boron and fluorine-substituted chiral allylic boronates, which could be readily converted into various valuable products as demonstrated by further synthetic applications in this Article.

## Results

### Optimization Studies of the Racemic Process

To commence the investigation of racemic diborylation, 1-(2-chloro-3,3,3-trifluoroprop-1-en-1-yl)-4-methoxybenzene (**1**) was chosen as a model compound, together with B_2_pin_2_ as the borylation reagent, ^*t*^BuONa as the base, in the presence of copper salt, and with PCy_3_ as the ligand (in Table 1). Pleasingly, the combination afforded the desired product **2a** in a 37% yield when catalytic CuCl_2_ was evaluated (Table 1, entry 1). Different copper salts, such as CuF_2_, CuBr_2_, CuSO_4_, CuOTf, CuTc, CuCl, and Cu(OTf)_2_, were explored (Table 1, entries 2–8); among these salts, Cu(OTf)_2_ provided the best result with 87% yield. Subsequently, various reaction temperatures were investigated, and it turned out that 60 °C was the temperature that led to the optimal outcome (Table 1, entries 9–12). Different amounts of base caused insignificant reductions in the yields of this reaction (Table 1, entries 13–15).

**Table 1:**
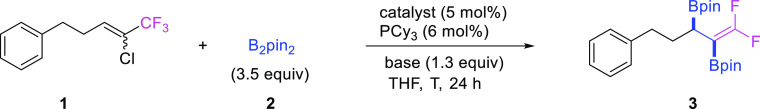
Optimization Studies of the Racemic Process^a^

entry	catalyst	base	*T* (°C)	yield (%)
1	CuCl_2_	^*t*^BuONa	60	37
2	CuF_2_	CuCl_2_	60	52
3	CuBr_2_	^*t*^BuONa	60	46
4	CuSO_4_	^*t*^BuONa	60	45
5	CuOTf	^*t*^BuONa	60	63
6	CuTc	^*t*^BuONa	60	26
7	CuCl	^*t*^BuONa	60	54
8	Cu(OTf)_2_	^*t*^BuONa	60	87 (81^b^)
9	Cu(OTf)_2_	^*t*^BuONa	40	49
10	Cu(OTf)_2_	^*t*^BuONa	50	68
11	Cu(OTf)_2_	^*t*^BuONa	70	59
12	Cu(OTf)_2_	^*t*^BuONa	80	63
13^c^	Cu(OTf)_2_	^*t*^BuONa	60	75
14^d^	Cu(OTf)_2_	^*t*^BuONa	60	82
15^e^	Cu(OTf)_2_	^*t*^BuONa	60	78

a Conditions: the reaction was carried out with **1** (0.5 mmol), B_2_pin_2_ (3.5 equiv), [Cu] (5 mol %), PCy_3_ (6 mol %), and ^*t*^BuONa (1.5 equiv) in THF (2.5 mL) at 60 °C for 24 h.

b Isolated yield.

c ^*t*^BuONa 1.0 equiv.

d ^*t*^BuONa 1.8 equiv.

e ^*t*^BuONa 2.0 equiv. The yield was determined by GC using *n*-dodecane as an internal standard.

### Substrate Scope for the Racemic Process

**Figure 2:**
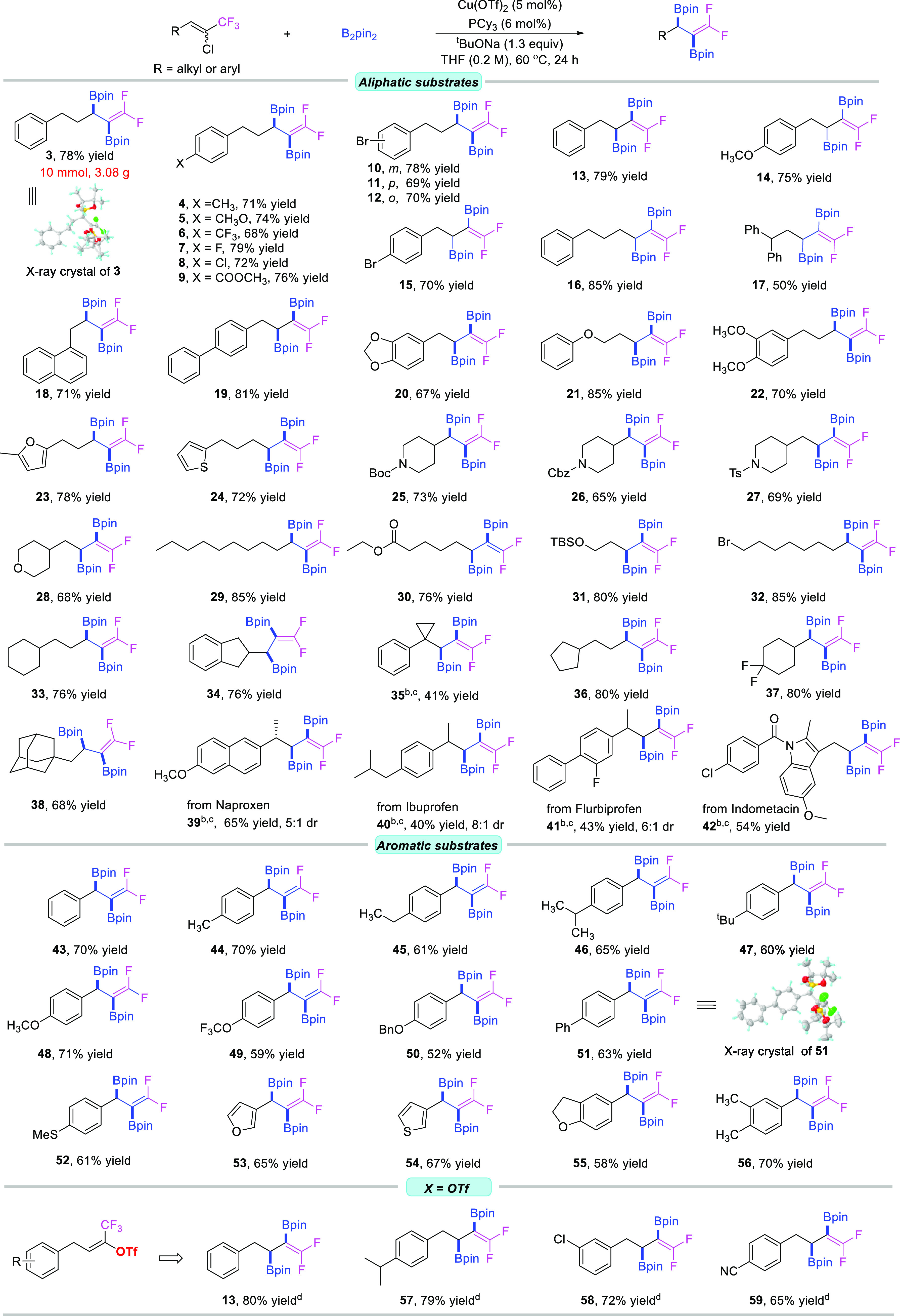
Scope of the racemic reaction. (a) Reaction conditions: Cu(OTf)_2_ (5 mol %), PCy_3_ (6 mol %), ^*t*^BuONa (1.3 equiv), and B_2_pin_2_ (4.0 equiv) were mixed in 2.5 mL of THF, and **1** (0.5 mmol) was added subsequently at room temperature; then, the resulting mixture was stirred at 60 °C for 24 h. (b) 80 °C. (c) 36 h. (d) 16 h.

After identification of the optimal reaction conditions (Table 1, entry 8), the substrate generality of this Cu-catalyzed diborylation of 1-chloro-1-trifluoromethylalkenes was investigated (Figure 2). Various unactivated aliphatic 1-chloro-1-trifluoromethylalkenes were examined and gave satisfactory to excellent reaction outcomes. A variety of substituents such as Me, MeO, CF_3_, F, Cl, COOMe, as well as Br on the benzene ring were very compatible with the transformation to afford the desired products **4**–**12** in good yields. Substitution position did not affect the outcomes at all, since bromo groups at the *meta* or *ortho* positions of the benzene ring also smoothly afforded the target products **10** and **12** in 78% and 70% yields, respectively; no loss of efficiency was ever observed compared with the *para* position (**11**, 69%). Aromatic rings containing aliphatic substituted alkenes with different chain lengths and different substitutions were all good candidates and transformed into the corresponding target products under our standard conditions in good to excellent yields (**13**–**22**). Heteroatomic cyclic rings, such as furan, thiophene, tetrahydropyran, and various *N*-protected piperidines, were all tolerated in this transformation, and the corresponding desired products were obtained in decent yields (**23**–**28**). Long, linear aliphatic chains with various functionalities (ether, ester, sily ether, and Br) also showed good capabilities to lead to the target molecules **29**–**32** in good to excellent yields. Cyclic rings, such as cyclohexyl (**33** and **37**), cyclopentyl (**34** and **36**), cyclopropyl (**35**), and adamantyl (**38**), also demonstrated good compatibility. In terms of the phenylcyclopropyl ring, initially, no desired product **35** was obtained due to the steric hindrance that comes from the cyclopropane ring, but after increasing the reaction temperature and prolonging the reaction time, the target product **35** was afforded in decent yield. A series of substrates that were derived from bioactive or drug molecules like naproxen, ibuprofen, flurbiprofen, and indomethacin were all suited under our standard conditions to render the corresponding products in moderate to good yields (**39**–**42**).

To our delight, this chemistry was not unique to aliphatic substituted 1-chloro-1-trifluoromethylalkenes; the (hetero)aromatic substituted substrates were also compatible with this transformation to generate the corresponding desired diborylation products. Various substituents such as alkyl groups (Me, Et, ^i^Pr, and ^*t*^Bu), ethers and thioethers (CH_3_O, CF_3_O, BnO, and CH_3_S), as well as a phenyl group on the benzene ring were all tolerable under our standard conditions, and the corresponding products (**43**–**52**) were obtained in a moderate to good yields. Heteroaromatic substrates such as furan, thiophene, and 2,3-dihydrobenzofuran demonstrated good reactivities as well, and the corresponding target compounds (**53**–**55**) were isolated in good yields. A disubstituted aromatic substrate was also a good candidate to lead to the appealing product **56** in 70% yield.

Inspired by the aforementioned outcomes, we envisage that substrates bearing other types of (pseudo)halogen instead of the Cl group should be compatible with our transformation as well to render the same products. Therefore, a similar substrate (*Z*)-1,1,1-trifluoro-4-phenylbut-2-en-2-yl trifluoromethanesulfonate was first prepared (Figure 2, bottom); gratifyingly, when the new substrate was subjected to our standard conditions, the reaction indeed occurred, and the corresponding desired product **13** was obtained in 80% yield with a shorter reaction time. Just like its chloro congener, the new substrates also demonstrated good functional group tolerance; both electron-donating and electron-withdrawing groups on the benzene rings were compatible with this standard condition, and the target products **57**–**59** were obtained in excellent yields.

Encouraged by the successful racemic synthesis of diborylative products, we were prompted to try an asymmetric version of this transformation. A set of structurally relevant chiral phosphine ligands (Table 2) were extensively evaluated. Ferrocene-type ligands (L1–L3) were first chosen and showed relatively good results, delivering 42–76% isolated yields of diborylation products **60** with fair to good er values (Table 2, entries 1–3). Following a systematic examination of commercial chiral bidentate ligands (Table 2, entries 4–7), we were delighted to find that ligand L5 could provide enantioenriched **60** in 90:10 er (Table 2, entry 5); further examination indicated that ligand L8 could enantioselectively provide **60** in 95:5 er while with 67% yield (Table 2, entry 8). Subsequently, we replaced THF with MTBE as the solvent, remarkably, the yield of the desired product **60** was dramatically increased to 81% yield along with 96.5:3.5 er (Table 2, entry 9). Of note, after everything was added to the reaction mixture, instead of controlling the reaction temperature at 15 °C, we allowed the mixture to warm to ambient temperature; optically active **60** was obtained in 83% isolated yield with 93:7 er (Table 2, entry 10).

**Table 2:**
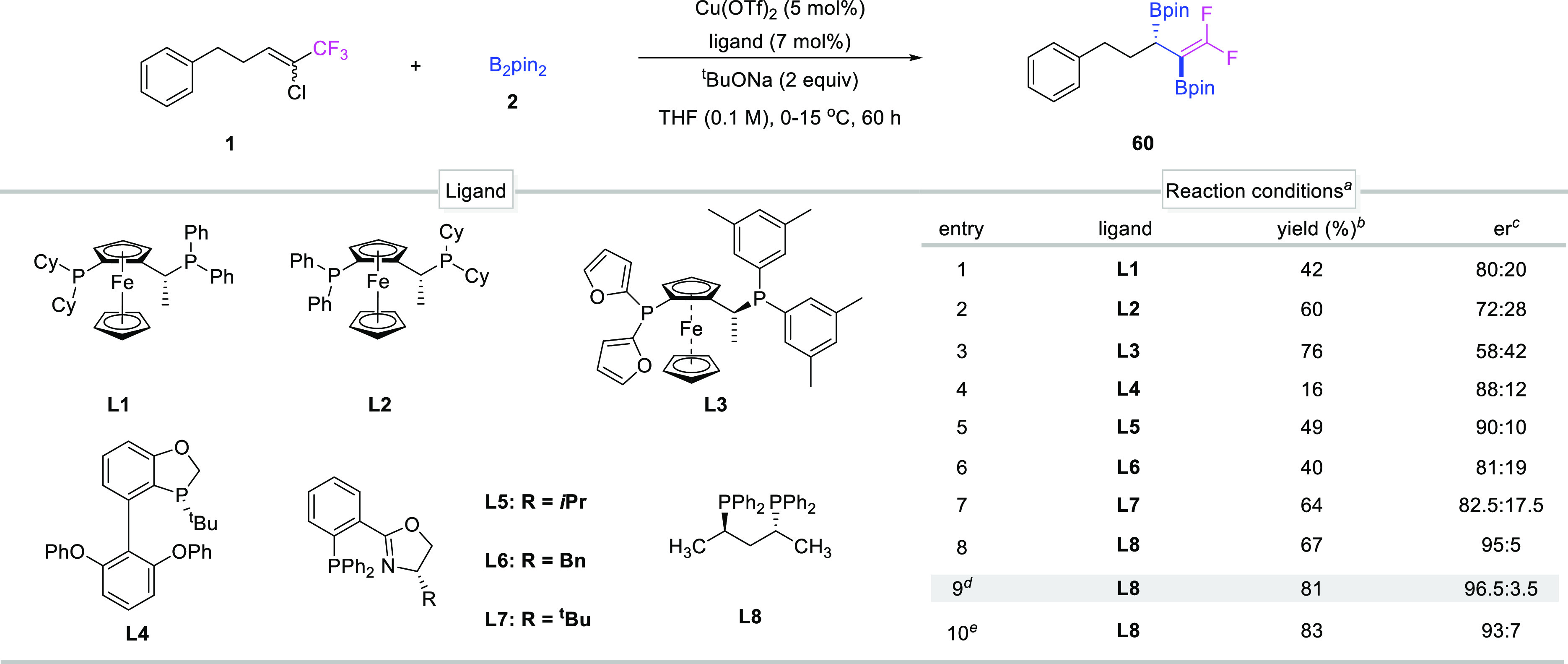
Discovery and Evaluation of Cu-Catalyzed Asymmetric Diborylation^a^

a Cu(OTf)_2_ (5 mol %), ligand (7 mol %), ^*t*^BuONa (1.3 equiv), and B_2_pin_2_ (4.0 equiv) were first mixed in 2.5 mL of THF, and **1** (0.5 mmol) was added subsequently at 0 °C; then, the temperature was slowly raised to 15 °C, and the mixture was stirred for 60 h.

b Isolated yield.

c The enantioselectivity was determined by chiral HPLC.

d MTBE (methyl *tert*-butyl ether) (3 mL) instead of THF (3 mL).

e 0 °C and then slowly raised to 25 °C and stirred for 60 h.

**Figure 3:**
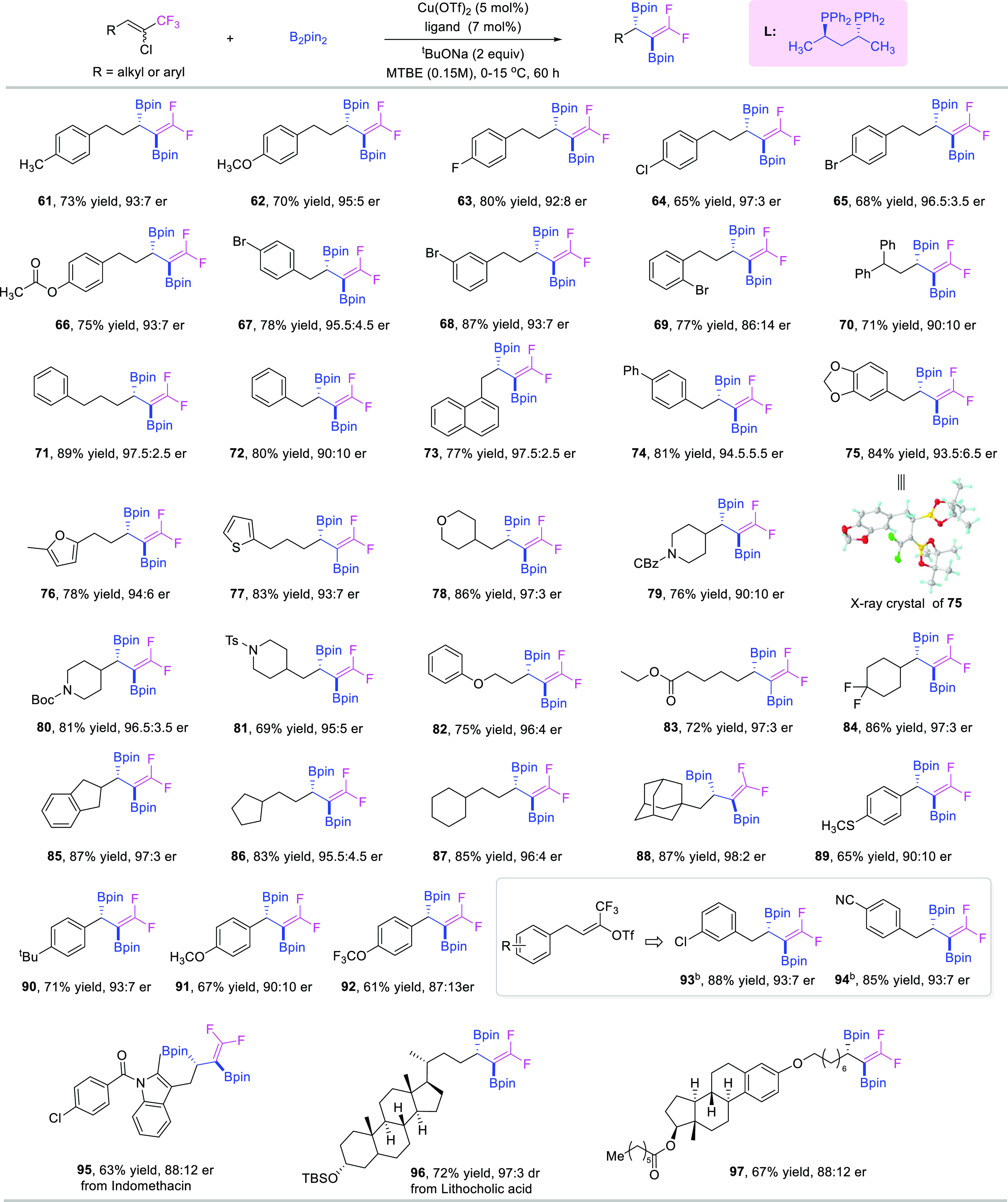
Scope of the asymmetric reaction. (a) Reaction conditions: Cu(OTf)_2_ (5 mol %), ligand (7 mol %), ^*t*^BuONa (2 equiv), and B_2_pin_2_ (4.0 equiv) were mixed in MTBE (3 mL), and **1** (0.3 mmol) was added subsequently at 0 °C; the resulting mixture was allowed to slowly rise to 15 °C and was stirred for 60 h. (b) 0 °C.

Having arrived at the optimized conditions for the enantiocontrolled reaction, we next explored the scope of 1-chloro-1-trifluoromethylalkenes (Figure 3). As with the racemic reactions, a wide range of aliphatic substituted alkenes could be successfully converted to enantioenriched forms of target products. A variety of substituents such as Me, MeO, F, Cl, Br, and ester on the *para* position of the benzene ring were all very compatible under the standard conditions, delivering the respective products in good to excellent yields with high enantioselectivities (**61**–**67**). When the bromo group was on the *meta* or *ortho* positions of the benzene ring, the corresponding products could be unperturbed and obtained in good yields albeit with a slight reduction in enantioselectivity (**68** and **69**). Aryl-substituted alkyl chains with different lengths were all suitable substrates and delivered the desired products (**70**–**75**) in excellent yields with high enantiocontrol. The absolute configuration of the major enantiomer was determined as **S** by the single crystal structure of **75**. Heteroatomic cyclic rings, including furan, thiophene, tetrahydropyran, and *N*-protected piperidines, were also tolerated very well under our chiral standard conditions, and the target molecules (**76**–**81**) were rendered in excellent yields with high enantioselectivity. In addition, ether and ester were also compatible in our transformations to lead to the corresponding enantioenriched products (**82** and **83**). Cyclic rings, such as cyclohexyl (**84** and **87**), cyclopentyl (**85** and **86**), and adamantane (**88**), were all smoothly transformed into the desired products in excellent yields and beautiful er values. Most remarkably, in addition to aliphatic substituted 1-chloro-1-trifluoromethylalkenes, we found that their styrene counterparts also demonstrated good reactivity under the chiral standard conditions with good functional group tolerance to afford the corresponding enantioenriched products with moderate yields and good er values (**89**–**92**). Furthermore, when the chloro groups on the starting alkenes were replaced with a triflate one, the desired products **93** and **94** were afforded in high yields and good er values. To further demonstrate the good functional group tolerance and broad substrate scope of our transformation, bioactive and complex molecule-derived substrates were evaluated in our chiral standard conditions as well; to our delight, those substrates were very compatible, and the desired enantioenriched diborylative target products were consistently generated in good yields with high stereoselectivities (**95**–**97**).

### Synthetic Applications

**Figure 4:**
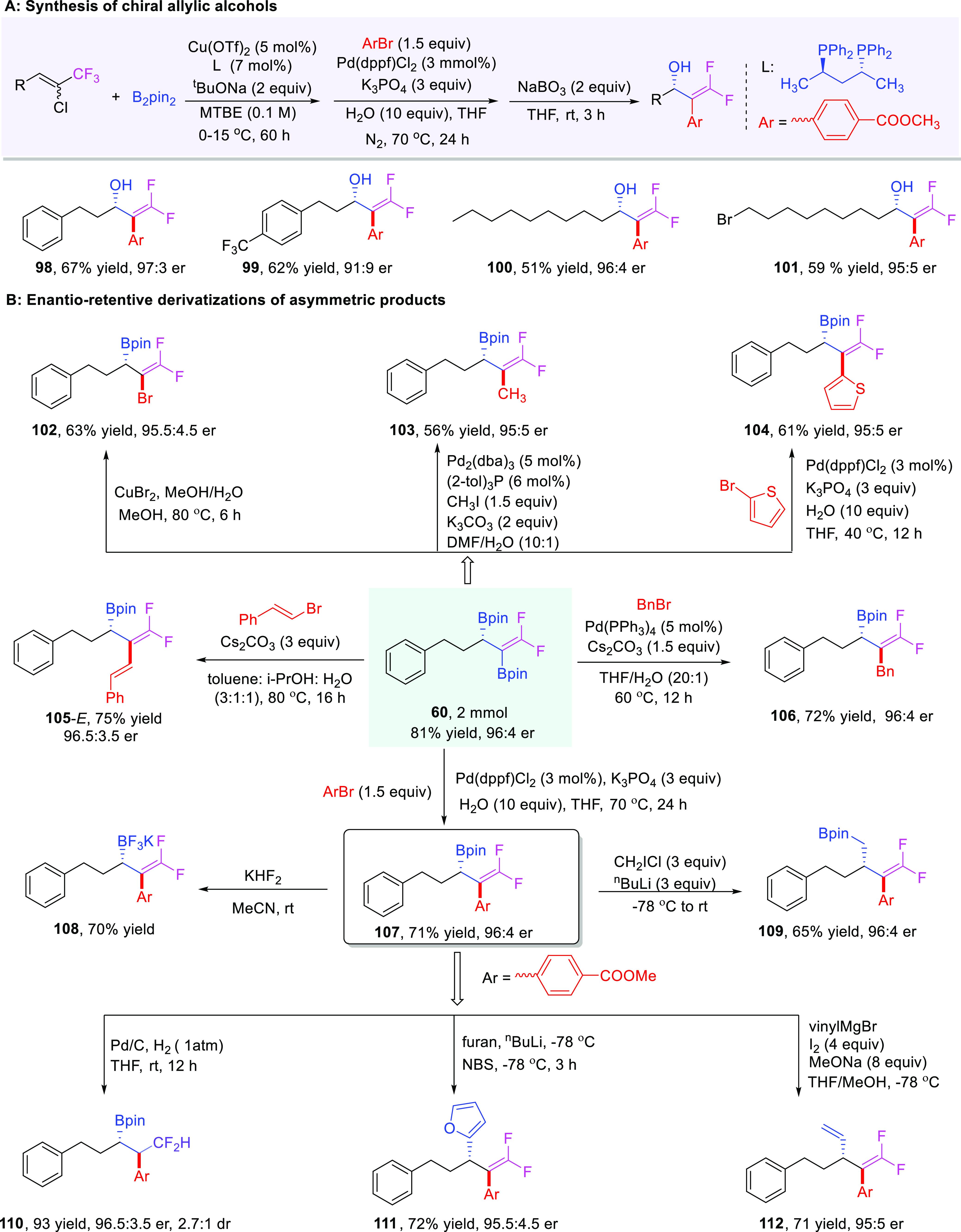
Synthetic applications and transformations of the diborylative products.

To exemplify the synthetic applications of our chiral diborylative products in asymmetric chemical synthesis, various stereospecific transformations on the optically active diborylative products were performed (Figure 4). First of all, chiral allylic alcohols **98**–**101**^43^ were readily accessible through three steps in a single-vessel reaction from the initial 1-chloro-1-trifluoromethylalkenes with moderate to good yields and high enantioselectivities (Figure 4A), which provides an alternative route to enable the very important allylic alcohols in a simple yet powerful way. Furthermore, the enantioenriched diborylative products are highly versatile chiral synthetic intermediates since two different types of C–B bonds exist on such molecules; therefore, they can be readily elaborated into a variety of valuable optically active compounds, by selective derivatization either on Csp^2^–B bonds or on Csp^3^–B bonds. For instance, selective functionalizations of Csp^2^–B bonds on chiral diboron **60**, such as bromination, methylation, thiophenolation, vinylation, benzylation, as well as arylation, could lead to the corresponding products **102**–**107**^80−83^ in good yields with excellent enantioretention. Starting from compound **107**, versatile transformations on Csp^3^–B bonds were successfully performed; for example, potassium trifluoroborate **108**(84) could be easily obtained with KHF_2_ from **107**, and **109**(43) could be obtained by one-carbon homologation of allylic boronates with ClCH_2_Li. The chiral boron **107** could undergo a complete hydrogenation with Pd/C catalyst to render a chiral boronate **110**(43) bearing a difluoromethyl group with a fair dr value but good er value; compound **111**(85) was formed by cross-coupling from **107** and furan with *n*-BuLi and NBS; in the presence of vinylMgBr, I_2_, and NaOMe, a sequential reaction occurred to lead to the vinylated product **112**(86) (Figure 4B).

### Mechanism Studies

**Figure 5:**
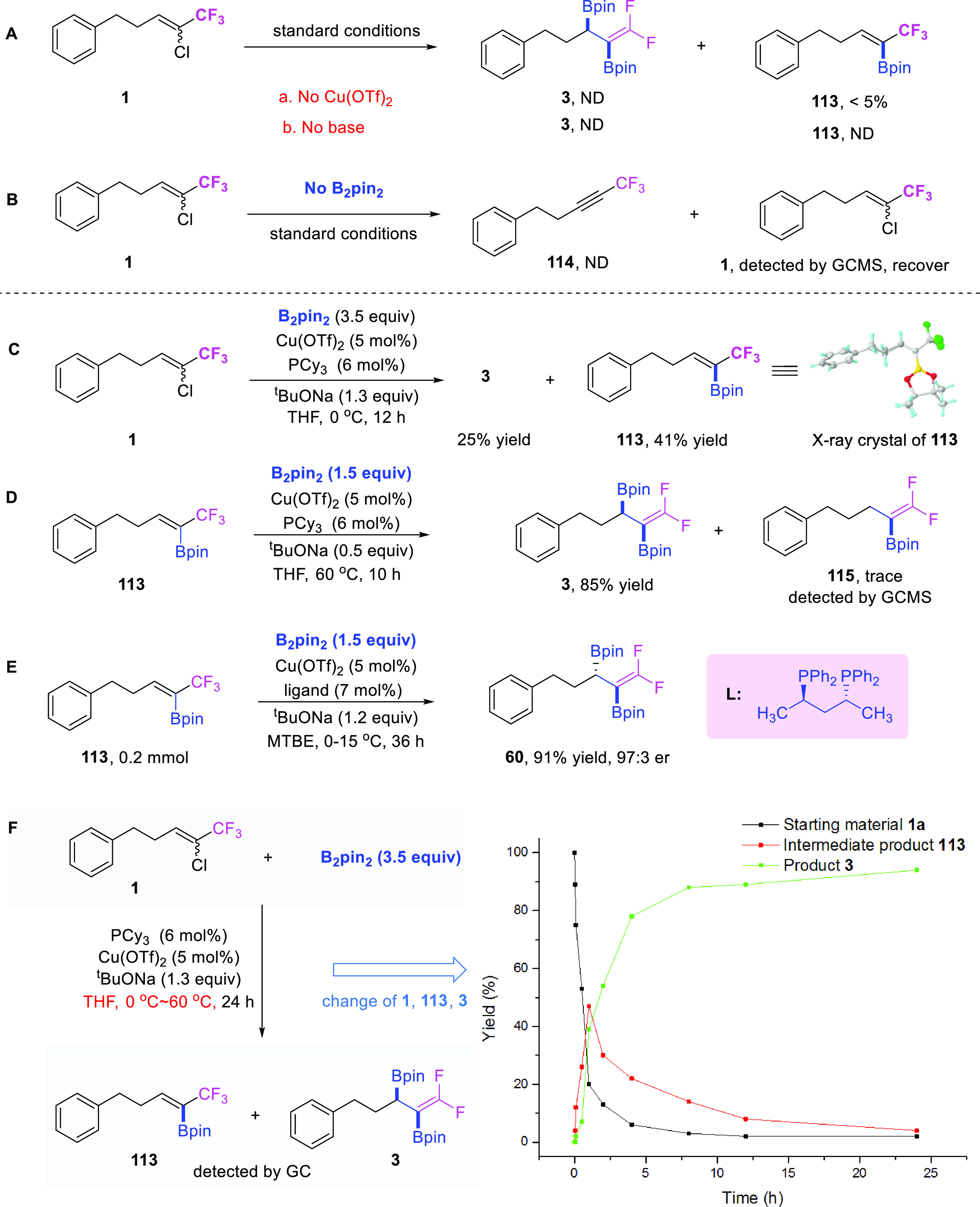
Mechanism studies.

In order to thoroughly understand the reaction mechanism, several control experiments were carried out. First, when the reaction was performed in the absence of copper salt or base, the target product **3** was not detected, which inferred that the Cu catalyst and base played key roles for the success of this transformation (Figure 5A). When the reaction was performed in the absence of B_2_pin_2_ (Figure 5B), compound **114** was not observed; the result of this experiment inferred that alkyne **114** was not the intermediate in this transformation. When we lowered the reaction temperature to 0 °C, compound **113** with only one boron moiety was obtained in 41% isolated yield, along with 25% yield of target product **3** (Figure 5C). When monoboron **113** was subjected to the reaction with only 1.5 equiv of B_2_pin_2_ and 0.5 equiv of base, the desired target product **3** was obtained in 85% isolated yield (Figure 5D). Intriguingly, when monoboron **113** was exposed to asymmetric conditions, the corresponding desired chiral product **60** was obtained in 91% yield with a 97:3 er value (Figure 5E). Based on the above experiments, we have sufficient reasons to believe that compound **113** is the key intermediate of the reaction. To further validate our hypothesis, we monitored the yield changes of intermediate **113** and product **3** over time, and we found that the yield of **3** gradually increased as time went on during the reaction; meanwhile, the yields of compound **113** initially increased in the first 2 h and then decreased gradually after that (Figure 5F). These results further confirmed the intermediacy of compound **113** in our transformation.

**Figure 6:**
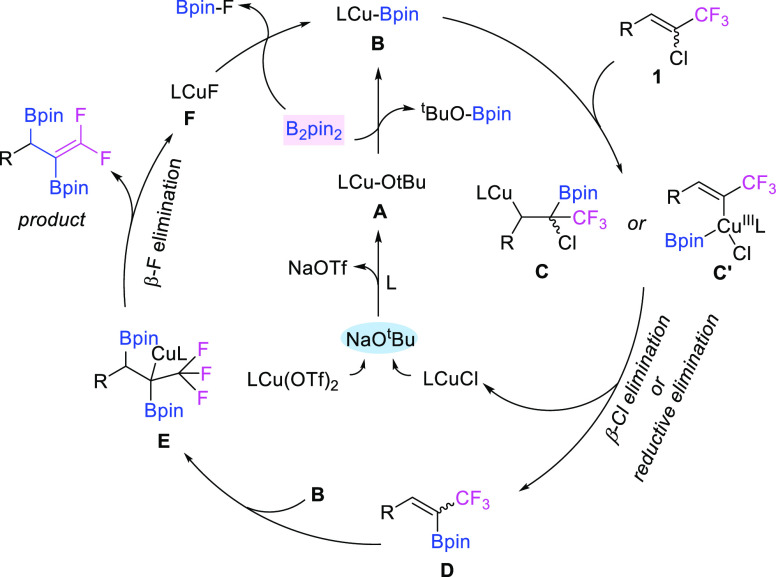
Proposed mechanisms.

On the basis of the above results and previous reports,^43,45,49,87^ we proposed a tentative reaction mechanism for this copper-catalyzed diborylation of 1-chloro-1-trifluoromethylalkenes, as shown in Figure 6. First, copper(I) alkoxide intermediate **A** is formed between Cu(OTf)_2_, ligand, and ^*t*^BuONa, which further reacts with diboron (B_2_pin_2_) to afford the Cu—B complex **B**. Subsequently, the C=C double bond of the starting material **1** inserts into the complex **B** to form copper(I) intermediate **C**; further β-chloro elimination of intermediate **C** gives the new olefin **D** (**path a**), or the Cu(I)–B complex goes through an oxidative addition to vinyl-Cl bond to afford intermediate **C**′, which further converts into olefin **D** after reductive elimination (**path b**). Then, the C=C double bond of olefin **D** regioselectively inserts into Cu—B complex **B** again to form copper intermediate **E**; subsequent β-fluoro elimination of **E** finally delivers the desired diborylative product along with LCuF (**F**). The formed copper(I) fluoride **F** reacts further with diboron (B_2_pin_2_)^88,89^ to regenerate Cu—B complex **B** to complete the catalytic cycle. Of note, after the second C=C bond insertion to the Cu—B complex, β—F elimination occurs to lead to a new alkene (*gem*-difluoroalkene; thus, the final chirality center has nothing to do with the Z isomer or E isomer of the starting 1-chloro-1-trifluoromethylalkenes, and both of them could give the same single enantiomer.

In conclusion, we developed a facile enantioselective copper-catalyzed diborylation reaction from readily accessible 1-chloro-1-trifluoromethylalkenes, commercially available diboron reagent, and inexpensive Cu catalyst to produce a diverse array of enantioenriched *gem*-difluoroallyl diboronates. In addition to the universality of substrates, this method is also suitable for bioactive and complex molecules and could be a very flexible conversion into versatile enantioenriched *gem*-difluoroallyl skeletons. We anticipate that this strategy based on the diversity of boron chemistry will simplify the synthesis and enhance structural elaborations of *gem*-difluoroalkene targets for chemistry, biology, and pharmaceutical and medicinal chemistry.

## Supporting Information Available

Experimental procedures, analytical and spectroscopic data for new compounds, and copies of NMR spectra (PDF)
