# Supplementary material for: Enantioselective Copper-Catalyzed sp2/sp3 Diborylation of 1-Chloro-1-Trifluoromethylalkenes
Source: ACS Cent Sci. 2022 Jul 20;8(8):1134–44. doi: 10.1021/acscentsci.2c00339 (PMC9413839; doi:10.1021/acscentsci.2c00339)
Supplement: Supplementary file 1 — oc2c00339_si_001.pdf [file oc2c00339_si_001.pdf]

Supplemental Information

for

Enantioselective Copper-Catalyzed *sp²/sp³* Diborylation of 1-Chloro-1-trifluoromethylalkenes

Zhenwei Fan,[1] Mingxing Ye,[1] Yahao Wang,[1] Jian Qiu,[1] Wangyang Li,[1] Xingxing Ma,[1] Kai Yang[1] and Qiuling Song*[1,2,3]

[1]Key Laboratory of Molecule Synthesis and Function Discovery, Fujian Province University, College of Chemistry at Fuzhou University, Fuzhou, Fujian 350108, China.

[2]Institute of Next Generation Matter Transformation, College of Materials Science Engineering at Huaqiao University, Xiamen, Fujian 361021, China.

[3]School of Chemistry and Chemical Engineering, Henan Normal University, Xinxiang, Henan, 453007, China

*Email: qsong@hqu.edu.cn

Total pages: 419

Total figures: 552

Total tables: 4

Table of Contents

1. General Information ……………………………………………..….……………….………S3

2. General Process for the Synthesis of Starting Materials……………………………………….….S4

2.1 General procedure A for the preparation of aldehydes……….…..…..…..…………………...S4

2.2 General procedure B for the preparation of 1-chloro-1-trifluoromethylalkenes……………...S4

2.3 General Procedure C for the preparation of (Z)-trifluoromethylated alkenyl triflates………..S5

3. General Process for the Synthesis of Racemic Products……………………………………….S5

3.1 General procedure D for the synthesis of **3-34**, **36-38**……………………………………...S5

3.2 General procedure E for the synthesis of **35**, **39-42**……………………………………..S6

3.3 General procedure F for the synthesis of **57-59**……………………………………….S6

4. General Process for the Synthesis of Asymmetric Products…………………………………S6

4.1 General procedure G for the synthesis of **60-92**, **95-97**……………………………….S6

4.2 General procedure H for the synthesis of **93**, **94**……………………………………...S7

5. General Process for the Synthesis of Chiral Allylic Alcohols……………..……..………………S7

6. Enantio-retentiveDerivatizations of Asymmetric Products…………………………………….S8

7. Mechanism Studies……………………………………………………………………....S12

8. Crystal Structure of Compound **3**, **51**, **75**, **113**……….…………………………….........S14

9. Characterization Data………………...……………………………………..……………...S19

10. NMR Spectroscopic Data….…………………………………………………………….S112

11. References……………………………………..…………………………………………S419

1. General Information

All experiments were conducted with a Schlenk tube under an argon atmosphere. Flash column chromatography was performed over silica gel (200-300 mesh). ^{1}H NMR, ^{11}B NMR, ^{13}C NMR and ^{19}F NMR spectra were recorded at ambient temperature using Bruker Ascend^{TM} 400 (400 MHz) spectrometer, Bruker AVANCE III 500M spectrometers or JNM-ECZ500R/S1 (500 MHz) spectrometer. ^{1}H NMR chemical shifts (in ppm) were referenced to $CDCl_3$ (δ = 7.26 ppm), DMSO-d_6 (δ = 2.50 ppm) and acetone-d_6 (δ = 2.05 ppm) as internal standards. ^{13}C NMR spectra were obtained by using the same NMR spectrometers and were calibrated with $CDCl_3$ (δ = 77.16 ppm), acetone-d_6 (δ = 29.84 ppm). The following abbreviations are used: s = singlet, d = doublet, t = triplet, q = quartet, dd, = double doublet, dt = double triplet, td = triple doublet, m = multiplet. Analytical thin-layer chromatography (TLC) was carried out on Merck 60 F254 pre-coated silica gel plate (0.2 mm thickness). Visualization was accomplished by UV light (254 nm), phosphomolybdic acid or $KMnO_4$ staining solutions followed by heating, also by Gas chromatograph-Mass spectrometer analysis (GC-MS). High resolution mass spectroscopy (HRMS) analysis was performed at an Exactive Plus (Thermo Scientific) or Agilent 8890-7250. Enantioselectivities were recorded on Waters or Agilent HPLC. The X-ray crystal structure was measured on Bruker D8 venture. Unless otherwise noted, materials obtained from commercial suppliers were used without further purification.

2. General Process for the Synthesis of Starting Materials [1].

2.1 General procedure A for the preparation of aldehydes.

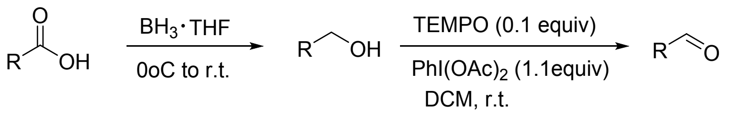

Step I:

To a solution of carboxylic acid (10 mmol, 1.0 equiv.) in anhydrous THF (10 mL) at 0 °C was added BH_3•THF (1.1 equiv, 1.0 M solution in THF). The resulting mixture was warmed slowly to room temperature and maintained at this temperature until the consumption of the starting carboxylic acid (monitored by TLC). Then the solvent was removed under vacuum and diluted with ethyl acetate, followed by quenched with water and the aqueous phase was extracted with ethyl acetate. The combined organic layers were then dried over Na_2SO_4, and concentrated under reduced pressure. Without further purification and go directly to the next reaction.

Step II:

To a dry round-bottom flask, the respective alcohol (1.0 equiv.), CH_2Cl_2, and TEMPO (0.1 equiv.) were added and stirred until dissolution. To the stirring solution, indobenzene diacetate (1.1 equiv.) was added and the reaction stirred at room temperature until the consumption of the starting alcohol (monitored by TLC). After completion, the reaction was diluted with CH_2Cl_2 and washed with saturated $Na_2S_2O_3$ solution, the saturated $NaHCO_3$ solution, and saturated brine solution. The combine organic extracts were dried over Na_2SO_4, then the solvent was removed under vacuum and the crude product was purified by column chromatography on silica gel.

Step III:

2.2 General procedure B for the preparation of 1-chloro-1-trifluoromethylalkenes[2].

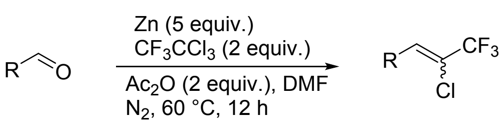

To a 100 mL Schlenk flask equipped with stirring bar, Zn (5 equiv.) and DMF (15 ml) was added and the flask was evacuated and refilled with Nitrogen (3 times).1,1,1-trichloro-2,2,2-trifluoroethane (2 equiv.), acetic anhydride (2 equiv.), aldehyde (10 mmol, with 5 mL DMF) was added with a syringe and the reaction was stirred for 15 minutes at room temperature, then place in an oil bath at 60 °C for 12 h. The reaction mixture was cooled to room temperature, then diluted with ethyl acetate, quenched by

dropping NH_4Cl (20 mL, sat. aq.), extracted with ethyl acetate (3 × 30 mL). The ethyl acetate layer was washed with water (3 × 30 mL), and washed with saturated brine once, dried over Na_2SO_4, then combined the organic phases, evaporated the solvent. The crude product was purified by flash column chromatography to give the corresponding product 1-chloro-1-trifluoromethylalkenes.

2.3 General procedure C for the preparation of (Z)-trifluoromethylated alkenyl triflates[3].

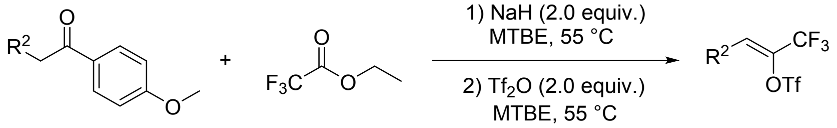

To a suspension of NaH (240 mg, 10.0 mmol, powder) in MTBE (5 mL) was added ethyl trifluoroacetate (1.2mL, 10.0 mmol) at room temperature under N_2 atmosphere. After 1 min of stirring, a solution of enolizable ketone (5.0 mmol) in MTBE (5 mL) was added, and the mixture was refluxed for 6−12 h. After the reaction was complete (monitored by TLC and GC analysis), the reaction solution was cooled to 0 °C. Tf_2O (2.82 g, 10 mmol) was added dropwise to the reaction mixture. After reaction completion (monitored by TLC and GC analysis), the reaction was quenched with ice-water. The aqueous layer was separated and extracted with EtOAc. The combined organic extracts were washed with brine and dried over Na_2SO_4, and solvent was removed under reduced pressure. The crude product was purified by column chromatography on silica gel (petroleum ether as eluent) to afford the corresponding (Z)-trifluoromethylated alkenyl triflates products.

3. General Process for the Synthesis of Racemic Products

3.1 General procedure D for the synthesis of 3-34, 36-38

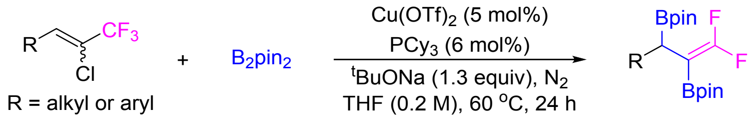

A 25 mL schlenk tube was charged with B_2pin_2 (3.5 equiv), $Cu(OTf)_2$ (5 mol%), PCy_3 (6 mol%), ^tBuONa (1.3 equiv), then 2.5 mL of THF, 1-chloro-1-trifluoromethylalkenes (0.5 mmol) was added in sequence under N_2 atmosphere. The reaction was allowed to stir at 60 °C for 24 h. After finished, the reaction mixture was concentrated and purified by column chromatography to afford the corresponding racemic products **3-34**, **36-38**.

3.2 General procedure E for the synthesis of 35, 39-42

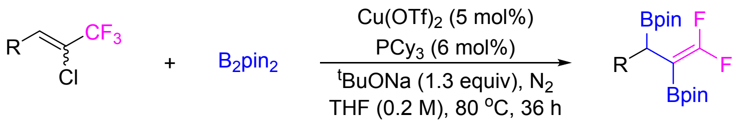

A 25 mL schlenk tube was charged with B_2pin_2 (3.5 equiv), $Cu(OTf)_2$ (5 mol%), PCy_3 (6 mol%), ^tBuONa (1.3 equiv), then 2.5 mL of THF, 1-chloro-1-trifluoromethylalkenes (0.5 mmol) was added in sequence under N_2 atmosphere. The reaction was allowed to stir at 80 °C for 36 h. After finished, the reaction mixture was concentrated and purified by column chromatography to afford the corresponding racemic products **35**, **39-42**.

3.3 General procedure F for the synthesis of 57-59

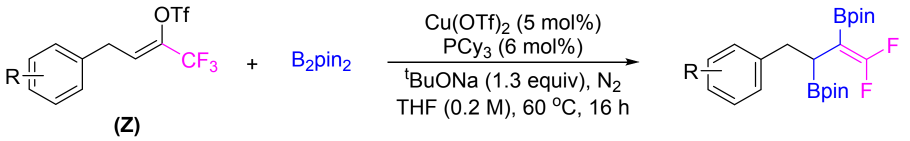

A 25 mL schlenk tube was charged with B_2pin_2 (3.5 equiv), $Cu(OTf)_2$ (5 mol%), PCy_3 (6 mol%), ^tBuONa (1.3 equiv), then 2.5 mL of THF, (Z)-trifluoromethylated alkenyl triflates (0.5 mmol) was added in sequence under N_2 atmosphere. The reaction was allowed to stir at 60 °C for 16 h. After finished, the reaction mixture was concentrated and purified by column chromatography to afford the corresponding racemic products **57-59**.

4. General Process for the Synthesis of Asymmetric Products

4.1 General procedure G for the synthesis of 60-92, 95-97

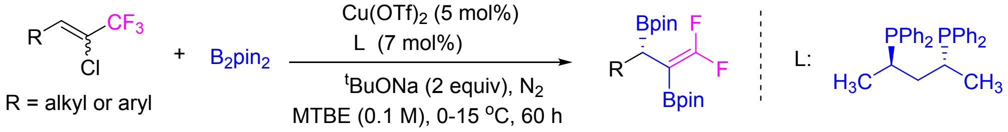

A 25 mL schlenk tube was charged with B_2pin_2 (4.0 equiv), $Cu(OTf)_2$ (5 mol%), **L** (7 mol%), ^tBuONa (2.0 equiv), then 3 mL of MTBE, 1-chloro-1-trifluoromethylalkenes (0.3 mmol) was added in sequence under N_2 atmosphere at 0 °C. Then slowly rise to 15 °C and the reaction was allowed to stir at 15 °C for 60 h. After finished, the reaction mixture was concentrated and purified by column chromatography to afford the corresponding Asymmetric products **60-92**, **95-97**.

4.2 General procedure H for the synthesis of 93, 94

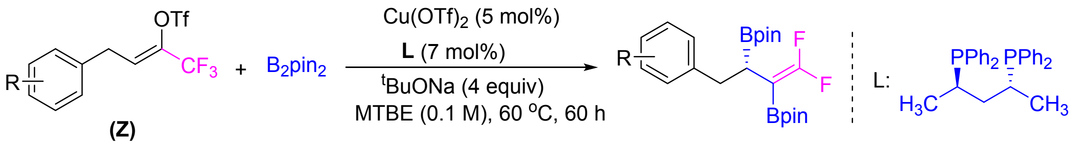

A 25 mL schlenk tube was charged with B_2pin_2 (4.0 equiv), $Cu(OTf)_2$ (5 mol%), **L** (7 mol%), ^{t}BuONa (2.0 equiv), then 3 mL of MTBE, (Z)-trifluoromethylated alkenyl triflates (0.3 mmol) was added in sequence under N_2 atmosphere at 0 °C. The reaction was allowed to stir at 0 °C for 60 h. After finished, the reaction mixture was concentrated and purified by column chromatography to afford the corresponding Asymmetric products **93**, **94**.

5. General Process for the Synthesis of Chiral Allylic Alcohols

5.1 General procedure I for the synthesis of 98-101.

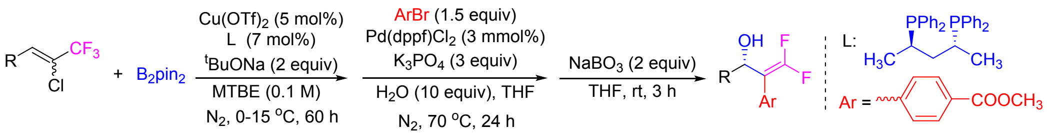

By analogy to general produce D and a modified literature procedure,[4,5] a 25 mL schlenk tube was charged with B_2pin_2 (4.0 equiv), $Cu(OTf)_2$ (5 mol%), **L** (7 mol%), ^{t}BuONa (2.0 equiv), then 3 mL of MTBE, 1-chloro-1-trifluoromethylalkenes (0.3 mmol) was added in sequence under N_2 atmosphere at 0 °C. Then slowly rise to 15 °C and the reaction was allowed to stir at 15 °C for 60 h. After finished, the reaction mixture was filtered through a short plug of silica gel and washed with EtOAc as the eluent. The filtrates were concentrated to near dryness, dissolved in the THF (3.0 mL). Then the mixture was added to another 25 mL Schlenk tube which was charged with methyl 4-bromobenzoate (83.9 mg, 0.39 mmol), K_3PO_4 (191.0 mg, 0.9 mmol) and [1,1′-bis (diphenyl phosphino)ferrocene] dichloropalladium(II) (6.6 mg, 0.009 mmol), 54 μL (10 equiv, 3 mmol) of H_2O was added under argon atmosphere. The tube was evacuated and filled with argon for three times. The reaction was allowed to stir at 70 °C for 24 h. After finished, the reaction was cooled to room temperature and filtered through a short plug of silica gel and washed with EtOAc as the eluent. The filtrates were concentrated to a 25 mL round-bottom flask to near dryness, then diluted with THF (1mL) and water (1 mL), then $NaBO_3 \cdot 4H_2O$ (92.3 mg, 0.6 mmol) was added and the mixture was allowed to stir at rt for 3 h. The mixture was washed with EtOAc (10 mL)

three times and the combined organic layers was dried over Na_2SO_4, concentrated and purified by silica gel chromatography to afford **98-101**.

6. Enantio-retentive Derivatizations of Asymmetric Products

6.1 General procedure J for the synthesis of 102

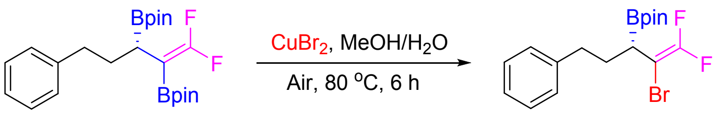

61 **102**

By analogy to a modified literature procedure,[6] a mixture of **61** (86.9 mg, 0.2 mmol), $CuBr_2$ (134.0 mg, 0.6 mmol) and MeOH/H_2O (1.0 mL/1.0 mL) was stirred at 80 °C for 6 h under air atmosphere. After the completion of reaction, the reaction mixture was quenched with saturated aqueous solution of NaCl (3 mL) and extracted with EtOAc (10 mL x 3). The combined organic layer was dried over Na_2SO_4, filtered and evaporated under vacuo. The residue was purified by column chromatography using PE/Et_2O = 10/1 as the eluent to afford **102** as a colorless liquid.

6.2 General Procedure K for the synthesis of 103

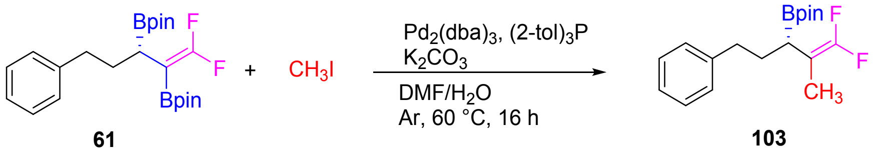

61 **103**

By analogy to a modified literature procedure,[7] to a mixture of **61** (86.9 mg, 0.2 mmol), $Pd_2(dba)_3$ (9.2 mg, 0.01 mmol), Tris(o-methylphenyl)phosphine (3.7 mg, 0.012 mmol), and K_2CO_3 (55.3 mg, 0.4 mmol) in 1.5 mL of DMF and 0.15 mL of H_2O was added CH_3I (42.6 mg, 0.3 mmol) under a nitrogen atmosphere. The reaction mixture was stirred at 60 °C for 16 h and then cooled to room temperature. The reaction mixture was quenched with water, extracted with EtOAc, washed with brine, dried over anhydrous Na_2SO_4, and evaporated under vacuo. The crude product was purified by flash column chromatography using PE/Et_2O = 10/1 as the eluent to give the corresponding product **102** as a colorless liquid.

6.3 General Procedure L for the synthesis of 104

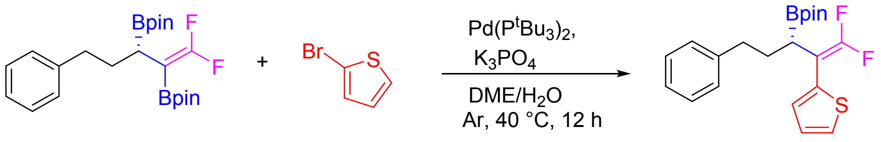

61 **104**

By analogy to a modified literature procedure,[8] boronate ester **61** (86.9 mg, 0.2 mmol), 2-Bromothiophene (65.2 mg, 0.4 mmol), K_3PO_4 (169.8 mg, 0.8 mmol), and Bis(tri-tert-butylphosphine)palladium(0) (5.1 mg, 0.01 mmol) was charged with a flask. After the atmosphere in the flask was replaced with argon, dimethoxyetane (2 mL) and water (0.2 mL) was added to reaction mixture. After the solution was stirred for 12 h at 40 °C in an oil bath, the reaction mixture was quenched with saturated NH_4Cl aqueous (10 mL), and the aqueous phase was extracted with Et_2O (10 mL × 3). The combined organic phase was washed with brine, dried over Na_2SO_4 and evaporated under vacuo. The residue was purified by column chromatography using PE/Et_2O = 10/1 as the eluent to afford **102** as a colorless liquid.

6.4 General Procedure M for the synthesis of 105-E

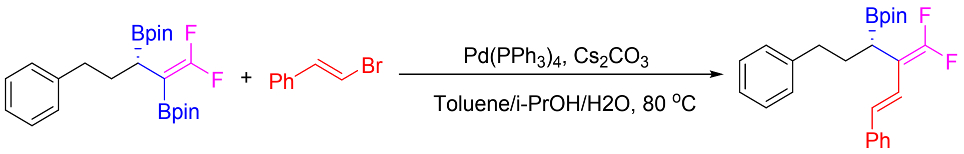

61 **105-E**

By analogy to a modified literature procedure,[7] to a mixture of **61** (86.9 mg, 0.2 mmol), $Pd(PPh_3)_4$ (6.9 mg, 0.006 mmol), and Cs_2CO_3 (325.8 mg, 0.6 mmol) in 1.5 mL of PhMe, 0.5 mL of i-PrOH and 0.5 ml of H_2O was added, then (E)-styrylbromide (43.9 mg, 0.24 mmol) under a nitrogen atmosphere was added. After stirring at 80 °C for 16 h, the reaction mixture was quenched with water, extracted with EtOAc, washed with brine, dried over anhydrous Na_2SO_4, then concentrated, and crude product was purified by flash column chromatography using PE/Et_2O = 10/1 as the eluent to give the corresponding product **105 (E)** as a colorless liquid.

6.5 General Procedure N for the synthesis of 106

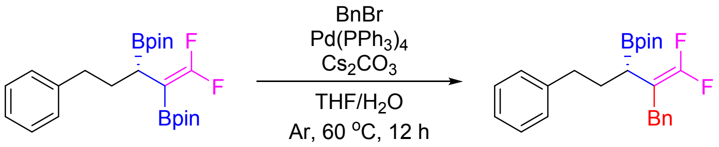

61 **106**

By analogy to a modified literature procedure,[7] to a mixture of **66** (86.9 mg, 0.2 mmol), $Pd(PPh_3)_4$ (11.6 mg, 0.01 mmol), and Cs_2CO_3 (97.8 mg, 0.3 mmol) in 1.5 mL of THF/H_2O (v/v = 20:1) was added BnBr (42.7 mg, 0.25 mmol) under a nitrogen atmosphere. After stirring at 60 °C for 12 h, cooled to room temperature, the mixture was filtered through a short plug of silica gel and washed with EtOAc as the eluent. The filtrates were concentrated and crude product was purified by flash column chromatography

using PE/Et_2O = 10/1 as the eluent to give the corresponding product **106** as a colorless liquid.

6.6 General Procedure O for the synthesis of 107

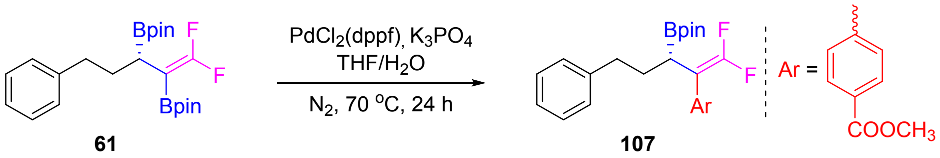

By analogy to a modified literature procedure,[4] a Schlenk tube equipped with a magnetic stirring bar was charged with **61** (86.9 mg, 0.2 mmol), methyl 4-bromobenzoate (55.9 mg, 0.26 mmol), K_3PO_4 (127.4 mg, 0.6 mmol) and [1,1′-bis (diphenyl phosphino)ferrocene] dichloropalladium(II) (4.4 mg, 0.006 mmol), then 3 mL of THF and 36 µL (10 equiv, 2 mmol) of H_2O was added under argon atmosphere. The reaction mixture was stirred at 70 °C for 24 h and then cooled to room temperature. The mixture was filtered through a short plug of silica gel and washed with EtOAc as the eluent. The filtrates were concentrated and crude product was purified by flash column chromatography using PE/Et_2O = 10/1 as the eluent to give the corresponding product **107** as a colorless liquid.

6.7 General Produce P for the Synthesis of 108

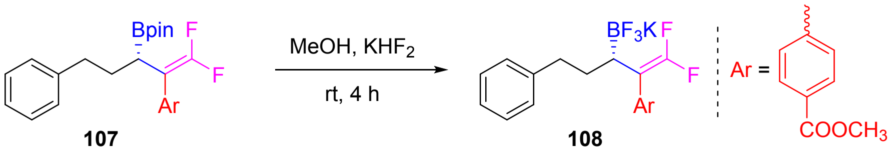

By analogy to a modified literature procedure,[9] in a 25 mL round-bottom flask, a solution of KHF_2 (62.5 mg, 0.8 mmol) in H_2O (0.2 mL) was added to a stirring solution of **107** (88.4 mg, 0.2 mmol) in MeOH (0.4 mL) at 0 °C. Once added, the ice bath was removed and the reaction mixture was stirred for 4 h. Upon finished, the resultant suspension was concentrated under reduced pressure. Then hot acetone (5 mL x 3) was added and filtered. The filtrate was concentrated to near dryness and Et_2O (4 mL) was added to yield a white precipitate. The precipitate was isolated by filtration, washed by CH_2Cl_2 (0.5 mL), to afford **108** as a white solid.

6.8 General Procedure Q for the synthesis of 109

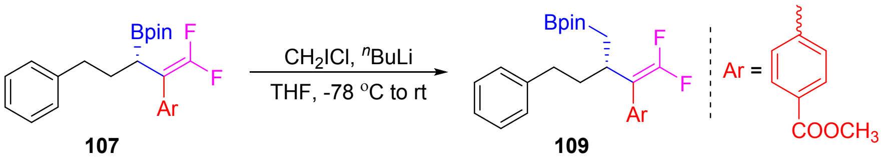

By analogy to a modified literature procedure,[5] to a 25 mL round bottomed flask containing a magnetic

stir bar, boronate ester **107** (44.2 mg, 0.1 mmol), chloroiodomethane (52.9 mg, 0.3 mmol) and THF (1 mL) were added. The reaction mixture was cooled to –78 °C and a solution of *n*-BuLi (0.19 mL, 0.3 mmol, 1.6 M in hexane) was slowly added at the same temperature. After stirring for 10 min, the reaction mixture was warmed to room temperature and additionally stirred for 2 h. The reaction mixture was quenched with saturated aqueous NH_4Cl solution (10 mL) and extracted with CH_2Cl_2 (10 mL x 3). The combined organic layers were dried over $MgSO_4$, purified by column chromatography on silica gel PE/Et_2O = 10/1 to afford **108** as a colorless oil.

6.9 General Procedure R for the synthesis of 110

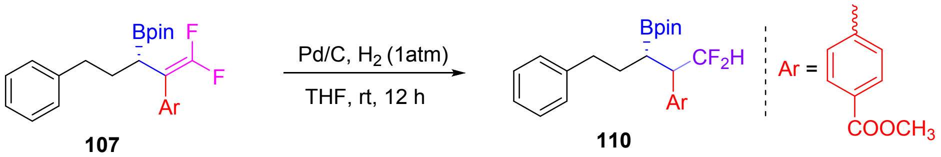

107 **110**

By analogy to a modified literature procedure,[5] to the alkene **107** (88.4 mg, 0.2 mmol) in THF (5 mL), was added palladium on activated carbon (5%, 0.1 equiv, 42.4 mg). The reaction solution was purged with hydrogen balloon for 15 minutes and then went overnight under hydrogen balloon. Then, the reaction was filtered over a short path of Celite, concentrated in vacuo, and the crude mixture was purified by flash column chromatography to afford the final product **110** as a colorless liquid.

6.10 General Procedure S for the synthesis of 111

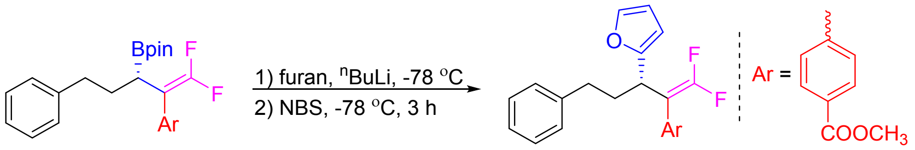

107 **111**

By analogy to a modified literature procedure,[10] a solution of furan (8.2 mg, 0.12 mmol) in THF (1.0 mL) was cooled to –78 °C and treated with n-BuLi (75 µL, 0.12 mmol, 1.6 M in hexanes). The cooling bath was removed and the mixture was stirred at room temperature for 1 h. The mixture was cooled to –78 °C and **107** (44.2 mg, 0.1 mmol) was added dropwise as a solution in THF (1.0 mL). The mixture was stirred at –78 °C for 1 h. A solution of the electrophile (0.12 mmol) in THF (1.0 mL) was added dropwise. After 1 h at –78°C, $Na_2S_2O_3$ sat (2 mL) was added and the reaction mixture was allowed to warm to room temperature. The reaction mixture was diluted with Et_2O and water. The layers were separated and the aqueous layer was extracted with Et_2O. The combined organic layers were dried ($MgSO_4$), filtered and concentrated under vacuum and purified by column chromatography on silica gel PE/Et_2O = 10/1 to

afford **111** as a colorless oil.

6.11 General Procedure T for the synthesis of 112

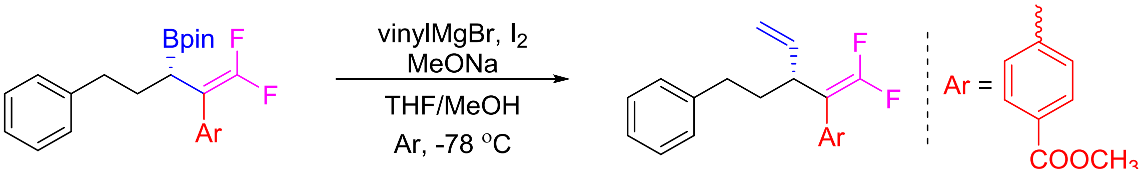

107 **112**

By analogy to a modified literature procedure,[11] an oven-dried 10 mL Schlenk tube equipped with a Teflon stir bar was added with a solution of **107** (44.2 mg, 0.1 mmol) in an hydrous THF (1 mL) under Argon atmosphere via syringe. Then a solution of vinyl magnesium bromide (0.4 mmol, 0.8 mL, 0.5 M in THF) was added dropwise, and the mixture was stirred for 30 min at room temperature. Then the tube was cooled to -78 °C and a solution of I_2 (0.4 mmol in 1 mL MeOH) was added dropwise. The solution was stirred for another 30 min at this temperature, followed by addition of a solution of NaOMe (0.8 mmol in 1 mL MeOH). The reaction mixture was allowed to warm to room temperature and stirred for another 2 hours. Once finished, the reaction was quenched with 2 mL saturated sodium thiosulfate aqueous. The aqueous solution was extracted with EtOAc three times. The combined organic layers were dried over anhydrous Na_2SO_4, filtered and concentrated. The crude material was purified by column chromatography on silica gel PE/Et_2O = 10/1 to afford **111** as a colorless oil.

7. Mechanism studies

Control experiments:

1)

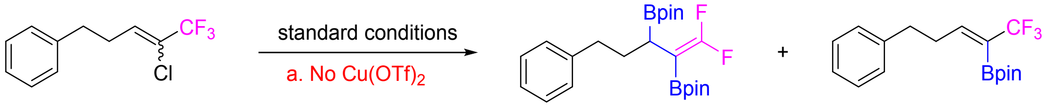

1 **3**, ND **113**, < 5%

A 25 mL Schlenk tube was charged with B_2pin_2 (3.5 equiv), PCy_3 (6 mol%), tBuONa (1.3 equiv), then 1.0 mL of THF, 1-chloro-1-trifluoromethylalkenes (0.2 mmol) was added in sequence under N_2 atmosphere. The reaction was allowed to stir at 60 °C for 24 h. After finished, the crude mixture was

detected by GC-MS.

2)

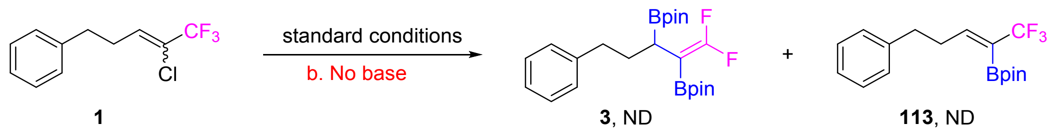

A 25 mL Schlenk tube was charged with B_2pin_2 (3.5 equiv), $Cu(OTf)_2$ (5 mol%), PCy_3 (6 mol%), then 1.0 mL of THF, 1-chloro-1-trifluoromethylalkenes (0.2 mmol) was added in sequence under N_2 atmosphere. The reaction was allowed to stir at 60 °C for 24 h. After finished, the crude mixture was detected by GC-MS.

3)

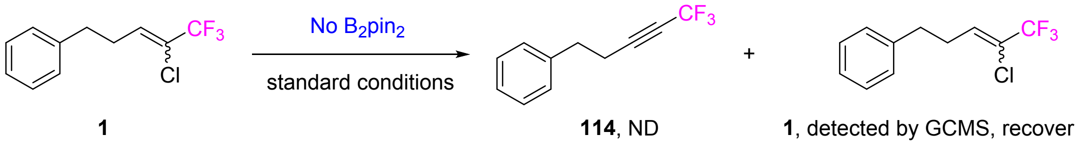

A 25 mL Schlenk tube was charged with $Cu(OTf)_2$ (5 mol%), PCy_3 (6 mol%), ^tBuONa (1.3 equiv), then 1.0 mL of THF, 1-chloro-1-trifluoromethylalkenes (0.2 mmol) was added in sequence under N_2 atmosphere. The reaction was allowed to stir at 60 °C for 24 h. After finished, the crude mixture was detected by GC-MS.

4)

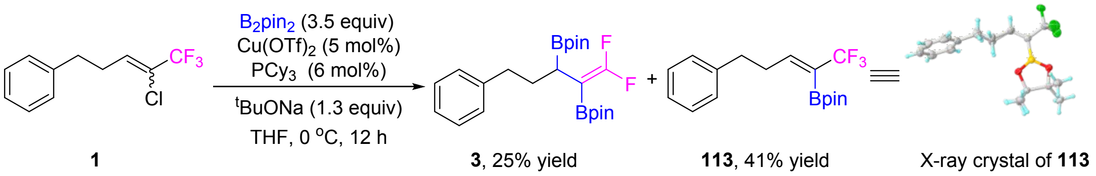

A 25 mL Schlenk tube was charged with B_2pin_2 (3.5 equiv), $Cu(OTf)_2$ (5 mol%), PCy_3 (6 mol%), ^tBuONa (1.3 equiv), then 1.0 mL of THF, 1-chloro-1-trifluoromethylalkenes (0.2 mmol) was added in sequence under N_2 atmosphere. The reaction was allowed to stir at 0 °C for 12 h. After finished, the reaction mixture was concentrated and purified by column chromatography to afford the corresponding products **3**, **113**.

5)

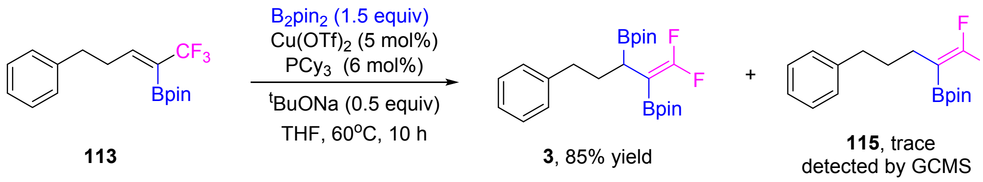

A 25 mL Schlenk tube was charged with B_2pin_2 (1.5 equiv), $Cu(OTf)_2$ (5 mol%), PCy_3 (6 mol%), ${}^tBuONa$ (0.5 equiv), then 1.0 mL of THF, 1-chloro-1-trifluoromethylalkenes (0.2 mmol) was added in sequence under N_2 atmosphere. The reaction was allowed to stir at 60 °C for 10 h. After finished, the crude mixture was detected by GC-MS. The reaction mixture was concentrated and purified by column chromatography to afford the corresponding products **3**.

6)

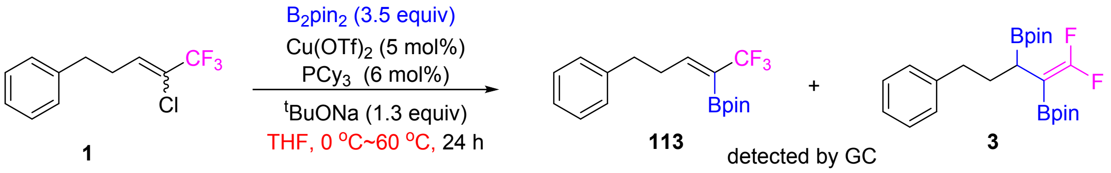

A 25 mL Schlenk tube was charged with B_2pin_2 (3.5 equiv), $Cu(OTf)_2$ (5 mol%), PCy_3 (6 mol%), ${}^tBuONa$ (1.3 equiv), then 1.0 mL of THF, 1-chloro-1-trifluoromethylalkenes (0.5 mmol) was added in sequence under N_2 atmosphere. The reaction was allowed to stir at 0 °C, slowly rise to 60 °C, the mixture was detected by GC, using dodecane as an internal standard.

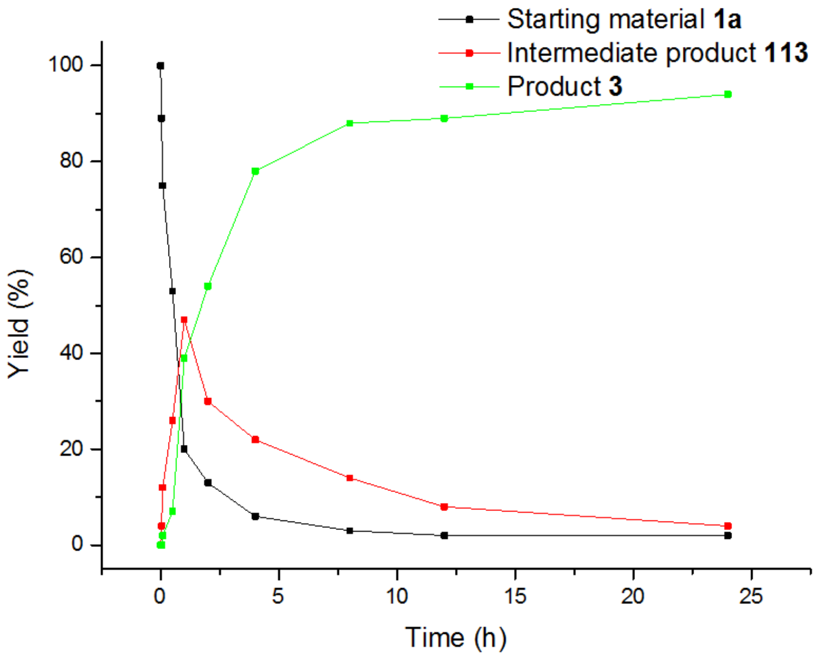

8. Crystal Structure of Compounds 3, 51, 75, 113

A small amount of ethyl acetate was used to dissolve the compound, and petroleum ether was slowly

added to the solution with a dropper. The single crystal was obtained by slowly evaporating mixed solvent at room temperature under the air conditions. X-ray crystal structure analysis were measured on Bruker D8 venture. The X-ray data have been deposited at the Cambridge Crystallographic Data Center. For **3** the data was collected by using molybdenum (Mo) irradiation source at 150K

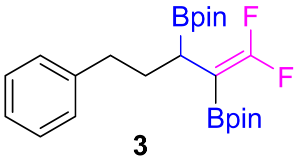

3

CCDC: 2126195

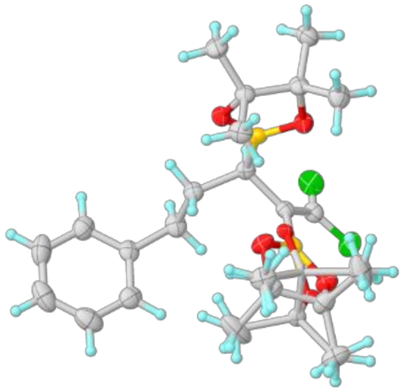

Crystal data and structure refinement for 3

Identification code	P21c
Empirical formula	$C_{23}H_{34}B_2F_2O_4$
Formula weight	434.12
Temperature/K	150
Crystal system	monoclinic
Space group	$P2_1/c$
a/Å	8.4802(6)
b/Å	24.243(2)
c/Å	11.9586(11)
α/°	90
β/°	105.007(3)
γ/°	90
Volume/Å³	2374.7(4)
Z	4
ρ_{calc}g/cm³	1.214
μ/mm⁻¹	0.090
F(000)	928.0
Crystal size/mm³	0.15 × 0.13 × 0.12
Radiation	MoKα (λ = 0.71073)
2θ range for data collection/°	3.906 to 50.728
Index ranges	-10 ≤ h ≤ 10, -29 ≤ k ≤ 29, -14 ≤ l ≤ 14
Reflections collected	41868

Independent reflections	4350 [R_{int} = 0.0826, R_{sigma} = 0.0426]
Data/restraints/parameters	4350/0/325
Goodness-of-fit on F^2	1.024
Final R indexes [I>=2σ (I)]	R_1 = 0.0464, wR_2 = 0.1023
Final R indexes [all data]	R_1 = 0.0772, wR_2 = 0.1157
Largest diff. peak/hole / e Å^{-3}	0.28/-0.27

For **51** the data was collected by using molybdenum (Mo) irradiation source at room temperature

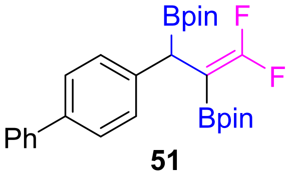

51

CCDC: 2126191

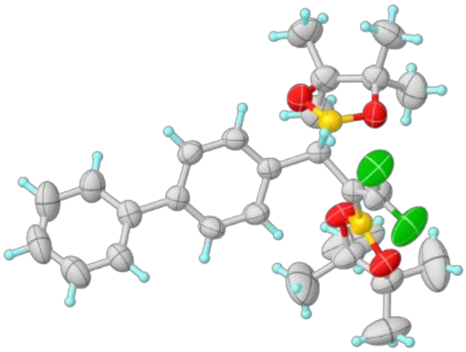

Crystal data and structure refinement for 51

Identification code	exp_7329
Empirical formula	$C_{27}H_{34}O_4F_2B_2$
Formula weight	482.16
Temperature/K	293(2)
Crystal system	monoclinic
Space group	$P2_1/c$
a/Å	12.1258(7)
b/Å	12.5854(7)
c/Å	17.4293(10)
α/°	90
β/°	93.808(5)
γ/°	90
Volume/Å^3	2654.0(3)
Z	4
ρ_{calc}g/cm^3	1.207
μ/mm^{-1}	0.087
F(000)	1024.0
Crystal size/mm^3	0.15 × 0.13 × 0.12
Radiation	MoKα (λ = 0.71073)
2θ range for data collection/°	7.3 to 58.448

Index ranges	$-16 \leq h \leq 15, -16 \leq k \leq 16, -21 \leq l \leq 23$
Reflections collected	13592
Independent reflections	6150 [R_{int} = 0.0314, R_{sigma} = 0.0553]
Data/restraints/parameters	6150/0/324
Goodness-of-fit on F^2	1.088
Final R indexes [I>=2σ (I)]	R_1 = 0.0715, wR_2 = 0.1795
Final R indexes [all data]	R_1 = 0.1458, wR_2 = 0.2198
Largest diff. peak/hole / e Å^{-3}	0.39/-0.22

For **75** the data was collected by using Cuprum (Cu) irradiation source at 150k

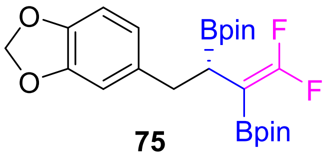

75

CCDC: 2126197

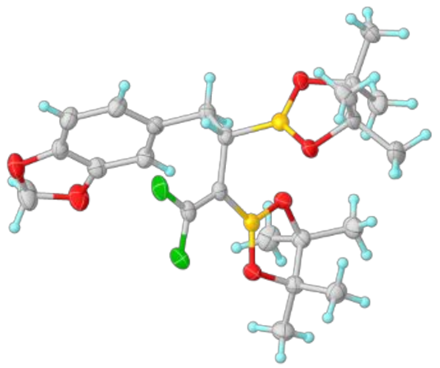

Crystal data and structure refinement for 75

Identification code	FZW090242_0m
Empirical formula	$C_{23}H_{32}B_2F_2O_6$
Formula weight	464.10
Temperature/K	150
Crystal system	monoclinic
Space group	$P2_1$
a/Å	9.0688(10)
b/Å	8.2338(9)
c/Å	16.5455(18)
α/°	90
β/°	98.299(8)
γ/°	90
Volume/Å^3	1222.5(2)
Z	2
ρ_{calc}g/cm^3	1.261
μ/mm^{-1}	0.817
F(000)	492.0
Crystal size/mm^3	0.15 × 0.13 × 0.12
Radiation	CuKα (λ = 1.54184)
2θ range for data collection/°	5.398 to 144.582
Index ranges	$-10 \leq h \leq 11, -10 \leq k \leq 10, -20 \leq l \leq 20$
Reflections collected	13223

Independent reflections	4343 [R_{int} = 0.0261, R_{sigma} = 0.0284]
Data/restraints/parameters	4343/1/306
Goodness-of-fit on F^2	1.048
Final R indexes [I>=2σ (I)]	R_1 = 0.0329, wR_2 = 0.0801
Final R indexes [all data]	R_1 = 0.0369, wR_2 = 0.0823
Largest diff. peak/hole / e Å^{-3}	0.17/-0.24
Flack parameter	0.01(5)

For **113** the data was collected by using molybdenum (Mo) irradiation source at room temperature

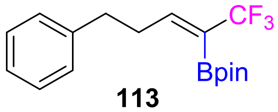

113

CCDC: 2126196

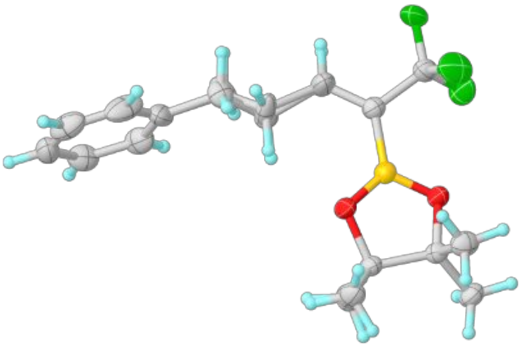

Crystal data and structure refinement for 113

Identification code	FZW004_0m
Empirical formula	$C_{17}H_{22}BF_3O_2$
Formula weight	326.15
Temperature/K	150
Crystal system	monoclinic
Space group	$P2_1/n$
a/Å	10.6529(8)
b/Å	13.1445(11)
c/Å	12.9336(11)
α/°	90
β/°	110.824(2)
γ/°	90
Volume/Å^3	1692.7(2)
Z	4
ρ_{calc}g/cm^3	1.280
μ/mm^{-1}	0.103
F(000)	688.0
Crystal size/mm^3	0.14 × 0.11 × 0.1
Radiation	MoKα (λ = 0.71073)
2θ range for data collection/°	4.276 to 64.462
Index ranges	-15 ≤ h ≤ 14, -18 ≤ k ≤ 19, -18 ≤ l ≤ 17
Reflections collected	37793

Independent reflections	4886 [R_{int} = 0.0720, R_{sigma} = 0.0675]
Data/restraints/parameters	4886/0/231
Goodness-of-fit on F^2	1.028
Final R indexes [I>=2σ (I)]	R_1 = 0.0606, wR_2 = 0.1215
Final R indexes [all data]	R_1 = 0.1298, wR_2 = 0.1414
Largest diff. peak/hole / e Å^{-3}	0.26/-0.32

9. Characterization Data

(4-chloro-5,5,5-trifluoropent-3-en-1-yl)benzene (1)

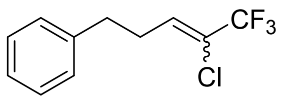

The reaction was performed following the **procedure B**. The residue was purified by flash column chromatograph (Rf = 0.8, silica gel, PE) to give the product as a colorless liquid (1.61 g, 69% yield). Z/E = 4.3:1, determined by ^{1}H NMR. **^{1}H NMR (500 MHz, $CDCl_3$)** δ 7.44 (t, *J* = 6.6 Hz, 2H), 7.33 (dd, *J* = 24.6, 7.7 Hz, 3H), 6.61 (t, *J* = 6.6 Hz, 0.82 x 1H), 6.32 (t, *J* = 8.0 Hz, 0.18 x 1H), 2.87 (q, *J* = 12.2, 9.9 Hz, 2H), 2.74 (dd, *J* = 17.4, 10.8 Hz, 2H). **^{13}C NMR (126 MHz, $CDCl_3$)** δ 140.3, 133.7 (q, *J* = 4.1 Hz), 128.8, 128.5, 126.6, 120.6 (q, *J* = 271.5 Hz), 120.5 (q, *J* = 38.0 Hz), 33.8, 29.8. **^{19}F NMR (471 MHz, $CDCl_3$)** δ -62.15, -68.83. **HRMS (EI)** calcd for $C_{11}H_{10}ClF_3$: 234.0423, found: 234.0413.

1-(4-chloro-5,5,5-trifluoropent-3-en-1-yl)-4-methylbenzene (1b)

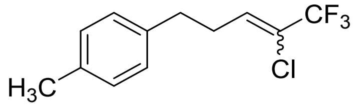

The reaction was performed following the **procedure B**. The residue was purified by flash column chromatograph (Rf = 0.8, silica gel, PE) to give the product as a colorless liquid (1.46 g, 59% yield). Z/E = 6.7:1, determined by ^{1}H NMR. **^{1}H NMR (500 MHz, $CDCl_3$)** δ 7.17 – 7.07 (m, 4H), 6.51 (t, *J* = 7.1 Hz, 0.87 x 1H), 6.23 (t, *J* = 7.9 Hz, 0.13 x 1H), 2.79 – 2.71 (m, 2H), 2.68 – 2.58 (m, 2H), 2.36 (s, 3H). **^{13}C NMR (126 MHz, $CDCl_3$)** δ 137.2, 136.1, 133.7 (q, *J* = 3.3 Hz), 129.4, 128.3, 121.9 (q, *J* = 37.3 Hz), 120.5 (q, *J* = 271.4 Hz), 33.3, 29.9, 21.1. **^{19}F NMR (471 MHz, $CDCl_3$)** δ -65.02, -71.68. **HRMS (EI)** calcd for: $C_{12}H_{12}ClF_3$ 248.0580, found: 248.0572.

1-(4-chloro-5,5,5-trifluoropent-3-en-1-yl)-4-methoxybenzene (1c)

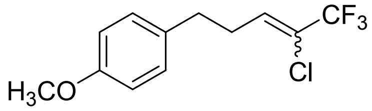

The reaction was performed following the **procedure B**. The residue was purified by flash column chromatograph (Rf = 0.6, silica gel, PE: EtOAc = 20:1) to give the product as a colorless liquid (1.40 g, 53% yield). Z/E = 6.1:1, determined by ^{1}H NMR. **^{1}H NMR (500 MHz, $CDCl_3$)** δ 7.12 (d, *J* = 8.6 Hz, 2H), 6.87 (d, *J* = 8.7 Hz, 2H), 6.49 (t, *J* = 7.1 Hz, 0.86 x 1H), 6.21 (t, *J* = 7.9 Hz, 0.14 x 1H), 3.81 (s, 3H), 2.74 (t, *J* = 7.6 Hz, 2H), 2.62 - 2.57 (m, 2H). **^{13}C NMR (126 MHz, $CDCl_3$)** δ 158.3,133.7 (d, *J* = 3.5 Hz), 132.3, 129.4, 121.9 (q, *J* = 37.3 Hz), 120.5 (q, *J* = 271.7 Hz), 114.1, 55.4, 32.9, 30.0. **^{19}F NMR (471 MHz, $CDCl_3$)** δ -62.21, -68.87. **HRMS (EI)** calcd for $C_{12}H_{12}ClF_3O$: 264.0529, found: 264.0537.

1-(4-chloro-5,5,5-trifluoropent-3-en-1-yl)-4-(trifluoromethyl)benzene (1d)

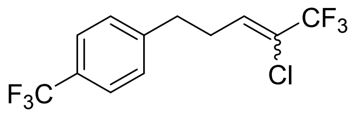

The reaction was performed following the **procedure B**. The residue was purified by flash column chromatograph (Rf = 0.85, silica gel, PE) to give the product as a colorless liquid (1.27 g, 42% yield). Z/E = 6.7:1, determined by ^{1}H NMR. **^{1}H NMR (500 MHz, CDCl$_3$)** δ 7.57 (d, *J* = 8.0 Hz, 2H), 7.31 (d, *J* = 8.0 Hz, 2H), 6.48 (t, J = 7.2 Hz, 0.87 x 1H), 6.19 (t, *J* = 8.1 Hz, 0.13 x 1H), 2.89 – 2.79 (m, 2H), 2.72 – 2.60 (m, 2H). **^{13}C NMR (126 MHz, CDCl$_3$)** δ 144.3, 137.4, 132.9 (q, *J* = 3.6 Hz), 129.1 (q, *J* = 32.5 Hz), 128.8, 125.7 (q, *J* = 4.3 Hz), 122.7 (q, *J* = 37.4 Hz), 120.4 (q, *J* = 271.6 Hz), 33.6, 29.4. **^{19}F NMR (471 MHz, CDCl$_3$)** δ -65.11, -65.19, -71.82. **HRMS (EI)** calcd for $C_{12}H_9ClF_6$: 302.0297, found: 302.0295.

1-(4-chloro-5,5,5-trifluoropent-3-en-1-yl)-4-fluorobenzene (1e)

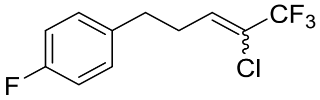

The reaction was performed following the **procedure B**. The residue was purified by flash column chromatograph (Rf = 0.75, silica gel, PE) to give the product as a colorless liquid (1.41 g, 56% yield). Z/E = 4:1, determined by ^{1}H NMR. **^{1}H NMR (500 MHz, CDCl$_3$)** δ 7.21 – 7.11 (m, 2H), 7.05 – 6.96 (m, 2H), 6.48 (t, *J* = 7.2 Hz, 0.8 x 1H), 6.20 (t, *J* = 8.0 Hz, 0.2 x 1H), 2.77 (t, *J* = 7.7 Hz, 2H), 2.61 (q, *J* = 7.7, 7.2 Hz, 2H). **^{13}C NMR (126 MHz, CDCl$_3$)** δ 161.8 (d, *J* = 244.5 Hz), 135.9 (d, *J* = 2.4 Hz), 133.4 (q, *J* = 3.8 Hz), 129.9 (d, *J* = 7.8 Hz), 122.3 (q, *J* = 37.5 Hz), 120.5 (q, *J* = 271.5 Hz), 115.5 (d, *J* = 21.1 Hz), 33.0, 29.9. **^{19}F NMR (471 MHz, CDCl$_3$)** δ -62.23, -68.93, -116.61. **HRMS (EI)** calcd for $C_{11}H_9ClF_4$: 252.0329, found: 252.0324.

1-chloro-4-(4-chloro-5,5,5-trifluoropent-3-en-1-yl)benzene (1f)

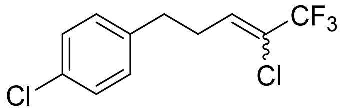

The reaction was performed following the **procedure B**. The residue was purified by flash column chromatograph (Rf = 0.75, silica gel, PE) to give the product as a colorless liquid (1.53 g, 57% yield). Z/E = 6.1:1, determined by ^{1}H NMR. **^{1}H NMR (500 MHz, CDCl$_3$)** δ 7.28 (d, *J* = 8.4 Hz, 2H), 7.12 (d, *J* = 8.5 Hz, 2H), 6.46 (t, *J* = 7.2 Hz, 0.86 x 1H), 6.18 (t, *J* = 8.0 Hz, 1H), 2.79 – 2.70 (m, 2H), 2.68 – 2.55 (m, 2H). **^{13}C NMR (126 MHz, CDCl$_3$)** δ 138.7, 133.1 (q, *J* = 3.7 Hz), 132.4, 129.8, 128.9, 122.4 (q, *J* = 37.8), 120.4 (q, *J* = 271.6 Hz), 33.1, 29.6. **^{19}F NMR (471 MHz, CDCl$_3$)** δ -65.06, -71.76. **HRMS (EI)** calcd for $C_{11}H_9Cl_2F_3$: 268.0033, found: 268.0028.

4-(4-chloro-5,5,5-trifluoropent-3-en-1-yl)phenyl acetate (1g)

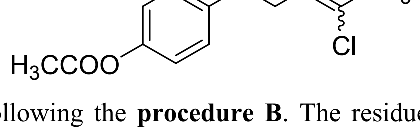

The reaction was performed following the **procedure B**. The residue was purified by flash column chromatograph (Rf = 0.6, silica gel, PE: EtOAc = 10:1) to give the product as a colorless liquid (1.40 g,

48% yield). Z/E = 9:1, determined by ^{1}H NMR. **^{1}H NMR (500 MHz, $CDCl_3$)** δ 7.21 (d, *J* = 8.5 Hz, 2H), 7.04 (d, *J* = 8.5 Hz, 2H), 6.50 (t, *J* = 7.0 Hz, 0.9 x 1H), 6.22 (t, *J* = 8.0 Hz, 0.1 x 1H), 2.80 – 2.74 (m, 2H), 2.61 (q, *J* = 7.2, 7.8 Hz, 2H), 2.30 (s, 3H). **^{13}C NMR (126 MHz, $CDCl_3$)** δ 169.7, 149.3, 137.8, 133.4 (q, *J* = 3.8 Hz), 129.3, 122.1 (q, *J* = 37.3 Hz), 121.8, 120.4 (q, *J* = 271.4 Hz), 33.1, 29.7, 21.1. **^{19}F NMR (471 MHz, $CDCl_3$)** δ -62.22, -68.88. **HRMS (ESI)** calcd for $C_{13}H_{13}ClF_3O_2$ $[M+H]^+$: 293.0551, found: 293.0550.

1-bromo-2-(4-chloro-5,5,5-trifluoropent-3-en-1-yl)benzene (1h)

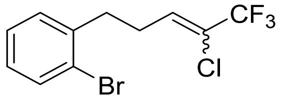

The reaction was performed following the **procedure B**. The residue was purified by flash column chromatograph (Rf = 0.75, silica gel, PE) to give the product as a colorless liquid (1.93 g, 62% yield). Z/E = 6.1:1, determined by ^{1}H NMR. **^{1}H NMR (500 MHz, $CDCl_3$)** δ 7.53 (d, *J* = 8.0 Hz, 1H), 7.23 (t, *J* = 7.4 Hz, 1H), 7.18 (d, *J* = 5.9 Hz, 1H), 7.07 (td, *J* = 7.7, 1.8 Hz, 1H), 6.50 (t, *J* = 7.3 Hz, 0.86 x 1H), 6.22 (t, *J* = 8.1 Hz, 0.14 x 1H), 2.90 – 2.86 (m, 2H), 2.64 – 2.59 (m, 2H). **^{19}F NMR (471 MHz, $CDCl_3$)** δ -65.16, -71.90. **^{13}C NMR (126 MHz, $CDCl_3$)** δ 139.5, 137.7, 133.2, 130.5, 128.4, 127.8, 124.5, 122.4 (q, *J* = 37.4 Hz), 120.4 (q, *J* = 271.5 Hz), 34.1, 28.2. **HRMS (EI)** calcd for $C_{11}H_9BrClF_3$: 311.9528, found: 311.9520.

1-bromo-3-(4-chloro-5,5,5-trifluoropent-3-en-1-yl)benzene (1i)

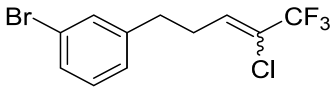

The reaction was performed following the **procedure B**. The residue was purified by flash column chromatograph (Rf = 0.75, silica gel, PE) to give the product as a colorless liquid (1.93 g, 62% yield). Z/E = 6.7:1, determined by ^{1}H NMR. **^{1}H NMR (500 MHz, $CDCl_3$)** δ 7.38 (d, *J* = 8.9 Hz, 2H), 7.19 (t, *J* = 7.7 Hz, 1H), 7.13 (d, *J* = 7.7 Hz, 1H), 6.48 (t, *J* = 7.2 Hz, 0.87 x 1H), 6.20 (t, *J* = 8.0 Hz, 0.13 x 1H), 2.76 (t, *J* = 7.7 Hz, 2H), 2.71 – 2.56 (m, 2H). **^{13}C NMR (126 MHz, $CDCl_3$)** δ 142.5, 133.0 (q, *J* = 3.8 Hz), 131.5, 130.3, 129.8, 127.1, 122.8, 122.5 (q, *J* = 37.6 Hz), 120.4 (q, *J* = 271.5 Hz), 33.4, 29.5. **^{19}F NMR (471 MHz, $CDCl_3$)** δ -64.77, -71.47. **HRMS (EI)** calcd for $C_{11}H_9BrClF_3$: 311.9528, found: 311.9518.

1-bromo-4-(4-chloro-5,5,5-trifluoropent-3-en-1-yl)benzene (1j)

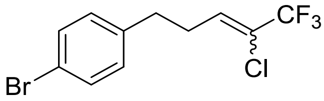

The reaction was performed following the **procedure B**. The residue was purified by flash column chromatograph (Rf = 0.75, silica gel, PE) to give the product as a colorless liquid (1.93 g, 62% yield). Z/E = 6.1:1, determined by ^{1}H NMR. **^{1}H NMR (500 MHz, $CDCl_3$)** δ 7.43 (d, *J* = 8.4 Hz, 2H), 7.06 (d, *J* = 8.6 Hz, 2H), 6.45 (t, *J* = 7.2 Hz, 0.86 x 1H), 6.17 (t, *J* = 8.2 Hz, 0.14 x 1H), 2.74 (t, *J* = 7.7 Hz, 2H), 2.62 – 2.57 (m, 2H). **^{13}C NMR (126 MHz, $CDCl_3$)** δ 139.2, 133.1 (q, *J* = 3.6 Hz), 131.8, 130.2, 122.4 (q, *J* = 37.3 Hz), 120.4, 120.4 (q, *J* = 271.6 Hz), 33.2, 29.5. **^{19}F NMR (471 MHz, $CDCl_3$)** δ -65.04, -71.75. **HRMS (ESI)** calcd for $C_{11}H_{10}BrClF_3$ $[M+H]^+$: 312.9601, found: 312.9600.

(3-chloro-4,4,4-trifluorobut-2-en-1-yl)benzene (1k)

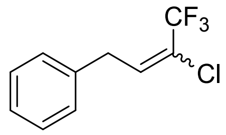

The reaction was performed following the **procedure B**. The residue was purified by flash column chromatograph (Rf = 0.8, silica gel, PE) to give the product as a colorless liquid (1.52 g, 69% yield). Z/E = 6.1:1, determined by ^{1}H NMR. **^{1}H NMR (500 MHz, CDCl$_3$)** δ 7.36 (t, *J* = 7.7 Hz, 2H), 7.29 (t, *J* = 7.3 Hz, 1H), 7.22 (d, *J* = 7.9 Hz, 2H), 6.67 (t, *J* = 7.3 Hz, 0.86 x 1H), 6.37 (t, *J* = 8.3 Hz, 0.14 x 1H), 3.66 (d, *J* = 7.2 Hz, 2H). **^{13}C NMR (126 MHz, CDCl$_3$)** δ 137.0, 133.3 (q, *J* = 4.2 Hz), 129.1, 128.6, 127.2, 122.0 (q, *J* = 37.6 Hz), 120.5 (q, *J* = 271.6 Hz), 34.3. **^{19}F NMR (471 MHz, CDCl$_3$)** δ -61.53, -68.82. **HRMS (ESI)** calcd for $C_{10}H_9ClF_3$ [M+H]$^+$: 221.0339, found: 221.0342.

1-(3-chloro-4,4,4-trifluorobut-2-en-1-yl)-4-methoxybenzene (1l)

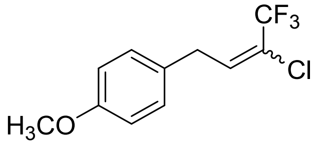

The reaction was performed following the **procedure B**. The residue was purified by flash column chromatograph (Rf = 0.65, silica gel, PE: EtOAc = 10:1) to give the product as a colorless liquid (1.40 g, 56% yield). Z/E = 9:1, determined by ^{1}H NMR. **^{1}H NMR (500 MHz, CDCl$_3$)** δ 7.15 (dd, *J* = 6.6, 2.0 Hz, 2H), 6.912 -6.89 (m, 2H), 6.66 (t, *J* = 7.5 Hz, 0.9 x 1H), 6.36 (t, *J* = 8.2 Hz, 0.1 x 1H), 3.83 (s, 3H), 3.61 (d, *J* = 7.1 Hz, 2H). **^{13}C NMR (126 MHz, CDCl$_3$)** δ 158.8, 133.6 (q, *J* = 3.6 Hz), 129.6, 129.0, 121.5 (q, *J* = 37.4 Hz), 120.5 (q, *J* = 271.4 Hz), 114.4, 55.4, 33.4. **^{19}F NMR (471 MHz, CDCl$_3$)** δ -64.12, -71.47. **HRMS (EI)** calcd for $C_{11}H_{10}ClF_3O$: 250.0372, found: 250.0362.

1-bromo-4-(3-chloro-4,4,4-trifluorobut-2-en-1-yl)benzene (1m)

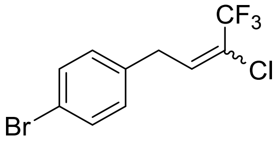

The reaction was performed following the **procedure B**. The residue was purified by flash column chromatograph (Rf = 0.75, silica gel, PE) to give the product as a colorless liquid (1.82 g, 61% yield). Z/E = 6.7:1, determined by ^{1}H NMR. **^{1}H NMR (500 MHz, CDCl$_3$)** δ 7.46 (d, *J* = 8.4 Hz, 2H), 7.08 (d, *J* = 8.4 Hz, 2H), 6.61 (t, *J* = 7.4 Hz, 0.87 x 1H), 6.31 (t, *J* = 8.2 Hz, 0.13 x 1H), 3.60 (d, *J* = 7.3 Hz, 1H). **^{13}C NMR (126 MHz, CDCl$_3$)** δ 136.0, 132.5 (q, *J* = 4.2 Hz), 132.1, 130.3, 122.5 (q, *J* = 37.4 Hz), 121.1, 120.4 (q, *J* = 271.6 Hz), 33.7. **^{19}F NMR (471 MHz, CDCl$_3$)** δ -61.57, -68.87. **HRMS (ESI)** calcd for $C_{10}H_8BrClF_3$ [M+H]$^+$: 298.9445, found: 298.9447.

(5-chloro-6,6,6-trifluorohex-4-en-1-yl)benzene (1n)

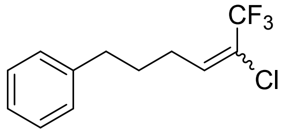

The reaction was performed following the **procedure B**. The residue was purified by flash column chromatograph (Rf = 0.8, silica gel, PE) to give the product as a colorless liquid (1.82 g, 69% yield). Z/E = 7.3:1, determined by ^{1}H NMR. **^{1}H NMR (500 MHz, CDCl$_3$)** δ 7.36 (t, J = 7.5 Hz, 2H), 7.26 (dd, J = 16.3, 7.2 Hz, 3H), 6.53 (t, J = 7.3 Hz, 0.88 x 1H), 6.25 (t, J = 8.1 Hz, 0.12 x 1H), 2.72 (t, J = 7.8 Hz, 2H), 2.41 -2.36 (m, 2H), 1.90 -1.80 (m, 2H). **^{13}C NMR (126 MHz, CDCl$_3$)** δ 141.4, 134.3 (q, J = 3.8 Hz), 128.6, 128.5, 126.2, 121.7 (q, J = 37.3 Hz), 120.5 (q, J = 271.2 Hz), 35.4, 29.4, 27.6. **^{19}F NMR (471 MHz, CDCl$_3$)** δ -64.71, -71.43. **HRMS (EI)** calcd for $C1_2H_{12}ClF_3$: 248.0580, found: 248.0570.

(4-chloro-5,5,5-trifluoropent-3-ene-1,1-diyl)dibenzene (1o)

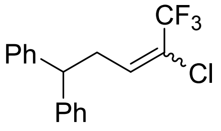

The reaction was performed following the **procedure B**. The residue was purified by flash column chromatograph (Rf = 0.75, silica gel, PE) to give the product as a colorless liquid (2.05 g, 66% yield). Z/E = 8.1:1, determined by ^{1}H NMR. **^{1}H NMR (500 MHz, CDCl$_3$)** δ 7.34 – 7.29 (m, 4H), 7.25 – 7.20 (m, 6H), 6.38 (t, J = 6.9 Hz, 0.89 x 1H), 6.09 (t, J = 7.4 Hz, 0.11 x 1H), 4.12 (t, J = 8.1 Hz, 1H), 3.13 – 3.00 (m, 2H).**^{13}C NMR (126 MHz, CDCl$_3$)** δ 143.2, 132.9 (q, J = 3.9 Hz), 128.9, 127.8, 126.9, 122.4 (q, J = 37.4 Hz), 120.3 (q, J = 271.6 Hz), 49.6, 34.2. **^{19}F NMR (471 MHz, CDCl$_3$)** δ -62.22, -68.88. **HRMS (ESI)** calcd for $C_{17}H_{14}ClF_3$ [M+H]+: 311.0809, found: 311.0813.

1-(3-chloro-4,4,4-trifluorobut-2-en-1-yl)naphthalene (1p)

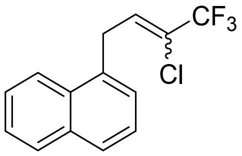

The reaction was performed following the **procedure B**. The residue was purified by flash column chromatograph (Rf = 0.65, silica gel, PE: EtOAc=20:1) to give the product as a colorless liquid (1.62 g, 60% yield). Z/E = 6.1:1, determined by ^{1}H NMR. **^{1}H NMR (600 MHz, CDCl$_3$)** δ 7.96 (t, J = 8.0 Hz, 2H), 7.86 (d, J = 8.2 Hz, 1H), 7.65 -7.58 (m, 2H), 7.49 (dd, J = 7.0, 8.3 Hz, 1H), 7.40 (d, J = 6.9 Hz, 1H), 6.77 (t, J = 7.0 Hz, 0.86 x 1H), 6.47 (t, J = 7.7 Hz, 0.11 x 1H), 4.10 (d, J = 7.0 Hz, 2H). **^{13}C NMR (126 MHz, CDCl$_3$)** δ 134.1, 133.4 (q, J = 3.9 Hz), 133.2, 131.8, 129.1, 128.1, 126.7, 126.7, 126.1, 125.8, 123.4, 122.0 (q, J = 37.6 Hz), 120.5 (q, J = 271.6 Hz), 32.0.**^{19}F NMR (471 MHz, CDCl$_3$)** δ -64.62, -71.42. **HRMS (EI)** calcd for $C_{14}H_{10}ClF_3$: 270.0423, found: 270.0416.

4-(3-chloro-4,4,4-trifluorobut-2-en-1-yl)-1,1'-biphenyl (1q)

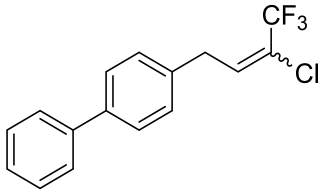

The reaction was performed following the **procedure B**. The residue was purified by flash column chromatograph (Rf = 0.75, silica gel, PE: EtOAc=20:1) to give the product as a colorless liquid (1.92 g, 65% yield). Z/E = 9:1, determined by ^{1}H NMR. **^{1}H NMR (500 MHz, Chloroform-*d*)** δ 7.58 – 7.52 (m,

4H), 7.43 (t, J = 7.7 Hz, 2H), 7.34 (t, J = 7.5 Hz, 1H), 7.25 (d, J = 8.3 Hz, 2H), 6.66 (t, J = 7.4 Hz, 0.9 x 1H), 6.36 (t, J = 8.1 Hz, 0.1 x 1H), 3.66 (d, J = 7.4 Hz, 2H). **^{13}C NMR (126 MHz, CDCl$_3$)** δ 140.8, 140.2, 136.0, 133.1 (q, J = 3.9 Hz), 129.0, 128.9, 127.8, 127.5, 127.2, 122.1 (q, J = 37.6 Hz), 120.5 (q, J = 271.6 Hz), 33.9. **^{19}F NMR (471 MHz, CDCl$_3$)** δ -61.44, -68.75. **HRMS (ESI)** calcd for $C_{16}H_{13}ClF_3[M+H]^+$:: 297.0652, found: 297.0650.

5-(3-chloro-4,4,4-trifluorobut-2-en-1-yl)benzo[d][1,3]dioxole (1r)

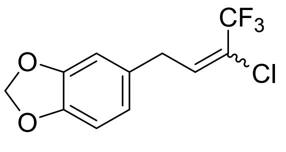

The reaction was performed following the **procedure B**. The residue was purified by flash column chromatograph (Rf = 0.45, silica gel, PE: EtOAc=20:1) to give the product as a colorless liquid (1.11 g, 42% yield). Z/E = 8.1:1, determined by ^{1}H NMR. **^{1}H NMR (500 MHz, CDCl$_3$)** δ 6.76 (d, J = 7.9 Hz, 1H), 6.69 – 6.62 (m, 2H), 6.59 (t, J = 7.9 Hz, 0.89 x 1H), 6.30 (t, J = 8.3 Hz, 0.11 x 1H), 5.95 (s, 2H), 3.55 (d, 7.3 Hz, 2H). **^{13}C NMR (126 MHz, CDCl$_3$)** δ 148.2, 146.8, 133.4 (q, J = 4.0 Hz), 130.6, 121.8 (q, J = 37.9 Hz), 121.6, 120.5 (q, J = 271.6 Hz), 109.0, 108.7, 101.2, 34.0. **^{19}F NMR (471 MHz, CDCl$_3$)** δ -62.93, -70.31. **HRMS (EI)** calcd for $C_{11}H_8ClF_3O_2$: 264.0165, found: 264.0175.

((4-chloro-5,5,5-trifluoropent-3-en-1-yl)oxy)benzene (1s)

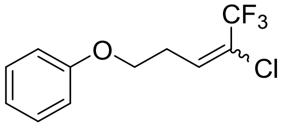

The reaction was performed following the **procedure B**. The residue was purified by flash column chromatograph (Rf = 0.6, silica gel, PE: EtOAc=10:1) to give the product as a colorless liquid (1.20 g, 48% yield). Z/E = 5.3:1, determined by ^{1}H NMR. **^{1}H NMR (500 MHz, CDCl$_3$)** δ 7.31 (dd, J = 8.5, 7.5 Hz, 2H), 6.99 (t, J = 7.4 Hz, 1H), 6.91 (d, J = 7.9 Hz, 2H), 6.68 (t, J = 6.9 Hz, 0.84 x 1H), 6.40 (t, J = 7.7 Hz, 0.16 x 1H), 4.09 (t, J = 6.2 Hz, 2H), 2.82 – 2.77 (m, 2H).**^{13}C NMR (126 MHz, CDCl$_3$)** δ 158.5, 131.1 (q, J = 4.5 Hz), 129.7, 123.2 (q, J = 37.3 Hz), 121.5, 120.6 (q, J = 296.8 Hz), 114.6, 65.2, 28.5. **^{19}F NMR (471 MHz, CDCl$_3$)** δ -65.28, -71.86. **HRMS (EI)** calcd for $C_{11}H_{10}ClF_3O$: 250.0372, found: 250.0362.

4-(4-chloro-5,5,5-trifluoropent-3-en-1-yl)-1,2-dimethoxybenzene (1t)

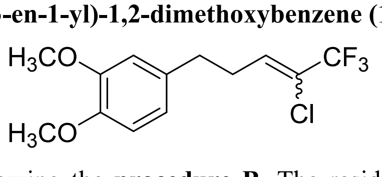

The reaction was performed following the **procedure B**. The residue was purified by flash column chromatograph (Rf = 0.5, silica gel, PE: EtOAc=10:1) to give the product as a colorless liquid (1.32 g, 45% yield). Z/E = 6.1:1, determined by ^{1}H NMR. **^{1}H NMR (500 MHz, Chloroform-*d*)** δ 6.80 (d, J = 8.1 Hz, 1H), 6.70 (q, J = 8.5 Hz, 2H), 6.47 (t, J = 7.1 Hz, 0.86 x 1H), 6.19 (t, J = 7.8 Hz, 0.14 x 1H), 3.85 (d, J = 7.5 Hz, 6H), 2.72 (t, J = 7.6 Hz, 2H), 2.61 -2.56 (m, 2H). **^{13}C NMR (126 MHz, Chloroform-*d*)** δ 148.9, 147.6, 133.7 (q, J = 3.6 Hz), 132.7, 121.7 (q, J = 37.4 Hz), 120.3 (q, J = 271.6 Hz), 120.2, 111.5, 111.3, 55.8, 55.7, 33.2, 29.8. **^{19}F NMR (471 MHz, CDCl$_3$)** δ -62.22, -68.89. **HRMS (EI)** calcd for

$C_{13}H_{14}ClF_3O_2$: 294.0634, found: 294.0629.

2-(4-chloro-5,5,5-trifluoropent-3-en-1-yl)-5-methylfuran (1u)

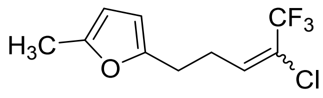

The reaction was performed following the **procedure B**. The residue was purified by flash column chromatograph (Rf = 0.6, silica gel, PE: EtOAc = 20:1) to give the product as a colorless liquid (1.09 g, 46% yield). Z/E = 5.3:1, determined by ^{1}H NMR. **^{1}H NMR (500 MHz, CDCl$_3$)** δ 6.50 (t, *J* = 7.5 Hz, 0.84 x 1H), 6.21 (t, *J* = 7.6 Hz, 0.16 x 1H), 5.88 (d, *J* = 17.7 Hz, 2H), 2.75 (t, *J* = 7.3 Hz, 2H), 2.70 – 2.59 (m, 2H), 2.26 (s, 3H).**^{13}C NMR (126 MHz, CDCl$_3$)** δ 152.0, 151.1, 133.5 (q, *J* = 3.9 Hz), 122.1 (q, *J* = 37.4 Hz), 120.5 (q, *J* = 271.3 Hz), 106.5, 106.1, 26.9, 26.2, 13.6. **^{19}F NMR (471 MHz, CDCl$_3$)** δ -62.35, -68.94. **HRMS (EI)** calcd for $C_{10}H_{10}ClF_3O$: 238.0372, found: 238.0368.

2-(5-chloro-6,6,6-trifluorohex-4-en-1-yl)thiophene (1v)

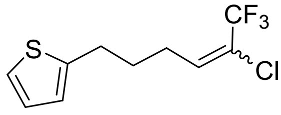

The reaction was performed following the **procedure B**. The residue was purified by flash column chromatograph (Rf = 0.6, silica gel, PE: EtOAc=20:1) to give the product as a colorless liquid (1.07 g, 42% yield). Z/E = 6.7:1, determined by ^{1}H NMR. **^{1}H NMR (500 MHz, CDCl$_3$)**δ 7.15 (d, *J* = 5.1 Hz, 1H), 6.95 (dd, *J* = 5.1, 3.4 Hz, 1H), 6.82 (d, *J* = 3.4 Hz, 1H), 6.49 (t, *J* = 7.3 Hz, 0.87 x 1H), 6.22 (t, *J* = 8.2 Hz, 0.13 x 1H), 2.90 (t, *J* = 7.5 Hz, 2H), 2.47 – 2.32 (m, 2H), 1.93 – 1.81 (m, 2H).**^{13}C NMR (126 MHz, CDCl$_3$)** δ 144.1, 133.9 (q, *J* = 3.6 Hz), 127.0, 124.7, 123.4, 122.0 (q, *J* = 37.2 Hz), 120.5 (q, *J* = 271.3 Hz), 29.7, 29.5, 27.4. **^{19}F NMR (471 MHz, CDCl$_3$)** δ -62.08, -68.83. **HRMS (EI)** calcd for $C_{10}H_{10}ClF_3S$: 254.0144, found: 254.0141.

tert-butyl 4-(2-chloro-3,3,3-trifluoroprop-1-en-1-yl)piperidine-1-carboxylate (1w)

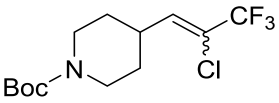

The reaction was performed following the **procedure B**. The residue was purified by flash column chromatograph (Rf = 0.4, silica gel, PE: EtOAc=4:1) to give the product as a yellow solid (1.06 g, 34% yield, mp: 73.6-78.4 °C). Z/E = 4.6:1, determined by ^{1}H NMR. **^{1}H NMR (500 MHz, CDCl$_3$)** δ 6.26 (d, *J* = 9.0 Hz, 0.82 x 1H), 5.99 (d, *J* = 10.5 Hz, 0.18 x 1H), 4.09 (s, 2H), 2.90 – 2.58 (m, 3H), 1.67 (t, *J* = 14.2 Hz, 2H), 1.44 (s, 9H), 1.33 (q, *J* = 12.5 Hz, 2H).**^{13}C NMR (126 MHz, CDCl$_3$)** δ 154.8, 137.2 (q, *J* = 3.5 Hz), 121.0 (q, *J* = 37.4 Hz), 120.4 (q, *J* = 271.6 Hz), 79.8, 35.8, 31.5, 30.0, 28.5.**^{19}F NMR (471 MHz, CDCl$_3$)** δ -64.60, -71.61. HRMS **(EI)** calcd for $C_{13}H_{19}ClF_3NO_2$: 313.1056, found: 313.1061.

benzyl 4-(2-chloro-3,3,3-trifluoroprop-1-en-1-yl)piperidine-1-carboxylate (1x)

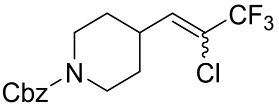

The reaction was performed following the **procedure B**. The residue was purified by flash column

chromatograph (Rf= 0.4, silica gel, PE: EtOAc=4:1) to give the product as a colorless liquid (1.28 g, 37% yield). Z/E = 8.1:1, determined by ^{1}H NMR. **^{1}H NMR (500 MHz, $CDCl_3$)** δ 7.37 (d, *J* = 4.4 Hz, 4H), 7.33 (dt, *J* = 4.5, 9.1 Hz, 1H), 6.28 (d, *J* = 9.1 Hz, 0.89 x 1H), 6.00 (d, *J* = 10.6 Hz, 0.11 x 1H), 5.14 (s, 2H), 4.21 (s, 2H), 2.89 (s, 2H), 2.77 – 2.68 (m, 1H), 1.72 (d, *J* = 10.6 Hz, 2H), 1.38 (d, *J* = 10.9 Hz, 2H).**^{13}C NMR (126 MHz, $CDCl_3$)** δ 155.2, 137.0, 136.8, 128.6, 128.1, 128.0, 121.2 (q, *J* = 37.7 Hz), 120.3 (q, *J* = 271.5 Hz), 67.2, 43.4, 35.6, 29.9. **^{19}F NMR (471 MHz, $CDCl_3$)** δ -61.93, -68.94. HRMS **(ESI)** calcd for $C_{16}H_{18}ClF_3NO_2$ $[M+H]^+$: 348.0973, found: 348.0978.

4-(3-chloro-4,4,4-trifluorobut-2-en-1-yl)-1-tosylpiperidine (1y)

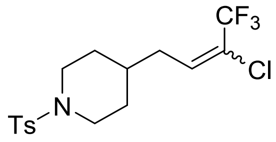

The reaction was performed following the **procedure B**. The residue was purified by flash column chromatograph (Rf= 0.35, silica gel, PE: EtOAc=4:1) to give the product as a white solid (1.37 g, 36% yield, mp: 41.7-45.7 °C). Z/E = 5.3:1, determined by ^{1}H NMR. **^{1}H NMR (500 MHz, $CDCl_3$)** δ 7.63 (d, *J* = 8.2 Hz, 2H), 7.32 (d, *J* = 8.1 Hz, 2H), 6.42 (t, *J* = 7.4 Hz, 0.84 x 1H), 6.14 (t, *J* = 8.4 Hz, 0.16 x 1H), 3.78 (d, *J* = 11.9 Hz, 2H), 2.43 (s, 3H), 2.31 – 2.17 (m, 4H), 1.82 – 1.66 (m, 2H), 1.46 – 1.38 (m, 2H), 1.35 (d, *J* = 14.9 Hz, 1H).**^{13}C NMR (126 MHz, $CDCl_3$)** δ 143.6, 133.1, 132.0 (d, *J* = 3.6 Hz), 129.7, 127.7, 122.8 (q, *J* = 37.4 Hz), 120.3 (q, *J* = 271.4 Hz), 46.2, 34.6, 34.1, 31.1, 21.5. **^{19}F NMR (471 MHz, $CDCl_3$)** δ -63.46, -70.49. **HRMS (EI)** calcd for $C_{16}H_{19}ClF_3NO_2S$: 381.0777, found: 381.0769.

4-(3-chloro-4,4,4-trifluorobut-2-en-1-yl)tetrahydro-2H-pyran (1z)

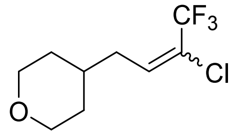

The reaction was performed following the **procedure B**. The residue was purified by flash column chromatograph (Rf= 0.6, silica gel, PE: EtOAc=10:1) to give the product as a colorless liquid (0.94 g, 41% yield). Z/E = 4:1, determined by ^{1}H NMR. **^{1}H NMR (400 MHz, $CDCl_3$)** δ 6.48 (t, *J* = 7.4 Hz, 0.79 x 1H), 6.20 (t, *J* = 8.3 Hz, 0.21 x 1H), 3.96 (dd, *J* = 11.2, 4.0 Hz, 2H), 3.36 (t, *J* = 11.8 Hz, 2H), 2.35 – 2.20 (m, 2H), 1.77 – 1.63 (m, 1H), 1.66 – 1.55 (m, 2H), 1.42 -1.31 (m, 2H).**^{13}C NMR (126 MHz, $CDCl_3$)** δ 132.3 (q, *J* = 3.7 Hz), 122.7 (q, *J* = 37.3 Hz), 120.4 (q, *J* = 271.4 Hz), 67.9, 35.0, 34.4, 32.7. **^{19}F NMR (376 MHz, $CDCl_3$)** δ -61.89, -68.92. **HRMS (EI)** calcd for $C_9H_{12}ClF_3O$: 228.0529, found: 228.0536.

2-chloro-1,1,1-trifluorododec-2-ene (1aa)

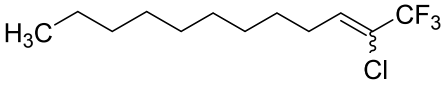

The reaction was performed following the **procedure B**. The residue was purified by flash column chromatograph (Rf= 0.85, silica gel, PE: EtOAc=20:1) to give the product as a colorless liquid (1.79 g, 70% yield). Z/E = 7.3:1, determined by ^{1}H NMR. **^{1}H NMR (500 MHz, $CDCl_3$)** δ 6.47 (t, *J* = 7.3 Hz, 0.88 x 1H), 6.19 (t, *J* = 8.2 Hz, 0.12 x 1H), 2.38 – 2.23 (m, 2H), 1.50 -1.46 (m 2H), 1.39 – 1.21 (m, 12H), 0.89 (t, *J* = 6.9 Hz, 3H).**^{13}C NMR (126 MHz, $CDCl_3$)** δ 134.8 (q, *J* = 3.8 Hz), 121.4 (q, *J* = 37.0 Hz),

120.6 (q, J = 271.2 Hz), 32.1, 29.6, 29.5, 29.5, 29.3, 28.1, 27.8, 22.9, 14.2. **^{19}F NMR (471 MHz, $CDCl_3$)** δ -65.02, -71.81. **HRMS (EI)** calcd for $C_{12}H_{20}ClF_3$: 256.1206, found: 256.1203

ethyl 7-chloro-8,8,8-trifluorooct-6-enoate (1ab)

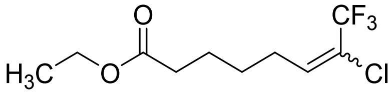

The reaction was performed following the **procedure B**. The residue was purified by flash column chromatograph (Rf = 0.65, silica gel, PE: EtOAc=10:1) to give the product as a colorless liquid (1.29 g, 50% yield). Z/E = 6.1:1, determined by ^{1}H NMR. **^{1}H NMR (500 MHz, $CDCl_3$)** δ 6.44 (t, J = 7.3 Hz, 0.86 x 1H), 6.17 (t, J = 8.2 Hz, 0.14 x 1H), 4.11 (q, J = 7.1 Hz, 2H), 2.32 -2.28 (m, 4H), 1.69 -1.63 (m, 2H), 1.53 – 1.45 (m, 2H), 1.24 (t, J = 7.2 Hz, 3H). **^{13}C NMR (126 MHz, $CDCl_3$)** δ 173.4, 134.1 (q, J = 3.9 Hz), 121.8 (q, J = 37.3 Hz), 120.5 (q, J = 271.3 Hz), 60.5, 34.0, 27.7, 27.2, 24.5, 14.3. **^{19}F NMR (471 MHz, $CDCl_3$)** δ -62.15, -68.96. **HRMS (ESI)** calcd for $C_{10}H_{15}ClF_3O_2$ $[M+H]^+$: 259.0707, found: 259.0709.

tert-butyl((4-chloro-5,5,5-trifluoropent-3-en-1-yl)oxy)dimethylsilane (1ac)

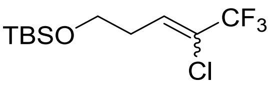

The reaction was performed following the **procedure B**. The residue was purified by flash column chromatograph (Rf = 0.7, silica gel, PE: EtOAc=20:1) to give the product as a colorless liquid (0.86 g, 30% yield). Z/E = 5.7:1, determined by ^{1}H NMR. **^{1}H NMR (500 MHz, $CDCl_3$)** δ 6.58 (t, J = 7.0 Hz, 0.85 x 1H), 6.30 (t, J = 7.8 Hz, 0.15 x 1H), 3.73 (t, J = 6.2 Hz, 2H), 2.59 – 2.48 (m, 2H), 0.89 (s, 9H), 0.06 (s, 6H). **^{13}C NMR (126 MHz, $CDCl_3$)** δ 132.1 (q, J = 3.7 Hz), 122.5 (q, J = 37.3 Hz), 120.5 (q, J = 271.2 Hz), 60.7, 31.8, 25.9, 18.4, -5.3. **^{19}F NMR (471 MHz, $CDCl_3$)** δ -71.87, -78.59. **HRMS (ESI)** calcd for $C_{11}H_{21}ClF_3OSi$ $[M+H]^+$: 289.0997, found: 289.0992.

10-bromo-2-chloro-1,1,1-trifluorodec-2-ene (1ad)

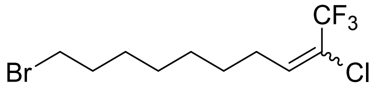

The reaction was performed following the **procedure B**. The residue was purified by flash column chromatograph (Rf = 0.8, silica gel, PE: EtOAc=20:1) to give the product as a colorless liquid (1.87 g, 61% yield). Z/E = 10.1:1, determined by ^{1}H NMR. **^{1}H NMR (500 MHz, $CDCl_3$)** δ 6.46 (t, J = 7.3 Hz, 0.91 x 1H), 6.19 (t, J = 8.2 Hz, 0.09 x 1H), 3.40 (t, J = 6.8 Hz, 2H), 2.32 – 2.27 (m, 2H), 1.88 -1.83 (m, 2H), 1.46 (tt, J = 7.2, 15.7 Hz, 4H), 1.37 -1.31 (m, 4H). **^{13}C NMR (126 MHz, $CDCl_3$)** δ 134.6 (q, J = 4.0 Hz), 121.5 (q, J = 37.2 Hz), 120.5 (q, J = 271.2 Hz), 34.0, 32.8, 29.1, 28.5, 28.1, 28.0, 27.6. **^{19}F NMR (471 MHz, $CDCl_3$)** δ -62.06, -68.85. **HRMS (ESI)** calcd for $C_{10}H_{16}BrClF_3$ $[M+H]^+$: 307.0071, found: 307.0074.

(4-chloro-5,5,5-trifluoropent-3-en-1-yl)cyclohexane (1ae)

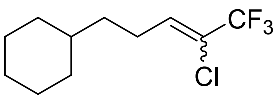

The reaction was performed following the **procedure B**. The residue was purified by flash column chromatograph (Rf = 0.75, silica gel, PE: EtOAc=20:1) to give the product as a colorless liquid (0.60 g, 25% yield). Z/E = 4.9:1, determined by ^{1}H NMR. **^{1}H NMR (500 MHz, CDCl$_3$)** δ 6.47 (t, J = 7.3 Hz, 0.83 x 1H), 6.20 (t, *J* = 8.2 Hz, 0.17 x 1H), 2.41 – 2.27 (m, 2H), 1.82 – 1.62 (m, 5H), 1.41 – 1.32 (m, 2H), 1.32 – 1.12 (m, 4H), 0.99 – 0.86 (m, 2H).**^{13}C NMR (126 MHz, CDCl$_3$)** δ 135.1 (q, *J* = 4.0 Hz), 121.2 (q, *J* = 37.2 Hz), 120.7 (q, *J* = 270.9 Hz), 37.5, 35.4, 33.3, 26.8, 26.5, 25.7. **^{19}F NMR (471 MHz, CDCl$_3$)** δ -62.11, -68.81. **HRMS (EI)** calcd for $C_{11}H_{16}ClF_3$: 240.0893, found: 240.0885.

2-(2-chloro-3,3,3-trifluoroprop-1-en-1-yl)-2,3-dihydro-1H-indene (1af)

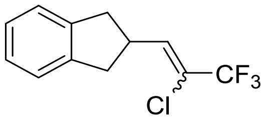

The reaction was performed following the **procedure B**. The residue was purified by flash column chromatograph (Rf = 0.75, silica gel, PE: EtOAc=20:1) to give the product as a white solid (1.55 g, 63% yield, m.p.=38.7-46.6). Z/E = 7.3:1, determined by ^{1}H NMR. **^{1}H NMR (500 MHz, CDCl$_3$)** δ 7.18 (d, *J* = 18.0 Hz, 4H), 6.54 (d, *J* = 9.2 Hz, 0.88 x 1H), 6.28 (d, *J* = 10.7 Hz, 0.12 x 1H), 3.56 (dt, *J* = 15.1, 7.9 Hz, 1H), 3.20 (dd, *J* = 15.6, 8.0 Hz, 2H), 2.77 (dd, *J* = 15.5, 7.0 Hz, 2H).**^{13}C NMR (126 MHz, CDCl$_3$)** δ 141.8, 138.3 (q, *J* = 3.7 Hz), 126.9, 124.7, 121.2 (q, *J* = 37.5 Hz), 120.6 (q, *J* = 271.5 Hz), 39.7, 38.5. **^{19}F NMR (471 MHz, CDCl$_3$)** δ -64.39, -71.53. **HRMS (ESI)** calcd for $C_{12}H_{11}ClF_3$: 247.0496, found: 247.0491.

(1-(2-chloro-3,3,3-trifluoroprop-1-en-1-yl)cyclopropyl)benzene (1ag)

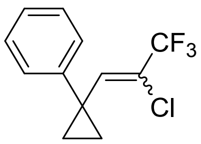

The reaction was performed following the **procedure B**. The residue was purified by flash column chromatograph (Rf = 0.75, silica gel, PE: EtOAc=20:1) to give the product as a colorless liquid (1.43 g, 58% yield). Z/E = 6.7:1, determined by ^{1}H NMR. **^{1}H NMR (500 MHz, Chloroform-*d*)** δ 7.32 – 7.22 (m, 5H), 6.69 (s, 0.13 x 1H), 6.68 (s, 0.87 x 1H), 1.30 -1.28 (m, 2H), 1.26 -1.23 (m, 2H). **^{13}C NMR (126 MHz, CDCl$_3$)** δ 141.7, 136.7 (q, *J* = 4.7 Hz), 128.7, 127.9, 126.9, 123.3 (q, *J* = 36.5 Hz), 120.7 (q, *J* = 271.9 Hz), 24.1, 16.6. **^{19}F NMR (471 MHz, CDCl$_3$)** δ -62.80, -68.83. **HRMS (EI)** calcd for $C_{12}H_{10}ClF_3$: 246.0423, found: 246.0416.

(4-chloro-5,5,5-trifluoropent-3-en-1-yl)cyclopentane (1ah)

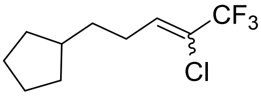

The reaction was performed following the **procedure B**. The residue was purified by flash column chromatograph (Rf = 0.75, silica gel, PE: EtOAc=20:1) to give the product as a colorless liquid (1.02 g, 45% yield). Z/E = 4.9:1, determined by ^{1}H NMR. **^{1}H NMR (500 MHz, Chloroform-*d*)** δ 6.48 (t, *J* = 7.2

Hz, 0.83 x 1H), 6.21 (t, J = 8.2 Hz, 0.17 x 1H), 2.40 – 2.27 (m, 2H), 1.84 – 1.71 (m, 3H), 1.65 – 1.58 (m, 2H), 1.56 -1.52 (m, 2H), 1.50 – 1.43 (m, 2H), 1.12 - 1.08 (m, 2H). **^{13}C NMR (126 MHz, $CDCl_3$)** δ 135.0 (q, J = 3.8 Hz), 121.2 (q, J = 37.1 Hz), 120.6 (q, J = 271.0 Hz), 39.9, 34.1, 32.7, 27.4, 25.3. **^{19}F NMR (471 MHz, $CDCl_3$)** δ -62.15, -68.90. **HRMS (EI)** calcd for $C_{10}H_{14}ClF_3$: 226.0736, found: 226.0727.

4-(2-chloro-3,3,3-trifluoroprop-1-en-1-yl)-1,1-difluorocyclohexane (1ai)

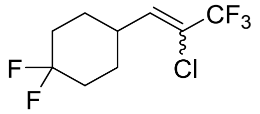

The reaction was performed following the **procedure B**. The residue was purified by flash column chromatograph (Rf = 0.75, silica gel, PE: EtOAc=20:1) to give the product as a colorless liquid (0.77 g, 31% yield). Z/E =4.6:1, determined by ^{1}H NMR. **^{1}H NMR (500 MHz, Chloroform-*d*)** δ 6.30 (dd, J = 9.1, 0.8 Hz, 0.82 x 1H), 6.04 (d, J = 10.6 Hz, 0.18 x 1H), 2.62 (q, J = 10.9 Hz, 1H), 2.18 – 2.09 (m, 2H), 1.87 -1.79 (m, 3H), 1.59 – 1.48 (m, 2H). **^{13}C NMR (126 MHz, Chloroform-*d*)** δ 141.8, 136.9 (t, J = 3.6 Hz), 122.6 (dd, J = 242.6, 239.6 Hz), 121.6 (q, J = 37.9 Hz), 120.4 (q, J = 271.7 Hz), 35.4, 33.0 (dd, J = 25.3, 23.6 Hz), 28.9 (d, J = 9.7 Hz), 27.4 (d, J = 9.7 Hz). **^{19}F NMR (471 MHz, $CDCl_3$)** δ -64.71, -71.68, -94.78 (d, J = 237.4 Hz), -104.58 (d, J = 233.2 Hz). **HRMS (EI)** calcd for $C_9H_{10}ClF_5$: 248.0391, found: 248.0398.

(3r,5r,7r)-1-(3-chloro-4,4,4-trifluorobut-2-en-1-yl)adamantane (1aj)

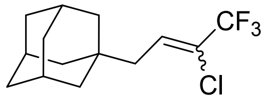

The reaction was performed following the **procedure B**. The residue was purified by flash column chromatograph (Rf = 0.75, silica gel, PE: EtOAc=20:1) to give the product as a colorless liquid (1.84 g, 66% yield). Z/E = 6.1:1, determined by ^{1}H NMR. **^{1}H NMR (500 MHz, Chloroform-*d*)** δ 6.54 (t, J = 7.6 Hz, 0.86 x 1H), 6.28 (t, J = 8.6 Hz, 0.14 x 1H), 2.08 (d, J = 6.0 Hz, 2H), 1.98 (s, 3H), 1.74 – 1.60 (m, 7H), 1.54 (s, 5H). **^{13}C NMR (126 MHz, $CDCl_3$)** δ 131.3 (q, J = 3.6 Hz), 122.5 (q, J = 37.0 Hz), 120.6 (q, J = 271.0 Hz), 42.5, 42.2, 36.9, 33.9, 28.7. **^{19}F NMR (471 MHz, $CDCl_3$)** δ -61.24, -68.59. **HRMS (ESI)** calcd for $C_{14}H_{19}ClF_3$ $[M+H]^+$: 279.1122, found: 279.1118.

(R)-2-(4-chloro-5,5,5-trifluoropent-3-en-2-yl)-6-methoxynaphthalene (1ak)

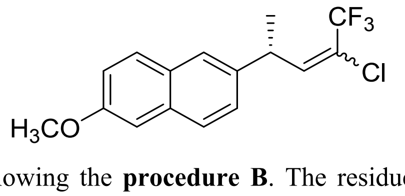

The reaction was performed following the **procedure B**. The residue was purified by flash column chromatograph (Rf = 0.65, silica gel, PE: EtOAc=10:1) to give the product as a white solid (1.41 g, 45% yield, mp: 52.2-55.7 °C). Z/E = 13.3:1, determined by ^{1}H NMR. **^{1}H NMR (500 MHz, $CDCl_3$)** δ 7.74 (dd, J = 4.4, 8.7 Hz, 2H), 7.63 (s, 1H), 7.35 (dd, J = 2.1, 8.5 Hz, 1H), 7.19 (dd, J = 2.7, 8.9 Hz, 1H), 7.14 (d, J = 2.5 Hz, 1H), 6.66 (d, J = 9.8 Hz, 0.93 x 1H), 6.38 (d, J = 11.3 Hz, 0.07 x 1H), 4.20 -4.14 (m, 1H), 3.94 (s, 3H), 1.54 (d, J = 7.0 Hz, 3H). **^{13}C NMR (126 MHz, $CDCl_3$)** δ 157.9, 138.6 (q, J = 3.8 Hz), 137.6, 133.8, 129.3, 129.2, 127.6, 126.0, 125.2, 120.6 (q, J = 271.5 Hz), 120.3 (q, J = 37.4 Hz), 119.3, 105.7, 55.4, 38.4, 20.1. **^{19}F NMR (471 MHz, $CDCl_3$)** δ -61.14, -68.73. **HRMS (ESI)** calcd for

$C_{16}H_{15}ClF_3O$ [M+H]$^+$: 315.0758, found: 315.0754.

1-(4-chloro-5,5,5-trifluoropent-3-en-2-yl)-4-isobutylbenzene (1al)

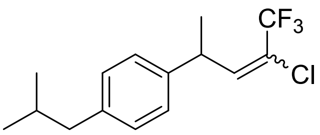

The reaction was performed following the **procedure B**. The residue was purified by flash column chromatograph (Rf = 0.75, silica gel, PE: EtOAc=20:1) to give the product as a colorless liquid (1.60 g, 55% yield). Z/E = 10.1:1, determined by ^{1}H NMR. **^{1}H NMR (500 MHz, CDCl$_3$)** δ 7.1 (d, *J* = 8.6 Hz, 2H), 7.1 (d, *J* = 7.8 Hz, 2H), 6.5 (d, *J* = 9.7 Hz, 0.91 x 1H), 6.26 (d, *J* = 10.9 Hz, 0.09 x 1H), 4.0 (p, *J* = 7.1 Hz, 1H), 2.4 (d, *J* = 7.2 Hz, 2H), 1.8 (dh, *J* = 13.5, 6.8 Hz, 1H), 1.4 (dd, *J* = 6.9, 3.3 Hz, 3H), 0.9 (d, *J* = 6.3 Hz, 6H). **^{13}C NMR (126 MHz, CDCl$_3$)** δ 140.7, 139.7, 138.8 (q, *J* = 3.6 Hz), 129.7, 126.8, 120.6 (q, *J* = 271.5 Hz), 120.2 (q, *J* = 37.4 Hz), 45.2, 38.2, 30.4, 22.5, 20.1.**^{19}F NMR (471 MHz, CDCl$_3$)** δ -61.23, -68.85. **HRMS (ESI)** calcd for $C_{15}H_{19}ClF_3$ [M+H]$^+$: 291.1122, found: 291.1122.

4-(4-chloro-5,5,5-trifluoropent-3-en-2-yl)-2-fluoro-1,1'-biphenyl (1am)

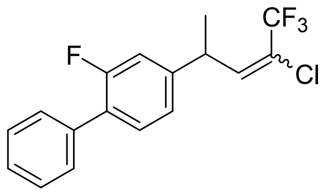

The reaction was performed following the **procedure B**. The residue was purified by flash column chromatograph (Rf = 0.75, silica gel, PE: EtOAc=20:1) to give the product as a colorless liquid (1.84 g, 56% yield). Z/E = 7.3:1, determined by ^{1}H NMR. **^{1}H NMR (500 MHz, CDCl$_3$)** δ 7.57 (d, *J* = 7.4 Hz, 2H), 7.52 – 7.35 (m, 4H), 7.10 (dd, *J* = 21.2, 9.7 Hz, 2H), 6.60 (d, *J* = 9.7 Hz, 0.88 x 1H), 6.31 (d, *J* = 11.3 Hz, 0.12 x 1H), 4.08 (p, *J* = 7.2 Hz, 1H), 1.50 (d, *J* = 7.0 Hz, 3H). **^{13}C NMR (126 MHz, CDCl$_3$)** δ 161.0, 159.0, 143.9 (d, *J* = 7.2 Hz), 137.7 (q, *J* = 3.6 Hz), 135.5, 131.2 (d, *J* = 3.5 Hz), 129.1, 128.6, 127.9, 123.1 (d, *J* = 3.6 Hz), 121.0 (q, *J* = 37.6 Hz), 120.5 (q, *J* = 271.7 Hz), 114.7 (d, *J* = 22.9 Hz), 38.0, 19.9. **^{19}F NMR (471 MHz, CDCl$_3$)** δ -62.26, -69.87, -118.09. **HRMS (ESI)** calcd for $C_{17}H_{14}ClF_4$ [M+H]$^+$: 329.0715, found: 329.0719.

(3-(3-chloro-4,4,4-trifluorobut-2-en-1-yl)-5-methoxy-2-methyl-1H-indol-1-yl)(4-chlorophenyl)methanone (1an)

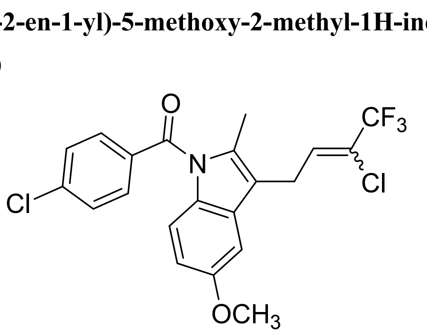

The reaction was performed following the **procedure B**. The residue was purified by flash column chromatograph (Rf = 0. 5, silica gel, PE: EtOAc=4:1) to give the product as a light yellow solid (1.81 g, 41% yield, mp: 101.5-103.9 °C). Z/E = 11.5:1, determined by ^{1}H NMR. **^{1}H NMR (500 MHz, CDCl$_3$)** δ 7.67 (d, *J* = 8.4 Hz, 2H), 7.48 (d, *J* = 8.4 Hz, 2H), 6.91 – 6.82 (m, 2H), 6.69 (dd, *J* = 9.0, 2.6 Hz, 1H),

6.55 (t, J = 7.2 Hz, 0.92 x 1H), 6.25 (t, J = 7.7 Hz, 0.08 x 1H), 3.84 (s, 3H), 3.67 (d, J = 7.1 Hz, 2H), 2.40 (s, 3H). **^{13}C NMR (126 MHz, CDCl$_3$)** δ 168.4, 156.1, 139.5, 135.3, 133.8, 132.2 (q, J = 3.6 Hz), 131.3, 130.9, 130.3, 129.3, 121.8 (q, J = 37.4 Hz), 120.4 (q, J = 271.6 Hz), 115.2, 114.7, 111.8, 100.9, 55.7, 23.3, 13.3. **^{19}F NMR (471 MHz, CDCl$_3$)** δ -61.62, -68.74. **HRMS (EI)** calcd for $C_{21}H_{16}Cl_2F_3NO_2$: 441.0510, found: 441.0496.

(Z)-(2-chloro-3,3,3-trifluoroprop-1-en-1-yl)benzene (1ao)

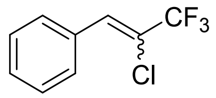

The reaction was performed following the **procedure B**. The residue was purified by flash column chromatograph (Rf = 0.8, silica gel, PE: EtOAc=20:1) to give the product as a colorless liquid (1.24 g, 60% yield). Z/E = 6.3:1, determined by ^{19}F NMR. **^{1}H NMR (500 MHz, CDCl$_3$)** δ 7.74 (dd, J = 7.5, 1.8 Hz, 2H), 7.45 (d, J = 7.1 Hz, 3H), 7.31 (s, 1H). **^{13}C NMR (126 MHz, CDCl$_3$)** δ 131.7, 130.8 (q, J = 4.3 Hz), 130.2, 130.1, 128.8, 121.1 (q, J = 271.8 Hz), 119.5 (q, J = 36.9 Hz). **^{19}F NMR (471 MHz, CDCl$_3$)** δ -61.44, -68.61. **HRMS (ESI)** calcd for $C_9H_7ClF_3$ $[M+H]^+$: 207.0183, found: 207.0178.

(Z)-1-(2-chloro-3,3,3-trifluoroprop-1-en-1-yl)-4-methylbenzene (1ap)

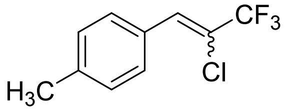

The reaction was performed following the **procedure B**. The residue was purified by flash column chromatograph (Rf = 0.8, silica gel, PE: EtOAc=20:1) to give the product as a colorless liquid (1.39 g, 63% yield). Z/E = 7.8:1, determined by ^{19}F NMR. **^{1}H NMR (500 MHz, CDCl$_3$)** δ 7.59 (d, J = 8.3 Hz, 2H), 7.21 (d, J = 2.1 Hz, 2H), 7.13 (s, 1H), 2.35 (s, 3H). **^{13}C NMR (126 MHz, CDCl$_3$)** δ 140.7, 130.7 (q, J = 4.4 Hz), 130.1, 129.5, 128.6, 121.2 (q, J = 271.6 Hz), 118.3 (q, J = 36.5 Hz), 21.5. **^{19}F NMR (471 MHz, CDCl$_3$)** δ -61.37, -68.49. **HRMS (EI)** calcd for $C_{10}H_9ClF_3$: 221.0339, found: 221.0336.

(Z)-1-(2-chloro-3,3,3-trifluoroprop-1-en-1-yl)-4-ethylbenzene (1aq)

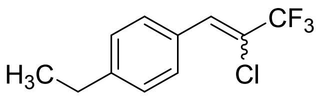

The reaction was performed following the **procedure B**. The residue was purified by flash column chromatograph (Rf = 0.8, silica gel, PE: EtOAc=20:1) to give the product as a colorless liquid (1.47 g, 63% yield). Z/E = 9.3:1, determined by ^{19}F NMR. **^{1}H NMR (500 MHz, CDCl$_3$)** δ 7.65 (d, J = 8.1 Hz, 2H), 7.26 (s, 1H), 7.25 (s, 2H), 2.68 (q, J = 7.6 Hz, 2H), 1.25 (t, J = 7.7 Hz, 3H). **^{13}C NMR (126 MHz, CDCl$_3$)** δ 146.9, 130.7 (q, J = 4.8 Hz), 130.2, 129.1, 128.3, 121.2 (q, J = 271.4 Hz), 118.4 (q, J = 36.5 Hz), 28.9, 15.4. **^{19}F NMR (471 MHz, CDCl$_3$)** δ -61.37, -68.45. **HRMS (EI)** calcd for $C_{11}H_{10}ClF_3$: 234.0423, found: 234.0416

(Z)-1-(2-chloro-3,3,3-trifluoroprop-1-en-1-yl)-4-isopropylbenzene (1ar)

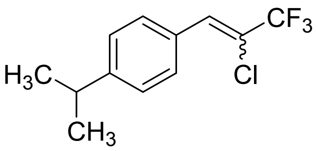

The reaction was performed following the **procedure B**. The residue was purified by flash column chromatograph (Rf = 0.8, silica gel, PE: EtOAc=20:1) to give the product as a colorless liquid (1.51 g, 61% yield). Z/E = 5.7:1, determined by ^{19}F NMR. **^{1}H NMR (500 MHz, CDCl$_3$)** δ 7.69 (d, J = 7.9 Hz, 2H), 7.31 (d, J = 7.8 Hz, 2H), 7.28 (s, 1H), 3.01 – 2.90 (m, J = 6.9 Hz, 1H), 1.30 (d, J = 1.5 Hz, 3H), 1.28 (d, J = 1.5 Hz, 3H). **^{13}C NMR (126 MHz, CDCl$_3$)** δ 151.5, 130.6 (q, J = 4.2 Hz), 130.3, 129.3, 126.9, 121.2 (q, J = 271.8 Hz), 118.4 (q, J = 36.8 Hz), 34.2, 23.9. **^{19}F NMR (471 MHz, CDCl$_3$)** δ -61.37, -68.44. **HRMS (ESI)** calcd for $C_{12}H_{13}ClF_3$ [M+H]$^+$: 249.0652, found: 249.0654.

(Z)-1-(tert-butyl)-4-(2-chloro-3,3,3-trifluoroprop-1-en-1-yl)benzene (1as)

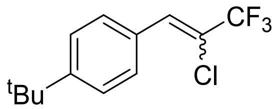

The reaction was performed following the **procedure B**. The residue was purified by flash column chromatograph (Rf = 0.8, silica gel, PE: EtOAc=20:1) to give the product as a colorless liquid (1.55 g, 59% yield). Z/E = 9.6:1, determined by ^{19}F NMR. **^{1}H NMR (500 MHz, CDCl$_3$)** δ 7.67 (d, J = 8.5 Hz, 2H), 7.44 (d, J = 8.6 Hz, 2H), 7.25 (s, 1H), 1.33 (s, 9H). **^{13}C NMR (126 MHz, CDCl$_3$)** δ 152.3, 129.1 (q, J = 4.2 Hz), 128.6, 127.4, 124.3, 119.7 (q, J = 271.6 Hz), 117.1 (q, J = 36.5 Hz), 33.6, 29.8. **^{19}F NMR (471 MHz, CDCl$_3$)** δ -62.74, -69.82. **HRMS (EI)** calcd for $C_{13}H_{14}ClF_3$: 262.0736, found: 262.0740

(Z)-1-(2-chloro-3,3,3-trifluoroprop-1-en-1-yl)-4-methoxybenzene (1at)

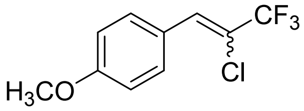

The reaction was performed following the **procedure B**. The residue was purified by flash column chromatograph (Rf = 0.7, silica gel, PE: EtOAc=10:1) to give the product as a colorless liquid (1.25 g, 53% yield). Z/E = 6.1:1, determined by ^{19}F NMR. **^{1}H NMR (500 MHz, CDCl$_3$)** δ 7.70 (d, J = 8.8 Hz, 2H), 7.19 (s, 1H), 6.93 (d, J = 8.6 Hz, 2H), 3.83 (s, 3H). **^{13}C NMR (126 MHz, CDCl$_3$)** δ 161.1, 132.0, 130.1 (q, J = 4.2 Hz), 124.3, 121.3 (q, J = 271.4 Hz), 116.8 (q, J = 36.8 Hz), 114.2, 55.4. **^{19}F NMR (471 MHz, CDCl$_3$)** δ -61.28, -68.23. **HRMS (ESI)** calcd for $C_{10}H_9ClF_3O$ [M+H]$^+$: 237.0289, found: 237.0295.

1-(2-chloro-3,3,3-trifluoroprop-1-en-1-yl)-4-(trifluoromethoxy)benzene (1au)

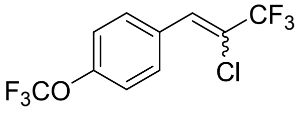

The reaction was performed following the **procedure B**. The residue was purified by flash column chromatograph (Rf = 0.75, silica gel, PE: EtOAc=10:1) to give the product as a colorless liquid (0.96 g, 33% yield). Z/E = 7.4:1, determined by ^{19}F NMR. **^{1}H NMR (500 MHz, CDCl$_3$)** δ 7.79 – 7.73 (m, 2H), 7.30 – 7.27 (m, 2H), 7.27 (s, 1H). **^{13}C NMR (126 MHz, CDCl$_3$)** δ 150.2, 131.7, 130.2, 129.4 (q, J = 4.7 Hz), 121.0, 120.9 (q, J = 272.1 Hz), 120.5 (q, J = 258.5 Hz), 120.4 (q, J = 37.1 Hz). **^{19}F NMR (471 MHz, CDCl$_3$)** δ -57.71, -61.57, -68.81. **HRMS (ESI)** calcd for $C_{10}H_6ClF_6O$ [M+H]$^+$: 291.0006, found:

291.0007.

1-(benzyloxy)-4-(2-chloro-3,3,3-trifluoroprop-1-en-1-yl)benzene (1av)

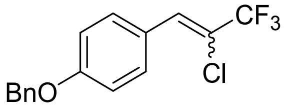

The reaction was performed following the **procedure B**. The residue was purified by flash column chromatograph (Rf = 0.65, silica gel, PE: EtOAc=10:1) to give the product as a white solid (1.03 g, 33% yield). Z/E = 6.4:1, determined by ^{19}F NMR. **^{1}H NMR (500 MHz, CDCl$_3$)** δ 7.75 (d, J = 8.7 Hz, 2H), 7.49 – 7.40 (m, 5H), 7.24 (s, 1H), 7.04 (d, J = 8.8 Hz, 2H), 5.13 (s, 2H). **^{13}C NMR (126 MHz, CDCl$_3$)** δ 160.2, 136.5, 132.0, 130.1 (q, J = 4.2 Hz), 128.8, 128.3, 127.6, 124.5, 121.3 (q, J = 271.4 Hz), 117.0 (q, J = 36.7 Hz), 115.1, 70.2. **^{19}F NMR (471 MHz, CDCl$_3$)** δ -61.22, -68.17. **HRMS (ESI)** calcd for $C_{16}H_{12}ClF_3O$ [M+H]$^+$: 313.0602, found: 313.0608.

4-(2-chloro-3,3,3-trifluoroprop-1-en-1-yl)-1,1'-biphenyl (1aw)

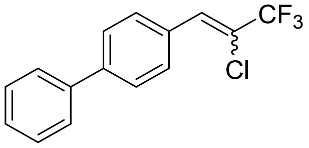

The reaction was performed following the **procedure B**. The residue was purified by flash column chromatograph (Rf = 0.7, silica gel, PE: EtOAc=20:1) to give the product as a white solid (1.58 g, 56% yield, mp: 70.3-75.3 °C). Z/E = 10.1:1, determined by ^{19}F NMR. **^{1}H NMR (500 MHz, CDCl$_3$)** δ 7.84 (d, J = 8.2 Hz, 2H), 7.68 (dd, J = 7.8, 17.0 Hz, 4H), 7.50 (t, J = 7.3 Hz, 2H), 7.45 – 7.37 (m, 1H), 7.36 (s, 1H). **^{13}C NMR (126 MHz, CDCl$_3$)** δ 143.0, 140.1, 130.6, 130.6, 130.4 (q, J = 4.2 Hz), 129.1, 128.1, 127.3, 127.2, 121.1 (q, J = 271.8 Hz), 119.3 (q, J = 36.7 Hz). **^{19}F NMR (471 MHz, CDCl$_3$)** δ -70.98, -78.13. **HRMS (ESI)** calcd for $C_{15}H_{11}ClF_3$ [M+H]$^+$: 283.0496, found: 283.0499.

(4-(2-chloro-3,3,3-trifluoroprop-1-en-1-yl)phenyl)(methyl)sulfane (1ax)

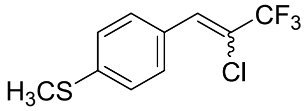

The reaction was performed following the **procedure B**. The residue was purified by flash column chromatograph (Rf = 0.6, silica gel, PE: EtOAc=10:1) to give the product as a colorless liquid (1.08 g, 43% yield). Z/E = 9.5:1, determined by ^{19}F NMR. **^{1}H NMR (600 MHz, CDCl$_3$)** δ 7.66 (d, J = 8.5 Hz, 2H), 7.28 – 7.23 (m, 2H), 7.22 (s, 1H), 2.50 (s, 3H). **^{13}C NMR (151 MHz, CDCl$_3$)** δ 142.1, 130.5, 130.1 (q, J = 4.5 Hz), 129.1, 128.0, 125.7, 121.1 (q, J = 271.8 Hz), 118.4 (q, J = 36.5 Hz), 15.1. **^{19}F NMR (565 MHz, CDCl$_3$)** δ -61.32, -68.40. **HRMS (ESI)** calcd for $C_{10}H_9ClF_3S$ [M+H]$^+$: 253.0060, found: 253.0052.

3-(2-chloro-3,3,3-trifluoroprop-1-en-1-yl)furan (1ay)

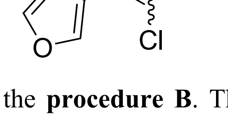

The reaction was performed following the **procedure B**. The residue was purified by flash column chromatograph (Rf = 0.85, silica gel, PE: EtOAc=10:1) to give the product as a colorless liquid (0.65 g,

33% yield). Z/E = 11.5:1, determined by ^{19}F NMR. **^{1}H NMR (500 MHz, CDCl$_3$)** δ 7.91 (s, 1H), 7.46 (t, *J* = 1.7 Hz, 1H), 7.13 (s, 1H), 6.84 (d, *J* = 1.7 Hz, 1H). **^{13}C NMR (126 MHz, CDCl$_3$)** δ 145.5, 143.8, 122.3 (q, *J* = 4.5 Hz), 121.1 (q, *J* = 271.3 Hz), 118.8, 118.5 (q, *J* = 37.4 Hz), 110.5. **^{19}F NMR (471 MHz, CDCl$_3$)** δ -61.67, -68.35. **HRMS (ESI)** calcd for $C_7H_5ClF_3O$ [M+H]$^+$: 196.9976, found: 196.9968.

3-(2-chloro-3,3,3-trifluoroprop-1-en-1-yl)thiophene (1az)

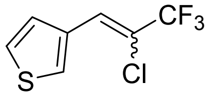

The reaction was performed following the **procedure B**. The residue was purified by flash column chromatograph (Rf = 0.85, silica gel, PE: EtOAc=10:1) to give the product as a yellow liquid (0.70 g, 33% yield). Z/E = 10.7:1, determined by ^{19}F NMR. **^{1}H NMR (600 MHz, CDCl$_3$)** δ 7.90 (d, *J* = 2.8 Hz, 1H), 7.52 (dd, *J* = 5.1, 1.2 Hz, 1H), 7.39 (dd, *J* = 5.1, 3.0 Hz, 1H), 7.32 (s, 1H). **^{13}C NMR (151 MHz, CDCl$_3$)** δ 133.0, 129.6, 128.6, 126.1, 124.9 (q, *J* = 4.7 Hz), 121.2 (q, *J* = 271.7 Hz), 118.0 (q, *J* = 37.2 Hz). **^{19}F NMR (565 MHz, CDCl$_3$)** δ -61.43, -68.18. **HRMS (ESI)** calcd for $C_7H_5ClF_3S$ [M+H]$^+$: 212.9747, found: 212.9745.

5-(2-chloro-3,3,3-trifluoroprop-1-en-1-yl)-2,3-dihydrobenzofuran (1aaa)

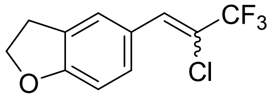

The reaction was performed following the **procedure B**. The residue was purified by flash column chromatograph (Rf = 0.65, silica gel, PE: EtOAc=10:1) to give the product as a colorless liquid (1.31 g, 53% yield). Z/E = 5.0:1, determined by ^{19}F NMR. **^{1}H NMR (500 MHz, CDCl$_3$)** δ 7.72 (s, 1H), 7.49 (d, *J* = 8.3 Hz, 1H), 7.20 (s, 1H), 6.83 (d, *J* = 8.4 Hz, 1H), 4.64 (t, *J* = 8.7 Hz, 2H), 3.25 (t, *J* = 8.7 Hz, 2H). **^{13}C NMR (126 MHz, CDCl$_3$)** δ 161.9, 131.7, 130.4 (q, *J* = 4.4 Hz), 127.9, 126.7, 124.2, 121.4 (q, *J* = 271.3 Hz), 116.0 (q, *J* = 36.5 Hz), 109.6, 72.0, 29.5. **^{19}F NMR (471 MHz, CDCl$_3$)** δ -61.14, -68.11. **HRMS (EI)** calcd for $C_{11}H_8ClF_3O$: 248.0216, found: 248.0215.

4-(2-chloro-3,3,3-trifluoroprop-1-en-1-yl)-1,2-dimethylbenzene (1aab)

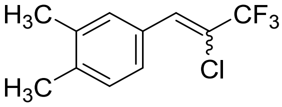

The reaction was performed following the **procedure B**. The residue was purified by flash column chromatograph (Rf = 0.8, silica gel, PE: EtOAc=20:1) to give the product as a colorless liquid (1.43 g, 61% yield). Z/E = 4.5:1, determined by ^{19}F NMR. **^{1}H NMR (500 MHz, CDCl$_3$)** δ 7.52 (d, *J* = 7.7 Hz, 1H), 7.50 (s, 1H), 7.25 (s, 1H), 7.21 (d, *J* = 7.6 Hz, 1H), 2.32 (s, 6H). **^{13}C NMR (126 MHz, CDCl$_3$)** δ 139.4, 137.1, 131.4, 130.8 (q, *J* = 4.4 Hz), 130.0, 129.3, 127.6, 121.2 (q, *J* = 271.6 Hz), 118.2 (q, *J* = 36.5 Hz), 19.9. **^{19}F NMR (471 MHz, CDCl$_3$)** δ -61.33, -68.44. **HRMS (EI)** calcd for $C_{11}H_{11}ClF_3$: 235.0496, found: 235.0492.

(Z)-1,1,1-trifluoro-4-phenylbut-2-en-2-yl trifluoromethanesulfonate (1aac)

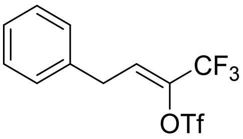

The reaction was performed following the **procedure B**. The residue was purified by flash column chromatograph (Rf = 0.45, silica gel, PE: EtOAc=4:1) to give the product as a colorless liquid (1.94 g, 58% yield). **^{1}H NMR (500 MHz, CDCl$_3$)** δ 7.34 (t, *J* = 7.1 Hz, 2H), 7.28 (t, *J* = 7.0 Hz, 1H), 7.20 (d, *J* = 7.2 Hz, 2H), 6.49 (t, *J* = 7.5 Hz, 1H), 3.67 (d, *J* = 7.0 Hz, 2H). **^{13}C NMR (126 MHz, CDCl$_3$)** δ 135.8, 134.1 (q, *J* = 39.3 Hz), 130.1 (q, *J* = 3.2 Hz), 129.2, 128.7, 127.6, 118.8 (q, *J* = 272.4 Hz), 118.6 (q, *J* = 320.4 Hz), 32.3. **^{19}F NMR (471 MHz, CDCl$_3$)** δ -69.85, -72.81. **HRMS (EI)** calcd for $C_{11}H_8F_6O_3S$: 334.0098, found: 334.0092.

(Z)-1,1,1-trifluoro-4-(4-isopropylphenyl)but-2-en-2-yl trifluoromethanesulfonate (1aad)

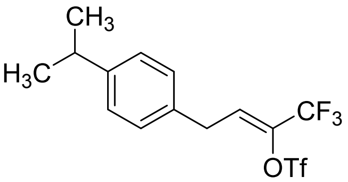

The reaction was performed following the **procedure B**. The residue was purified by flash column chromatograph (Rf = 0.45, silica gel, PE: EtOAc=4:1) to give the product as a colorless liquid (2.11 g, 56% yield). **^{1}H NMR (500 MHz, CDCl$_3$)** δ 7.24 (d, *J* = 8.2 Hz, 2H), 7.16 (d, *J* = 8.0 Hz, 2H), 6.52 (t, *J* = 7.6 Hz, 1H), 3.68 (d, *J* = 7.5 Hz, 2H), 2.93 (hept, *J* = 6.9 Hz, 1H), 1.28 (d, *J* = 0.9 Hz, 3H), 1.26 (d, *J* = 0.9 Hz, 3H). **^{13}C NMR (126 MHz, CDCl$_3$)** δ 148.4, 133.9 (q, *J* = 39.5 Hz), 133.1, 130.4 (q, *J* = 3.8 Hz), 128.6, 127.3, 118.8 (q, *J* = 273.2 Hz), 118.2 (q, *J* = 319.6 Hz), 33.9, 31.9, 24.1. **^{19}F NMR (471 MHz, CDCl$_3$)** δ -69.78, -72.77. **HRMS (EI)** calcd for $C_{14}H_{14}F_6O_3S$: 376.0568, found: 376.0559.

(Z)-4-(3-chlorophenyl)-1,1,1-trifluorobut-2-en-2-yl trifluoromethanesulfonate (1aae)

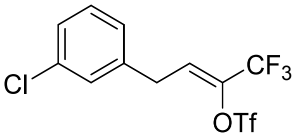

The reaction was performed following the **procedure B**. The residue was purified by flash column chromatograph (Rf = 0.45, silica gel, PE: EtOAc=4:1) to give the product as a colorless liquid (1.88 g, 51% yield). **^{1}H NMR (500 MHz, CDCl$_3$)** δ 7.33 – 7.24 (m, 2H), 7.21 (s, 1H), 7.15 – 7.06 (m, 1H), 6.47 (t, *J* = 7.6 Hz, 1H), 3.67 (d, *J* = 7.5, 1.7 Hz, 2H). **^{13}C NMR (126 MHz, CDCl$_3$)** δ 137.6, 135.0, 134.5 (q, *J* = 39.5 Hz), 130.5, 129.2 (q, *J* = 3.5 Hz), 128.8, 127.9, 126.8, 118.6 (q, *J* = 272.4 Hz), 118.6 (q, *J* = 320.3 Hz), 31.9. **^{19}F NMR (471 MHz, CDCl$_3$)** δ -69.88, -72.66. **HRMS (ESI)** calcd for $C_{11}H_8ClF_6O_3S$ $[M+H]^+$: 368.9781, found: 368.9784.

(Z)-4-(4-cyanophenyl)-1,1,1-trifluorobut-2-en-2-yl trifluoromethanesulfonate (1aaf)

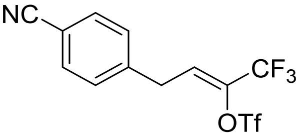

The reaction was performed following the **procedure B**. The residue was purified by flash column

chromatograph (Rf= 0.3, silica gel, PE: EtOAc=4:1) to give the product as a colorless liquid (1.51 g, 42% yield). **^{1}H NMR (500 MHz, $CDCl_3$)** δ 7.64 (d, *J* = 6.7 Hz, 2H), 7.34 (d, *J* = 8.3 Hz, 2H), 6.48 (t, *J* = 7.6 Hz, 1H), 3.75 (d, *J* = 7.5 Hz, 2H). **^{13}C NMR (126 MHz, $CDCl_3$)** δ 141.0, 135.0 (q, *J* = 39.1, 39.9 Hz), 132.9, 129.5, 128.3, 118.5 (q, *J* = 272.8 Hz), 118.5 (q, *J* = 320.4 Hz), 118.5, 111.7, 32.1. **^{19}F NMR (471 MHz, $CDCl_3$)** δ -69.98, -72.69. **HRMS (ESI)** calcd for $C_{12}H_8F_6NO_3S$ [M+H]$^+$: 360.0124, found: 360.0124.

tert-butyl(((3R,8R,9S,10S,13R,14S,17R)-17-((R)-6-chloro-7,7,7-trifluorohept-5-en-2-yl)-10,13-dimethylhexadecahydro-1H-cyclopenta[a]phenanthren-3-yl)oxy)dimethylsilane (1aag)

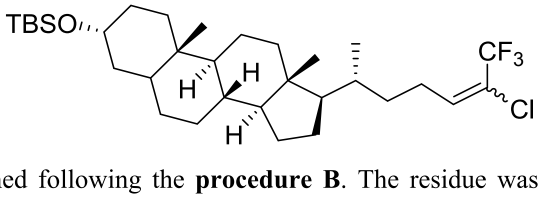

The reaction was performed following the **procedure B**. The residue was purified by flash column chromatograph (Rf= 0.65, silica gel, PE: EtOAc=10:1) to give the product as a white solid (35% yield, mp: 79.2-82.7 °C). Z/E = 9.2:1, determined by ^{19}F NMR. **^{1}H NMR (500 MHz, $CDCl_3$)** δ 6.45 (t, *J* = 7.3 Hz, 1H), 3.58 (td, *J* = 5.4, 10.9 Hz, 1H), 2.38 – 2.28 (m, 1H), 2.24 – 2.15 (m, 1H), 1.95 (dt, *J* = 3.2, 12.3 Hz, 1H), 1.87 – 1.72 (m, 4H), 1.59 – 1.49 (m, 3H), 1.45 – 1.31 (m, 8H), 1.27 – 1.15 (m, 5H), 1.14 – 1.02 (m, 5H), 0.95 (d, *J* = 6.6 Hz, 3H), 0.90 (s, 3H), 0.89 (s, 9H), 0.64 (s, 3H), 0.06 (s, 6H). **^{13}C NMR (126 MHz, $CDCl_3$)** δ 135.3 (q, *J* = 3.7 Hz), 121.1 (q, *J* = 37.2 Hz), 120.6 (q, *J* = 270.5 Hz), 73.0, 56.5, 56.1, 42.9, 42.4, 40.4, 40.3, 37.1, 36.0, 35.8, 35.7, 34.7, 33.9, 31.2, 28.4, 27.4, 26.5, 26.1, 25.0, 24.4, 23.5, 20.9, 18.5, 18.5, 12.1, -4.5. **^{19}F NMR (471 MHz, $CDCl_3$)** δ -62.06, -68.78. **HRMS (ESI)** calcd for $C_{32}H_{55}ClF_3OSi$ [M+H]$^+$: 575.3657, found: 575.3665.

(8R,9S,13S,14S,17S)-3-((9-chloro-10,10,10-trifluorodec-8-en-1-yl)oxy)-13-methyl-7,8,9,11,12,13,14,15,16,17-decahydro-6H-cyclopenta[a]phenanthren-17-yl heptanoate (1aah)

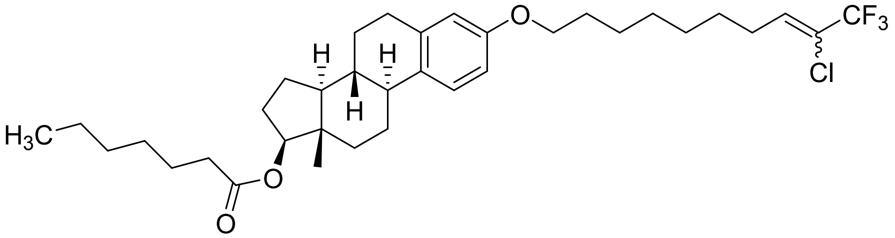

The reaction was performed following the **procedure B**. The residue was purified by flash column chromatograph (Rf= 0.55, silica gel, PE: EtOAc=10:1) to give the product as a colorless liquid (33% yield). Z/E = 8.7:1, determined by ^{19}F NMR. **^{1}H NMR (500 MHz, $CDCl_3$)** δ 7.20 (d, *J* = 8.6 Hz, 1H), 6.71 (d, *J* = 8.6 Hz, 1H), 6.64 (s, 1H), 6.48 (t, *J* = 7.3 Hz, 1H), 4.72 (t, *J* = 8.5 Hz, 1H), 3.94 (t, *J* = 6.4 Hz, 2H), 2.90 – 2.80 (m, 2H), 2.36 – 2.27 (m, 5H), 2.27 – 2.18 (m, 2H), 1.90 (td, *J* = 9.4, 8.3, 5.1 Hz, 2H), 1.81 – 1.74 (m, 3H), 1.70 – 1.61 (m, 2H), 1.52 – 1.43 (m, 7H), 1.41 – 1.27 (m, 14H), 0.93 -0.90 (m, 3H), 0.85 (s, 3H). **^{13}C NMR (126 MHz, $CDCl_3$)** δ 174.0, 157.1, 137.9, 134.7 (q, *J* = 4.3 Hz), 132.5, 126.4, 121.4 (q, *J* = 36.8, 37.2 Hz), 120.5 (q, *J* = 271.1 Hz), 114.5, 112.1, 82.5, 67.8, 49.9, 43.9, 43.1, 38.7, 37.0, 34.7, 31.6, 29.9, 29.4, 29.2, 29.1, 28.9, 28.0, 27.7, 27.6, 27.4, 26.3, 26.0, 25.2, 23.4, 22.6, 14.1, 12.2. **^{19}F NMR (471 MHz, $CDCl_3$)** δ -61.99, -68.77. **HRMS (EI)** calcd for $C_{35}H_{51}ClF_3O_3$: 611.3473, found: 611.3470.

2,2'-(1,1-difluoro-5-phenylpent-1-ene-2,3-diyl)bis(4,4,5,5-tetramethyl-1,3,2-dioxaborolane) (3)

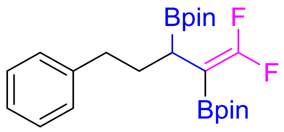

The reaction was performed following the **procedure D**. The residue was purified by flash column chromatograph (Rf = 0.4, PE: Et_2O = 10:1) to give the product as a white solid (175.9 mg, 81% yield, mp: 45.3 - 47 °C.).

^{1}H NMR (500 MHz, $CDCl_3$) δ 7.27 – 7.22 (m, 2H), 7.19 – 7.11 (m, 3H), 2.68 – 2.58 (m, 1H), 2.53 – 2.43 (m, 1H), 2.03 – 1.87 (m, 2H), 1.85 – 1.74 (m, 1H), 1.49 – 0.81 (m, 24H). **^{13}C NMR (126 MHz, $CDCl_3$)** δ 159.8 (dd, *J* = 299.7, 296.7 Hz)., 143.0, 128.6, 128.3, 125.6, 83.7, 83.4, 35.4, 32.3, 25.1, 24.9, 24.8, 24.4. **^{19}F NMR (470MHz, CDCl3)** δ -71.58 (d, *J* = 16.2 Hz), -72.69 (d, *J* = 16.1 Hz). **^{11}B NMR (128 MHz, $CDCl_3$)** δ 30.28. **HRMS (ESI)** calcd for $C_{23}H_{35}B_2H_2O_4$ $[M+H]^+$: 435.2684, found: 435.2680.

2,2'-(1,1-difluoro-5-(p-tolyl)pent-1-ene-2,3-diyl)bis(4,4,5,5-tetramethyl-1,3,2-dioxaborolane) (4)

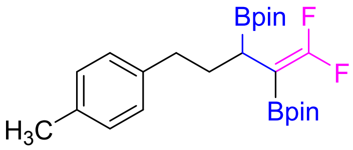

The reaction was performed following the **procedure D**. The residue was purified by flash column chromatograph (Rf = 0.4, PE: Et_2O = 10:1) to give the product as a white solid (156.9 mg, 70% yield, mp: 40.8-42 °C.).

^{1}H NMR (500 MHz, $CDCl_3$) δ 7.08 (s, 4H), 2.66 – 2.56 (m, 1H), 2.52 – 2.42 (m, 1H), 2.32 (s, 3H), 2.03 – 1.88 (m, 2H), 1.83 -1.73 (m, 1H), 1.32 – 1.22 (m, 24H). **^{13}C NMR (126 MHz, $CDCl_3$)** δ 159.8 (t, *J* = 296.3 Hz), 139.9, 134.9, 129.0, 128.5, 83.6, 83.4, 35.0, 32.4, 25.1, 25.0, 24.8, 24.5, 21.1. **^{19}F NMR (470 MHz, $CDCl_3$)** δ -71.69 (d, *J* = 15.7 Hz), -72.84 (d, *J* = 18.2 Hz). **^{11}B NMR (128 MHz, $CDCl_3$)** δ 32.92. **HRMS (ESI)** calcd for $C_{24}H_{37}B_2F_2O_4^+$ $[M+H]^+$: 449.2841, found: 449.2846.

2,2'-(1,1-difluoro-5-(4-methoxyphenyl)pent-1-ene-2,3-diyl)bis(4,4,5,5-tetramethyl-1,3,2-dioxaborolane) (5)

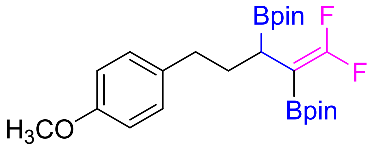

The reaction was performed following the **procedure D**. The residue was purified by flash column chromatograph (Rf = 0.35, PE: Et_2O = 10:1) to give the product as a white solid (181.4 mg, 74% yield, mp: 47.3 – 58.6 °C.).

^{1}H NMR (500 MHz, $CDCl_3$) δ 7.08 (d, *J* = 8.6 Hz, 2H), 6.80 (d, *J* = 8.6 Hz, 2H), 3.77 (s, 3H), 2.61 – 2.55 (m, 1H), 2.46 – 2.40 (m, 1H), 2.01 – 1.85 (m, 2H), 1.83 – 1.71 (m, 1H), 1.31 – 1.19 (m, 24H). **^{13}C NMR (126 MHz, $CDCl_3$)** δ 159.7 (dd, *J* = 301.1, 297.4 Hz), 157.6, 135.1, 129.4, 113.7, 83.6, 83.4, 55.3, 34.4, 32.5, 25.1, 24.9, 24.8, 24.4. **^{19}F NMR NMR (470 MHz, $CDCl_3$)** δ -71.66 (d, *J* = 16.2 Hz), -72.80 (d, *J* = 16.1 Hz). **^{11}B NMR (128 MHz, $CDCl_3$)** δ 30.83. **HRMS (ESI)** calcd for $C_{24}H_{37}B_2F_2O_5$ $[M+H]^+$: 465.2790, found: 465.2787.

2,2'-(1,1-difluoro-5-(4-(trifluoromethyl)phenyl)pent-1-ene-2,3-diyl)bis(4,4,5,5-tetramethyl-1,3,2-dioxaborolane) (6)

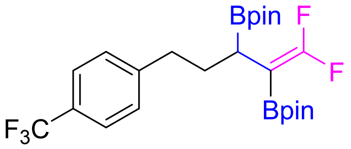

The reaction was performed following the **procedure D**. The residue was purified by flash column chromatograph (Rf = 0.45, PE: Et_2O = 10:1) to give the product as a white solid (170.8 mg, 68% yield, mp: 33.2-34 °C.).

^{1}H NMR (500 MHz, $CDCl_3$) δ 7.49 (d, *J* = 8.1 Hz, 2H), 7.25 (d, *J* = 8.2 Hz, 2H), 2.72 -2.66 (m, 1H), 2.60 – 2.47 (m, 1H), 2.03 – 1.85 (m, 2H), 1.85 – 1.68 (m, 1H), 1.48 – 0.79 (m, 24H). **^{13}C NMR (126 MHz, $CDCl_3$)** δ 159.9 (dd, *J* = 300.5, 296.9 Hz), 147.2, 128.9, 128.0 (q, *J* = 32.0 Hz), 125.2 (q, *J* = 3.7 Hz), 124.6 (q, *J* = 272.7), 83.7, 83.5, 35.2, 32.0, 25.1, 24.9, 24.8, 24.4 **^{19}F NMR (471 MHz, $CDCl_3$)** δ -62.12, -71.47 (d, *J* = 15.2 Hz), -72.44 (d, *J* = 15.2 Hz). **^{11}B NMR (128 MHz, $CDCl_3$)** δ 30.64. **HRMS (ESI)** calcd for $C_{24}H_{34}B_2F_5O_4$ $[M+H]^+$: 503.2558, found: 503.2555.

2,2'-(1,1-difluoro-5-(4-fluorophenyl)pent-1-ene-2,3-diyl)bis(4,4,5,5-tetramethyl-1,3,2-dioxaborolane) (7)

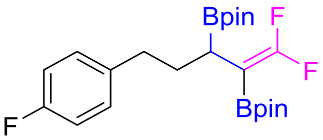

The reaction was performed following the **procedure D**. The residue was purified by flash column chromatograph (Rf = 0.4, PE: Et_2O = 10:1) to give the product as a white solid (178.6 mg, 79% yield, mp: 38.5-40 °C.).

^{1}H NMR (500 MHz, $CDCl_3$) δ 7.10 (dd, *J* = 8.5, 5.6 Hz, 2H), 6.92 (t, *J* = 8.8 Hz, 2H), 2.63 -2.57 (m, 1H), 2.48 -2.42 (m, 1H), 2.03 – 1.83 (m, 2H), 1.82 – 1.70 (m, 1H), 1.29 – 1.21 (m, 24H). **^{13}C NMR (126 MHz, $CDCl_3$)** δ 161.2 (d, *J* = 242.6 Hz), 159.8 (dd, *J* = 299.9, 297.4Hz) 138.5 (d, *J* = 3.7 Hz), 129.9 (d, *J* = 7.3 Hz), 114.9 (d, *J* = 21.4 Hz), 83.7, 83.5, 34.5, 32.4, 25.1, 24.9, 24.8, 24.4. **^{19}F NMR (470 MHz, $CDCl_3$)** δ -71.57 (d, *J* = 15.0 Hz), -72.63 (d, *J* = 14.8 Hz), -118.22. **^{11}B NMR (128 MHz, $CDCl_3$)** δ 30.62 **HRMS (EI)** calcd for $C_{23}H_{33}B_2F_3O_4$: 452.2517, found: 452.2508.

2,2'-(5-(4-chlorophenyl)-1,1-difluoropent-1-ene-2,3-diyl)bis(4,4,5,5-tetramethyl-1,3,2-dioxaborolane) (8)

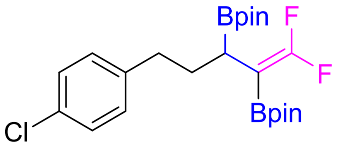

The reaction was performed following the **procedure D**. The residue was purified by flash column chromatograph (Rf = 0.4, PE: Et_2O = 10:1) to give the product as a white solid (168.6 mg, 72% yield, mp: 45.2-47.5 °C.).

^{1}H NMR (500 MHz, $CDCl_3$) δ 7.21 (d, *J* = 8.4 Hz, 2H), 7.08 (d, *J* = 8.3 Hz, 2H), 2.63 – 2.57 (m, 1H), 2.49 - 2.43 (m, 1H), 2.01 – 1.84 (m, 2H), 1.80 -1.72 (m, 1H), 1.28 – 1.22 (m, 24H). **^{13}C NMR (126**

MHz, $CDCl_3$) δ 159.8 (dd, *J* = 297.5, 295.0 Hz), 141.4, 131.3, 130.0, 128.3, 83.7, 83.5, 34.7, 32.1, 25.1, 24.9, 24.8, 24.5 **^{19}F NMR (471 MHz, $CDCl_3$)** δ -71.57 (d, *J* = 18.1 Hz), -72.62 (d, *J* = 14.7 Hz). **^{11}B NMR** (128 MHz, **CDCl3)** δ 30.62. **HRMS (ESI)** calcd for $C_{23}H_{34}B_2ClF_2O_4$ $[M+H]^+$: 429.2294, found: 429.2290.

4-(5,5-difluoro-3,4-bis(4,4,5,5-tetramethyl-1,3,2-dioxaborolan-2-yl)pent-4-en-1-yl)phenyl acetate (9)

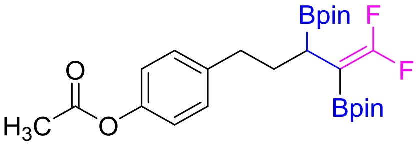

The reaction was performed following the **procedure D**. The residue was purified by flash column chromatograph (R_f = 0.3, PE: Et_2O = 10:1) to give the product as a white solid (187.1 mg, 76% yield, mp: 52.1-53.5 °C.).

^{1}H NMR (500 MHz, $CDCl_3$) δ 7.16 (d, *J* = 8.4 Hz, 2H), 6.95 (d, *J* = 8.4 Hz, 2H), 2.64 – 2.59 (m, 1H), 2.50 – 2.45 (m, 1H), 2.27 (s, 3H), 2.01 – 1.87 (m, 2H), 1.83 – 1.73 (m, 1H), 1.27 – 1.22 (m, 24H). **^{13}C NMR (126 MHz, $CDCl_3$)** δ 169.8, 159.8 (dd, *J* = 300.0, 296.9 Hz), 148.7, 140.6, 129.5, 121.3, 83.7, 83.5, 34.8, 32.3, 25.1, 25.0, 24.8, 24.5, 21.3. **^{19}F NMR (471 MHz, $CDCl_3$)** δ -72.13 (dd, *J* = 544.4, 15.3 Hz). **^{11}B NMR (128 MHz, $CDCl_3$)** δ 28.82. **HRMS (ESI)** calcd for $C_{25}H_{37}B_2F_2O_6$ $[M+H]^+$: 493.2739, found: 493.2742.

2,2'-(5-(2-bromophenyl)-1,1-difluoropent-1-ene-2,3-diyl)bis(4,4,5,5-tetramethyl-1,3,2-dioxaborolane) (10)

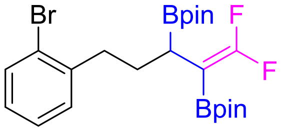

The reaction was performed following the **procedure D**. The residue was purified by flash column chromatograph (R_f = 0.4, PE: Et_2O = 20:1) to give the product as a white solid (179.3 mg, 70% yield, mp: 37-38 °C.).

^{1}H NMR (500 MHz, $CDCl_3$) δ 7.48 (d, *J* = 7.5 Hz, 1H), 7.23 – 7.16 (m, 2H), 7.04 – 6.98 (m, 1H), 2.82 – 2.72 (m, 1H), 2.66 – 2.55 (m, 1H), 2.00 – 1.88 (m, 2H), 1.82 – 1.69 (m, 1H), 1.29 – 1.21 (m, 24H). **^{13}C NMR (126 MHz, $CDCl_3$)** δ 160.0 (dd, *J* = 301.1, 298.6 Hz), 142.3, 132.7, 130.6, 127.4, 124.5, 83.7, 83.5, 35.8, 30.7, 25.2, 25.0, 24.8, 24.6. **^{19} FNMR (470 MHz, $CDCl_3$)** δ -71.40 (d, *J* = 15.0 Hz), -72.52 (d, *J* = 15.8 Hz). **^{11}B NMR (128 MHz, $CDCl_3$)** δ 30.80. **HRMS (ESI)** calcd for $C_{23}H_{34}B_2BrF_2O_4$ $[M+H]^+$: 513.1789, found: 513.1786.

2,2'-(5-(3-bromophenyl)-1,1-difluoropent-1-ene-2,3-diyl)bis(4,4,5,5-tetramethyl-1,3,2-dioxaborolane) (11)

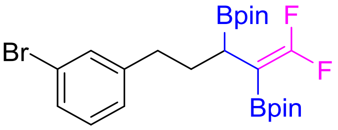

The reaction was performed following the **procedure D**. The residue was purified by flash column

chromatograph (Rf = 0.4, PE: Et_2O = 20:1) to give the product as a white solid (199.7 mg, 78% yield, mp: 35.1-36 °C.).

^{1}H NMR (500 MHz, $CDCl_3$) δ 7.32 (s, 1H), 7.28 (d, J = 7.7 Hz, 1H), 7.13 – 7.06 (m, 2H), 2.65 – 2.54 (m, 1H), 2.51 – 2.41 (m, 1H), 2.02 – 1.85 (m, 2H), 1.82 – 1.68 (m, 1H), 1.29 – 1.22 (m, 24H). **^{13}C NMR (126 MHz, $CDCl_3$)** δ 159.8 (dd, J = 300.2, 297.3 Hz), 145.4, 131.7, 129.8, 128.7, 127.3, 122.4, 83.7, 83.5, 35.1, 32.1, 25.1, 24.9, 24.8, 24.5. **^{19}F NMR (471 MHz, $CDCl_3$)** δ -72.85 (d, J = 15.1 Hz), -73.92 (d, J = 14.9 Hz). **^{11}B NMR (128 MHz, $CDCl_3$)** δ 30.48. **HRMS (EI)** calcd for $C_{23}H_{33}B_2BrF_2O_4$: 512.1716, found: 512.1708.

2,2'-(5-(4-bromophenyl)-1,1-difluoropent-1-ene-2,3-diyl)bis(4,4,5,5-tetramethyl-1,3,2-dioxaborolane) (12)

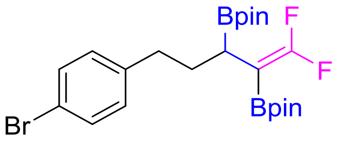

The reaction was performed following the **procedure D**. The residue was purified by flash column chromatograph (Rf = 0.4, PE: Et_2O = 10:1) to give the product as a white solid (176.7 mg, 69% yield, mp: 28.7 - 30 °C.).

^{1}H NMR (500 MHz, $CDCl_3$) δ 7.4 (d, J = 8.3 Hz, 2H), 7.0 (d, J = 8.3 Hz, 2H), 2.6 – 2.5 (m, 1H), 2.5 – 2.4 (m, 1H), 2.0 – 1.8 (m, 2H), 1.79 – 1.71 (m, 1H), 1.3 – 1.2 (m, 24H). **^{13}C NMR (126 MHz, $CDCl_3$)** δ 159.8 (dd, J = 300.4, 297.0 Hz), 141.9, 131.3, 130.4, 119.3, 83.7, 83.5, 34.7, 32.1, 25.2, 24.9, 24.8, 24.4. **^{19}F NMR (470 MHz, $CDCl_3$)** δ -71.45 (d, J = 14.1 Hz), -72.51 (d, J = 18.8 Hz). **^{11}B NMR (128 MHz, $CDCl_3$)** δ 30.53. **HRMS (EI)** calcd for $C_{23}H_{33}B_2BrF_2O_4$: 512.1716, found: 512.1714.

2,2'-(1,1-difluoro-4-phenylbut-1-ene-2,3-diyl)bis(4,4,5,5-tetramethyl-1,3,2-dioxaborolane) (13)

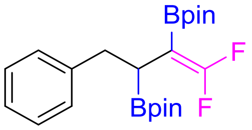

The reaction was performed following the **procedure D**. The residue was purified by flash column chromatograph (Rf = 0.4, PE: Et_2O = 10:1) to give the product as a white solid (166.0 mg, 79% yield, mp: 47.5 - 49 °C.).

^{1}H NMR (500 MHz, $CDCl_3$) δ 7.24 – 7.19 (m, 2H), 7.16 – 7.10 (m, 3H), 3.07 – 3.03 (m, 1H), 2.72 (dd, J = 13.9, 11.3 Hz, 1H), 2.21 – 2.17 (m, 1H), 1.28 – 1.22 (m, 24H). **^{13}C NMR (126 MHz, $CDCl_3$)** δ 159.8 (dd, J = 301.1, 297.4 Hz), 142.1, 129.0, 128.1, 125.7, 83.6, 36.1, 25.1, 25.0, 24.8, 24.5. **^{19}F NMR (471 MHz, $CDCl_3$)** δ -72.35 (d, J = 13.4 Hz), -74.05 (d, J = 13.4 Hz). **^{11}B NMR (128 MHz, $CDCl_3$)** δ 32.74, 30.45. **HRMS (ESI)** calcd for $C_{22}H_{33}B_2F_2O_4$ $[M+H]^+$: 421.2528, found: 421.2531.

2,2'-(1,1-difluoro-4-(4-methoxyphenyl)but-1-ene-2,3-diyl)bis(4,4,5,5-tetramethyl-1,3,2-dioxaborolane) (14)

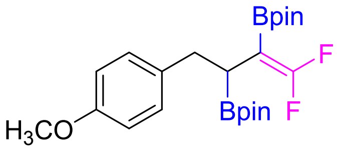

The reaction was performed following the **procedure D**. The residue was purified by flash column chromatograph (Rf = 0.35, PE: Et_2O = 10:1) to give the product as a white solid (168.8 mg, 75% yield, mp: 30-31 °C.).

^{1}H NMR (500 MHz, $CDCl_3$) δ 7.04 (d, J = 8.6 Hz, 2H), 6.77 (d, J = 8.6 Hz, 2H), 3.76 (s, 3H), 2.99 (dd, J = 13.9, 4.8 Hz, 1H), 2.67 (dd, J = 13.7, 11.5 Hz, 1H), 2.14 (dd, J = 11.3, 4.8 Hz, 1H), 1.31 – 1.17 (m, 24 H). **^{13}C NMR (125MHz, $CDCl_3$)** δ159.8 (dd, J = 301.1, 298.6 Hz) 157.7, 134.2, 129.8, 113.5, 83.5, 83.5, 55.2, 35.2, 25.1, 24.9, 24.8, 24.4 **^{19}F NMR (470 MHz, CDCl3)** δ -71.30 (d, J = 14.3 Hz), -72.89 (d, J = 14.7 Hz). **^{11}B NMR (128 MHz, $CDCl_3$)** δ 30.18. **HRMS (ESI)** calcd for $C_{23}H_{35}B_2F_2O_5$ $[M+H]^+$: 451.2633, found: 451.2629.

2,2'-(4-(4-bromophenyl)-1,1-difluorobut-1-ene-2,3-diyl)bis(4,4,5,5-tetramethyl-1,3,2-dioxaborolane) (15)

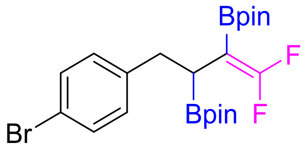

The reaction was performed following the **procedure D**. The residue was purified by flash column chromatograph (Rf = 0.4, PE: Et_2O = 10:1) to give the product as a white solid (174.4 mg, 70% yield, mp: 46.7 – 47.5 °C.).

^{1}H NMR (500 MHz, $CDCl_3$) δ 7.33 (d, J = 8.3 Hz, 2H), 7.00 (d, J = 8.3 Hz, 2H), 3.00 – 2.96 (m, 1H), 2.68 (dd, J = 13.9, 11.2 Hz, 1H), 2.16 – 2.12 (m, 1H), 1.26 – 1.22 (m, 24H). **^{13}C NMR (126 MHz, $CDCl_3$)** δ 159.9 (dd, J = 300.9, 298.2 Hz), 141.2, 131.1, 130.8, 119.5, 83.7, 83.7, 35.6, 25.1, 25.0, 24.8, 24.5. **^{19}F NMR (471 MHz, $CDCl_3$)** δ -71.95 (d, J = 13.6 Hz), -73.64 (d, J = 13.0 Hz). **^{11}B NMR (128 MHz, $CDCl_3$)** δ 30.50. **HRMS (ESI)** calcd for $C_{22}H_{32}B_2BrF_2O_4$ $[M+H]^+$: 499.1633, found: 499.1629.

2,2'-(1,1-difluoro-6-phenylhex-1-ene-2,3-diyl)bis(4,4,5,5-tetramethyl-1,3,2-dioxaborolane) (16)

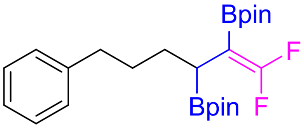

The reaction was performed following the **procedure D**. The residue was purified by flash column chromatograph (Rf = 0.4, PE: Et_2O = 10:1) to give the product as a colorless liquid (190.5 mg, 85% yield).

^{1}H NMR (500 MHz, $CDCl_3$) δ 7.26 – 7.22 (m, 2H), 7.18 – 7.12 (m, 3H), 2.67 – 2,61 (m, 1H), 2.55 – 2.49 (m, 1H), 1.90 – 1.87 (m, 1H), 1.77 – 1.69 (m, 1H), 1.68 – 1.59 (m, 1H), 1.59 – 1.48 (m, 2H), 1.25 – 1.19 (m, 24H). **^{13}C NMR (126 MHz, $CDCl_3$)** δ 159.8 (dd, J = 299.4, 296.9 Hz), 143.0, 128.5, 128.3, 125.6, 83.6, 83.4, 36.0, 30.9, 29.8, 25.1, 24.9, 24.8, 24.4. **^{19}F NMR (471 MHz, $CDCl_3$)** δ -74.83 (d, J = 16.5 Hz), -75.72 (d, J = 16.6 Hz). **^{11}B NMR (128 MHz, $CDCl_3$)** δ 30.54. **HRMS (EI)** calcd for $C_{24}H_{36}B_2F_2O_4$: 448.2768, found: 448.2758.

2,2'-(1,1-difluoro-5,5-diphenylpent-1-ene-2,3-diyl)bis(4,4,5,5-tetramethyl-1,3,2-dioxaborolane) (17)

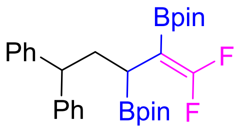

The reaction was performed following the **procedure D**. The residue was purified by flash column chromatograph (R_f = 0.4, PE: Et_2O = 10:1) to give the product as a white solid (127.6 mg, 50% yield, mp: 40-41.5 °C.).

^{1}H NMR (500 MHz, $CDCl_3$) δ 7.27 (d, J = 4.4 Hz, 4H), 7.22 – 7.19 (m, 4H), 7.18 – 7.14 (m, 1H), 7.12 – 7.09 (m, 1H), 3.97 (dd, J = 10.2, 5.6 Hz, 1H), 2.48 – 2.42 (m, 1H), 2.25 – 2.14 (m, 1H), 1.83 – 1.80 (m, 1H), 1.26 – 1.19 (m, 24H). **^{13}C NMR (126 MHz, $CDCl_3$)** δ 159.7 (dd, J = 300.7, 296.0 Hz), 146.0, 143.9, 128.5, 128.4, 128.3, 128.0, 126.0, 125.9, 83.6, 83.4, 50.0, 36.1, 25.2, 24.9, 24.8, 24.4. **^{19}F NMR (471 MHz, $CDCl_3$)** δ -71.21 (d, J = 14.6 Hz), -72.66 (d, J = 14.6 Hz). **^{11}B NMR (128 MHz, $CDCl_3$)** δ 30.71, 22.14. **HRMS (EI)** calcd for $C_{29}H_{38}B_2F_2O_4$: 510.2924, found: 510.2925.

2,2'-(1,1-difluoro-4-(naphthalen-1-yl)but-1-ene-2,3-diyl)bis(4,4,5,5-tetramethyl-1,3,2-dioxaborolane) (18)

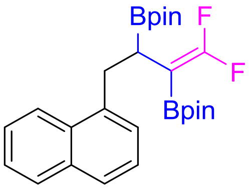

The reaction was performed following the **procedure D**. The residue was purified by flash column chromatograph (R_f = 0.4, PE: Et_2O = 10:1) to give the product as a white solid (117.6 mg, 50% yield, mp: 50.5-52 °C.).

^{1}H NMR (500 MHz, $CDCl_3$) δ 8.12 (d, J = 8.3 Hz, 1H), 7.80 (d, J = 8.2 Hz, 1H), 7.66 (d, J = 7.9 Hz, 1H), 7.50 – 7.40 (m, 2H), 7.33 (t, J = 7.6 Hz, 1H), 7.26 – 7.22 (m, 1H), 3.61 (dd, J = 14.2, 5.0 Hz, 1H), 3.08 (dd, J = 14.1, 10.6 Hz, 1H), 2.37 (dd, J = 10.7, 5.0 Hz, 1H), 1.27 – 1.15 (m, 24H). **^{13}C NMR (126 MHz, $CDCl_3$)** δ 159.9 (dd, J = 300.8, 297.8 Hz), 138.0, 134.0, 132.2, 128.7, 127.1, 126.6, 125.5, 125.2, 124.2, 83.7, 83.6, 33.3, 25.1, 25.0, 24.9, 24.5. **^{19}F NMR (471 MHz, $CDCl_3$)** δ -70.68 (d, J = 14.1 Hz), -72.72 (d, J = 11.5 Hz). **^{11}B NMR (128 MHz, $CDCl_3$)** δ 30.13. **HRMS (ESI)** calcd for $C_{26}H_{35}B_2F_2O_4$ $[M+H]^+$: 471.2684, found: 471.2681.

2,2'-(4-([1,1'-biphenyl]-4-yl)-1,1-difluorobut-1-ene-2,3-diyl)bis(4,4,5,5-tetramethyl-1,3,2-dioxaborolane) (19)

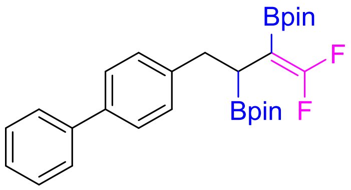

The reaction was performed following the **procedure D**. The residue was purified by flash column chromatograph (R_f = 0.4, PE: Et_2O = 10:1) to give the product as a white solid (201.0 mg, 81% yield, mp: 81.5 - 83 °C.).

^{1}H NMR (500 MHz, $CDCl_3$) δ 7.57 (dd, J = 8.3, 1.2 Hz, 2H), 7.46 (d, J = 8.1 Hz, 2H), 7.41 (t, J = 7.8

Hz, 2H), 7.34 – 7.29 (m, 1H), 7.23 – 7.18 (m, 2H), 3.11 – 3.07 (m, 1H), 2.77 (dd, *J* = 13.8, 11.4 Hz, 1H), 2.23 (dd, *J* = 11.0, 4.3 Hz, 1H), 1.29 – 1.22 (m, 24H). **^{13}C NMR (126 MHz, $CDCl_3$)** δ 159.8 (dd, *J* = 300.4, 297.7 Hz), 141.4, 141.3, 138.6, 129.5, 128.8, 127.1, 127.0, 126.8, 83.6, 35.8, 25.1, 25.0, 24.8, 24.5. **^{19}F NMR (471 MHz, $CDCl_3$)** δ -70.74 (d, *J* = 13.4 Hz), -72.64 (d, *J* = 14.0 Hz). **^{11}B NMR (128 MHz, $CDCl_3$)** δ 28.56. **HRMS (ESI)** calcd for $C_{26}H_{35}B_2F_2O_4$ $[M+H]^+$: 497.2841,found: 497.2838

2,2'-(4-(benzo[d][1,3]dioxol-5-yl)-1,1-difluorobut-1-ene-2,3-diyl)bis(4,4,5,5-tetramethyl-1,3,2-dioxaborolane) (20)

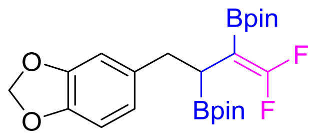

The reaction was performed following the **procedure D**. The residue was purified by flash column chromatograph (R_f = 0.3, PE: Et_2O = 10:1) to give the product as a white solid (155.5 mg, 67% yield, mp: 85 -86.5 °C.).

^{1}H NMR (500 MHz, $CDCl_3$) δ 6.68 – 6.63 (m, 2H), 6.58 – 6.54 (m, 1H), 5.88 (s, 2H), 2.96 (dd, *J* = 14.1, 2.9 Hz, 1H), 2.65 (dd, *J* = 13.9, 11.2 Hz, 1H), 2.13 (dd, *J* = 11.1, 4.1 Hz, 1H), 1.26 – 1.22 (m, 24H). **^{13}C NMR (126 MHz, $CDCl_3$)** δ 159.8 (dd, *J* = 299.9, 297.4 Hz), 147.3, 145.5, 136.0, 121.8, 109.4, 107.9, 100.7, 83.6, 35.8, 25.1, 24.9, 24.8, 24.5. **^{19}F NMR (471 MHz, $CDCl_3$)** δ -72.79 (d, *J* = 5.6 Hz), -74.40 (d, *J* = 14.0 Hz). **^{11}B NMR (128 MHz, $CDCl_3$)** δ 30.46. **HRMS (ESI)** calcd for $C_{23}H_{33}B_2F_2O_6$ $[M+H]^+$: 465.2426, found: 465.2434.

2,2'-(1,1-difluoro-5-phenoxypent-1-ene-2,3-diyl)bis(4,4,5,5-tetramethyl-1,3,2-dioxaborolane) (21)

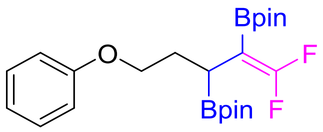

The reaction was performed following the **procedure D**. The residue was purified by flash column chromatograph (R_f = 0.3, PE: Et_2O = 20:1) to give the product as a white solid (146.3 mg, 65% yield, mp: 41.5-43 °C.).

^{1}H NMR (500 MHz, $CDCl_3$) δ 7.27 – 7.22 (m, 2H), 6.93 – 6.85 (m, 3H), 4.01 – 3.86 (m, 2H), 2.24 – 2.09 (m, 2H), 1.97 – 1.86 (m, 1H), 1.27 – 1.22 (m, 24H). **^{13}C NMR (126 MHz, $CDCl_3$)** δ 159.9 (dd, *J* = 300.7, 297.2 Hz), 159.2, 129.4, 120.4, 114.7, 83.7, 83.6, 66.9, 29.7, 25.2, 25.0, 24.8, 24.4. **^{19}F NMR NMR (470 MHz, $CDCl_3$)** δ -71.31 (d, *J* = 14.3 Hz), -72.24 (d, *J* = 14.1 Hz). **^{11}B NMR (128 MHz, $CDCl_3$)** δ 30.38. **HRMS (EI)** calcd for $C_{23}H_{34}B_2F_2O_5$: 450.2560, found: 450.2570.

2,2'-(5-(3,4-dimethoxyphenyl)-1,1-difluoropent-1-ene-2,3-diyl)bis(4,4,5,5-tetramethyl-1,3,2-dioxaborolane) (22)

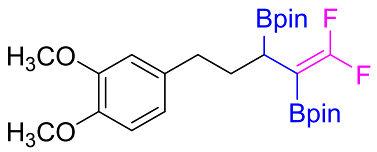

The reaction was performed following the **procedure D**. The residue was purified by flash column chromatograph (R_f = 0.2, PE: Et_2O = 10:1) to give the product as a colorless liquid (173 mg, 70% yield).

^{1}H NMR (500 MHz, CDCl$_3$) δ 6.75 (d, *J* = 8.7 Hz, 1H), 6.69 (d, *J* = 7.0 Hz, 2H), 3.84 (s, 3H), 3.83 (s, 3H), 2.60 – 2.54 (m, 1H), 2.44 – 2.38 (m, 1H), 2.00 – 1.85 (m, 2H), 1.81 – 1.71 (m, 1H), 1.26 – 1.21 (m, 24H). **^{13}C NMR (126 MHz, CDCl$_3$)** δ 159.7 (dd, *J* = 299.7, 296.7 Hz), 148.7, 147.0, 135.7, 120.3, 112.0, 111.2, 83.6, 83.4, 56.0, 55.8, 34.9, 32.4, 25.1, 24.9, 24.8, 24.4. **^{19}F NMR (471 MHz, CDCl$_3$)** δ -71.70 (d, *J* = 16.8 Hz), -72.82 (d, *J* = 16.4 Hz). **^{11}B NMR (128 MHz, CDCl$_3$)** δ 30.26. **HRMS (ESI)** calcd for $C_{25}H_{39}B_2F_2O_6$ [M+H]$^+$: 495.2895, found: 495.2891.

2,2'-(1,1-difluoro-5-(5-methylfuran-2-yl)pent-1-ene-2,3-diyl)bis(4,4,5,5-tetramethyl-1,3,2-dioxaborolane) (23)

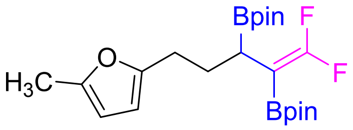

The reaction was performed following the **procedure D**. The residue was purified by flash column chromatograph (Rf = 0.35, PE: Et$_2$O = 10:1) to give the product as a white solid (170.9 mg, 78% yield, mp: 37.2-38.5 ^{o}C.).

^{1}H NMR (500 MHz, CDCl$_3$) δ 5.86 – 5.74 (m, 2H), 2.61 – 2.55 (m, 1H), 2.50 – 2.44 (m, 1H), 2.22 (s, 3H), 2.03 – 1.96 (m, 1H), 1.88 (dd, *J* = 11.3, 3.8 Hz, 1H), 1.81 – 1.71 (m, 1H), 1.27 – 1.21 (m, 24H). **^{13}C NMR (126 MHz, CDCl$_3$)** δ 159.9 (t, *J* = 296.3 Hz), 154.7, 150.0, 105.8, 105.3, 83.7, 83.4, 28.8, 27.4, 25.1, 24.9, 24.8, 24.4, 13.6 **^{19}F NMR (470 MHz, CDCl3)** δ -71.48 (d, *J* = 14.8 Hz), -72.63 (d, *J* = 15.5 Hz). **^{11}B NMR (128 MHz, CDCl$_3$)** δ 30.62. **HRMS (EI)** calcd for $C_{22}H_{34}B_2F_2O_5$: 438.2560, found: 438.2552.

2,2'-(1,1-difluoro-6-(thiophen-2-yl)hex-1-ene-2,3-diyl)bis(4,4,5,5-tetramethyl-1,3,2-dioxaborolane) (24)

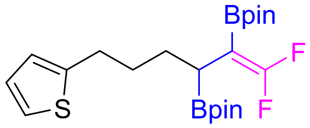

The reaction was performed following the **procedure D**. The residue was purified by flash column chromatograph (Rf = 0.45, PE: Et$_2$O = 10:1) to give the product as a colorless liquid (193.0 mg, 85% yield).

^{1}H NMR (500 MHz, CDCl$_3$) δ 7.10 – 7.05 (m, 1H), 6.89 (dd, *J* = 5.1, 3.4 Hz, 1H), 6.79 – 6.74 (m, 1H), 2.88 – 2.82 (m, 1H), 2.78 – 2.72 (m, 1H), 1.93 – 1.84 (m, 1H), 1.82 – 1.66 (m, 2H), 1.65 – 1.52 (m, 2H), 1.26 – 1.21 (m, 24H). **^{13}C NMR (126 MHz, CDCl$_3$)** δ 159.8 (dd, *J* = 299.9, 298.6 Hz), 145.8, 126.6, 123.9, 122.7, 83.6, 83.3, 31.1, 29.9, 29.6, 25.0, 24.8, 24.7, 24.4. **^{19}F NMR (470 MHz, CDCl$_3$)** δ -71.91 (d, *J* = 14.9 Hz), -72.82 (d, *J* = 15.2 Hz). **^{11}B NMR** (128 MHz**, CDCl$_3$)** δ 30.63. **HRMS (ESI)** calcd for $C_{22}H_{35}B_2F_2O_4S$ [M+H]$^+$: 455.2405, found: 455.2401.

tert-butyl4-(3,3-difluoro-1,2-bis(4,4,5,5-tetramethyl-1,3,2-dioxaborolan-2-yl)allyl)piperidine-1-carboxylate (25)

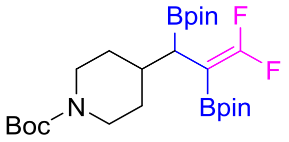

The reaction was performed following the **procedure D**. The residue was purified by flash column chromatograph (Rf = 0.35, PE: Et_2O = 10:1) to give the product as a white solid (187.4 mg, 73% yield, mp: 40.5-42 ^{o}C).

^{1}H NMR (500 MHz, CDCl$_3$) δ 4.00 (s, 2H), 2.63 (d, *J* = 44.6 Hz, 2H), 1.78 (d, *J* = 9.9 Hz, 2H), 1.66 (d, *J* = 8.6 Hz, 1H), 1.62 – 1.51 (m, 1H), 1.40 (d, *J* = 2.1 Hz, 9H), 1.23 – 1.17 (m, 24H), 1.10 – 1.05 (m, 1H), 1.02 – 0.91 (m, 1H). **^{13}C NMR (126 MHz, CDCl$_3$)** δ 159.6 (dd, *J* = 300.6, 297.4 Hz), 155.0, 83.6, 83.3, 79.1, 44.2, 36.3, 32.2, 31.3, 28.5, 24.9, 24.8, 24.7, 24.5 **^{19}F NMR (471 MHz, CDCl$_3$)** δ -69.84, -72.18. **^{11}B NMR (128 MHz, CDCl$_3$)** δ 30.50. **HRMS (EI)** calcd for $C_{25}H_{43}B_2F_2NO_6$: 513.3245, found: 513.3241.

benzyl4-(3,3-difluoro-1,2-bis(4,4,5,5-tetramethyl-1,3,2-dioxaborolan-2-yl)allyl)piperidine-1-carboxylate (26)

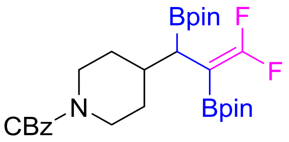

The reaction was performed following the **procedure D**. The residue was purified by flash column chromatograph (Rf = 0.25, PE: Et_2O = 4:1) to give the product as a colorless liquid (177.9 mg, 65% yield).

^{1}H NMR (500 MHz, CDCl$_3$) δ 7.34 (d, *J* = 4.4 Hz, 4H), 7.32 – 7.27 (m, 1H), 5.11 (s, 2H), 4.14 (s, 2H), 2.76 (d, *J* = 39.5 Hz, 2H), 1.91 – 1.80 (m, 2H), 1.72 (d, *J* = 8.6 Hz, 1H), 1.68 – 1.57 (m, 1H), 1.26 – 1.21 (m, 24H), 1.18 – 1.10 (m, 1H), 1.09 – 0.99 (m, 1H). **^{13}C NMR (126 MHz, CDCl$_3$)** δ 159.7 (dd, *J* = 300.6, 297.6 Hz), 155.4, 137.2, 128.6, 128.0, 127.9, 83.7, 83.4, 67.0, 44.5, 44.4, 36.3, 27.0, 25.0, 24.8, 24.8, 24.6. **^{19}F NMR (471 MHz, CDCl$_3$)** δ -69.45 (d, *J* = 29.6 Hz), -71.94 (d, *J* = 67.1 Hz). **^{11}B NMR (128 MHz, CDCl$_3$)** δ 30.70. **HRMS (ESI)** calcd for $C_{28}H_{42}B_2F_2NO_6$ $[M+H]^+$: 548.3161, found: 548.3164.

4-(4,4-difluoro-2,3-bis(4,4,5,5-tetramethyl-1,3,2-dioxaborolan-2-yl)but-3-en-1-yl)-1-tosylpiperidine (27)

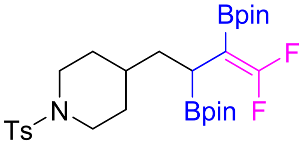

The reaction was performed following the **procedure D**. The residue was purified by flash column chromatograph (Rf = 0.2, PE: Et_2O = 4:1) to give the product as a white solid (200.5 mg, 69% yield, mp: 48.9-50 ^{o}C.).

^{1}H NMR (500 MHz, CDCl$_3$) δ 7.61 (d, *J* = 8.2 Hz, 2H), 7.30 (d, *J* = 8.0 Hz, 2H), 3.72 (t, *J* = 11.7 Hz, 2H), 2.42 (s, 3H), 2.16 – 2.07 (m, 2H), 1.92 – 1.88 (m, 1H), 1.79 (d, *J* = 12.7 Hz, 1H), 1.59 – 1.47 (m, 2H), 1.44 – 1.37 (m, 1H), 1.36 – 1.30 (m, 1H), 1.23 – 1.17 (m, 24H), 1.16 – 1.09 (m, 1H), 1.09 – 0.98

(m, 1H). **¹³C NMR (126 MHz, CDCl₃)** δ 159.5 (dd, J = 299.4, 296.9 Hz), 143.5, 133.0, 129.7, 127.8, 83.7, 83.5, 46.7, 46.7, 35.8, 33.6, 32.6, 30.2, 25.2, 24.9, 24.8, 24.3, 21.6. **¹⁹F NMR (376 MHz, CDCl₃)** δ -72.92 (d, J = 14.4 Hz), -73.36 (d, J = 15.3 Hz). **¹¹B NMR (128 MHz, CDCl₃)** δ 29.45. **HRMS (ESI)** calcd for $C_{22}H_{44}B_2F_2NO_6S$ $[M+H]^+$: 582.3038, found:582.3041

2,2'-(1,1-difluoro-4-(tetrahydro-2H-pyran-4-yl)but-1-ene-2,3-diyl)bis(4,4,5,5-tetramethyl-1,3,2-dioxaborolane) (28)

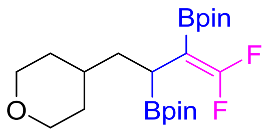

The reaction was performed following the **procedure D**. The residue was purified by flash column chromatograph (R_f = 0.35, PE: Et_2O = 10:1) to give the product as a colorless liquid (145.6 mg, 68% yield).

¹H NMR (500 MHz, CDCl₃) δ 3.95 – 3.86 (m, 2H), 3.43 – 3.20 (m, 2H), 1.98 – 1.95 (m, 1H), 1.67 (d, J = 13.3 Hz, 1H), 1.58 – 1.50 (m, 1H), 1.49 – 1.29 (m, 4H), 1.22 (dd, J = 7.7, 5.5 Hz, 24H), 1.15 – 1.06 (m, 1H). **¹³C NMR (126 MHz, CDCl₃)** δ 159.6 (dd, J = 299.5, 296.9 Hz), 83.6, 83.4, 68.3, 68.3, 36.8, 34.1, 33.3, 32.1, 25.1, 24.9, 24.8, 24.4. **¹⁹F NMR (471 MHz, CDCl₃)** δ -74.69 (d, J = 17.5 Hz), -75.48 (d, J = 17.3 Hz). **¹¹B NMR (128 MHz, CDCl₃)** δ 30.37. **HRMS (ESI)** calcd for $C_{21}H_{37}B_2F_2O_5$ $[M+H]^+$: 429.2790, found: 429.2786.

2,2'-(1,1-difluorododec-1-ene-2,3-diyl)bis(4,4,5,5-tetramethyl-1,3,2-dioxaborolane) (29)

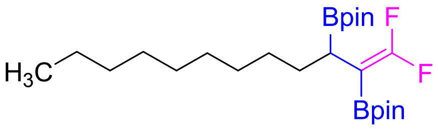

The reaction was performed following the **procedure D**. The residue was purified by flash column chromatograph (R_f = 0.55, PE: Et_2O = 10:1) to give the product as a colorless liquid (193.9 mg, 85% yield).

¹H NMR (500 MHz, CDCl₃) δ 1.86 – 1.70 (m, 1H), 1.68 – 1.54 (m, 1H), 1.48 – 1.38 (m, 1H), 1.33 – 1.05 (m, 38H), 0.86 (t, J = 6.9 Hz, 3H). **¹³C NMR (126 MHz, CDCl₃)** δ 159.8 (dd, J = 299.4, 296.9 Hz), 83.6, 83.3, 32.0, 30.0, 29.8, 29.7, 29.5, 29.0, 25.1, 24.9, 24.8, 24.4, 22.8, 14.2. **¹⁹FNMR (471 MHz, CDCl₃)** δ -72.25 (d, J = 17.4 Hz), -73.27 (d, J = 16.6 Hz). **¹¹B NMR (128 MHz, CDCl₃)** δ 30.42. **HRMS (EI)** calcd for $C_{24}H_{44}B_2F_2O_4$: 456.3394, found: 456.3393

ethyl (R)-8,8-difluoro-6,7-bis(4,4,5,5-tetramethyl-1,3,2-dioxaborolan-2-yl)oct-7-enoate (30)

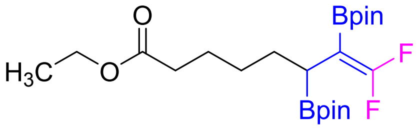

The reaction was performed following the **procedure D**. The residue was purified by flash column chromatograph (R_f = 0.4, PE: Et_2O = 10:1) to give the product as a white solid (174.1 mg, 76% yield, mp: 25.6-27 °C.).

¹H NMR (500 MHz, CDCl₃) δ 4.08 (q, J = 7.3 Hz, 2H), 2.24 (t, J = 7.9 Hz, 2H), 1.85 – 1.76 (m, 1H),

1.69 – 1.49 (m, 3H), 1.49 – 1.38 (m, 1H), 1.31 – 1.18 (m, 29H). **^{13}C NMR (126 MHz, $CDCl_3$)** δ 174.0, 159.8 (dd, *J* = 299.9, 296.6 Hz), 83.6, 83.4, 60.2, 34.5, 29.6, 28.5, 25.1, 25.0, 24.9, 24.8, 24.4, 14.3. **^{19}F NMR (471 MHz, $CDCl_3$)** δ -73.00 (d, *J* = 15.3 Hz), -73.97 (d, *J* = 14.8 Hz). **^{11}B NMR (128 MHz, $CDCl_3$)** δ 30.44. **HRMS (ESI)** calcd for $C_{22}H_{39}B_2F_2O_6$ $[M+H]^+$: 459.2895, found: 459.2892.

tert-butyl((5,5-difluoro-3,4-bis(4,4,5,5-tetramethyl-1,3,2-dioxaborolan-2-yl)pent-4-en-1-yl)oxy)dimethylsilane (31)

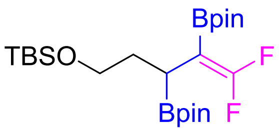

The reaction was performed following the **procedure D**. The residue was purified by flash column chromatograph (R_f = 0.5, PE: Et_2O = 10:1) to give the product as a colorless liquid (195.3 mg, 80% yield).

^{1}H NMR (500 MHz, $CDCl_3$) δ 3.67 – 3.43 (m, 2H), 2.01 – 1.98 (m, 1H), 1.95 – 1.85 (m, 1H), 1.69 – 1.56 (m, 1H), 1.25 – 1.21 (m, 24H), 0.87 (s, 9H), 0.01 (d, *J* = 1.3 Hz, 6H). **^{13}C NMR (126 MHz, $CDCl_3$)** δ 159.8 (dd, *J* = 299.9, 296.4 Hz), 83.6, 83.4, 62.3, 33.3, 26.1, 25.1, 24.9, 24.8, 24.5, 18.4, -5.2. **^{19}F NMR (471 MHz, $CDCl_3$)** δ -73.37 (d, *J* = 14.4 Hz), -74.77 (d, *J* = 14.7 Hz). **^{11}B NMR (128 MHz, $CDCl_3$)** δ 30.00. **HRMS (ESI)** calcd for $C_{23}H_{45}B_2F_2O_4Si$ $[M+H]^+$: 473.3236, found: 489.3188.

2,2'-(11-bromo-1,1-difluoroundec-1-ene-2,3-diyl)bis(4,4,5,5-tetramethyl-1,3,2-dioxaborolane) (32)

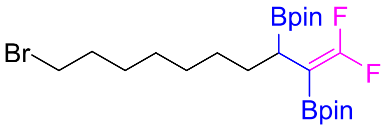

The reaction was performed following the **procedure D**. The residue was purified by flash column chromatograph (R_f = 0.5, PE: Et_2O = 20:1) to give the product as a colorless liquid (215.1 mg, 85% yield).

^{1}H NMR (500 MHz, $CDCl_3$) δ 3.38 (t, *J* = 6.9 Hz, 2H), 1.88 – 1.77 (m, 3H), 1.65 (d, *J* = 7.1 Hz, 1H), 1.48 – 1.34 (m, 3H), 1.32 – 1.18 (m, 30H). **^{13}C NMR (126 MHz, $CDCl_3$)** δ 159.7 (dd, *J* = 299.9, 297.4 Hz), 83.5, 83.2, 34.0, 32.9, 29.9, 29.3, 28.8, 28.2, 25.0, 24.8, 24.7, 24.4. **^{19}F NMR (471 MHz, $CDCl_3$)** δ -73.73 (d, *J* = 17.2 Hz), -74.75 (d, *J* = 17.3 Hz). **^{11}B NMR (128 MHz, $CDCl_3$)** δ 30.86. **HRMS (ESI)** calcd for $C_{22}H_{40}B_2BrF_2O_4$ $[M+H]^+$: 507.2259, found: 507.2262 .

2,2'-(5-cyclohexyl-1,1-difluoropent-1-ene-2,3-diyl)bis(4,4,5,5-tetramethyl-1,3,2-dioxaborolane) (33)

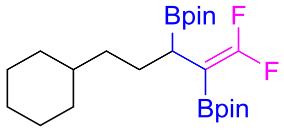

The reaction was performed following the **procedure D**. The residue was purified by flash column chromatograph (R_f = 0.4, PE: Et_2O = 10:1) to give the product as a colorless liquid (167.3 mg, 76% yield).

^{1}H NMR (500 MHz, $CDCl_3$) δ 1.76 (dt, *J* = 10.6, 4.5 Hz, 1H), 1.71 – 1.57 (m, 6H), 1.51 – 1.38 (m, 1H), 1.29 – 1.02 (m, 30H), 0.89 – 0.73 (m, 2H). **^{13}C NMR (126 MHz, $CDCl_3$)** δ 159.7 (dd, *J* = 299.9, 297.4

Hz), 83.5, 83.3, 37.8, 36.9, 33.9, 33.3, 27.2, 26.9, 26.6, 26.5, 25.1, 24.9, 24.8, 24.4. **^{19}F NMR (471 MHz, $CDCl_3$)** δ -72.21 – -72.35 (m), -73.26 (d, *J* = 17.5 Hz). **^{11}B NMR (128 MHz, $CDCl_3$)** δ 30.26. **HRMS (ESI)** calcd for $C_{23}H_{41}B_2F_2O_4$ [M+H]$^+$: 441.3154, found: 441.3158.

2,2'-(1-(2,3-dihydro-1H-inden-2-yl)-3,3-difluoroprop-2-ene-1,2-diyl)bis(4,4,5,5-tetramethyl-1,3,2-dioxaborolane) (34)

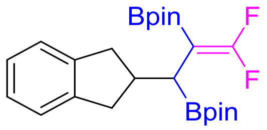

The reaction was performed following the **procedure D**. The residue was purified by flash column chromatograph (R_f = 0.4, PE: Et_2O = 10:1) to give the product as a white solid (169.6 mg, 76% yield, mp: 37.9 - 41 °C.).

^{1}H NMR (500 MHz, $CDCl_3$) δ 7.22 – 7.18 (m, 1H), 7.18 – 7.15 (m, 1H), 7.14 – 7.09 (m, 2H), 3.25 (dd, *J* = 15.7, 7.5 Hz, 1H), 2.99 – 2.80 (m, 2H), 2.65 – 2.57 (m, 2H), 2.01 (dd, *J* = 10.3, 1.9 Hz, 1H), 1.32 – 1.25 (m, 24H). **^{13}C NMR (126 MHz, $CDCl_3$)** δ 159.8 (dd, *J* = 299.6, 296.8 Hz), 144.2, 143.6, 126.0, 125.9, 124.3, 83.7, 83.4, 41.1, 39.8, 39.2, 25.0, 24.9, 24.5. **^{19}F NMR (471 MHz, $CDCl_3$)** δ -73.85 (d, *J* = 16.5 Hz), -75.87 (d, *J* = 14.6 Hz). **^{11}B NMR (128 MHz, $CDCl_3$)** δ 30.76. **HRMS (ESI)** calcd for $C_{24}H_{35}B_2F_2O_4$ [M+H]$^+$: 447.2684, found: 447.2684.

2,2'-(3,3-difluoro-1-(1-phenylcyclopropyl)prop-2-ene-1,2-diyl)bis(4,4,5,5-tetramethyl-1,3,2-dioxaborolane) (35)

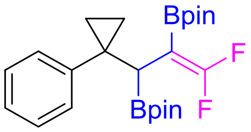

The reaction was performed following the **procedure E**. The residue was purified by flash column chromatograph (R_f = 0.4, PE: Et_2O = 10:1) to give the product as a colorless liquid (167.3 mg, 75% yield).

^{1}H NMR (500 MHz, $CDCl_3$) δ 7.43 (d, *J* = 7.8 Hz, 2H), 7.19 (t, *J* = 7.5 Hz, 2H), 7.11 (t, *J* = 7.1 Hz, 1H), 1.97 (s, 1H), 1.25 – 1.17 (m, 24H), 1.00 – 0.87 (m, 2H), 0.85 – 0.81 (m, 1H), 0.65 – 0.62 (m, 1H). **^{13}C NMR (126 MHz, $CDCl_3$)** δ 160.3 (dd, *J* = 302.3, 297.5 Hz), 145.9, 131.1, 127.6, 126.0, 83.6, 83.3, 27.3, 25.0, 25.0, 24.8, 12.7, 12.4. **^{19}F NMR (471 MHz, $CDCl_3$)** δ -70.68 (d, *J* = 11.2 Hz), -75.73 (d, *J* = 11.1 Hz). **^{11}B NMR (128 MHz, $CDCl_3$)** δ 30.50. **HRMS (ESI)** calcd for $C_{24}H_{35}B_2F_2O_4$ [M+H]$^+$: 447.2684, found: 447.2680.

2,2'-(5-cyclopentyl-1,1-difluoropent-1-ene-2,3-diyl)bis(4,4,5,5-tetramethyl-1,3,2-dioxaborolane) (36)

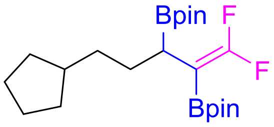

The reaction was performed following the **procedure D**. The residue was purified by flash column

chromatograph (Rf = 0.4, PE: Et_2O = 10:1) to give the product as a colorless liquid (170.5 mg, 80% yield).

^{1}H NMR (500 MHz, CDCl$_3$) δ 1.85 – 1.59 (m, 5H), 1.56 – 1.51 (m, 2H), 1.48 – 1.40 (m, 3H), 1.30 – 1.15 (m, 26H), 1.12 – 0.95 (m, 2H). **^{13}C NMR (126 MHz, CDCl$_3$)** δ 159.7 (dd, *J* = 299.8, 296.1 Hz), 83.6, 83.3, 40.2, 35.7, 33.1, 32.7, 29.1, 25.3, 25.3, 25.1, 24.9, 24.8, 24.4. **^{19}FNMR (471 MHz, CDCl$_3$)** δ -72.32 (d, *J* = 16.8 Hz), -73.30 (d, *J* = 16.7 Hz). **^{11}B NMR (128 MHz, CDCl$_3$)** δ 30.38. **HRMS (ESI)** calcd for $C_{22}H_{39}B_2F_2O_4$ $[M+H]^+$: 427.2997, found: 427.2994.

2,2'-(1-(4,4-difluorocyclohexyl)-3,3-difluoroprop-2-ene-1,2-diyl)bis(4,4,5,5-tetramethyl-1,3,2-dioxaborolane) (37)

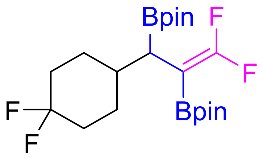

The reaction was performed following the **procedure D**. The residue was purified by flash column chromatograph (Rf = 0.5, PE: Et_2O = 10:1) to give the product as a colorless liquid (168.1 mg, 75% yield).

^{1}H NMR (500 MHz, CDCl$_3$) δ 2.07 – 1.96 (m, 2H), 1.91 (d, *J* = 13.2 Hz, 1H), 1.82 – 1.58 (m, 5H), 1.32 – 1.14 (m, 26H). **^{13}C NMR (126 MHz, CDCl$_3$)** δ 159.7 (dd, *J* = 300.7, 297.1 Hz), 123.9 (t, *J* = 240.6 Hz), 83.7, 83.4, 36.1, 33.8 (dd, *J* = 25.2, 22.6 Hz), 29.0 (d, *J* = 9.5 Hz), 28.1 (d, *J* = 9.2 Hz), 25.0, 24.8, 24.8, 24.5. **^{19}F NMR (471 MHz, CDCl$_3$)** δ -72.37, -74.71 (d, *J* = 14.4 Hz), -94.04 (d, *J* = 233.0 Hz), -104.21 (d, *J* = 231.1 Hz). **^{11}B NMR (128 MHz, CDCl$_3$)** δ 30.45. **HRMS (ESI)** calcd for $C_{21}H_{35}B_2F_4O_4$ $[M+H]^+$: 449.2652, found: 449.2655.

2,2'-(4-((3r,5r,7r)-adamantan-1-yl)-1,1-difluorobut-1-ene-2,3-diyl)bis(4,4,5,5-tetramethyl-1,3,2-dioxaborolane) (38)

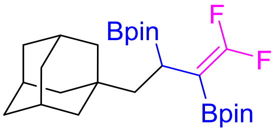

The reaction was performed following the **procedure D**. The residue was purified by flash column chromatograph (Rf = 0.4, PE: Et_2O = 10:1) to give the product as a colorless liquid (162.6 mg, 68% yield).

^{1}H NMR (500 MHz, CDCl$_3$) δ 2.01 – 1.93 (m, 1H), 1.89 (s, 3H), 1.68 – 1.52 (m, 7H), 1.49 (d, *J* = 12.2 Hz, 3H), 1.41 (d, *J* = 12.2 Hz, 3H), 1.26 – 1.17 (m, 24H). **^{13}C NMR (126 MHz, CDCl$_3$)** δ 158.5 (dd, *J* = 300.2, 295.7 Hz), 83.6, 83.3, 44.2, 42.6, 37.3, 33.3, 28.9, 25.1, 24.8, 24.7, 24.5. **^{19}F NMR (471 MHz, CDCl$_3$)** δ -71.69 (d, *J* = 18.1 Hz), -74.40 (d, *J* = 18.4 Hz). **^{11}B NMR (128 MHz, CDCl$_3$)** δ 33.13, 30.53. **HRMS (ESI)** calcd for $C_{26}H_{43}B_2F_2O_4$ $[M+H]^+$: 479.3310, found: 479.3307.

2,2'-((3S,4R)-1,1-difluoro-4-(6-methoxynaphthalen-2-yl)pent-1-ene-2,3-diyl)bis(4,4,5,5-tetramethyl-1,3,2-dioxaborolane) (39)

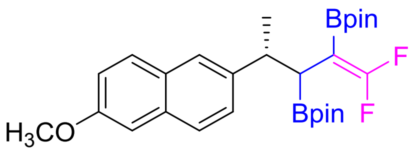

The reaction was performed following the **procedure E**. The residue was purified by flash column chromatograph (Rf = 0.3, PE: Et_2O = 1:1) to give the product as a white solid (167.1 mg, 65% yield, mp: 31.5 - 33 °C.).

^{1}H NMR (500 MHz, $CDCl_3$) δ 7.61 (dd, *J* = 9.1, 4.9 Hz, 2H), 7.49 (s, 1H), 7.29 (d, *J* = 8.4 Hz, 1H), 7.08 (dd, *J* = 7.0, 2.4 Hz, 2H), 3.88 (s, 3H), 3.47 – 3.33 (m, 1H), 2.28 (d, *J* = 10.6 Hz, 1H), 1.38 (d, *J* = 6.8 Hz, 3H), 1.27 – 1.26 (m, 12H), 1.18 – 1.16 (m, 12H). **^{13}C NMR (126 MHz, $CDCl_3$)** δ 159.6 (dd, *J* = 300.6, 297.9 Hz), 157.1, 142.7, 133.2, 129.1, 126.7, 126.4, 125.8, 118.4, 105.7, 83.4, 55.4, 39.7, 24.9, 24.8, 24.8, 24.7, 23.4. **^{19}F NMR (471 MHz, $CDCl_3$)** δ -69.33 (d, *J* = 14.5 Hz), -72.93 (d, *J* = 13.5 Hz). **^{11}B NMR (128 MHz, $CDCl_3$)** δ 30.39. **HRMS (ESI)** calcd for $C_{28}H_{38}B_2F_2O_5$: 515.2946, found: 515.2954.

2,2'-((3S)-1,1-difluoro-4-(4-isobutylphenyl)pent-1-ene-2,3-diyl)bis(4,4,5,5-tetramethyl-1,3,2-dioxaborolane) (40)

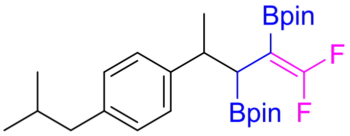

The reaction was performed following the **procedure E**. The residue was purified by flash column chromatograph (Rf = 0.4, PE: Et_2O = 10:1) to give the product as a colorless liquid (98 mg, 40% yield.).

^{1}H NMR (500 MHz, $CDCl_3$) δ 7.05 (d, *J* = 7.9 Hz, 2H), 6.98 (d, *J* = 8.0 Hz, 2H), 3.26 – 3.18 (m, 1H), 2.40 (d, *J* = 7.3 Hz, 2H), 2.16 (d, *J* = 11.3 Hz, 1H), 1.86 – 1.78 (m, 1H), 1.32 (d, *J* = 6.5 Hz, 3H), 1.28 – 1.24 (m, 12H), 1.23 – 1.19 (m, 12H), 0.87 (d, *J* = 6.6 Hz, 6H). **^{13}C NMR (126 MHz, $CDCl_3$)** δ 159.6 (dd, *J* = 300.8, 297.3 Hz), 144.6, 138.9, 128.8, 127.1, 83.4, 83.3, 45.2, 39.3, 30.3, 24.9, 24.8, 24.8, 24.7, 23.3, 22.5, 22.5. **^{19}F NMR (471 MHz, $CDCl_3$)** δ -69.09 (d, *J* = 14.1 Hz), -73.36 (d, *J* = 14.3 Hz). **^{11}B NMR (128 MHz, $CDCl_3$)** δ 30.35. **HRMS (ESI)** calcd for $C_{27}H_{43}B_2F_2O_4$ $[M+H]^+$: 491.3310, found: 491.3319.

2,2'-((3S)-1,1-difluoro-4-(2-fluoro-[1,1'-biphenyl]-4-yl)pent-1-ene-2,3-diyl)bis(4,4,5,5-tetramethyl-1,3,2-dioxaborolane) (41)

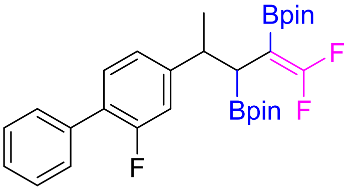

The reaction was performed following the **procedure E**. The residue was purified by flash column chromatograph (Rf = 0.4, PE: Et_2O = 10:1) to give the product as a white solid (113.5 mg, 43% yield, mp: 35.6 – 37.5 °C.).

^{1}H NMR (500 MHz, $CDCl_3$) δ 7.52 (d, *J* = 7.7 Hz, 2H), 7.41 (t, *J* = 7.7 Hz, 2H), 7.34 – 7.27 (m, 2H), 7.01 – 6.94 (m, 2H), 3.33 - 3.24 (m, 1H), 2.19 (d, *J* = 11.2 Hz, 1H), 1.34 (d, *J* = 6.9 Hz, 3H), 1.28 – 1.25

(m, 12H), 1.23 – 1.20 (m, 12H). **^{13}C NMR (126 MHz, CDCl$_3$)** δ 159.7 (dd, *J* = 302.4, 298.6 Hz), 159.7 (d, *J* = 3.6 Hz), 149.4 (d, *J* = 7.3 Hz), 136.2, 130.2 (d, *J* = 3.8 Hz), 129.1 (d, *J* = 3.2 Hz), 128.4, 127.4, 126.3 (d, *J* = 13.5 Hz), 123.5 (d, *J* = 1.9 Hz), 115.0 (d, *J* = 22.1 Hz), 83.6, 83.5, 39.5, 24.9, 24.7, 24.7, 23.1. **^{19}F NMR (471 MHz, CDCl$_3$)** δ -72.44 (d, *J* = 14.4 Hz), -119.03 (d, *J* = 10.9 Hz). **^{11}B NMR (128 MHz, CDCl$_3$)** δ 28.40. **HRMS (EI)** calcd for $C_{22}H_{34}B_2F_2O_5$: 528.2830, found: 528.2830.

(4-chlorophenyl)(3-(4,4-difluoro-2,3-bis(4,4,5,5-tetramethyl-1,3,2-dioxaborolan-2-yl)but-3-en-1-yl)-2-methyl-1H-indol-1-yl)methanone (42)

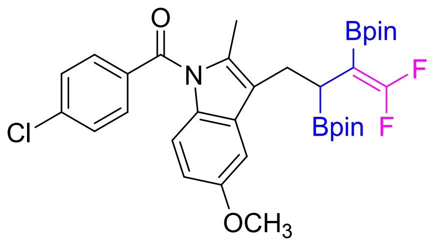

The reaction was performed following the **procedure E**. The residue was purified by flash column chromatograph (Rf = 0.25, PE: Et_2O = 4:1) to give the product as a Light yellow liquid (173.1 mg, 54% yield.).

^{1}H NMR (500 MHz, CDCl$_3$) δ 7.60 (d, *J* = 8.5 Hz, 2H), 7.44 (d, *J* = 8.4 Hz, 2H), 7.06 – 6.98 (m, 2H), 6.65 (dd, *J* = 8.9, 2.6 Hz, 1H), 3.84 (s, 3H), 3.07 (dd, *J* = 14.7, 5.4 Hz, 1H), 2.72 (dd, *J* = 14.2, 10.3 Hz, 1H), 2.33 – 2.24 (m, 1H), 2.23 (s, 3H), 1.27 – 1.17 (m, 24H) **^{13}C NMR (126 MHz, CDCl$_3$)** δ 168.4, 160.1 (dd, *J* = 301.1, 298.6 Hz), 155.9, 138.9, 134.6, 134.4, 131.5, 131.3, 131.2, 129.1, 119.7, 114.8, 110.8, 102.4, 83.7, 83.6, 55.8, 27.0, 25.1, 24.8, 24.6, 14.0. **^{19}F NMR (471 MHz, CDCl$_3$)** δ -70.75 (d, *J* = 12.5 Hz), -72.46 (d, *J* = 12.7 Hz). **^{11}B NMR (128 MHz, CDCl$_3$)** δ 30.01. **HRMS (ESI)** calcd for $C_{33}H_{41}B_2ClF_2NO_6$ $[M+H]^+$: 642.2771, found: 642.2775 .

2,2'-(3,3-difluoro-1-phenylprop-2-ene-1,2-diyl)bis(4,4,5,5-tetramethyl-1,3,2-dioxaborolane) (43)

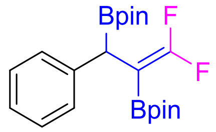

The reaction was performed following the **procedure D**. The residue was purified by flash column chromatograph (Rf = 0. 45, PE: Et_2O = 10:1) to give the product as a white solid (138.1 mg, 68% yield, mp: 38.3-40 ^{o}C.).

^{1}H NMR (500 MHz, CDCl$_3$) δ 7.31 – 7.24 (m, 2H), 7.23 – 7.17 (m, 2H), 7.14 – 7.07 (m, 1H), 3.28 (s, 1H), 1.28 – 1.16 (m, 24H). **^{13}C NMR (126 MHz, CDCl$_3$)** δ 160.2 (dd, *J* = 301.1, 298.6 Hz), 142.2, 129.4, 128.1, 125.6, 83.9, 25.0, 24.7, 24.6. **^{19}F NMR (471 MHz, CDCl$_3$)** δ -70.15 (d, *J* = 11.8 Hz), -73.18 (d, *J* = 13.0 Hz). **^{11}B NMR (128 MHz, CDCl$_3$)** δ 31.64, 30.00. **HRMS (ESI)** calcd for $C_{21}H_{31}B_2F_2O_4$ $[M+H]^+$: 407.2371, found: 407.2375.

2,2'-(3,3-difluoro-1-(p-tolyl)prop-2-ene-1,2-diyl)bis(4,4,5,5-tetramethyl-1,3,2-dioxaborolane) (44)

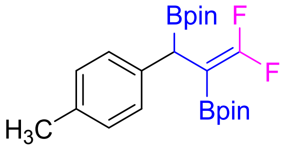

The reaction was performed following the **procedure D**. The residue was purified by flash column chromatograph (R_f = 0. 45, PE: Et_2O = 10:1) to give the product as a white solid (147.1 mg, 70% yield, mp: 46.8-48 °C.).

^{1}H NMR (500 MHz, $CDCl_3$) δ 7.19 (d, J = 7.6 Hz, 2H), 7.04 (d, *J* = 7.7 Hz, 2H), 3.24 (s, 1H), 2.28 (s, 3H), 1.28 – 1.21 (m, 24H). **^{13}C NMR (126 MHz, $CDCl_3$)** δ 160.3 (dd, *J* = 301.0, 298.0 Hz). 139.1, 134.9, 129.2, 128.9, 83.9, 83.8, 25.0, 25.0, 24.7, 24.6, 21.1 **^{19}FNMR (471 MHz, $CDCl_3$)** δ -70.45 (d, J = 11.8 Hz), -73.37 (d, J = 12.7 Hz). **^{11}B NMR (128 MHz, $CDCl_3$)** δ 30.05. **HRMS (ESI)** calcd for $C_{22}H_{33}B_2F_2O_4$ $[M+H]^+$: 421.2528, found: 421.2524.

2,2'-(1-(4-ethylphenyl)-3,3-difluoroprop-2-ene-1,2-diyl)bis(4,4,5,5-tetramethyl-1,3,2-dioxaborolane) (45)

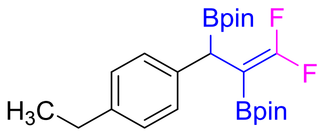

The reaction was performed following the **procedure D**. The residue was purified by flash column chromatograph (R_f = 0. 45, PE: Et_2O = 10:1) to give the product as a colorless liquid (132.4 mg, 61% yield.).

^{1}H NMR (500 MHz, $CDCl_3$) δ 7.22 (d, *J* = 7.3 Hz, 2H), 7.06 (d, *J* = 7.8 Hz, 2H), 3.25 (s, 1H), 2.69 – 2.53 (m, 2H), 1.33 – 1.17 (m, 27H). **^{13}C NMR (126 MHz, $CDCl_3$)** δ 160.2 (dd, *J* = 302.4, 299.9 Hz), 141.3, 139.3, 129.3, 127.6, 83.9, 28.5, 25.0, 25.0, 24.7, 24.6, 15.6. **^{19}F NMR (471 MHz, $CDCl_3$)** δ -70.30 (d, *J* = 12.3 Hz), -73.3 (d, *J* = 13.0 Hz). **^{11}B NMR (128 MHz, $CDCl_3$)** δ 30.69. **HRMS (ESI)** calcd for $C_{23}H_{35}B_2F_2O_4$ $[M+H]^+$: 435.2684, found: 435.2680 .

2,2'-(3,3-difluoro-1-(4-isopropylphenyl)prop-2-ene-1,2-diyl)bis(4,4,5,5-tetramethyl-1,3,2-dioxaborolane) (46)

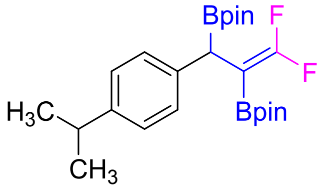

The reaction was performed following the **procedure D**. The residue was purified by flash column chromatograph (R_f = 0. 45, PE: Et_2O = 10:1) to give the product as a colorless liquid (145.7 mg, 65% yield.).

^{1}H NMR (500 MHz, $CDCl_3$) δ 7.22 (d, *J* = 7.4 Hz, 2H), 7.08 (d, *J* = 7.9 Hz, 2H), 3.25 (s, 1H), 2.91 – 2.78 (m, 1H), 1.31 – 1.20 (m, 30H). **^{13}C NMR (126 MHz, $CDCl_3$)** δ 160.2 (dd, *J* = 301.1, 298.6 Hz), 145.8, 139.3, 129.3, 126.2, 83.9, 83.8, 33.7, 25.0, 25.0, 24.7, 24.6, 24.1. **^{19}F NMR (471 MHz, $CDCl_3$)** δ -70.42 (d, *J* = 12.5 Hz), -73.41 (d, *J* = 12.7 Hz). **^{11}B NMR (128 MHz, $CDCl_3$)** δ 31.33. **HRMS (ESI)** calcd for $C_{24}H_{37}B_2F_2O_4$ $[M+H]^+$: 449.2841, found: 449.2838 .

2,2'-(1-(4-(tert-butyl)phenyl)-3,3-difluoroprop-2-ene-1,2-diyl)bis(4,4,5,5-tetramethyl-1,3,2-dioxaborolane) (47)

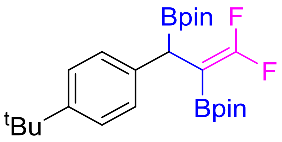

The reaction was performed following the **procedure D**. The residue was purified by flash column chromatograph (R_f = 0.5, PE: Et_2O = 10:1) to give the product as a colorless liquid (134.5 mg, 60% yield).

^{1}H NMR (500 MHz, $CDCl_3$) δ 7.24 (s, 4H), 3.26 (s, 1H), 1.32 – 1.26 (m, 24H), 1.23 (s, 9H). **^{13}C NMR (126 MHz, $CDCl_3$)** δ 160.2 (dd, J = 301.1, 297.4 Hz), 148.1, 138.9, 129.1, 125.1, 83.9, 83.8, 34.4, 31.6, 25.0, 25.0, 24.7, 24.6. **^{19}F NMR (471 MHz, $CDCl_3$)** δ -71.81 (d, J = 14.9 Hz), -74.83 (d, J = 14.4 Hz). **^{11}B NMR (128 MHz, $CDCl_3$)** δ 30.53. **HRMS (ESI)** calcd for $C_{25}H_{39}B_2F_2O_4$ $[M+H]^+$: 463.2997, found: 463.2994.

(S)-2,2'-(3,3-difluoro-1-(4-methoxyphenyl)prop-2-ene-1,2-diyl)bis(4,4,5,5-tetramethyl-1,3,2-dioxaborolane) (48)

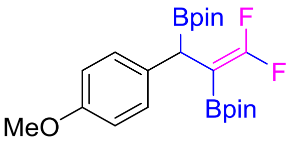

The reaction was performed following the **procedure D**. The residue was purified by flash column chromatograph (R_f = 0.4, PE: Et_2O = 10:1) to give the product as a colorless liquid (131.5 mg, 60% yield).

^{1}H NMR (400 MHz, $CDCl_3$) δ 7.23 (d, J = 7.5 Hz, 2H), 6.77 (d, J = 7.6 Hz, 2H), 3.76 (s, 3H), 3.22 (s, 1H), 1.30 – 1.18 (m, 24H). **^{13}C NMR (101 MHz, $CDCl_3$)** δ 160.2 (dd, J = 300.8, 297.9 Hz), 157.7, 134.3, 130.4, 113.6, 83.9, 83.9, 55.3, 25.0, 25.0, 24.7, 24.6. **^{19}F NMR (376 MHz, $CDCl_3$)** δ -70.77 (d, J = 13.1 Hz), -73.63 (d, J = 12.9 Hz). **^{11}B NMR (128 MHz, $CDCl_3$)** δ 33.26, 31.27. **HRMS (EI)** calcd for $C_{22}H_{32}B_2F_2O_5$: 436.2404, found: 436.2400.

2,2'-(3,3-difluoro-1-(4-(trifluoromethoxy)phenyl)prop-2-ene-1,2-diyl)bis(4,4,5,5-tetramethyl-1,3,2-dioxaborolane) (49)

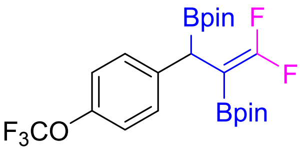

The reaction was performed following the **procedure D**. The residue was purified by flash column chromatograph (R_f = 0.4, PE: Et_2O = 10:1) to give the product as a colorless liquid (144.6 mg, 59% yield.).

^{1}H NMR (500 MHz, $CDCl_3$) δ 7.32 (d, J = 8.7 Hz, 2H), 7.07 (d, J = 7.7 Hz, 2H), 3.28 (s, 1H), 1.29 – 1.20 (m, 24H). **^{13}C NMR (126 MHz, $CDCl_3$)** δ 160.3 (t, J = 300 Hz), 147.3, 140.9, 130.7, 121.1 (q, J = 253.8 Hz), 120.6, 84.1, 84.0, 25.0, 24.7, 24.5. **^{19}F NMR (470MHz, $CDCl_3$)** δ -57.71, -70.04 (d, J = 12.6 Hz), -72.83 (d, J = 11.1 Hz). **^{11}B NMR (128 MHz, $CDCl_3$)** δ 30.74. **HRMS (ESI)** calcd for $C_{22}H_{30}B_2F_5O_5$ $[M+H]^+$: 491.2194, found: 491.2196.

2,2'-(1-(4-(benzyloxy)phenyl)-3,3-difluoroprop-2-ene-1,2-diyl)bis(4,4,5,5-tetramethyl-1,3,2-dioxaborolane) (50)

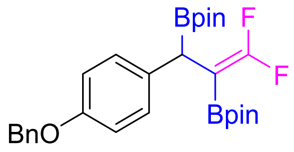

The reaction was performed following the **procedure D**. The residue was purified by flash column chromatograph (Rf = 0.35, PE: Et_2O = 10:1) to give the product as a white solid (133.2 mg, 52% yield, mp: 50.7-52.2 °C.).

^{1}H NMR (500 MHz, $CDCl_3$) δ 7.41 (d, *J* = 7.0 Hz, 2H), 7.36 (t, *J* = 7.4 Hz, 2H), 7.30 (t, *J* = 7.1 Hz, 1H), 7.26 – 7.20 (m, 2H), 6.85 (d, *J* = 8.6 Hz, 2H), 5.01 (s, 2H), 3.23 (s, 1H), 1.29 – 1.18 (m, 24H). **^{13}C NMR (126 MHz, $CDCl_3$)** δ 160.2 (dd, *J* = 301.1, 298.6 Hz), 156.9, 137.5, 134.6, 130.5, 128.6, 127.9, 127.6, 114.5, 83.9, 70.0, 25.0, 25.0, 24.7, 24.6. **^{19}F NMR (471 MHz, $CDCl_3$)** δ -72.07 (d, *J* = 14.1 Hz), -74.90 (d, *J* = 14.6 Hz). **^{11}B NMR (128 MHz, $CDCl_3$)** δ 30.95. **HRMS (EI)** calcd for $C_{28}H_{36}B_2F_2O_5$: 512.2717, found: 512.2714.

(S)-2,2'-(1-([1,1'-biphenyl]-4-yl)-3,3-difluoroprop-2-ene-1,2-diyl)bis(4,4,5,5-tetramethyl-1,3,2-dioxaborolane) (51)

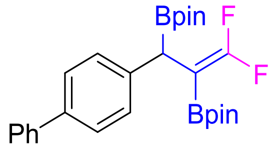

The reaction was performed following the **procedure D**. The residue was purified by flash column chromatograph (Rf = 0.4, PE: Et_2O = 10:1) to give the product as a white solid (125.3 mg, 52% yield, mp: 50.7-52.2 °C.).

^{1}H NMR (500 MHz, $CDCl_3$) δ 7.60 (dd, *J* = 8.2, 1.3 Hz, 2H), 7.50 (d, *J* = 8.3 Hz, 2H), 7.42 (q, *J* = 7.7 Hz, 4H), 7.33 (dd, *J* = 7.8, 5.8 Hz, 1H), 3.36 (s, 1H), 1.33 – 1.24 (m, 24H). **^{13}C NMR (126 MHz, CDCl3)** δ 160.3 (dd, *J* = 301.1, 298.4 Hz), 141.4, 141.3, 138.5, 129.8, 128.8, 127.1, 126.9, 84.0, 25.0, 25.0, 24.8, 24.6. **^{19}F NMR (471 MHz, $CDCl_3$)** δ -70.00 (d, *J* = 11.4 Hz), -72.91 (d, *J* = 11.2 Hz). **^{11}B NMR (128 MHz, $CDCl_3$)** δ 31.61. **HRMS (EI)** calcd for $C_{27}H_{34}B_2F_2O_4$: 482.2611, found: 482.2610.

2,2'-(3,3-difluoro-1-(4-(methylthio)phenyl)prop-2-ene-1,2-diyl)bis(4,4,5,5-tetramethyl-1,3,2-dioxaborolane) (52)

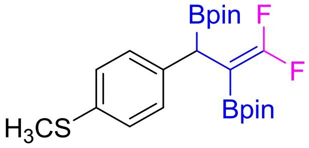

The reaction was performed following the **procedure D**. The residue was purified by flash column chromatograph (Rf = 0.4, PE: Et_2O = 10:1) to give the product as a white solid (101.7 mg, 45% yield, mp: 29.7 - 31 °C.).

^{1}H NMR (500 MHz, $CDCl_3$) δ 7.24 (d, *J* = 8.1 Hz, 2H), 7.15 (d, *J* = 8.4 Hz, 2H), 3.23 (s, 1H), 2.45 (s, 3H), 1.28 – 1.22 (m, 24H). **^{13}C NMR (126 MHz, $CDCl_3$)** δ 160.3 (dd, *J* = 302.4, 299.9 Hz), 139.4, 134.9, 129.9, 126.9, 83.9, 25.0, 25.0, 24.7, 24.6, 16.3. **^{19}F NMR (471 MHz, $CDCl_3$)** δ -70.04, -72.93 (d, *J* =

11.0 Hz). **^{11}B NMR (128 MHz, CDCl$_3$)** δ 30.22. **HRMS (EI)** calcd for $C_{22}H_{32}B_2F_2O_4S$: 452.2175, found: 452.2168.

2,2'-(3,3-difluoro-1-(furan-3-yl)prop-2-ene-1,2-diyl)bis(4,4,5,5-tetramethyl-1,3,2-dioxaborolane) (53)

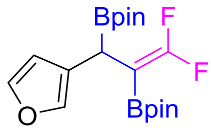

The reaction was performed following the **procedure D**. The residue was purified by flash column chromatograph (Rf = 0.4, PE: Et$_2$O = 10:1) to give the product as a white solid (128.8 mg, 65% yield, mp: 27.9 – 28.5 °C.).

^{1}H NMR (500 MHz, CDCl$_3$) δ 7.31 (s, 1H), 7.28 (t, *J* = 1.6 Hz, 1H), 6.35 (s, 1H), 3.10 (s, 1H), 1.28 – 1.26 (m, 12H), 1.24 – 1.20 (m, 12H). **^{13}C NMR (126 MHz, CDCl$_3$)** δ 160.2 (dd, *J* = 301.1, 298.6 Hz), 142.1, 139.8, 124.6, 112.1, 83.9, 83.9, 25.0, 24.7, 24.5. **^{19}F NMR (471 MHz, CDCl$_3$)** δ -71.68 (d, *J* = 12.9 Hz), -73.56 (d, *J* = 13.6 Hz). **^{11}B NMR (128 MHz, CDCl$_3$)** δ 30.35. **HRMS (ESI)** calcd for [M+H]$^+$: $C_{19}H_{29}B_2F_2O_5$ [M+H]$^+$: 397.2164, found: 397.2169.

2,2'-(3,3-difluoro-1-(thiophen-3-yl)prop-2-ene-1,2-diyl)bis(4,4,5,5-tetramethyl-1,3,2-dioxaborolane) (54)

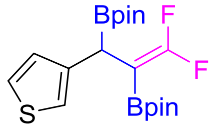

The reaction was performed following the **procedure D**. The residue was purified by flash column chromatograph (Rf = 0.45, PE: Et$_2$O = 10:1) to give the product as a white solid (138.1 mg, 67% yield, mp: 25.2-26 °C.).

^{1}H NMR (500 MHz, CDCl$_3$) δ 7.30 – 7.14 (m, 1H), 7.14 – 6.96 (m, 2H), 3.34 (s, 1H), 1.34 – 1.19 (m, 24H). **^{13}C NMR (126 MHz, CDCl$_3$)** δ 160.1 (t, *J* = 299.9 Hz), 141.6, 129.5, 124.4, 121.2, 83.9, 25.0, 24.7, 24.5. **^{19}F NMR (471 MHz, CDCl$_3$)** δ -71.27 (d, *J* = 12.8 Hz), -73.60 (d, *J* = 12.5 Hz). **^{11}B NMR (128 MHz, CDCl$_3$)** δ 30.36. **HRMS (ESI)** calcd for $C_{19}H_{29}B_2F_2O_4S$ [M+H]$^+$: 413.1935, found: 413.1932 .

2,2'-(1-(2,3-dihydrobenzofuran-5-yl)-3,3-difluoroprop-2-ene-1,2-diyl)bis(4,4,5,5-tetramethyl-1,3,2-dioxaborolane) (55)

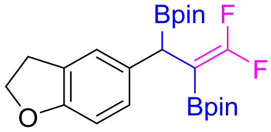

The reaction was performed following the **procedure D**. The residue was purified by flash column chromatograph (Rf = 0.35, PE: Et$_2$O = 10:1) to give the product as a white solid (130.0 mg, 58% yield, mp: 37.7 - 39 °C.).

^{1}H NMR (500 MHz, CDCl$_3$) δ 7.13 (s, 1H), 7.06 (d, *J* = 7.8 Hz, 1H), 6.65 (d, *J* = 8.2 Hz, 1H), 4.51 (t, *J* = 8.7 Hz, 2H), 3.21 (s, 1H), 3.19 – 3.09 (m, 2H), 1.29 – 1.22 (m, 24H). **^{13}C NMR (126 MHz, CDCl$_3$)**

δ 160.1 (dd, *J* = 300.5, 297.8 Hz), 158.2,134.1, 129.0, 126.6, 126.1, 108.8, 83.9, 71.2, 30.0, 25.02, 24.96, 24.7, 24.6. **^{19}F NMR (471 MHz, CDCl$_3$)** δ -70.84 (d, *J* = 13.2 Hz), -73.64 (d, *J* = 14.2 Hz). **^{11}B NMR (128 MHz, CDCl$_3$)** δ 30.58. **HRMS (EI)** calcd for $C_{23}H_{32}B_2F_2O_5$: 448.2404, found: 448.2398.

2,2'-(1-(3,4-dimethylphenyl)-3,3-difluoroprop-2-ene-1,2-diyl)bis(4,4,5,5-tetramethyl-1,3,2-dioxaborolane) (56)

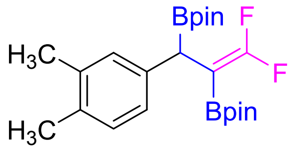

The reaction was performed following the **procedure D**. The residue was purified by flash column chromatograph (Rf = 0.4, PE: Et$_2$O = 20:1) to give the product as a colorless liquid (152.0 mg, 70% yield.).

^{1}H NMR (500 MHz, CDCl$_3$) δ 7.07 (d, *J* = 9.4 Hz, 2H), 7.01 (d, *J* = 7.2 Hz, 1H), 3.23 (s, 1H), 2.24 – 2.20 (m, 6H), 1.31 – 1.24 (m, 24H). **^{13}C NMR (126 MHz, CDCl$_3$)** δ 160.1 (t, *J* = 299.9 Hz), 139.6, 136.0, 133.6, 130.7, 129.5, 126.8, 83.9, 83.8, 25.1, 25.0, 24.7, 24.6, 19.9, 19.4. **^{19}F NMR (471 MHz, CDCl$_3$)** δ -70.26 (d, *J* = 11.1 Hz), -73.07 (d, *J* = 14.6 Hz) **^{11}B NMR (128 MHz, CDCl$_3$)** δ 30.70. **HRMS (ESI)** calcd for $C_{23}H_{35}B_2F_2O_4$ [M+H]$^+$: 435.2684, found: 435.2686.

2,2'-(1,1-difluoro-4-(4-isopropylphenyl)but-1-ene-2,3-diyl)bis(4,4,5,5-tetramethyl-1,3,2-dioxaborolane) (57)

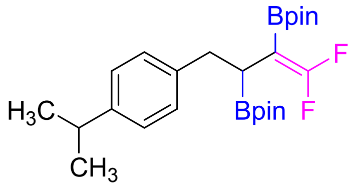

The reaction was performed following the **procedure F**. The residue was purified by flash column chromatograph (Rf = 0.45, PE: Et$_2$O = 20:1) to give the product as a colorless liquid (182.6 mg, 79% yield.).

^{1}H NMR (500 MHz, CDCl$_3$) δ 7.14 – 7.03 (m, 4H), 3.03 (d, *J* = 14.0 Hz, 1H), 2.86 (p, *J* = 6.9 Hz, 1H), 2.70 (t, *J* = 12.6 Hz, 1H), 2.19 (dd, *J* = 11.3, 4.8 Hz, 1H), 1.29 – 1.21 (m, 30H). **^{13}C NMR (126 MHz, CDCl$_3$)** δ 159.7 (dd, *J* = 300.7, 296.9 Hz), 146.0, 139.4, 128.9, 126.1, 83.5, 35.7, 33.8, 25.1, 24.9,24.8, 24.5, 24.2. **^{19}FNMR (471 MHz, CDCl$_3$)** δ -70.98 (d, *J* = 15.0 Hz), -72.92 (d, *J* = 13.5 Hz). **^{11}B NMR (128 MHz, CDCl$_3$)** δ 30.33. **HRMS (ESI)** calcd for $C_{25}H_{39}B_2F_2O_4$ [M+H]$^+$: 463.2997, found: 463.2997.

2,2'-(4-(3-chlorophenyl)-1,1-difluorobut-1-ene-2,3-diyl)bis(4,4,5,5-tetramethyl-1,3,2-dioxaborolane) (58)

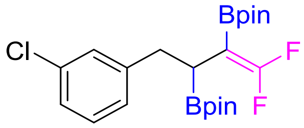

The reaction was performed following the **procedure F**. The residue was purified by flash column chromatograph (Rf = 0.4, PE: Et$_2$O = 10:1) to give the product as a white solid (163.5 mg, 72% yield, mp: 46.3-47 °C.).

^{1}H NMR (500 MHz, $CDCl_3$) δ 7.16 (d, *J* = 1.9 Hz, 1H), 7.15 – 7.09 (m, 2H), 7.00 (d, *J* = 7.3 Hz, 1H), 3.03 – 2.99 (m, 1H), 2.71 (dd, *J* = 13.9, 10.9 Hz, 1H), 2.25 – 2.07 (m, 1H), 1.27 – 1.21 (m, 24H). **^{13}C NMR (126 MHz, $CDCl_3$)** δ 159.9 (dd, *J* = 300.8, 297.9 Hz), 144.3, 133.8, 129.3, 129.2, 127.2, 125.9, 83.7, 83.7, 35.9, 25.1, 24.9, 24.8, 24.5. **^{19}F NMR (471 MHz, $CDCl_3$)** δ -70.68 (d, *J* = 13.0 Hz), -72.45 (d, *J* = 13.2 Hz). **^{11}B NMR (128 MHz, $CDCl_3$)** δ 30.18. **HRMS (ESI)** calcd for $C_{22}H_{32}B_2ClF_2O_4$ $[M+H]^+$: 455.2138, found: 455.2135 .

4-(4,4-difluoro-2,3-bis(4,4,5,5-tetramethyl-1,3,2-dioxaborolan-2-yl)but-3-en-1-yl)benzonitrile (59)

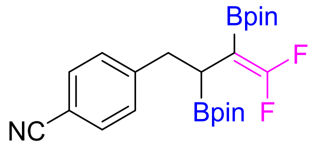

The reaction was performed following the **procedure F**. The residue was purified by flash column chromatograph (R_f = 0.3, PE: Et_2O = 10:1) to give the product as a white solid (144.7 mg, 65% yield, mp: 81.3-82.6 °C.).

^{1}H NMR (500 MHz, $CDCl_3$) δ 7.52 (d, *J* = 8.3 Hz, 2H), 7.24 (d, *J* = 8.2 Hz, 2H), 3.11 – 3.04 (m, 1H), 2.79 (dd, *J* = 13.8, 11.1 Hz, 1H), 2.23 – 2.12 (m, 1H), 1.27 – 1.23 (m, 24H). **^{13}C NMR (126 MHz, $CDCl_3$)** δ 159.9 (dd, *J* = 299.9, 298.6 Hz), 148.1, 132.0, 129.8, 119.4, 109.6, 83.9, 83.8, 36.4, 25.1, 25.0, 24.8, 24.5. **^{19}F NMR (471 MHz, $CDCl_3$)** δ -70.51 (d, *J* = 12.4 Hz), -72.12 (d, *J* = 12.4 Hz). **^{11}B NMR (128 MHz, $CDCl_3$)** δ 33.02. **HRMS (ESI)** calcd for $C_{23}H_{32}B_2F_2NO_4$ $[M+H]^+$: 446.2480, found: 446.2483.

(S)-2,2'-(1,1-difluoro-5-phenylpent-1-ene-2,3-diyl)bis(4,4,5,5-tetramethyl-1,3,2-dioxaborolane) (60)

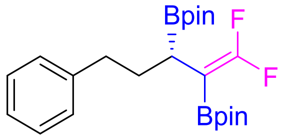

The reaction was performed following the **procedure G**. The residue was purified by flash column chromatograph (Rf = 0.4, PE: Et_2O = 10:1) to give the product as a white solid (105.2 mg, 81% yield, m p: 45.3 - 47 °C.).

^{1}H NMR (500 MHz, $CDCl_3$) δ 7.27 – 7.22 (m, 2H), 7.19 – 7.11 (m, 3H), 2.68 – 2.58 (m, 1H), 2.53 – 2.43 (m, 1H), 2.03 – 1.87 (m, 2H), 1.85 – 1.74 (m, 1H), 1.49 – 0.81 (m, 24H). **^{13}C NMR (126 MHz, $CDCl_3$)** δ 159.8 (dd, *J* = 299.7, 296.7 Hz), 143.08, 128.6, 128.3, 125.6, 83.7, 83.41, 35.4, 32.3, 25.1, 24.9, 24.8, 24.4. **^{19}F NMR (470MHz, $CDCl_3$)** δ -71.58 (d, *J* = 16.2 Hz), -72.69 (d, *J* = 16.1 Hz). **^{11}B NMR (128 MHz, $CDCl_3$)** δ 30.28. **HRMS (ESI)** calcd for $C_{23}H_{35}B_2H_2O_4$ $[M+H]^+$: 435.2684, found: 435.2680. **HPLC analysis**: DAICEL CHIRALCEL OZ-3, hexane/isopropanol = 99.9/0.1, 0.5mL/min, λ = 210 nm, t_R (major) = 10.6 min, t_R (minor) = 12.6 min, 96.5:3.5 er **$[\alpha]^{25}_D$**: +13.6 (*c* 0.5, $CHCl_3$).

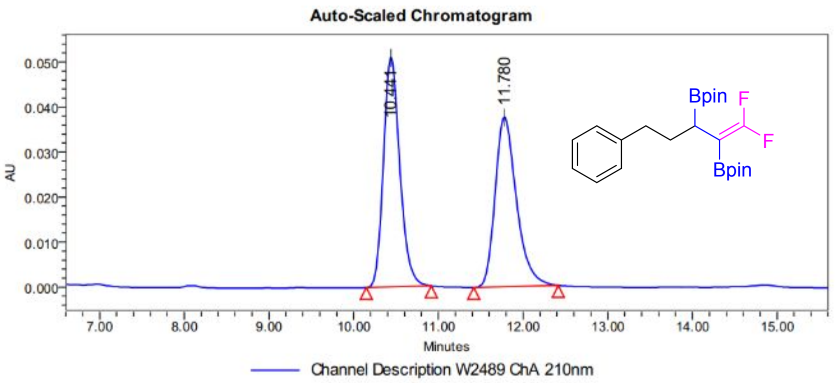

Peak Results

	Retention Time (min)	Int Type	Width (sec)	Area (μV*sec)	Height (μV)	% Area
1	10.441	bb	46.000	685368	51056	50.97
2	11.780	Bb	60.000	659263	37615	49.03

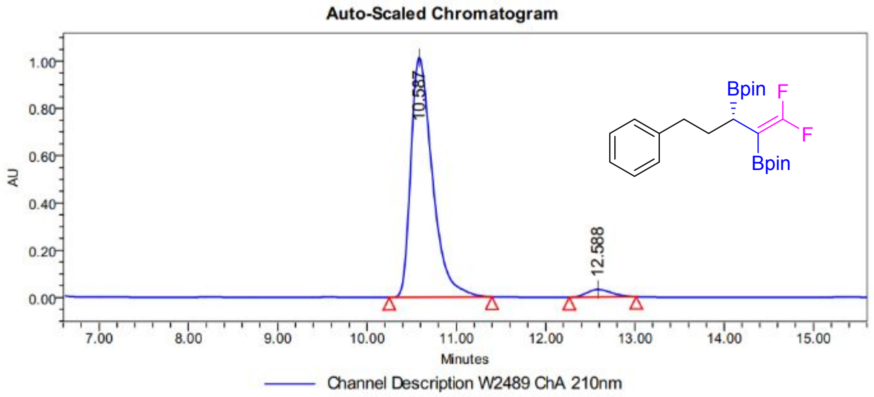

Peak Results

	Retention Time (min)	Int Type	Width (sec)	Area (μV*sec)	Height (μV)	% Area
1	10.587	bb	69.000	16986706	1015296	96.51
2	12.588	bb	45.000	613603	31536	3.49

(S)-2,2'-(1,1-difluoro-5-(p-tolyl)pent-1-ene-2,3-diyl)bis(4,4,5,5-tetramethyl-1,3,2-dioxaborolane) (61)

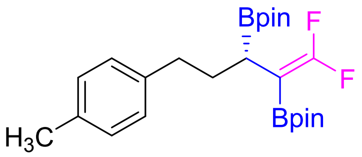

The reaction was performed following the Condition B. The residue was purified by flash column chromatograph (Rf = 0.4, PE: Et_2O = 10:1) to give the product as a white solid (98.1 mg, 73% yield, mp: 40.8-42 ^{o}C.).

^{1}H NMR (500 MHz, $CDCl_3$) δ 7.08 (s, 4H), 2.66 – 2.56 (m, 1H), 2.52 – 2.42 (m, 1H), 2.32 (s, 3H), 2.03 – 1.88 (m, 2H), 1.83 -1.73 (m, 1H), 11.6, 4.5 Hz, 1H), 1.32 – 1.22 (m, 24H). **^{13}C NMR (126 MHz, $CDCl_3$)** δ 159.8 (t, J = 296.3 Hz), 139.9, 134.9, 129.0, 128.5, 83.6, 83.4, 35.0, 32.4, 25.1, 25.0, 24.8, 24.5, 21.1. **^{19}F NMR (470 MHz, $CDCl_3$)** δ -71.69 (d, J = 15.7 Hz), -72.84 (d, J = 18.2 Hz). **^{11}B NMR (128 MHz, $CDCl_3$)** δ 32.92. **HRMS (ESI)** calcd for $C_{24}H_{37}B_2F_2O_4^+$ $[M+H]^+$: 449.2841, found: 449.2846. **HPLC analysis**: DAICEL CHIRALCEL OD-H, hexane/isopropanol = 99.9/0.1, 0.5mL/min, λ = 210 nm, t_R (major) = 9.4 min, t_R (minor) = 10.3 min, 94:6 er. **$[\alpha]^{25}_D$**: +10.0 (c 0.5, $CHCl_3$).

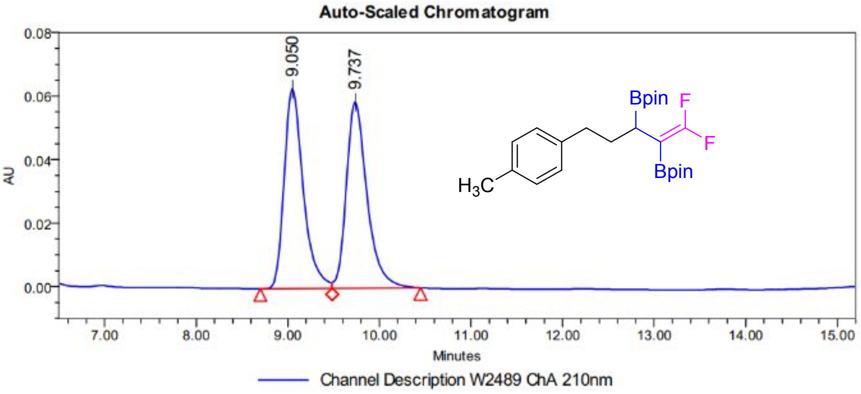

Peak Results

	Retention Time (min)	Int Type	Width (sec)	Area (μV*sec)	Height (μV)	% Area
1	9.050	BV	47.000	911086	62810	49.73
2	9.737	VB	58.000	920852	58648	50.27

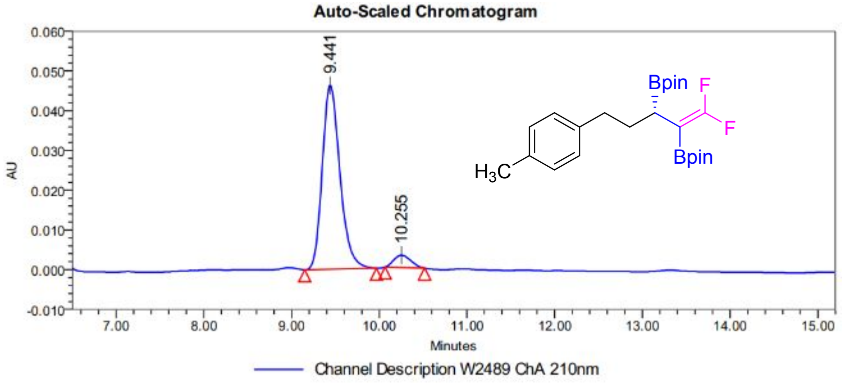

Peak Results

	Retention Time (min)	Int Type	Width (sec)	Area (μV*sec)	Height (μV)	% Area
1	9.441	bb	49.000	648729	46372	93.96
2	10.255	bb	27.000	41670	3126	6.04

(S)-2,2'-(1,1-difluoro-5-(4-methoxyphenyl)pent-1-ene-2,3-diyl)bis(4,4,5,5-tetramethyl-1,3,2-dioxaborolane) (62)

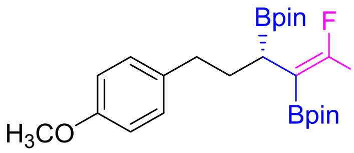

The reaction was performed following the **procedure G**. The residue was purified by flash column chromatograph (Rf = 0.35, PE: Et_2O = 10:1) to give the product as a white solid (97.4 mg, 74% yield, mp: 47.3 – 58.6 °C.).

^{1}H NMR (500 MHz, $CDCl_3$) 7.08 (d, J = 8.6 Hz, 2H), 6.80 (d, J = 8.6 Hz, 2H), 3.77 (s, 3H), 2.61 – 2.55 (m, 1H), 2.46 – 2.40 (m, 1H), 2.01 – 1.85 (m, 2H), 1.83 – 1.71 (m, 1H), 1.31 – 1.19 (m, 24H). **^{13}C NMR (126 MHz, $CDCl_3$)** δ 159.7 (dd, J = 301.1, 297.4 Hz), 157.6, 135.1, 129.4, 113.7, 83.6, 83.4, 55.3, 34.4, 32.5, 25.1, 24.9, 24.8, 24.4. **^{19}F NMR NMR (470 MHz, $CDCl_3$)** δ -71.66 (d, J = 16.2 Hz), -72.80 (d, J = 16.1 Hz). **^{11}B NMR (128 MHz, $CDCl_3$)** δ 30.83. **HRMS (ESI)** calcd for $C_{24}H_{37}B_2F_2O_5$ $[M+H]^+$: 465.2790, found: 465.2787. **HPLC analysis**: DAICEL CHIRALCEL OD-H, hexane/isopropanol = 99.8/0.2, 0.5mL/min, λ = 210 nm, t_R (major) = 14.2 min, t_R (minor) = 15.5 min, 94:6 er. **$[\alpha]^{25}_D$**: +9.8 (*c*

0.5, $CHCl_3$).

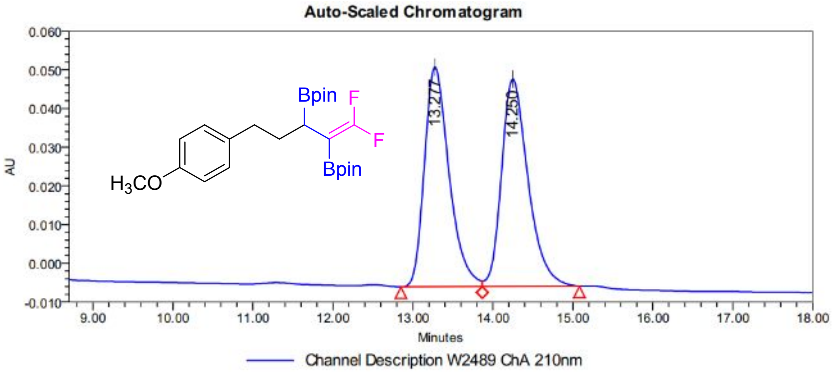

Peak Results

	Retention Time (min)	Int Type	Width (sec)	Area (µV*sec)	Height (µV)	% Area
1	13.277	BV	61.000	1215852	56752	49.57
2	14.250	VB	73.000	1237145	53628	50.43

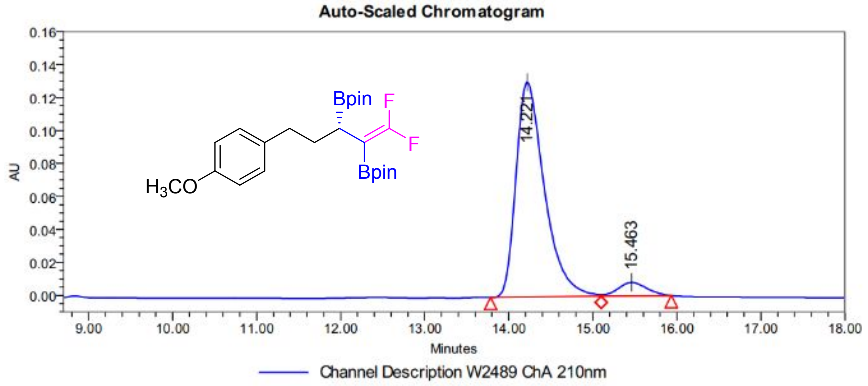

Peak Results

	Retention Time (min)	Int Type	Width (sec)	Area (µV*sec)	Height (µV)	% Area
1	14.221	bV	79.000	3068157	130436	94.01
2	15.463	Vb	50.000	195659	8204	5.99

(S)-2,2'-(1,1-difluoro-5-(4-fluorophenyl)pent-1-ene-2,3-diyl)bis(4,4,5,5-tetramethyl-1,3,2-dioxaborolane) (63)

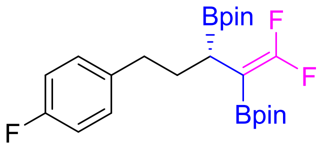

The reaction was performed following the **procedure G**. The residue was purified by flash column chromatograph (R_f = 0.4, PE: Et_2O = 10:1) to give the product as a white solid (108.5 mg, 80% yield, mp: 38.5-40 °C.).

^{1}H NMR (500 MHz, $CDCl_3$) 7.10 (dd, J = 8.5, 5.6 Hz, 2H), 6.92 (t, J = 8.8 Hz, 2H), 2.63 -2.57 (m, 1H), 2.48 -2.42 (m, 1H), 2.03 – 1.83 (m, 2H), 1.82 – 1.70 (m, 1H), 1.29 – 1.21 (m, 24H). **^{13}C NMR (126 MHz, $CDCl_3$)** 161.2 (d, J = 242.6 Hz), 159.8 (dd, J = 299.9, 297.4Hz) 138.5 (d, J = 3.7 Hz), 129.9 (d, J = 7.3 Hz), 114.9 (d, J = 21.4 Hz), 83.7, 83.5, 34.5, 32.4, 25.1, 24.9, 24.8, 24.4. **^{19}F NMR (470 MHz, $CDCl_3$)** δ -71.57 (d, J = 15.0 Hz), -72.63 (d, J = 14.8 Hz), -118.22. **^{11}B NMR (128 MHz, $CDCl_3$)** δ 30.62 **HRMS (EI)** calcd for $C_{23}H_{33}B_2F_3O_4$: 452.2517, found: 452.2508. **HPLC analysis**: DAICEL CHIRALCEL IB N-3, hexane/isopropanol = 99.9/0.1, 0.5mL/min, λ = 210 nm, t_R (major) = 8.8 min, t_R

(minor) = 9.2 min, 90:10 er. **[α]$^{25}_{D}$**: +10.0 (*c* 0.5, CHCl3).

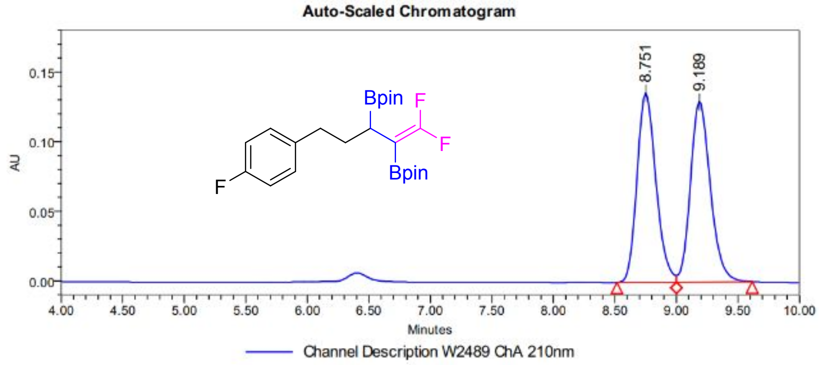

Peak Results

	Retention Time (min)	Int Type	Width (sec)	Area (μV*sec)	Height (μV)	% Area
1	8.751	bV	29.000	1458100	135778	50.00
2	9.189	Vb	37.000	1457948	129811	50.00

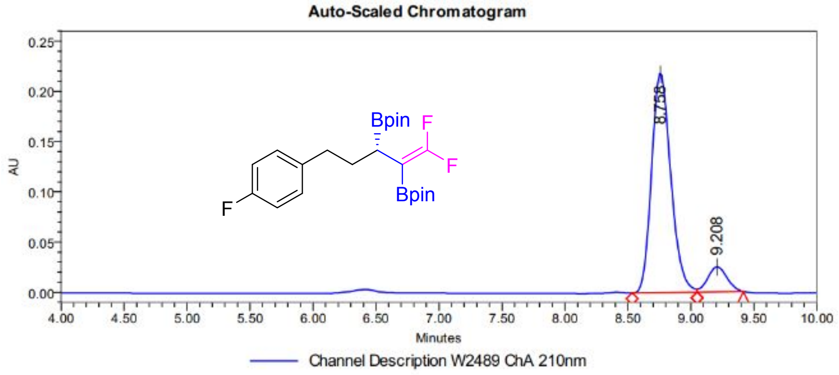

Peak Results

	Retention Time (min)	Int Type	Width (sec)	Area (μV*sec)	Height (μV)	% Area
1	8.758	VV	31.000	2322403	218918	89.81
2	9.208	Vb	22.000	263619	24935	10.19

(S)-2,2'-(5-(4-chlorophenyl)-1,1-difluoropent-1-ene-2,3-diyl)bis(4,4,5,5-tetramethyl-1,3,2-dioxaborolane) (64)

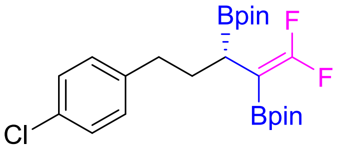

The reaction was performed following the **procedure G**. The residue was purified by flash column chromatograph (Rf = 0.4, PE: Et_2O = 10:1) to give the product as a white solid (91.3 mg, 72% yield, mp: 45.2-47.5 °C.).

^{1}H NMR (500 MHz, $CDCl_3$) δ 7.21 (d, *J* = 8.4 Hz, 2H), 7.08 (d, *J* = 8.3 Hz, 2H), 2.63 – 2.57 (m, 1H), 2.49 - 2.43 (m, 1H), 2.01 – 1.84 (m, 2H), 1.80 -1.72 (m, 1H), 1.28 – 1.22 (m, 24H). **^{13}C NMR (126 MHz, $CDCl_3$)** δ 159.8 (dd, *J* = 297.5, 295.0 Hz), 141.4, 131.3, 130.0, 128.3, 83.7, 83.5, 34.7, 32.1, 25.1, 24.9, 24.78, 24.45 **^{19}F NMR (471 MHz, $CDCl_3$)** δ -71.57 (d, *J* = 18.1 Hz), -72.62 (d, *J* = 14.7 Hz). **^{11}B NMR (128 MHz, $CDCl_3$)** δ 30.62. **HRMS (ESI)** calcd for: $C_{23}H_{34}B_2ClF_2O_4$ $[M+H]^+$: 429.2294, found: 429.2290. **HPLC analysis**: DAICEL CHIRALCEL OD-H, hexane/isopropanol = 99.9/0.1, 0.5mL/min, λ = 210 nm, t_R (major) = 8.8 min, t_R (minor) = 9.4 min, 97:3 er. **[α]$^{25}_{D}$**: +13.4 (*c* 0.5, CHCl3).

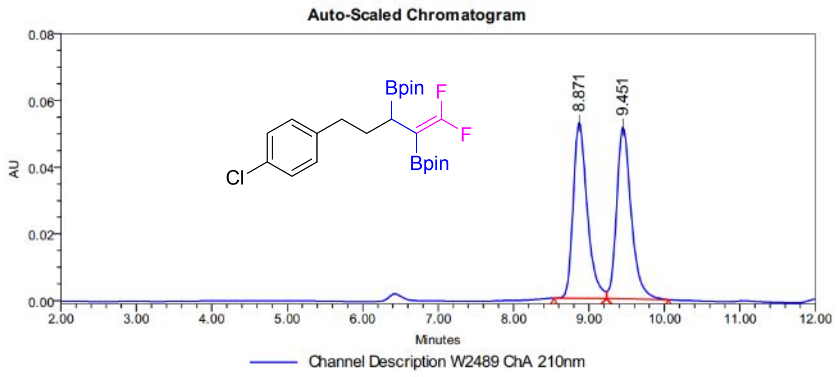

Peak Results

	Retention Time (min)	Int Type	Width (sec)	Area (μV*sec)	Height (μV)	% Area
1	8.871	BV	42.000	681956	52700	49.69
2	9.451	VB	49.000	690417	51509	50.31

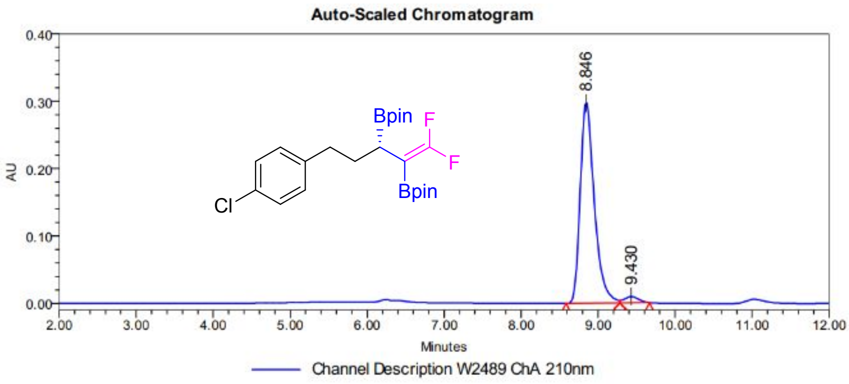

Peak Results

	Retention Time (min)	Int Type	Width (sec)	Area (μV*sec)	Height (μV)	% Area
1	8.846	bV	42.000	3785167	297859	97.01
2	9.430	Vb	23.000	116773	9276	2.99

(S)-2,2'-(5-(4-bromophenyl)-1,1-difluoropent-1-ene-2,3-diyl)bis(4,4,5,5-tetramethyl-1,3,2-dioxaborolane) (65)

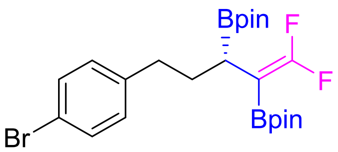

The reaction was performed following the **procedure G**. The residue was purified by flash column chromatograph (R_f = 0.4, PE: Et_2O = 10:1) to give the product as a white solid (104.4 mg, 68% yield, mp: 28.7 - 30 °C.).

^{1}H NMR (500 MHz, $CDCl_3$) δ 7.4 (d, J = 8.3 Hz, 2H), 7.0 (d, J = 8.3 Hz, 2H), 2.6 – 2.5 (m, 1H), 2.5 – 2.4 (m, 1H), 2.0 – 1.8 (m, 2H), 1.79 – 1.71 (m, 1H), 1.3 – 1.2 (m, 24H). **^{13}C NMR (126 MHz, $CDCl_3$)** δ 159.8 (dd, J = 300.4, 297.0 Hz), 141.9, 131.3, 130.4, 119.3, 83.7, 83.5, 34.7, 32.1, 25.2, 24.9, 24.8, 24.4. **^{19}F NMR (470 MHz, $CDCl_3$)** δ -71.45 (d, J = 14.1 Hz), -72.51 (d, J = 18.8 Hz). **^{11}B NMR (128 MHz, $CDCl_3$)** δ 30.53. **HRMS (EI)** calcd for $C_{23}H_{33}B_2BrF_2O_4$: 512.1716, found: 512.1714. **HPLC analysis**: DAICEL CHIRALCEL OD-H, hexane/isopropanol = 99.9/0.1, 0.5mL/min, λ = 210 nm, t_R (major) = 10.3 min, t_R (minor) = 11.0 min, 96:4 er. **$[\alpha]^{25}_D$**: +4.3 (c 0.5, $CHCl_3$).

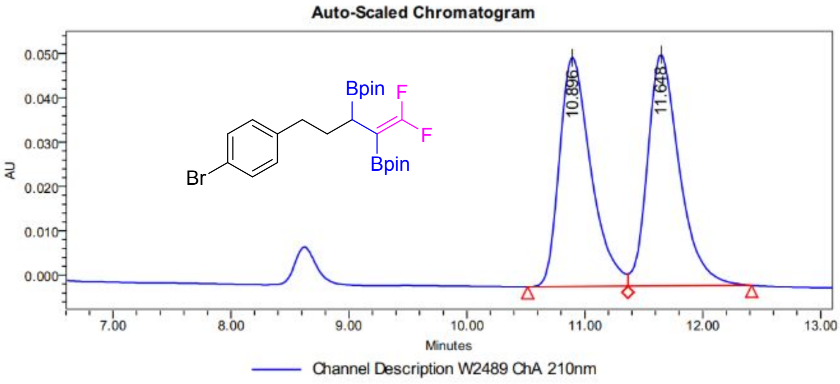

Peak Results

	Retention Time (min)	Int Type	Width (sec)	Area (μV*sec)	Height (μV)	% Area
1	10.896	BV	51.000	958364	51699	49.34
2	11.648	VB	63.000	983855	52189	50.66

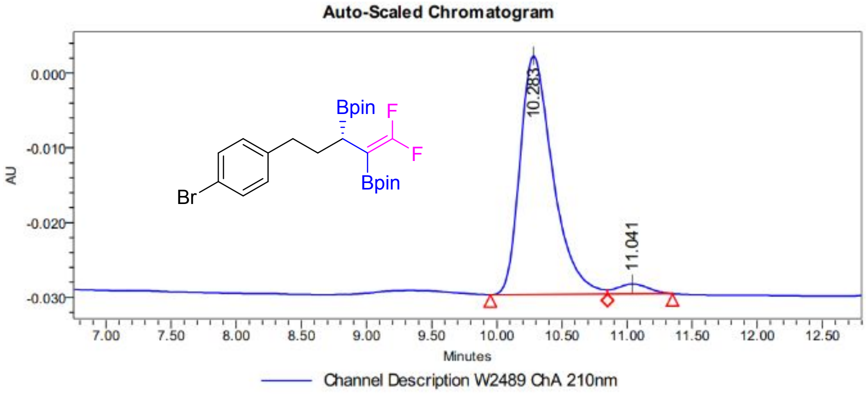

Peak Results

	Retention Time (min)	Int Type	Width (sec)	Area (μV*sec)	Height (μV)	% Area
1	10.283	BV	54.000	562001	31945	96.20
2	11.041	VB	30.000	22196	1331	3.80

(S)-4-(5,5-difluoro-3,4-bis(4,4,5,5-tetramethyl-1,3,2-dioxaborolan-2-yl)pent-4-en-1-yl)phenyl acetate (66)

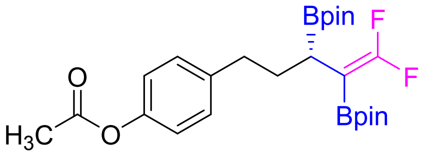

The reaction was performed following the **procedure G**. The residue was purified by flash column chromatograph (R_f = 0.3, PE: Et_2O = 10:1) to give the product as a white solid (110.7 mg, 75% yield, mp: 52.1-53.5 °C.).

^{1}H NMR (500 MHz, $CDCl_3$) δ 7.16 (d, J = 8.4 Hz, 2H), 6.95 (d, J = 8.4 Hz, 2H), 2.64 – 2.59 (m, 1H), 2.50 – 2.45 (m, 1H), 2.27 (s, 3H), 2.01 – 1.87 (m, 2H), 1.83 – 1.73 (m, 1H), 1.27 – 1.22 (m, 24H). **^{13}C NMR (126 MHz, $CDCl_3$)** δ 169.8, 159.8 (dd, J = 300.0, 296.9 Hz), 148.7, 140.6, 129.5, 121.3, 83.7, 83.5, 34.8, 32.3, 25.1, 25.0, 24.8, 24.5, 21.3. **^{19}F NMR (471 MHz, $CDCl_3$)** δ -72.13 (dd, J = 544.4, 15.3 Hz). **^{11}B NMR (128 MHz, $CDCl_3$)** δ 28.82. **HRMS (ESI)** calcd for $C_{25}H_{37}B_2F_2O_6$ $[M+H]^+$: 493.2739, found: 493.2742. **HPLC analysis**: DAICEL CHIRALCEL OZ-3, hexane/isopropanol = 99.8/0.2, 0.5mL/min, λ = 210 nm, t_R (major) = 20.0 min, t_R (minor) = 23.9 min, 93:7 er. **$[\alpha]^{25}_D$**:-8.5 (c 0.5, $CHCl_3$).

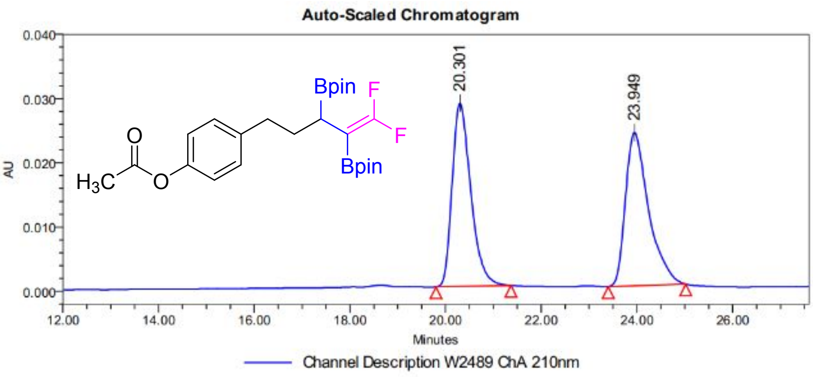

Peak Results

	Retention Time (min)	Int Type	Width (sec)	Area (μV*sec)	Height (μV)	% Area
1	20.301	Bb	94.000	772141	28530	49.07
2	23.949	BB	97.000	801432	23800	50.93

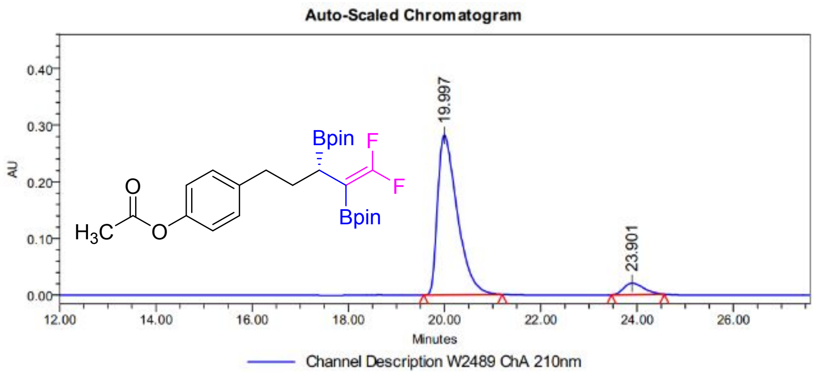

Peak Results

	Retention Time (min)	Int Type	Width (sec)	Area (μV*sec)	Height (μV)	% Area
1	19.997	bb	98.000	8020011	281941	93.09
2	23.901	bb	66.000	595469	20316	6.91

(S)-2,2'-(4-(4-bromophenyl)-1,1-difluorobut-1-ene-2,3-diyl)bis(4,4,5,5-tetramethyl-1,3,2-dioxaborolane) (67)

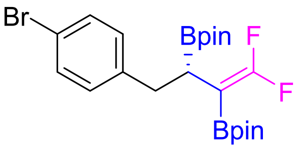

The reaction was performed following the **procedure G**. The residue was purified by flash column chromatograph (Rf = 0.4, PE: Et_2O = 10:1) to give the product as a white solid (116.5 mg, 78% yield, mp: 46.7 – 47.5 °C.).

^{1}H NMR (500 MHz, $CDCl_3$) δ 7.33 (d, *J* = 8.3 Hz, 2H), 7.00 (d, *J* = 8.3 Hz, 2H), 2.98 (ddd, *J* = 13.9, 4.9, 1.9 Hz, 1H), 2.68 (dd, *J* = 13.9, 11.2 Hz, 1H), 2.14 (ddd, *J* = 11.0, 4.8, 1.3 Hz, 1H), 1.26 – 1.22 (m, 24H) 7.33 (d, *J* = 8.3 Hz, 2H), 7.00 (d, *J* = 8.3 Hz, 2H), 3.00 – 2.96 (m, 1H), 2.68 (dd, *J* = 13.9, 11.2 Hz, 1H), 2.16 – 2.12 (m, 1H), 1.26 – 1.22 (m, 24H). **^{13}C NMR (126 MHz, $CDCl_3$)** δ 159.9 (dd, *J* = 300.9, 298.2 Hz), 141.2, 131.1, 130.8, 119.5, 83.7, 83.7, 35.6, 25.1, 25.0, 24.8, 24.5. **^{19}F NMR (471 MHz, $CDCl_3$)** δ -71.95 (d, *J* = 13.6 Hz), -73.64 (d, *J* = 13.0 Hz). **^{11}B NMR (128 MHz, $CDCl_3$)** δ 30.50. **HRMS (ESI)** calcd for $C_{22}H_{32}B_2BrF_2O_4$ $[M+H]^+$: 499.1633, found 499.1629. **HPLC analysis**: DAICEL CHIRALCEL OD-H hexane/isopropanol = 99.9/0.1, 0.5mL/min, λ = 210 nm, t_R (major) = 9.5 min, t_R

(minor) = 10.2 min, 95.5:4.5 er. **[α]$^{25}_{D}$**: 40.2 (*c* 0.5, CHCl3).

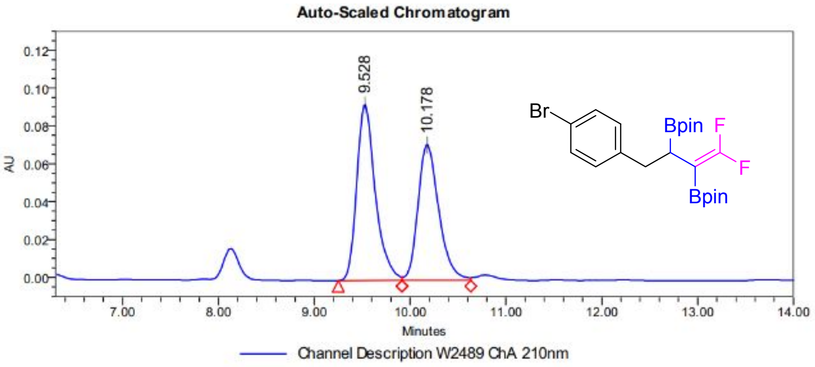

Peak Results

	Retention Time (min)	Int Type	Width (sec)	Area (µV*sec)	Height (µV)	% Area
1	9.528	bV	40.000	1254881	92950	53.70
2	10.178	VV	43.000	1081802	71717	46.30

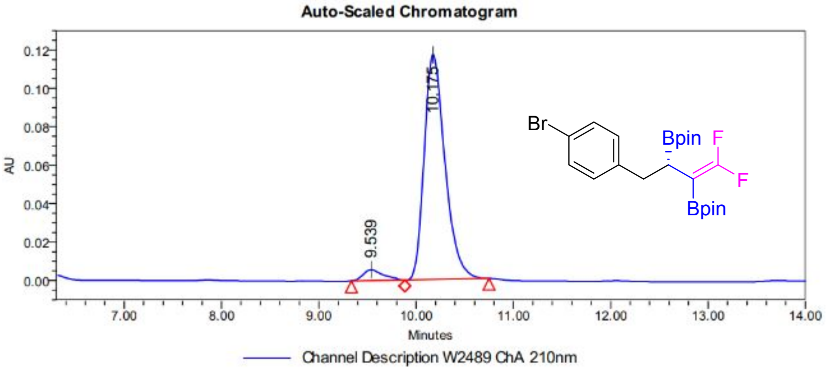

Peak Results

	Retention Time (min)	Int Type	Width (sec)	Area (µV*sec)	Height (µV)	% Area
1	9.539	bV	33.000	81888	5686	4.52
2	10.175	Vb	52.000	1729194	117351	95.48

(S)-2,2'-(5-(3-bromophenyl)-1,1-difluoropent-1-ene-2,3-diyl)bis(4,4,5,5-tetramethyl-1,3,2-dioxaborolane) (68)

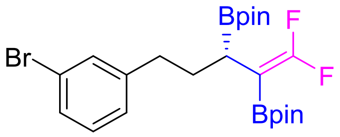

The reaction was performed following the **procedure G**. The residue was purified by flash column chromatograph (Rf = 0.4, PE: Et_2O = 20:1) to give the product as a white solid (133.6 mg, 87% yield, mp: 35.1-36 °C.).

^{1}H NMR (500 MHz, $CDCl_3$) δ 7.32 (s, 1H), 7.28 (d, *J* = 7.7 Hz, 1H), 7.13 – 7.06 (m, 2H), 2.65 – 2.54 (m, 1H), 2.51 – 2.41 (m, 1H), 2.02 – 1.85 (m, 2H), 1.82 – 1.68 (m, 1H), 1.29 – 1.22 (m, 24H). **^{13}C NMR (126 MHz, $CDCl_3$)** δ 159.8 (dd, *J* = 300.2, 297.3 Hz), 145.4, 131.7, 129.9, 128.7, 127.3, 122.4, 83.7, 83.5, 35.1, 32.1, 25.1, 24.9, 24.8, 24.5. **^{19}F NMR (471 MHz, $CDCl_3$)** δ -72.85 (d, *J* = 15.1 Hz), -73.92 (d, *J* = 14.9 Hz). **^{11}B NMR (128 MHz, $CDCl_3$)** δ 30.48. **HRMS (EI)** calcd for $C_{23}H_{33}B_2BrF_2O_4$: 512.1716, found: 512.1708. **HPLC analysis**: DAICEL CHIRALCEL OZ-3, hexane/isopropanol = 99.8/0.2, 0.5mL/min, λ = 210 nm, t_R (major) = 10.8 min, t_R (minor) = 11.6 min, 93:7 er. **[α]$^{25}_{D}$**: +1.3 (*c* 0.5, CHCl3).

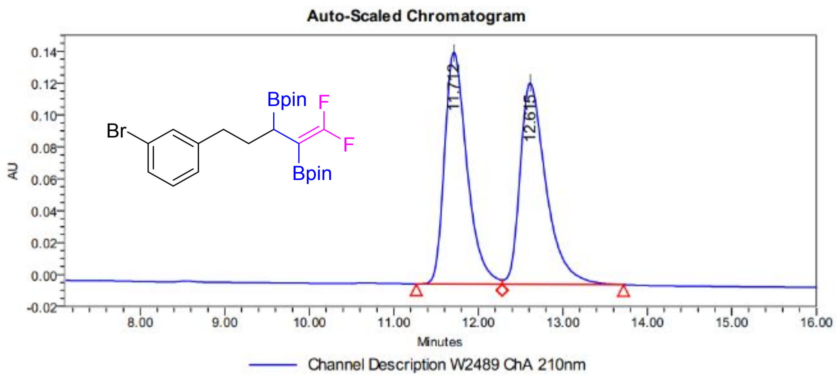

Peak Results

	Retention Time (min)	Int Type	Width (sec)	Area (µV*sec)	Height (µV)	% Area
1	11.712	BV	61.000	2687191	145309	49.77
2	12.615	VB	86.000	2712043	126324	50.23

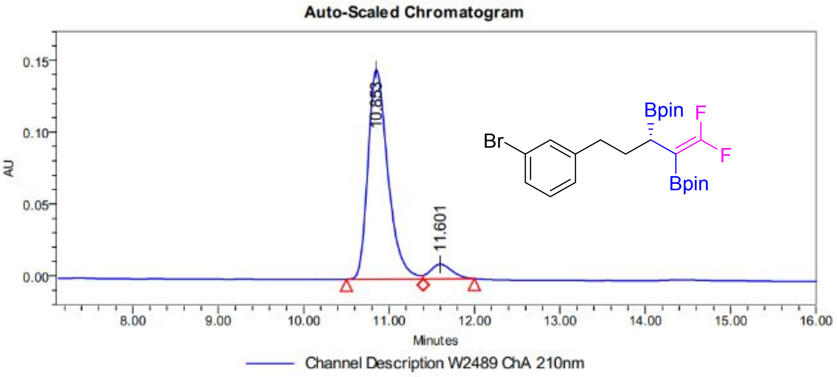

Peak Results

	Retention Time (min)	Int Type	Width (sec)	Area (µV*sec)	Height (µV)	% Area
1	10.853	bv	54.000	2369449	145998	93.07
2	11.601	vb	36.000	176416	10216	6.93

(S)-2,2'-(5-(2-bromophenyl)-1,1-difluoropent-1-ene-2,3-diyl)bis(4,4,5,5-tetramethyl-1,3,2-dioxaborolane) (69)

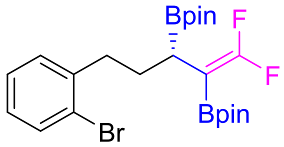

The reaction was performed following the **procedure G**. The residue was purified by flash column chromatograph (R_f = 0.4, PE: Et_2O = 20:1) to give the product as a white solid (118.3 mg, 77% yield, mp: 37-38 ^oC.).

^{1}H NMR (500 MHz, $CDCl_3$) δ 7.48 (d, J = 7.5 Hz, 1H), 7.23 – 7.16 (m, 2H), 7.04 – 6.98 (m, 1H), 2.82 – 2.72 (m, 1H), 2.66 – 2.55 (m, 1H), 2.00 – 1.88 (m, 2H), 1.82 – 1.69 (m, 1H), 1.29 – 1.21 (m, 24H). **^{13}C NMR (126 MHz, $CDCl_3$)** δ 160.0 (dd, J = 301.1, 298.6 Hz), 142.3, 132.7, 130.6, 127.4, 124.5, 83.7, 83.5, 35.8, 30.7, 25.2, 25.0, 24.8, 24.6. **^{19} FNMR (470 MHz, $CDCl_3$)** δ -71.40 (d, J = 15.0 Hz), -72.52 (d, J = 15.8 Hz). **^{11}B NMR (128 MHz, $CDCl_3$)** δ 30.80. **HRMS (ESI)** calcd for $C_{23}H_{34}B_2BrF_2O_4$ $[M+H]^+$: 513.1789, found: 513.1786. **HPLC analysis**: DAICEL CHIRALCEL OZ-3 hexane/isopropanol = 99.9/0.1, 0.5mL/min, λ = 210 nm, t_R (major) = 11.1 min, t_R (minor) = 13.1 min, 86:14 er **$[\alpha]^{25}_D$**: 7.8 (c 0.5,

CHCl3)

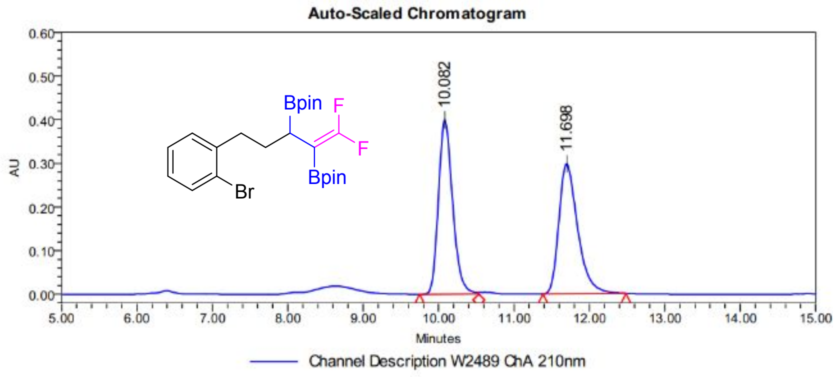

Peak Results

	Retention Time (min)	Int Type	Width (sec)	Area (μV*sec)	Height (μV)	% Area
1	10.082	BV	47.000	5225776	399952	50.30
2	11.698	bb	66.000	5164399	298389	49.70

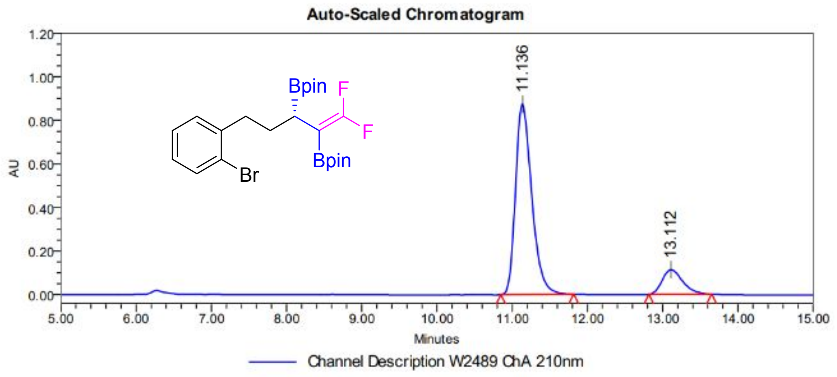

Peak Results

	Retention Time (min)	Int Type	Width (sec)	Area (μV*sec)	Height (μV)	% Area
1	11.136	bb	58.000	13102737	875449	85.92
2	13.112	bb	50.000	2146337	112997	14.08

(S)-2,2'-(1,1-difluoro-5,5-diphenylpent-1-ene-2,3-diyl)bis(4,4,5,5-tetramethyl-1,3,2-dioxaborolane) (70)

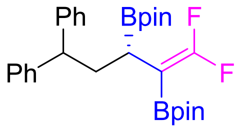

The reaction was performed following the **procedure G**. The residue was purified by flash column chromatograph (R_f = 0.4, PE: Et_2O = 10:1) to give the product as a white solid (108.6 mg, 71% yield, mp: 40-41.5 °C.).

^{1}H NMR (500 MHz, $CDCl_3$) δ 7.27 (d, J = 4.4 Hz, 4H), 7.22 – 7.19 (m, 4H), 7.18 – 7.14 (m, 1H), 7.12 – 7.09 (m, 1H), 3.97 (dd, J = 10.2, 5.6 Hz, 1H), 2.48 – 2.42 (m, 1H), 2.25 – 2.14 (m, 1H), 1.83 – 1.80 (m, 1H), 1.26 – 1.19 (m, 24H). **^{13}C NMR (126 MHz, $CDCl_3$)** δ 159.7 (dd, J = 300.7, 296.0 Hz), 146.0, 143.9, 128.5, 128.4, 128.3, 128.0, 126.0, 125.9, 83.6, 83.4, 50.0, 36.1, 25.2, 24.9, 24.8, 24.4. **^{19}F NMR (471 MHz, $CDCl_3$)** δ -71.21 (d, J = 14.6 Hz), -72.66 (d, J = 14.6 Hz). **^{11}B NMR (128 MHz, $CDCl_3$)** δ 30.71, 22.14. **HRMS (EI)** calcd for $C_{29}H_{38}B_2F_2O_4$: 510.2924, found: 510.2925. **HPLC analysis**:

DAICEL CHIRALCEL OZ-3 hexane/isopropanol = 99.9/0.1, 0.5mL/min, λ = 214 nm, t_R (major) = 15.9 min, t_R (minor) = 17.8 min, 95:5 er **[α]$^{25}_D$**: -9.6 (*c* 0.5, $CHCl_3$).

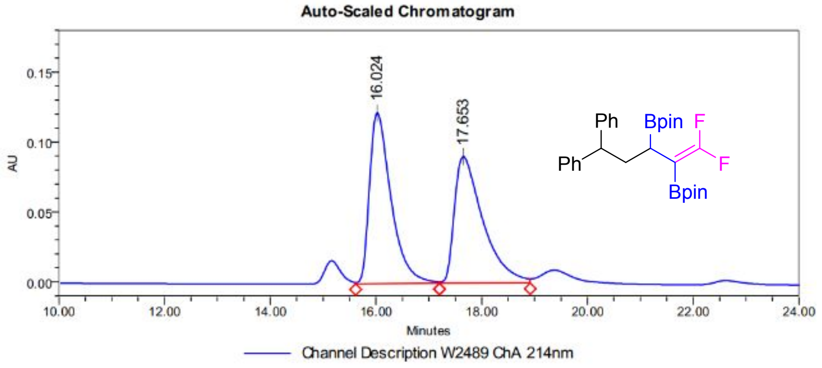

Peak Results

	Retention Time (min)	Int Type	Width (sec)	Area (µV*sec)	Height (µV)	% Area
1	16.024	VV	95.000	3475225	122329	50.53
2	17.653	VV	103.000	3402344	90545	49.47

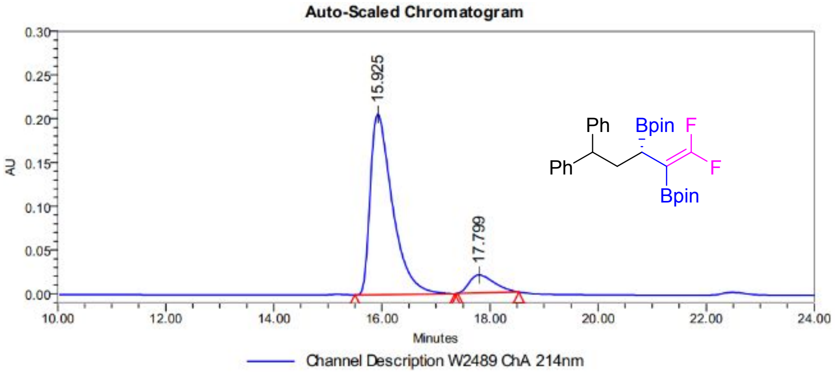

Peak Results

	Retention Time (min)	Int Type	Width (sec)	Area (µV*sec)	Height (µV)	% Area
1	15.925	bb	111.000	5880285	206489	89.95
2	17.799	bb	68.000	656653	20465	10.05

(S)-2,2'-(1,1-difluoro-6-phenylhex-1-ene-2,3-diyl)bis(4,4,5,5-tetramethyl-1,3,2-dioxaborolane) (71)

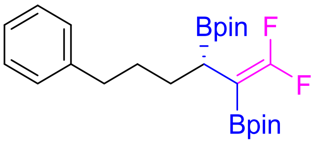

The reaction was performed following the **procedure G**. The residue was purified by flash column chromatograph (R_f = 0.4, PE: Et_2O = 10:1) to give the product as a colorless liquid (119.6 mg, 89% yield).

^{1}H NMR (500 MHz, $CDCl_3$) δ 7.26 – 7.22 (m, 2H), 7.18 – 7.12 (m, 3H), 2.67 – 2,61 (m, 1H), 2.55 – 2.49 (m, 1H), 1.90 – 1.87 (m, 1H), 1.77 – 1.69 (m, 1H), 1.68 – 1.59 (m, 1H), 1.59 – 1.48 (m, 2H), 1.25 – 1.19 (m, 24H). **^{13}C NMR (126 MHz, $CDCl_3$)** δ 159.8 (dd, *J* = 299.4, 296.9 Hz), 143.0, 128.5, 128.3, 125.6, 83.6, 83.4, 36.0, 30.9, 29.8, 25.1, 24.9, 24.8, 24.4. **^{19}F NMR (471 MHz, $CDCl_3$)** δ -74.83 (d, *J* = 16.5 Hz), -75.72 (d, *J* = 16.6 Hz). **^{11}B NMR (128 MHz, $CDCl_3$)** δ 30.54. **HRMS (EI)** calcd for $C_{24}H_{36}B_2F_2O_4$: 448.2768, found: 448.2758. **HPLC analysis**: DAICEL CHIRALCEL OZ-3

hexane/isopropanol = 99.9/0.1, 0.5mL/min, λ = 210 nm, t_R (major) = 10.5 min, t_R (minor) = 11.1 min, 97.5:2.5 er. **$[\alpha]^{25}_D$**: 24.4 (*c* 0.5, $CHCl_3$).

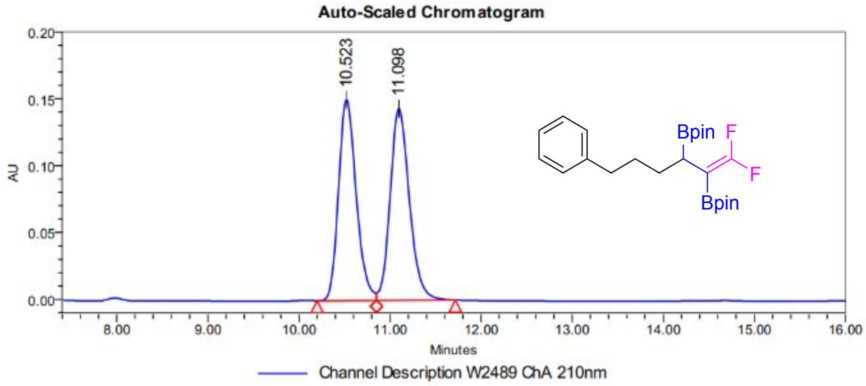

Peak Results

	Retention Time (min)	Int Type	Width (sec)	Area (μV*sec)	Height (μV)	% Area
1	10.523	bV	39.000	2030382	150462	49.22
2	11.098	Vb	52.000	2094395	143389	50.78

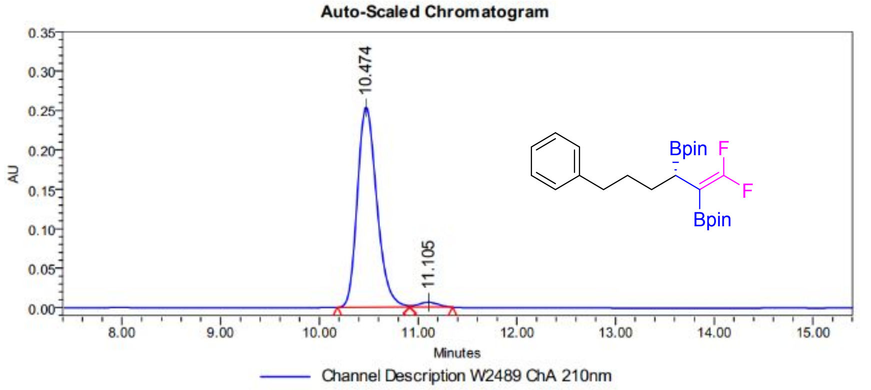

Peak Results

	Retention Time (min)	Int Type	Width (sec)	Area (μV*sec)	Height (μV)	% Area
1	10.474	bV	44.000	3457539	254210	97.51
2	11.105	Vb	26.000	88444	6266	2.49

(S)-2,2'-(1,1-difluoro-4-phenylbut-1-ene-2,3-diyl)bis(4,4,5,5-tetramethyl-1,3,2-dioxaborolane) (72)

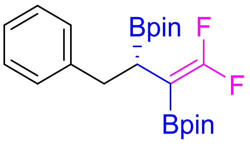

he reaction was performed following the **procedure G**. The residue was purified by flash column chromatograph (R_f = 0.4, PE: Et_2O = 10:1) to give the product as a white solid (100.8 mg, 80% yield, mp: 47.5 - 49 °C.).

^{1}H NMR (500 MHz, $CDCl_3$) δ 7.24 – 7.19 (m, 2H), 7.16 – 7.10 (m, 3H), 3.07 – 3.03 (m, 1H), 2.72 (dd, *J* = 13.9, 11.3 Hz, 1H), 2.21 – 2.17 (m, 1H), 1.28 – 1.22 (m, 24H). **^{13}C NMR (126 MHz, $CDCl_3$)** 159.8 (dd, *J* = 301.1, 297.4 Hz), 142.1, 129.0, 128.1, 125.7, 83.6, 36.1, 25.1, 25.0, 24.8, 24.5. **^{19}F NMR (471 MHz, $CDCl_3$)** δ -72.35 (d, *J* = 13.4 Hz), -74.05 (d, *J* = 13.4 Hz). **^{11}B NMR (128 MHz, $CDCl_3$)** δ 32.74, 30.45. **HRMS (ESI)** calcd for $C_{22}H_{33}B_2F_2O_4$ $[M+H]^+$: 421.2528, found: 421.2531. **HPLC analysis**: DAICEL CHIRALCEL OZ-3 hexane/isopropanol = 99.9/0.1, 0.5mL/min, λ = 210 nm, t_R (major) = 10.1

min, t_R (minor) = 10.8 min, 92: 8 er **[α]^{25}$_D$**: 94.6 (*c* 0.5, $CHCl_3$).

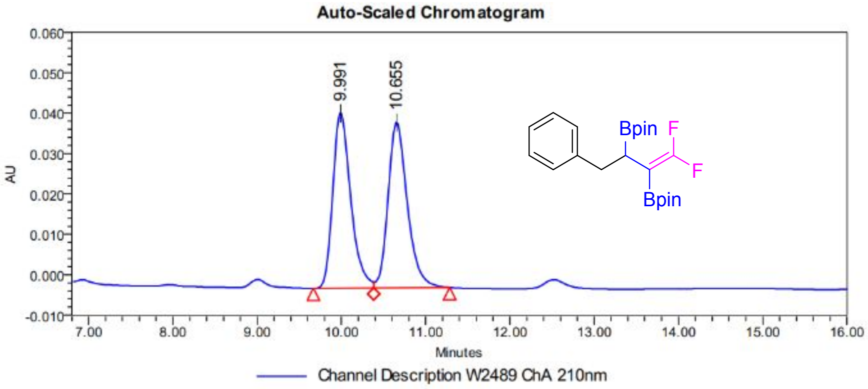

Peak Results

	Retention Time (min)	Int Type	Width (sec)	Area (µV*sec)	Height (µV)	% Area
1	9.991	BV	43.000	641982	43444	49.64
2	10.655	VB	54.000	651170	40968	50.36

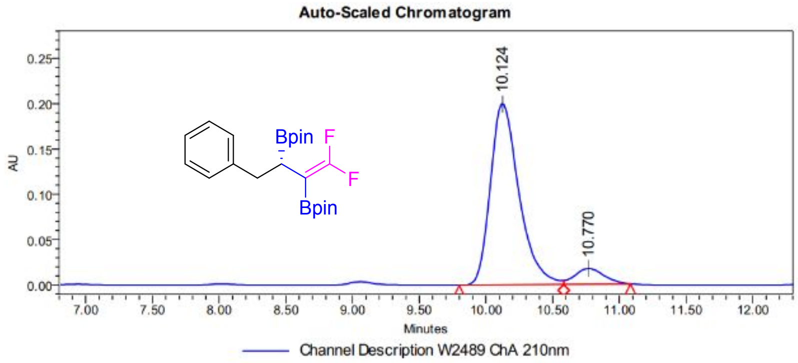

Peak Results

	Retention Time (min)	Int Type	Width (sec)	Area (µV*sec)	Height (µV)	% Area
1	10.124	bv	47.000	2981866	200140	91.94
2	10.770	vb	30.000	261251	17412	8.06

(S)-2,2'-(1,1-difluoro-4-(naphthalen-1-yl)but-1-ene-2,3-diyl)bis(4,4,5,5-tetramethyl-1,3,2-dioxaborolane) (73)

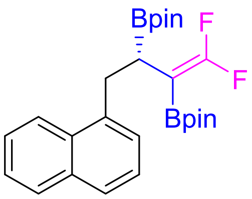

The reaction was performed following the **procedure G**. The residue was purified by flash column chromatograph (R_f = 0.4, PE: Et_2O = 10:1) to give the product as a white solid (108.6 mg, 77% yield, mp: 50.5-52 °C.).

^{1}H NMR (500 MHz, $CDCl_3$) δ 8.12 (d, *J* = 8.3 Hz, 1H), 7.80 (d, *J* = 8.2 Hz, 1H), 7.66 (d, *J* = 7.9 Hz, 1H), 7.50 – 7.40 (m, 2H), 7.33 (t, *J* = 7.6 Hz, 1H), 7.26 – 7.22 (m, 1H), 3.61 (dd, *J* = 14.2, 5.0 Hz, 1H), 3.08 (dd, *J* = 14.1, 10.6 Hz, 1H), 2.37 (dd, *J* = 10.7, 5.0 Hz, 1H), 1.27 – 1.15 (m, 24H). **^{13}C NMR (126 MHz, $CDCl_3$)** δ 159.9 (dd, *J* = 300.8, 297.8 Hz), 138.0, 134.0, 132.2, 128.7, 127.1, 126.6, 125.5, 125.2, 124.2, 83.7, 83.6, 33.3, 25.1, 25.0, 24.9, 24.5. **^{19}F NMR (471 MHz, $CDCl_3$)** δ -70.68 (d, *J* = 14.1 Hz), -

72.72 (d, J = 11.5 Hz). **^{11}B NMR (128 MHz, $CDCl_3$)** δ 30.13. **HRMS (ESI)** calcd for $C_{26}H_{35}B_2F_2O_4$ $[M+H]^+$: 471.2684, found: 471.2681. **HPLC analysis**: DAICEL CHIRALCEL OZ-3 hexane/isopropanol = 99.9/0.1, 0.5mL/min, λ = 210 nm, t_R (minor) = 12.0 min, t_R (major) = 14.3 min, 97.5:2 2.5. er **$[\alpha]^{25}_D$**: 48.8 (c 0.5, $CHCl_3$).

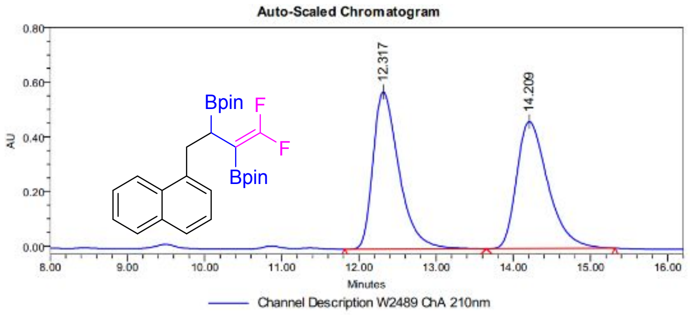

Peak Results

	Retention Time (min)	Int Type	Width (sec)	Area (μV*sec)	Height (μV)	% Area
1	12.317	BV	110.000	13457718	574886	50.64
2	14.209	Vb	100.000	13117156	464973	49.36

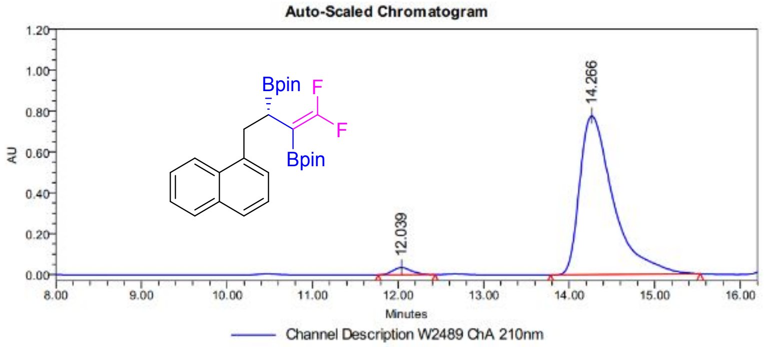

Peak Results

	Retention Time (min)	Int Type	Width (sec)	Area (μV*sec)	Height (μV)	% Area
1	12.039	bb	40.000	554907	35400	2.55
2	14.266	bb	105.000	21243962	778313	97.45

(S)-2,2'-(4-([1,1'-biphenyl]-4-yl)-1,1-difluorobut-1-ene-2,3-diyl)bis(4,4,5,5-tetramethyl-1,3,2-dioxaborolane) (74)

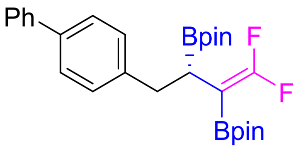

The reaction was performed following the **procedure G**. The residue was purified by flash column chromatograph (R_f = 0.4, PE: Et_2O = 10:1) to give the product as a white solid (120.5 mg, 81% yield, mp: 81.5 - 83 °C.).

^{1}H NMR (500 MHz, $CDCl_3$) δ 7.57 (dd, J = 8.3, 1.2 Hz, 2H), 7.46 (d, J = 8.1 Hz, 2H), 7.41 (t, J = 7.8 Hz, 2H), 7.34 – 7.29 (m, 1H), 7.23 – 7.18 (m, 2H), 3.11 – 3.07 (m, 1H), 2.77 (dd, J = 13.8, 11.4 Hz, 1H), 2.23 (dd, J = 11.0, 4.3 Hz, 1H), 1.29 – 1.22 (m, 24H). **^{13}C NMR (126 MHz, $CDCl_3$)** δ 159.8 (dd, J = 300.4, 297.7 Hz), 141.4, 141.3, 138.6, 129.5, 128.8, 127.1, 127.0, 126.8, 83.6, 35.8, 25.1, 25.0, 24.8, 24.5. **^{19}F NMR (471 MHz, $CDCl_3$)** δ -70.74 (d, J = 13.4 Hz), -72.64 (d, J = 14.0 Hz). **^{11}B NMR (128 MHz, $CDCl_3$)** δ 28.56. **HRMS (ESI)** calcd for $C_{26}H_{35}B_2F_2O_4$ $[M+H]^+$: 497.2841,found: 497.2838. **HPLC analysis**: DAICEL CHIRALCEL OZ-3 hexane/isopropanol = 99.9/0.1, 0.5mL/min, λ = 210 nm,

t_R (major) = 15.9 min, t_R (minor) = 20.5 min, 94.5:5.5 er **[α]^{25}$_D$**: 47.0 (*c* 0.5, $CHCl_3$).

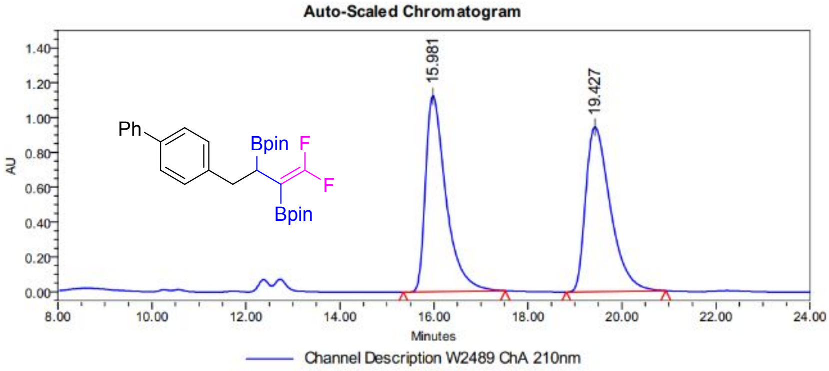

Peak Results

	Retention Time (min)	Int Type	Width (sec)	Area (µV*sec)	Height (µV)	% Area
1	15.981	bb	130.000	34128973	1122715	49.71
2	19.427	bb	127.000	34528170	945011	50.29

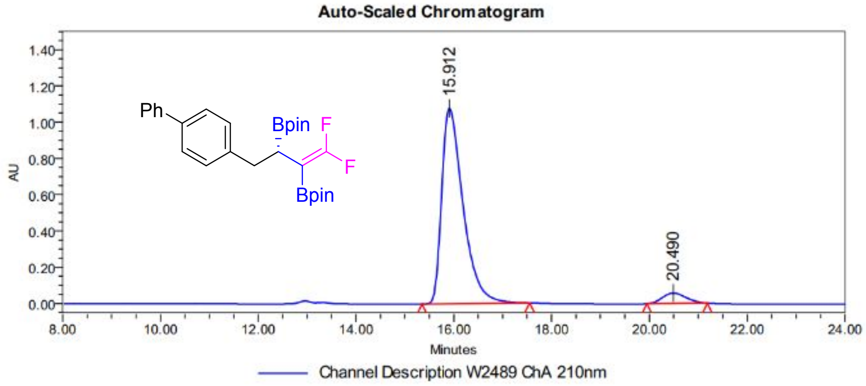

Peak Results

	Retention Time (min)	Int Type	Width (sec)	Area (µV*sec)	Height (µV)	% Area
1	15.912	Bb	132.000	32796346	1077892	94.48
2	20.490	bb	74.000	1917381	57068	5.52

(S)-2,2'-(4-(benzo[d][1,3]dioxol-5-yl)-1,1-difluorobut-1-ene-2,3-diyl)bis(4,4,5,5-tetramethyl-1,3,2-dioxaborolane) (75)

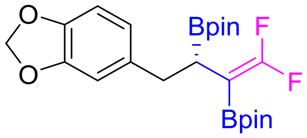

The reaction was performed following the **procedure G**. The residue was purified by flash column chromatograph (R_f = 0.3, PE: Et_2O = 10:1) to give the product as a white solid (117.0 mg, 84% yield, mp: 85 -86.5 ^oC.).

^{1}H NMR (500 MHz, $CDCl_3$) δ 6.68 – 6.63 (m, 2H), 6.58 – 6.54 (m, 1H), 5.88 (s, 2H), 2.96 (dd, *J* = 14.1, 2.9 Hz, 1H), 2.65 (dd, *J* = 13.9, 11.2 Hz, 1H), 2.13 (dd, *J* = 11.1, 4.1 Hz, 1H), 1.26 – 1.22 (m, 24H). **^{13}C NMR (126 MHz, $CDCl_3$)** δ 159.8 (dd, *J* = 299.9, 297.4 Hz), 147.3, 145.5, 136.0, 121.8, 109.4, 107.9, 100.7, 83.6, 35.8, 25.1, 24.9, 24.8, 24.5. **^{19}F NMR (471 MHz, $CDCl_3$)** δ -72.79 (d, *J* = 5.6 Hz), -74.40 (d, *J* = 14.0 Hz). **^{11}B NMR (128 MHz, $CDCl_3$)** δ 30.46. **HRMS (ESI)** calcd for $C_{23}H_{33}B_2F_2O_6$ $[M+H]^+$: 465.2426, found: 465.2434. **HPLC analysis**: DAICEL CHIRALCEL IB N-3 hexane/isopropanol = 99.8/0.2, 0.5mL/min, λ = 210 nm, t_R (minor) = 14.4 min, t_R (major) = 14.9 min, 93.5:6.5 er **[α]^{25}$_D$**: 10.2 (*c*

0.5, $CHCl_3$).

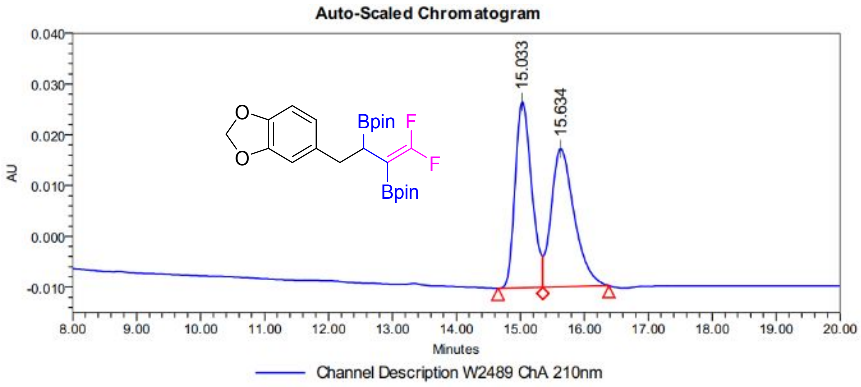

Peak Results

	Retention Time (min)	Int Type	Width (sec)	Area (µV*sec)	Height (µV)	% Area
1	15.033	BV	42.000	658197	36606	49.02
2	15.634	Vb	62.000	684559	27214	50.98

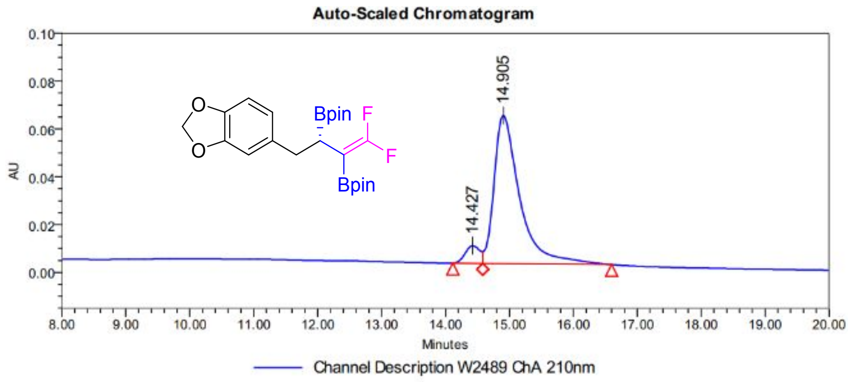

Peak Results

	Retention Time (min)	Int Type	Width (sec)	Area (µV*sec)	Height (µV)	% Area
1	14.427	bV	28.000	117505	7386	6.36
2	14.905	Vb	121.000	1729835	61979	93.64

(S)-2,2'-(1,1-difluoro-5-(5-methylfuran-2-yl)pent-1-ene-2,3-diyl)bis(4,4,5,5-tetramethyl-1,3,2-dioxaborolane) (76)

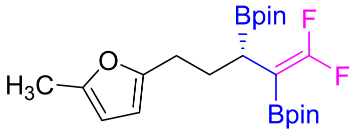

The reaction was performed following the **procedure G**. The residue was purified by flash column chromatograph (R_f = 0.35, PE: Et_2O = 10:1) to give the product as a white solid (102.5 mg, 78% yield, mp: 37.2-38.5 ^oC.).

^{1}H NMR (500 MHz, $CDCl_3$) δ 5.86 – 5.74 (m, 2H), 2.61 – 2.55 (m, 1H), 2.50 – 2.44 (m, 1H), 2.22 (s, 3H), 2.03 – 1.96 (m, 1H), 1.88 (dd, J = 11.3, 3.8 Hz, 1H), 1.81 – 1.71 (m, 1H), 1.27 – 1.21 (m, 24H). **^{13}C NMR (126 MHz, $CDCl_3$)** δ 159.9 (t, J = 296.3 Hz), 154.7, 150.0, 105.8, 105.3, 83.7, 83.4, 28.8, 27.4, 25.1, 24.9, 24.8, 24.4, 13.6 **^{19}F NMR (470 MHz, CDCl3)** δ -71.48 (d, J = 14.8 Hz), -72.63 (d, J = 15.5 Hz). **^{11}B NMR (128 MHz, $CDCl_3$)** δ 30.62. **HRMS (EI)** calcd for $C_{22}H_{34}B_2F_2O_5$: 438.2560, found: 438.2552. **HPLC analysis**: DAICEL CHIRALCEL OZ-3 hexane/isopropanol = 99.9/0.1, 0.5mL/min, λ

= 210 nm, t_R (major) = 12.0 min, t_R (minor) = 13.4 min, 94:6 er. **[α]$^{25}_{D}$**: 16.4 (*c* 0.5, CHCl3).

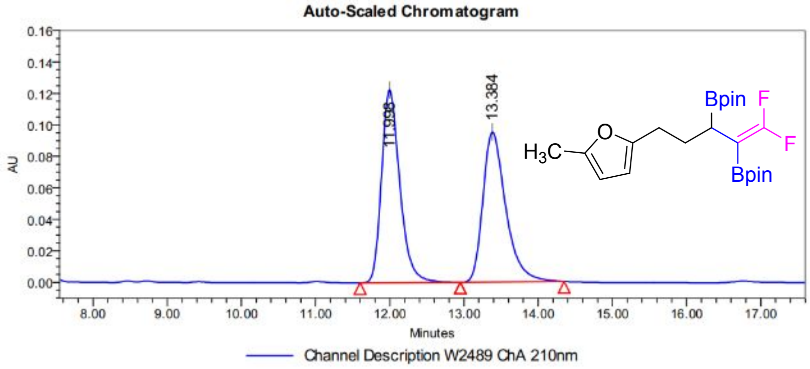

Peak Results

	Retention Time (min)	Int Type	Width (sec)	Area (µV*sec)	Height (µV)	% Area
1	11.998	BB	81.000	2094669	122601	50.62
2	13.384	BB	84.000	2043705	95278	49.38

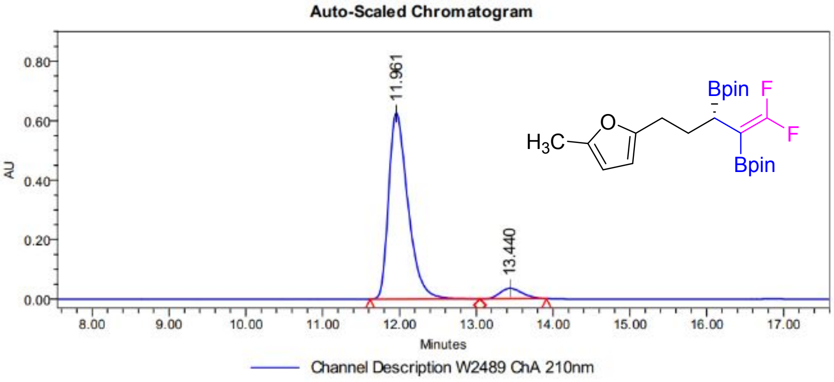

Peak Results

	Retention Time (min)	Int Type	Width (sec)	Area (µV*sec)	Height (µV)	% Area
1	11.961	bV	86.000	10912198	625696	93.91
2	13.440	Vb	52.000	707126	35318	6.09

(S)-2,2'-(1,1-difluoro-6-(thiophen-2-yl)hex-1-ene-2,3-diyl)bis(4,4,5,5-tetramethyl-1,3,2-dioxaborolane) (77)

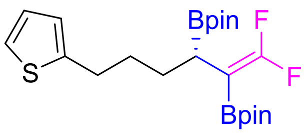

The reaction was performed following the **procedure G**. The residue was purified by flash column chromatograph (R_f = 0.45, PE: Et_2O = 10:1) to give the product as a colorless liquid (113.1 mg, 83% yield).

^{1}H NMR (500 MHz, CDCl$_3$) δ 7.10 – 7.05 (m, 1H), 6.89 (dd, *J* = 5.1, 3.4 Hz, 1H), 6.79 – 6.74 (m, 1H), 2.88 – 2.82 (m, 1H), 2.78 – 2.72 (m, 1H), 1.93 – 1.84 (m, 1H), 1.82 – 1.66 (m, 2H), 1.65 – 1.52 (m, 2H), 1.26 – 1.21 (m, 24H). **^{13}C NMR (126 MHz, CDCl$_3$)** δ 159.8 (dd, *J* = 299.9, 298.6 Hz), 145.8, 126.6, 123.9, 122.7, 83.6, 83.3, 31.1, 29.9, 29.6, 25.0, 24.8, 24.7, 24.4. **^{19}F NMR (470 MHz, CDCl$_3$)** δ -71.91 (d, *J* = 14.9 Hz), -72.82 (d, *J* = 15.2 Hz). **^{11}B NMR** (128 MHz, **CDCl$_3$)** δ 30.63. **HRMS (ESI)** calcd for $C_{22}H_{35}B_2F_2O_4S$ $[M+H]^+$: 455.2405, found: 455.2401. **HPLC analysis**: DAICEL CHIRALCEL OZ-3 hexane/isopropanol = 99.9/0.1, 0.5mL/min, λ = 210 nm, t_R (major) = 10.5 min, t_R (minor) = 11.8 min,

93:7 er. **[α]^{25}$_{D}$**: 16.0 (*c* 0.5, $CHCl_3$).

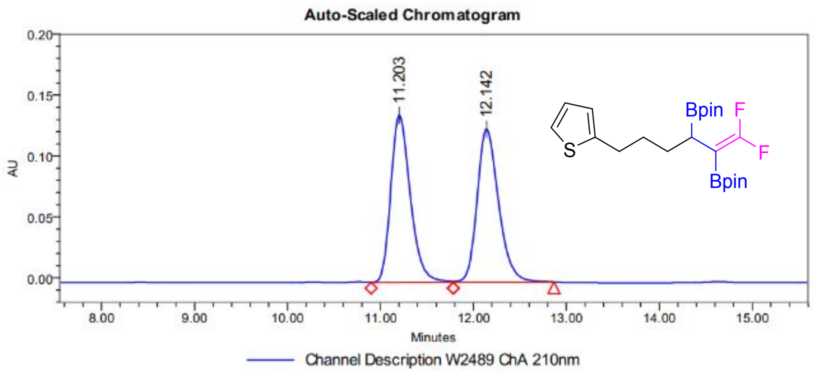

Peak Results

	Retention Time (min)	Int Type	Width (sec)	Area (μV*sec)	Height (μV)	% Area
1	11.203	VV	53.000	2006382	137361	49.77
2	12.142	Vb	65.000	2024695	125849	50.23

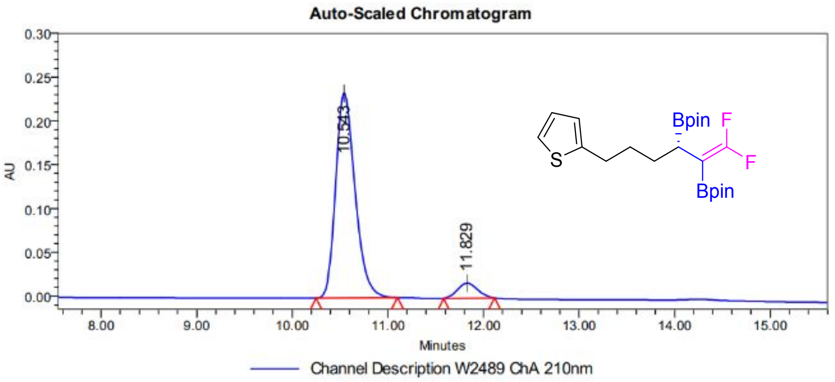

Peak Results

	Retention Time (min)	Int Type	Width (sec)	Area (μV*sec)	Height (μV)	% Area
1	10.543	bb	51.000	3307495	234008	92.94
2	11.829	bb	32.000	251140	17066	7.06

(S)-2,2'-(1,1-difluoro-4-(tetrahydro-2H-pyran-4-yl)but-1-ene-2,3-diyl)bis(4,4,5,5-tetramethyl-1,3,2-dioxaborolane) (78)

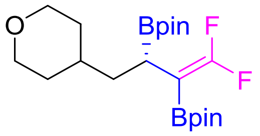

The reaction was performed following the **procedure G**. The residue was purified by flash column chromatograph (R_f = 0.35, PE: Et_2O = 10:1) to give the product as a colorless liquid (110.4 mg, 86% yield).

^{1}H NMR (500 MHz, $CDCl_3$) δ 3.95 – 3.86 (m, 2H), 3.43 – 3.20 (m, 2H), 1.98 – 1.95 (m, 1H), 1.67 (d, *J* = 13.3 Hz, 1H), 1.58 – 1.50 (m, 1H), 1.49 – 1.29 (m, 4H), 1.22 (dd, *J* = 7.7, 5.5 Hz, 24H), 1.15 – 1.06 (m, 1H). **^{13}C NMR (126 MHz, $CDCl_3$)** δ 159.6 (dd, *J* = 299.5, 296.9 Hz), 83.6, 83.4, 68.3, 68.3, 36.8, 34.1, 33.3, 32.1, 25.1, 24.9, 24.8, 24.4. **^{19}F NMR (471 MHz, $CDCl_3$)** δ -74.69 (d, *J* = 17.5 Hz), -75.48 (d, *J* = 17.3 Hz). **^{11}B NMR (128 MHz, $CDCl_3$)** δ 30.37. **HRMS (ESI)** calcd for $C_{21}H_{37}B_2F_2O_5$ [M+H]$^+$: 429.2790, found: 429.2786. **HPLC analysis**: DAICEL CHIRALCEL IC-3, hexane/isopropanol = 99.9/0.1, 0.5mL/min, λ = 210 nm, t_R (major) = 13.2 min, t_R (minor) = 16.5 min, 97:3 er. **[α]^{25}$_{D}$**: 20.8 (*c*

0.5, CHCl3).

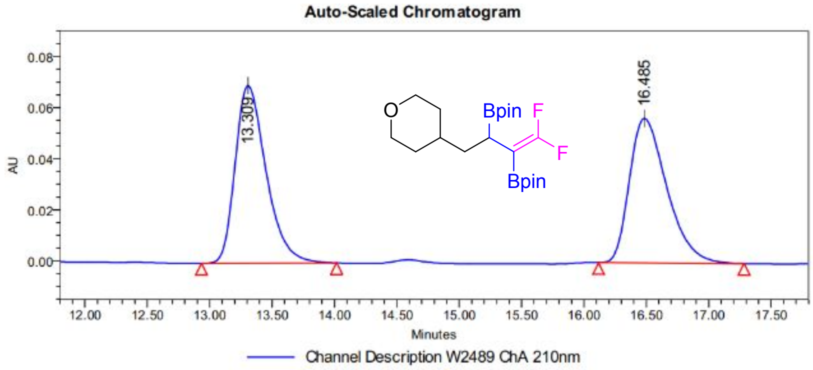

Peak Results

	Retention Time (min)	Int Type	Width (sec)	Area (μV*sec)	Height (μV)	% Area
1	13.309	BB	65.000	1195287	69657	50.31
2	16.485	BB	70.000	1180462	56588	49.69

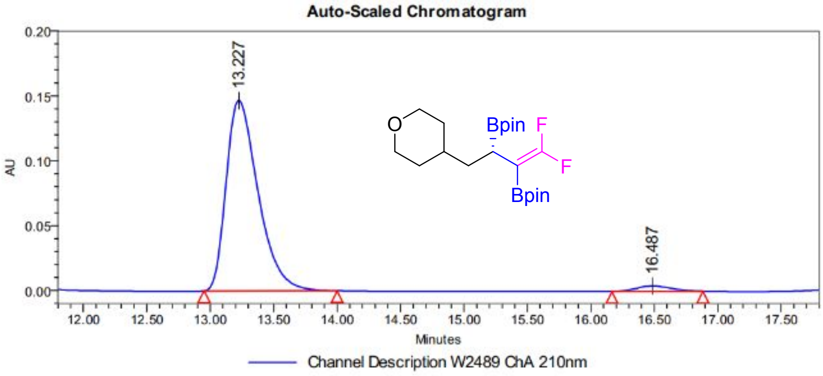

Peak Results

	Retention Time (min)	Int Type	Width (sec)	Area (μV*sec)	Height (μV)	% Area
1	13.227	bb	63.000	2566719	146919	97.01
2	16.487	bb	43.000	79103	4177	2.99

Benzyl(S)-4-(3,3-difluoro-1,2-bis(4,4,5,5-tetramethyl-1,3,2-dioxaborolan-2-yl)allyl)piperidine-1-carboxylate (79)

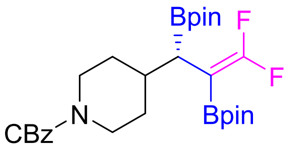

The reaction was performed following the **procedure G**. The residue was purified by flash column chromatograph (Rf = 0.25, PE: Et_2O = 4:1) to give the product as a colorless liquid (120.7 mg, 76% yield.).

^{1}H NMR (500 MHz, CDCl$_3$) δ 7.34 (d, J = 4.4 Hz, 4H), 7.32 – 7.27 (m, 1H), 5.11 (s, 2H), 4.14 (s, 2H), 2.76 (d, J = 39.5 Hz, 2H), 1.91 – 1.80 (m, 2H), 1.72 (d, J = 8.6 Hz, 1H), 1.68 – 1.57 (m, 1H), 1.26 – 1.21 (m, 24H), 1.18 – 1.10 (m, 1H), 1.09 – 0.99 (m, 1H). **^{13}C NMR (126 MHz, CDCl$_3$)** δ 159.7 (dd, J = 300.6, 297.6 Hz), 155.4, 137.2, 128.6, 128.0, 127.9, 83.7, 83.4, 67.0, 44.5, 44.4, 36.3, 27.0, 25.0, 24.8, 24.8, 24.6. **^{19}F NMR (471 MHz, CDCl$_3$)** δ -69.45 (d, J = 29.6 Hz), -71.94 (d, J = 67.1 Hz). **^{11}B NMR (128 MHz, CDCl$_3$)** δ 30.70. **HRMS (ESI)** calcd for $C_{28}H_{42}B_2F_2NO_6$ $[M+H]^+$: 548.3161, found: 548.3164. **HPLC analysis**: DAICEL CHIRALCEL OD-3 hexane/isopropanol = 99.5/0.5, 0.5mL/min, λ = 210 nm,

t_R (minor) = 30.4 min, t_R (major) = 33.2 min, 90:10 er. **[α]^{25}$_D$**: 11.6 (*c* 0.5, $CHCl_3$).

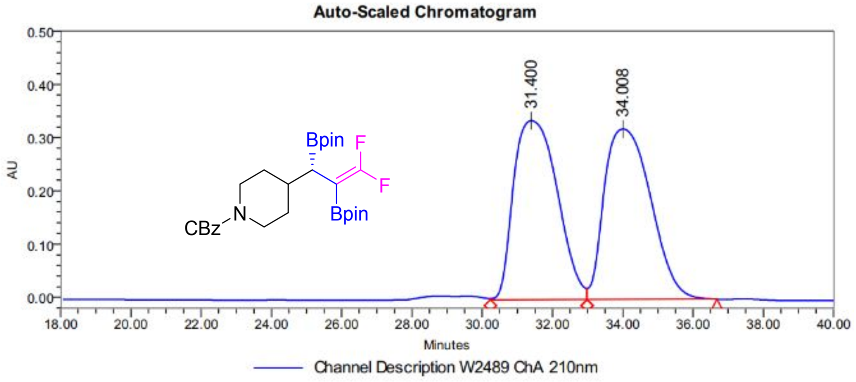

Peak Results

	Retention Time (min)	Int Type	Width (sec)	Area (µV*sec)	Height (µV)	% Area
1	31.400	VV	165.000	29096831	336035	49.61
2	34.008	VB	222.000	29549964	320036	50.39

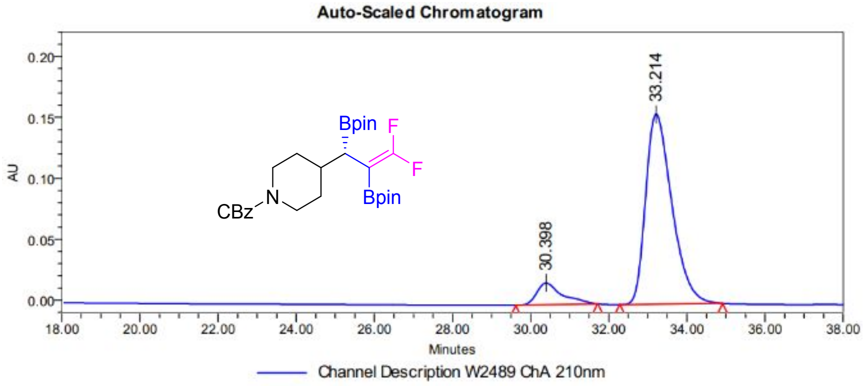

Peak Results

	Retention Time (min)	Int Type	Width (sec)	Area (µV*sec)	Height (µV)	% Area
1	30.398	Bb	126.000	808606	17927	9.97
2	33.214	BB	158.000	7299750	155985	90.03

tert-butyl(S)-4-(3,3-difluoro-1,2-bis(4,4,5,5-tetramethyl-1,3,2-dioxaborolan-2-yl)allyl)piperidine-1-carboxylate (80)

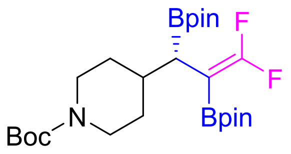

The reaction was performed following the **procedure G**. The residue was purified by flash column chromatograph (R_f = 0.35, PE: Et_2O = 10:1) to give the product as a white solid (123.8 mg, 81% yield, mp: 40.5-42 °C.).

^{1}H NMR (500 MHz, CDCl$_3$) δ 4.00 (s, 2H), 2.63 (d, *J* = 44.6 Hz, 2H), 1.78 (d, *J* = 9.9 Hz, 2H), 1.66 (d, *J* = 8.6 Hz, 1H), 1.62 – 1.51 (m, 1H), 1.40 (d, *J* = 2.1 Hz, 9H), 1.23 – 1.17 (m, 24H), 1.10 – 1.05 (m, 1H), 1.02 – 0.91 (m, 1H). **^{13}C NMR (126 MHz, CDCl$_3$)** δ 159.6 (dd, *J* = 300.6, 297.4 Hz), 155.0, 83.6, 83.3, 79.1, 44.2, 36.3, 32.2, 31.3, 28.5, 24.9, 24.8, 24.7, 24.5 **^{19}F NMR (471 MHz, CDCl$_3$)** δ -69.84, -72.18. **^{11}B NMR (128 MHz, CDCl$_3$)** δ 30.50. **HRMS (EI)** calcd for $C_{25}H_{43}B_2F_2NO_6$: 513.3245, found: 513.3241. **HPLC analysis**: DAICEL CHIRALCEL OZ-3, hexane/isopropanol = 99.9/0.1, 0.5mL/min, λ

= 210 nm, t_R (minor) = 17.5 min, t_R (major) = 19.8 min, 96.5:3.5 er. **[α]^{25}$_D$**: 9.8 (*c* 0.5, $CHCl_3$).

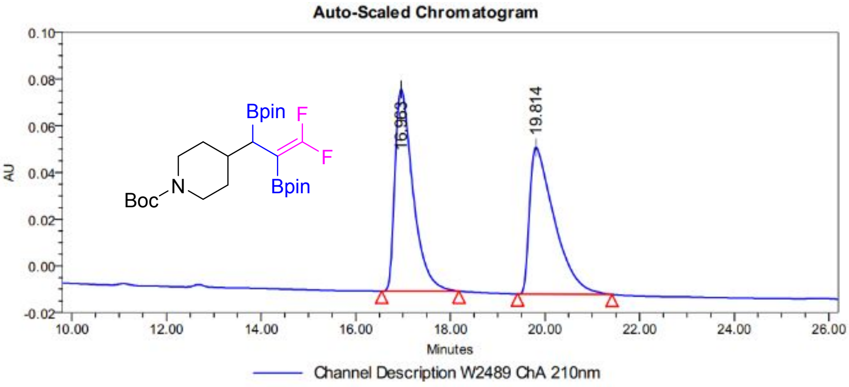

Peak Results

	Retention Time (min)	Int Type	Width (sec)	Area (µV*sec)	Height (µV)	% Area
1	16.963	BB	98.000	2286264	86467	49.90
2	19.814	BB	120.000	2295005	62795	50.10

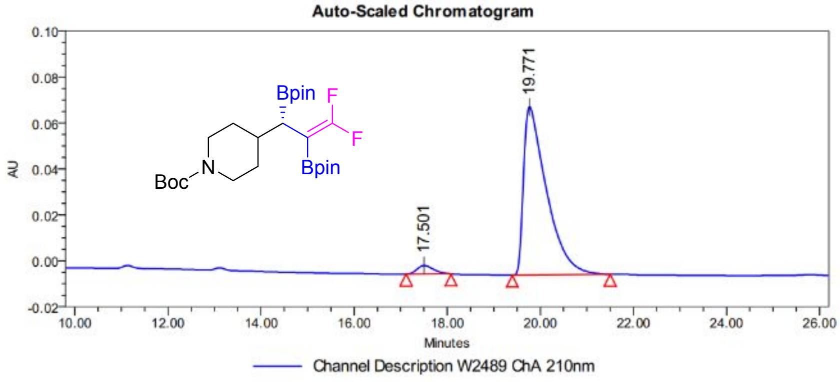

Peak Results

	Retention Time (min)	Int Type	Width (sec)	Area (µV*sec)	Height (µV)	% Area
1	17.501	bb	58.000	91932	3711	3.54
2	19.771	Bb	126.000	2505285	73197	96.46

(S)-4-(4,4-difluoro-2,3-bis(4,4,5,5-tetramethyl-1,3,2-dioxaborolan-2-yl)but-3-en-1-yl)-1-tosylpiperidine (81)

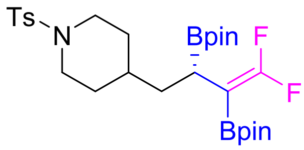

The reaction was performed following the **procedure G**. The residue was purified by flash column chromatograph (R_f = 0.2, PE: Et_2O = 4:1) to give the product as a white solid (102.3 mg, 69% yield, mp: 48.9-50 ^oC.).

^{1}H NMR (500 MHz, $CDCl_3$) δ 7.61 (d, *J* = 8.2 Hz, 2H), 7.30 (d, *J* = 8.0 Hz, 2H), 3.72 (t, *J* = 11.7 Hz, 2H), 2.42 (s, 3H), 2.16 – 2.07 (m, 2H), 1.92 – 1.88 (m, 1H), 1.79 (d, *J* = 12.7 Hz, 1H), 1.59 – 1.47 (m, 2H), 1.44 – 1.37 (m, 1H), 1.36 – 1.30 (m, 1H), 1.23 – 1.17 (m, 24H), 1.16 – 1.09 (m, 1H), 1.09 – 0.98 (m, 1H). **^{13}C NMR (126 MHz, $CDCl_3$)** δ 159.5 (dd, *J* = 299.4, 296.9 Hz), 143.5, 133.0, 129.7, 127.8, 83.7, 83.5, 46.7, 46.7, 35.8, 33.6, 32.6, 30.2, 25.2, 24.9, 24.8, 24.3, 21.6. **^{19}F NMR (376 MHz, $CDCl_3$)** δ -72.92 (d, *J* = 14.4 Hz), -73.36 (d, *J* = 15.3 Hz). **^{11}B NMR (128 MHz, $CDCl_3$)** δ 29.45. **HRMS (ESI)**

calcd for $C_{22}H_{44}B_2F_2NO_6S$ $[M+H]^+$: 582.3038, found:582.3041 **HPLC analysis**: DAICEL CHIRALCEL OZ-3 hexane/isopropanol = 98/2, 0.5mL/min, λ = 254 nm, t_R (major) = 34.5 min, t_R (minor) = 38.8 min, 95: 5 er **$[\alpha]^{25}_D$**: 22.6 (*c* 0.5, $CHCl_3$).

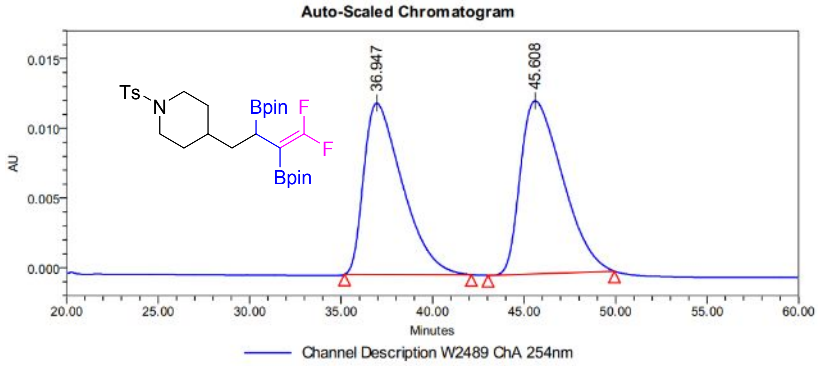

Peak Results

	Retention Time (min)	Int Type	Width (sec)	Area (µV*sec)	Height (µV)	% Area
1	36.947	Bb	415.000	1803018	12272	47.53
2	45.608	bb	414.000	1990030	12398	52.47

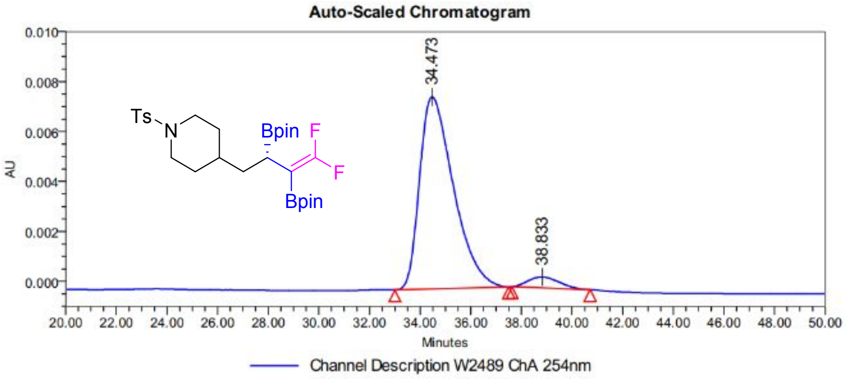

Peak Results

	Retention Time (min)	Int Type	Width (sec)	Area (µV*sec)	Height (µV)	% Area
1	34.473	bb	270.000	714506	7680	95.05
2	38.833	bb	185.000	37220	434	4.95

(S)-2,2'-(1,1-difluoro-5-phenoxypent-1-ene-2,3-diyl)bis(4,4,5,5-tetramethyl-1,3,2-dioxaborolane) (82)

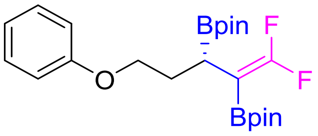

The reaction was performed following the **procedure G**. The residue was purified by flash column chromatograph (R_f = 0.3, PE: Et_2O = 20:1) to give the product as a white solid (101.3 mg, 75% yield, mp: 41.5-43 °C.).

^{1}H NMR (500 MHz, $CDCl_3$) δ 7.27 – 7.22 (m, 2H), 6.93 – 6.85 (m, 3H), 4.01 – 3.86 (m, 2H), 2.24 – 2.09 (m, 2H), 1.97 – 1.86 (m, 1H), 1.27 – 1.22 (m, 24H). **^{13}C NMR (126 MHz, $CDCl_3$)** δ 159.9 (dd, *J* = 300.7, 297.2 Hz), 159.2, 129.4, 120.4, 114.7, 83.7, 83.6, 66.9, 29.7, 25.2, 25.0, 24.8, 24.4. **^{19}F NMR NMR (470 MHz, $CDCl_3$)** δ -71.31 (d, *J* = 14.3 Hz), -72.24 (d, *J* = 14.1 Hz). **^{11}B NMR (128 MHz, $CDCl_3$)** δ 30.38. **HRMS (EI)** calcd for $C_{23}H_{34}B_2F_2O_5$: 450.2560, found: 450.2570. **HPLC analysis**: DAICEL

CHIRALCEL OD-3, hexane/isopropanol = 99.8/0.2, 0.5mL/min, λ = 210 nm, t_R (minor) = 10.8 min, t_R (major) = 11.8 min, 96:4 er. **[α]$^{25}_{D}$**: +16.0 (*c* 0.5, $CHCl_3$).

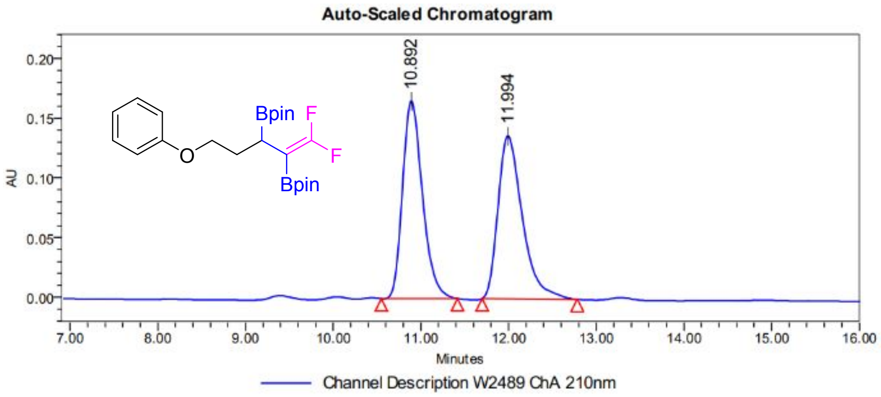

Peak Results

	Retention Time (min)	Int Type	Width (sec)	Area (μV*sec)	Height (μV)	% Area
1	10.892	bb	52.000	2616740	165691	49.85
2	11.994	bb	65.000	2632609	136312	50.15

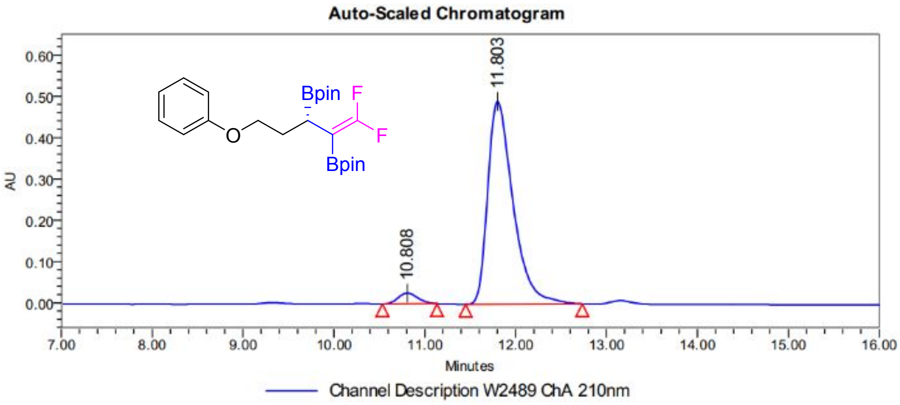

Peak Results

	Retention Time (min)	Int Type	Width (sec)	Area (μV*sec)	Height (μV)	% Area
1	10.808	bb	36.000	390307	26271	3.96
2	11.803	bb	77.000	9464254	491393	96.04

ethyl (S)-8,8-difluoro-6,7-bis(4,4,5,5-tetramethyl-1,3,2-dioxaborolan-2-yl)oct-7-enoate (83)

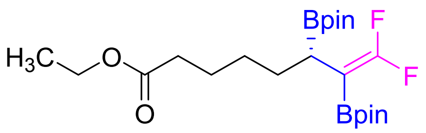

The reaction was performed following the **procedure G**. The residue was purified by flash column chromatograph (R_f = 0.4, PE: Et_2O = 10:1) to give the product as a white solid (98.9 mg, 72% yield, mp: 25.6-27 ^oC.).

^{1}H NMR (500 MHz, $CDCl_3$) δ 4.08 (q, J = 7.3 Hz, 2H), 2.24 (t, J = 7.9 Hz, 2H), 1.85 – 1.76 (m, 1H), 1.69 – 1.49 (m, 3H), 1.49 – 1.38 (m, 1H), 1.31 – 1.18 (m, 29H). **^{13}C NMR (126 MHz, $CDCl_3$)** δ 174.0, 159.8 (dd, J = 299.9, 296.6 Hz), 83.6, 83.4, 60.2, 34.5, 29.6, 28.5, 25.1, 25.0, 24.9, 24.8, 24.4, 14.3. **^{19}F NMR (471 MHz, $CDCl_3$)** δ -73.00 (d, J = 15.3 Hz), -73.97 (d, J = 14.8 Hz). **^{11}B NMR (128 MHz, $CDCl_3$)** δ 30.44. **HRMS (ESI)** calcd for $C_{22}H_{39}B_2F_2O_6$ $[M+H]^+$: 459.2895, found: 459.2892. **HPLC analysis**:

DAICEL CHIRALCEL OD-H hexane/isopropanol = 99.9/0.1, 0.5mL/min, λ = 210 nm, t_R (major) = 14.8 min, t_R (minor) = 15.7 min, 97:3 er. **[α]^{25}$_D$**: 16.6 (*c* 0.5, $CHCl_3$).

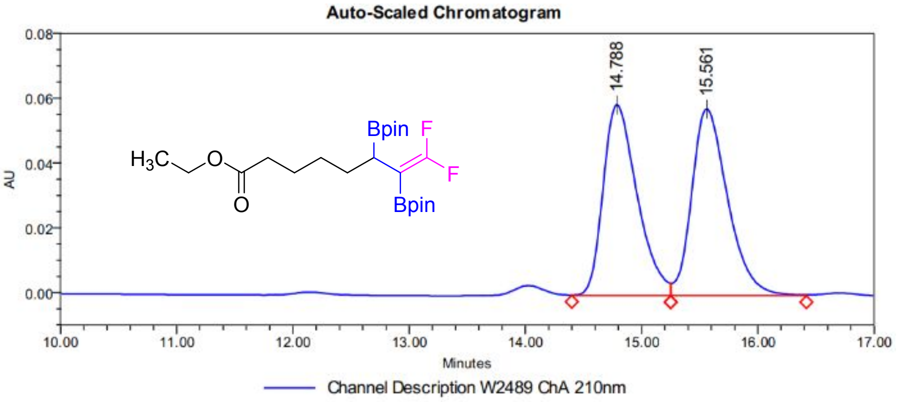

Peak Results

	Retention Time (min)	Int Type	Width (sec)	Area (µV*sec)	Height (µV)	% Area
1	14.788	VV	51.000	1198265	58989	49.11
2	15.561	VV	70.000	1241509	57683	50.89

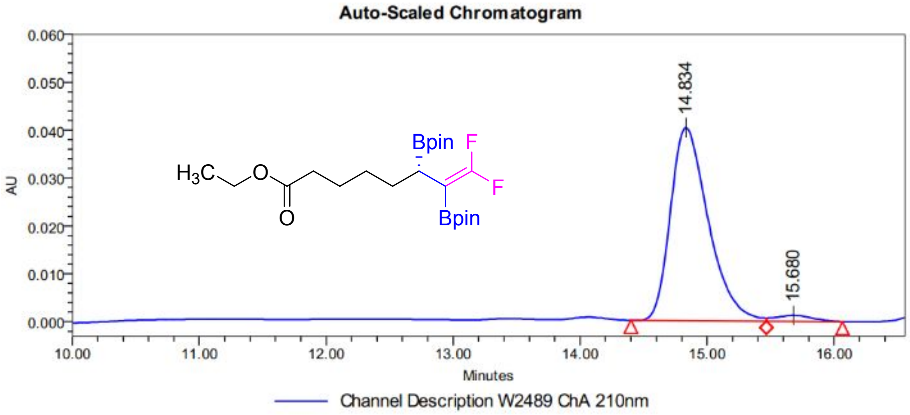

Peak Results

	Retention Time (min)	Int Type	Width (sec)	Area (µV*sec)	Height (µV)	% Area
1	14.834	bV	64.000	836596	40346	97.11
2	15.680	Vb	36.000	24936	1227	2.89

(S)-2,2'-(1-(4,4-difluorocyclohexyl)-3,3-difluoroprop-2-ene-1,2-diyl)bis(4,4,5,5-tetramethyl-1,3,2-dioxaborolane) (84)

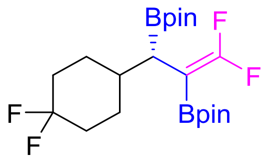

The reaction was performed following the **procedure G**. The residue was purified by flash column chromatograph (Rf = 0.5, PE: Et_2O = 10:1) to give the product as a colorless liquid (115.6 mg, 86% yield).

^{1}H NMR (500 MHz, $CDCl_3$) δ 2.07 – 1.96 (m, 2H), 1.91 (d, *J* = 13.2 Hz, 1H), 1.82 – 1.58 (m, 5H), 1.32 – 1.14 (m, 26H). **^{13}C NMR (126 MHz, $CDCl_3$)** δ 159.7 (dd, *J* = 300.7, 297.1 Hz), 123.9 (t, *J* = 240.6 Hz), 83.7, 83.4, 36.1, 33.8 (dd, *J* = 25.2, 22.6 Hz), 29.0 (d, *J* = 9.5 Hz), 28.1 (d, *J* = 9.2 Hz), 25.0, 24.8, 24.8, 24.5. **^{19}F NMR (471 MHz, $CDCl_3$)** δ -72.37, -74.71 (d, *J* = 14.4 Hz), -94.04 (d, *J* = 233.0 Hz), -

104.21 (d, J = 231.1 Hz). **^{11}B NMR (128 MHz, $CDCl_3$)** δ 30.45. **HRMS (ESI)** calcd for $C_{21}H_{35}B_2F_4O_4$ $[M+H]^+$: 449.2652, found: 449.2655. **HPLC analysis**: DAICEL CHIRALCEL OZ-3 hexane/isopropanol = 99.9/0.1, 0.5mL/min, λ = 210 nm, t_R (minor) = 14.7 min, t_R (major) = 19.1 min, 97:3 er **$[\alpha]^{25}_D$**:12.4 (c 0.5, $CHCl_3$)

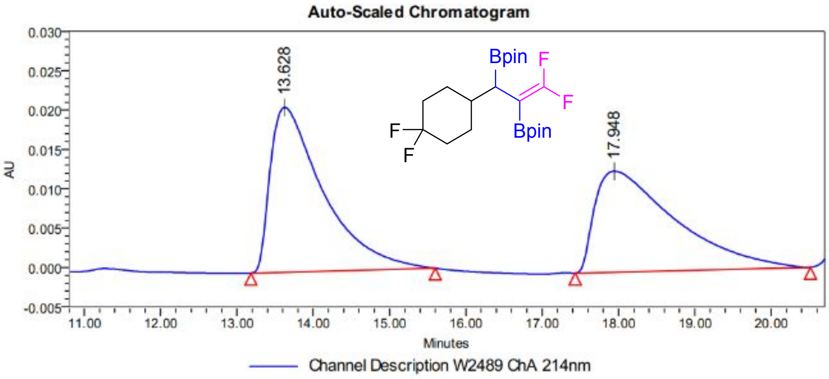

Peak Results

	Retention Time (min)	Int Type	Width (sec)	Area (µV*sec)	Height (µV)	% Area
1	13.628	Bb	145.000	1015121	20953	51.98
2	17.948	Bb	185.000	937932	12832	48.02

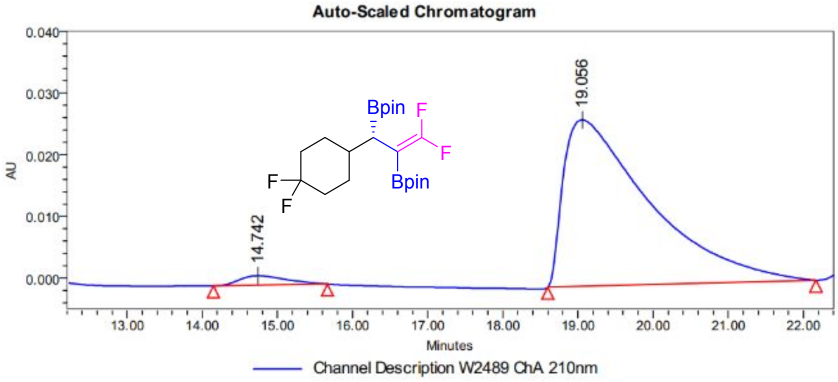

Peak Results

	Retention Time (min)	Int Type	Width (sec)	Area (µV*sec)	Height (µV)	% Area
1	14.742	bb	91.000	66068	1525	2.98
2	19.056	bb	214.000	2151285	26966	97.02

(S)-2,2'-(1-(2,3-dihydro-1H-inden-2-yl)-3,3-difluoroprop-2-ene-1,2-diyl)bis(4,4,5,5-tetramethyl-1,3,2-dioxaborolane) (85)

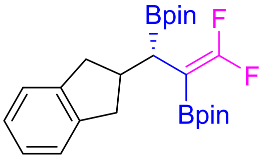

The reaction was performed following the **procedure G**. The residue was purified by flash column chromatograph (R_f = 0.4, PE: Et_2O = 10:1) to give the product as a white solid (116.4 mg, 87% yield, mp: 37.9 - 41 °C.).

^{1}H NMR (500 MHz, $CDCl_3$) δ 7.22 – 7.18 (m, 1H), 7.18 – 7.15 (m, 1H), 7.14 – 7.09 (m, 2H), 3.25 (dd, J = 15.7, 7.5 Hz, 1H), 2.99 – 2.80 (m, 2H), 2.65 – 2.57 (m, 2H), 2.01 (dd, J = 10.3, 1.9 Hz, 1H), 1.32 – 1.25 (m, 24H). **^{13}C NMR (126 MHz, $CDCl_3$)** δ 159.8 (dd, J = 299.6, 296.8 Hz), 144.2, 143.6, 126.0, 126.0, 124.3, 83.7, 83.4, 41.1, 39.8, 39.2, 25.0, 24.9, 24.5. **^{19}F NMR (471 MHz, $CDCl_3$)** δ -73.85 (d, J

= 16.5 Hz), -75.87 (d, J = 14.6 Hz). **^{11}B NMR (128 MHz, $CDCl_3$)** δ 30.76. **HRMS (ESI)** calcd for $C_{24}H_{35}B_2F_2O_4$ $[M+H]^+$: 447.2684, found: 447.2684. **HPLC analysis**: DAICEL CHIRALCEL IC-3, hexane/isopropanol = 99.9/0.1, 0.5mL/min, λ = 210 nm, t_R (minor) = 14.6 min, t_R (major) = 17.4 min, 97:3 er. **$[\alpha]^{25}_D$**: 1.6 (c 0.5, $CHCl_3$).

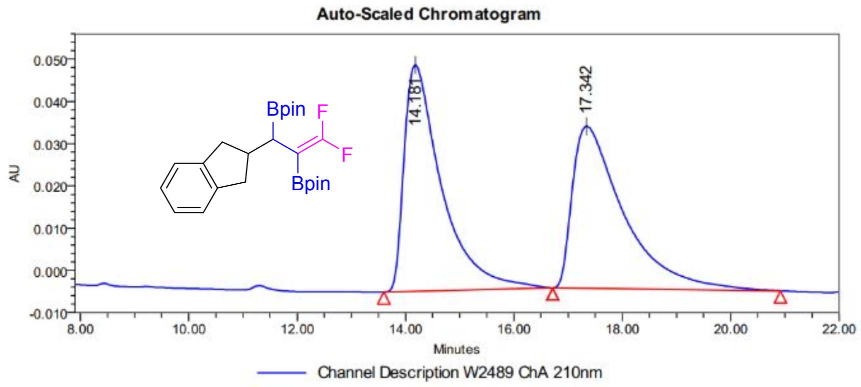

Peak Results

	Retention Time (min)	Int Type	Width (sec)	Area (µV*sec)	Height (µV)	% Area
1	14.181	BB	187.000	2576199	53526	50.53
2	17.342	Bb	252.000	2522142	38327	49.47

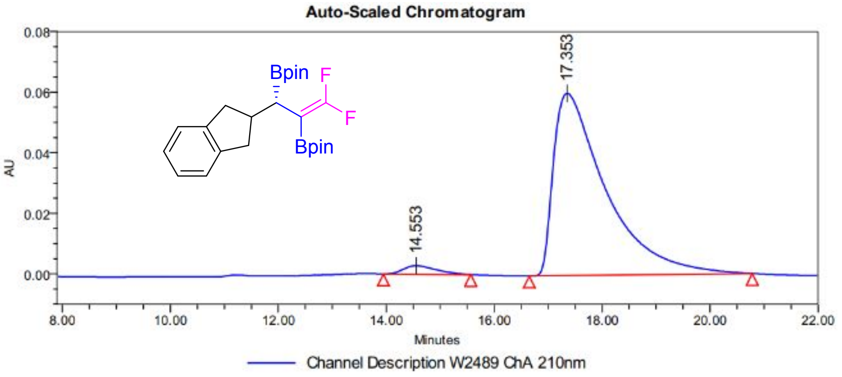

Peak Results

	Retention Time (min)	Int Type	Width (sec)	Area (µV*sec)	Height (µV)	% Area
1	14.553	bb	97.000	118452	2792	2.83
2	17.353	bb	248.000	4061654	60166	97.17

(S)-2,2'-(5-cyclopentyl-1,1-difluoropent-1-ene-2,3-diyl)bis(4,4,5,5-tetramethyl-1,3,2-dioxaborolane) (86)

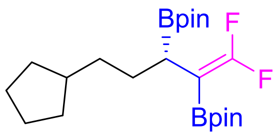

The reaction was performed following the **procedure G**. The residue was purified by flash column chromatograph (R_f = 0.4, PE: Et_2O = 10:1) to give the product as a colorless liquid (110.0 mg, 83% yield).

^{1}H NMR (500 MHz, $CDCl_3$) δ 1.85 – 1.59 (m, 5H), 1.56 – 1.51 (m, 2H), 1.48 – 1.40 (m, 3H), 1.30 – 1.15 (m, 26H), 1.12 – 0.95 (m, 2H). **^{13}C NMR (126 MHz, $CDCl_3$)** δ 159.7 (dd, J = 299.8, 296.1 Hz), 83.6, 83.3, 40.2, 35.7, 33.1, 32.7, 29.1, 25.3, 25.3, 25.1, 24.9, 24.8, 24.4. **^{19}FNMR (471 MHz, $CDCl_3$)**

δ -72.32 (d, J = 16.8 Hz), -73.30 (d, J = 16.7 Hz). **^{11}B NMR (128 MHz, CDCl$_3$)** δ 30.38. **HRMS (ESI)** calcd for $C_{22}H_{39}B_2F_2O_4$ $[M+H]^+$: 427.2997, found: 427.2994. **HPLC analysis**: DAICEL CHIRALCEL OZ-3 hexane/isopropanol = 99.9/0.1, 0.5mL/min, λ = 210 nm, t_R (major) = 8.6 min, t_R (minor) = 9.3 min, 95.5:4.5 er. **[α]^{25}$_D$**: 24.2 (c 0.5, CHCl3).

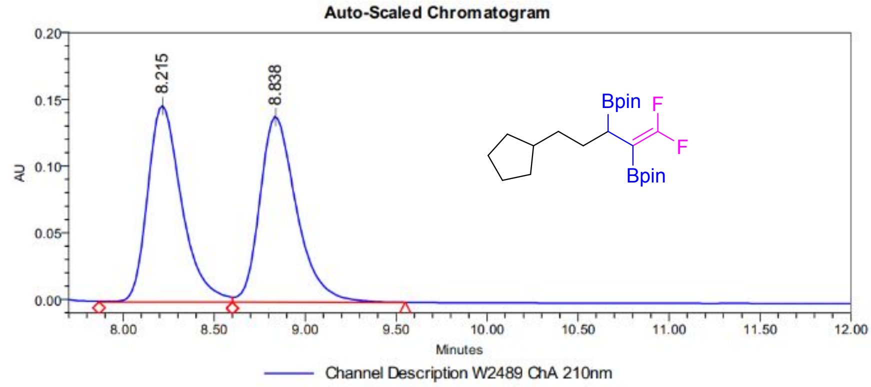

Peak Results

	Retention Time (min)	Int Type	Width (sec)	Area (µV*sec)	Height (µV)	% Area
1	8.215	VV	44.000	1912785	147023	49.57
2	8.838	VB	57.000	1945914	138989	50.43

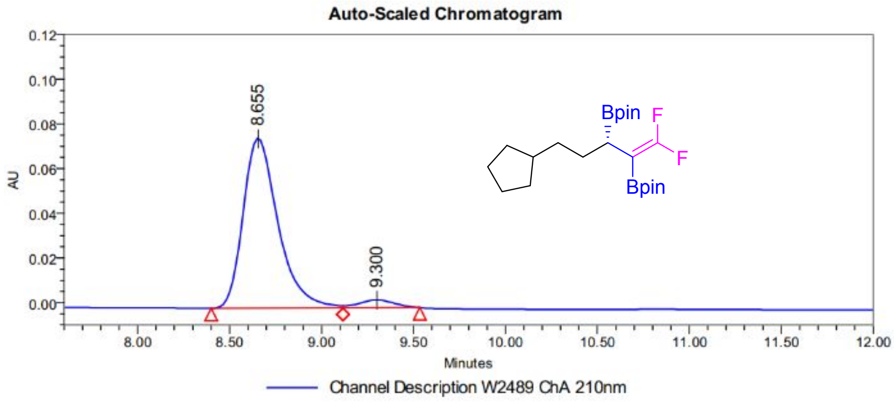

Peak Results

	Retention Time (min)	Int Type	Width (sec)	Area (µV*sec)	Height (µV)	% Area
1	8.655	bV	43.000	1005474	76154	95.52
2	9.300	Vb	25.000	47193	3517	4.48

(S)-2,2'-(5-cyclohexyl-1,1-difluoropent-1-ene-2,3-diyl)bis(4,4,5,5-tetramethyl-1,3,2-dioxaborolane) (87)

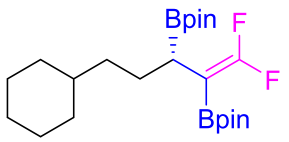

The reaction was performed following the **procedure G**. The residue was purified by flash column chromatograph (Rf = 0.4, PE: Et_2O = 10:1) to give the product as a colorless liquid (112.3 mg, 85% yield).

^{1}H NMR (500 MHz, CDCl$_3$) δ 1.76 (dt, J = 10.6, 4.5 Hz, 1H), 1.71 – 1.57 (m, 6H), 1.51 – 1.38 (m, 1H), 1.29 – 1.02 (m, 30H), 0.89 – 0.73 (m, 2H). **^{13}C NMR (126 MHz, CDCl$_3$)** δ 159.7 (dd, J = 299.9, 297.4 Hz), 83.5, 83.3, 37.8, 36.9, 33.9, 33.3, 27.2, 26.9, 26.6, 26.5, 25.1, 24.9, 24.8, 24.4. **^{19}F NMR (471 MHz,**

CDCl$_3$) δ -72.21 – -72.35 (m), -73.26 (d, *J* = 17.5 Hz). **^{11}B NMR (128 MHz, CDCl$_3$)** δ 30.26. **HRMS (ESI)** calcd for $C_{23}H_{41}B_2F_2O_4$ [M+H]$^+$: 441.3154, found: 441.3158. **HPLC analysis**: DAICEL CHIRALCEL OZ-3 hexane/isopropanol = 99.9/0.1, 0.5mL/min, λ = 210 nm, t_R (major) = 8.5 min, t_R (minor) = 9.2 min, 96:4 er. **[α]$^{25}_D$**: 10.2 (*c* 0.5, CHCl$_3$).

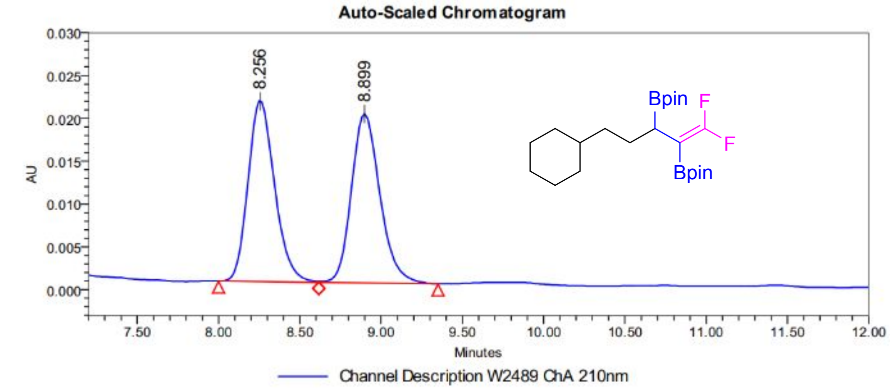

Peak Results

	Retention Time (min)	Int Type	Width (sec)	Area (µV*sec)	Height (µV)	% Area
1	8.256	BV	37.000	239617	21142	49.92
2	8.899	VB	44.000	240368	19721	50.08

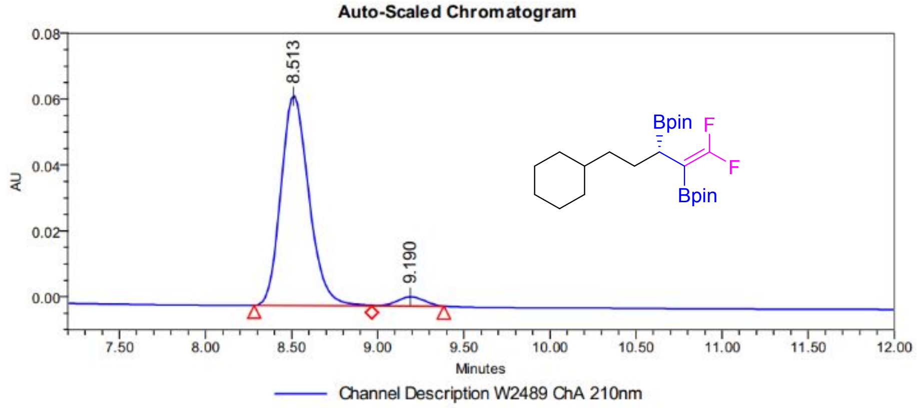

Peak Results

	Retention Time (min)	Int Type	Width (sec)	Area (µV*sec)	Height (µV)	% Area
1	8.513	bv	41.000	718625	63704	95.91
2	9.190	vb	25.000	30626	2761	4.09

2,2'-((S)-4-((3S,5S,7S)-adamantan-1-yl)-1,1-difluorobut-1-ene-2,3-diyl)bis(4,4,5,5-tetramethyl-1,3,2-dioxaborolane) (88)

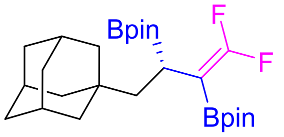

The reaction was performed following the **procedure G**. The residue was purified by flash column chromatograph (Rf = 0.4, PE: Et_2O = 10:1) to give the product as a colorless liquid (124.8 mg, 88% yield).

^{1}H NMR (500 MHz, CDCl$_3$) δ 2.01 – 1.93 (m, 1H), 1.89 (s, 3H), 1.68 – 1.52 (m, 7H), 1.49 (d, *J* = 12.2 Hz, 3H), 1.41 (d, *J* = 12.2 Hz, 3H), 1.26 – 1.17 (m, 24H). **^{13}C NMR (126 MHz, CDCl$_3$)** δ 158.5 (dd, *J*

= 300.2, 295.7 Hz), 83.6, 83.3, 44.2, 42.6, 37.3, 33.3, 28.9, 25.1, 24.8, 24.7, 24.5. **^{19}F NMR (471 MHz, $CDCl_3$)** δ -71.69 (d, J = 18.1 Hz), -74.40 (d, J = 18.4 Hz). **^{11}B NMR (128 MHz, $CDCl_3$)** δ 33.13, 30.53. **HRMS (ESI)** calcd for $C_{26}H_{43}B_2F_2O_4$ $[M+H]^+$: 479.3310, found: 479.3307 **HPLC analysis**: DAICEL CHIRALCEL OZ-3 hexane/isopropanol = 99.9/0.1, 0.5mL/min, λ = 210 nm, t_R (major) = 9.0 min, t_R (minor) = 9.7 min, 98:2 er. **$[\alpha]^{25}_D$**: 14.8 (c 0.5, $CHCl_3$).

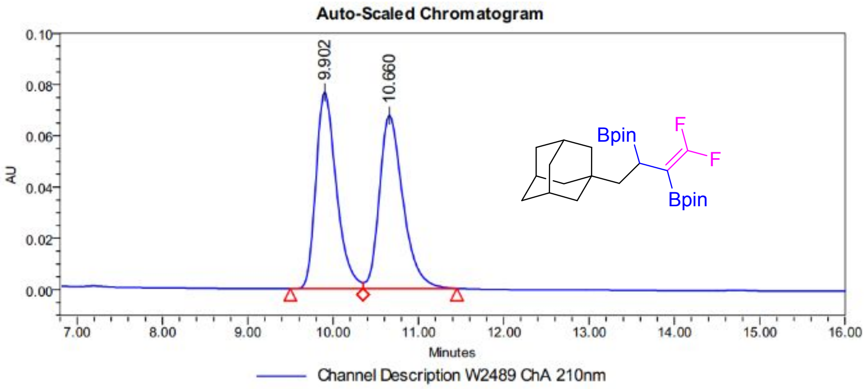

Peak Results

	Retention Time (min)	Int Type	Width (sec)	Area (μV*sec)	Height (μV)	% Area
1	9.902	BV	51.000	1281867	76712	49.62
2	10.660	VB	66.000	1301380	67574	50.38

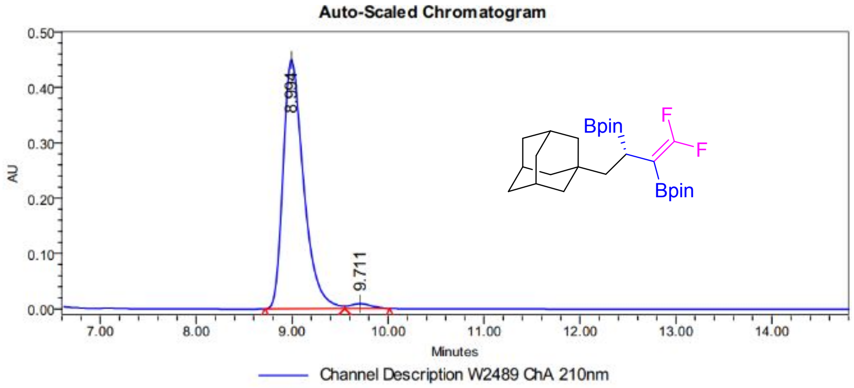

Peak Results

	Retention Time (min)	Int Type	Width (sec)	Area (μV*sec)	Height (μV)	% Area
1	8.994	bV	50.000	6700116	449772	98.05
2	9.711	Vb	28.000	133499	8540	1.95

(S)-2,2'-(3,3-difluoro-1-(4-(methylthio)phenyl)prop-2-ene-1,2-diyl)bis(4,4,5,5-tetramethyl-1,3,2-dioxaborolane) (89)

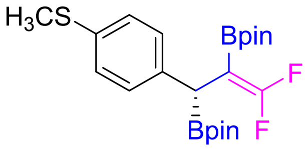

The reaction was performed following the **procedure G**. The residue was purified by flash column chromatograph (R_f = 0.4, PE: Et_2O = 10:1) to give the product as a white solid (88.0 mg, 65% yield, mp: 29.7 - 31 °C.).

^{1}H NMR (500 MHz, $CDCl_3$) δ 7.24 (d, J = 8.1 Hz, 2H), 7.15 (d, J = 8.4 Hz, 2H), 3.23 (s, 1H), 2.45 (s, 3H), 1.28 – 1.22 (m, 24H). **^{13}C NMR (126 MHz, $CDCl_3$)** δ 160.3 (dd, J = 302.4, 299.9 Hz), 139.4, 134.9,

129.9, 126.9, 83.9, 25.01, 24.97, 24.7, 24.6, 16.3. **^{19}F NMR (471 MHz, $CDCl_3$)** δ -70.04, -72.93 (d, *J* = 11.0 Hz). **^{11}B NMR (128 MHz, $CDCl_3$)** δ 30.22. **HRMS (EI)** calcd for $C_{22}H_{32}B_2F_2O_4S$: 452.2175, found: 452.2168. **HPLC analysis**: DAICEL CHIRALCEL OD-H, hexane/isopropanol = 99.9/0.1, 0.5mL/min, λ = 210 nm, t_R (minor) = 13.5 min, t_R (major) = 14.6 min, 90:10 er. **$[\alpha]^{25}_D$**: -1.4 (*c* 0.5, $CHCl_3$).

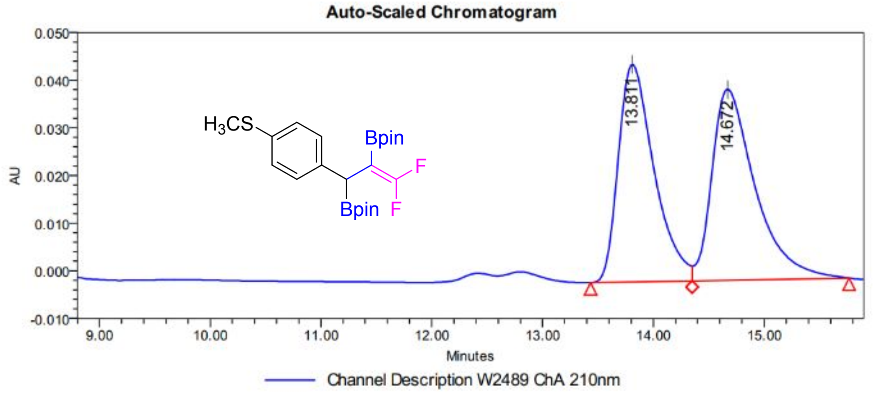

Peak Results

	Retention Time (min)	Int Type	Width (sec)	Area (μV*sec)	Height (μV)	% Area
1	13.811	bV	55.000	1025575	45667	48.56
2	14.672	Vb	85.000	1086471	40100	51.44

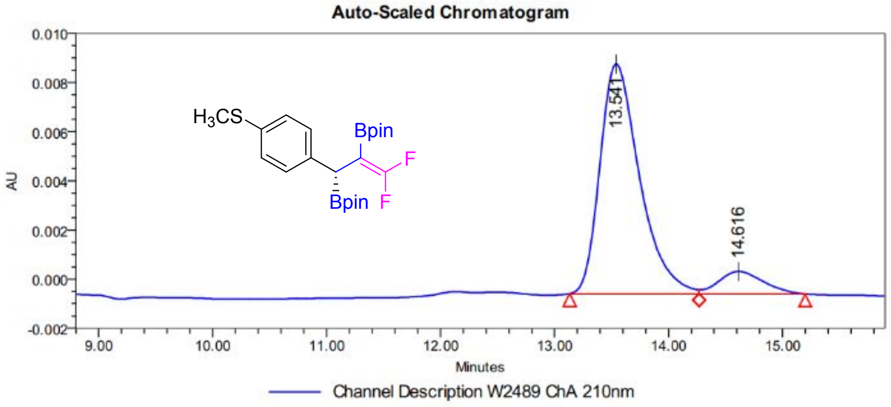

Peak Results

	Retention Time (min)	Int Type	Width (sec)	Area (μV*sec)	Height (μV)	% Area
1	13.541	BV	68.000	225572	9357	90.11
2	14.616	Vb	56.000	24756	913	9.89

(S)-2,2'-(1-(4-(tert-butyl)phenyl)-3,3-difluoroprop-2-ene-1,2-diyl)bis(4,4,5,5-tetramethyl-1,3,2-dioxaborolane) (90)

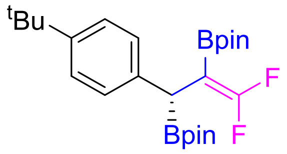

The reaction was performed following the **procedure G**. The residue was purified by flash column chromatograph (Rf= 0.5, PE: Et_2O = 10:1) to give the product as a colorless liquid (98.5 mg, 71% yield). **^{1}H NMR (500 MHz, $CDCl_3$)** δ 7.24 (s, 4H), 3.26 (s, 1H), 1.32 – 1.26 (m, 24H), 1.23 (s, 9H). **^{13}C NMR (126 MHz, $CDCl_3$)** δ 160.2 (dd, *J* = 301.1, 297.4 Hz), 148.1, 138.9, 129.1, 125.1, 83.9, 83.8, 34.4, 31.6, 25.0, 25.0, 24.7, 24.6. **^{19}F NMR (471 MHz, $CDCl_3$)** δ -71.81 (d, *J* = 14.9 Hz), -74.83 (d, *J* = 14.4 Hz).

^{11}B NMR (128 MHz, $CDCl_3$) δ 30.53. **HRMS (ESI)** calcd for $C_{25}H_{39}B_2F_2O_4$ $[M+H]^+$: 463.2997, found: 463.2994. **HPLC analysis**: DAICEL CHIRALCEL OZ-3 hexane/isopropanol = 99.9/0.1, 0.5mL/min, λ = 210 nm, t_R (major) = 11.8 min, t_R (minor) = 13.7 min, 93:7 er. **$[\alpha]^{25}_D$**: 12.8 (*c* 0.5, $CHCl_3$).

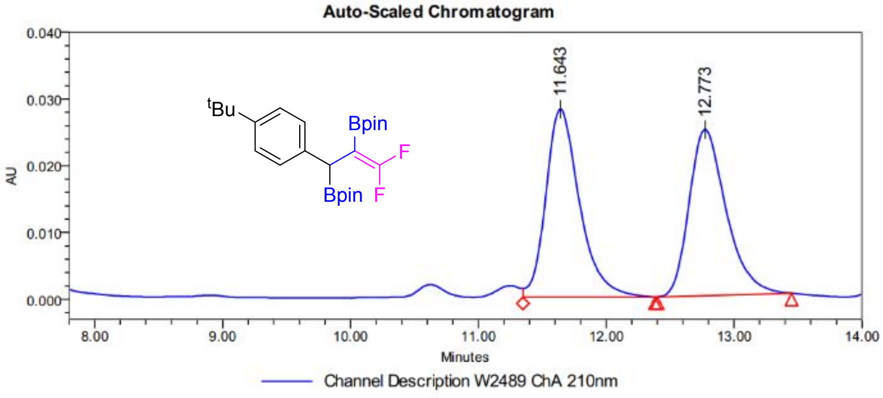

Peak Results

	Retention Time (min)	Int Type	Width (sec)	Area (μV*sec)	Height (μV)	% Area
1	11.643	VB	62.000	516179	28189	50.78
2	12.773	BB	63.000	500230	24910	49.22

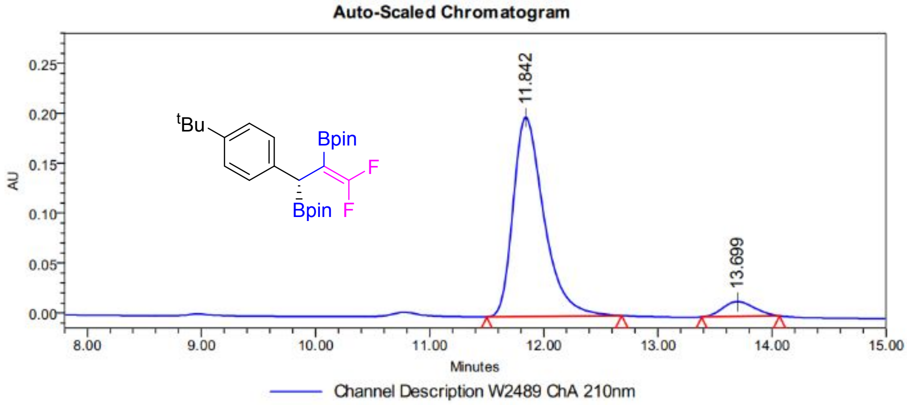

Peak Results

	Retention Time (min)	Int Type	Width (sec)	Area (μV*sec)	Height (μV)	% Area
1	11.842	bb	71.000	3744691	199715	92.90
2	13.699	bb	41.000	286282	14739	7.10

(S)-2,2'-(3,3-difluoro-1-(4-methoxyphenyl)prop-2-ene-1,2-diyl)bis(4,4,5,5-tetramethyl-1,3,2-dioxaborolane) (91)

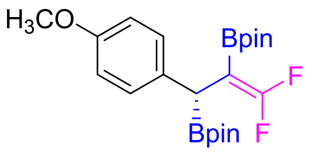

The reaction was performed following the **procedure G**. The residue was purified by flash column chromatograph (Rf = 0.4, PE: Et_2O = 10:1) to give the product as a colorless liquid (87.7 mg, 67% yield). **^{1}H NMR (400 MHz, $CDCl_3$)** δ 7.23 (d, *J* = 7.5 Hz, 2H), 6.77 (d, *J* = 7.6 Hz, 2H), 3.76 (s, 3H), 3.22 (s, 1H), 1.30 – 1.18 (m, 24H). **^{13}C NMR (101 MHz, $CDCl_3$)** δ 160.1 (dd, J = 300.8, 297.8 Hz), 157.5, 134.2, 130.3, 113.5, 83.8, 83.7, 55.1, 24.9, 24.9, 24.6, 24.5. **^{19}F NMR (376 MHz, $CDCl_3$)** δ -70.77 (d, *J* = 13.1 Hz), -73.63 (d, *J* =12.9 Hz). **^{11}B NMR (128 MHz, $CDCl_3$)** δ 33.26, 31.27. **HRMS (EI)** calcd for

$C_{22}H_{32}B_2F_2O_5$: 436.2404, found: 436.2400. DAICEL CHIRALCEL OZ-3 hexane/isopropanol = 99.9/0.1, 0.5mL/min, λ = 210 nm, t_R (major) = 14.1 min, t_R (minor) = 15.9 min, 93:7 er. **[α]$^{25}_D$**: 16.0 (*c* 0.5, CHCl3).

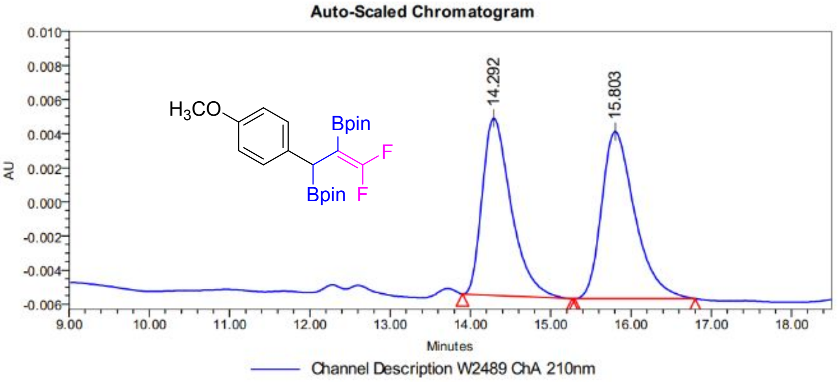

Peak Results

	Retention Time (min)	Int Type	Width (sec)	Area (μV*sec)	Height (μV)	% Area
1	14.292	BB	82.000	260587	10381	48.02
2	15.803	Bb	90.000	282049	9798	51.98

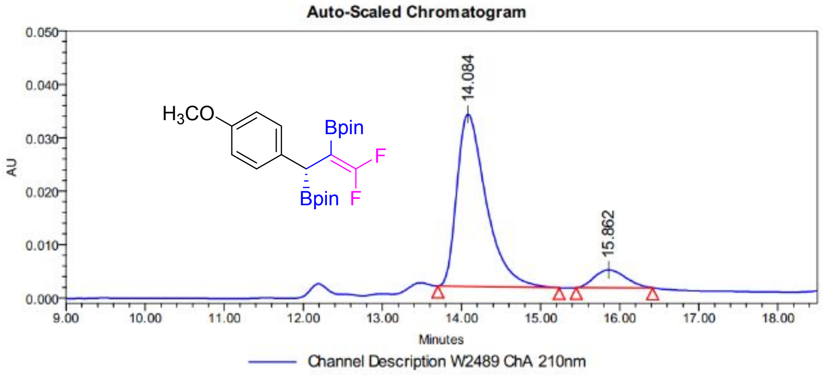

Peak Results

	Retention Time (min)	Int Type	Width (sec)	Area (μV*sec)	Height (μV)	% Area
1	14.084	bb	92.000	832467	32298	89.95
2	15.862	bb	58.000	92989	3340	10.05

(S)-2,2'-(3,3-difluoro-1-(4-(trifluoromethoxy)phenyl)prop-2-ene-1,2-diyl)bis(4,4,5,5-tetramethyl-1,3,2-dioxaborolane) (92)

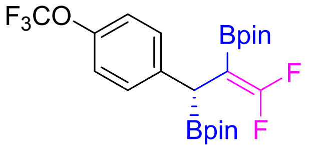

The reaction was performed following the **procedure G**. The residue was purified by flash column chromatograph (Rf = 0.4, PE: Et_2O = 10:1) to give the product as a colorless liquid (89.7 mg, 61% yield.). **^{1}H NMR (500 MHz, CDCl$_3$)** δ 7.32 (d, *J* = 8.7 Hz, 2H), 7.07 (d, *J* = 7.7 Hz, 2H), 3.28 (s, 1H), 1.29 – 1.20 (m, 24H). **^{13}C NMR (126 MHz, CDCl$_3$)** δ 160.3 (t, *J* = 300 Hz), 147.3, 140.9, 130.7, 121.1 (q, *J* = 253.8 Hz), 120.6, 84.1, 84.0, 25.0, 24.7, 24.5. **^{19}F NMR (470MHz, CDCl$_3$)** δ -57.71, -70.04 (d, *J* = 12.6 Hz), -72.83 (d, *J* = 11.1 Hz). **^{11}B NMR (128 MHz, CDCl$_3$)** δ 30.74. **HRMS (ESI)** calcd for $C_{22}H_{30}B_2F_5O_5$ $[M+H]^+$: 491.2194, found: 491.2196. DAICEL CHIRALCEL OZ-3 hexane/isopropanol = 99.8/0.2, 0.5mL/min, λ = 210 nm, t_R (major) = 7.5 min, t_R (minor) = 8.4 min, 87:13 er **[α]$^{25}_D$**: 10.8 (*c* 0.5, CHCl3).

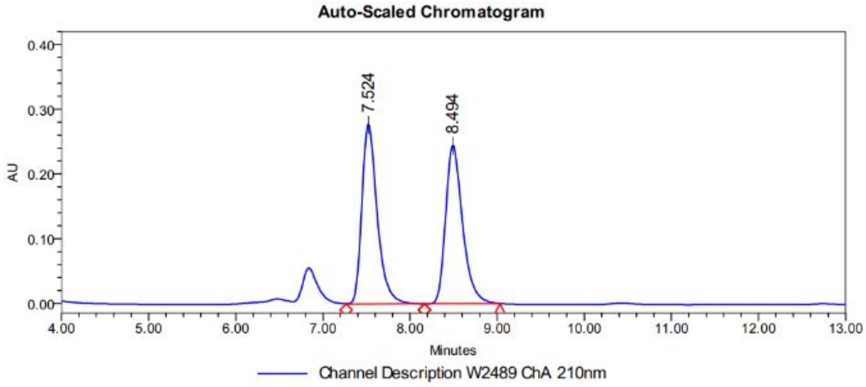

Peak Results

	Retention Time (min)	Int Type	Width (sec)	Area (μV*sec)	Height (μV)	% Area
1	7.524	VV	54.000	3398766	278543	50.49
2	8.494	Vb	52.000	3332777	244534	49.51

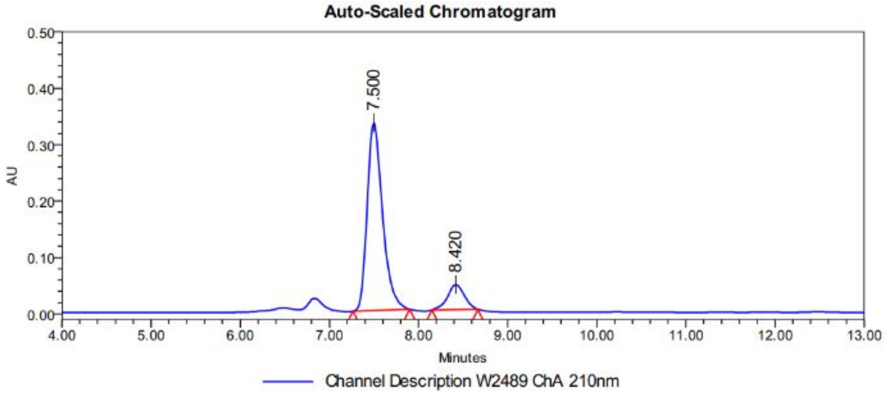

Peak Results

	Retention Time (min)	Int Type	Width (sec)	Area (μV*sec)	Height (μV)	% Area
1	7.500	bb	38.000	3978219	332387	87.20
2	8.420	bb	31.000	583961	44392	12.80

(S)-2,2'-(4-(3-chlorophenyl)-1,1-difluorobut-1-ene-2,3-diyl)bis(4,4,5,5-tetramethyl-1,3,2-dioxaborolane) (93)

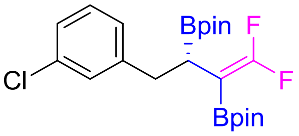

The reaction was performed following the **procedure H**. The residue was purified by flash column chromatograph (R_f = 0.4, PE: Et_2O = 10:1) to give the product as a white solid (119.8 mg, 88% yield, mp: 46.3-47 ^oC.).

^{1}H NMR (500 MHz, $CDCl_3$) δ 7.16 (d, J = 1.9 Hz, 1H), 7.15 – 7.09 (m, 2H), 7.00 (d, J = 7.3 Hz, 1H), 3.03 – 2.99 (m, 1H), 2.71 (dd, J = 13.9, 10.9 Hz, 1H), 2.25 – 2.07 (m, 1H), 1.27 – 1.21 (m, 24H). **^{13}C NMR (126 MHz, $CDCl_3$)** δ 159.9 (dd, J = 300.8, 297.9 Hz), 144.3, 133.8, 129.3, 129.2, 127.2, 125.9, 83.7, 83.7, 35.9, 25.1, 24.9, 24.8, 24.5. **^{19}F NMR (471 MHz, $CDCl_3$)** δ -70.68 (d, J = 13.0 Hz), -72.45 (d, J = 13.2 Hz). **^{11}B NMR (128 MHz, $CDCl_3$)** δ 30.18. **HRMS (ESI)** calcd for $C_{22}H_{32}B_2ClF_2O_4$ $[M+H]^+$: 455.2138, found: 455.2135. **HPLC analysis**: DAICEL CHIRALCEL OZ-3 hexane/isopropanol = 99.9/0.1, 0.5mL/min, λ = 210 nm, t_R (major) = 9.8 min, t_R (minor) = 10.7 min, 93:7 er **$[\alpha]^{25}_D$**: 33.4 (c 0.5,

CHCl3).

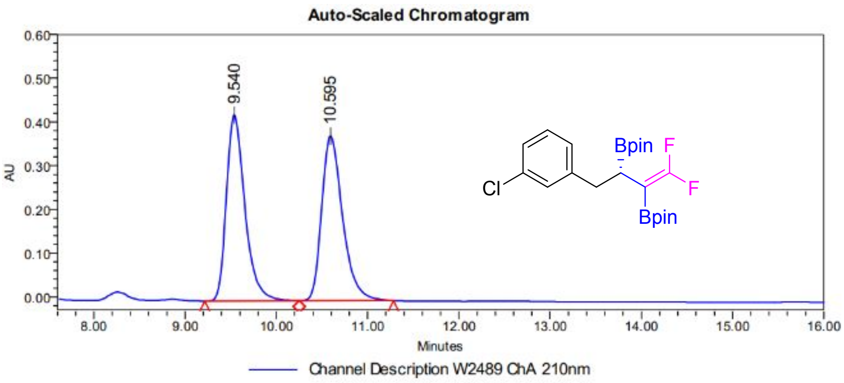

Peak Results

	Retention Time (min)	Int Type	Width (sec)	Area (µV*sec)	Height (µV)	% Area
1	9.540	BV	62.000	6001950	426205	49.98
2	10.595	Vb	62.000	6005748	377055	50.02

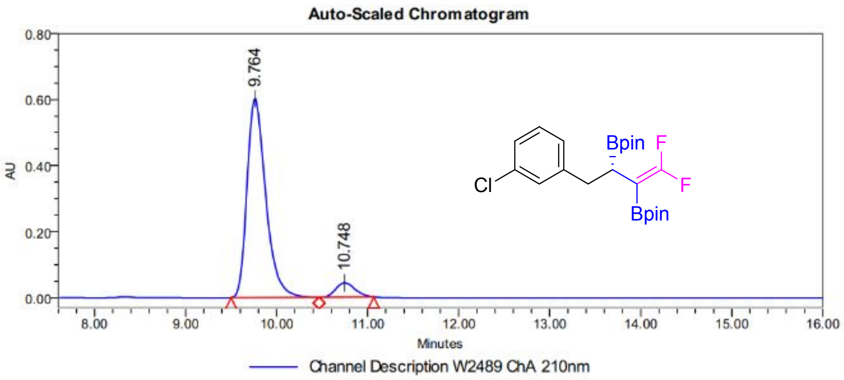

Peak Results

	Retention Time (min)	Int Type	Width (sec)	Area (µV*sec)	Height (µV)	% Area
1	9.764	bV	58.000	8605750	602881	93.15
2	10.748	Vb	36.000	632911	43131	6.85

(S)-4-(4,4-difluoro-2,3-bis(4,4,5,5-tetramethyl-1,3,2-dioxaborolan-2-yl)but-3-en-1-yl)benzonitrile (94)

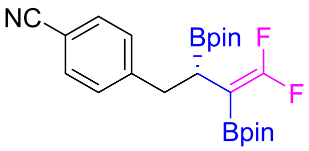

The reaction was performed following the **procedure H**. The residue was purified by flash column chromatograph (R_f = 0.3, PE: Et_2O = 10:1) to give the product as a white solid (113.5 mg, 85% yield, mp: 81.3-82.6 °C.).

^{1}H NMR (500 MHz, $CDCl_3$) δ 7.52 (d, J = 8.3 Hz, 2H), 7.24 (d, J = 8.2 Hz, 2H), 3.11 – 3.04 (m, 1H), 2.79 (dd, J = 13.8, 11.1 Hz, 1H), 2.23 – 2.12 (m, 1H), 1.27 – 1.23 (m, 24H). **^{13}C NMR (126 MHz, $CDCl_3$)** δ 159.9 (dd, J = 299.9, 298.6 Hz), 148.1, 132.0, 129.8, 119.4, 109.6, 83.9, 83.8, 36.4, 25.1, 25.0, 24.8, 24.5. **^{19}F NMR (471 MHz, $CDCl_3$)** δ -70.51 (d, J = 12.24 Hz), -72.12 (d, J = 12.4 Hz). **^{11}B NMR (128 MHz, $CDCl_3$)** δ 33.02. **HRMS (ESI)** calcd for $C_{23}H_{32}B_2F_2NO_4$ $[M+H]^+$: 446.2480, found: 446.2483. **HPLC analysis**: DAICEL CHIRALCEL ID-3 hexane/isopropanol = 99.9/0.1, 0.5mL/min, λ = 210 nm,

t_R (major) = 21.4 min, t_R (minor) = 23.5 min, 93:7 er **[α]^{25}$_D$**:12.8 (*c* 0.5, $CHCl_3$).

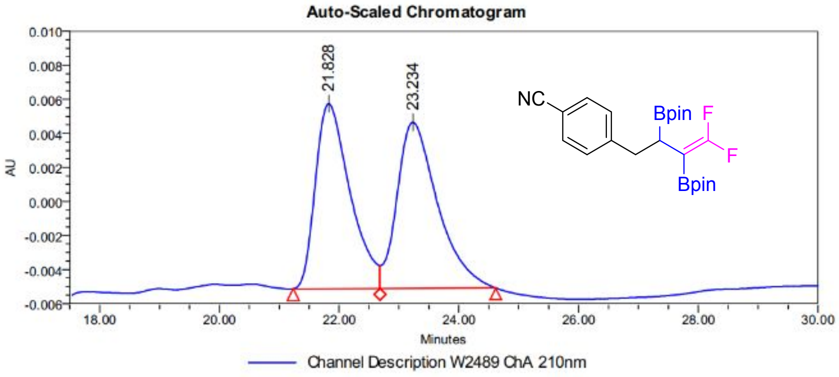

Peak Results

	Retention Time (min)	Int Type	Width (sec)	Area (µV*sec)	Height (µV)	% Area
1	21.828	BV	87.000	437152	10876	48.70
2	23.234	Vb	116.000	460433	9758	51.30

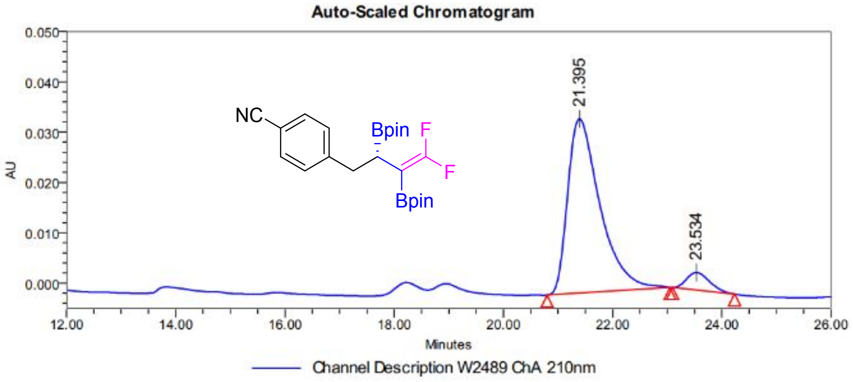

Peak Results

	Retention Time (min)	Int Type	Width (sec)	Area (µV*sec)	Height (µV)	% Area
1	21.395	Bb	135.000	1424124	34639	93.28
2	23.534	Bb	68.000	102667	3488	6.72

(S)-(4-chlorophenyl)(3-(4,4-difluoro-2,3-bis(4,4,5,5-tetramethyl-1,3,2-dioxaborolan-2-yl)but-3-en-1-yl)-2-methyl-1H-indol-1-yl)methanone (95)

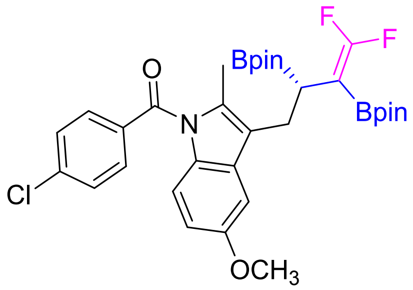

The reaction was performed following the **procedure G**. The residue was purified by flash column chromatograph (R_f = 0.25, PE: Et_2O = 4:1) to give the product as a Light yellow liquid (115.5 mg, 63% yield.).

^{1}H NMR (500 MHz, $CDCl_3$) δ 7.60 (d, *J* = 8.5 Hz, 2H), 7.44 (d, *J* = 8.4 Hz, 2H), 7.06 – 6.98 (m, 2H), 6.65 (dd, *J* = 8.9, 2.6 Hz, 1H), 3.84 (s, 3H), 3.07 (dd, *J* = 14.7, 5.4 Hz, 1H), 2.72 (dd, *J* = 14.2, 10.3 Hz,

1H), 2.33 – 2.24 (m, 1H), 2.23 (s, 3H), 1.27 – 1.17 (m, 24H) **^{13}C NMR (126 MHz, $CDCl_3$)** δ 168.4, 160.1 (dd, *J* = 301.1, 298.6 Hz), 155.9, 138.9, 134.6, 134.4, 131.5, 131.3, 131.2, 129.1, 119.7, 114.8, 110.8, 102.4, 83.7, 83.6, 55.8, 27.0, 25.1, 24.8, 24.6, 14.0. **^{19}F NMR (471 MHz, $CDCl_3$)** δ -70.75 (d, *J* = 12.5 Hz), -72.46 (d, *J* = 12.7 Hz). **^{11}B NMR (128 MHz, $CDCl_3$)** δ 30.01. **HRMS (ESI)** calcd for $C_{33}H_{41}B_2ClF_2NO_6$ $[M+H]^+$: 642.2771, found: 642.2775. DAICEL CHIRALCEL OD-3 hexane/isopropanol = 99.5/0.5, 0.5mL/min, λ = 254 nm, t_R (minor) = 24.8 min, t_R (major) = 26.4 min, 88:12 er **$[\alpha]^{25}_D$**: 8.7 (*c* 0.5, $CHCl_3$).

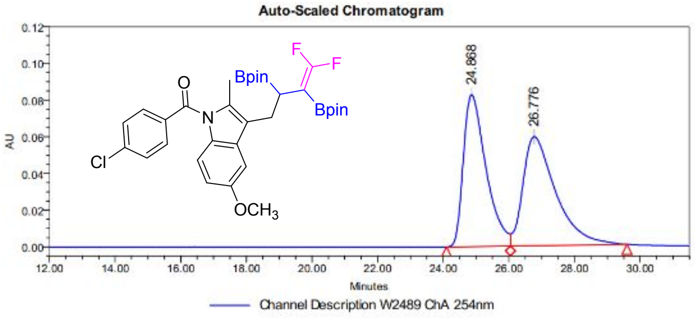

Peak Results

	Retention Time (min)	Int Type	Width (sec)	Area (µV*sec)	Height (µV)	% Area
1	24.868	BV	117.000	3971566	82830	49.02
2	26.776	Vb	213.000	4130711	59401	50.98

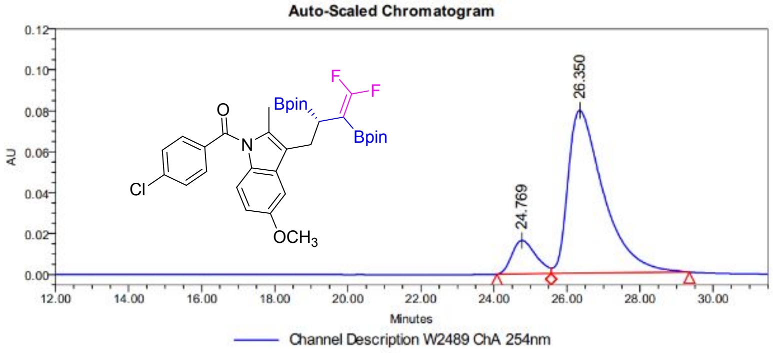

Peak Results

	Retention Time (min)	Int Type	Width (sec)	Area (µV*sec)	Height (µV)	% Area
1	24.769	bv	89.000	742286	16351	12.09
2	26.350	vb	227.000	5397493	79519	87.91

tert-butyl(((3R,8R,9S,10S,13R,14S,17R)-17-((2R,5S)-7,7-difluoro-5,6-bis(4,4,5,5-tetramethyl-1,3,2-dioxaborolan-2-yl)hept-6-en-2-yl)-10,13-dimethylhexadecahydro-1H-cyclopenta[a]phenanthren-3-yl)oxy)dimethylsilane (96)

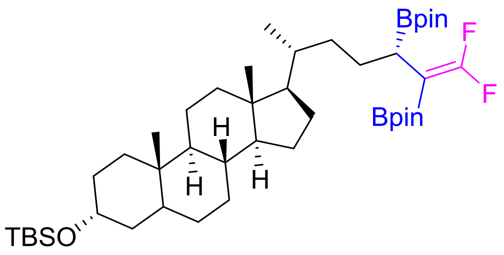

The reaction was performed following the **procedure G**. The residue was purified by flash column chromatograph (R_f = 0.35, PE: Et_2O = 20:1) to give the product as a white solid (167.0 mg, 72% yield,

mp: 73-74.5 °C.).

^{1}H NMR (500 MHz, CDCl$_3$) δ 3.59 – 3.53 (m, 1H), 1.94 – 1.90 (m, 1H), 1.84 -1.71 (m, 5H), 1.57 – 1.47 (m, 2H), 1.43 – 1.28 (m, 14H), 1.25 – 1.21 (m, 29H), 1.13 – 1.03 (m, 2H), 1.02 – 0.95 (m, 2H), 0.89 – 0.84 (m, 20H), 0.59 (s, 3H), 0.04 (s, 10H). **^{13}C NMR (126 MHz, CDCl$_3$)** δ 159.7 (dd, J = 299.3, 296.5 Hz), 83.5, 83.3, 73.0, 56.6, 56.2, 42.8, 42.5, 40.4, 40.3, 37.1, 36.0, 35.7, 35.6, 35.3, 34.7, 31.2, 28.3, 27.5, 26.6, 26.2, 26.1, 25.1, 24.9, 24.8, 24.5, 24.3, 23.5, 20.9, 18.8, 18.5, 12.1, -4.5. **^{19}F NMR (471 MHz, CDCl$_3$)** δ -72.31 (d, J = 16.5 Hz), -73.27 (d, J = 18.3 Hz). **^{11}B NMR (128 MHz, CDCl$_3$)** δ 29.36. **HRMS (ESI)** calcd for $C_{44}H_{79}B_2F_2O_5Si$ [M+H]$^+$: 775.5845, found: 775.5848. **HPLC analysis**: DAICEL CHIRALCEL OZ-3 hexane/isopropanol = 99.9/0.1, 0.5mL/min, λ = 210 nm, t_R (minor) = 10.4 min, t_R (major) = 11.0 min, 97:3 er **[α]^{25}$_D$**: 30.2 (c 0.5, CHCl3)

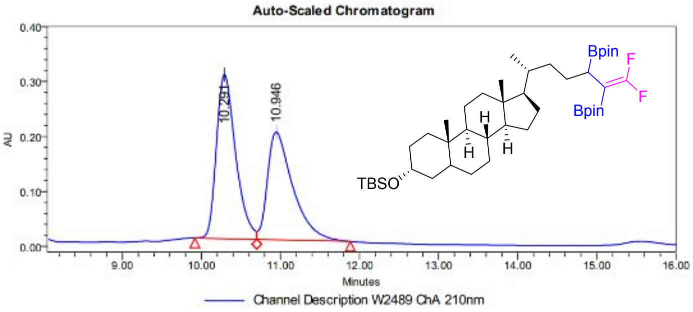

Peak Results

	Retention Time (min)	Int Type	Width (sec)	Area (µV*sec)	Height (µV)	% Area
1	10.291	bV	47.000	5036647	299124	53.40
2	10.946	Vb	71.000	4395266	196194	46.60

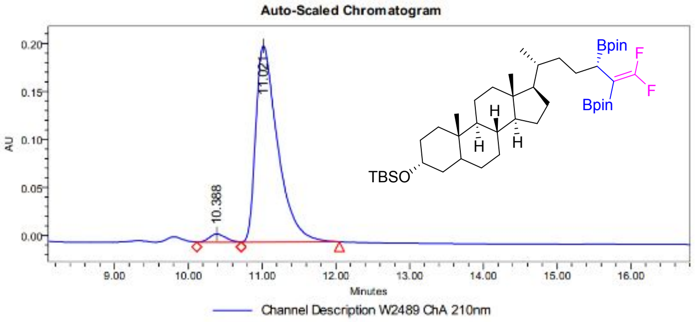

Peak Results

	Retention Time (min)	Int Type	Width (sec)	Area (µV*sec)	Height (µV)	% Area
1	10.388	VV	36.000	129632	8363	2.97
2	11.021	Vb	80.000	4237675	204566	97.03

(8R,9S,13S,14S,17S)-3-(((S)-5,5-difluoro-3,4-bis(4,4,5,5-tetramethyl-1,3,2-dioxaborolan-2-yl)pent-4-en-1-yl)oxy)-13-methyl-7,8,9,11,12,13,14,15,16,17-decahydro-6H-cyclopenta[a]phenanthren-17-yl propionate (97)

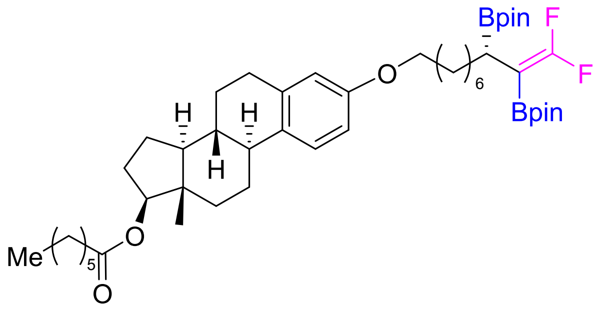

The reaction was performed following the **procedure G**. The residue was purified by flash column chromatograph (Rf = 0.35, PE: Et_2O = 20:1) to give the product as a colorless liquid (137.5 mg, 67% yield.).

^{1}H NMR (500 MHz, $CDCl_3$) δ 7.17 (d, J = 9.1 Hz, 1H), 6.69 (dd, J = 8.6, 2.7 Hz, 1H), 6.62 (d, J = 2.6 Hz, 1H), 4.73 – 4.65 (m, 1H), 3.90 (t, J = 6.6 Hz, 2H), 2.90 – 2.78 (m, 2H), 2.34 – 2.25 (m, 3H), 1.91 – 1.79 (m, 3H), 1.78 – 1.69 (m, 3H), 1.67 – 1.58 (m, 3H), 1.56 – 1.28 (m, 24H), 1.26 – 1.22 (m, 24H), 0.91 – 0.87 (m, 3H), 0.82 (s, 3H). **^{13}C NMR (126 MHz, $CDCl_3$)** δ 174.2, 159.8 (dd, J = 299.9, 297.4 Hz), 157.2, 137.9, 132.4, 126.4, 114.6, 112.2, 83.6, 83.3, 82.6, 68.0, 49.9, 43.9, 43.1, 38.7, 37.1, 34.7, 31.6, 30.0, 29.9, 29.6, 29.5, 29.5, 28.9, 27.7, 27.4, 26.3, 26.2, 25.2, 25.1, 24.9, 24.8, 24.7, 24.4, 23.4, 22.6, 14.1, 12.2. **^{19}F NMR (471 MHz, $CDCl_3$)** δ -72.16 (d, J = 17.6 Hz), -73.20 (d, J = 16.7 Hz). **^{11}B NMR (128 MHz, $CDCl_3$)** δ 30.22. **HRMS (ESI)** calcd for $C_{47}H_{75}B_2F_2O_7$ $[M+H]^+$: 811.5661, found: 811.5666. **HPLC analysis**: DAICEL CHIRALCEL OZ-3 hexane/isopropanol = 99/1, 0.5mL/min, λ = 210 nm, t_R (major) = 14.3 min, t_R (minor) = 17.0 min, 88:12 er **$[\alpha]^{25}_D$**: 18.0 (c 0.5, $CHCl_3$)

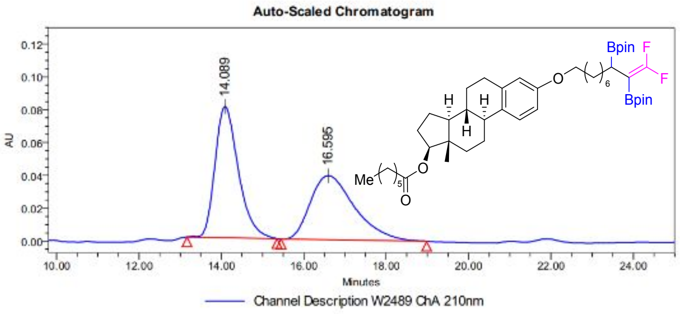

Peak Results

	Retention Time (min)	Int Type	Width (sec)	Area (µV*sec)	Height (µV)	% Area
1	14.089	bb	131.000	3143049	79905	51.89
2	16.595	bb	212.000	2914433	39067	48.11

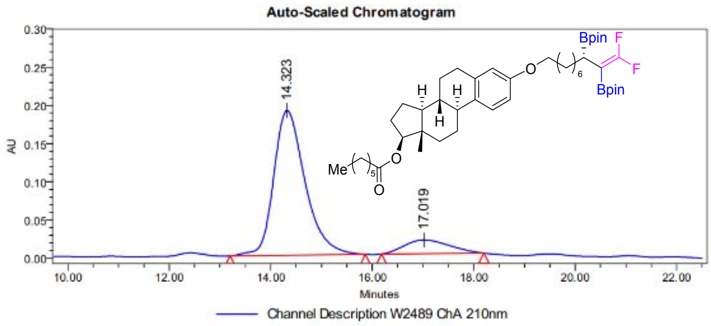

Peak Results

	Retention Time (min)	Int Type	Width (sec)	Area (µV*sec)	Height (µV)	% Area
1	14.323	bb	160.000	8132482	189401	87.97
2	17.019	bb	121.000	1112566	17863	12.03

methyl (S)-4-(1,1-difluoro-3-hydroxy-5-phenylpent-1-en-2-yl)benzoate (98)

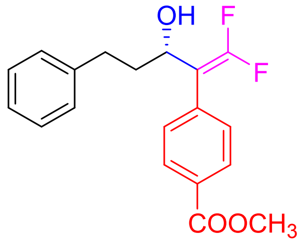

The reaction was performed following the **procedure I**. The residue was purified by flash column

chromatograph (Rf = 0.35, PE: Et_2O = 6:1) to give the product as a colorless liquid (66.7 mg, 67% yield). **^{1}H NMR (500 MHz, $CDCl_3$)** δ 8.02 (d, J = 7.2 Hz, 2H), 7.48 (d, J = 7.5 Hz, 2H), 7.25 (t, J = 7.0 Hz, 2H), 7.17 (t, J = 7.5 Hz, 1H), 7.09 (d, J = 7.2 Hz, 2H), 4.73 (s, 1H), 3.91 (s, 3H), 2.71 – 2.57 (m, 2H), 2.25 (s, 1H), 1.92 – 1.85 (m, 1H), 1.8 – 1.71 (m, 1H). **^{13}C NMR (126 MHz, $CDCl_3$)** δ 167.0, 154.4 (t, J = 292.9 Hz), 141.1, 135.8, 130.1, 129.7, 128.6, 128.4, 126.2, 95.5 (dd, J = 18.1, 11.7 Hz), 68.2 (d, J = 4.2 Hz), 52.3, 36.9, 32.1. **^{19}F NMR (471 MHz, $CDCl_3$)** δ -87.02 (d, J = 36.1 Hz), -88.88 (d, J = 34.5 Hz). **HRMS (ESI)** calcd for $C_{19}H_{19}F_2O_3$ $[M+H]^+$: 333.1297, found: 333.1300. **HPLC analysis**: DAICEL CHIRALCEL AD-H hexane/isopropanol = 90/10, 1.0mL/min, λ = 254 nm, t_R (minor) = 10.1 min, t_R (major) = 13.6 min, 97:3 er **$[\alpha]^{25}_D$**: -28.2 (c 0.5, $CHCl_3$)

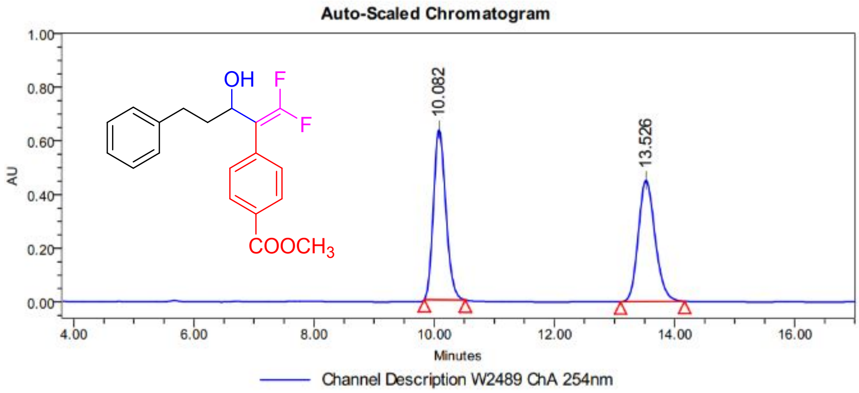

Peak Results

	Retention Time (min)	Int Type	Width (sec)	Area (µV*sec)	Height (µV)	% Area
1	10.082	bb	41.000	9064335	633781	50.74
2	13.526	bb	64.000	8799816	451476	49.26

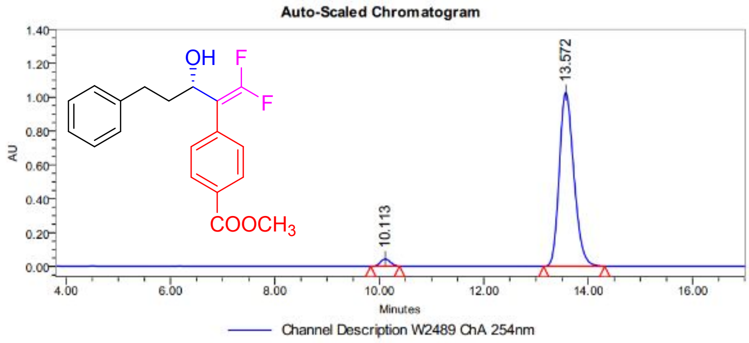

Peak Results

	Retention Time (min)	Int Type	Width (sec)	Area (µV*sec)	Height (µV)	% Area
1	10.113	bb	33.000	569530	42555	2.78
2	13.572	bb	70.000	19920233	1026786	97.22

methyl (S)-4-(1,1-difluoro-3-hydroxy-5-(4-(trifluoromethyl)phenyl)pent-1-en-2-yl)benzoate (99)

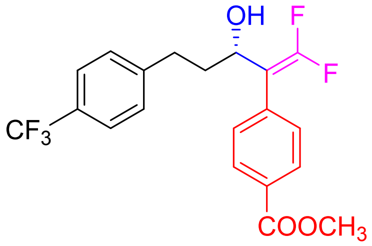

The reaction was performed following the **procedure I**. The residue was purified by flash column chromatograph (Rf = 0.35, PE: Et_2O = 6:1) to give the product as a colorless liquid (80.4 mg, 67% yield). **^{1}H NMR (500 MHz, CDCl$_3$)** δ 7.93 (d, J = 8.3 Hz, 2H), 7.39 (t, J = 7.4 Hz, 4H), 7.10 (d, J = 8.2 Hz, 2H), 4.63 (t, J = 7.1 Hz, 1H), 3.82 (s, 3H), 2.71 – 2.51 (m, 2H), 1.84 – 1.60 (m, 2H). **^{13}C NMR (126 MHz, CDCl$_3$)** δ 166.9, 154.4 (t, J = 293.6 Hz), 145.3, 135.6, 130.0, 129.9, 129.7, 128.8, 128.6 (q, J = 32.1 Hz), 125.5 (q, J = 3.6 Hz), 124.4 (q, J = 272.2 Hz), 95.4 (dd, J = 18.1, 12.1 Hz), 68.0 (d, J = 3.6 Hz), 52.4, 36.6, 32.0. **^{19}F NMR (471 MHz, CDCl$_3$)** δ -62.27, -86.75 (d, J = 34.8 Hz), -88.73 (d, J = 34.8 Hz). **HRMS (ESI)** calcd for $C_{20}H_{18}F_5O_3$ $[M+H]^+$: 401.1171, found: 401.1175. **HPLC analysis**: DAICEL CHIRALCEL AD-H hexane/isopropanol = 90/10, 1.0mL/min, λ = 254 nm, t_R (minor) = 9.3 min, t_R (major) = 11.6 min, 91:9 er **$[\alpha]^{25}_D$**: -16.8 (c 0.5, CHCl$_3$).

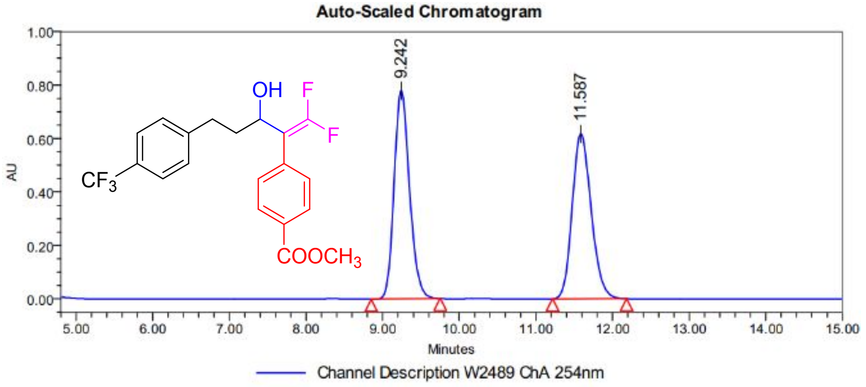

Peak Results

	Retention Time (min)	Int Type	Width (sec)	Area (µV*sec)	Height (µV)	% Area
1	9.242	bb	54.000	10876523	780968	50.54
2	11.587	bb	58.000	10643628	617473	49.46

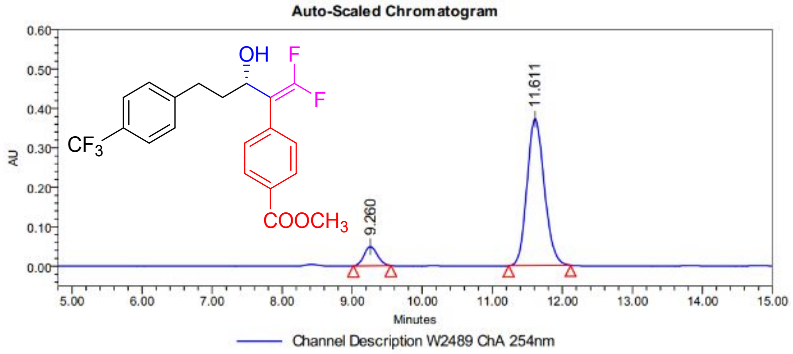

Peak Results

	Retention Time (min)	Int Type	Width (sec)	Area (µV*sec)	Height (µV)	% Area
1	9.260	bb	32.000	634590	48237	9.02
2	11.611	bb	53.000	6398264	372509	90.98

methyl (S)-4-(1,1-difluoro-3-hydroxydodec-1-en-2-yl)benzoate (100)

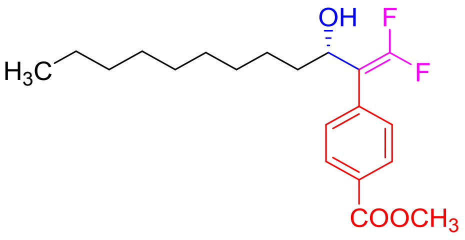

he reaction was performed following the **procedure I**. The residue was purified by flash column chromatograph (Rf = 0.4, PE: Et_2O = 6:1) to give the product as a colorless liquid (54.19 mg, 51% yield). **^{1}H NMR (500 MHz, $CDCl_3$)** δ 8.02 (d, *J* = 8.4 Hz, 2H), 7.47 (d, *J* = 7.7 Hz, 2H), 4.70 (t, *J* = 7.1 Hz, 1H), 3.92 (s, 3H), 1.26 – 1.18 (m, 15H), 0.87 (t, *J* = 7.0 Hz, 4H). **^{13}C NMR (126 MHz, $CDCl_3$)** δ 166.9, 154.4 (t, *J* = 293.6 Hz), 135.9 (d, *J* = 3.0 Hz), 130.1, 129.7, 129.6, 95.5 (dd, *J* = 18.3, 11.4 Hz), 68.9 (d, *J* = 4.1 Hz), 52.3, 35.3, 32.0, 29.6, 29.4, 29.3, 25.7, 22.8, 14.2. **^{19}F NMR (471 MHz, $CDCl_3$)** δ -87.38 (d, *J* = 36.5 Hz), -89.52 (d, *J* = 36.3 Hz). **HRMS (ESI)** calcd for $C_{20}H_{29}F_2O_3$ $[M+H]^+$: 354.2007, found: 355.2083. **HPLC analysis**: DAICEL CHIRALCEL AD-H hexane/isopropanol = 90/10, 1.0mL/min, λ = 254 nm, t_R (minor) = 5.3 min, t_R (major) = 8.2 min, 96:4 er **$[\alpha]^{25}_D$**: -8.6 (*c* 0.5, $CHCl_3$)

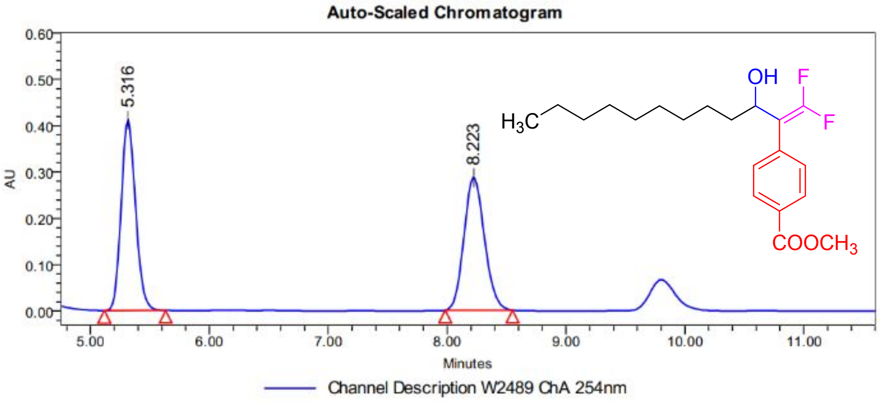

Peak Results

	Retention Time (min)	Int Type	Width (sec)	Area (μV*sec)	Height (μV)	% Area
1	5.316	bb	31.000	3471774	410914	50.20
2	8.223	bb	34.000	3443538	287133	49.80

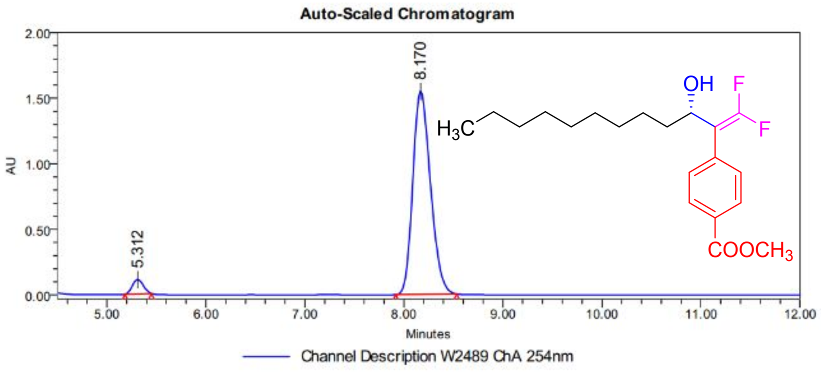

Peak Results

	Retention Time (min)	Int Type	Width (sec)	Area (μV*sec)	Height (μV)	% Area
1	5.312	bb	16.000	837540	109062	4.18
2	8.170	bb	37.000	19210723	1551608	95.82

methyl (S)-4-(11-bromo-1,1-difluoro-3-hydroxyundec-1-en-2-yl)benzoate (101)

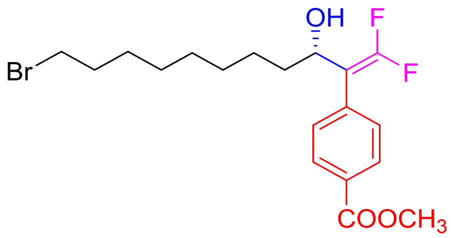

The reaction was performed following the **procedure I**. The residue was purified by flash column chromatograph (Rf = 0.35, PE: Et_2O = 6:1) to give the product as a colorless liquid (74.00 mg, 59% yield).

^{1}H NMR (500 MHz, $CDCl_3$) δ 8.01 (dd, J = 8.4, 1.9 Hz, 2H), 7.46 (d, J = 8.1 Hz, 2H), 4.69 (s, 1H), 3.91 (s, 3H), 3.37 (t, J = 6.8 Hz, 2H), 2.08 – 1.95 (m, 1H), 1.86 – 1.76 (m, 2H), 1.62 – 1.50 (m, 1H), 1.45 – 1.32 (m, 4H), 1.24 (s, 6H). **^{13}C NMR (126 MHz, $CDCl_3$)** δ 167.0, 154.4 (t, J = 292.5 Hz), 135.9 (dd, J = 4.0, 1.8 Hz), 130.1, 129.7, 129.6, 95.5 (dd, J = 18.3, 11.3 Hz), 68.8 (d, J = 3.3 Hz), 52.3, 35.2, 34.0, 32.8, 29.1, 28.6, 28.1, 25.6. **^{19}F NMR (471 MHz, $CDCl_3$)** δ -87.33 (d, J = 35.3 Hz), -89.43 (d, J = 36.7 Hz). **HRMS (ESI)** calcd for $C_{19}H_{26}BrF_2O_3$ $[M+H]^+$: 419.1028, found: 419.1031. **HPLC analysis**: DAICEL CHIRALCEL AD-H hexane/isopropanol = 90/10, 1.0mL/min, λ = 254 nm, t_R (minor) = 10.0 min, t_R (major) = 19.0 min, 95:5 er **$[\alpha]^{25}_D$**: -13.1(c 0.5, $CHCl_3$)

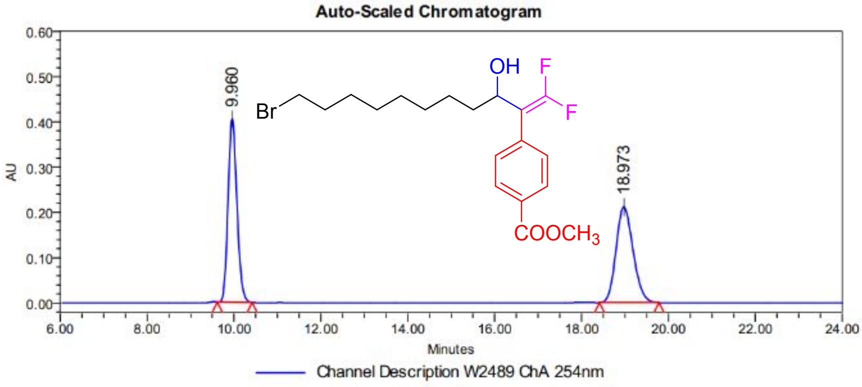

Peak Results

	Retention Time (min)	Int Type	Width (sec)	Area (μV*sec)	Height (μV)	% Area
1	9.960	bb	48.000	5748302	405016	49.87
2	18.973	bb	82.000	5778696	211621	50.13

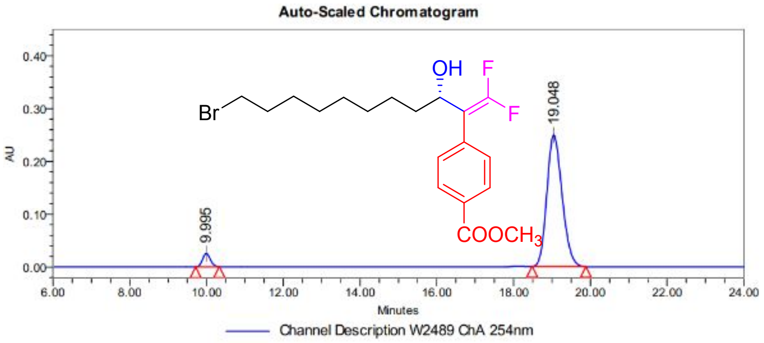

Peak Results

	Retention Time (min)	Int Type	Width (sec)	Area (μV*sec)	Height (μV)	% Area
1	9.995	bb	37.000	357261	25529	4.93
2	19.048	bb	84.000	6888842	249126	95.07

(S)-2-(2-bromo-1,1-difluoro-5-phenylpent-1-en-3-yl)-4,4,5,5-tetramethyl-1,3,2-dioxaborolane (102)

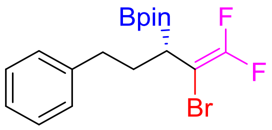

The reaction was performed following the **procedure J**. The residue was purified by flash column

chromatograph (Rf = 0.65, PE: Et_2O = 10:1) to give the product as a colorless liquid (48.65 mg, 63% yield).

^{1}H NMR (500 MHz, $CDCl_3$) δ 7.28 (t, J = 7.6 Hz, 2H), 7.21 – 7.17 (m, 3H), 2.71 – 2.65 (m, 1H), 2.54 – 2.48 (m, 1H), 2.37 – 2.30 (m, 1H), 2.04 – 1.97 (m, 1H), 1.94 – 1.86 (m, 1H), 1.26 (d, J = 1.6 Hz, 12H). **^{13}C NMR (126 MHz, $CDCl_3$)** δ 153.4 (dd, J = 285.8, 283.4 Hz), 141.9, 128.7, 128.5, 126.0, 84.2, 82.7 (dd, J = 41.1, 20.2 Hz), 34.6, 30.5, 25.0, 24.7. **^{19}F NMR (471 MHz, $CDCl_3$)** δ -83.98 (d, J = 45.8 Hz), -90.29 (d, J = 45.7 Hz). **^{11}B NMR (128 MHz, $CDCl_3$)** δ 32.72. **HRMS (ESI)** calcd for $C_{17}H_{23}BBrF_2O_2$ $[M+H]^+$: 387.0937, found: 387.0942. **HPLC analysis**: **102** was determined by corresponding alcohol. DAICEL CHIRALCEL AD-H hexane/isopropanol = 95/5, 0.5/min, λ = 220 nm, t_R (minor) = 22.9 min, t_R (major) = 32.2min, 95.5:4.55 er **[α]$^{25}_D$**: -0.8 (c 0.5, $CHCl_3$)

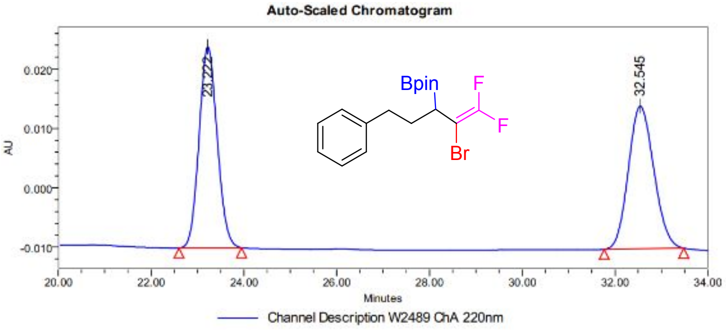

Peak Results

	Retention Time (min)	Int Type	Width (sec)	Area (μV*sec)	Height (μV)	% Area
1	23.222	BB	81.000	916832	33872	50.00
2	32.545	BB	103.000	916660	24042	50.00

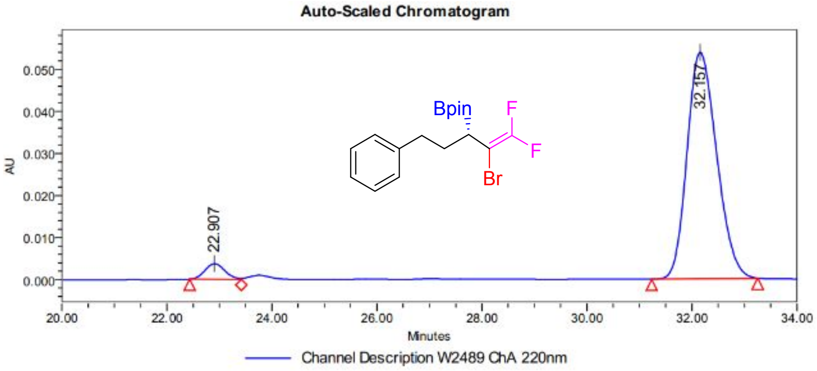

Peak Results

	Retention Time (min)	Int Type	Width (sec)	Area (μV*sec)	Height (μV)	% Area
1	22.907	BV	59.000	98391	3721	4.45
2	32.157	BB	121.000	2112404	53818	95.55

(S)-2-(1,1-difluoro-2-methyl-5-phenylpent-1-en-3-yl)-4,4,5,5-tetramethyl-1,3,2-dioxaborolane (103)

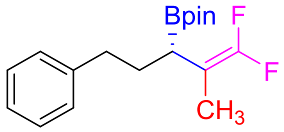

The reaction was performed following the **procedure K**. The residue was purified by flash column chromatograph (Rf = 0.65, PE: Et_2O = 10:1) to give the product as a colorless liquid (36.09 mg, 56% yield).

^{1}H NMR (500 MHz, CDCl$_3$) δ 7.32 – 7.20 (m, 2H), 7.17 (d, J = 7.3 Hz, 3H), 2.65 – 2.57 (m, 1H), 2.56 – 2.46 (m, 1H), 2.05 – 1.83 (m, 2H), 1.75 – 1.65 (m, 1H), 1.58 (t, J = 3.2 Hz, 3H), 1.23 (d, J = 2.0 Hz, 12H). **^{13}C NMR (151 MHz, CDCl$_3$)** δ 153.1 (dd, J = 280.3, 282.5 Hz), 142.6, 128.6, 128.4, 125.8, 1.58, 85.2 (t, J = 15.1 Hz), 83.6, 35.3, 30.3, 25.0, 24.8, 11.0. **^{19}F NMRNMR (471 MHz, CDCl$_3$)** δ -95.95 (q, J = 58.9 Hz). **^{11}B NMR (128 MHz, CDCl$_3$)** δ 33.07. **HRMS (ESI)** calcd for $C_{18}H_{26}BF_2O_2$ [M+H]$^+$: 323.1988, found: 323.1990. **HPLC analysis**: **103** was determined by corresponding alcohol. DAICEL CHIRALCEL AD-H hexane/isopropanol = 95/5, 0.5/min, λ = 220 nm, t_R (minor) = 22.2 min, t_R (major) = 24.5min, 95:5 er **[α]^{25}$_D$**: 2.0 (c 0.5, CHCl3)

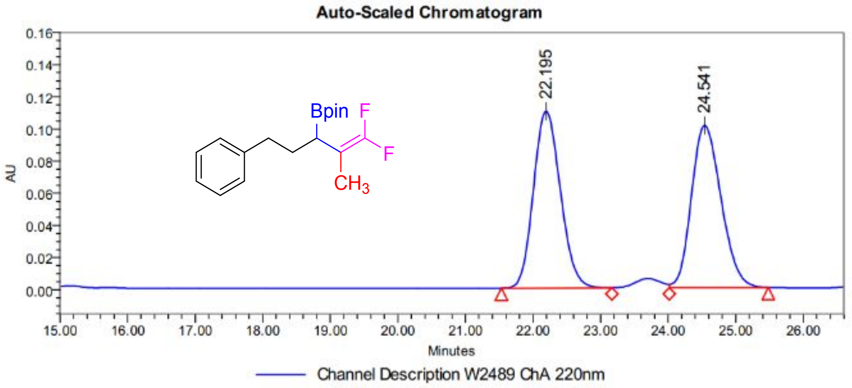

Peak Results

	Retention Time (min)	Int Type	Width (sec)	Area (μV*sec)	Height (μV)	% Area
1	22.195	BV	98.000	3050975	109986	49.84
2	24.541	VB	88.000	3070333	100976	50.16

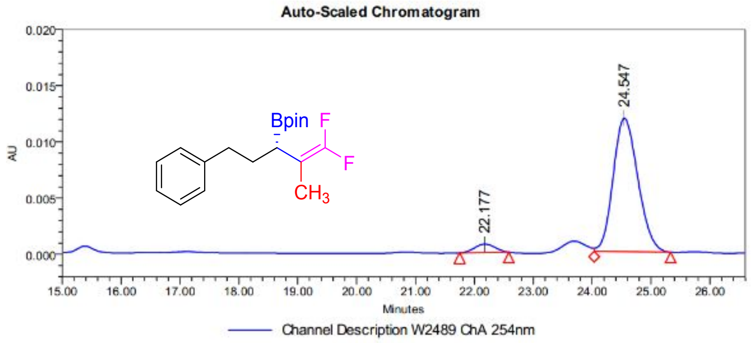

Peak Results

	Retention Time (min)	Int Type	Width (sec)	Area (μV*sec)	Height (μV)	% Area
1	22.177	bb	50.000	18089	755	4.95
2	24.547	Vb	78.000	347443	11896	95.05

(S)-2-(1,1-difluoro-5-phenyl-2-(thiophen-2-yl)pent-1-en-3-yl)-4,4,5,5-tetramethyl-1,3,2-dioxaborolane (104)

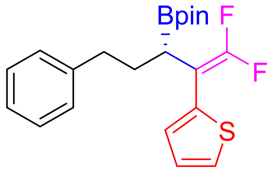

The reaction was performed following the **procedure L**. The residue was purified by flash column chromatograph (Rf = 0.65, PE: Et$_2$O = 10:1) to give the product as a colorless liquid (47.60 mg, 61%

yield).

^{1}H NMR (500 MHz, CDCl$_3$) δ 7.27 – 7.22 (m, 3H), 7.18 – 7.12 (m, 3H), 7.03 (dd, J = 1.1, 3.6 Hz, 1H), 6.99 – 6.96 (m, 1H), 2.72 – 2.57 (m, 2H), 2.24 (dd, J = 6.6, 8.6 Hz, 1H), 2.12 – 2.03 (m, 1H), 1.93 – 1.83 (m, 1H), 1.22 (d, J = 10.6 Hz, 12H). **^{13}C NMR (126 MHz, CDCl$_3$)** δ 154.1 (dd, J = 287.2, 294.5 Hz), 142.4, 136.1 (t, J = 5.4 Hz), 128.7, 128.4, 126.8, 126.3 (t, J = 4.2 Hz), 125.8, 125.0 (dd, J = 2.1, 5.1 Hz), 89.2 (dd, J = 12.5, 26.3 Hz), 83.9, 35.3, 31.1, 25.0, 24.8. **^{19}F NMRNMR (471 MHz, CDCl$_3$)** δ -84.91 (d, J = 34.9 Hz), -89.26 (d, J = 34.9 Hz). **^{11}B NMR (128 MHz, CDCl$_3$)** δ 33.17. **HRMS (ESI)** calcd for $C_{21}H_{26}BF_2O_2S$ [M+H]$^+$: 391.1709, found: 391.1706. **HPLC analysis**: DAICEL CHIRALCEL OZ-3 hexane/isopropanol = 99.9/0.1, 0.5/min, λ = 210 nm, t_R (major) = 9.6 min, t_R (minor) = 10.5min, 95.5:4.5 er **[α]^{25}$_D$**: -3.6. (c 0.5, CHCl3)

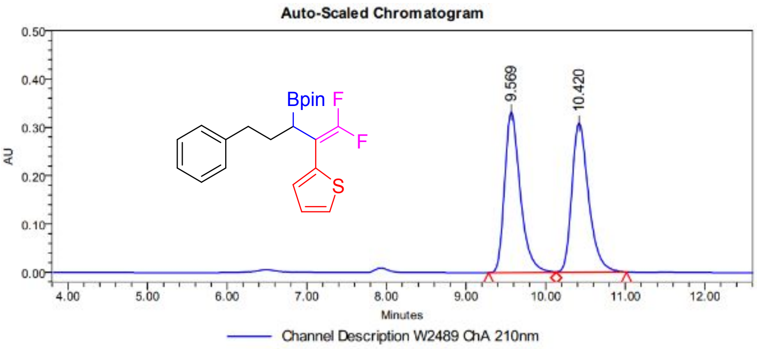

Peak Results

	Retention Time (min)	Int Type	Width (sec)	Area (µV*sec)	Height (µV)	% Area
1	9.569	bV	51.000	4544605	332875	50.13
2	10.420	Vb	53.000	4520157	308777	49.87

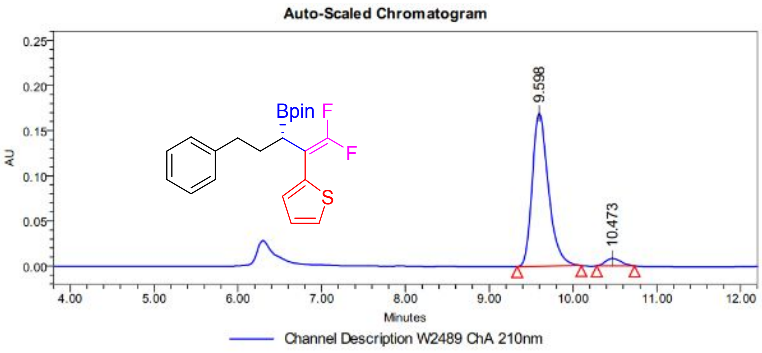

Peak Results

	Retention Time (min)	Int Type	Width (sec)	Area (µV*sec)	Height (µV)	% Area
1	9.598	bb	46.000	2283958	169383	95.57
2	10.473	bb	27.000	105984	8300	4.43

(S,E)-2-(4-(difluoromethylene)-1,6-diphenylhex-5-en-3-yl)-4,4,5,5-tetramethyl-1,3,2-dioxaborolane (105-E)

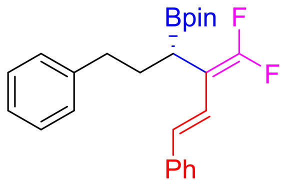

The reaction was performed following the **procedure M**. The residue was purified by flash column

chromatograph (Rf = 0.65, PE: Et_2O = 10:1) to give the product as a colorless liquid (61.53 mg, 75% yield).

^{1}H NMR (500 MHz, CDCl$_3$) δ 7.36 – 7.14 (m, 12H), 6.79 (d, J = 16.4 Hz, 1H), 6.44 (d, J = 16.6 Hz, 1H), 2.71 – 2.59 (m, 2H), 2.16 – 2.09 (m, 2H), 1.93 – 1.84 (m, 1H), 1.25 (d, J = 14.9 Hz, 12H). **^{13}C NMR (126 MHz, CDCl$_3$)** δ 155.4 (dd, J = 290.7, 297.4 Hz), 142.4, 137.4, 128.7 (d, J = 2.4 Hz), 128.4, 127.5, 126.3, 125.9, 120.7 (d, J = 2.4 Hz), 93.5 (dd, J = 10.8, 21.1 Hz), 83.9, 35.3, 31.1, 25.1, 24.8. **^{19}F NMR (471 MHz, CDCl$_3$)** δ -85.37 – -91.18 (m). **^{11}B NMR (128 MHz, CDCl$_3$)** δ 34.28. **HRMS (ESI)** calcd for $C_{25}H_{30}BF_2O_2$ [M+H]$^+$: 410.2229, found: 410.2309. **HPLC analysis**: DAICEL CHIRALCEL OZ-3 hexane/isopropanol = 99.9/0.1, 0.5/min, λ = 254 nm, t_R (major) = 9.8 min, t_R (minor) = 11.8min, 96.5:3.5 er **[α]$^{25}_D$**: -5.5 (c 0.5, CHCl3)

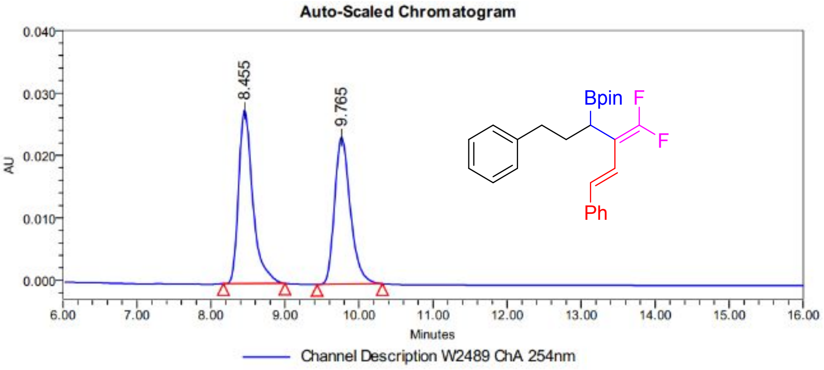

Peak Results

	Retention Time (min)	Int Type	Width (sec)	Area (μV*sec)	Height (μV)	% Area
1	8.455	Bb	50.000	370529	27781	51.46
2	9.765	BB	53.000	349560	23521	48.54

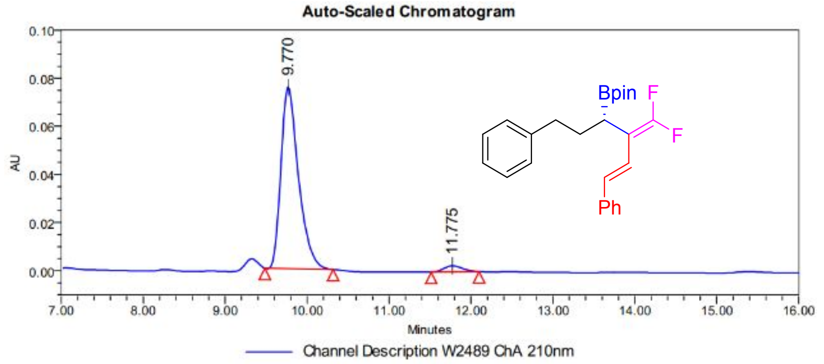

Peak Results

	Retention Time (min)	Int Type	Width (sec)	Area (μV*sec)	Height (μV)	% Area
1	9.770	bb	50.000	1123841	75593	96.59
2	11.775	bb	35.000	39658	2499	3.41

(S)-2-(2-benzyl-1,1-difluoro-5-phenylpent-1-en-3-yl)-4,4,5,5-tetramethyl-1,3,2-dioxaborolane(106)

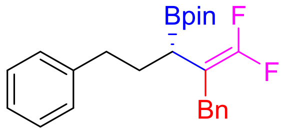

The reaction was performed following the **procedure N**. The residue was purified by flash column chromatograph (Rf = 0.6, PE: Et_2O = 10:1) to give the product as a colorless liquid (57.34 mg, 72% yield).

^{1}H NMR (500 MHz, CDCl$_3$) δ 7.29 – 7.17 (m, 7H), 7.12 (t, *J* = 7.3 Hz, 1H), 6.96 (d, *J* = 7.8 Hz, 2H), 3.43 – 3.27 (m, 2H), 2.50 – 2.34 (m, 2H), 1.86 – 1.76 (m, 2H), 1.64 – 1.55 (m, 1H), 1.15 (s, 12H). **^{13}C NMR (126 MHz, CDCl$_3$)** δ 154.4 (t, *J* = 284.3 Hz), 142.5, 139.2, 129.1, 128.5, 128.5, 128.3, 126.5, 125.7, 89.6 (t, *J* = 16.6 Hz), 83.5, 35.4, 33.7, 33.7, 31.1, 24.9, 24.8. **^{19}FNMR (471 MHz, CDCl$_3$)** δ -93.49 (d, *J* = 55.0 Hz), -93.75 (d, *J* = 53.2 Hz). **^{11}B NMR (128 MHz, CDCl$_3$)** δ 32.86. **HRMS (ESI)** calcd for $C_{24}H_{30}BF_2O_2$ $[M+H]^+$: 399.2301, found: 399.2302. **HPLC analysis**: DAICEL CHIRALCEL OZ-3 hexane/isopropanol = 99.9/0.1, 0.5/min, λ = 210 nm, t_R (major) = 9.5 min, t_R (minor) = 10.3min, 96:4 er **[α]$^{25}_{D}$**: -0.6 (*c* 0.5, CHCl3)

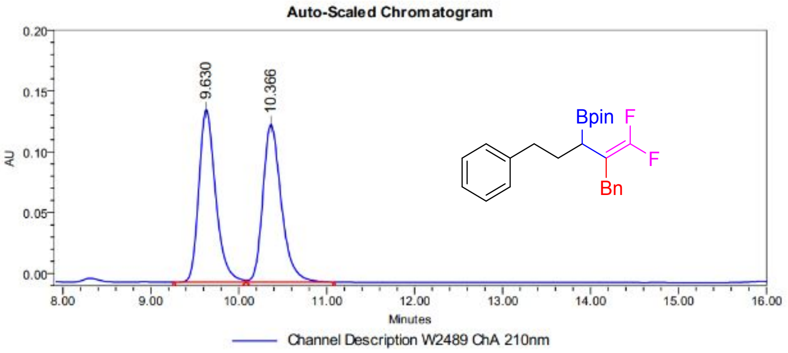

Peak Results

	Retention Time (min)	Int Type	Width (sec)	Area (µV*sec)	Height (µV)	% Area
1	9.630	BV	49.000	1876277	141596	49.88
2	10.366	VB	60.000	1885675	129532	50.12

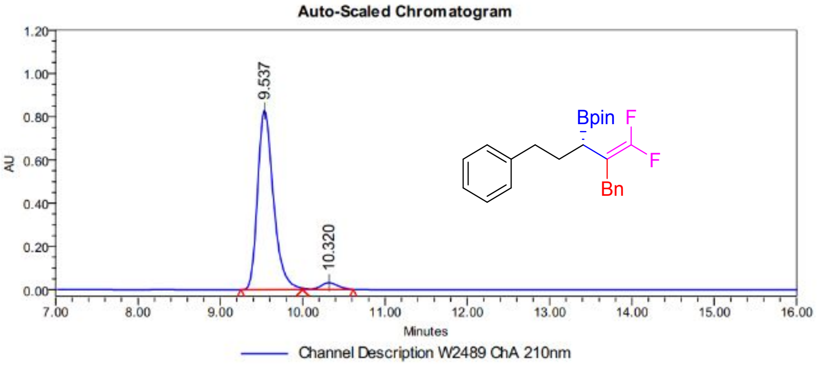

Peak Results

	Retention Time (min)	Int Type	Width (sec)	Area (µV*sec)	Height (µV)	% Area
1	9.537	bv	45.000	11043663	828986	96.14
2	10.320	vb	37.000	443190	30572	3.86

(S)-4-(1,1-difluoro-5-phenyl-3-(4,4,5,5-tetramethyl-1,3,2-dioxaborolan-2-yl)pent-1-en-2-yl)benzoate (107)

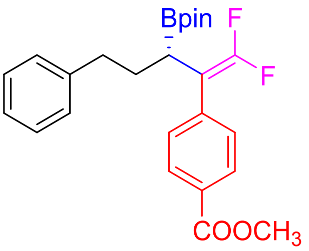

The reaction was performed following the **procedure O**. The residue was purified by flash column chromatograph (Rf = 0.4, PE: Et_2O = 10:1) to give the product as a colorless liquid (62.79 mg, 71% yield).

^{1}H NMR (500 MHz, $CDCl_3$) δ 7.99 (s, 2H), 7.41 (d, *J* = 7.2 Hz, 2H), 7.23 (t, *J* = 7.4 Hz, 2H), 7.15 (t, *J* = 7.3 Hz, 1H), 7.07 (d, *J* = 7.1 Hz, 2H), 3.91 (s, 3H), 2.70 – 2.53 (m, 2H), 2.23 – 2.14 (m, 1H), 2.06 – 1.96 (m, 1H), 1.80 – 1.68 (m, 1H), 1.24 – 1.20 (m, 12H). **^{13}C NMR (126 MHz, $CDCl_3$)** δ 167.0, 153.9 (dd, *J* = 292.0, 288.6 Hz), 142.1, 139.7 (dd, *J* = 5.4, 3.1 Hz), 129.6, 129.0, 128.9, 128.5, 128.4, 125.9, 93.3 (dd, *J* = 22.3, 12.9 Hz), 83.9, 52.2, 35.3, 31.1, 24.9, 24.8. **^{19}F NMR (471 MHz, $CDCl_3$)** δ -88.37 (d, *J* = 40.0 Hz), -89.10 (d, *J* = 40.2 Hz). **^{11}B NMR (128 MHz, $CDCl_3$)** δ 33.75. **HRMS (ESI)** calcd for $C_{25}H_{30}BF_2O_4$ $[M+H]^+$: 443.2200, found: 443.2197. **HPLC analysis**: DAICEL CHIRALCEL OD-H hexane/isopropanol = 99.5/0.5, 0.5/min, λ = 210 nm, t_R (major) = 18.0 min, t_R (minor) = 24.5min, 96:4 er **$[\alpha]^{25}_D$**: -8.2 (*c* 0.5, $CHCl_3$)

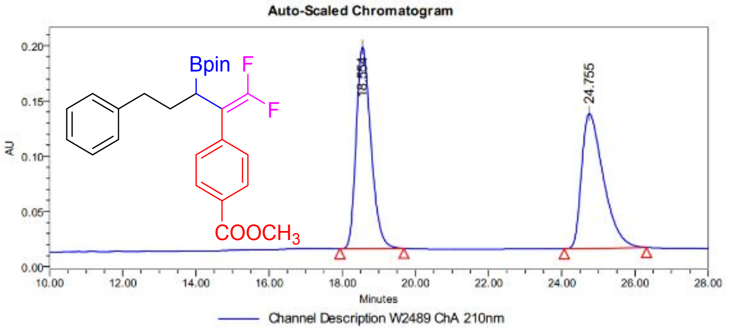

Peak Results

	Retention Time (min)	Int Type	Width (sec)	Area (µV*sec)	Height (µV)	% Area
1	18.554	BB	105.000	5111387	182197	50.22
2	24.755	BB	135.000	5066116	122013	49.78

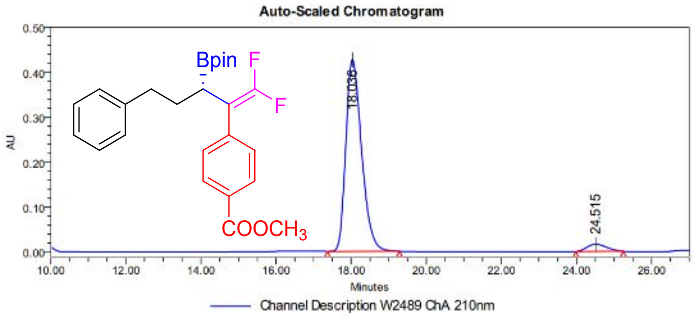

Peak Results

	Retention Time (min)	Int Type	Width (sec)	Area (µV*sec)	Height (µV)	% Area
1	18.036	bb	115.000	12437742	426577	95.47
2	24.515	bb	76.000	590484	16107	4.53

methyl(S)-4-(1,1-difluoro-5-phenyl-3-(trifluoro-l4-boraneyl)pent-1-en-2-yl)benzoate,potassium salt (108)

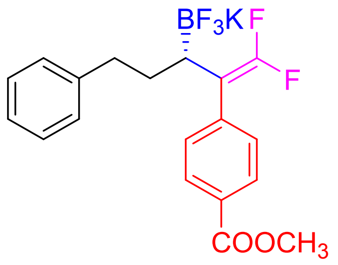

The reaction was performed following the **procedure P**.
^{1}H NMR (500 MHz, Acetone-d_6) δ 7.95 (dd, J = 14.9, 7.8 Hz, 2H), 7.67 (d, J = 6.6 Hz, 2H), 7.16 (d, J = 6.1 Hz, 2H), 7.11 – 6.99 (m, 3H), 3.87 (s, 3H), 2.62 (d, J = 13.3 Hz, 1H), 2.44 (dd, J = 10.7, 6.2 Hz, 1H), 1.76 (d, J = 47.7 Hz, 2H), 1.63 – 1.48 (m, 1H). **^{13}C NMR (126 MHz, Acetone-d_6)** δ 167.3, 153.7 (t, J = 283.5 Hz), 144.8, 142.8 (d, J = 6.2 Hz), 131.0, 129.3, 129.1, 128.8, 128.8, 125.9, 98.2 (dd, J = 25.1, 12.6 Hz), 52.1, 37.1, 31.6. **^{19}F NMR (471 MHz, Acetone-d_6)** δ -94.56 (d, J = 54.4 Hz), -95.10 (d, J = 54.2 Hz), -142.26. **^{11}B NMR (128 MHz, Acetone-d_6)** δ 4.14.

(S)-2-(3-argio-4,4-difluoro-2-phenethylbut-3-en-1-yl)-4,4,5,5-tetramethyl-1,3,2-dioxaborolane (109)

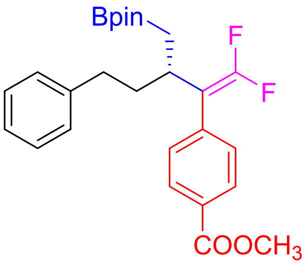

The reaction was performed following the **procedure Q**. The residue was purified by flash column chromatograph (R_f = 0.4, PE: Et_2O = 10:1) to give the product as a colorless liquid (29.65 mg, 65% yield).
^{1}H NMR (500 MHz, $CDCl_3$) δ 8.02 (d, J = 8.4 Hz, 2H), 7.38 (d, J = 8.1 Hz, 2H), 7.28 – 7.24 (m, 2H), 7.17 (t, J = 7.4 Hz, 1H), 7.12 (d, J = 7.3 Hz, 2H), 3.92 (s, 3H), 2.94 – 2.86 (m, 1H), 2.75 – 2.66 (m, 1H), 2.61 (dt, J = 14.4, 8.4 Hz, 1H), 1.71 – 1.66 (m, 2H), 1.23 (s, 12H), 1.01 (d, J = 8.2 Hz, 2H). **^{13}C NMR (126 MHz, $CDCl_3$)** δ 166.9, 153.6 (t, J = 289.8 Hz), 142.1, 138.1 (d, J = 4.9 Hz), 130.2, 129.6, 129.4, 128.5, 128.4, 125.9, 96.0 (dd, J = 20.2, 12.6 Hz), 83.4, 52.3, 37.7, 34.9, 34.2, 25.0, 24.9. **^{19}F NMR (471 MHz, $CDCl_3$)** δ -88.54 (d, J = 42.1 Hz), -89.68 (d, J = 42.1 Hz). **^{11}B NMR (128 MHz, $CDCl_3$)** δ 34.02 **HRMS (ESI)** calcd for $C_{26}H_{32}BF_2O_4$ $[M+H]^+$: 457.2356, found: 457.2359. **HPLC analysis**: DAICEL CHIRALCEL ID-3 hexane/isopropanol = 99.8/0.2, 0.5/min, λ = 254 nm, t_R (minor) = 51.8 min, t_R (major) = 54.9 min, 96:4 er **$[\alpha]^{25}_D$**: -10.8 (c 0.5, $CHCl_3$).

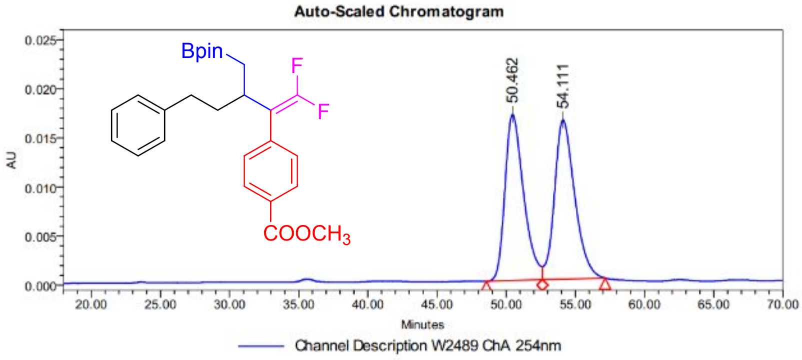

Peak Results

	Retention Time (min)	Int Type	Width (sec)	Area (µV*sec)	Height (µV)	% Area
1	50.462	bV	244.000	1589439	16904	49.13
2	54.111	Vb	271.000	1645804	16207	50.87

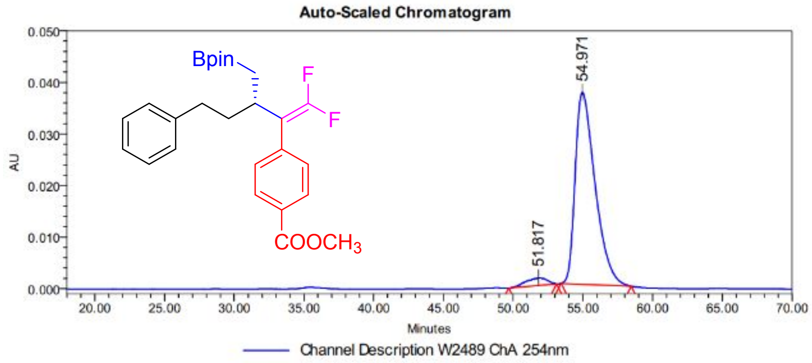

Peak Results

	Retention Time (min)	Int Type	Width (sec)	Area (μV*sec)	Height (μV)	% Area
1	51.817	bb	206.000	154993	1448	4.02
2	54.971	bb	302.000	3696637	37217	95.98

methyl4-((3S)-1,1-difluoro-5-phenyl-3-(4,4,5,5-tetramethyl-1,3,2-dioxaborolan-2-yl)pentan-2-yl)benzoate (110)

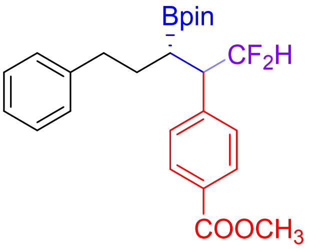

The reaction was performed following the **procedure R**. The residue was purified by flash column chromatograph (Rf = 0.4, PE: Et_2O = 10:1) to give the product as a colorless liquid (41.31 mg, 93% yield).

^{1}H NMR (500 MHz, $CDCl_3$) δ 7.97 (d, J = 8.3 Hz, 2H), 7.38 (d, J = 8.3 Hz, 1H), 7.31 – 7.11 (m, 6H), 6.97 (d, J = 6.7 Hz, 1H), 3.90 (s, 3H), 3.36 – 3.15 (m, 1H), 2.73 – 2.51 (m, 1H), 2.50 – 2.33 (m, 1H), 1.91 – 1.79 (m, 1H), 1.68 – 1.52 (m, 2H), 1.33 -1.0 (m, 12H). **^{13}C NMR (126 MHz, $CDCl_3$)** δ 167.0, 142.3 (t, J = 3.8 Hz), 141.9, 129.9, 129.4, 128.4, 126.0, 117.8 (t, J = 244.1 Hz), 83.9, 52.2, 51.4 (t, J = 19.2 Hz), 34.8, 31.1, 25.2, 25.1. **^{19}F NMR (471 MHz, $CDCl_3$)** δ -116.82 – -120.32 (m). **^{11}B NMR (128 MHz, $CDCl_3$)** δ 34.85. **HRMS (ESI)** calcd for $C_{25}H_{32}BF_2O_4$ $[M+H]^+$: 445.2356, found: 445.2359. **HPLC analysis**: DAICEL CHIRALCEL OD-H hexane/isopropanol = 99.5/0.5, 0.5/min, λ = 254 nm, tR (major) = 20.4 min, tR (minor) = 24.9min, 96.5:3.5 er, 2.7:1dr. **[α]$^{25}_{D}$**: 14.8 (c 0.5, CHCl3)

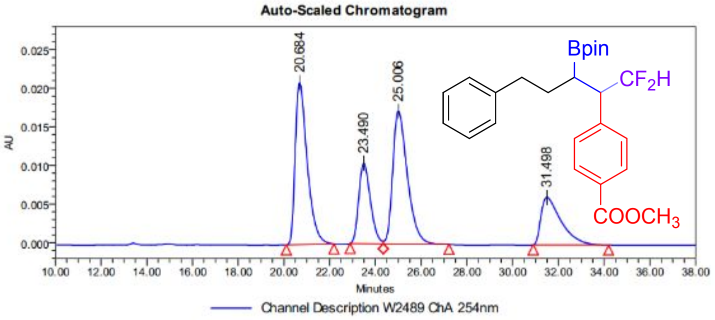

Peak Results

	Retention Time (min)	Int Type	Width (sec)	Area (µV*sec)	Height (µV)	% Area
1	20.684	Bb	125.000	764650	20979	33.60
2	23.490	BV	87.000	369128	10414	16.22
3	25.006	Vb	172.000	766113	17197	33.67
4	31.498	Bb	198.000	375787	6147	16.51

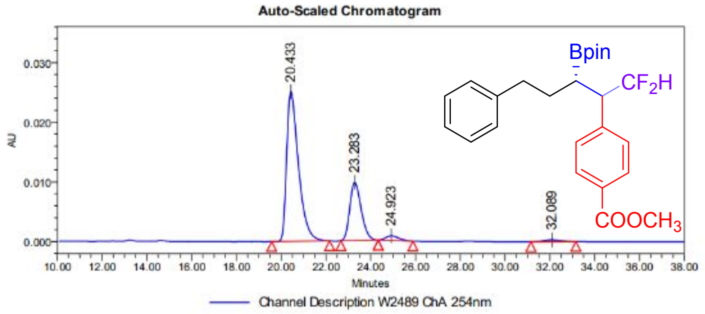

Peak Results

	Retention Time (min)	Int Type	Width (sec)	Area (µV*sec)	Height (µV)	% Area
1	20.433	bb	156.000	921225	25086	70.08
2	23.283	Bb	100.000	346322	9746	26.35
3	24.923	bb	93.000	32354	799	2.46
4	32.089	bb	121.000	14635	270	1.11

methyl (S)-4-(1,1-difluoro-3-(furan-2-yl)-5-phenylpent-1-en-2-yl)benzoate (111)

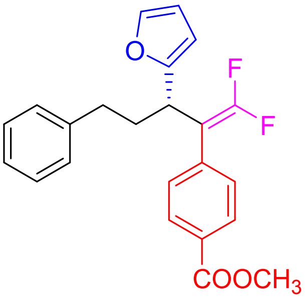

The reaction was performed following the **procedure S**. The residue was purified by flash column chromatograph (R_f = 0.45, PE: Et_2O = 10:1) to give the product as a colorless liquid (27.51 mg, 72% yield).

^{1}H NMR (500 MHz, CDCl$_3$) δ 7.95 (d, *J* = 7.6 Hz, 2H), 7.38 (s, 1H), 7.30 – 7.22 (m, 2H), 7.18 (t, *J* = 7.3 Hz, 1H), 7.10 (d, *J* = 7.1 Hz, 2H), 7.05 (d, *J* = 8.0 Hz, 2H), 6.31 (s, 1H), 6.03 (s, 1H), 3.97 (t, *J* = 7.3 Hz, 1H), 3.90 (s, 3H), 2.77 – 2.57 (m, 2H), 2.17 – 2.10 (m, 1H), 1.94 – 1.80 (m, 1H). **^{13}C NMR (126 MHz, CDCl$_3$)** δ 166.9, 154.9, 154.5 (t, *J* = 290.6 Hz), 141.7, 141.3, 136.9 (d, *J* = 3.2 Hz), 129.7, 129.6, 129.5, 128.6, 128.5, 126.3, 110.5, 106.6, 93.3 (dd, *J* = 19.9, 14.6 Hz), 52.3, 37.5, 33.6, 32.1. **^{19}F NMR**

(471 MHz, CDCl3) δ -87.65 (d, *J* = 37.7 Hz), -90.04 (d, *J* = 37.0 Hz) **HRMS (ESI)** calcd for $C_{23}H_{21}F_2O_3$ $[M+H]^+$: 383.1453, found: 383.1456. **HPLC analysis**: DAICEL CHIRALCEL ID-3 hexane/isopropanol = 99.5/0.5, 0.5/min, λ = 254 nm, t_R (minor) = 17.3 min, t_R (major r) = 18.4 min, 96:4 er **$[\alpha]^{25}_D$**: -2.6 (*c* 0.5, CHCl3)

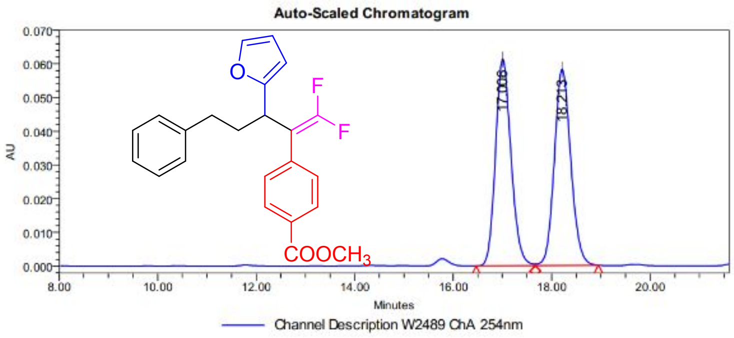

Peak Results

	Retention Time (min)	Int Type	Width (sec)	Area (μV*sec)	Height (μV)	% Area
1	17.006	BV	72.000	1341591	61352	49.83
2	18.213	VB	77.000	1350634	58192	50.17

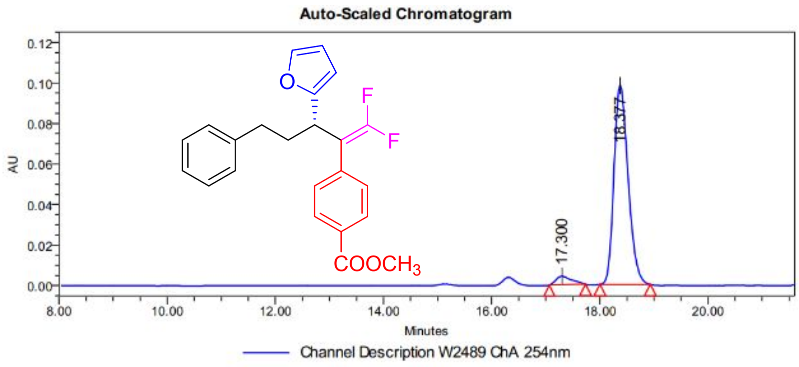

Peak Results

	Retention Time (min)	Int Type	Width (sec)	Area (μV*sec)	Height (μV)	% Area
1	17.300	bb	40.000	84944	4229	4.56
2	18.377	bb	56.000	1779162	98204	95.44

methyl (S)-4-(1,1-difluoro-3-phenethylpenta-1,4-dien-2-yl)benzoate (112)

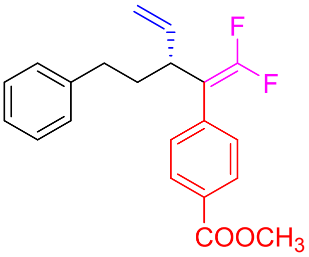

The reaction was performed following the **procedure T**. The residue was purified by flash column chromatograph (Rf = 0.5, PE: Et_2O = 10:1) to give the product as a colorless liquid (24.29 mg, 71% yield).

^{1}H NMR (500 MHz, CDCl$_3$) δ 8.01 (d, *J* = 8.4 Hz, 2H), 7.36 – 7.32 (m, 2H), 7.26 (t, *J* = 7.3 Hz, 2H), 7.18 (t, *J* = 7.5 Hz, 1H), 7.10 (d, *J* = 7.1 Hz, 2H), 5.91 – 5.78 (m, 1H), 5.20 – 5.11 (m, 2H), 3.93 (s, 3H), 3.24 (q, *J* = 7.3 Hz, 1H), 2.62 (t, *J* = 8.0 Hz, 2H), 1.88 – 1.78 (m, 2H). **^{13}C NMR (126 MHz, CDCl$_3$)** δ

166.9, 153.9 (t, J = 291.1 Hz), 141.7, 138.9, 138.0 (d, J = 3.2 Hz), 129.6, 129.5, 128.5, 128.5, 126.1, 116.1, 94.5 (dd, J = 20.3, 13.1 Hz), 52.3, 42.7, 34.1, 33.8. **^{19}F NMR (471 MHz, $CDCl_3$)** δ -88.34 (d, J = 40.3 Hz), -89.36 (d, J = 40.2 Hz). **HRMS (ESI)** calcd for $C_{21}H_{21}F_2O_2$ $[M+H]^+$: 343.1504, found: 343.1507. **HPLC analysis**: DAICEL CHIRALCEL IB N-3 hexane/isopropanol = 99.9/0.1, 0.5mL/min, λ = 254 nm, t_R (major) = 18.4 min, t_R (minor) = 21.8 min, 95:5 er **$[\alpha]^{25}_D$**: -7.8 (c 0.5, $CHCl_3$).

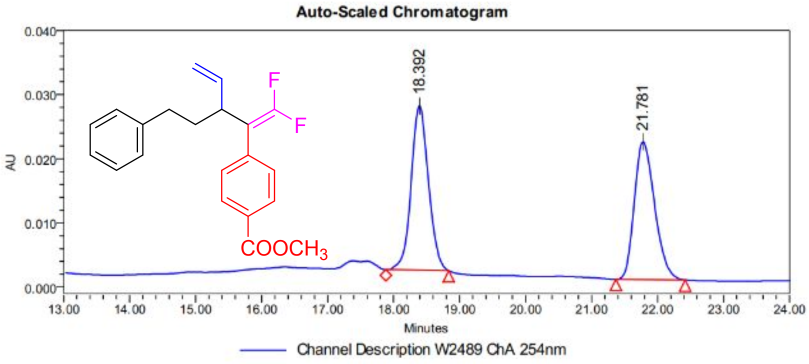

Peak Results

	Retention Time (min)	Int Type	Width (sec)	Area (µV*sec)	Height (µV)	% Area
1	18.392	Vb	57.000	466936	25612	50.44
2	21.781	BB	63.000	458879	21472	49.56

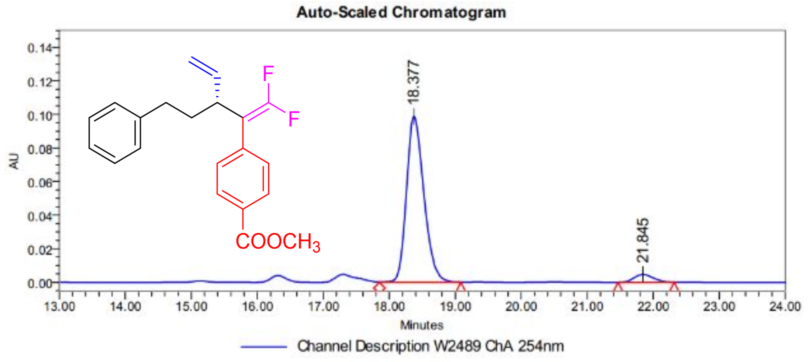

Peak Results

	Retention Time (min)	Int Type	Width (sec)	Area (µV*sec)	Height (µV)	% Area
1	18.377	VB	74.000	1814334	98753	94.96
2	21.845	BB	51.000	96358	4682	5.04

(E)-4,4,5,5-tetramethyl-2-(1,1,1-trifluoro-5-phenylpent-2-en-2-yl)-1,3,2-dioxaborolane (113)

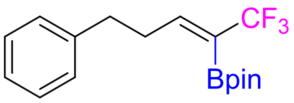

The reaction was performed following the **procedure U**. The residue was purified by flash column chromatograph (Rf= 0.5, PE: Et_2O = 10:1) to give the product as a white solid (66.8 mg, 41% yield). **^{1}H NMR (500 MHz, $CDCl_3$)** δ 7.33 – 7.28 (m, 2H), 7.24 – 7.18 (m, 3H), 6.95 (t, J = 6.2 Hz, 1H), 2.83 – 2.72 (m, 4H), 1.30 (s, 12H). **^{13}C NMR (126 MHz, $CDCl_3$)** δ 153.50 (q, J = 7.6 Hz), 141.10, 128.57, 126.25, 124.08, 84.11, 35.33, 32.47, 24.78. **^{19}F NMR (471 MHz, $CDCl_3$)** δ -64.92. **^{11}B NMR (128 MHz, $CDCl_3$)** δ 28.98. **HRMS (ESI)** calcd for $C_{17}H_{23}BF_3O_2$ $[M+H]^+$: 327.1738, found: 327.1747.

10. NMR Spectroscopic Data

(4-chloro-5,5,5-trifluoropent-3-en-1-yl)benzene (1)

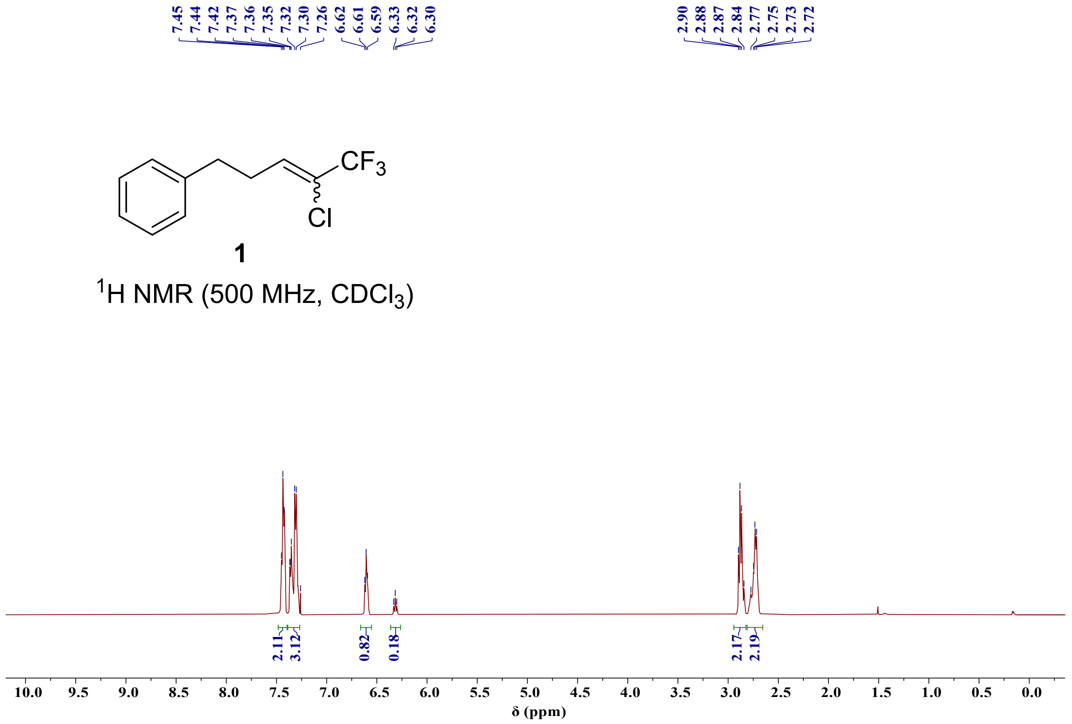

(4-chloro-5,5,5-trifluoropent-3-en-1-yl)benzene (1)

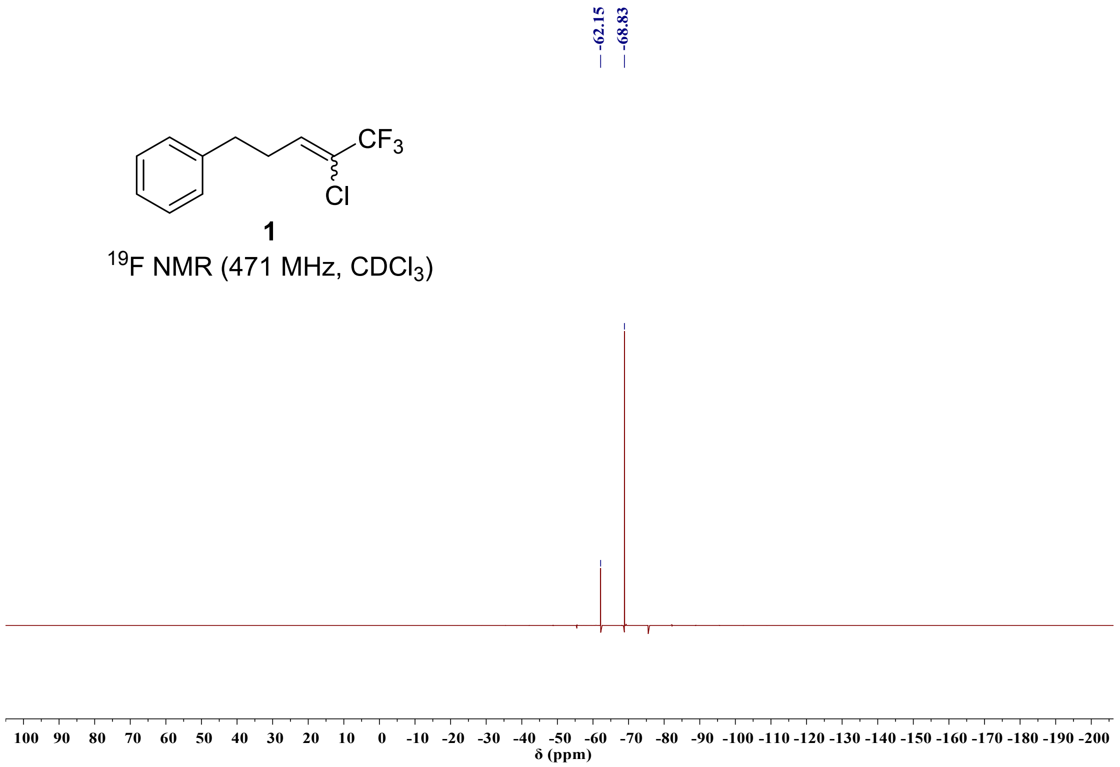

(4-chloro-5,5,5-trifluoropent-3-en-1-yl)benzene (1)

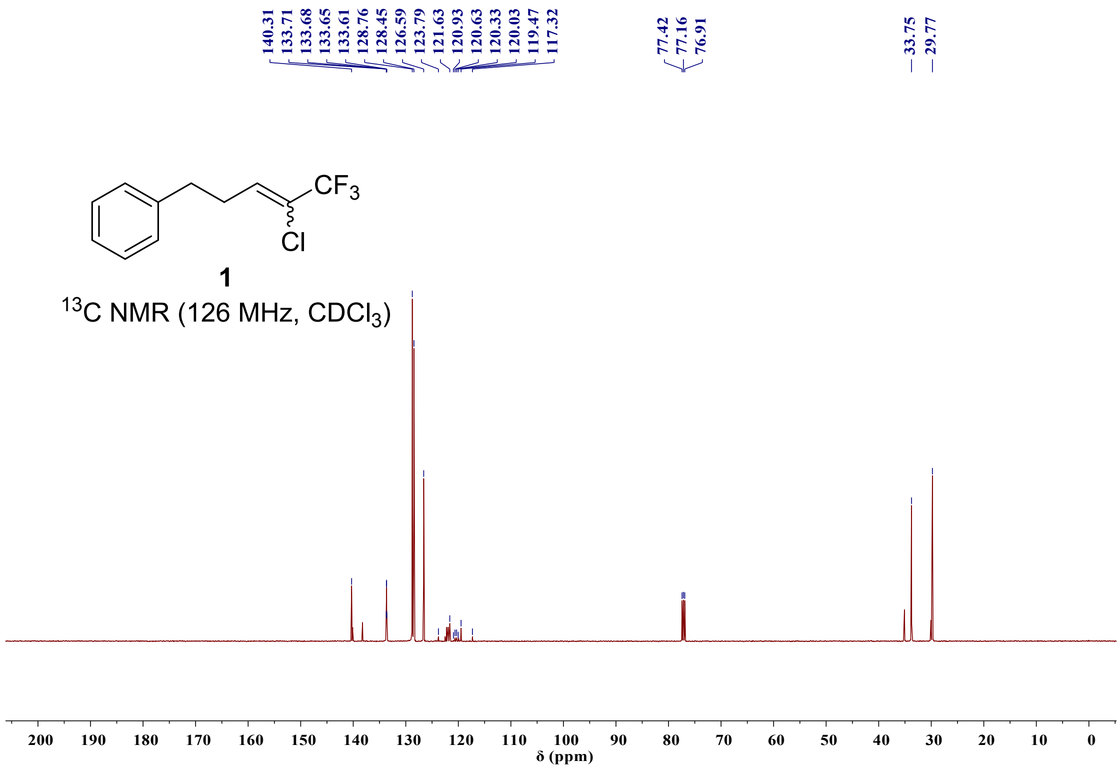

1-(4-chloro-5,5,5-trifluoropent-3-en-1-yl)-4-methylbenzene (1b)

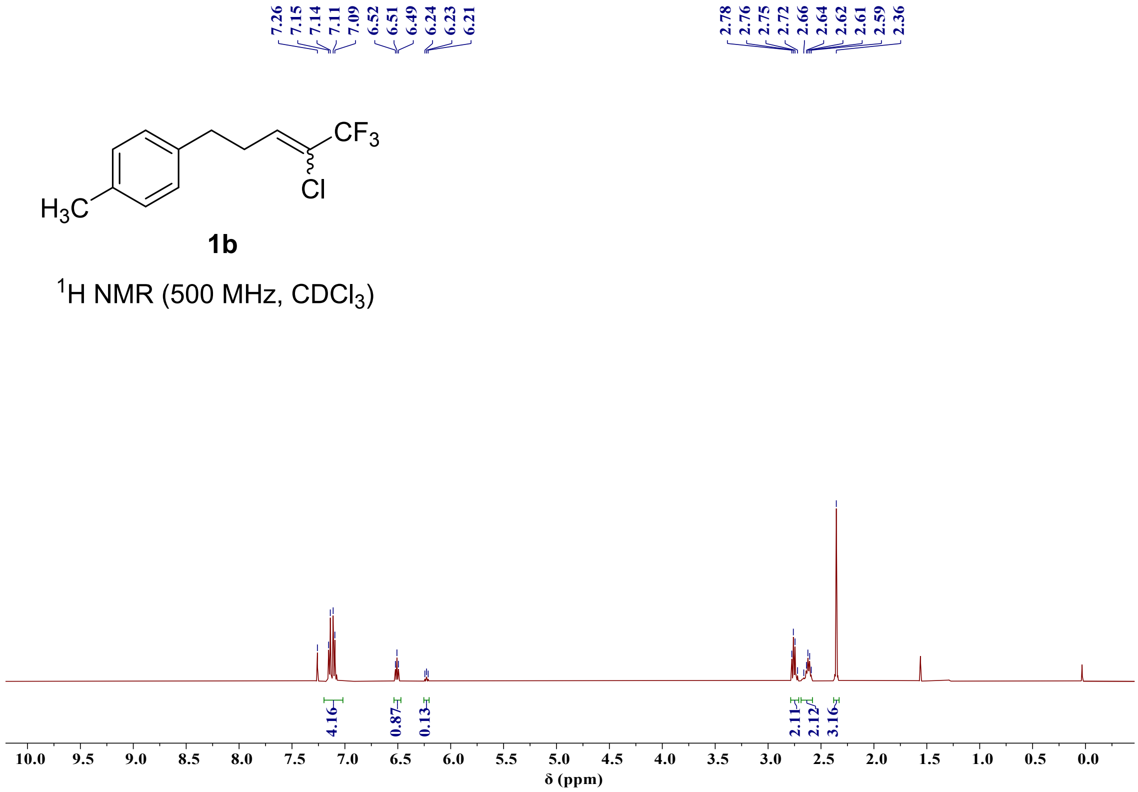

1-(4-chloro-5,5,5-trifluoropent-3-en-1-yl)-4-methylbenzene (1b)

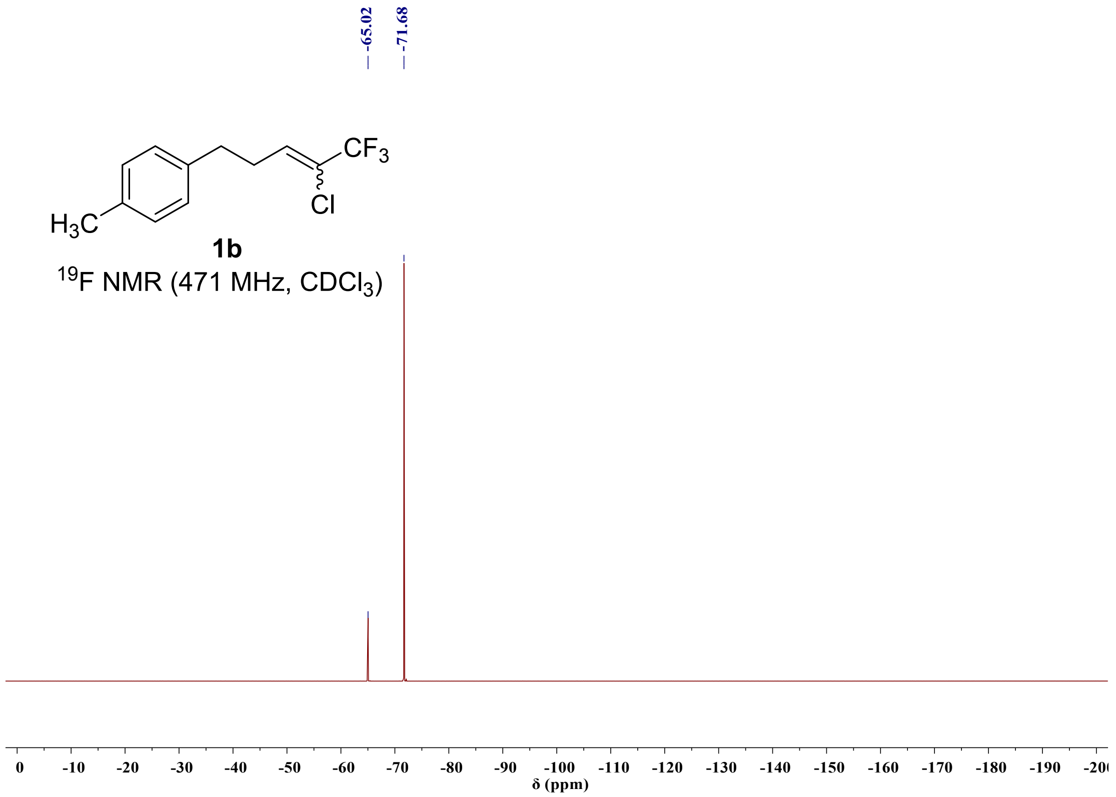

1-(4-chloro-5,5,5-trifluoropent-3-en-1-yl)-4-methylbenzene (1b)

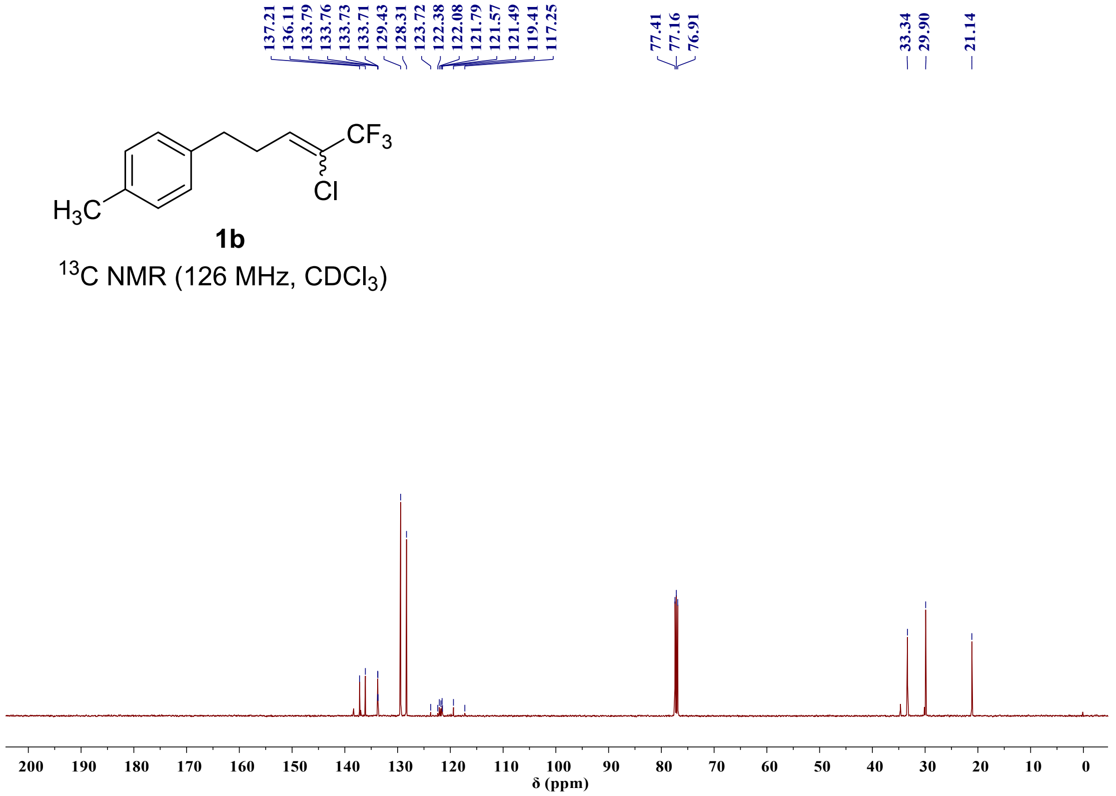

1-(4-chloro-5,5,5-trifluoropent-3-en-1-yl)-4-methoxybenzene (1c)

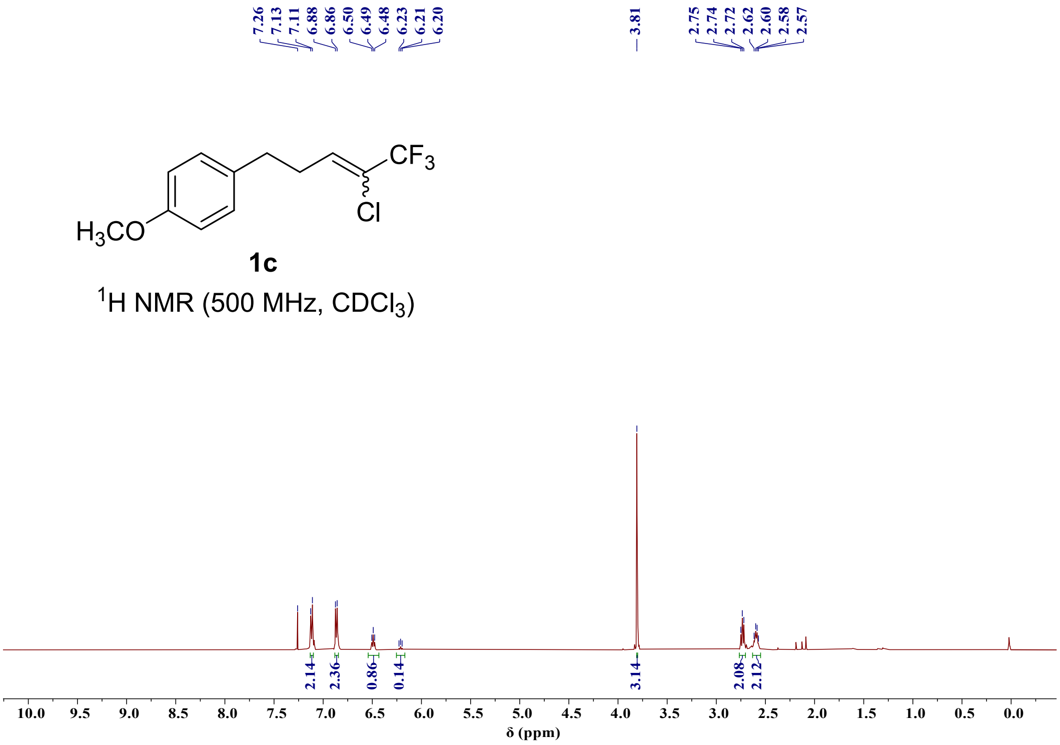

1-(4-chloro-5,5,5-trifluoropent-3-en-1-yl)-4-methoxybenzene (1c)

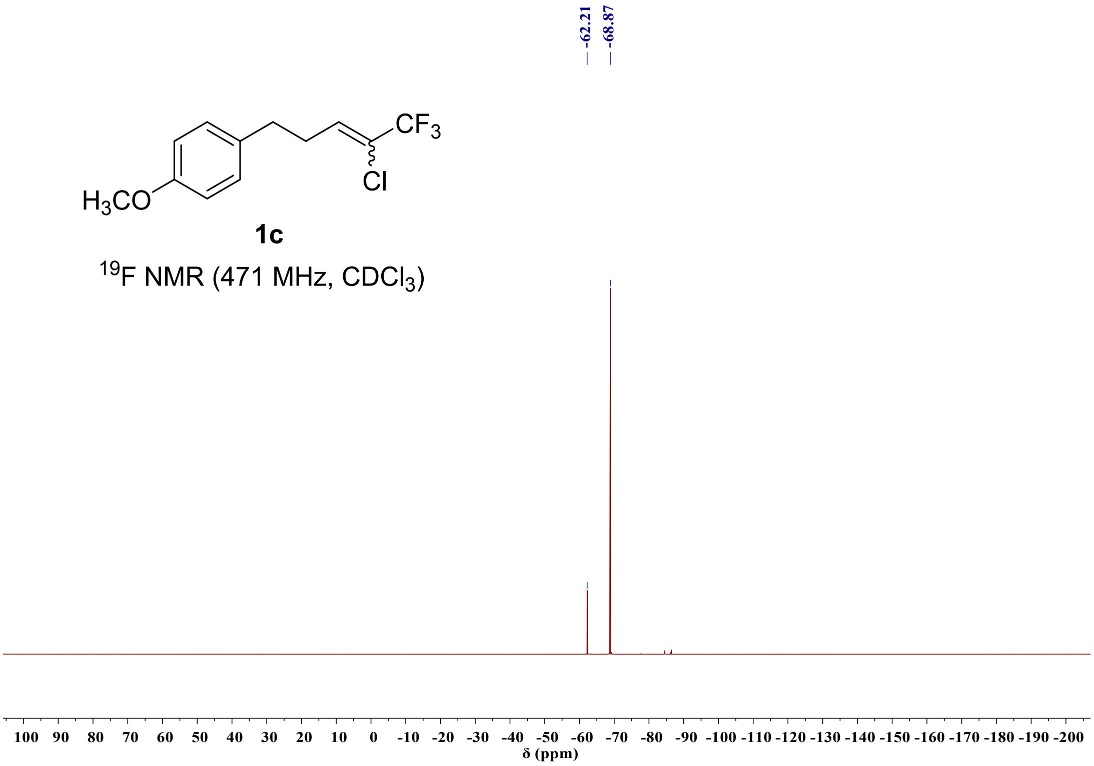

1-(4-chloro-5,5,5-trifluoropent-3-en-1-yl)-4-methoxybenzene (1c)

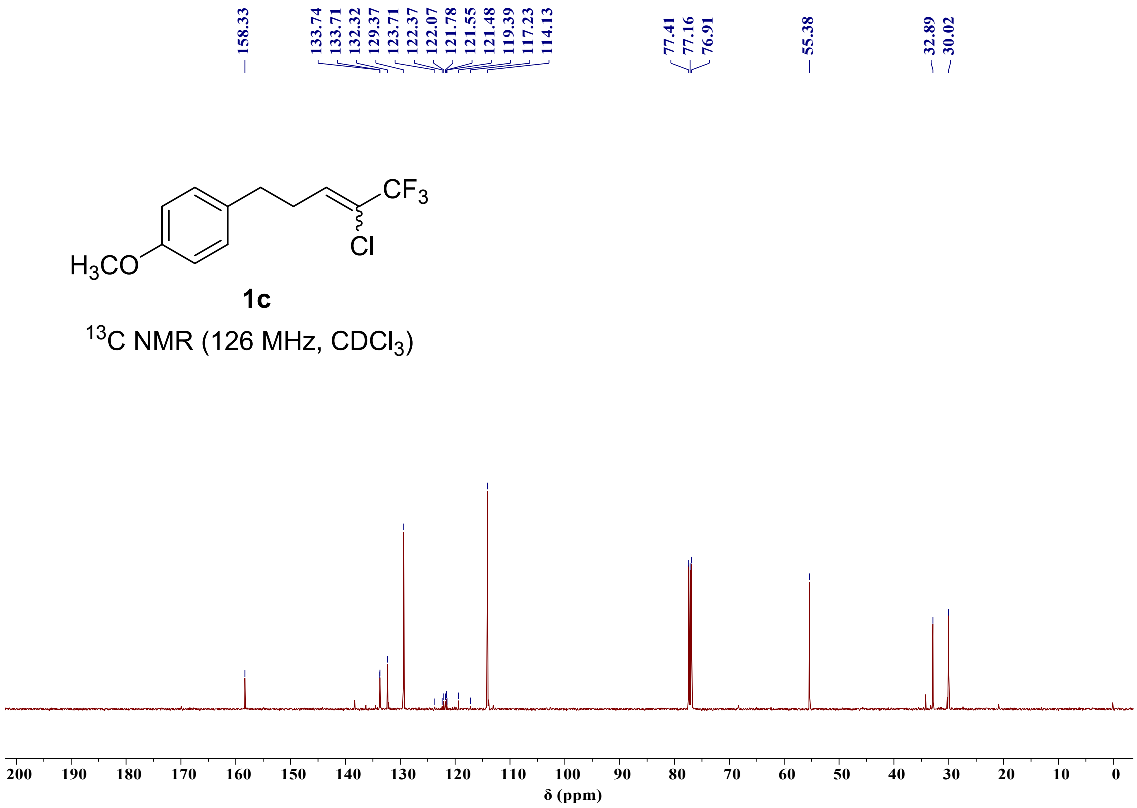

1-(4-chloro-5,5,5-trifluoropent-3-en-1-yl)-4-(trifluoromethyl)benzene (1d)

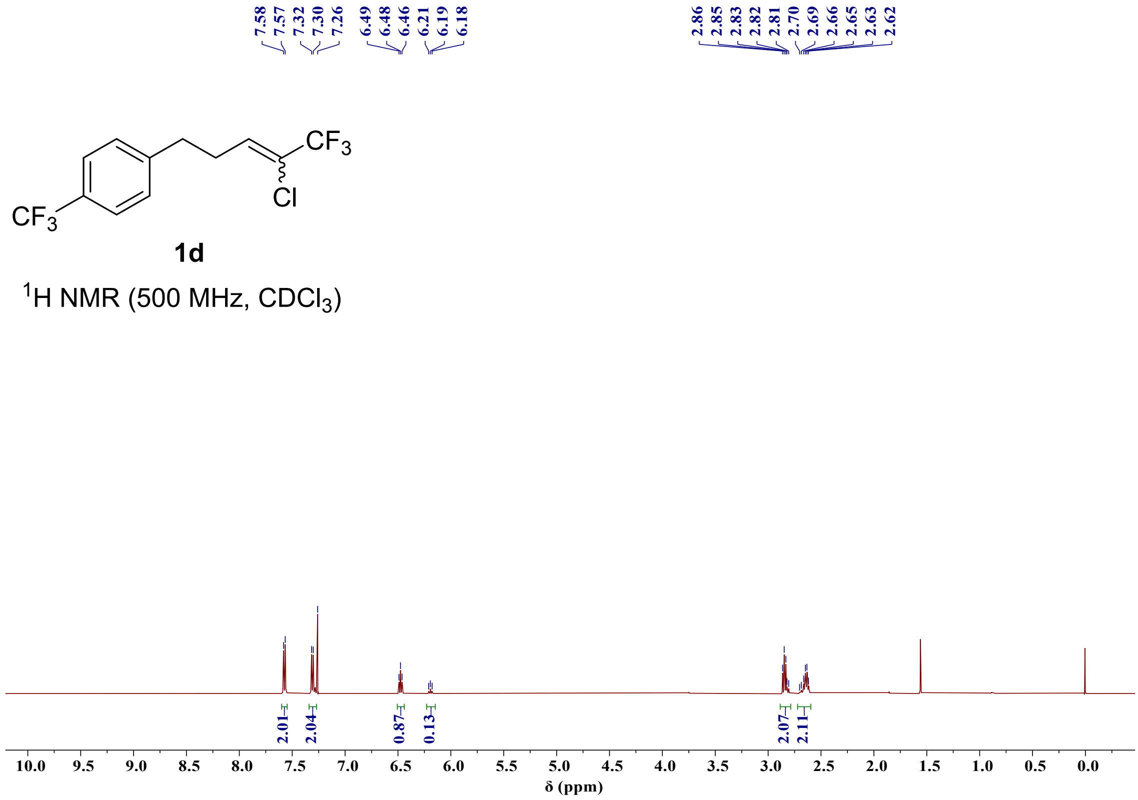

1-(4-chloro-5,5,5-trifluoropent-3-en-1-yl)-4-(trifluoromethyl)benzene (1d)

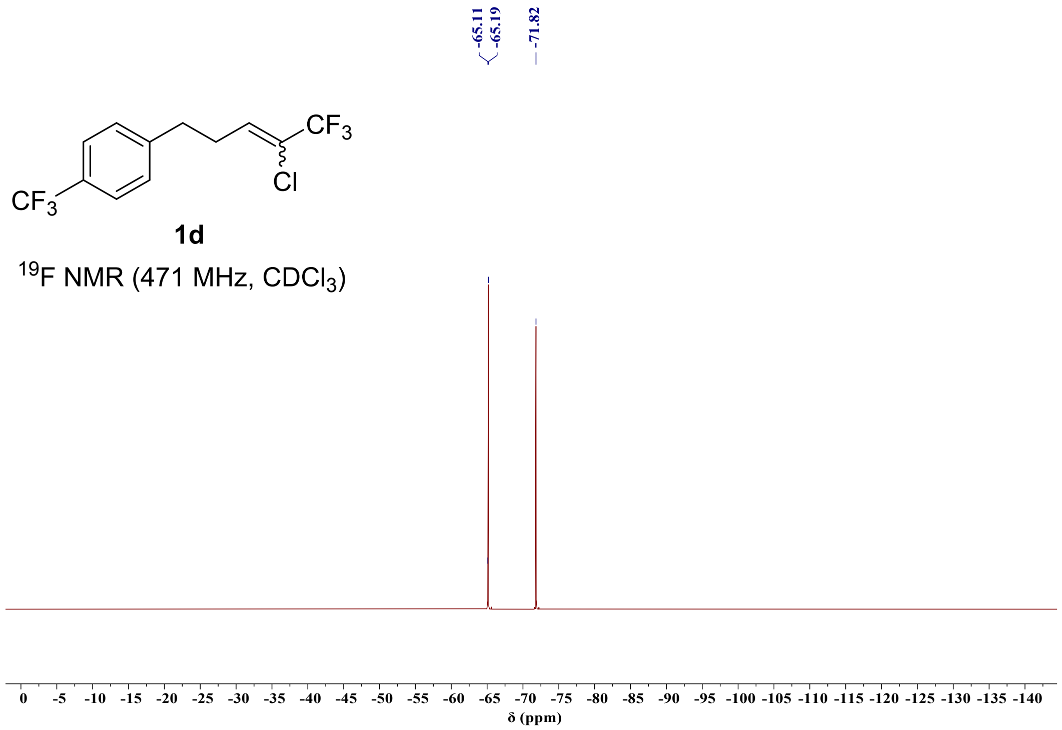

1-(4-chloro-5,5,5-trifluoropent-3-en-1-yl)-4-(trifluoromethyl)benzene (1d)

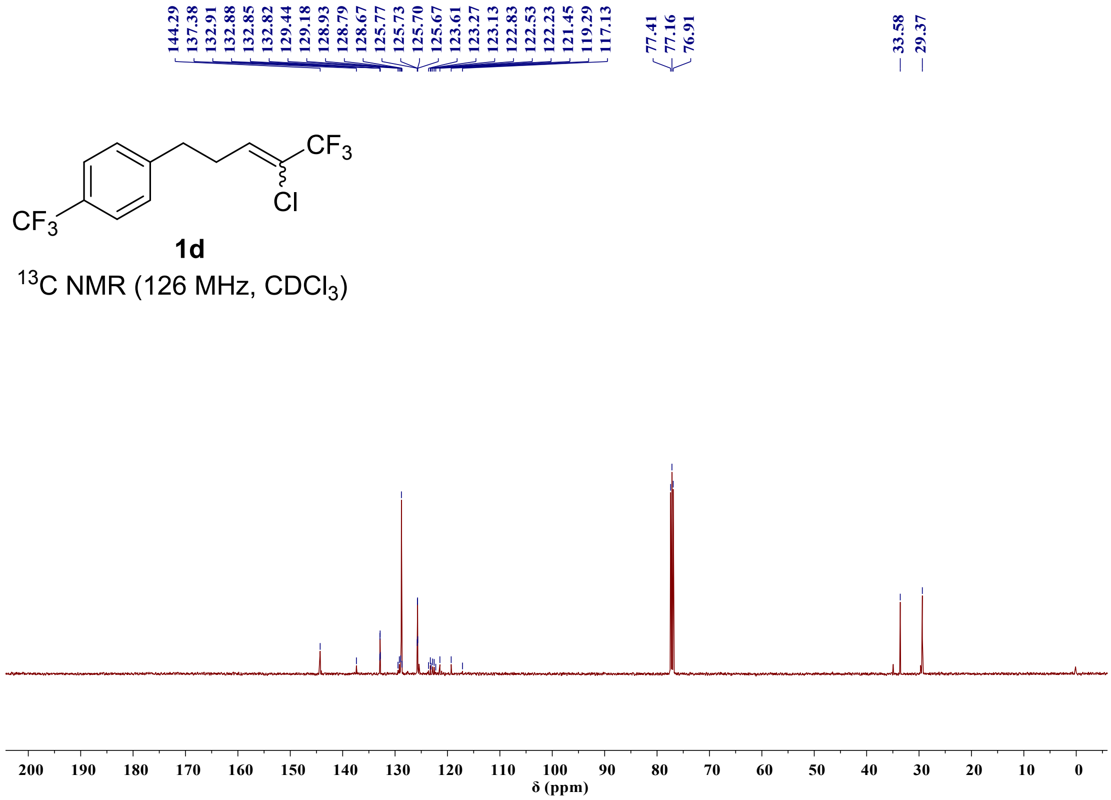

1-(4-chloro-5,5,5-trifluoropent-3-en-1-yl)-4-fluorobenzene (1e)

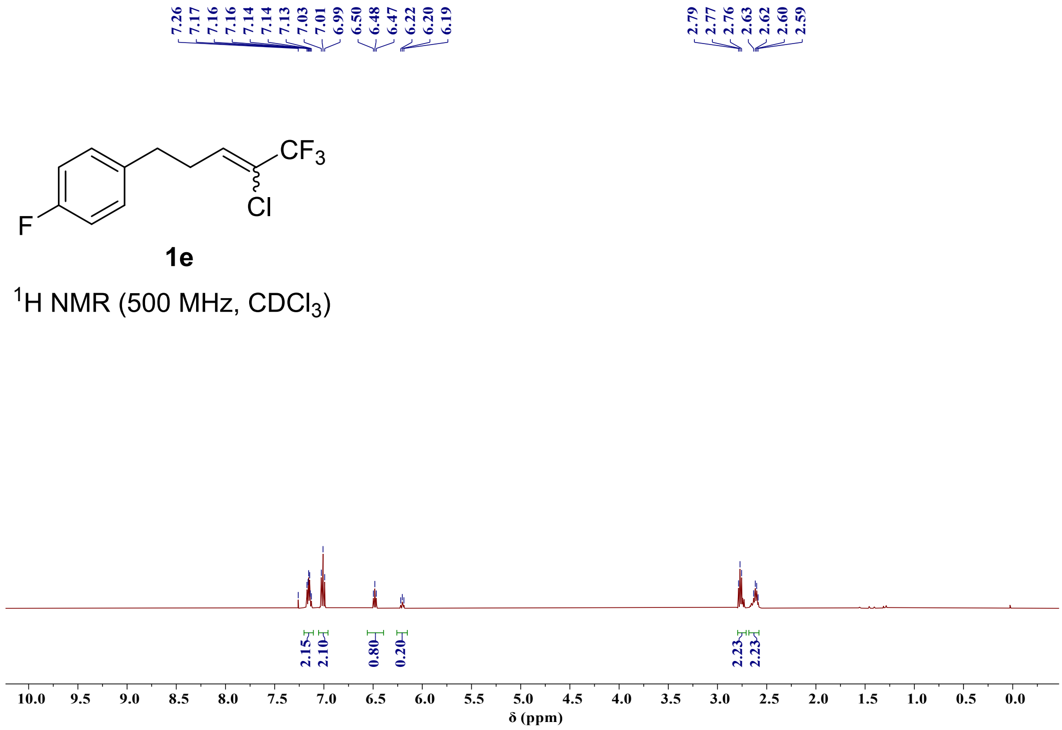

1e

^{1}H NMR (500 MHz, $CDCl_3$)

1-(4-chloro-5,5,5-trifluoropent-3-en-1-yl)-4-fluorobenzene (1e)

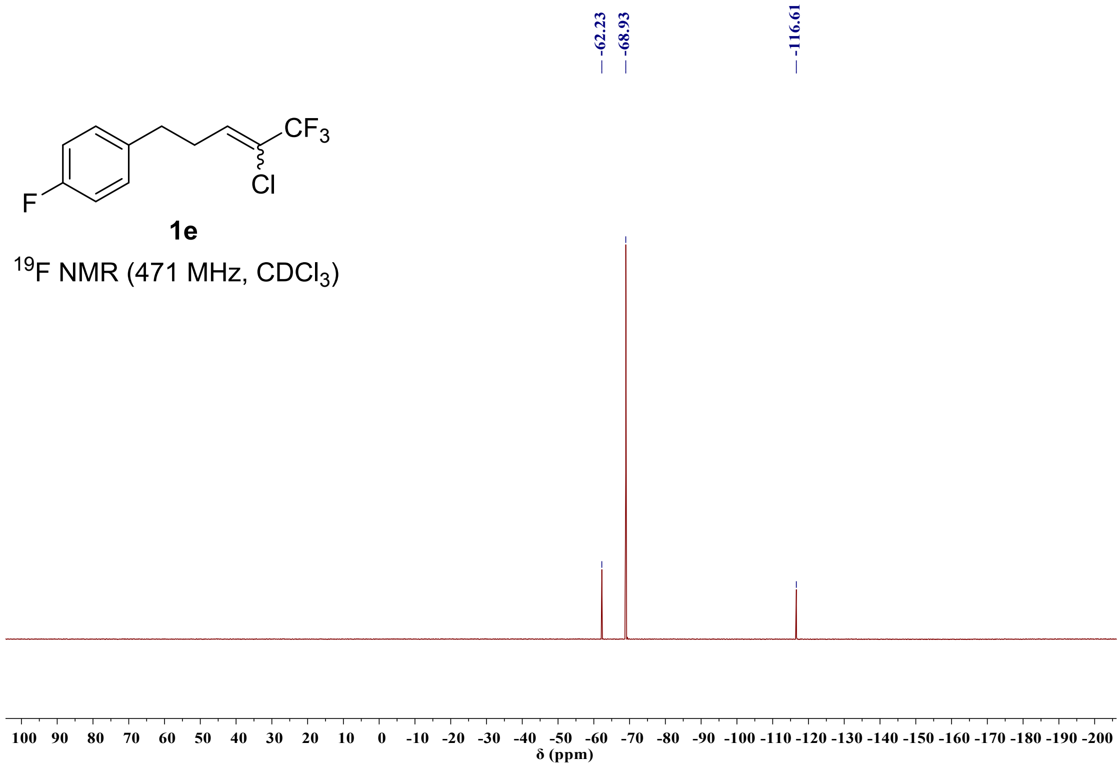

1e

^{19}F NMR (471 MHz, $CDCl_3$)

1-(4-chloro-5,5,5-trifluoropent-3-en-1-yl)-4-fluorobenzene (1e)

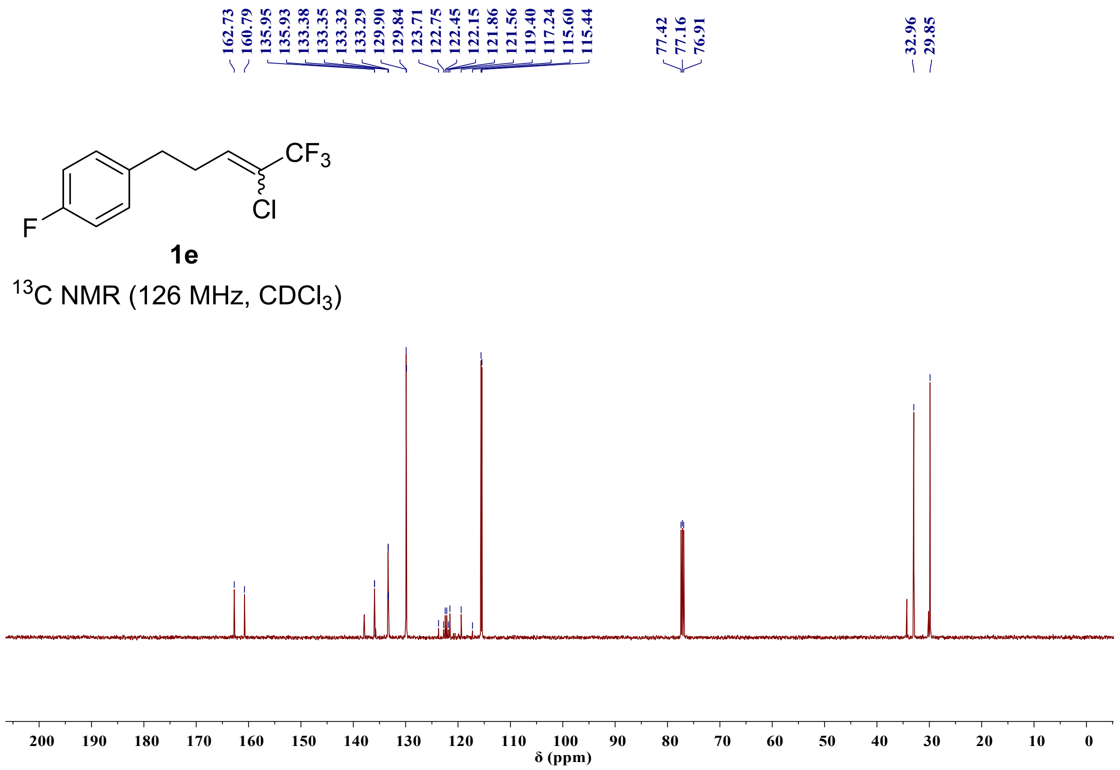

1-chloro-4-(4-chloro-5,5,5-trifluoropent-3-en-1-yl)benzene (1f)

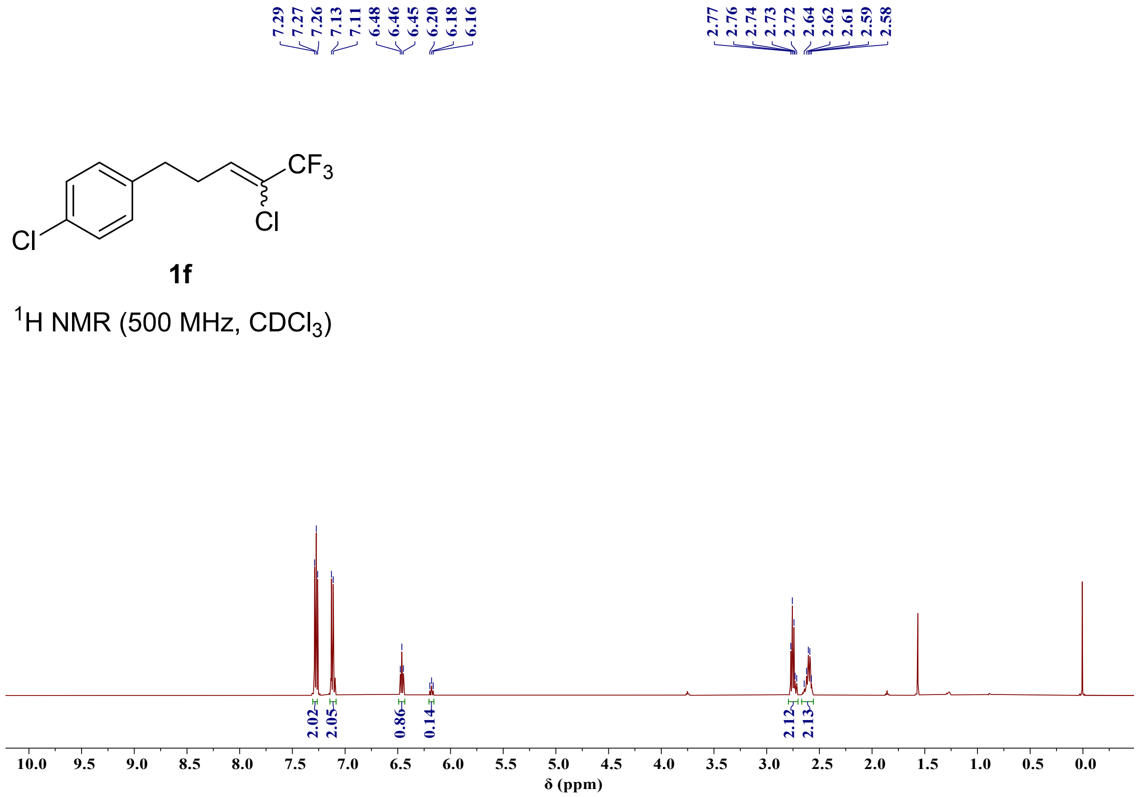

1-chloro-4-(4-chloro-5,5,5-trifluoropent-3-en-1-yl)benzene (1f)

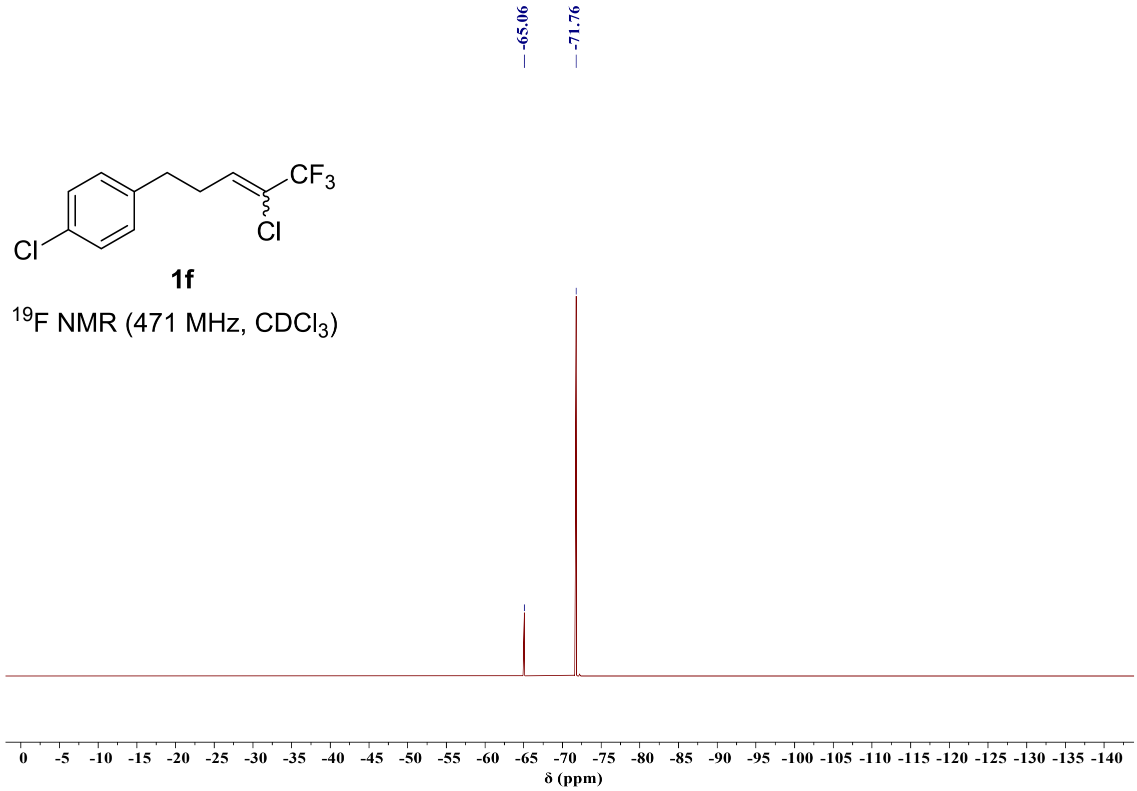

1-chloro-4-(4-chloro-5,5,5-trifluoropent-3-en-1-yl)benzene (1f)

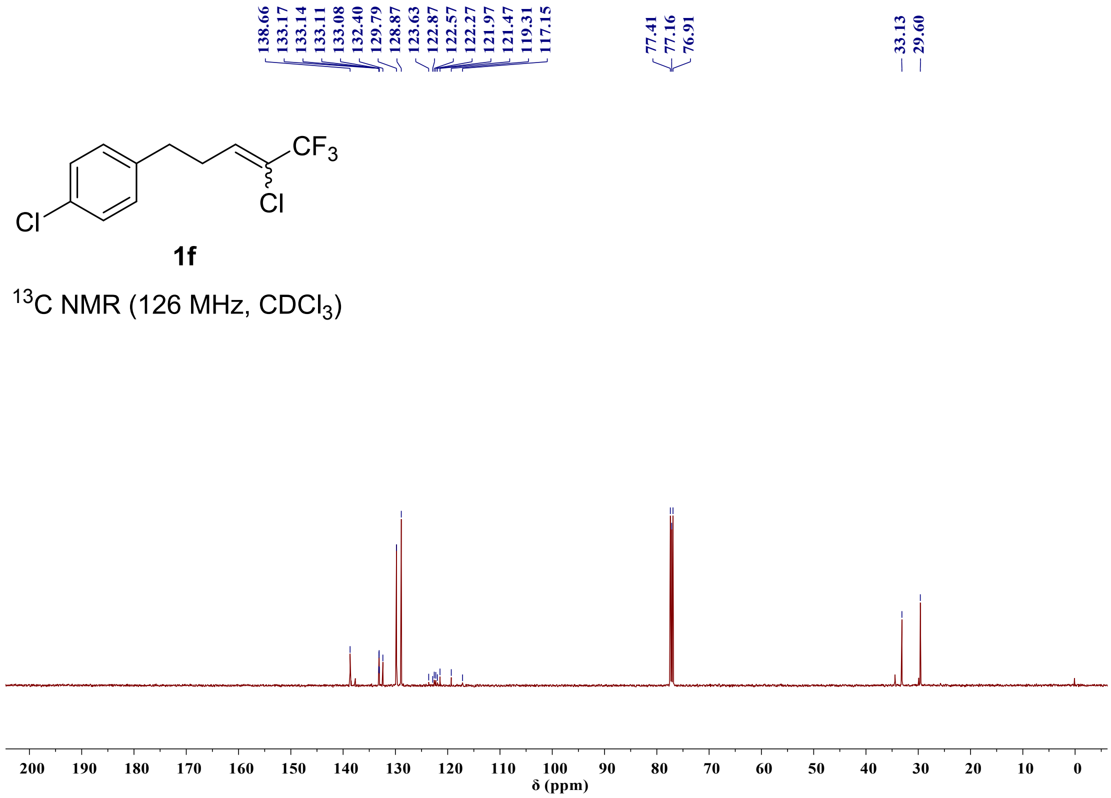

4-(4-chloro-5,5,5-trifluoropent-3-en-1-yl)phenyl acetate (1g)

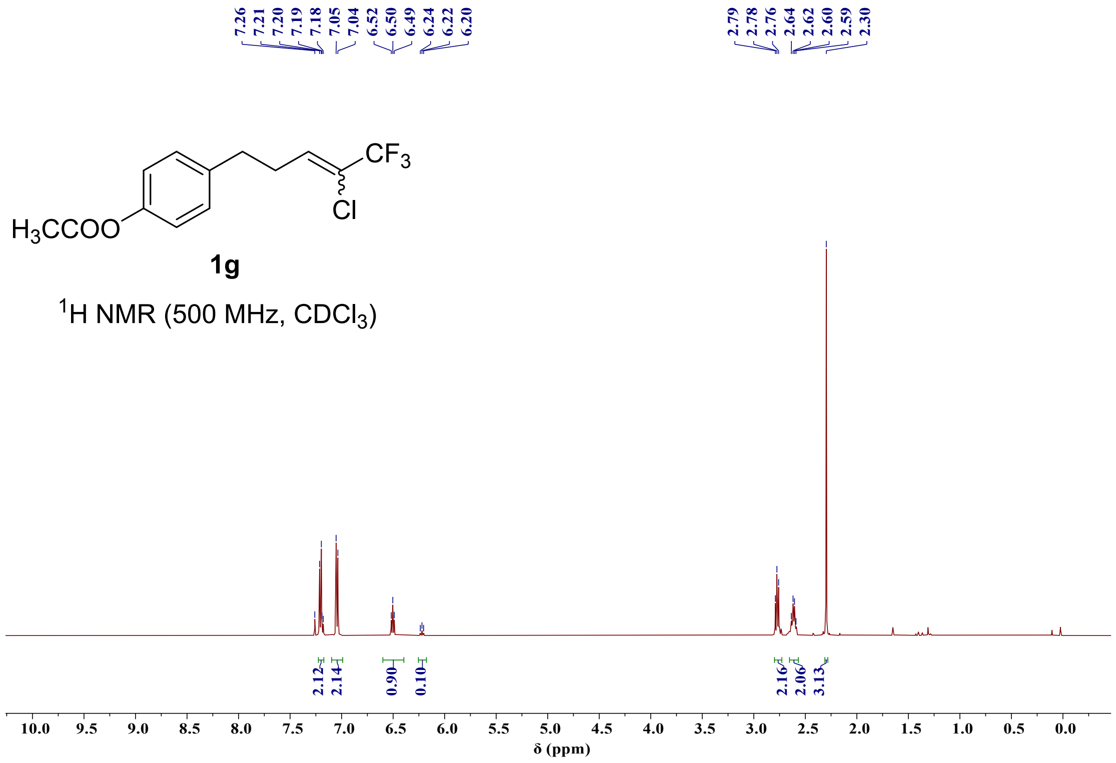

4-(4-chloro-5,5,5-trifluoropent-3-en-1-yl)phenyl acetate (1g)

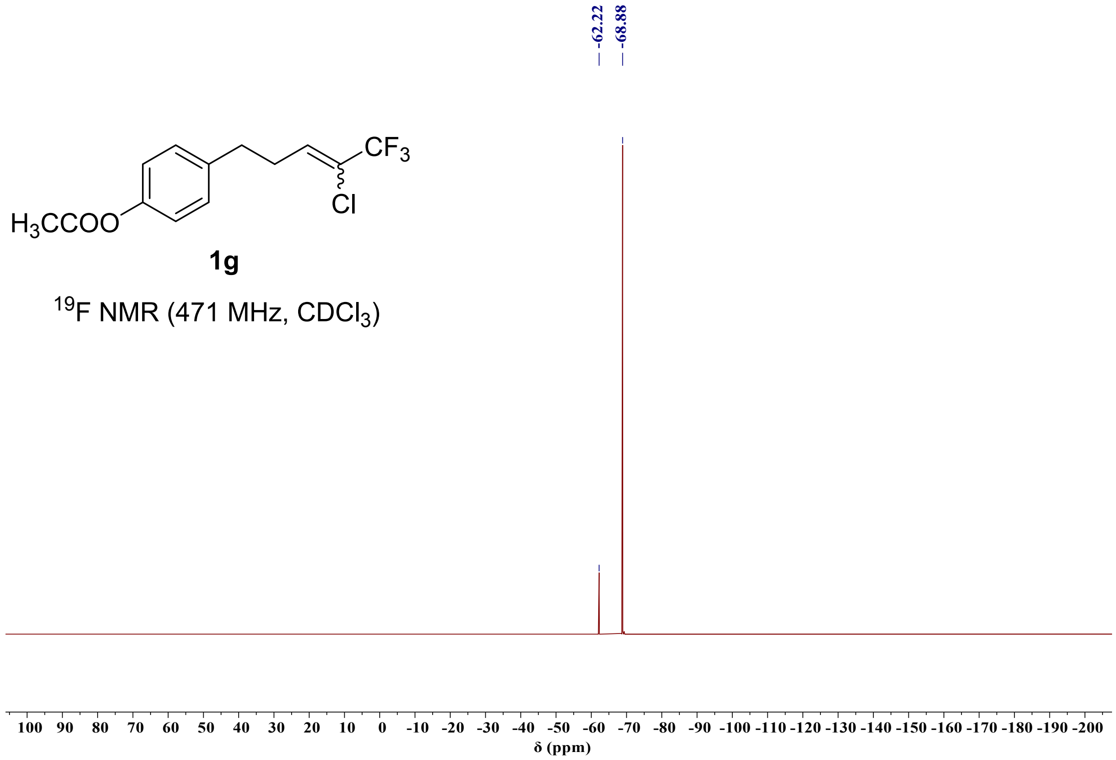

4-(4-chloro-5,5,5-trifluoropent-3-en-1-yl)phenyl acetate (1g)

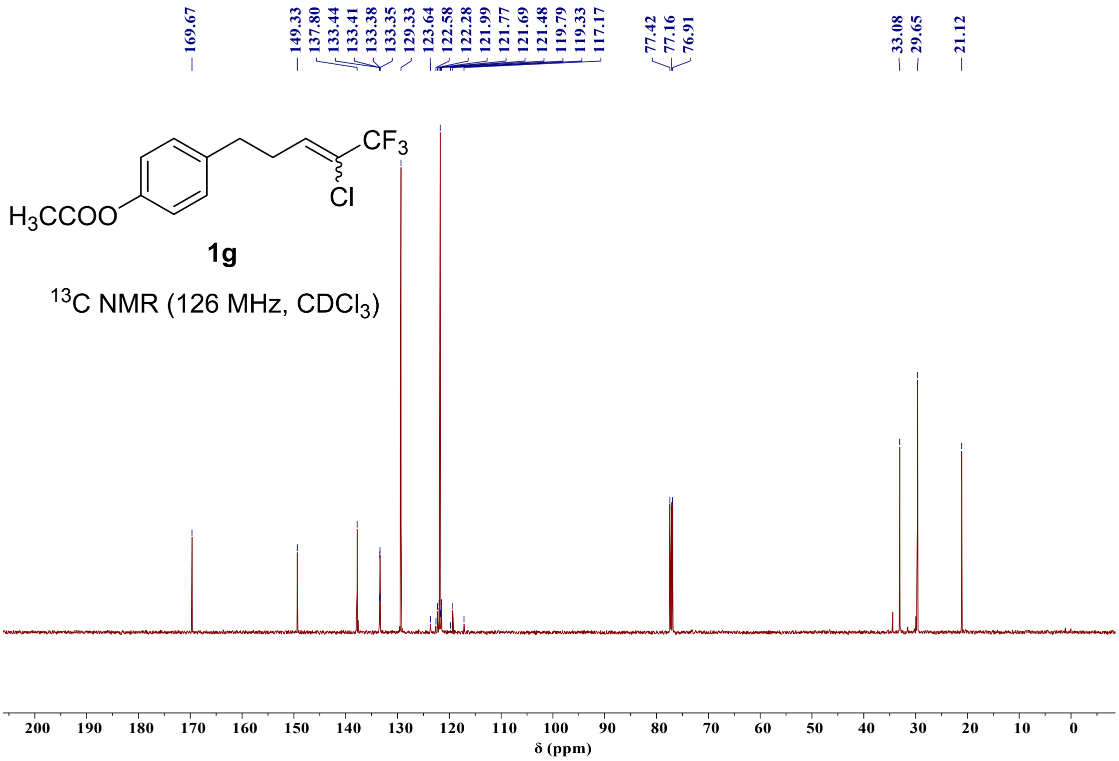

1-bromo-2-(4-chloro-5,5,5-trifluoropent-3-en-1-yl)benzene (1h)

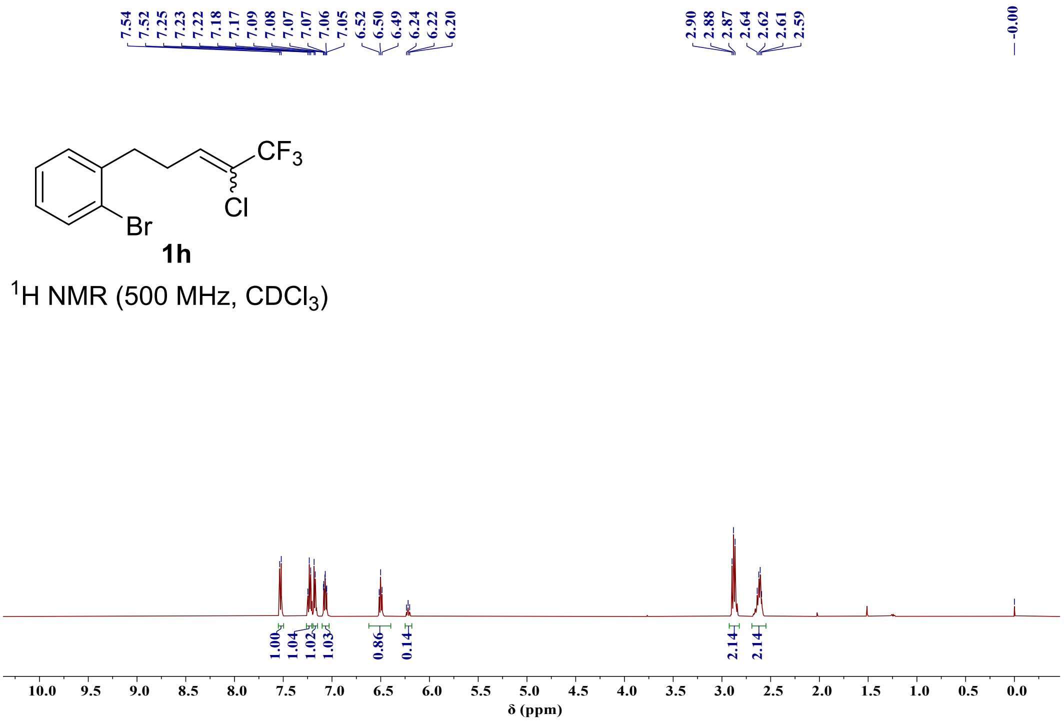

1-bromo-2-(4-chloro-5,5,5-trifluoropent-3-en-1-yl)benzene (h)

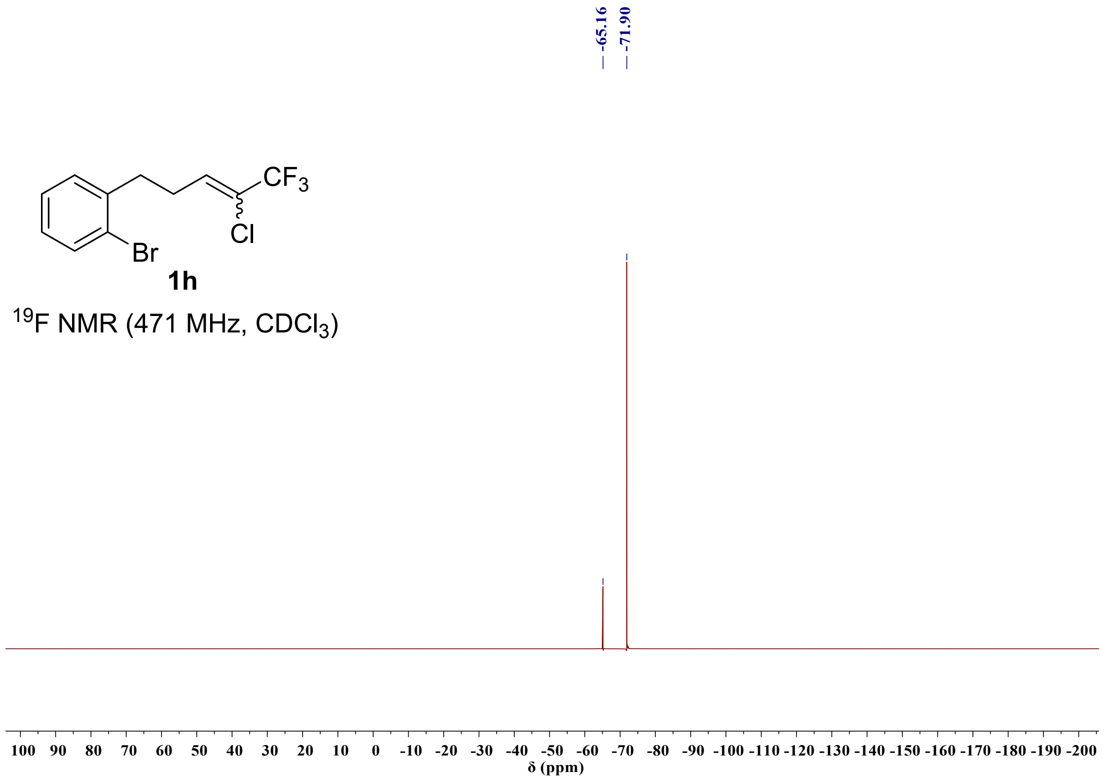

1-bromo-2-(4-chloro-5,5,5-trifluoropent-3-en-1-yl)benzene (h)

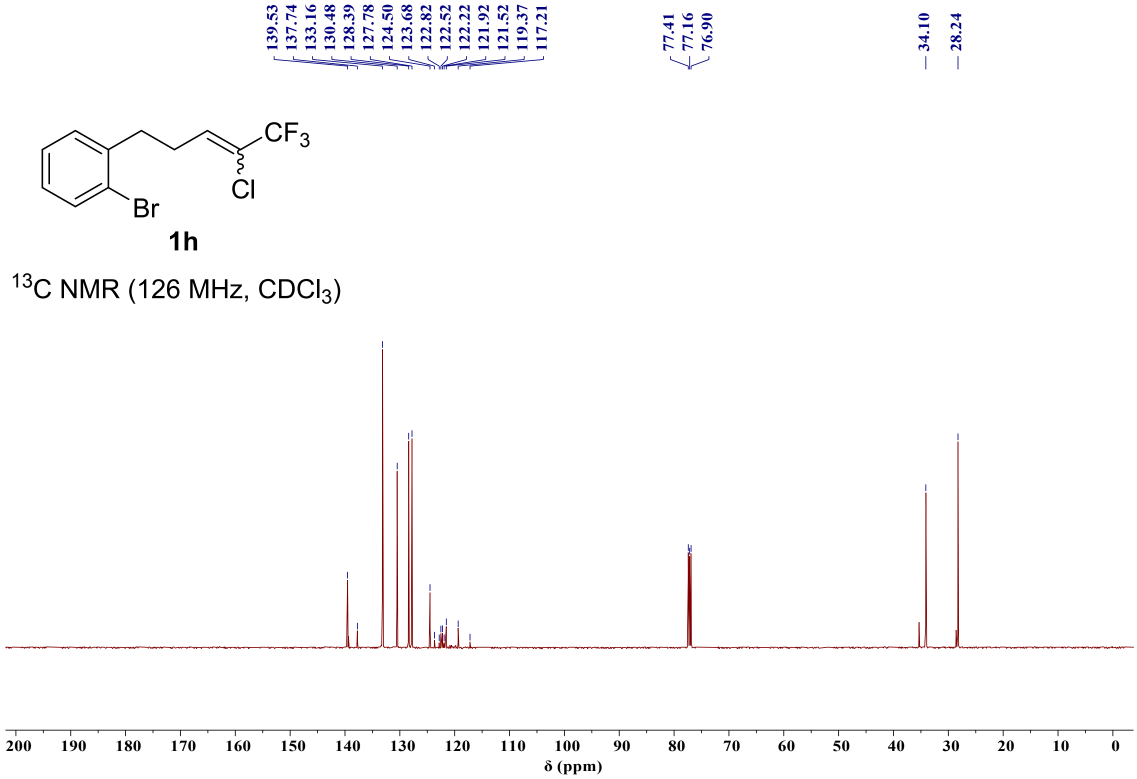

1-bromo-3-(4-chloro-5,5,5-trifluoropent-3-en-1-yl)benzene (1i)

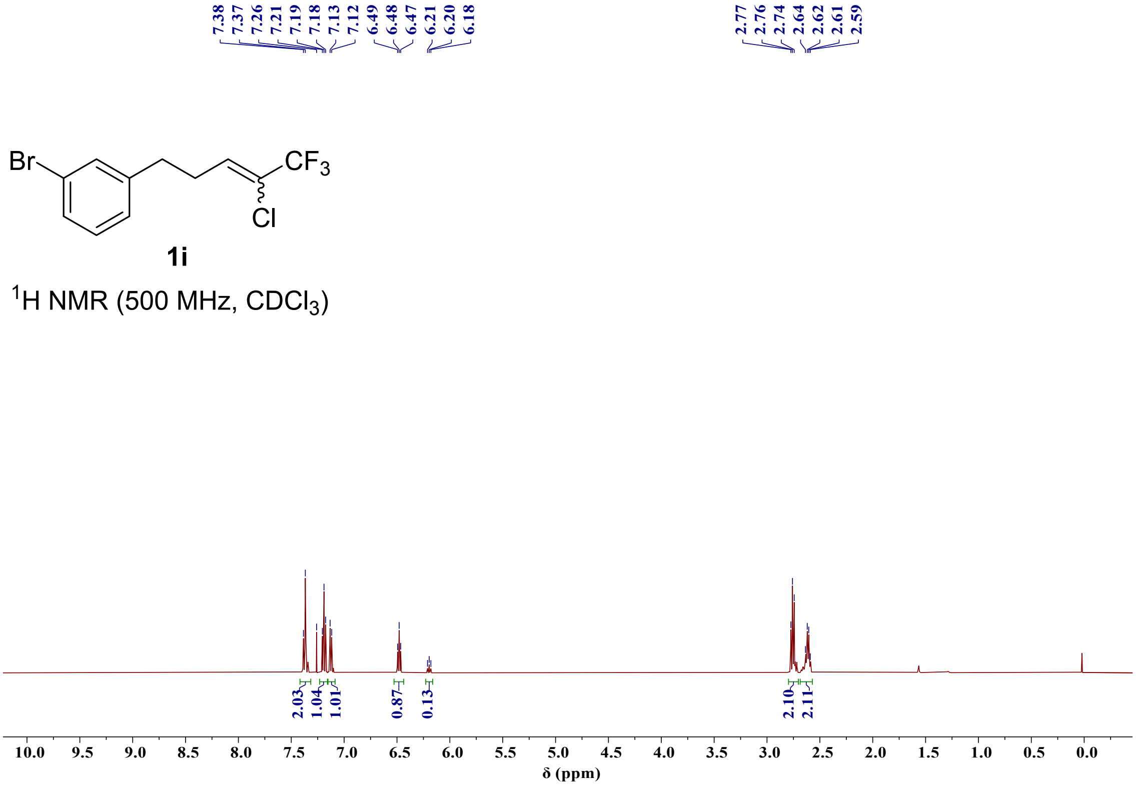

1-bromo-3-(4-chloro-5,5,5-trifluoropent-3-en-1-yl)benzene (1i)

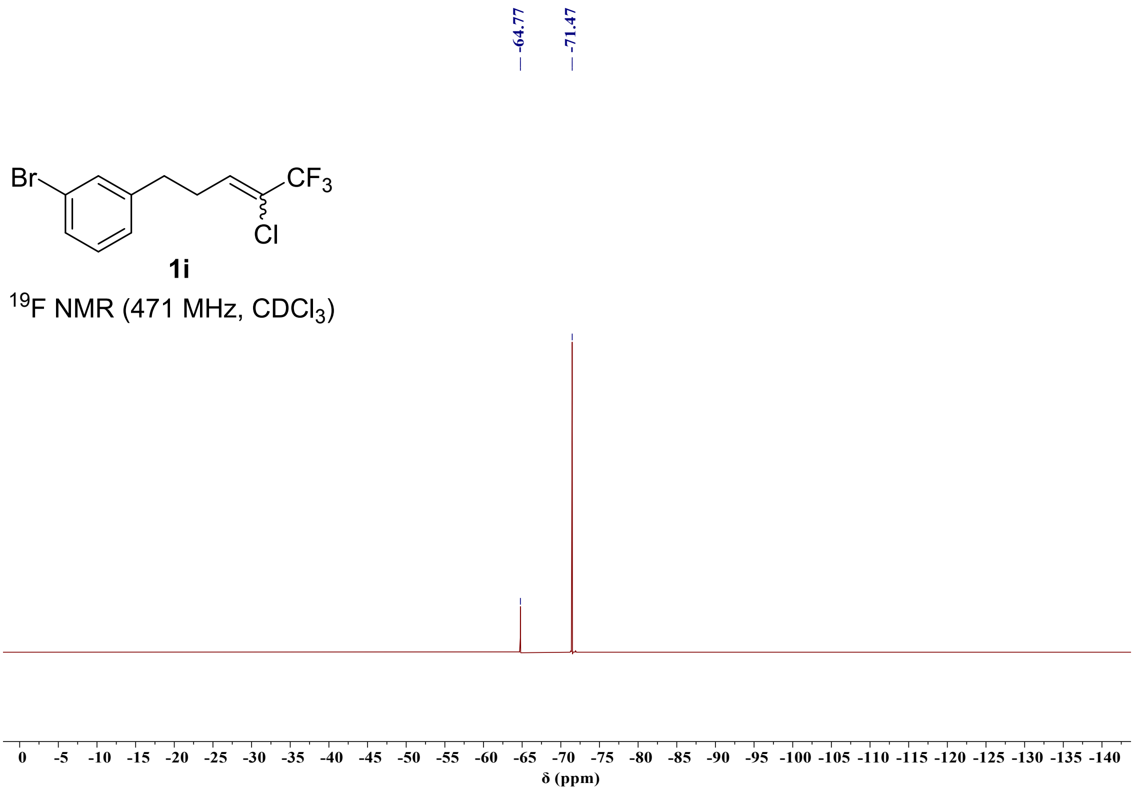

1-bromo-3-(4-chloro-5,5,5-trifluoropent-3-en-1-yl)benzene (1i)

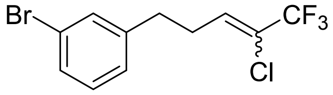

1i

^{13}C NMR (126 MHz, $CDCl_3$)

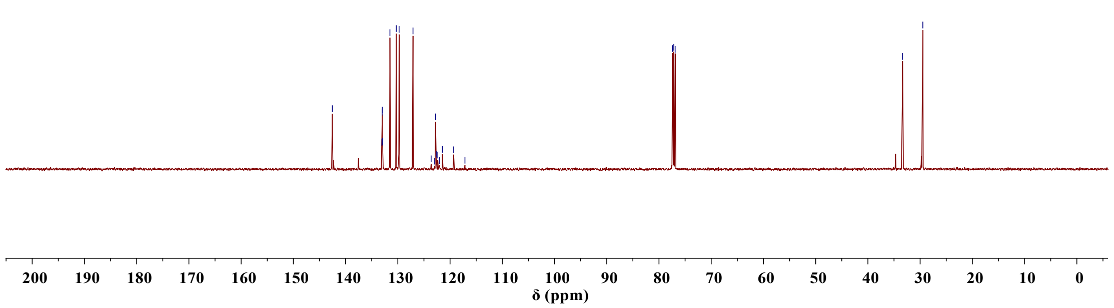

1-bromo-4-(4-chloro-5,5,5-trifluoropent-3-en-1-yl)benzene (1j)

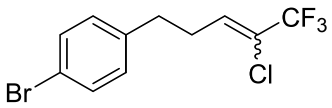

1j

^{1}H NMR (500 MHz, $CDCl_3$)

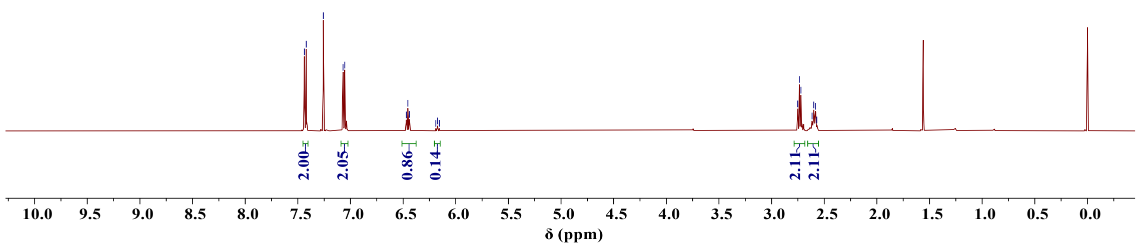

1-bromo-4-(4-chloro-5,5,5-trifluoropent-3-en-1-yl)benzene (1j)

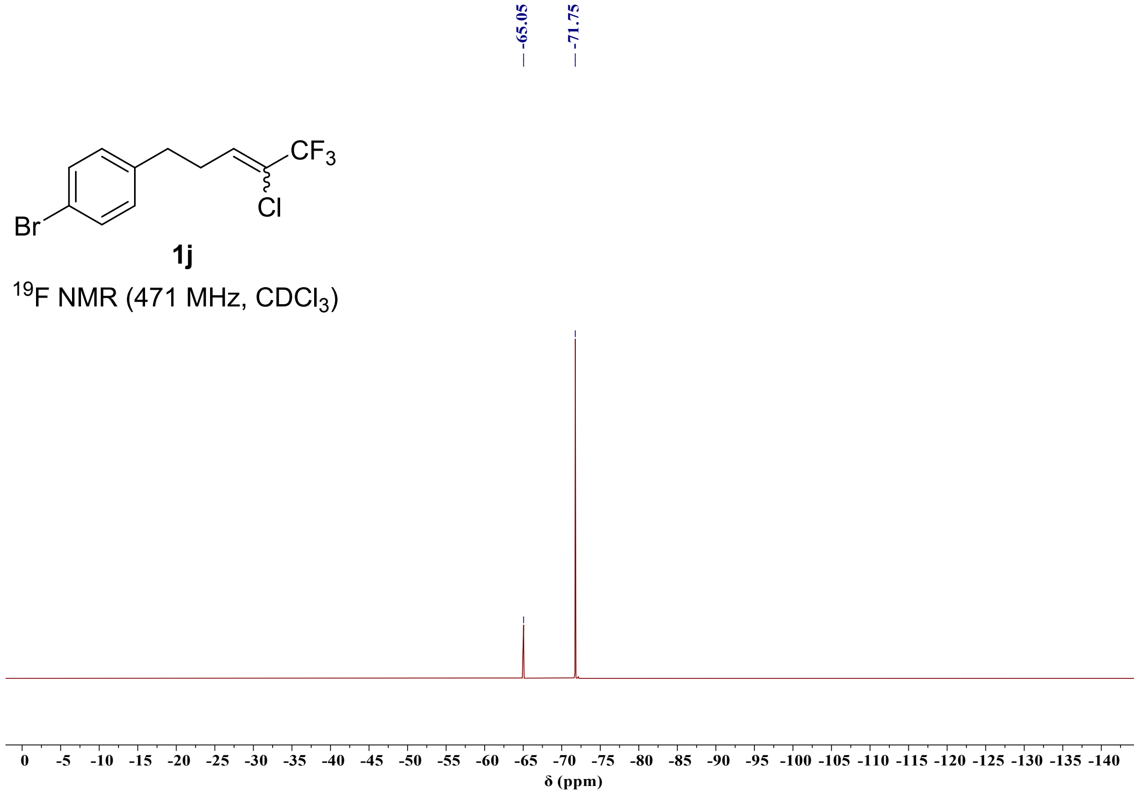

1-bromo-4-(4-chloro-5,5,5-trifluoropent-3-en-1-yl)benzene (1j)

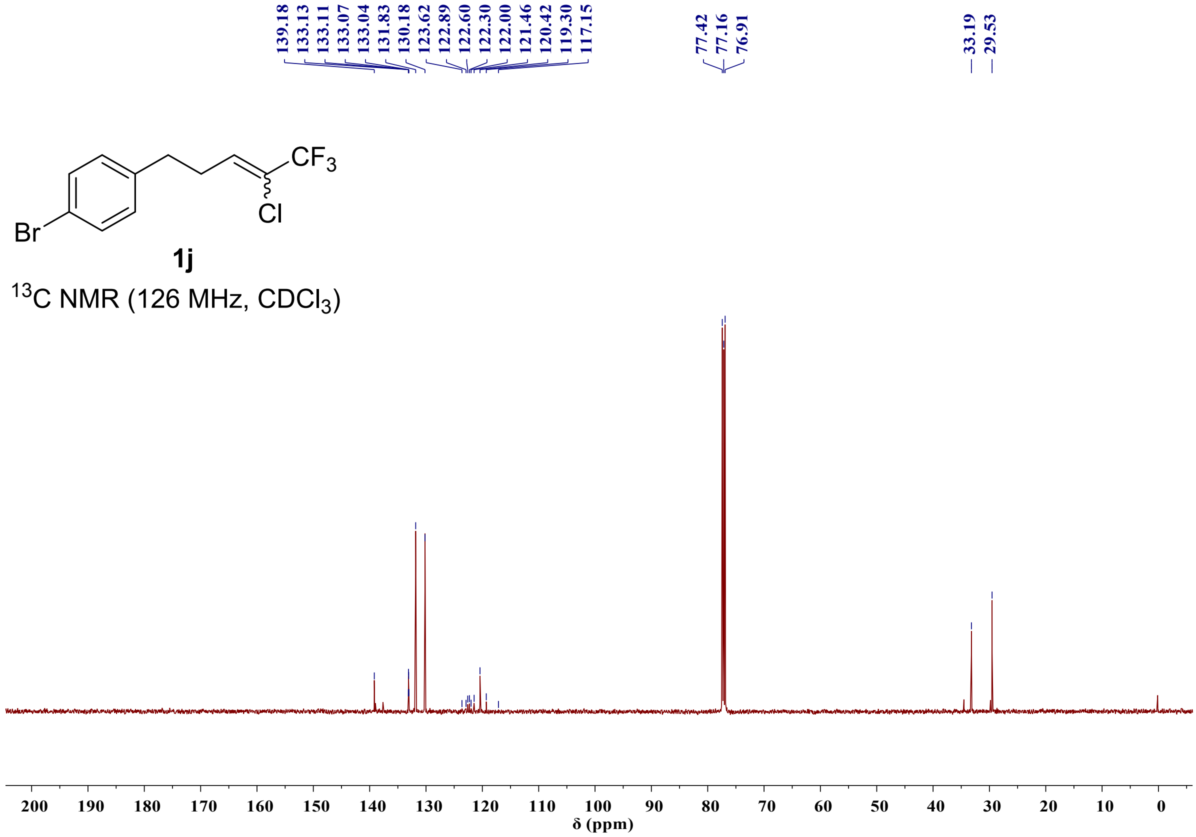

(3-chloro-4,4,4-trifluorobut-2-en-1-yl)benzene (1k)

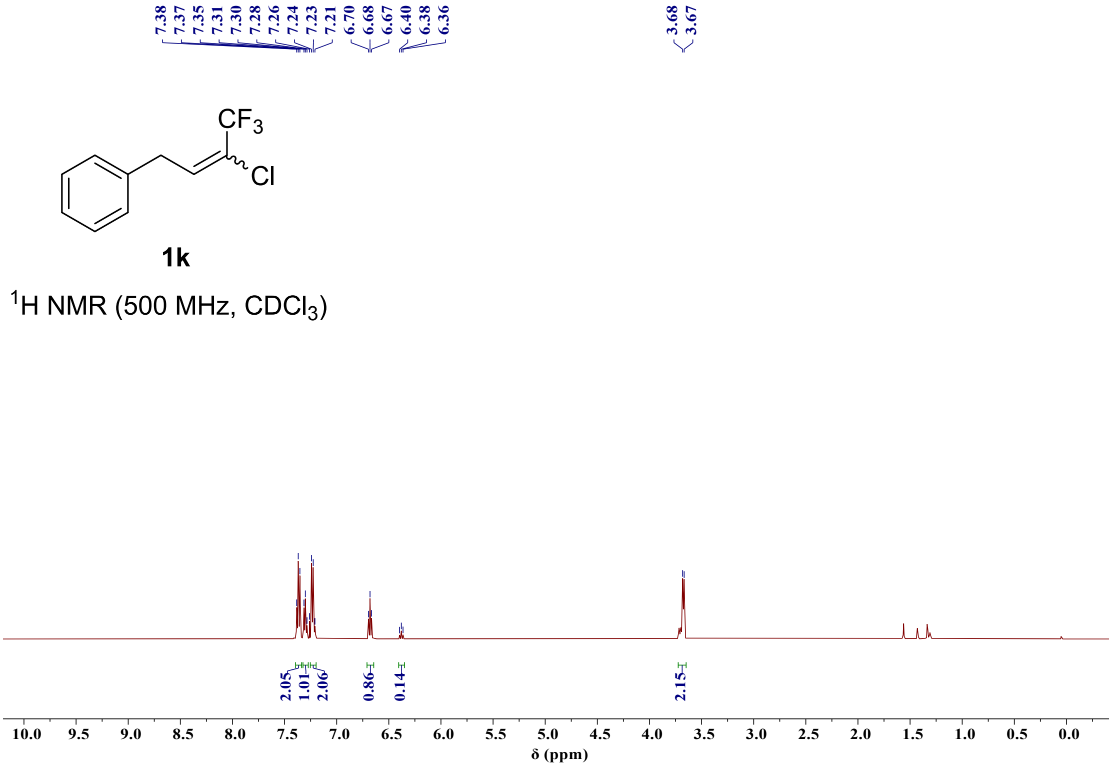

(3-chloro-4,4,4-trifluorobut-2-en-1-yl)benzene (1k)

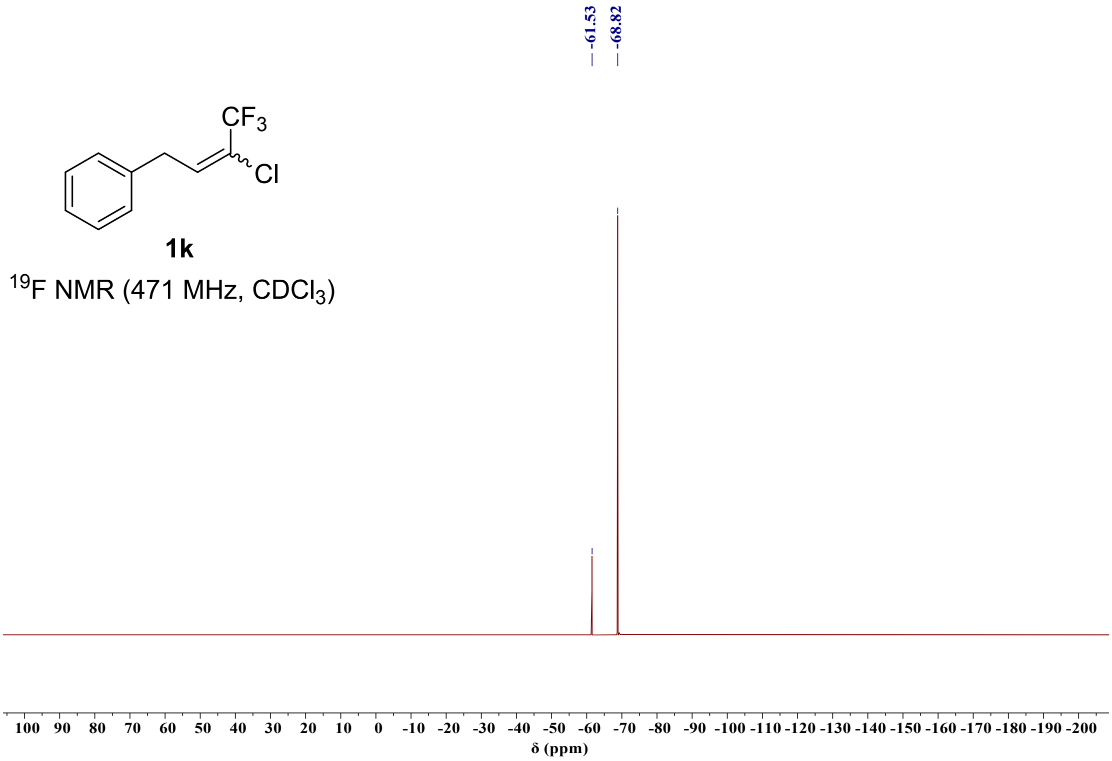

(3-chloro-4,4,4-trifluorobut-2-en-1-yl)benzene (1k)

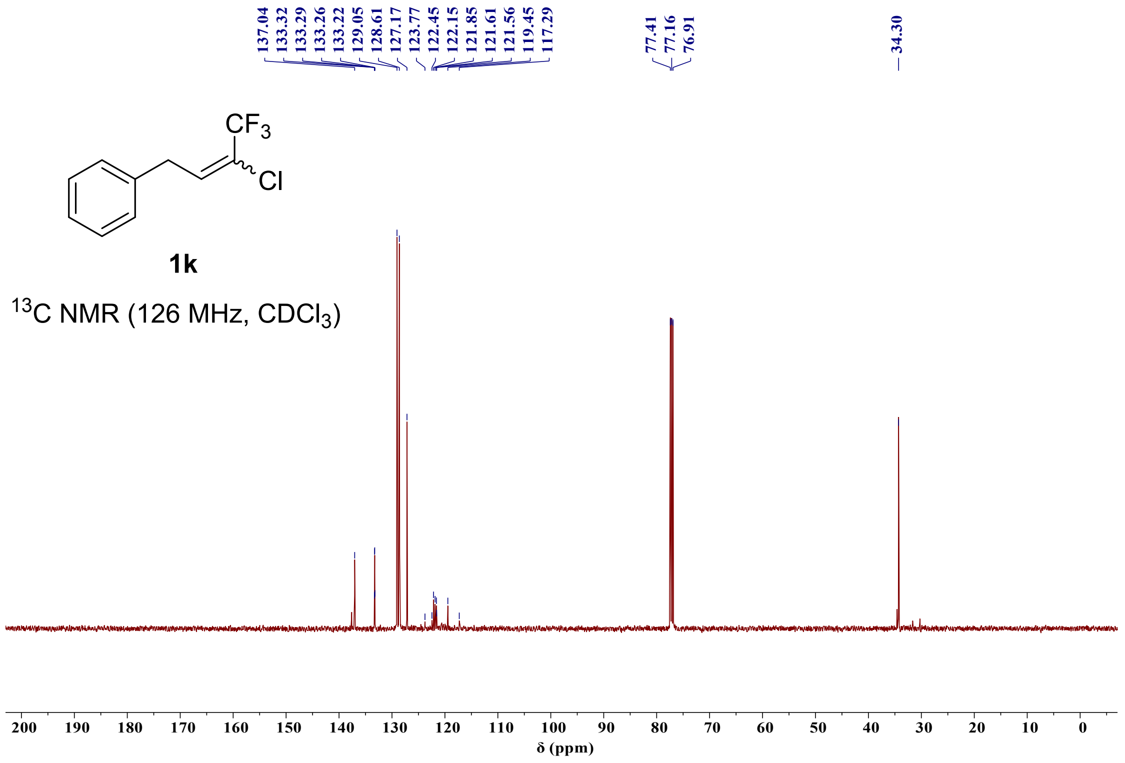

1-(3-chloro-4,4,4-trifluorobut-2-en-1-yl)-4-methoxybenzene (1l)

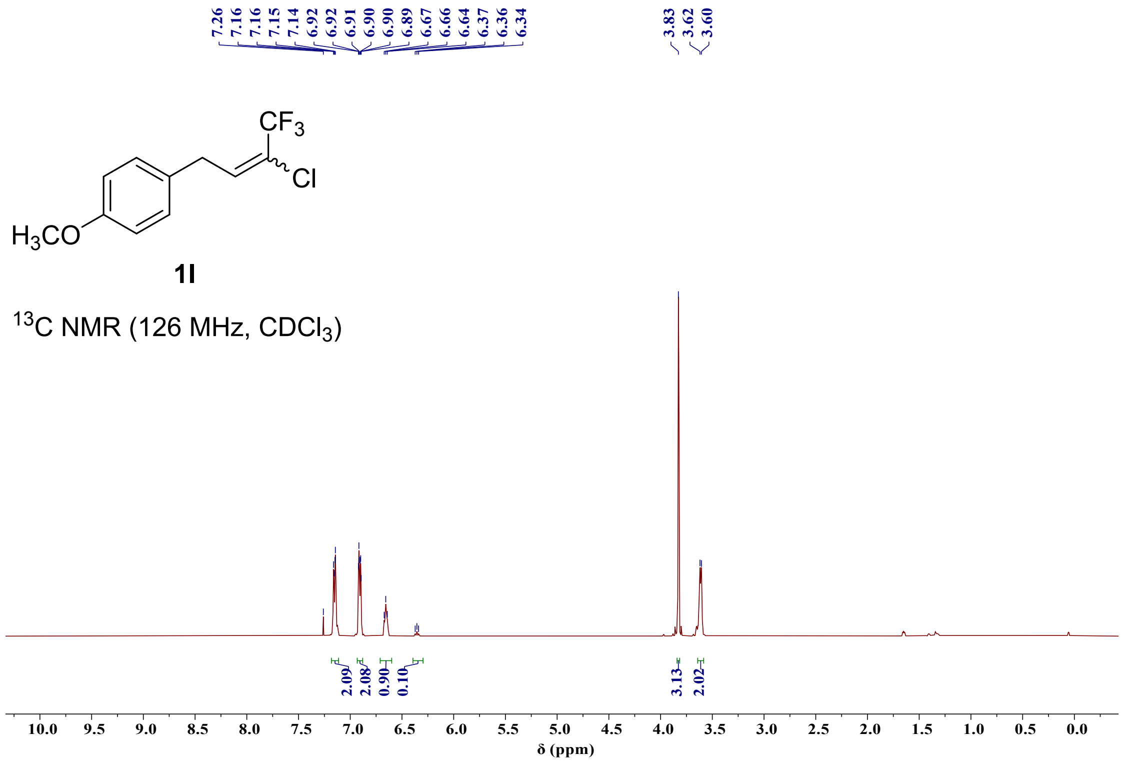

1-(3-chloro-4,4,4-trifluorobut-2-en-1-yl)-4-methoxybenzene (1l)

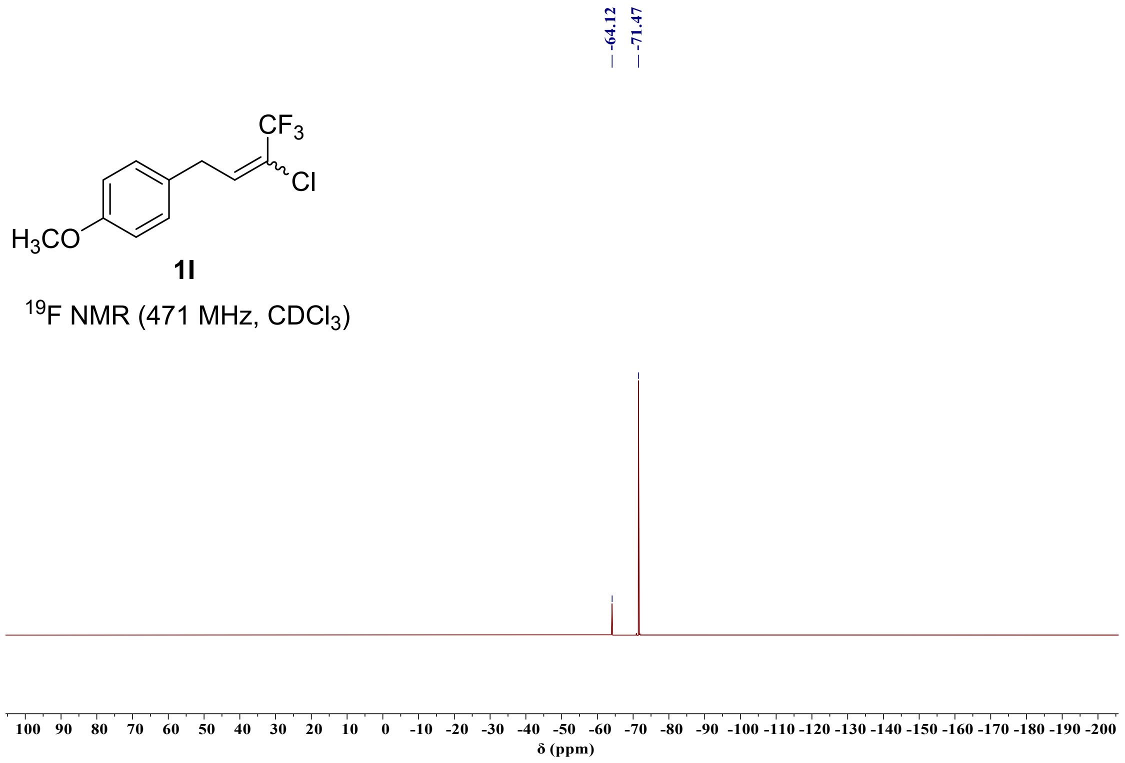

1-(3-chloro-4,4,4-trifluorobut-2-en-1-yl)-4-methoxybenzene (1l)

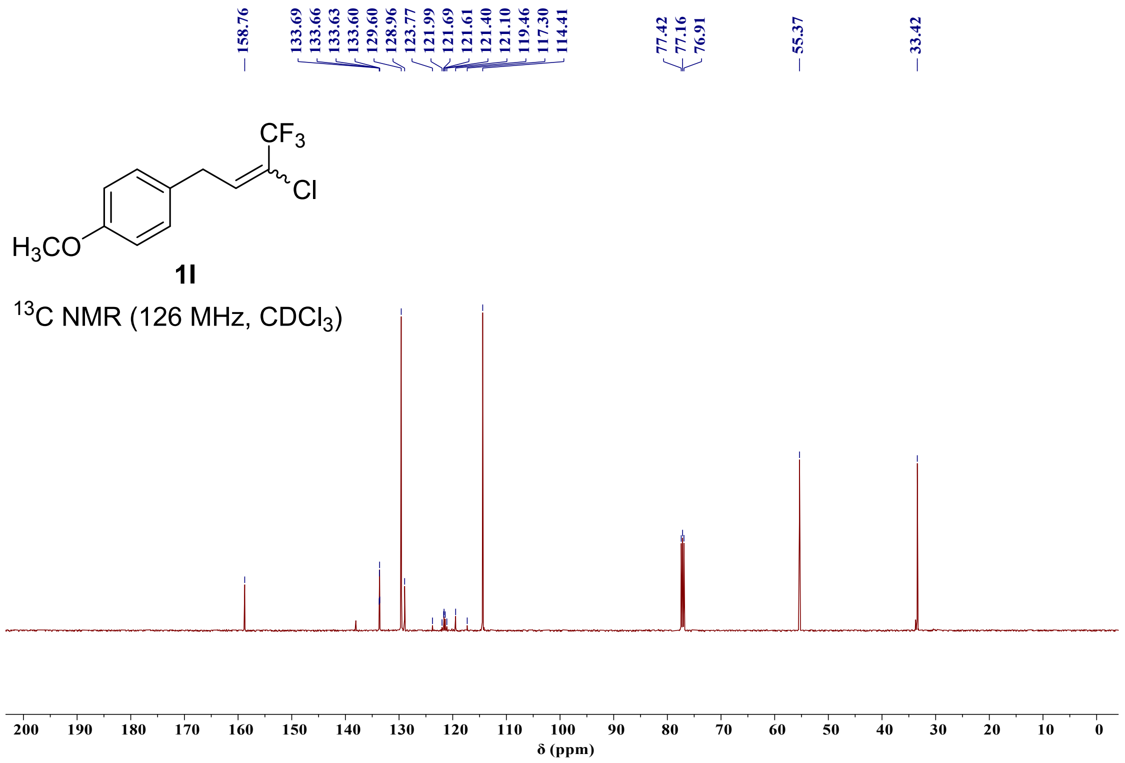

1-bromo-4-(3-chloro-4,4,4-trifluorobut-2-en-1-yl)benzene (1m)

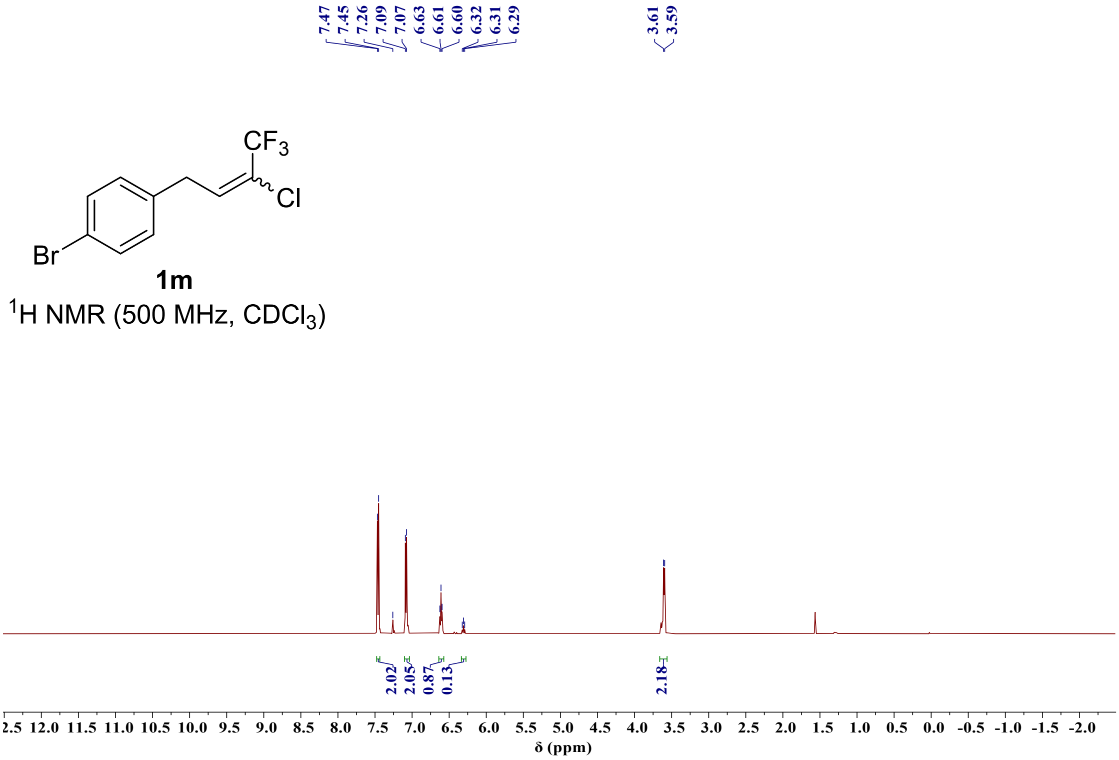

1-bromo-4-(3-chloro-4,4,4-trifluorobut-2-en-1-yl)benzene (1m)

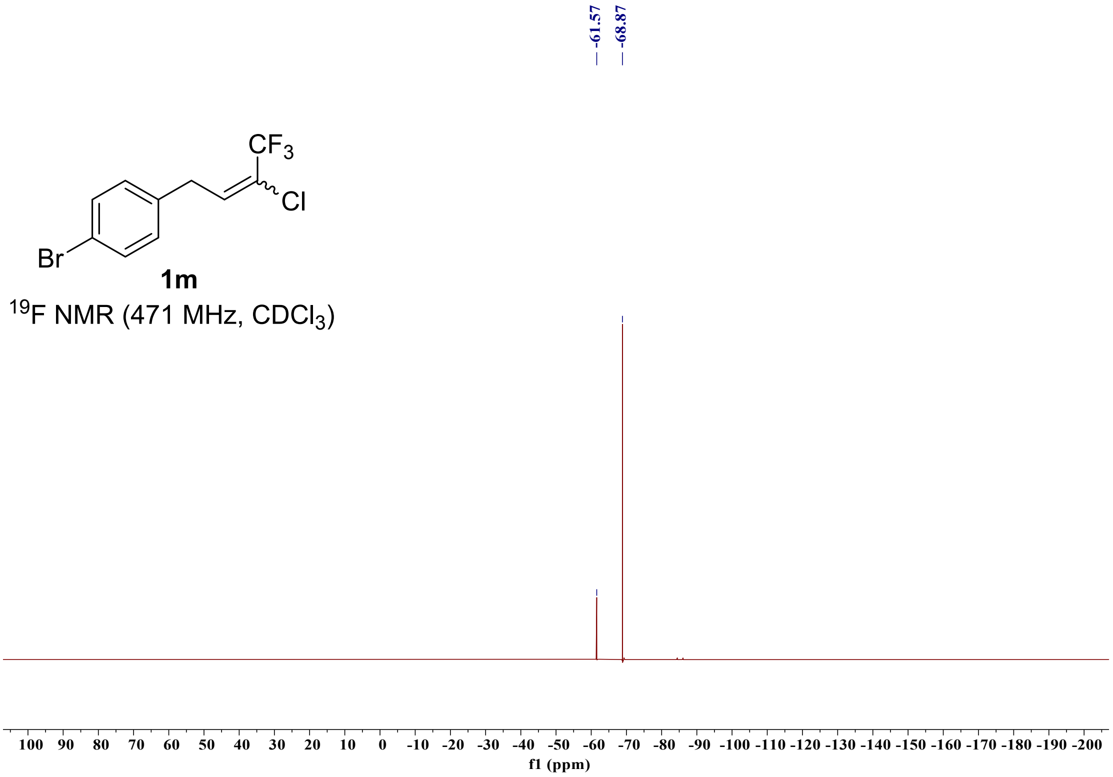

1-bromo-4-(3-chloro-4,4,4-trifluorobut-2-en-1-yl)benzene (1m)

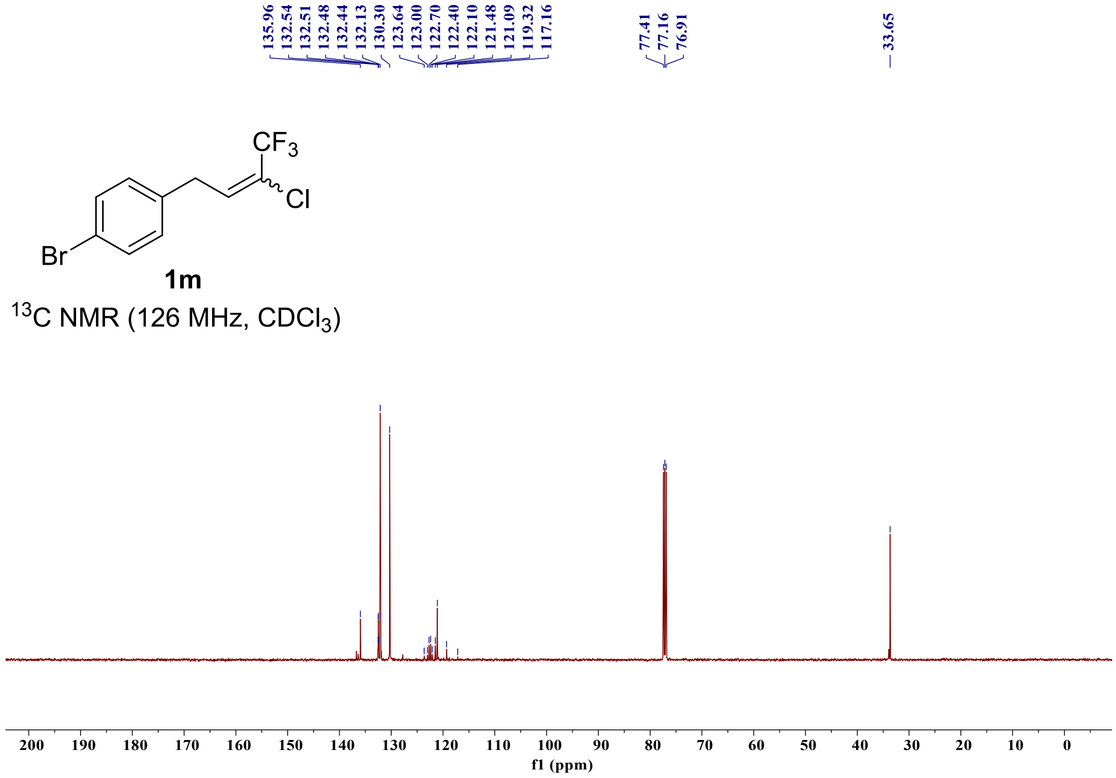

(5-chloro-6,6,6-trifluorohex-4-en-1-yl)benzene (1n)

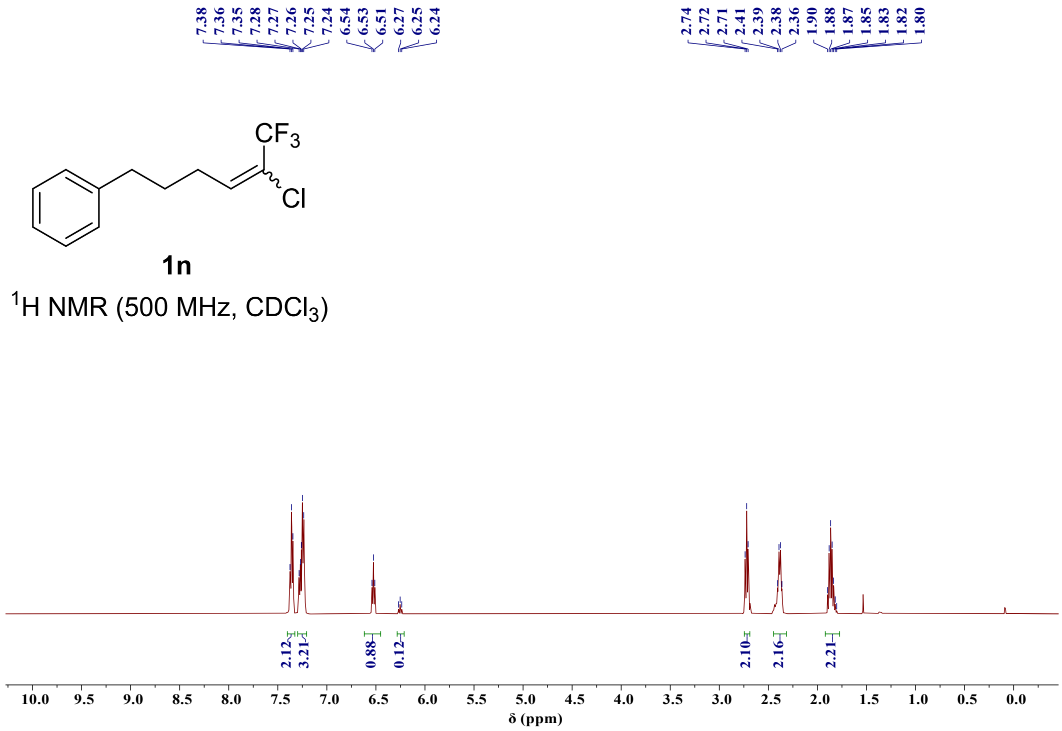

(5-chloro-6,6,6-trifluorohex-4-en-1-yl)benzene (1n)

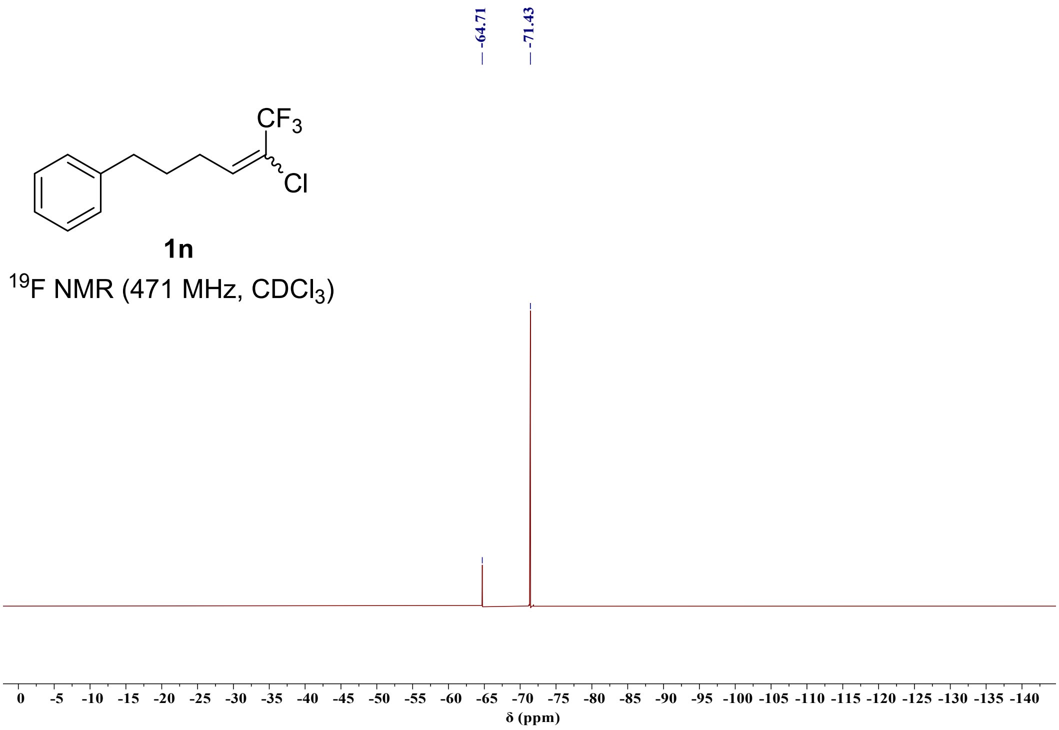

(5-chloro-6,6,6-trifluorohex-4-en-1-yl)benzene (1n)

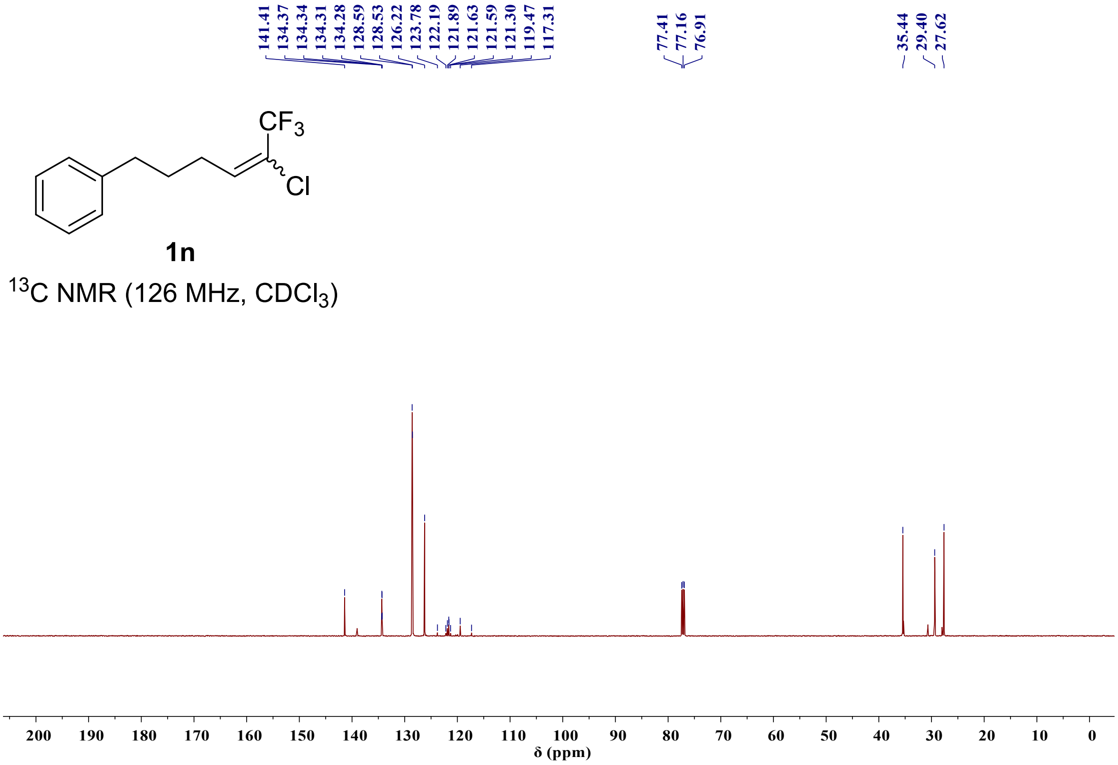

(4-chloro-5,5,5-trifluoropent-3-ene-1,1-diyl)dibenzene (1o)

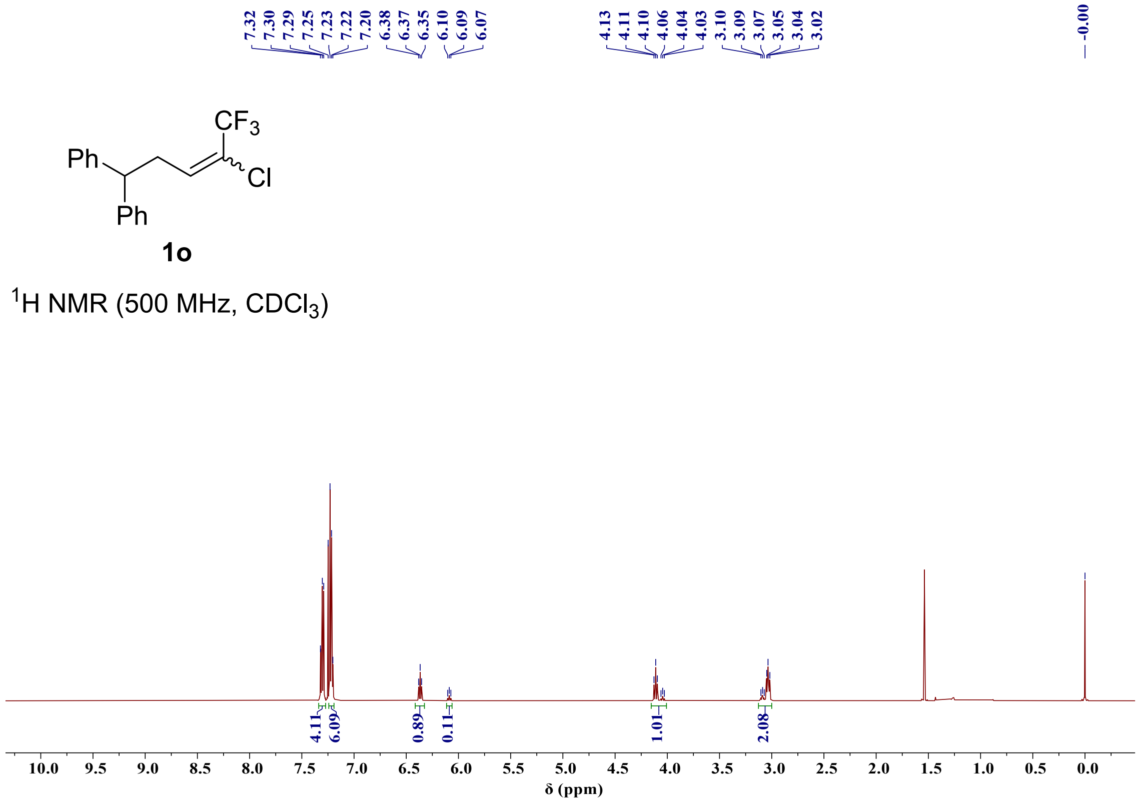

(4-chloro-5,5,5-trifluoropent-3-ene-1,1-diyl)dibenzene (1o)

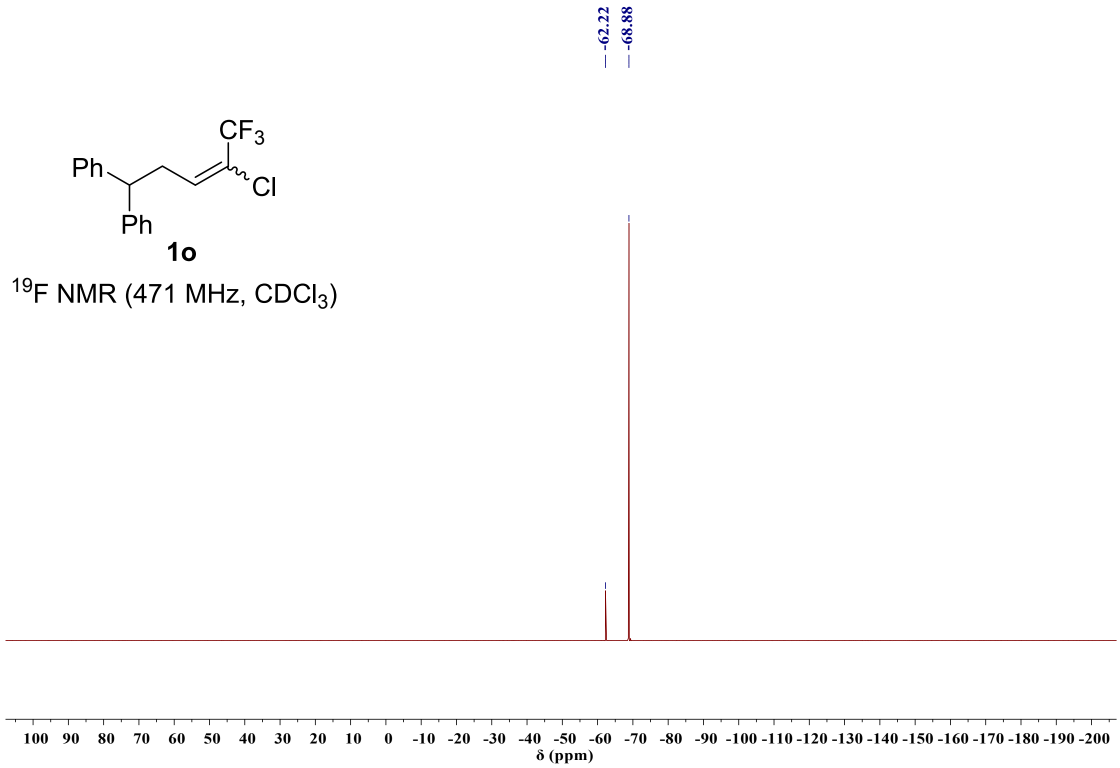

(4-chloro-5,5,5-trifluoropent-3-ene-1,1-diyl)dibenzene (1o)

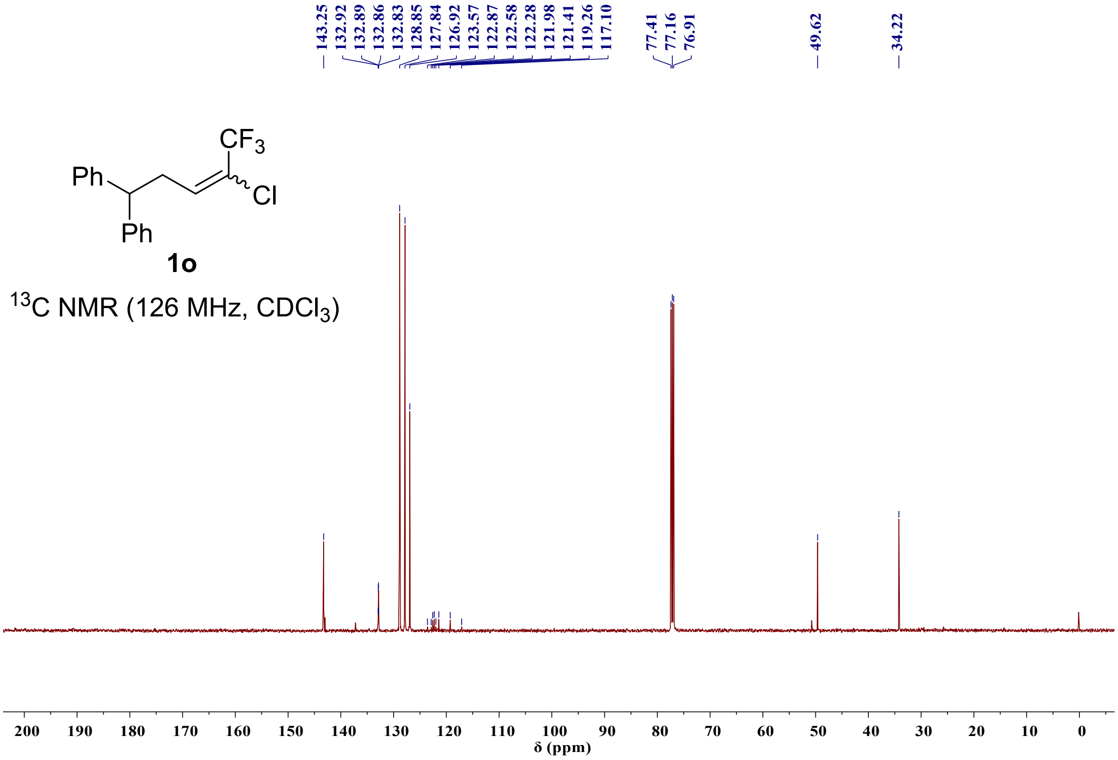

1-(3-chloro-4,4,4-trifluorobut-2-en-1-yl)naphthalene (1p)

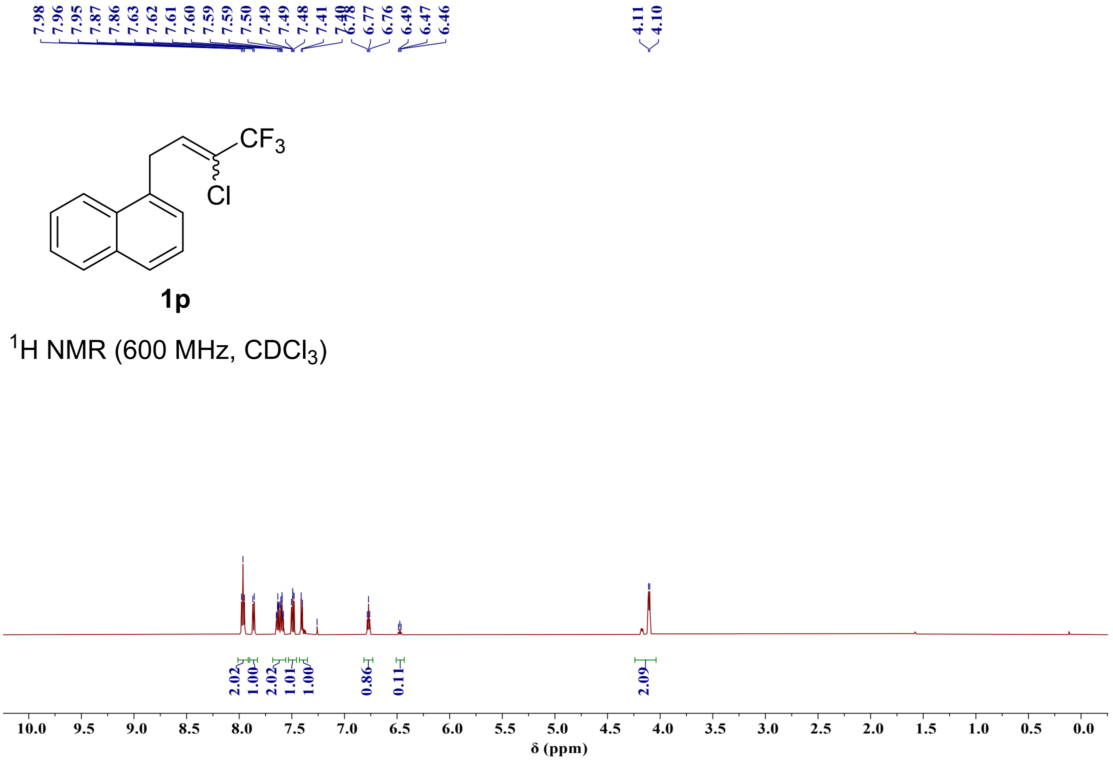

1-(3-chloro-4,4,4-trifluorobut-2-en-1-yl)naphthalene (1p)

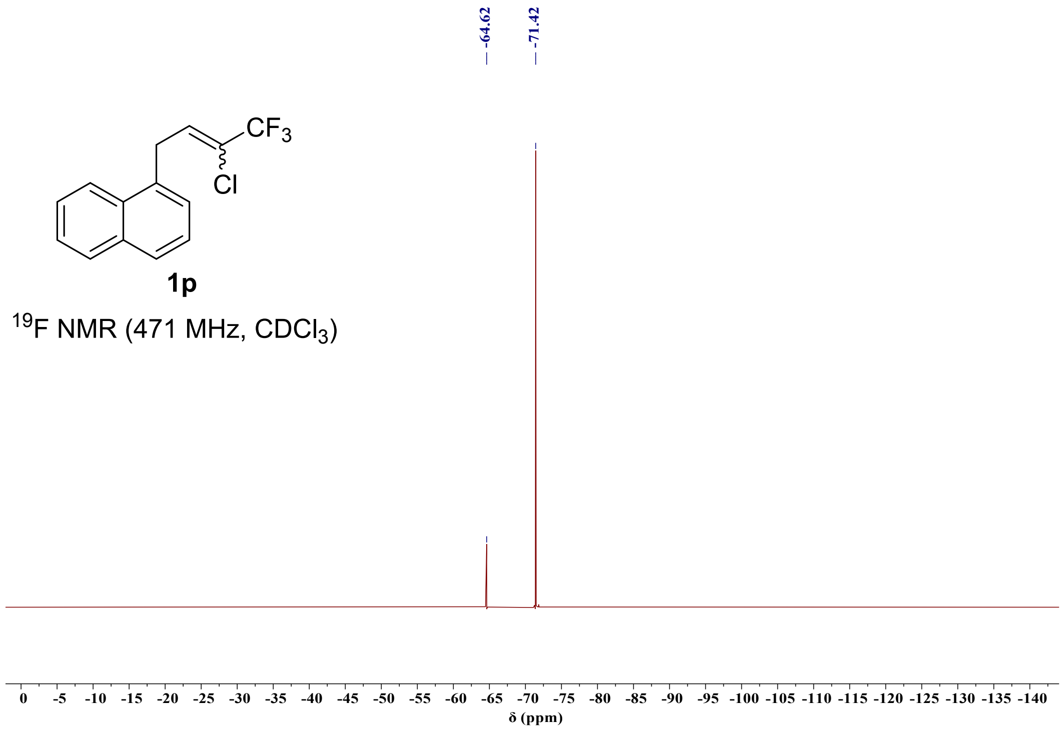

1-(3-chloro-4,4,4-trifluorobut-2-en-1-yl)naphthalene (1p)

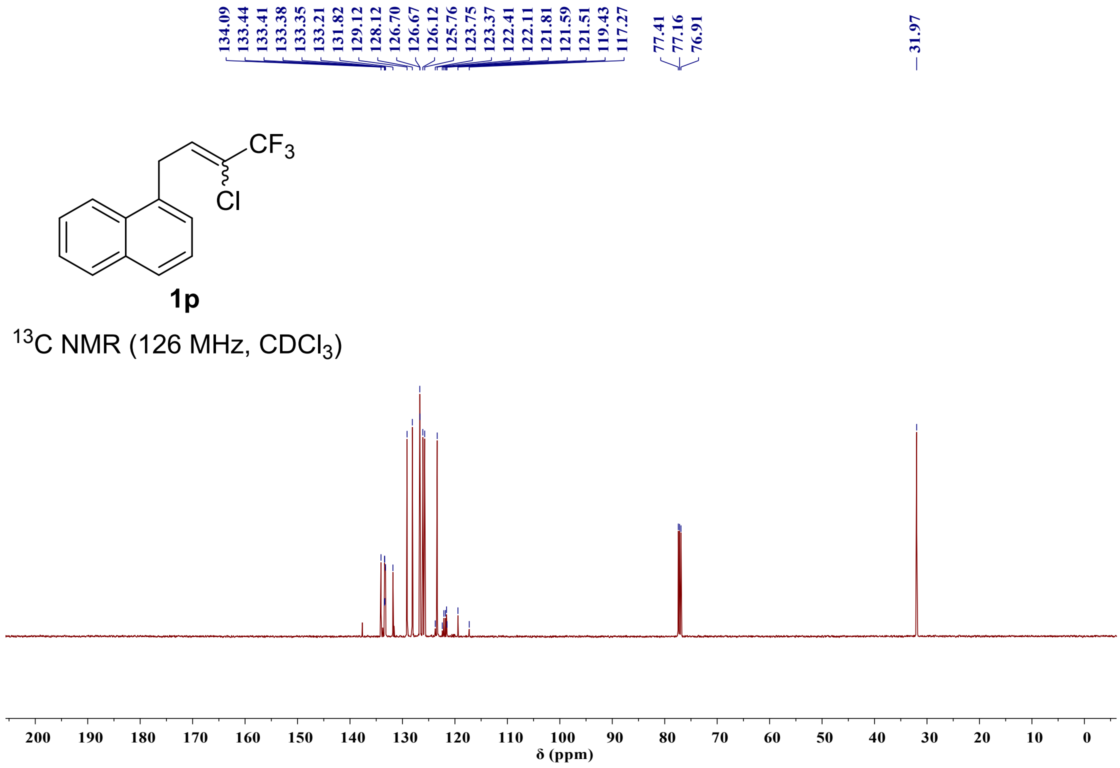

4-(3-chloro-4,4,4-trifluorobut-2-en-1-yl)-1,1'-biphenyl (1q)

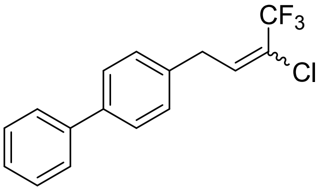

1q

^{1}H NMR (500 MHz, CDCl$_3$)

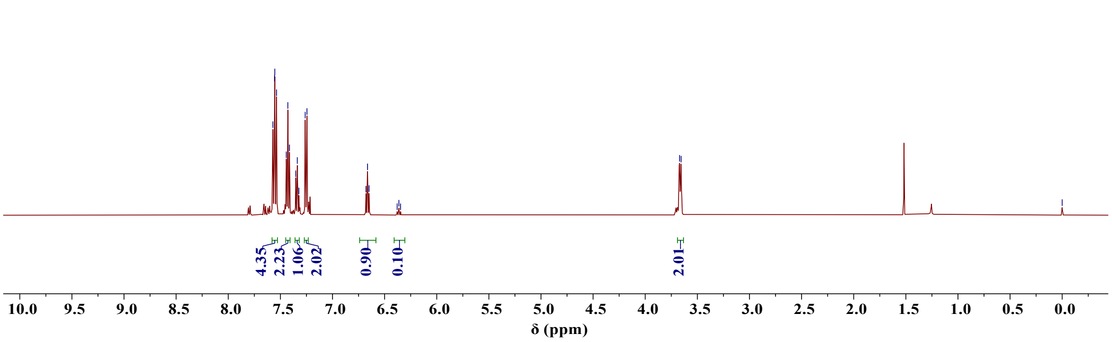

4-(3-chloro-4,4,4-trifluorobut-2-en-1-yl)-1,1'-biphenyl (1q)

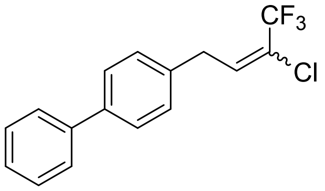

1q

^{19}F NMR (471 MHz, CDCl$_3$)

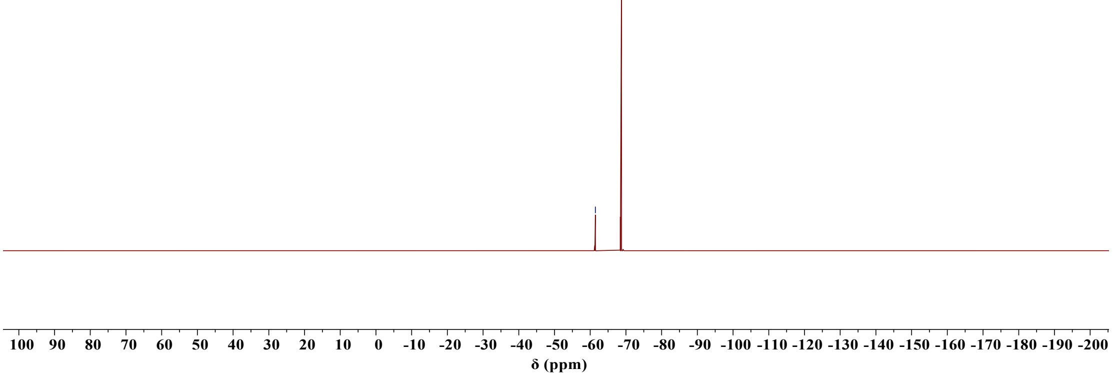

4-(3-chloro-4,4,4-trifluorobut-2-en-1-yl)-1,1'-biphenyl (1q)

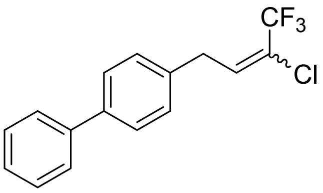

1q

^{13}C NMR (126 MHz, $CDCl_3$)

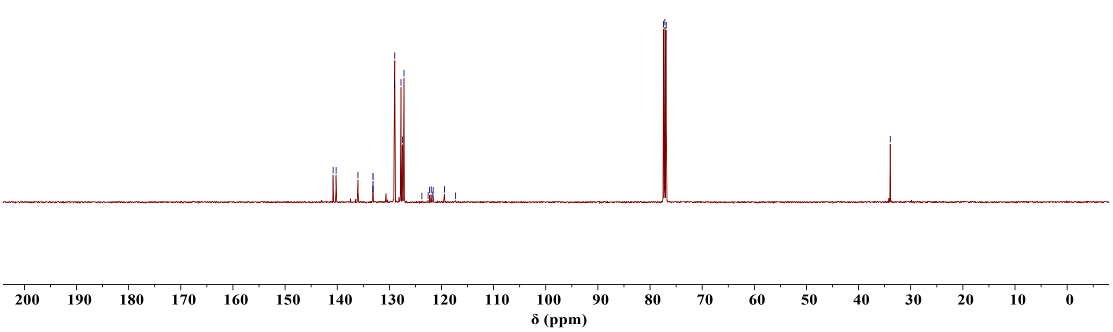

5-(3-chloro-4,4,4-trifluorobut-2-en-1-yl)benzo[d][1,3]dioxole (1r)

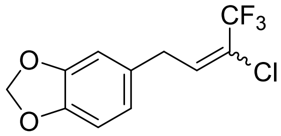

1r

^{1}H NMR (500 MHz, $CDCl_3$)

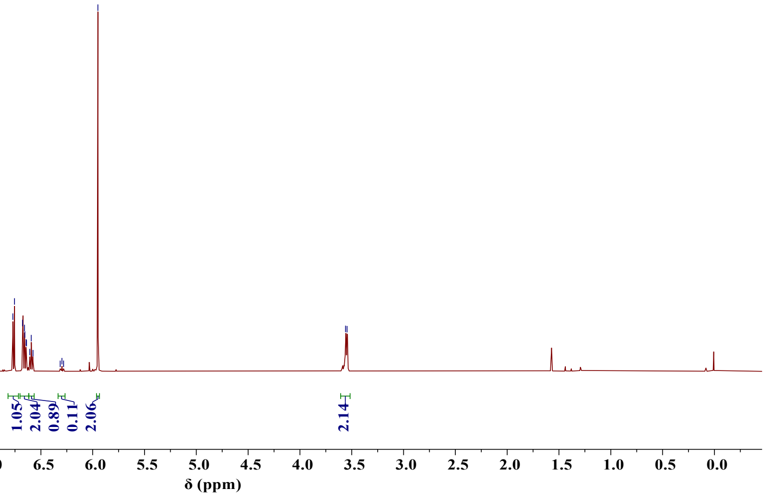

5-(3-chloro-4,4,4-trifluorobut-2-en-1-yl)benzo[d][1,3]dioxole (1r)

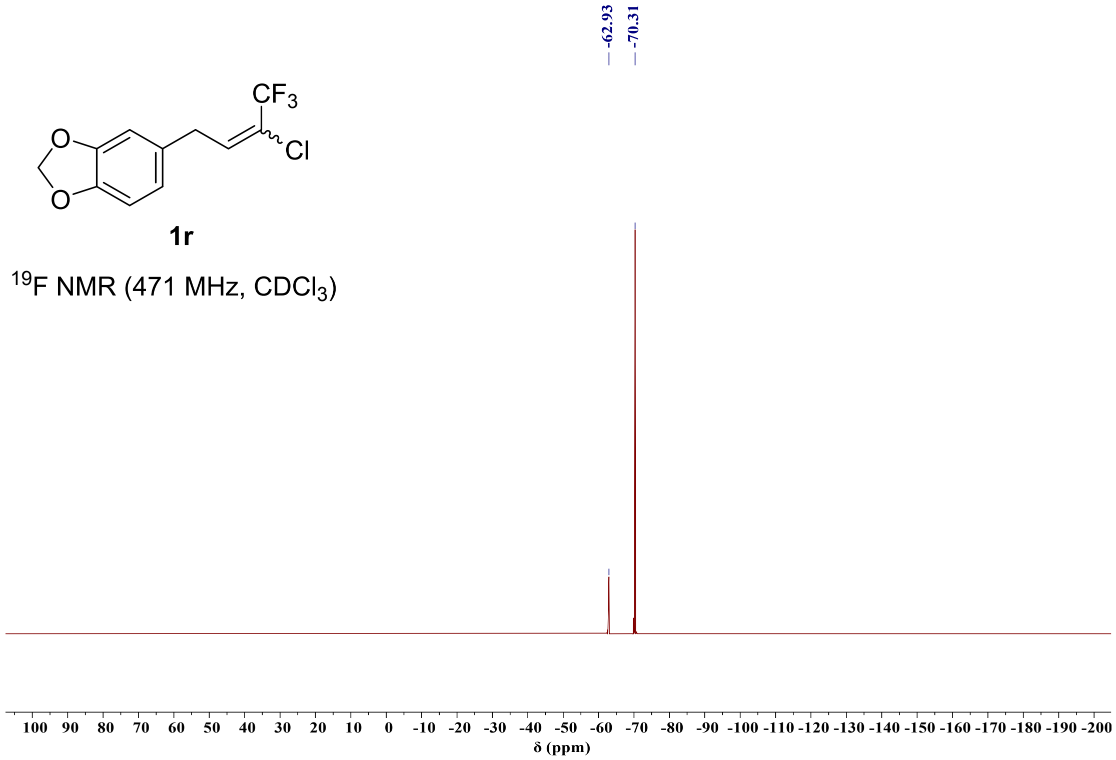

5-(3-chloro-4,4,4-trifluorobut-2-en-1-yl)benzo[d][1,3]dioxole (1r)

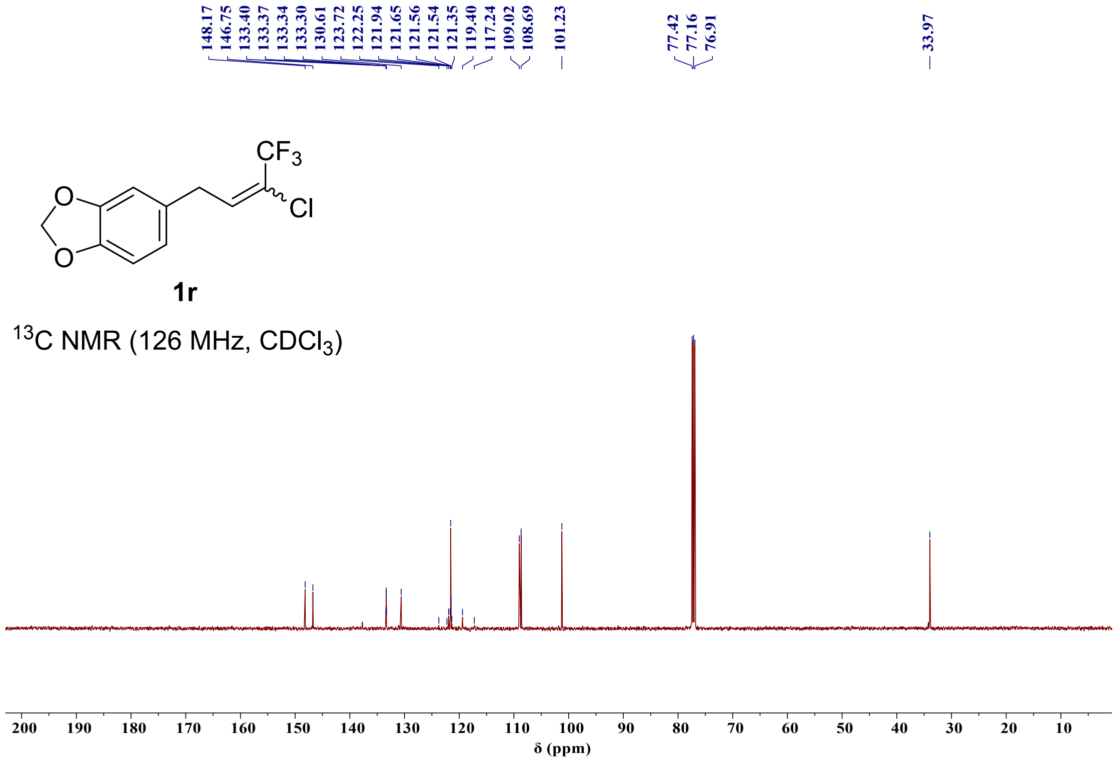

((4-chloro-5,5,5-trifluoropent-3-en-1-yl)oxy)benzene (1s)

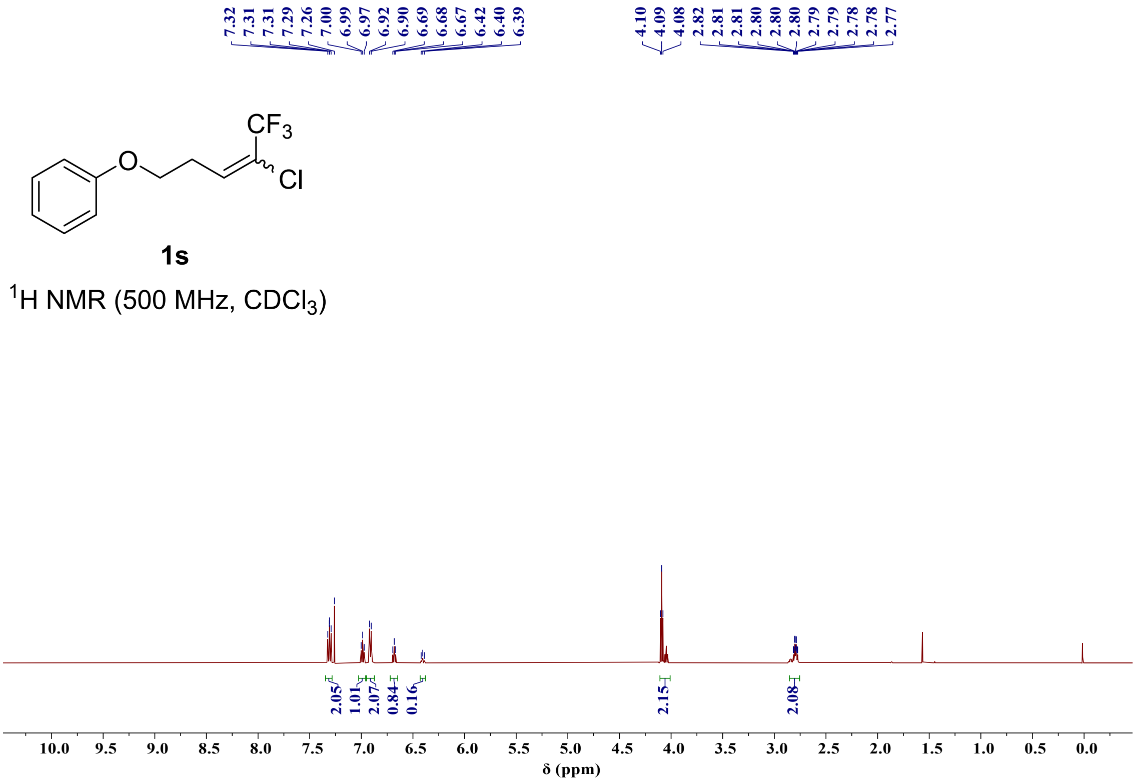

((4-chloro-5,5,5-trifluoropent-3-en-1-yl)oxy)benzene (1s)

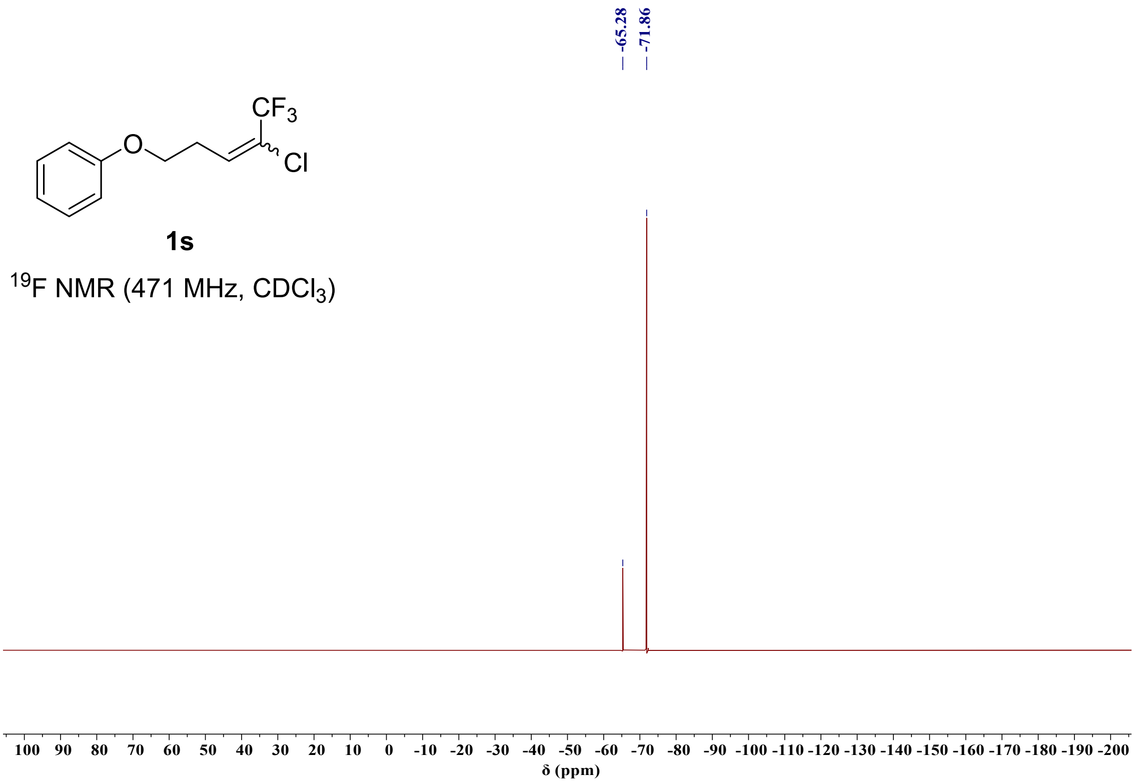

((4-chloro-5,5,5-trifluoropent-3-en-1-yl)oxy)benzene (1s)

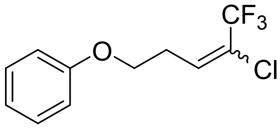

1s

^{13}C NMR (126 MHz, $CDCl_3$)

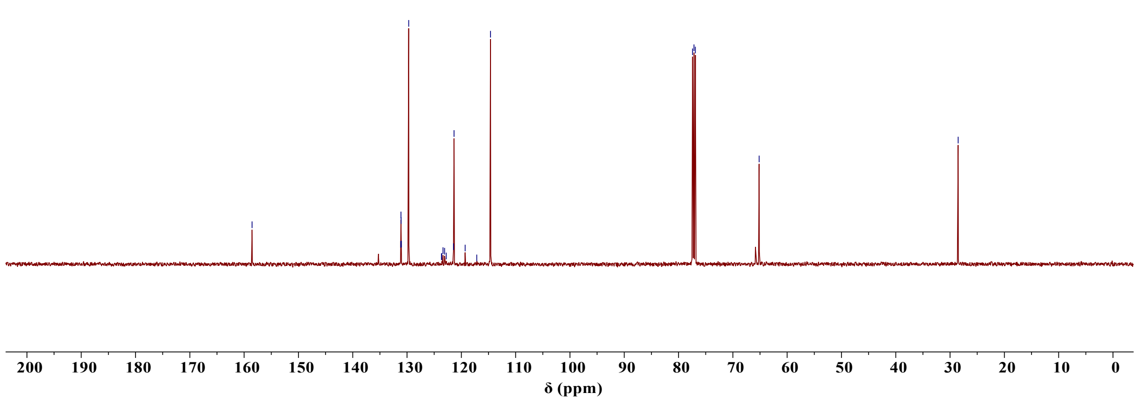

4-(4-chloro-5,5,5-trifluoropent-3-en-1-yl)-1,2-dimethoxybenzene (1t)

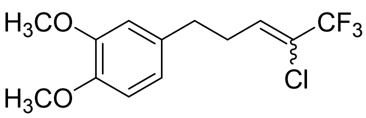

1t

^{1}H NMR (500 MHz, $CDCl_3$)

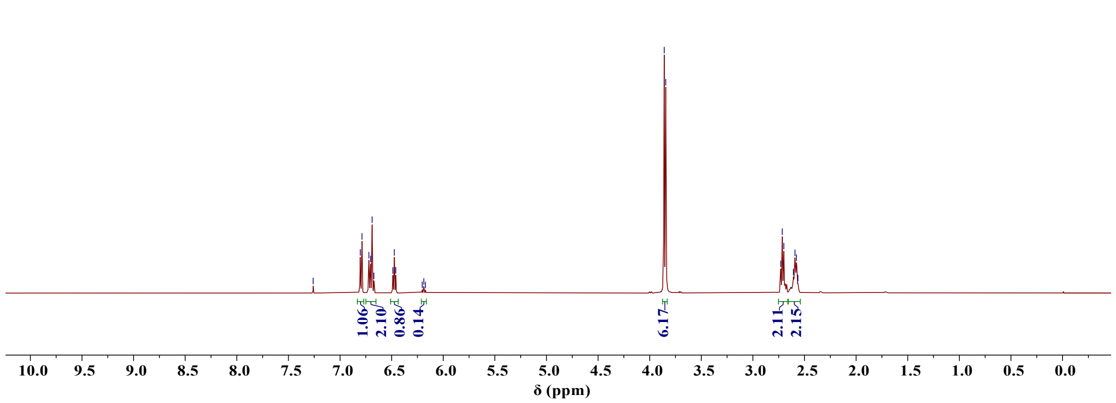

4-(4-chloro-5,5,5-trifluoropent-3-en-1-yl)-1,2-dimethoxybenzene (1t)

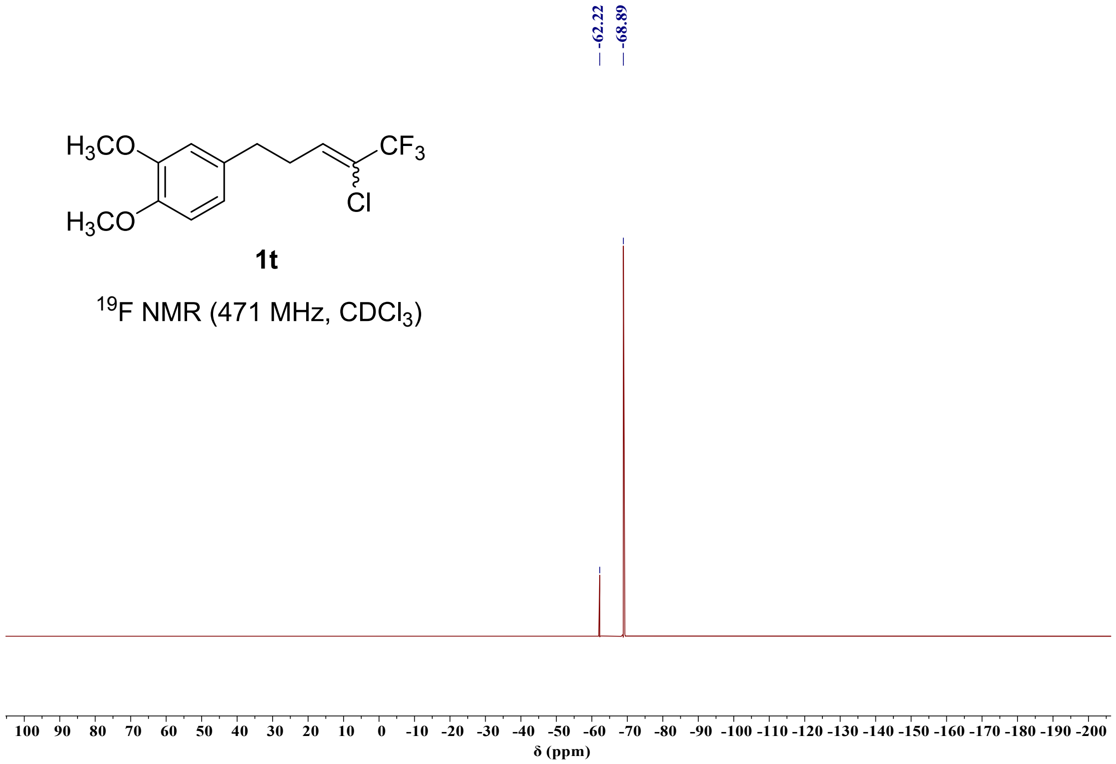

4-(4-chloro-5,5,5-trifluoropent-3-en-1-yl)-1,2-dimethoxybenzene (1t)

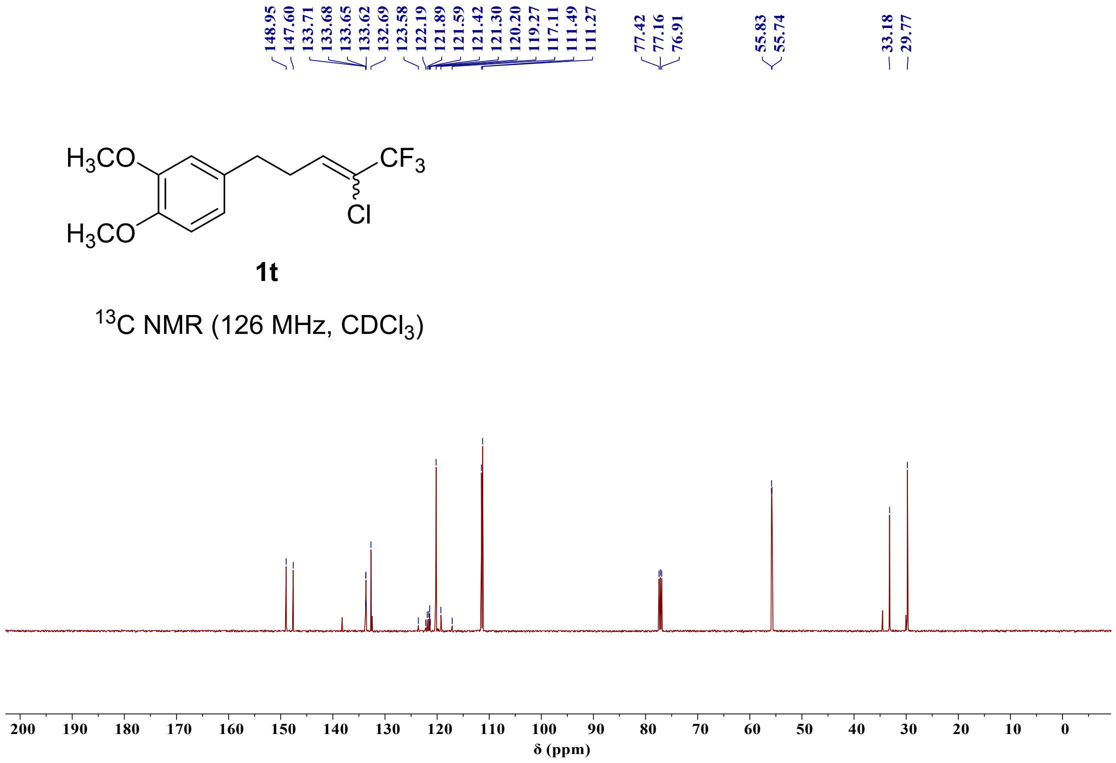

2-(4-chloro-5,5,5-trifluoropent-3-en-1-yl)-5-methylfuran (1u)

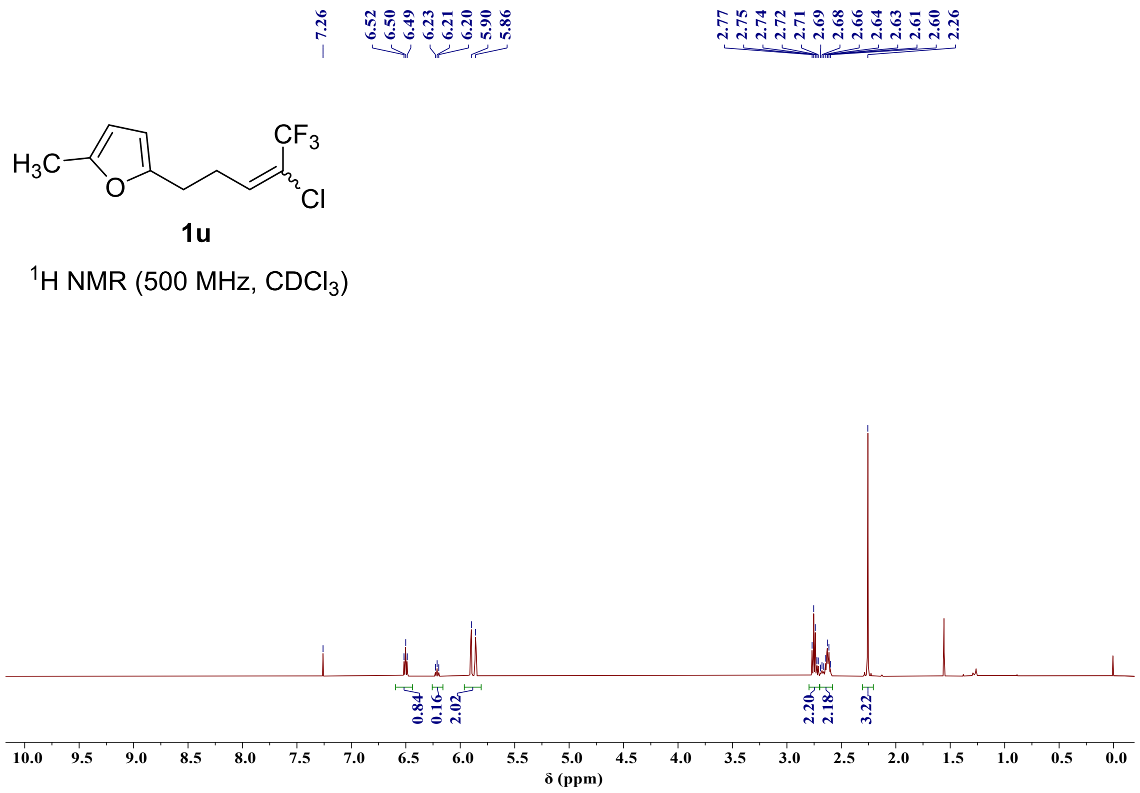

2-(4-chloro-5,5,5-trifluoropent-3-en-1-yl)-5-methylfuran (1u)

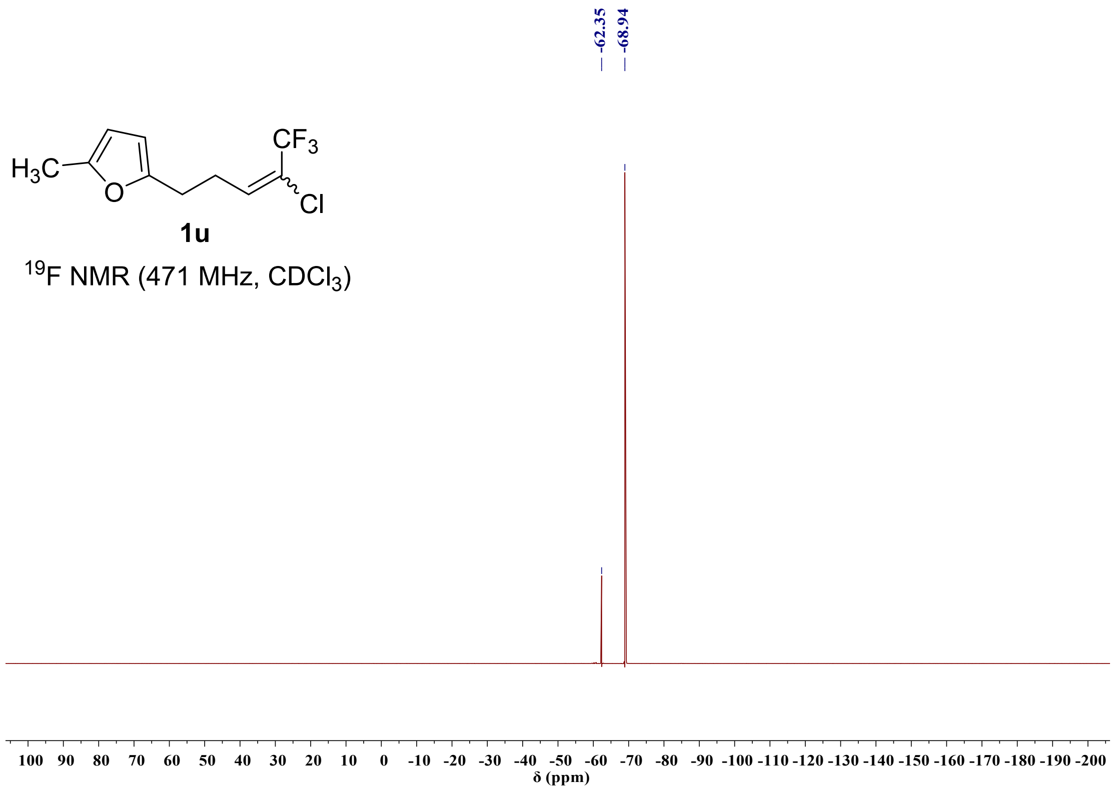

2-(4-chloro-5,5,5-trifluoropent-3-en-1-yl)-5-methylfuran (1u)

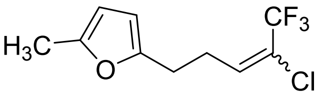

1u

^{13}C NMR (126 MHz, CDCl$_3$)

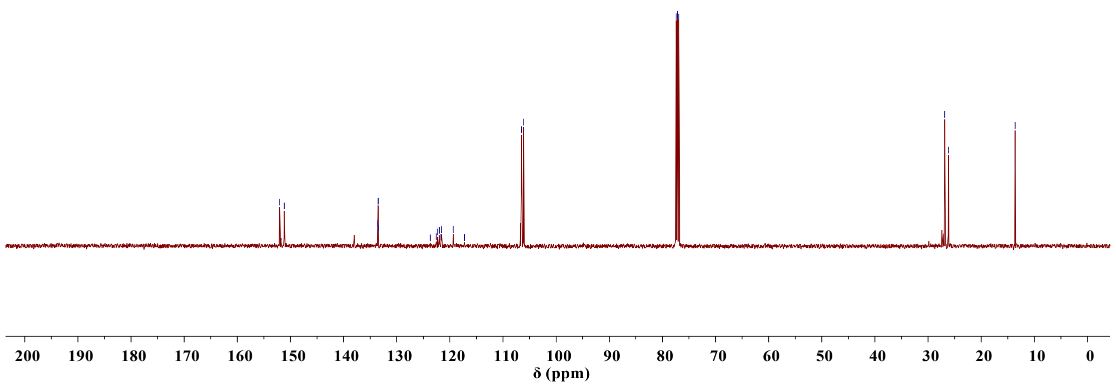

2-(5-chloro-6,6,6-trifluorohex-4-en-1-yl)thiophene (1v)

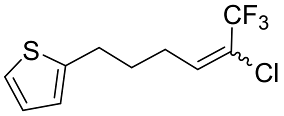

1v

^{1}H NMR (500 MHz, CDCl$_3$)

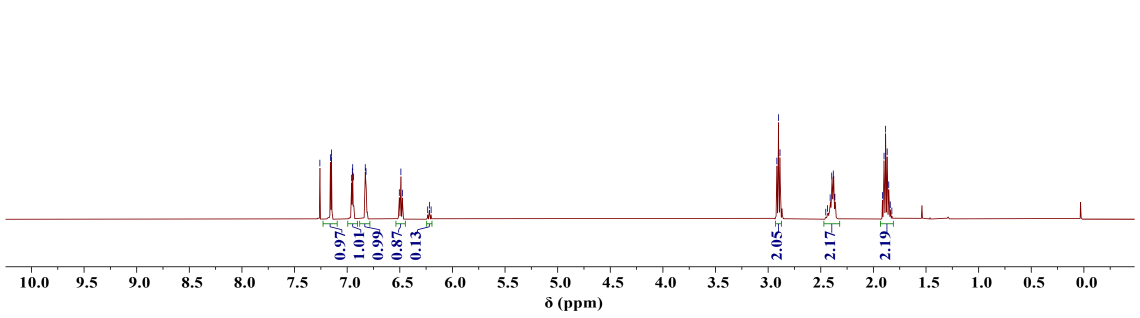

2-(5-chloro-6,6,6-trifluorohex-4-en-1-yl)thiophene (1v)

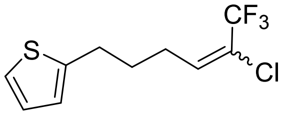

1v

^{19}F NMR (471 MHz, $CDCl_3$)

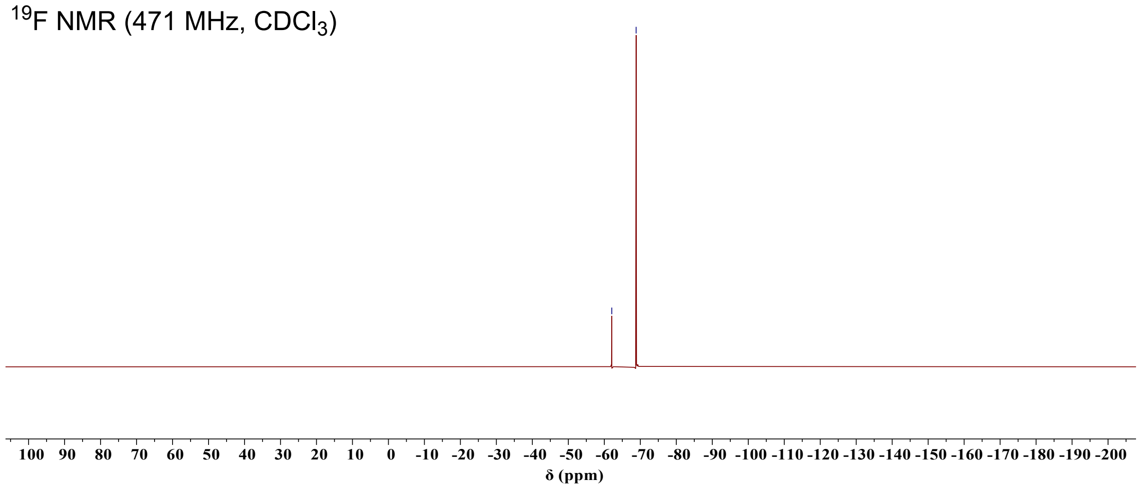

2-(5-chloro-6,6,6-trifluorohex-4-en-1-yl)thiophene (1v)

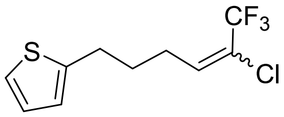

1v

^{13}C NMR (126 MHz, $CDCl_3$)

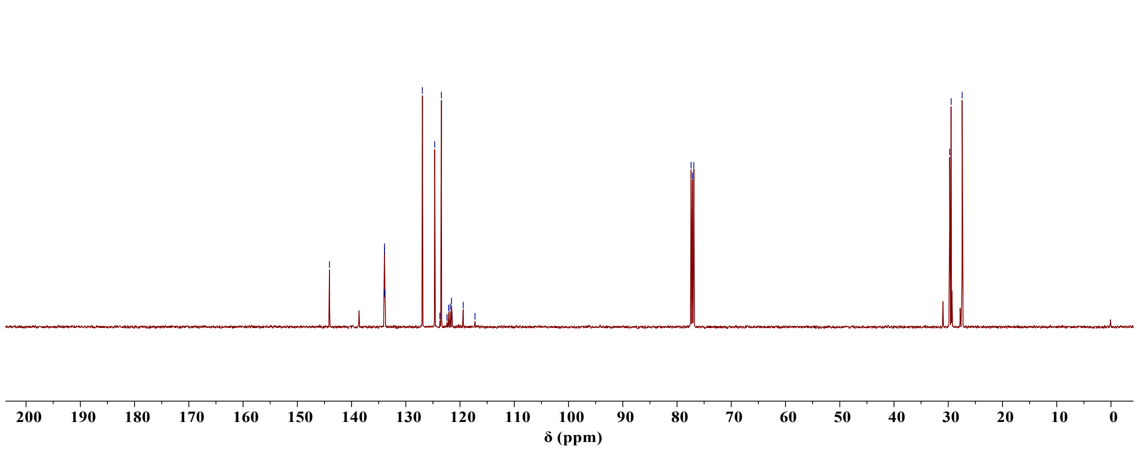

tert-butyl 4-(2-chloro-3,3,3-trifluoroprop-1-en-1-yl)piperidine-1-carboxylate (1w)

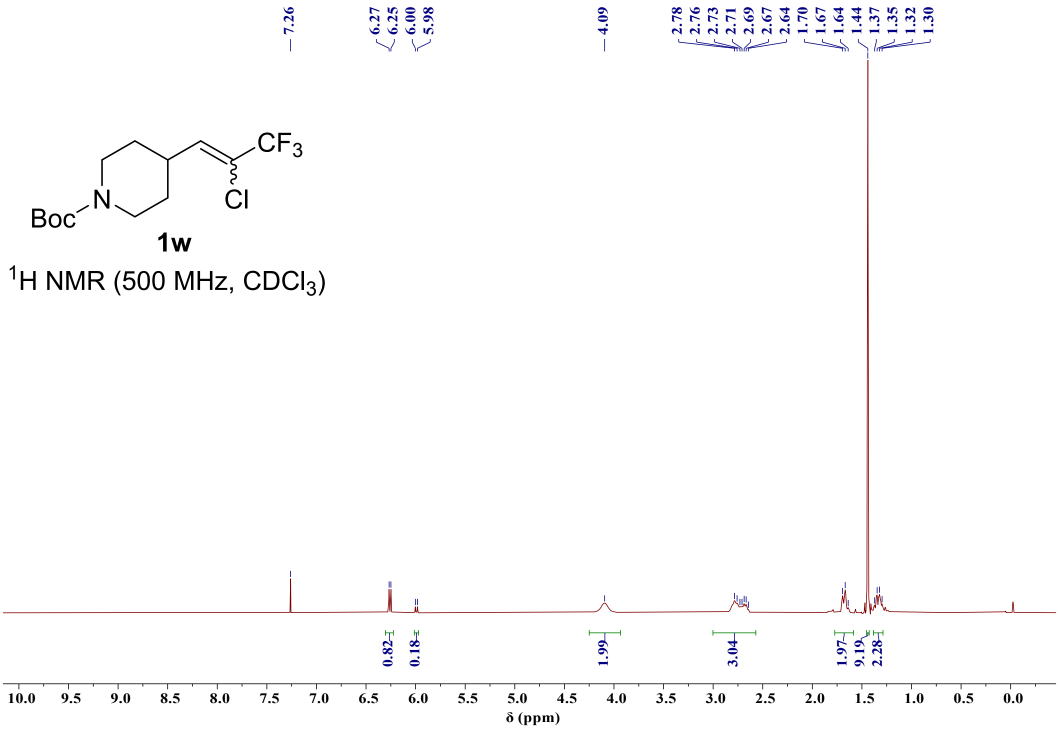

tert-butyl 4-(2-chloro-3,3,3-trifluoroprop-1-en-1-yl)piperidine-1-carboxylate (1w)

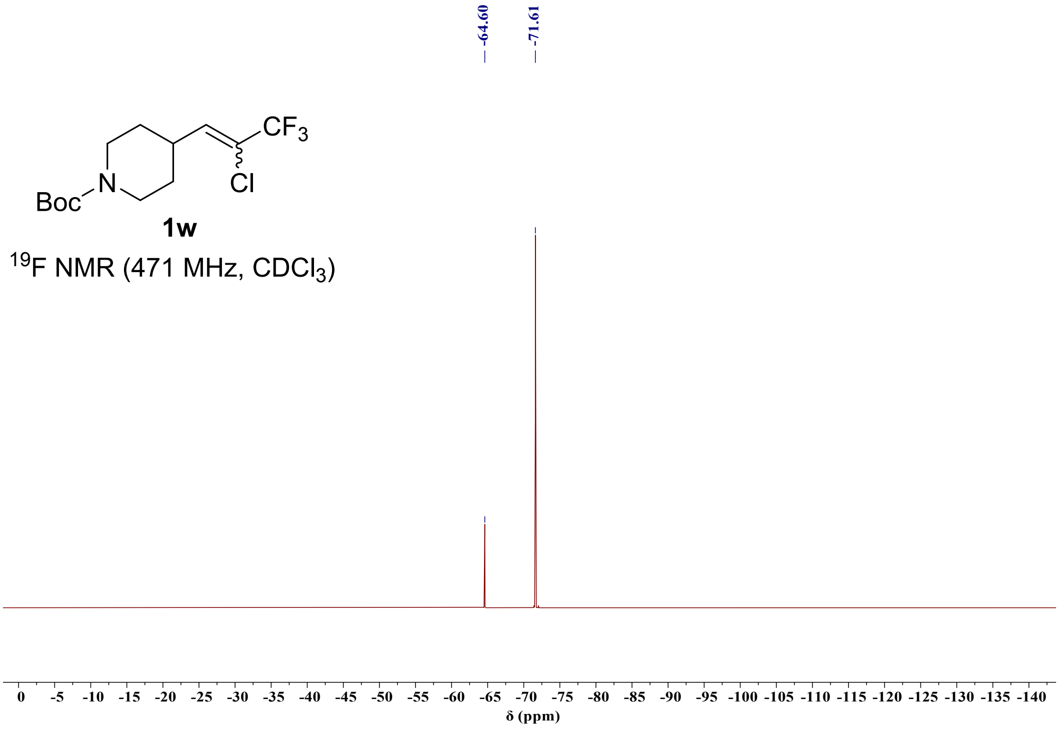

tert-butyl 4-(2-chloro-3,3,3-trifluoroprop-1-en-1-yl)piperidine-1-carboxylate (1w)

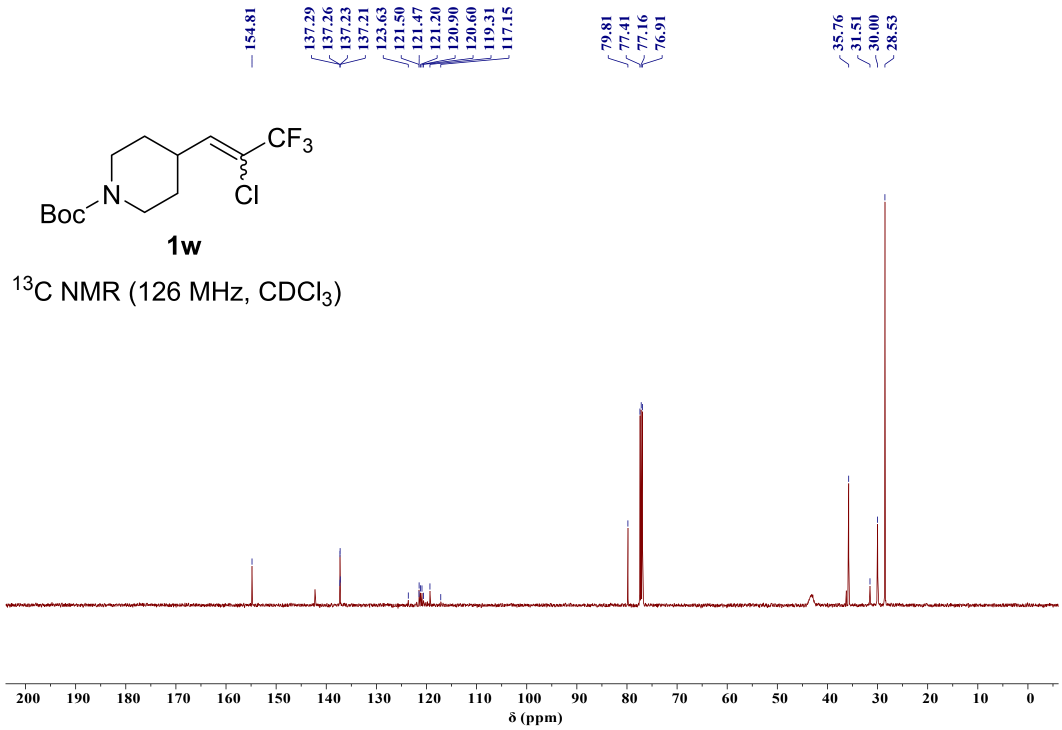

benzyl 4-(2-chloro-3,3,3-trifluoroprop-1-en-1-yl)piperidine-1-carboxylate (1x)

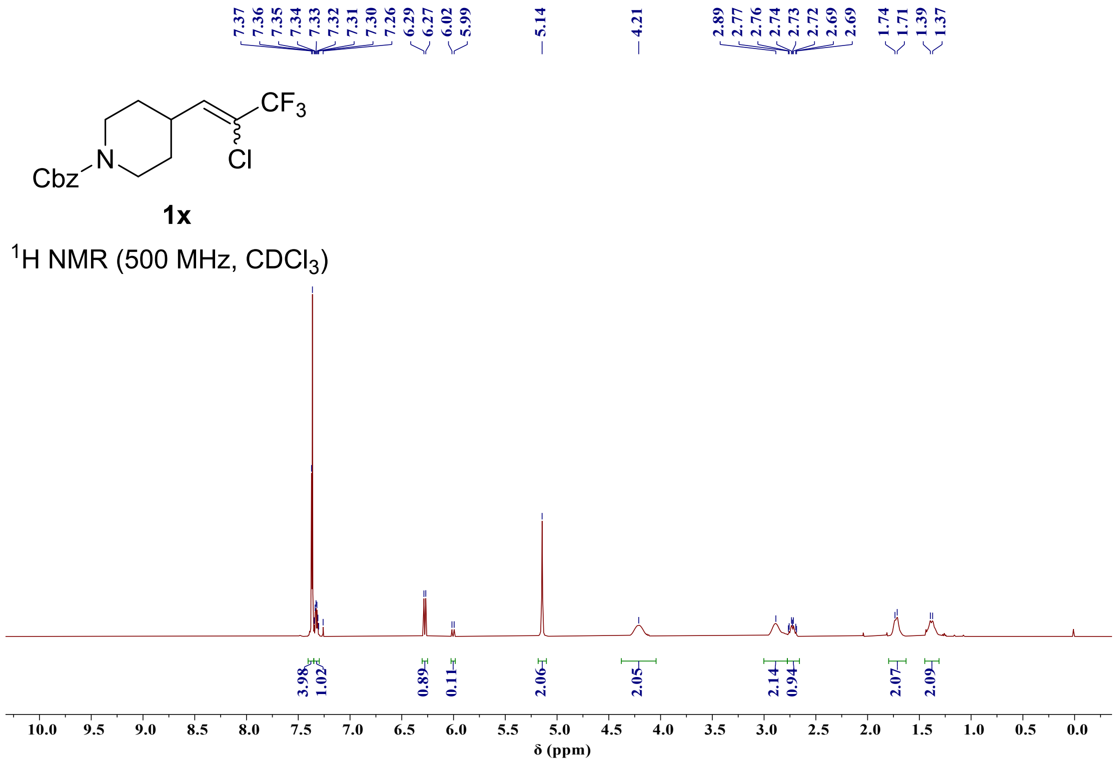

benzyl 4-(2-chloro-3,3,3-trifluoroprop-1-en-1-yl)piperidine-1-carboxylate (1x)

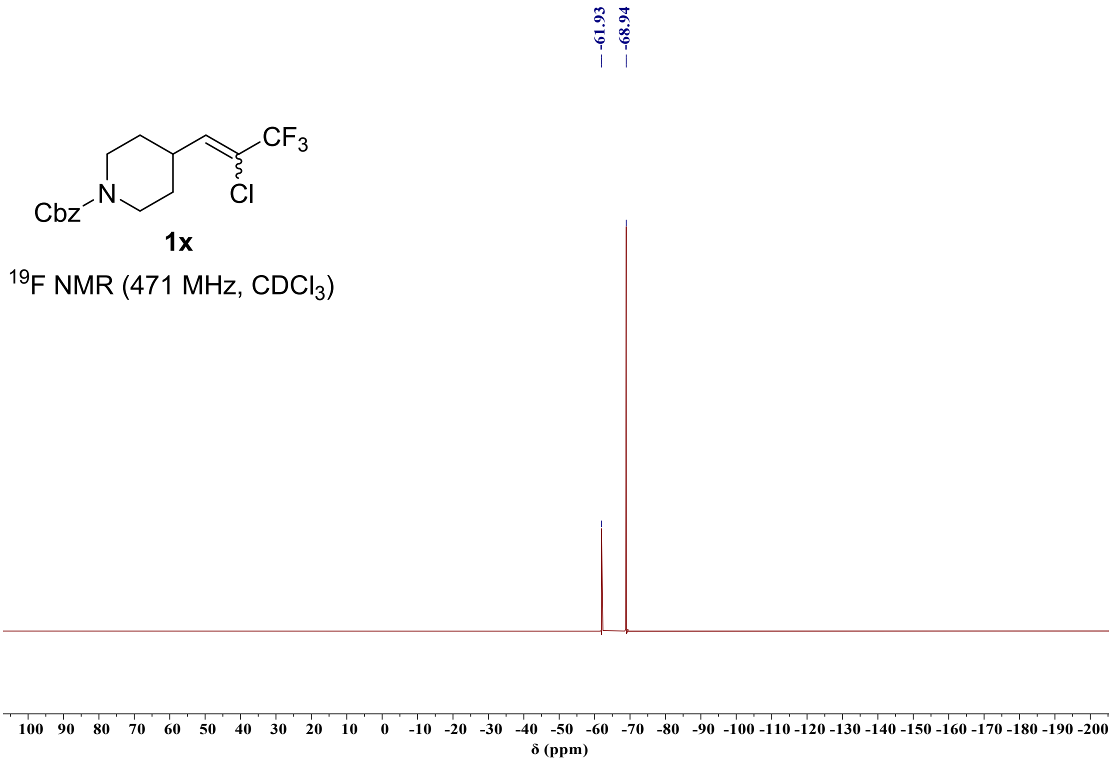

benzyl 4-(2-chloro-3,3,3-trifluoroprop-1-en-1-yl)piperidine-1-carboxylate (1x)

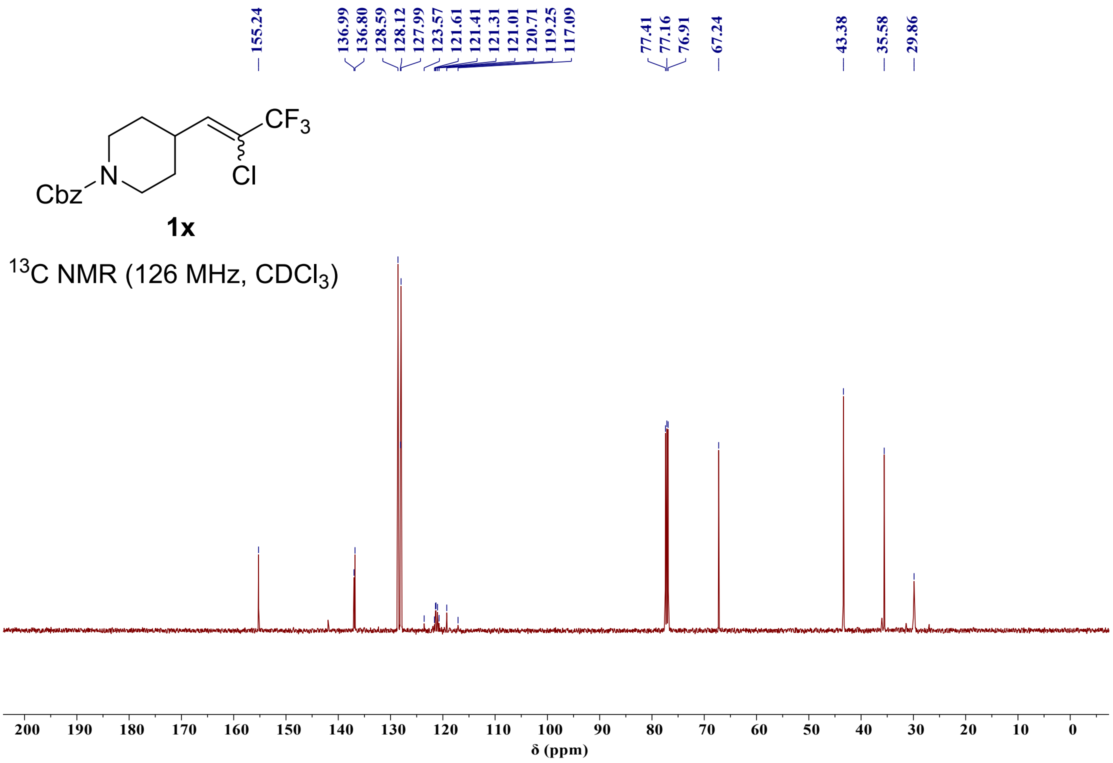

4-(3-chloro-4,4,4-trifluorobut-2-en-1-yl)-1-tosylpiperidine (1y)

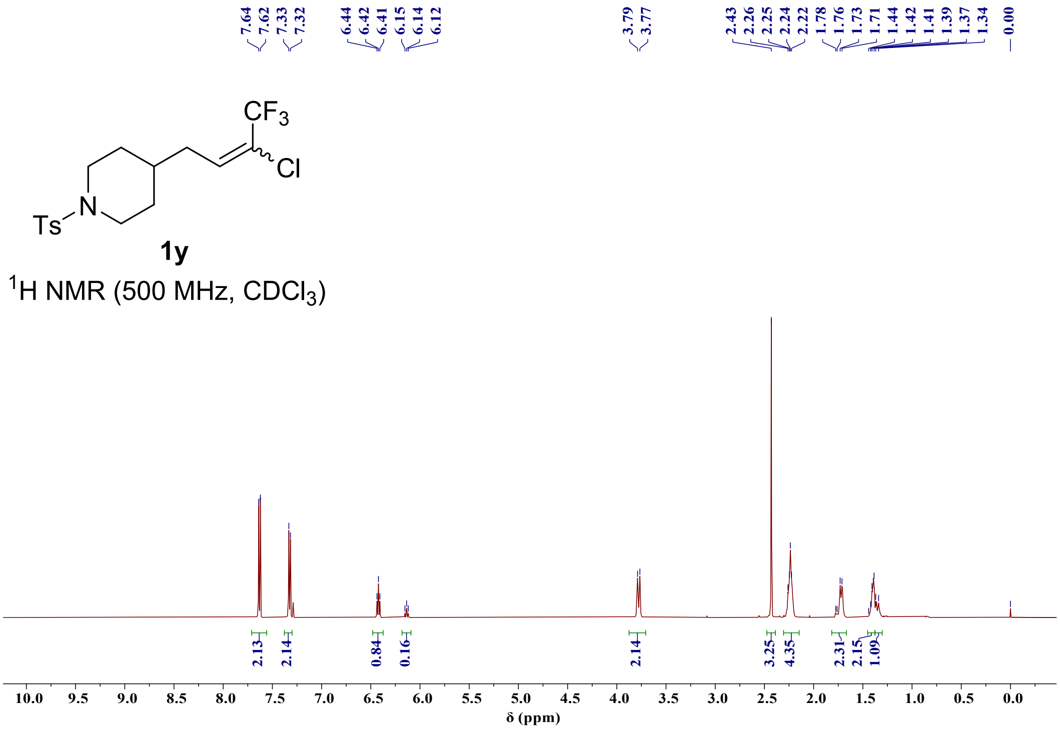

4-(3-chloro-4,4,4-trifluorobut-2-en-1-yl)-1-tosylpiperidine (1y)

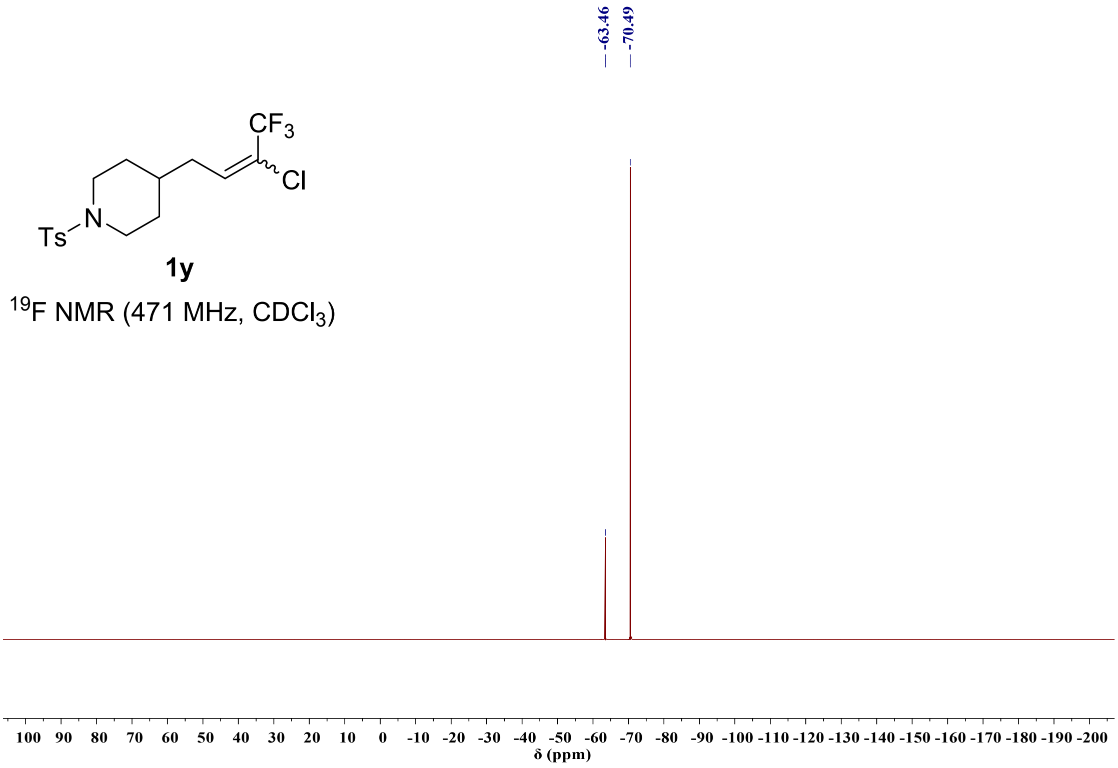

4-(3-chloro-4,4,4-trifluorobut-2-en-1-yl)-1-tosylpiperidine (1y)

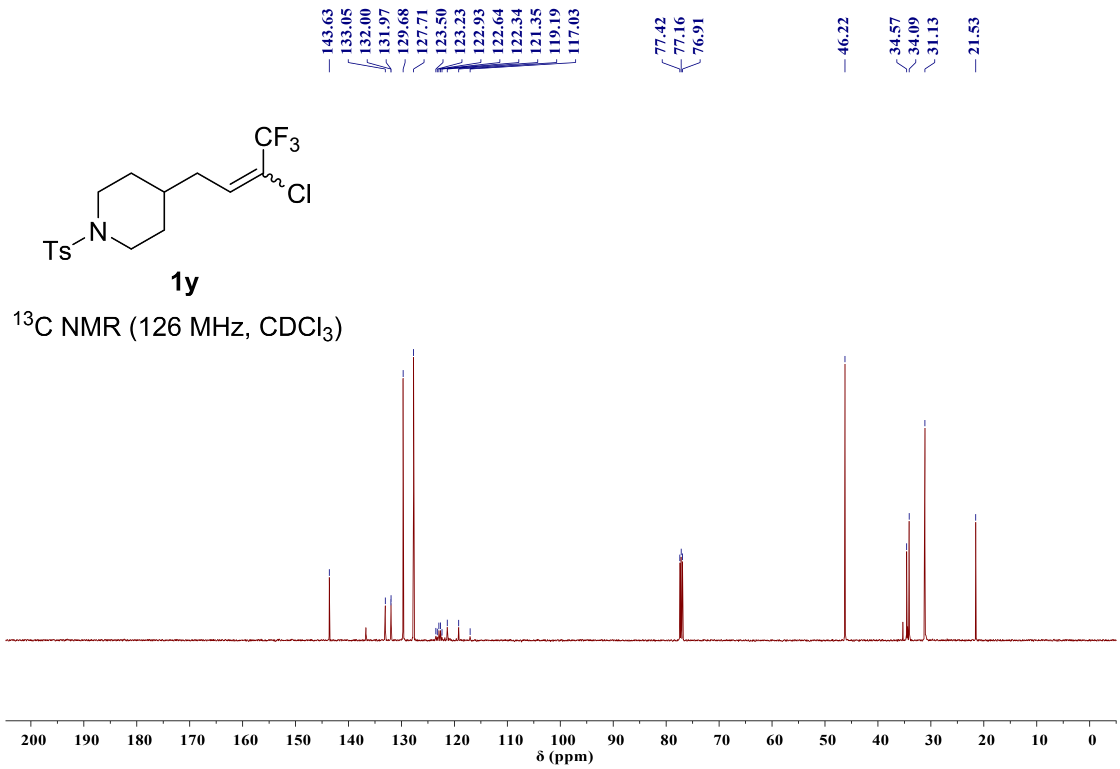

4-(3-chloro-4,4,4-trifluorobut-2-en-1-yl)tetrahydro-2H-pyran (1z)

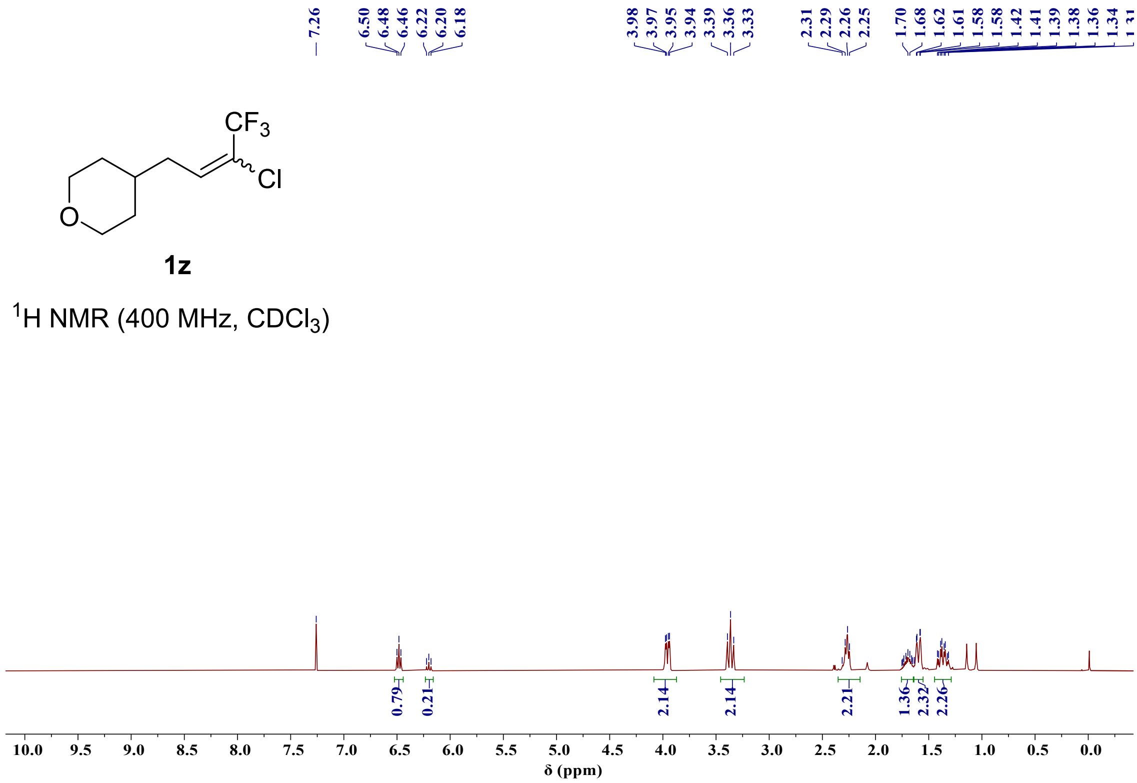

4-(3-chloro-4,4,4-trifluorobut-2-en-1-yl)tetrahydro-2H-pyran (1z)

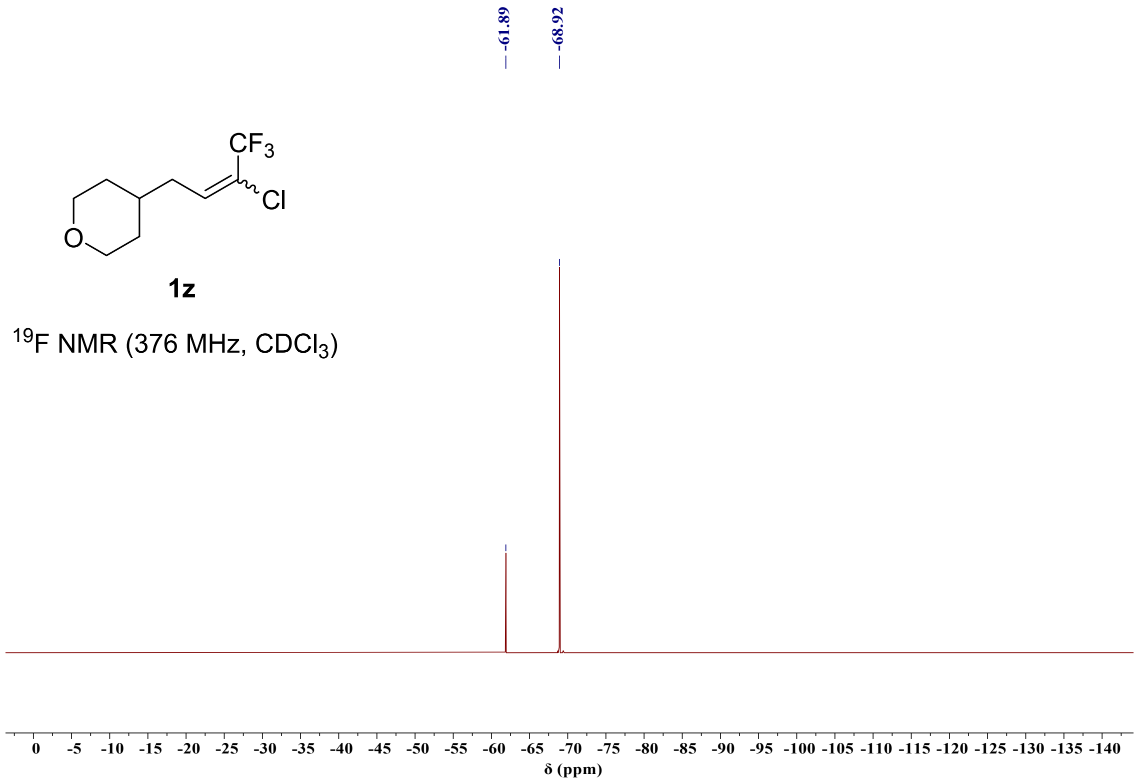

4-(3-chloro-4,4,4-trifluorobut-2-en-1-yl)tetrahydro-2H-pyran (1z)

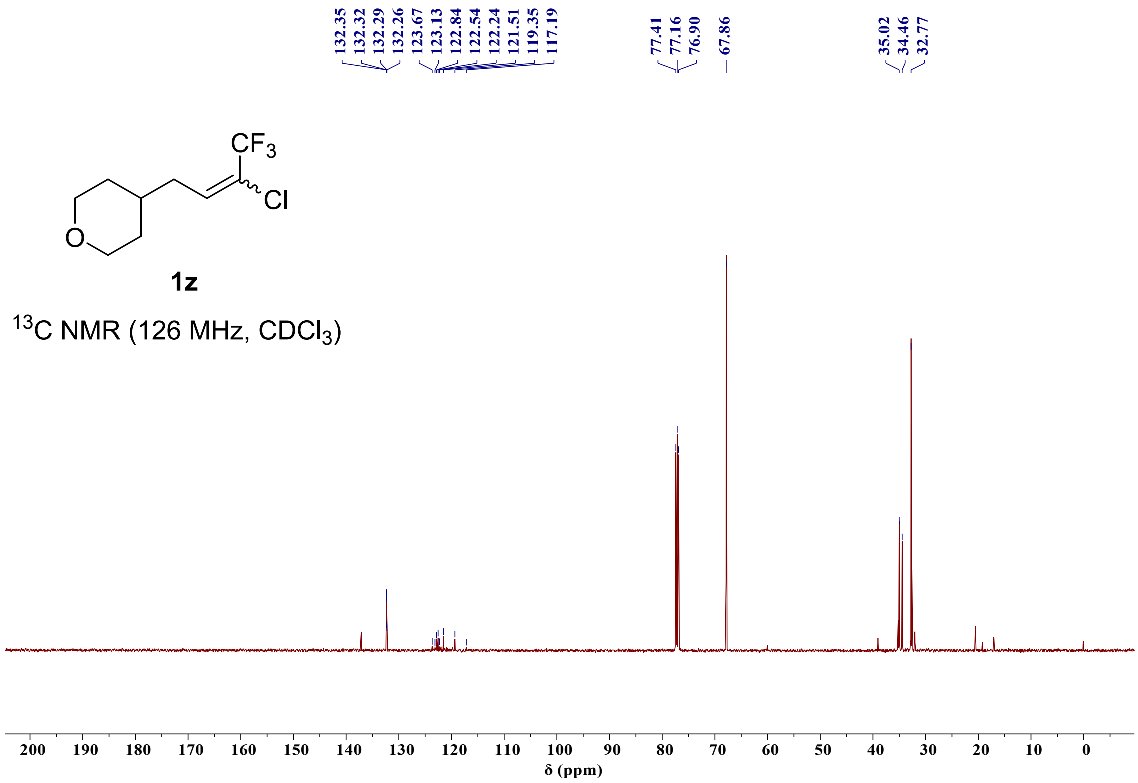

2-chloro-1,1,1-trifluorododec-2-ene (1aa)

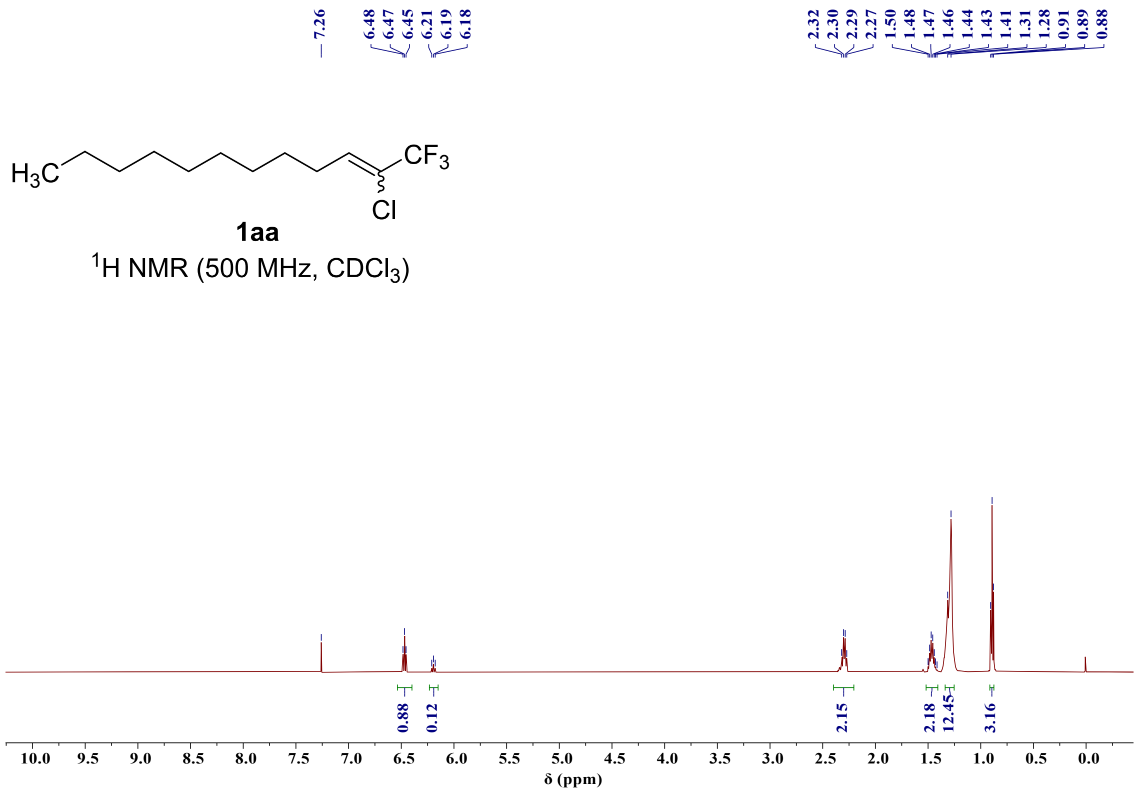

2-chloro-1,1,1-trifluorododec-2-ene (1aa)

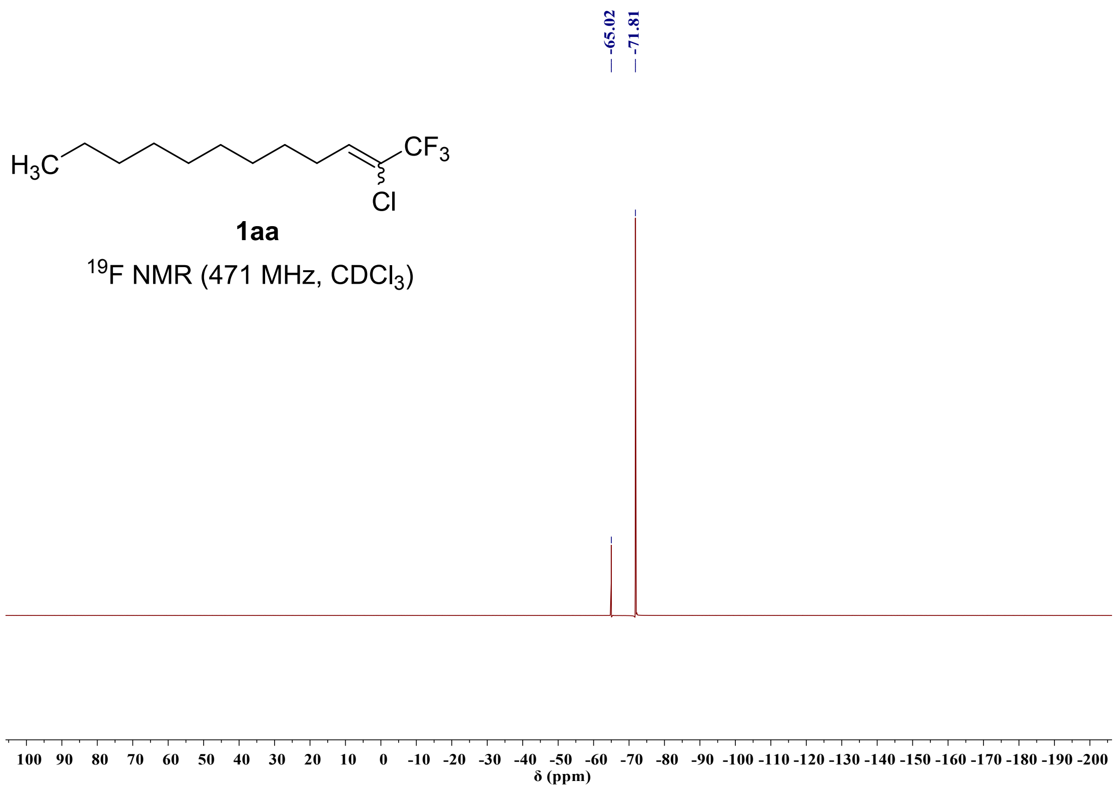

2-chloro-1,1,1-trifluorododec-2-ene (1aa)

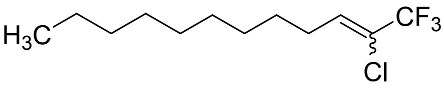

1aa

^{13}C NMR (126 MHz, $CDCl_3$)

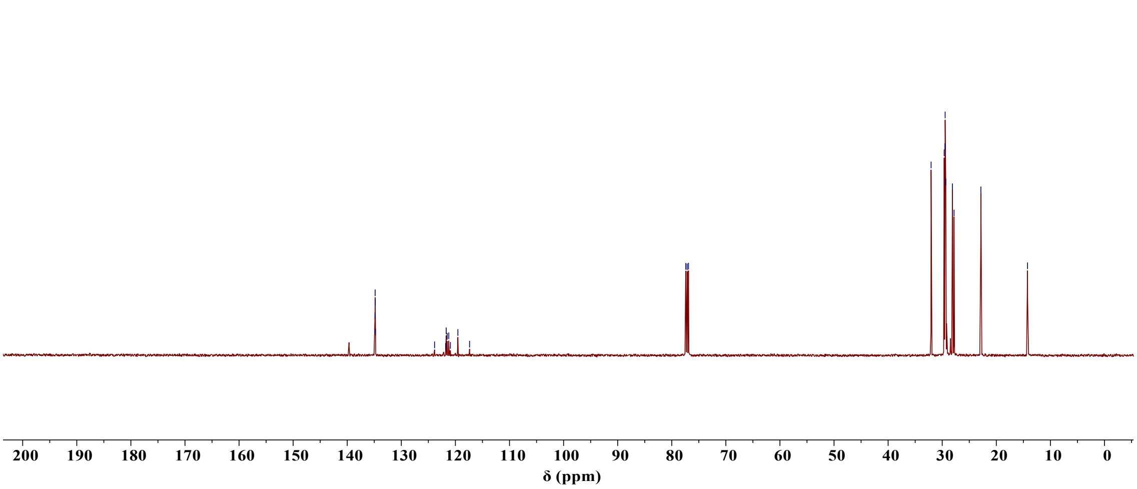

ethyl 7-chloro-8,8,8-trifluorooct-6-enoate (1ab)

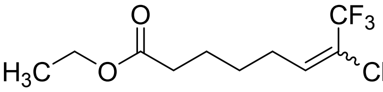

1ab

^{1}H NMR (500 MHz, $CDCl_3$)

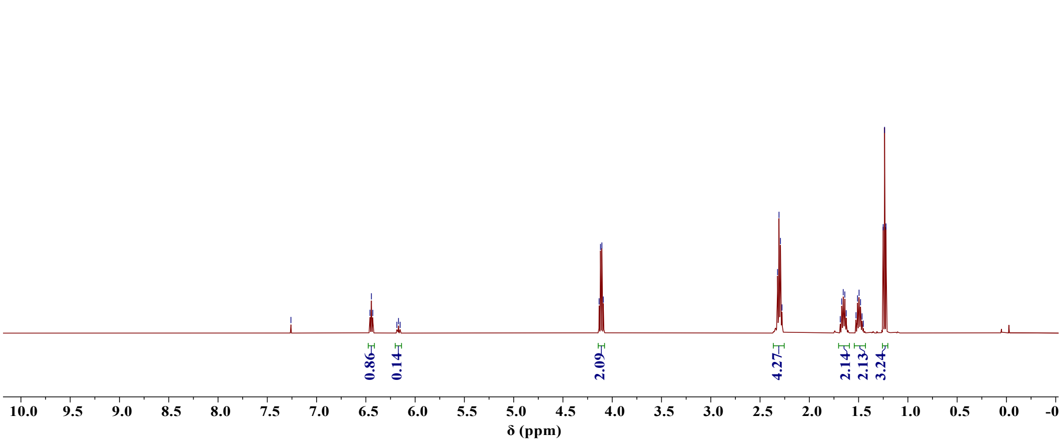

ethyl 7-chloro-8,8,8-trifluorooct-6-enoate (1ab)

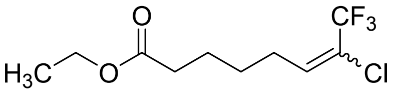

1ab

^{19}F NMR (471 MHz, $CDCl_3$)

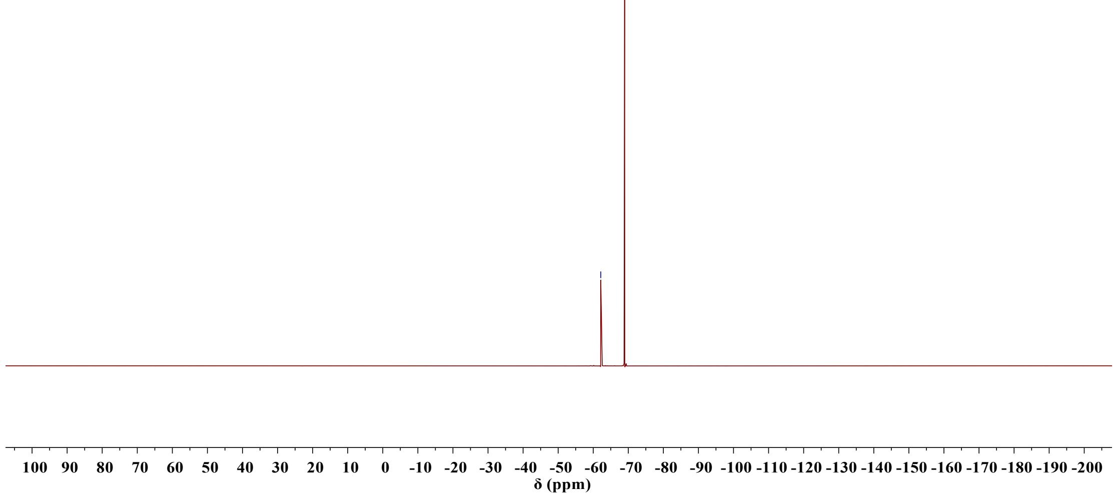

ethyl 7-chloro-8,8,8-trifluorooct-6-enoate (1ab)

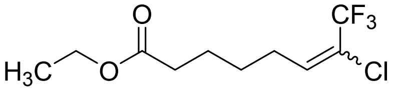

1ab

^{13}C NMR (126 MHz, $CDCl_3$)

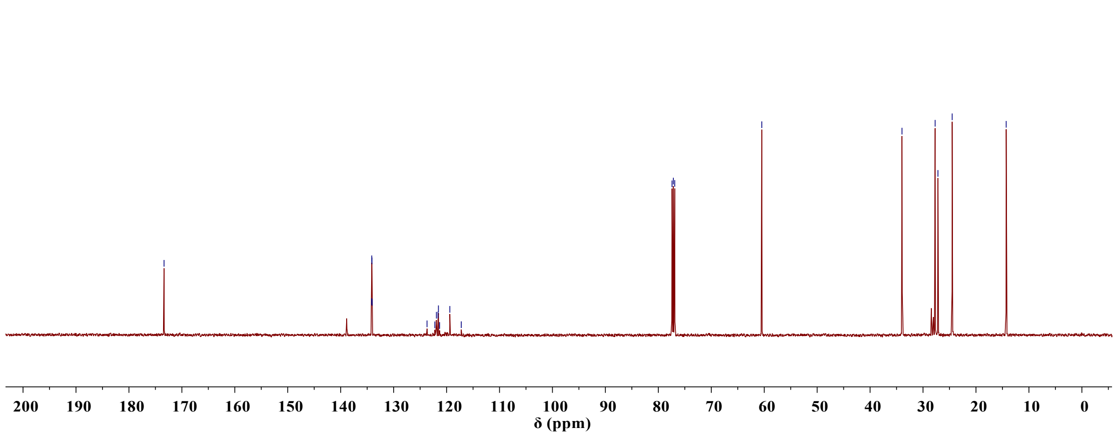

tert-butyl((4-chloro-5,5,5-trifluoropent-3-en-1-yl)oxy)dimethylsilane (1ac)

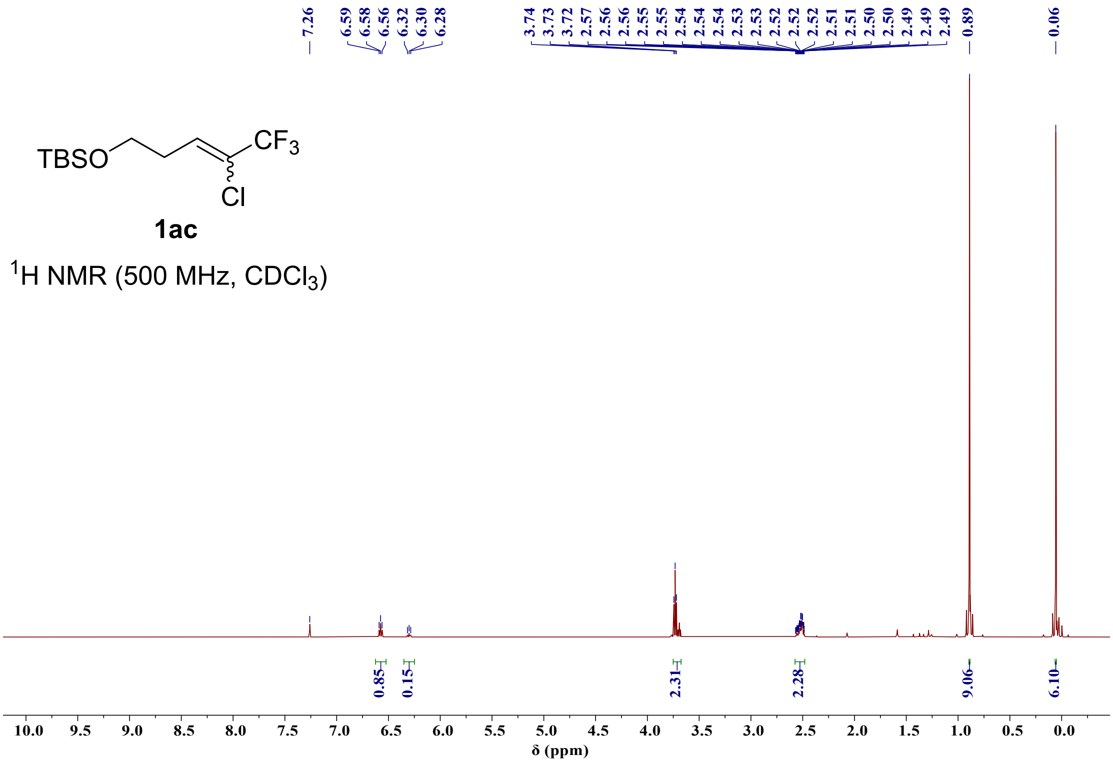

tert-butyl((4-chloro-5,5,5-trifluoropent-3-en-1-yl)oxy)dimethylsilane (1ac)

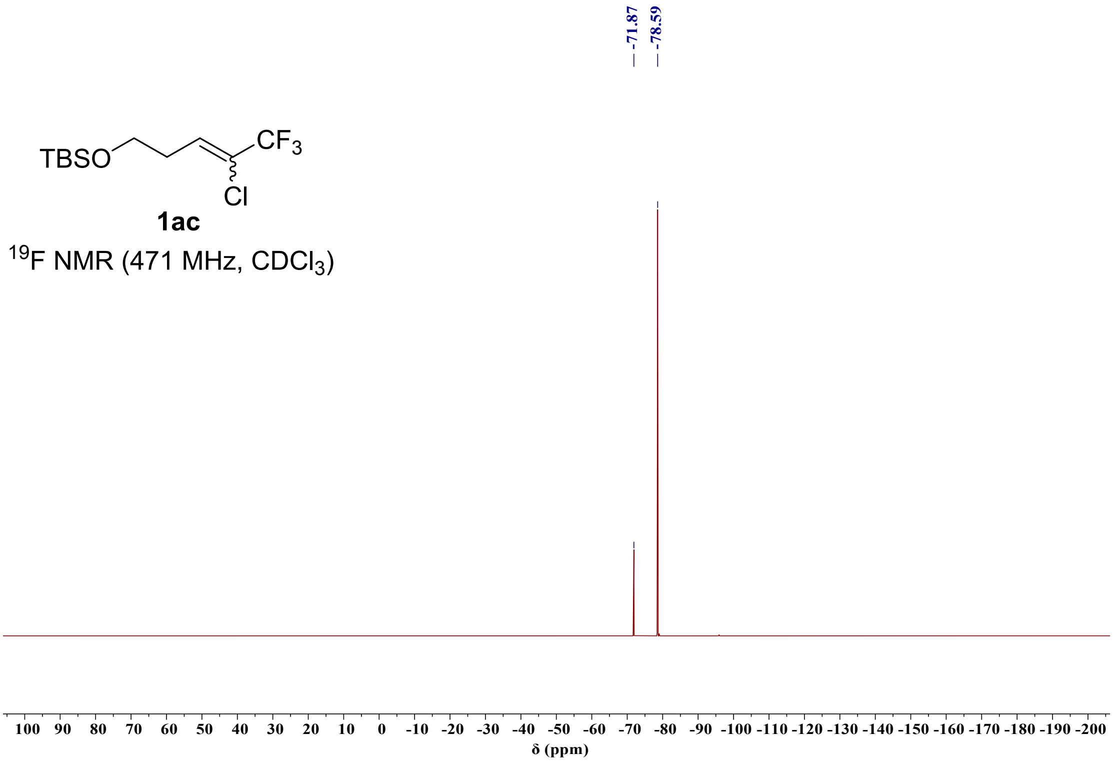

tert-butyl((4-chloro-5,5,5-trifluoropent-3-en-1-yl)oxy)dimethylsilane (1ac)

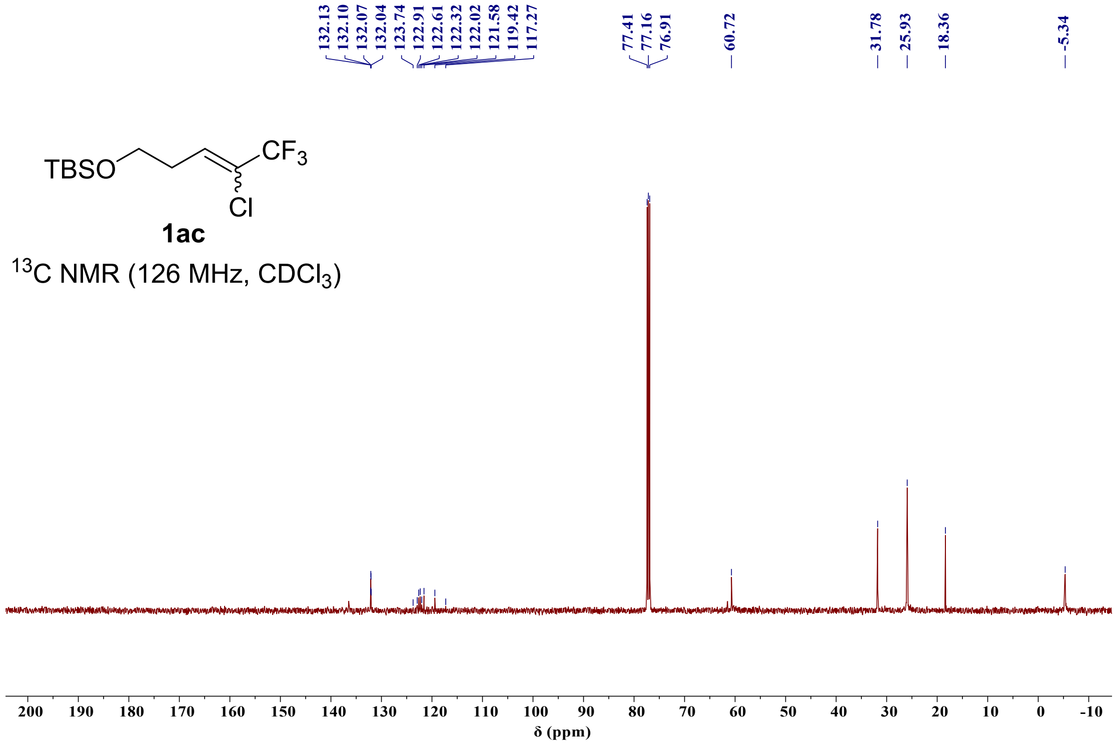

10-bromo-2-chloro-1,1,1-trifluorodec-2-ene (1ad)

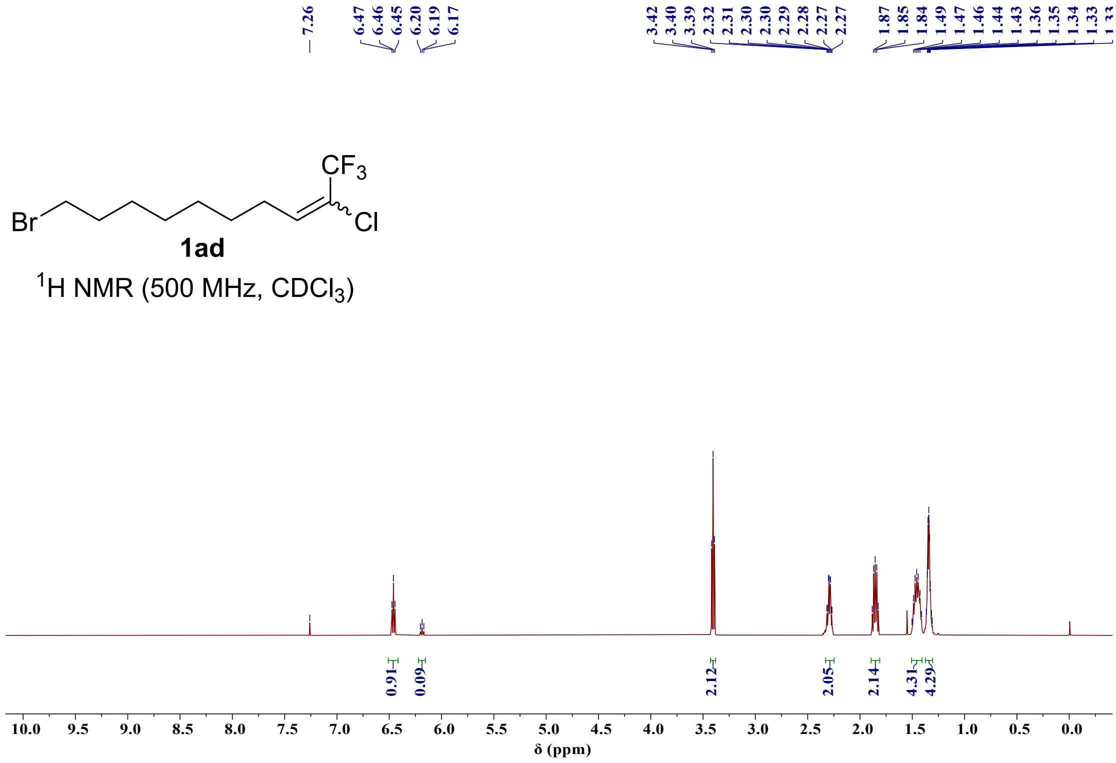

10-bromo-2-chloro-1,1,1-trifluorodec-2-ene (1ad)

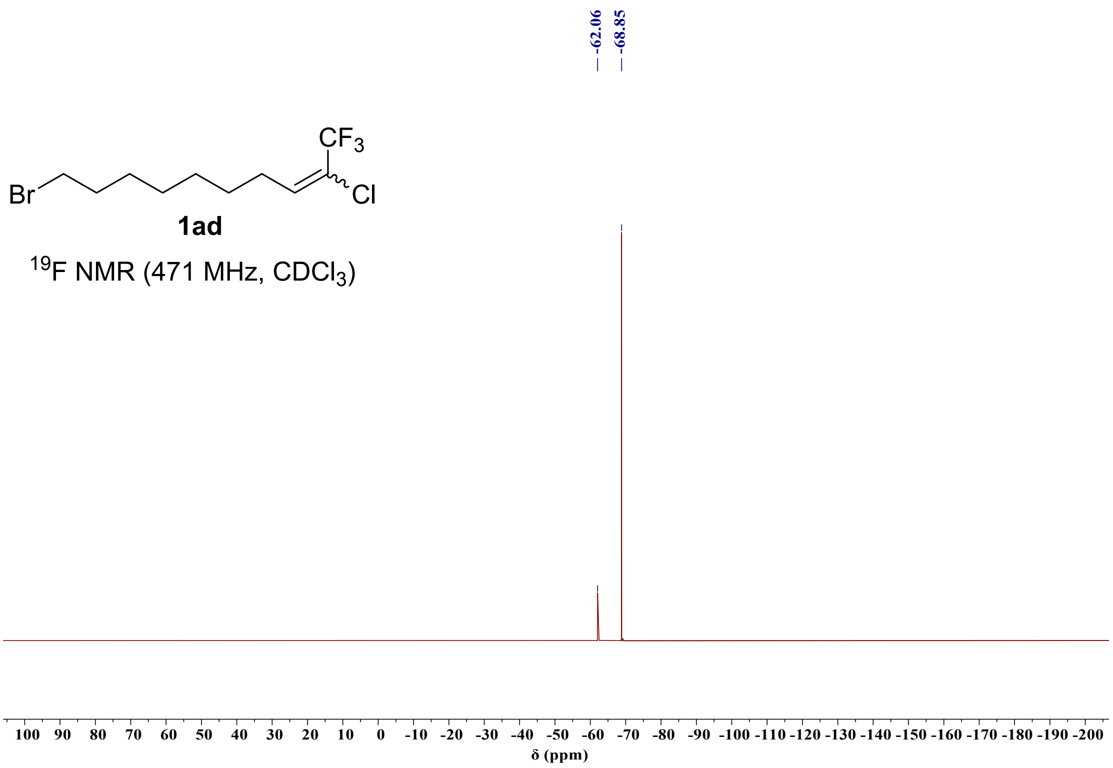

10-bromo-2-chloro-1,1,1-trifluorodec-2-ene (1ad)

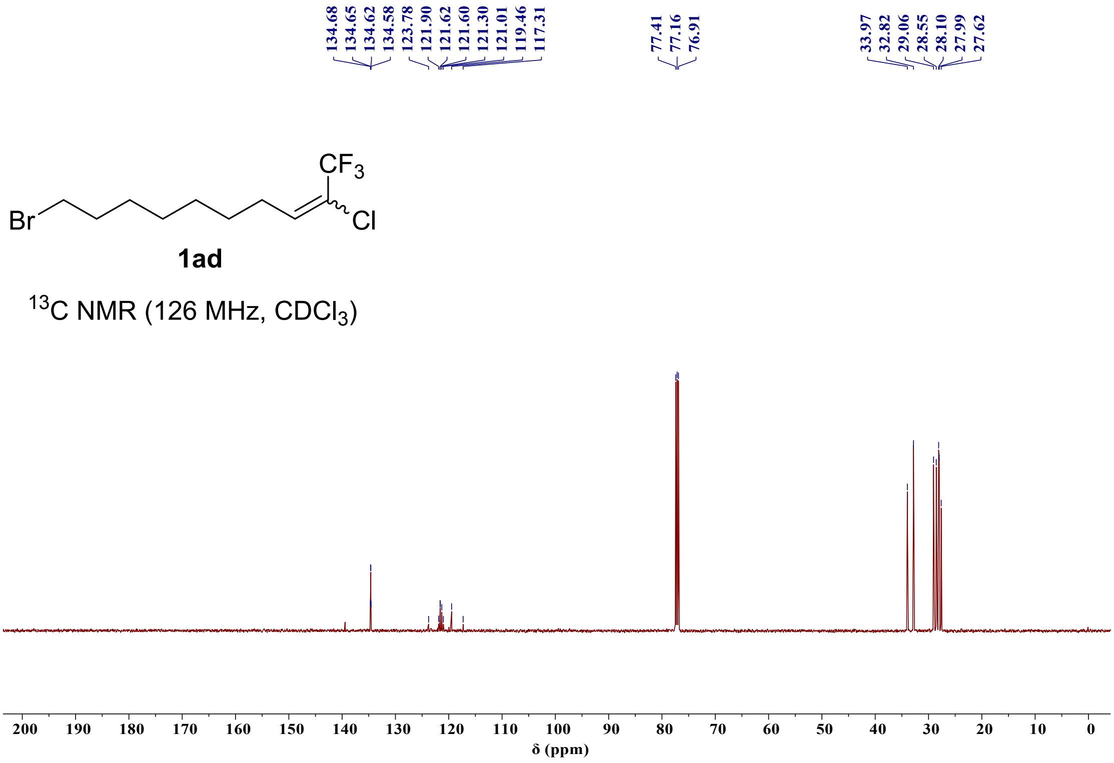

(4-chloro-5,5,5-trifluoropent-3-en-1-yl)cyclohexane (1ae)

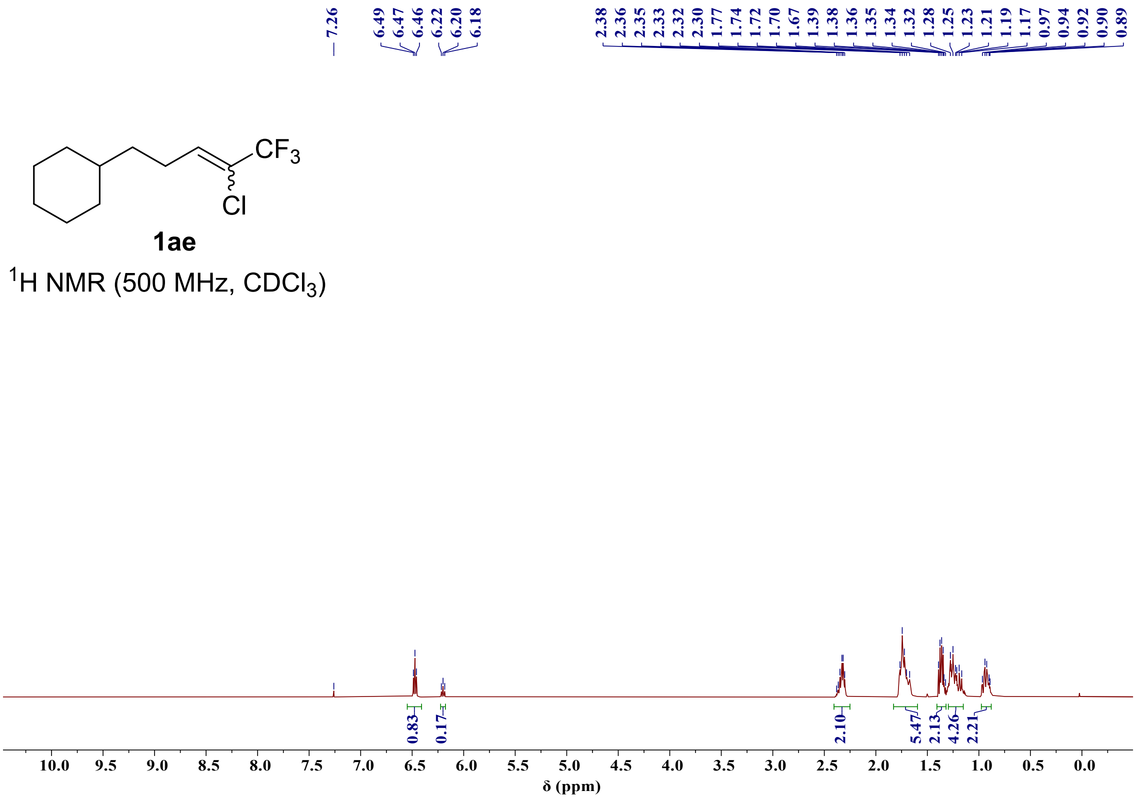

(4-chloro-5,5,5-trifluoropent-3-en-1-yl)cyclohexane (1ae)

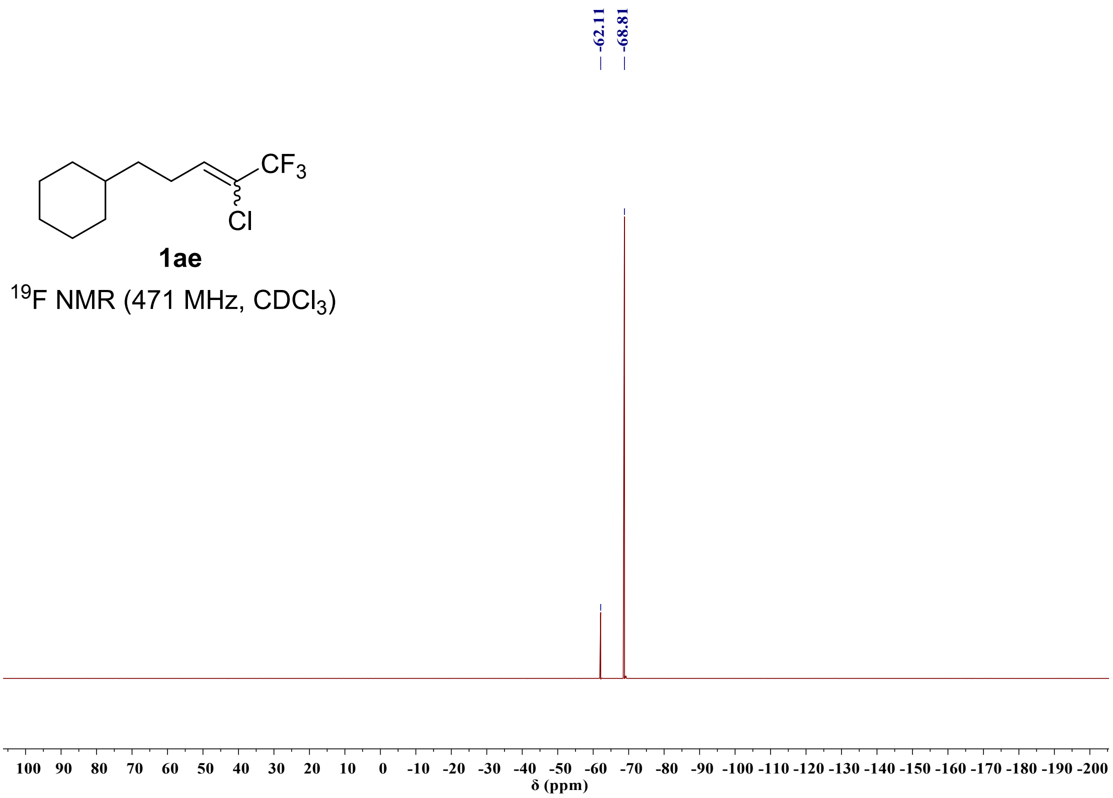

(4-chloro-5,5,5-trifluoropent-3-en-1-yl)cyclohexane (1ae)

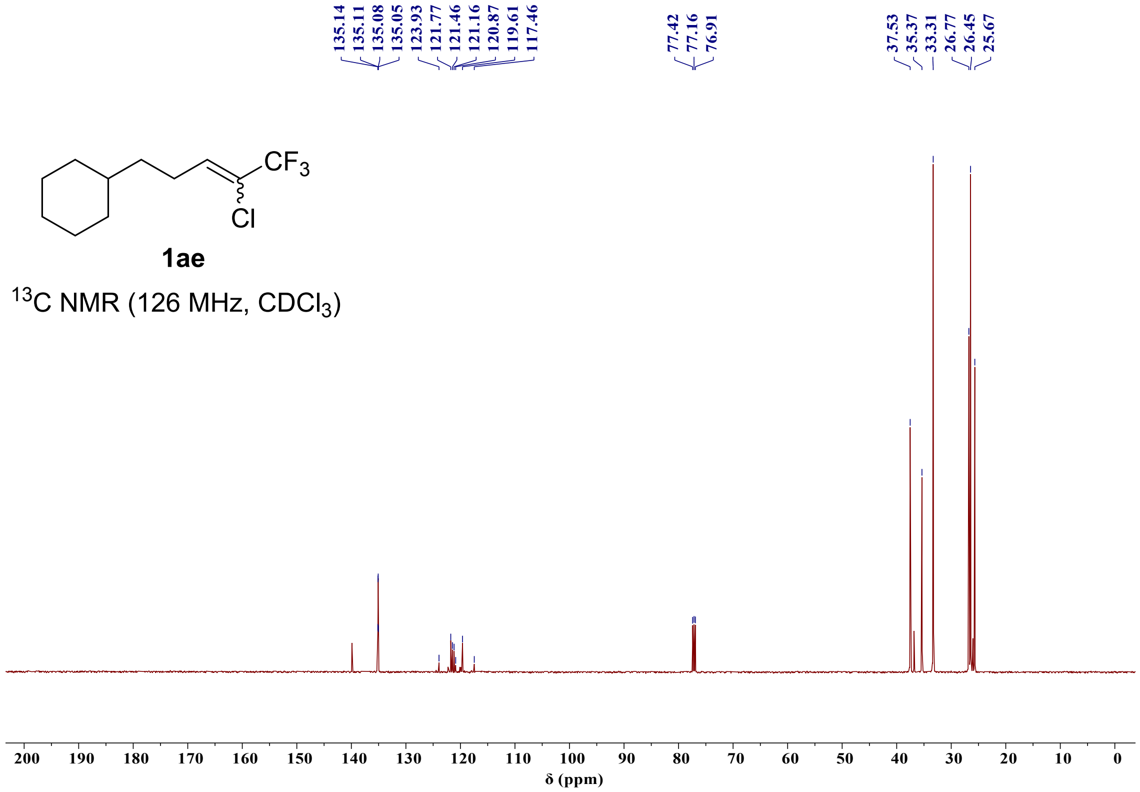

2-(2-chloro-3,3,3-trifluoroprop-1-en-1-yl)-2,3-dihydro-1H-indene (1af)

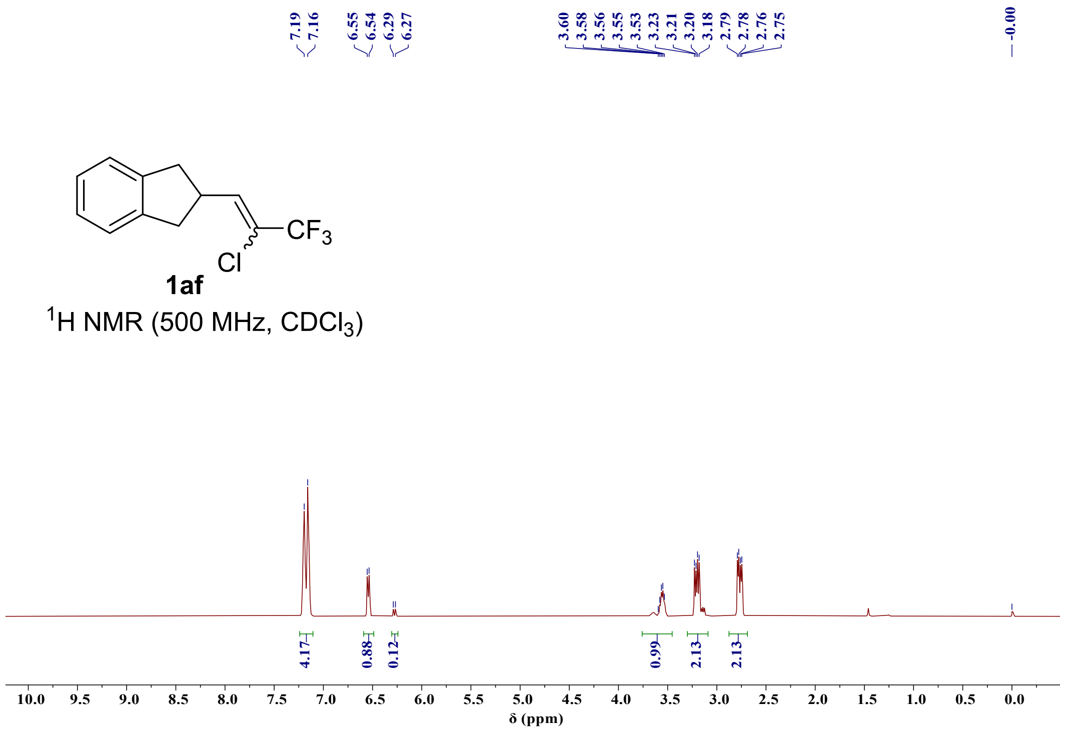

2-(2-chloro-3,3,3-trifluoroprop-1-en-1-yl)-2,3-dihydro-1H-indene (1af)

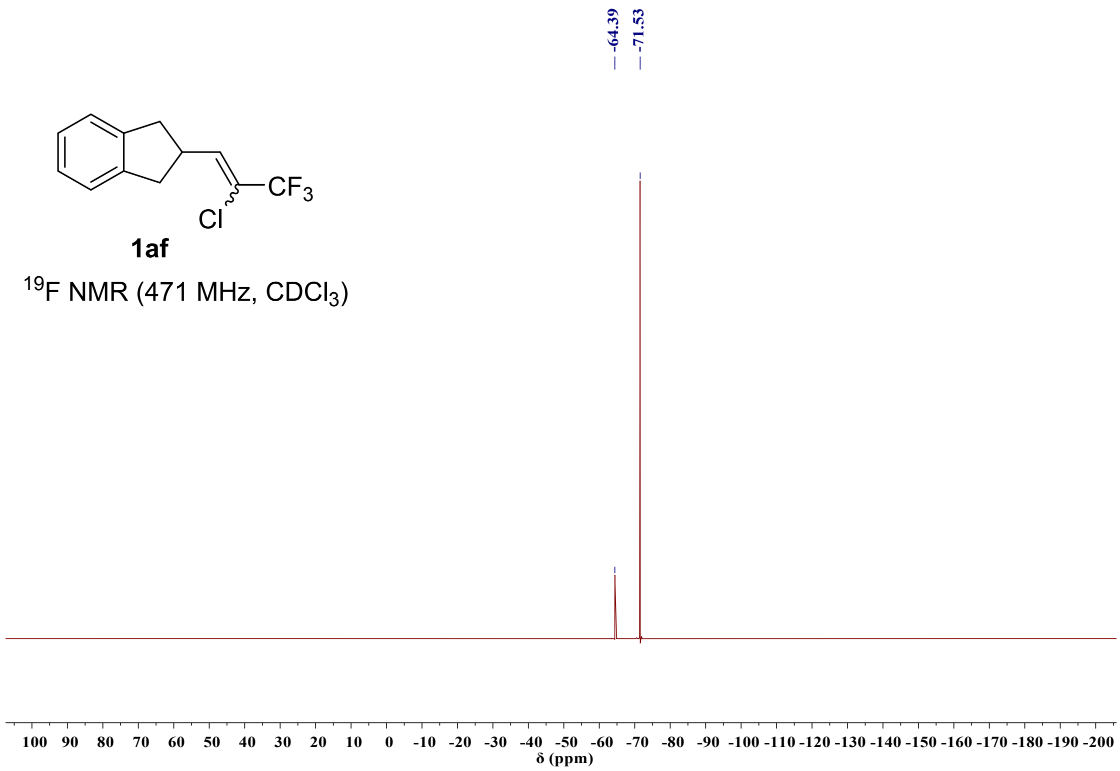

2-(2-chloro-3,3,3-trifluoroprop-1-en-1-yl)-2,3-dihydro-1H-indene (1af)

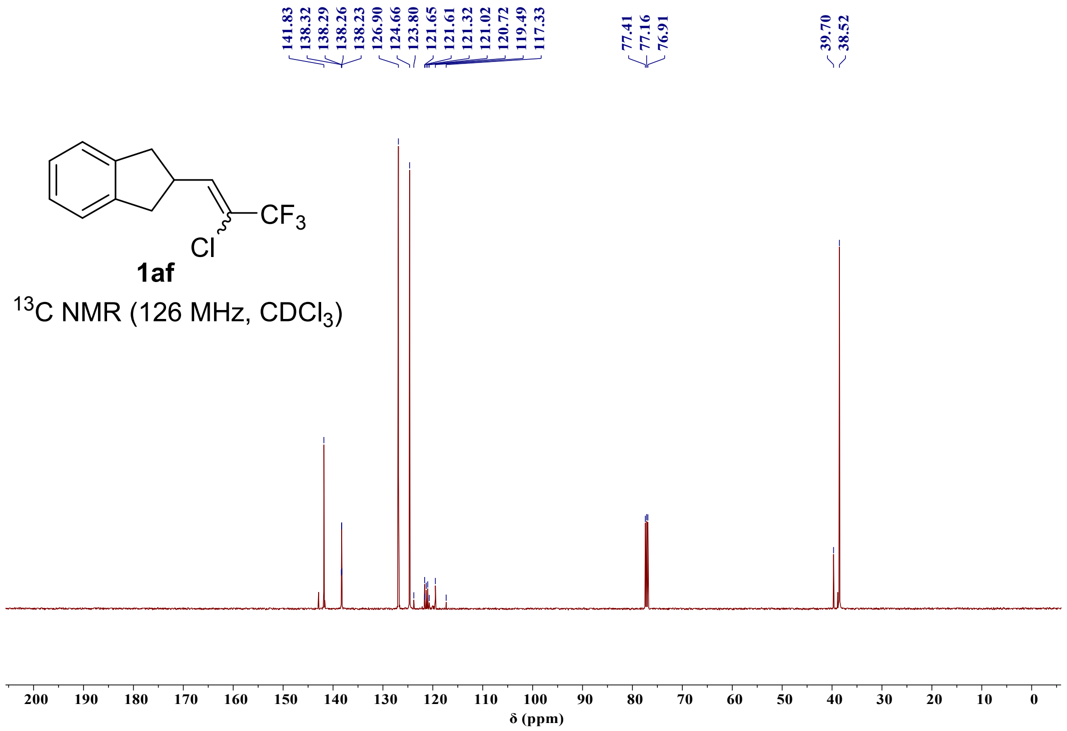

(1-(2-chloro-3,3,3-trifluoroprop-1-en-1-yl)cyclopropyl)benzene (1ag)

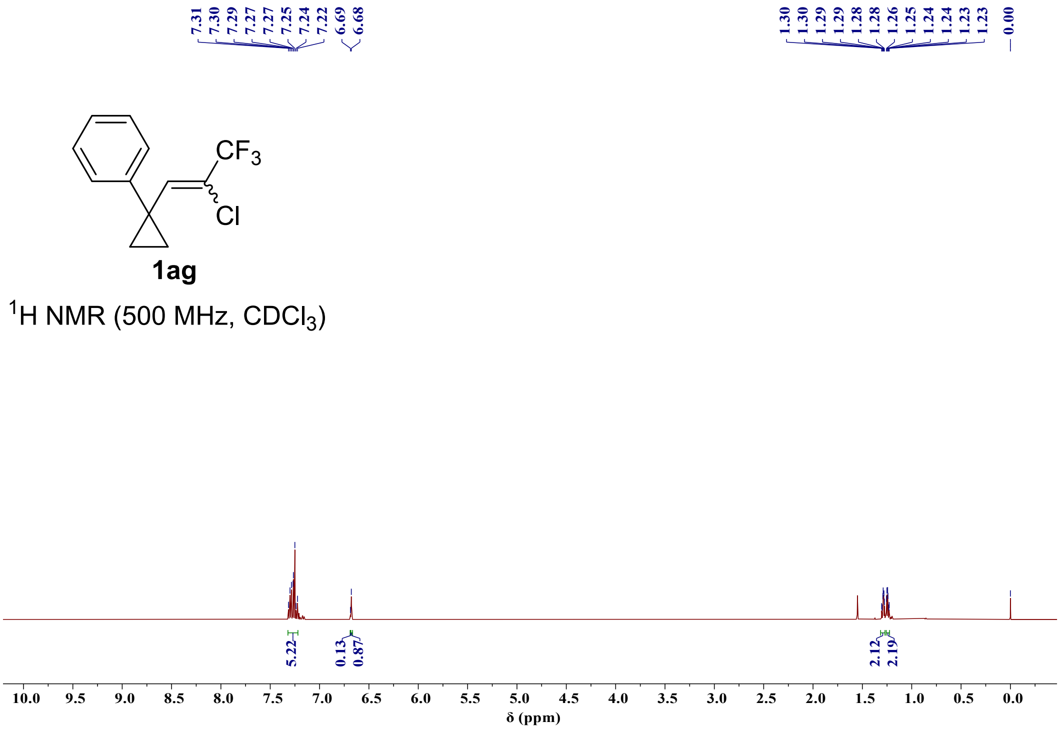

(1-(2-chloro-3,3,3-trifluoroprop-1-en-1-yl)cyclopropyl)benzene (1ag)

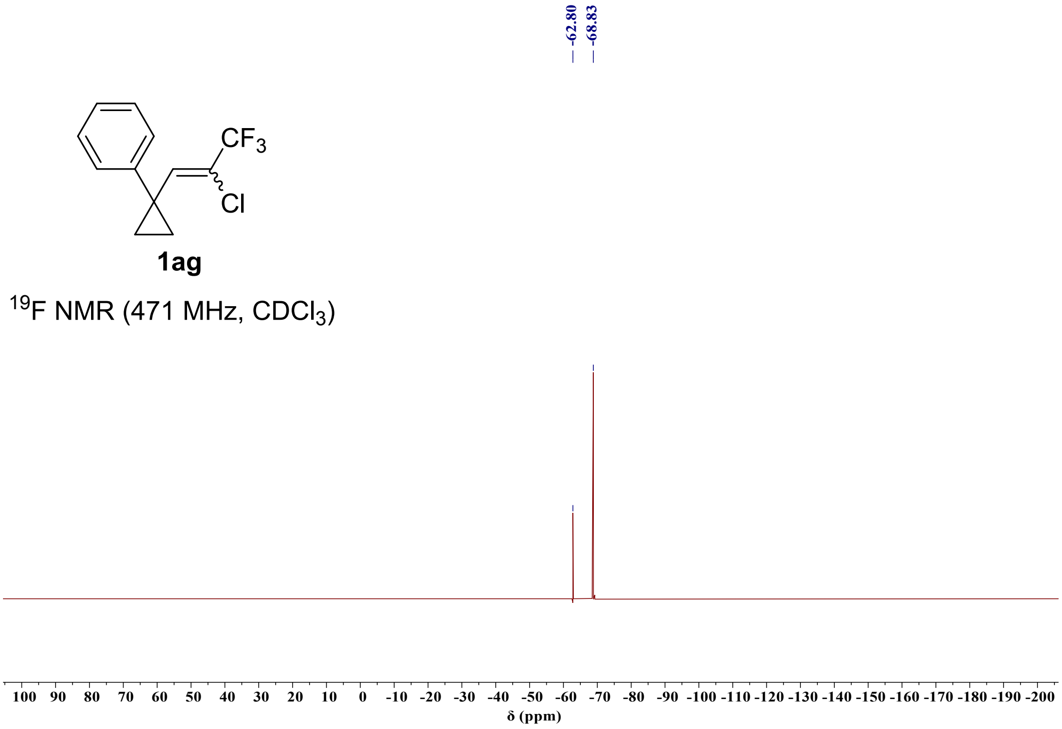

(1-(2-chloro-3,3,3-trifluoroprop-1-en-1-yl)cyclopropyl)benzene (1ag)

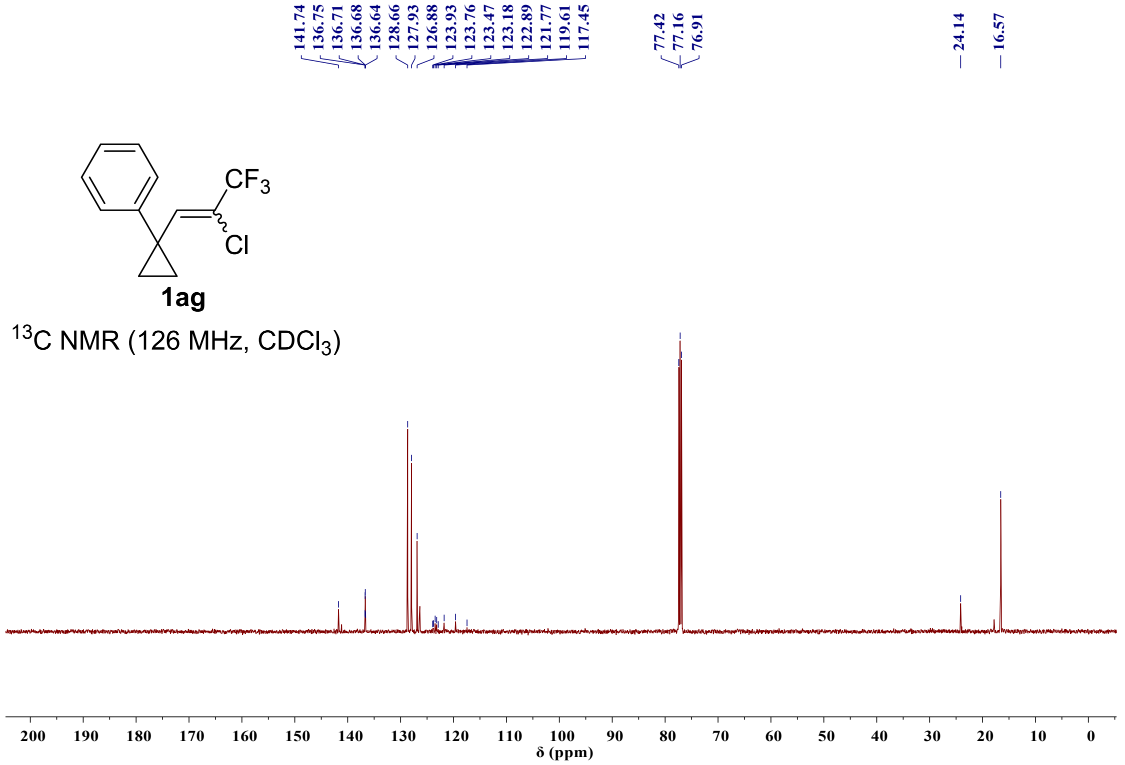

1ag

^{13}C NMR (126 MHz, $CDCl_3$)

(4-chloro-5,5,5-trifluoropent-3-en-1-yl)cyclopentane (1ah)

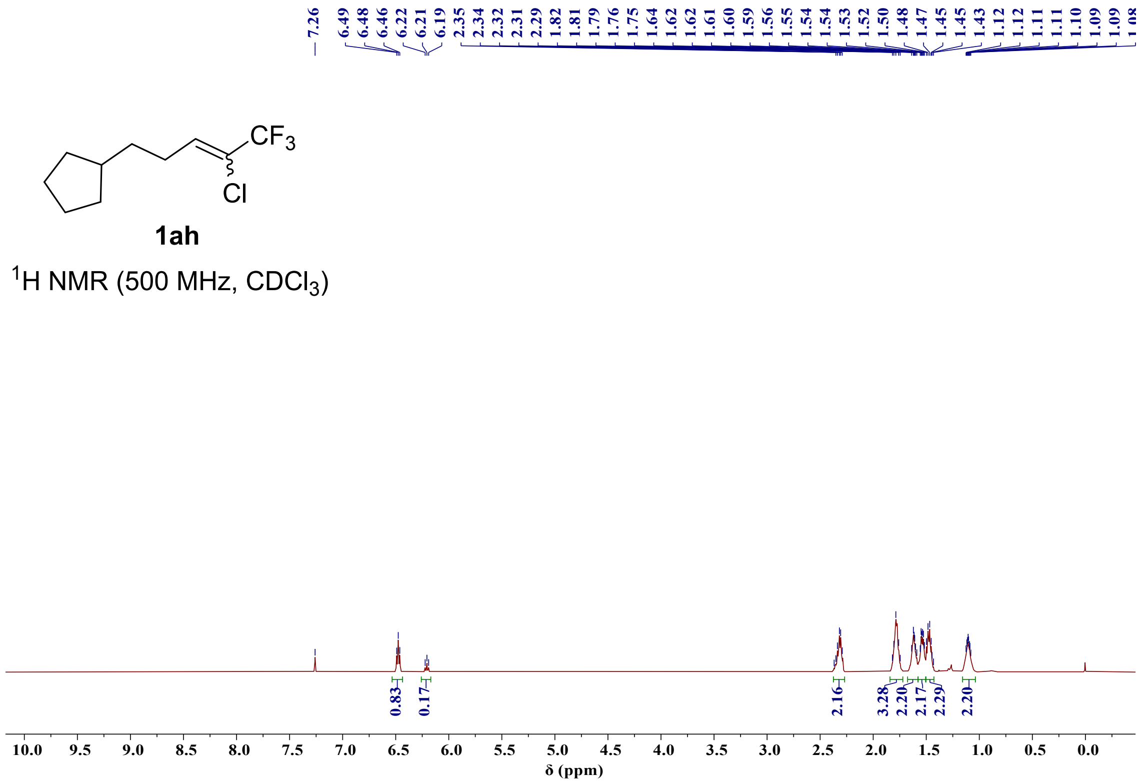

1ah

^{1}H NMR (500 MHz, $CDCl_3$)

(4-chloro-5,5,5-trifluoropent-3-en-1-yl)cyclopentane (1ah)

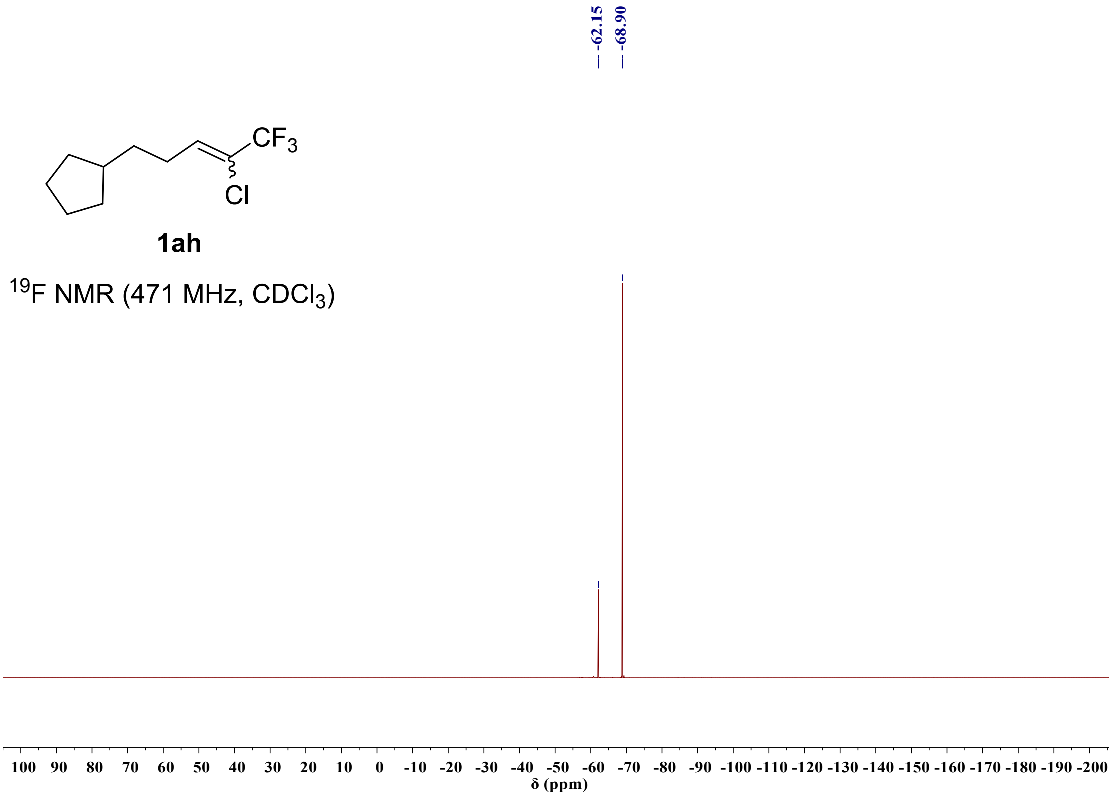

(4-chloro-5,5,5-trifluoropent-3-en-1-yl)cyclopentane (1ah)

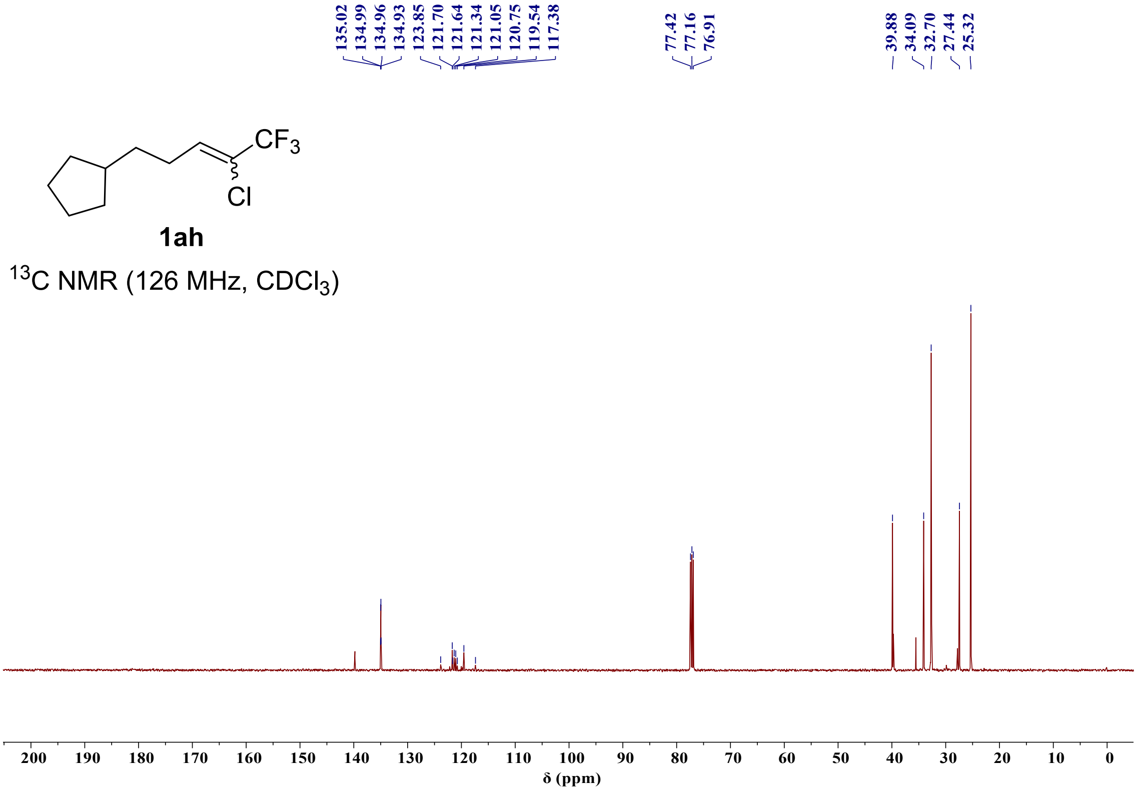

4-(2-chloro-3,3,3-trifluoroprop-1-en-1-yl)-1,1-difluorocyclohexane (1ai)

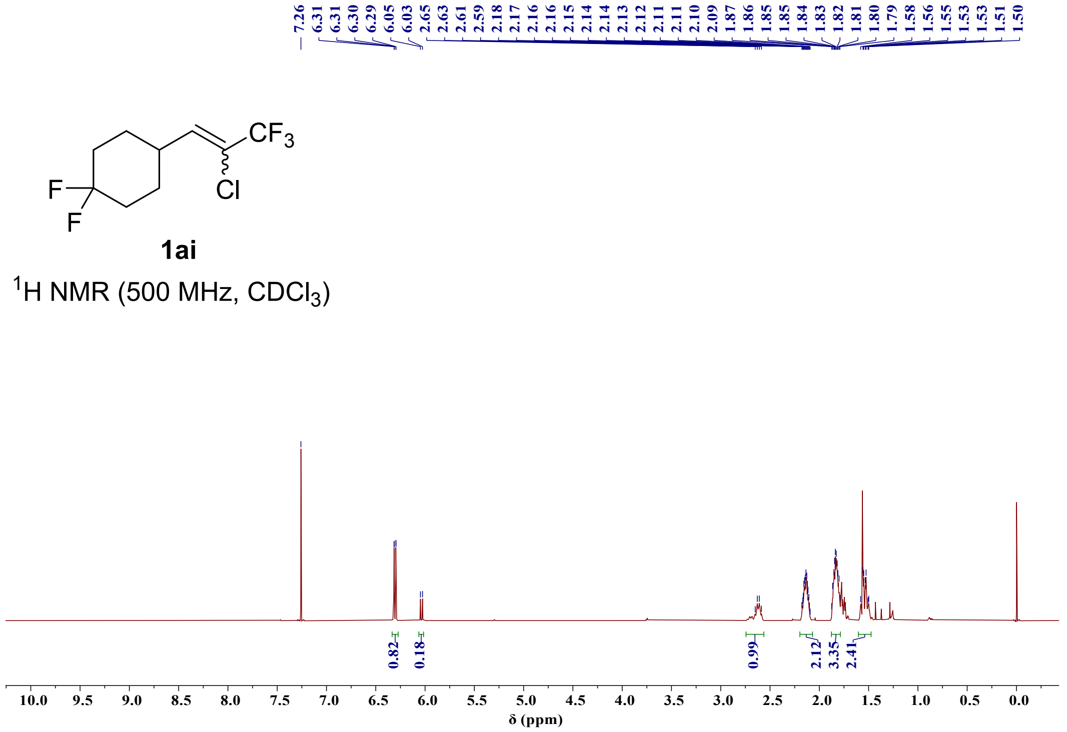

4-(2-chloro-3,3,3-trifluoroprop-1-en-1-yl)-1,1-difluorocyclohexane (1ai)

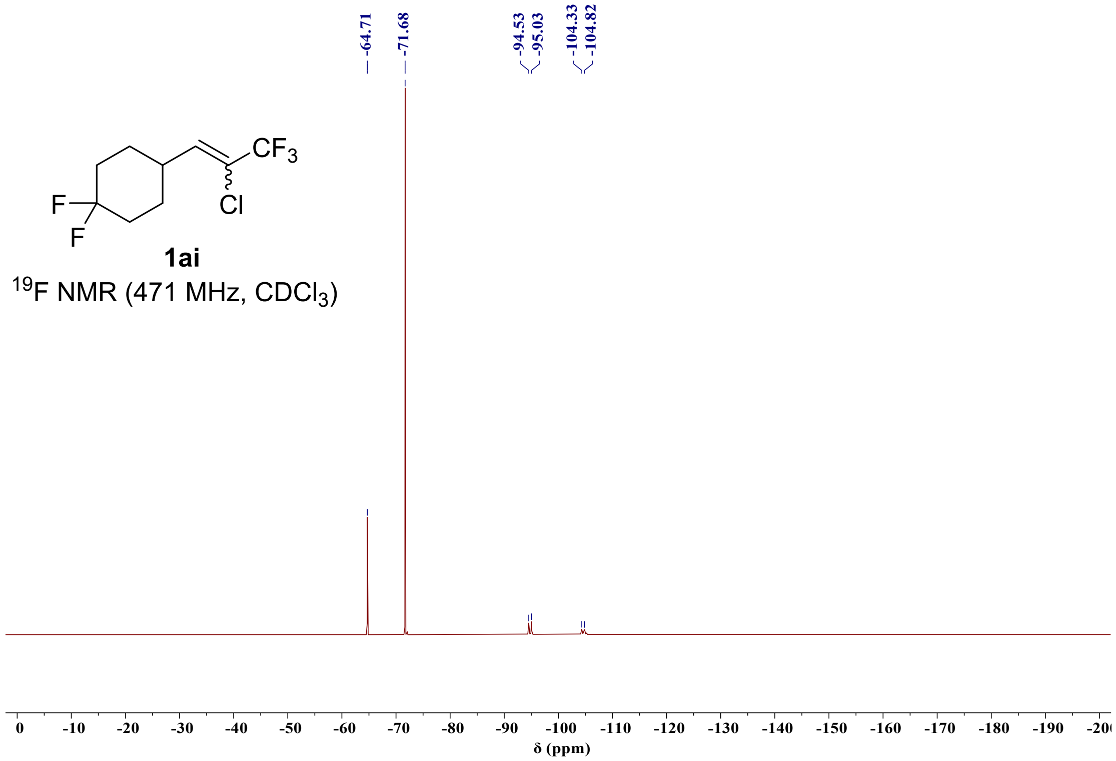

4-(2-chloro-3,3,3-trifluoroprop-1-en-1-yl)-1,1-difluorocyclohexane (1ai)

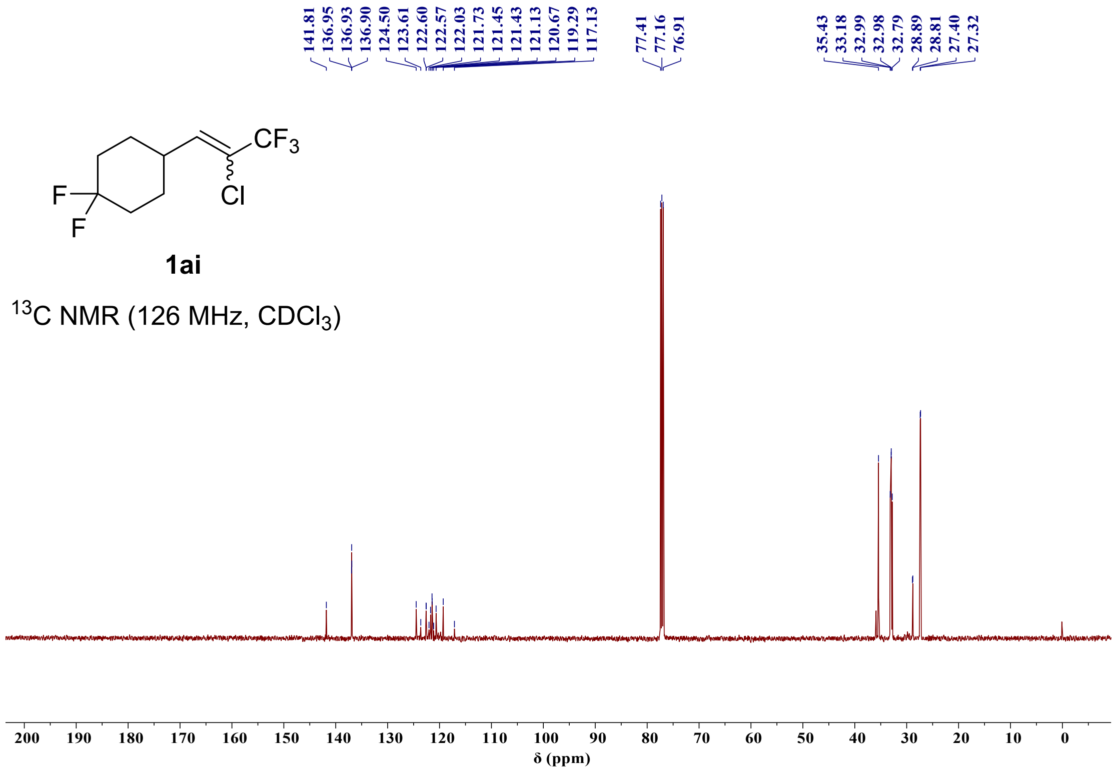

(3r,5r,7r)-1-(3-chloro-4,4,4-trifluorobut-2-en-1-yl)adamantane (1aj)

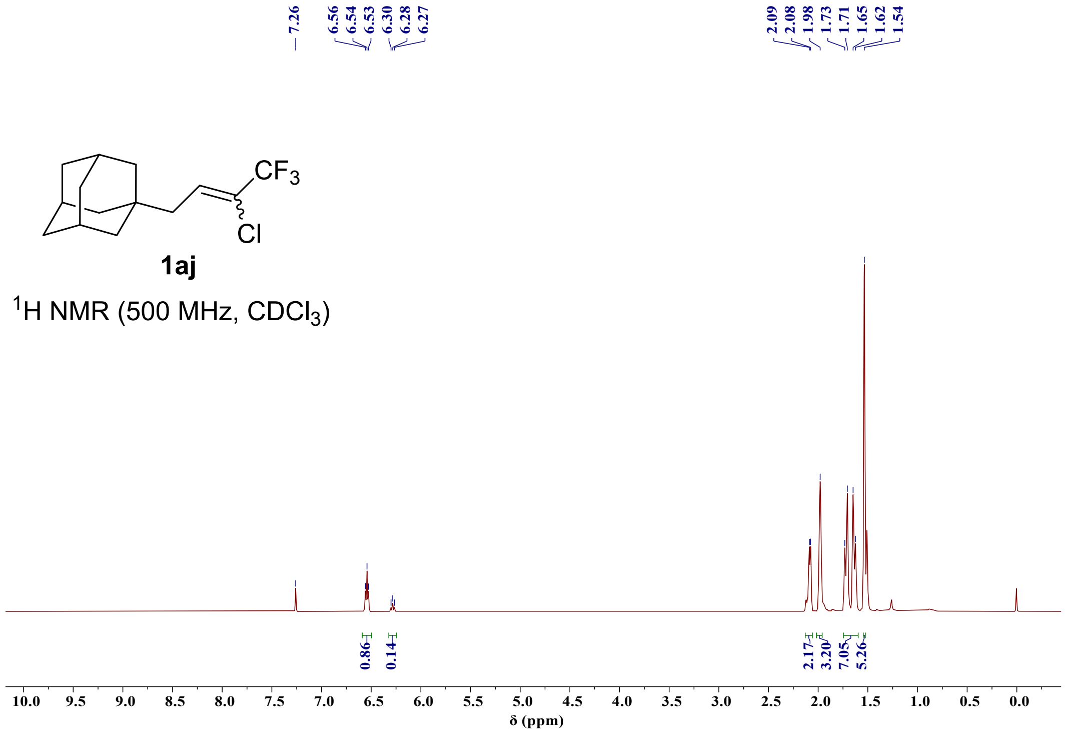

(3r,5r,7r)-1-(3-chloro-4,4,4-trifluorobut-2-en-1-yl)adamantane (1aj)

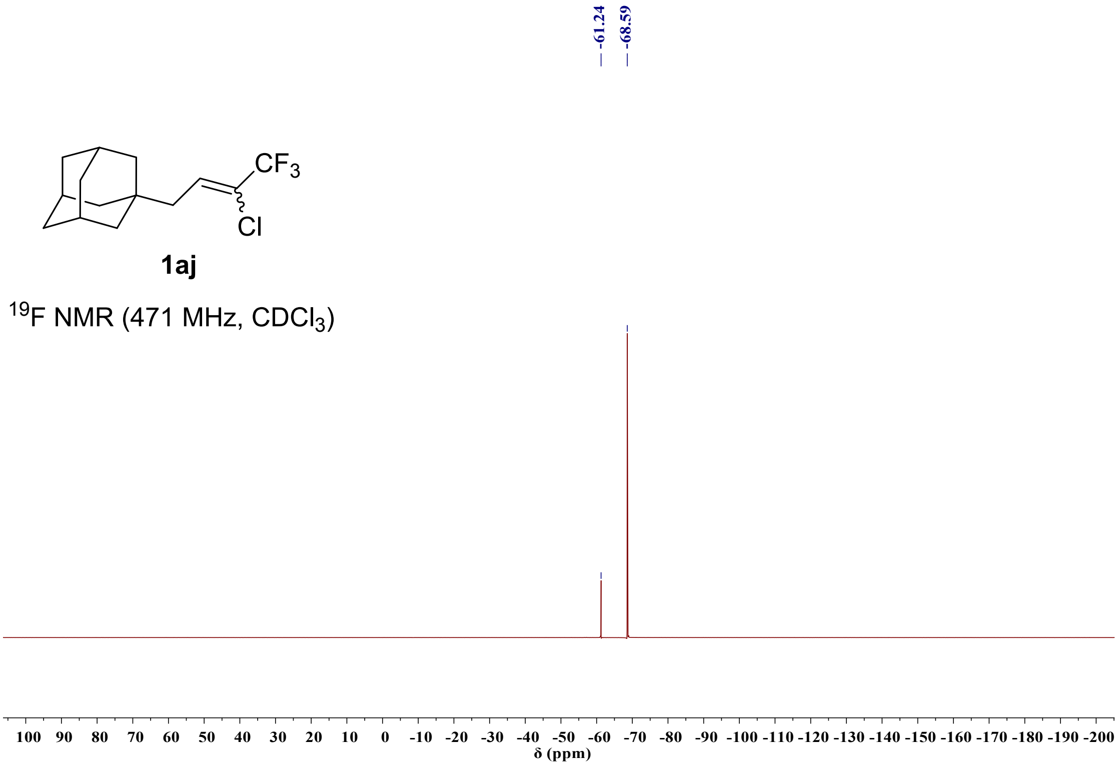

(3r,5r,7r)-1-(3-chloro-4,4,4-trifluorobut-2-en-1-yl)adamantane (1aj)

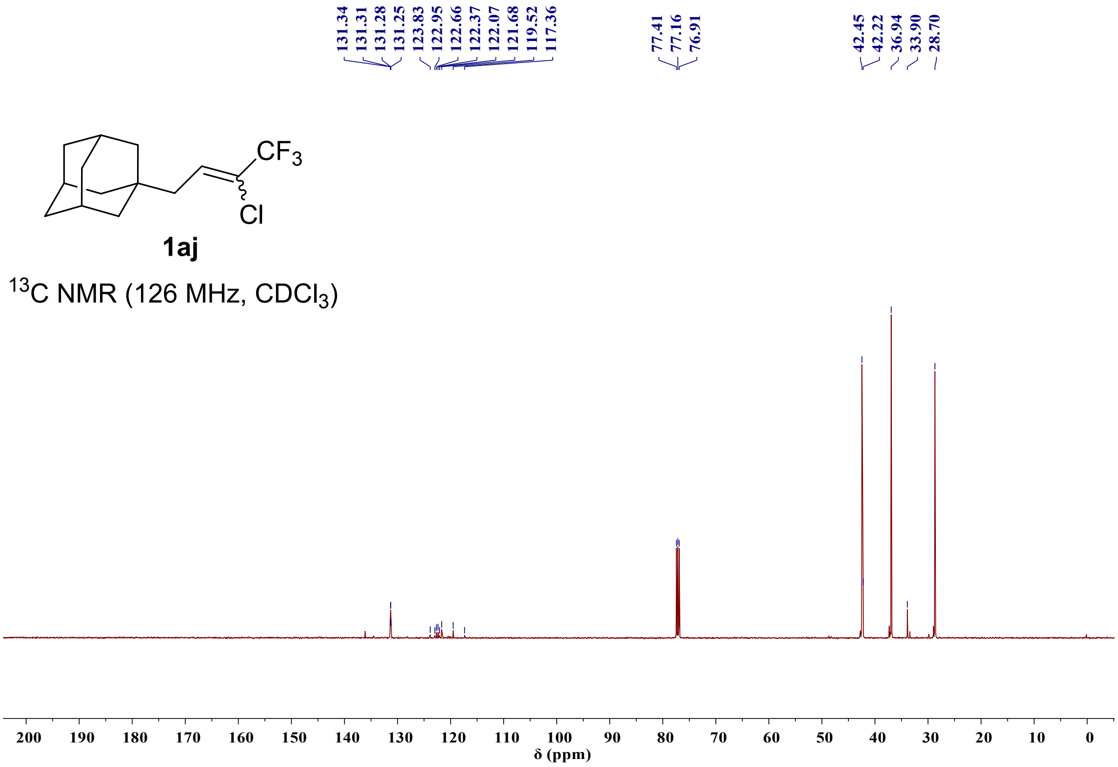

(R)-2-(4-chloro-5,5,5-trifluoropent-3-en-2-yl)-6-methoxynaphthalene (1ak)

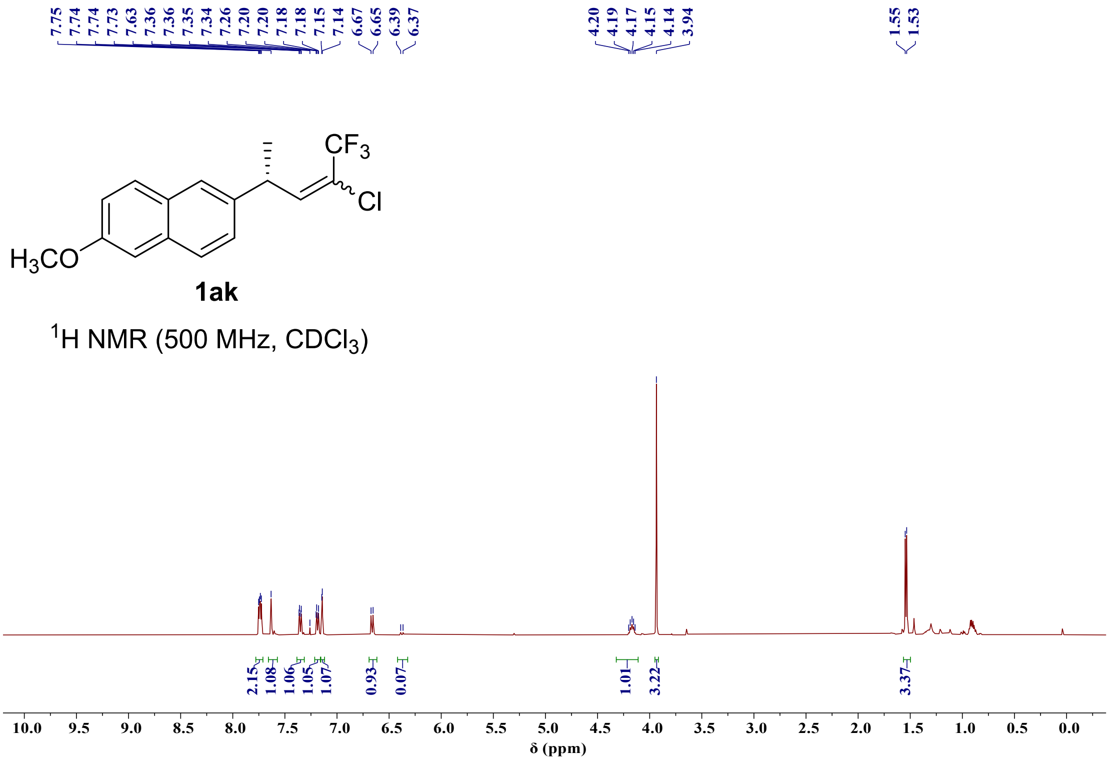

(R)-2-(4-chloro-5,5,5-trifluoropent-3-en-2-yl)-6-methoxynaphthalene (1ak)

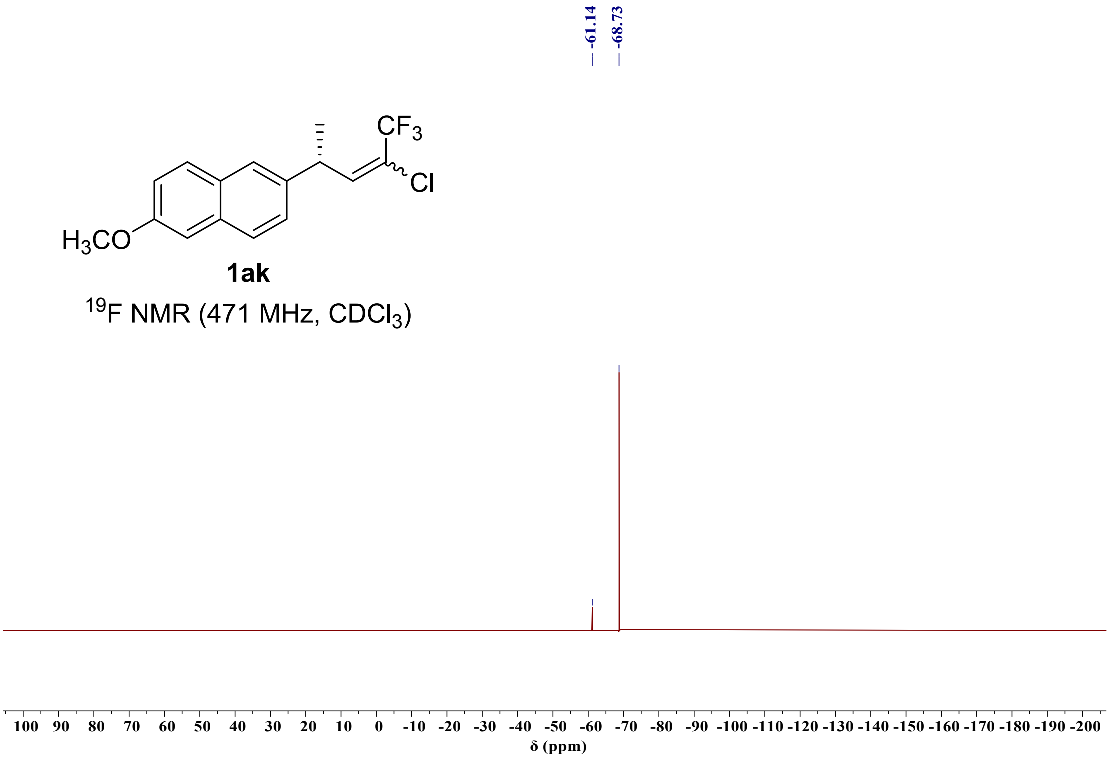

(R)-2-(4-chloro-5,5,5-trifluoropent-3-en-2-yl)-6-methoxynaphthalene (1ak)

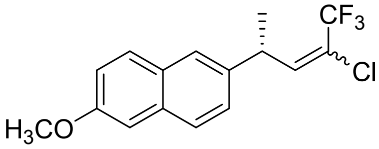

1ak

^{13}C NMR (126 MHz, CDCl$_3$)

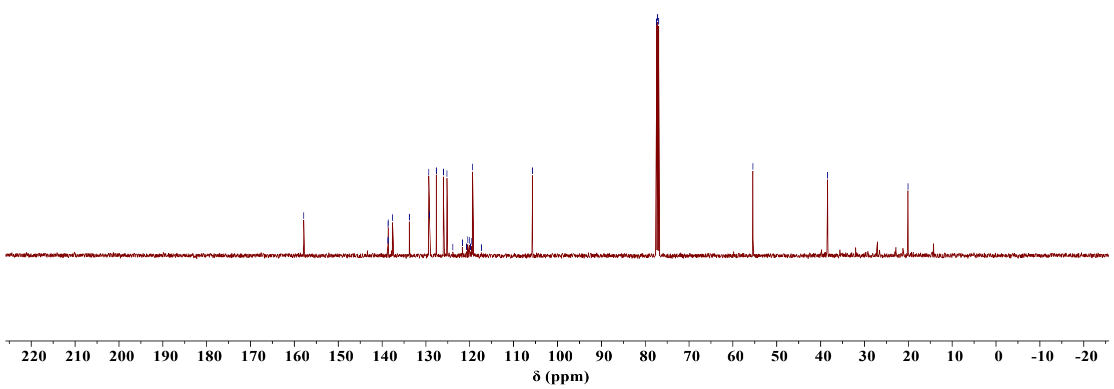

1-(4-chloro-5,5,5-trifluoropent-3-en-2-yl)-4-isobutylbenzene (1al)

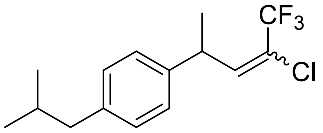

1al

^{1}H NMR (500 MHz, CDCl$_3$)

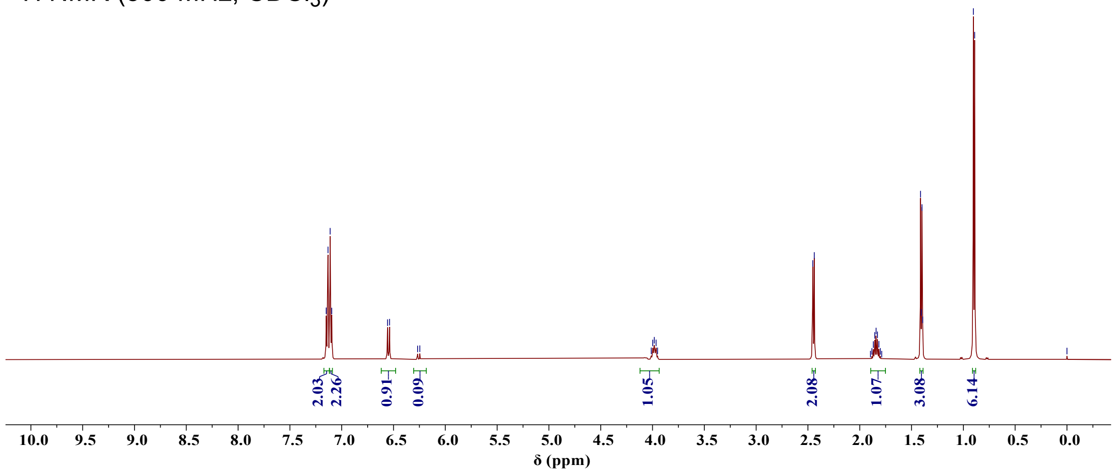

1-(4-chloro-5,5,5-trifluoropent-3-en-2-yl)-4-isobutylbenzene (1al)

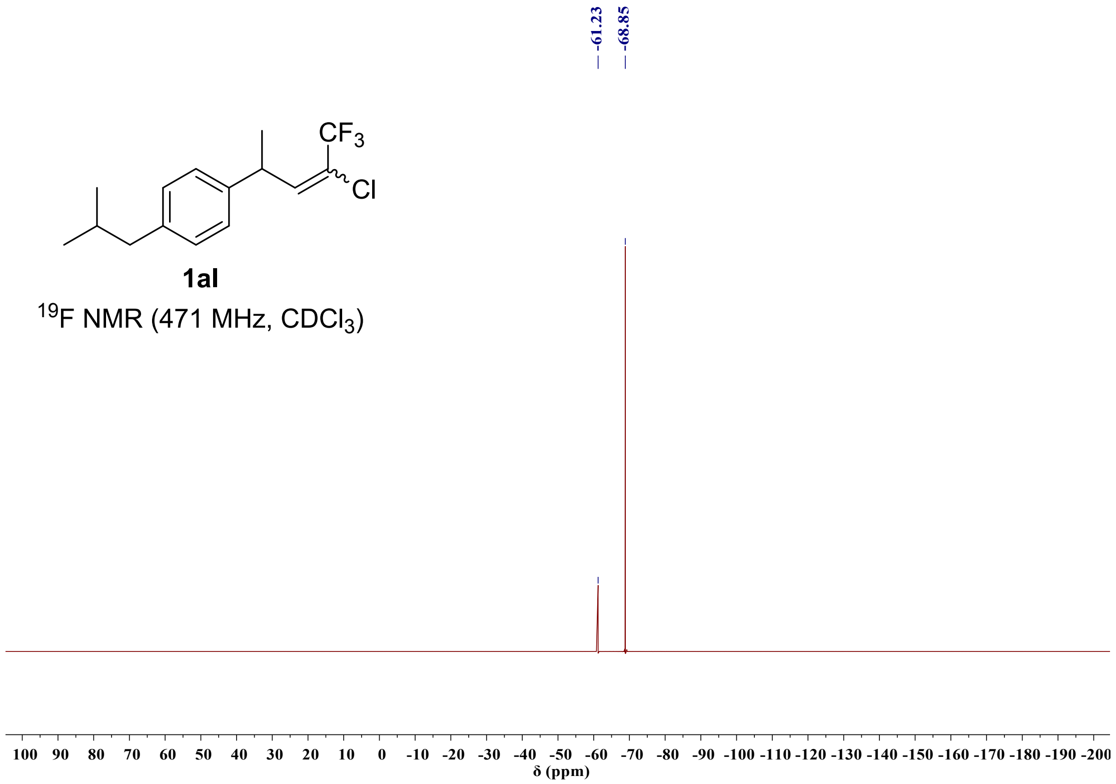

1-(4-chloro-5,5,5-trifluoropent-3-en-2-yl)-4-isobutylbenzene (1al)

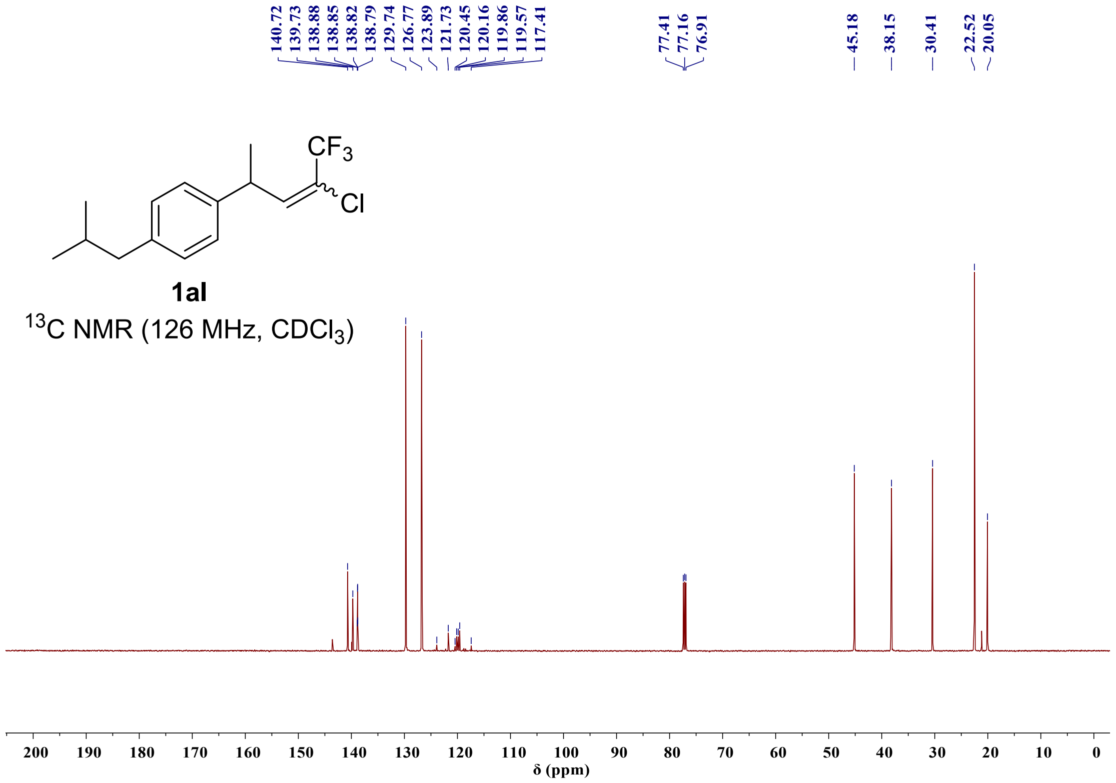

4-(4-chloro-5,5,5-trifluoropent-3-en-2-yl)-2-fluoro-1,1'-biphenyl (1am)

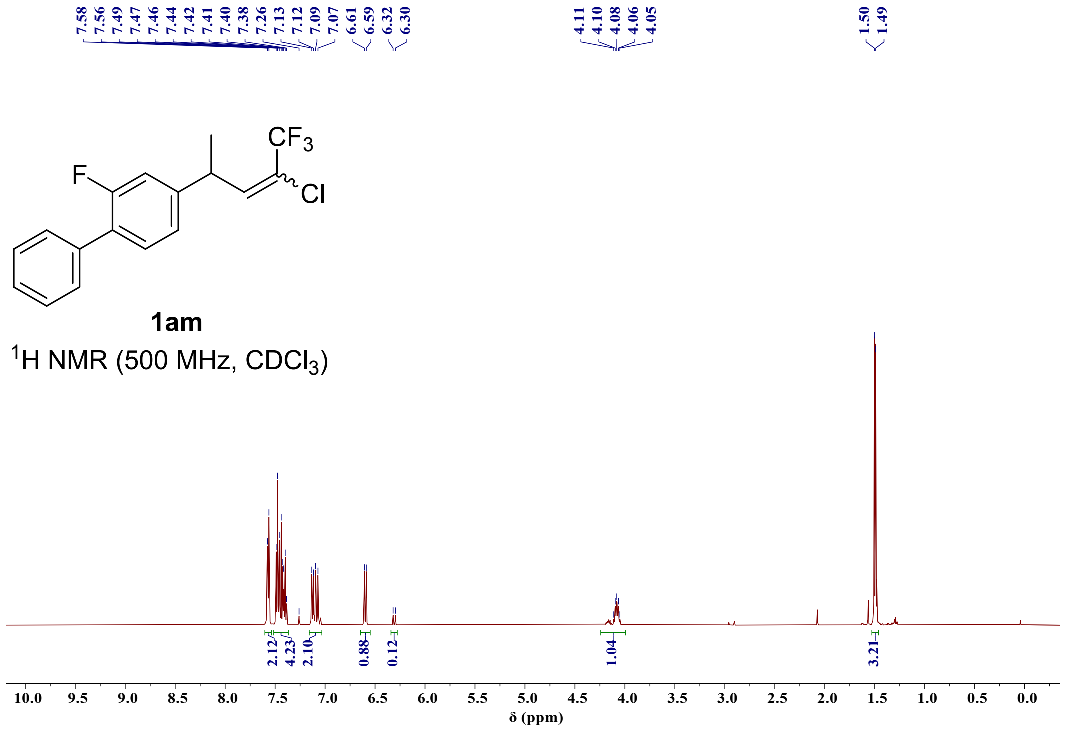

4-(4-chloro-5,5,5-trifluoropent-3-en-2-yl)-2-fluoro-1,1'-biphenyl (1am)

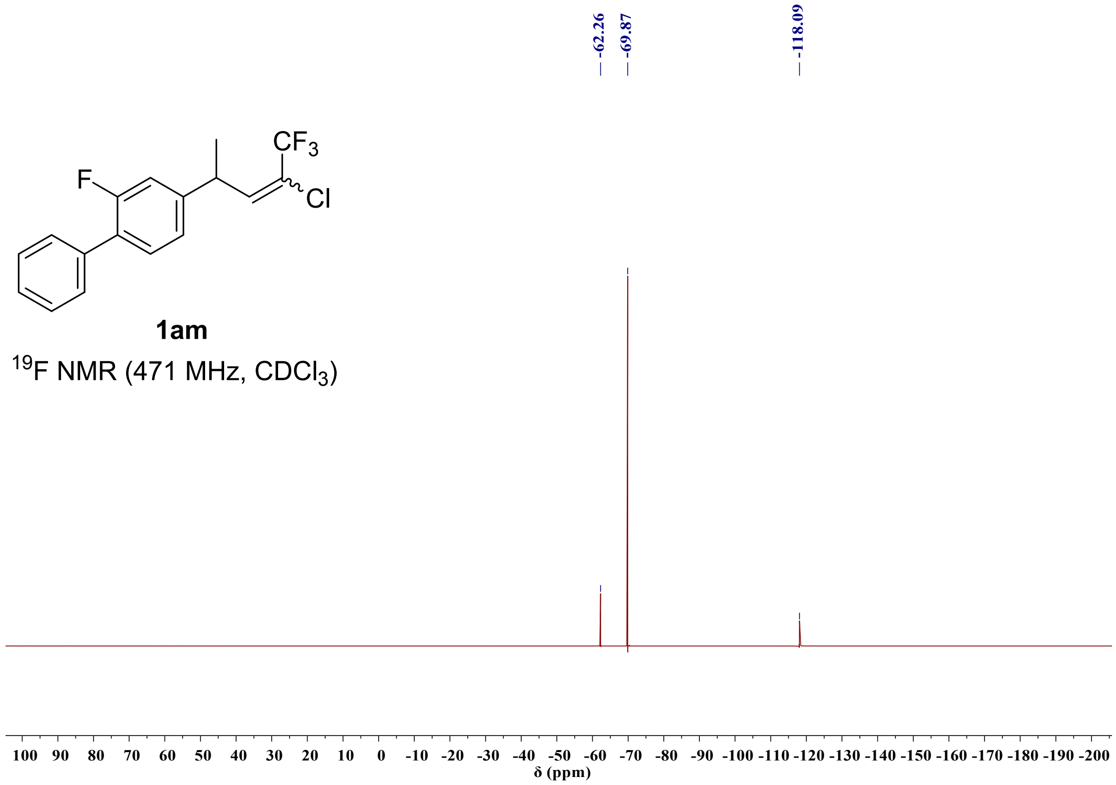

4-(4-chloro-5,5,5-trifluoropent-3-en-2-yl)-2-fluoro-1,1'-biphenyl (1am)

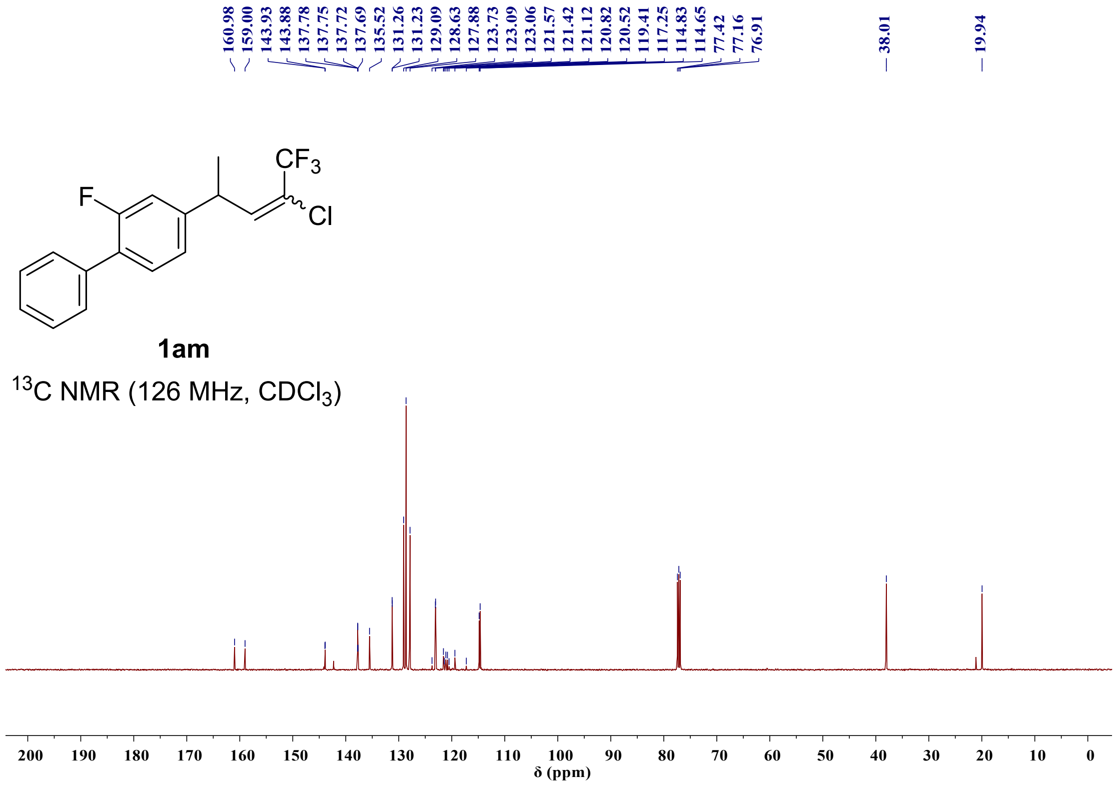

(3-(3-chloro-4,4,4-trifluorobut-2-en-1-yl)-5-methoxy-2-methyl-1H-indol-1-yl)(4-chlorophenyl)methanone (1an)

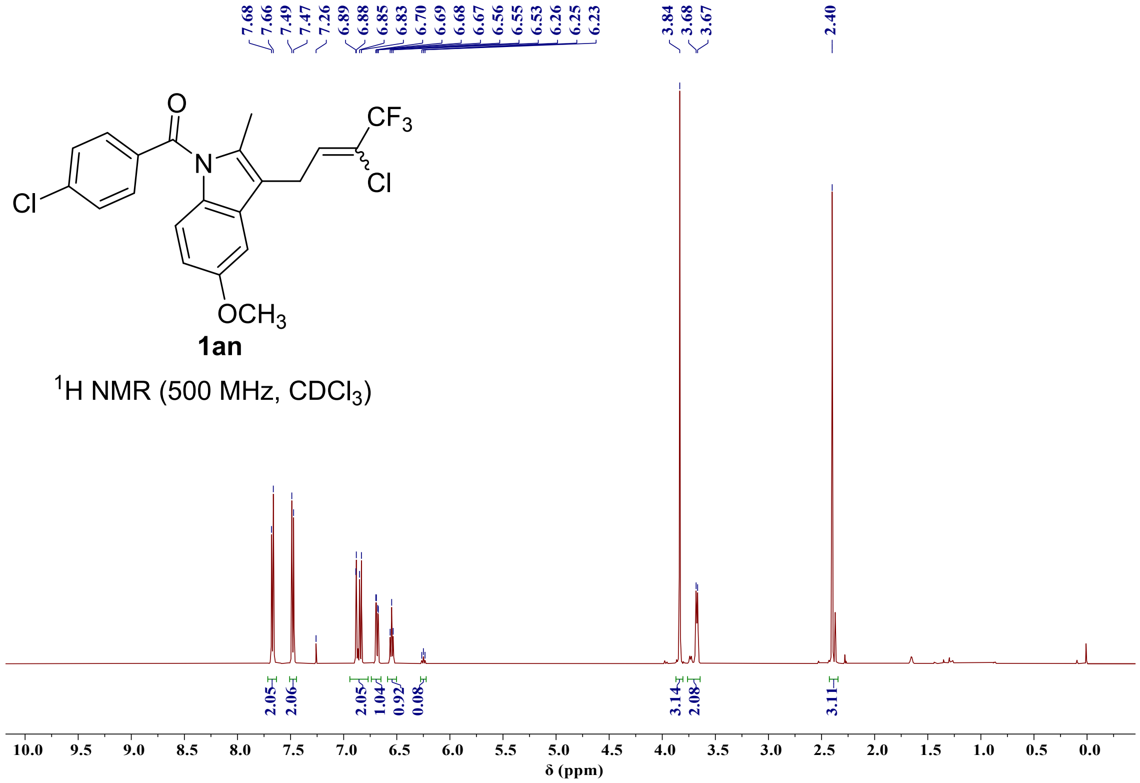

(3-(3-chloro-4,4,4-trifluorobut-2-en-1-yl)-5-methoxy-2-methyl-1H-indol-1-yl)(4-chlorophenyl)methanone (1an)

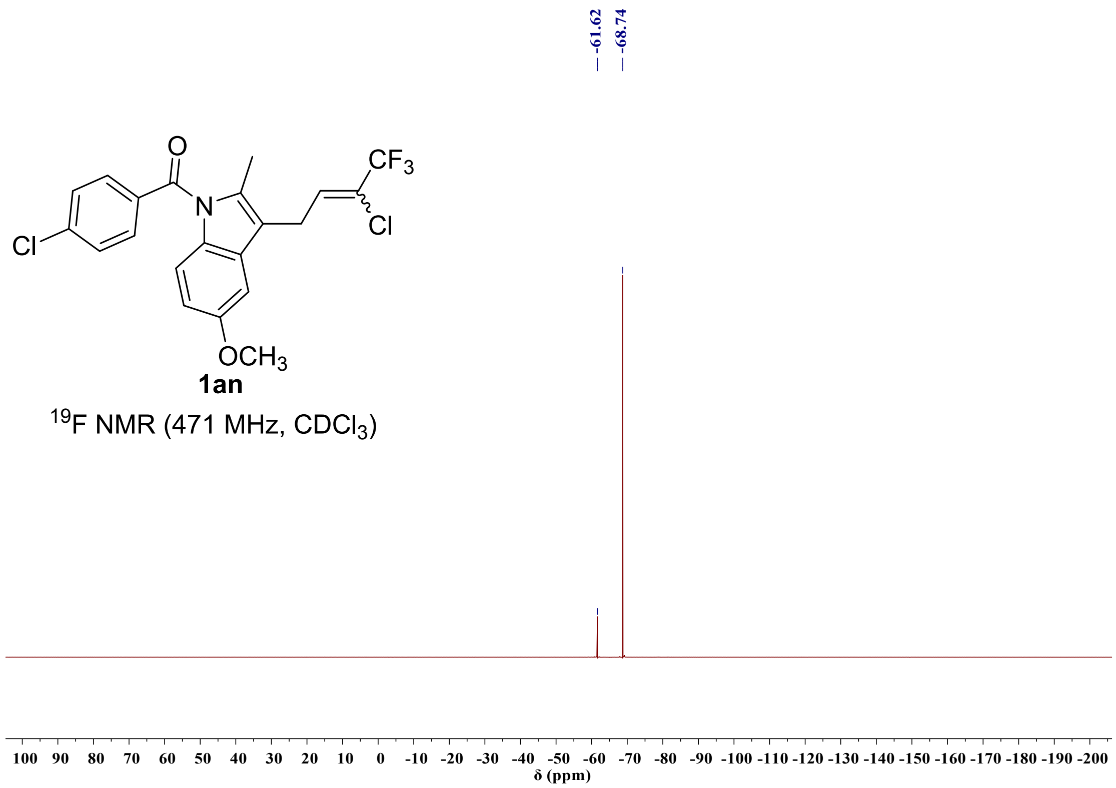

(3-(3-chloro-4,4,4-trifluorobut-2-en-1-yl)-5-methoxy-2-methyl-1H-indol-1-yl)(4-chlorophenyl)methanone (1an)

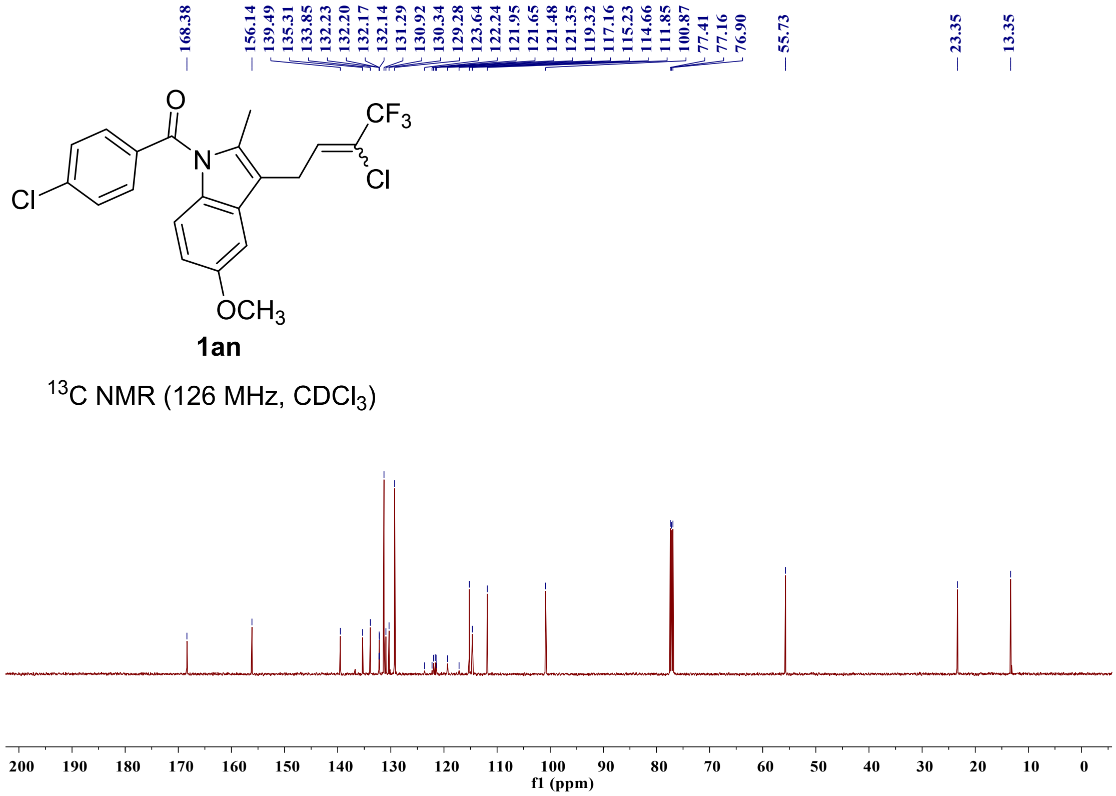

(Z)-(2-chloro-3,3,3-trifluoroprop-1-en-1-yl)benzene (1ao)

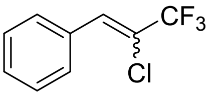

1ao

^1H NMR (500 MHz, $CDCl_3$)

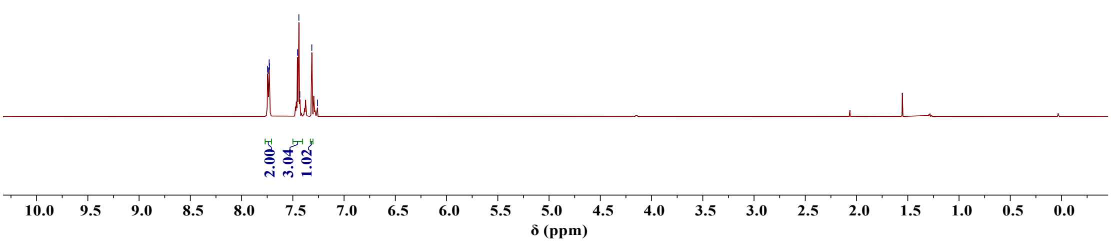

(Z)-(2-chloro-3,3,3-trifluoroprop-1-en-1-yl)benzene (1ao)

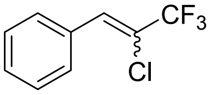

1ao

^{19}F NMR (471 MHz, $CDCl_3$)

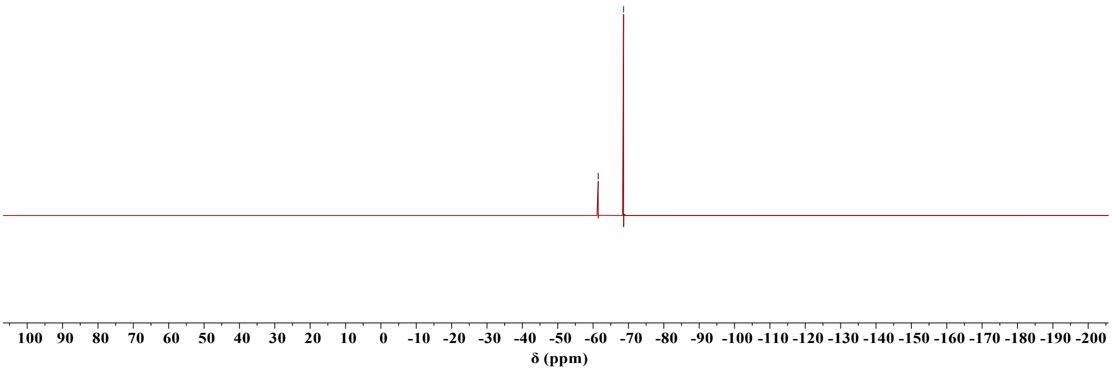

(Z)-(2-chloro-3,3,3-trifluoroprop-1-en-1-yl)benzene (1ao)

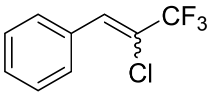

1ao

^{13}C NMR (126 MHz, $CDCl_3$)

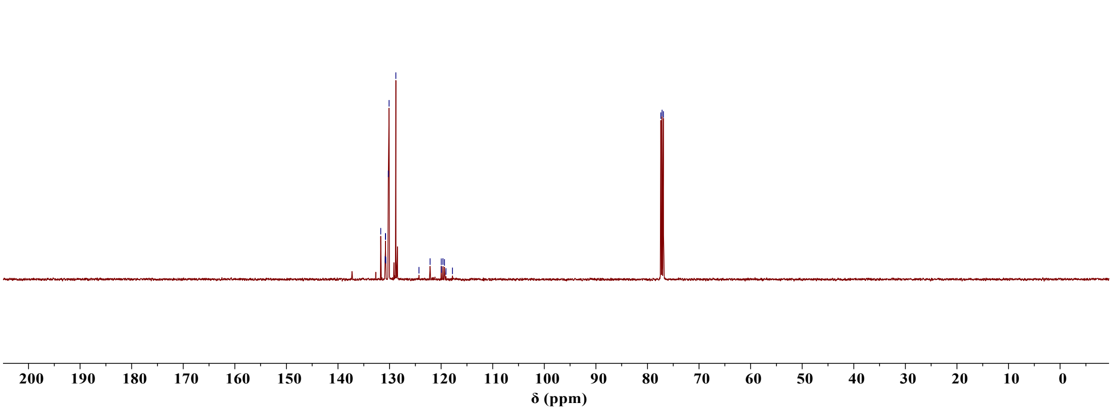

(Z)-1-(2-chloro-3,3,3-trifluoroprop-1-en-1-yl)-4-methylbenzene (1ap)

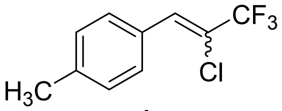

1ap

^{1}H NMR (500 MHz, $CDCl_3$)

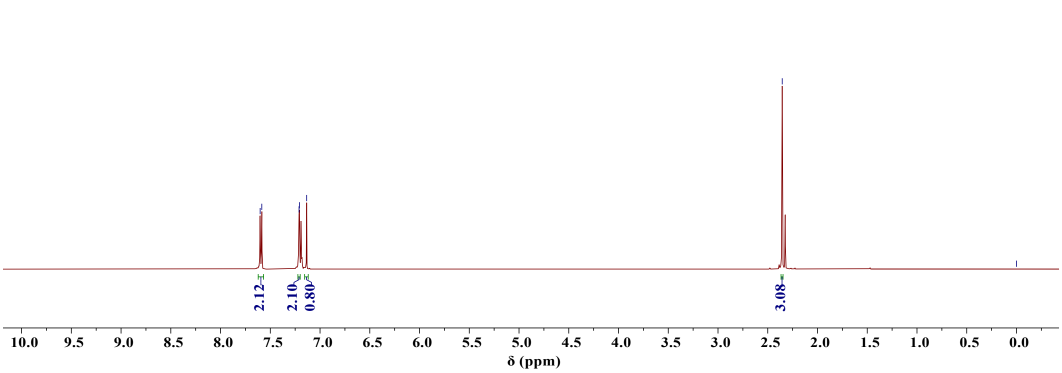

(Z)-1-(2-chloro-3,3,3-trifluoroprop-1-en-1-yl)-4-methylbenzene (1ap)

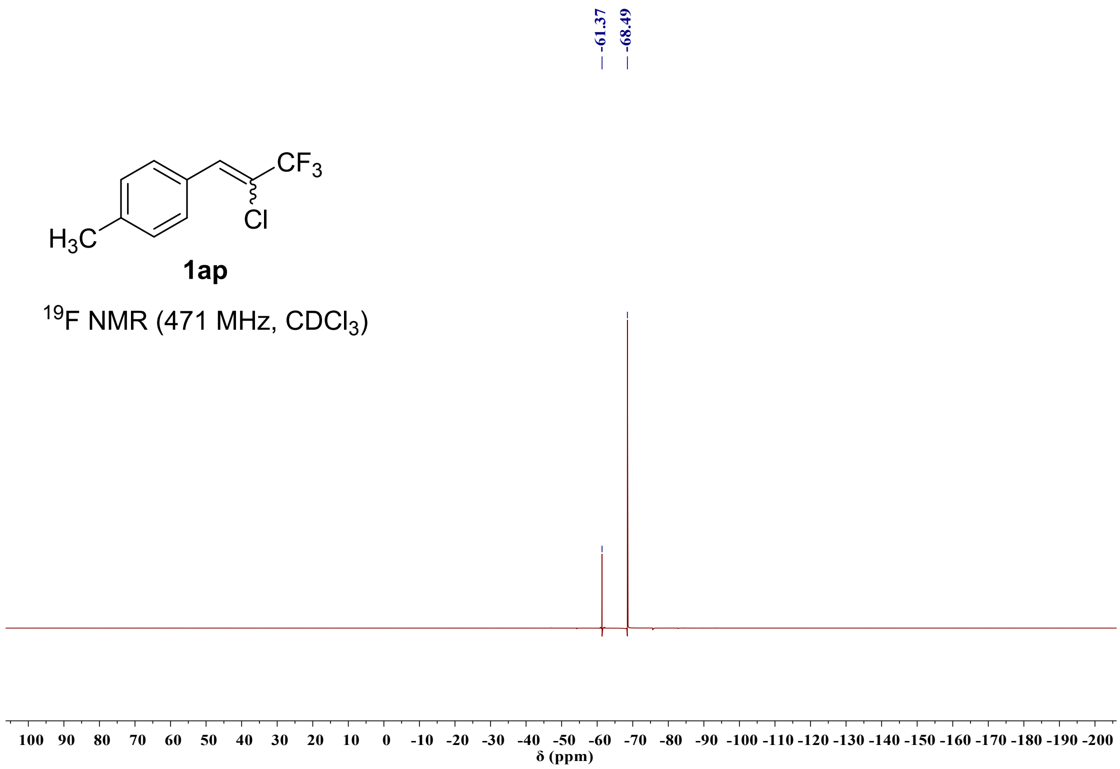

(Z)-1-(2-chloro-3,3,3-trifluoroprop-1-en-1-yl)-4-methylbenzene (1ap)

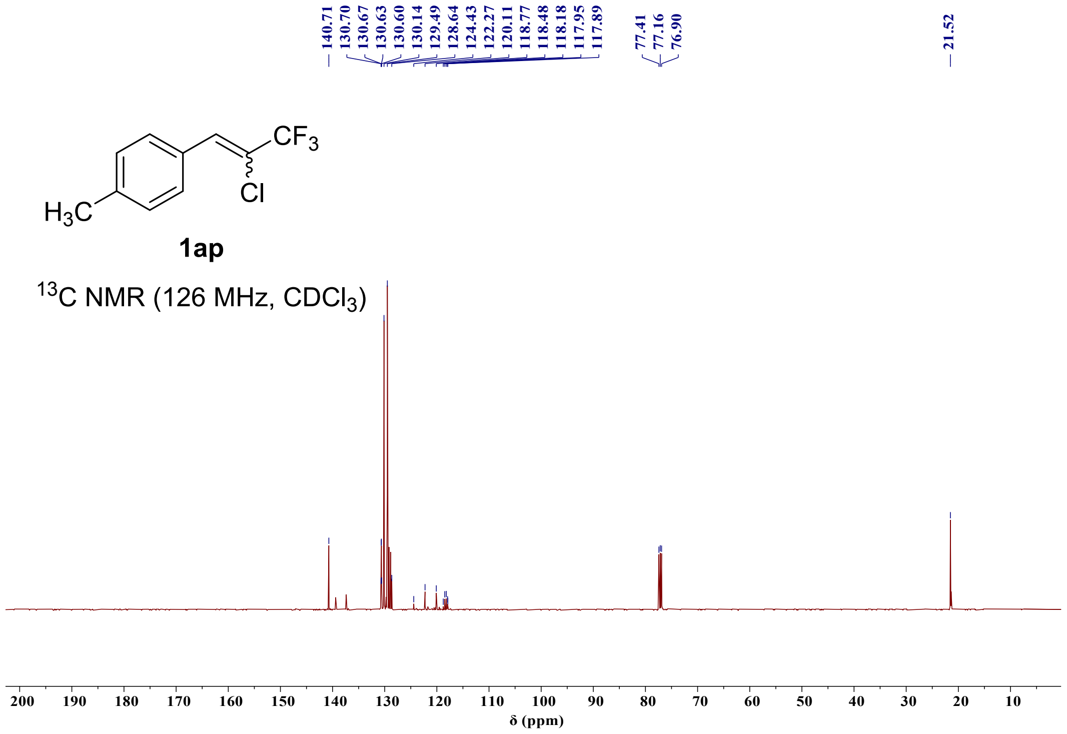

(Z)-1-(2-chloro-3,3,3-trifluoroprop-1-en-1-yl)-4-ethylbenzene (1aq)

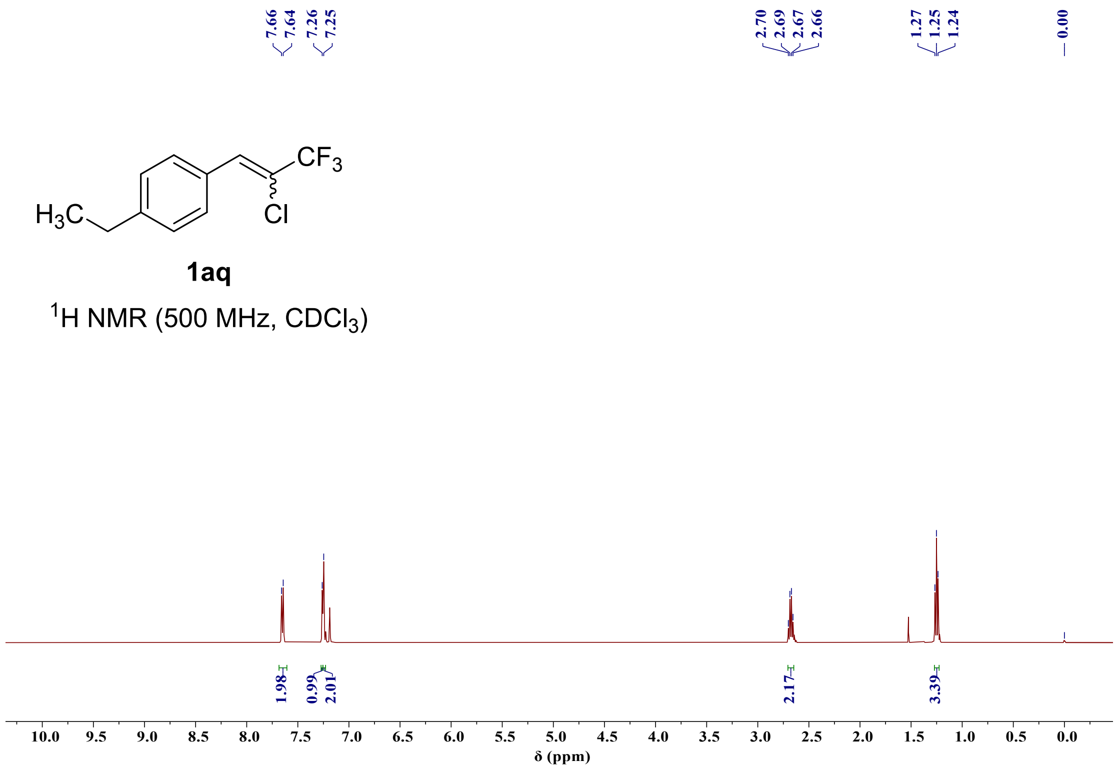

(Z)-1-(2-chloro-3,3,3-trifluoroprop-1-en-1-yl)-4-ethylbenzene (1aq)

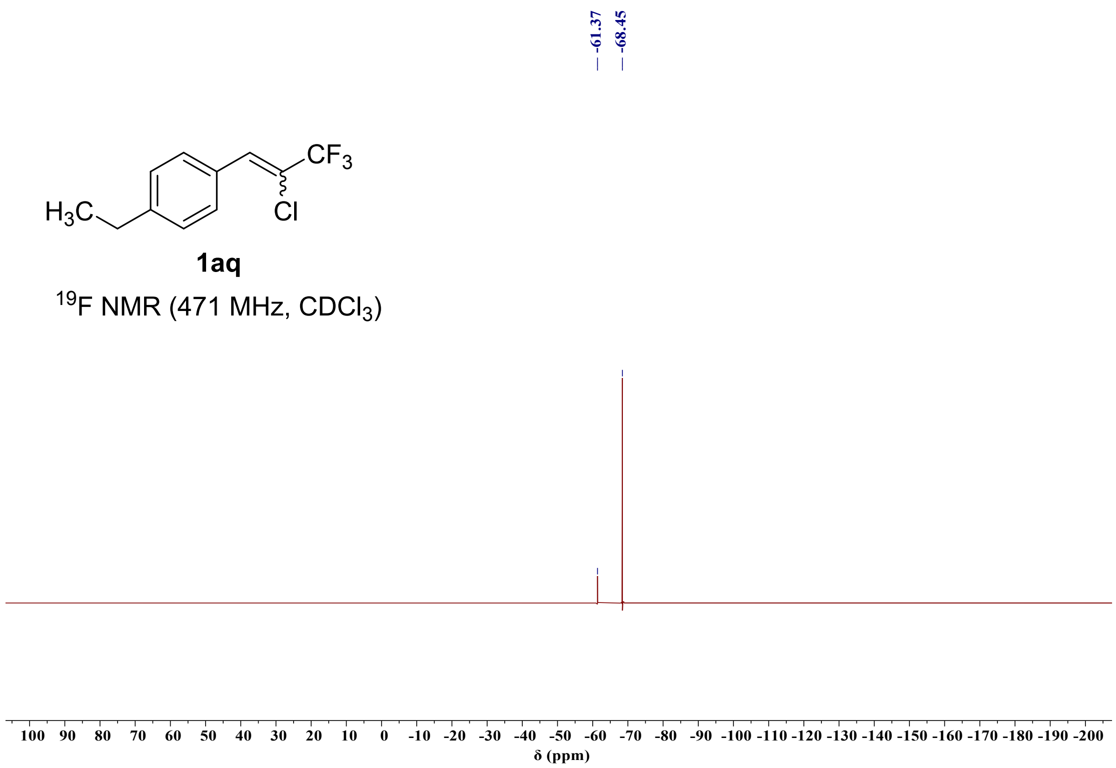

(Z)-1-(2-chloro-3,3,3-trifluoroprop-1-en-1-yl)-4-ethylbenzene (1aq)

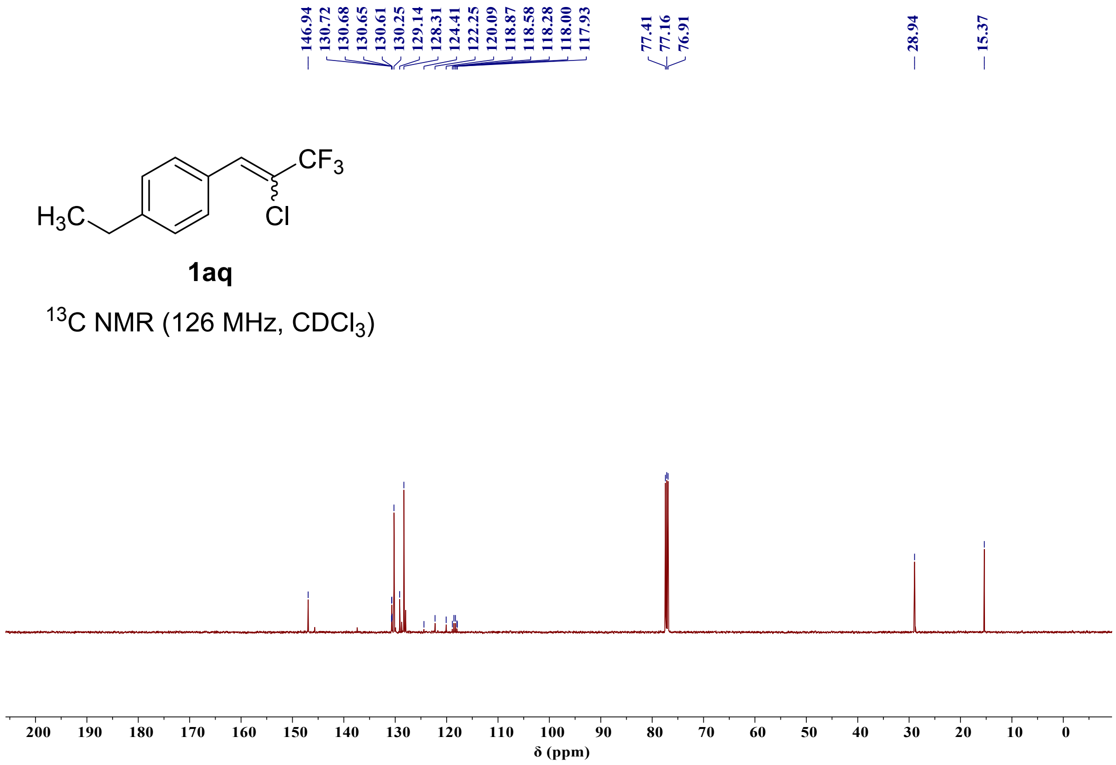

(Z)-1-(2-chloro-3,3,3-trifluoroprop-1-en-1-yl)-4-isopropylbenzene (1ar)

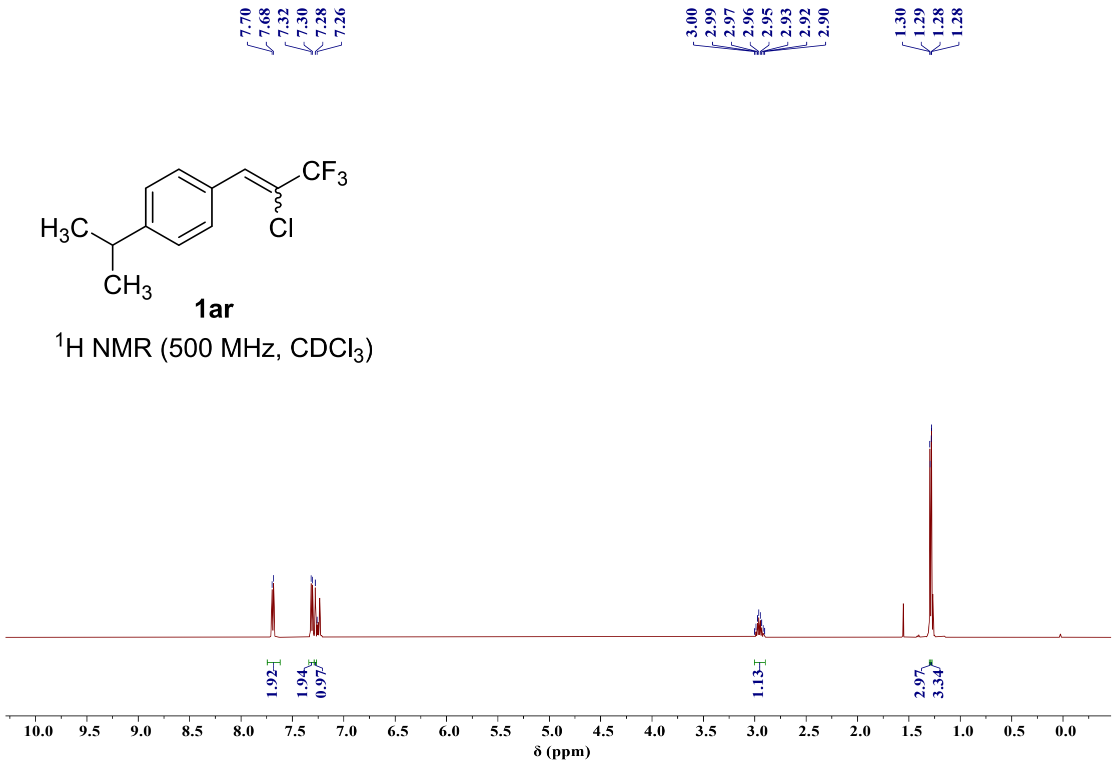

(Z)-1-(2-chloro-3,3,3-trifluoroprop-1-en-1-yl)-4-isopropylbenzene (1ar)

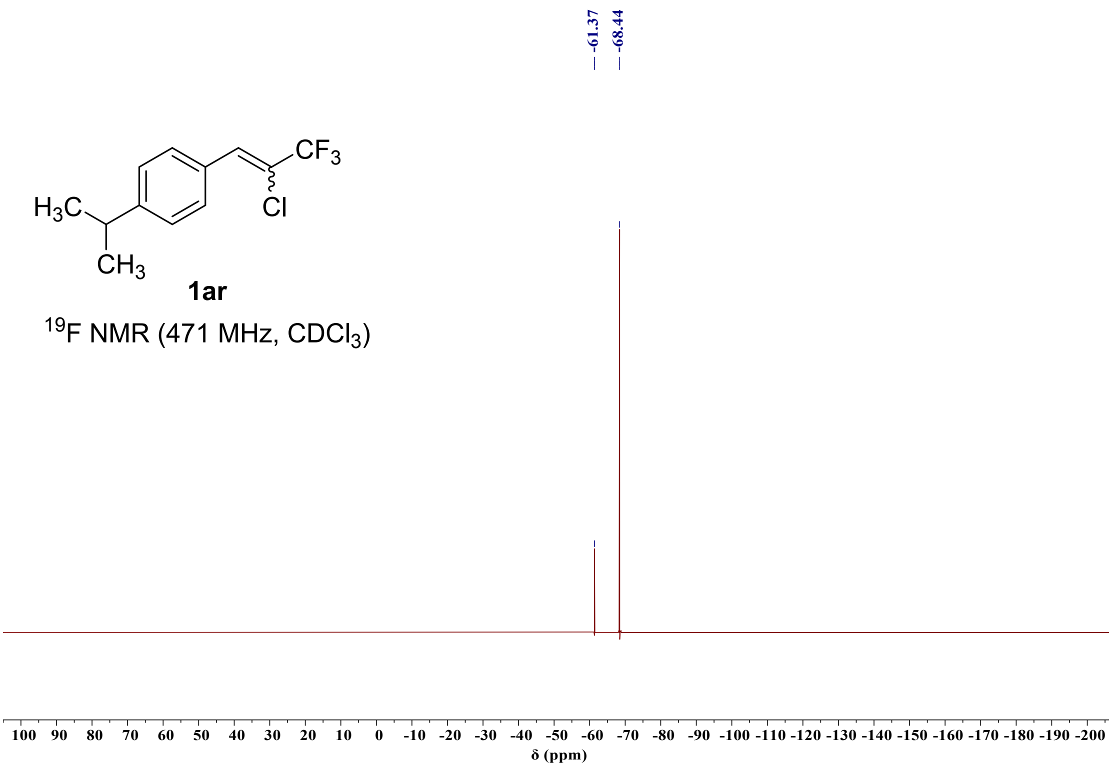

(Z)-1-(2-chloro-3,3,3-trifluoroprop-1-en-1-yl)-4-isopropylbenzene (1ar)

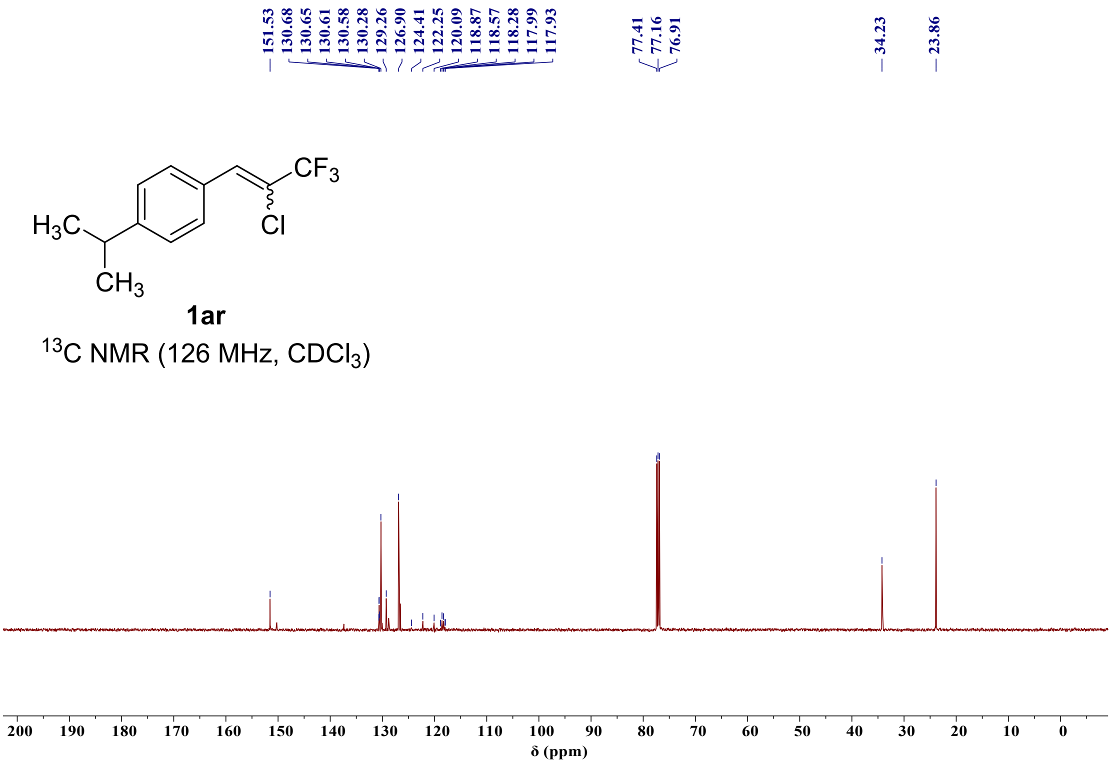

(Z)-1-(tert-butyl)-4-(2-chloro-3,3,3-trifluoroprop-1-en-1-yl)benzene (1as)

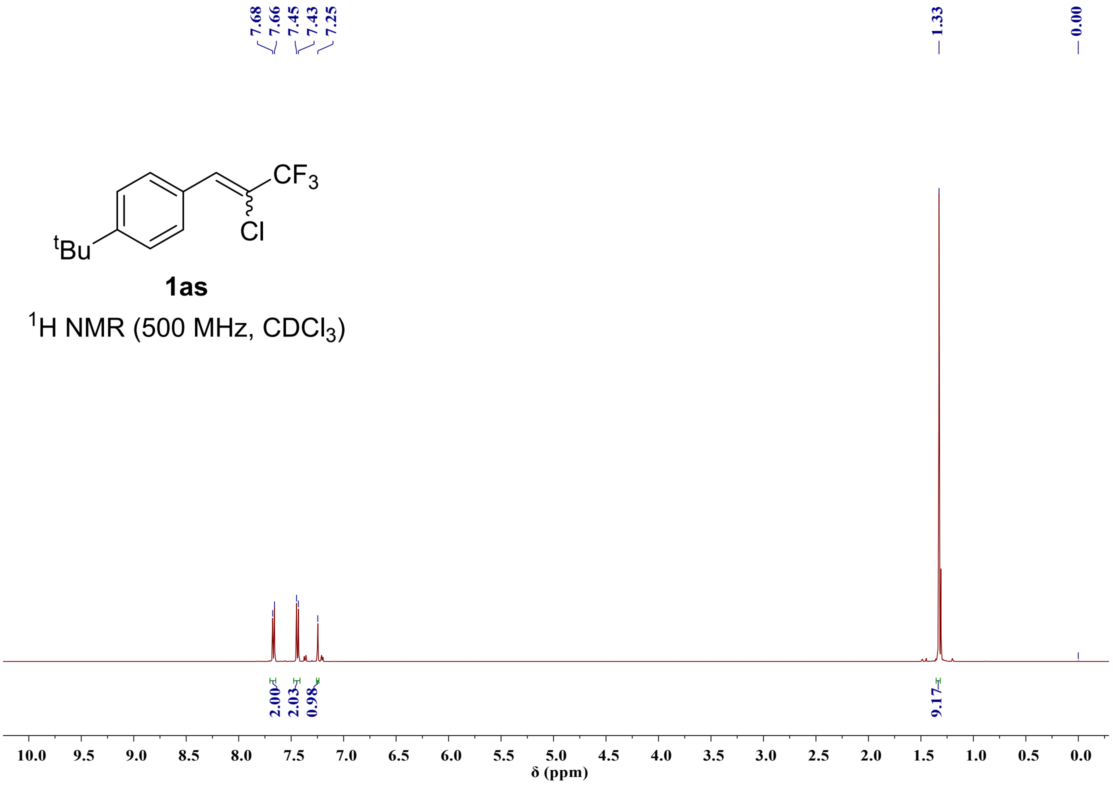

(Z)-1-(tert-butyl)-4-(2-chloro-3,3,3-trifluoroprop-1-en-1-yl)benzene (1as)

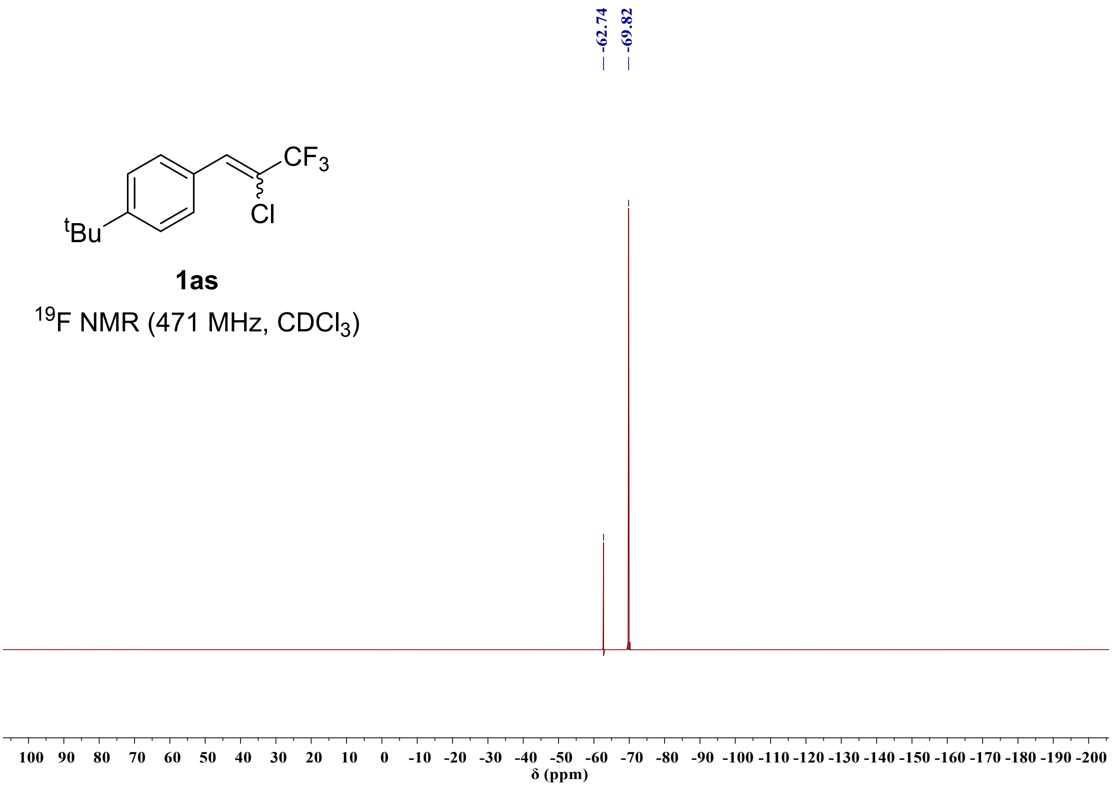

(Z)-1-(tert-butyl)-4-(2-chloro-3,3,3-trifluoroprop-1-en-1-yl)benzene (1as)

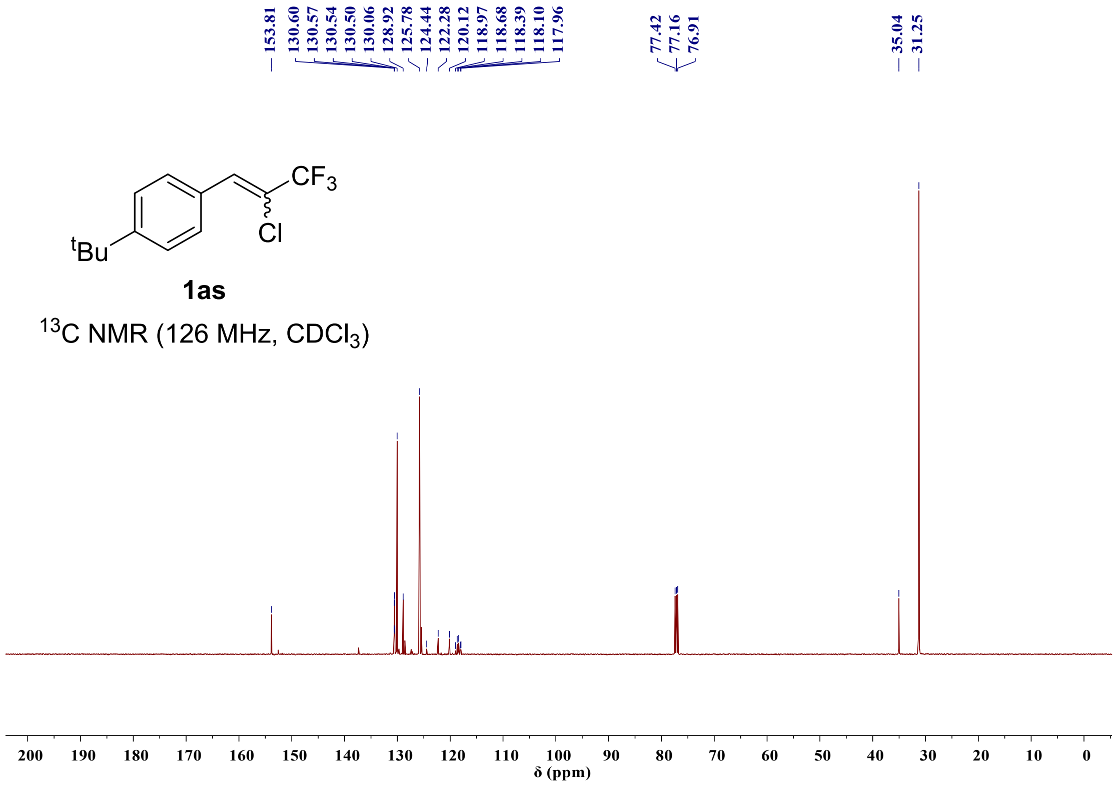

(Z)-1-(2-chloro-3,3,3-trifluoroprop-1-en-1-yl)-4-methoxybenzene (1at)

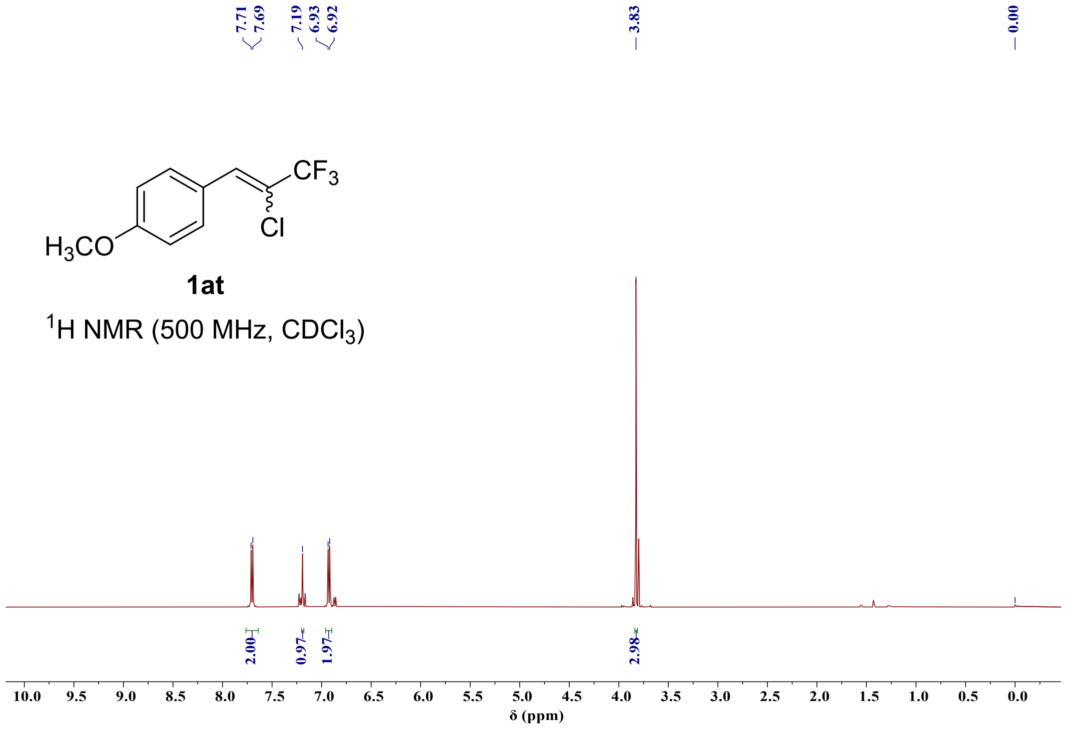

(Z)-1-(2-chloro-3,3,3-trifluoroprop-1-en-1-yl)-4-methoxybenzene (1at)

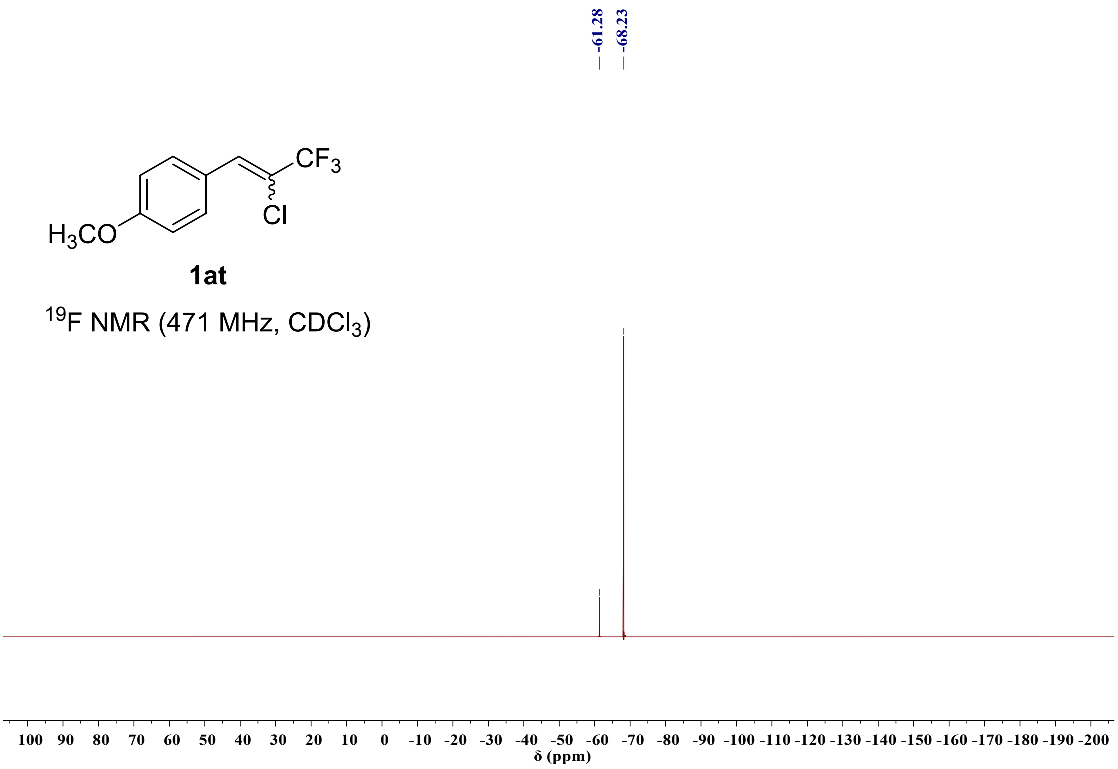

(Z)-1-(2-chloro-3,3,3-trifluoroprop-1-en-1-yl)-4-methoxybenzene (1at)

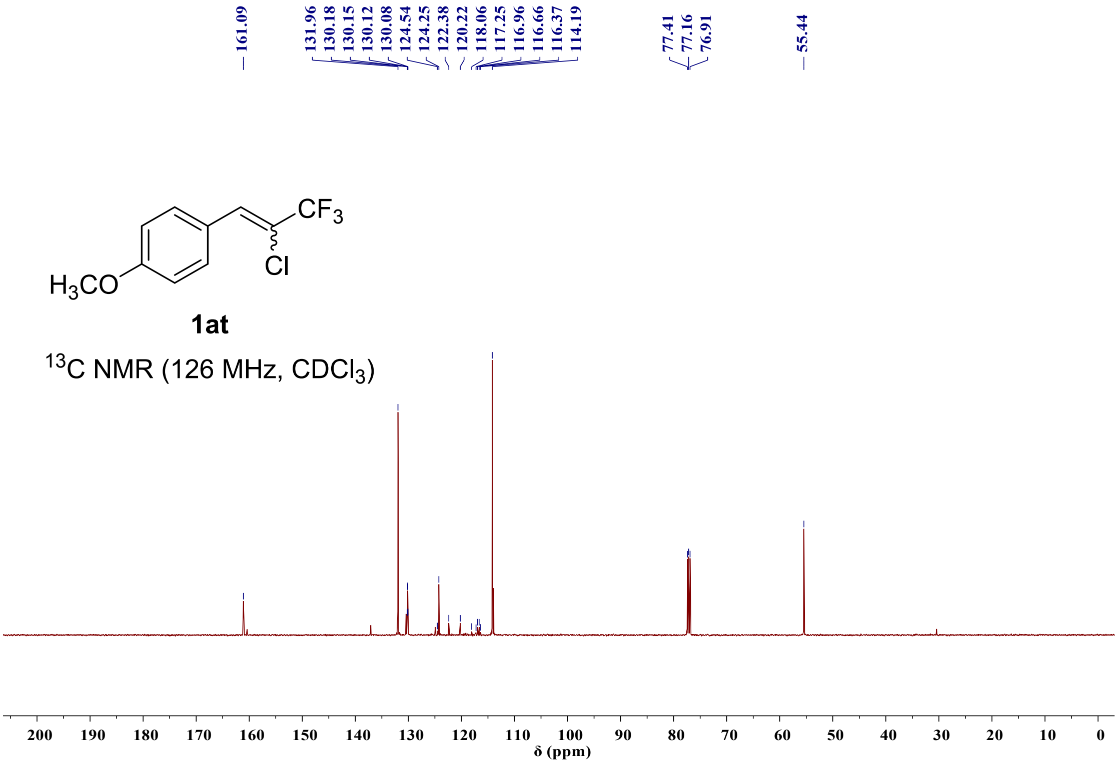

1-(2-chloro-3,3,3-trifluoroprop-1-en-1-yl)-4-(trifluoromethoxy)benzene (1au)

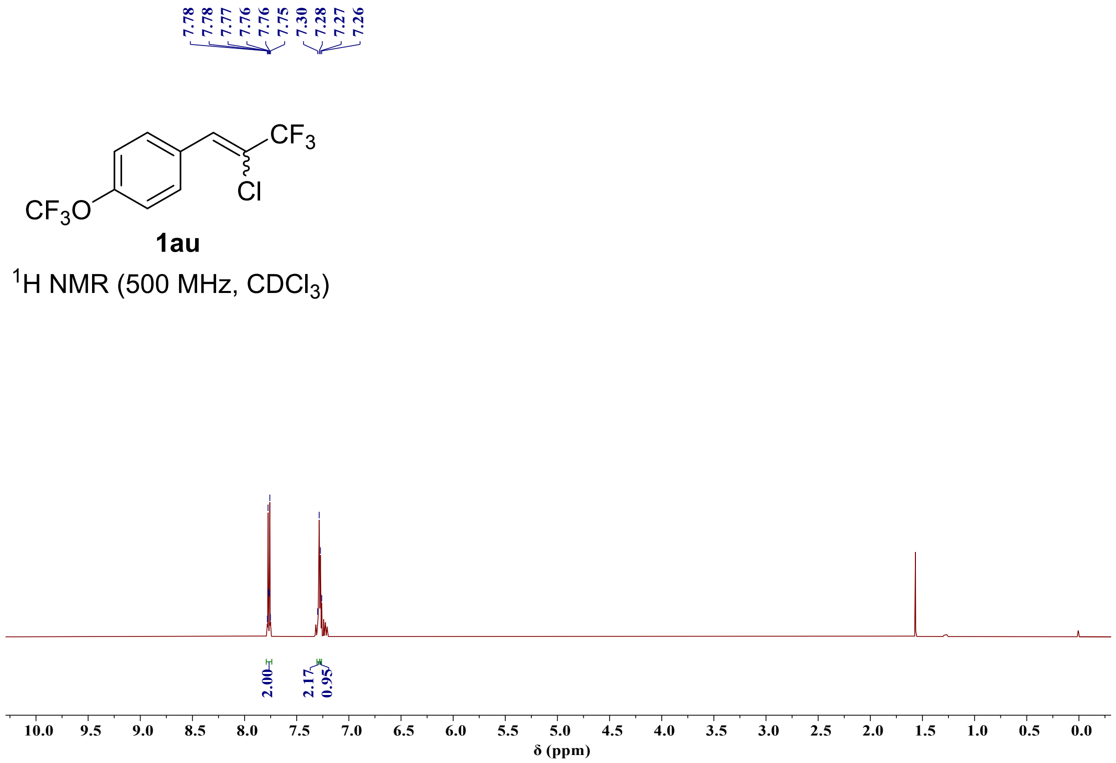

1-(2-chloro-3,3,3-trifluoroprop-1-en-1-yl)-4-(trifluoromethoxy)benzene (1au)

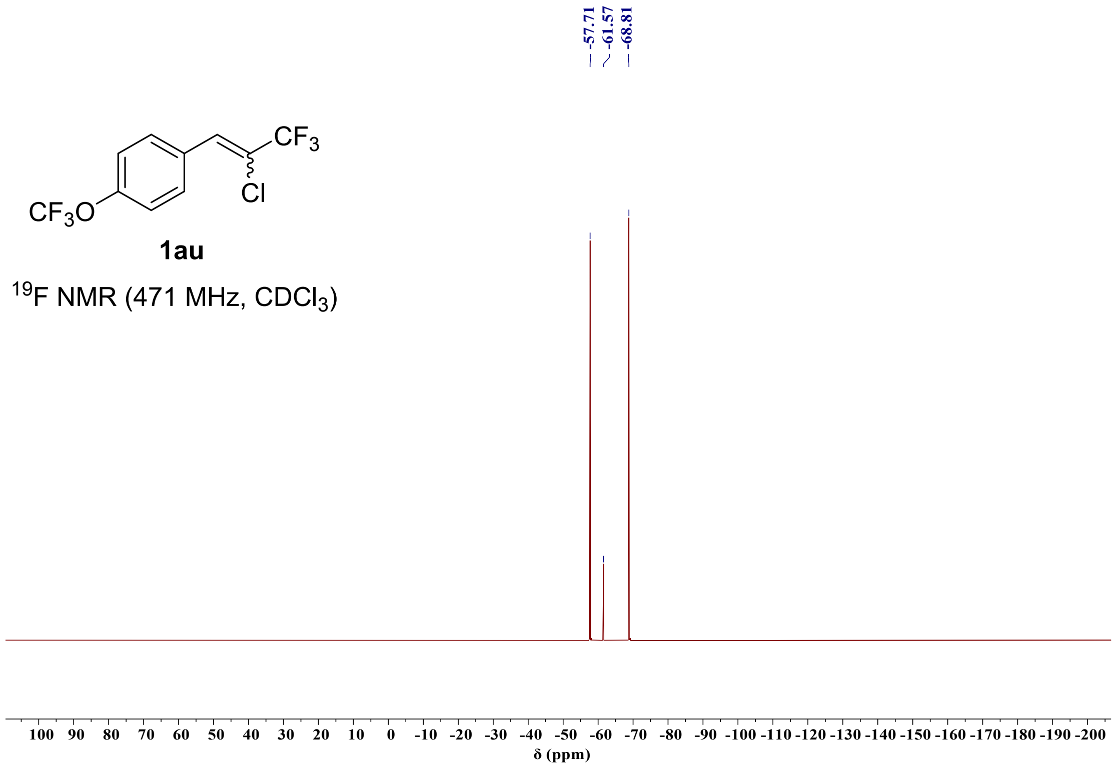

1-(2-chloro-3,3,3-trifluoroprop-1-en-1-yl)-4-(trifluoromethoxy)benzene (1au)

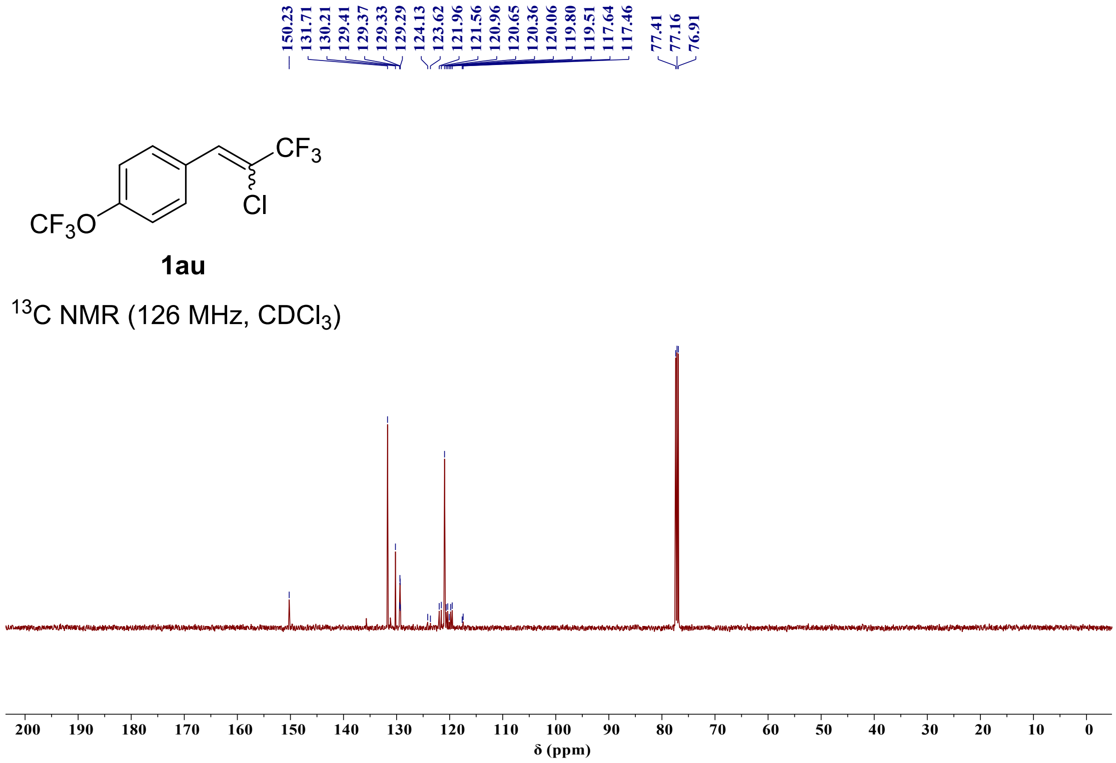

1-(benzyloxy)-4-(2-chloro-3,3,3-trifluoroprop-1-en-1-yl)benzene (1av)

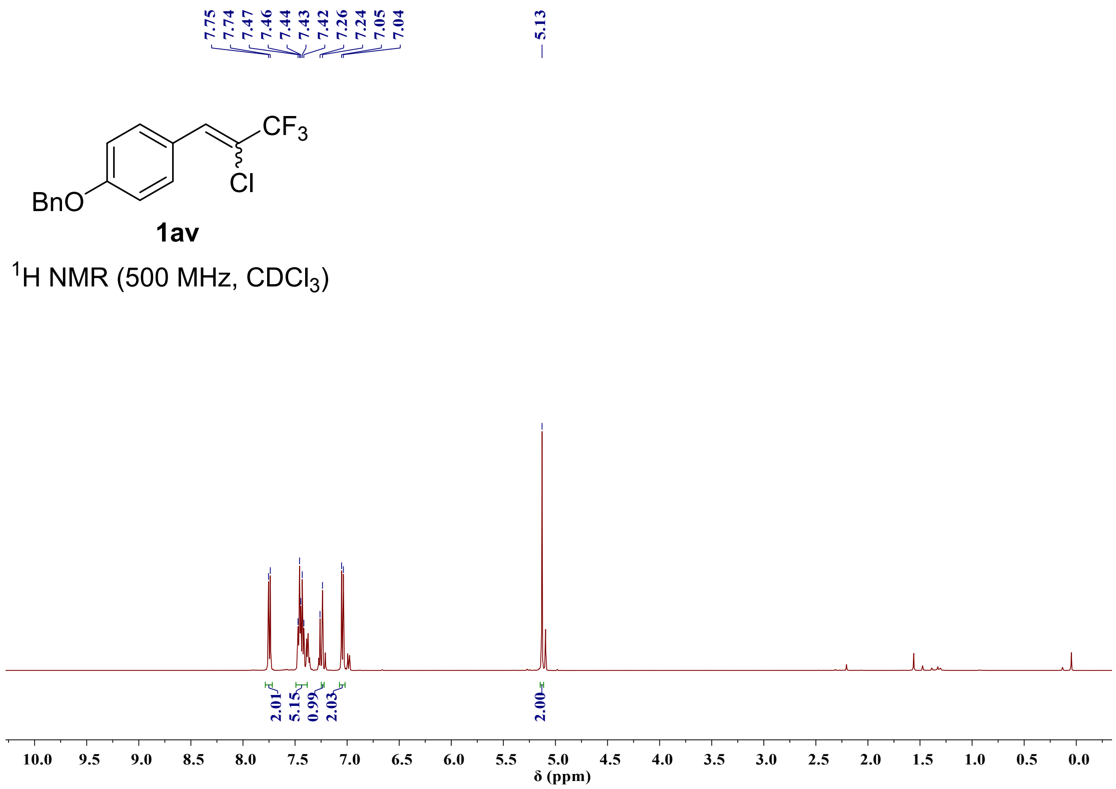

1-(benzyloxy)-4-(2-chloro-3,3,3-trifluoroprop-1-en-1-yl)benzene (1av)

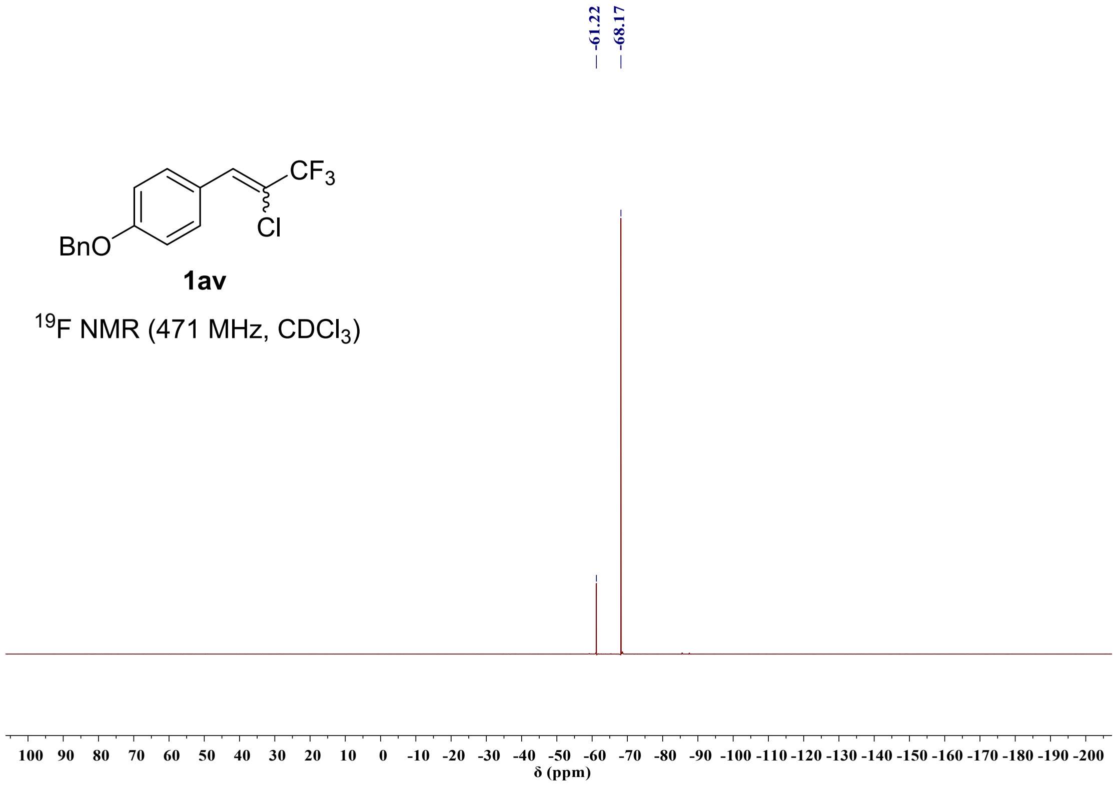

1-(benzyloxy)-4-(2-chloro-3,3,3-trifluoroprop-1-en-1-yl)benzene (1av)

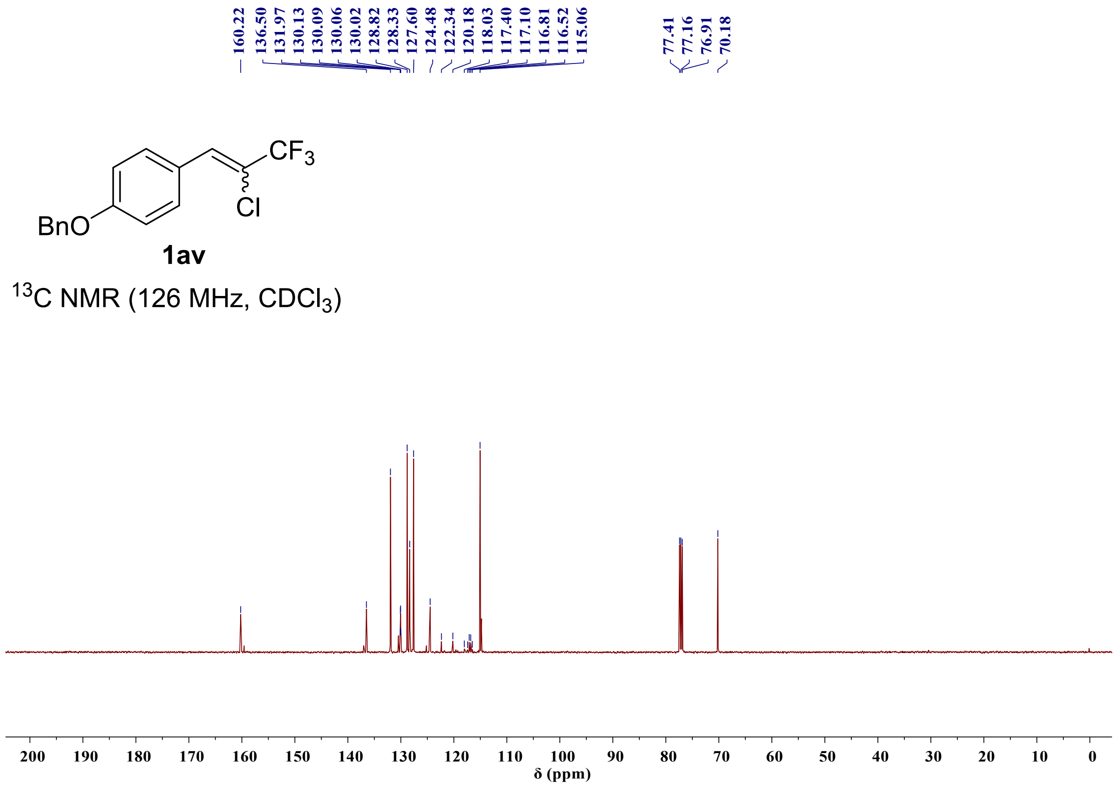

4-(2-chloro-3,3,3-trifluoroprop-1-en-1-yl)-1,1'-biphenyl (1aw)

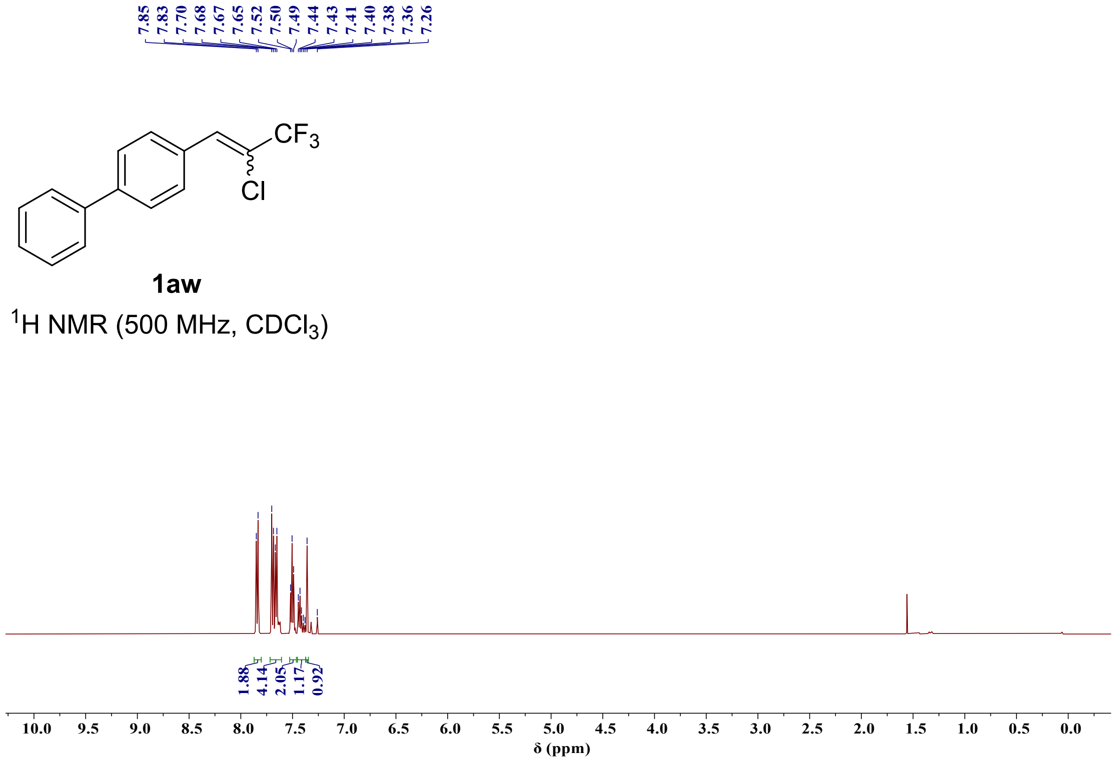

4-(2-chloro-3,3,3-trifluoroprop-1-en-1-yl)-1,1'-biphenyl (1aw)

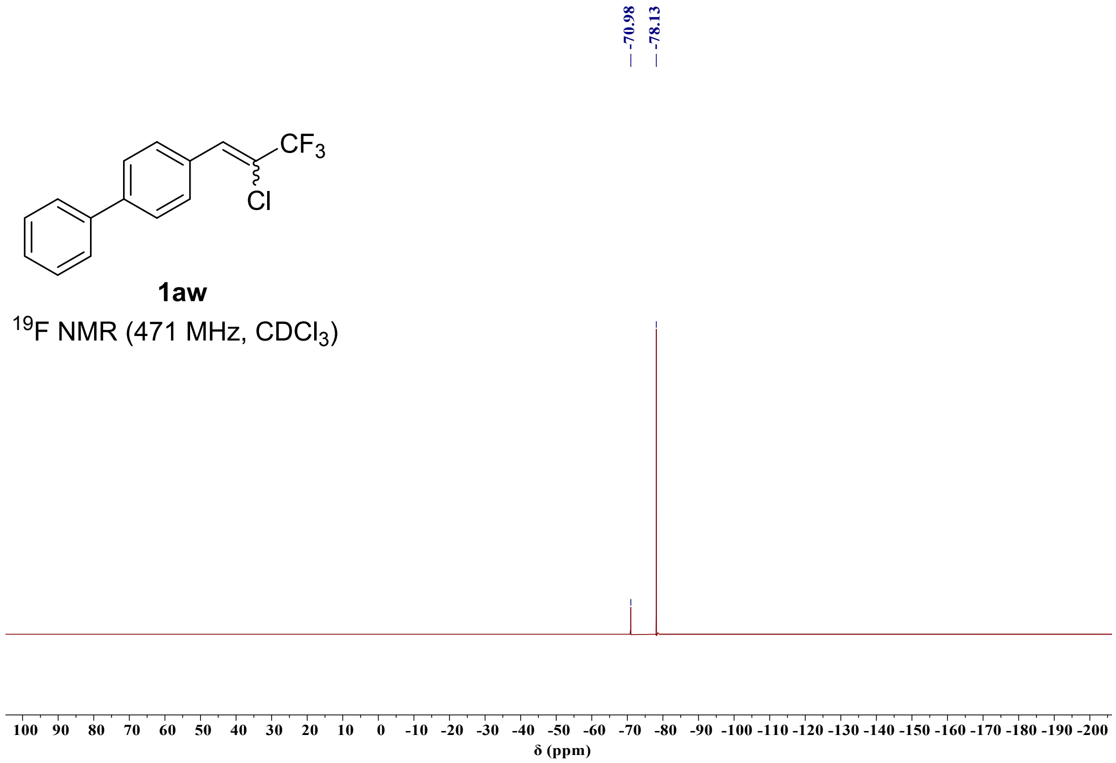

4-(2-chloro-3,3,3-trifluoroprop-1-en-1-yl)-1,1'-biphenyl (1aw)

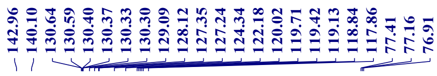

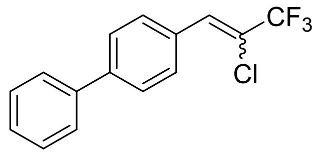

1aw

^{13}C NMR (126 MHz, $CDCl_3$)

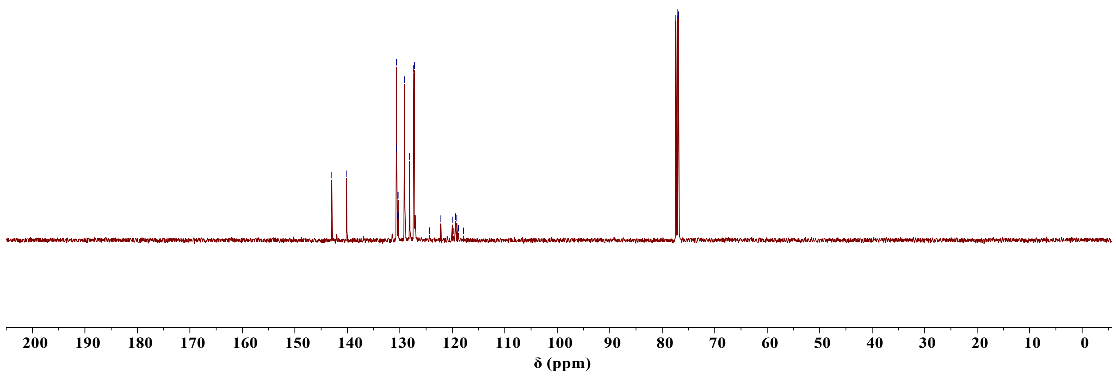

(4-(2-chloro-3,3,3-trifluoroprop-1-en-1-yl)phenyl)(methyl)sulfane (1ax)

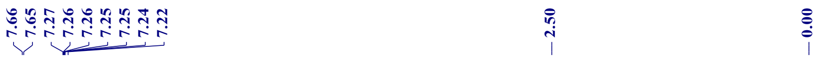

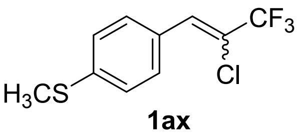

1ax

^{1}H NMR (600 MHz, $CDCl_3$)

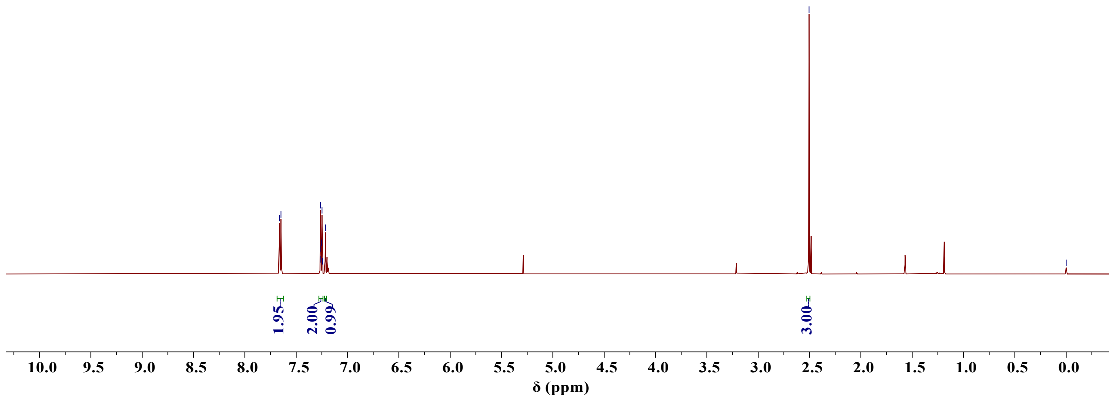

(4-(2-chloro-3,3,3-trifluoroprop-1-en-1-yl)phenyl)(methyl)sulfane (1ax)

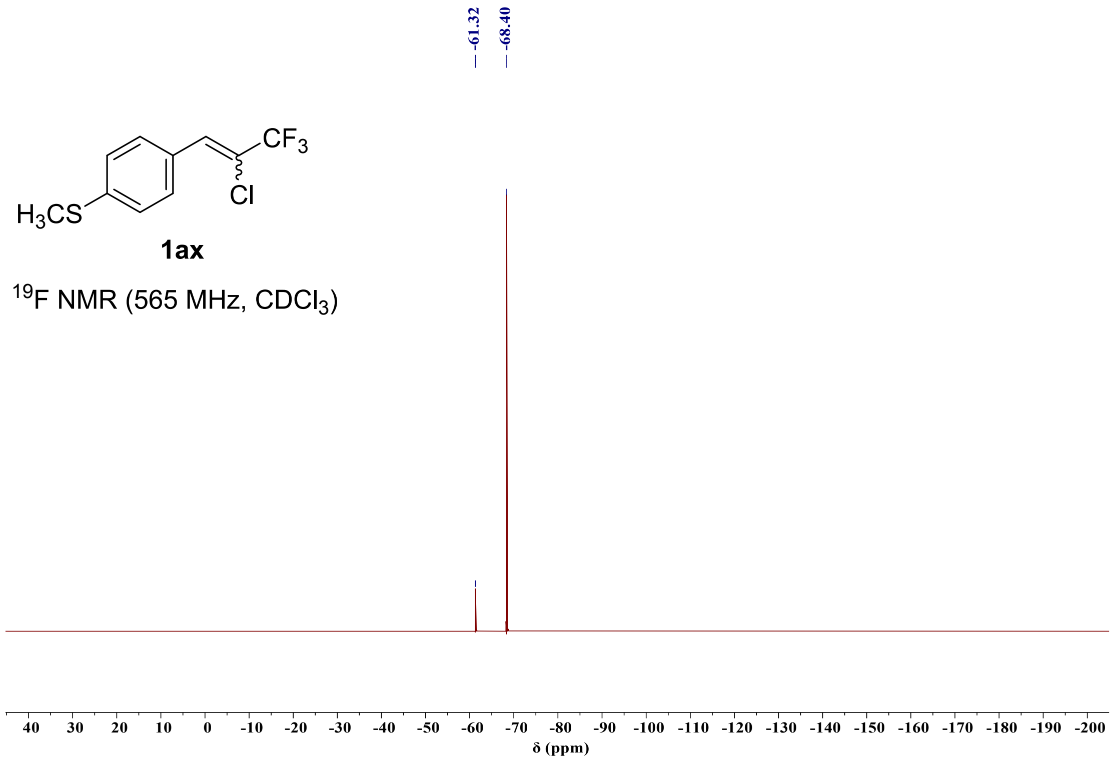

(4-(2-chloro-3,3,3-trifluoroprop-1-en-1-yl)phenyl)(methyl)sulfane (1ax)

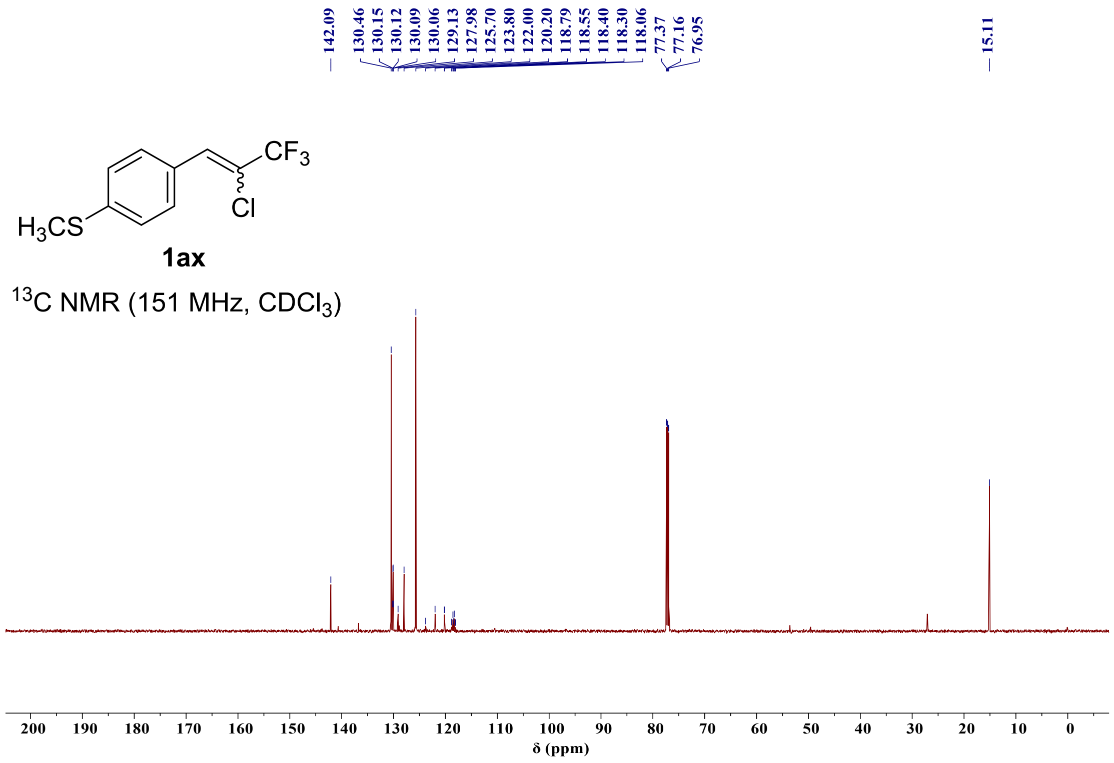

3-(2-chloro-3,3,3-trifluoroprop-1-en-1-yl)furan (1ay)

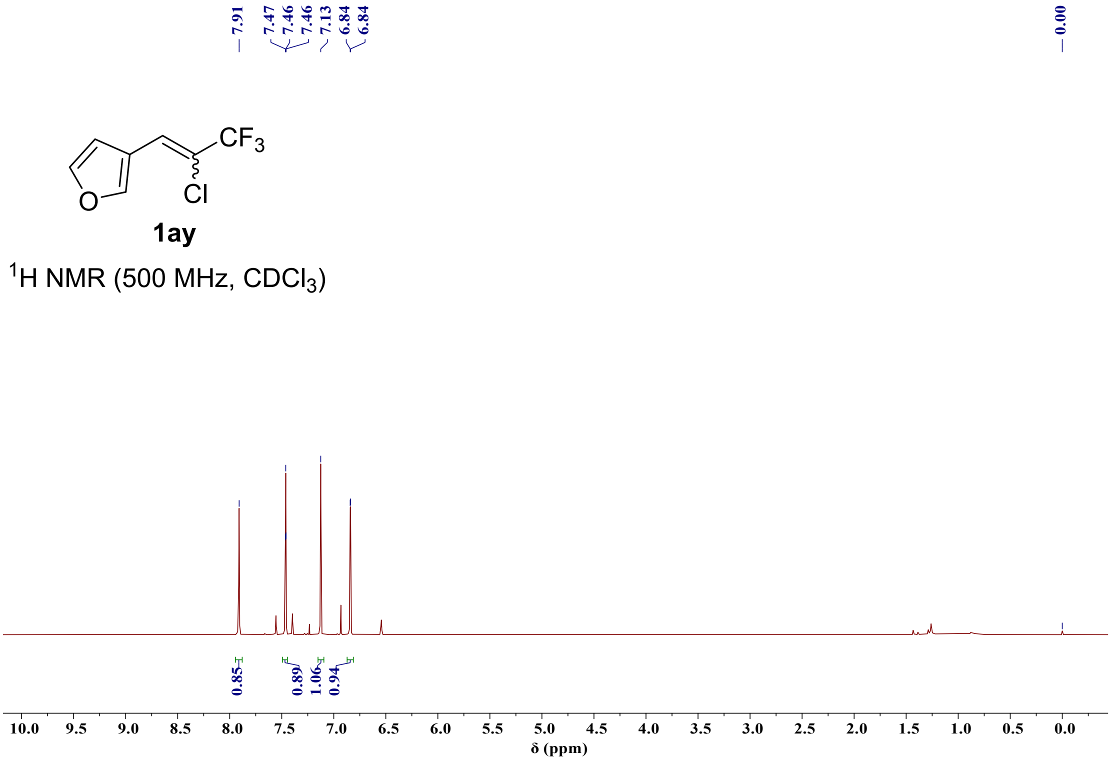

3-(2-chloro-3,3,3-trifluoroprop-1-en-1-yl)furan (1ay)

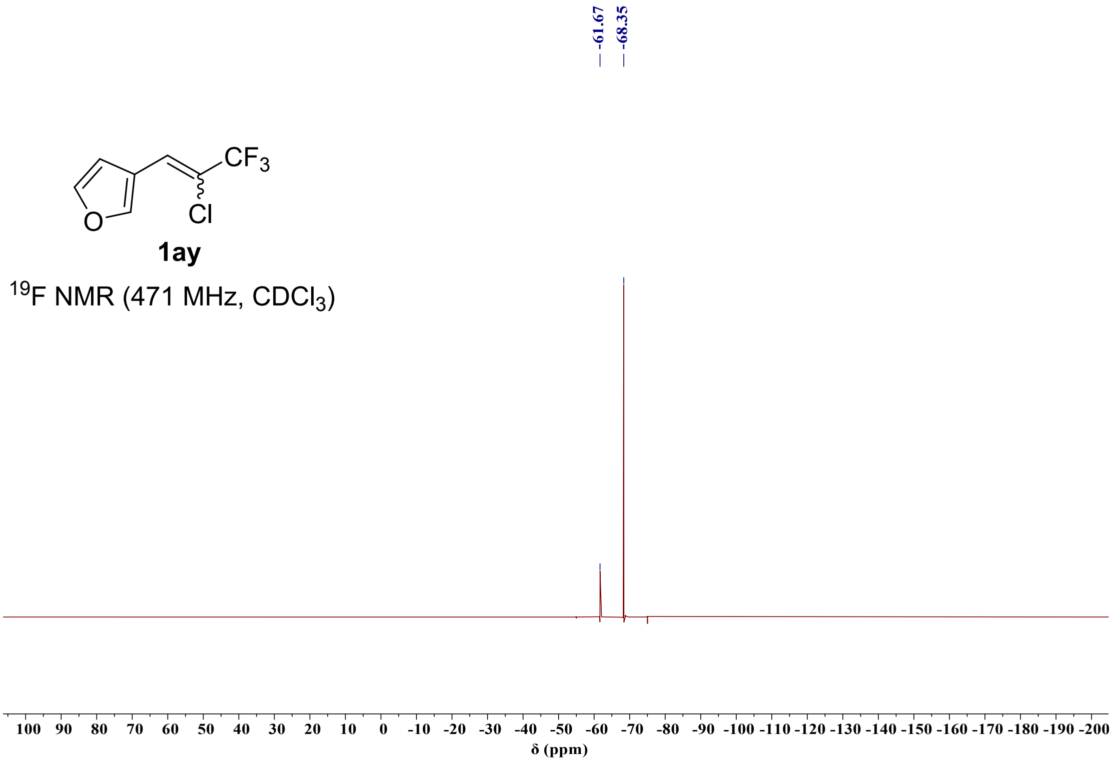

3-(2-chloro-3,3,3-trifluoroprop-1-en-1-yl)furan (1ay)

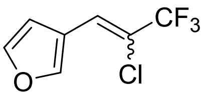

1ay

^{13}C NMR (151 MHz, $CDCl_3$)

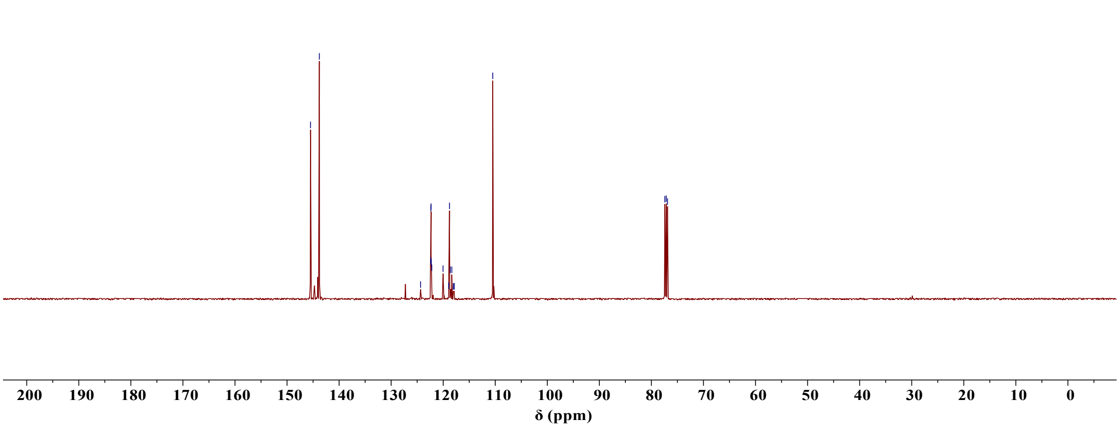

3-(2-chloro-3,3,3-trifluoroprop-1-en-1-yl)thiophene (1az)

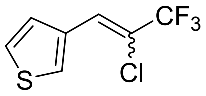

1az

^{1}H NMR (600 MHz, $CDCl_3$)

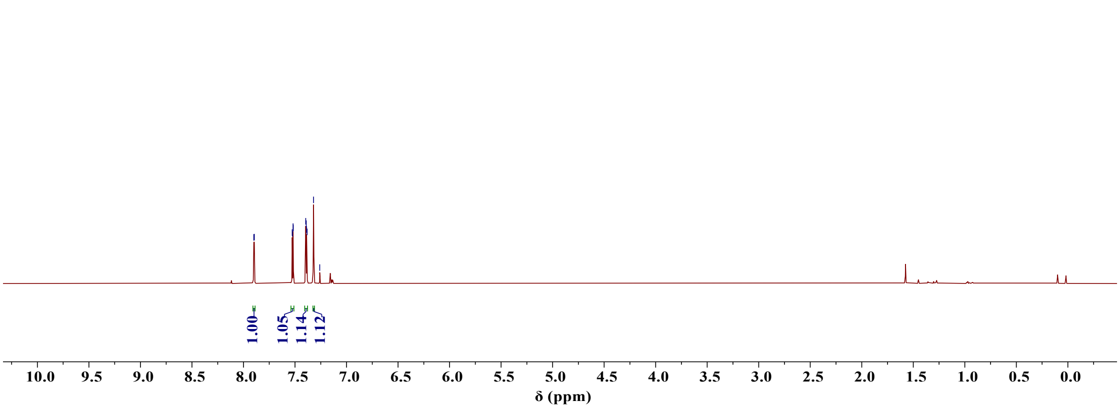

3-(2-chloro-3,3,3-trifluoroprop-1-en-1-yl)thiophene (1az)

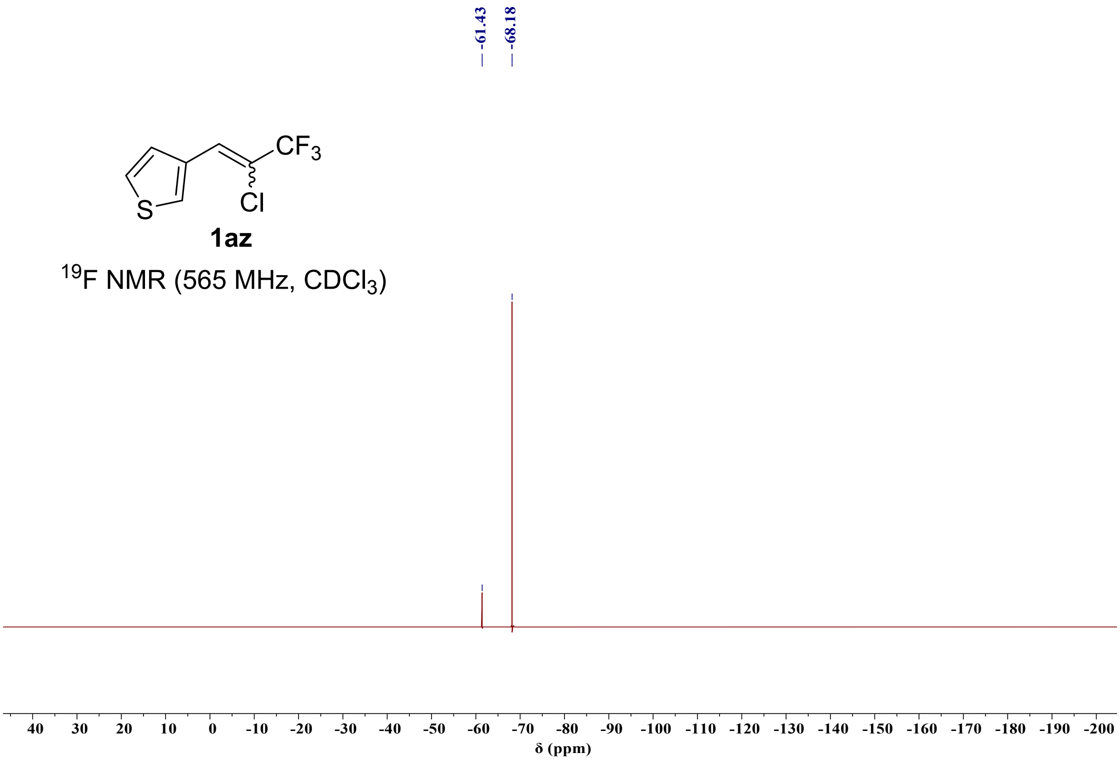

3-(2-chloro-3,3,3-trifluoroprop-1-en-1-yl)thiophene (1az)

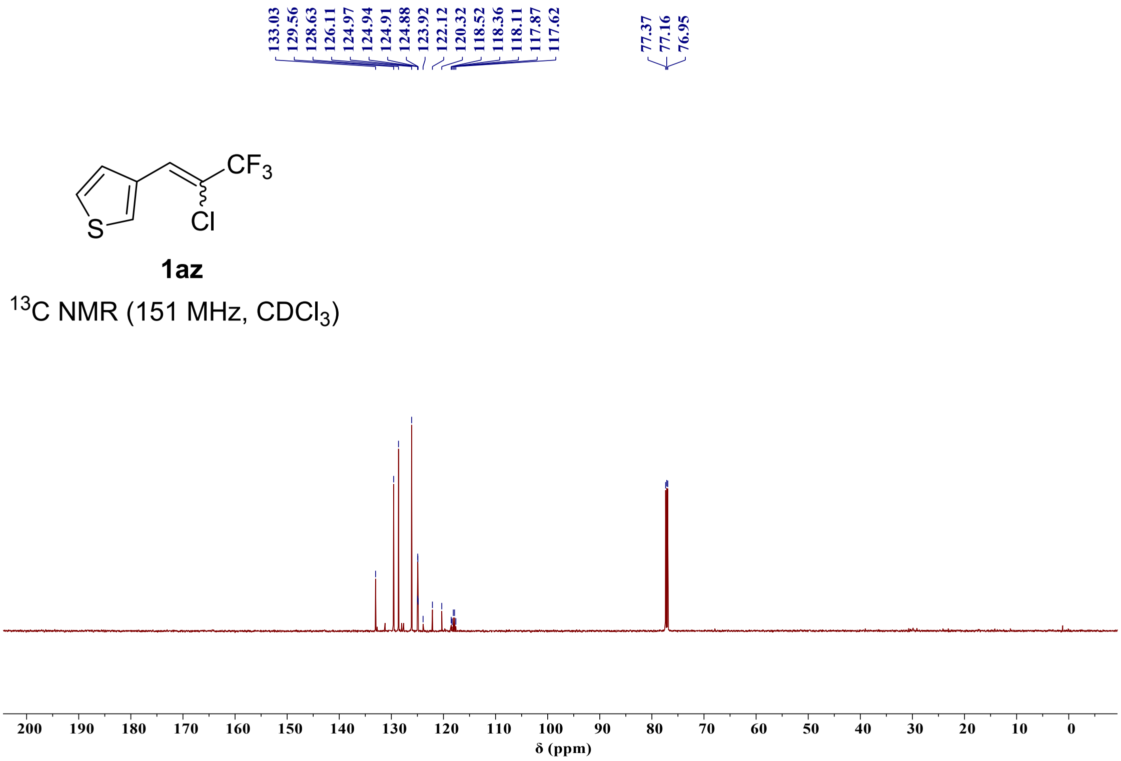

5-(2-chloro-3,3,3-trifluoroprop-1-en-1-yl)-2,3-dihydrobenzofuran (1aaa)

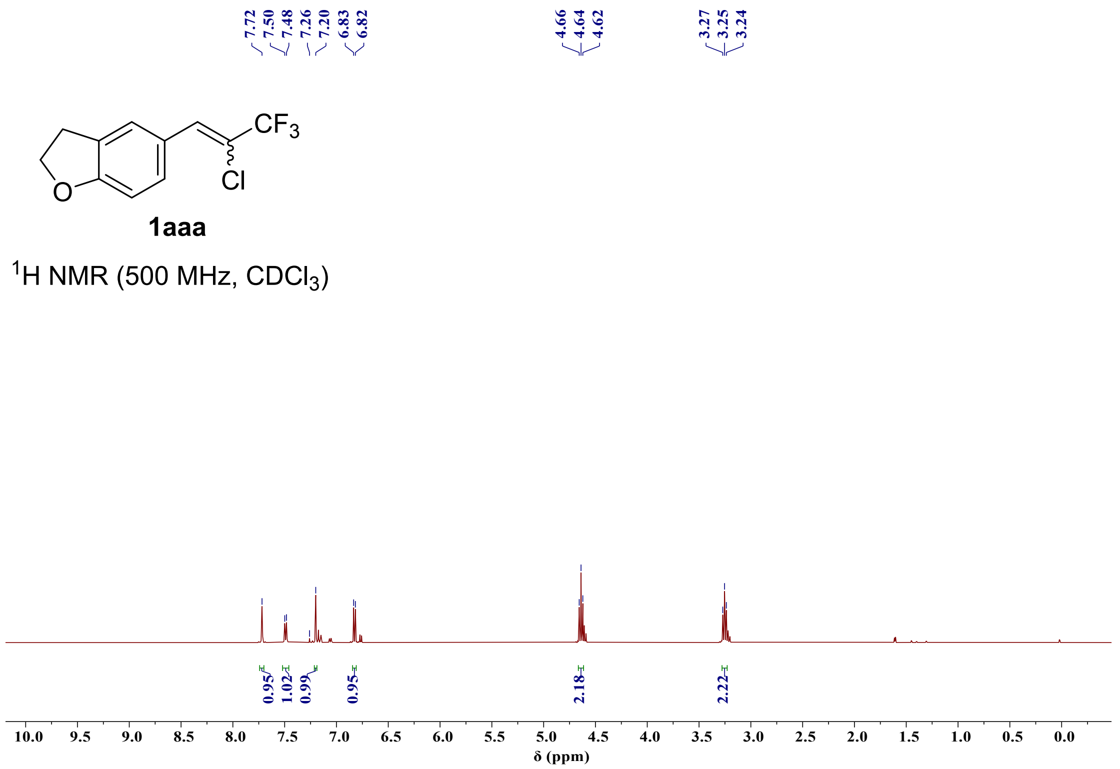

5-(2-chloro-3,3,3-trifluoroprop-1-en-1-yl)-2,3-dihydrobenzofuran (1aaa)

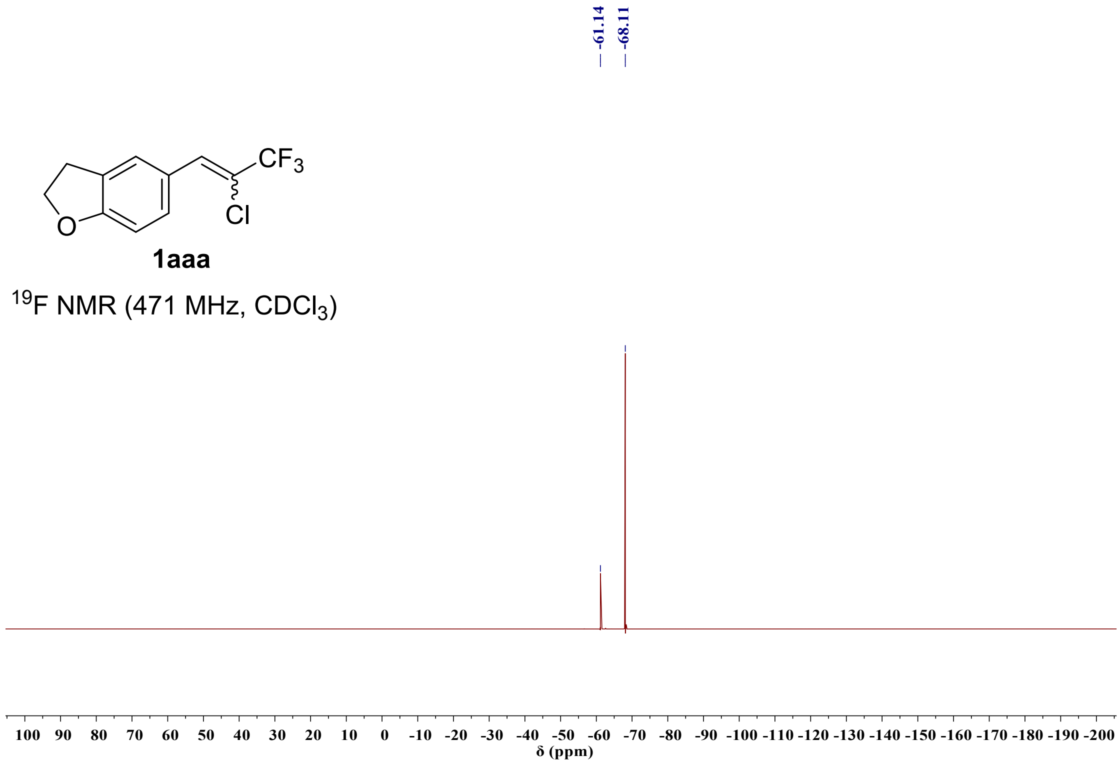

5-(2-chloro-3,3,3-trifluoroprop-1-en-1-yl)-2,3-dihydrobenzofuran (1aaa)

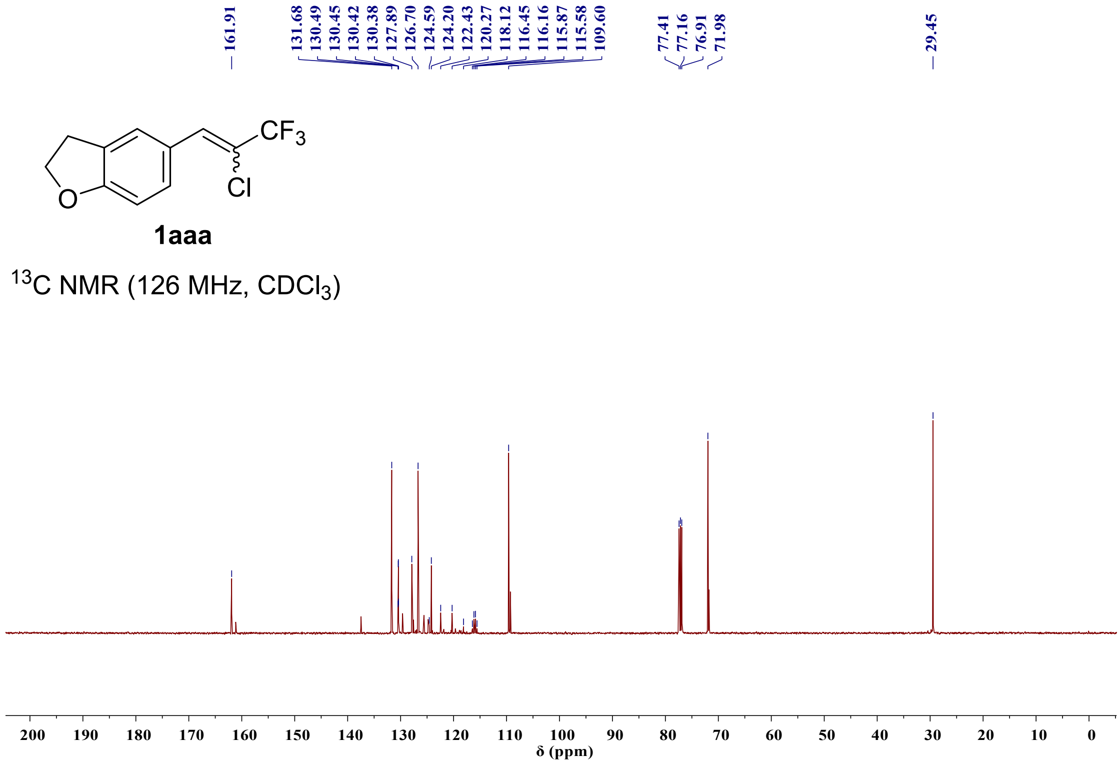

4-(2-chloro-3,3,3-trifluoroprop-1-en-1-yl)-1,2-dimethylbenzene (1aab)

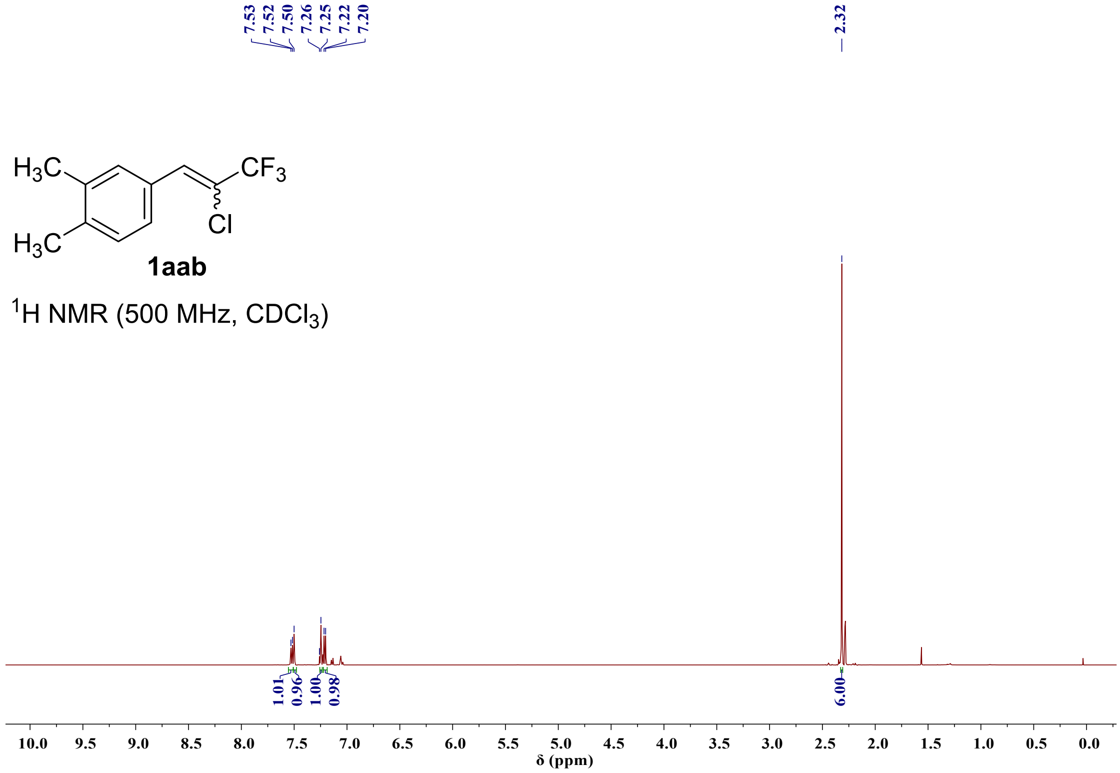

.

4-(2-chloro-3,3,3-trifluoroprop-1-en-1-yl)-1,2-dimethylbenzene (1aab)

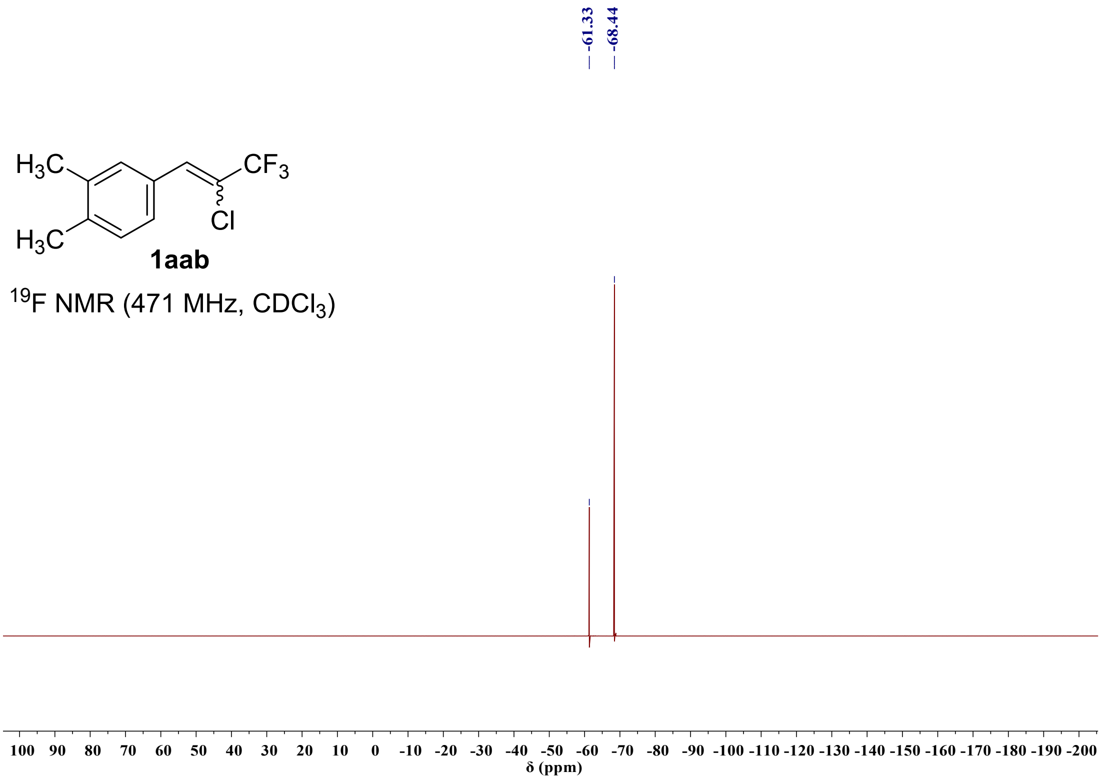

4-(2-chloro-3,3,3-trifluoroprop-1-en-1-yl)-1,2-dimethylbenzene (1aab)

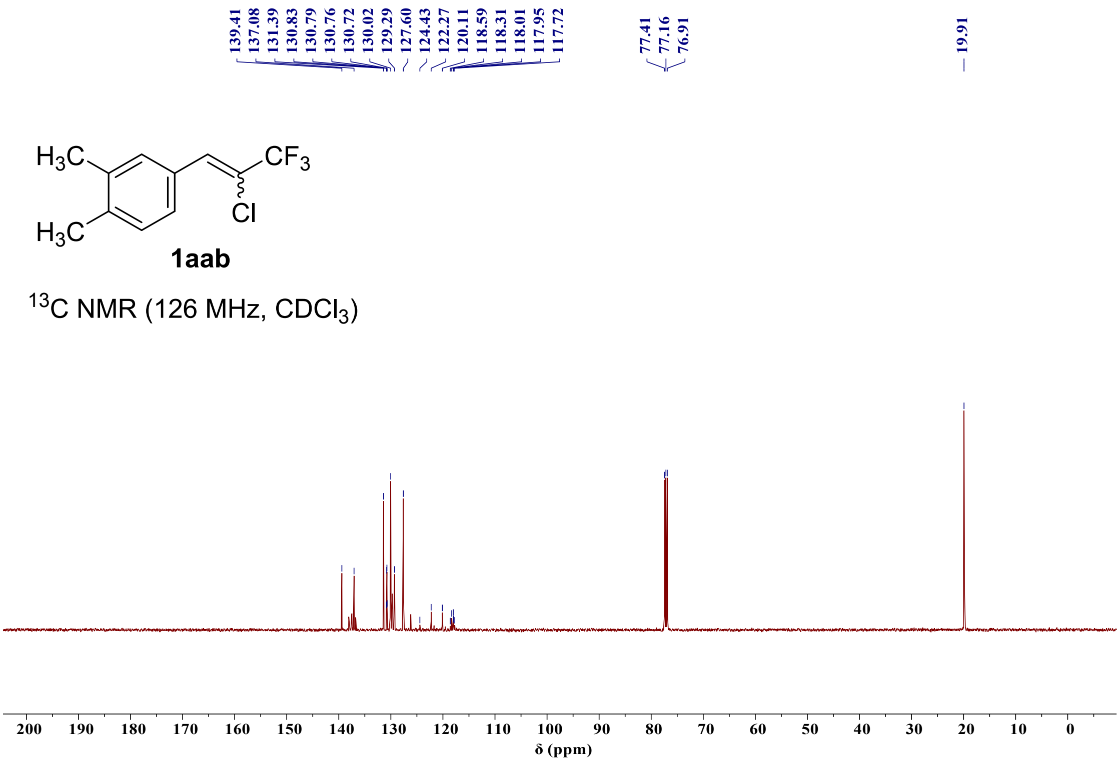

(Z)-1,1,1-trifluoro-4-phenylbut-2-en-2-yl trifluoromethanesulfonate (1aac)

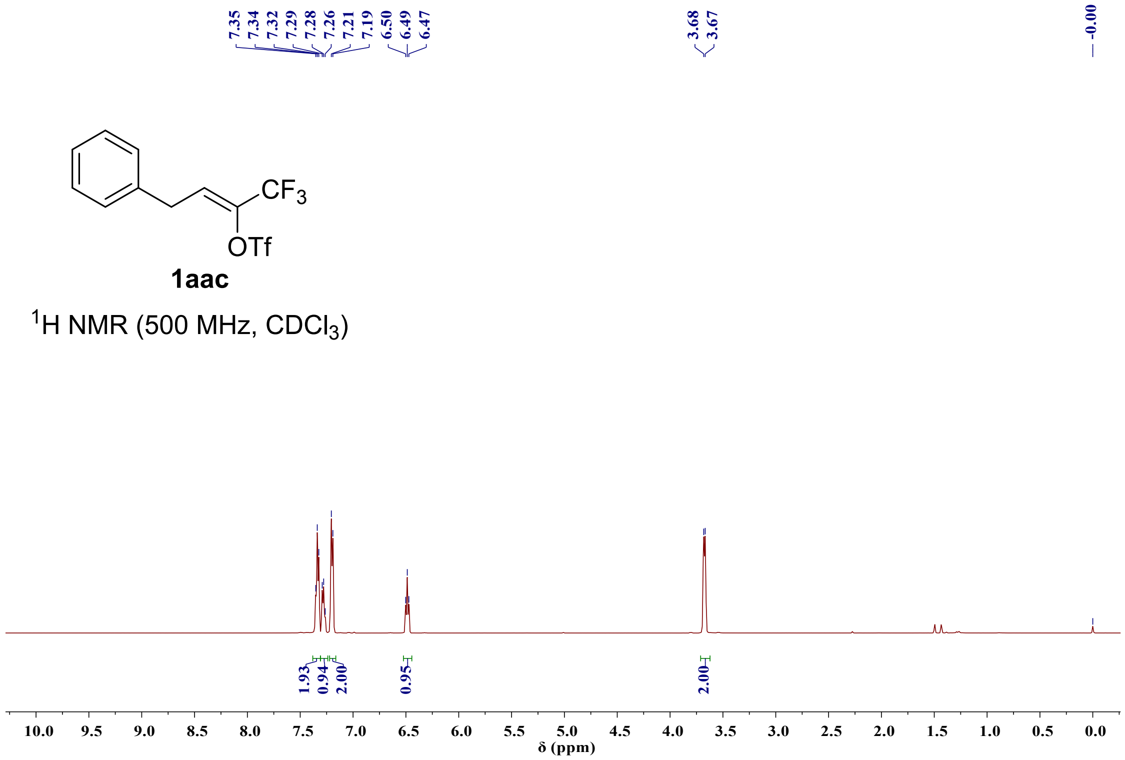

(Z)-1,1,1-trifluoro-4-phenylbut-2-en-2-yl trifluoromethanesulfonate (1aac)

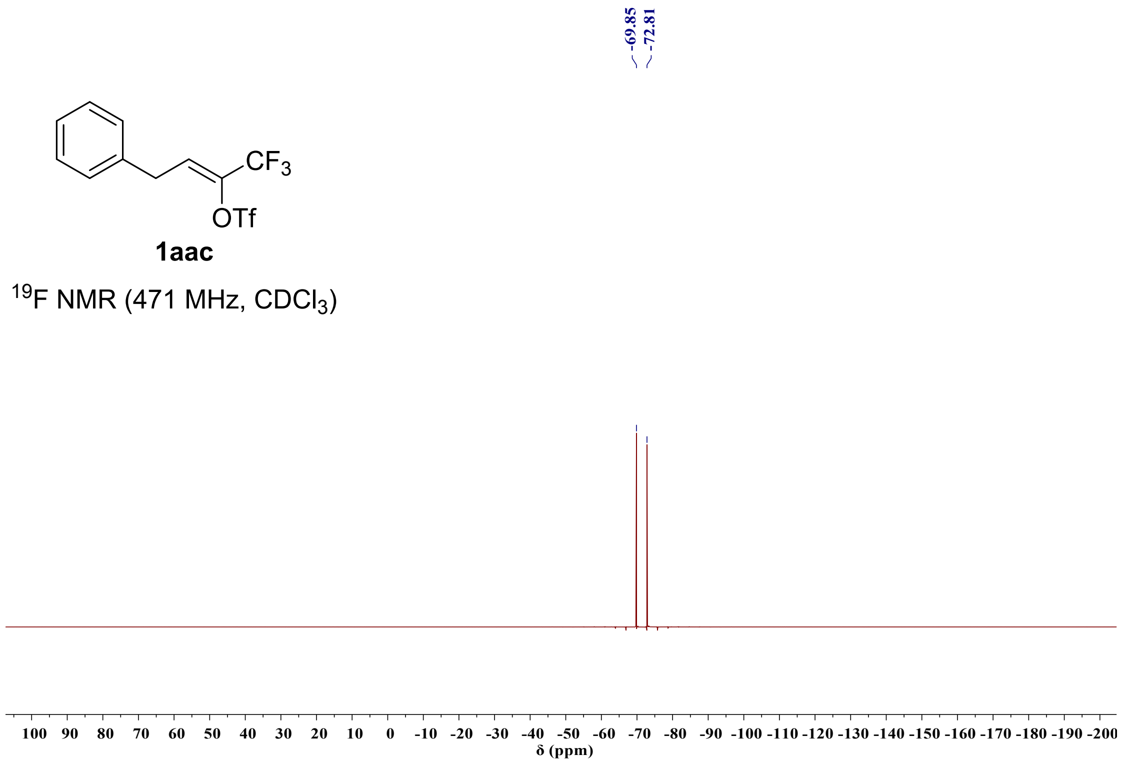

(Z)-1,1,1-trifluoro-4-phenylbut-2-en-2-yl trifluoromethanesulfonate (1aac)

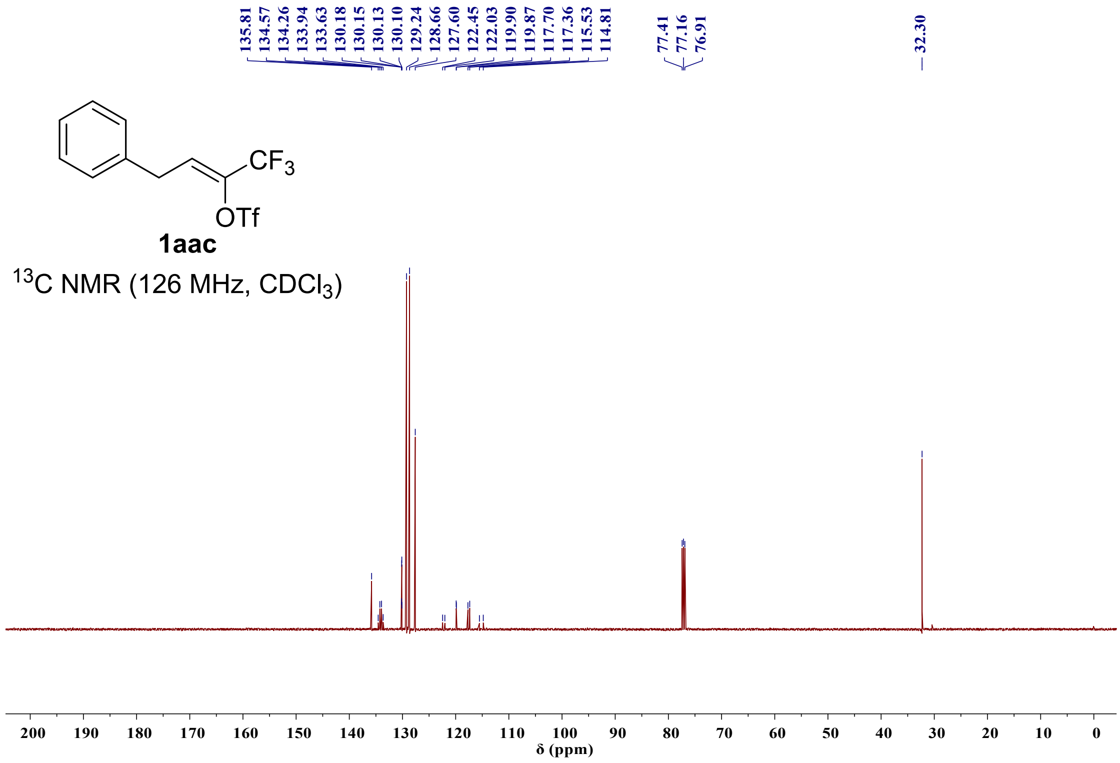

(Z)-1,1,1-trifluoro-4-(4-isopropylphenyl)but-2-en-2-yl trifluoromethanesulfonate (1aad)

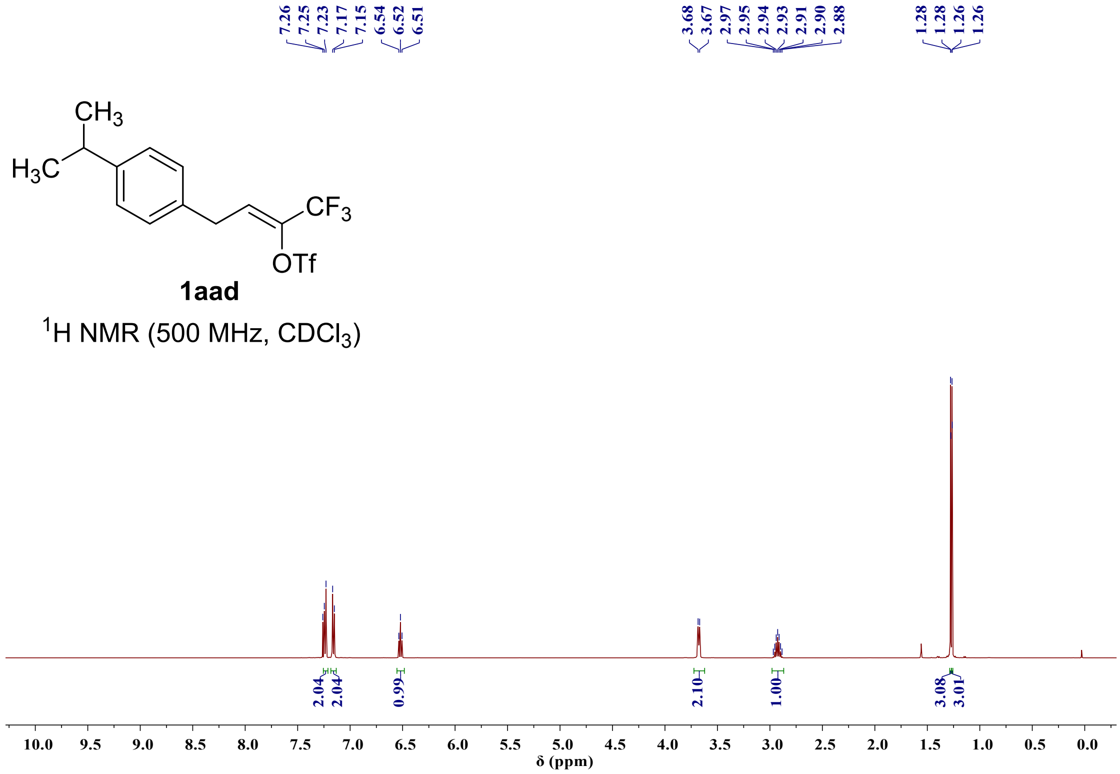

(Z)-1,1,1-trifluoro-4-(4-isopropylphenyl)but-2-en-2-yl trifluoromethanesulfonate (1aad)

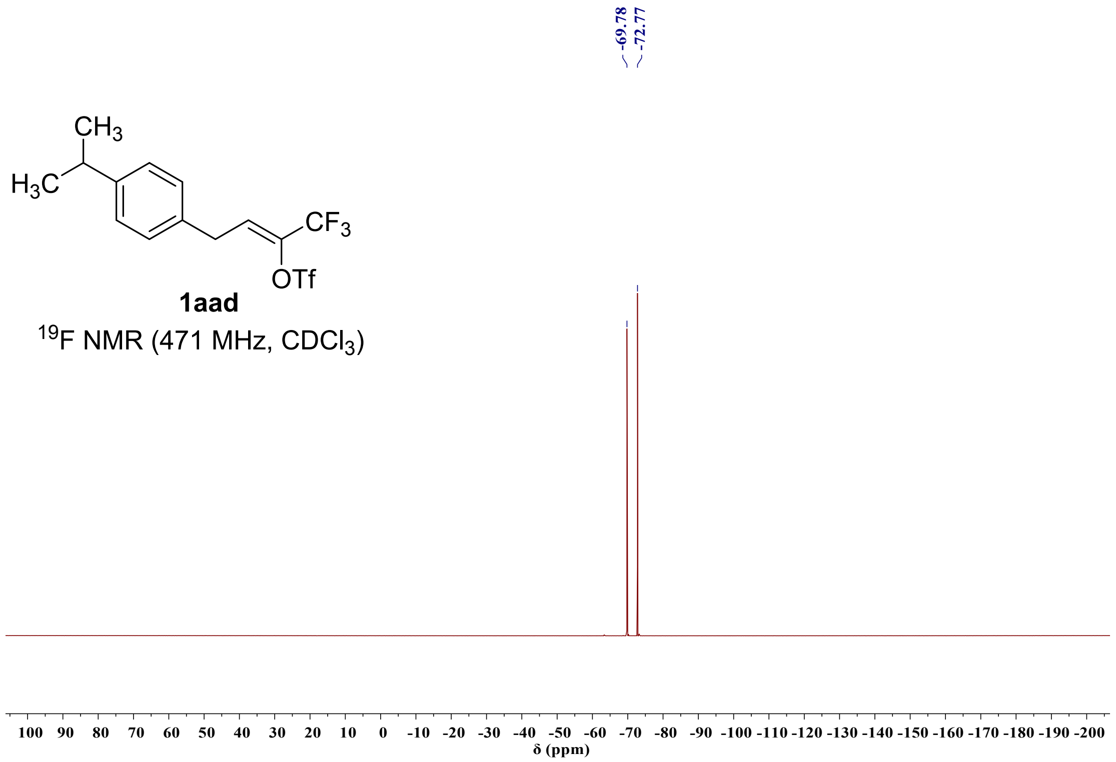

(Z)-1,1,1-trifluoro-4-(4-isopropylphenyl)but-2-en-2-yl trifluoromethanesulfonate (1aad)

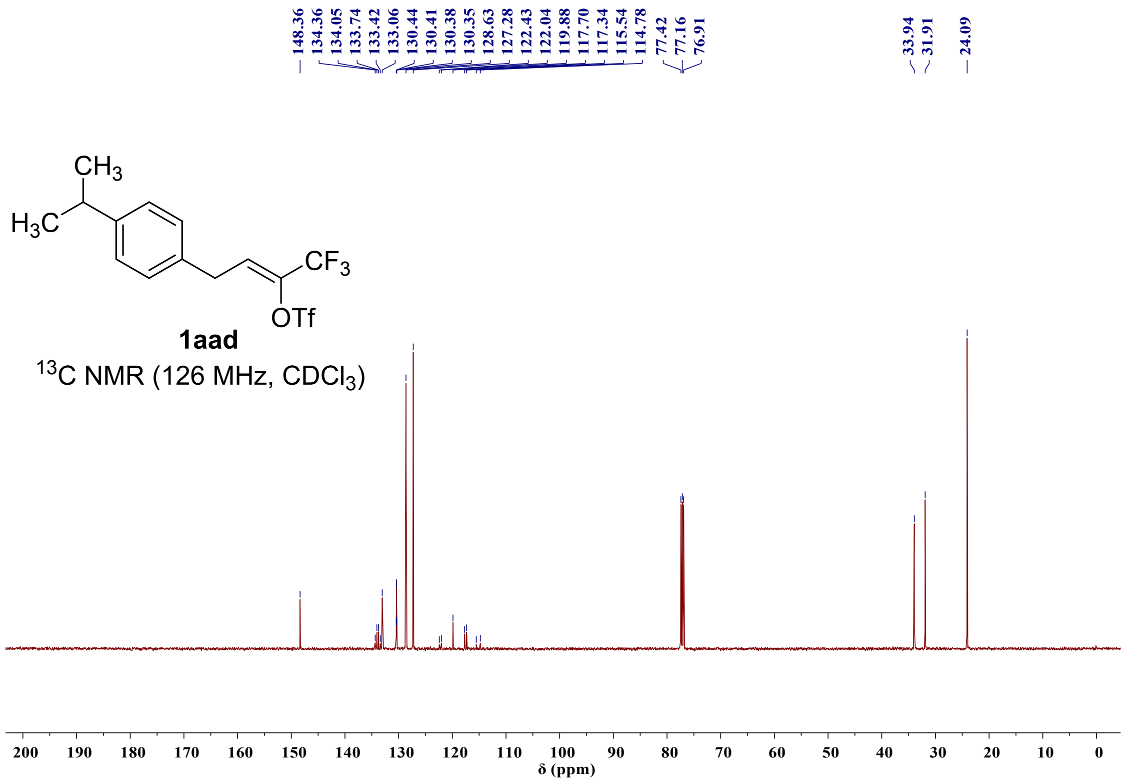

(Z)-4-(3-chlorophenyl)-1,1,1-trifluorobut-2-en-2-yl trifluoromethanesulfonate (1aae)

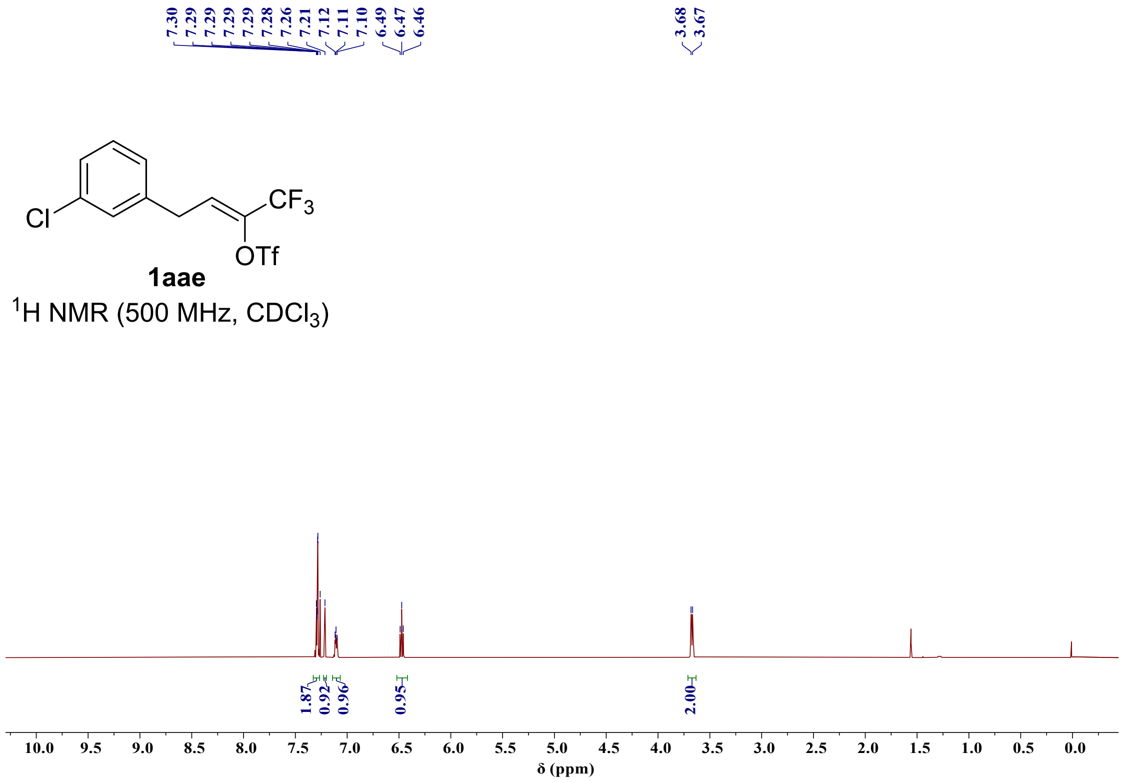

(Z)-4-(3-chlorophenyl)-1,1,1-trifluorobut-2-en-2-yl trifluoromethanesulfonate (1aae)

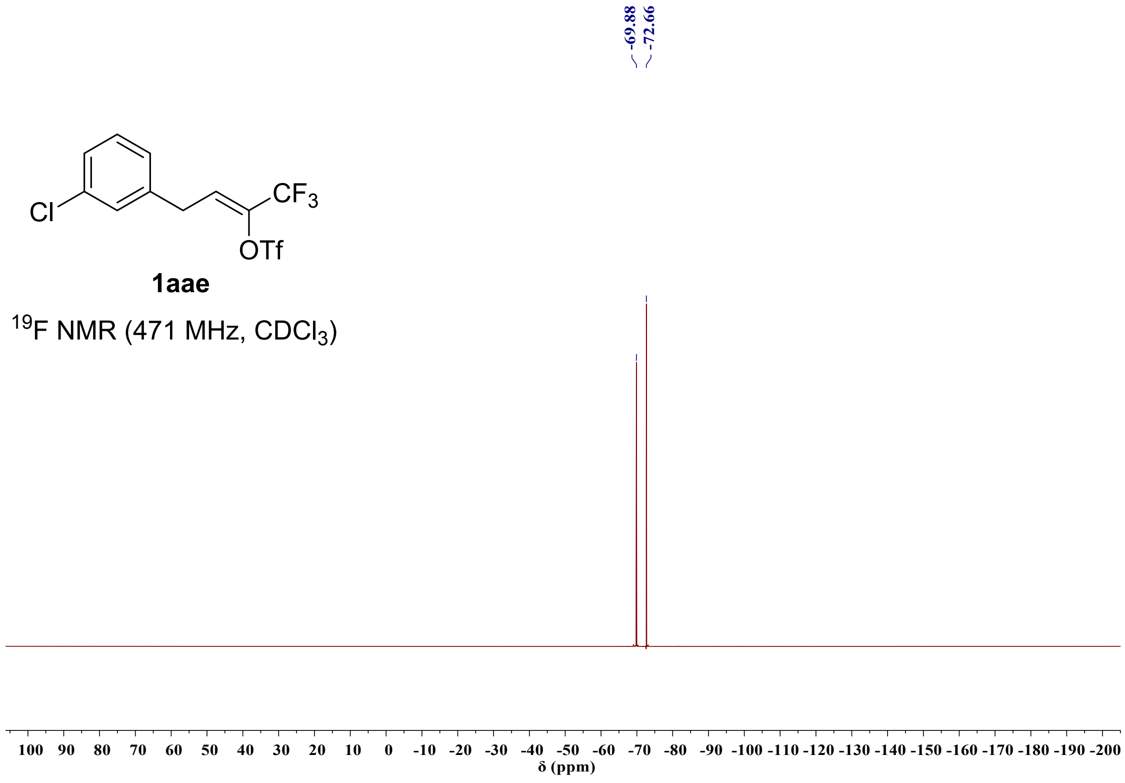

(Z)-4-(3-chlorophenyl)-1,1,1-trifluorobut-2-en-2-yl trifluoromethanesulfonate (1aae)

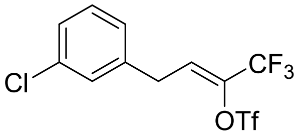

1aae

^{13}C NMR (126 MHz, $CDCl_3$)

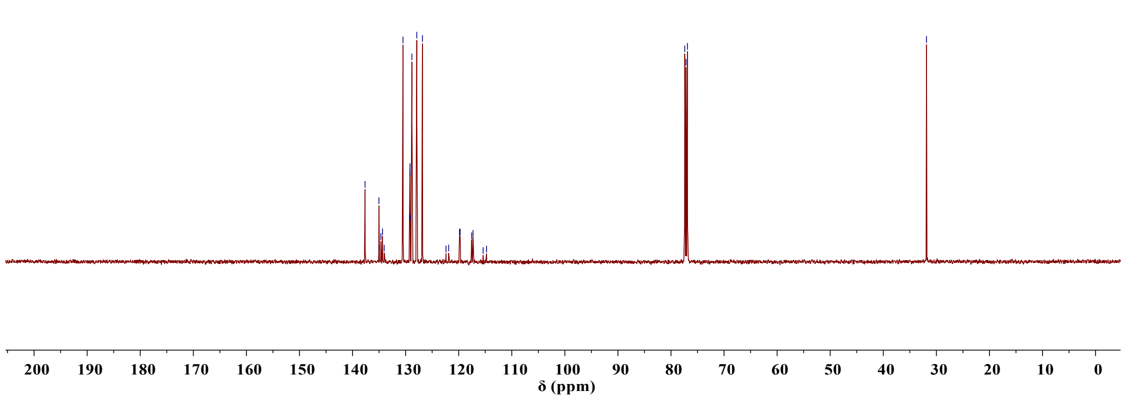

(Z)-4-(4-cyanophenyl)-1,1,1-trifluorobut-2-en-2-yl trifluoromethanesulfonate (1aaf)

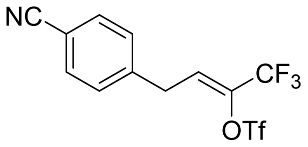

1aaf

^{1}H NMR (500 MHz, $CDCl_3$)

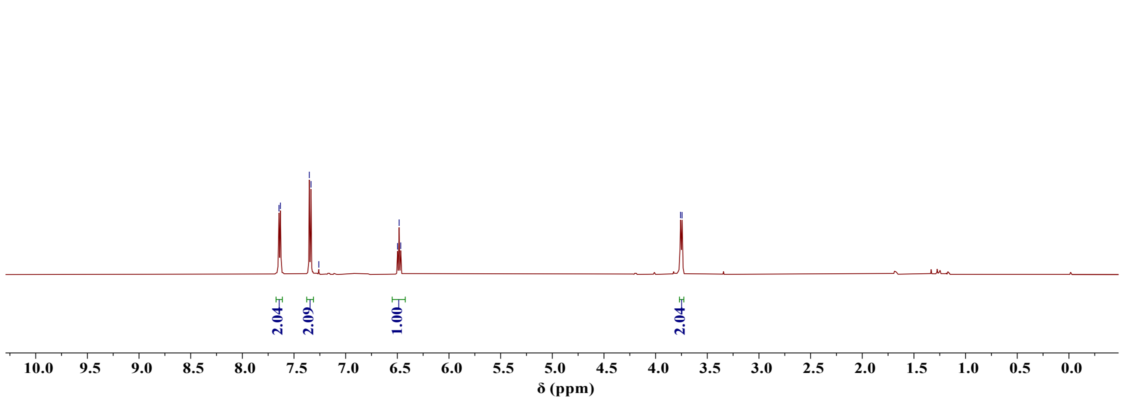

(Z)-4-(4-cyanophenyl)-1,1,1-trifluorobut-2-en-2-yl trifluoromethanesulfonate (1aaf)

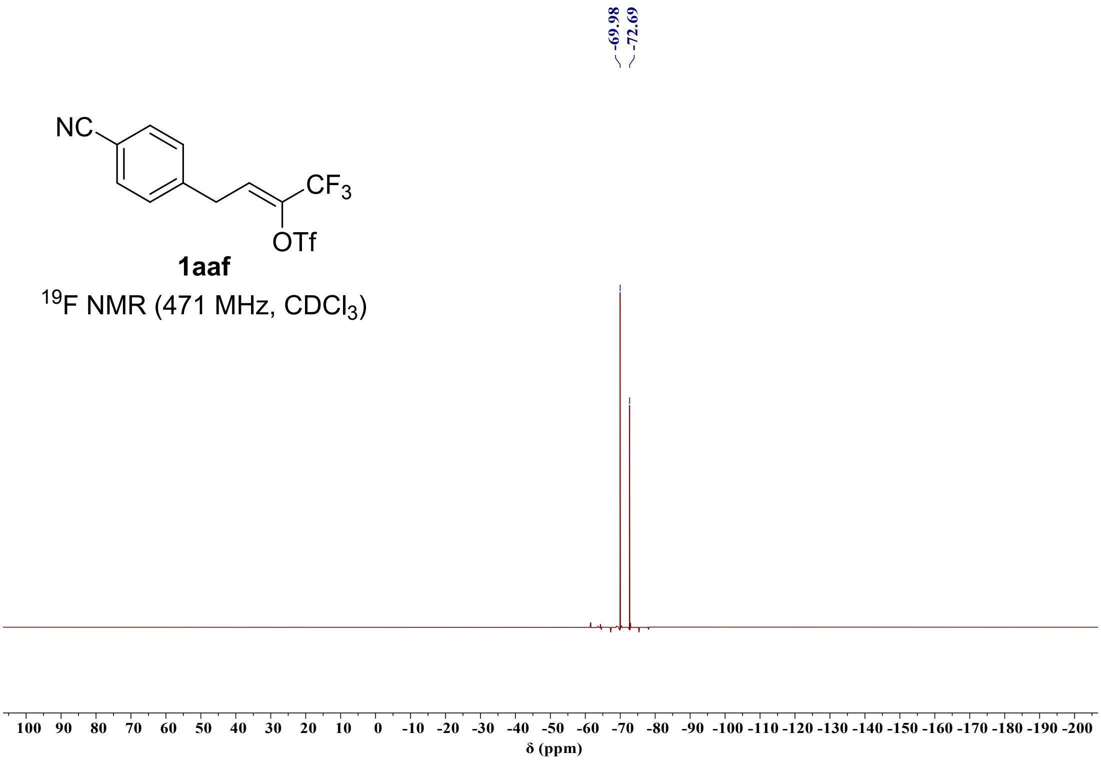

(Z)-4-(4-cyanophenyl)-1,1,1-trifluorobut-2-en-2-yl trifluoromethanesulfonate (1aaf)

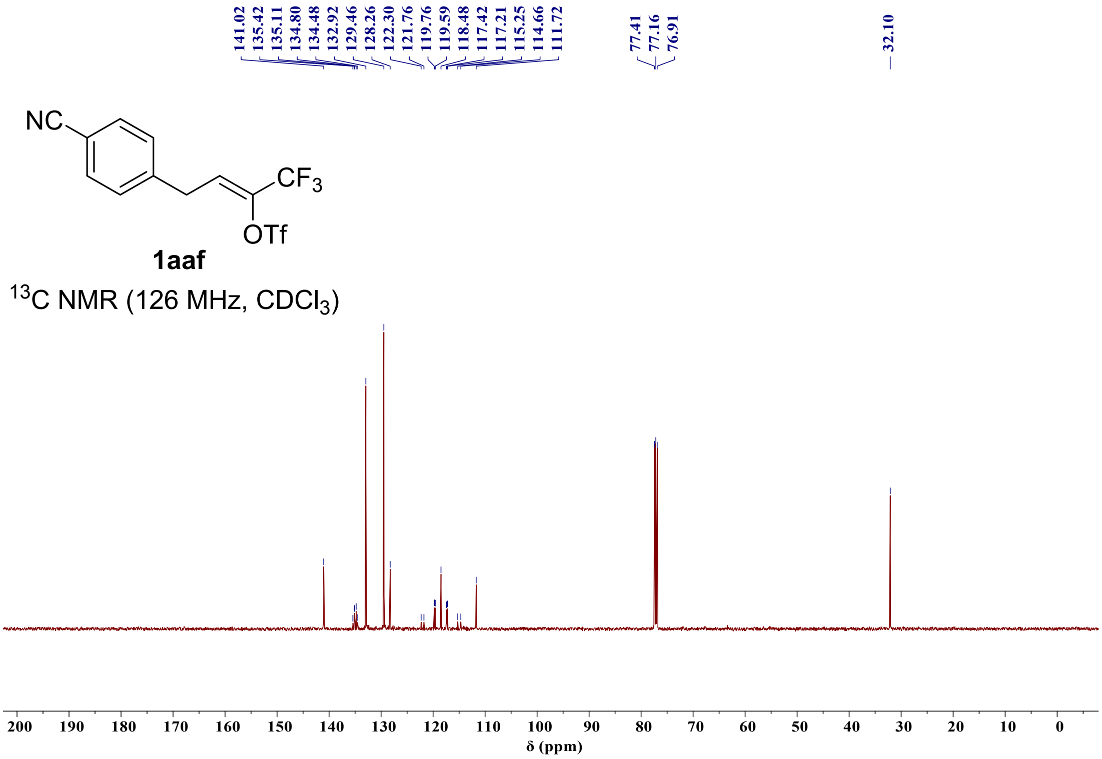

tert-butyl(((3R,8R,9S,10S,13R,14S,17R)-17-((R)-6-chloro-7,7,7-trifluorohept-5-en-2-yl)-10,13-dimethylhexadecahydro-1H-cyclopenta[a]phenanthren-3-yl)oxy)dimethylsilane (1aag)

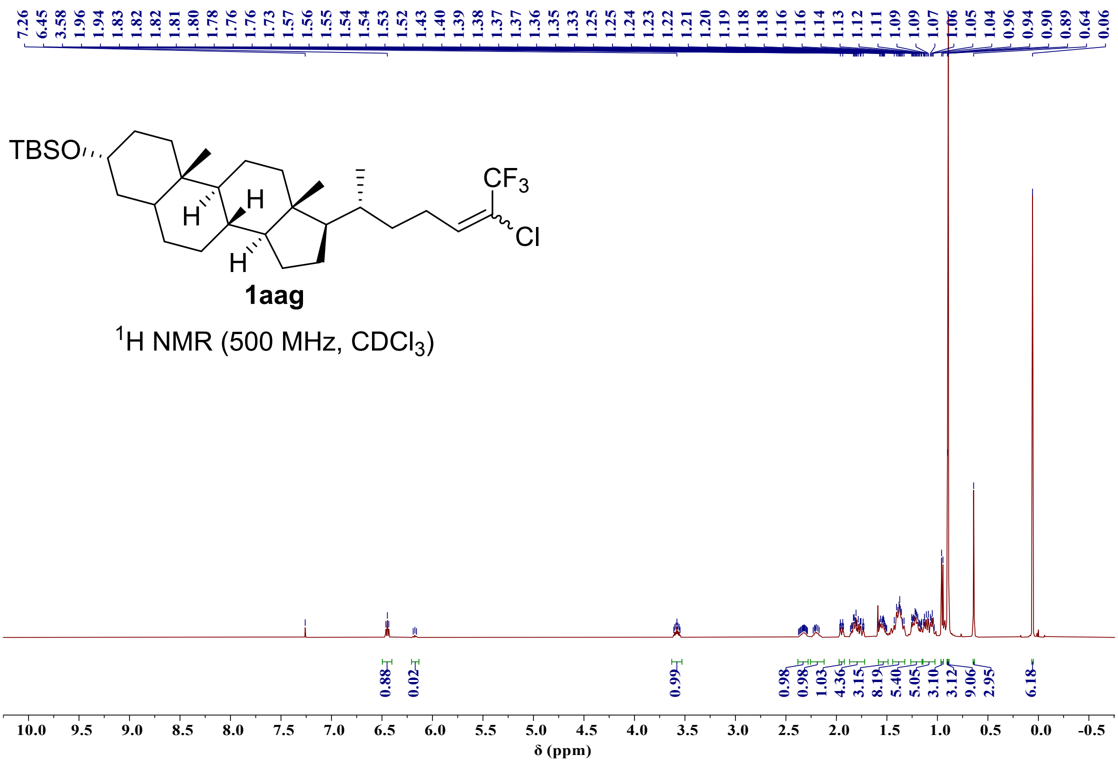

tert-butyl(((3R,8R,9S,10S,13R,14S,17R)-17-((R)-6-chloro-7,7,7-trifluorohept-5-en-2-yl)-10,13-dimethylhexadecahydro-1H-cyclopenta[a]phenanthren-3-yl)oxy)dimethylsilane (1aag)

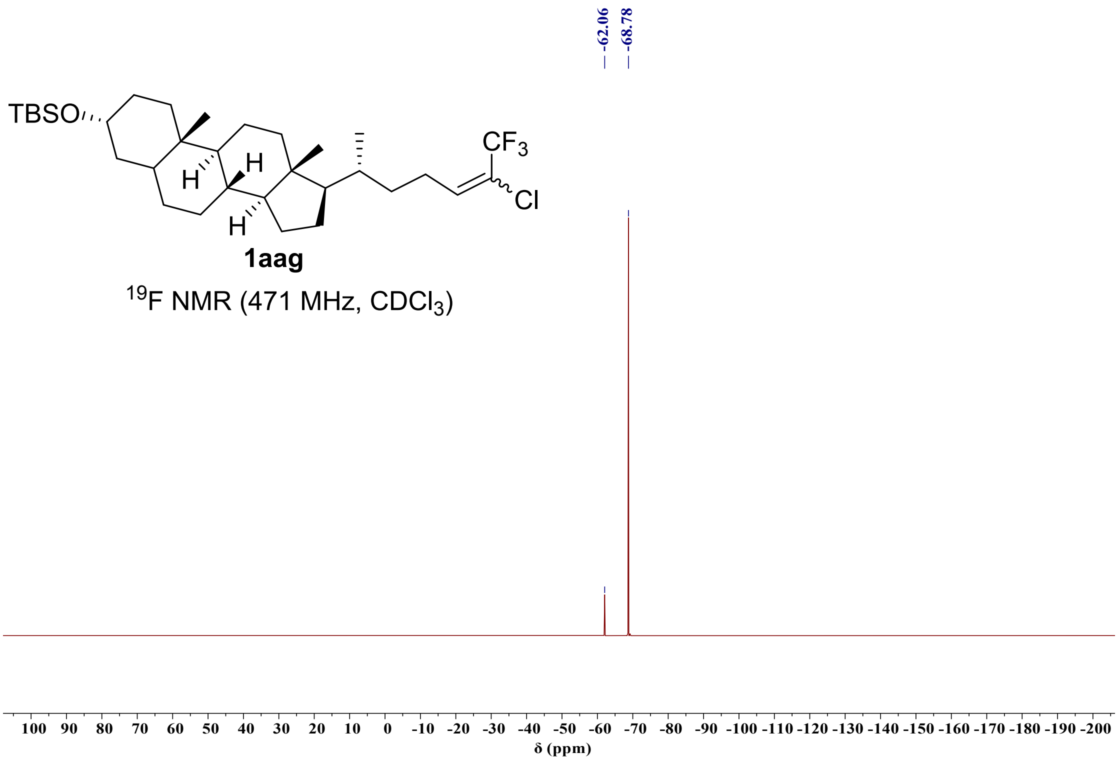

tert-butyl(((3R,8R,9S,10S,13R,14S,17R)-17-((R)-6-chloro-7,7,7-trifluorohept-5-en-2-yl)-10,13-dimethylhexadecahydro-1H-cyclopenta[a]phenanthren-3-yl)oxy)dimethylsilane (1aag)

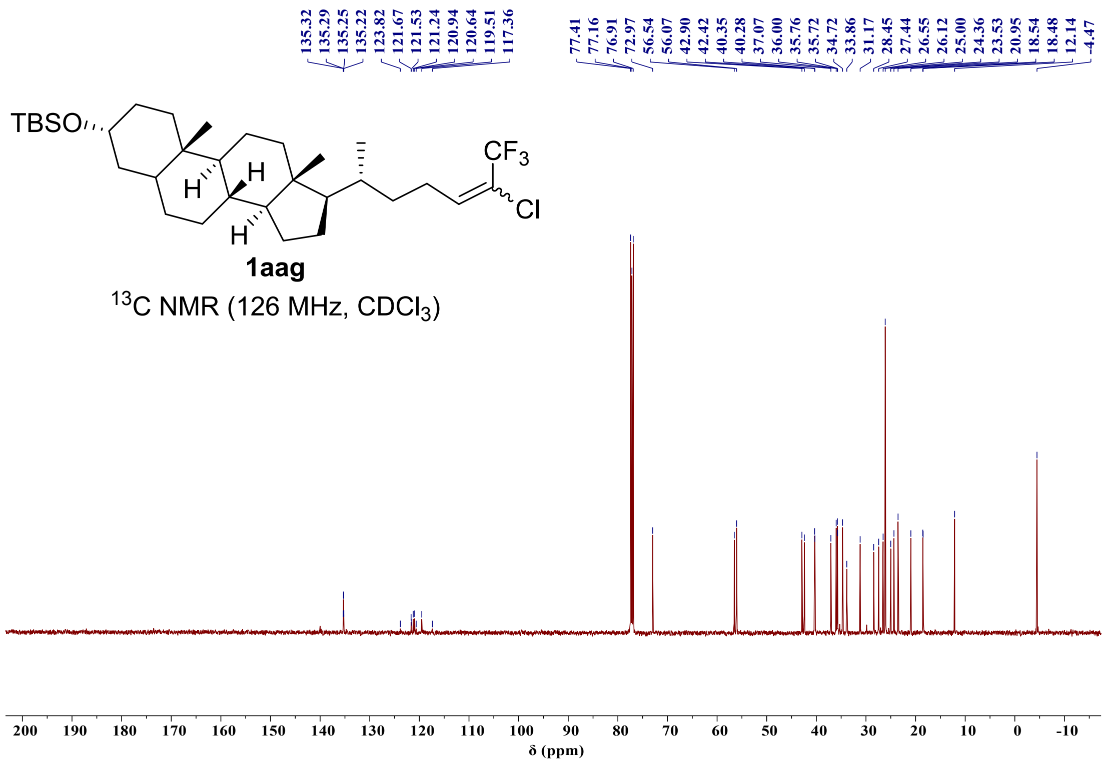

(8R,9S,13S,14S,17S)-3-((9-chloro-10,10,10-trifluorodec-8-en-1-yl)oxy)-13-methyl-7,8,9,11,12,13,14,15,16,17-decahydro-6H-cyclopenta[a]phenanthren-17-yl heptanoate (1aah)

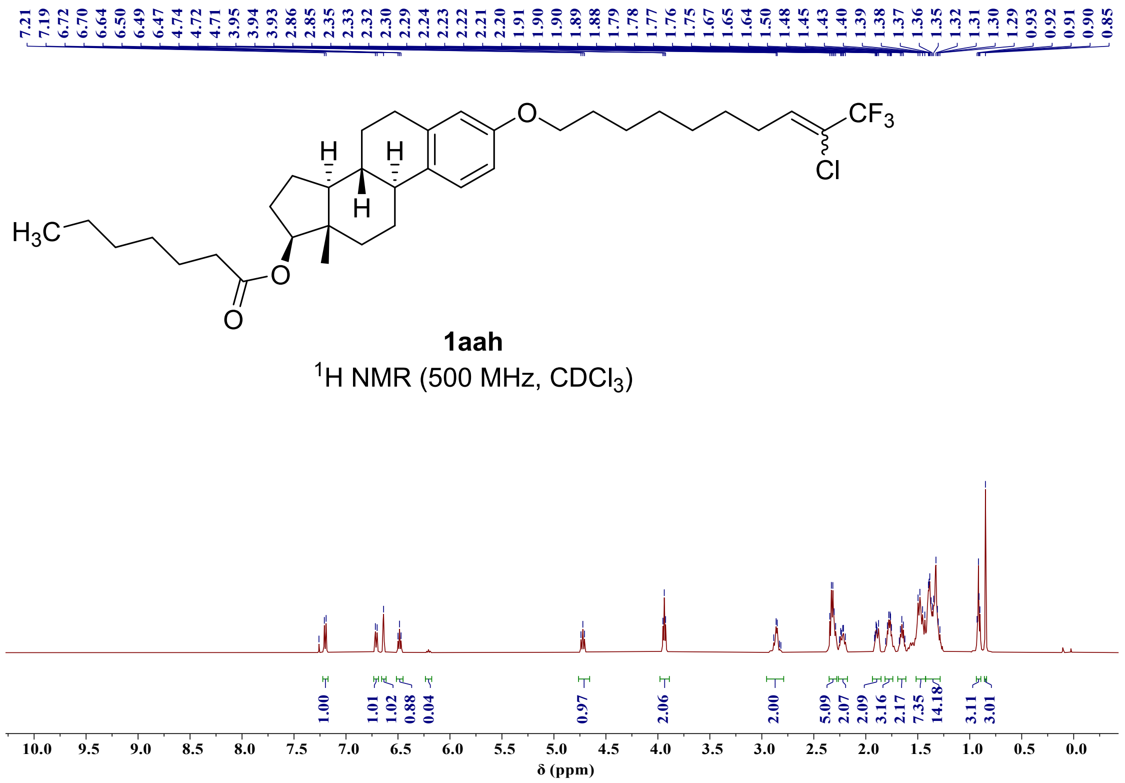

(8R,9S,13S,14S,17S)-3-((9-chloro-10,10,10-trifluorodec-8-en-1-yl)oxy)-13-methyl-7,8,9,11,12,13,14,15,16,17-decahydro-6H-cyclopenta[a]phenanthren-17-yl heptanoate (1aah)

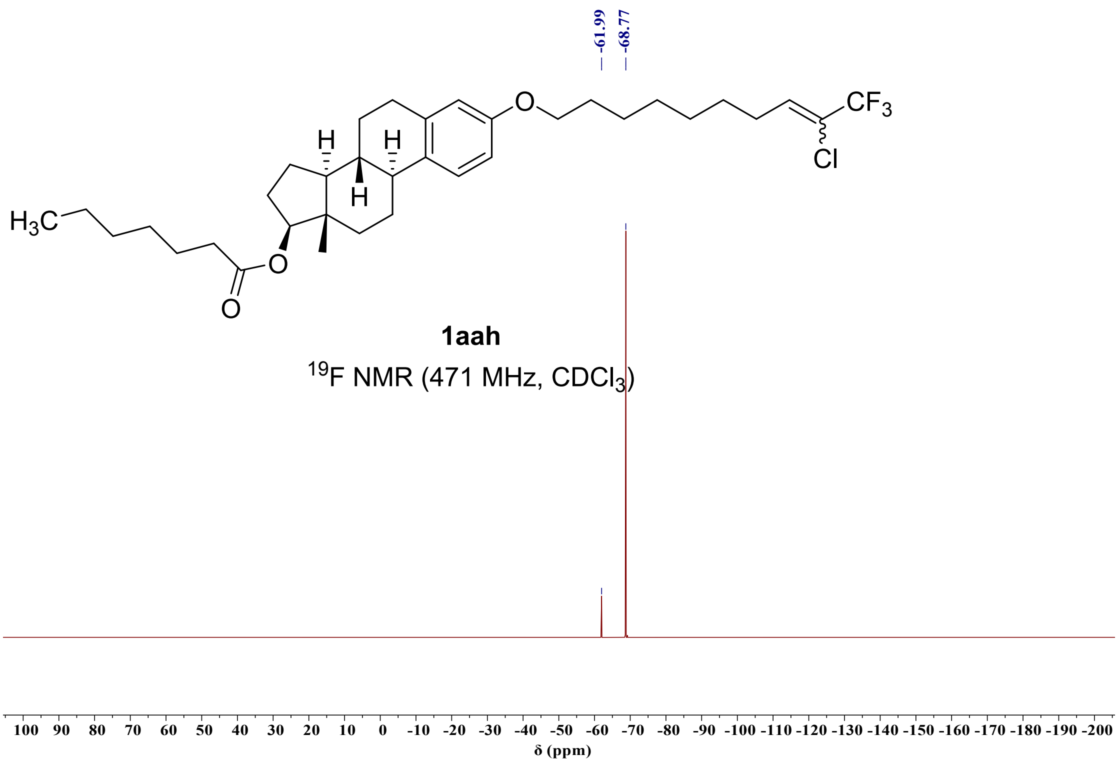

(8R,9S,13S,14S,17S)-3-((9-chloro-10,10,10-trifluorodec-8-en-1-yl)oxy)-13-methyl-7,8,9,11,12,13,14,15,16,17-decahydro-6H-cyclopenta[a]phenanthren-17-yl heptanoate (1aah)

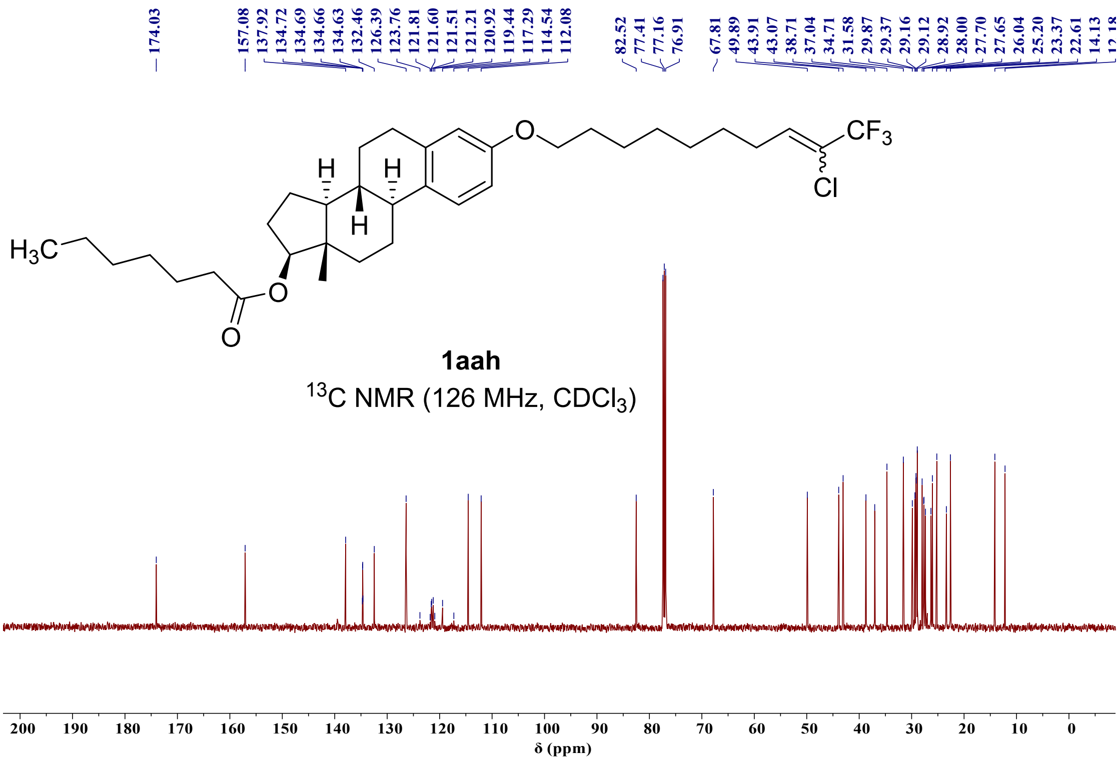

2,2'-(1,1-difluoro-5-phenylpent-1-ene-2,3-diyl)bis(4,4,5,5-tetramethyl-1,3,2-dioxaborolane) (3)

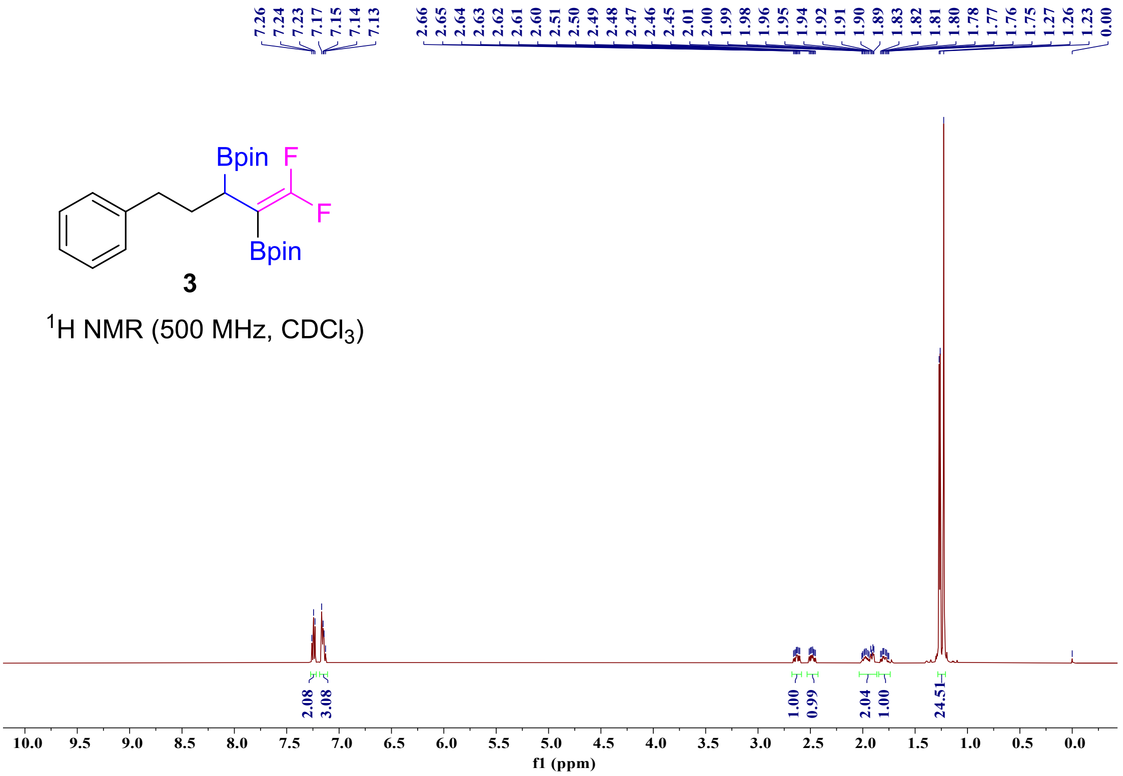

2,2'-(1,1-difluoro-5-phenylpent-1-ene-2,3-diyl)bis(4,4,5,5-tetramethyl-1,3,2-dioxaborolane) (3)

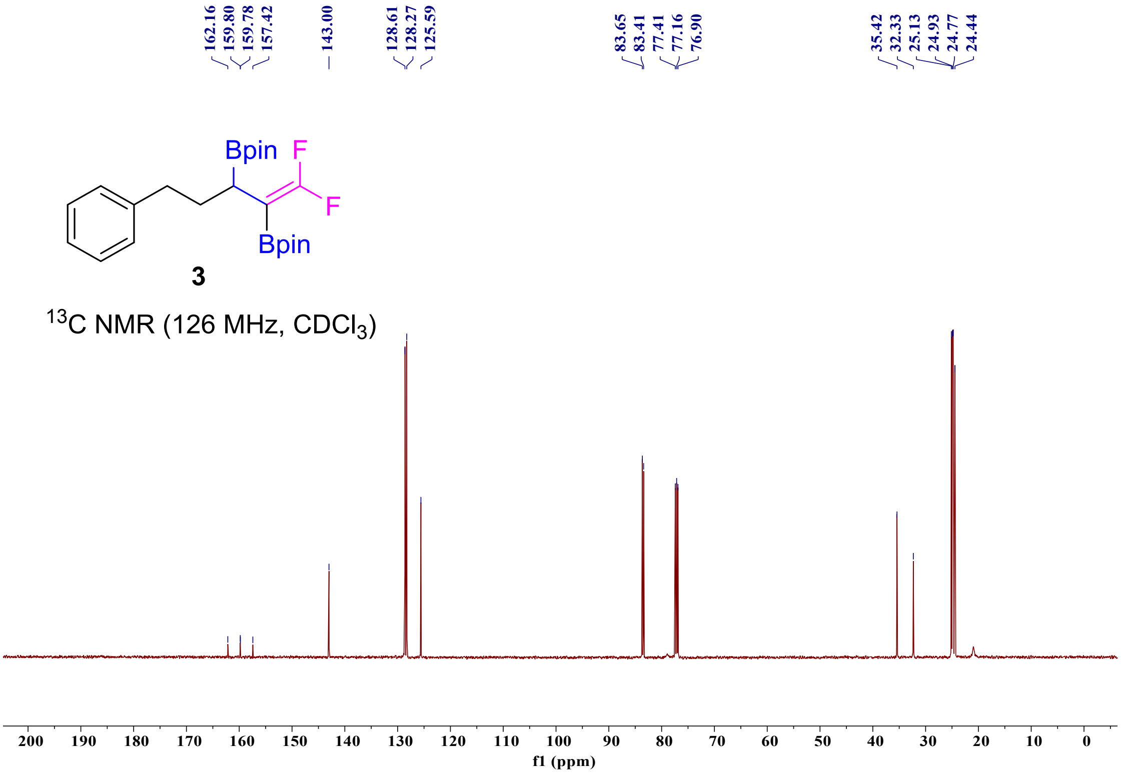

2,2'-(1,1-difluoro-5-phenylpent-1-ene-2,3-diyl)bis(4,4,5,5-tetramethyl-1,3,2-dioxaborolane) (3)

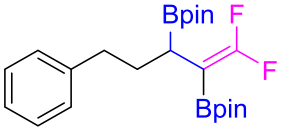

3

^{19}F NMR (470 MHz, CDCl$_3$)

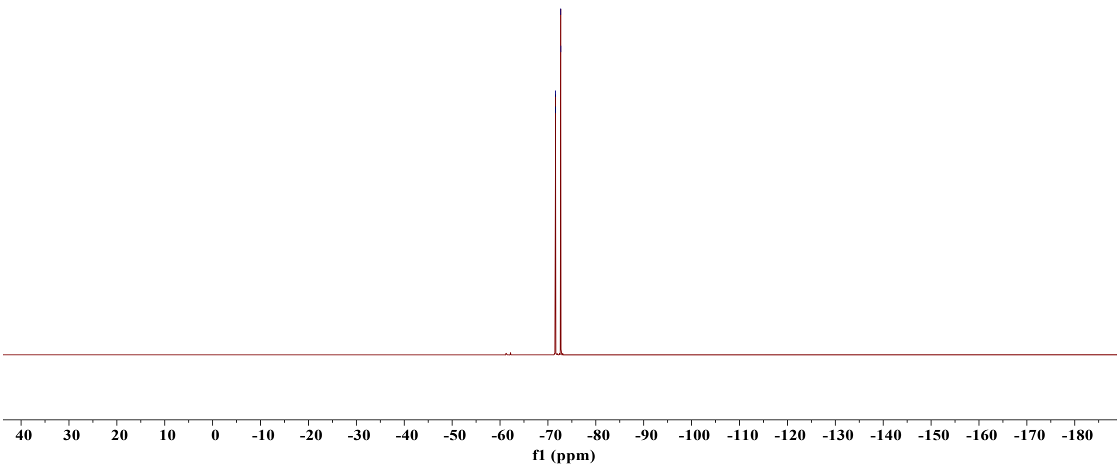

2,2'-(1,1-difluoro-5-phenylpent-1-ene-2,3-diyl)bis(4,4,5,5-tetramethyl-1,3,2-dioxaborolane) (3)

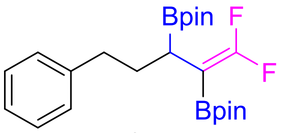

3

^{11}B NMR (128 MHz, CDCl$_3$)

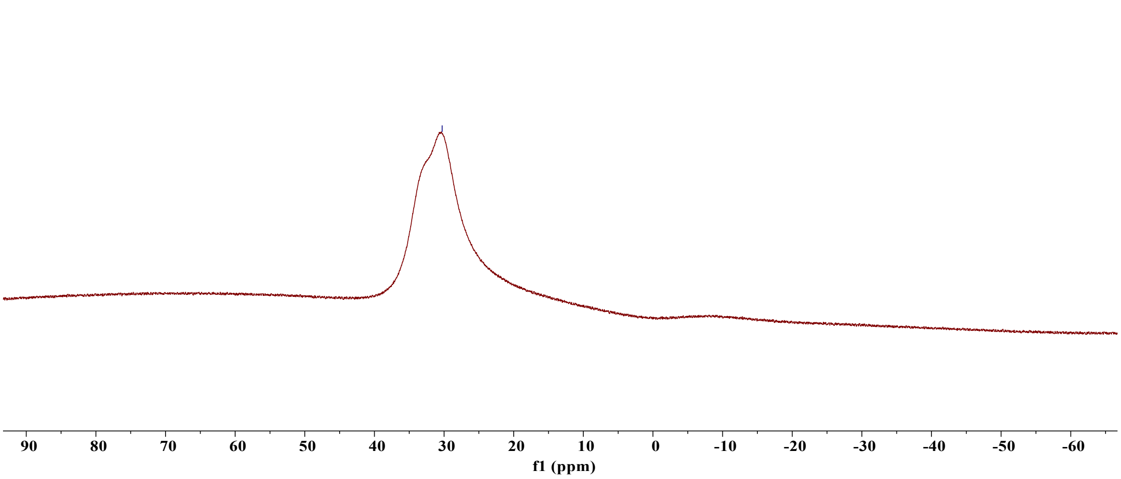

2,2'-(1,1-difluoro-5-(p-tolyl)pent-1-ene-2,3-diyl)bis(4,4,5,5-tetramethyl-1,3,2-dioxaborolane) (4)

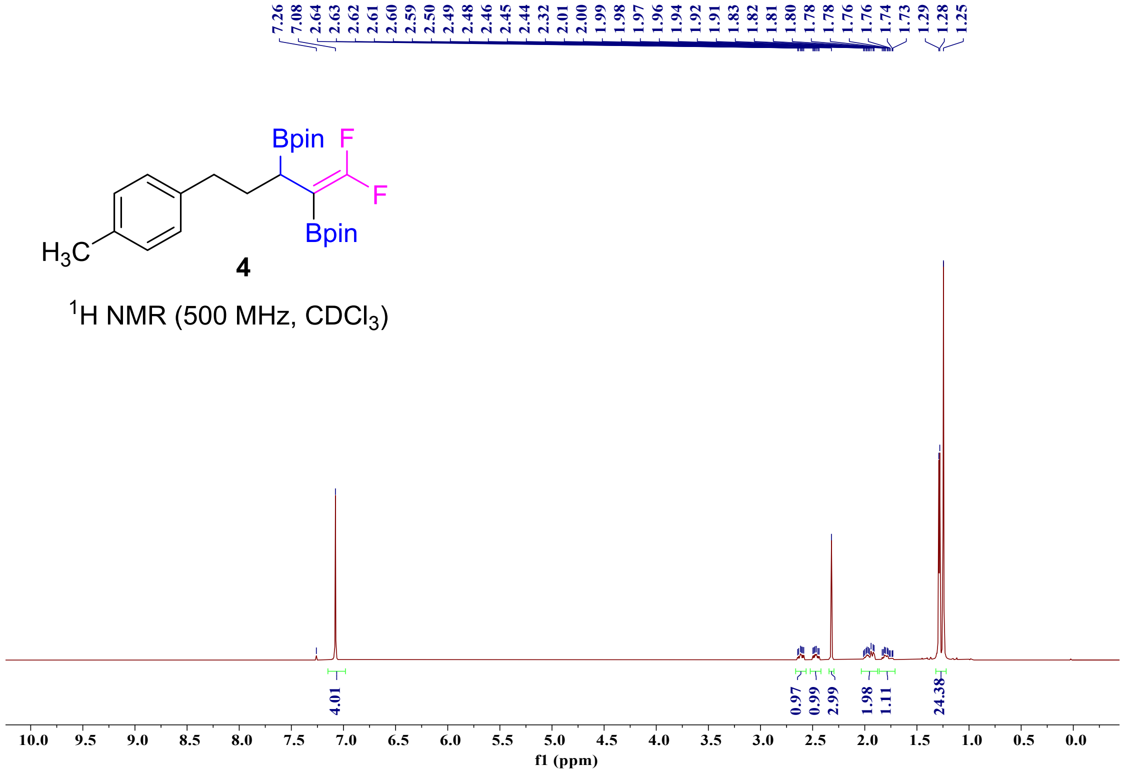

2,2'-(1,1-difluoro-5-(p-tolyl)pent-1-ene-2,3-diyl)bis(4,4,5,5-tetramethyl-1,3,2-dioxaborolane) (4)

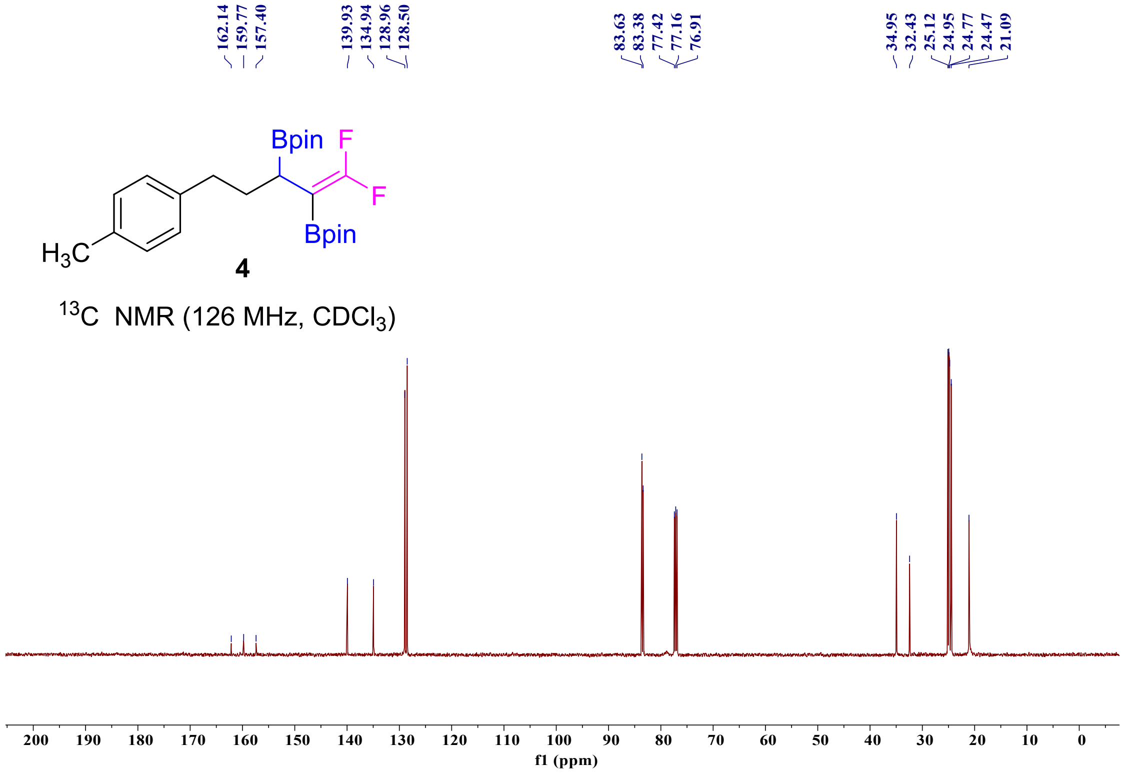

2,2'-(1,1-difluoro-5-(p-tolyl)pent-1-ene-2,3-diyl)bis(4,4,5,5-tetramethyl-1,3,2-dioxaborolane) (4)

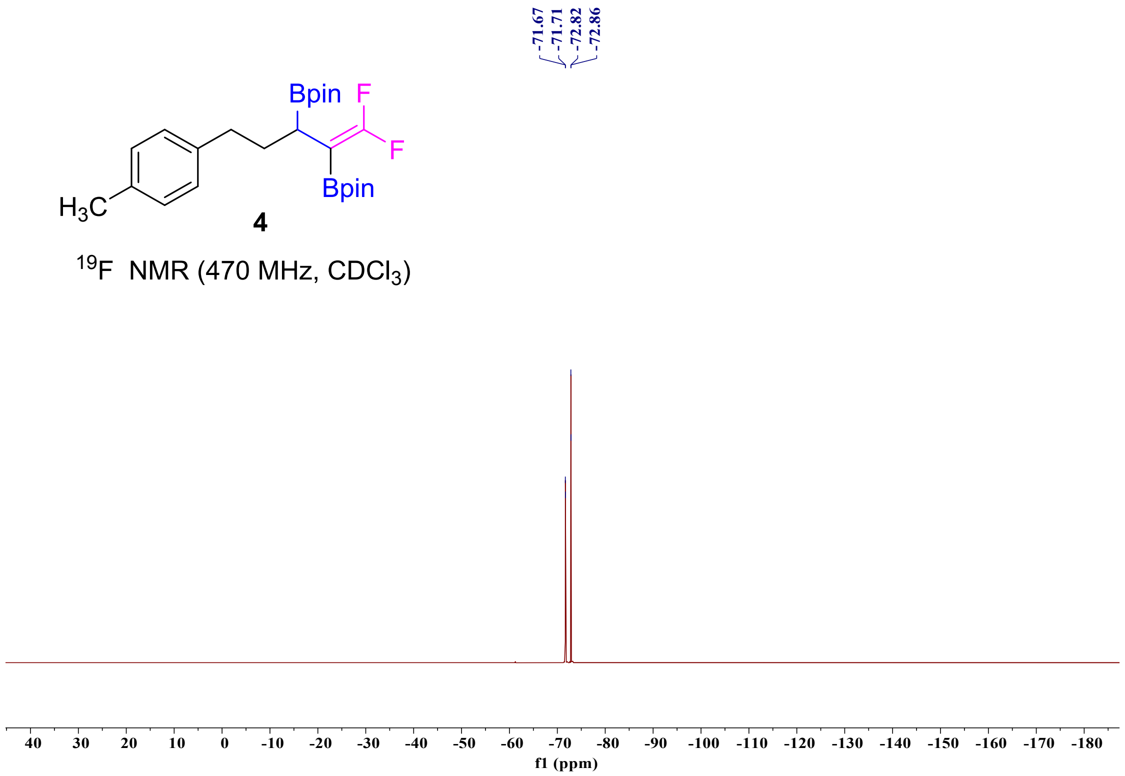

2,2'-(1,1-difluoro-5-(p-tolyl)pent-1-ene-2,3-diyl)bis(4,4,5,5-tetramethyl-1,3,2-dioxaborolane) (4)

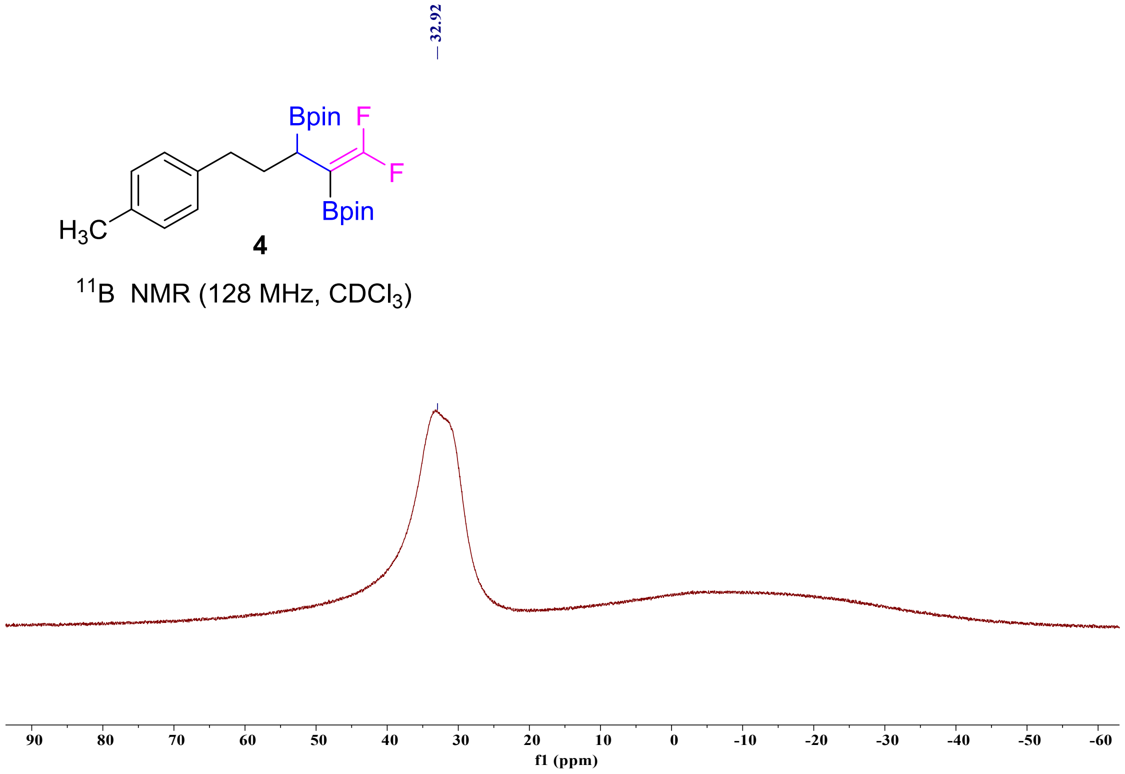

2,2'-(1,1-difluoro-5-(4-methoxyphenyl)pent-1-ene-2,3-diyl)bis(4,4,5,5-tetramethyl-1,3,2-dioxaborolane) (5)

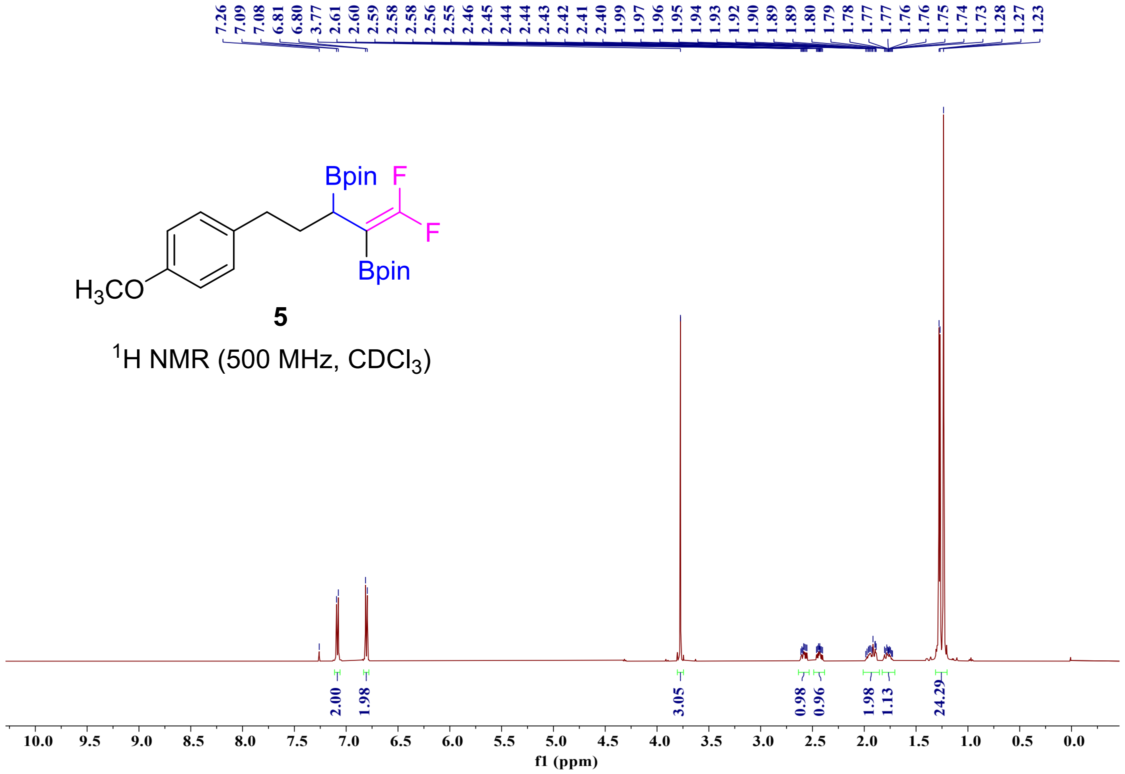

2,2'-(1,1-difluoro-5-(4-methoxyphenyl)pent-1-ene-2,3-diyl)bis(4,4,5,5-tetramethyl-1,3,2-dioxaborolane) (5)

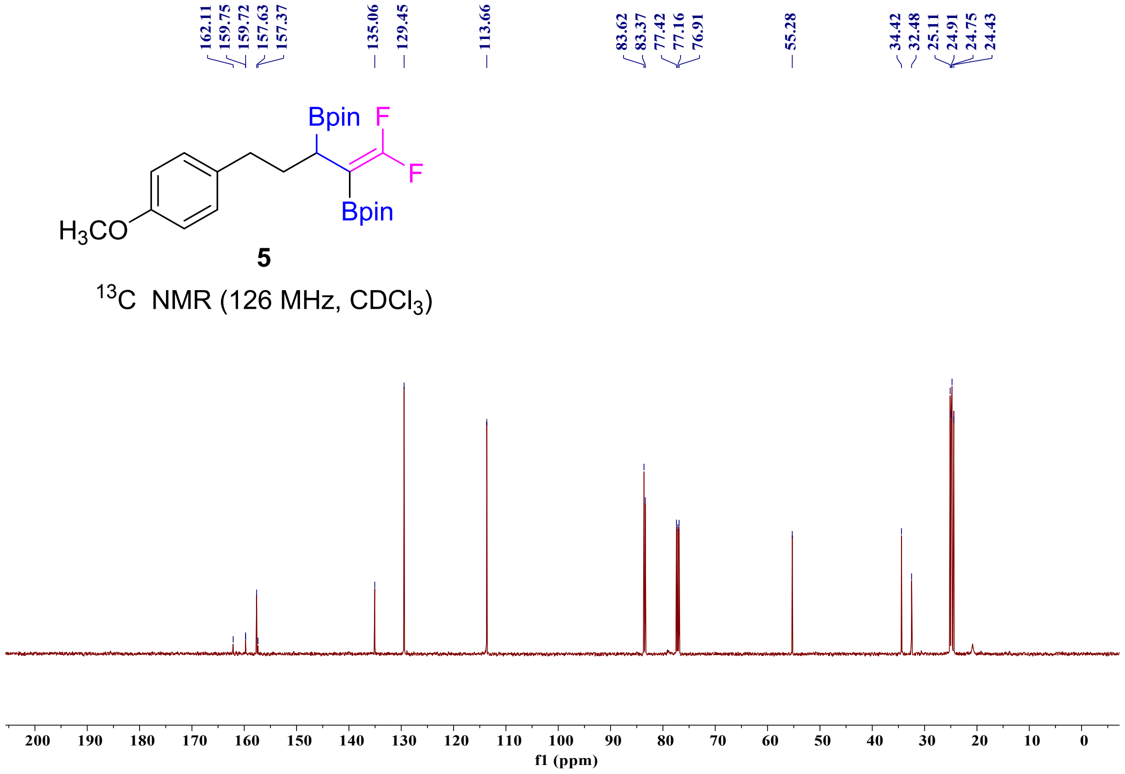

2,2'-(1,1-difluoro-5-(4-methoxyphenyl)pent-1-ene-2,3-diyl)bis(4,4,5,5-tetramethyl-1,3,2-dioxaborolane) (5)

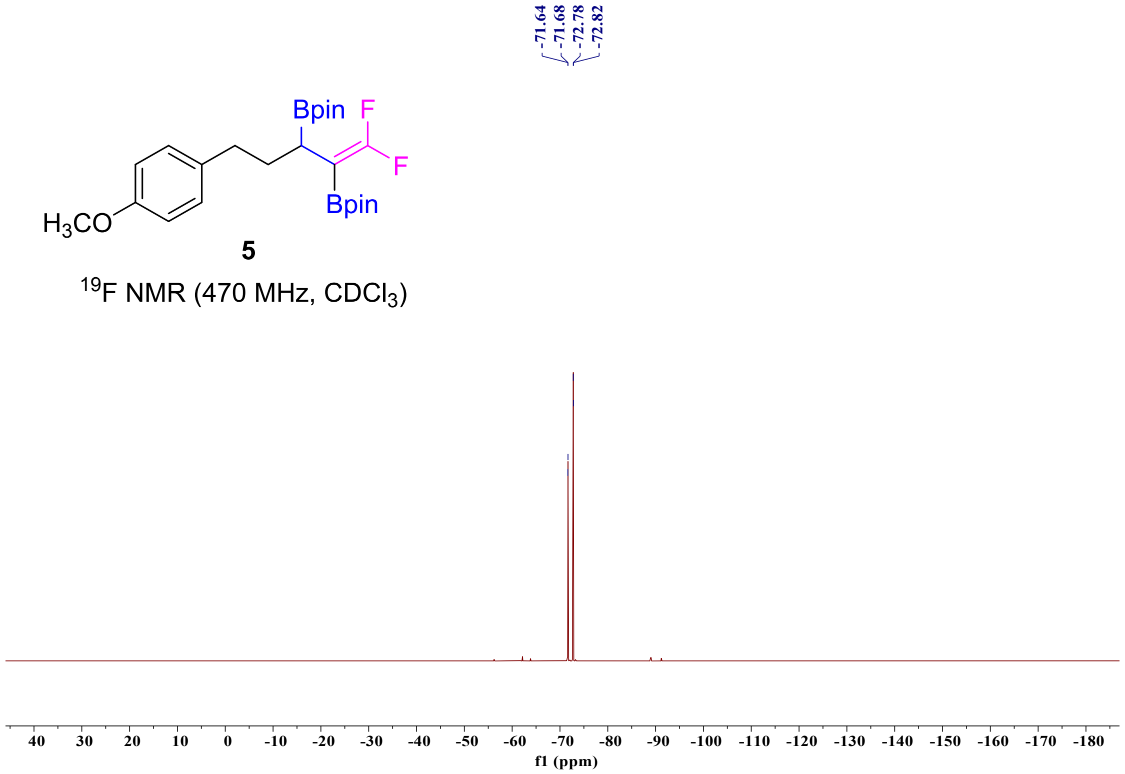

5

^{19}F NMR (470 MHz, $CDCl_3$)

2,2'-(1,1-difluoro-5-(4-methoxyphenyl)pent-1-ene-2,3-diyl)bis(4,4,5,5-tetramethyl-1,3,2-dioxaborolane) (5)

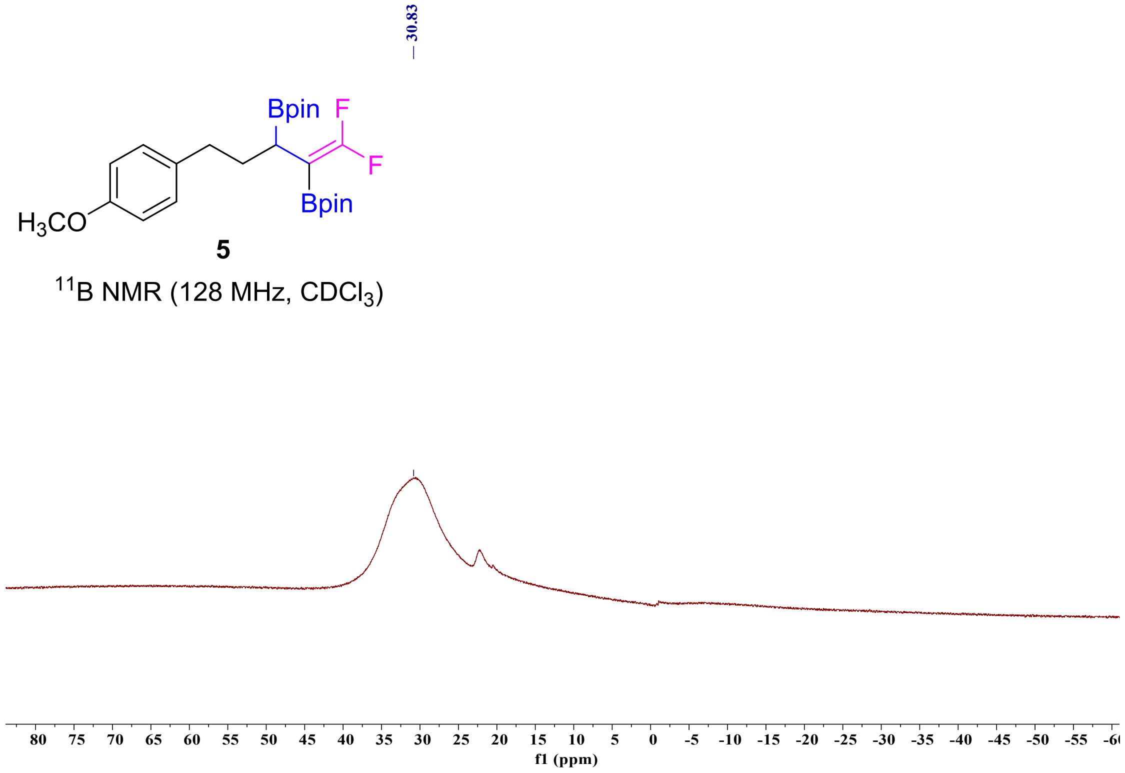

5

^{11}B NMR (128 MHz, $CDCl_3$)

2,2'-(1,1-difluoro-5-(4-(trifluoromethyl)phenyl)pent-1-ene-2,3-diyl)bis(4,4,5,5-tetramethyl-1,3,2-dioxaborolane) (6)

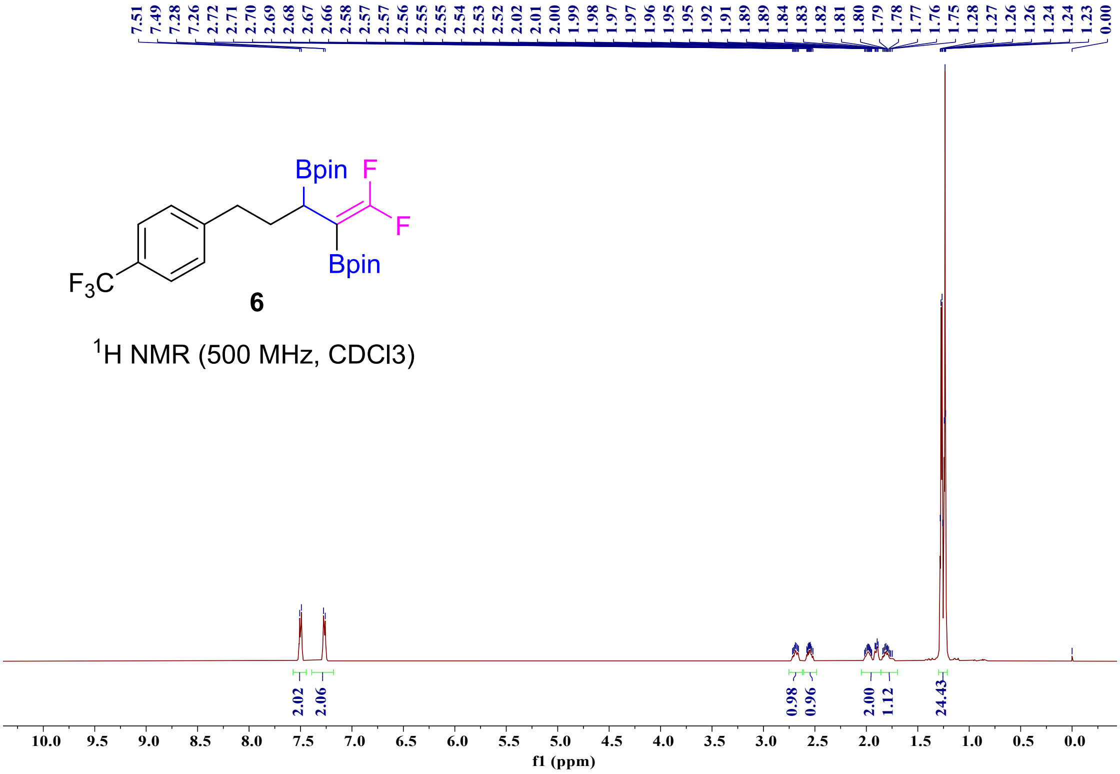

2,2'-(1,1-difluoro-5-(4-(trifluoromethyl)phenyl)pent-1-ene-2,3-diyl)bis(4,4,5,5-tetramethyl-1,3,2-dioxaborolane) (6)

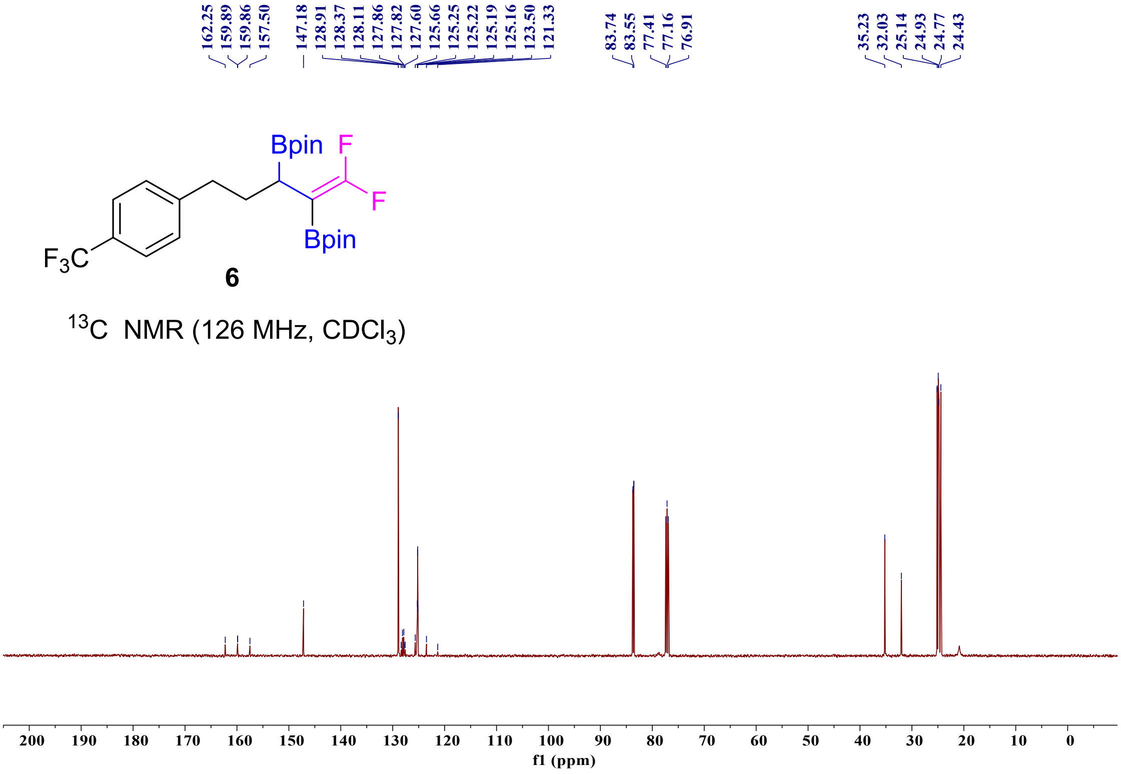

2,2'-(1,1-difluoro-5-(4-(trifluoromethyl)phenyl)pent-1-ene-2,3-diyl)bis(4,4,5,5-tetramethyl-1,3,2-dioxaborolane) (6)

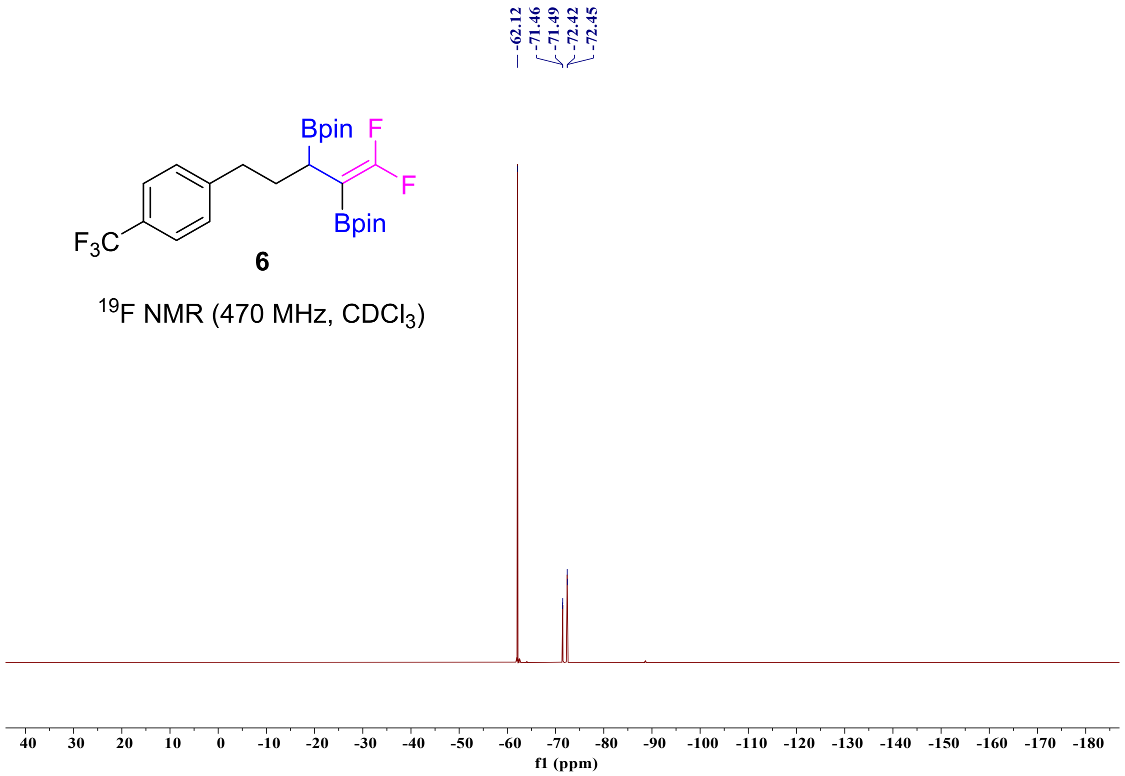

2,2'-(1,1-difluoro-5-(4-(trifluoromethyl)phenyl)pent-1-ene-2,3-diyl)bis(4,4,5,5-tetramethyl-1,3,2-dioxaborolane) (6)

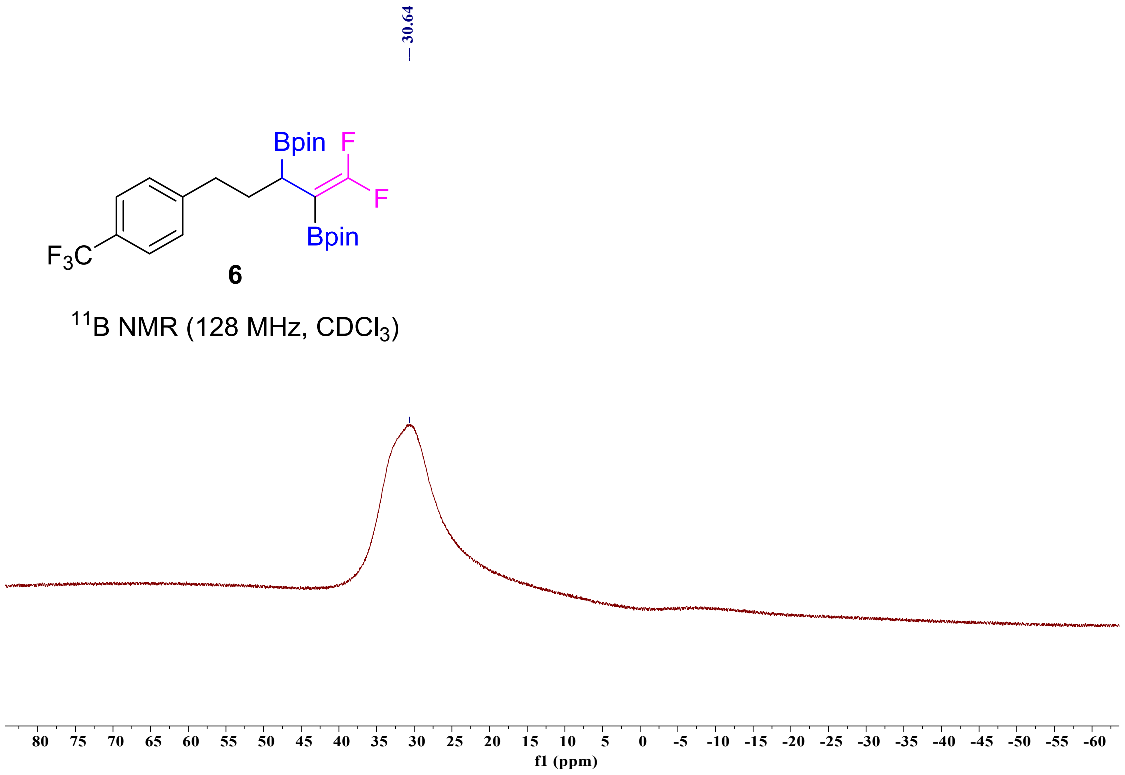

2,2'-(1,1-difluoro-5-(4-fluorophenyl)pent-1-ene-2,3-diyl)bis(4,4,5,5-tetramethyl-1,3,2-dioxaborolane) (7)

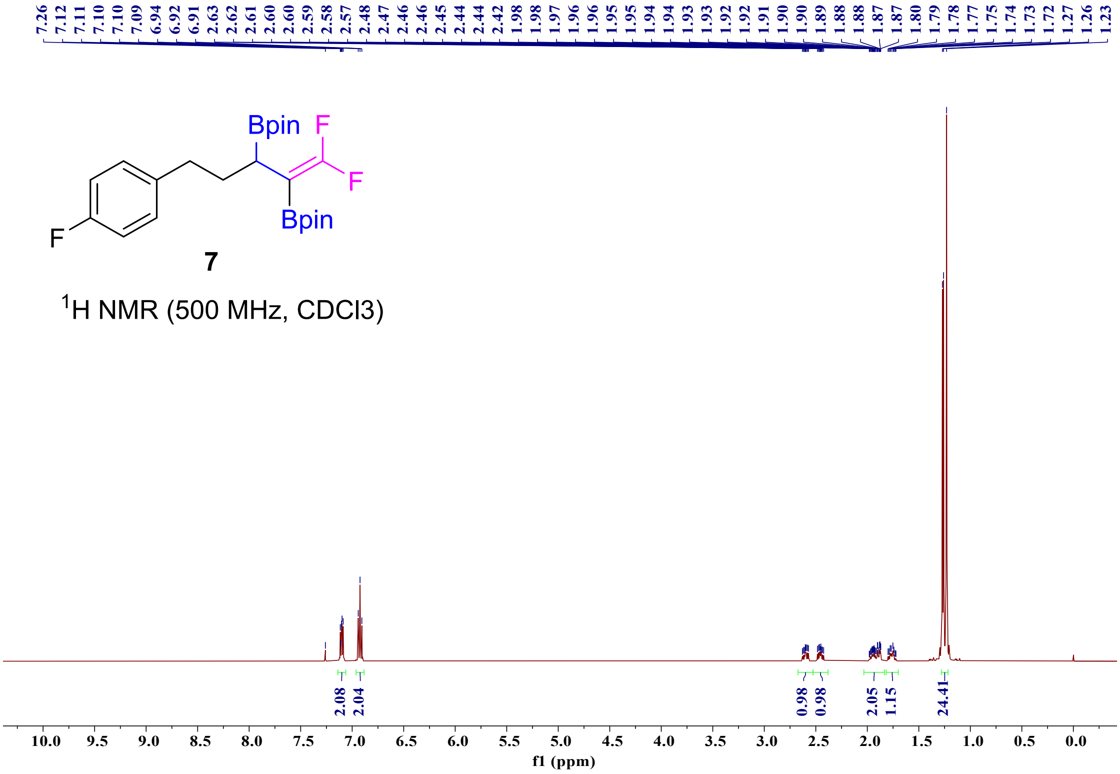

2,2'-(1,1-difluoro-5-(4-fluorophenyl)pent-1-ene-2,3-diyl)bis(4,4,5,5-tetramethyl-1,3,2-dioxaborolane) (7)

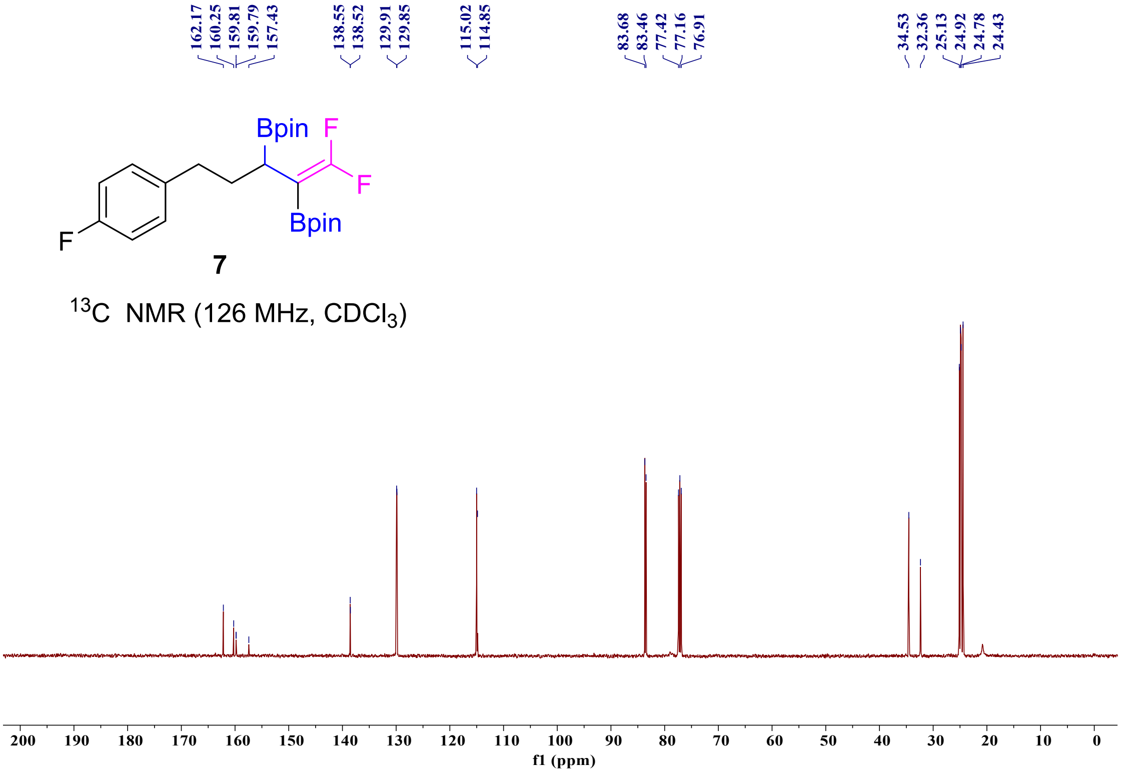

2,2'-(1,1-difluoro-5-(4-fluorophenyl)pent-1-ene-2,3-diyl)bis(4,4,5,5-tetramethyl-1,3,2-dioxaborolane) (7)

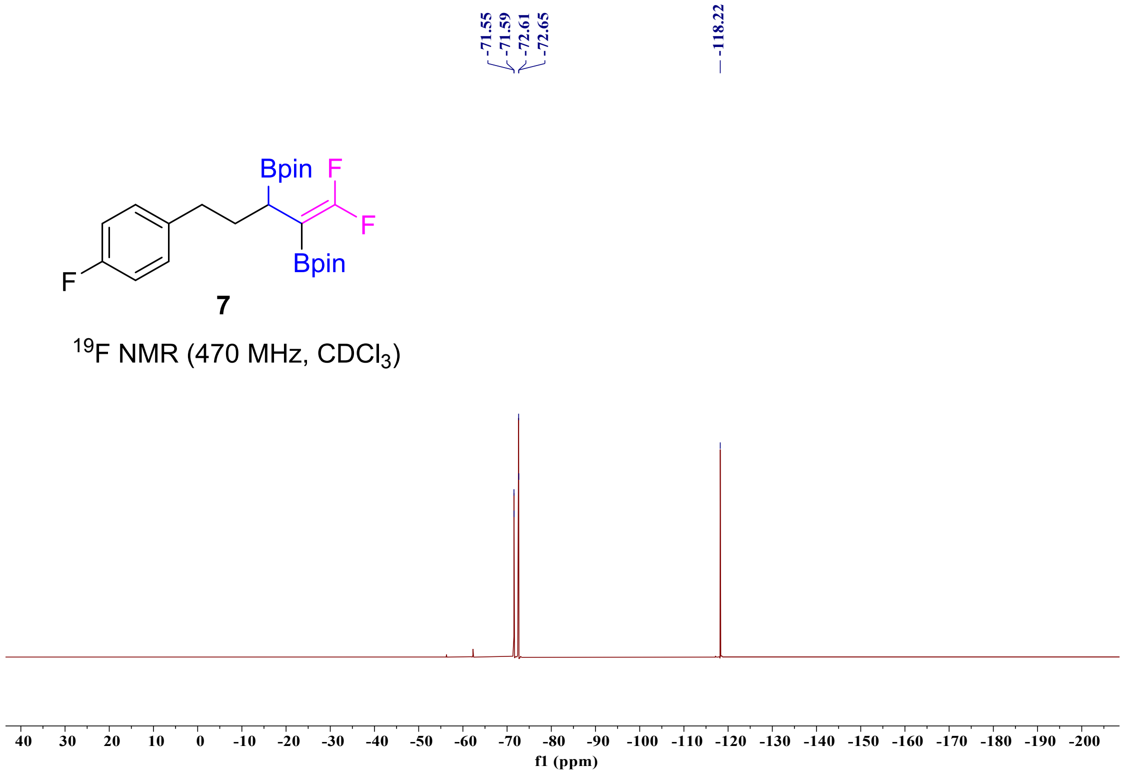

2,2'-(1,1-difluoro-5-(4-fluorophenyl)pent-1-ene-2,3-diyl)bis(4,4,5,5-tetramethyl-1,3,2-dioxaborolane) (7)

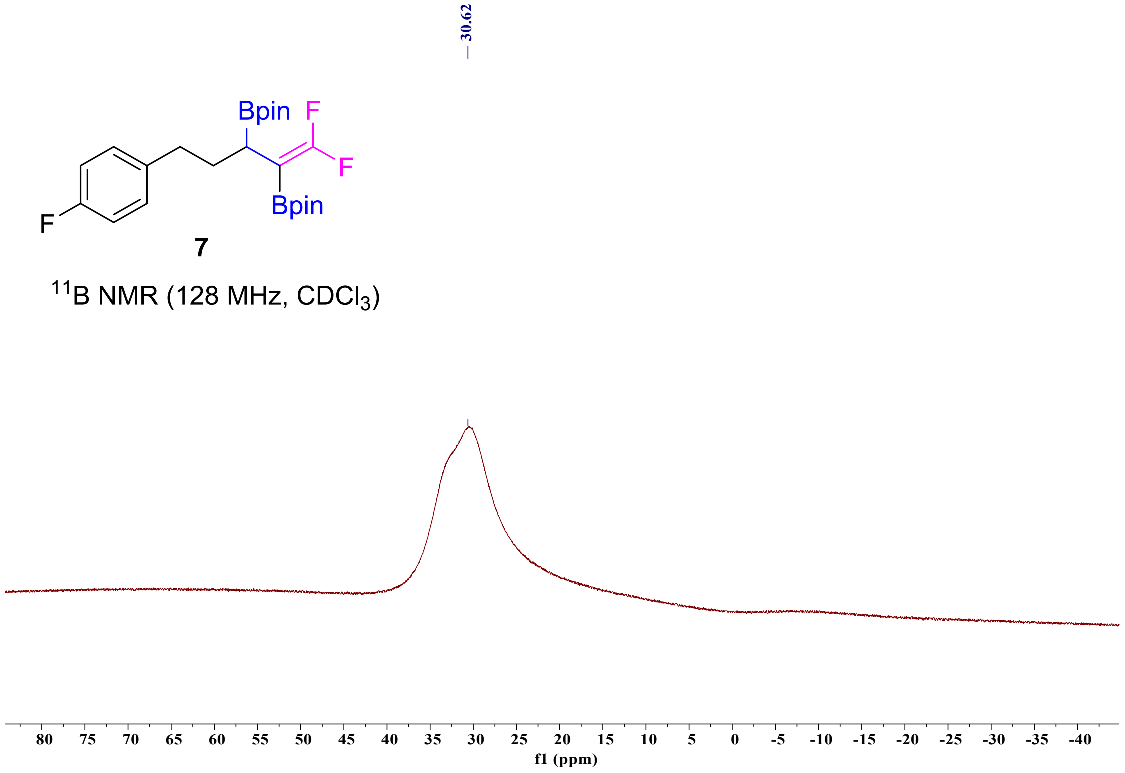

2,2'-(5-(4-chlorophenyl)-1,1-difluoropent-1-ene-2,3-diyl)bis(4,4,5,5-tetramethyl-1,3,2-dioxaborolane) (8)

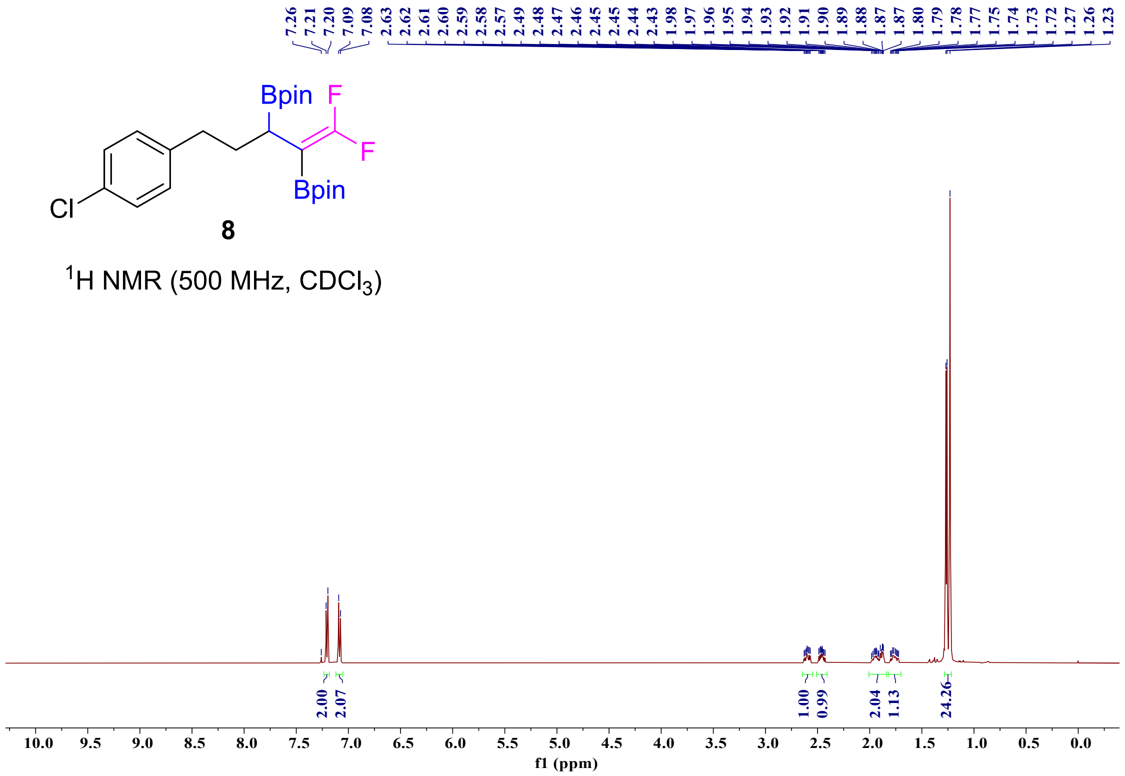

2,2'-(5-(4-chlorophenyl)-1,1-difluoropent-1-ene-2,3-diyl)bis(4,4,5,5-tetramethyl-1,3,2-dioxaborolane) (8)

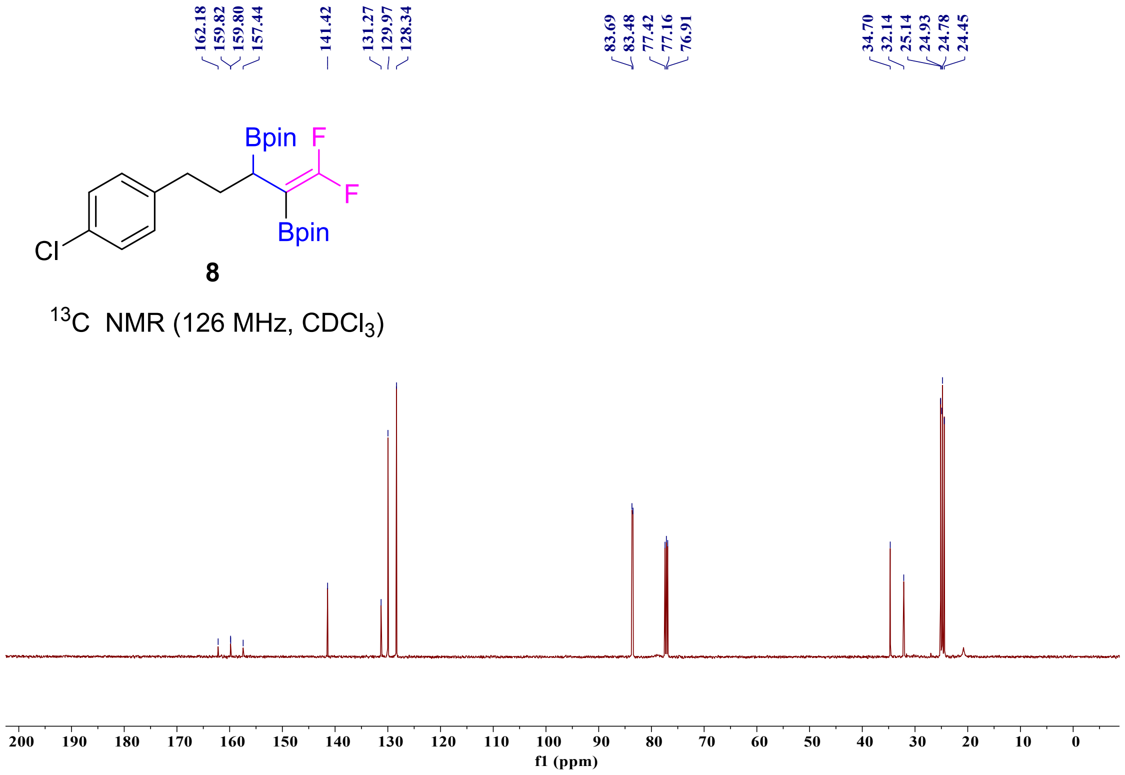

2,2'-(5-(4-chlorophenyl)-1,1-difluoropent-1-ene-2,3-diyl)bis(4,4,5,5-tetramethyl-1,3,2-dioxaborolane) (8)

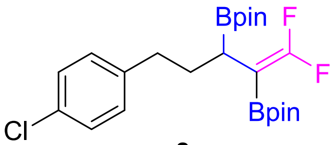

8

^{19}F NMR (470 MHz, $CDCl_3$)

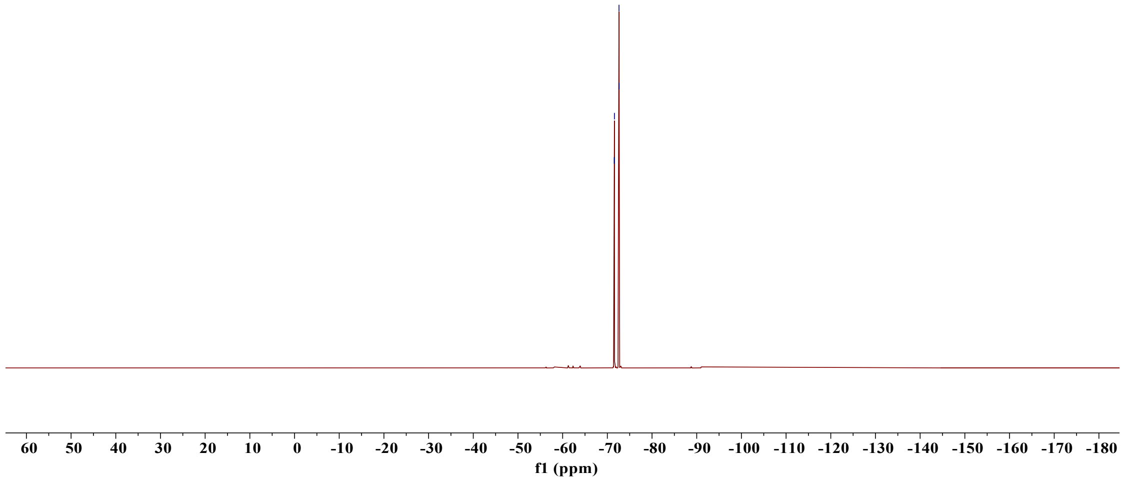

2,2'-(5-(4-chlorophenyl)-1,1-difluoropent-1-ene-2,3-diyl)bis(4,4,5,5-tetramethyl-1,3,2-dioxaborolane) (8)

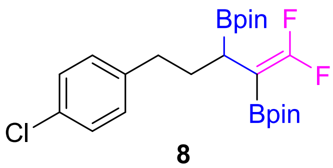

8

^{11}B NMR (128 MHz, $CDCl_3$)

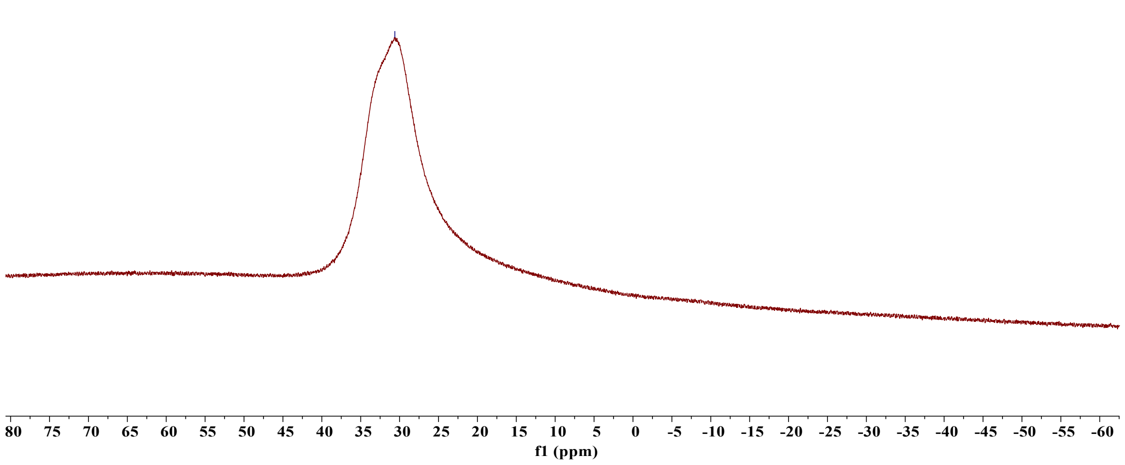

4-(5,5-difluoro-3,4-bis(4,4,5,5-tetramethyl-1,3,2-dioxaborolan-2-yl)pent-4-en-1-yl)phenyl acetate (9)

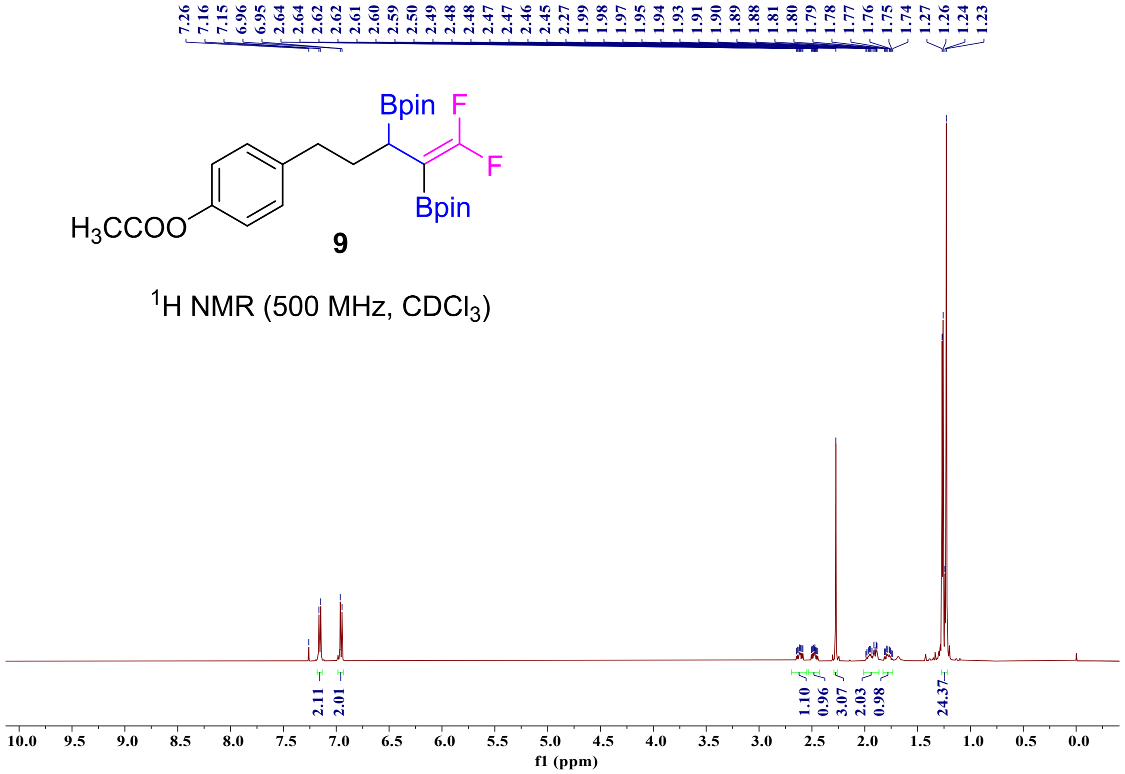

4-(5,5-difluoro-3,4-bis(4,4,5,5-tetramethyl-1,3,2-dioxaborolan-2-yl)pent-4-en-1-yl)phenyl acetate (9)

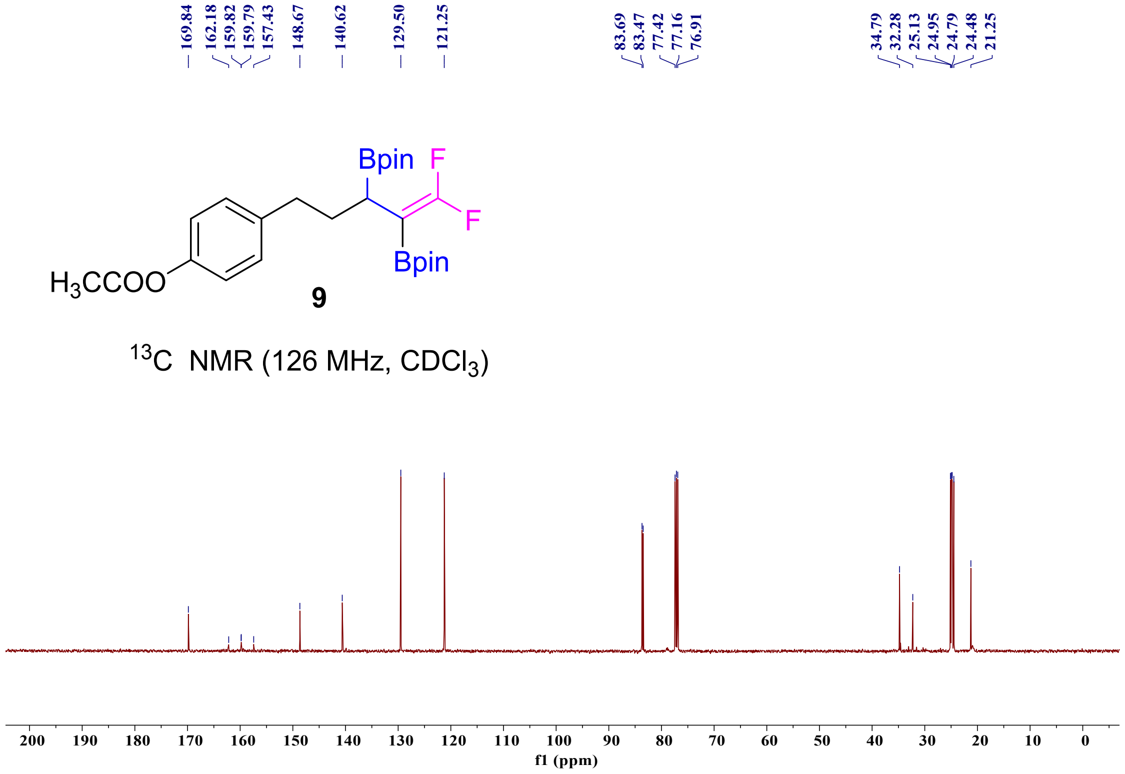

4-(5,5-difluoro-3,4-bis(4,4,5,5-tetramethyl-1,3,2-dioxaborolan-2-yl)pent-4-en-1-yl)phenyl acetate (9)

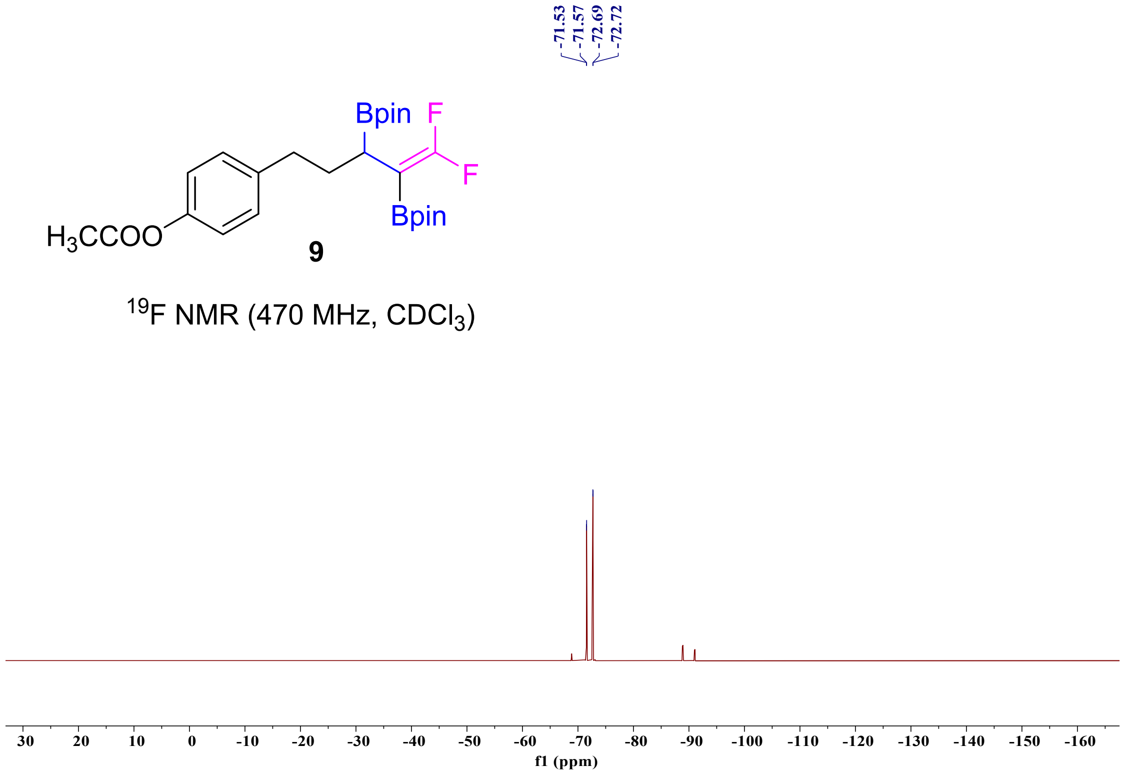

4-(5,5-difluoro-3,4-bis(4,4,5,5-tetramethyl-1,3,2-dioxaborolan-2-yl)pent-4-en-1-yl)phenyl acetate (9)

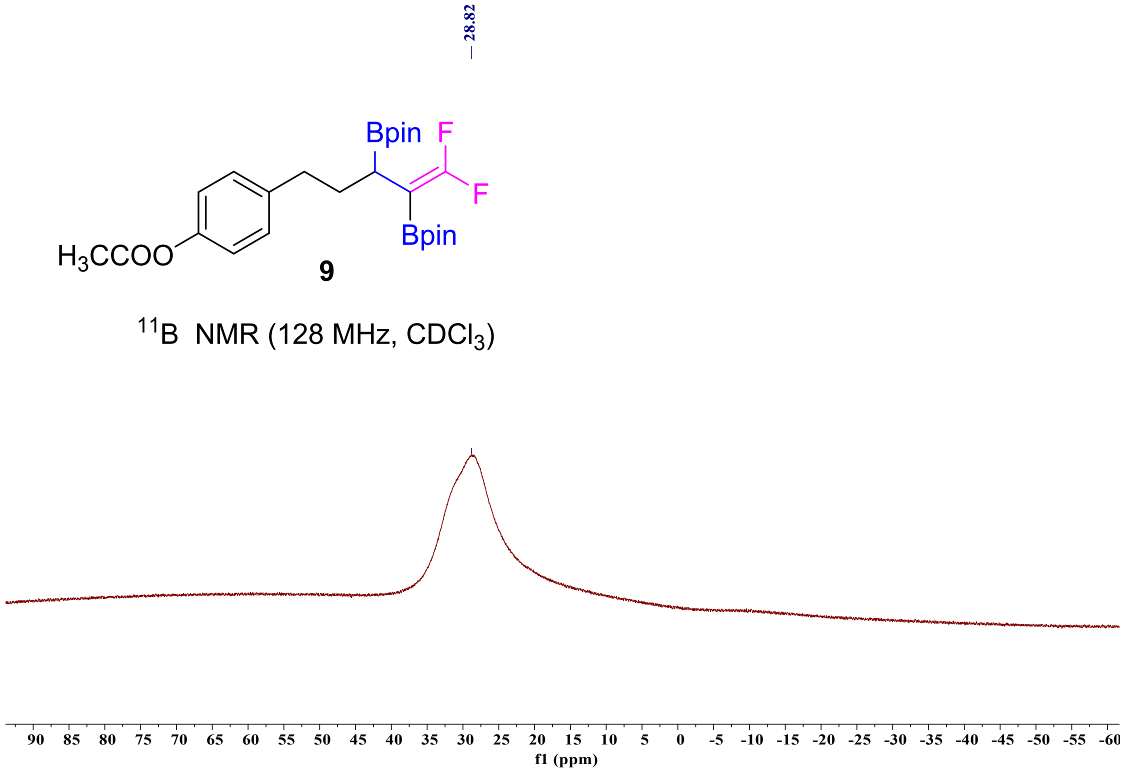

2,2'-(5-(2-bromophenyl)-1,1-difluoropent-1-ene-2,3-diyl)bis(4,4,5,5-tetramethyl-1,3,2-dioxaborolane) (10)

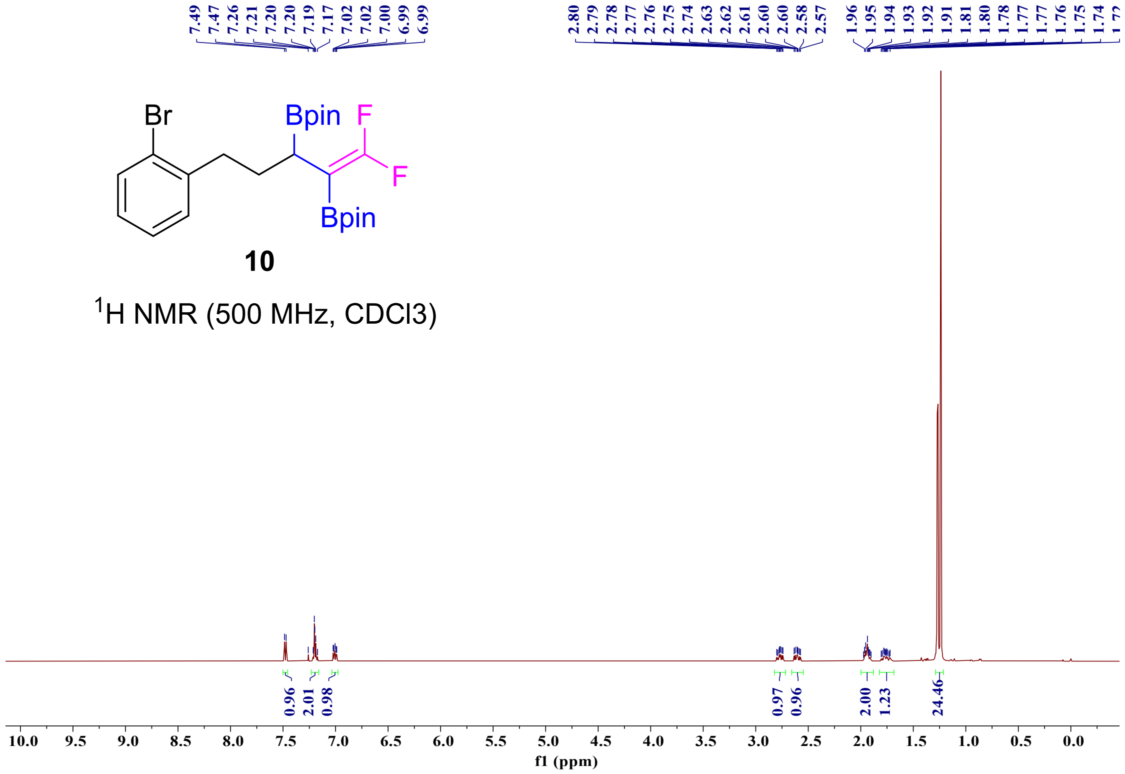

2,2'-(5-(2-bromophenyl)-1,1-difluoropent-1-ene-2,3-diyl)bis(4,4,5,5-tetramethyl-1,3,2-dioxaborolane) (10)

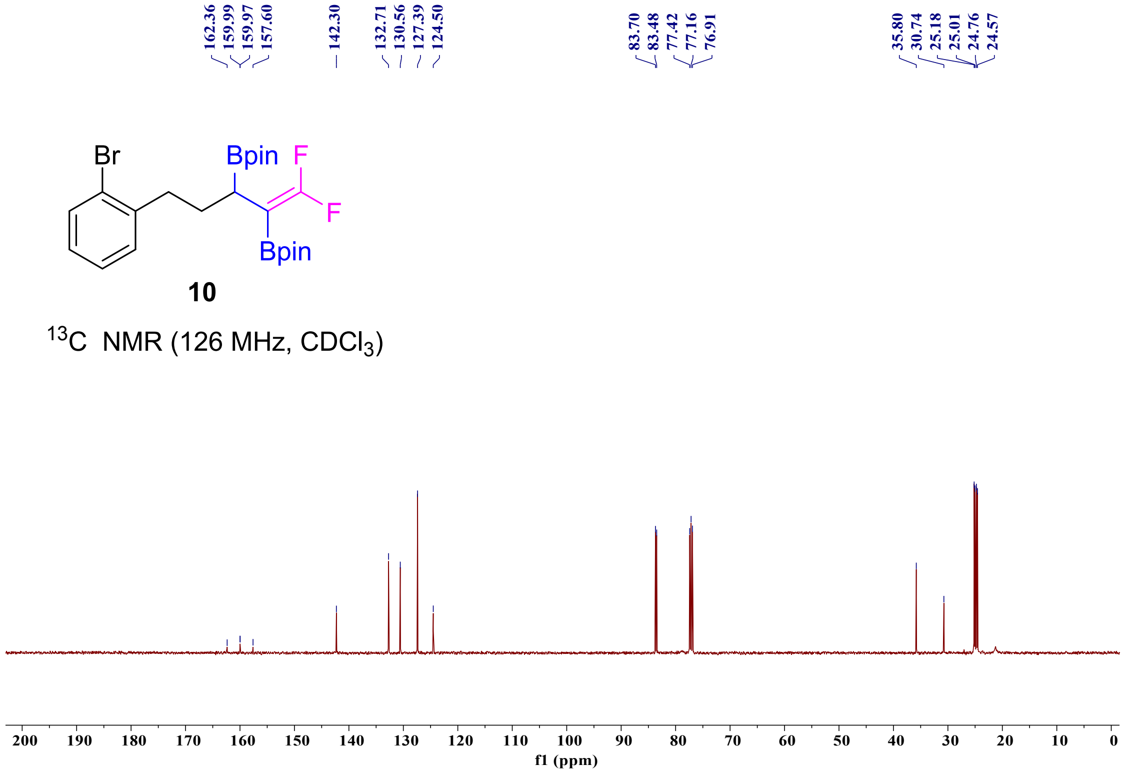

2,2'-(5-(2-bromophenyl)-1,1-difluoropent-1-ene-2,3-diyl)bis(4,4,5,5-tetramethyl-1,3,2-dioxaborolane) (10)

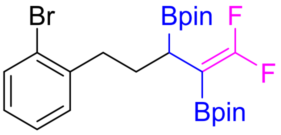

10

^{19}F NMR (470 MHz, $CDCl_3$)

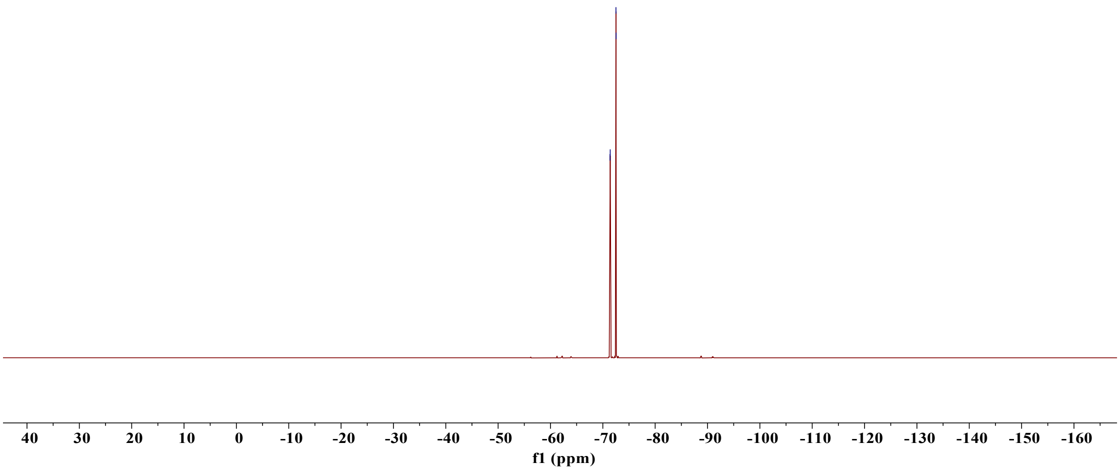

2,2'-(5-(2-bromophenyl)-1,1-difluoropent-1-ene-2,3-diyl)bis(4,4,5,5-tetramethyl-1,3,2-dioxaborolane) (10)

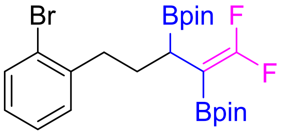

10

^{11}B NMR (128 MHz, $CDCl_3$)

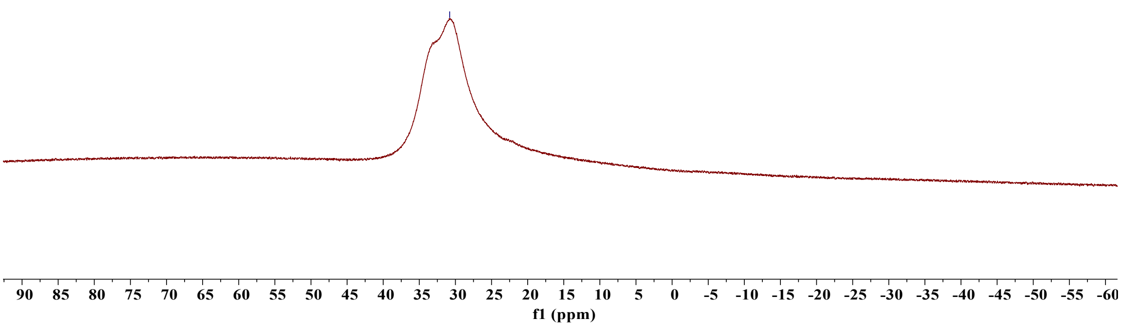

2,2'-(5-(3-bromophenyl)-1,1-difluoropent-1-ene-2,3-diyl)bis(4,4,5,5-tetramethyl-1,3,2-dioxaborolane) (11)

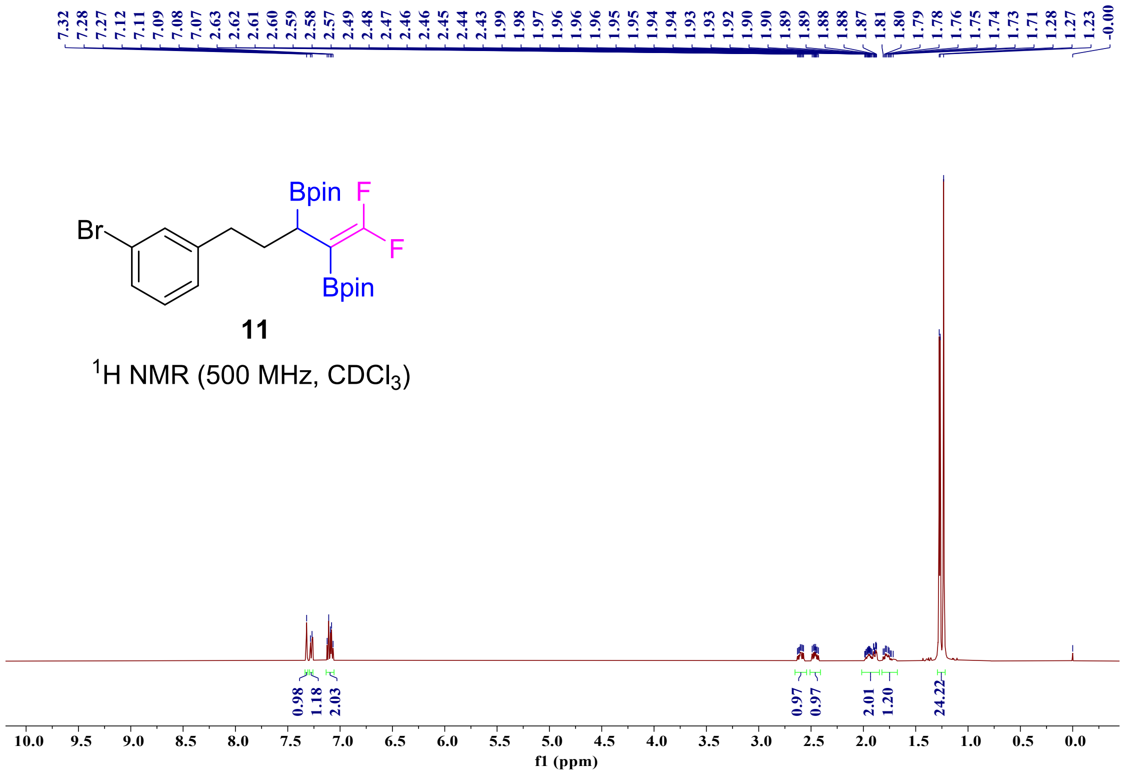

2,2'-(5-(3-bromophenyl)-1,1-difluoropent-1-ene-2,3-diyl)bis(4,4,5,5-tetramethyl-1,3,2-dioxaborolane) (11)

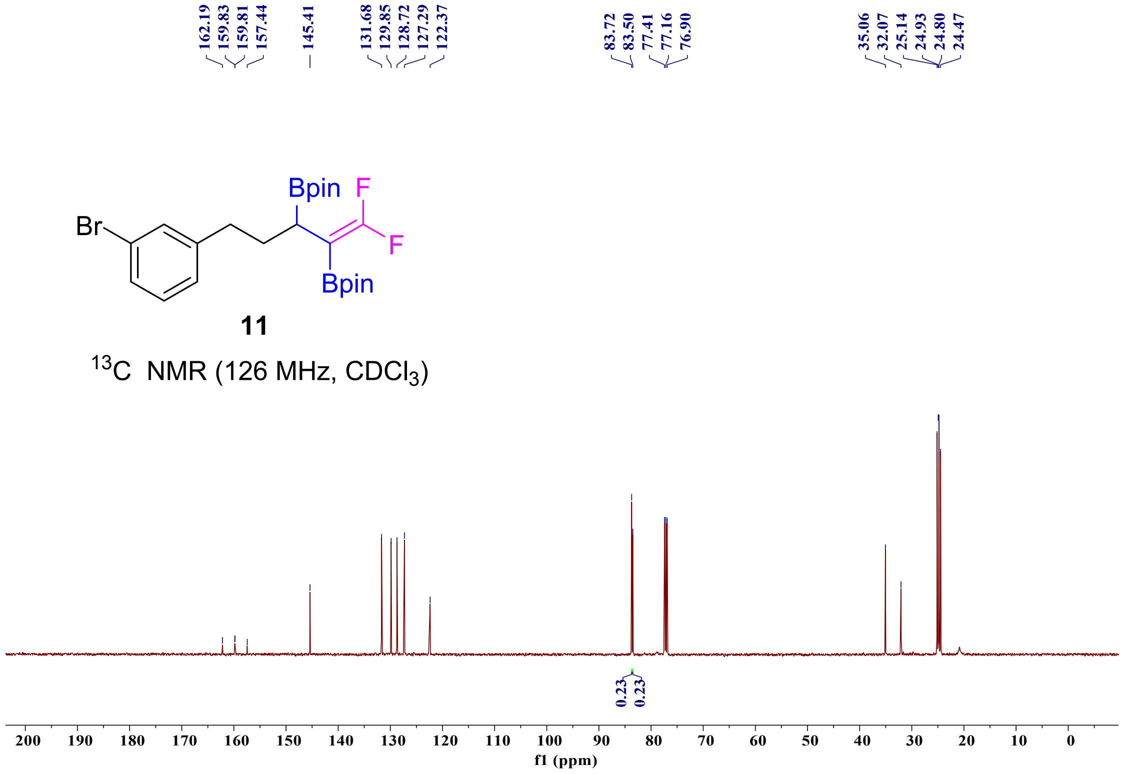

2,2'-(5-(3-bromophenyl)-1,1-difluoropent-1-ene-2,3-diyl)bis(4,4,5,5-tetramethyl-1,3,2-dioxaborolane) (11)

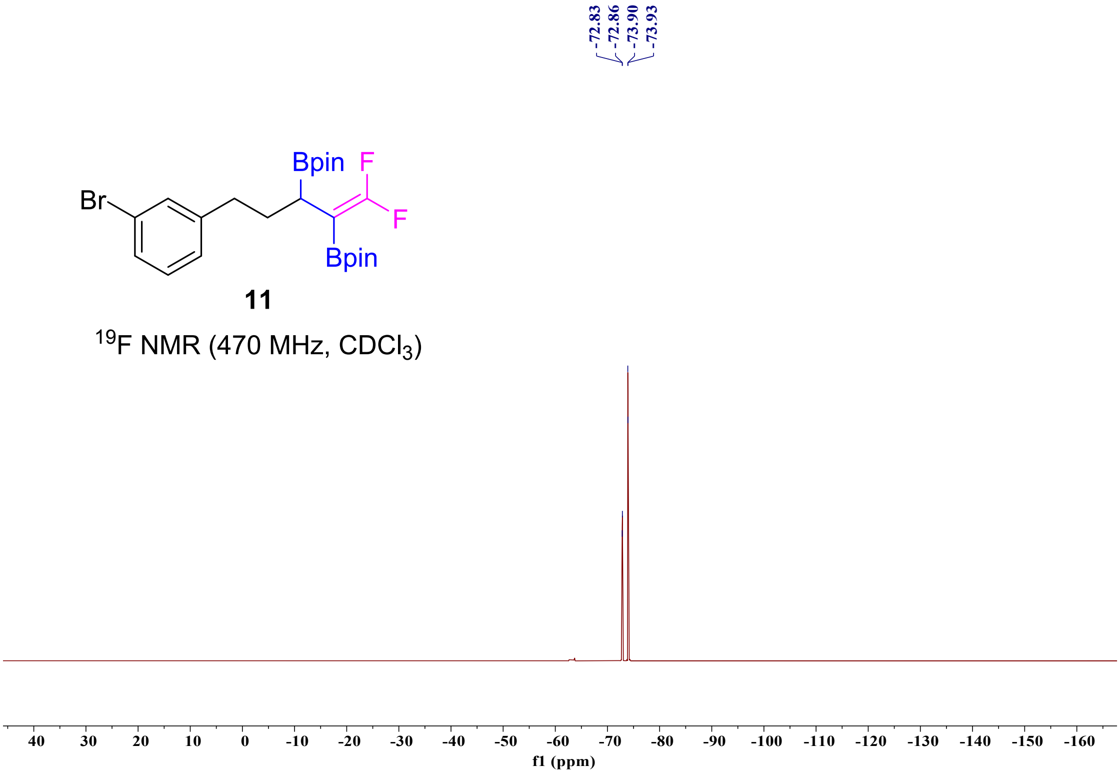

2,2'-(5-(3-bromophenyl)-1,1-difluoropent-1-ene-2,3-diyl)bis(4,4,5,5-tetramethyl-1,3,2-dioxaborolane) (11)

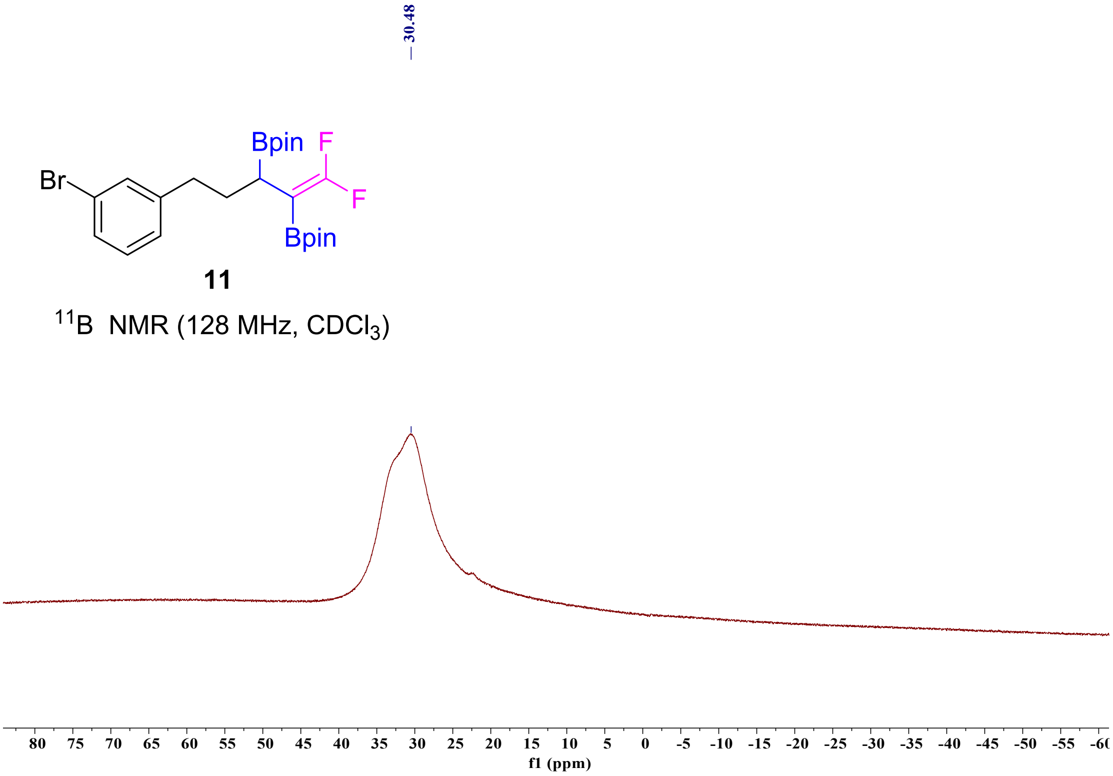

2,2'-(5-(4-bromophenyl)-1,1-difluoropent-1-ene-2,3-diyl)bis(4,4,5,5-tetramethyl-1,3,2-dioxaborolane) (12)

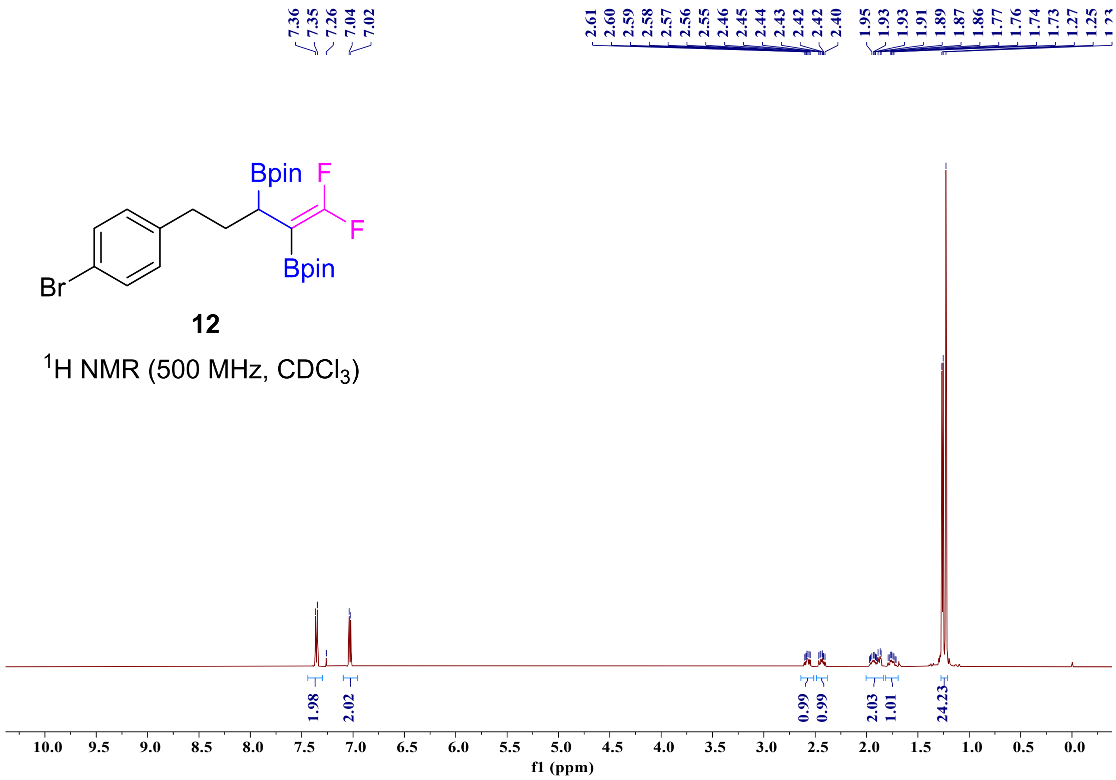

2,2'-(5-(4-bromophenyl)-1,1-difluoropent-1-ene-2,3-diyl)bis(4,4,5,5-tetramethyl-1,3,2-dioxaborolane) (12)

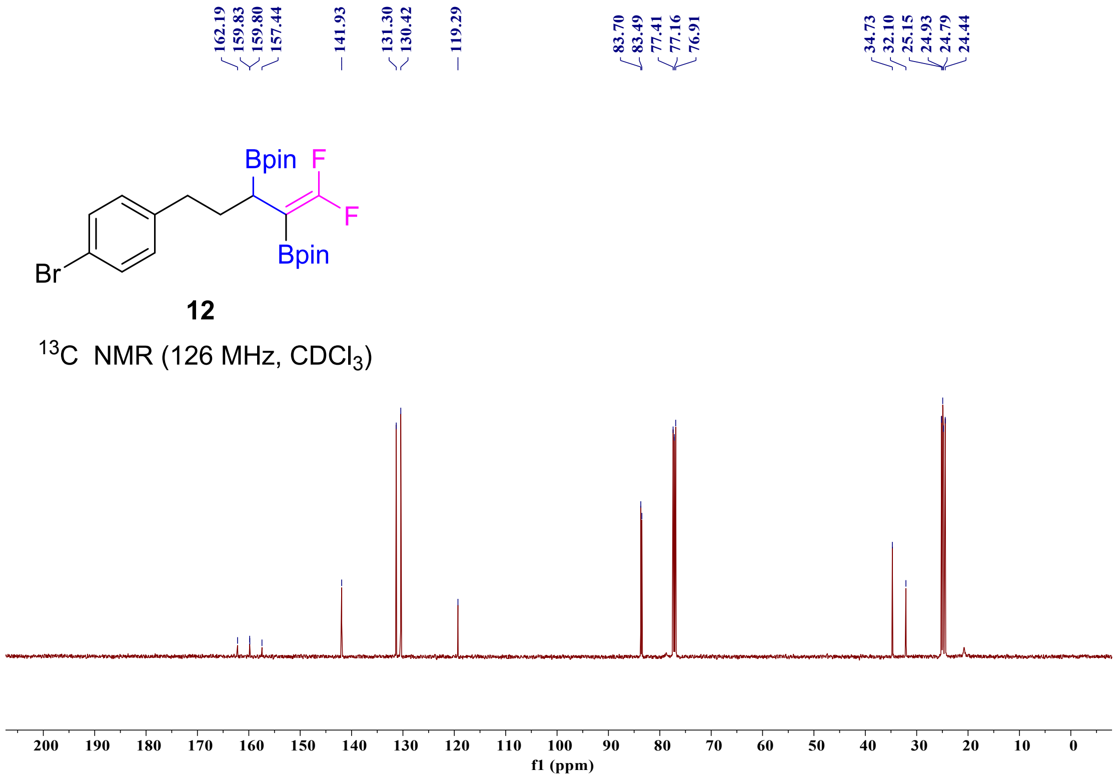

2,2'-(5-(4-bromophenyl)-1,1-difluoropent-1-ene-2,3-diyl)bis(4,4,5,5-tetramethyl-1,3,2-dioxaborolane) (12)

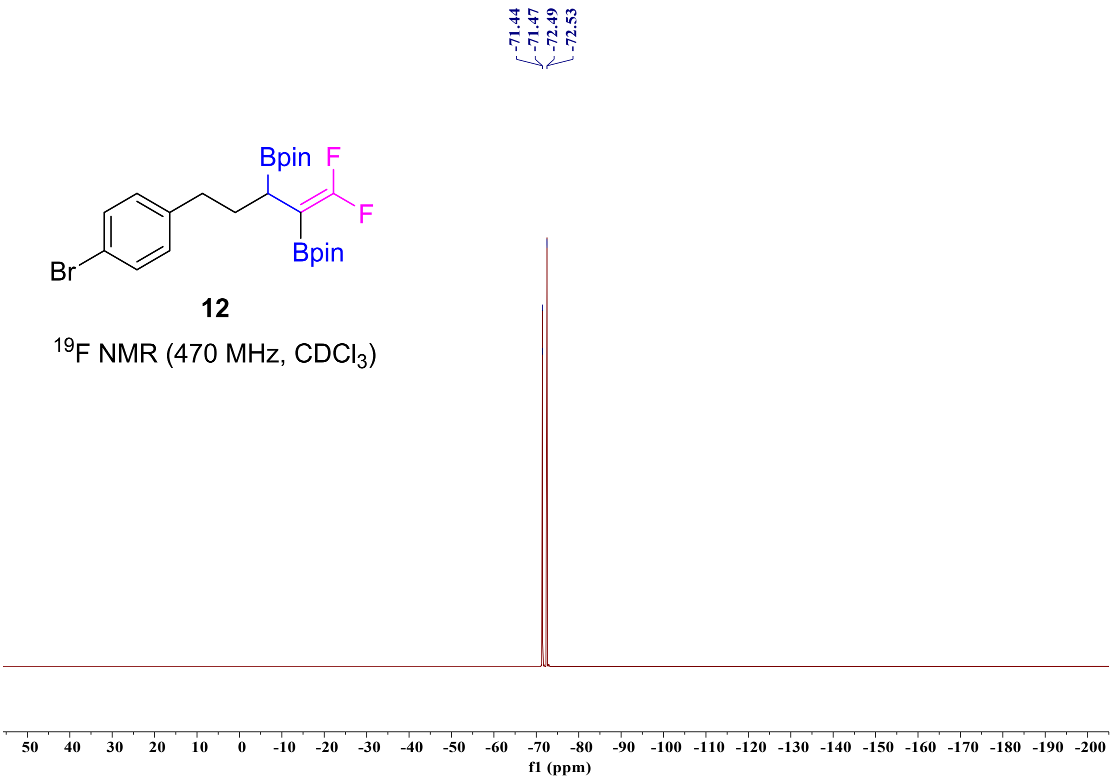

2,2'-(5-(4-bromophenyl)-1,1-difluoropent-1-ene-2,3-diyl)bis(4,4,5,5-tetramethyl-1,3,2-dioxaborolane) (12)

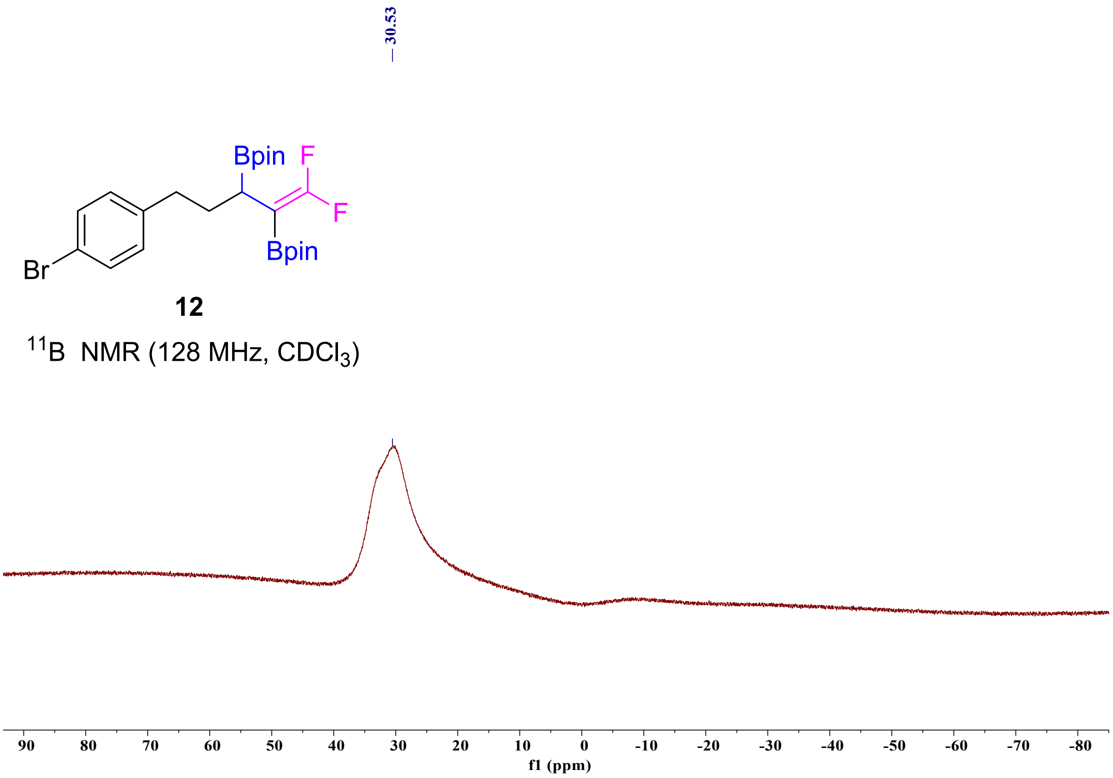

2,2'-(1,1-difluoro-4-phenylbut-1-ene-2,3-diyl)bis(4,4,5,5-tetramethyl-1,3,2-dioxaborolane) (13)

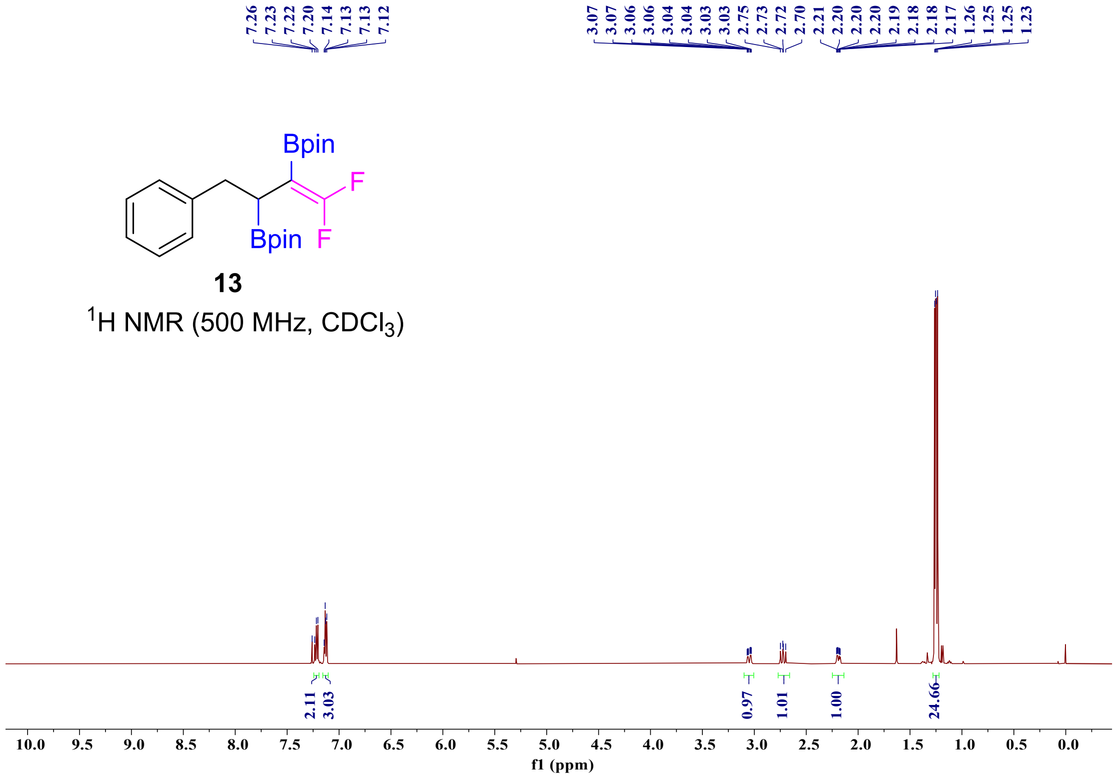

2,2'-(1,1-difluoro-4-phenylbut-1-ene-2,3-diyl)bis(4,4,5,5-tetramethyl-1,3,2-dioxaborolane) (13)

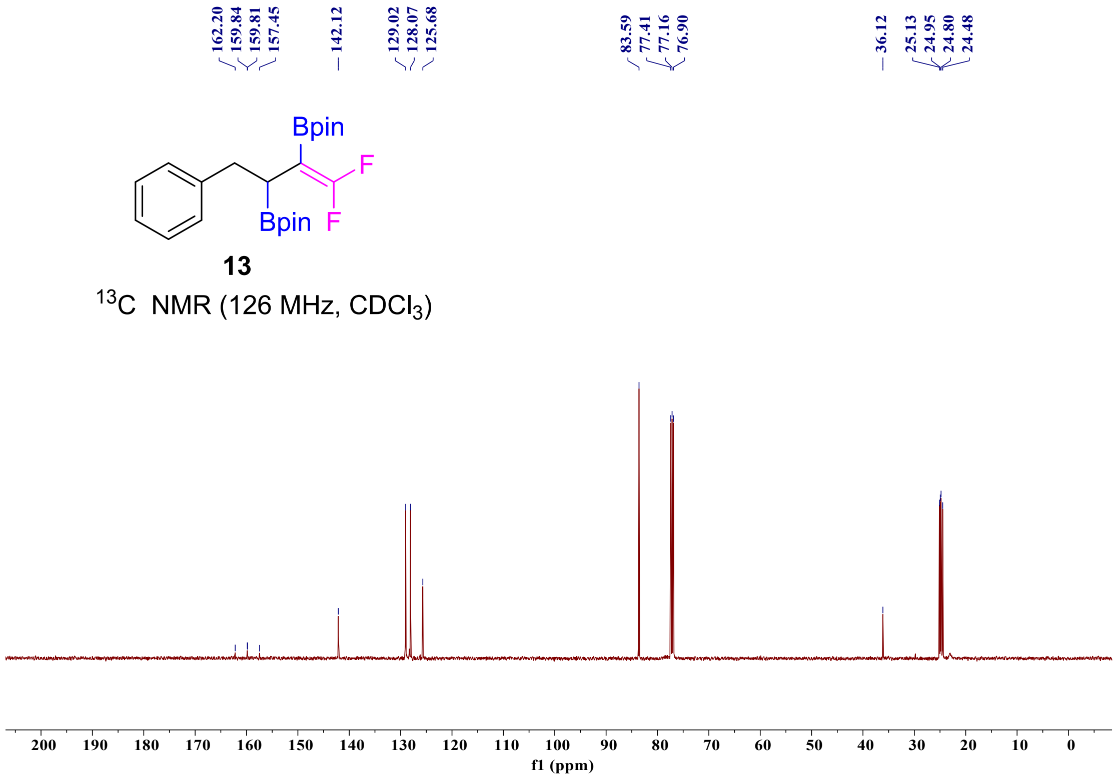

2,2'-(1,1-difluoro-4-phenylbut-1-ene-2,3-diyl)bis(4,4,5,5-tetramethyl-1,3,2-dioxaborolane) (13)

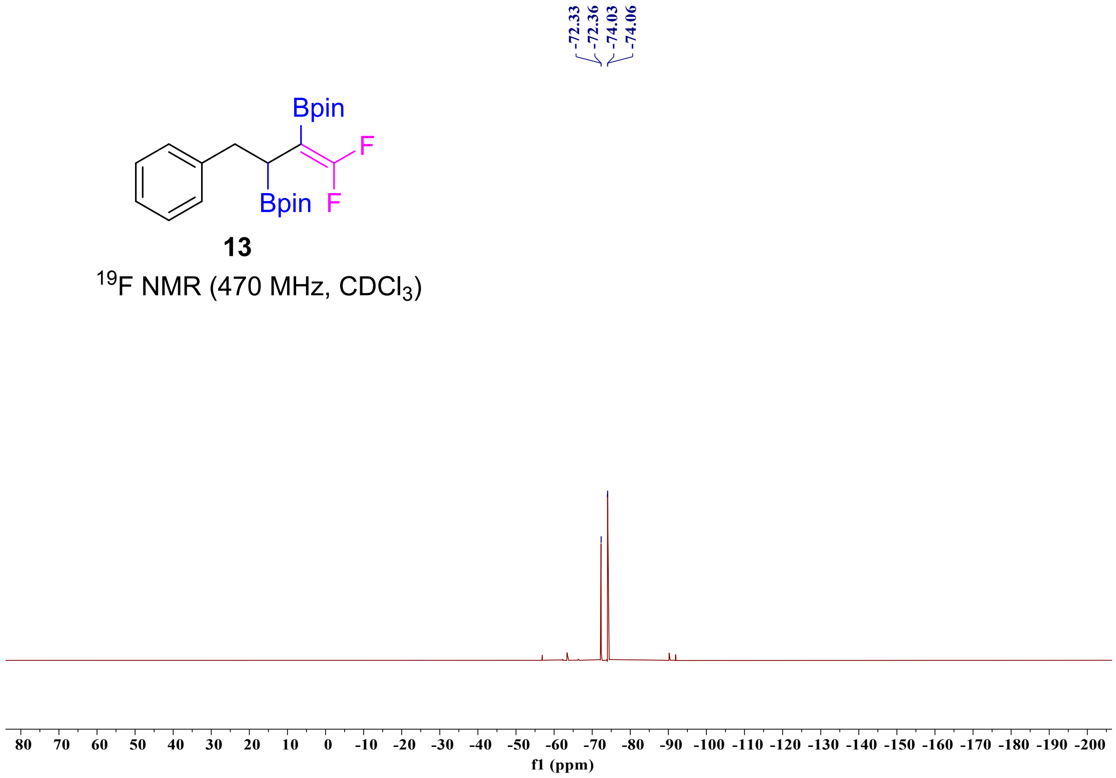

2,2'-(1,1-difluoro-4-phenylbut-1-ene-2,3-diyl)bis(4,4,5,5-tetramethyl-1,3,2-dioxaborolane) (13)

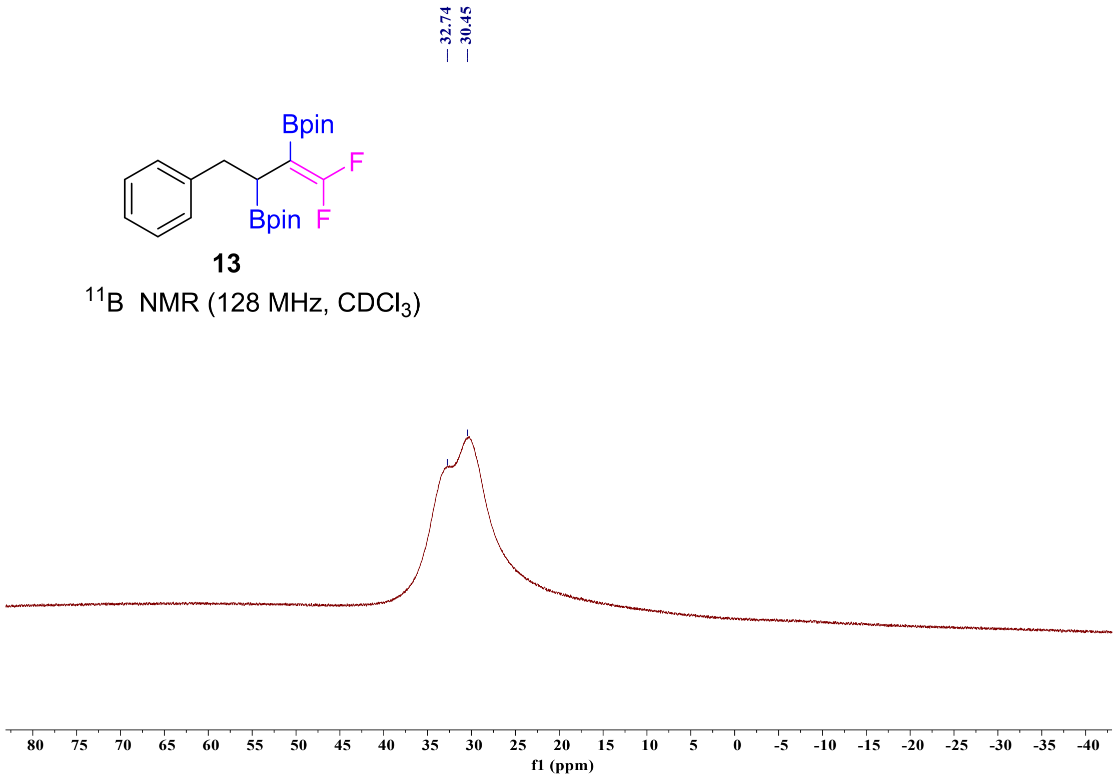

2,2'-(1,1-difluoro-4-(4-methoxyphenyl)but-1-ene-2,3-diyl)bis(4,4,5,5-tetramethyl-1,3,2-dioxaborolane) (14)

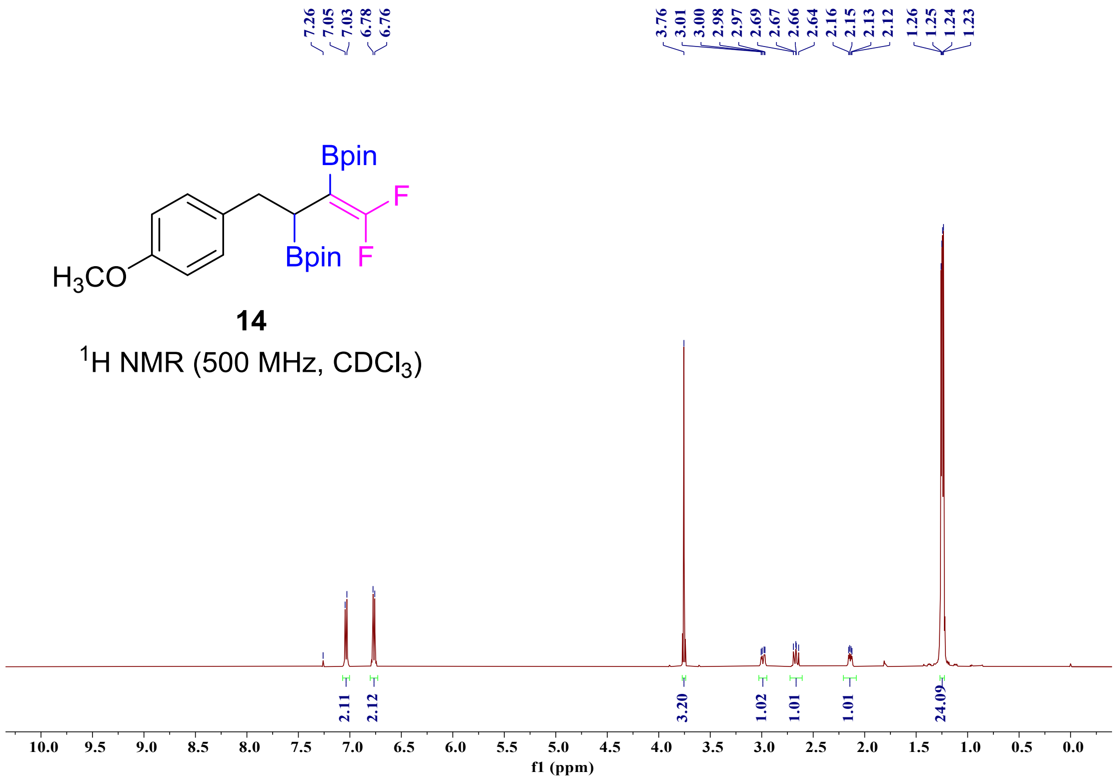

2,2'-(1,1-difluoro-4-(4-methoxyphenyl)but-1-ene-2,3-diyl)bis(4,4,5,5-tetramethyl-1,3,2-dioxaborolane) (14)

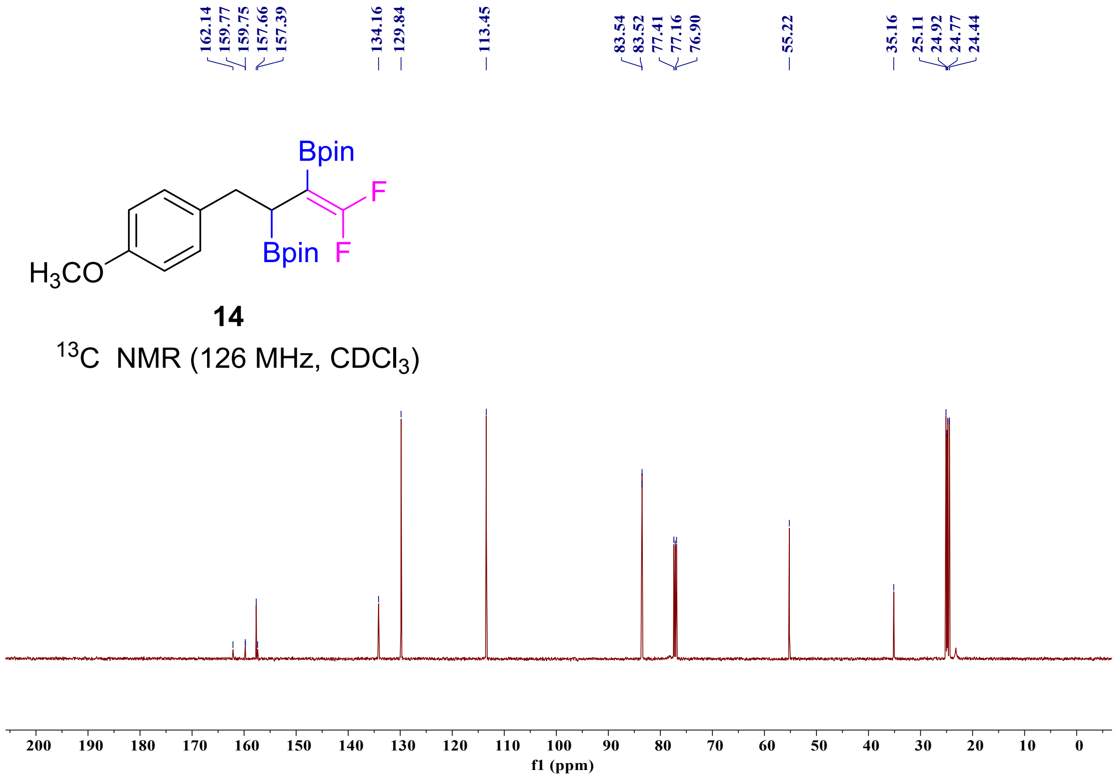

2,2'-(1,1-difluoro-4-(4-methoxyphenyl)but-1-ene-2,3-diyl)bis(4,4,5,5-tetramethyl-1,3,2-dioxaborolane) (14)

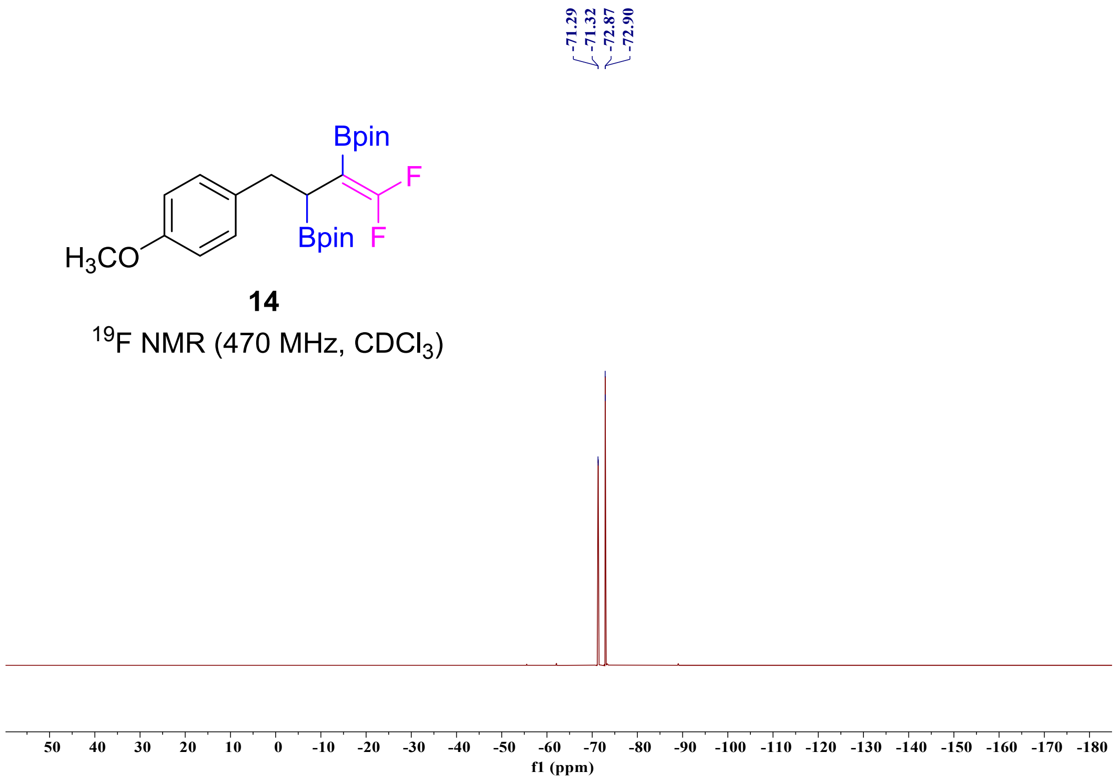

2,2'-(1,1-difluoro-4-(4-methoxyphenyl)but-1-ene-2,3-diyl)bis(4,4,5,5-tetramethyl-1,3,2-dioxaborolane) (14)

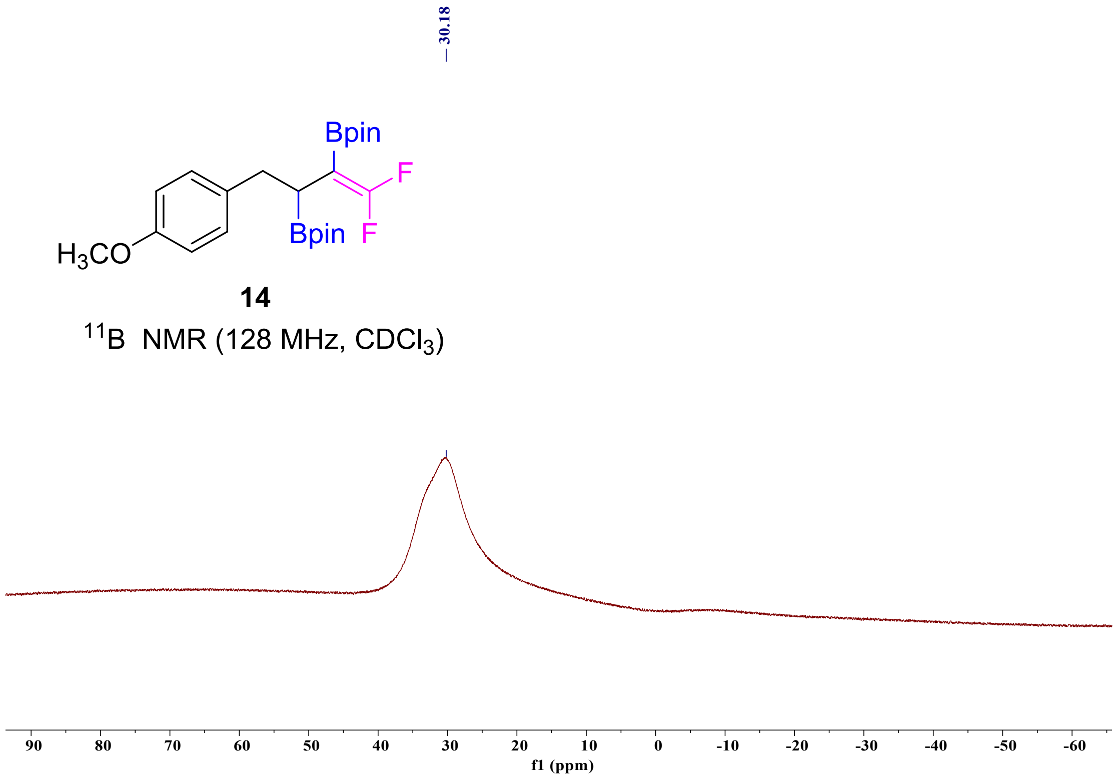

2,2'-(4-(4-bromophenyl)-1,1-difluorobut-1-ene-2,3-diyl)bis(4,4,5,5-tetramethyl-1,3,2-dioxaborolane) (15)

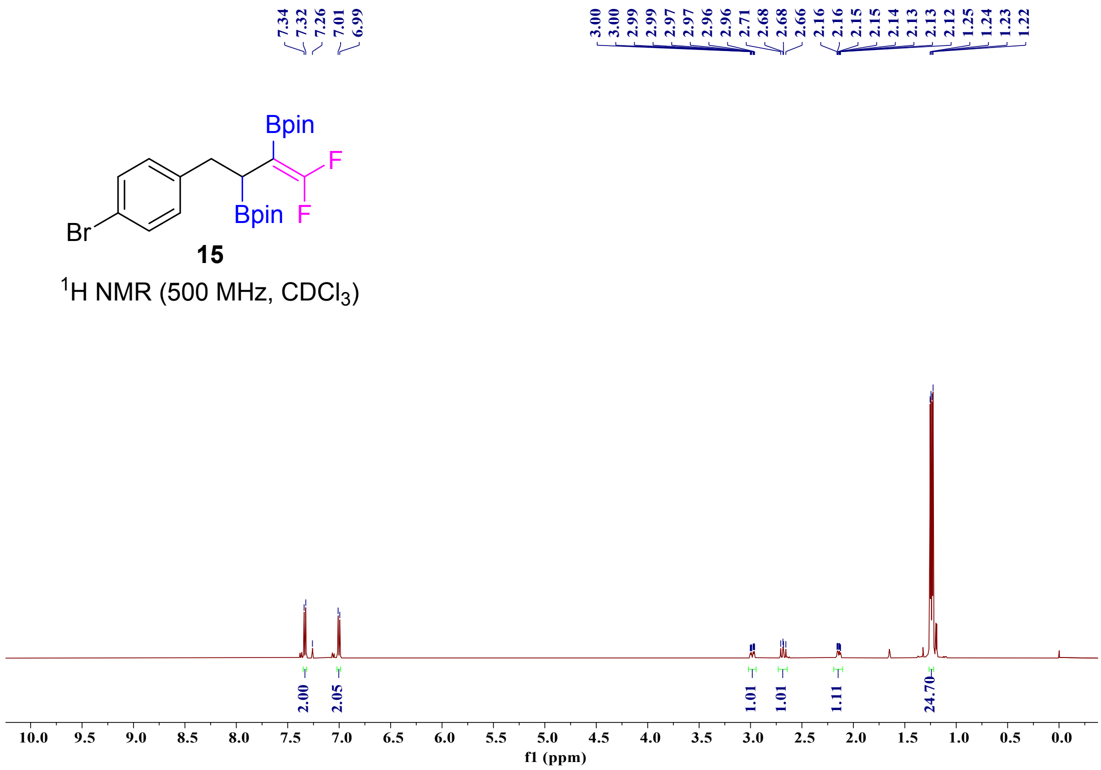

2,2'-(4-(4-bromophenyl)-1,1-difluorobut-1-ene-2,3-diyl)bis(4,4,5,5-tetramethyl-1,3,2-dioxaborolane) (15)

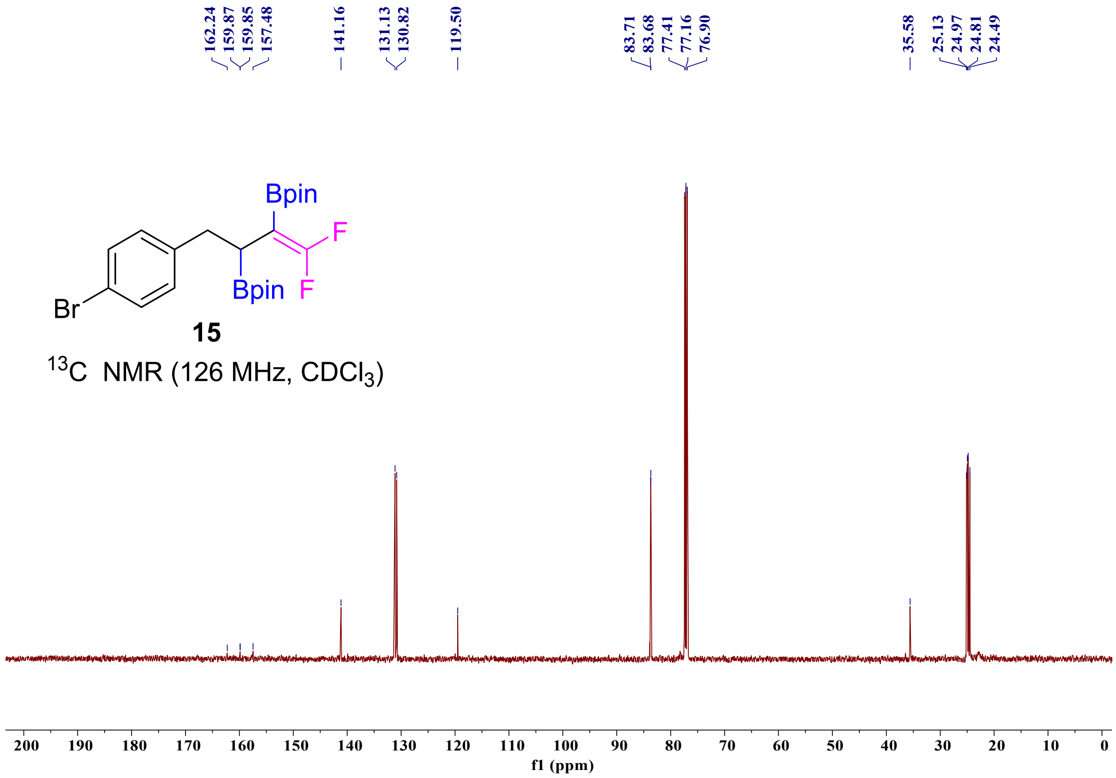

2,2'-(4-(4-bromophenyl)-1,1-difluorobut-1-ene-2,3-diyl)bis(4,4,5,5-tetramethyl-1,3,2-dioxaborolane) (15)

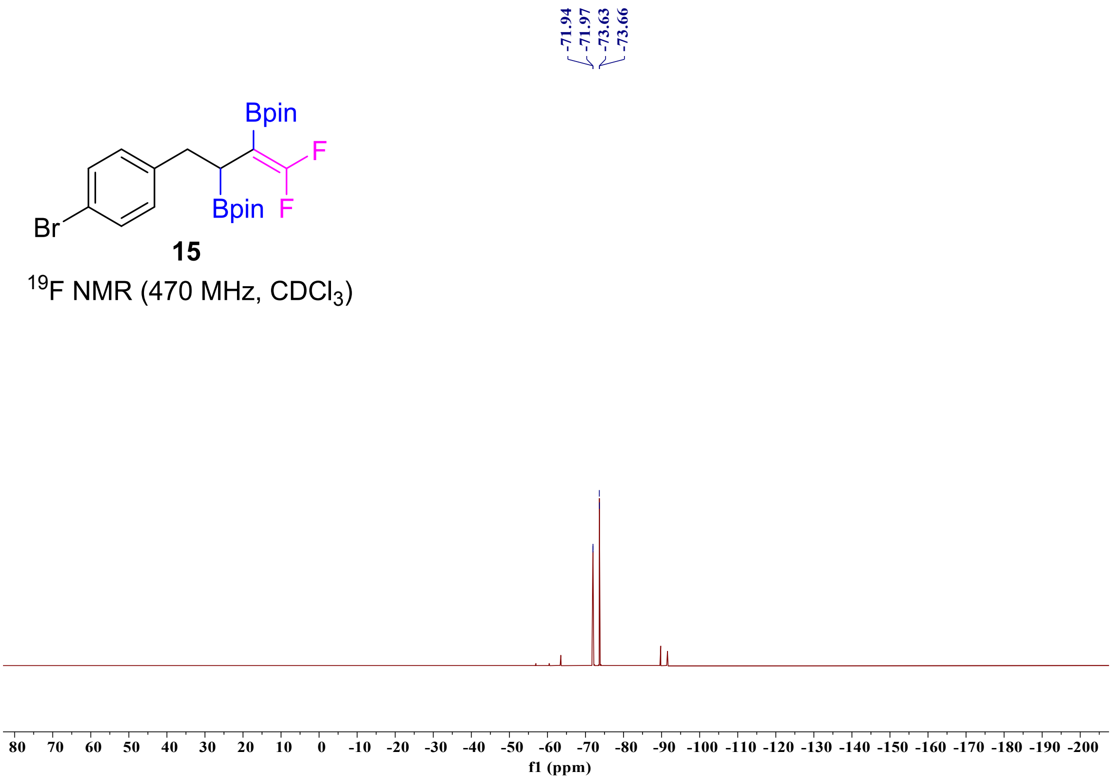

2,2'-(4-(4-bromophenyl)-1,1-difluorobut-1-ene-2,3-diyl)bis(4,4,5,5-tetramethyl-1,3,2-dioxaborolane) (15)

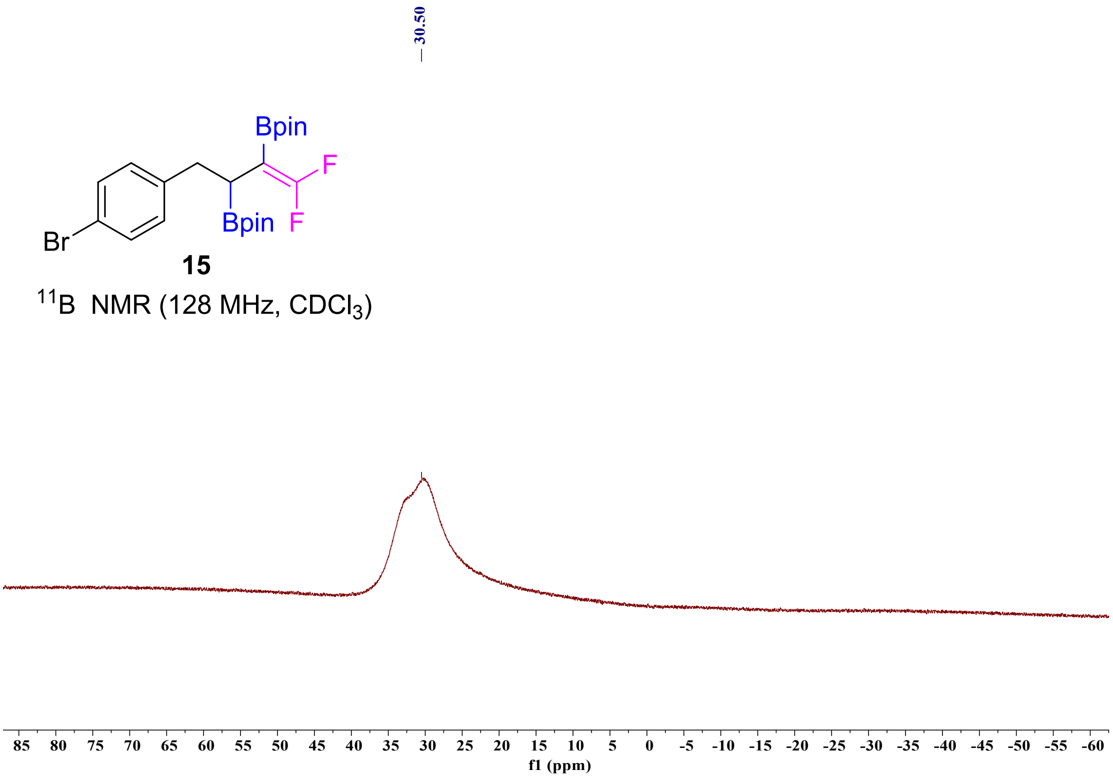

2,2'-(1,1-difluoro-6-phenylhex-1-ene-2,3-diyl)bis(4,4,5,5-tetramethyl-1,3,2-dioxaborolane) (16)

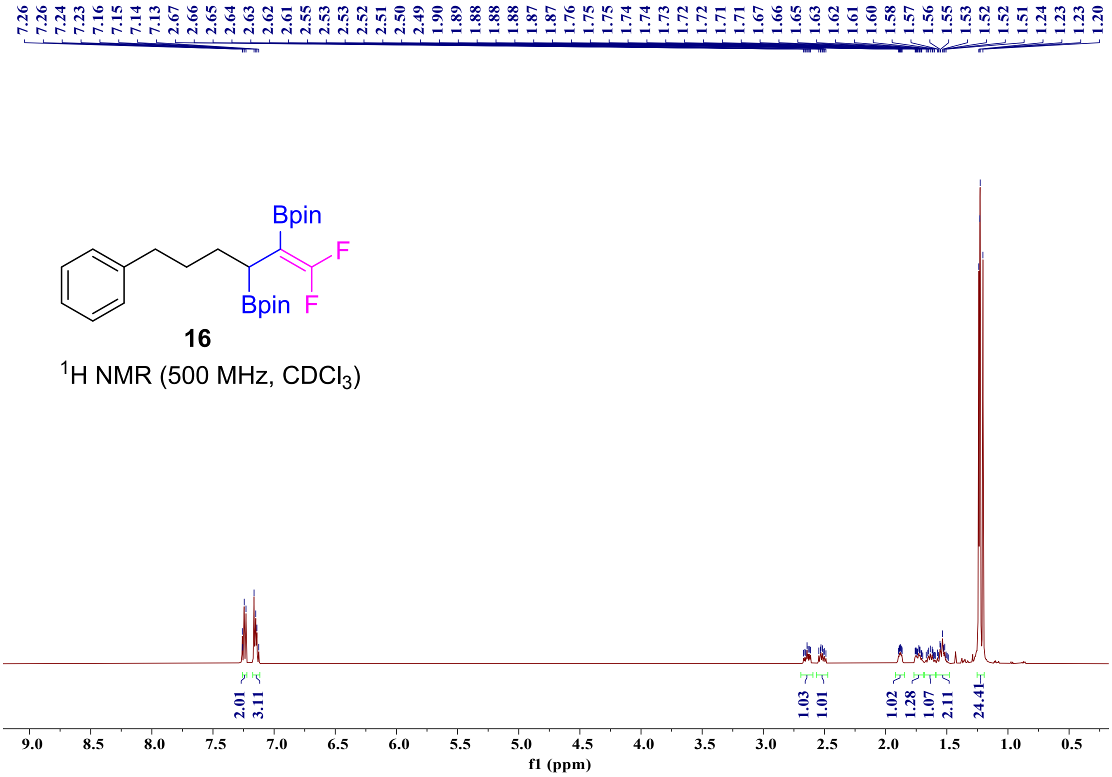

2,2'-(1,1-difluoro-6-phenylhex-1-ene-2,3-diyl)bis(4,4,5,5-tetramethyl-1,3,2-dioxaborolane) (16)

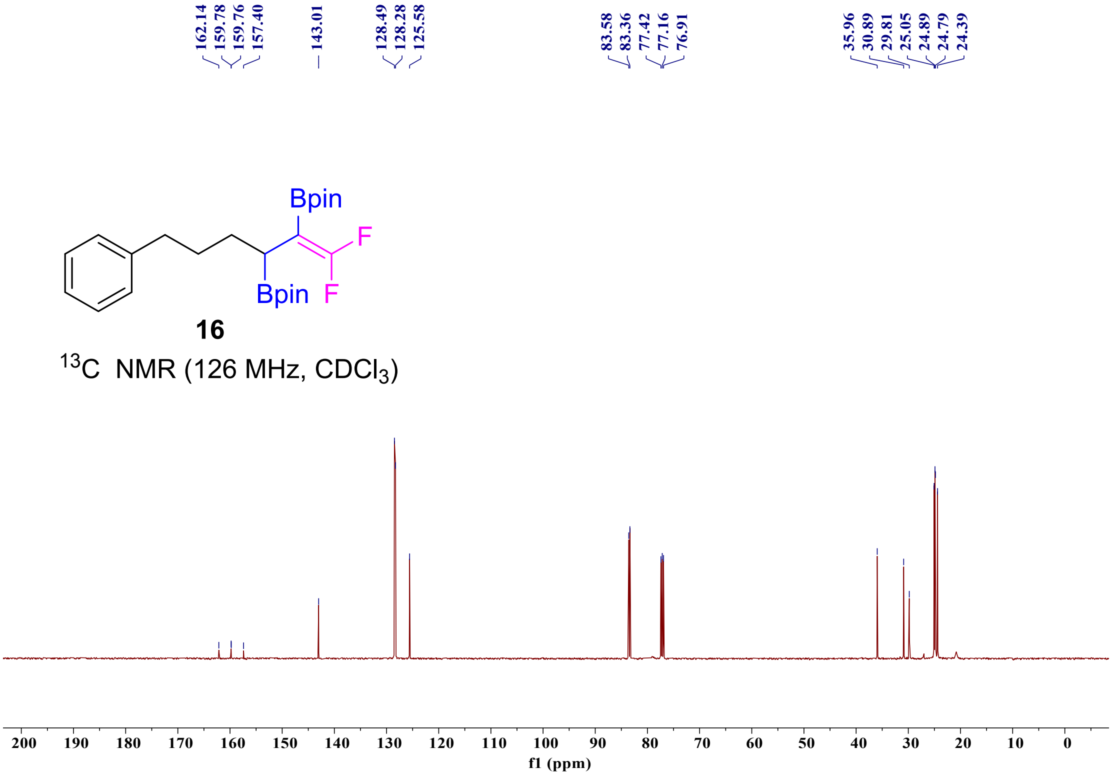

2,2'-(1,1-difluoro-6-phenylhex-1-ene-2,3-diyl)bis(4,4,5,5-tetramethyl-1,3,2-dioxaborolane) (16)

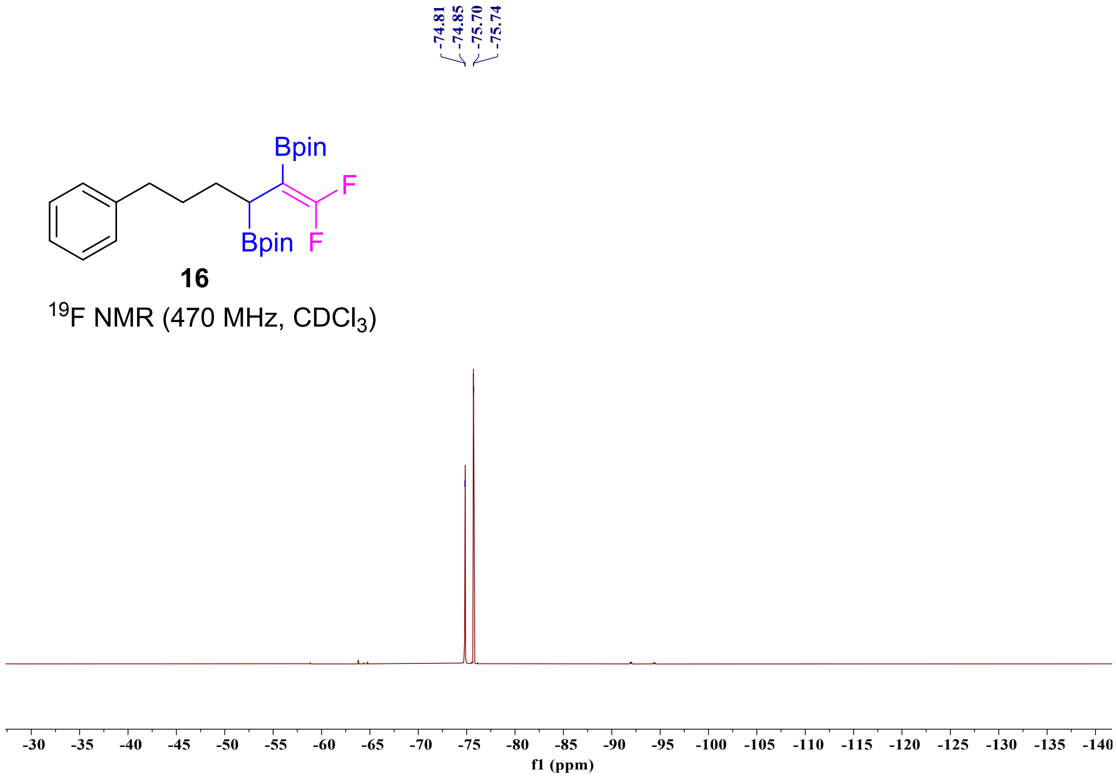

2,2'-(1,1-difluoro-6-phenylhex-1-ene-2,3-diyl)bis(4,4,5,5-tetramethyl-1,3,2-dioxaborolane) (16)

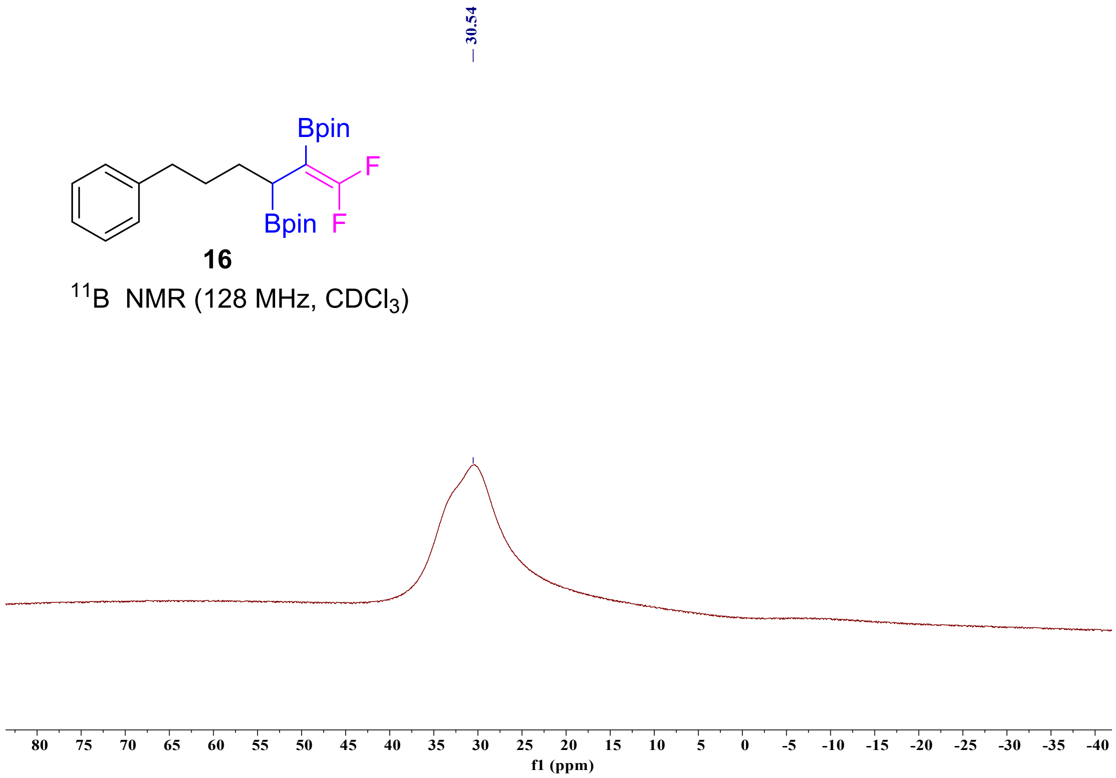

2,2'-(1,1-difluoro-5,5-diphenylpent-1-ene-2,3-diyl)bis(4,4,5,5-tetramethyl-1,3,2-dioxaborolane) (17)

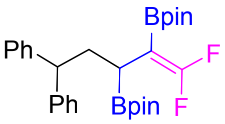

17

^{1}H NMR (500 MHz, $CDCl_3$)

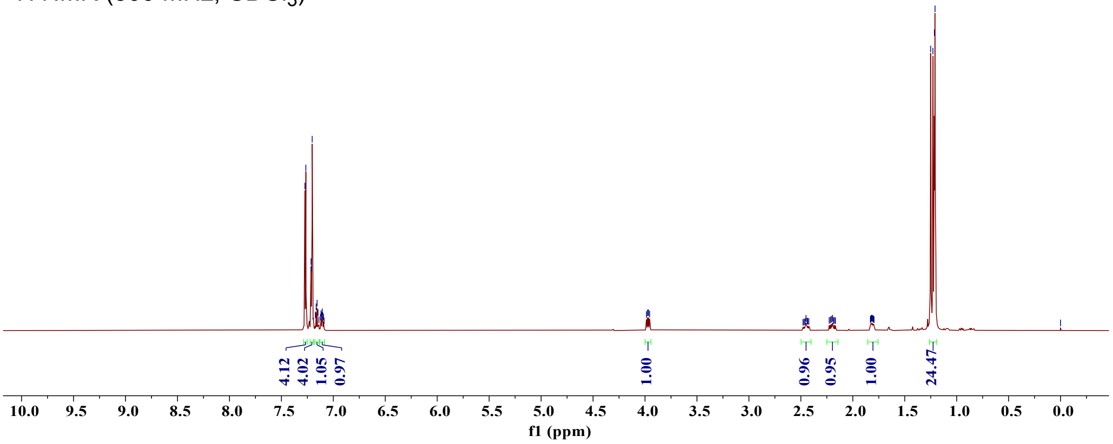

2,2'-(1,1-difluoro-5,5-diphenylpent-1-ene-2,3-diyl)bis(4,4,5,5-tetramethyl-1,3,2-dioxaborolane) (17)

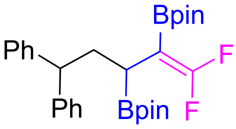

17

^{13}C NMR (126 MHz, $CDCl_3$)

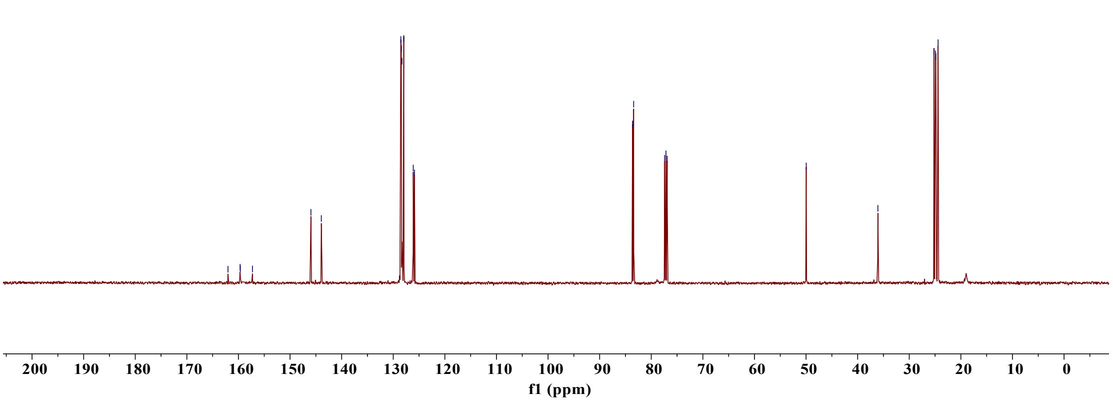

2,2'-(1,1-difluoro-5,5-diphenylpent-1-ene-2,3-diyl)bis(4,4,5,5-tetramethyl-1,3,2-dioxaborolane) (17)

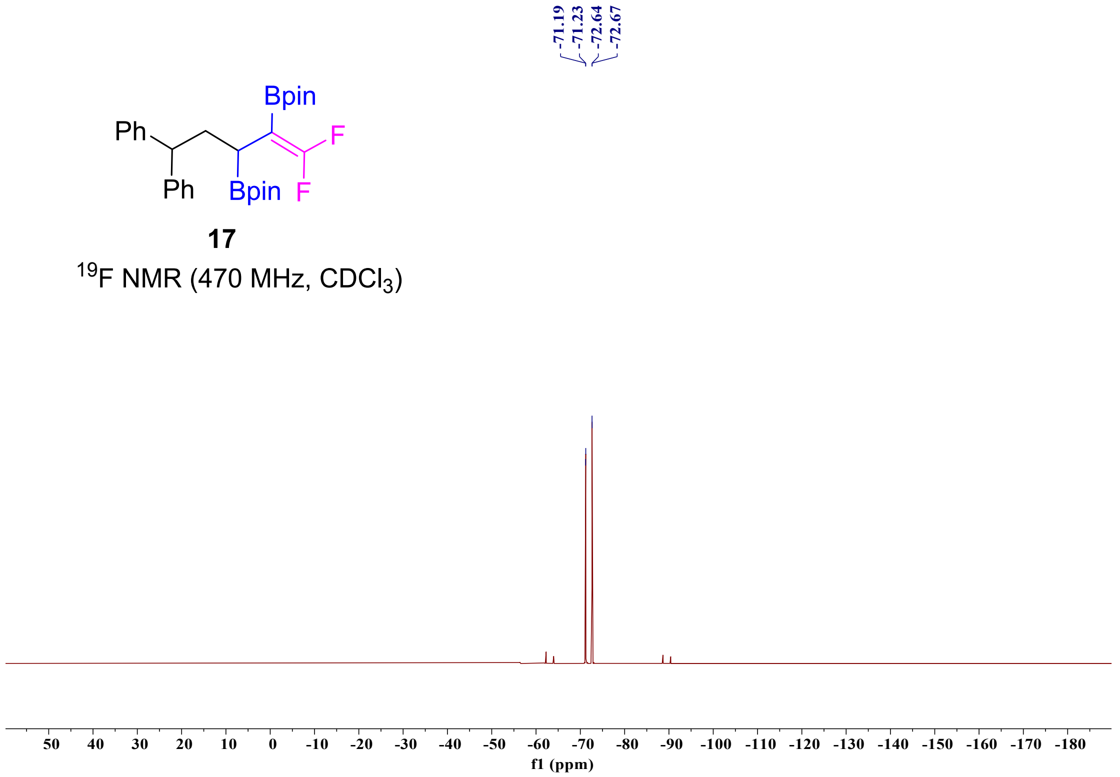

2,2'-(1,1-difluoro-5,5-diphenylpent-1-ene-2,3-diyl)bis(4,4,5,5-tetramethyl-1,3,2-dioxaborolane) (17)

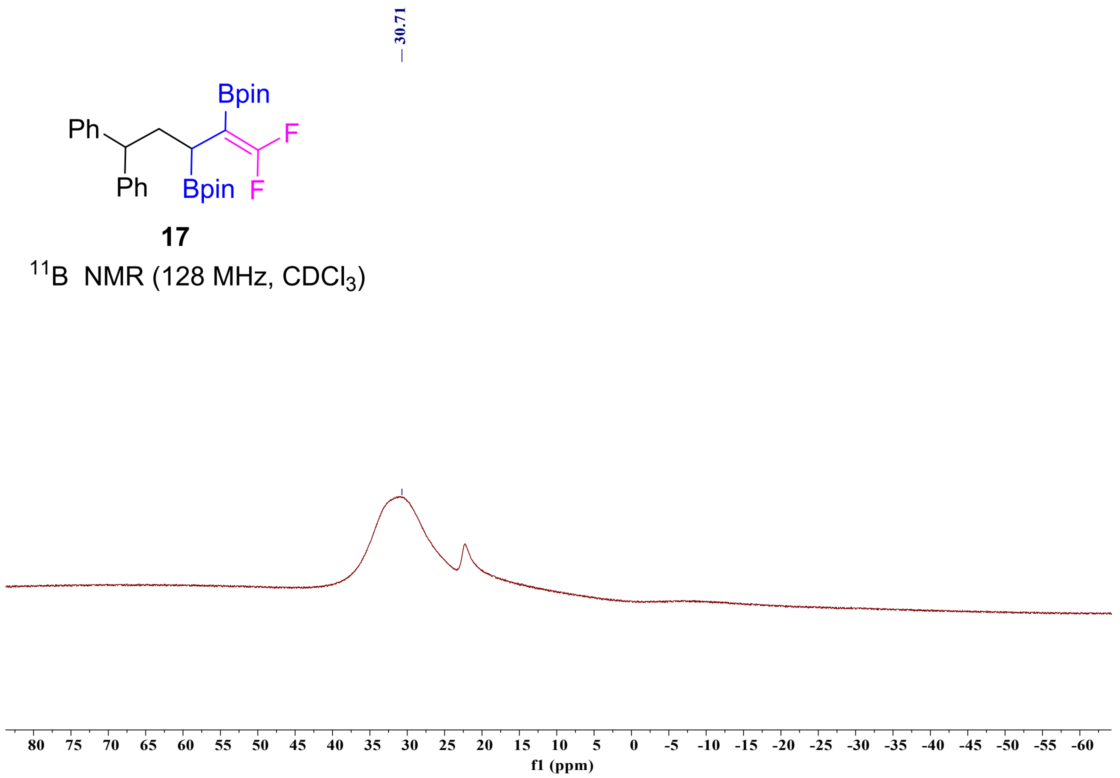

2,2'-(1,1-difluoro-4-(naphthalen-1-yl)but-1-ene-2,3-diyl)bis(4,4,5,5-tetramethyl-1,3,2-dioxaborolane) (18)

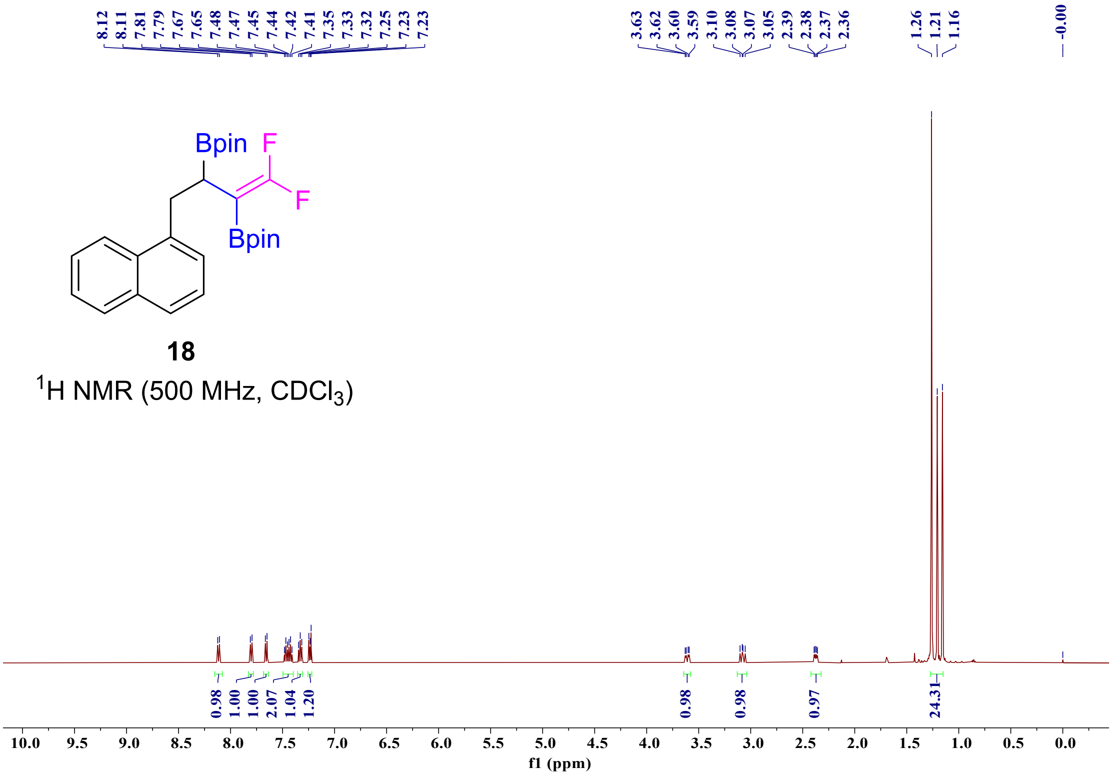

2,2'-(1,1-difluoro-4-(naphthalen-1-yl)but-1-ene-2,3-diyl)bis(4,4,5,5-tetramethyl-1,3,2-dioxaborolane) (18)

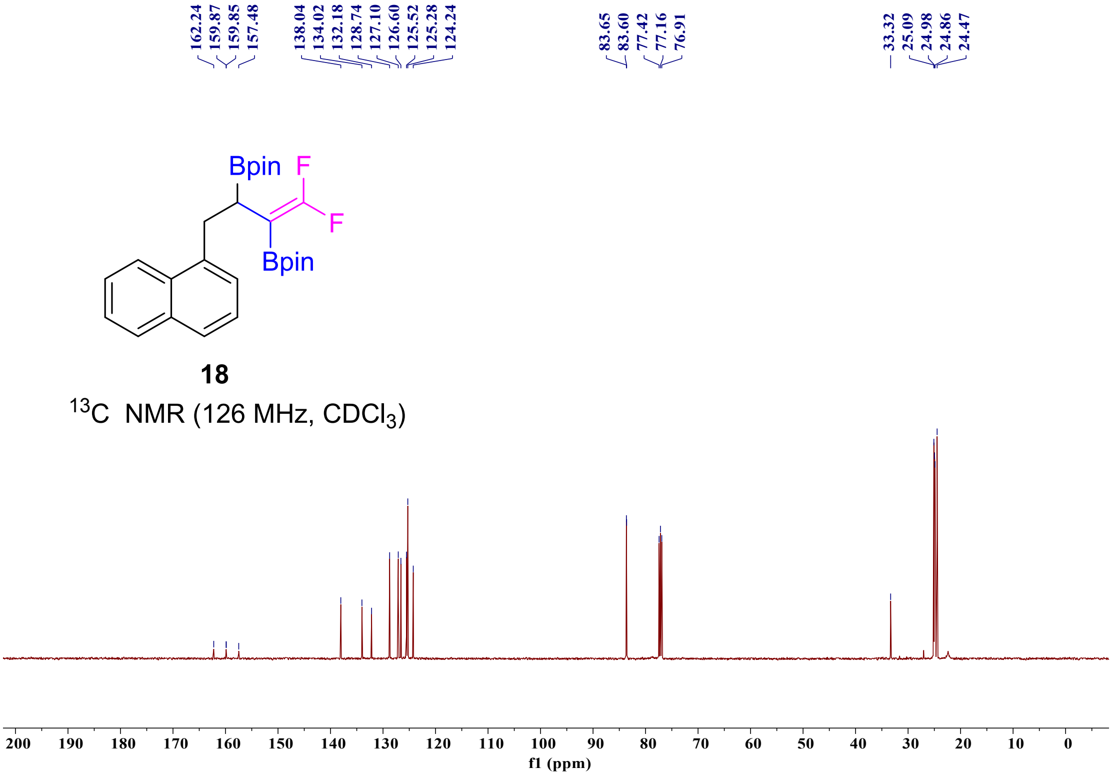

2,2'-(1,1-difluoro-4-(naphthalen-1-yl)but-1-ene-2,3-diyl)bis(4,4,5,5-tetramethyl-1,3,2-dioxaborolane) (18)

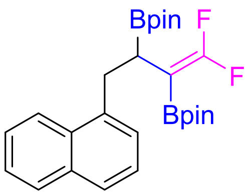

18

^{19}F NMR (470 MHz, $CDCl_3$)

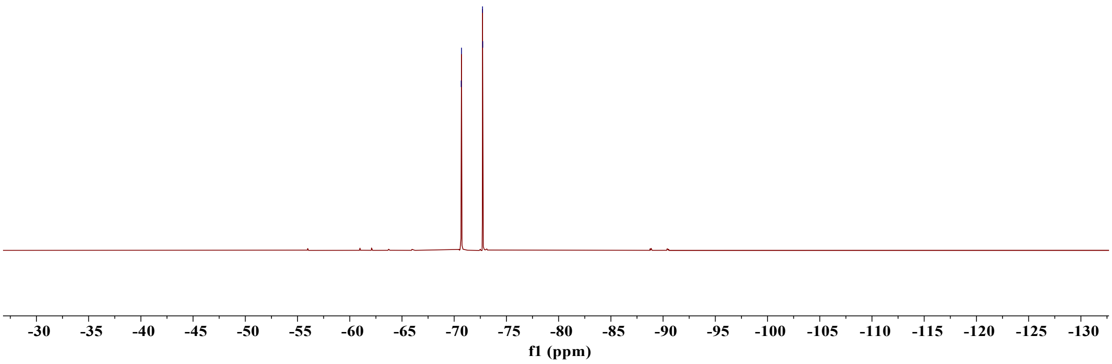

2,2'-(1,1-difluoro-4-(naphthalen-1-yl)but-1-ene-2,3-diyl)bis(4,4,5,5-tetramethyl-1,3,2-dioxaborolane) (18)

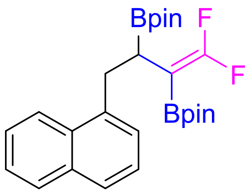

18

^{11}B NMR (128 MHz, $CDCl_3$)

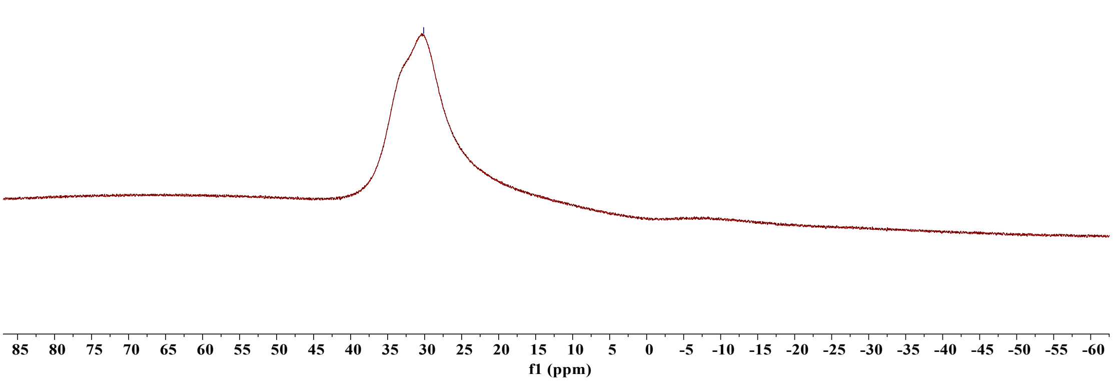

2,2'-(4-([1,1'-biphenyl]-4-yl)-1,1-difluorobut-1-ene-2,3-diyl)bis(4,4,5,5-tetramethyl-1,3,2-dioxaborolane) (19)

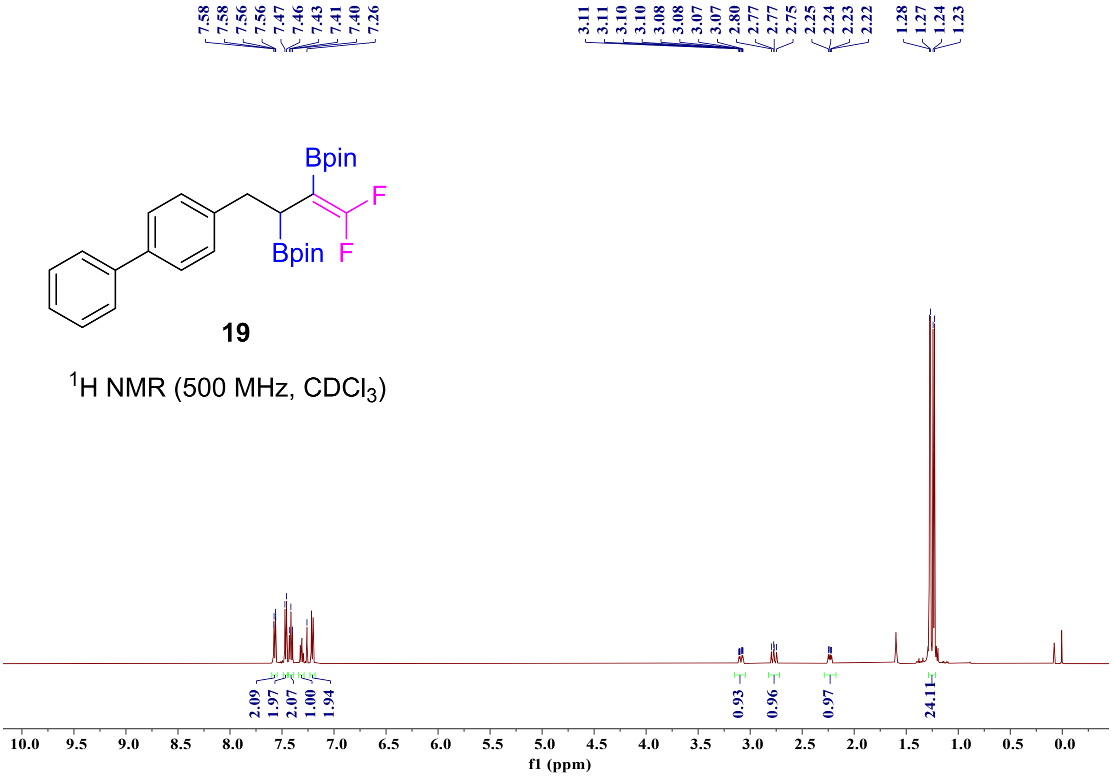

2,2'-(4-([1,1'-biphenyl]-4-yl)-1,1-difluorobut-1-ene-2,3-diyl)bis(4,4,5,5-tetramethyl-1,3,2-dioxaborolane) (19)

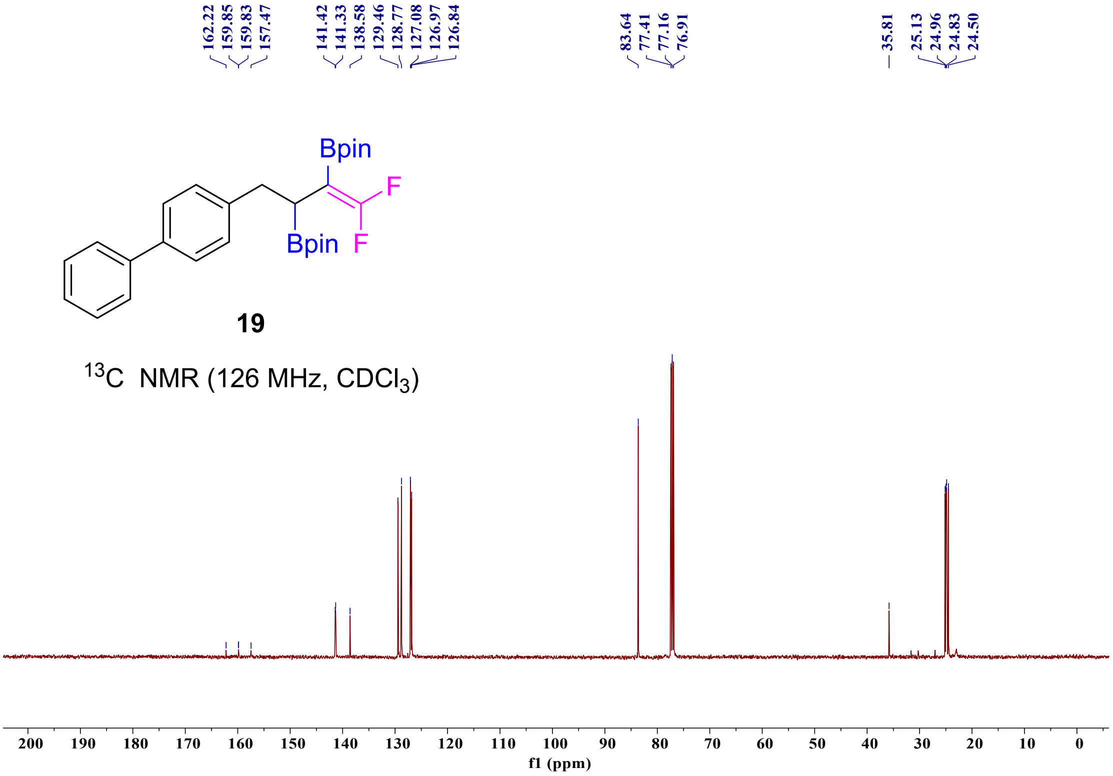

2,2'-(4-([1,1'-biphenyl]-4-yl)-1,1-difluorobut-1-ene-2,3-diyl)bis(4,4,5,5-tetramethyl-1,3,2-dioxaborolane) (19)

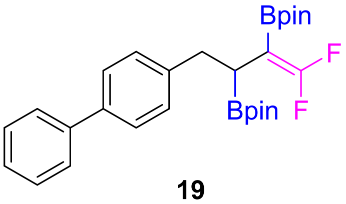

19

^{19}F NMR (470 MHz, $CDCl_3$)

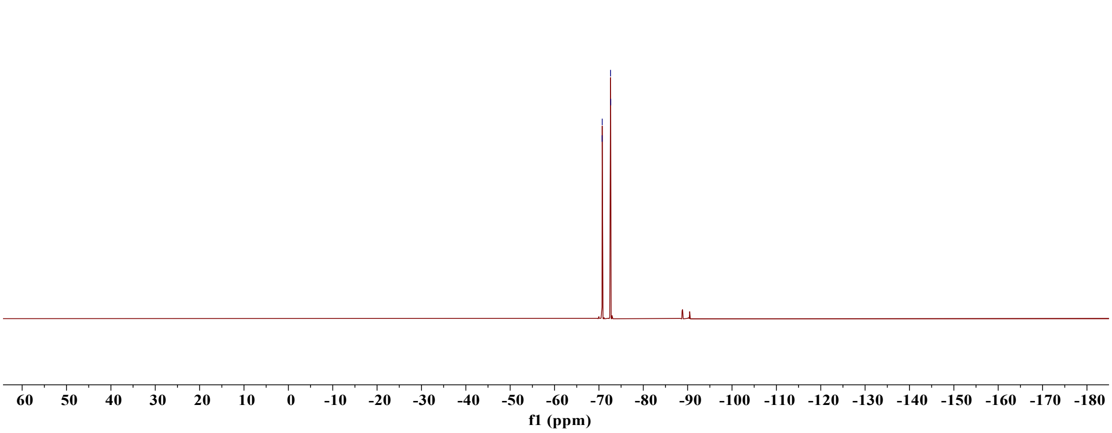

2,2'-(4-([1,1'-biphenyl]-4-yl)-1,1-difluorobut-1-ene-2,3-diyl)bis(4,4,5,5-tetramethyl-1,3,2-dioxaborolane) (19)

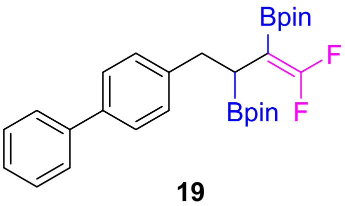

19

^{11}B NMR (128 MHz, $CDCl_3$)

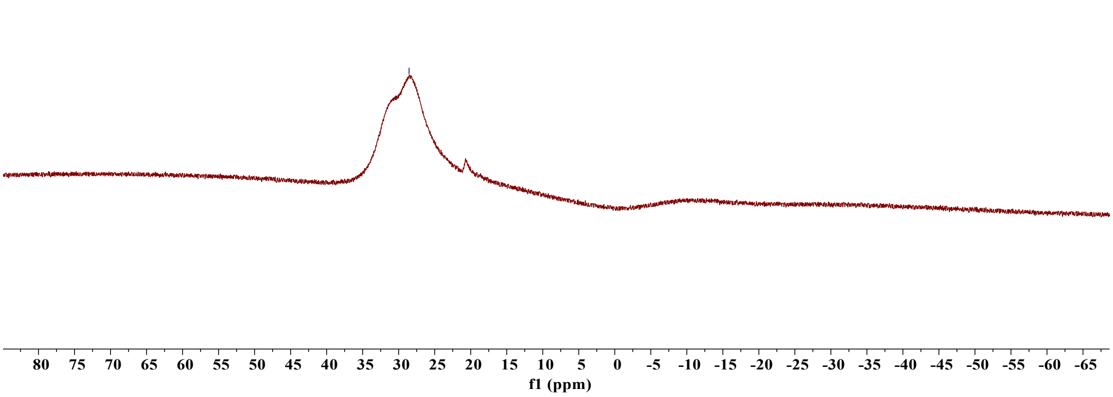

2,2'-(4-(benzo[d][1,3]dioxol-5-yl)-1,1-difluorobut-1-ene-2,3-diyl)bis(4,4,5,5-tetramethyl-1,3,2-dioxaborolane) (20)

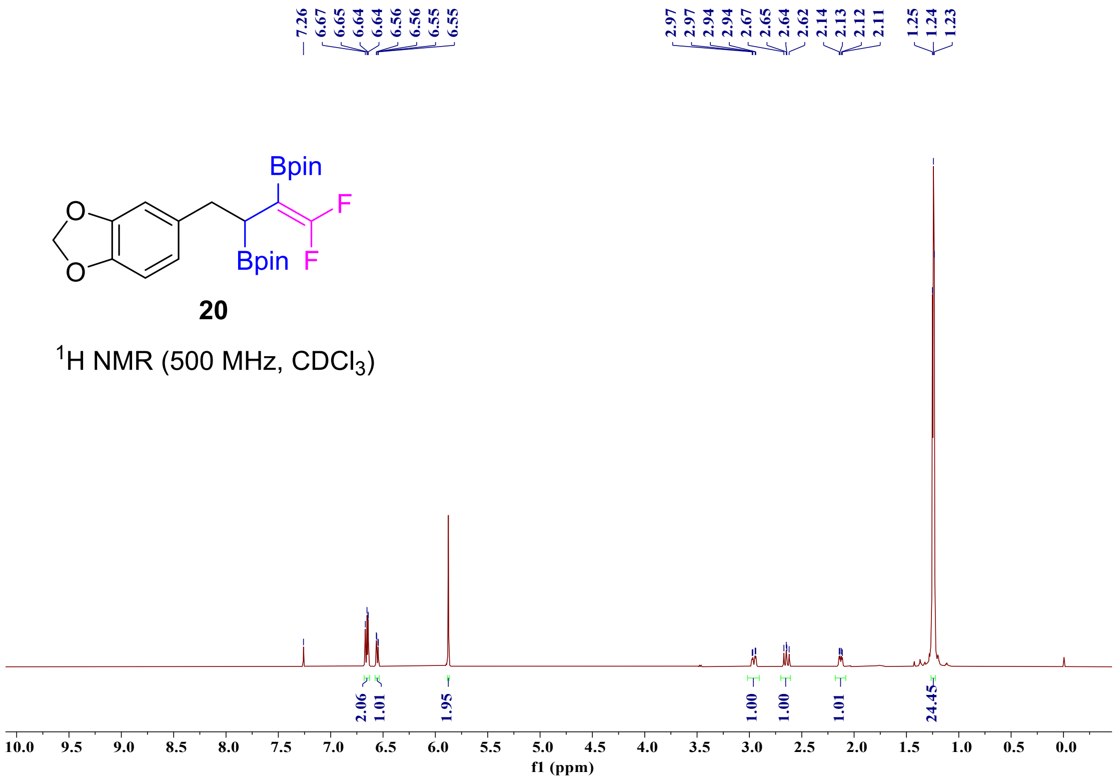

2,2'-(4-(benzo[d][1,3]dioxol-5-yl)-1,1-difluorobut-1-ene-2,3-diyl)bis(4,4,5,5-tetramethyl-1,3,2-dioxaborolane) (20)

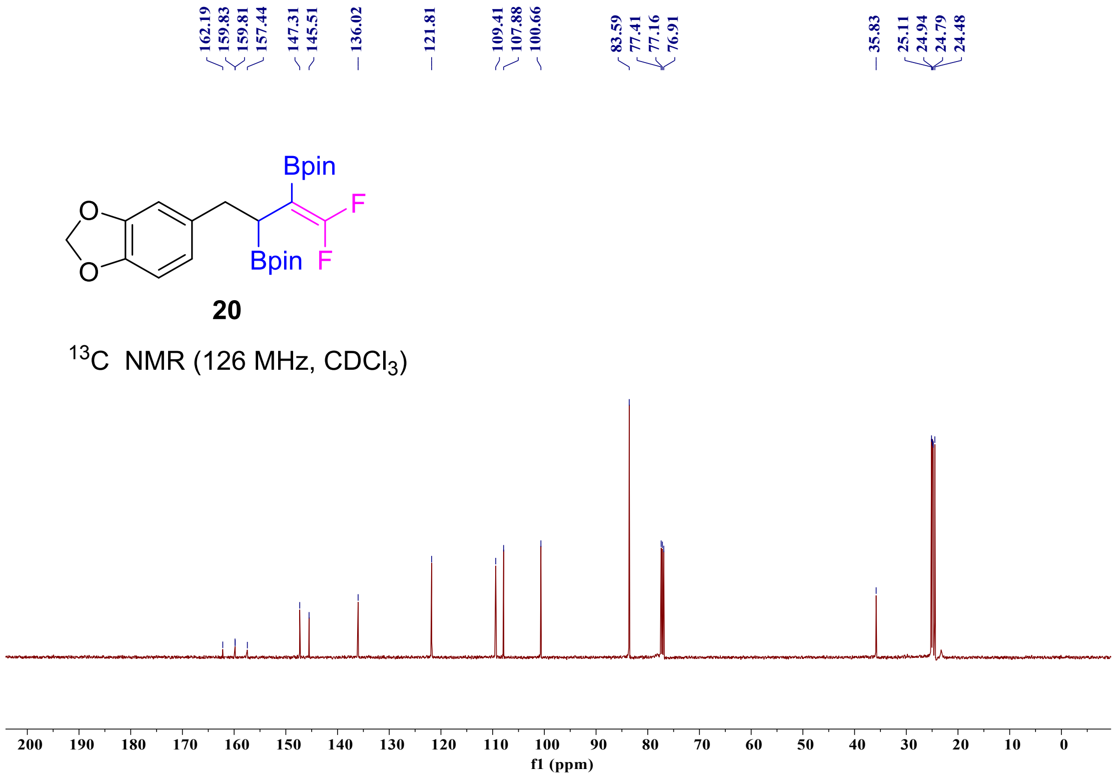

2,2'-(4-(benzo[d][1,3]dioxol-5-yl)-1,1-difluorobut-1-ene-2,3-diyl)bis(4,4,5,5-tetramethyl-1,3,2-dioxaborolane) (20)

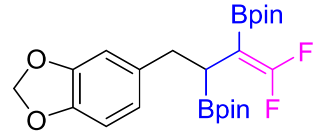

20

^{19}F NMR (470 MHz, $CDCl_3$)

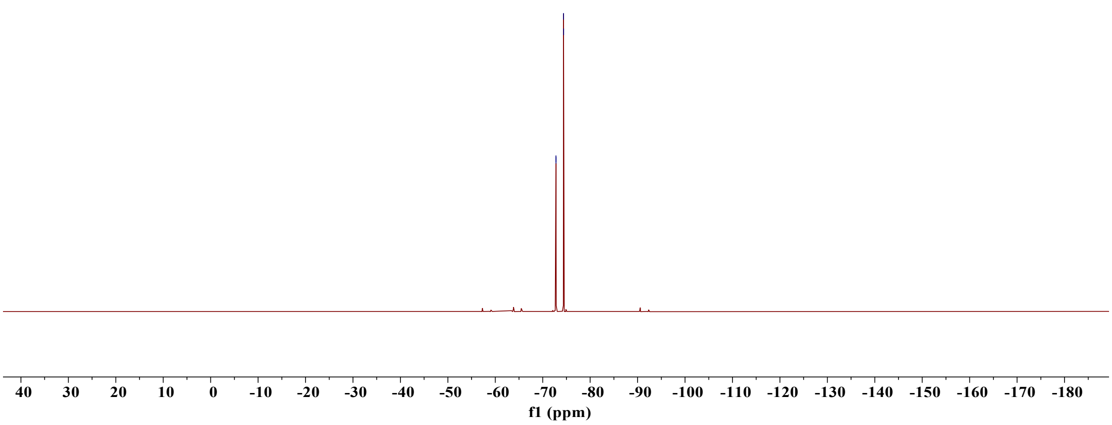

2,2'-(4-(benzo[d][1,3]dioxol-5-yl)-1,1-difluorobut-1-ene-2,3-diyl)bis(4,4,5,5-tetramethyl-1,3,2-dioxaborolane) (20)

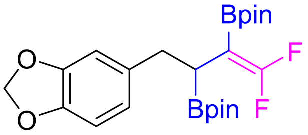

20

^{11}B NMR (128 MHz, $CDCl_3$)

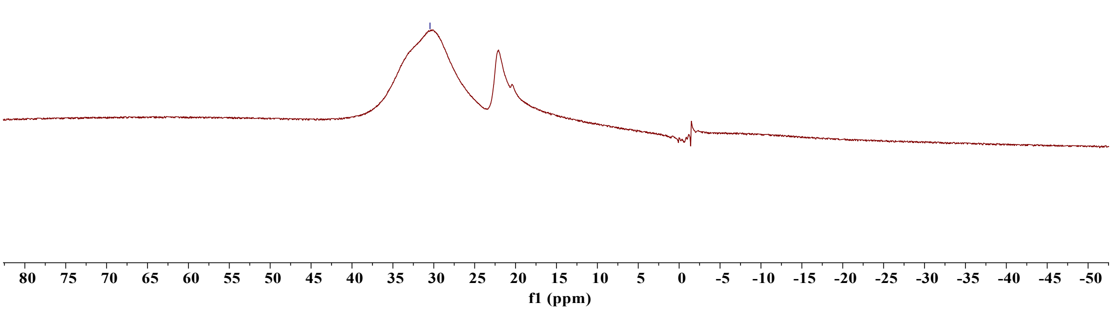

2,2'-(1,1-difluoro-5-phenoxypent-1-ene-2,3-diyl)bis(4,4,5,5-tetramethyl-1,3,2-dioxaborolane) (21)

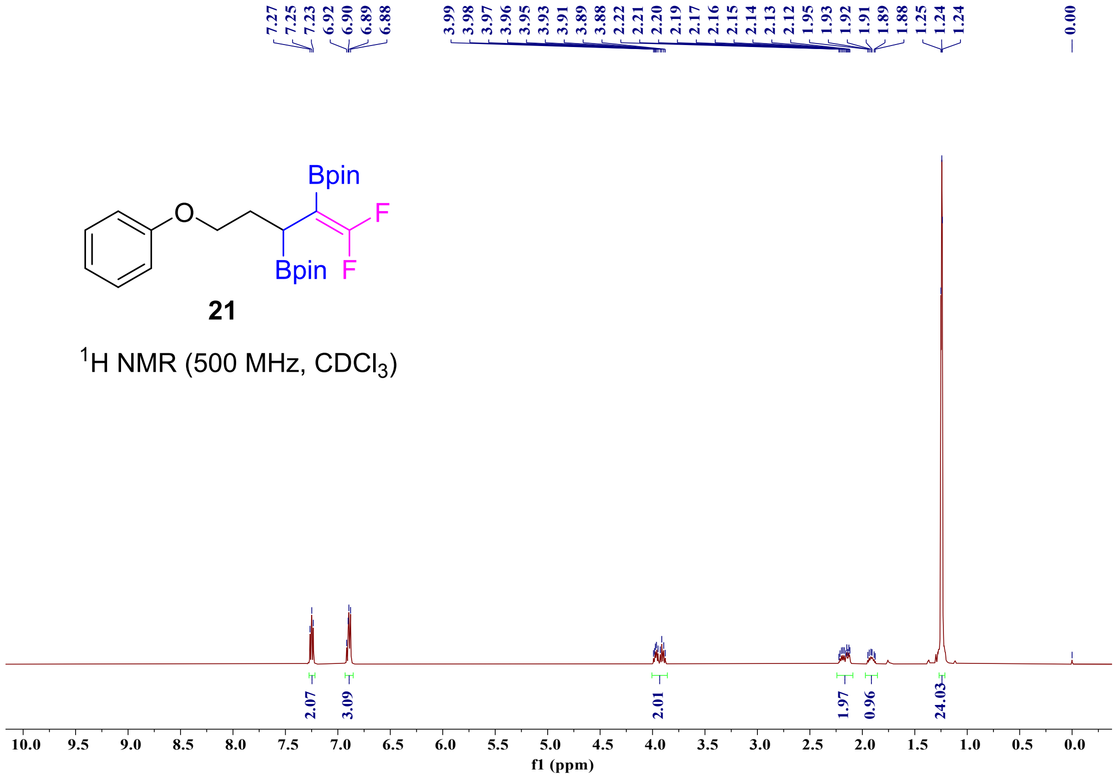

2,2'-(1,1-difluoro-5-phenoxypent-1-ene-2,3-diyl)bis(4,4,5,5-tetramethyl-1,3,2-dioxaborolane) (21)

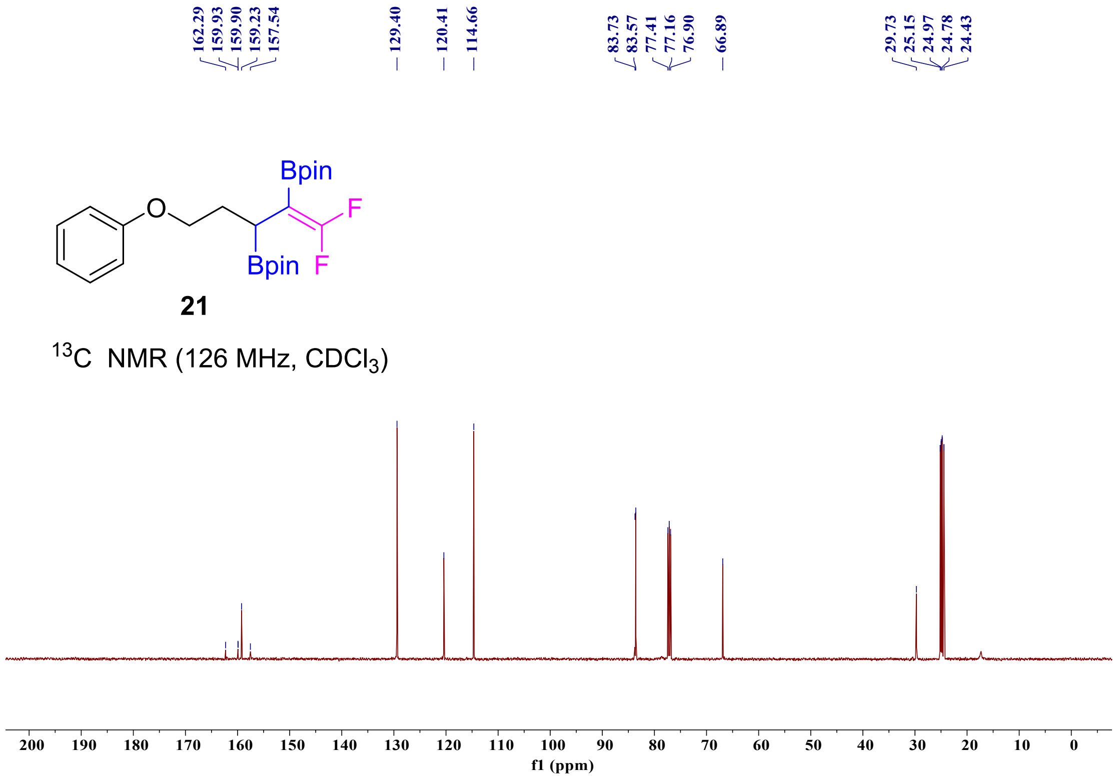

2,2'-(1,1-difluoro-5-phenoxypent-1-ene-2,3-diyl)bis(4,4,5,5-tetramethyl-1,3,2-dioxaborolane) (21)

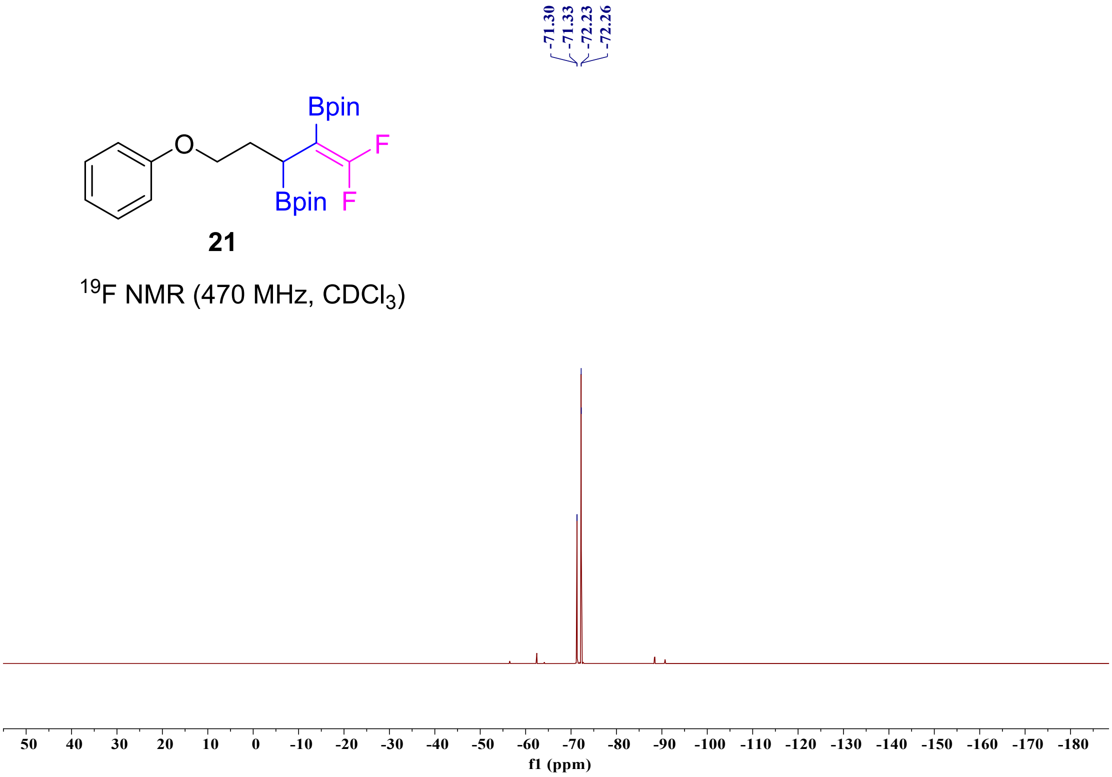

2,2'-(1,1-difluoro-5-phenoxypent-1-ene-2,3-diyl)bis(4,4,5,5-tetramethyl-1,3,2-dioxaborolane) (21)

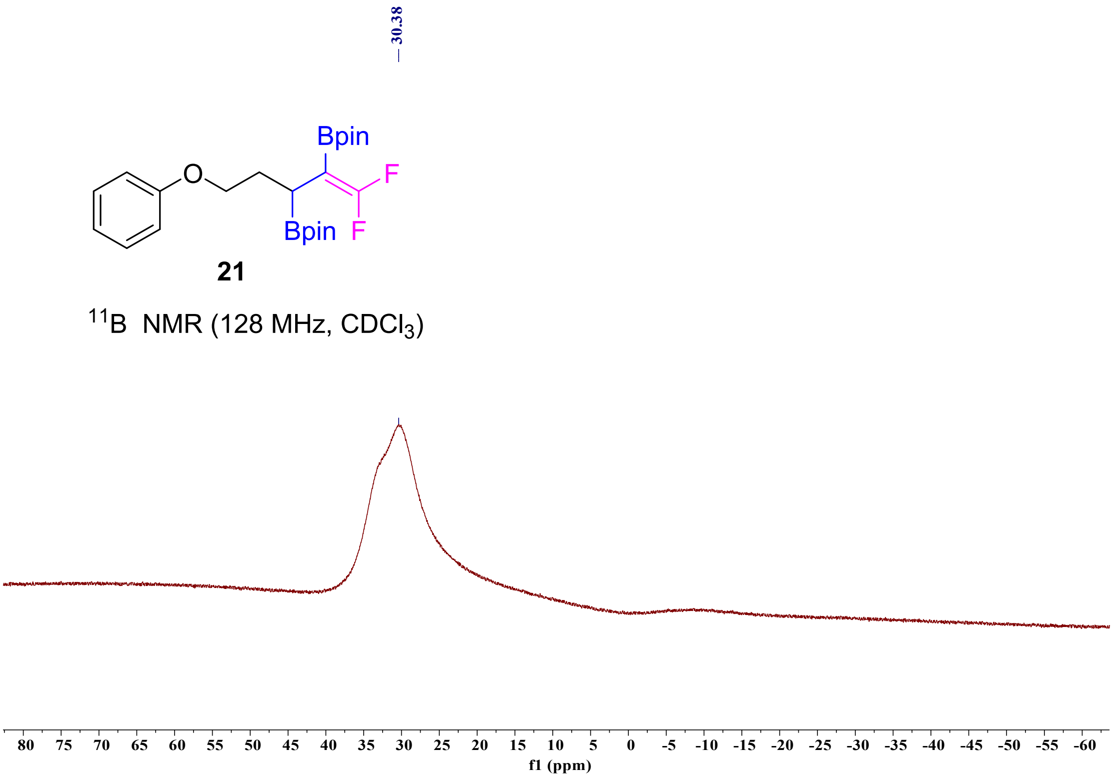

2,2'-(5-(3,4-dimethoxyphenyl)-1,1-difluoropent-1-ene-2,3-diyl)bis(4,4,5,5-tetramethyl-1,3,2-dioxaborolane) (22)

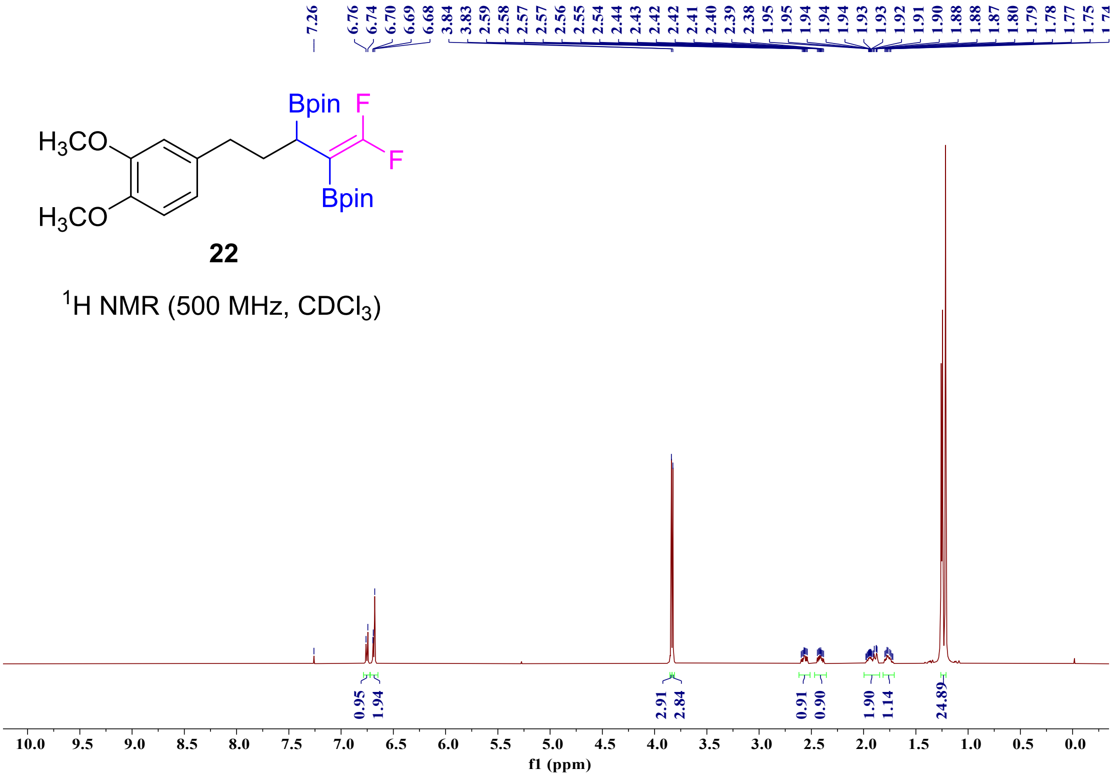

2,2'-(5-(3,4-dimethoxyphenyl)-1,1-difluoropent-1-ene-2,3-diyl)bis(4,4,5,5-tetramethyl-1,3,2-dioxaborolane) (22)

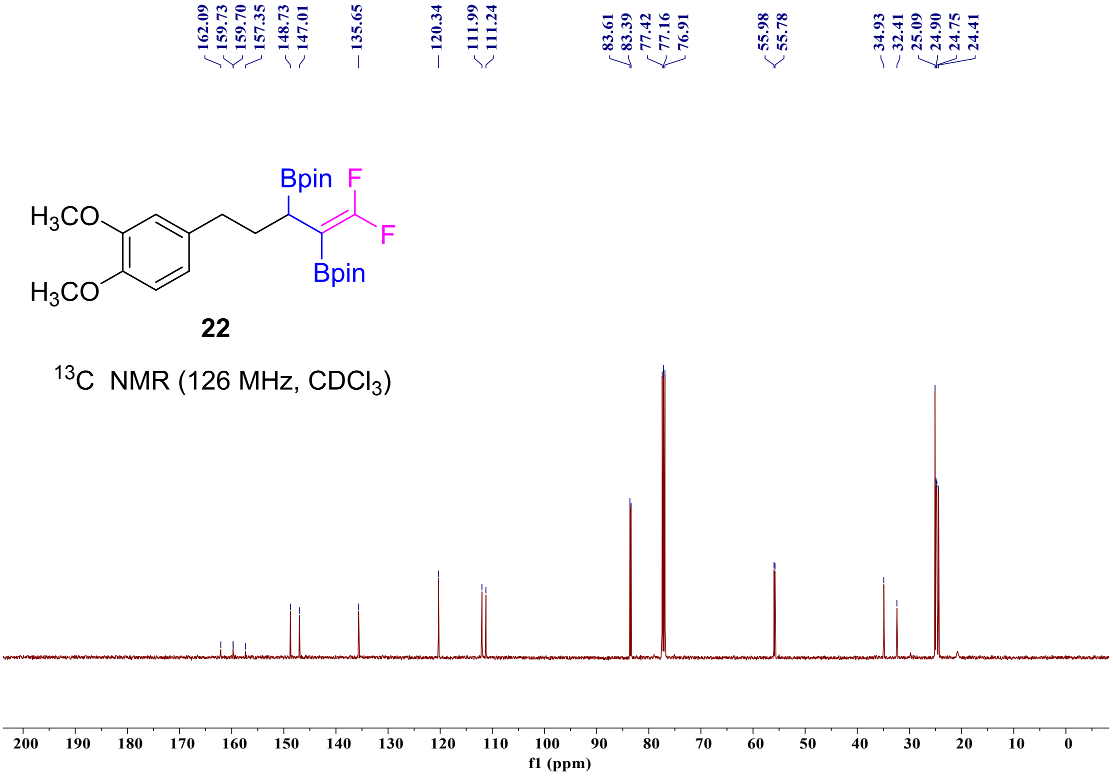

2,2'-(5-(3,4-dimethoxyphenyl)-1,1-difluoropent-1-ene-2,3-diyl)bis(4,4,5,5-tetramethyl-1,3,2-dioxaborolane) (22)

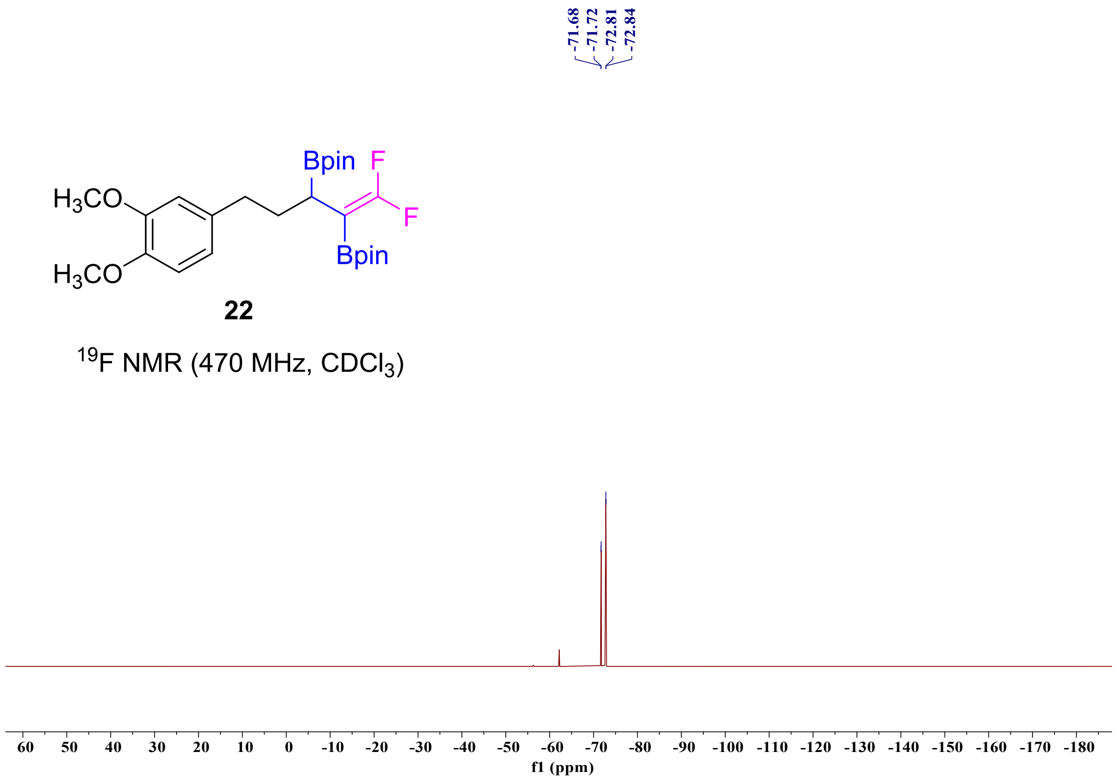

2,2'-(5-(3,4-dimethoxyphenyl)-1,1-difluoropent-1-ene-2,3-diyl)bis(4,4,5,5-tetramethyl-1,3,2-dioxaborolane) (22)

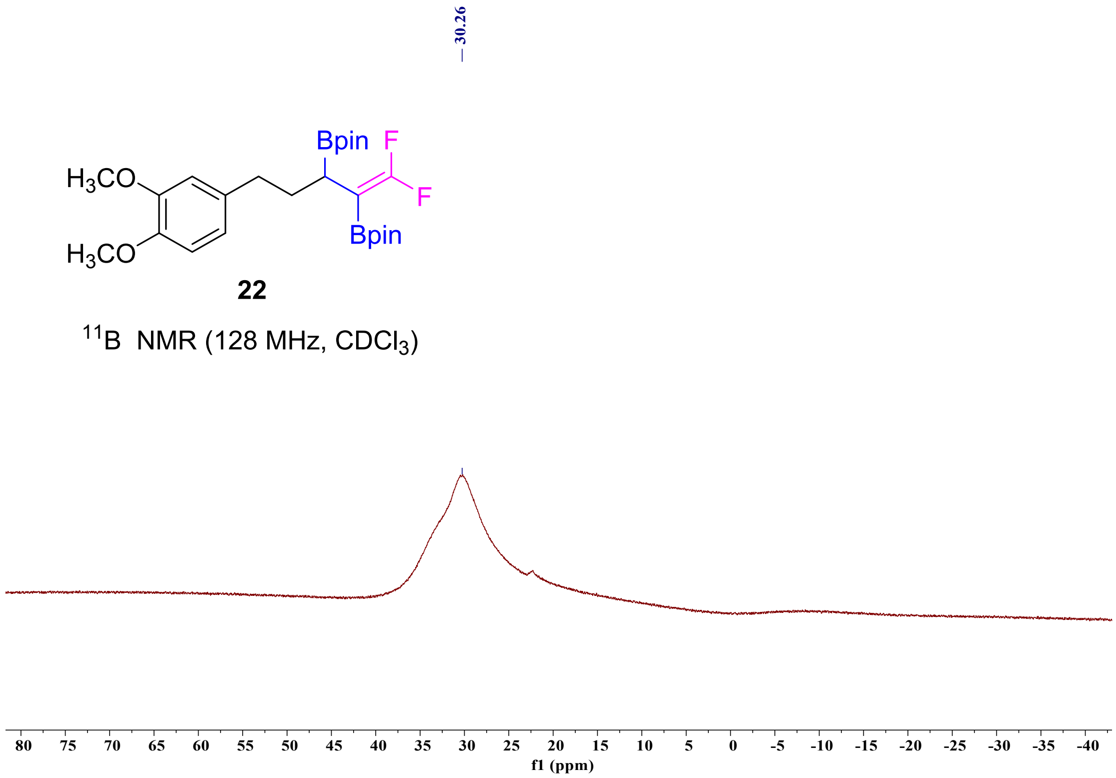

2,2'-(1,1-difluoro-5-(5-methylfuran-2-yl)pent-1-ene-2,3-diyl)bis(4,4,5,5-tetramethyl-1,3,2-dioxaborolane) (23)

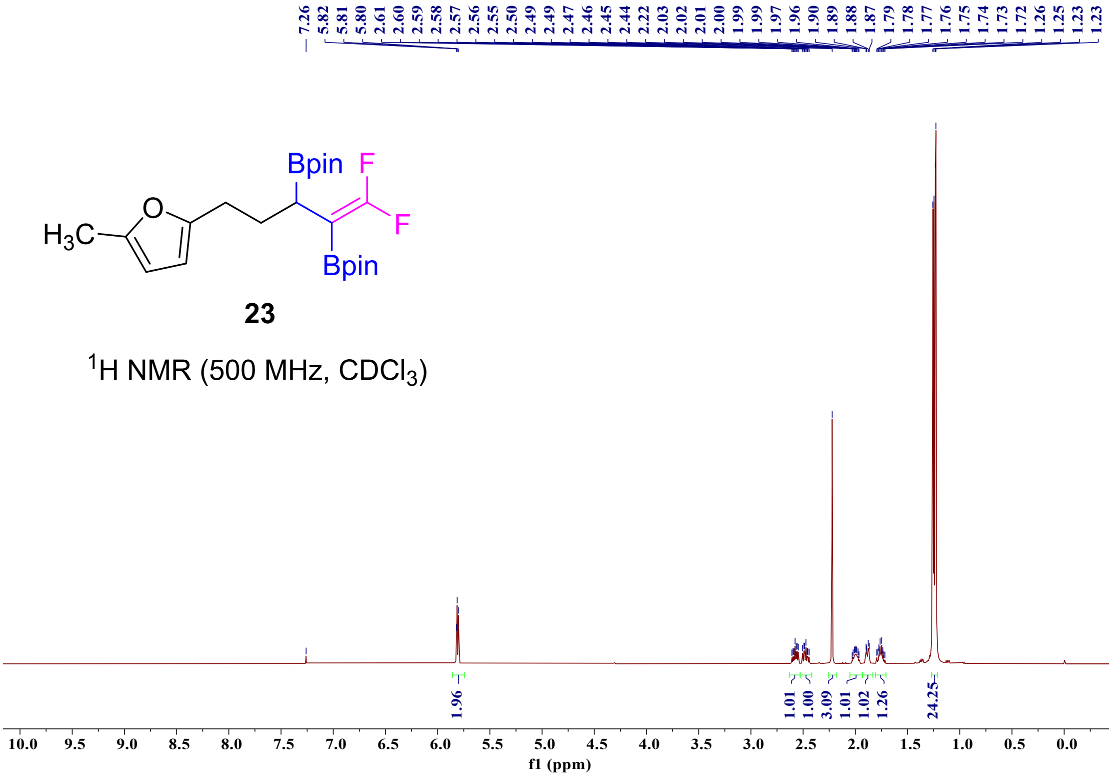

2,2'-(1,1-difluoro-5-(5-methylfuran-2-yl)pent-1-ene-2,3-diyl)bis(4,4,5,5-tetramethyl-1,3,2-dioxaborolane) (23)

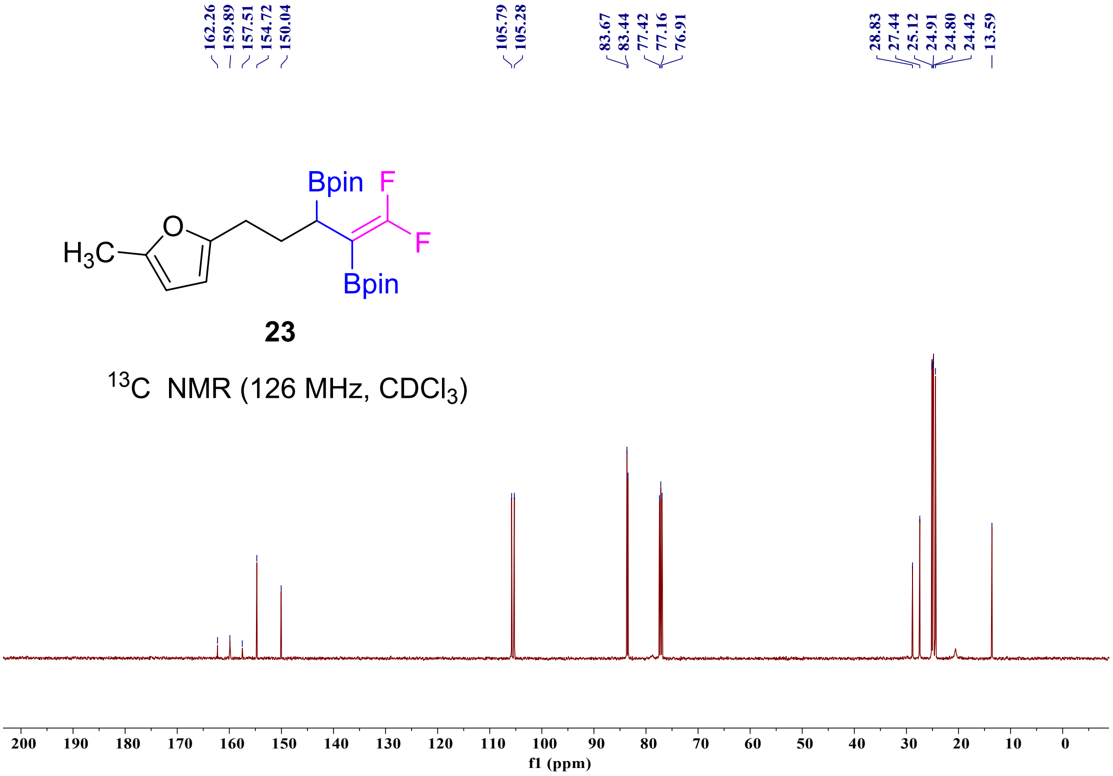

2,2'-(1,1-difluoro-5-(5-methylfuran-2-yl)pent-1-ene-2,3-diyl)bis(4,4,5,5-tetramethyl-1,3,2-dioxaborolane) (23)

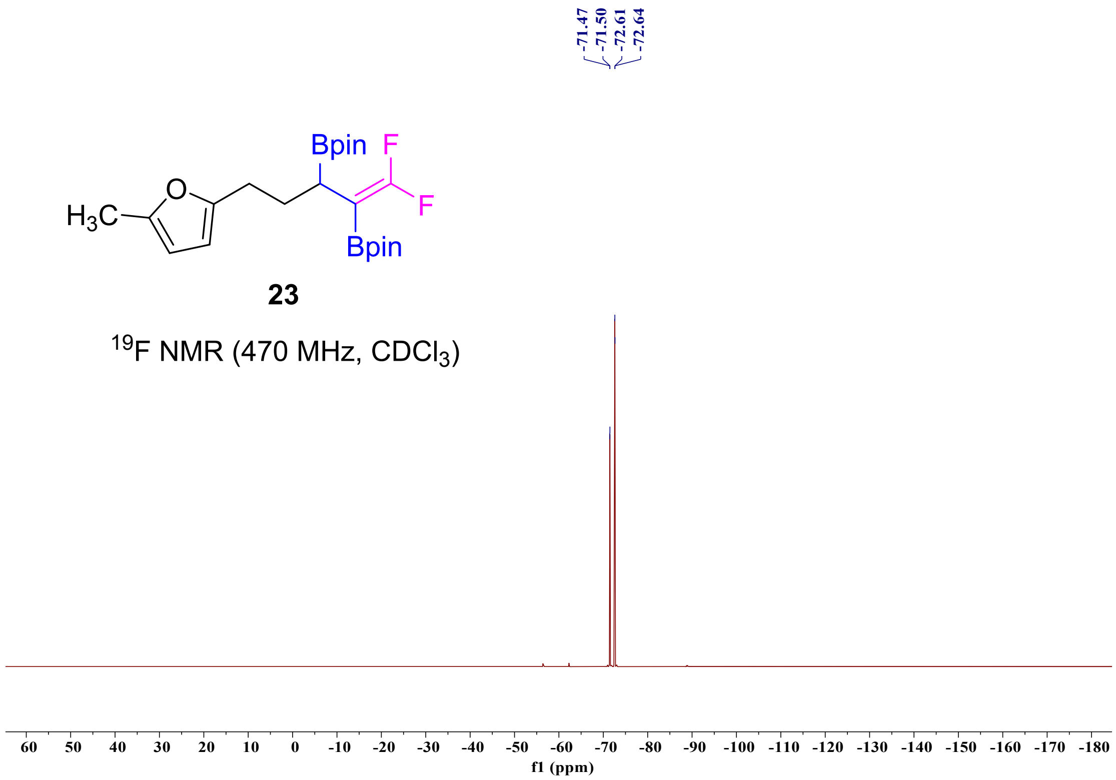

2,2'-(1,1-difluoro-5-(5-methylfuran-2-yl)pent-1-ene-2,3-diyl)bis(4,4,5,5-tetramethyl-1,3,2-dioxaborolane) (23)

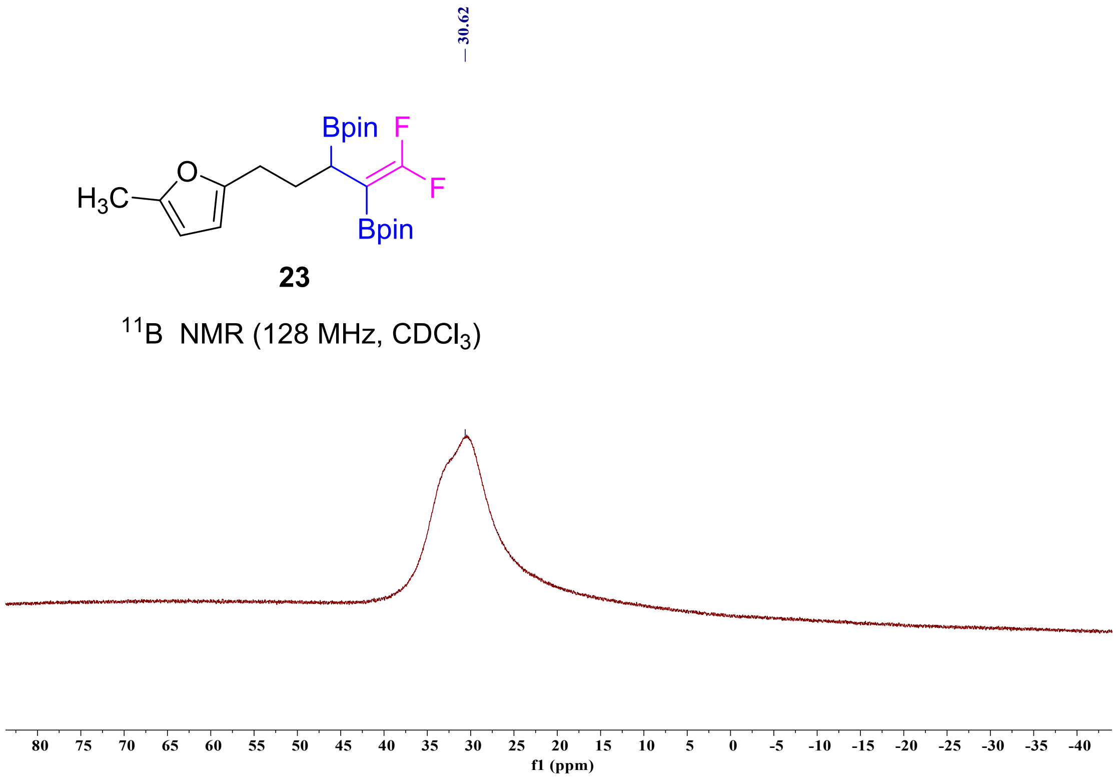

2,2'-(1,1-difluoro-6-(thiophen-2-yl)hex-1-ene-2,3-diyl)bis(4,4,5,5-tetramethyl-1,3,2-dioxaborolane) (24)

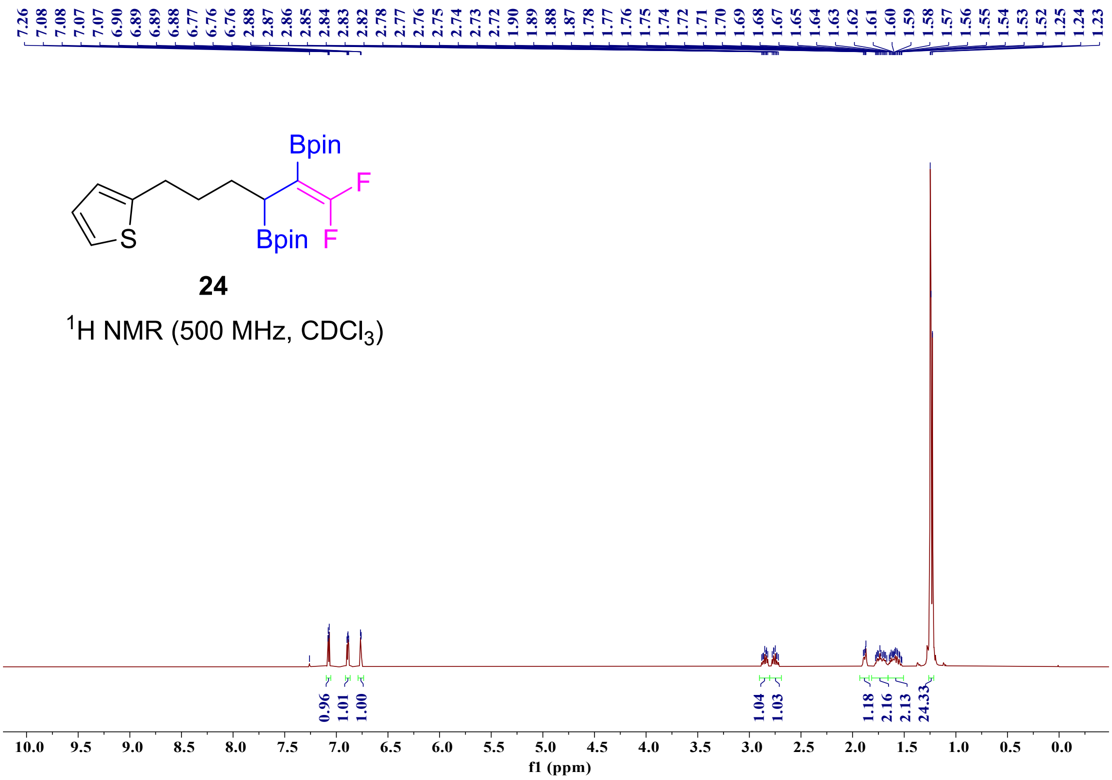

2,2'-(1,1-difluoro-6-(thiophen-2-yl)hex-1-ene-2,3-diyl)bis(4,4,5,5-tetramethyl-1,3,2-dioxaborolane) (24)

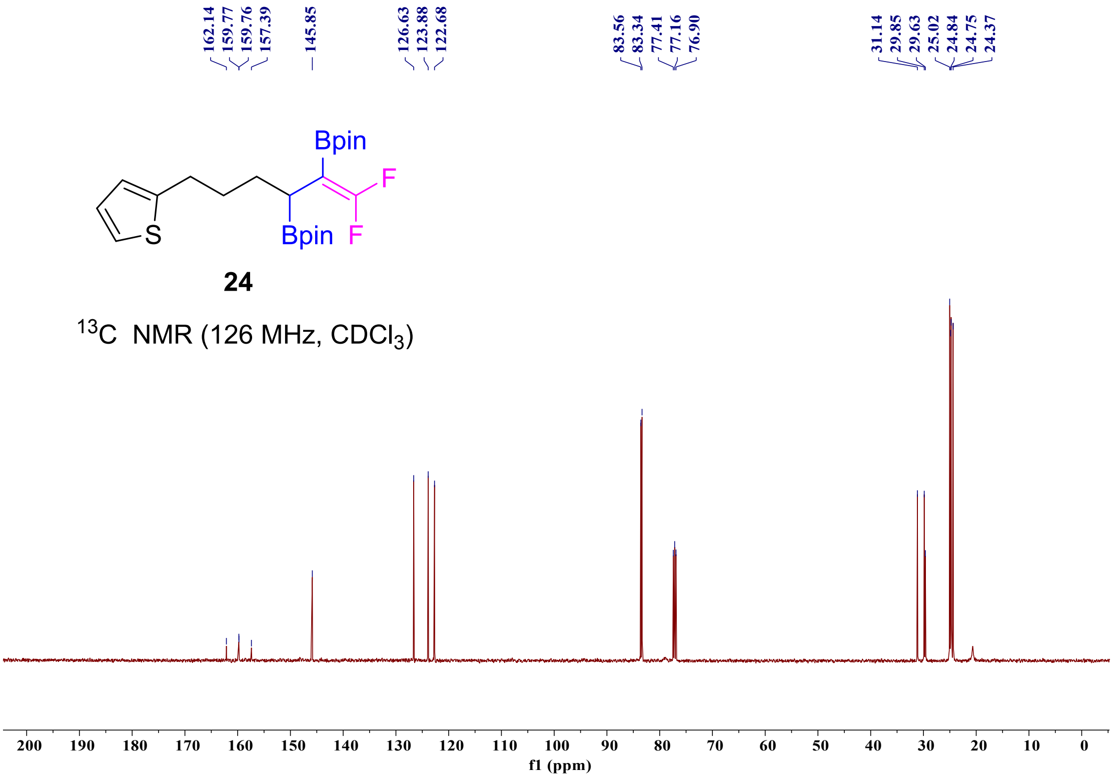

2,2'-(1,1-difluoro-6-(thiophen-2-yl)hex-1-ene-2,3-diyl)bis(4,4,5,5-tetramethyl-1,3,2-dioxaborolane) (24)

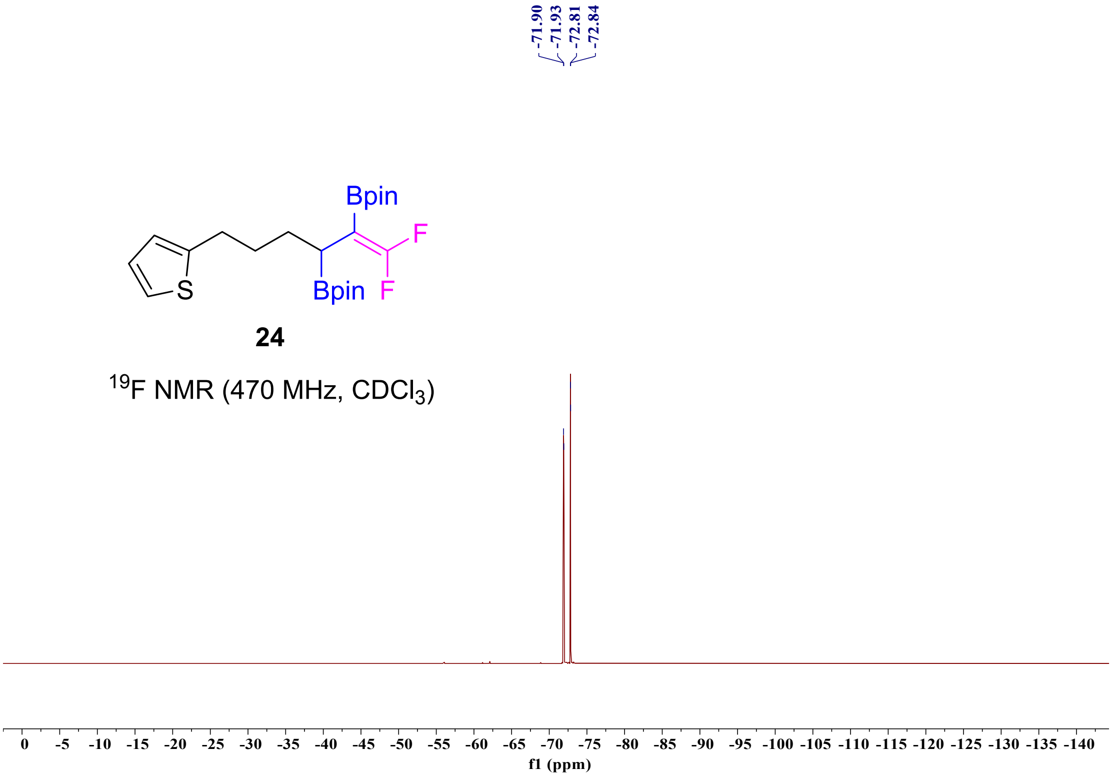

2,2'-(1,1-difluoro-6-(thiophen-2-yl)hex-1-ene-2,3-diyl)bis(4,4,5,5-tetramethyl-1,3,2-dioxaborolane) (24)

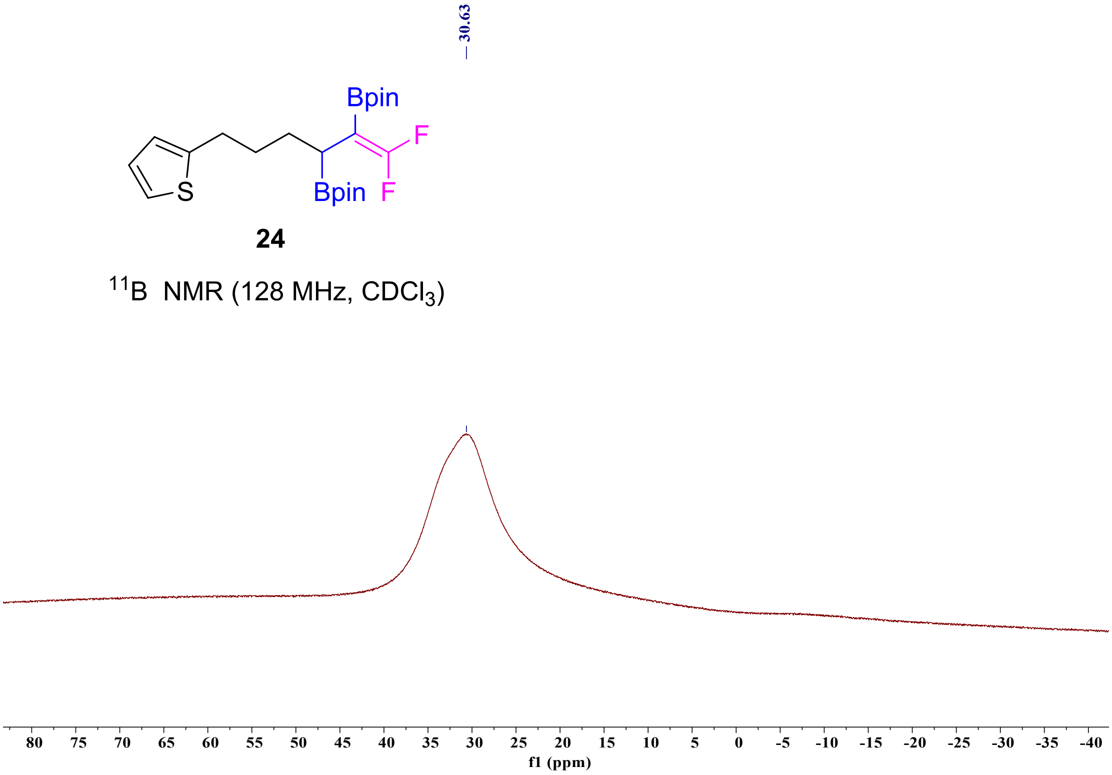

tert-butyl4-(3,3-difluoro-1,2-bis(4,4,5,5-tetramethyl-1,3,2-dioxaborolan-2-yl)allyl)piperidine-1-carboxylate(25)

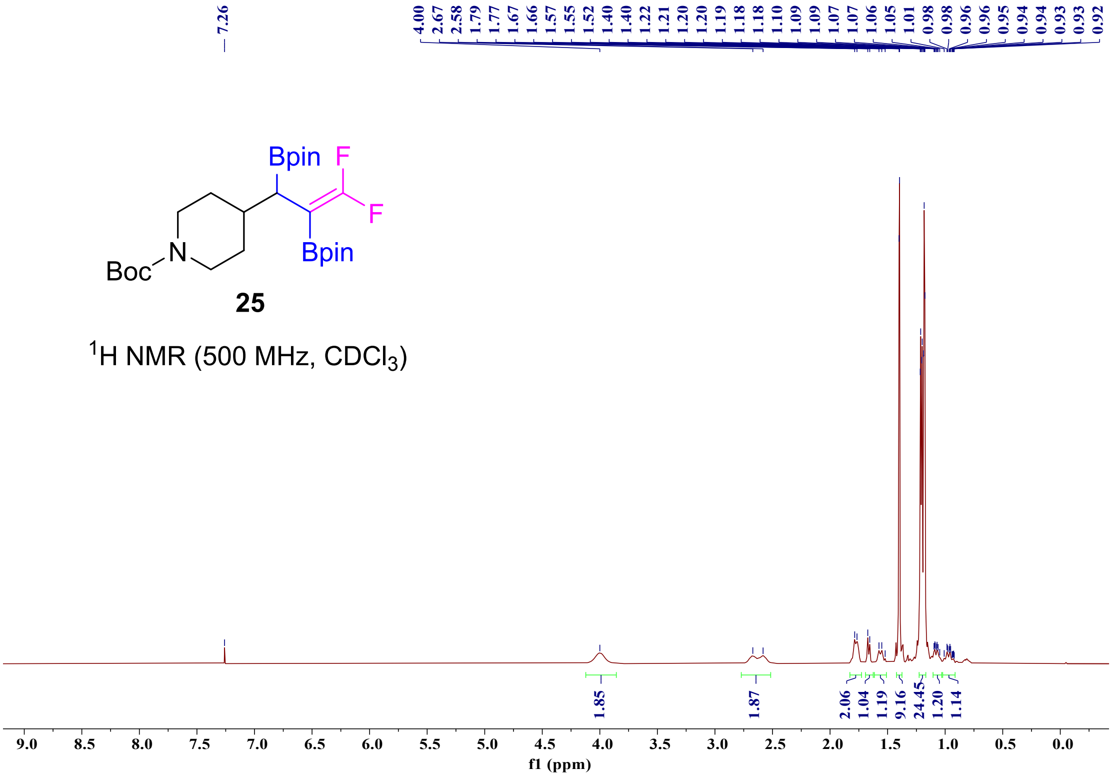

tert-butyl4-(3,3-difluoro-1,2-bis(4,4,5,5-tetramethyl-1,3,2-dioxaborolan-2-yl)allyl)piperidine-1-carboxylate(25)

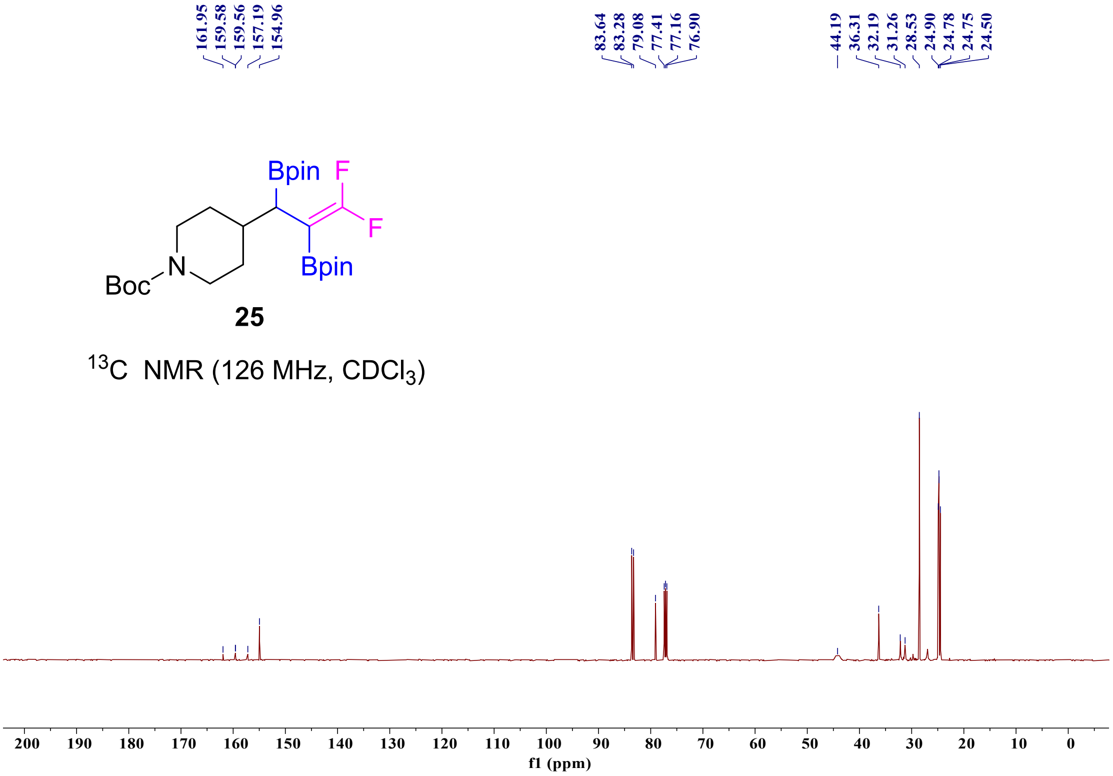

tert-butyl4-(3,3-difluoro-1,2-bis(4,4,5,5-tetramethyl-1,3,2-dioxaborolan-2-yl)allyl)piperidine-1-carboxylate(25)

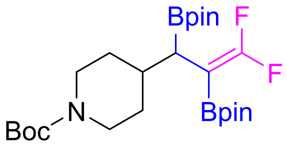

25

^{19}F NMR (470 MHz, $CDCl_3$)

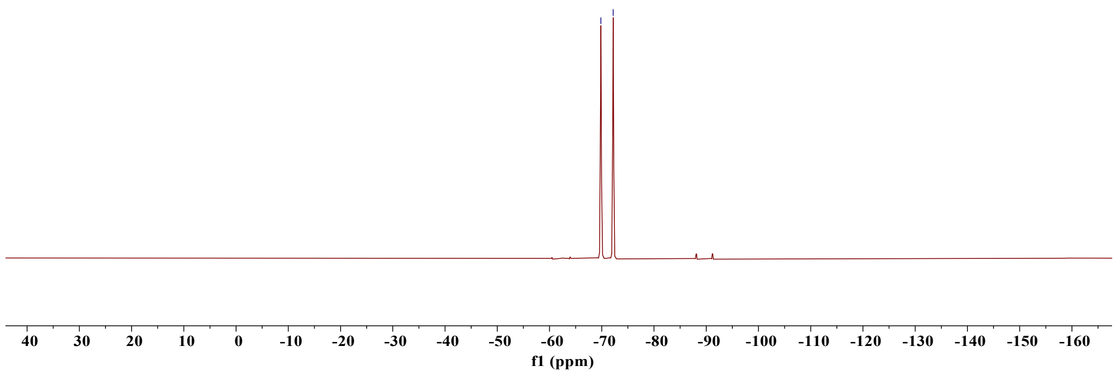

tert-butyl4-(3,3-difluoro-1,2-bis(4,4,5,5-tetramethyl-1,3,2-dioxaborolan-2-yl)allyl)piperidine-1-carboxylate(25)

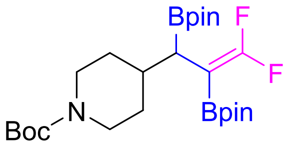

25

^{11}B NMR (128 MHz, $CDCl_3$)

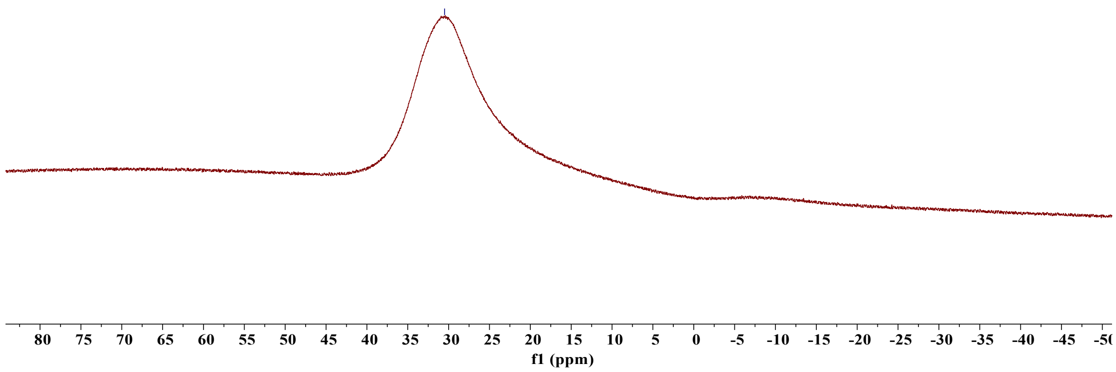

benzyl4-(3,3-difluoro-1,2-bis(4,4,5,5-tetramethyl-1,3,2-dioxaborolan-2-yl)allyl)piperidine-1-carboxylate(26)

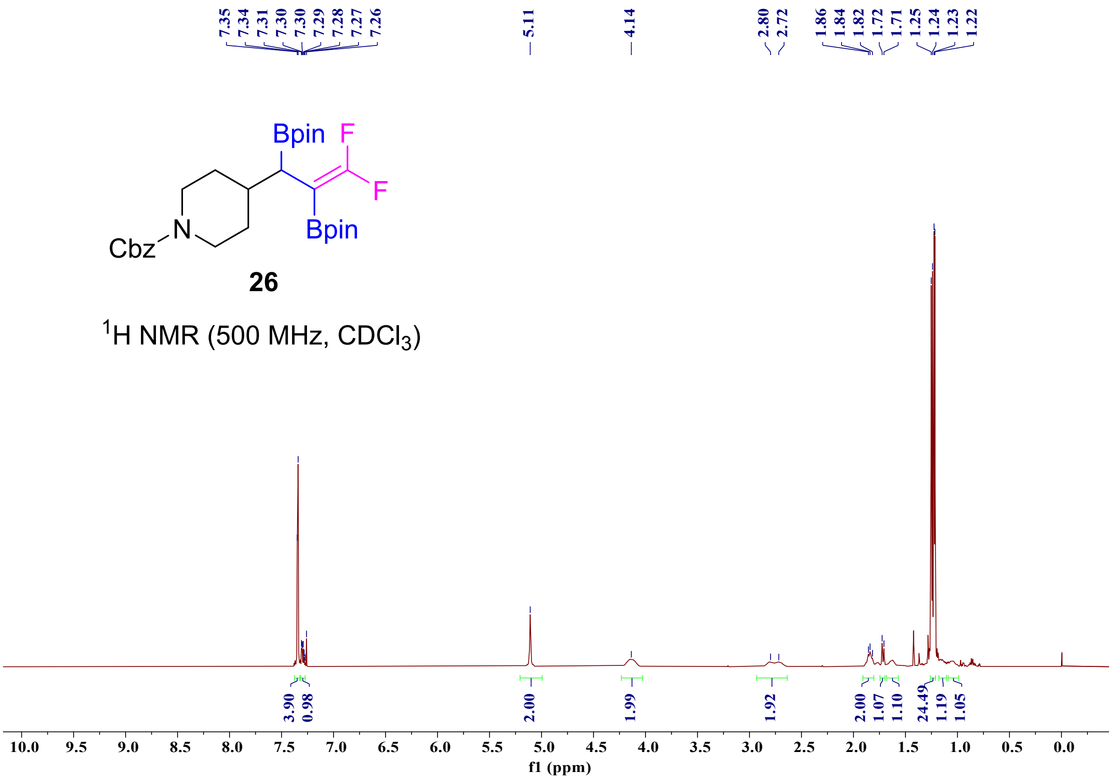

benzyl4-(3,3-difluoro-1,2-bis(4,4,5,5-tetramethyl-1,3,2-dioxaborolan-2-yl)allyl)piperidine-1-carboxylate(26)

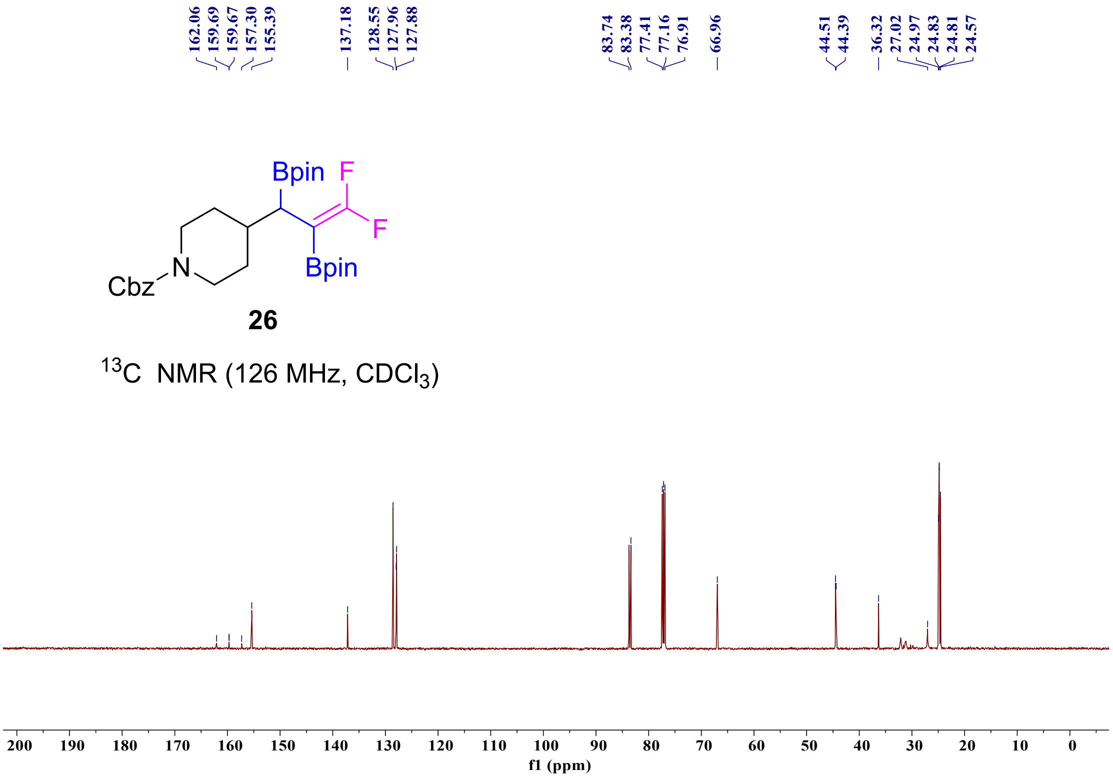

benzyl4-(3,3-difluoro-1,2-bis(4,4,5,5-tetramethyl-1,3,2-dioxaborolan-2-yl)allyl)piperidine-1-carboxylate(26)

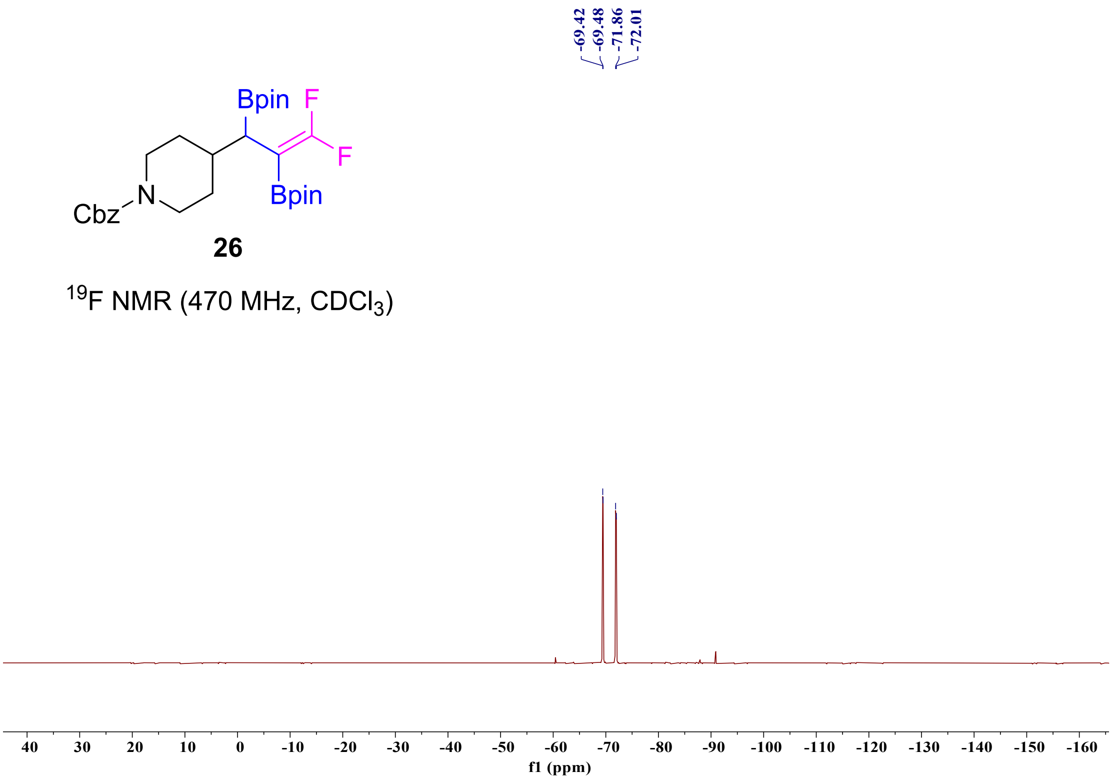

benzyl4-(3,3-difluoro-1,2-bis(4,4,5,5-tetramethyl-1,3,2-dioxaborolan-2-yl)allyl)piperidine-1-carboxylate(26)

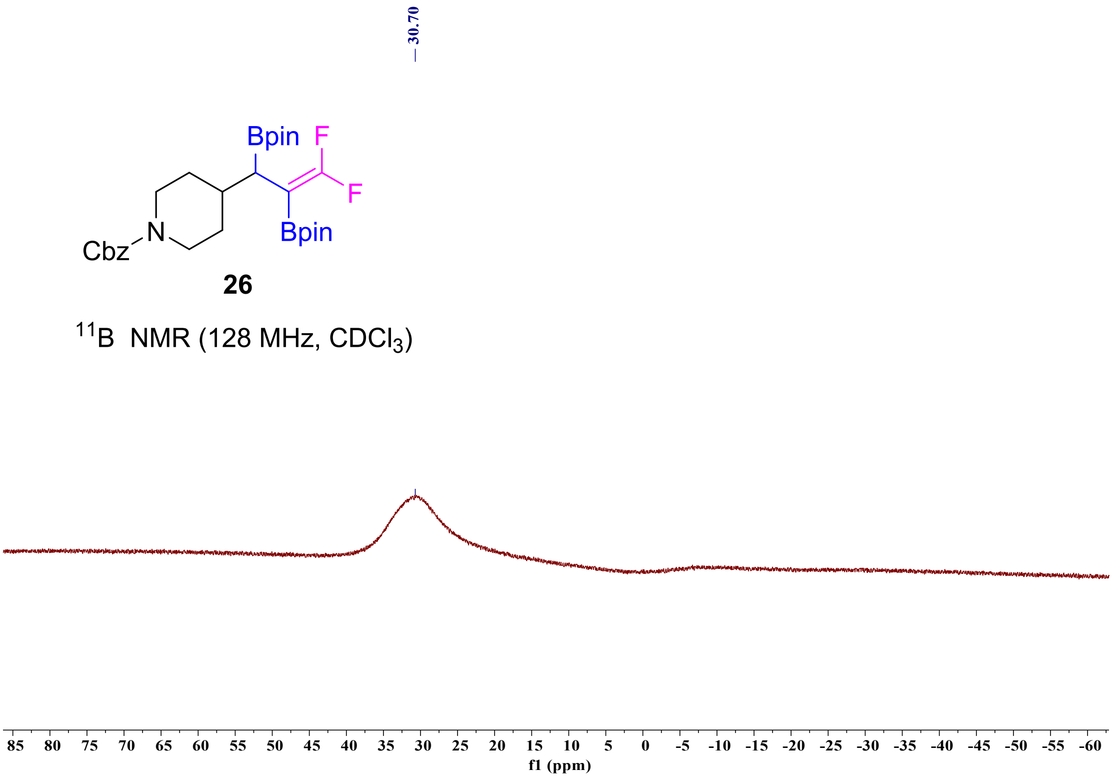

4-(4,4-difluoro-2,3-bis(4,4,5,5-tetramethyl-1,3,2-dioxaborolan-2-yl)but-3-en-1-yl)-1-tosylpiperidine(27)

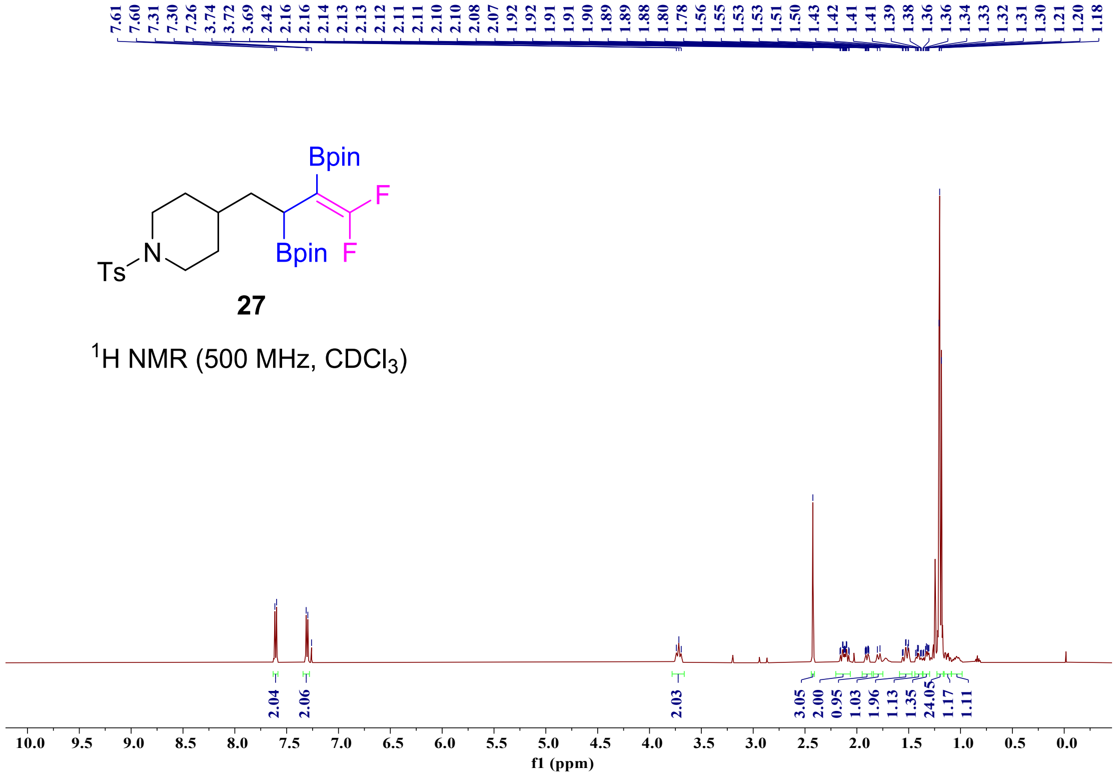

4-(4,4-difluoro-2,3-bis(4,4,5,5-tetramethyl-1,3,2-dioxaborolan-2-yl)but-3-en-1-yl)-1-tosylpiperidine(27)

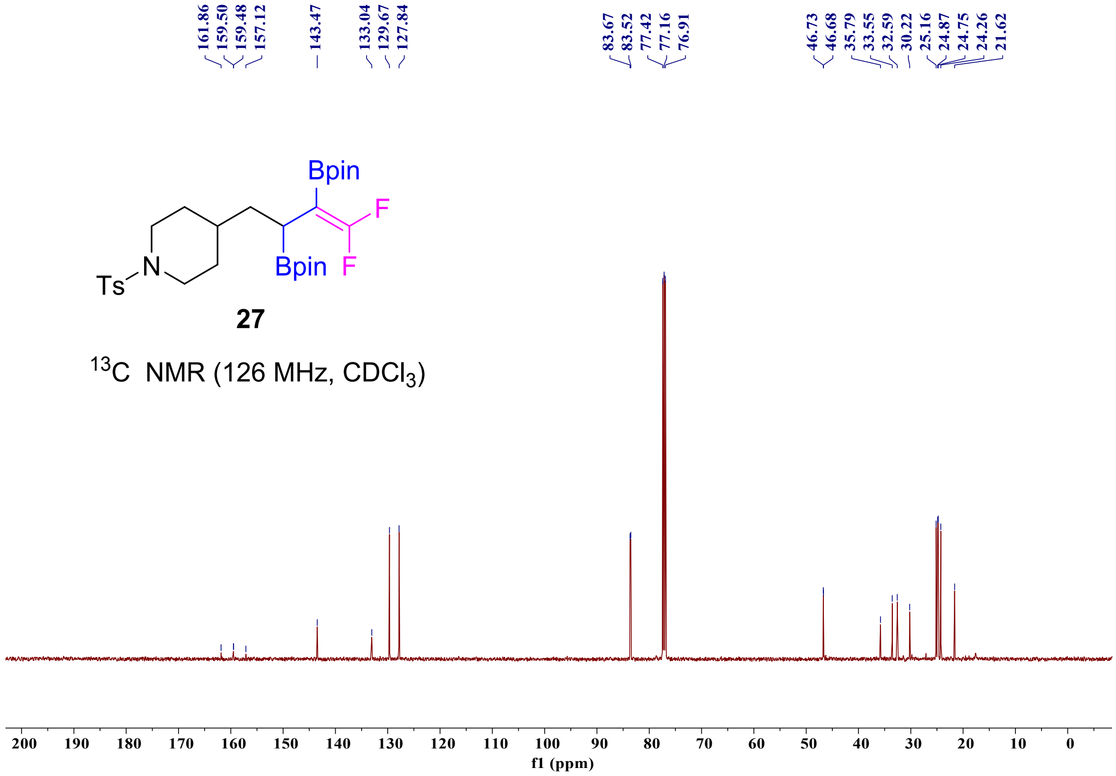

4-(4,4-difluoro-2,3-bis(4,4,5,5-tetramethyl-1,3,2-dioxaborolan-2-yl)but-3-en-1-yl)-1-tosylpiperidine(27)

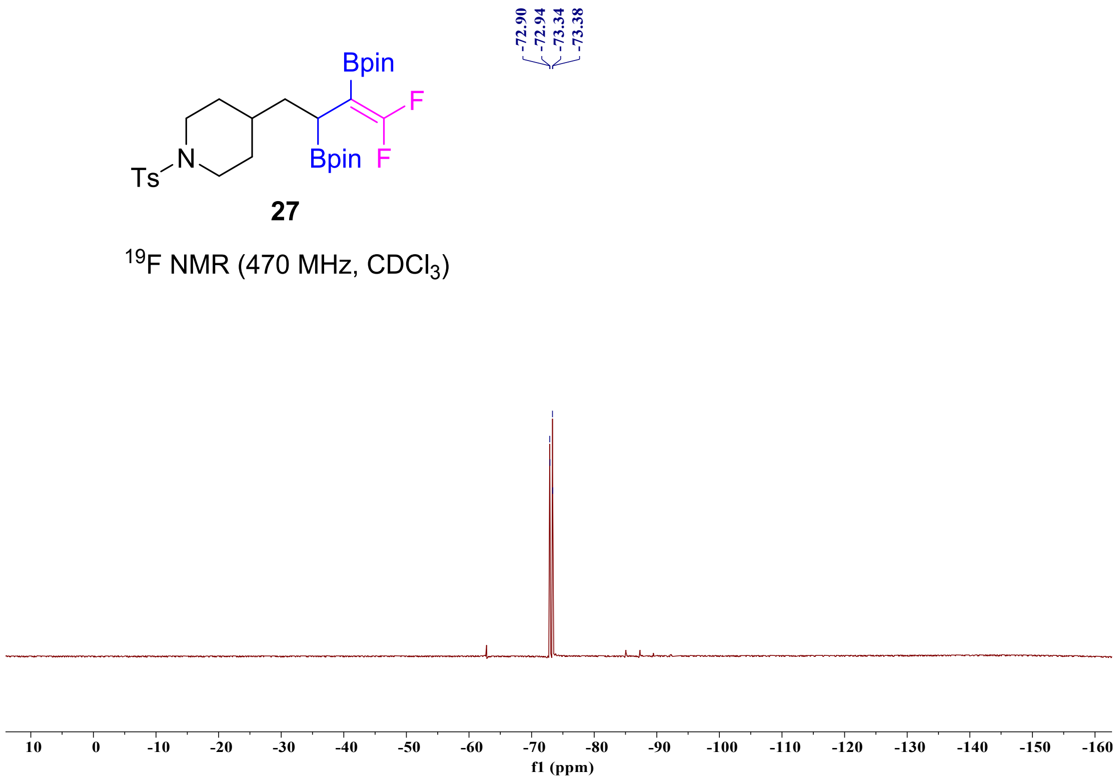

27

^{19}F NMR (470 MHz, $CDCl_3$)

4-(4,4-difluoro-2,3-bis(4,4,5,5-tetramethyl-1,3,2-dioxaborolan-2-yl)but-3-en-1-yl)-1-tosylpiperidine(27)

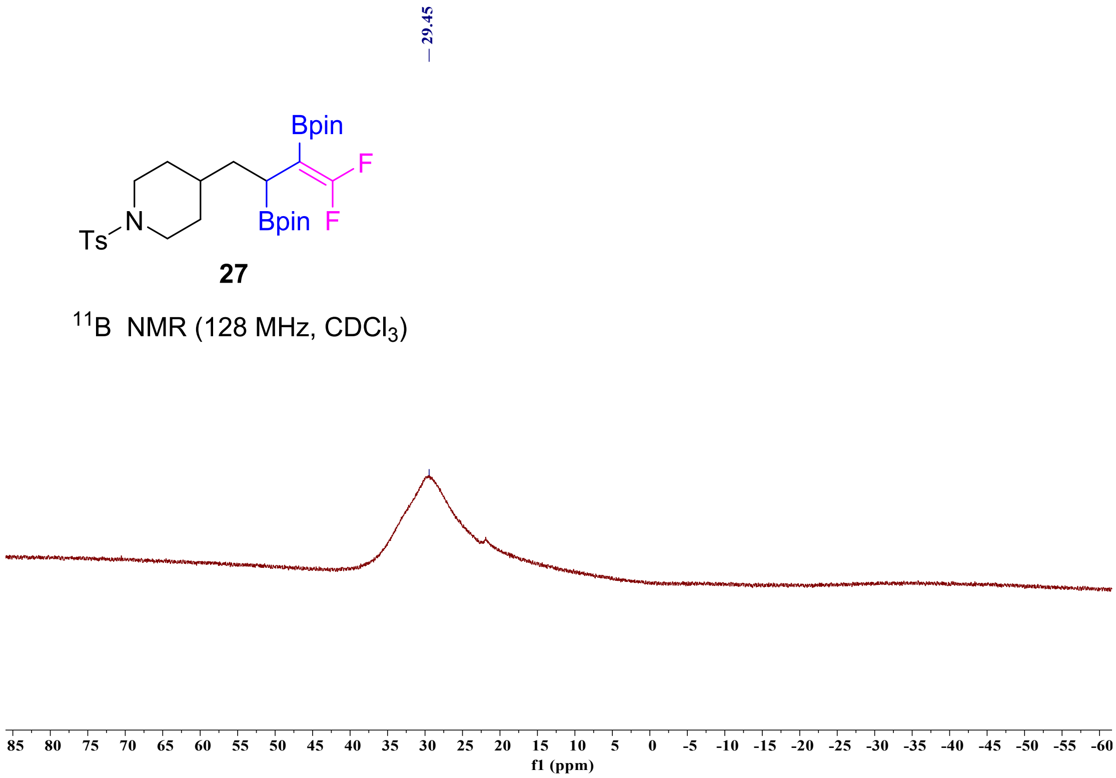

27

^{11}B NMR (128 MHz, $CDCl_3$)

2,2'-(1,1-difluoro-4-(tetrahydro-2H-pyran-4-yl)but-1-ene-2,3-diyl)bis(4,4,5,5-tetramethyl-1,3,2-dioxaborolane) (28)

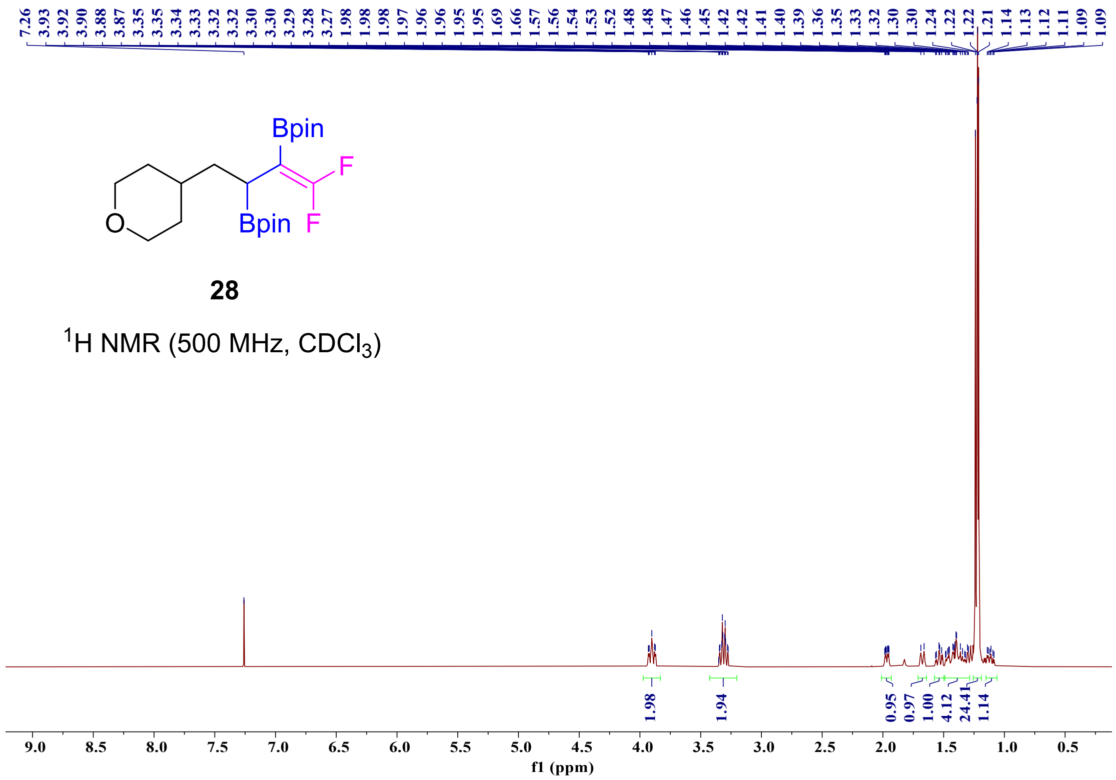

2,2'-(1,1-difluoro-4-(tetrahydro-2H-pyran-4-yl)but-1-ene-2,3-diyl)bis(4,4,5,5-tetramethyl-1,3,2-dioxaborolane) (28)

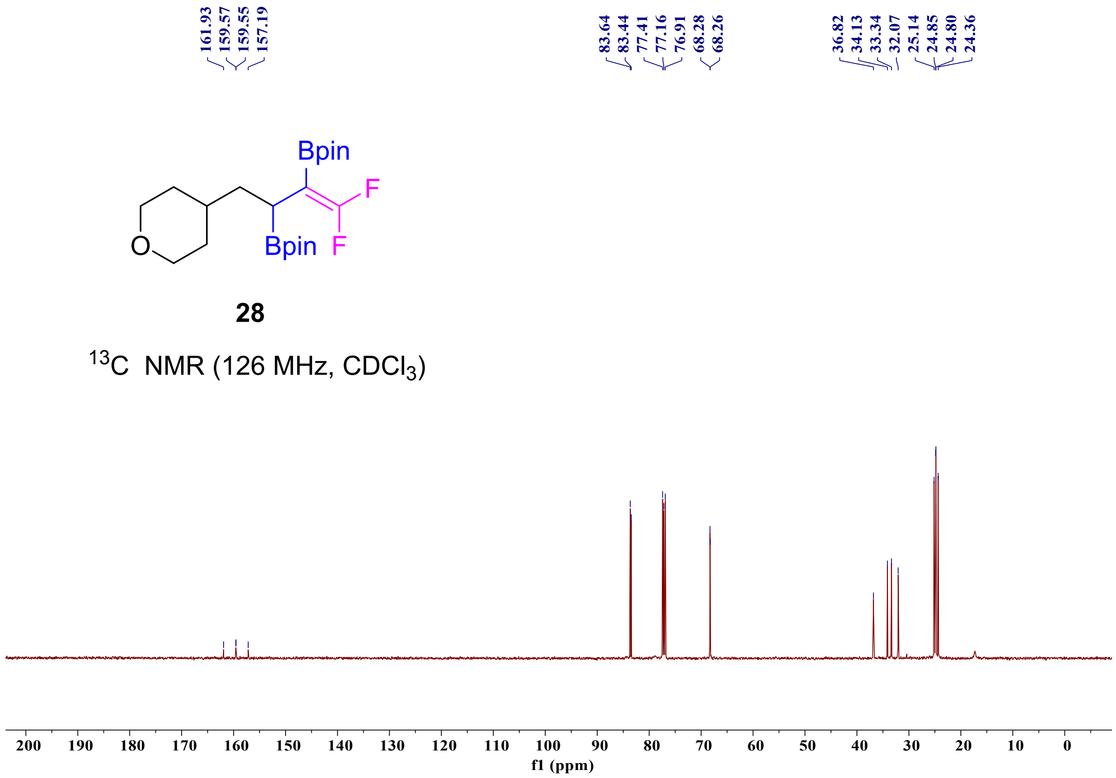

2,2'-(1,1-difluoro-4-(tetrahydro-2H-pyran-4-yl)but-1-ene-2,3-diyl)bis(4,4,5,5-tetramethyl-1,3,2-dioxaborolane) (28)

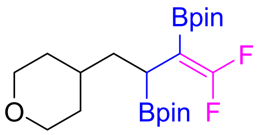

28

^{19}F NMR (470 MHz, $CDCl_3$)

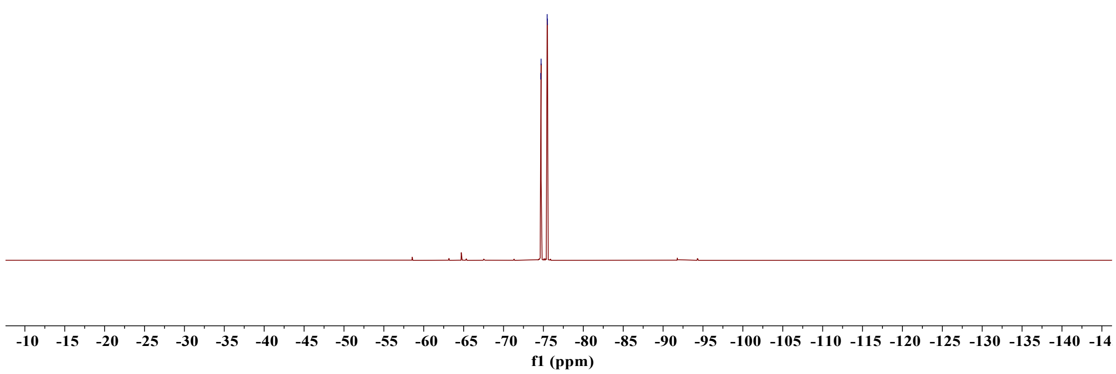

2,2'-(1,1-difluoro-4-(tetrahydro-2H-pyran-4-yl)but-1-ene-2,3-diyl)bis(4,4,5,5-tetramethyl-1,3,2-dioxaborolane) (28)

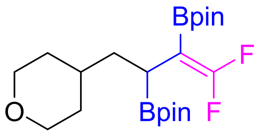

28

^{11}B NMR (128 MHz, $CDCl_3$)

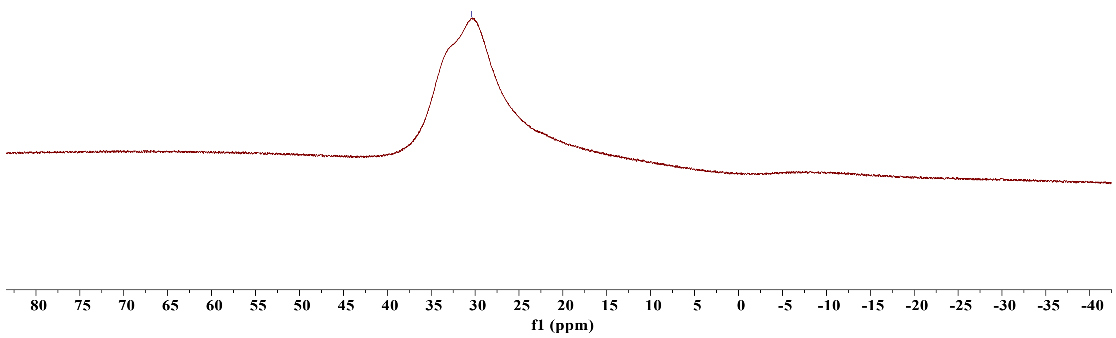

2,2'-(1,1-difluorododec-1-ene-2,3-diyl)bis(4,4,5,5-tetramethyl-1,3,2-dioxaborolane) (29)

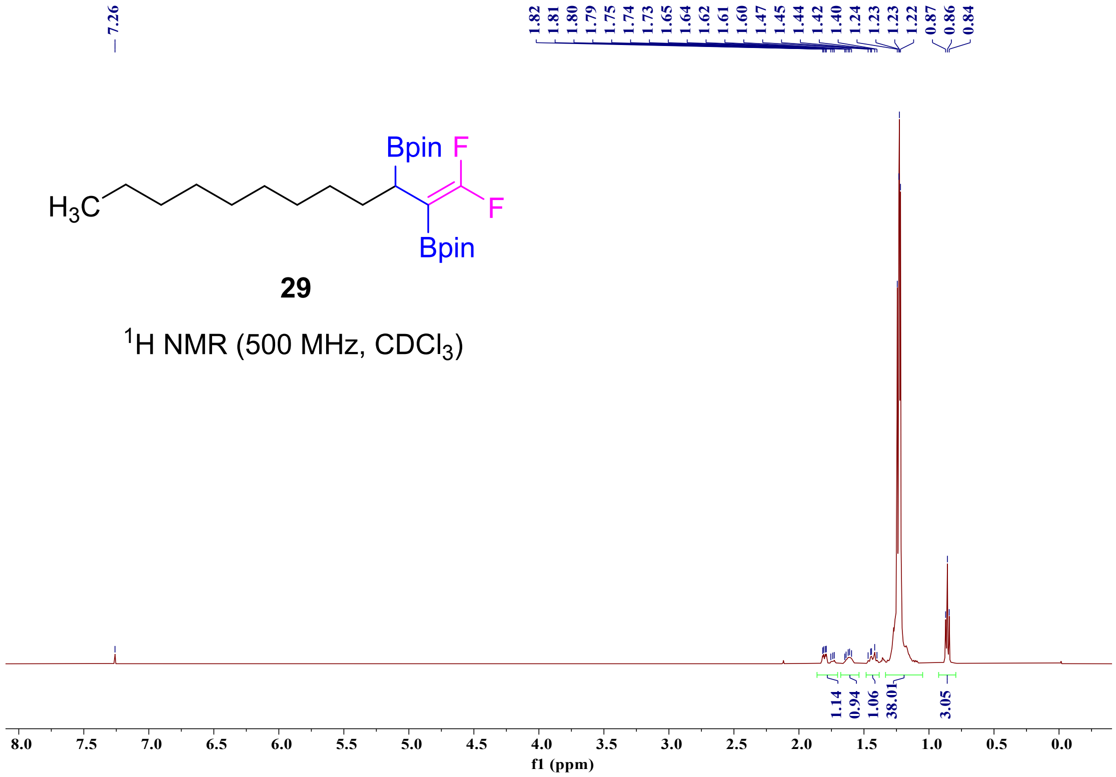

2,2'-(1,1-difluorododec-1-ene-2,3-diyl)bis(4,4,5,5-tetramethyl-1,3,2-dioxaborolane) (29)

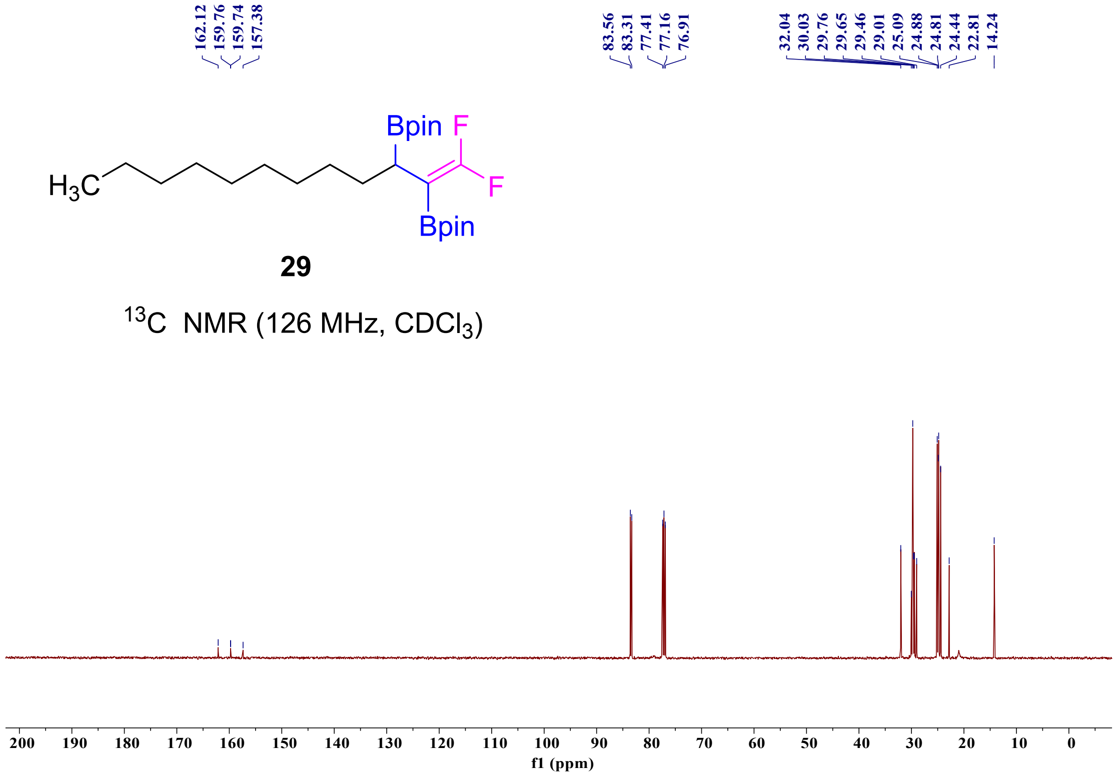

2,2'-(1,1-difluorododec-1-ene-2,3-diyl)bis(4,4,5,5-tetramethyl-1,3,2-dioxaborolane) (29)

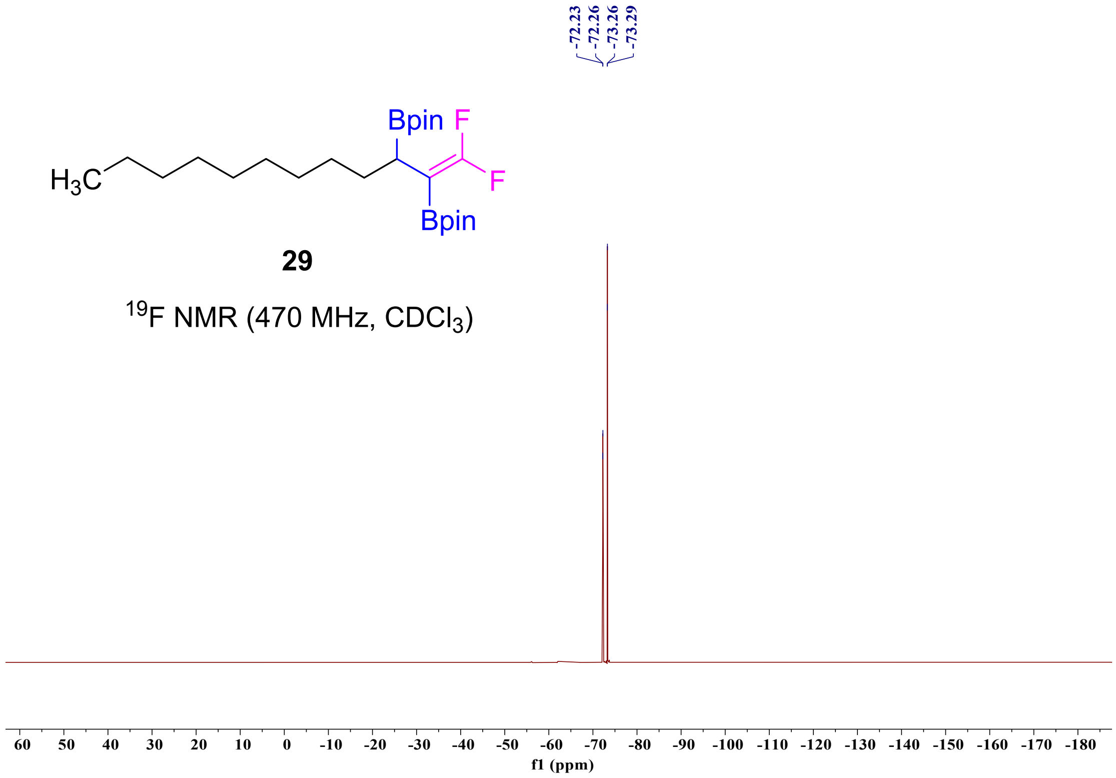

2,2'-(1,1-difluorododec-1-ene-2,3-diyl)bis(4,4,5,5-tetramethyl-1,3,2-dioxaborolane) (29)

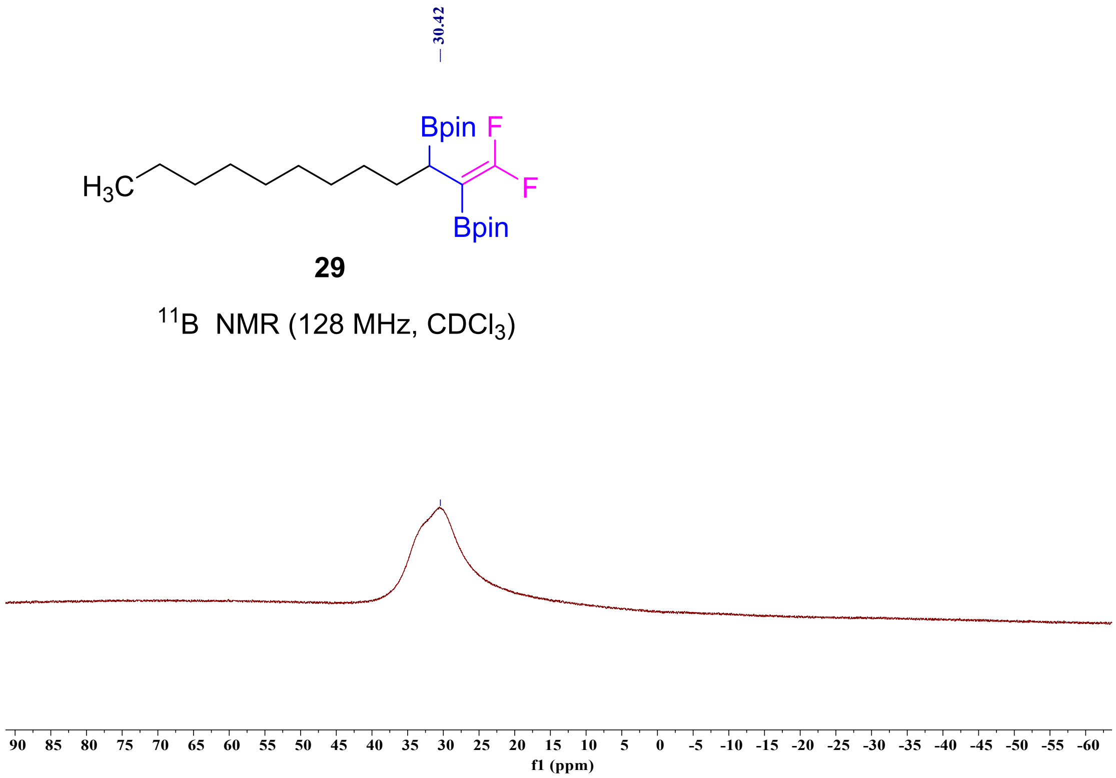

ethyl (R)-8,8-difluoro-6,7-bis(4,4,5,5-tetramethyl-1,3,2-dioxaborolan-2-yl)oct-7-enoate (30)

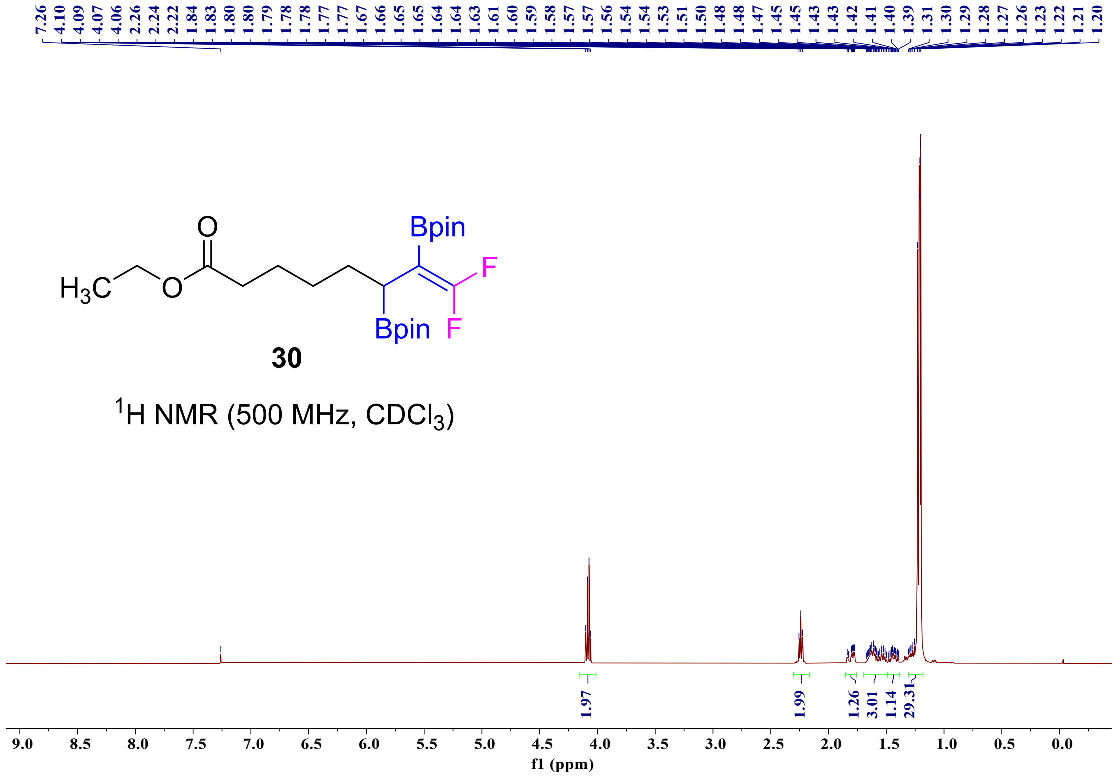

ethyl (R)-8,8-difluoro-6,7-bis(4,4,5,5-tetramethyl-1,3,2-dioxaborolan-2-yl)oct-7-enoate (30)

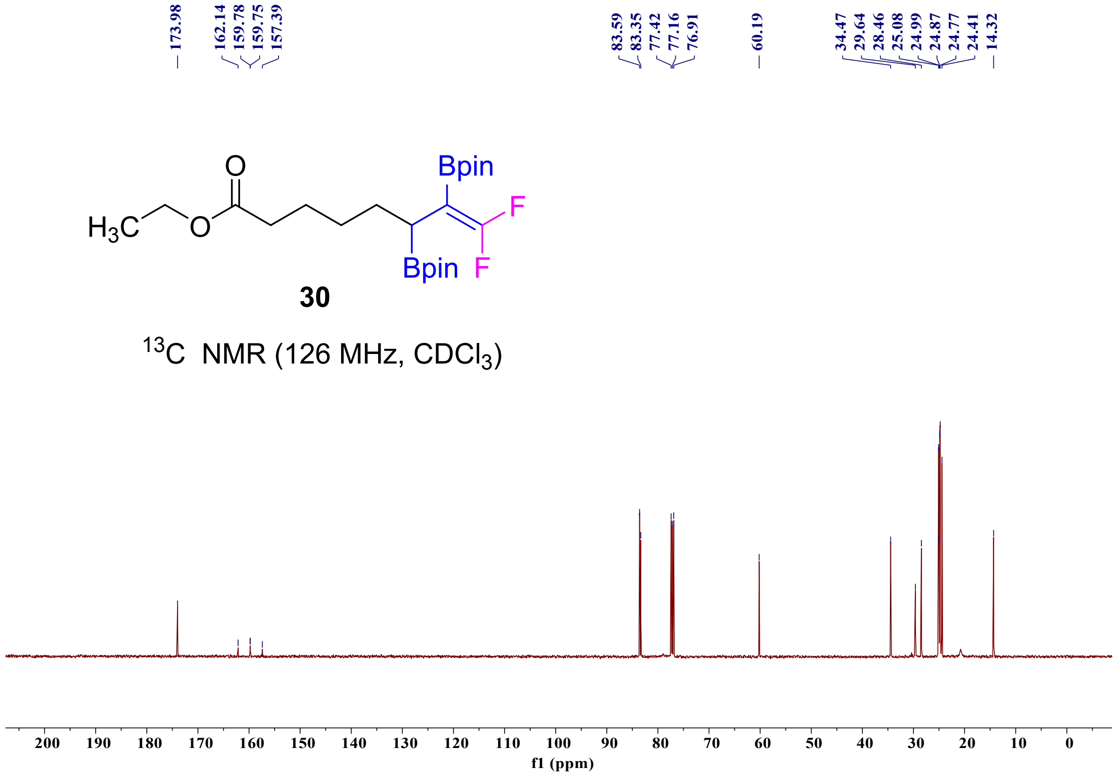

ethyl (R)-8,8-difluoro-6,7-bis(4,4,5,5-tetramethyl-1,3,2-dioxaborolan-2-yl)oct-7-enoate (30)

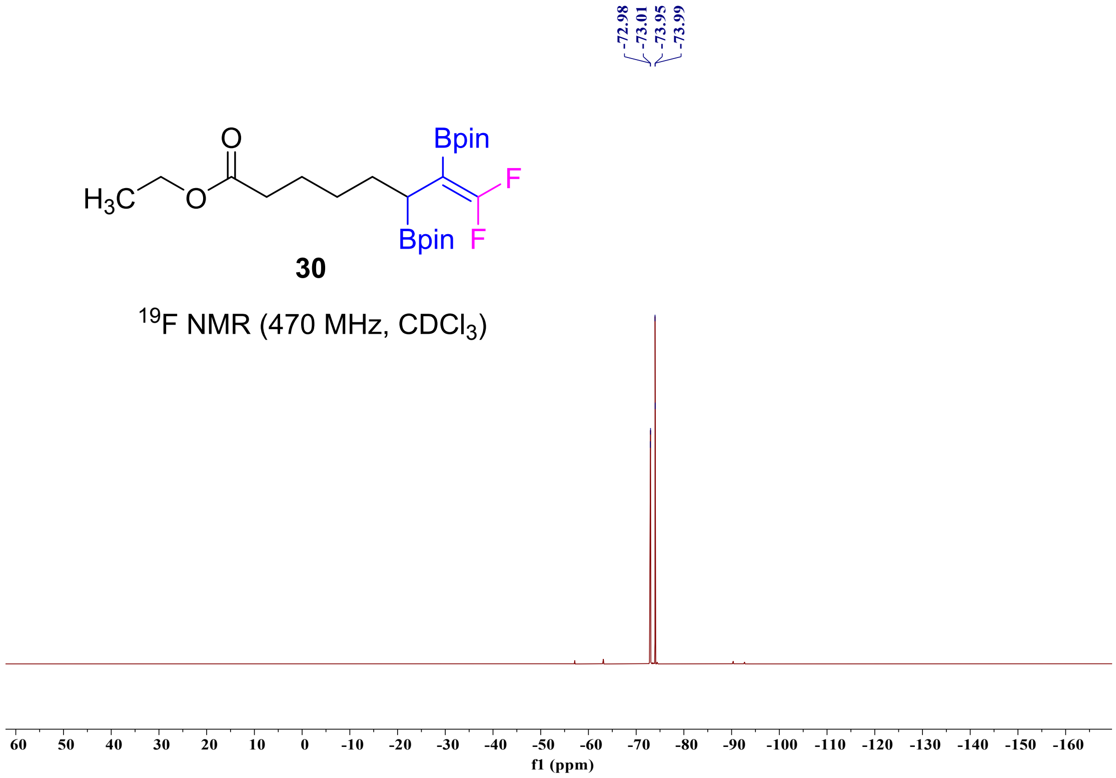

ethyl (R)-8,8-difluoro-6,7-bis(4,4,5,5-tetramethyl-1,3,2-dioxaborolan-2-yl)oct-7-enoate (30)

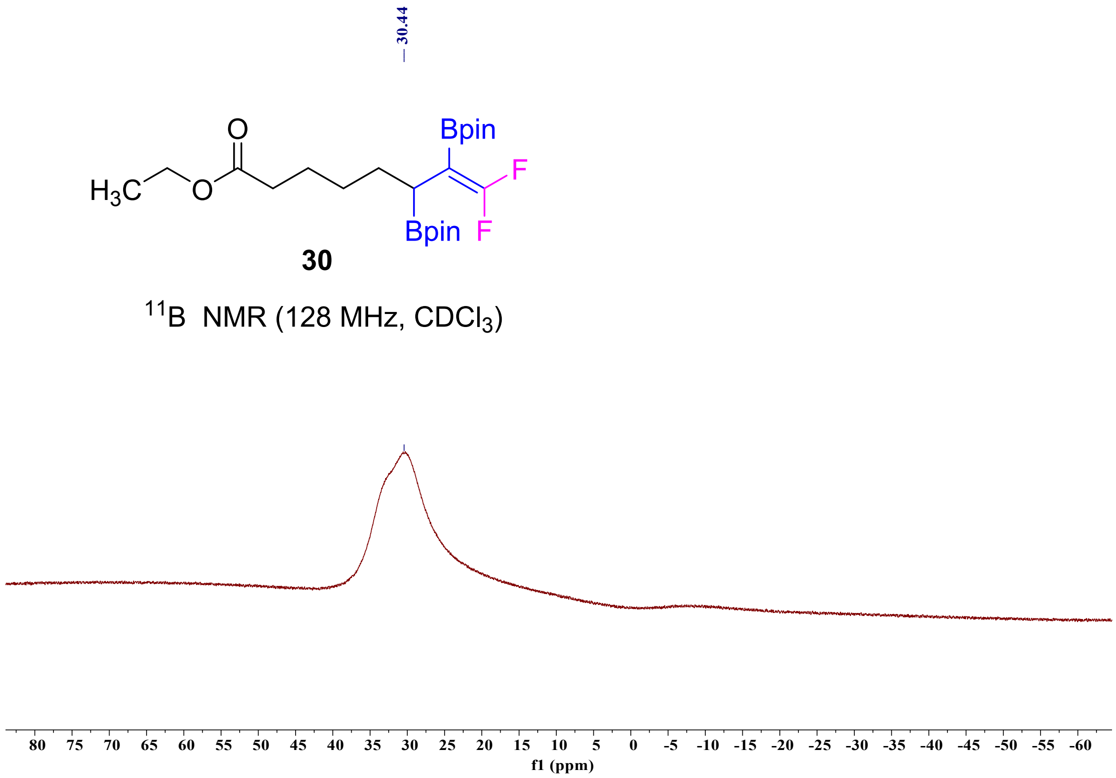

tert-butyl((5,5-difluoro-3,4-bis(4,4,5,5-tetramethyl-1,3,2-dioxaborolan-2-yl)pent-4-en-1-yl)oxy)dimethylsilane (31)

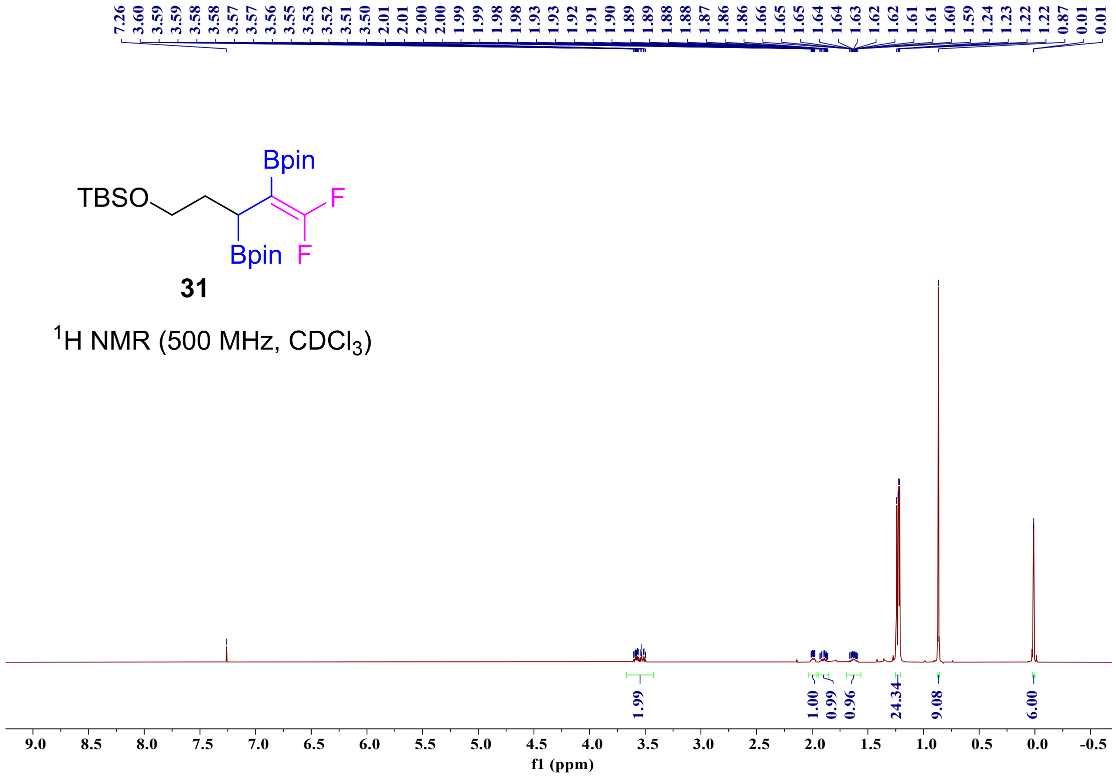

tert-butyl((5,5-difluoro-3,4-bis(4,4,5,5-tetramethyl-1,3,2-dioxaborolan-2-yl)pent-4-en-1-yl)oxy)dimethylsilane (31)

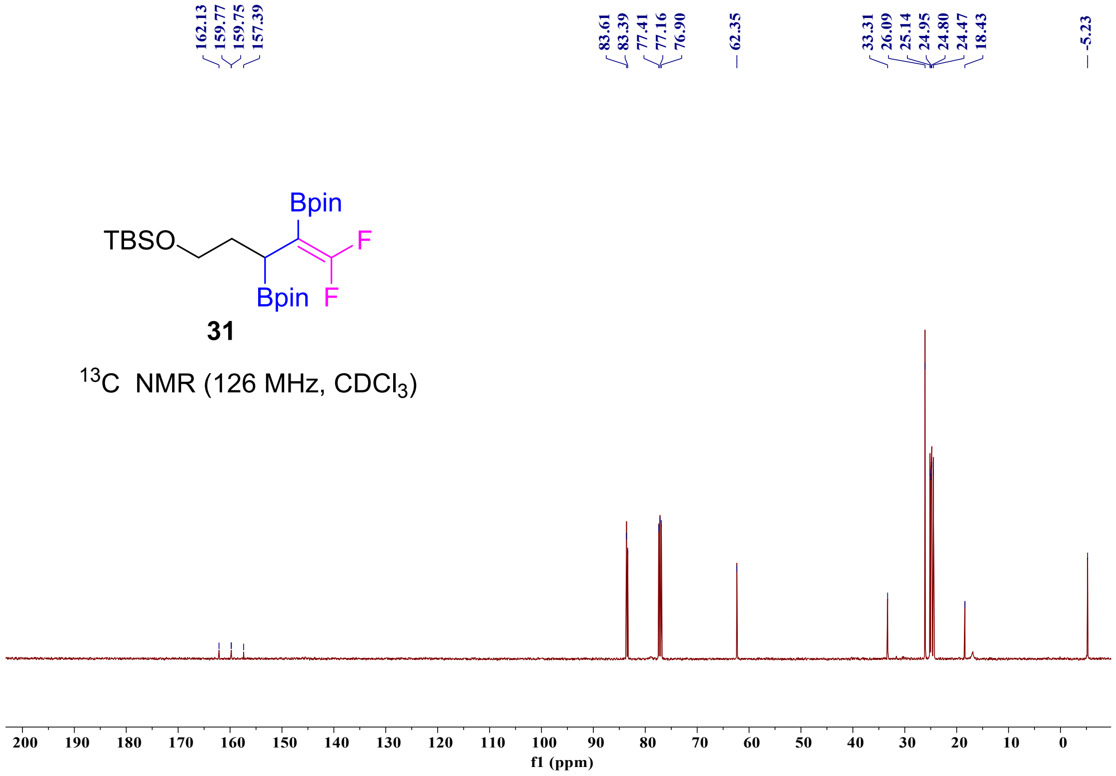

tert-butyl((5,5-difluoro-3,4-bis(4,4,5,5-tetramethyl-1,3,2-dioxaborolan-2-yl)pent-4-en-1-yl)oxy)dimethylsilane (31)

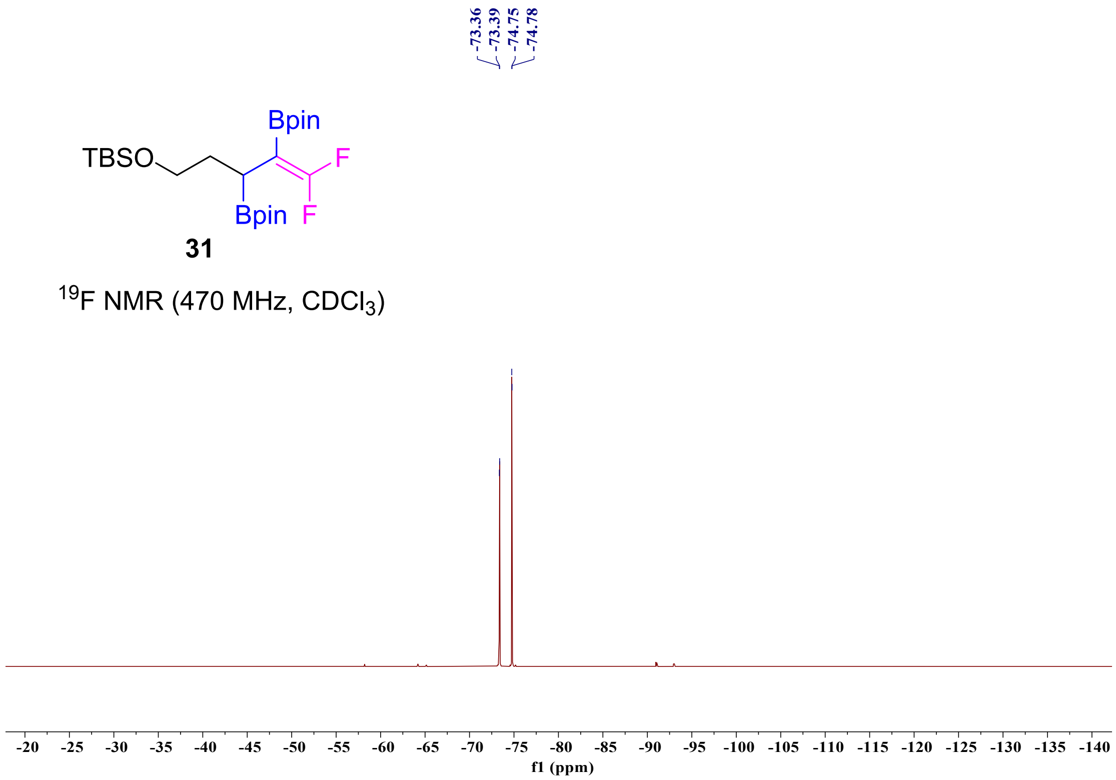

tert-butyl((5,5-difluoro-3,4-bis(4,4,5,5-tetramethyl-1,3,2-dioxaborolan-2-yl)pent-4-en-1-yl)oxy)dimethylsilane (31)

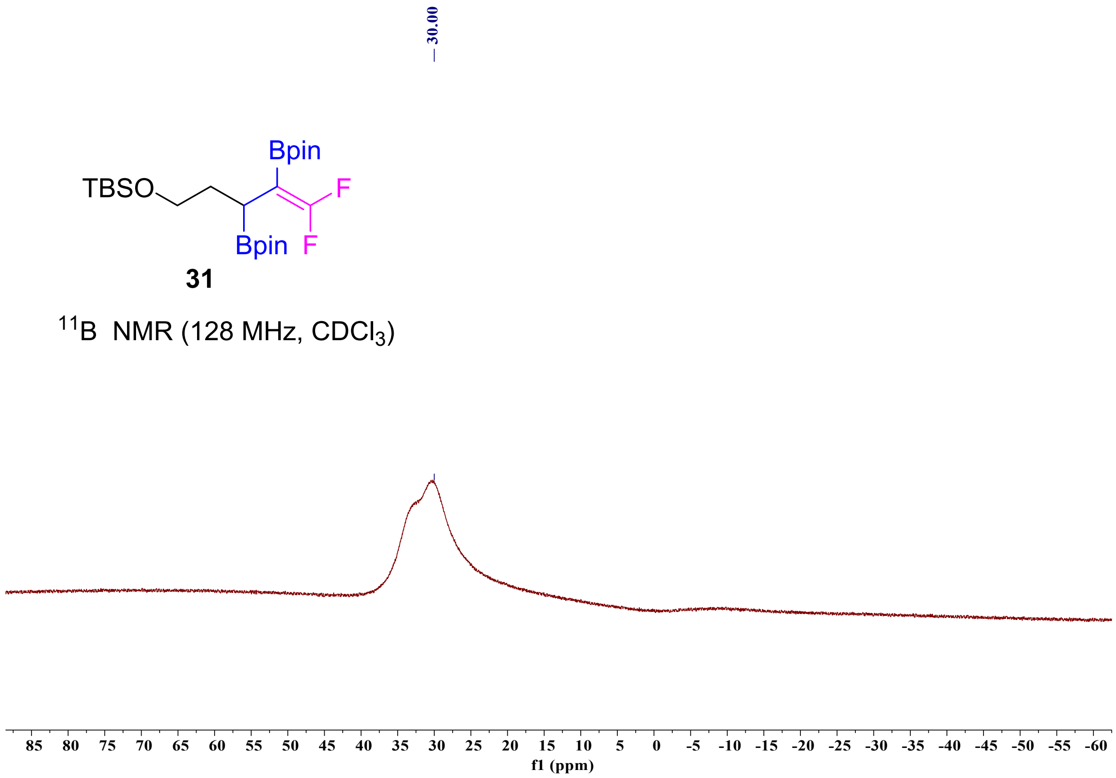

2,2'-(11-bromo-1,1-difluoroundec-1-ene-2,3-diyl)bis(4,4,5,5-tetramethyl-1,3,2-dioxaborolane) (32)

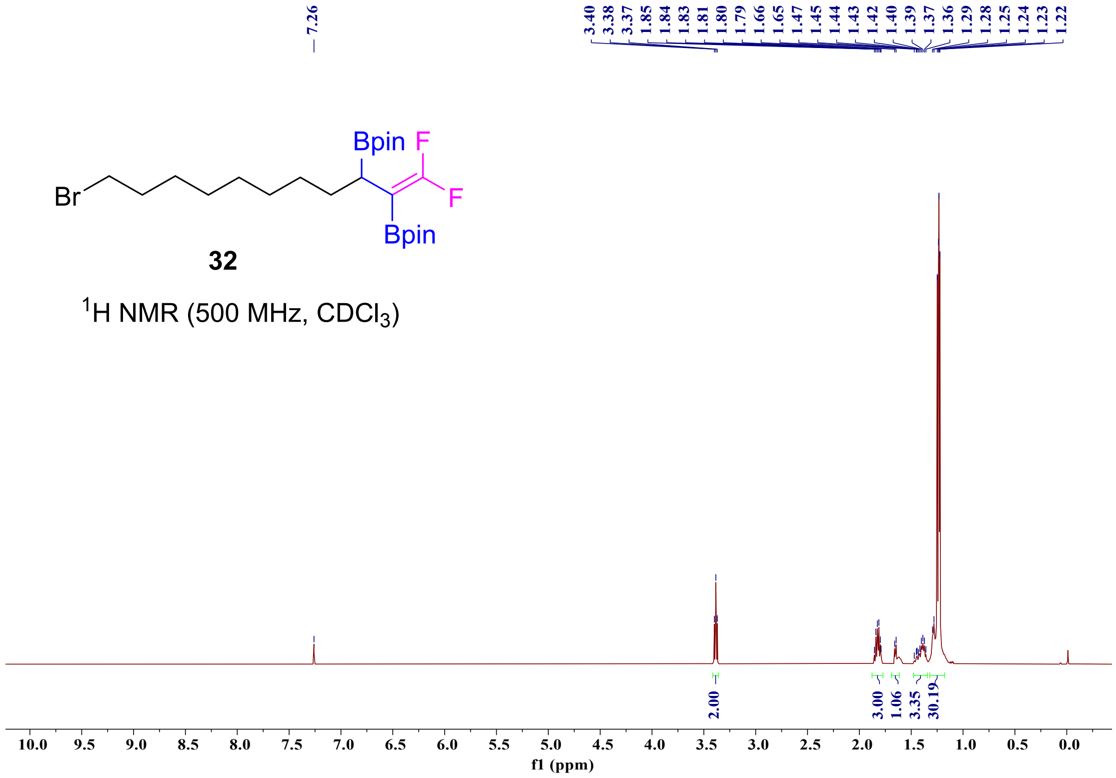

2,2'-(11-bromo-1,1-difluoroundec-1-ene-2,3-diyl)bis(4,4,5,5-tetramethyl-1,3,2-dioxaborolane) (32)

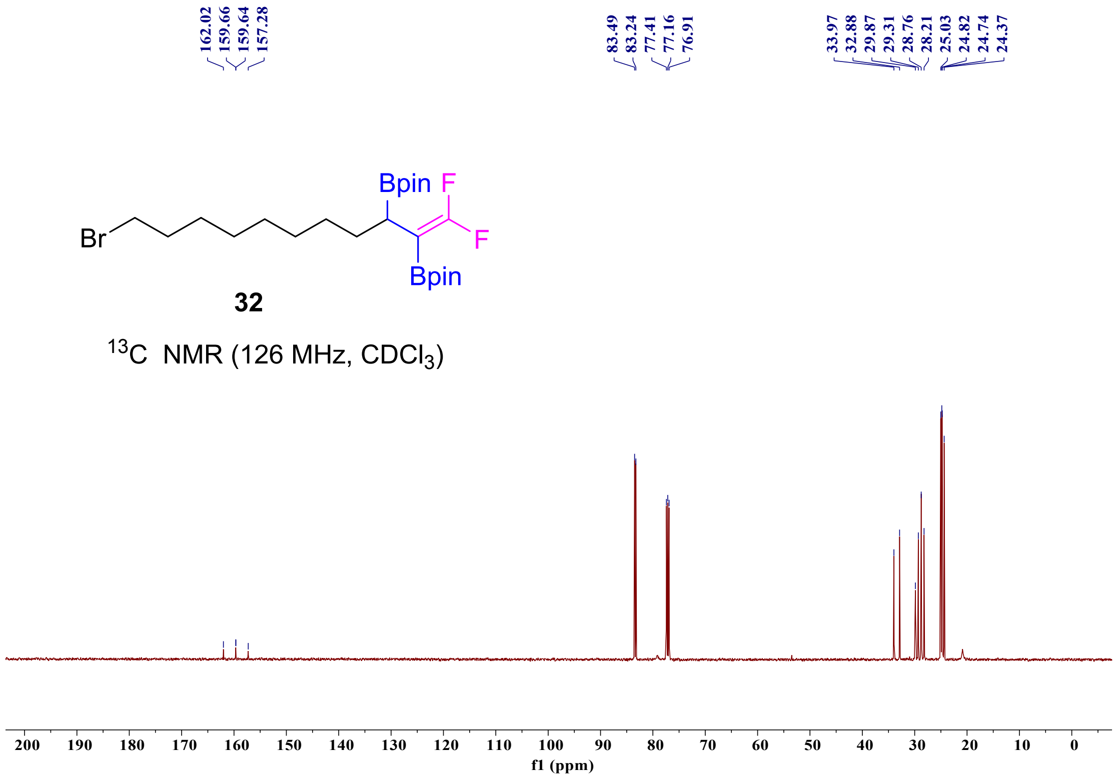

2,2'-(11-bromo-1,1-difluoroundec-1-ene-2,3-diyl)bis(4,4,5,5-tetramethyl-1,3,2-dioxaborolane) (32)

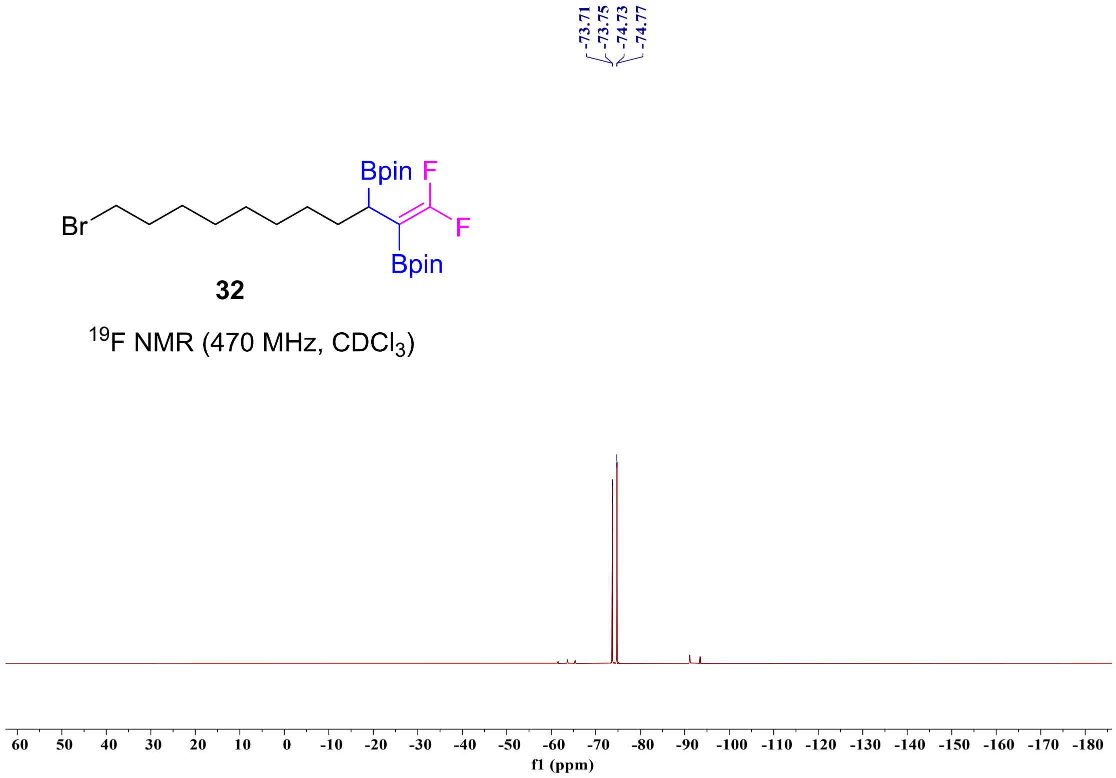

2,2'-(11-bromo-1,1-difluoroundec-1-ene-2,3-diyl)bis(4,4,5,5-tetramethyl-1,3,2-dioxaborolane) (32)

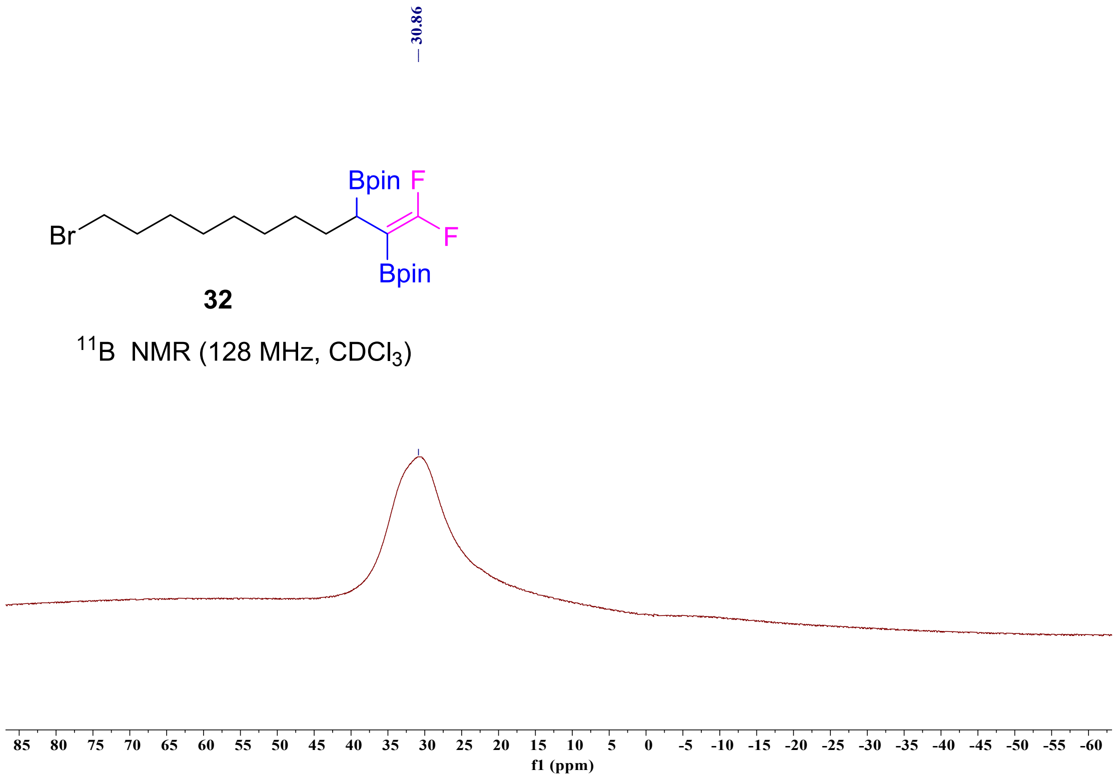

2,2'-(5-cyclohexyl-1,1-difluoropent-1-ene-2,3-diyl)bis(4,4,5,5-tetramethyl-1,3,2-dioxaborolane) (33)

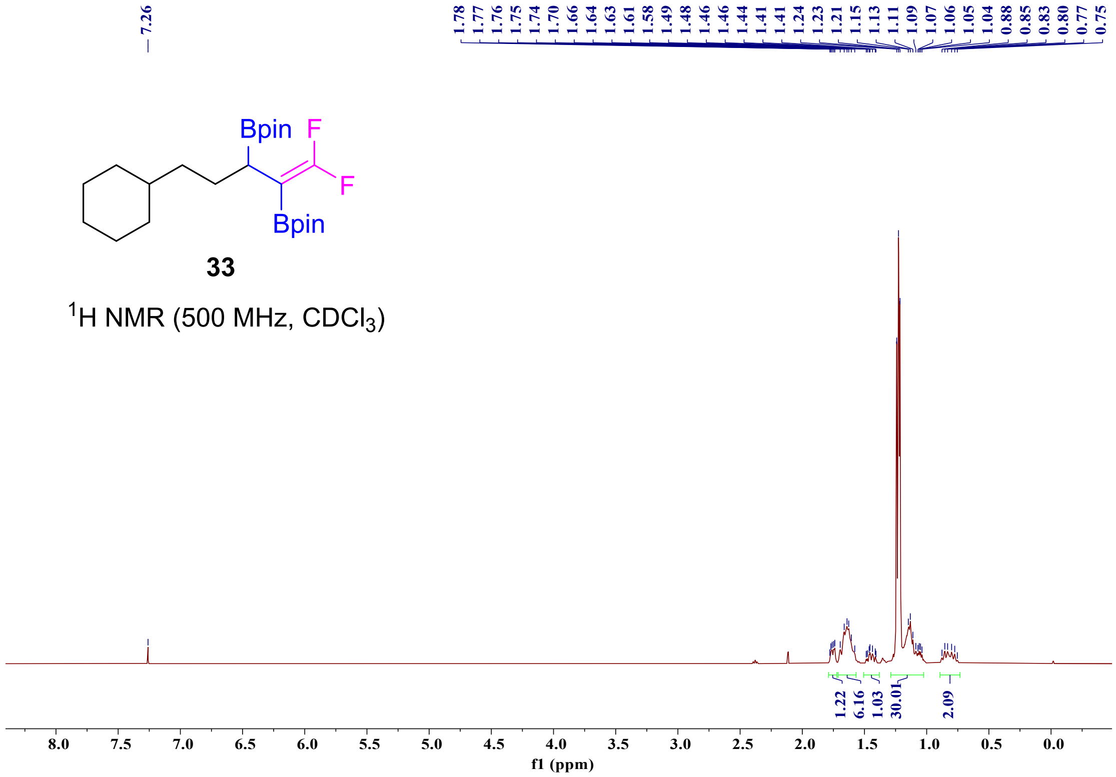

2,2'-(5-cyclohexyl-1,1-difluoropent-1-ene-2,3-diyl)bis(4,4,5,5-tetramethyl-1,3,2-dioxaborolane) (33)

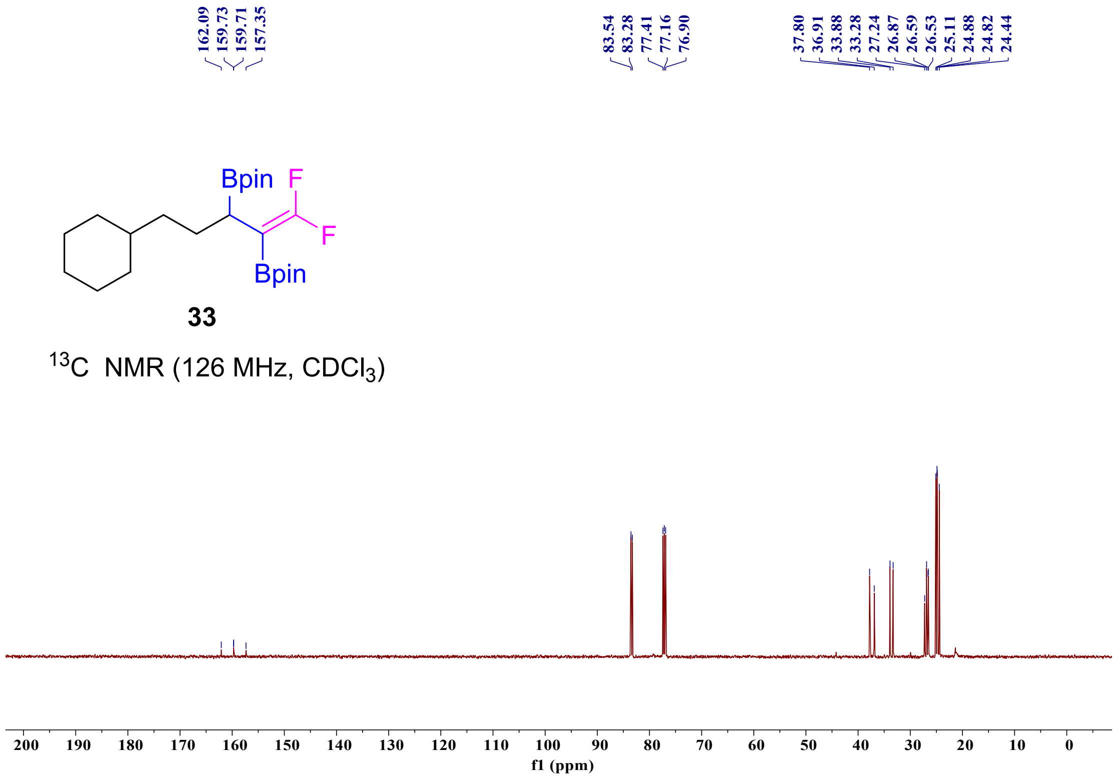

2,2'-(5-cyclohexyl-1,1-difluoropent-1-ene-2,3-diyl)bis(4,4,5,5-tetramethyl-1,3,2-dioxaborolane) (33)

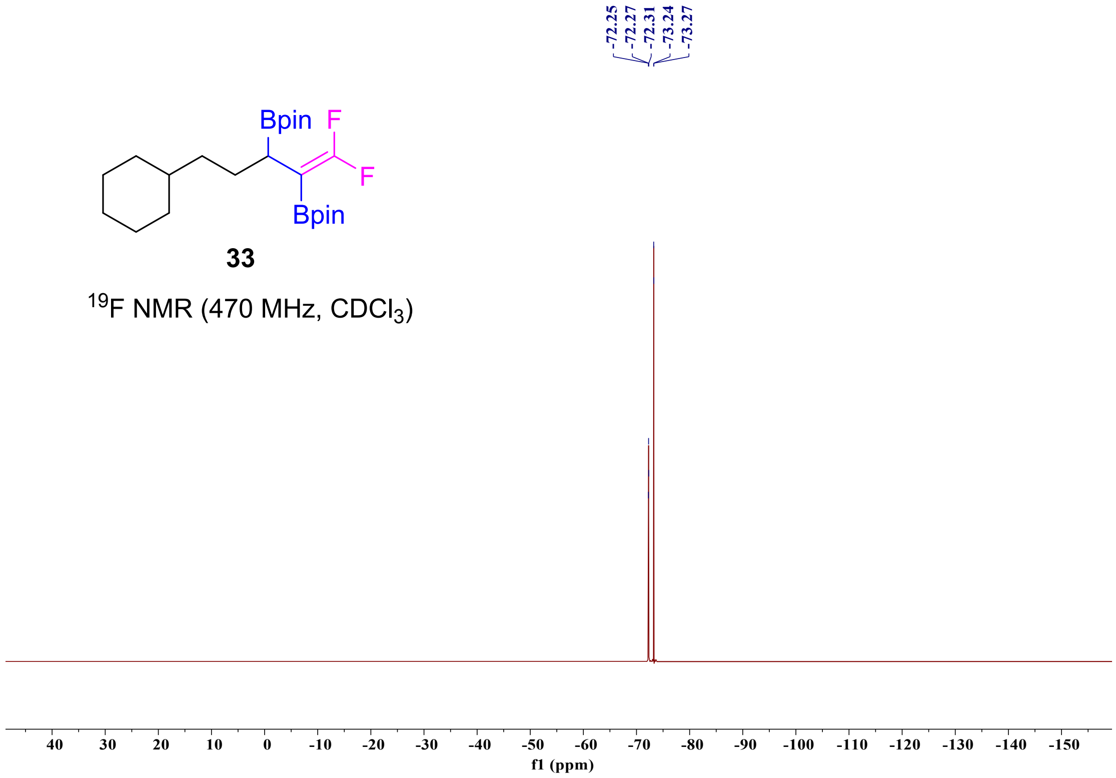

2,2'-(5-cyclohexyl-1,1-difluoropent-1-ene-2,3-diyl)bis(4,4,5,5-tetramethyl-1,3,2-dioxaborolane) (33)

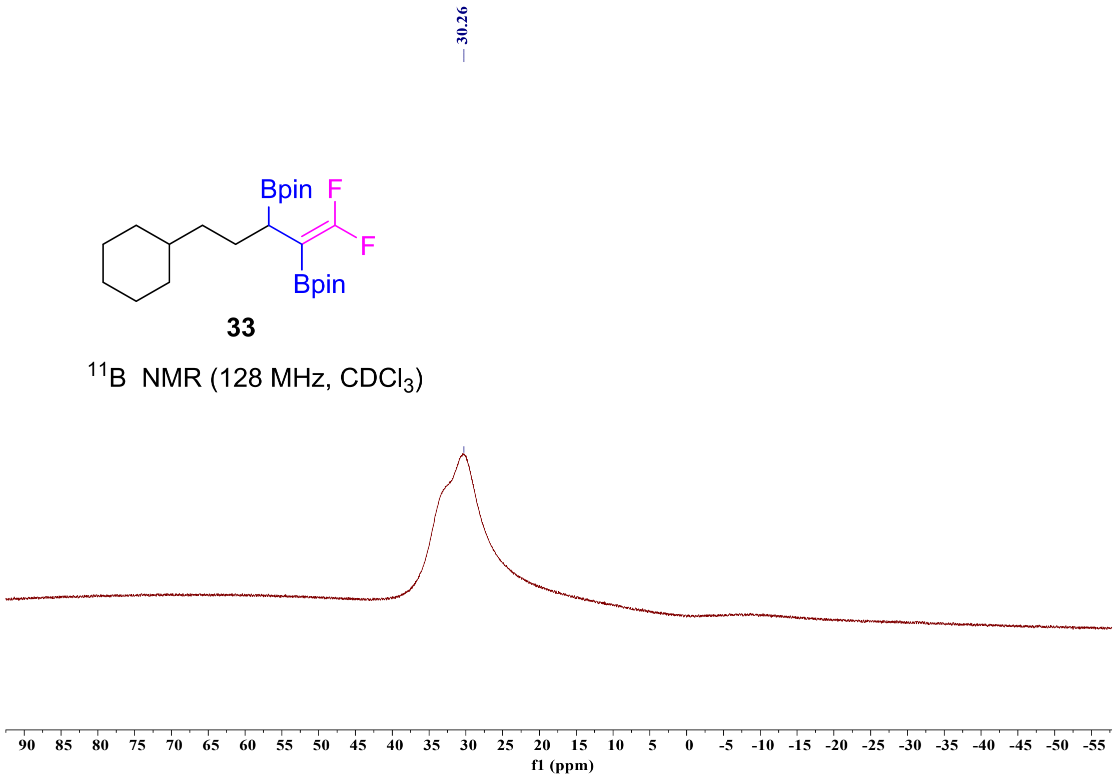

2,2'-(1-(2,3-dihydro-1H-inden-2-yl)-3,3-difluoroprop-2-ene-1,2-diyl)bis(4,4,5,5-tetramethyl-1,3,2-dioxaborolane) (34)

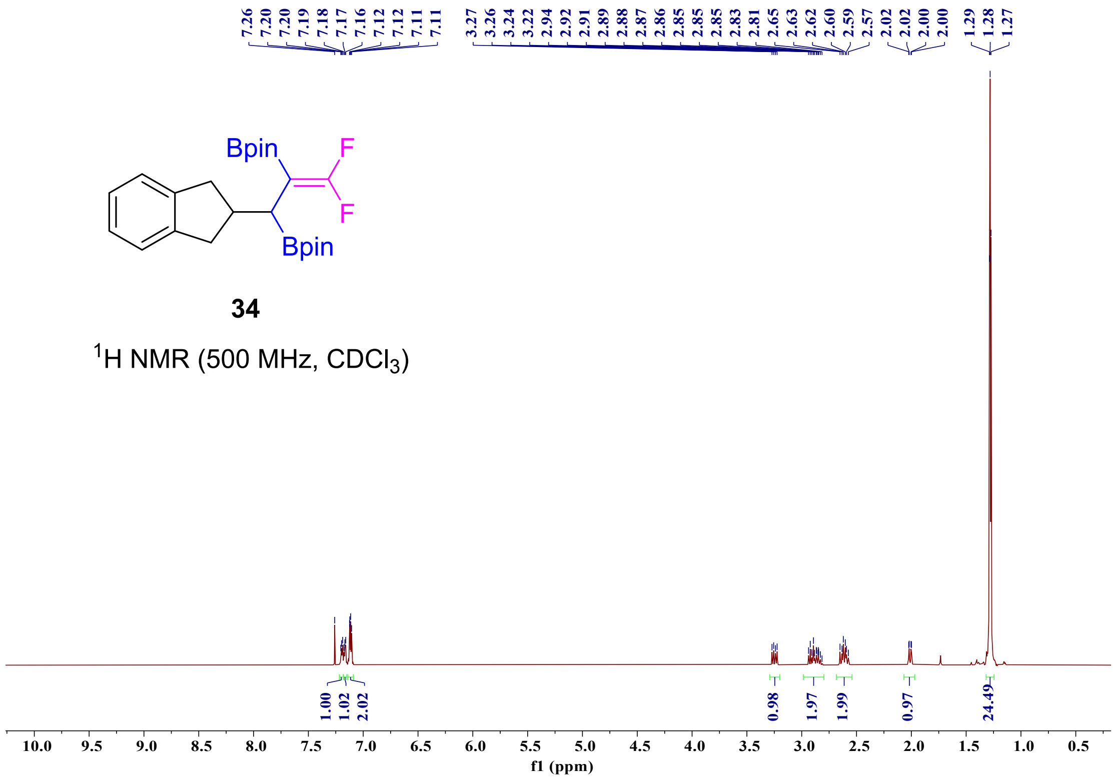

2,2'-(1-(2,3-dihydro-1H-inden-2-yl)-3,3-difluoroprop-2-ene-1,2-diyl)bis(4,4,5,5-tetramethyl-1,3,2-dioxaborolane) (34)

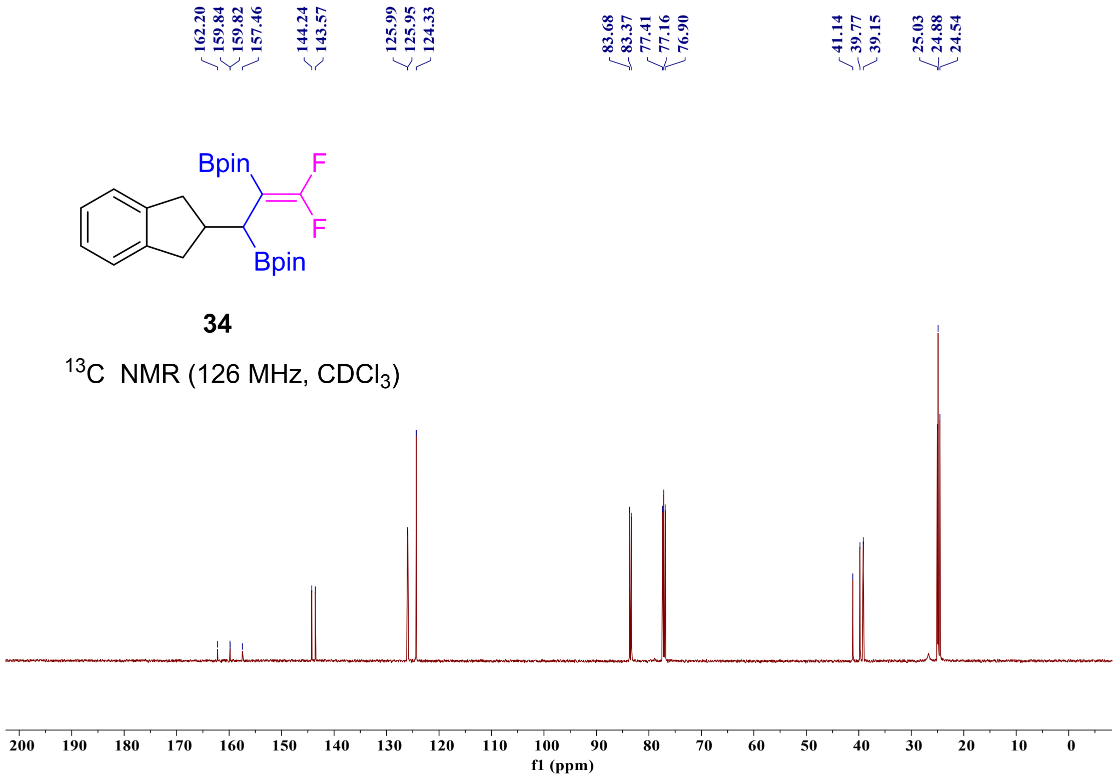

2,2'-(1-(2,3-dihydro-1H-inden-2-yl)-3,3-difluoroprop-2-ene-1,2-diyl)bis(4,4,5,5-tetramethyl-1,3,2-dioxaborolane) (34)

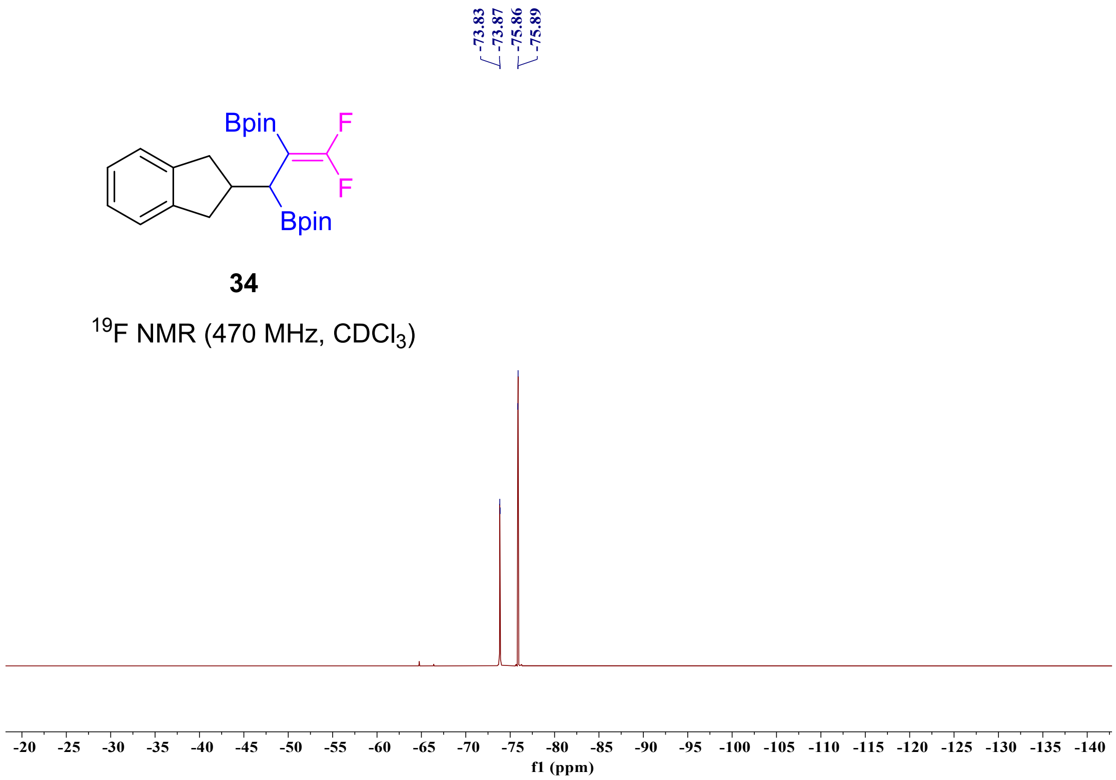

2,2'-(1-(2,3-dihydro-1H-inden-2-yl)-3,3-difluoroprop-2-ene-1,2-diyl)bis(4,4,5,5-tetramethyl-1,3,2-dioxaborolane) (34)

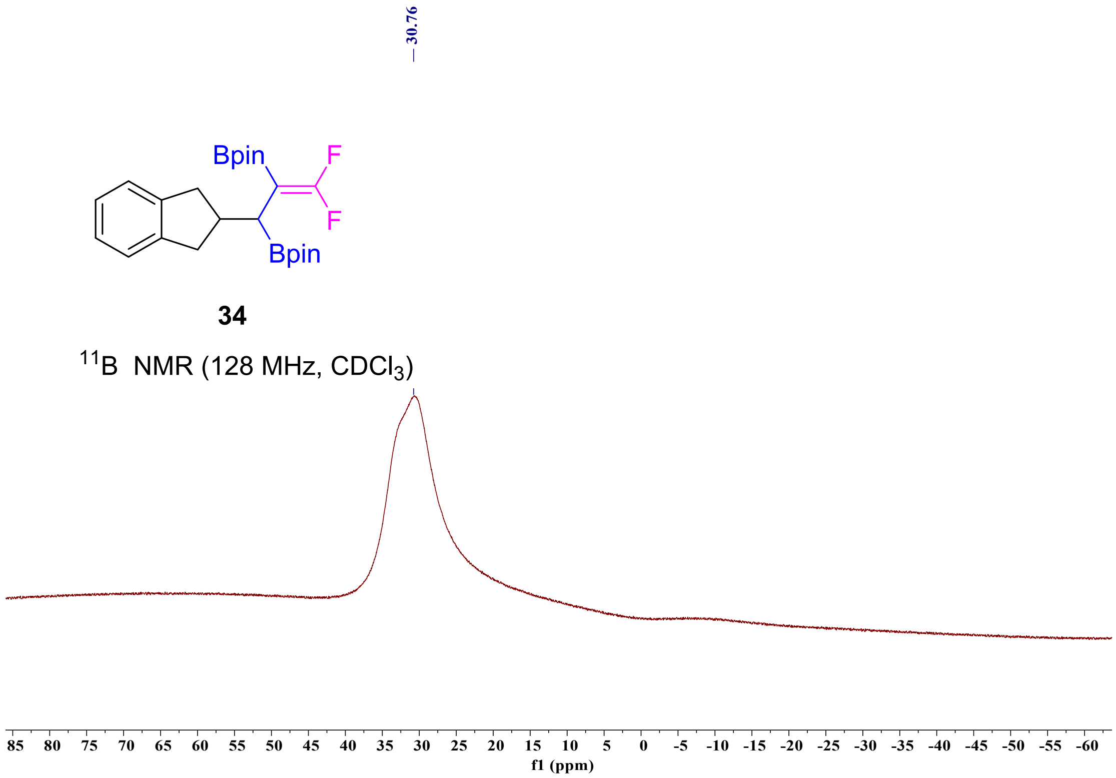

2,2'-(3,3-difluoro-1-(1-phenylcyclopropyl)prop-2-ene-1,2-diyl)bis(4,4,5,5-tetramethyl-1,3,2-dioxaborolane) (35)

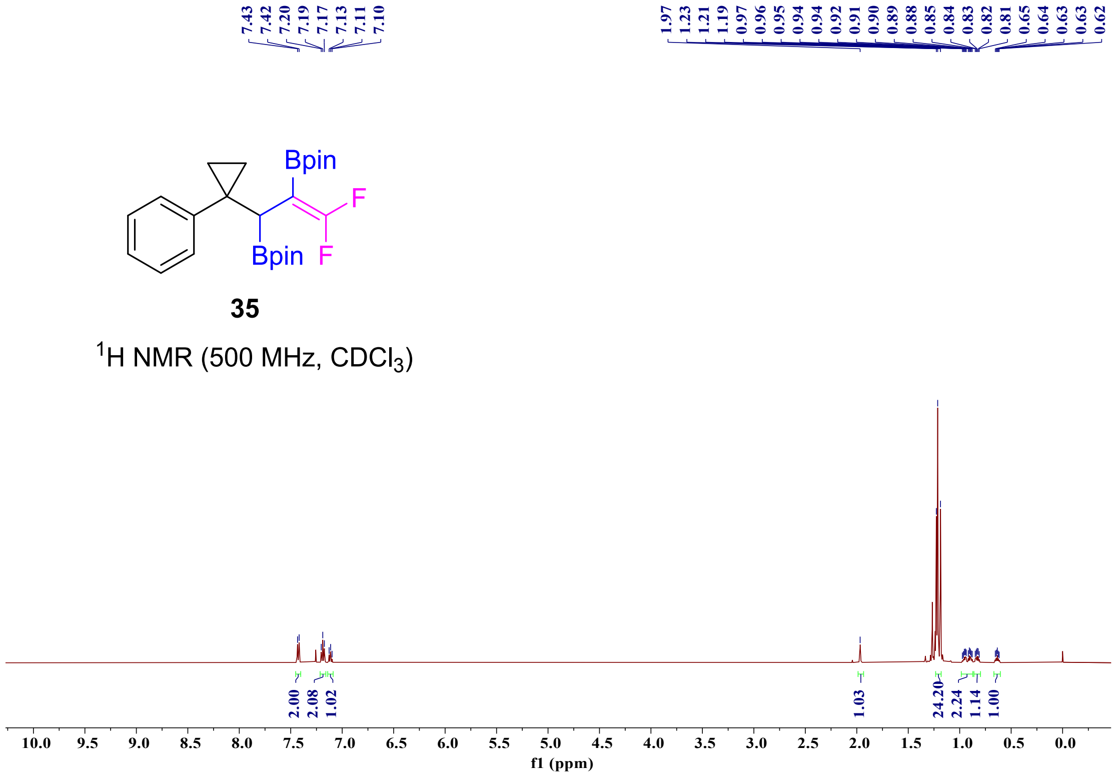

2,2'-(3,3-difluoro-1-(1-phenylcyclopropyl)prop-2-ene-1,2-diyl)bis(4,4,5,5-tetramethyl-1,3,2-dioxaborolane) (35)

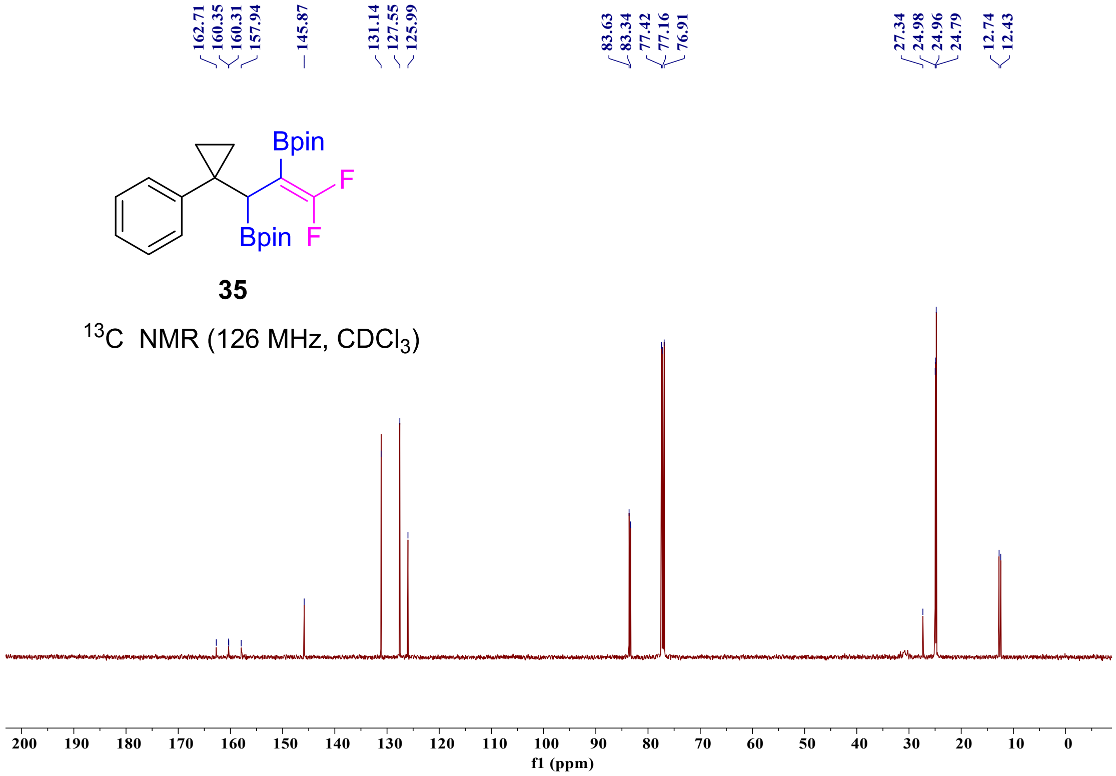

2,2'-(3,3-difluoro-1-(1-phenylcyclopropyl)prop-2-ene-1,2-diyl)bis(4,4,5,5-tetramethyl-1,3,2-dioxaborolane) (35)

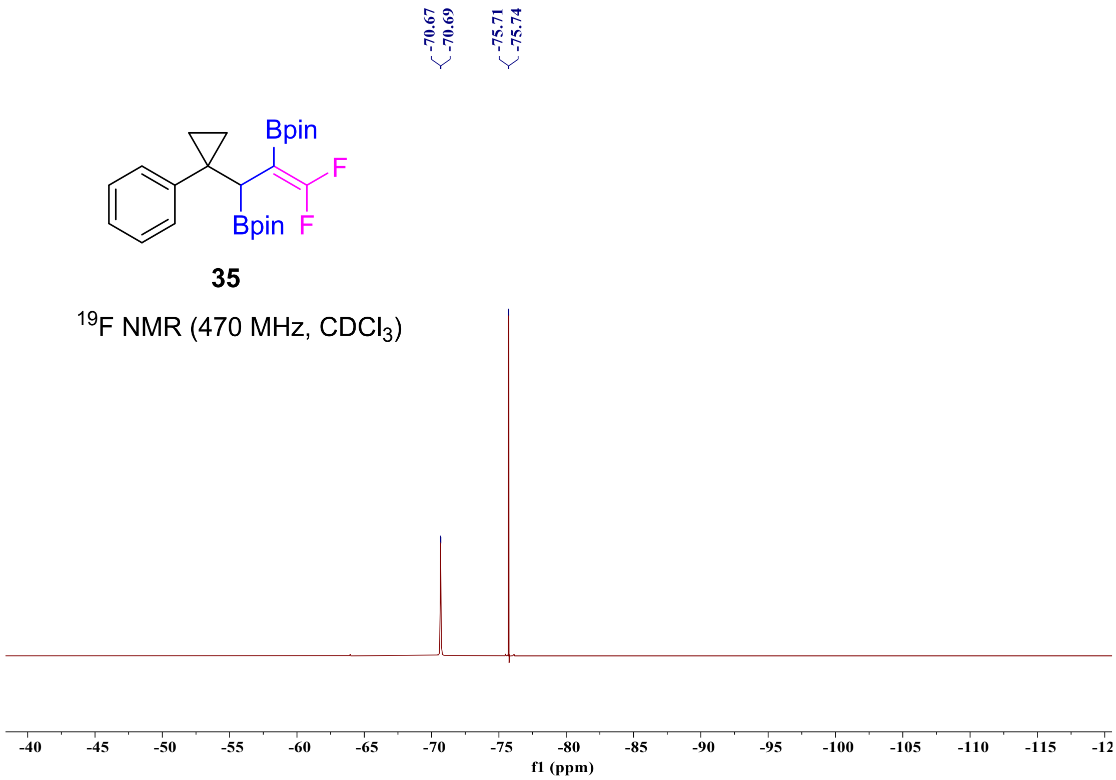

2,2'-(3,3-difluoro-1-(1-phenylcyclopropyl)prop-2-ene-1,2-diyl)bis(4,4,5,5-tetramethyl-1,3,2-dioxaborolane) (35)

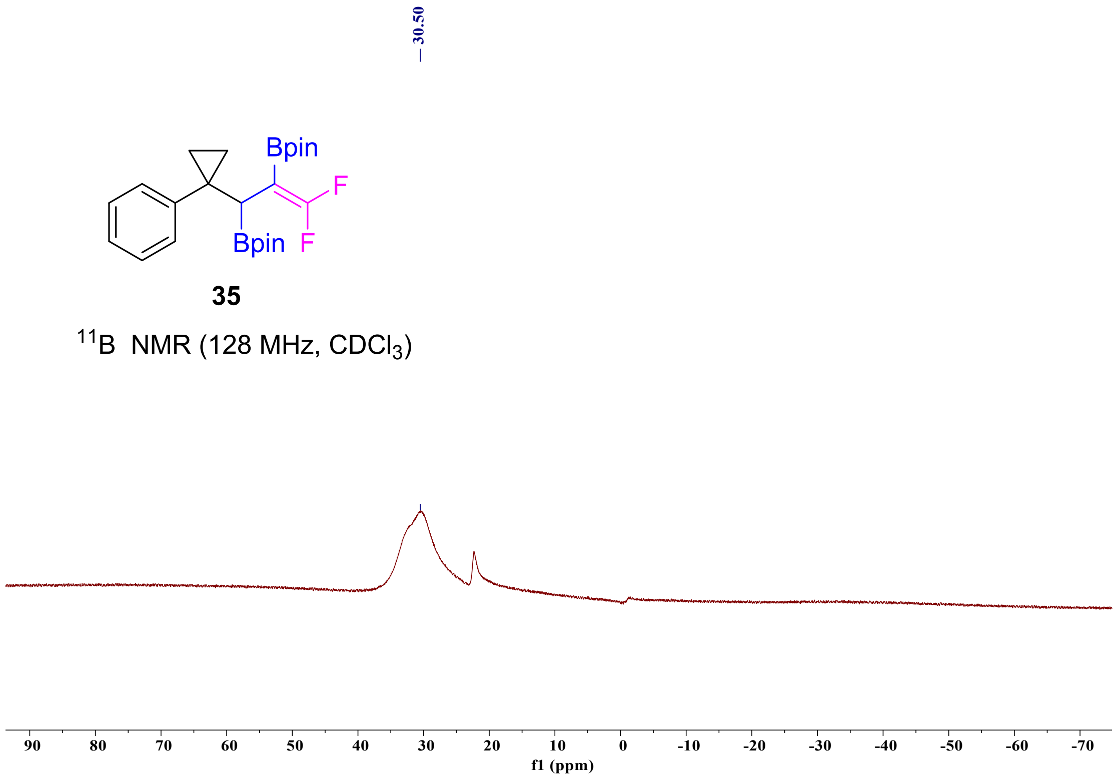

2,2'-(5-cyclopentyl-1,1-difluoropent-1-ene-2,3-diyl)bis(4,4,5,5-tetramethyl-1,3,2-dioxaborolane) (36)

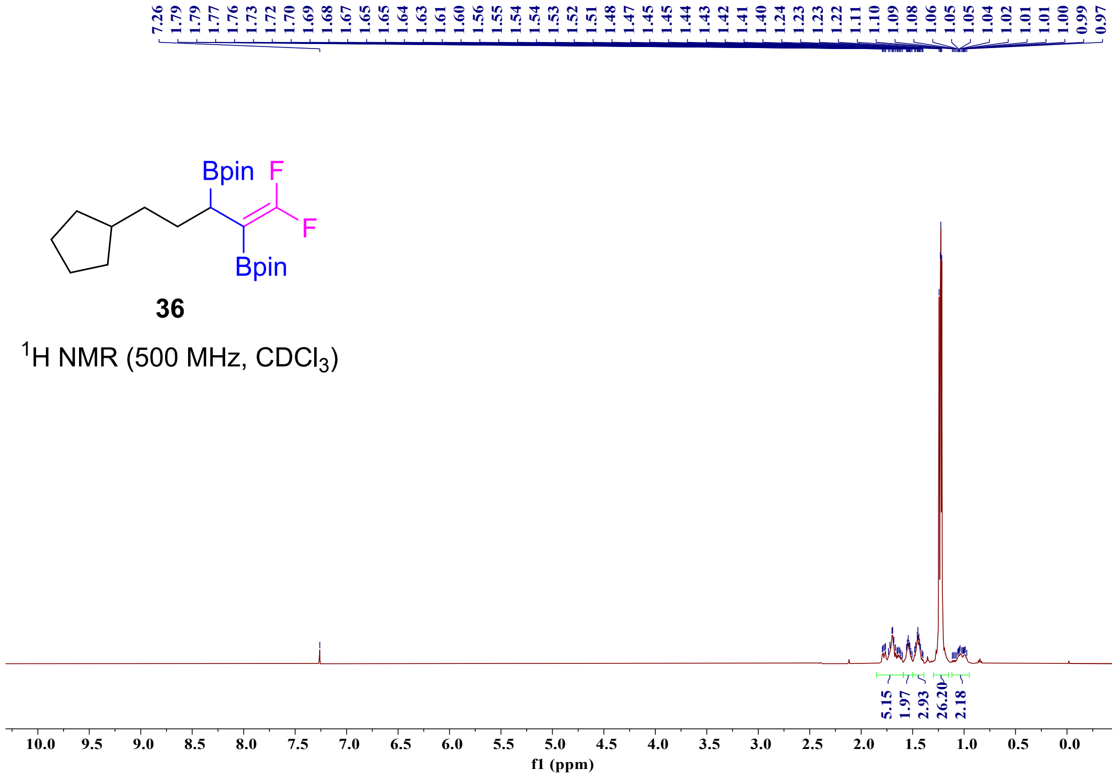

2,2'-(5-cyclopentyl-1,1-difluoropent-1-ene-2,3-diyl)bis(4,4,5,5-tetramethyl-1,3,2-dioxaborolane) (36)

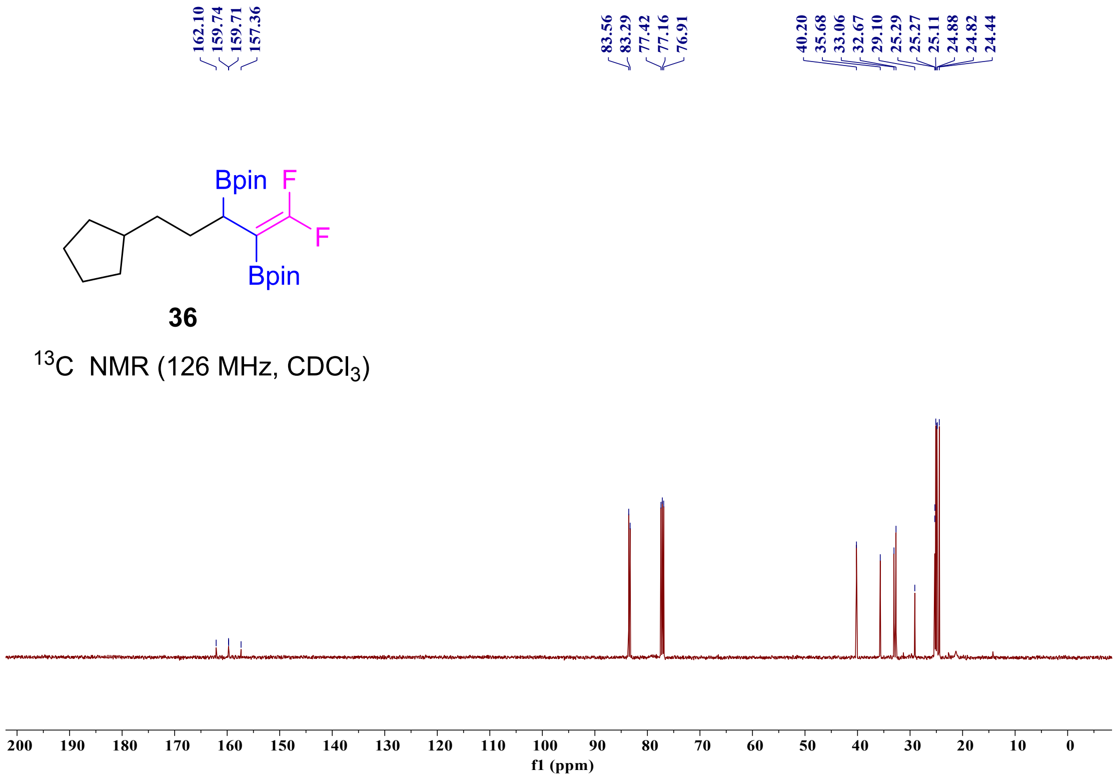

2,2'-(5-cyclopentyl-1,1-difluoropent-1-ene-2,3-diyl)bis(4,4,5,5-tetramethyl-1,3,2-dioxaborolane) (36)

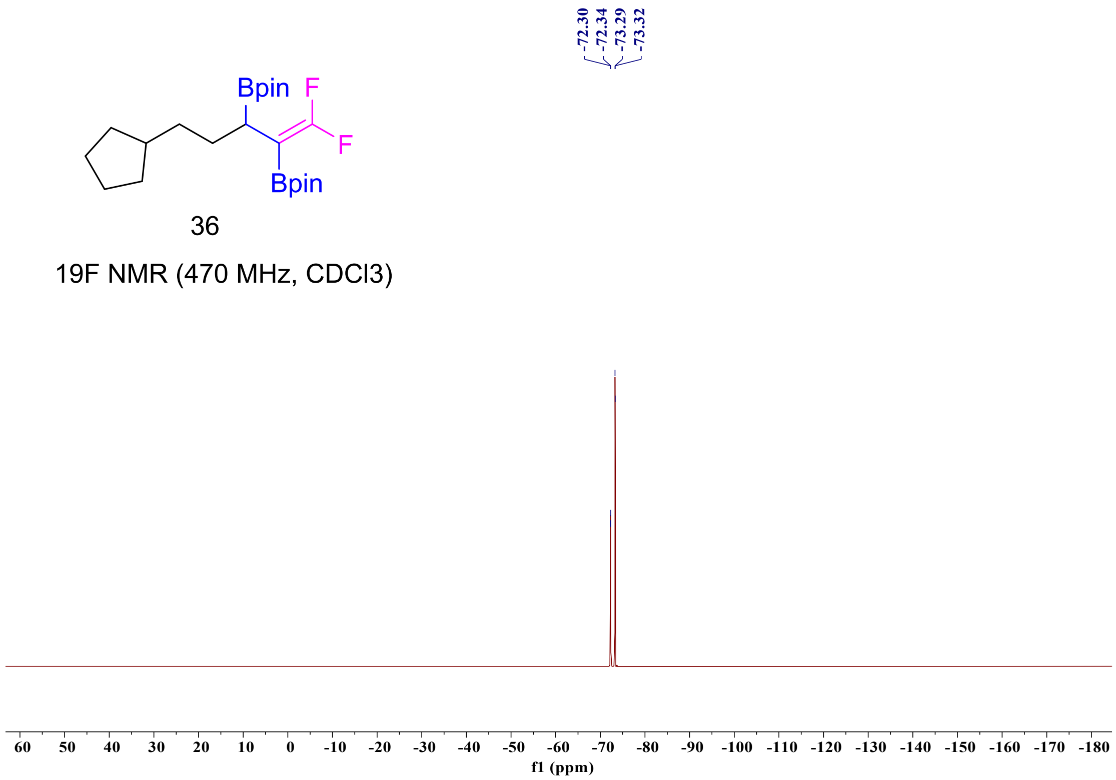

2,2'-(5-cyclopentyl-1,1-difluoropent-1-ene-2,3-diyl)bis(4,4,5,5-tetramethyl-1,3,2-dioxaborolane) (36)

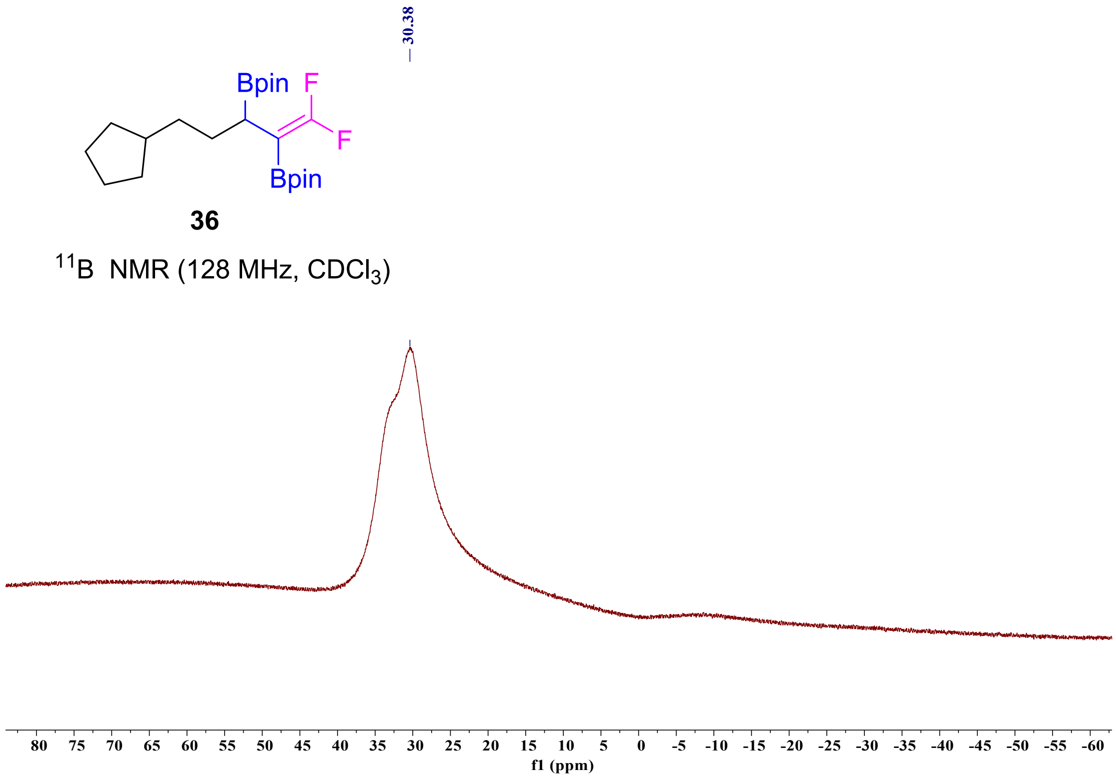

2,2'-(1-(4,4-difluorocyclohexyl)-3,3-difluoroprop-2-ene-1,2-diyl)bis(4,4,5,5-tetramethyl-1,3,2-dioxaborolane) (37)

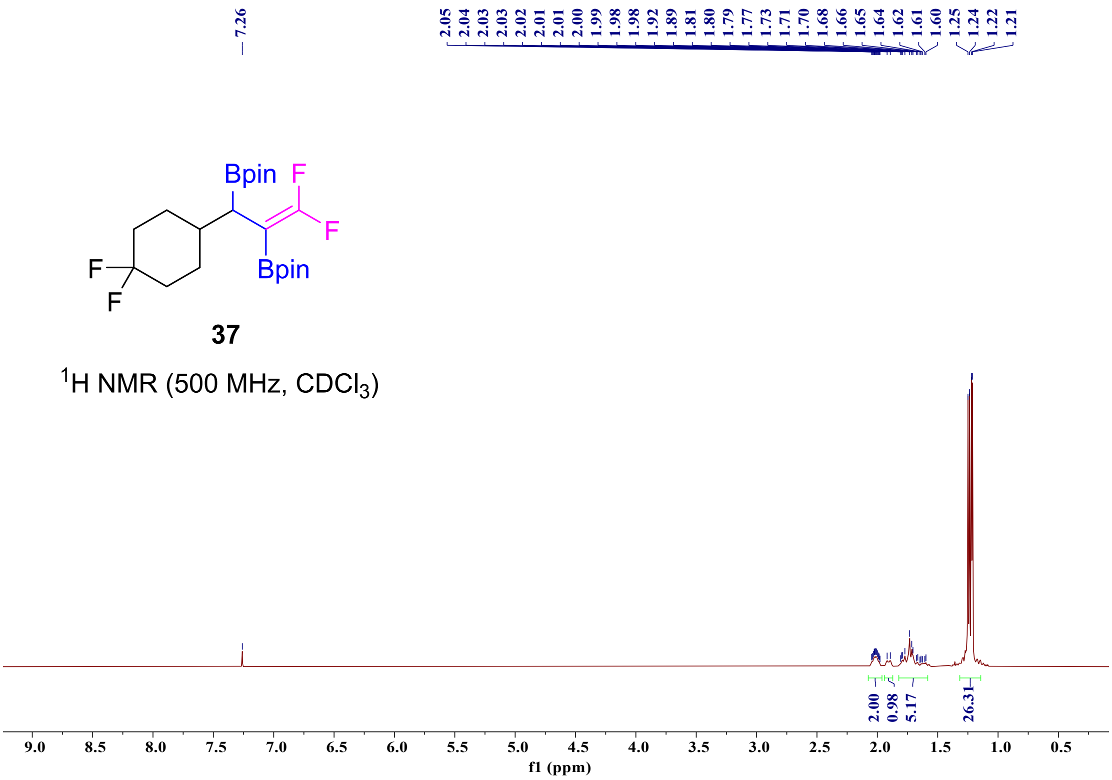

2,2'-(1-(4,4-difluorocyclohexyl)-3,3-difluoroprop-2-ene-1,2-diyl)bis(4,4,5,5-tetramethyl-1,3,2-dioxaborolane) (37)

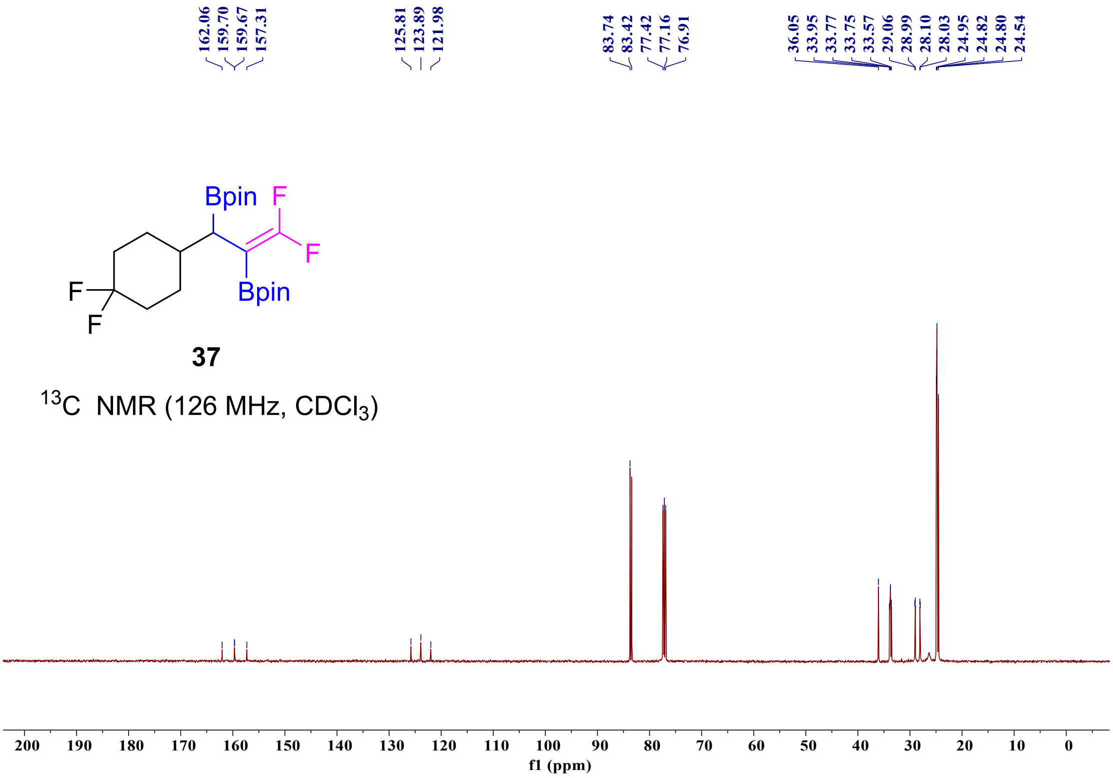

2,2'-(1-(4,4-difluorocyclohexyl)-3,3-difluoroprop-2-ene-1,2-diyl)bis(4,4,5,5-tetramethyl-1,3,2-dioxaborolane) (37)

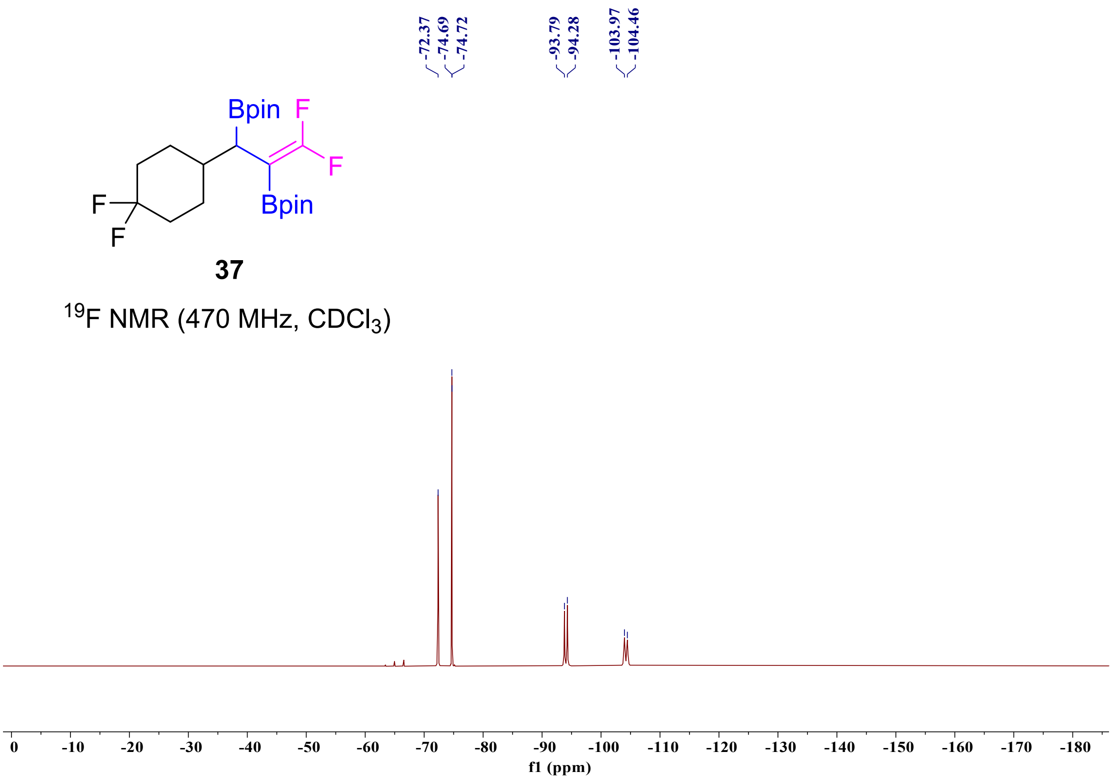

2,2'-(1-(4,4-difluorocyclohexyl)-3,3-difluoroprop-2-ene-1,2-diyl)bis(4,4,5,5-tetramethyl-1,3,2-dioxaborolane) (37)

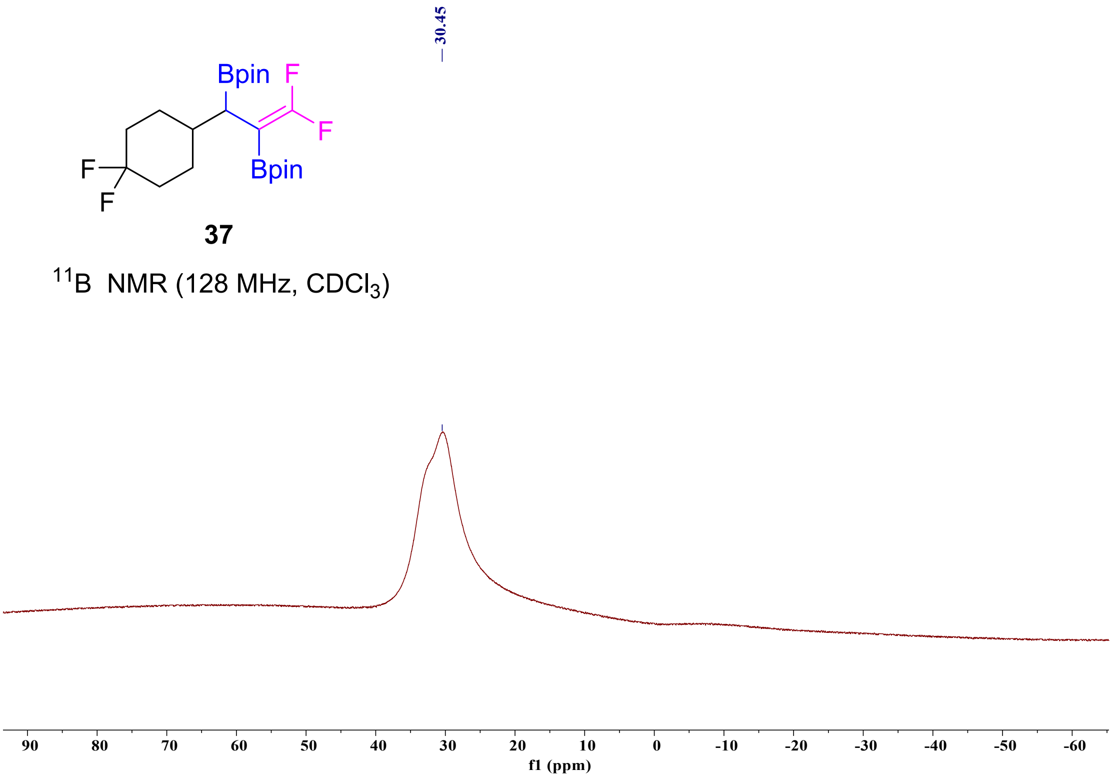

2,2'-(4-((3r,5r,7r)-adamantan-1-yl)-1,1-difluorobut-1-ene-2,3-diyl)bis(4,4,5,5-tetramethyl-1,3,2-dioxaborolane) (38)

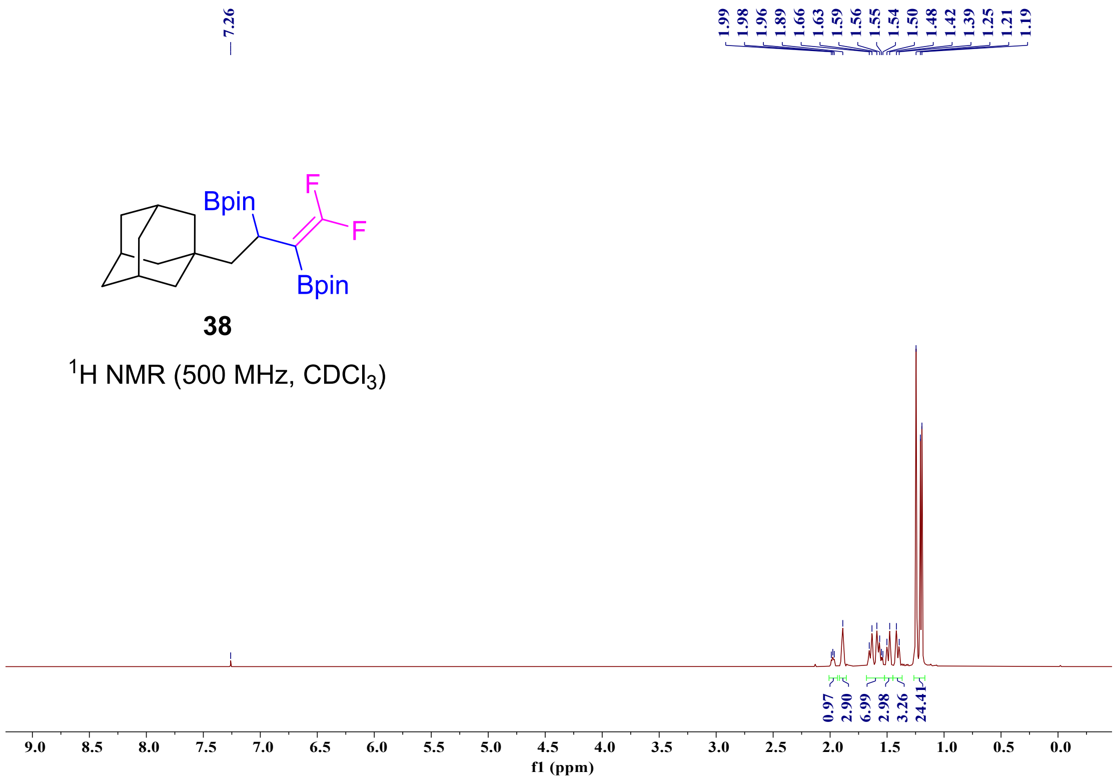

2,2'-(4-((3r,5r,7r)-adamantan-1-yl)-1,1-difluorobut-1-ene-2,3-diyl)bis(4,4,5,5-tetramethyl-1,3,2-dioxaborolane) (38)

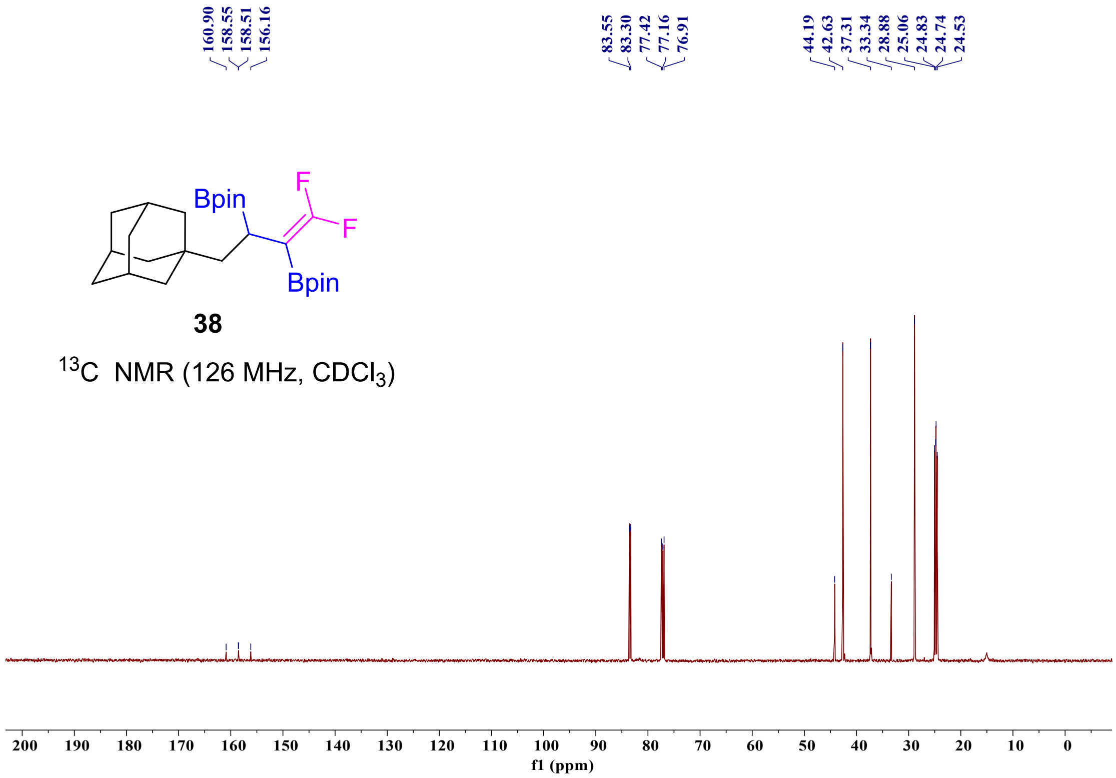

2,2'-(4-((3r,5r,7r)-adamantan-1-yl)-1,1-difluorobut-1-ene-2,3-diyl)bis(4,4,5,5-tetramethyl-1,3,2-dioxaborolane) (38)

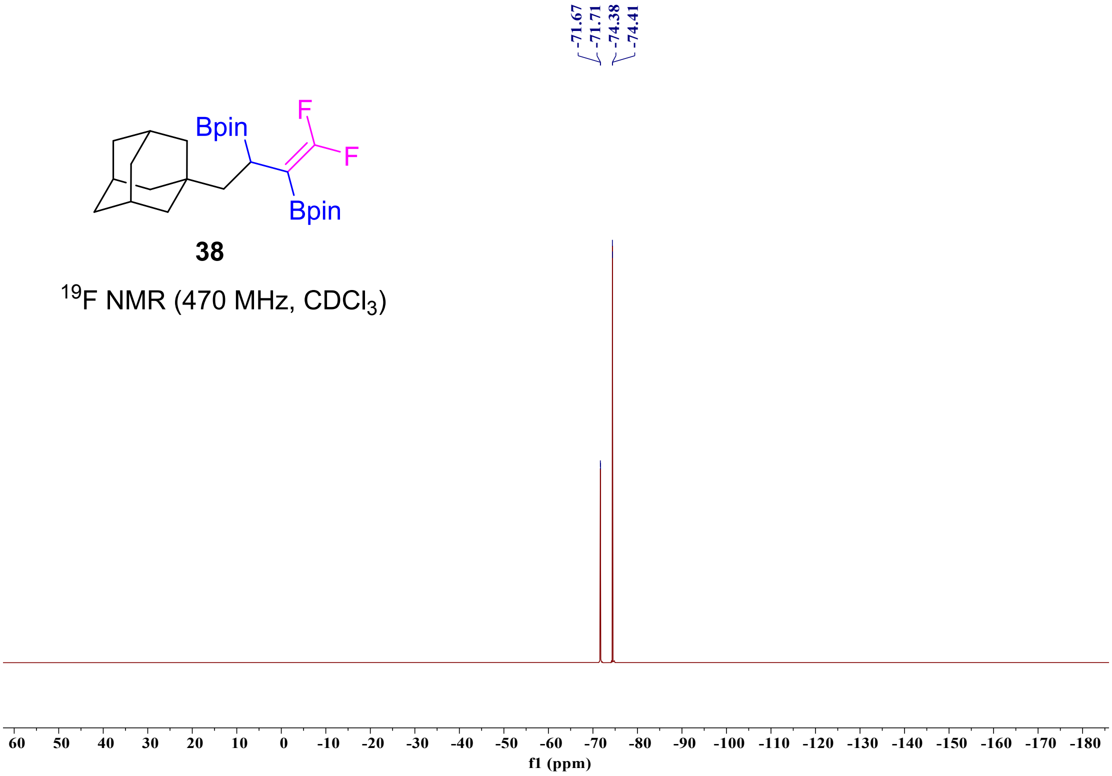

2,2'-(4-((3r,5r,7r)-adamantan-1-yl)-1,1-difluorobut-1-ene-2,3-diyl)bis(4,4,5,5-tetramethyl-1,3,2-dioxaborolane) (38)

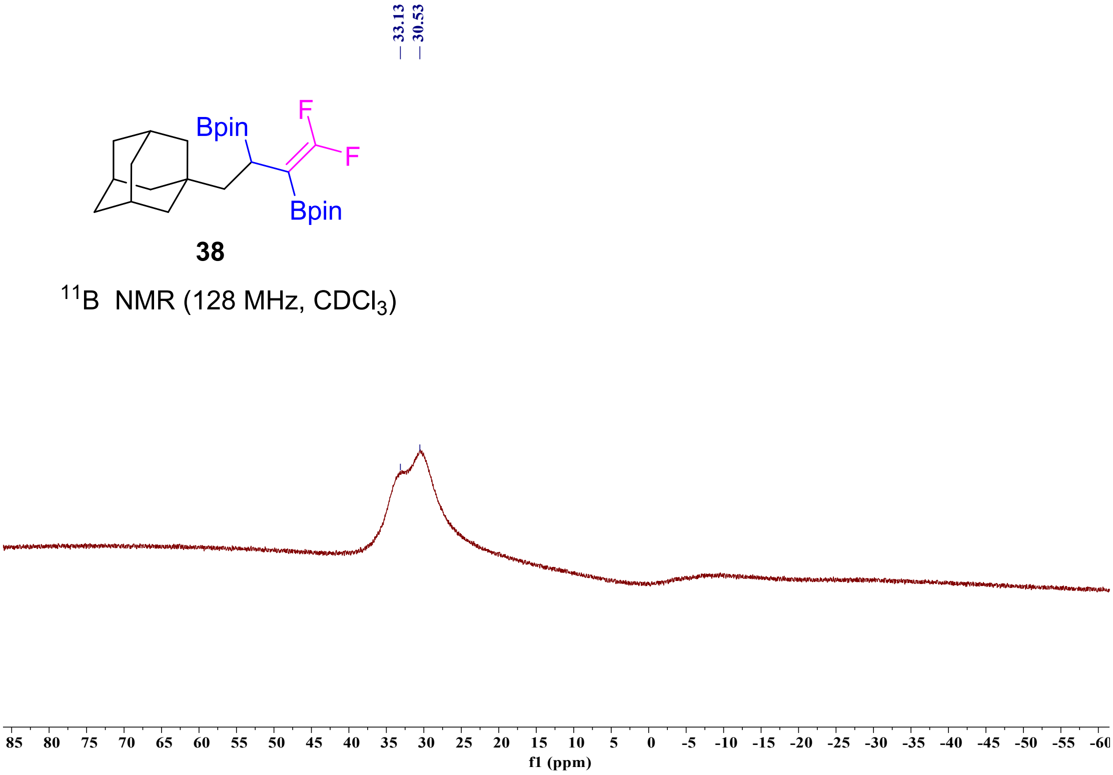

2,2'-((4R)-1,1-difluoro-4-(6-methoxynaphthalen-2-yl)pent-1-ene-2,3-diyl)bis(4,4,5,5-tetramethyl-1,3,2-dioxaborolane) (39)

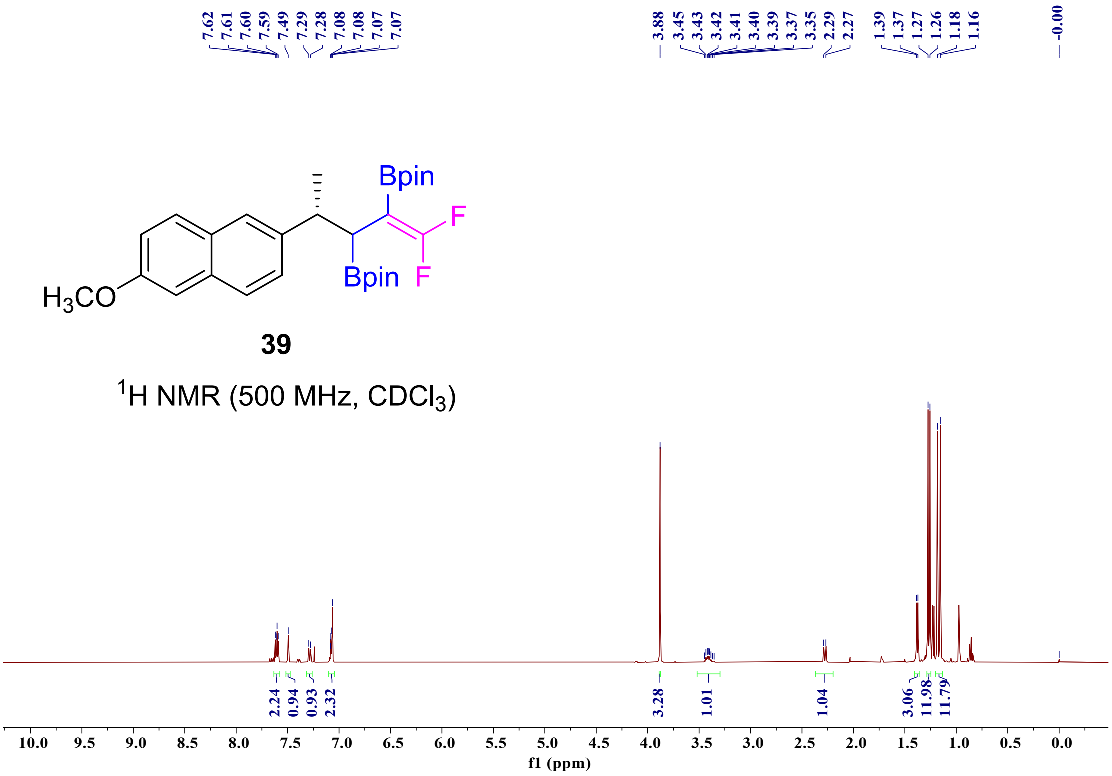

2,2'-((4R)-1,1-difluoro-4-(6-methoxynaphthalen-2-yl)pent-1-ene-2,3-diyl)bis(4,4,5,5-tetramethyl-1,3,2-dioxaborolane) (39)

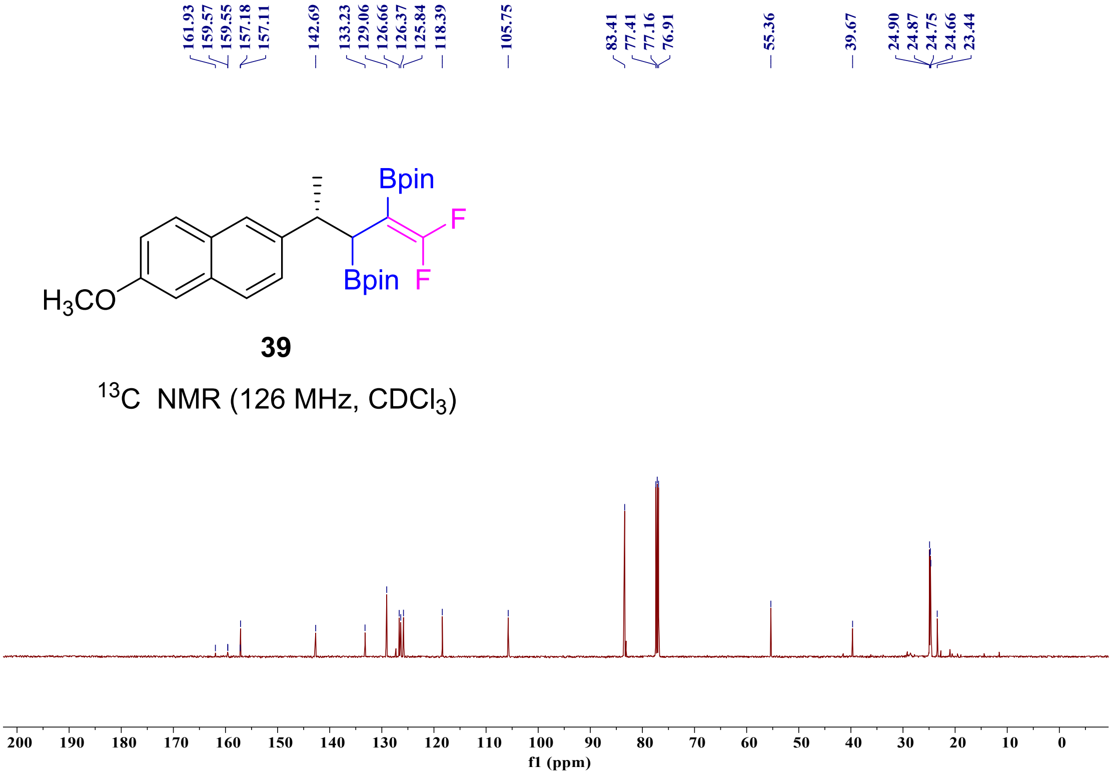

2,2'-((4R)-1,1-difluoro-4-(6-methoxynaphthalen-2-yl)pent-1-ene-2,3-diyl)bis(4,4,5,5-tetramethyl-1,3,2-dioxaborolane) (39)

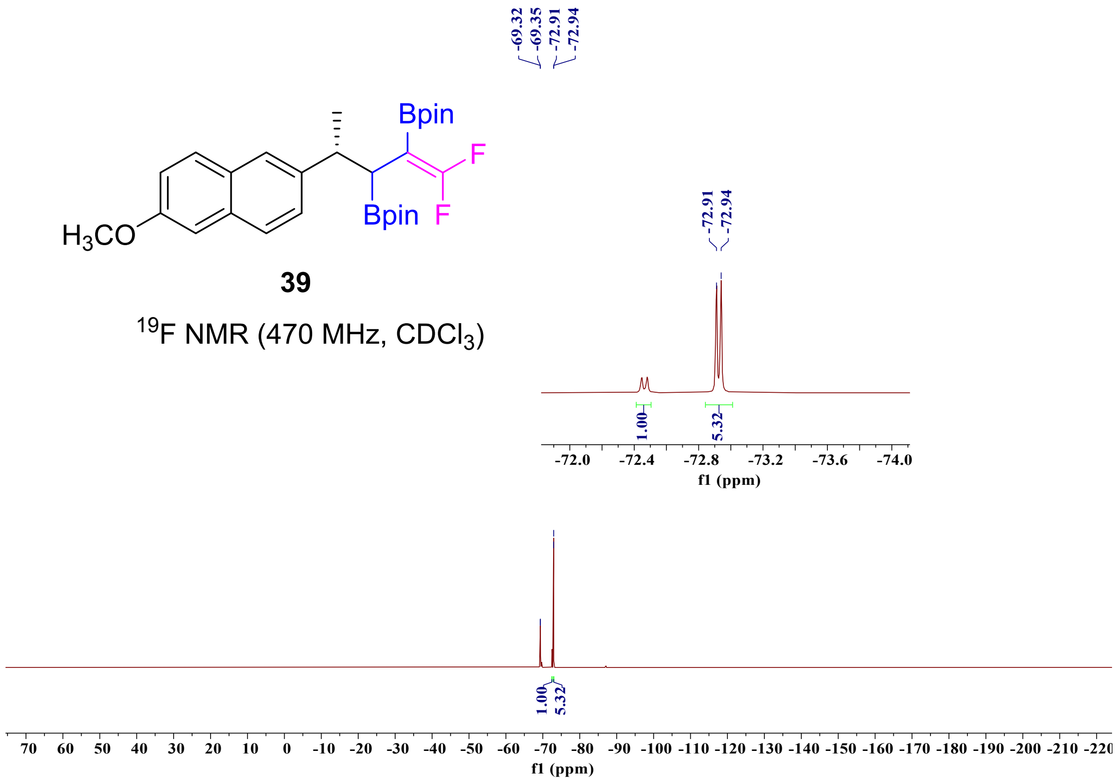

2,2'-((4R)-1,1-difluoro-4-(6-methoxynaphthalen-2-yl)pent-1-ene-2,3-diyl)bis(4,4,5,5-tetramethyl-1,3,2-dioxaborolane) (39)

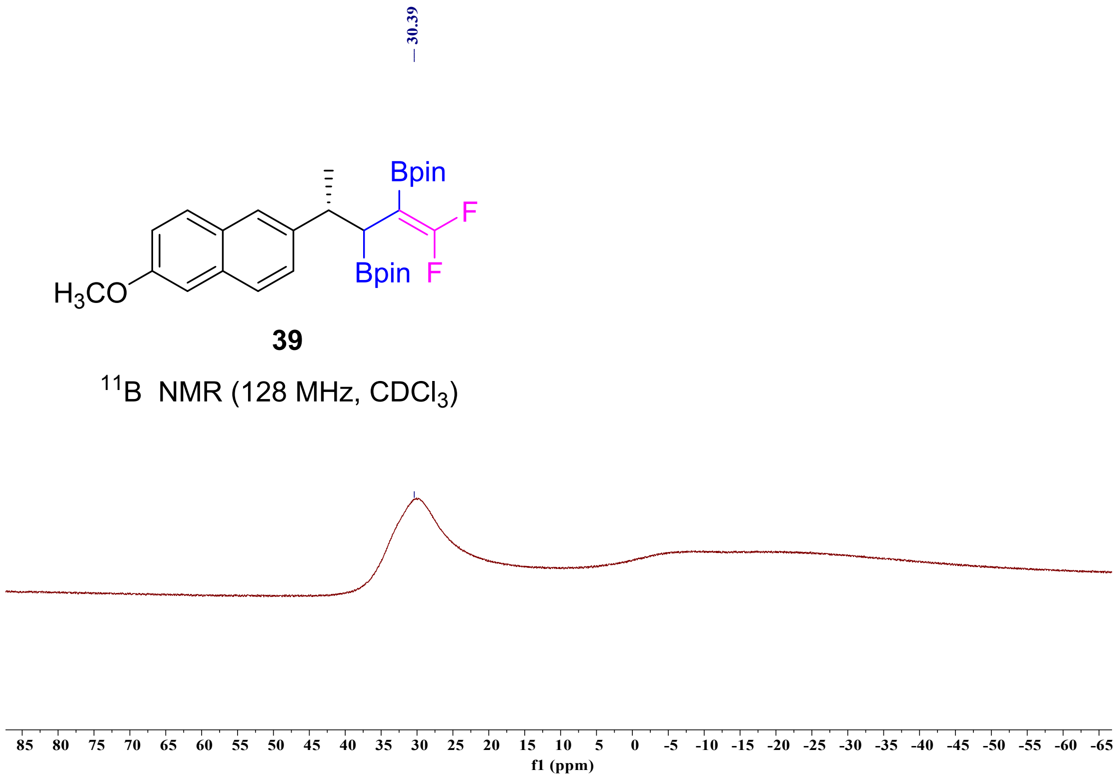

2,2'-(1,1-difluoro-4-(4-isobutylphenyl)pent-1-ene-2,3-diyl)bis(4,4,5,5-tetramethyl-1,3,2-dioxaborolane) (40)

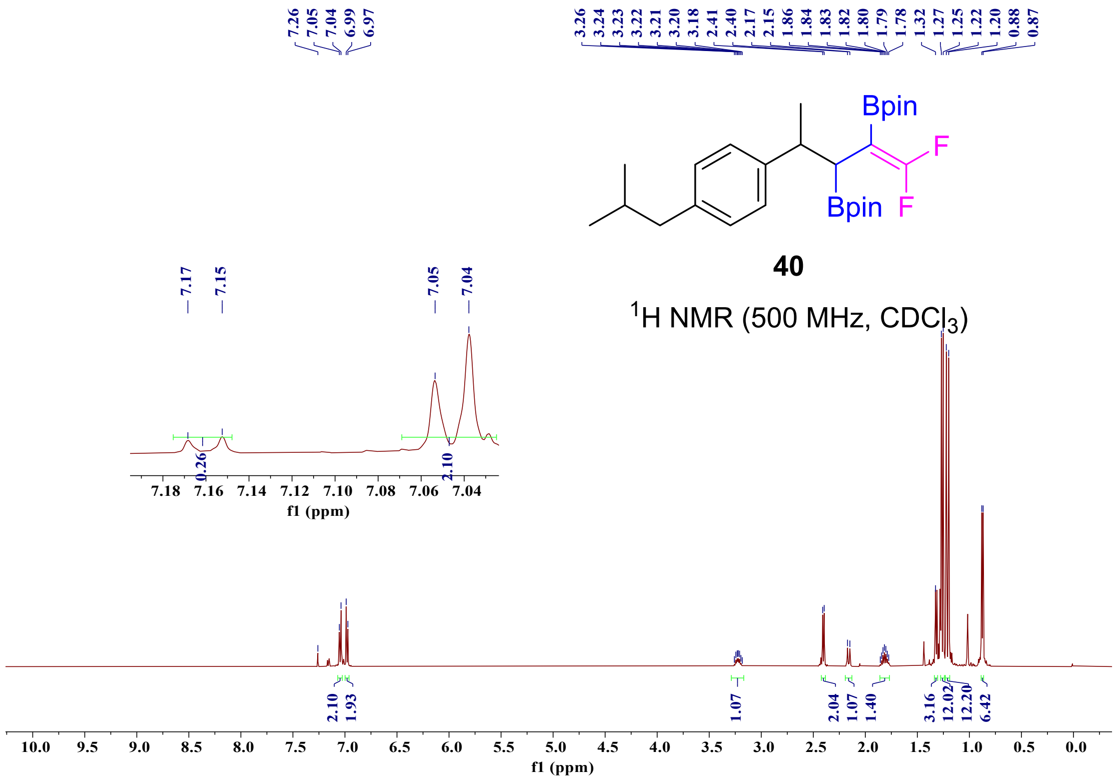

2,2'-(1,1-difluoro-4-(4-isobutylphenyl)pent-1-ene-2,3-diyl)bis(4,4,5,5-tetramethyl-1,3,2-dioxaborolane) (40)

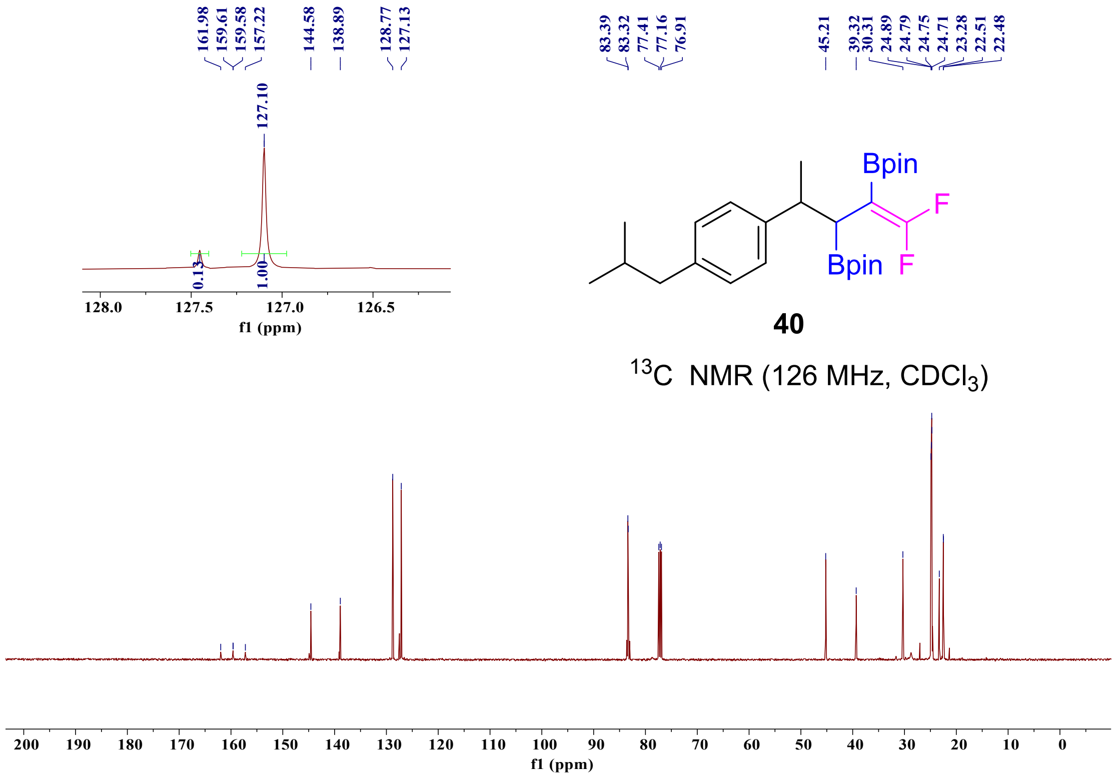

2,2'-(1,1-difluoro-4-(4-isobutylphenyl)pent-1-ene-2,3-diyl)bis(4,4,5,5-tetramethyl-1,3,2-dioxaborolane) (40)

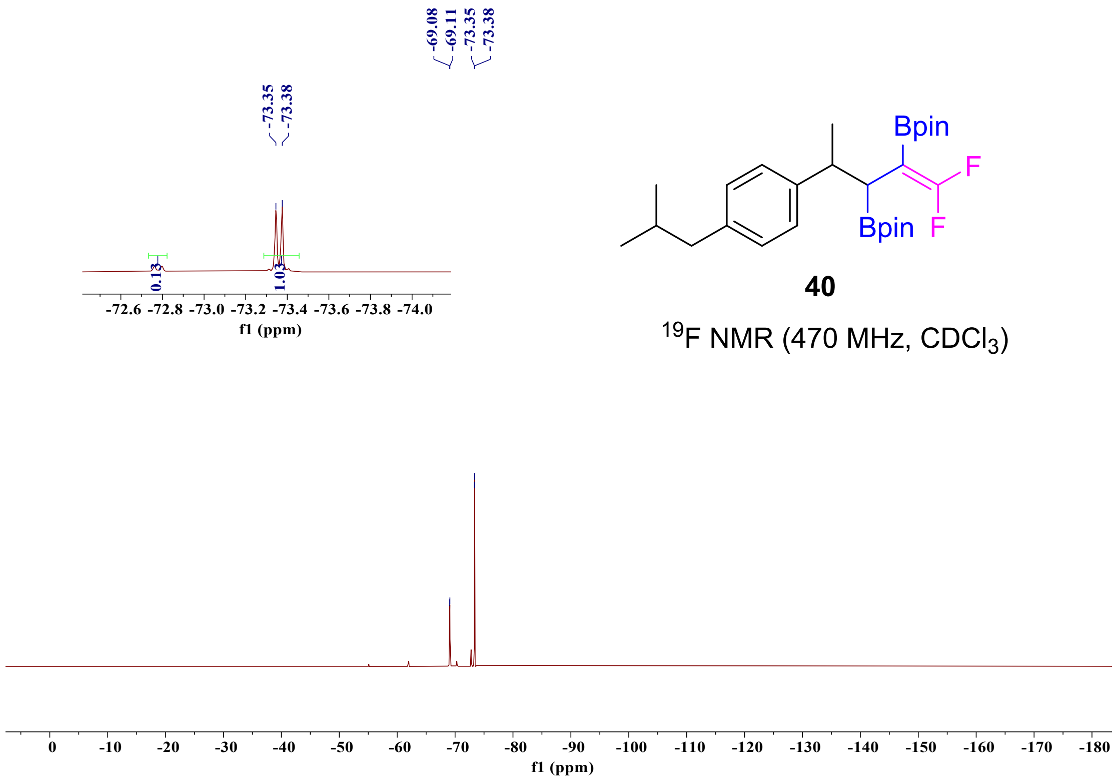

2,2'-(1,1-difluoro-4-(4-isobutylphenyl)pent-1-ene-2,3-diyl)bis(4,4,5,5-tetramethyl-1,3,2-dioxaborolane) (40)

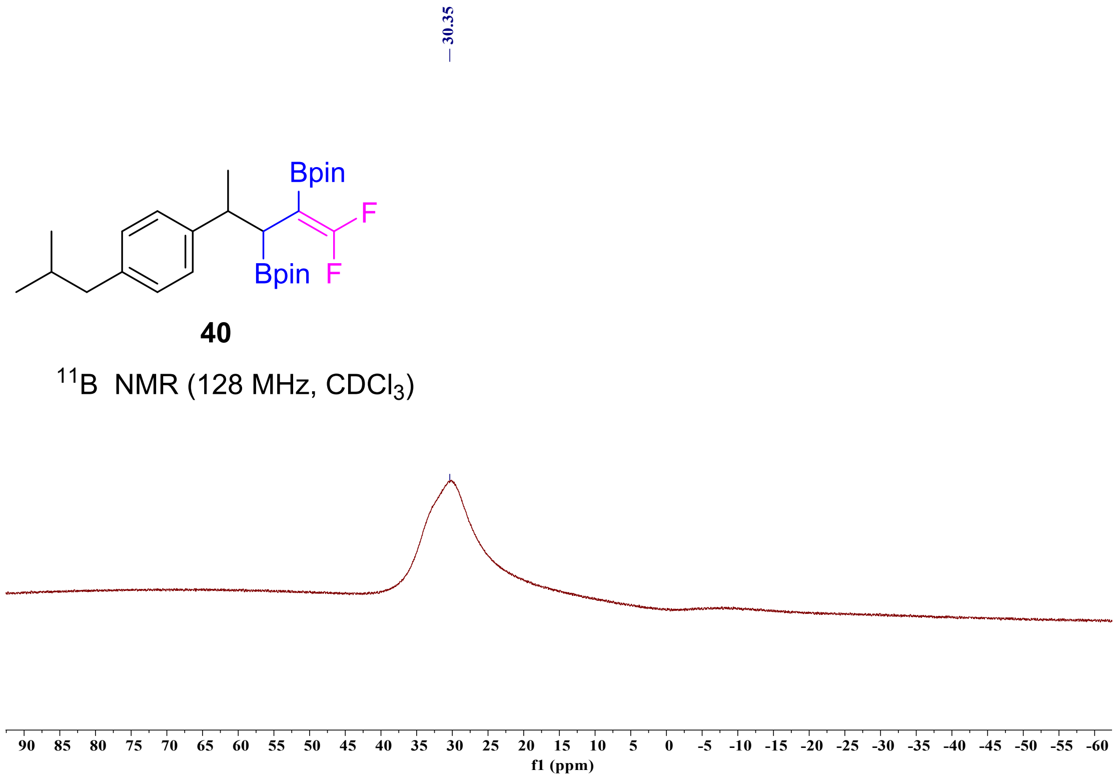

2,2'-(1,1-difluoro-4-(2-fluoro-[1,1'-biphenyl]-4-yl)pent-1-ene-2,3-diyl)bis(4,4,5,5-tetramethyl-1,3,2-dioxaborolane) (41)

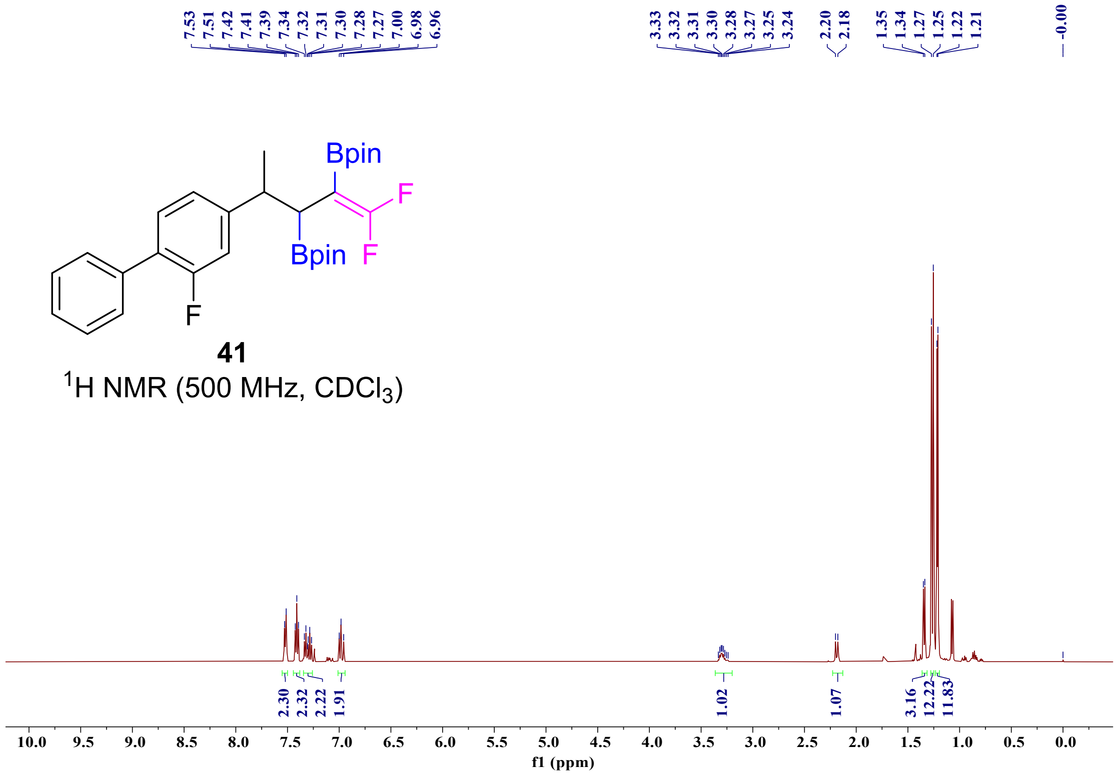

2,2'-(1,1-difluoro-4-(2-fluoro-[1,1'-biphenyl]-4-yl)pent-1-ene-2,3-diyl)bis(4,4,5,5-tetramethyl-1,3,2-dioxaborolane) (41)

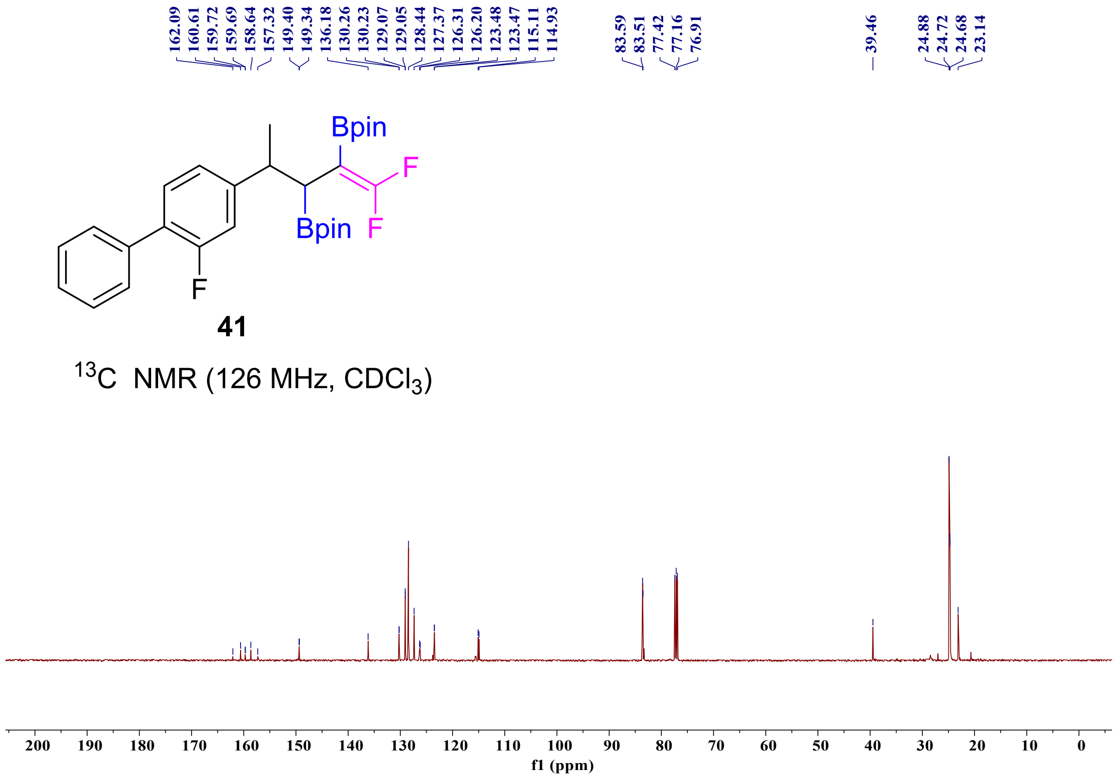

2,2'-(1,1-difluoro-4-(2-fluoro-[1,1'-biphenyl]-4-yl)pent-1-ene-2,3-diyl)bis(4,4,5,5-tetramethyl-1,3,2-dioxaborolane) (41)

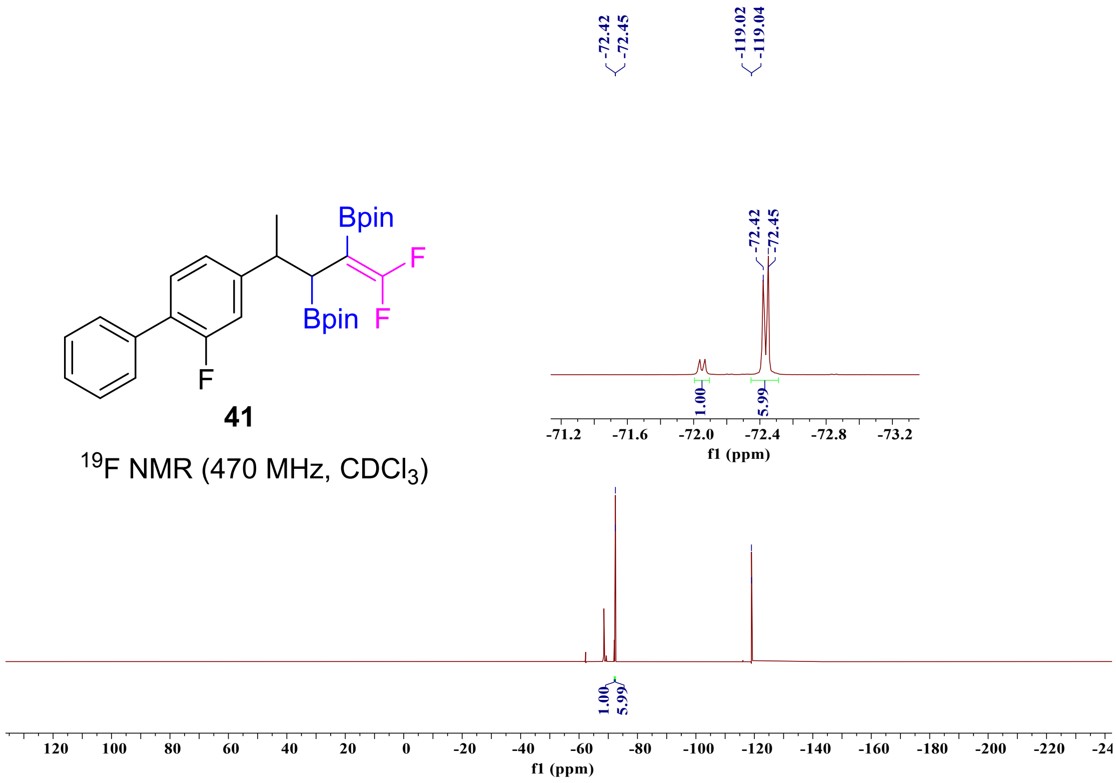

2,2'-(1,1-difluoro-4-(2-fluoro-[1,1'-biphenyl]-4-yl)pent-1-ene-2,3-diyl)bis(4,4,5,5-tetramethyl-1,3,2-dioxaborolane) (41)

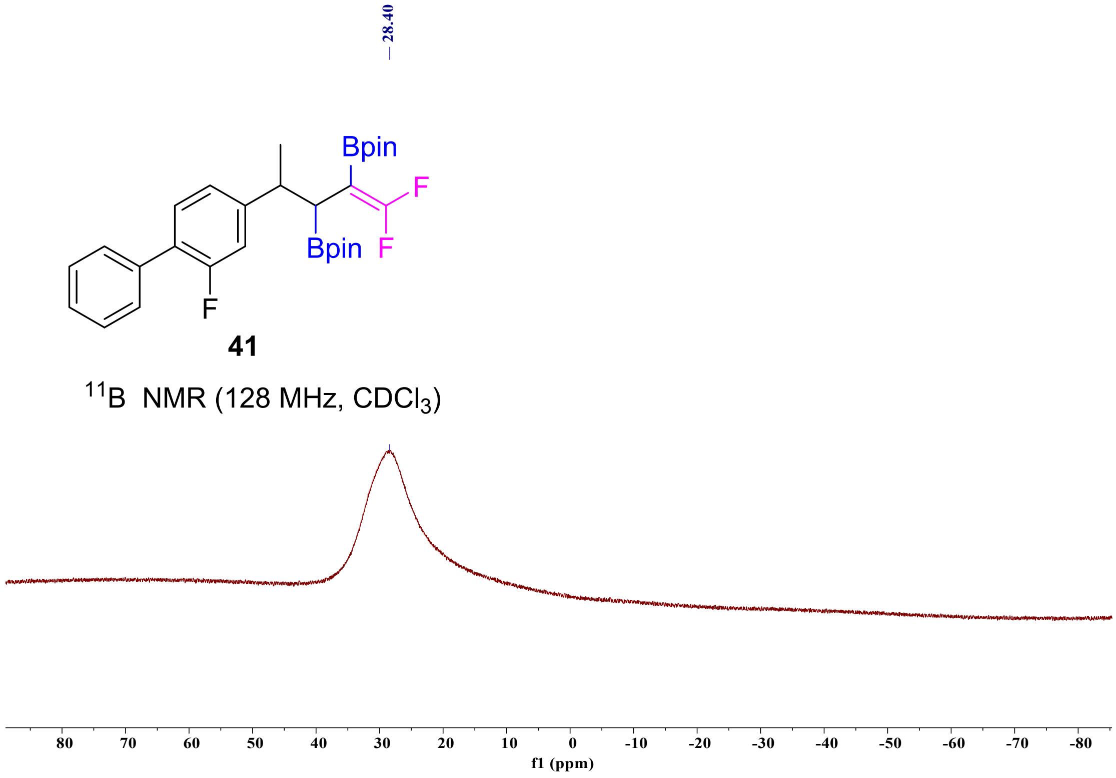

(4-chlorophenyl)(3-(4,4-difluoro-2,3-bis(4,4,5,5-tetramethyl-1,3,2-dioxaborolan-2-yl)but-3-en-1-yl)-2-methyl-1H-indol-1-yl)methanone (42)

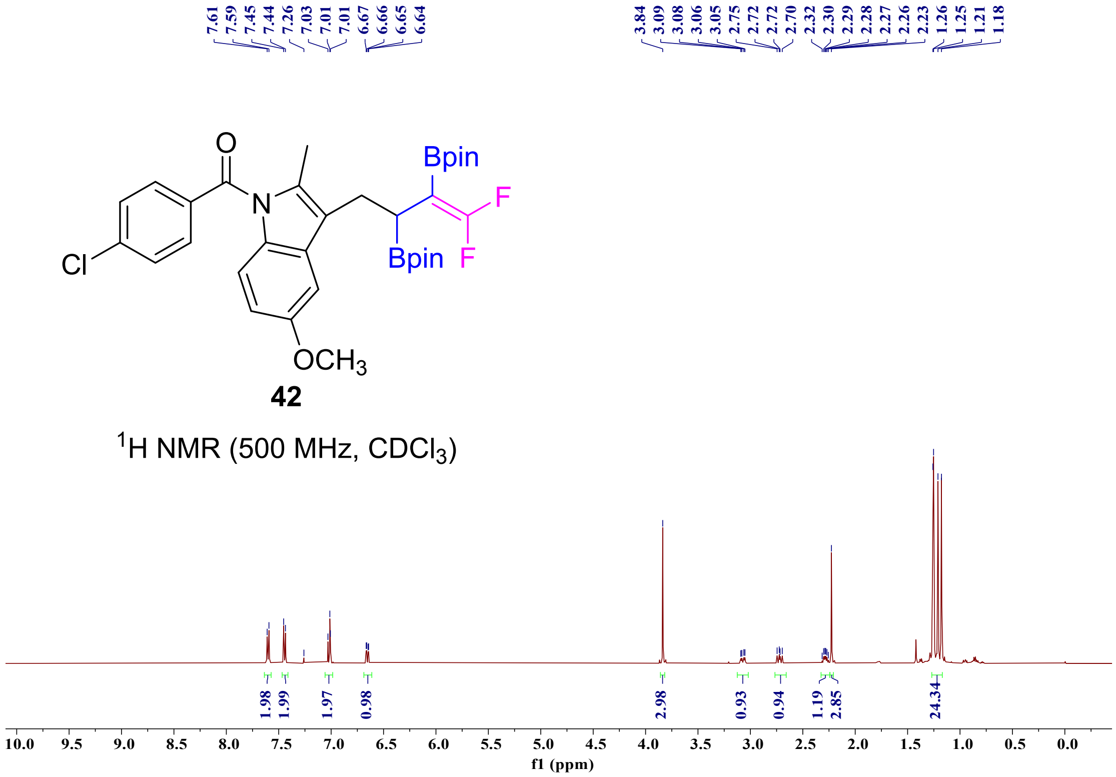

(4-chlorophenyl)(3-(4,4-difluoro-2,3-bis(4,4,5,5-tetramethyl-1,3,2-dioxaborolan-2-yl)but-3-en-1-yl)-2-methyl-1H-indol-1-yl)methanone (42)

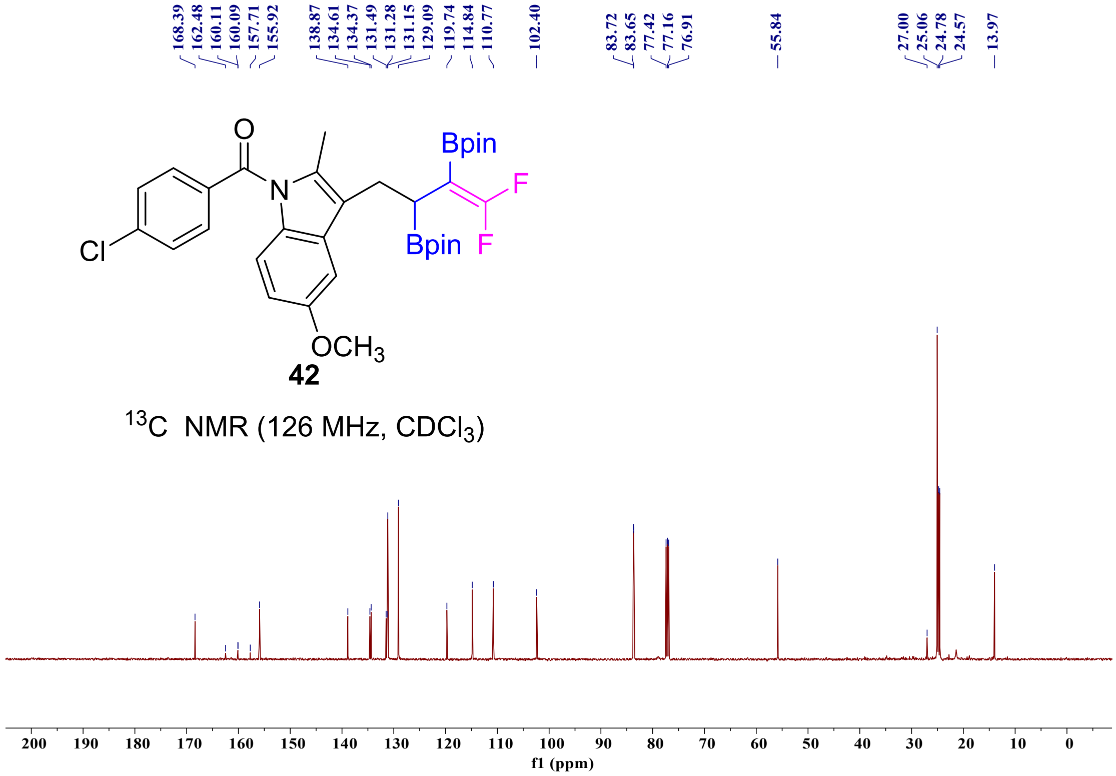

(4-chlorophenyl)(3-(4,4-difluoro-2,3-bis(4,4,5,5-tetramethyl-1,3,2-dioxaborolan-2-yl)but-3-en-1-yl)-2-methyl-1H-indol-1-yl)methanone (42)

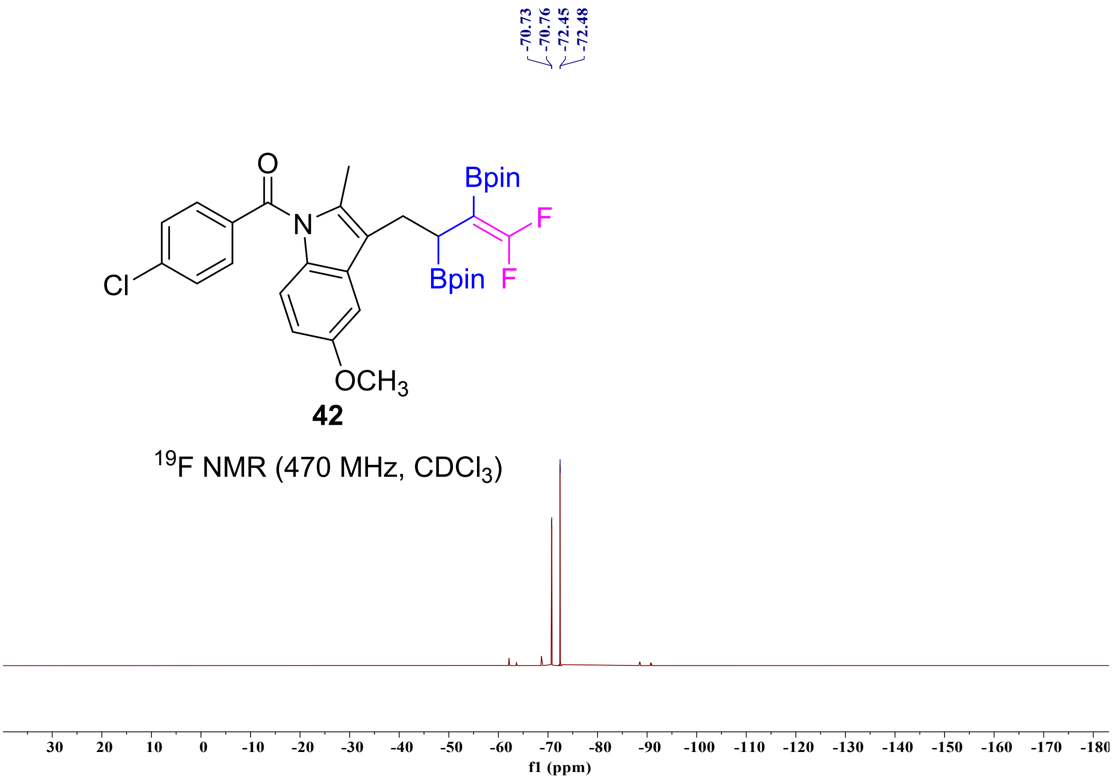

(4-chlorophenyl)(3-(4,4-difluoro-2,3-bis(4,4,5,5-tetramethyl-1,3,2-dioxaborolan-2-yl)but-3-en-1-yl)-2-methyl-1H-indol-1-yl)methanone (42)

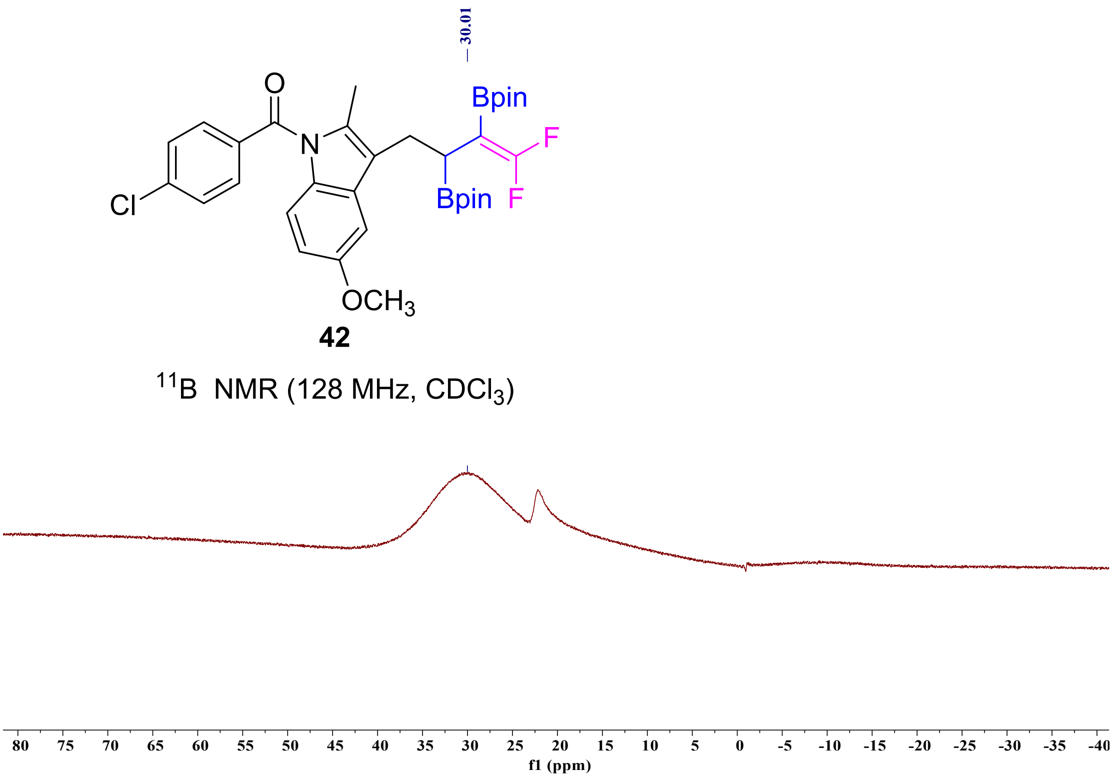

2,2'-(3,3-difluoro-1-phenylprop-2-ene-1,2-diyl)bis(4,4,5,5-tetramethyl-1,3,2-dioxaborolane) (43)

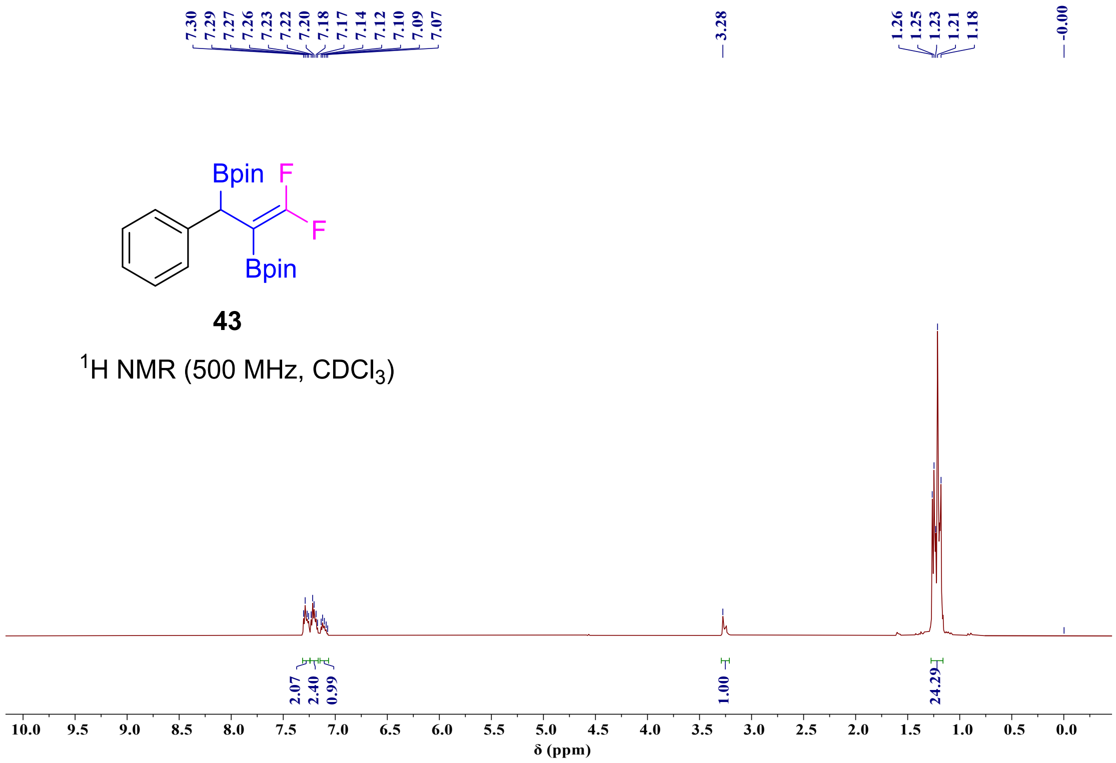

2,2'-(3,3-difluoro-1-phenylprop-2-ene-1,2-diyl)bis(4,4,5,5-tetramethyl-1,3,2-dioxaborolane) (43)

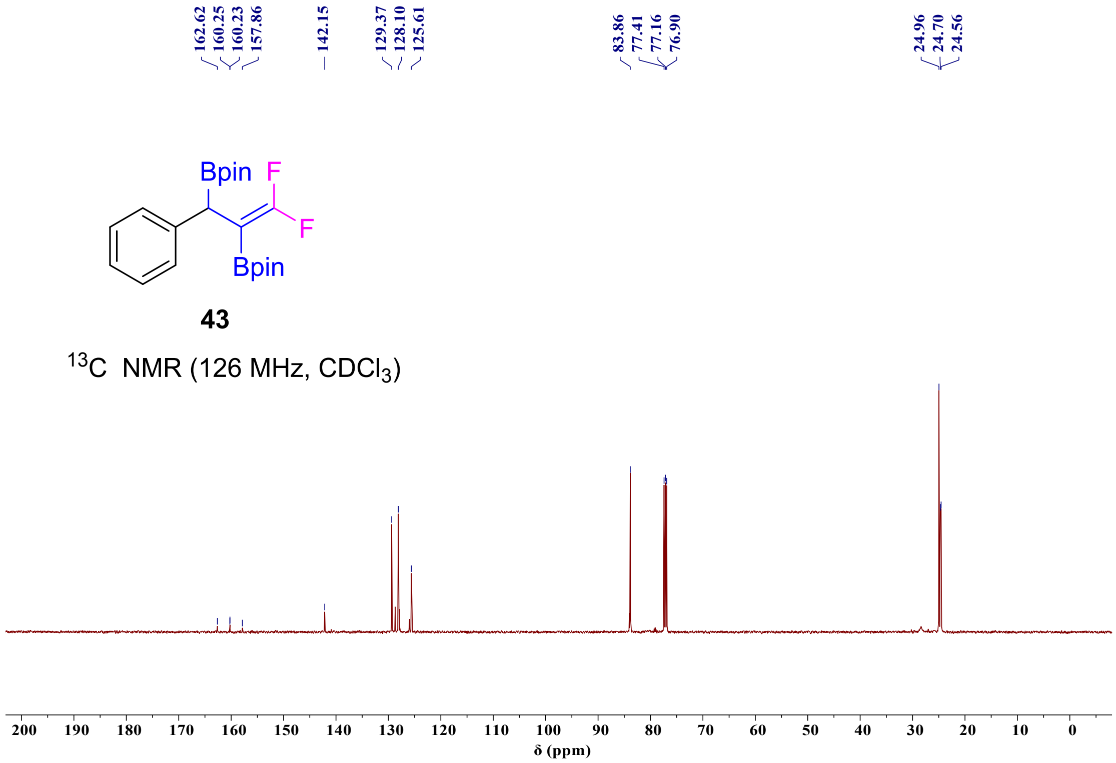

2,2'-(3,3-difluoro-1-phenylprop-2-ene-1,2-diyl)bis(4,4,5,5-tetramethyl-1,3,2-dioxaborolane) (43)

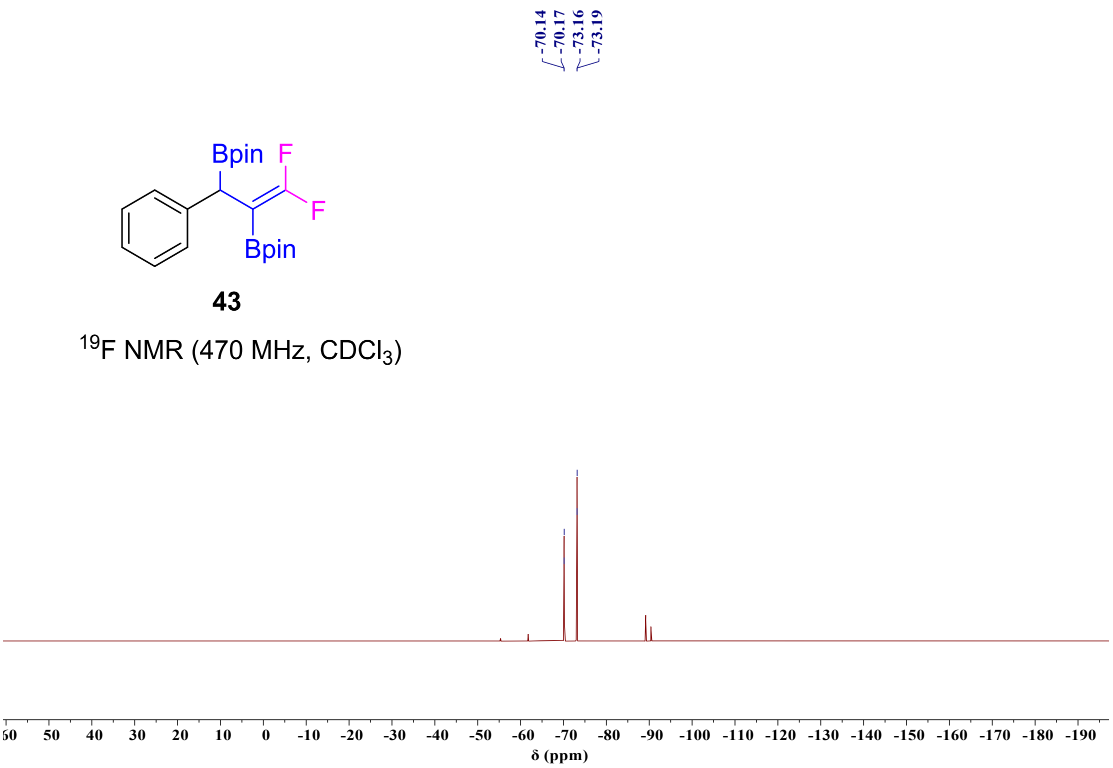

2,2'-(3,3-difluoro-1-phenylprop-2-ene-1,2-diyl)bis(4,4,5,5-tetramethyl-1,3,2-dioxaborolane) (43)

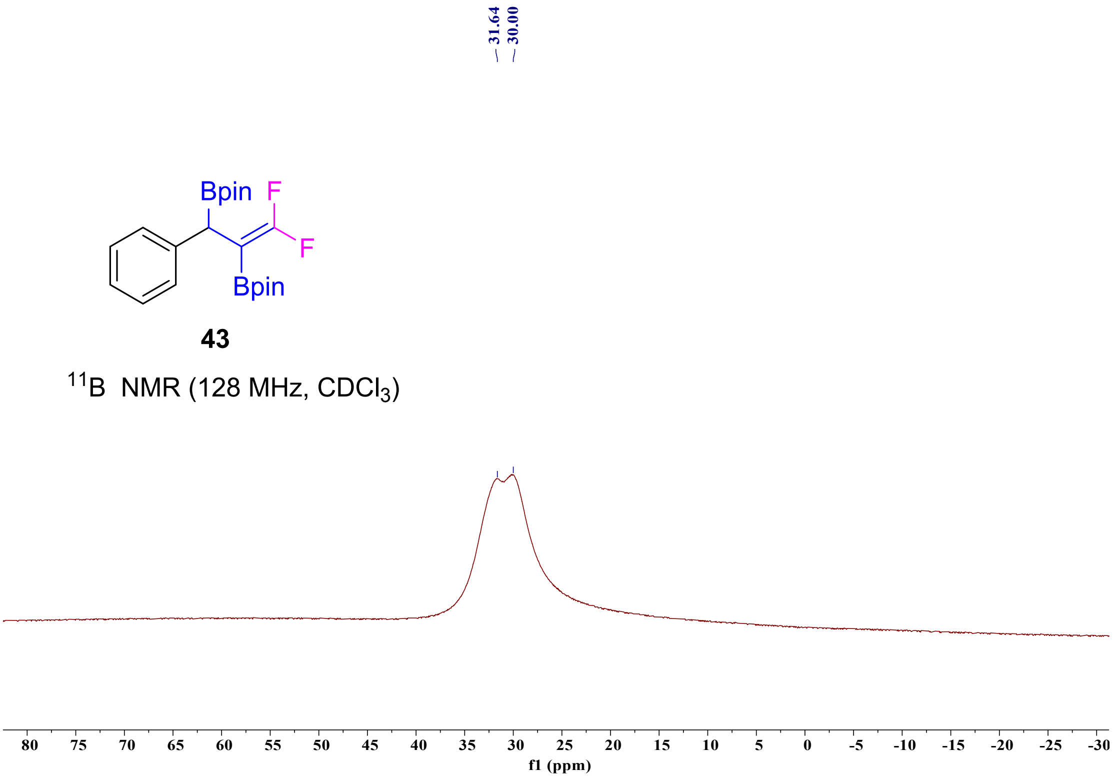

2,2'-(3,3-difluoro-1-(p-tolyl)prop-2-ene-1,2-diyl)bis(4,4,5,5-tetramethyl-1,3,2-dioxaborolane) (44)

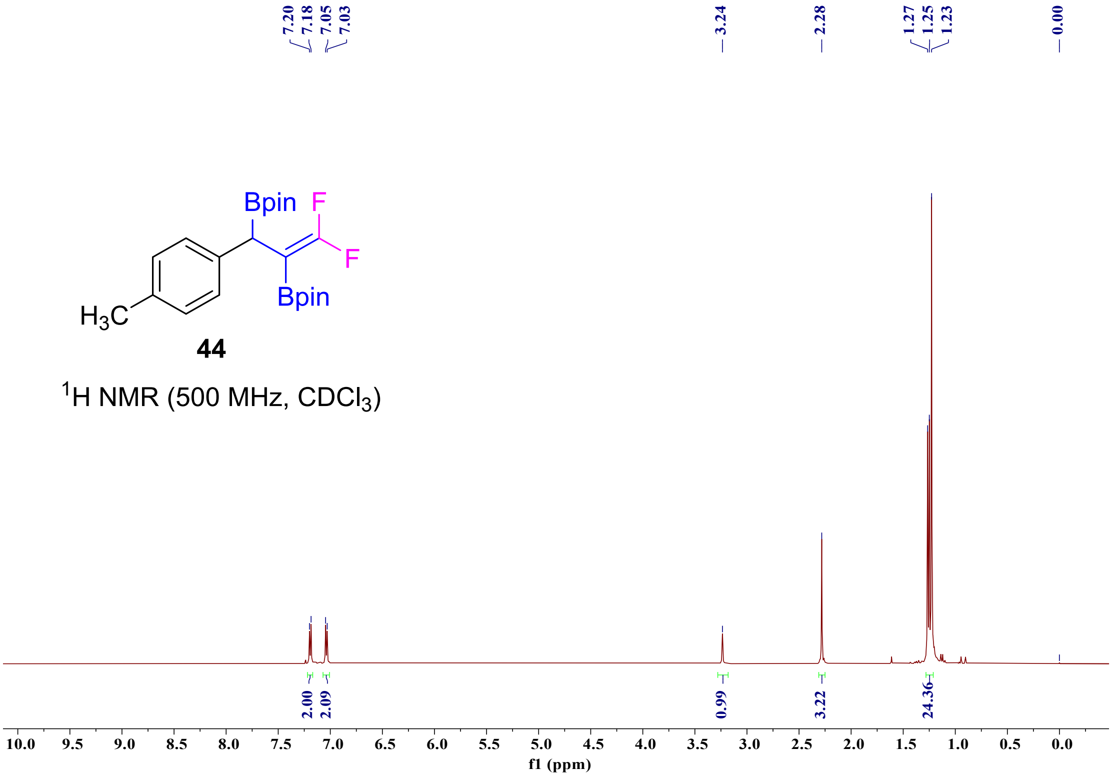

2,2'-(3,3-difluoro-1-(p-tolyl)prop-2-ene-1,2-diyl)bis(4,4,5,5-tetramethyl-1,3,2-dioxaborolane) (44)

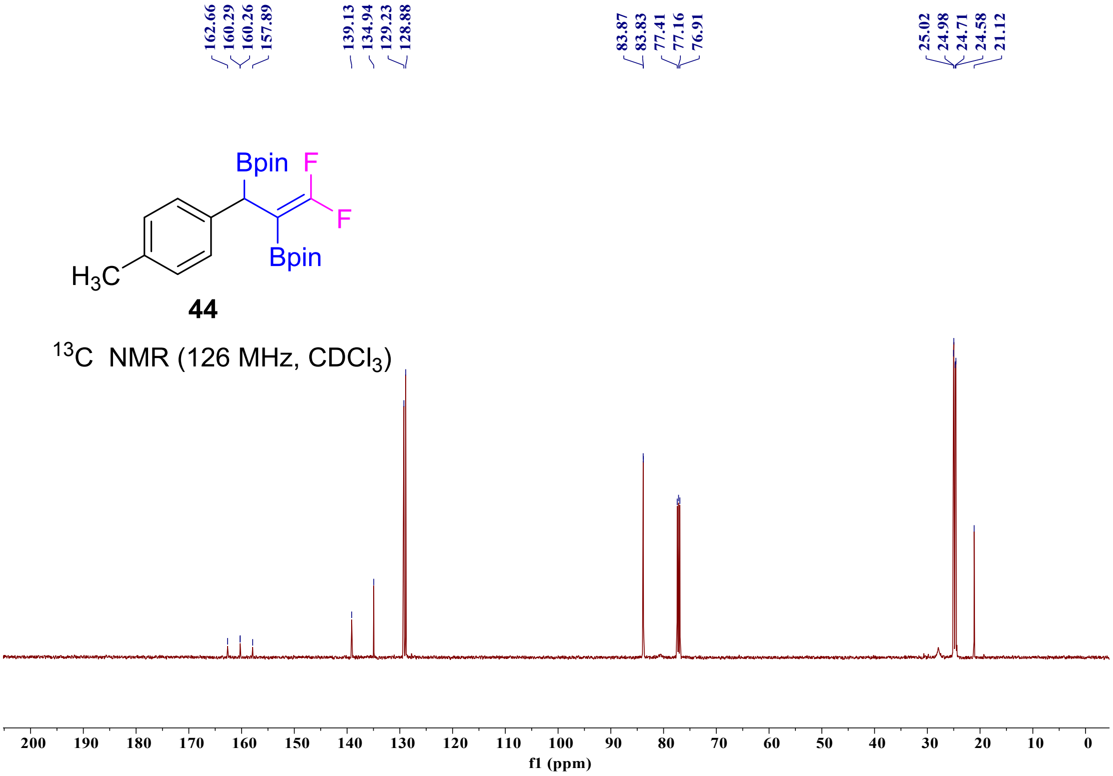

2,2'-(3,3-difluoro-1-(p-tolyl)prop-2-ene-1,2-diyl)bis(4,4,5,5-tetramethyl-1,3,2-dioxaborolane) (44)

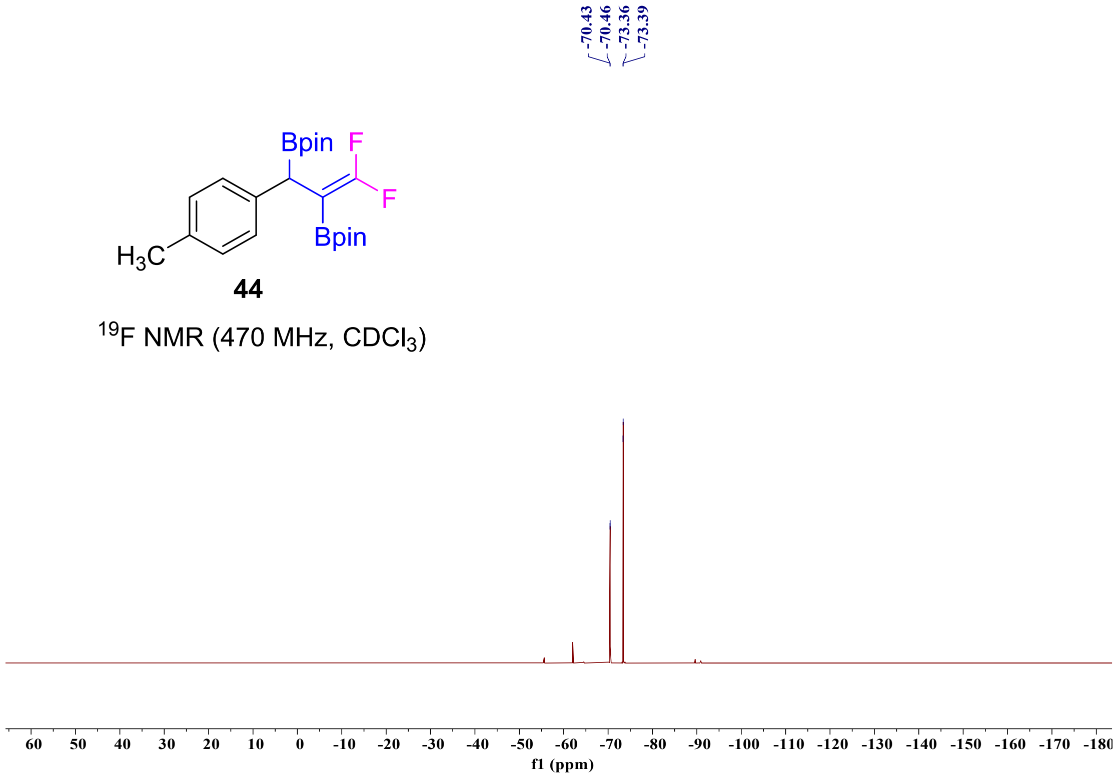

2,2'-(3,3-difluoro-1-(p-tolyl)prop-2-ene-1,2-diyl)bis(4,4,5,5-tetramethyl-1,3,2-dioxaborolane) (44)

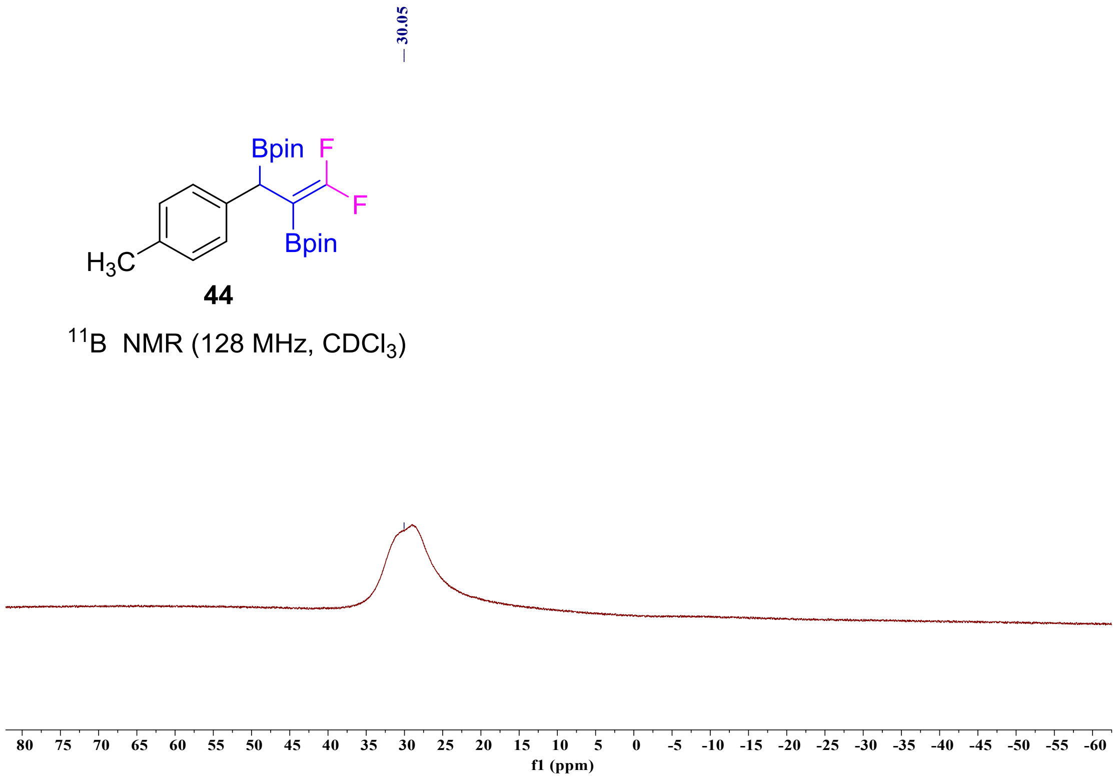

2,2'-(1-(4-ethylphenyl)-3,3-difluoroprop-2-ene-1,2-diyl)bis(4,4,5,5-tetramethyl-1,3,2-dioxaborolane) (45)

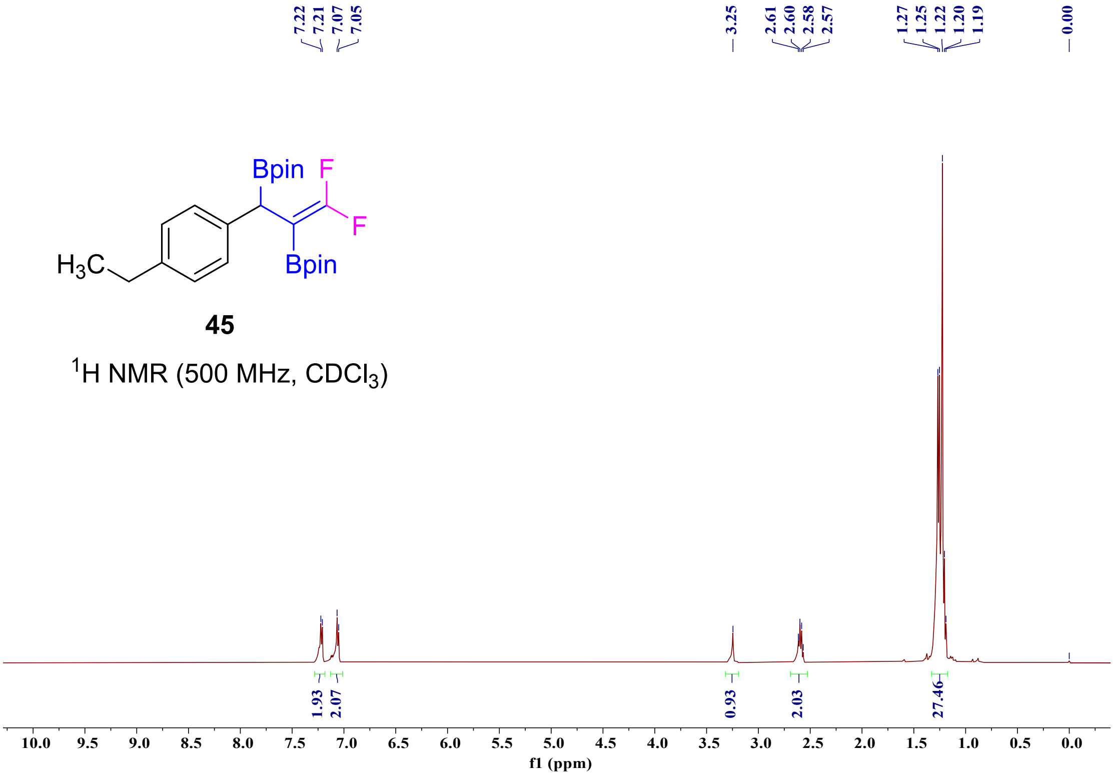

2,2'-(1-(4-ethylphenyl)-3,3-difluoroprop-2-ene-1,2-diyl)bis(4,4,5,5-tetramethyl-1,3,2-dioxaborolane) (45)

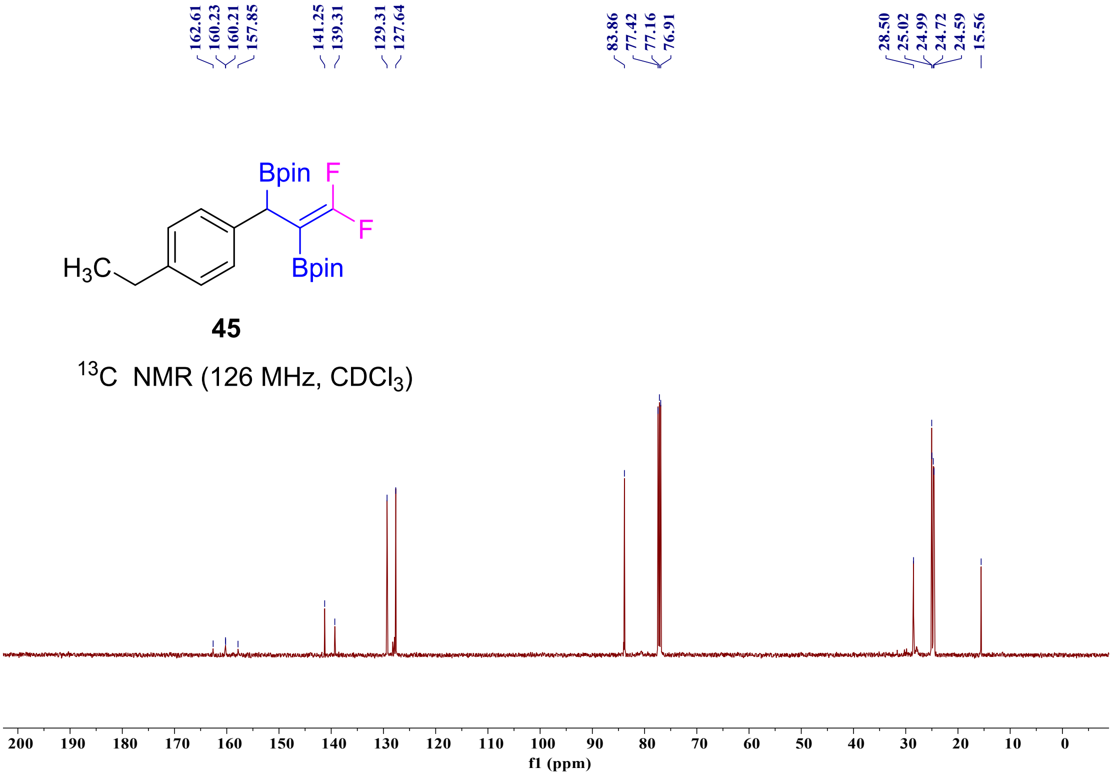

2,2'-(1-(4-ethylphenyl)-3,3-difluoroprop-2-ene-1,2-diyl)bis(4,4,5,5-tetramethyl-1,3,2-dioxaborolane) (45)

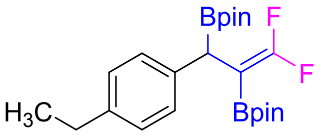

45

^{19}F NMR (470 MHz, $CDCl_3$)

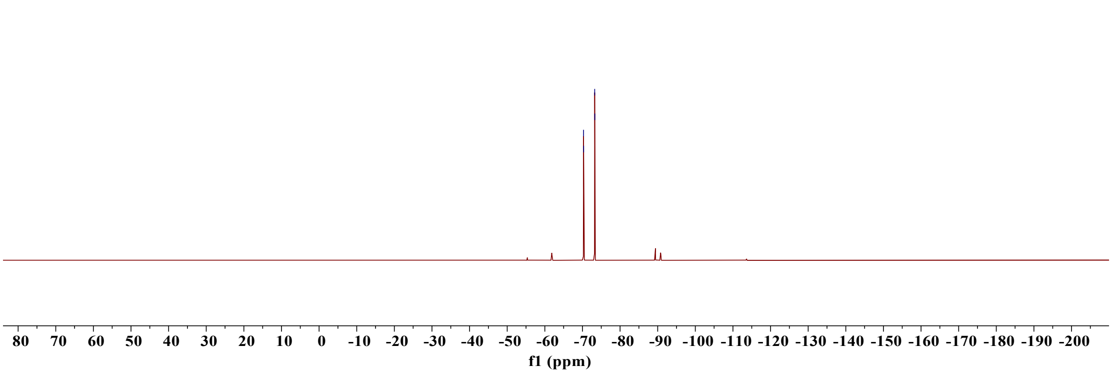

2,2'-(1-(4-ethylphenyl)-3,3-difluoroprop-2-ene-1,2-diyl)bis(4,4,5,5-tetramethyl-1,3,2-dioxaborolane) (45)

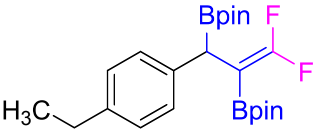

45

^{11}B NMR (128 MHz, $CDCl_3$)

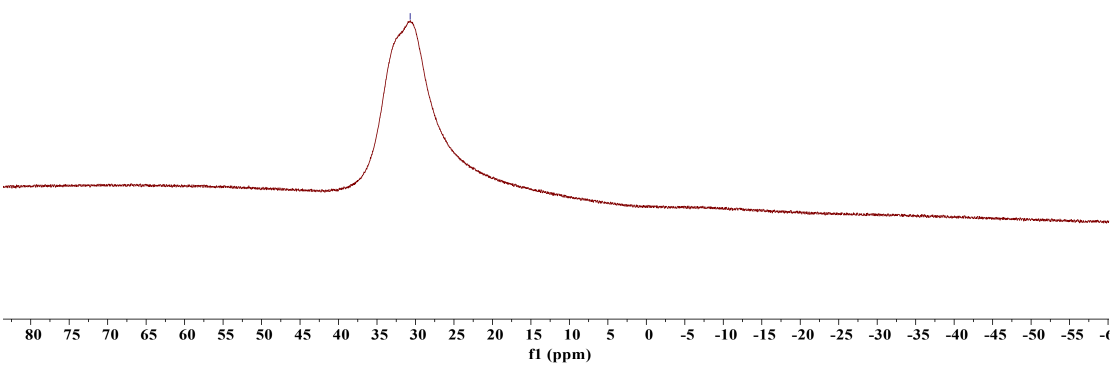

2,2'-(3,3-difluoro-1-(4-isopropylphenyl)prop-2-ene-1,2-diyl)bis(4,4,5,5-tetramethyl-1,3,2-dioxaborolane) (46)

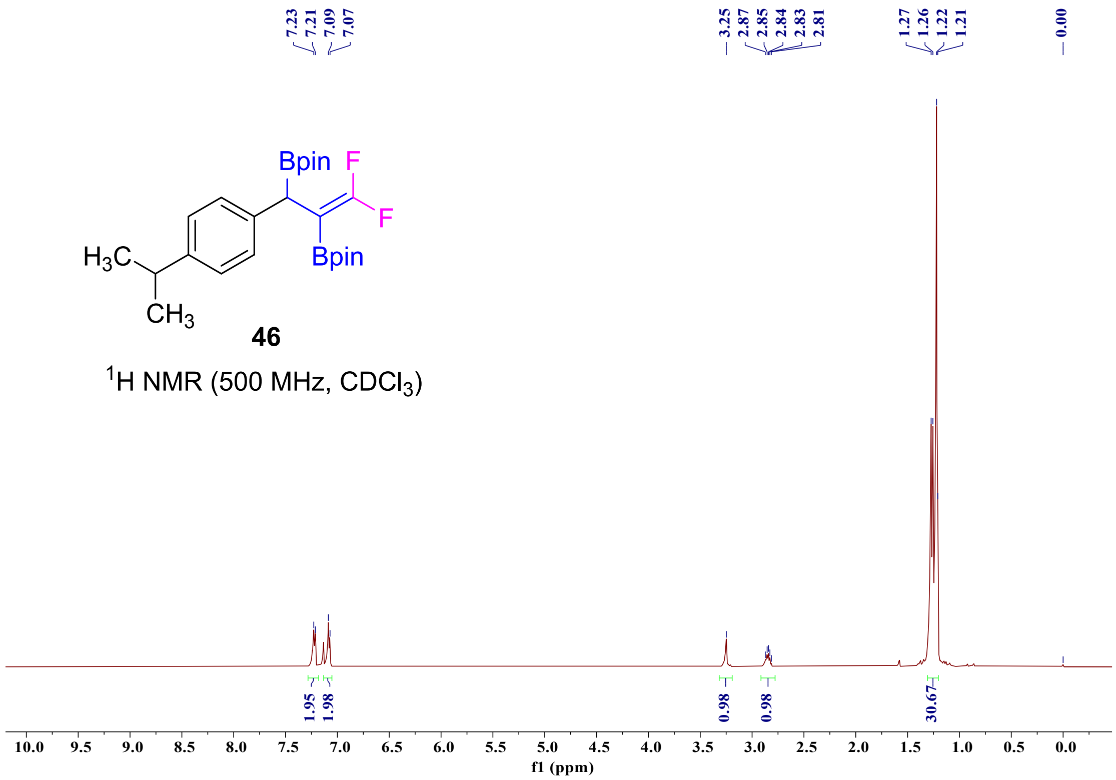

2,2'-(3,3-difluoro-1-(4-isopropylphenyl)prop-2-ene-1,2-diyl)bis(4,4,5,5-tetramethyl-1,3,2-dioxaborolane) (46)

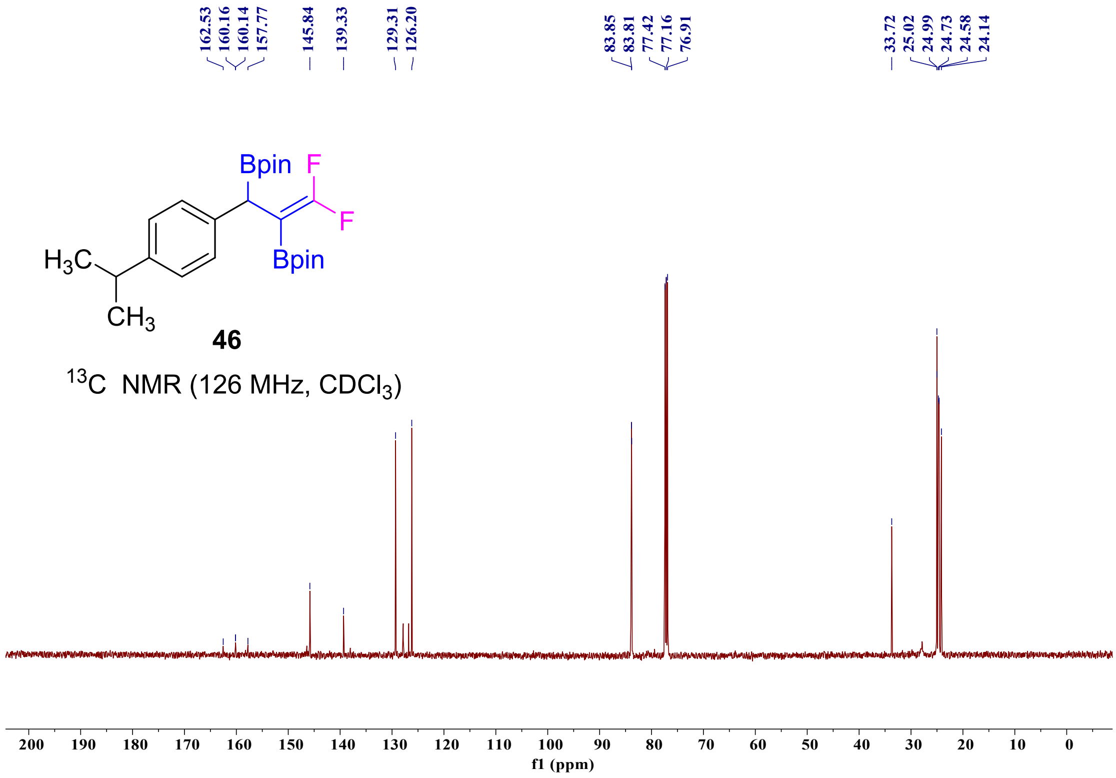

2,2'-(3,3-difluoro-1-(4-isopropylphenyl)prop-2-ene-1,2-diyl)bis(4,4,5,5-tetramethyl-1,3,2-dioxaborolane) (46)

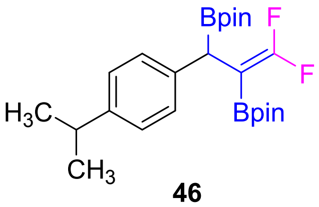

46

^{19}F NMR (470 MHz, $CDCl_3$)

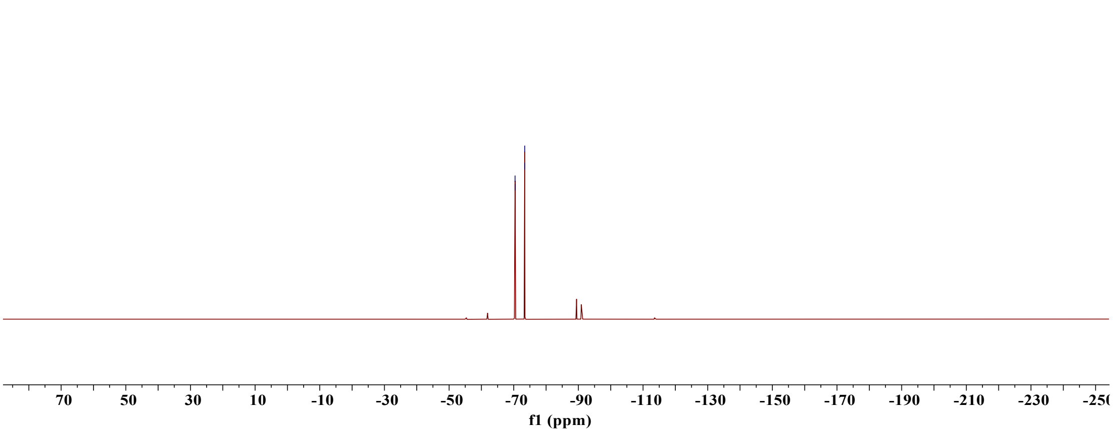

2,2'-(3,3-difluoro-1-(4-isopropylphenyl)prop-2-ene-1,2-diyl)bis(4,4,5,5-tetramethyl-1,3,2-dioxaborolane) (46)

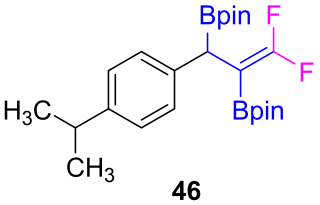

46

^{11}B NMR (128 MHz, $CDCl_3$)

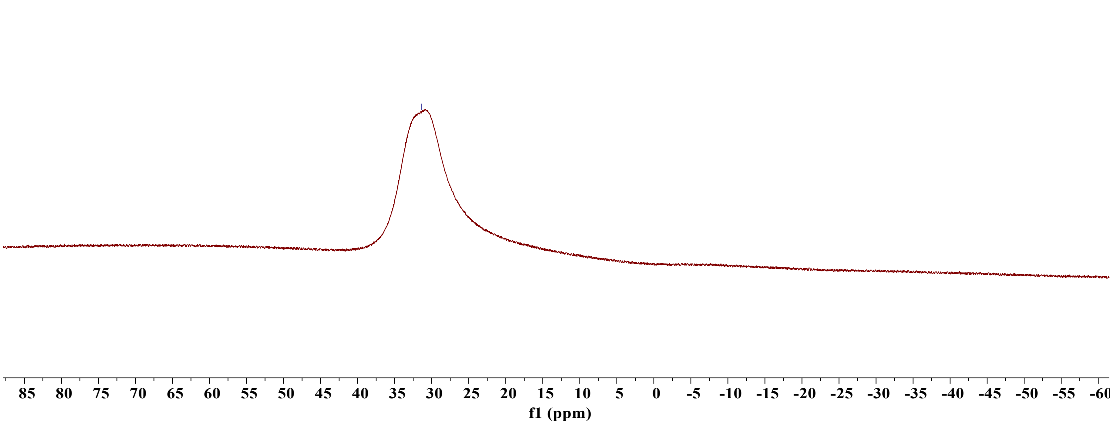

2,2'-(1-(4-(tert-butyl)phenyl)-3,3-difluoroprop-2-ene-1,2-diyl)bis(4,4,5,5-tetramethyl-1,3,2-dioxaborolane) (47)

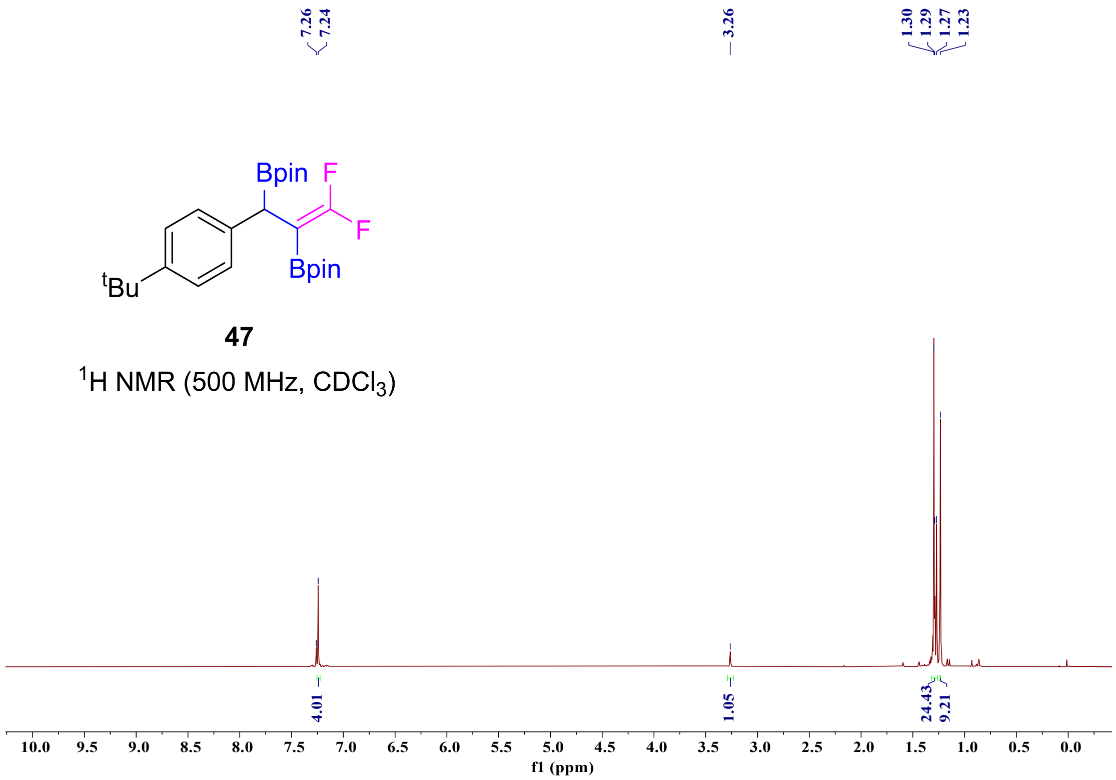

2,2'-(1-(4-(tert-butyl)phenyl)-3,3-difluoroprop-2-ene-1,2-diyl)bis(4,4,5,5-tetramethyl-1,3,2-dioxaborolane) (47)

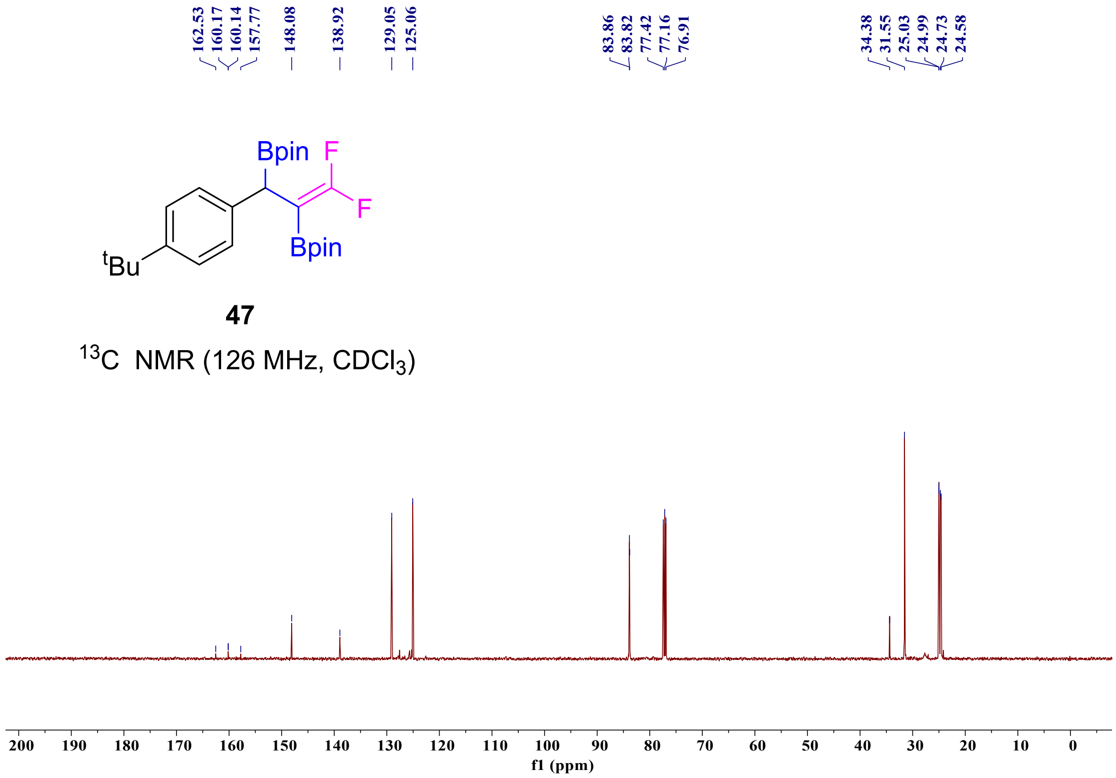

2,2'-(1-(4-(tert-butyl)phenyl)-3,3-difluoroprop-2-ene-1,2-diyl)bis(4,4,5,5-tetramethyl-1,3,2-dioxaborolane) (47)

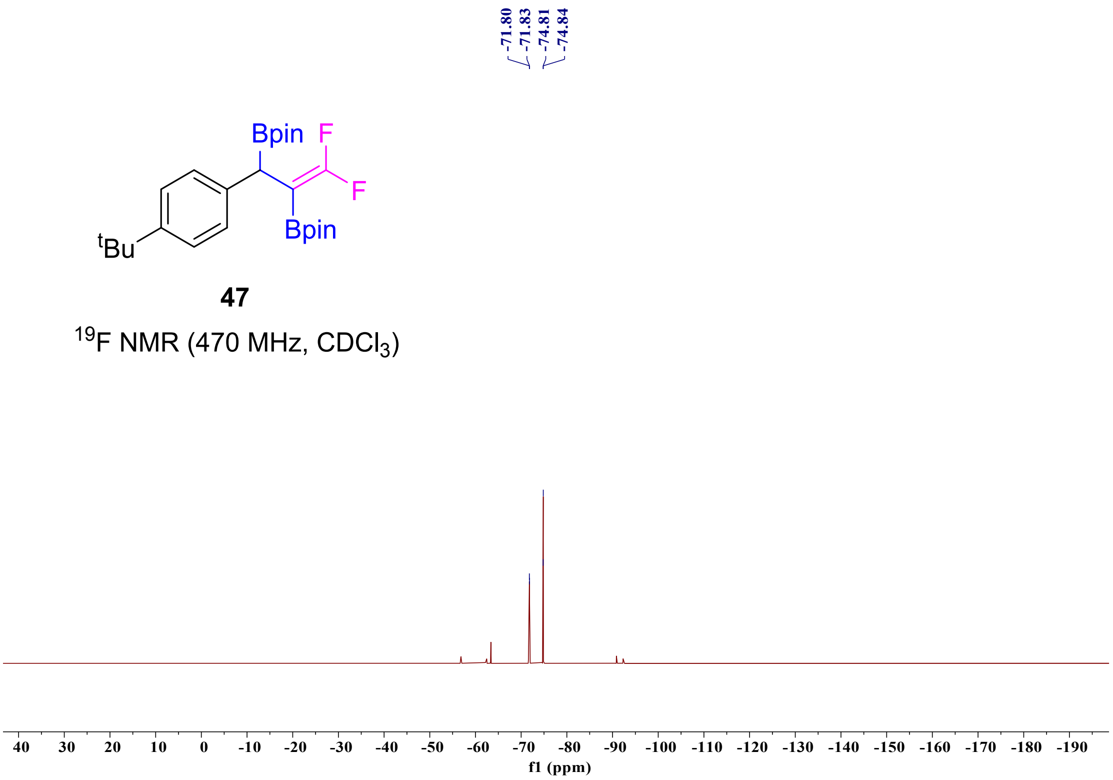

2,2'-(1-(4-(tert-butyl)phenyl)-3,3-difluoroprop-2-ene-1,2-diyl)bis(4,4,5,5-tetramethyl-1,3,2-dioxaborolane) (47)

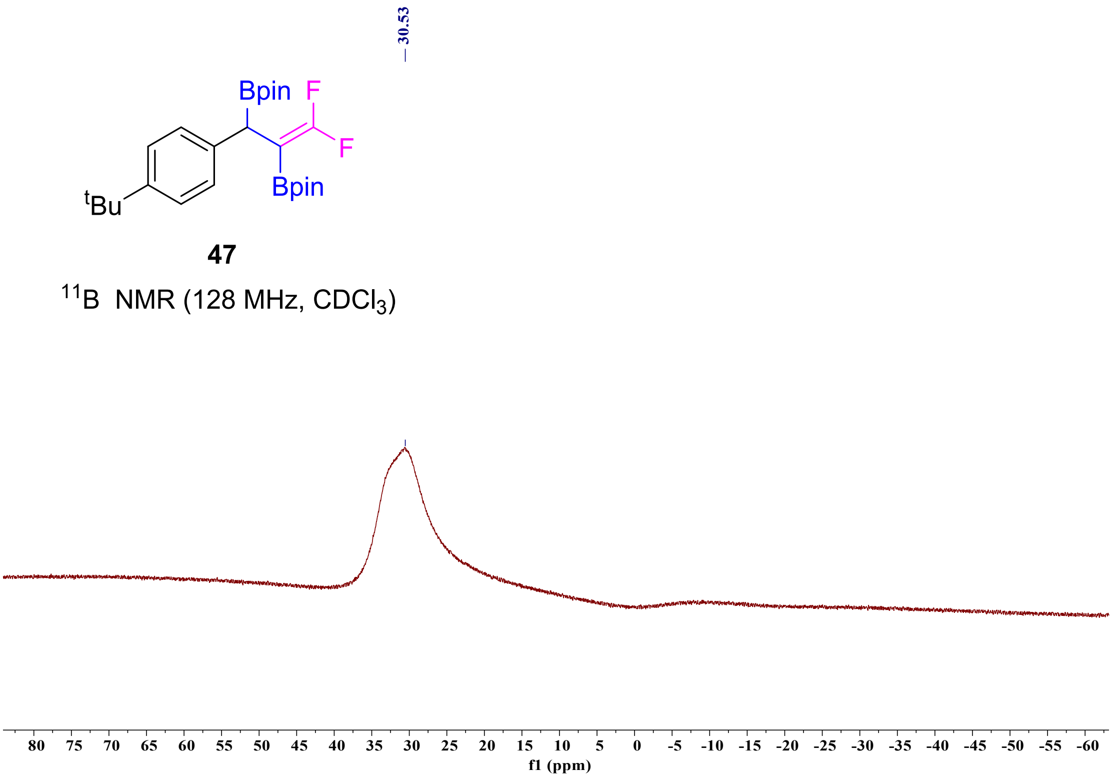

2,2'-(3,3-difluoro-1-(4-methoxyphenyl)prop-2-ene-1,2-diyl)bis(4,4,5,5-tetramethyl-1,3,2-dioxaborolane) (48)

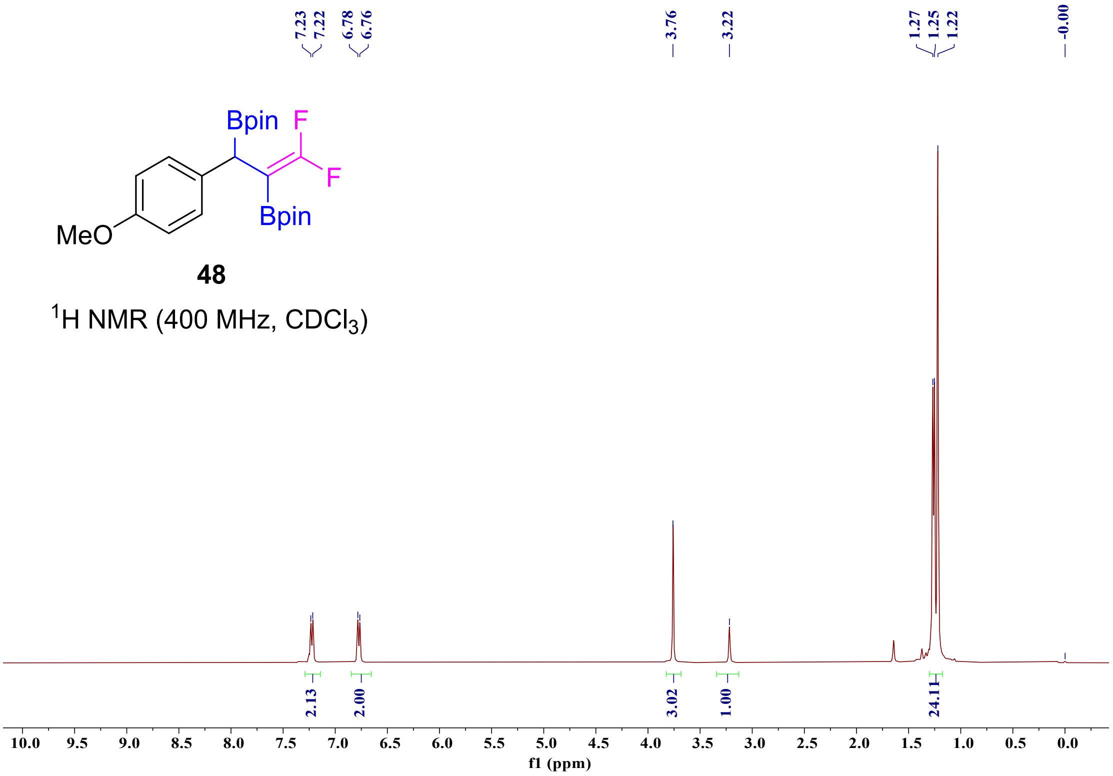

2,2'-(3,3-difluoro-1-(4-methoxyphenyl)prop-2-ene-1,2-diyl)bis(4,4,5,5-tetramethyl-1,3,2-dioxaborolane) (48)

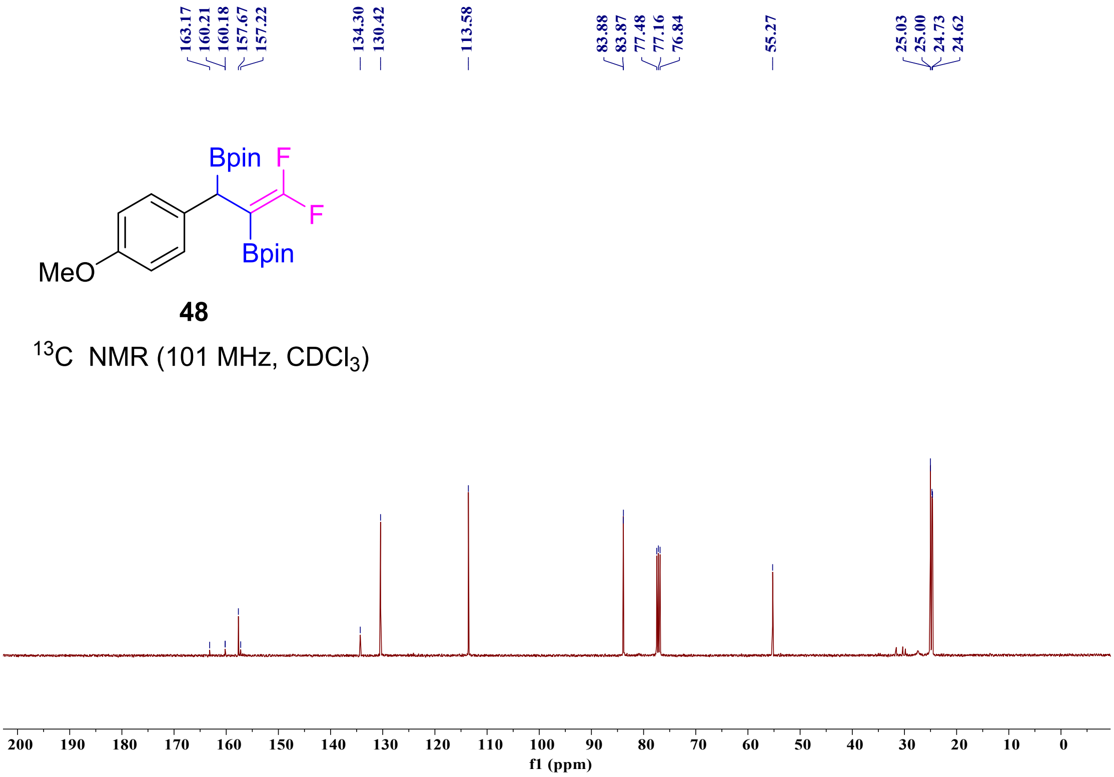

2,2'-(3,3-difluoro-1-(4-methoxyphenyl)prop-2-ene-1,2-diyl)bis(4,4,5,5-tetramethyl-1,3,2-dioxaborolane) (48)

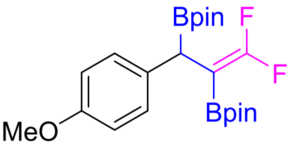

48

^{19}F NMR (376 MHz, $CDCl_3$)

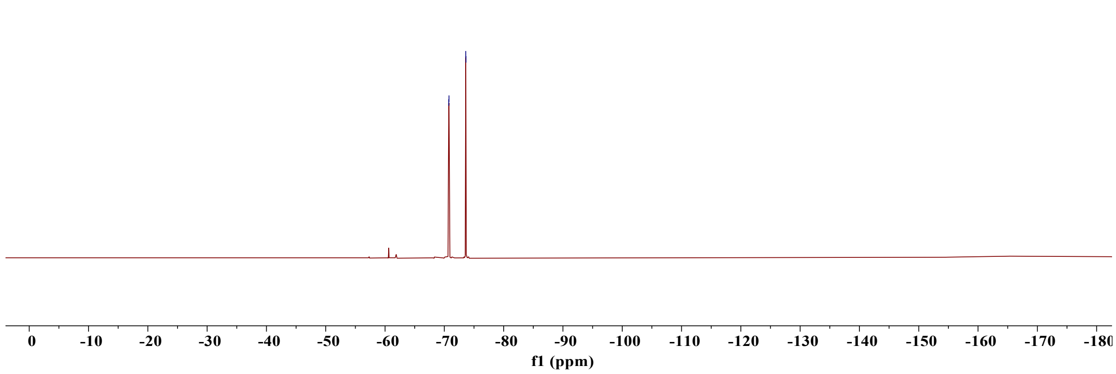

2,2'-(3,3-difluoro-1-(4-methoxyphenyl)prop-2-ene-1,2-diyl)bis(4,4,5,5-tetramethyl-1,3,2-dioxaborolane) (48)

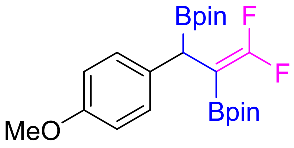

48

^{11}B NMR (128 MHz, $CDCl_3$)

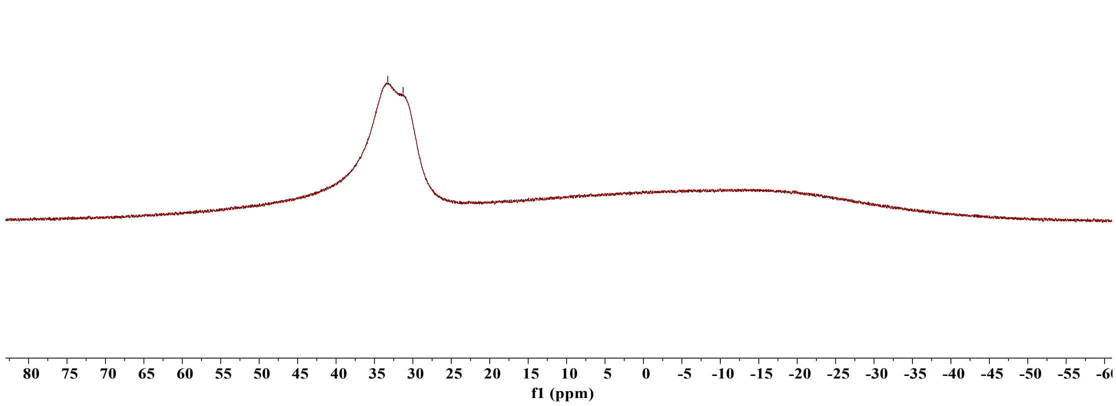

2,2'-(3,3-difluoro-1-(4-(trifluoromethoxy)phenyl)prop-2-ene-1,2-diyl)bis(4,4,5,5-tetramethyl-1,3,2-dioxaborolane) (49)

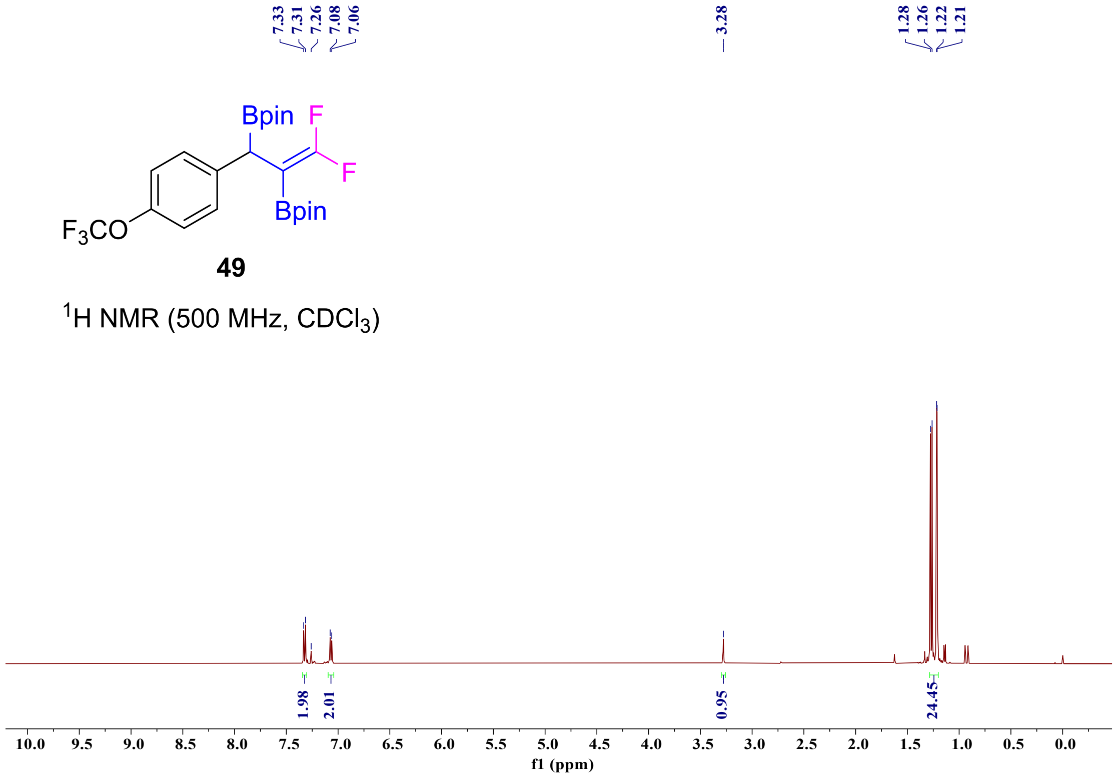

2,2'-(3,3-difluoro-1-(4-(trifluoromethoxy)phenyl)prop-2-ene-1,2-diyl)bis(4,4,5,5-tetramethyl-1,3,2-dioxaborolane) (49)

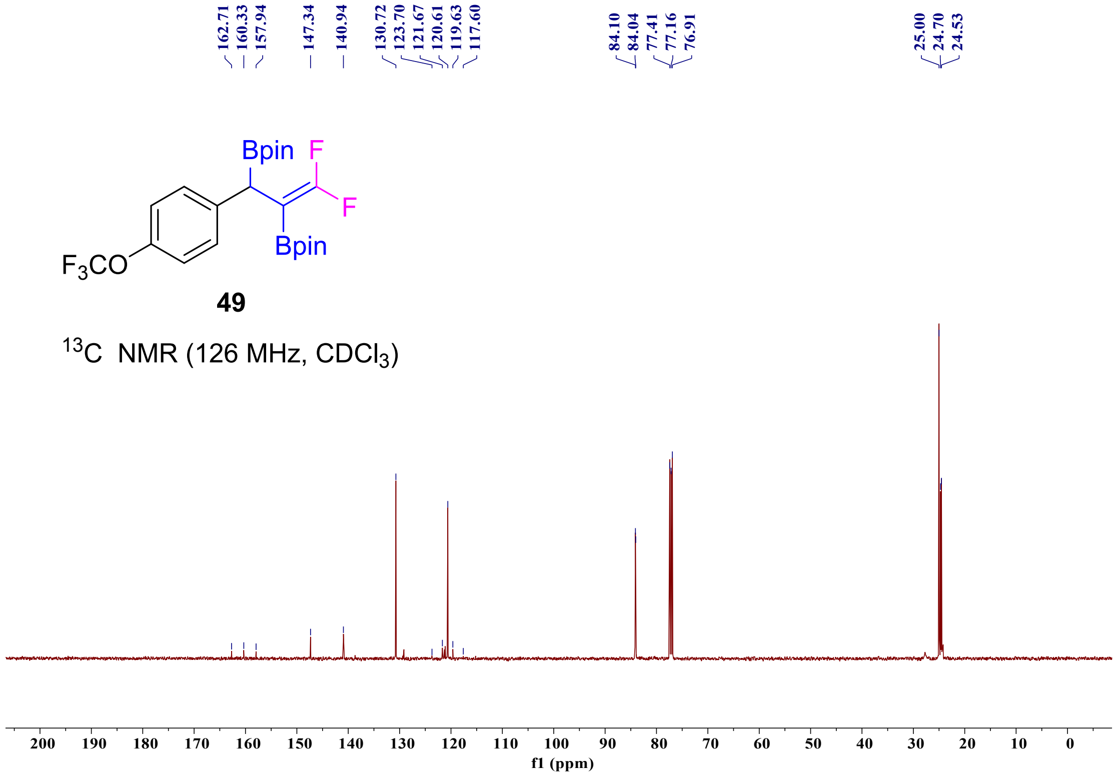

2,2'-(3,3-difluoro-1-(4-(trifluoromethoxy)phenyl)prop-2-ene-1,2-diyl)bis(4,4,5,5-tetramethyl-1,3,2-dioxaborolane) (49)

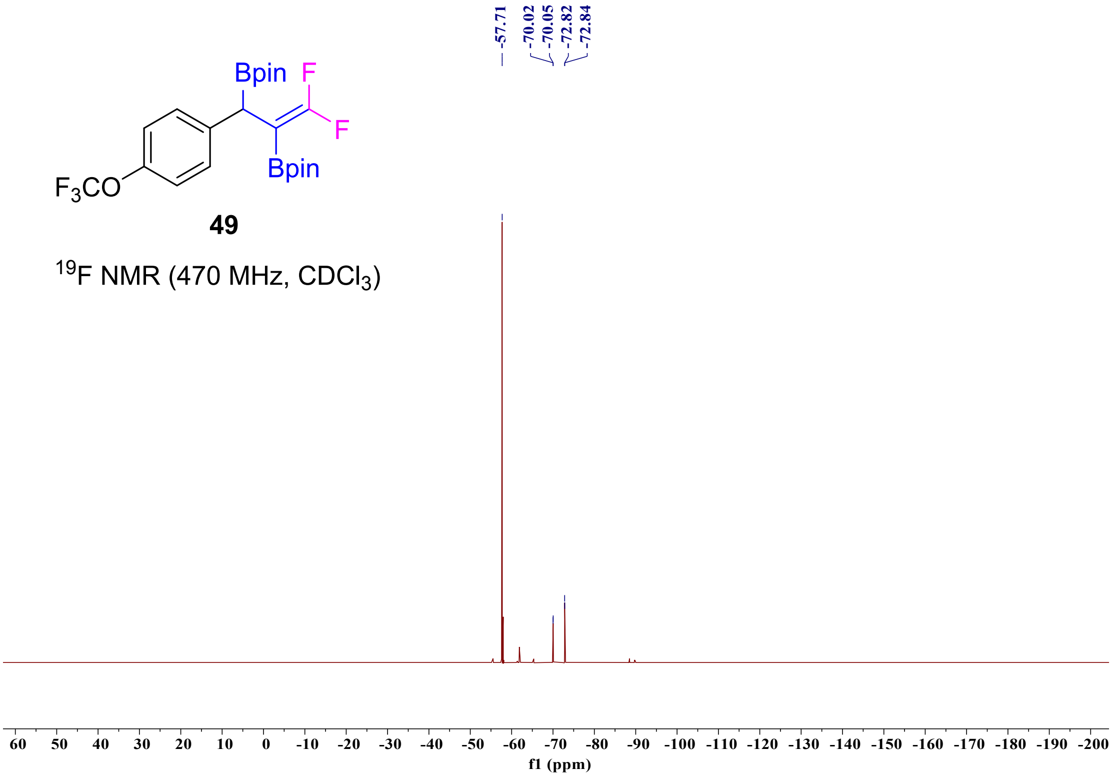

2,2'-(3,3-difluoro-1-(4-(trifluoromethoxy)phenyl)prop-2-ene-1,2-diyl)bis(4,4,5,5-tetramethyl-1,3,2-dioxaborolane) (49)

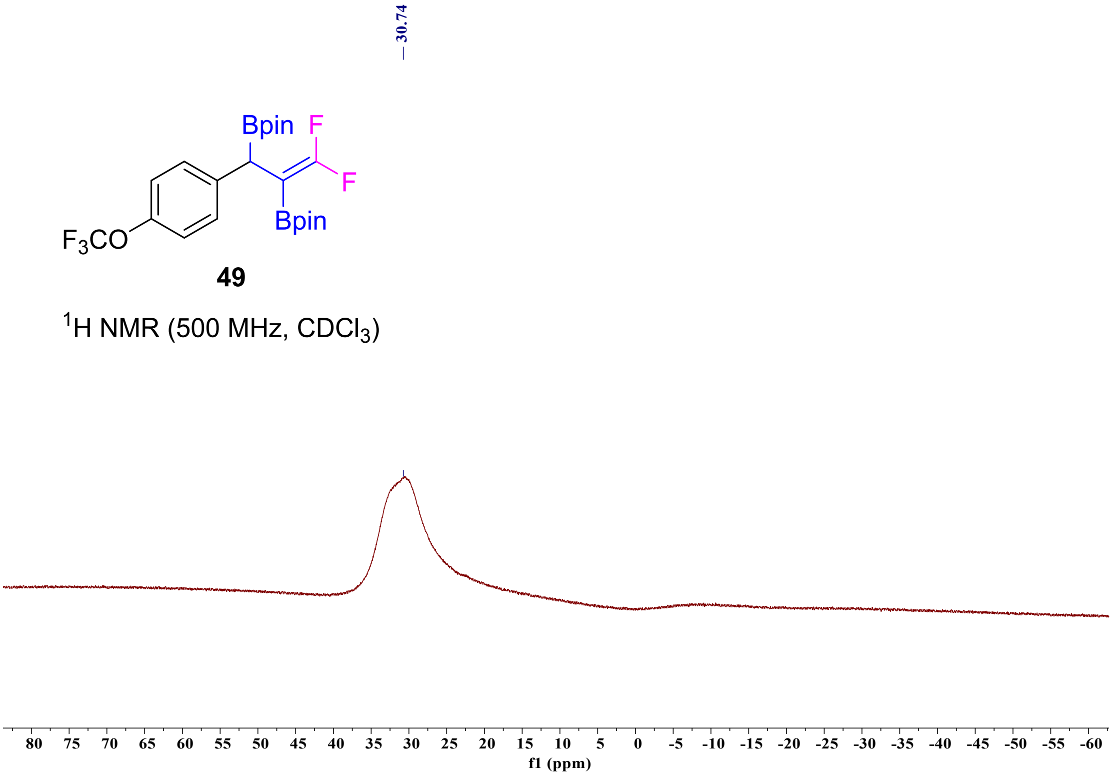

2,2'-(1-(4-(benzyloxy)phenyl)-3,3-difluoroprop-2-ene-1,2-diyl)bis(4,4,5,5-tetramethyl-1,3,2-dioxaborolane) (50)

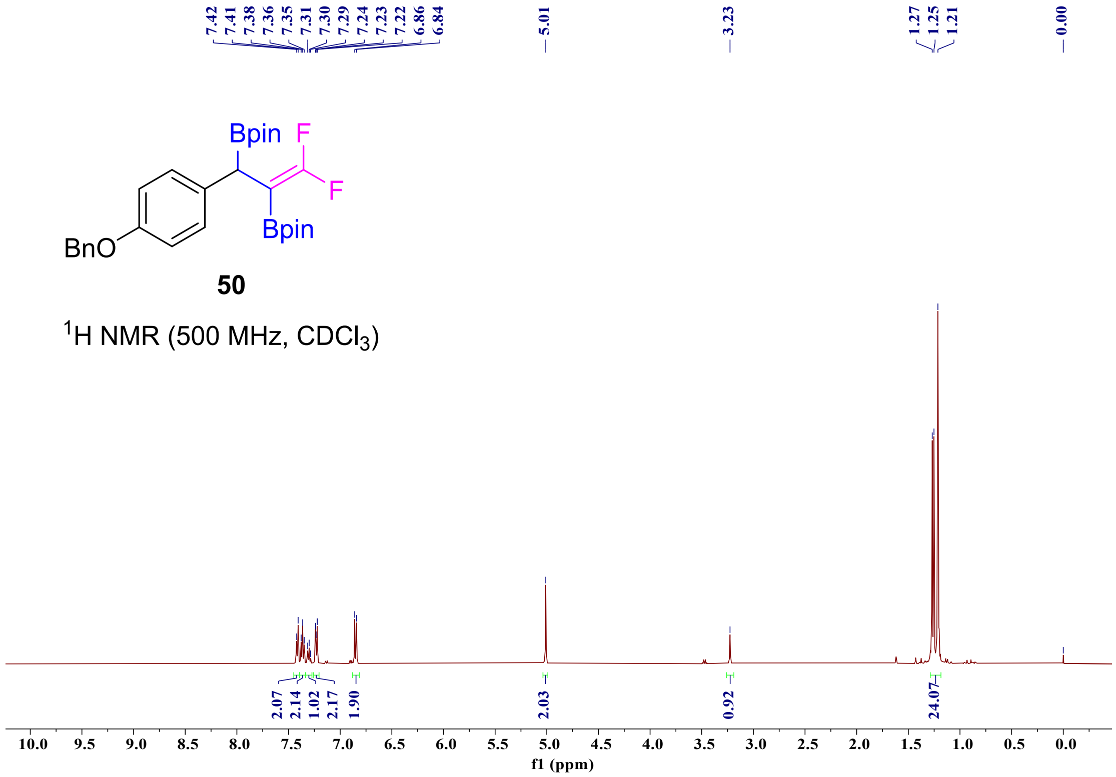

2,2'-(1-(4-(benzyloxy)phenyl)-3,3-difluoroprop-2-ene-1,2-diyl)bis(4,4,5,5-tetramethyl-1,3,2-dioxaborolane) (50)

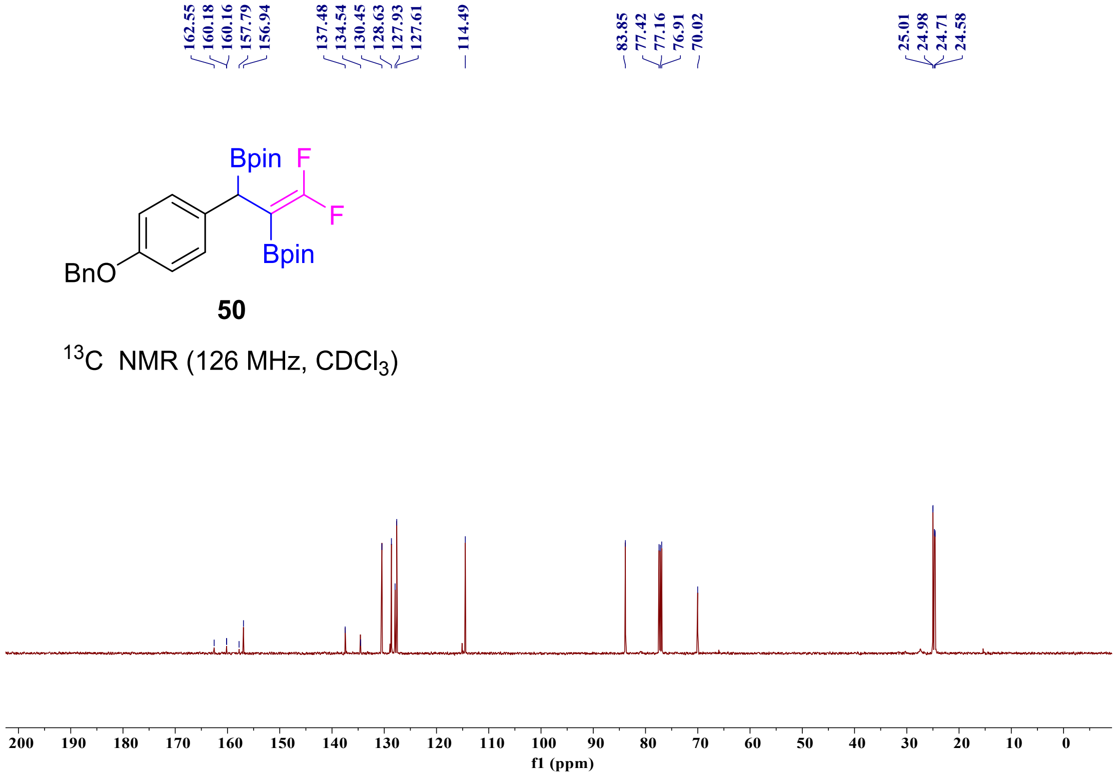

2,2'-(1-(4-(benzyloxy)phenyl)-3,3-difluoroprop-2-ene-1,2-diyl)bis(4,4,5,5-tetramethyl-1,3,2-dioxaborolane) (50)

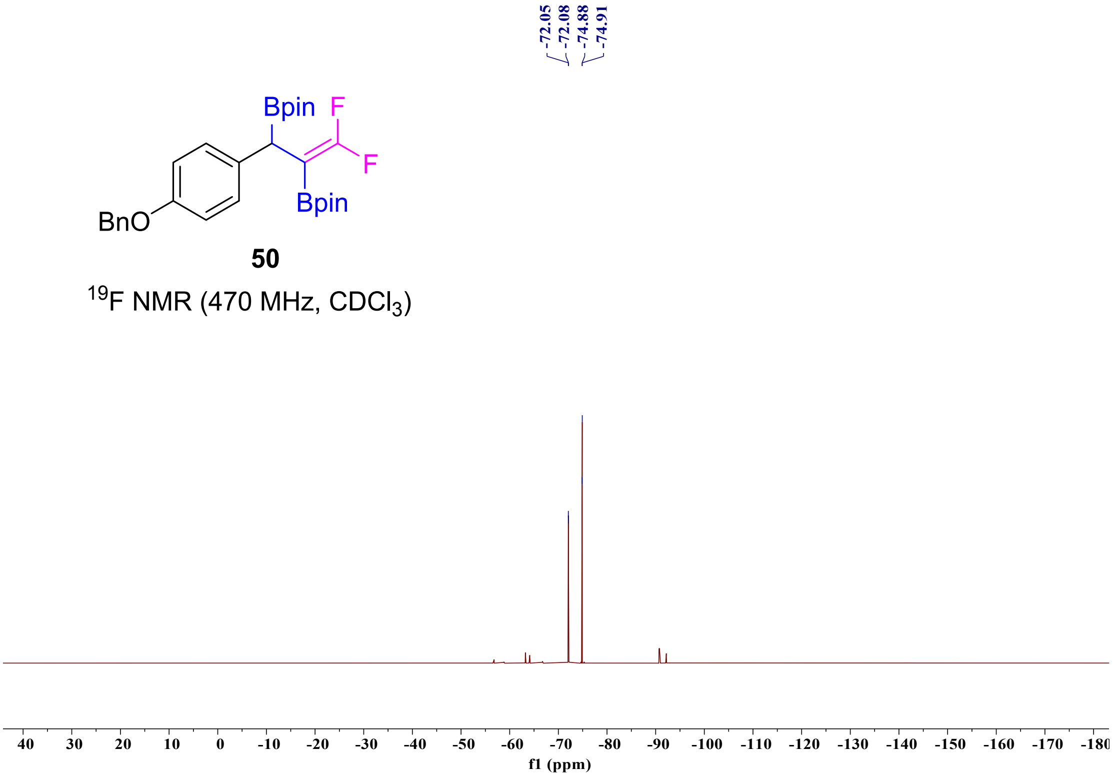

2,2'-(1-(4-(benzyloxy)phenyl)-3,3-difluoroprop-2-ene-1,2-diyl)bis(4,4,5,5-tetramethyl-1,3,2-dioxaborolane) (50)

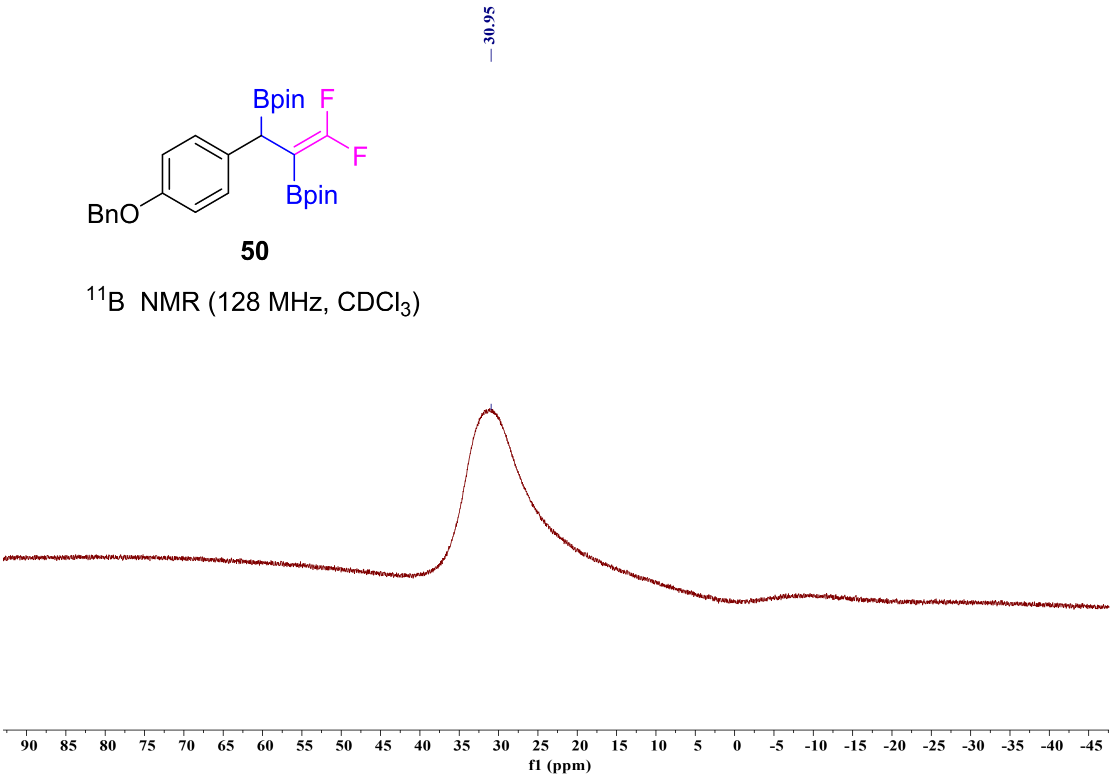

2,2'-(1-([1,1'-biphenyl]-4-yl)-3,3-difluoroprop-2-ene-1,2-diyl)bis(4,4,5,5-tetramethyl-1,3,2-dioxaborolane) (51)

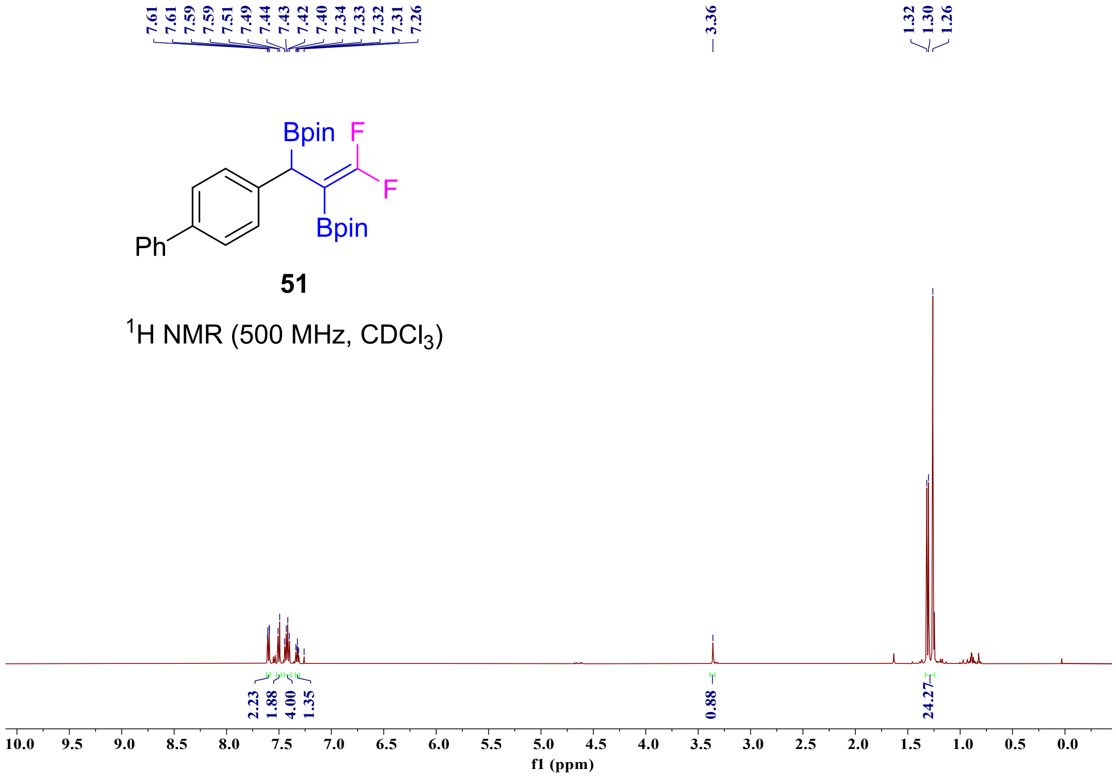

2,2'-(1-([1,1'-biphenyl]-4-yl)-3,3-difluoroprop-2-ene-1,2-diyl)bis(4,4,5,5-tetramethyl-1,3,2-dioxaborolane) (51)

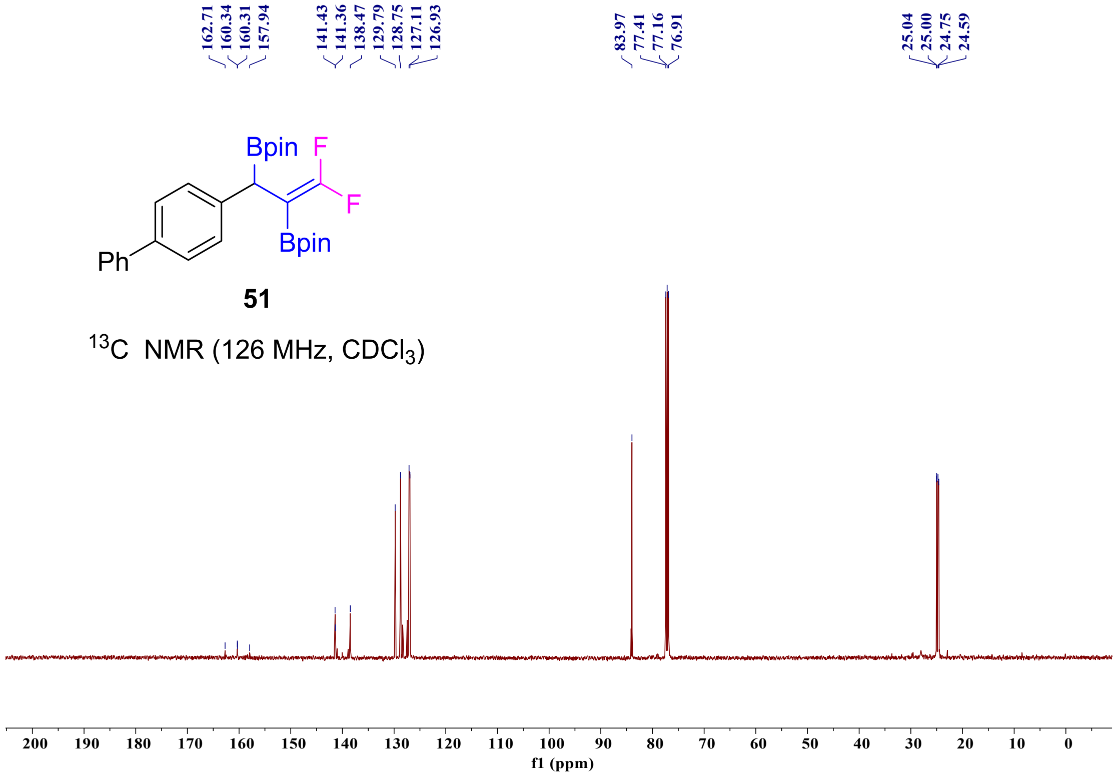

2,2'-(1-([1,1'-biphenyl]-4-yl)-3,3-difluoroprop-2-ene-1,2-diyl)bis(4,4,5,5-tetramethyl-1,3,2-dioxaborolane) (51)

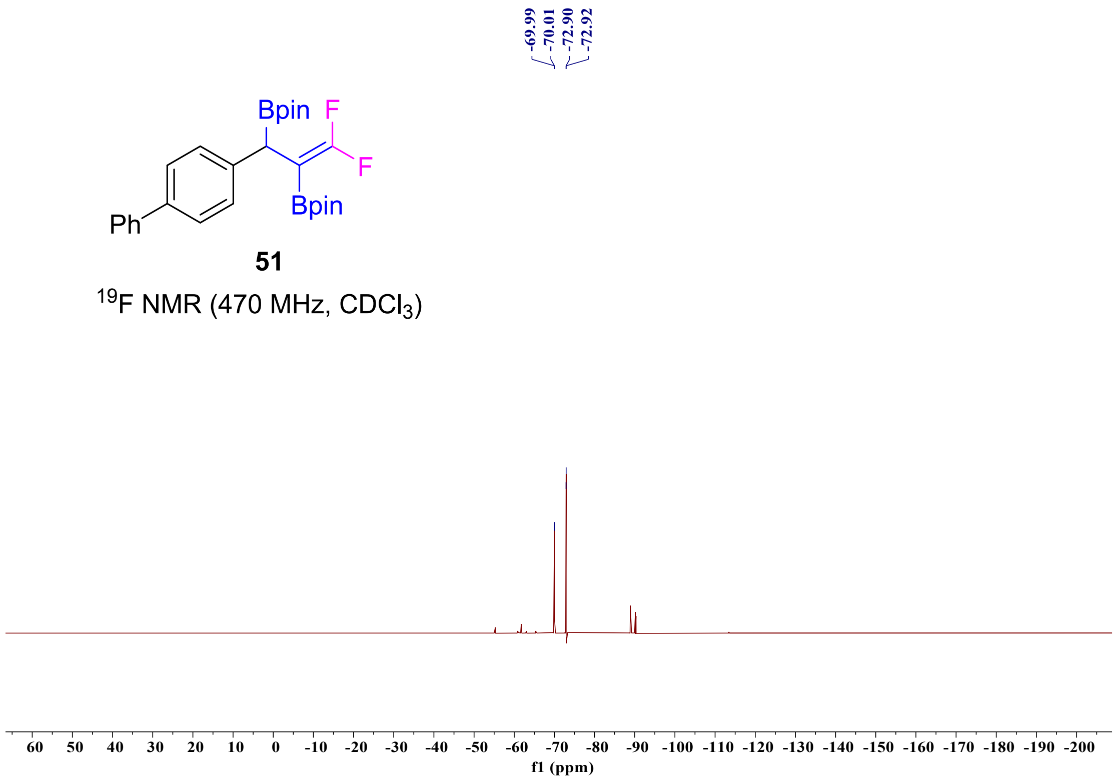

2,2'-(1-([1,1'-biphenyl]-4-yl)-3,3-difluoroprop-2-ene-1,2-diyl)bis(4,4,5,5-tetramethyl-1,3,2-dioxaborolane) (51)

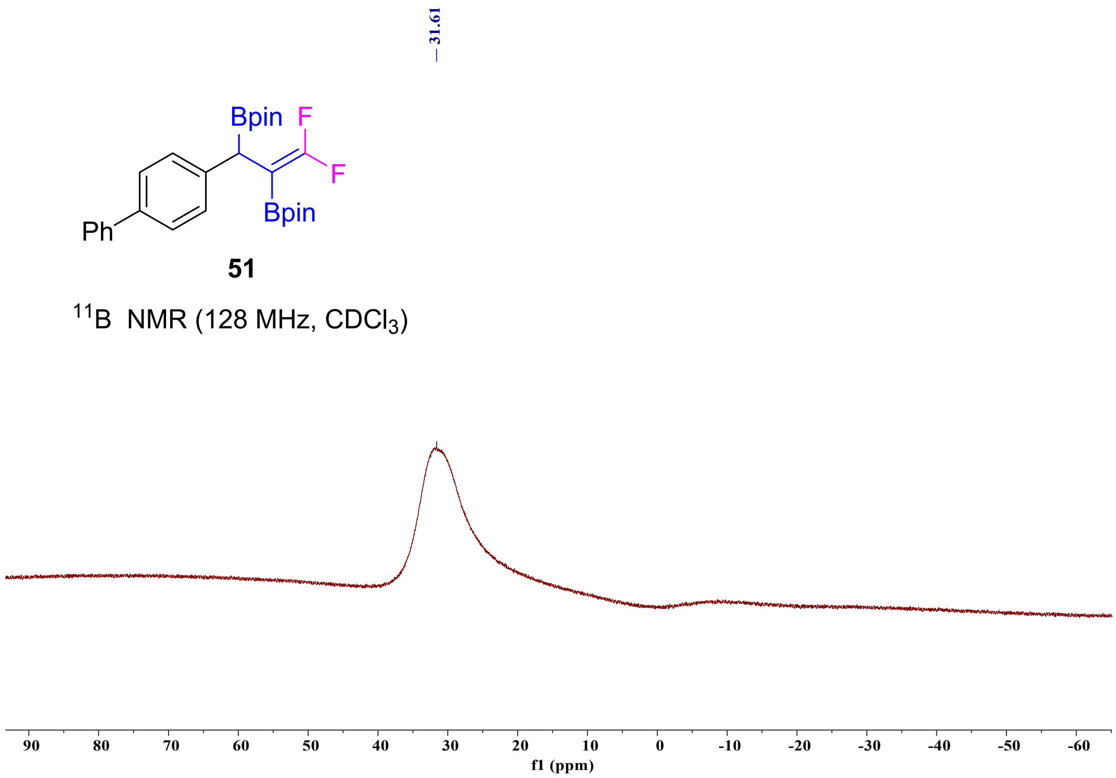

2,2'-(3,3-difluoro-1-(4-(methylthio)phenyl)prop-2-ene-1,2-diyl)bis(4,4,5,5-tetramethyl-1,3,2-dioxaborolane) (52)

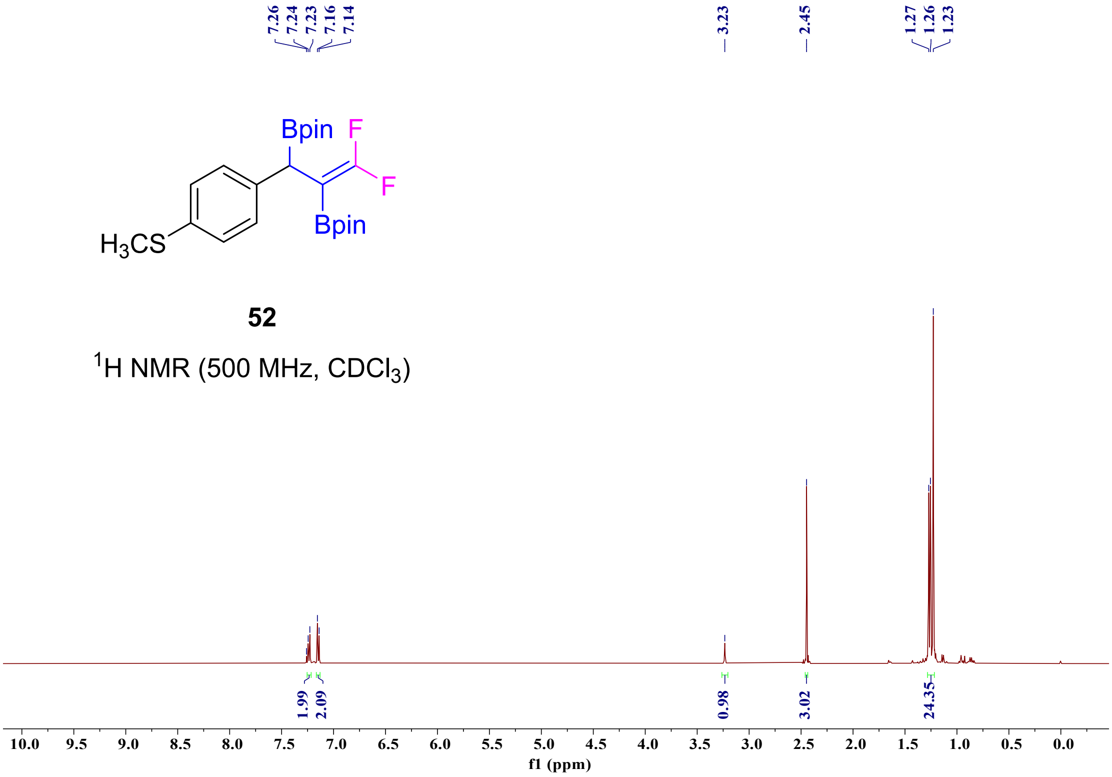

2,2'-(3,3-difluoro-1-(4-(methylthio)phenyl)prop-2-ene-1,2-diyl)bis(4,4,5,5-tetramethyl-1,3,2-dioxaborolane) (52)

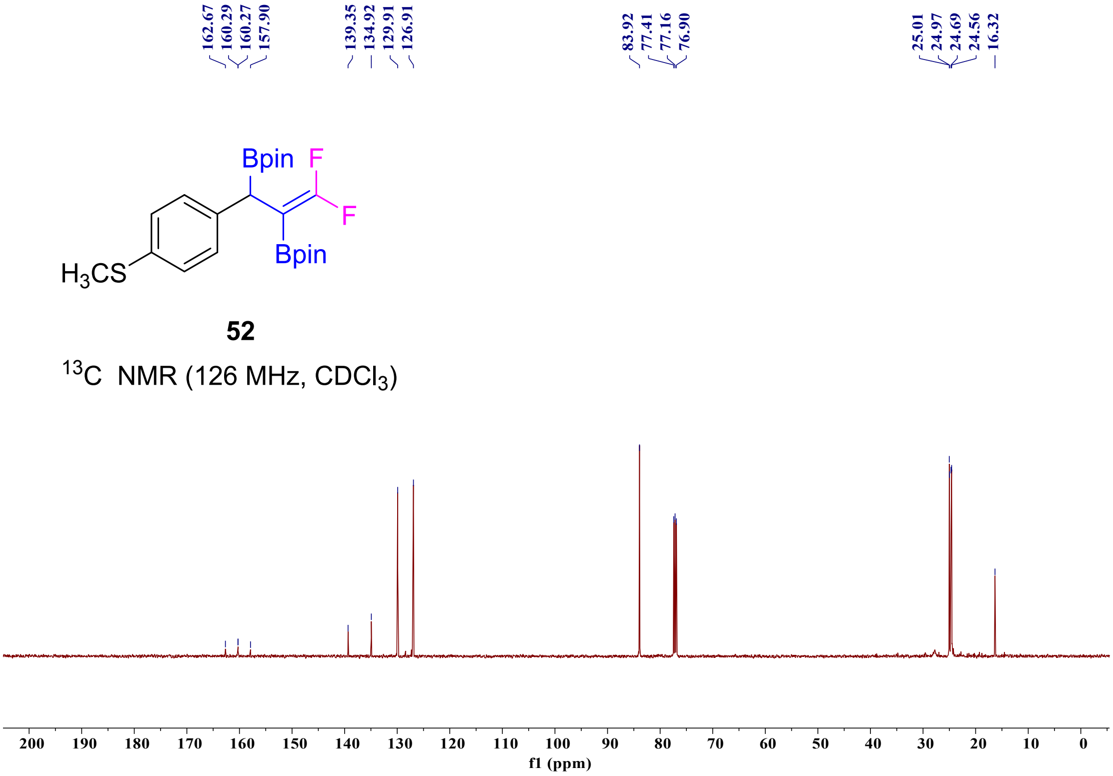

2,2'-(3,3-difluoro-1-(4-(methylthio)phenyl)prop-2-ene-1,2-diyl)bis(4,4,5,5-tetramethyl-1,3,2-dioxaborolane) (52)

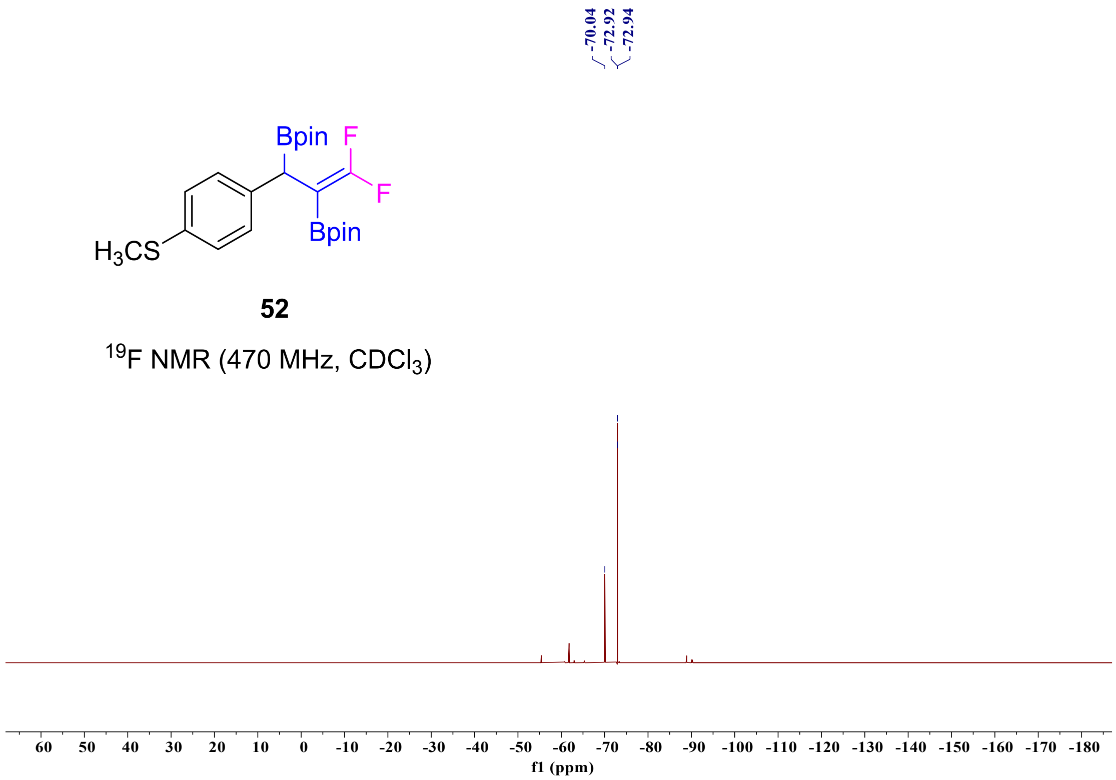

2,2'-(3,3-difluoro-1-(4-(methylthio)phenyl)prop-2-ene-1,2-diyl)bis(4,4,5,5-tetramethyl-1,3,2-dioxaborolane) (52)

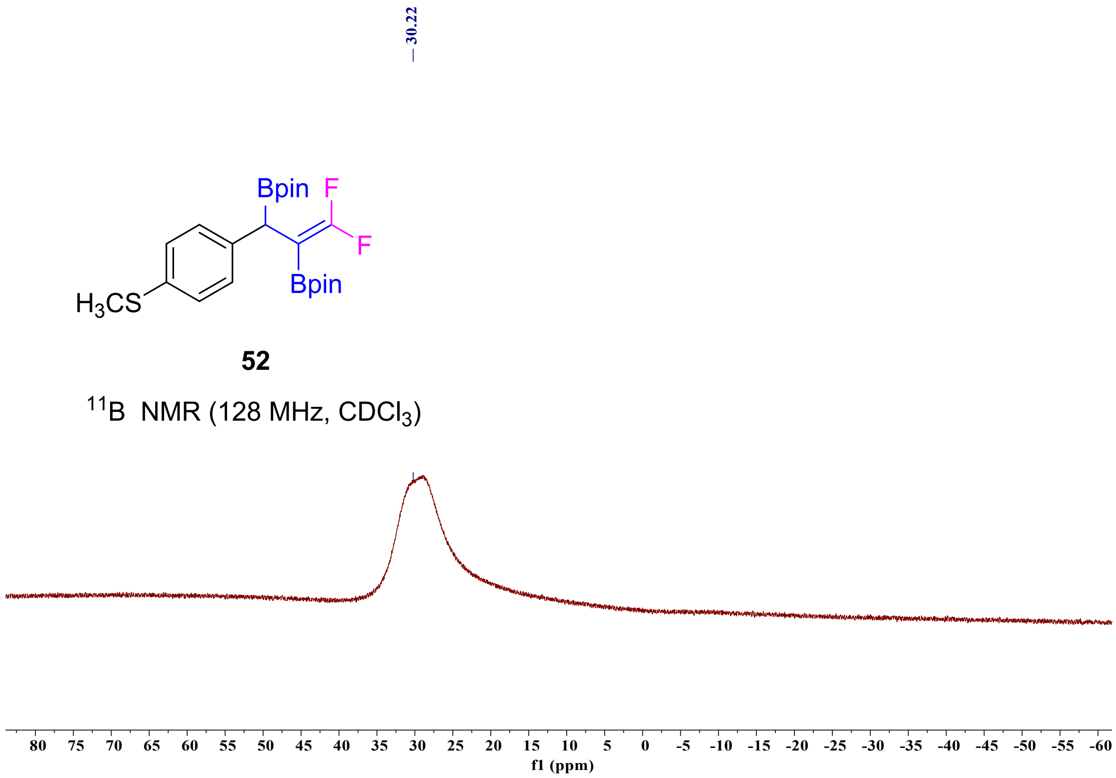

2,2'-(3,3-difluoro-1-(furan-3-yl)prop-2-ene-1,2-diyl)bis(4,4,5,5-tetramethyl-1,3,2-dioxaborolane) (53)

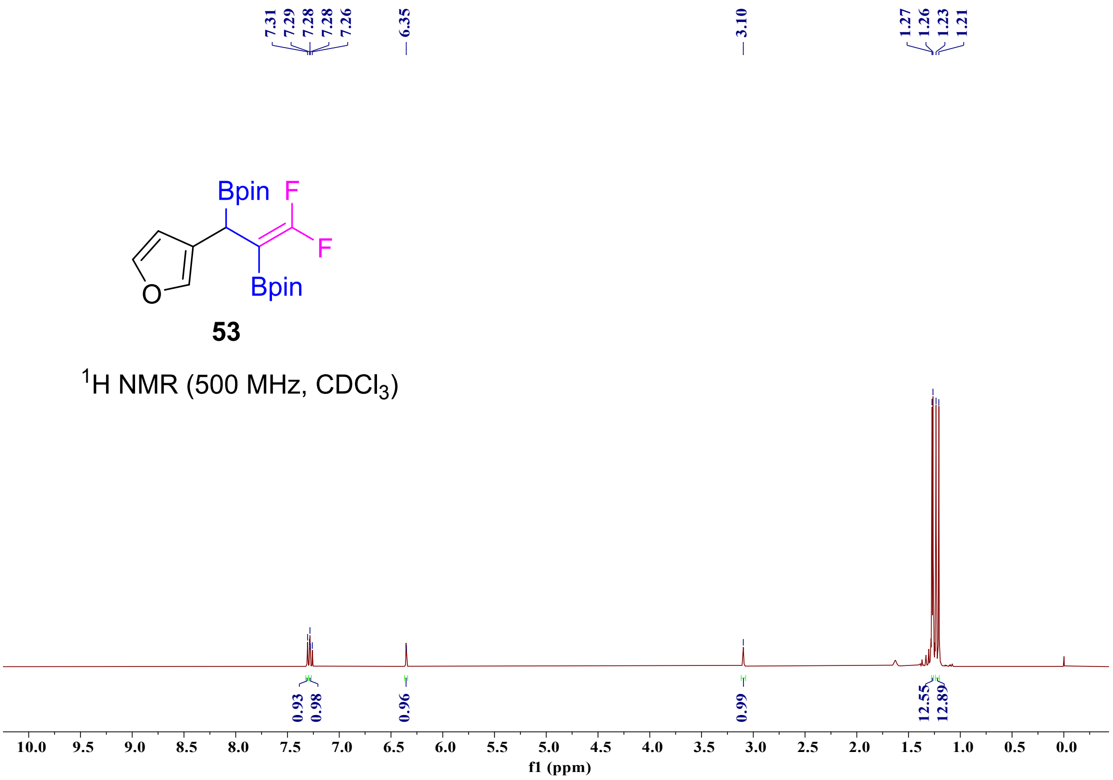

2,2'-(3,3-difluoro-1-(furan-3-yl)prop-2-ene-1,2-diyl)bis(4,4,5,5-tetramethyl-1,3,2-dioxaborolane) (53)

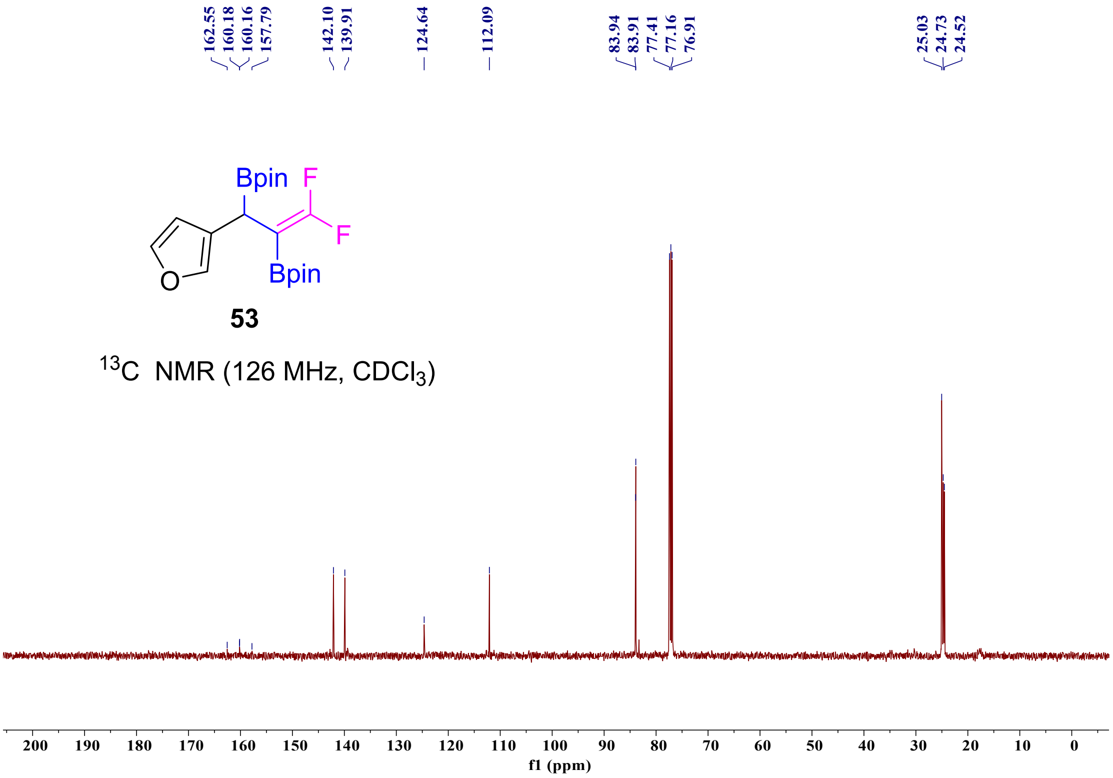

2,2'-(3,3-difluoro-1-(furan-3-yl)prop-2-ene-1,2-diyl)bis(4,4,5,5-tetramethyl-1,3,2-dioxaborolane) (53)

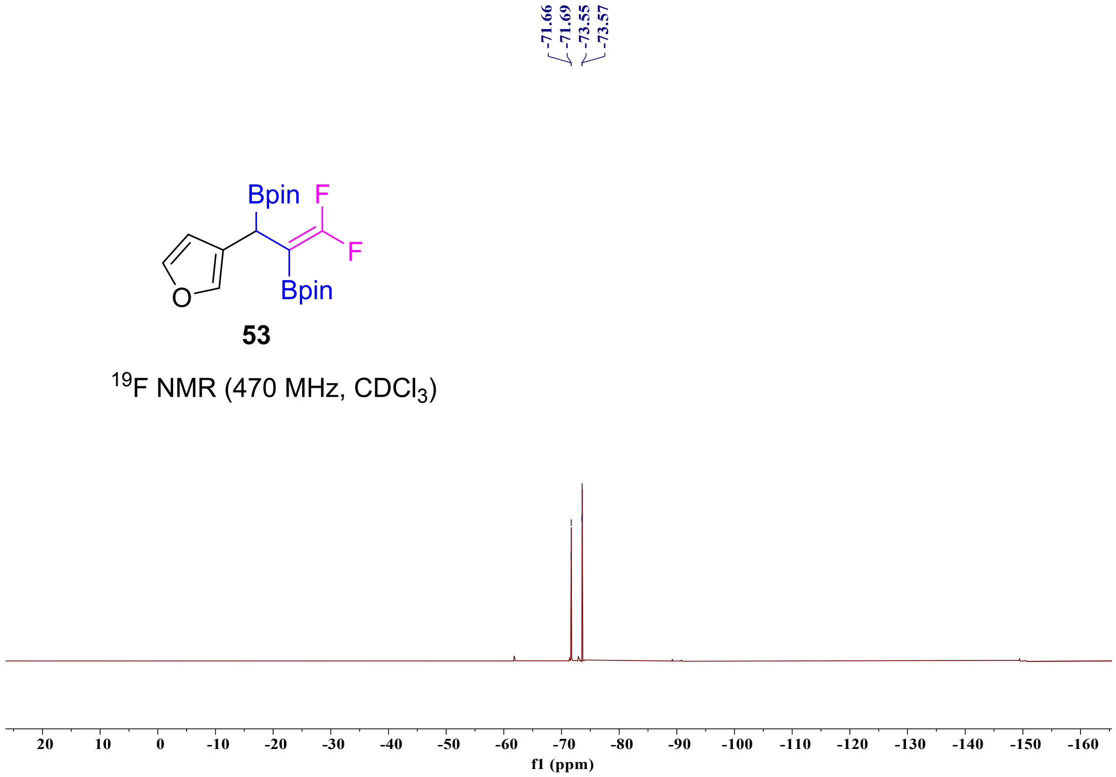

2,2'-(3,3-difluoro-1-(furan-3-yl)prop-2-ene-1,2-diyl)bis(4,4,5,5-tetramethyl-1,3,2-dioxaborolane) (53)

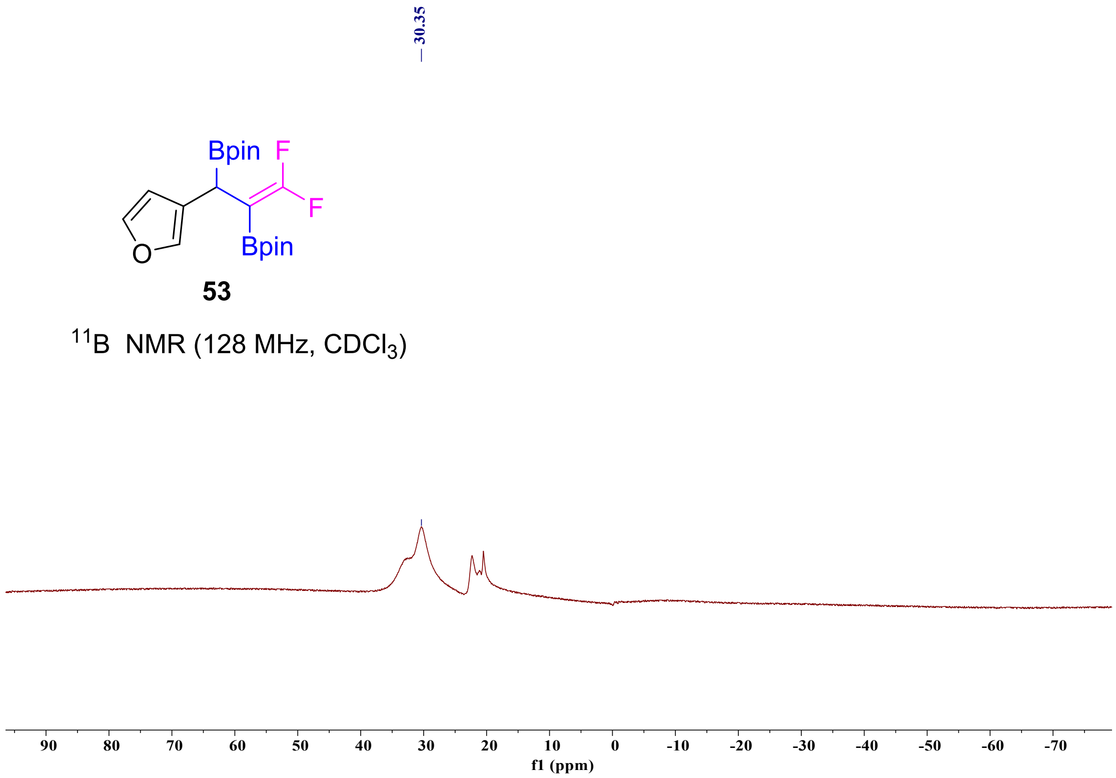

2,2'-(3,3-difluoro-1-(thiophen-3-yl)prop-2-ene-1,2-diyl)bis(4,4,5,5-tetramethyl-1,3,2-dioxaborolane) (54)

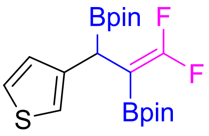

54

^{1}H NMR (500 MHz, $CDCl_3$)

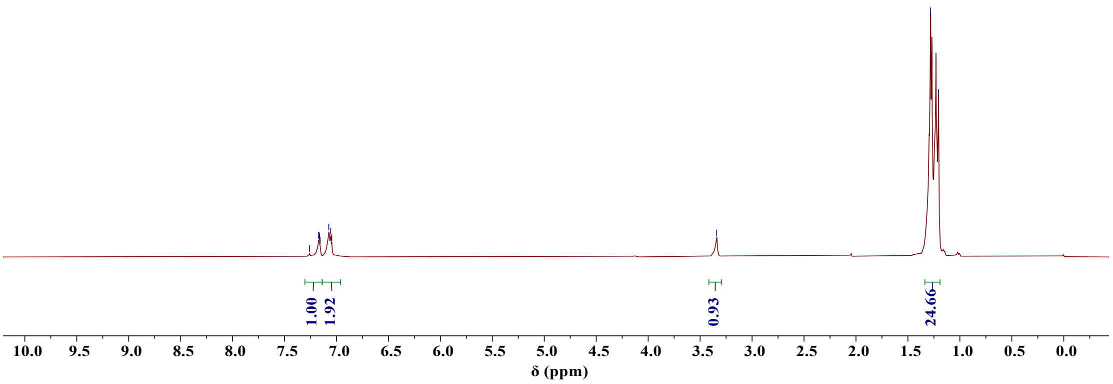

2,2'-(3,3-difluoro-1-(thiophen-3-yl)prop-2-ene-1,2-diyl)bis(4,4,5,5-tetramethyl-1,3,2-dioxaborolane) (54)

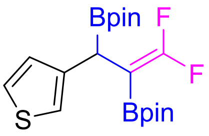

54

^{13}C NMR (126 MHz, $CDCl_3$)

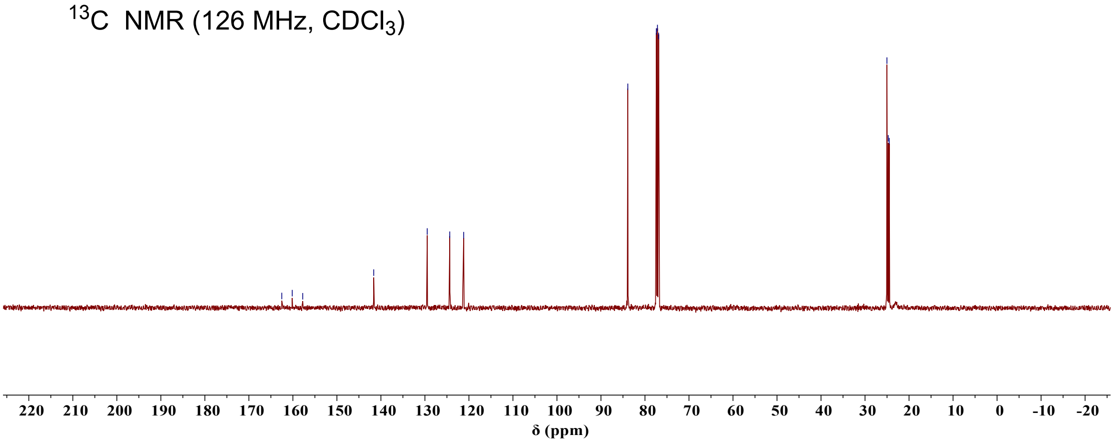

2,2'-(3,3-difluoro-1-(thiophen-3-yl)prop-2-ene-1,2-diyl)bis(4,4,5,5-tetramethyl-1,3,2-dioxaborolane) (54)

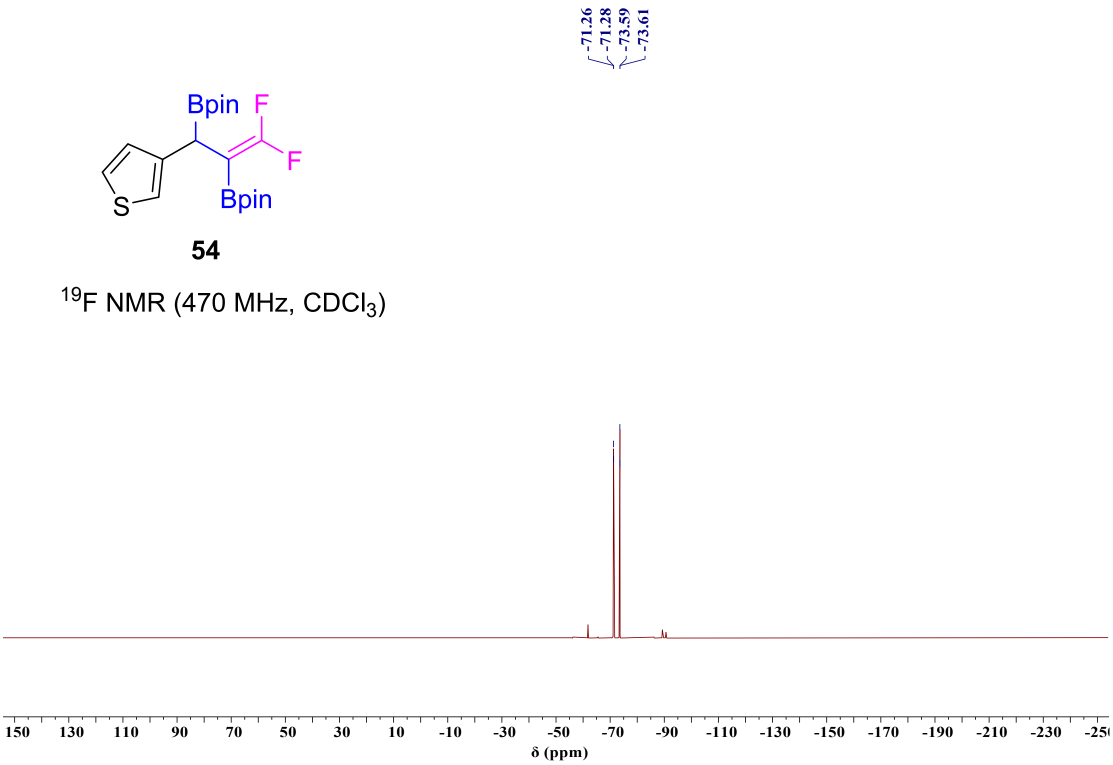

2,2'-(3,3-difluoro-1-(thiophen-3-yl)prop-2-ene-1,2-diyl)bis(4,4,5,5-tetramethyl-1,3,2-dioxaborolane) (54)

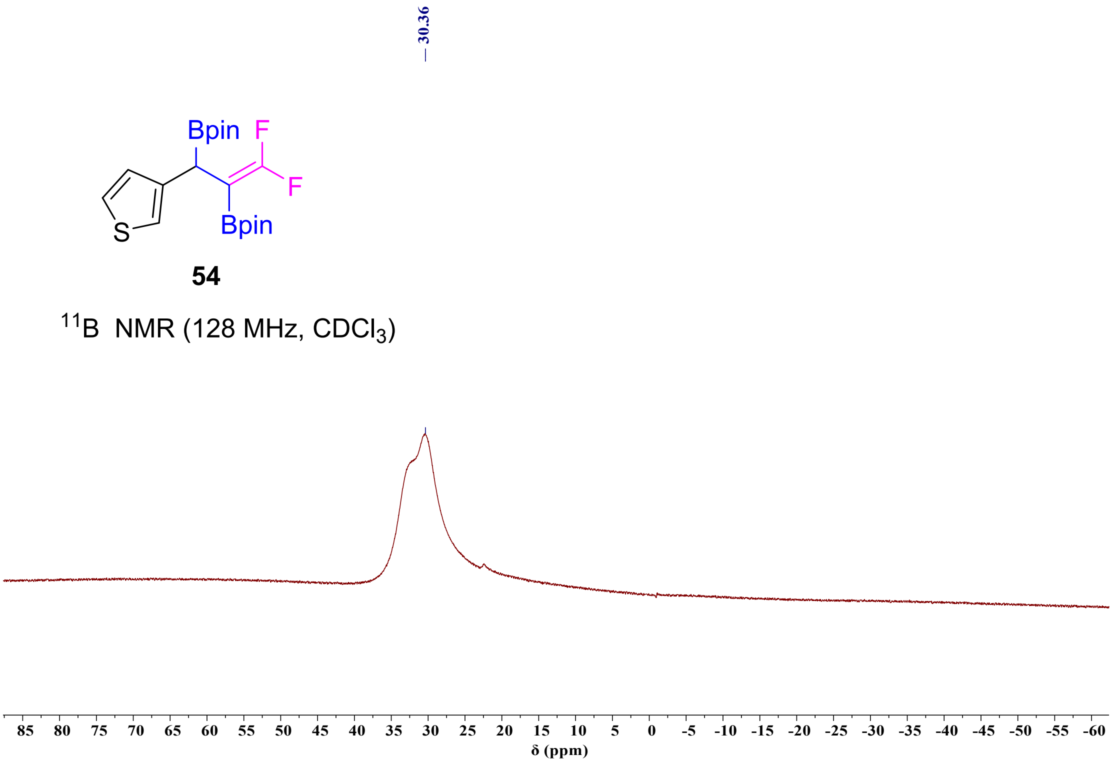

2,2'-(1-(2,3-dihydrobenzofuran-5-yl)-3,3-difluoroprop-2-ene-1,2-diyl)bis(4,4,5,5-tetramethyl-1,3,2-dioxaborolane) (55)

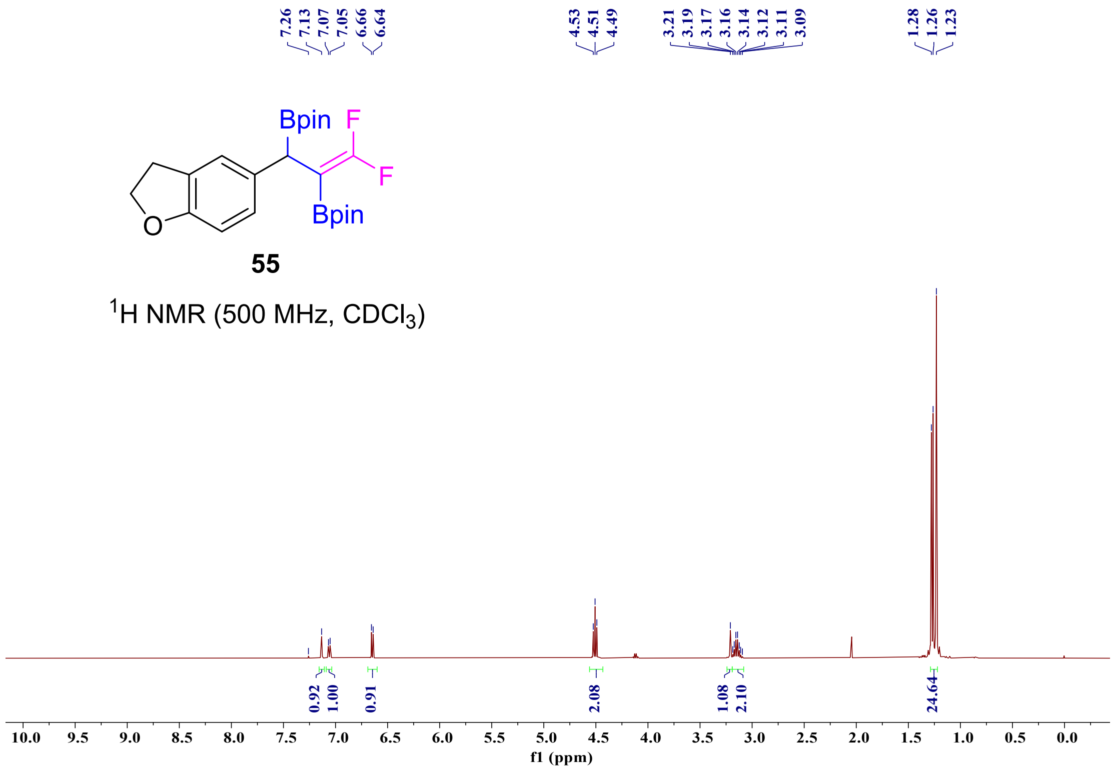

2,2'-(1-(2,3-dihydrobenzofuran-5-yl)-3,3-difluoroprop-2-ene-1,2-diyl)bis(4,4,5,5-tetramethyl-1,3,2-dioxaborolane) (55)

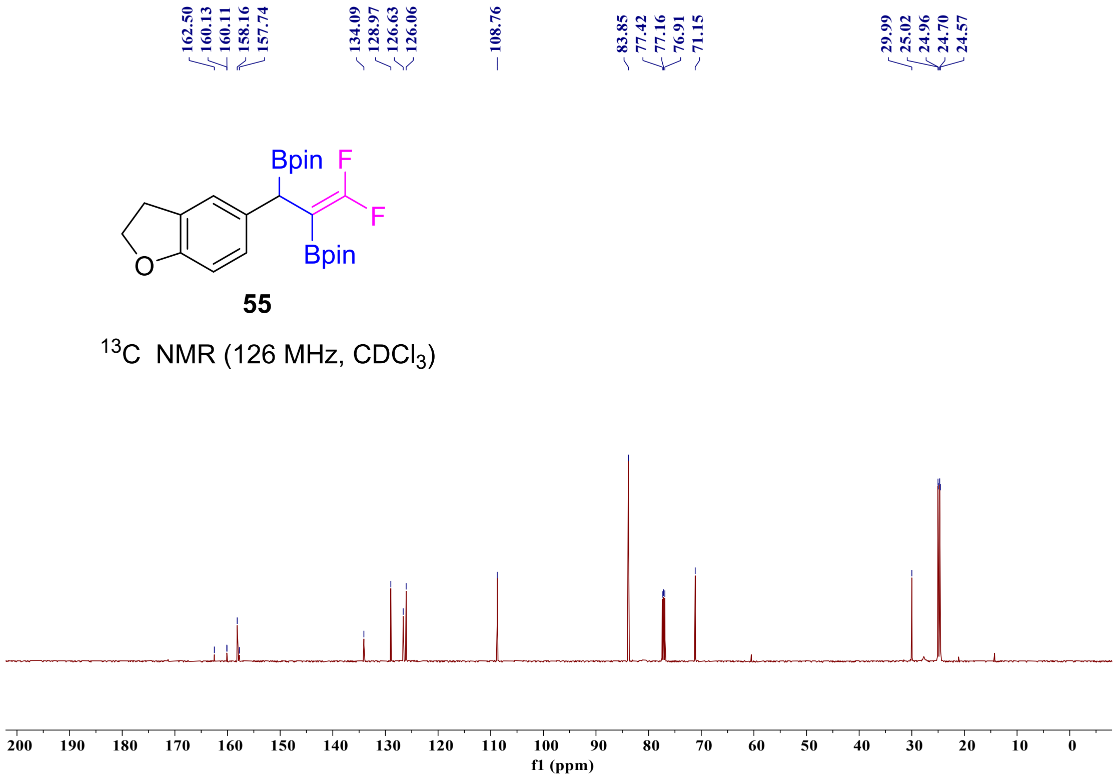

2,2'-(1-(2,3-dihydrobenzofuran-5-yl)-3,3-difluoroprop-2-ene-1,2-diyl)bis(4,4,5,5-tetramethyl-1,3,2-dioxaborolane) (55)

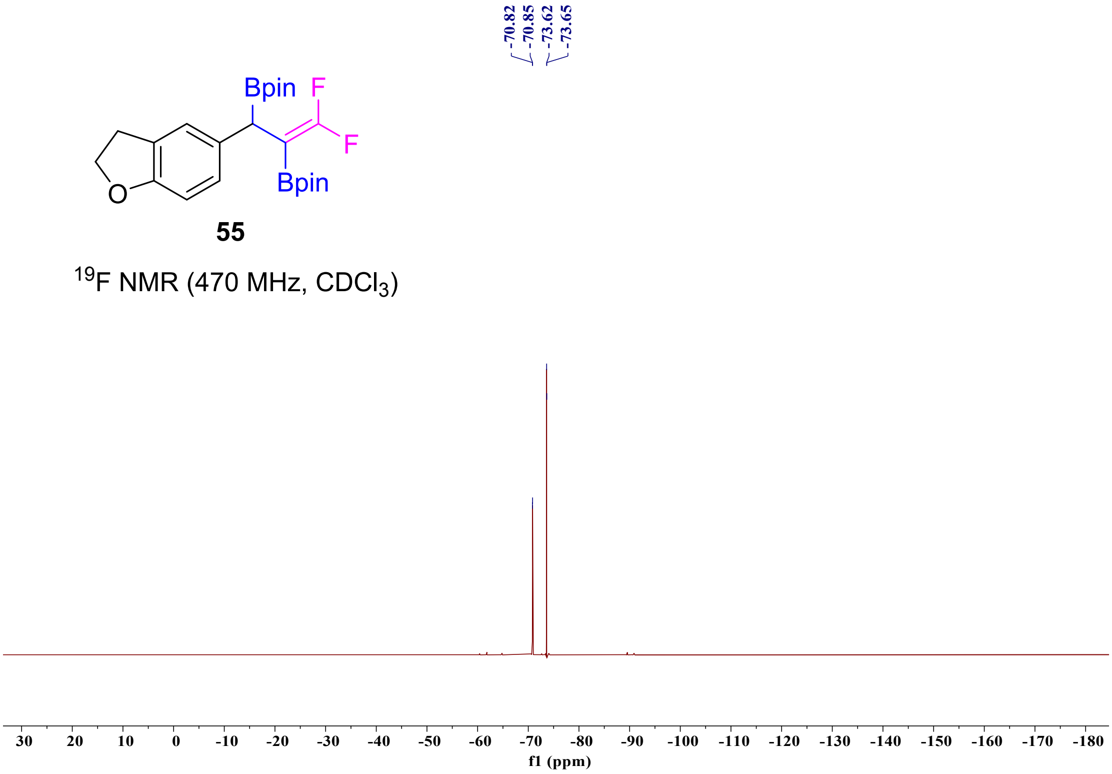

2,2'-(1-(2,3-dihydrobenzofuran-5-yl)-3,3-difluoroprop-2-ene-1,2-diyl)bis(4,4,5,5-tetramethyl-1,3,2-dioxaborolane) (55)

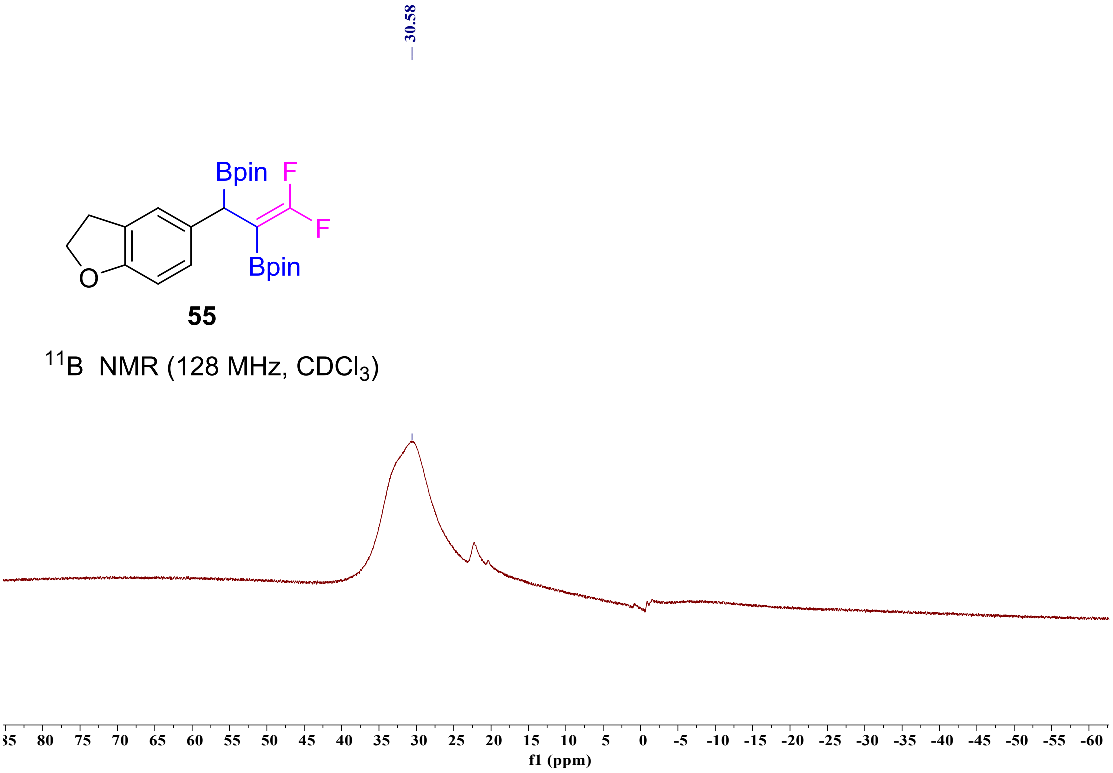

2,2'-(1-(3,4-dimethylphenyl)-3,3-difluoroprop-2-ene-1,2-diyl)bis(4,4,5,5-tetramethyl-1,3,2-dioxaborolane) (56)

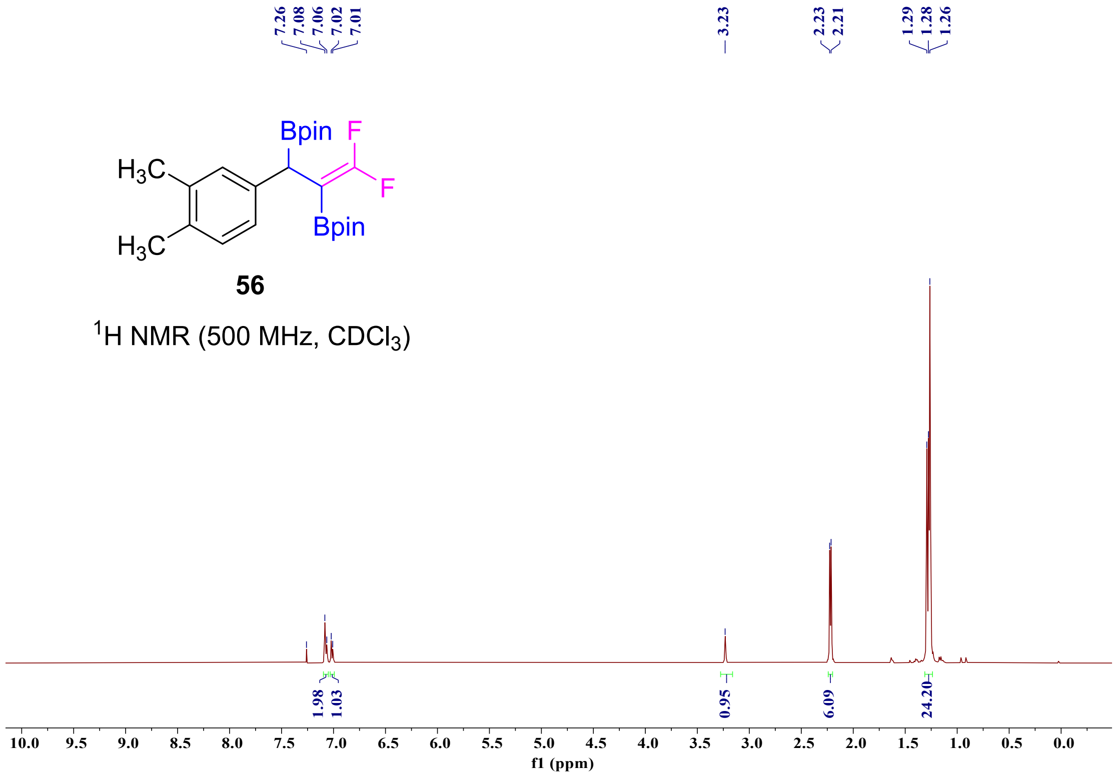

2,2'-(1-(3,4-dimethylphenyl)-3,3-difluoroprop-2-ene-1,2-diyl)bis(4,4,5,5-tetramethyl-1,3,2-dioxaborolane) (56)

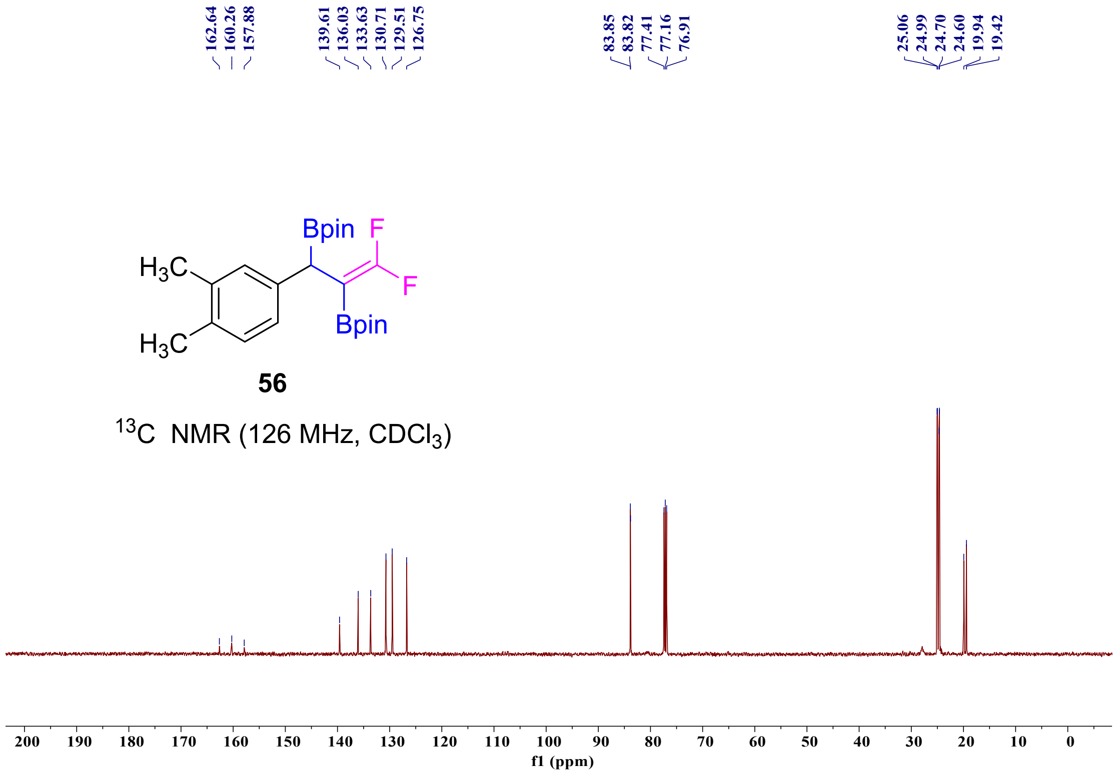

2,2'-(1-(3,4-dimethylphenyl)-3,3-difluoroprop-2-ene-1,2-diyl)bis(4,4,5,5-tetramethyl-1,3,2-dioxaborolane) (56)

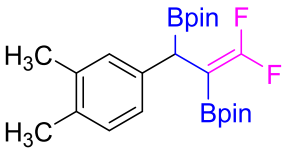

56

^{19}F NMR (470 MHz, $CDCl_3$)

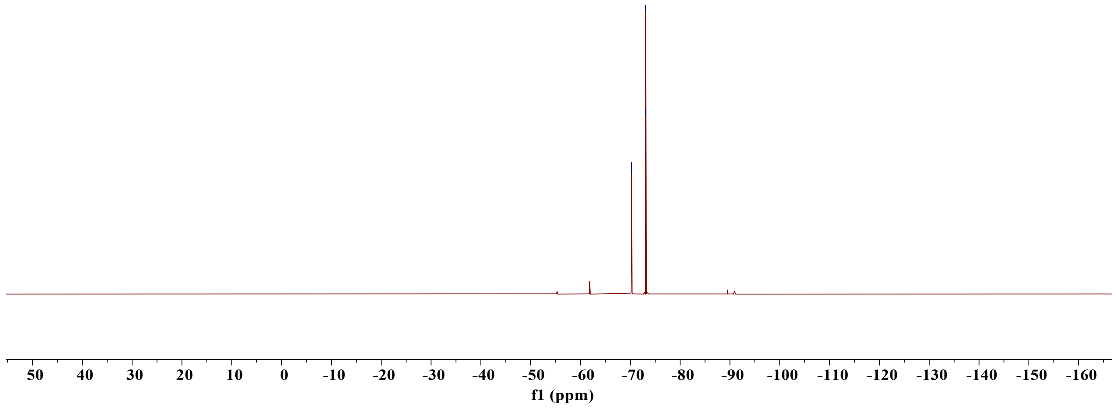

2,2'-(1-(3,4-dimethylphenyl)-3,3-difluoroprop-2-ene-1,2-diyl)bis(4,4,5,5-tetramethyl-1,3,2-dioxaborolane) (56)

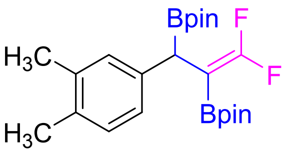

56

^{11}B NMR (128 MHz, $CDCl_3$)

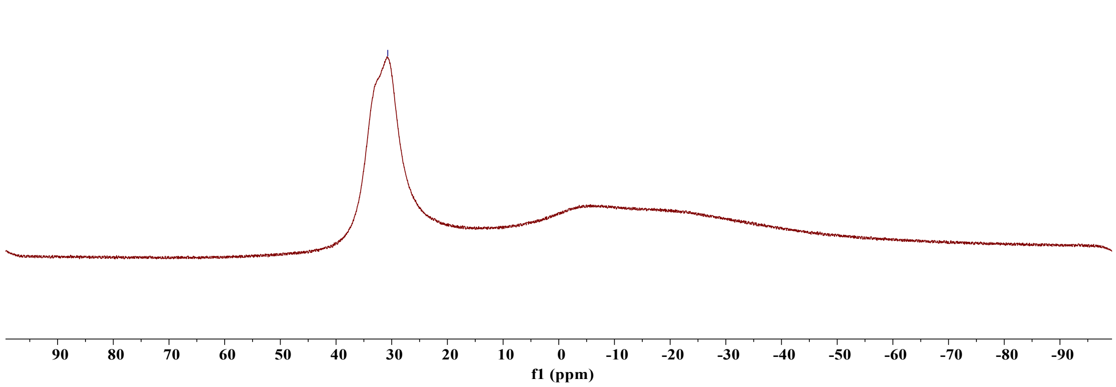

2,2'-(1,1-difluoro-4-(4-isopropylphenyl)but-1-ene-2,3-diyl)bis(4,4,5,5-tetramethyl-1,3,2-dioxaborolane) (57)

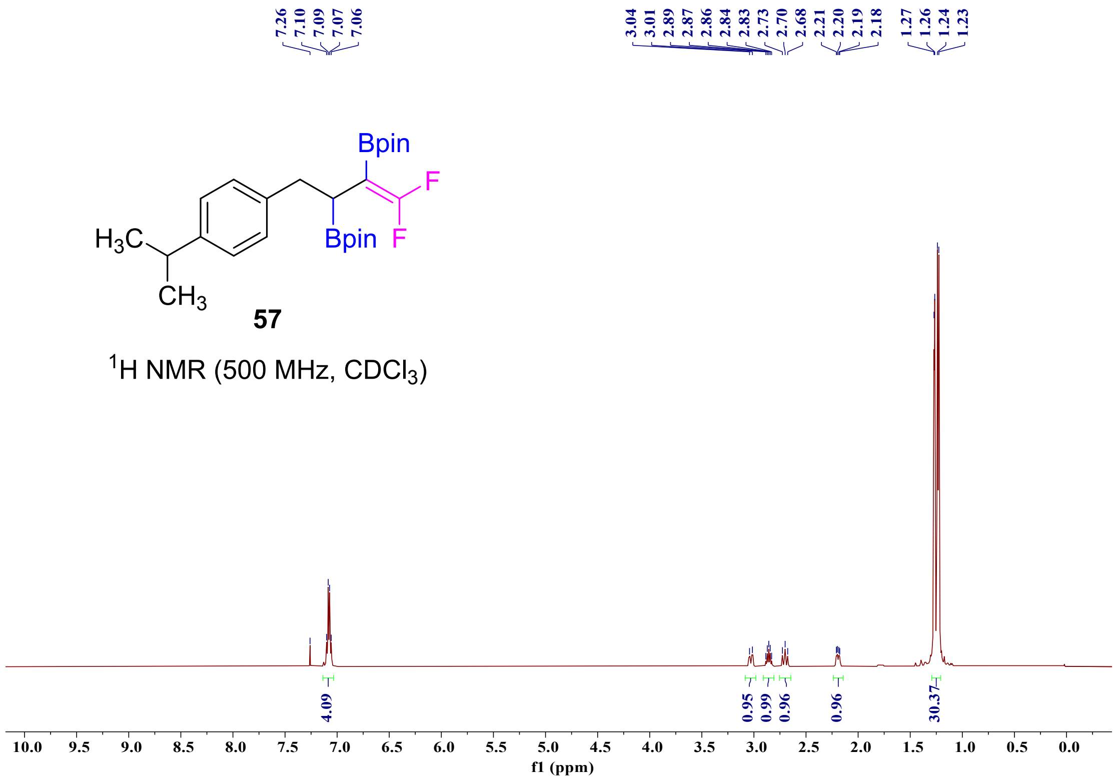

2,2'-(1,1-difluoro-4-(4-isopropylphenyl)but-1-ene-2,3-diyl)bis(4,4,5,5-tetramethyl-1,3,2-dioxaborolane) (57)

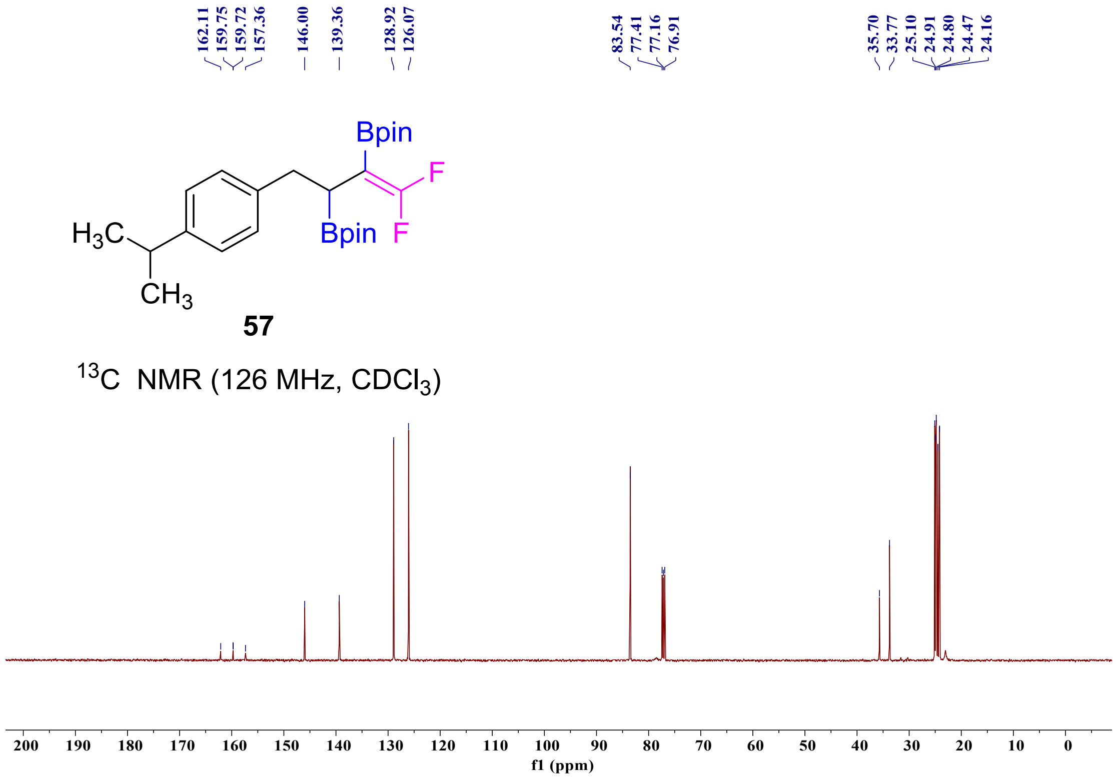

2,2'-(1,1-difluoro-4-(4-isopropylphenyl)but-1-ene-2,3-diyl)bis(4,4,5,5-tetramethyl-1,3,2-dioxaborolane) (57)

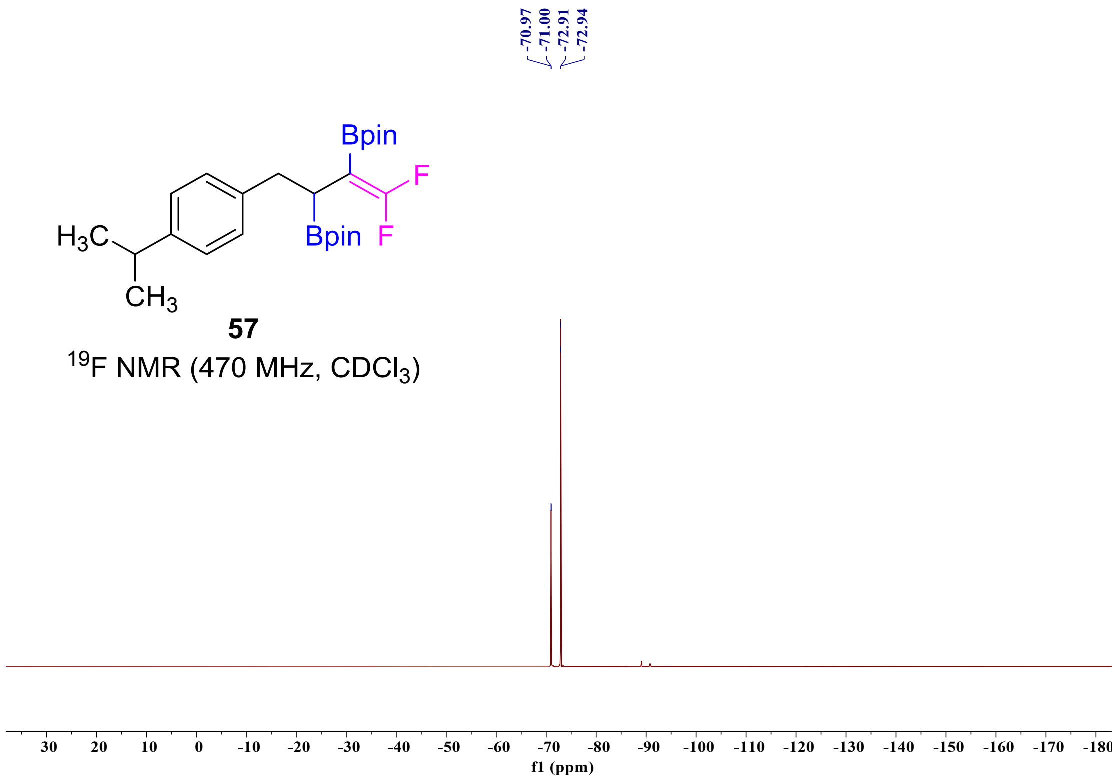

2,2'-(1,1-difluoro-4-(4-isopropylphenyl)but-1-ene-2,3-diyl)bis(4,4,5,5-tetramethyl-1,3,2-dioxaborolane) (57)

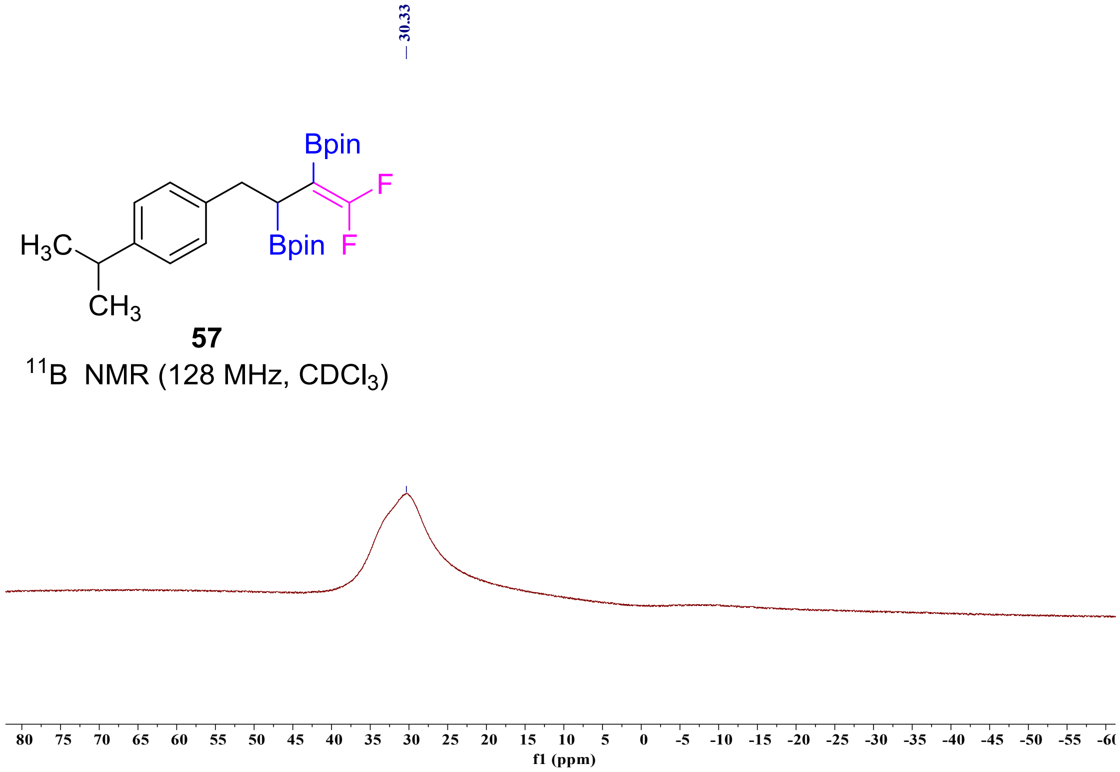

2,2'-(4-(3-chlorophenyl)-1,1-difluorobut-1-ene-2,3-diyl)bis(4,4,5,5-tetramethyl-1,3,2-dioxaborolane) (58)

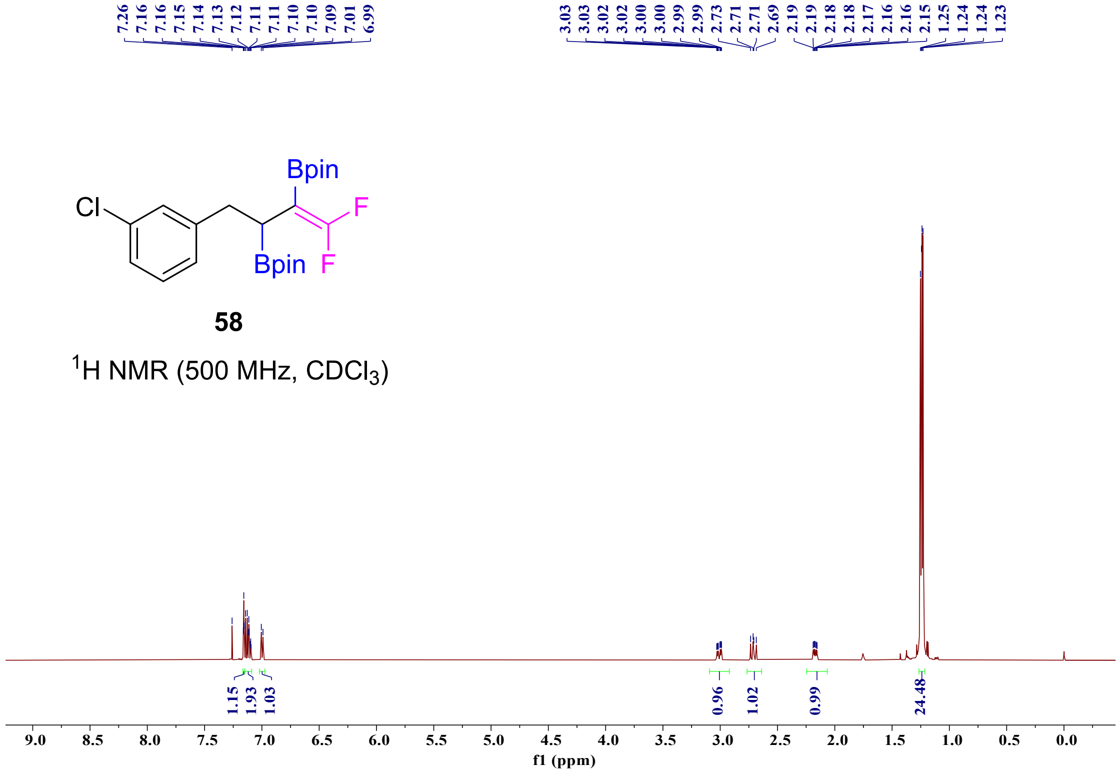

2,2'-(4-(3-chlorophenyl)-1,1-difluorobut-1-ene-2,3-diyl)bis(4,4,5,5-tetramethyl-1,3,2-dioxaborolane) (58)

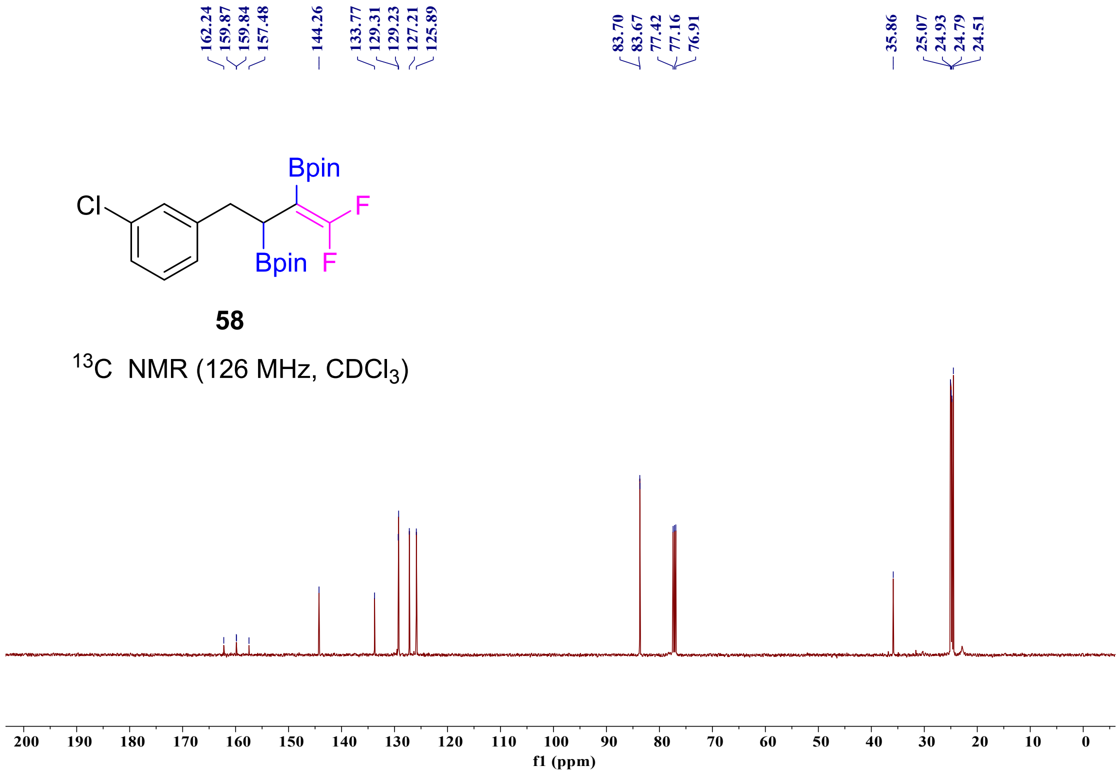

2,2'-(4-(3-chlorophenyl)-1,1-difluorobut-1-ene-2,3-diyl)bis(4,4,5,5-tetramethyl-1,3,2-dioxaborolane) (58)

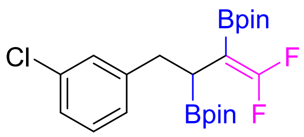

58

^{19}F NMR (470 MHz, CDCl$_3$)

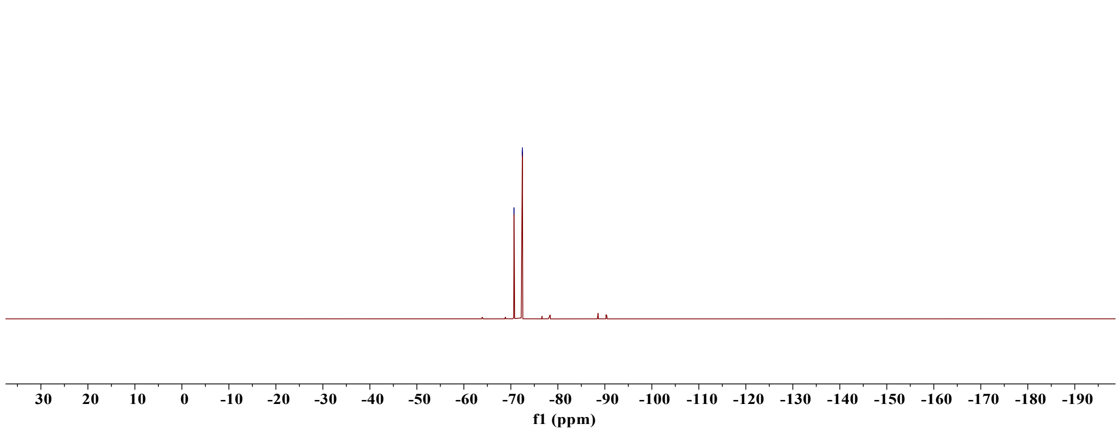

2,2'-(4-(3-chlorophenyl)-1,1-difluorobut-1-ene-2,3-diyl)bis(4,4,5,5-tetramethyl-1,3,2-dioxaborolane) (58)

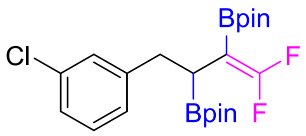

58

^{11}B NMR (128 MHz, CDCl$_3$)

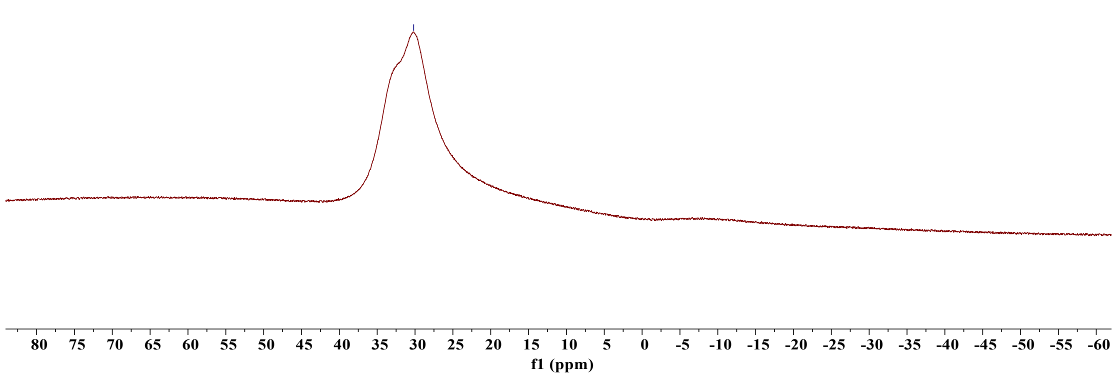

4-(4,4-difluoro-2,3-bis(4,4,5,5-tetramethyl-1,3,2-dioxaborolan-2-yl)but-3-en-1-yl)benzonitrile(59)

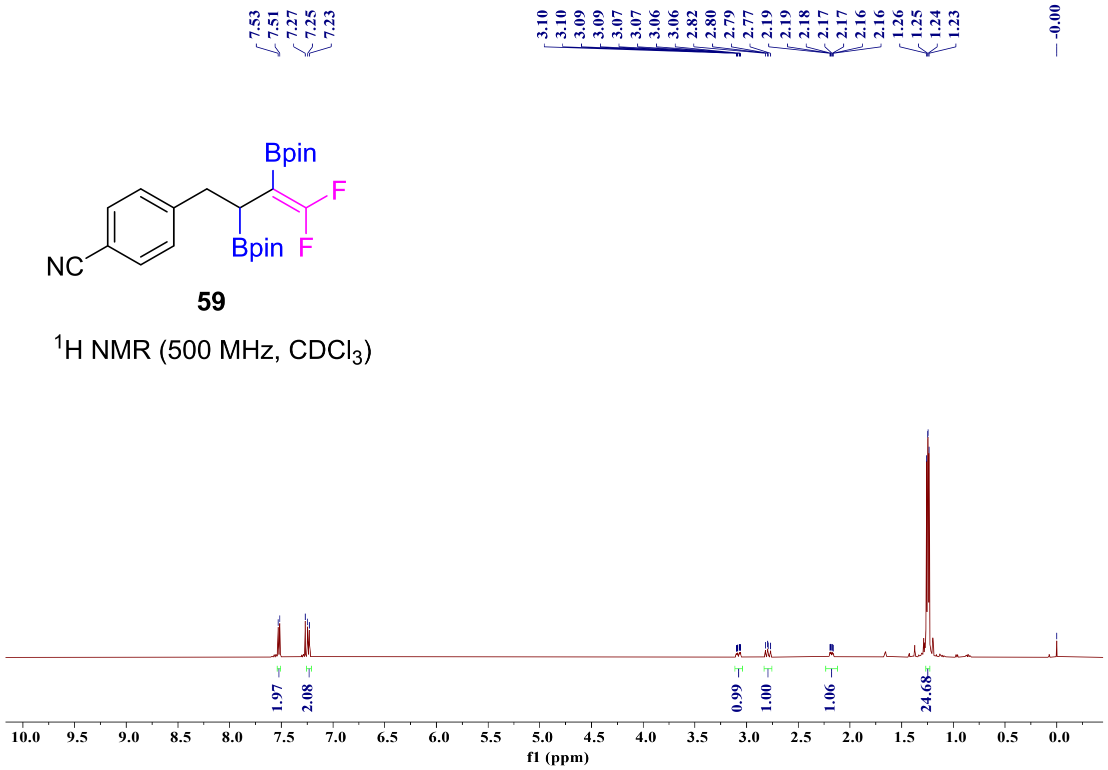

4-(4,4-difluoro-2,3-bis(4,4,5,5-tetramethyl-1,3,2-dioxaborolan-2-yl)but-3-en-1-yl)benzonitrile(59)

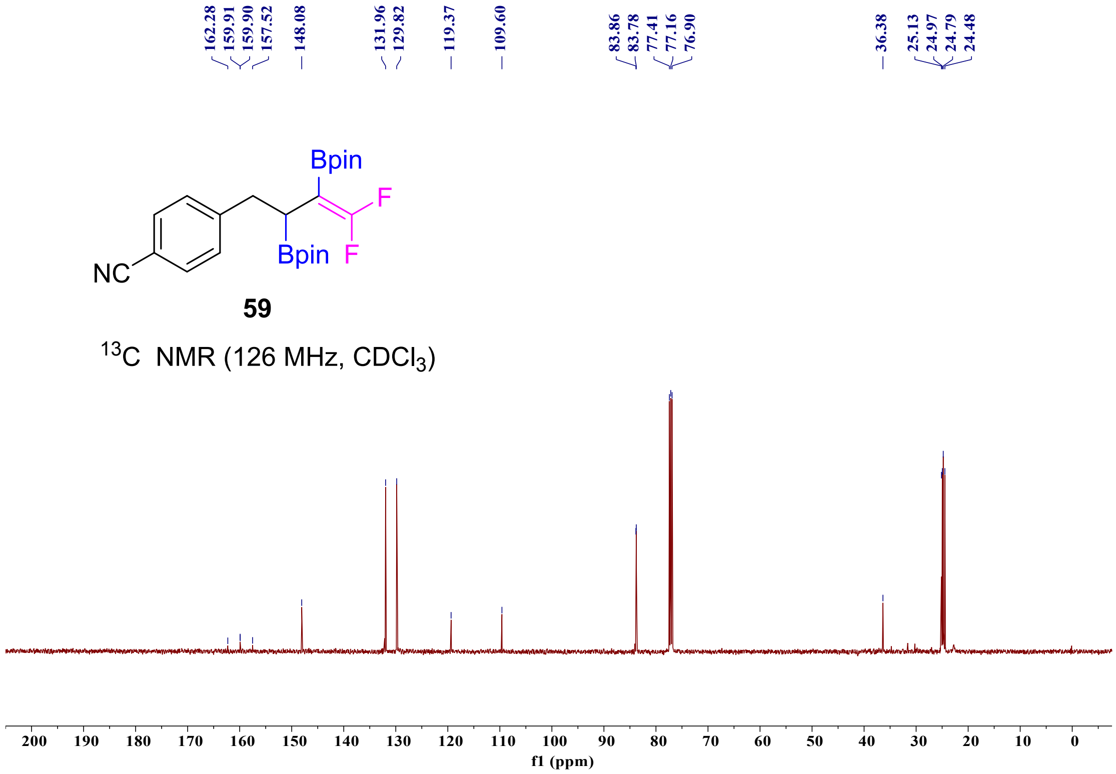

4-(4,4-difluoro-2,3-bis(4,4,5,5-tetramethyl-1,3,2-dioxaborolan-2-yl)but-3-en-1-yl)benzonitrile(59)

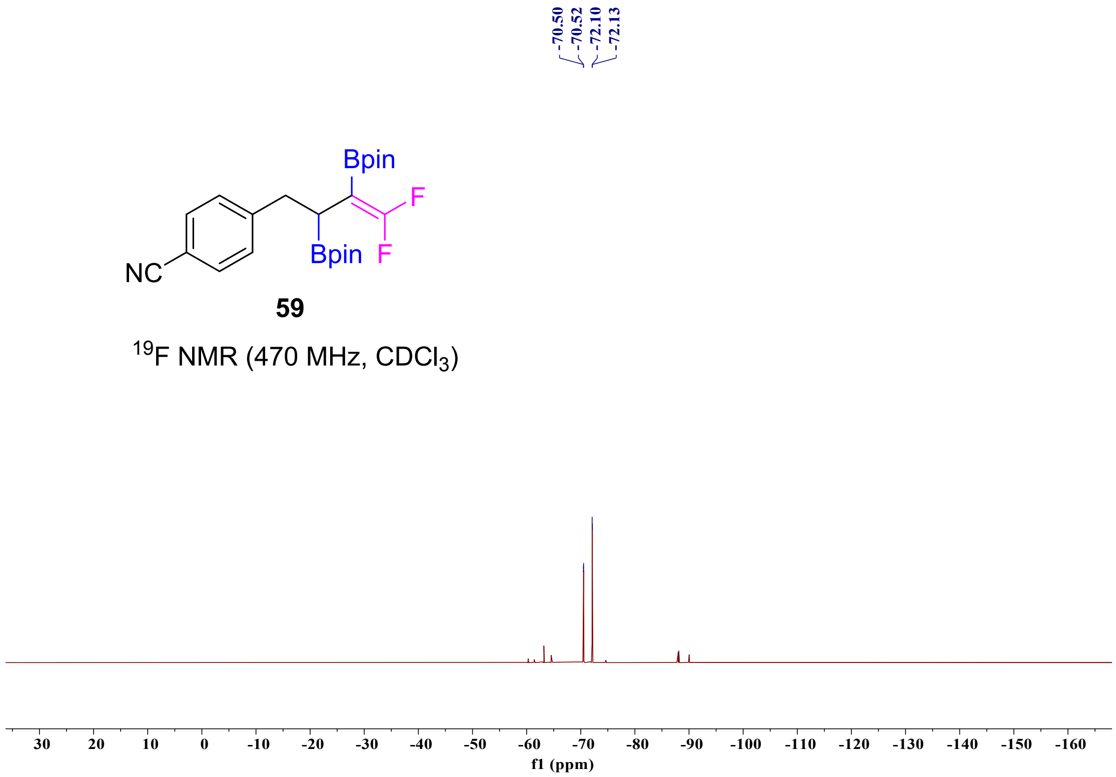

4-(4,4-difluoro-2,3-bis(4,4,5,5-tetramethyl-1,3,2-dioxaborolan-2-yl)but-3-en-1-yl)benzonitrile(59)

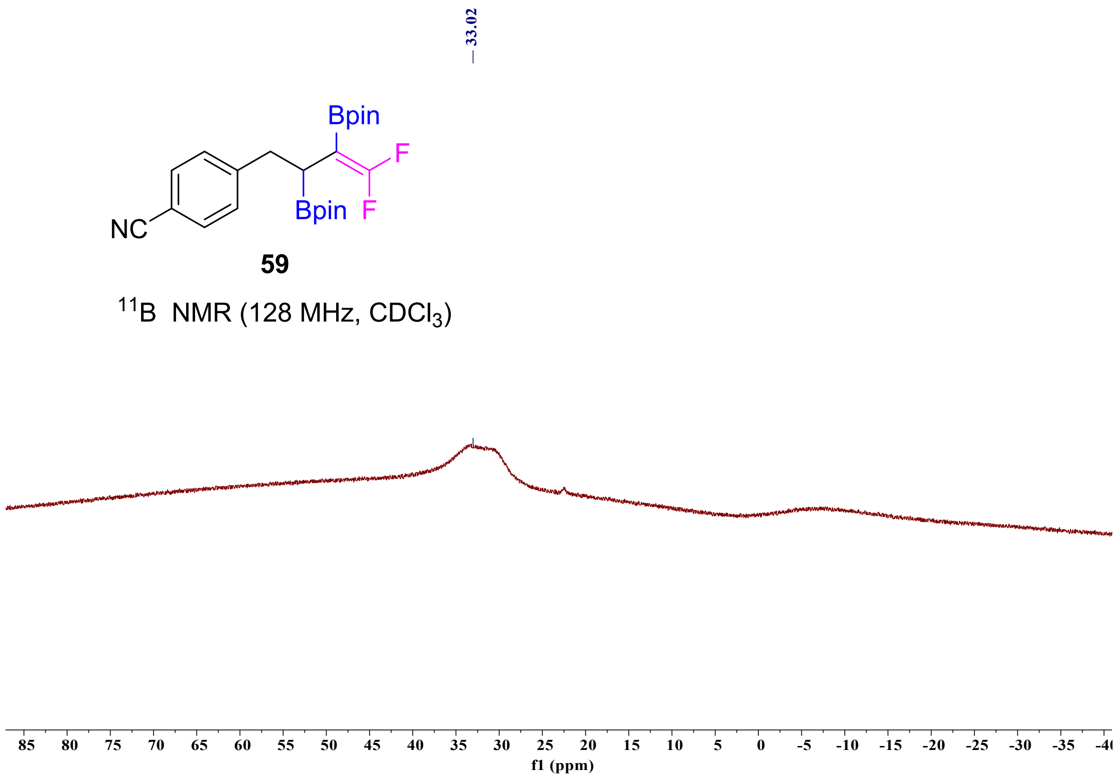

(S)-2,2'-(1,1-difluoro-5-phenylpent-1-ene-2,3-diyl)bis(4,4,5,5-tetramethyl-1,3,2-dioxaborolane) (60)

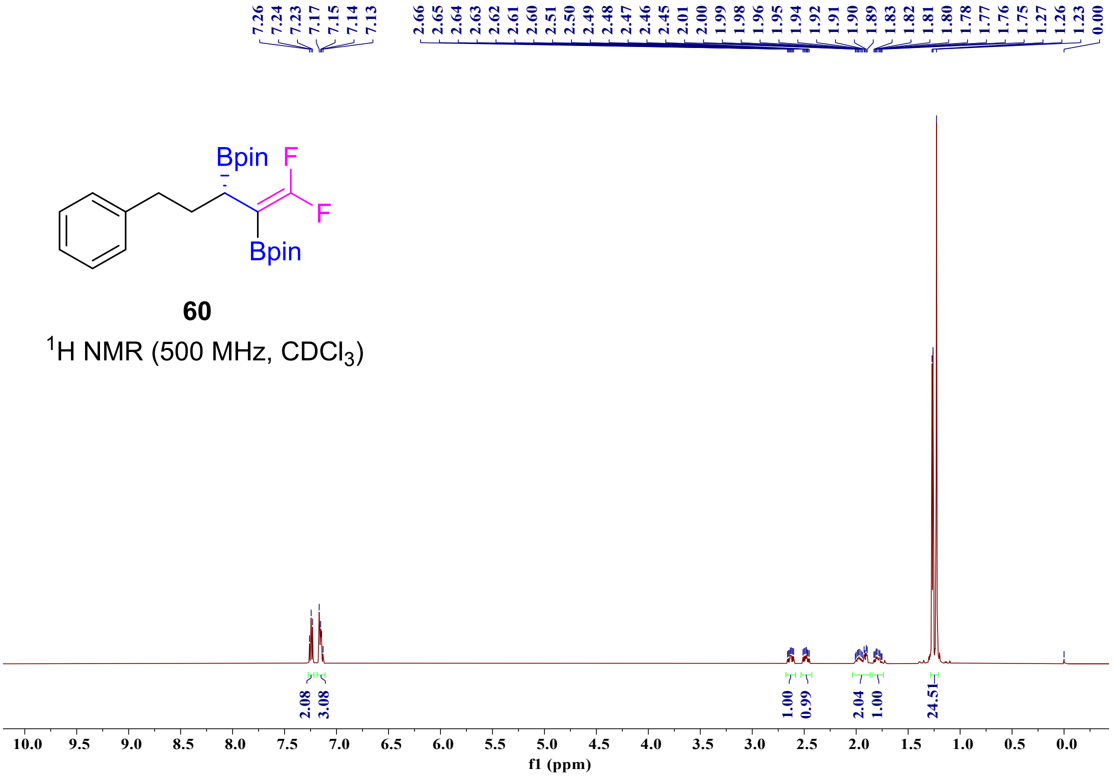

(S)-2,2'-(1,1-difluoro-5-phenylpent-1-ene-2,3-diyl)bis(4,4,5,5-tetramethyl-1,3,2-dioxaborolane) (60)

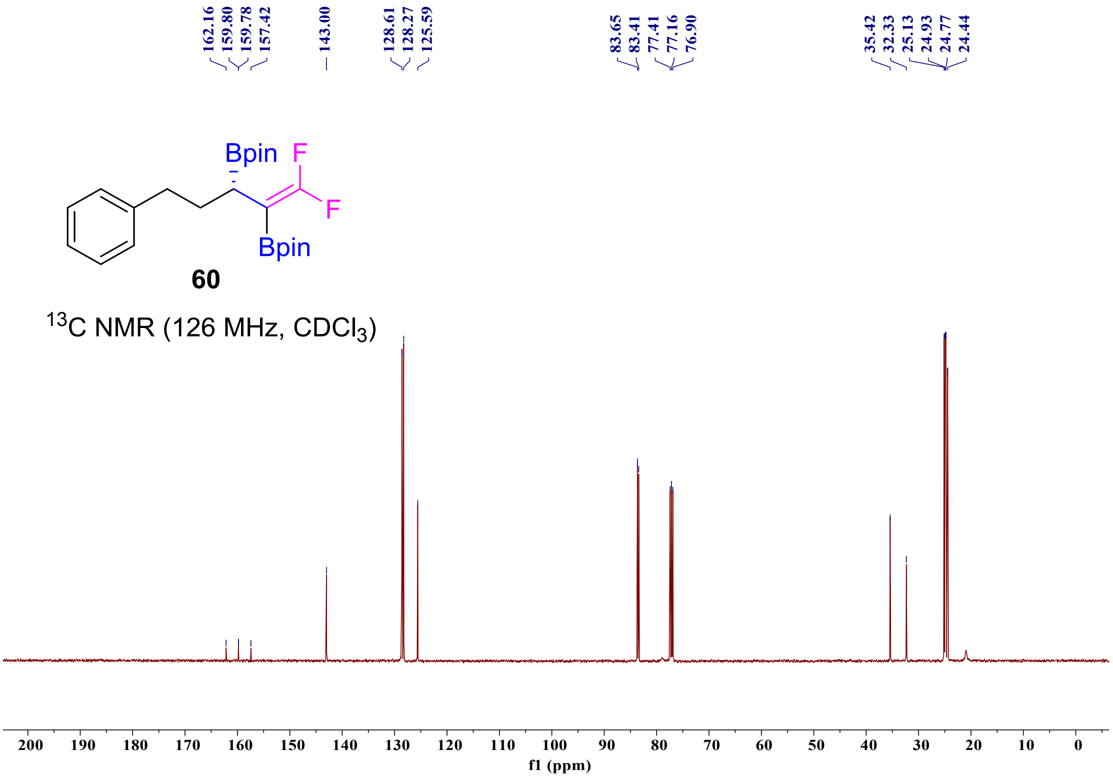

(S)-2,2'-(1,1-difluoro-5-phenylpent-1-ene-2,3-diyl)bis(4,4,5,5-tetramethyl-1,3,2-dioxaborolane) (60)

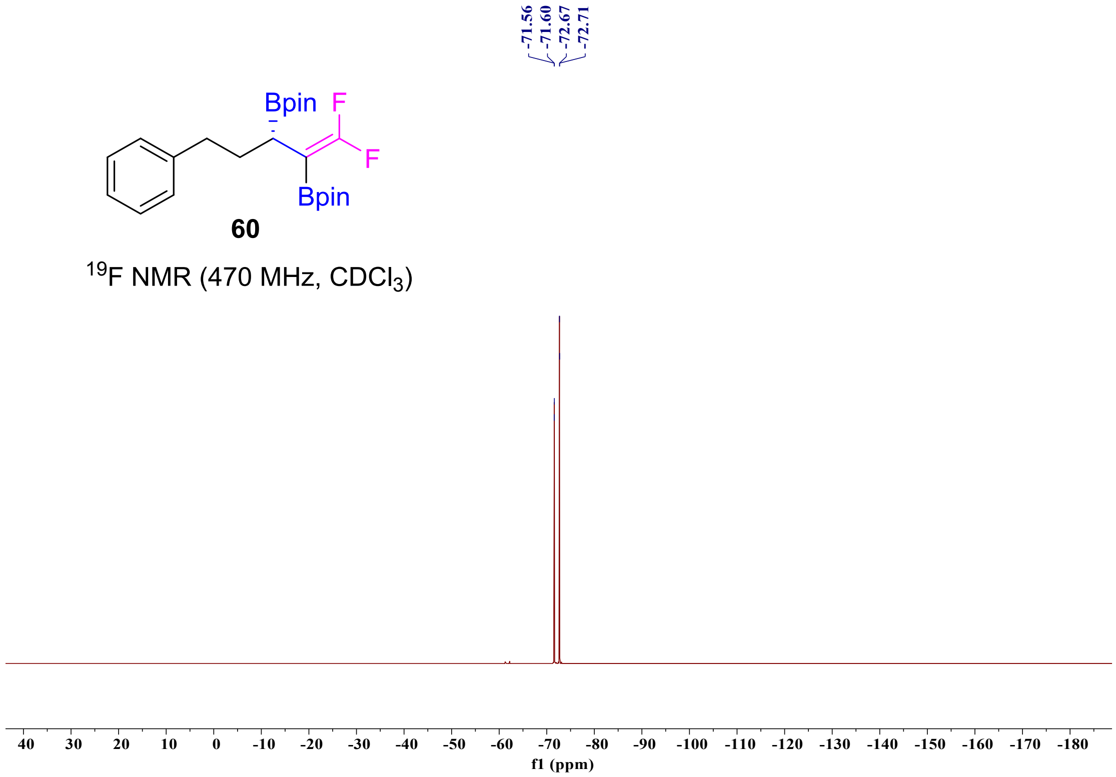

(S)-2,2'-(1,1-difluoro-5-phenylpent-1-ene-2,3-diyl)bis(4,4,5,5-tetramethyl-1,3,2-dioxaborolane) (60)

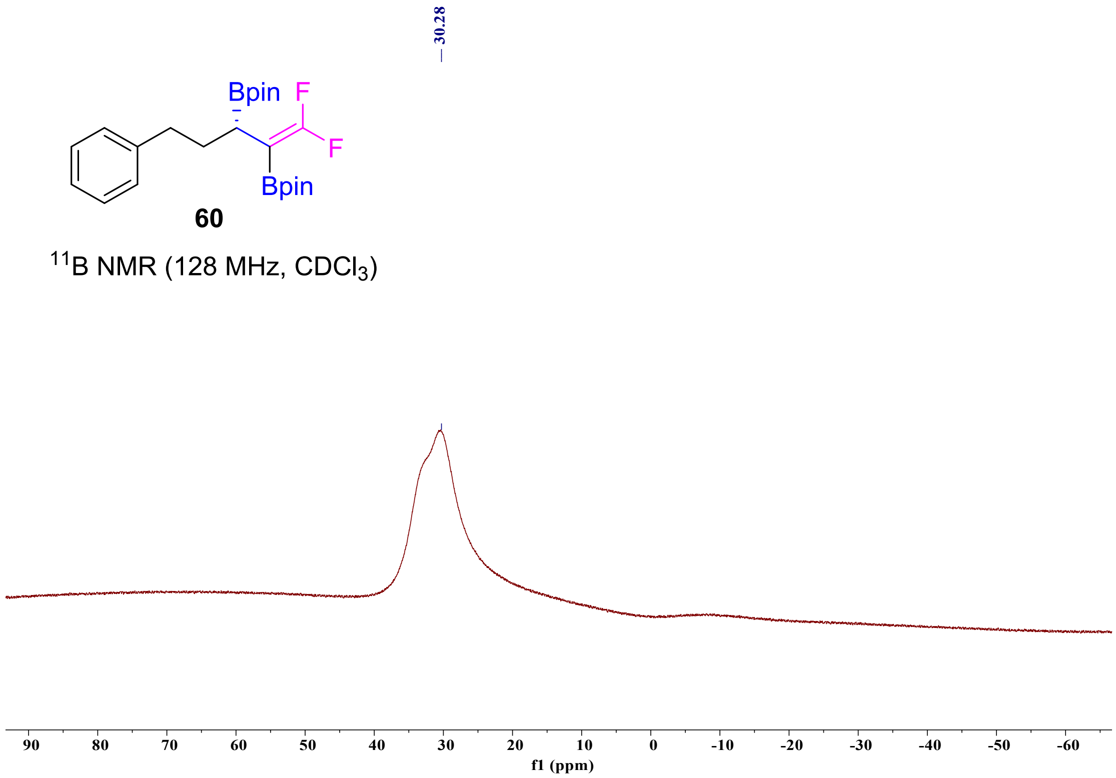

(S)-2,2'-(1,1-difluoro-5-(p-tolyl)pent-1-ene-2,3-diyl)bis(4,4,5,5-tetramethyl-1,3,2-dioxaborolane)(61)

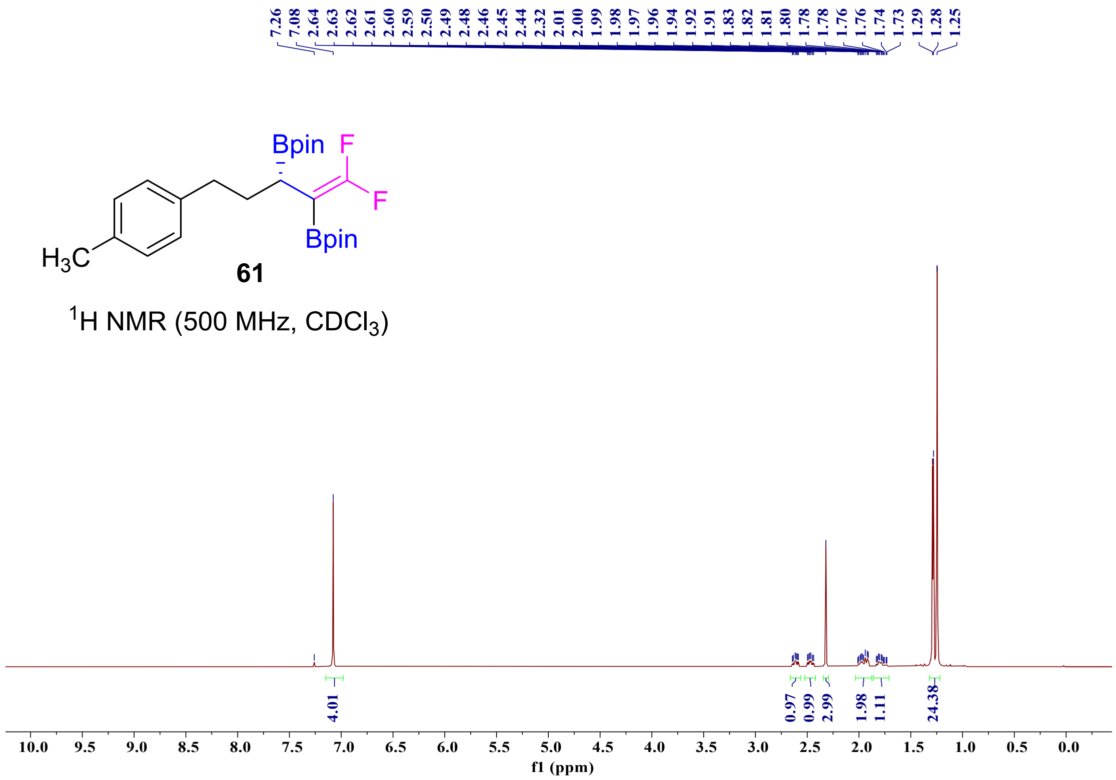

(S)-2,2'-(1,1-difluoro-5-(p-tolyl)pent-1-ene-2,3-diyl)bis(4,4,5,5-tetramethyl-1,3,2-dioxaborolane)(61)

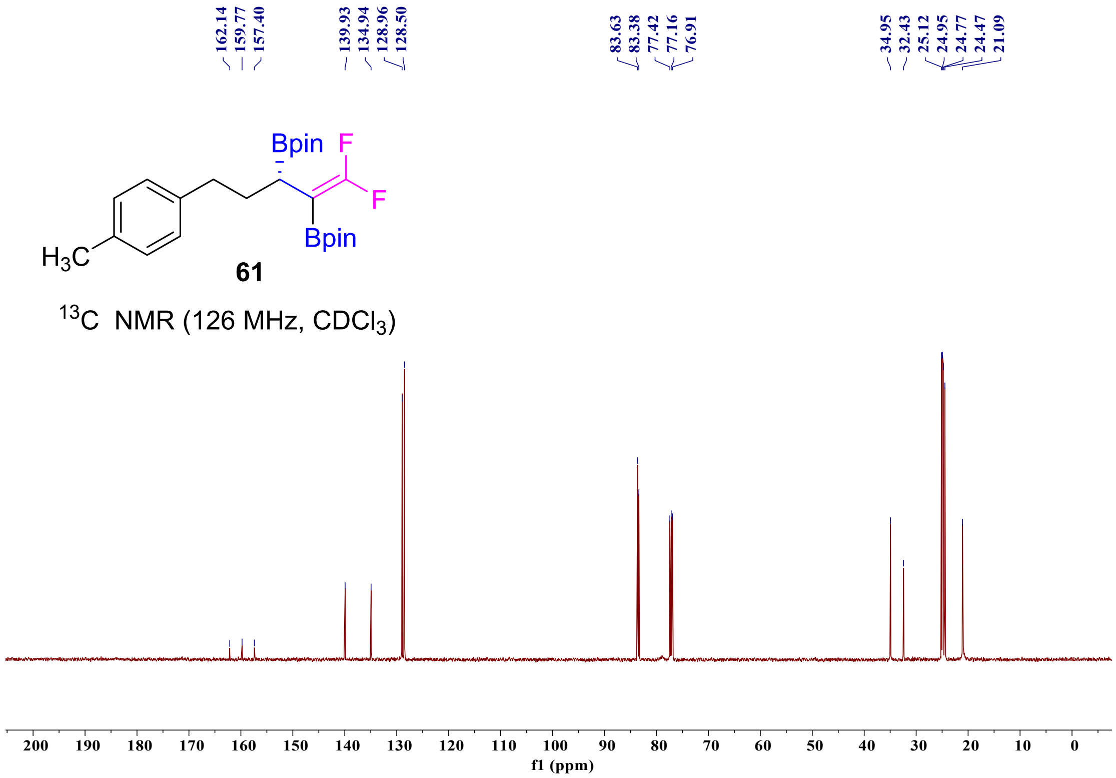

(S)-2,2'-(1,1-difluoro-5-(p-tolyl)pent-1-ene-2,3-diyl)bis(4,4,5,5-tetramethyl-1,3,2-dioxaborolane)(61)

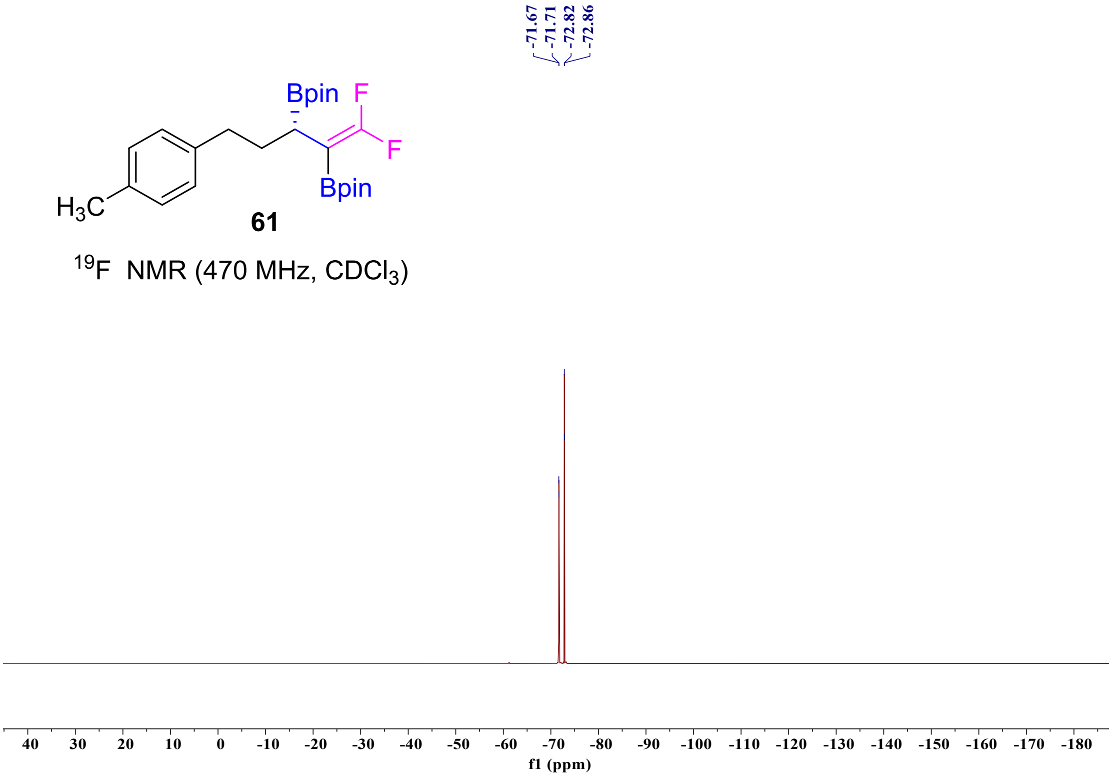

(S)-2,2'-(1,1-difluoro-5-(p-tolyl)pent-1-ene-2,3-diyl)bis(4,4,5,5-tetramethyl-1,3,2-dioxaborolane)(61)

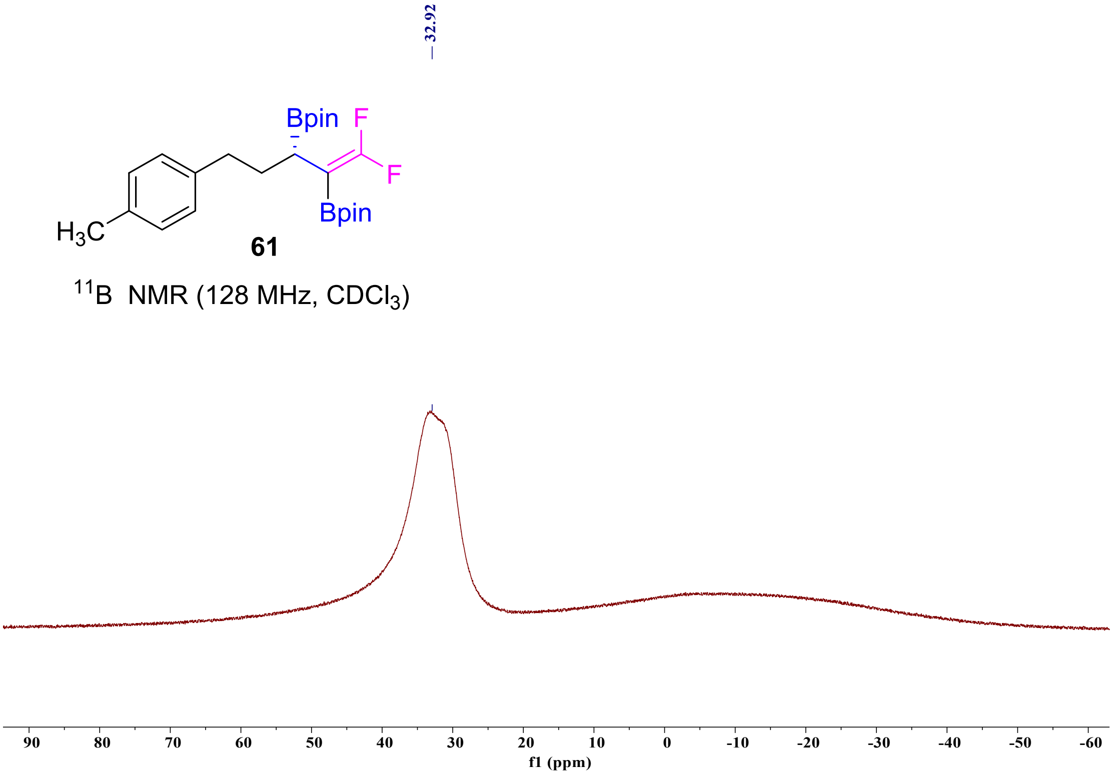

(S)-2,2'-(1,1-difluoro-5-(4-methoxyphenyl)pent-1-ene-2,3-diyl)bis(4,4,5,5-tetramethyl-1,3,2-dioxaborolane) (62)

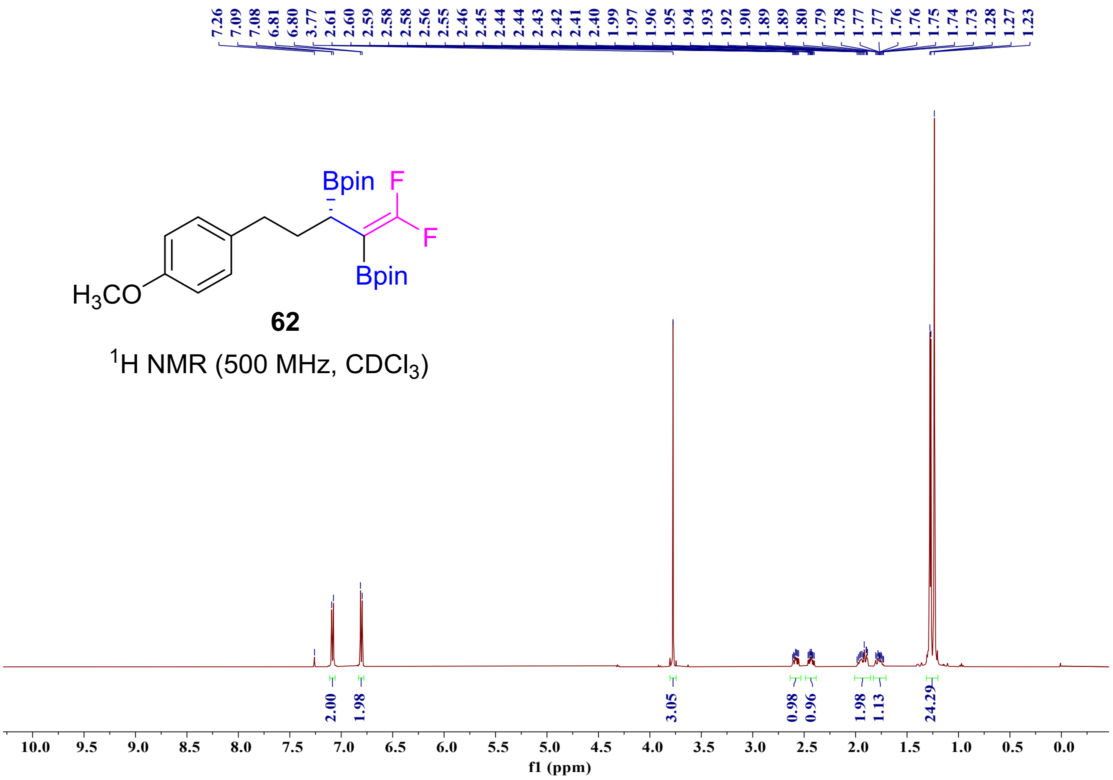

(S)-2,2'-(1,1-difluoro-5-(4-methoxyphenyl)pent-1-ene-2,3-diyl)bis(4,4,5,5-tetramethyl-1,3,2-dioxaborolane) (62)

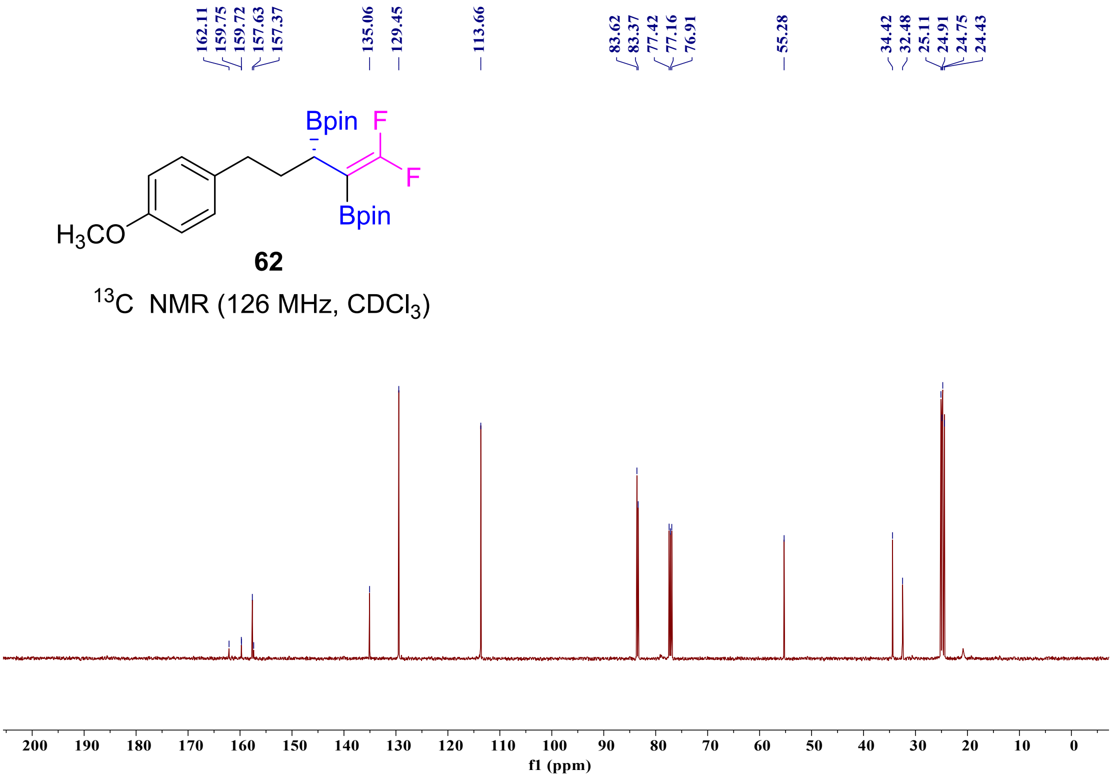

(S)-2,2'-(1,1-difluoro-5-(4-methoxyphenyl)pent-1-ene-2,3-diyl)bis(4,4,5,5-tetramethyl-1,3,2-dioxaborolane) (62)

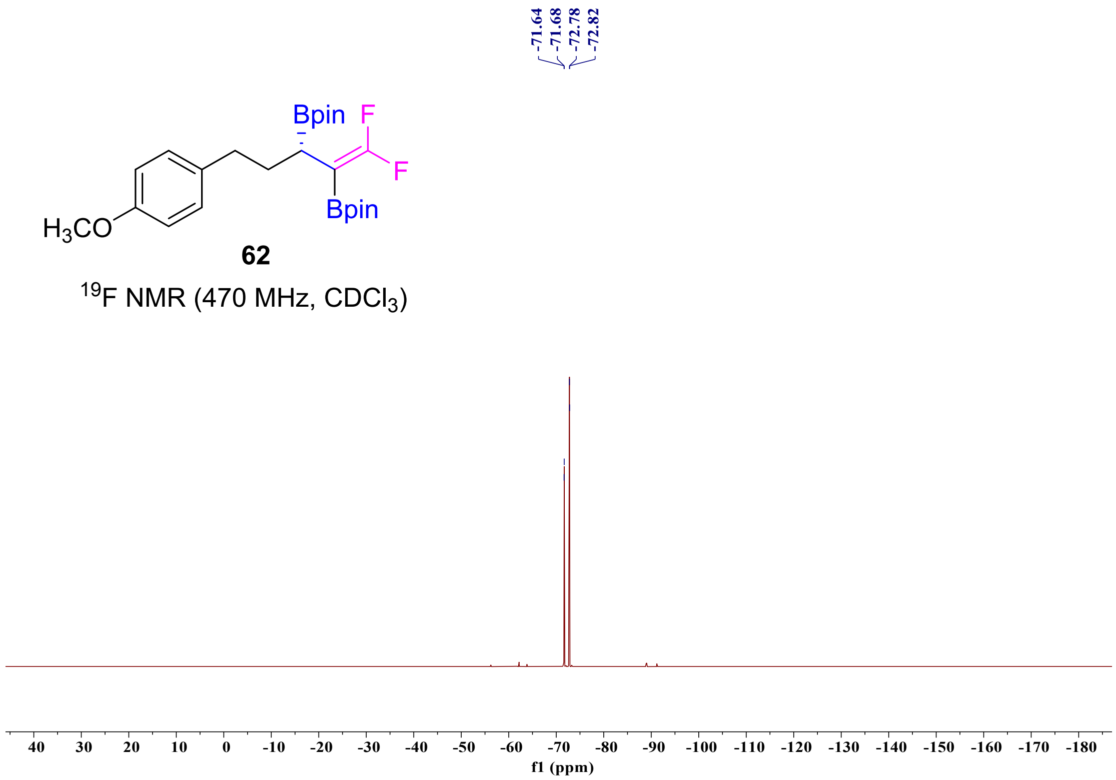

(S)-2,2'-(1,1-difluoro-5-(4-methoxyphenyl)pent-1-ene-2,3-diyl)bis(4,4,5,5-tetramethyl-1,3,2-dioxaborolane) (62)

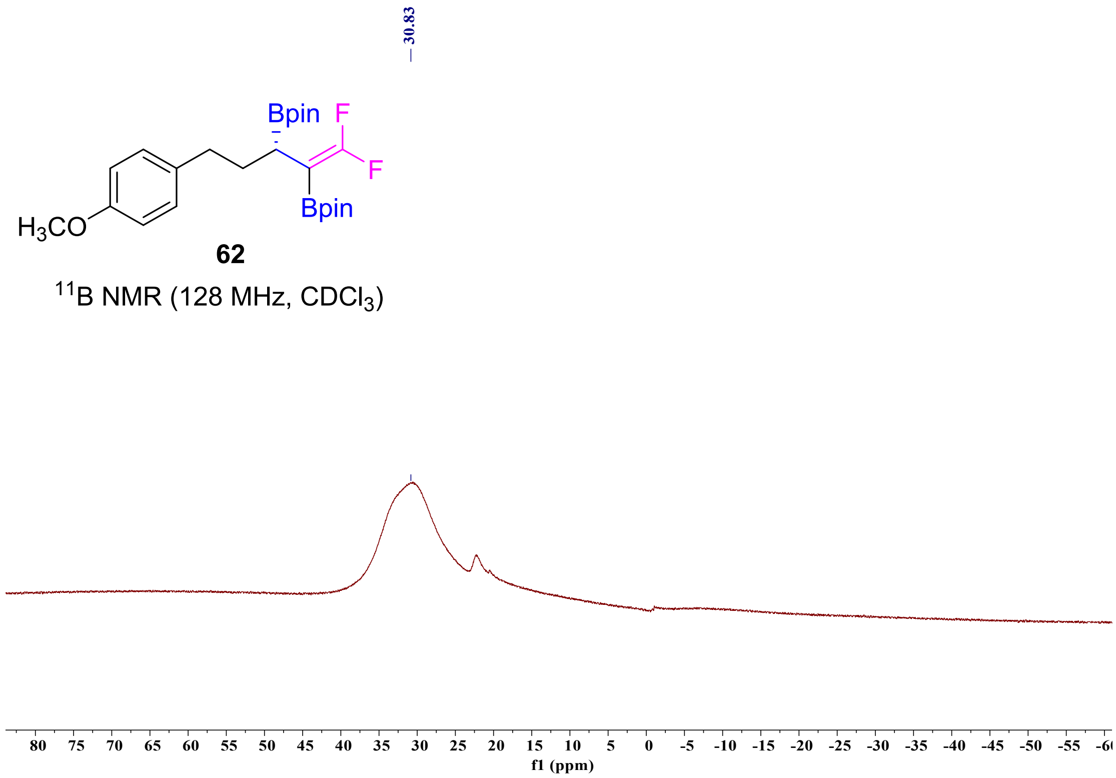

(S)2,2'-(1,1-difluoro-5-(4-fluorophenyl)pent-1-ene-2,3-diyl)bis(4,4,5,5-tetramethyl-1,3,2-dioxaborolane) (63)

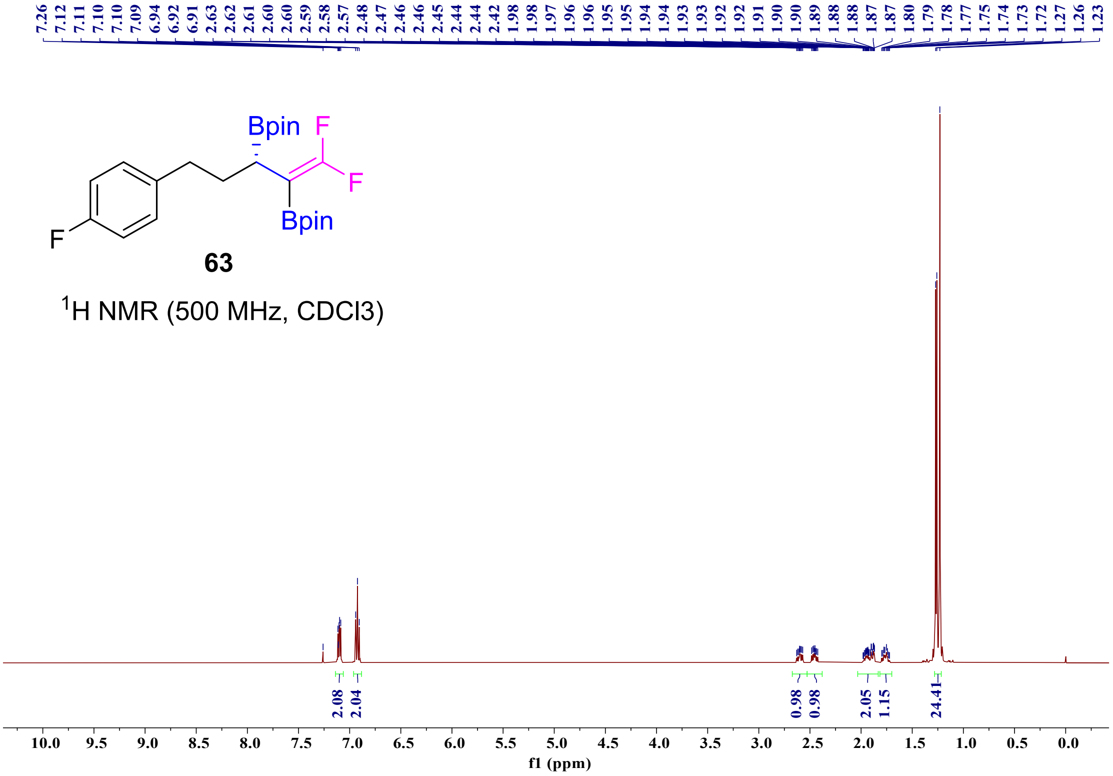

(S)2,2'-(1,1-difluoro-5-(4-fluorophenyl)pent-1-ene-2,3-diyl)bis(4,4,5,5-tetramethyl-1,3,2-dioxaborolane) (63)

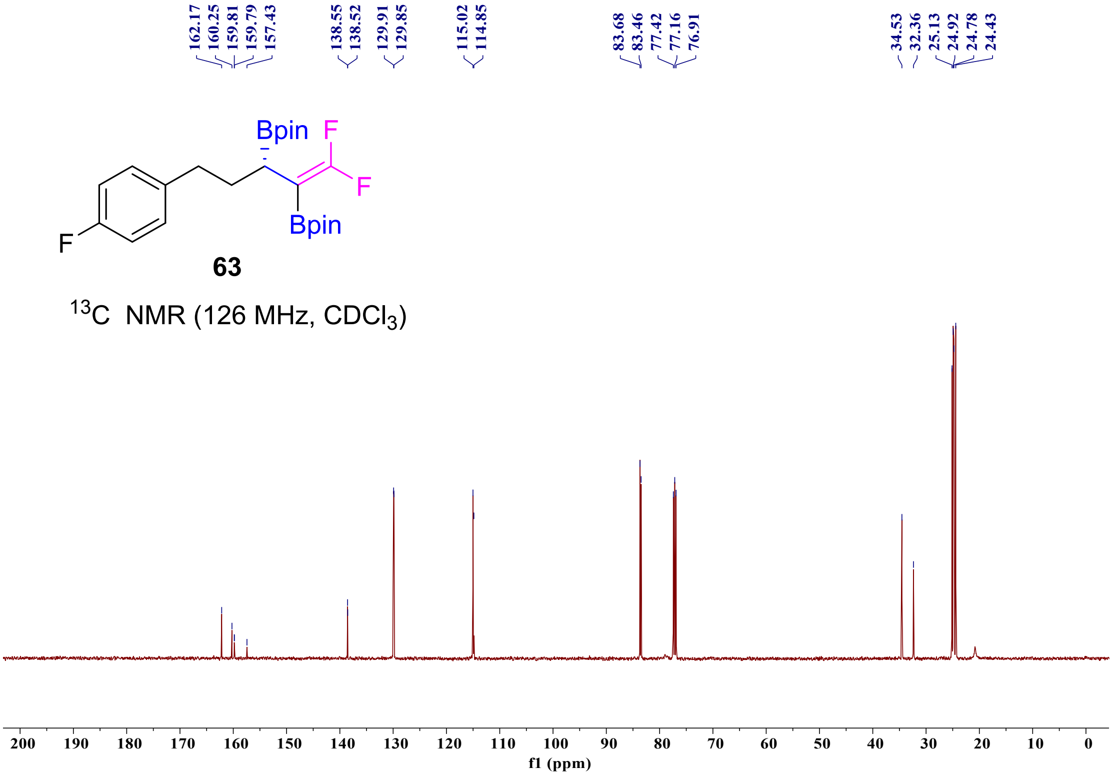

(S)2,2'-(1,1-difluoro-5-(4-fluorophenyl)pent-1-ene-2,3-diyl)bis(4,4,5,5-tetramethyl-1,3,2-dioxaborolane) (63)

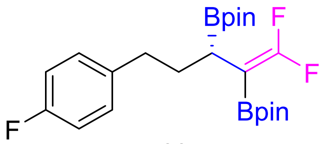

63

^{19}F NMR (470 MHz, $CDCl_3$)

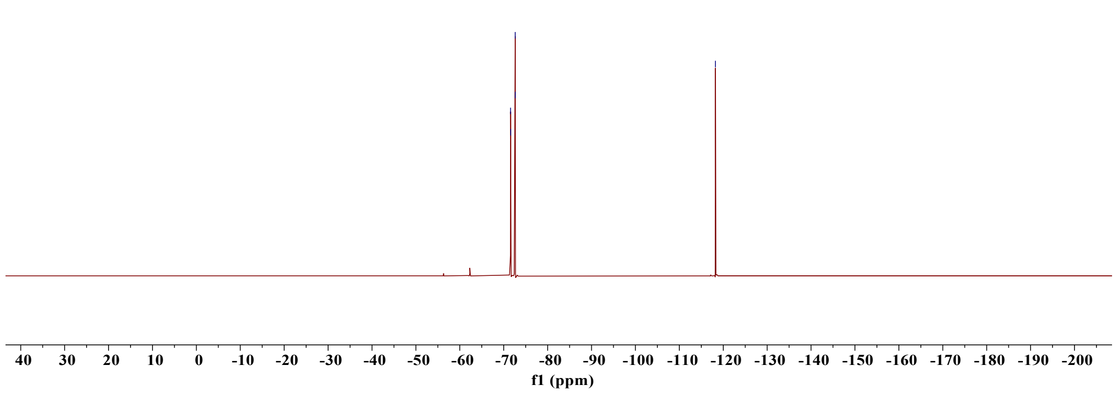

(S)2,2'-(1,1-difluoro-5-(4-fluorophenyl)pent-1-ene-2,3-diyl)bis(4,4,5,5-tetramethyl-1,3,2-dioxaborolane) (63)

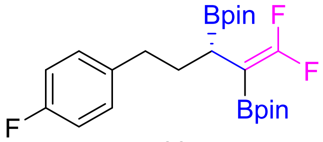

63

^{11}B NMR (128 MHz, $CDCl_3$)

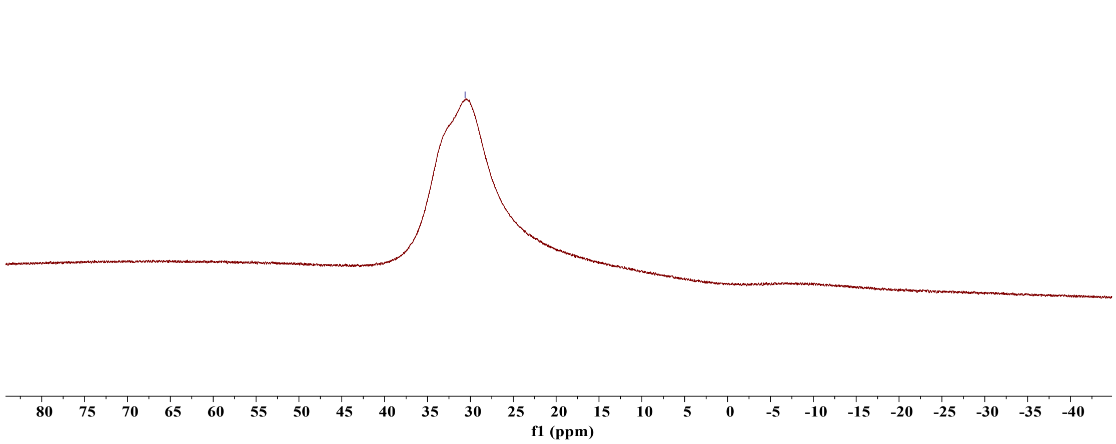

(S)-2,2'-(5-(4-chlorophenyl)-1,1-difluoropent-1-ene-2,3-diyl)bis(4,4,5,5-tetramethyl-1,3,2-dioxaborolane) (64)

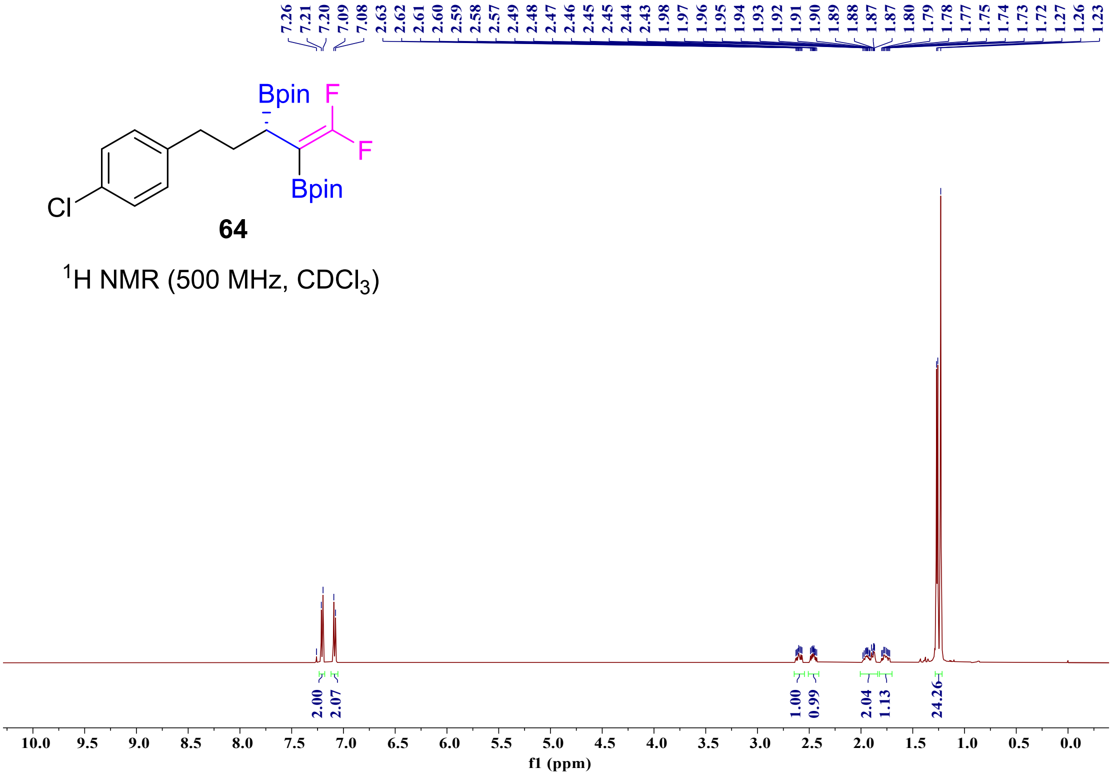

(S)-2,2'-(5-(4-chlorophenyl)-1,1-difluoropent-1-ene-2,3-diyl)bis(4,4,5,5-tetramethyl-1,3,2-dioxaborolane) (64)

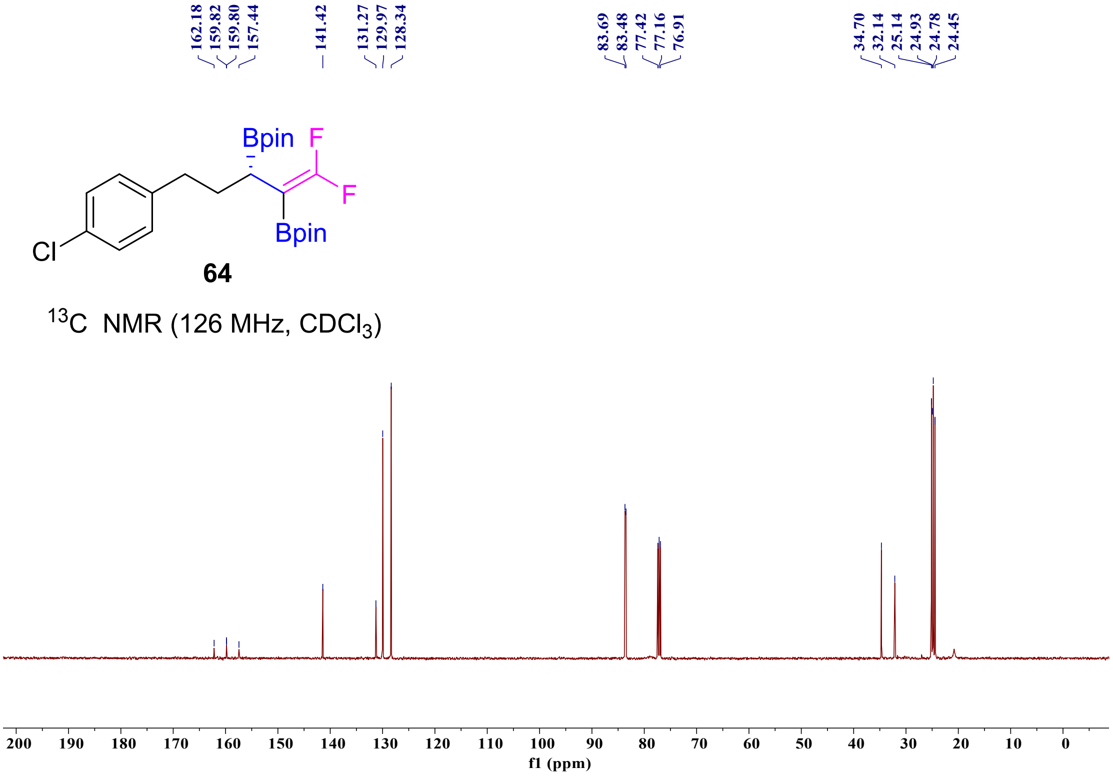

(S)-2,2'-(5-(4-chlorophenyl)-1,1-difluoropent-1-ene-2,3-diyl)bis(4,4,5,5-tetramethyl-1,3,2-dioxaborolane) (64)

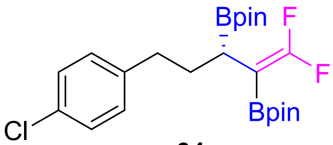

64

^{19}F NMR (470 MHz, $CDCl_3$)

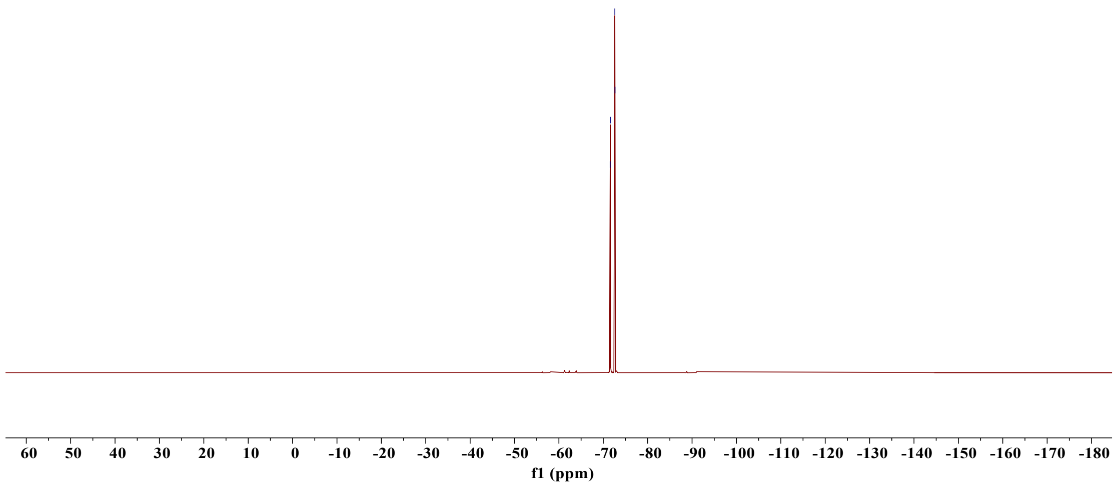

(S)-2,2'-(5-(4-chlorophenyl)-1,1-difluoropent-1-ene-2,3-diyl)bis(4,4,5,5-tetramethyl-1,3,2-dioxaborolane) (64)

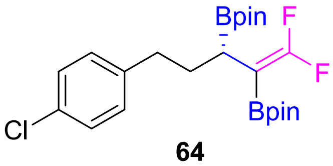

64

^{11}B NMR (128 MHz, $CDCl_3$)

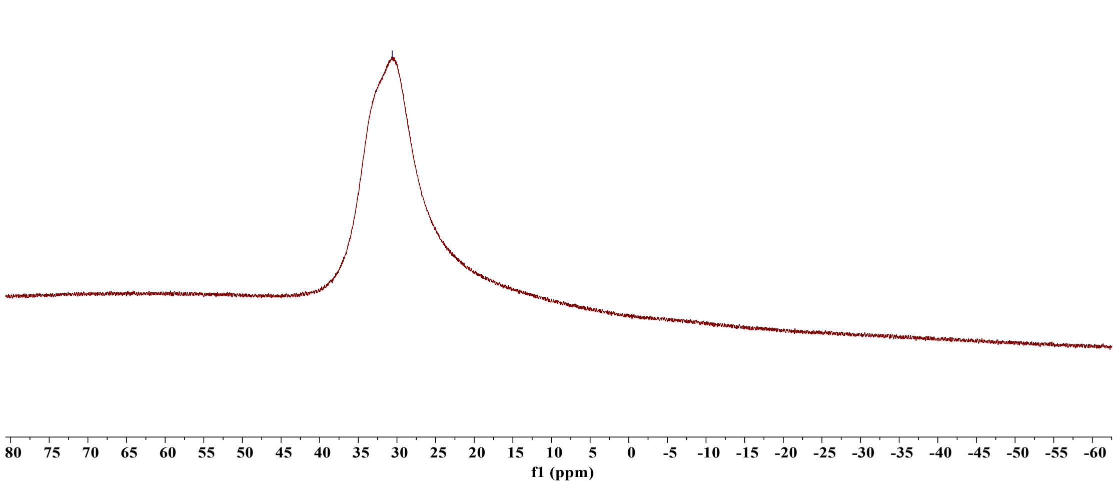

(S)-2,2'-(5-(4-bromophenyl)-1,1-difluoropent-1-ene-2,3-diyl)bis(4,4,5,5-tetramethyl-1,3,2-dioxaborolane) (65)

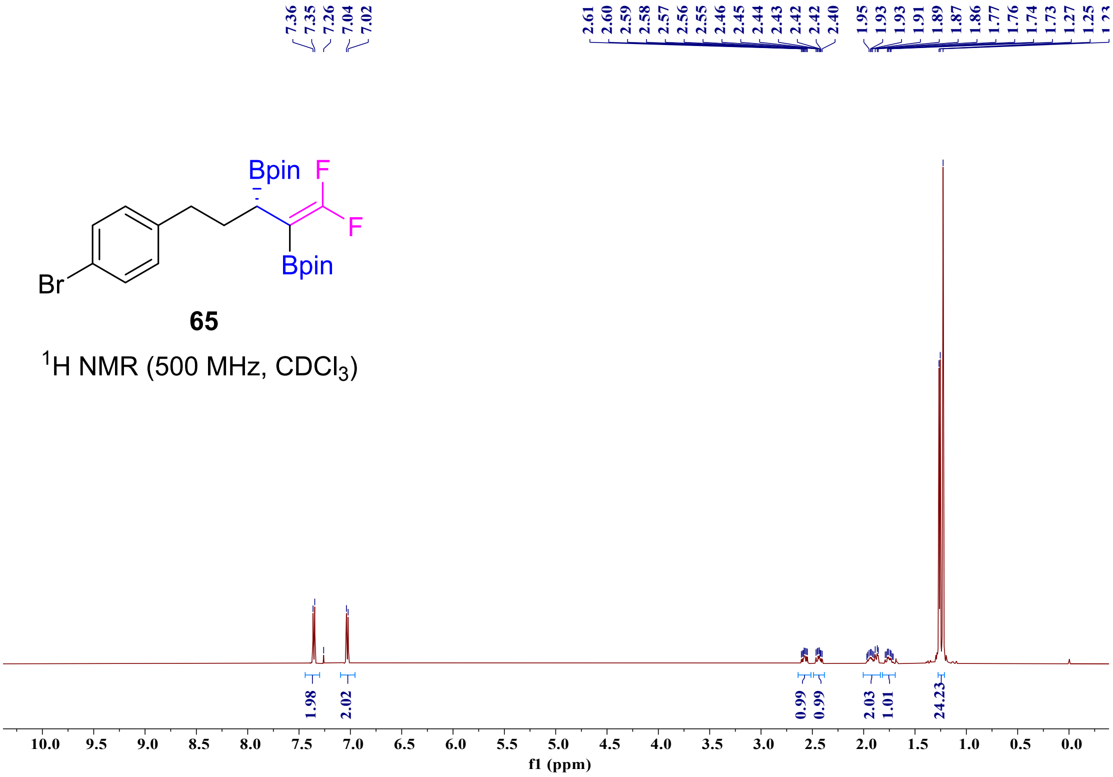

(S)-2,2'-(5-(4-bromophenyl)-1,1-difluoropent-1-ene-2,3-diyl)bis(4,4,5,5-tetramethyl-1,3,2-dioxaborolane) (65)

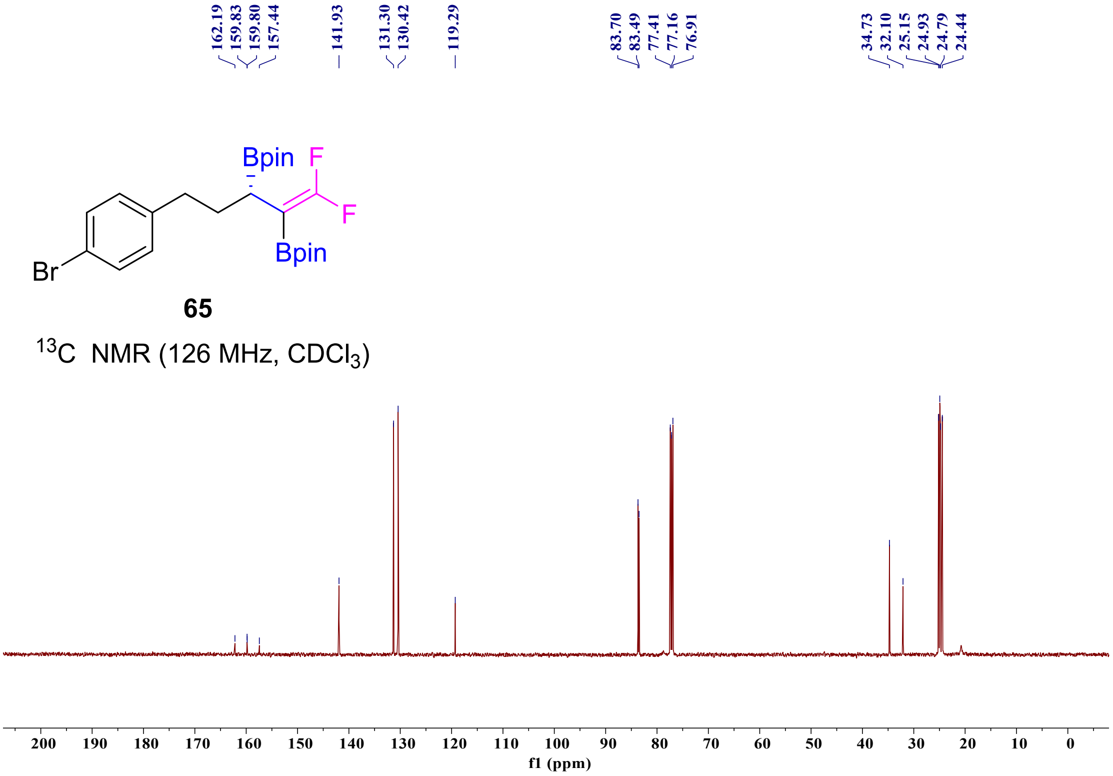

(S)-2,2'-(5-(4-bromophenyl)-1,1-difluoropent-1-ene-2,3-diyl)bis(4,4,5,5-tetramethyl-1,3,2-dioxaborolane) (65)

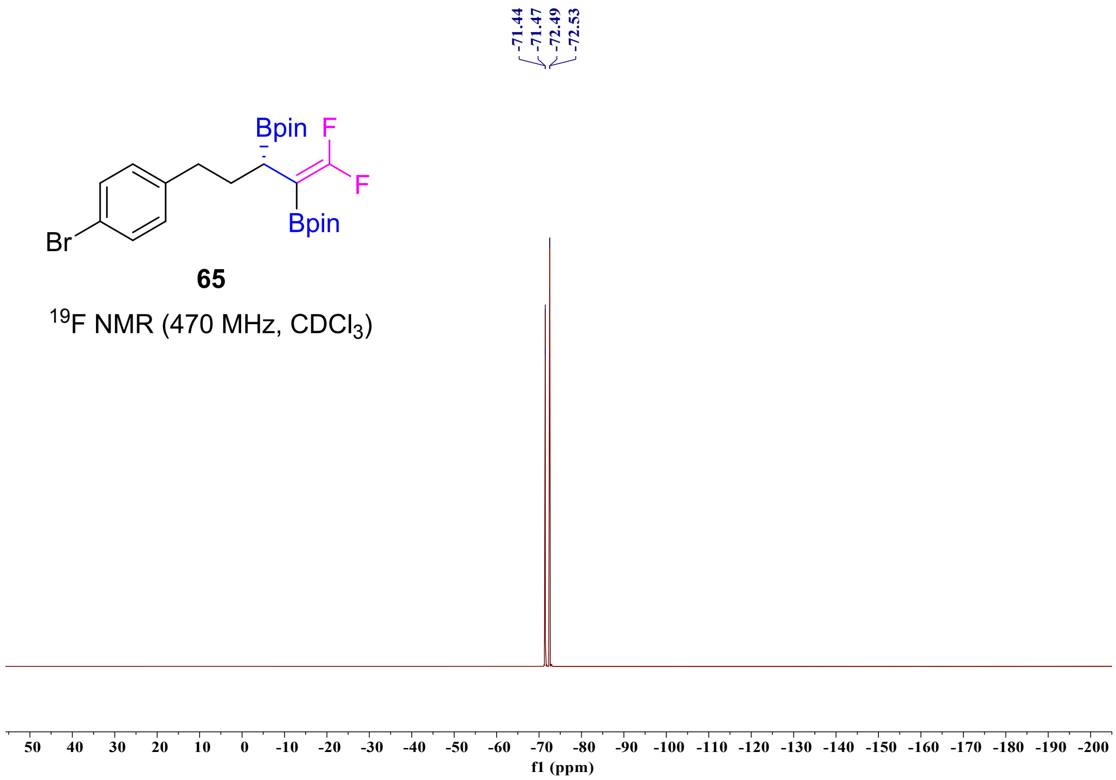

(S)-2,2'-(5-(4-bromophenyl)-1,1-difluoropent-1-ene-2,3-diyl)bis(4,4,5,5-tetramethyl-1,3,2-dioxaborolane) (65)

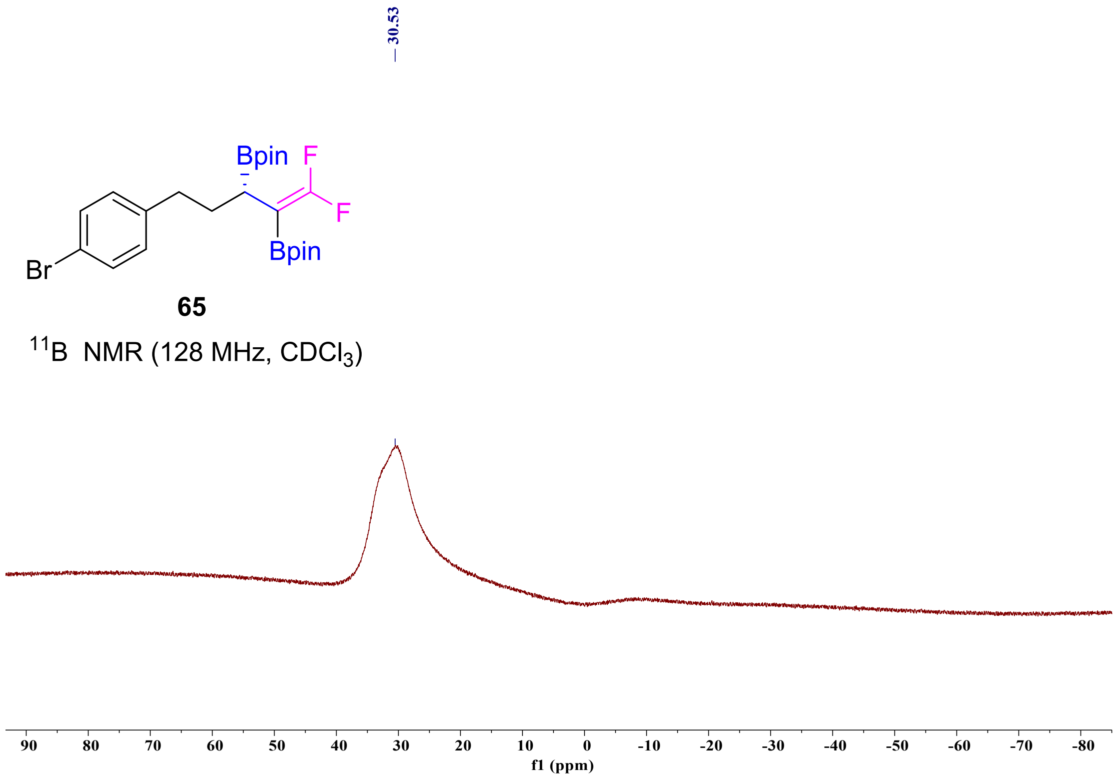

(S)4-(5,5-difluoro-3,4-bis(4,4,5,5-tetramethyl-1,3,2-dioxaborolan-2-yl)pent-4-en-1-yl)phenyl acetate (66)

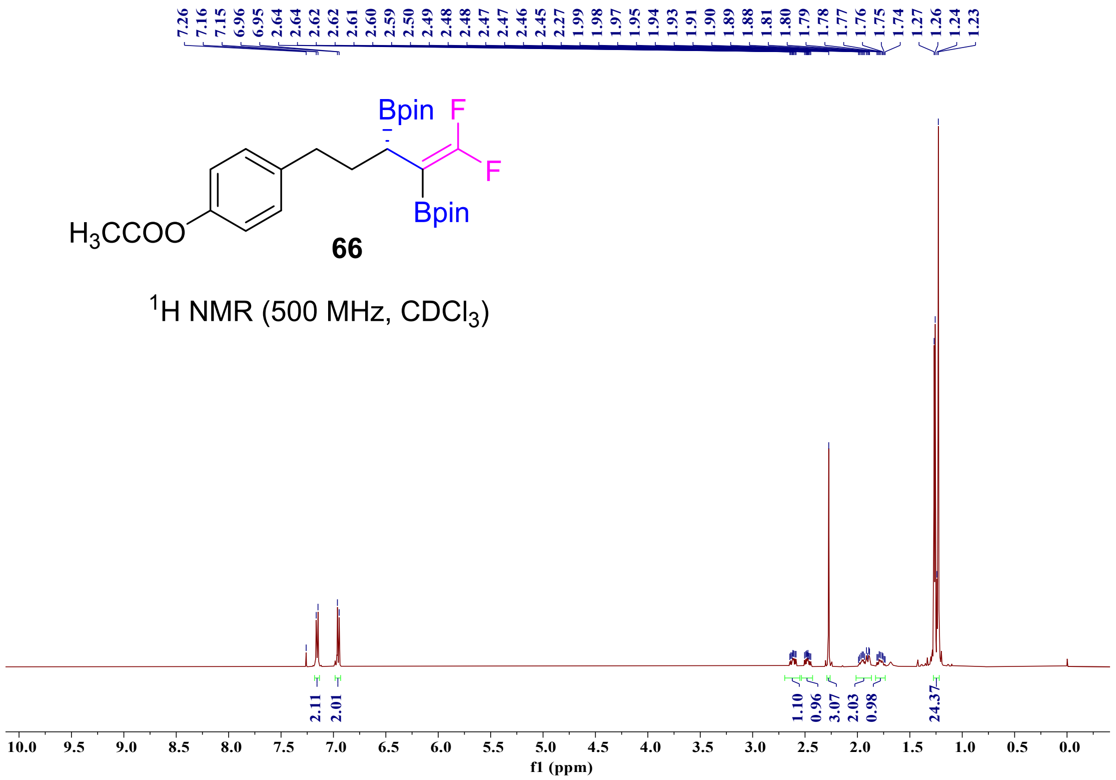

(S)4-(5,5-difluoro-3,4-bis(4,4,5,5-tetramethyl-1,3,2-dioxaborolan-2-yl)pent-4-en-1-yl)phenyl acetate(66)

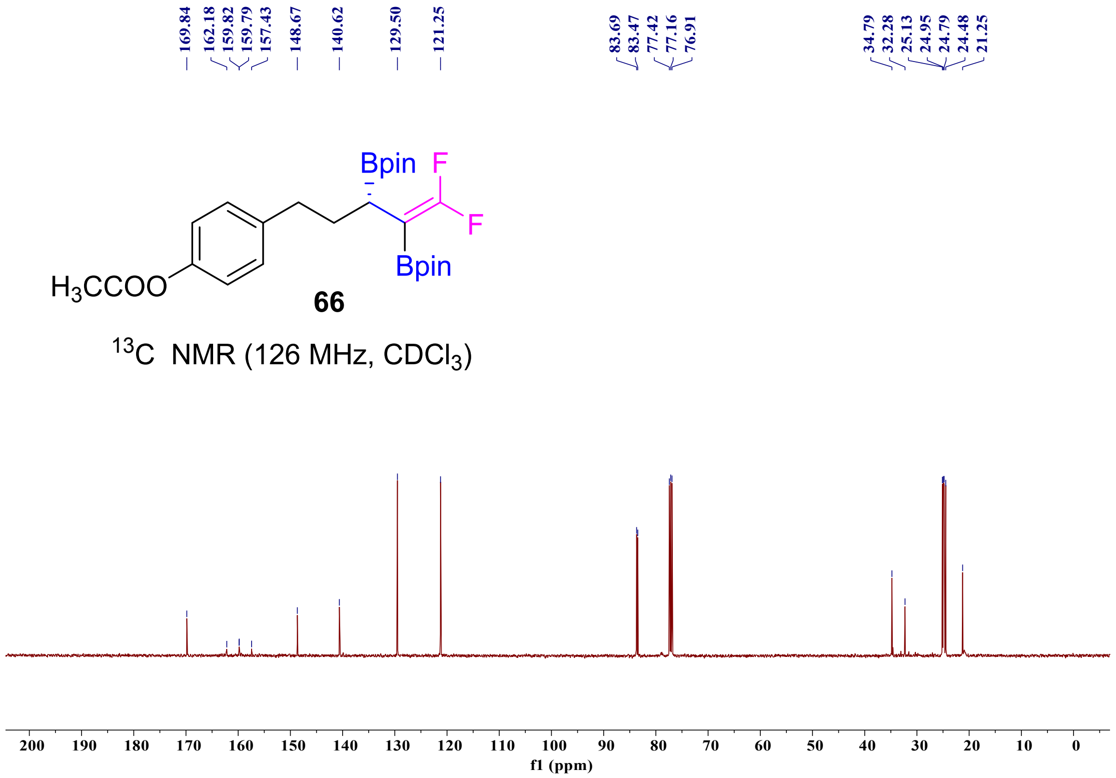

(S)4-(5,5-difluoro-3,4-bis(4,4,5,5-tetramethyl-1,3,2-dioxaborolan-2-yl)pent-4-en-1-yl)phenyl acetate(66)

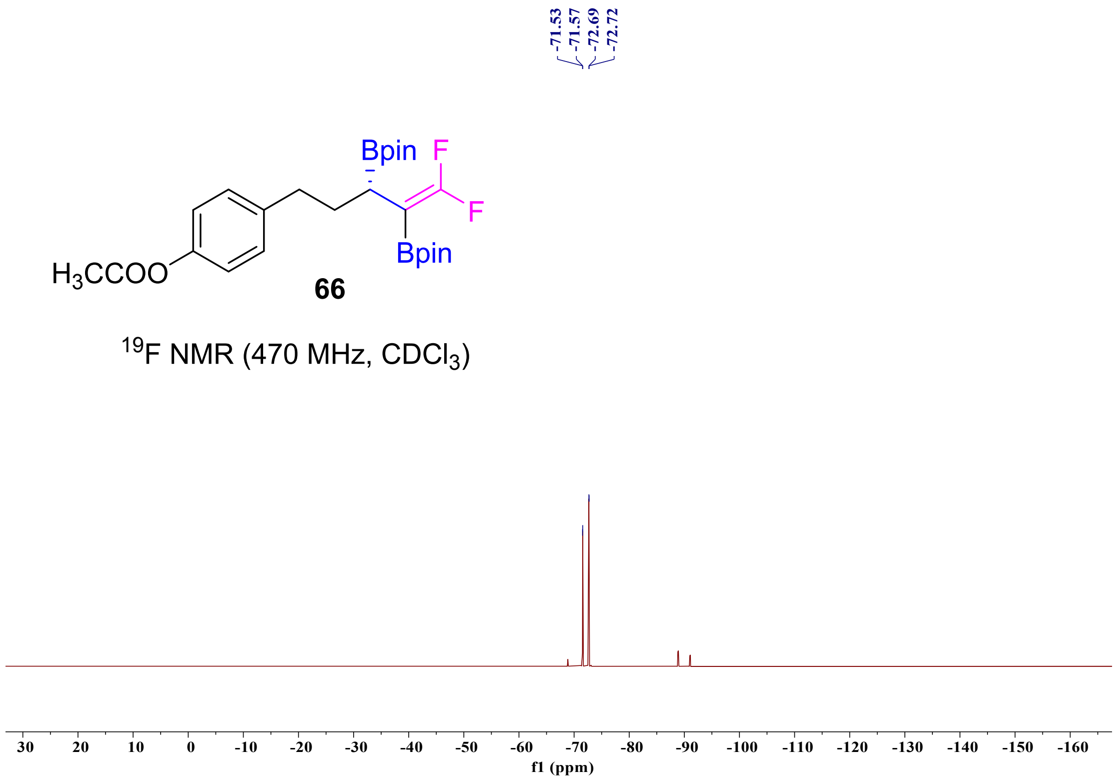

(S)4-(5,5-difluoro-3,4-bis(4,4,5,5-tetramethyl-1,3,2-dioxaborolan-2-yl)pent-4-en-1-yl)phenyl acetate(66)

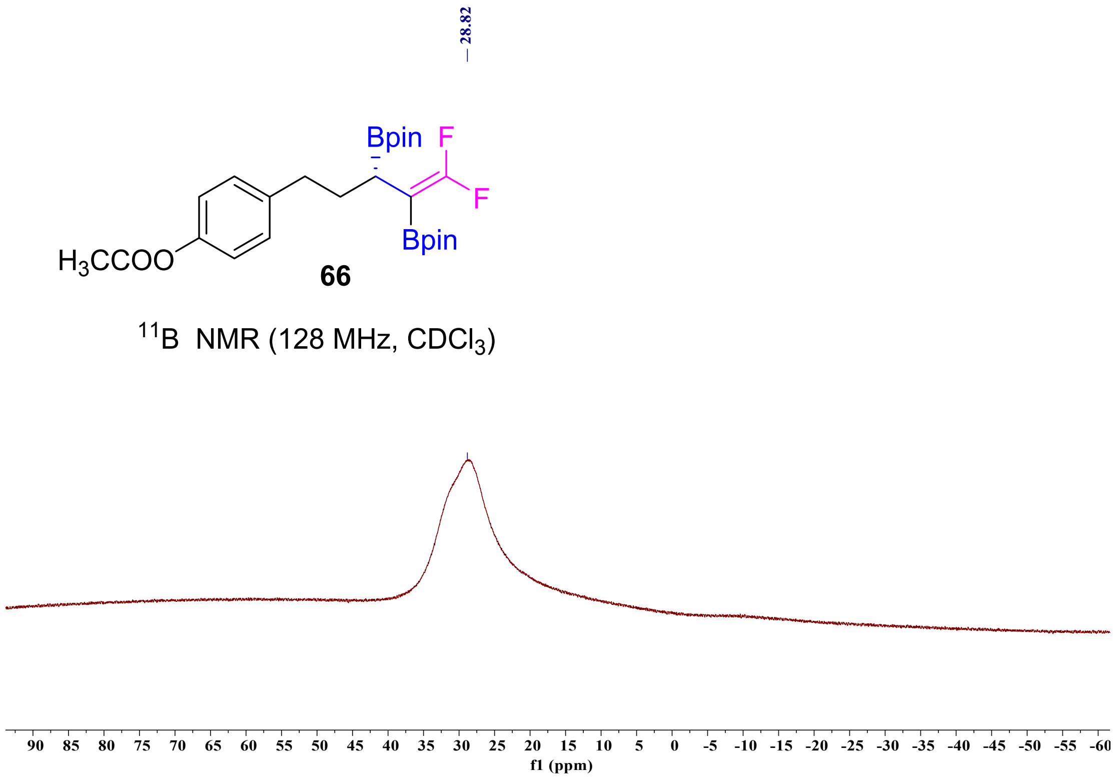

(S)2,2'-(4-(4-bromophenyl)-1,1-difluorobut-1-ene-2,3-diyl)bis(4,4,5,5-tetramethyl-1,3,2-dioxaborolane) (67)

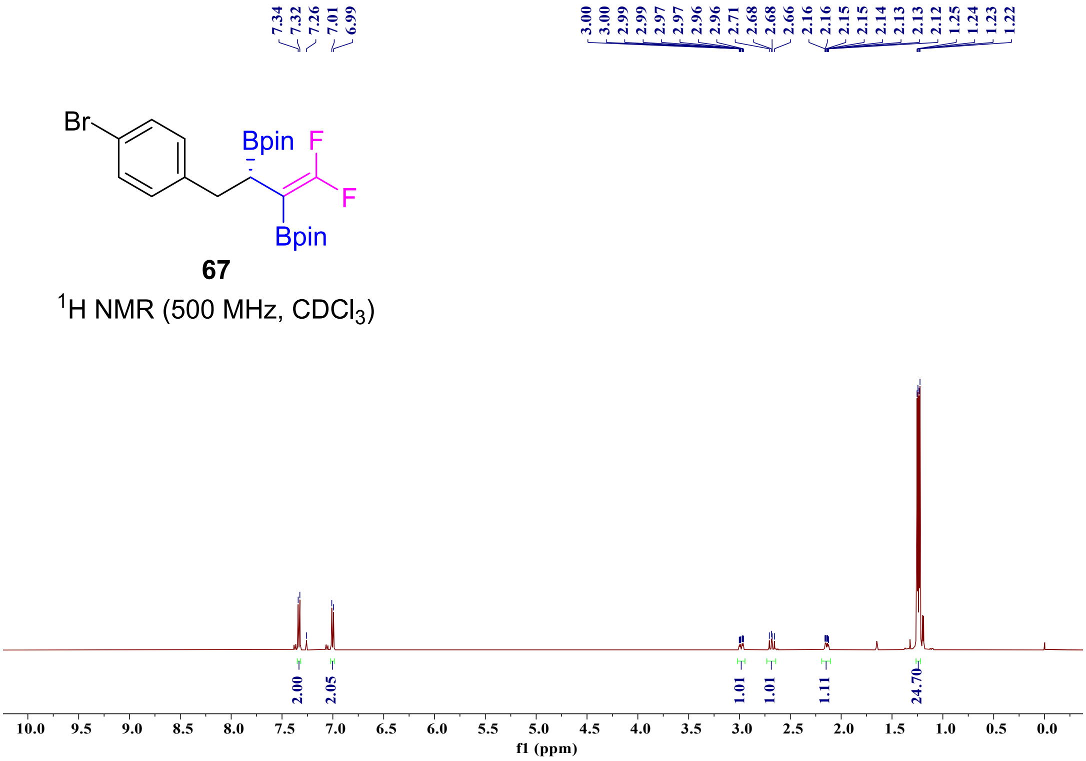

(S)2,2'-(4-(4-bromophenyl)-1,1-difluorobut-1-ene-2,3-diyl)bis(4,4,5,5-tetramethyl-1,3,2-dioxaborolane) (67)

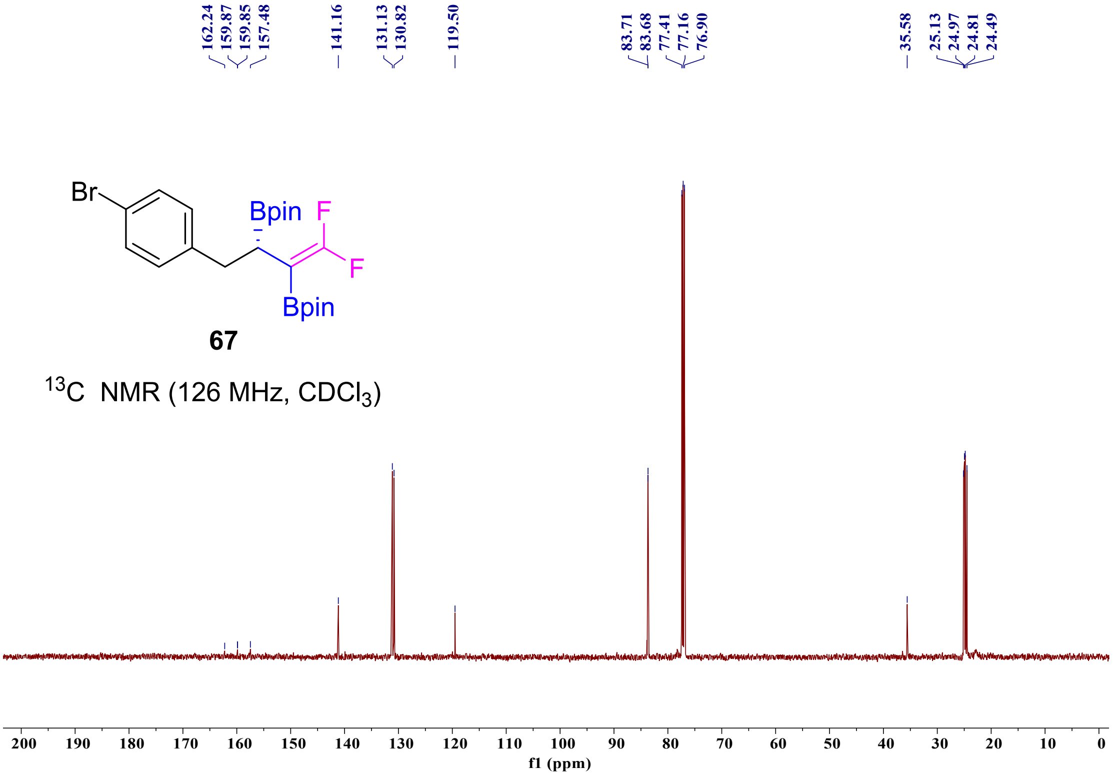

(S)2,2'-(4-(4-bromophenyl)-1,1-difluorobut-1-ene-2,3-diyl)bis(4,4,5,5-tetramethyl-1,3,2-dioxaborolane) (67)

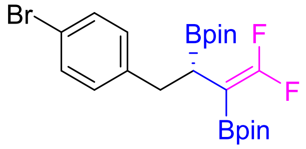

67

^{19}F NMR (470 MHz, $CDCl_3$)

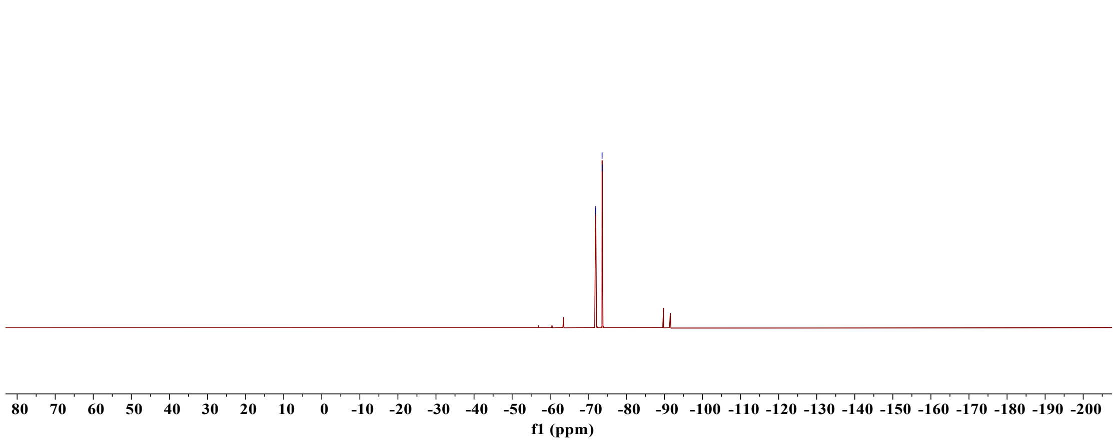

(S)2,2'-(4-(4-bromophenyl)-1,1-difluorobut-1-ene-2,3-diyl)bis(4,4,5,5-tetramethyl-1,3,2-dioxaborolane) (67)

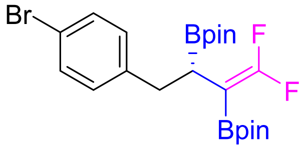

67

^{11}B NMR (128 MHz, $CDCl_3$)

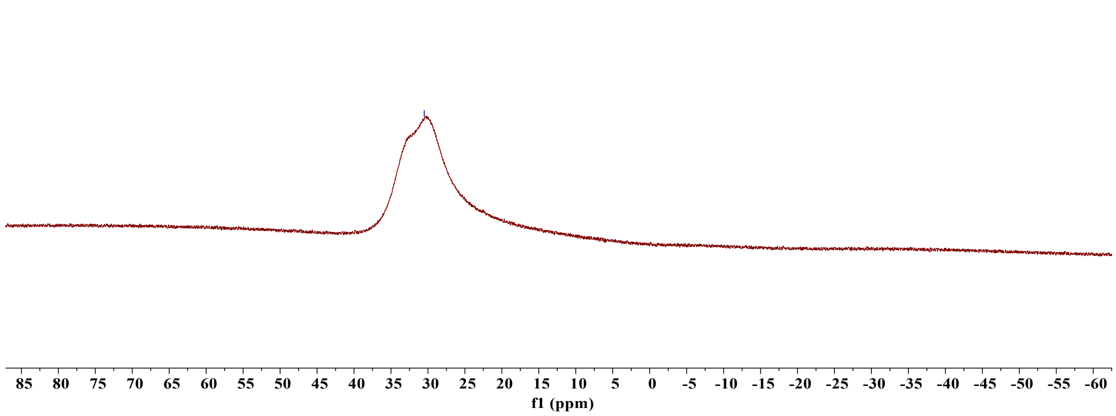

(S)-2,2'-(5-(3-bromophenyl)-1,1-difluoropent-1-ene-2,3-diyl)bis(4,4,5,5-tetramethyl-1,3,2-dioxaborolane) (68)

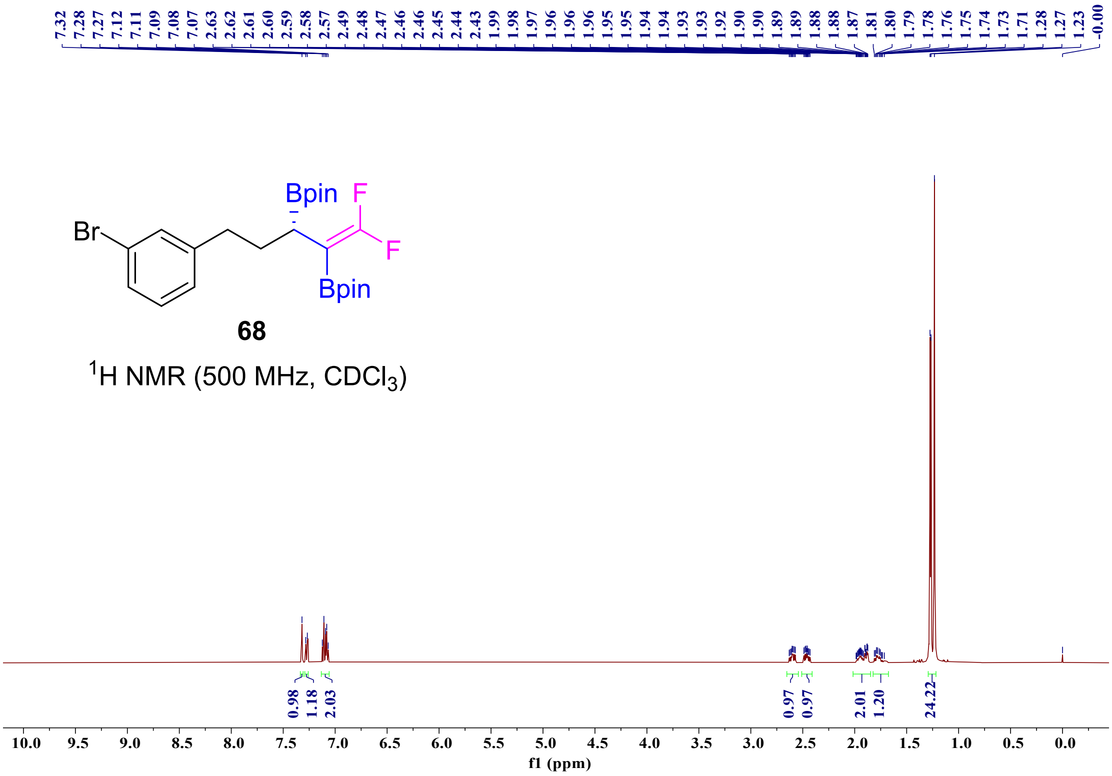

(S)-2,2'-(5-(3-bromophenyl)-1,1-difluoropent-1-ene-2,3-diyl)bis(4,4,5,5-tetramethyl-1,3,2-dioxaborolane) (68)

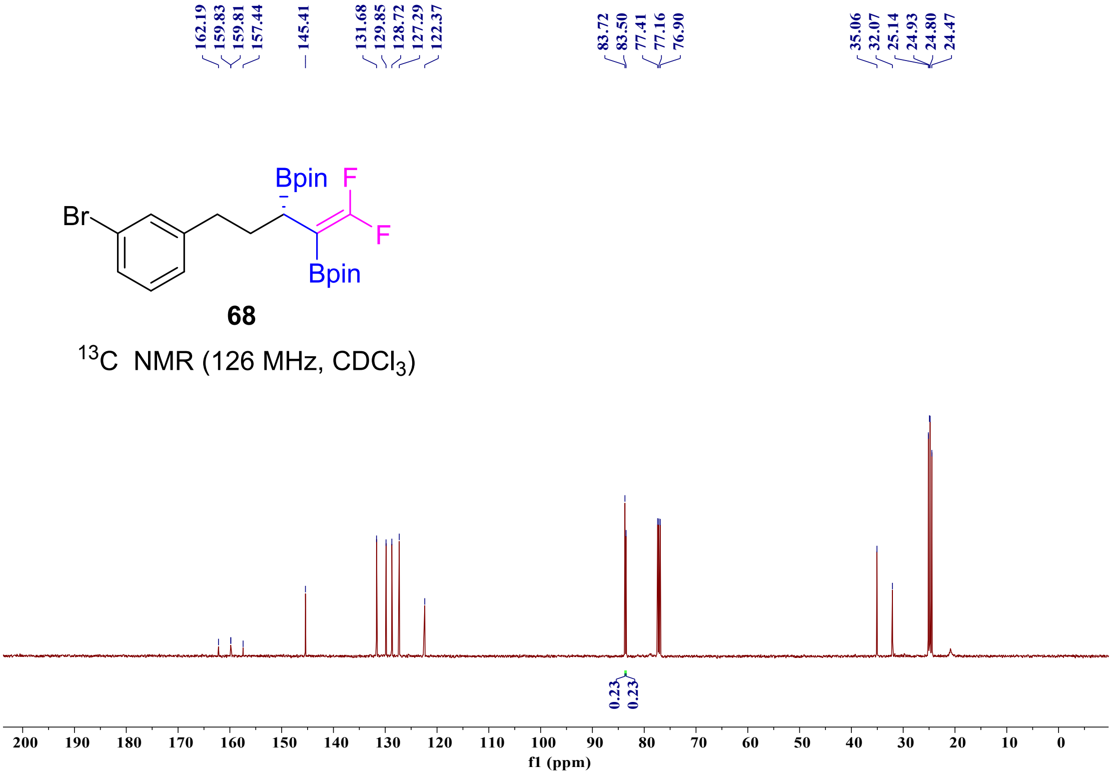

(S)-2,2'-(5-(3-bromophenyl)-1,1-difluoropent-1-ene-2,3-diyl)bis(4,4,5,5-tetramethyl-1,3,2-dioxaborolane) (68)

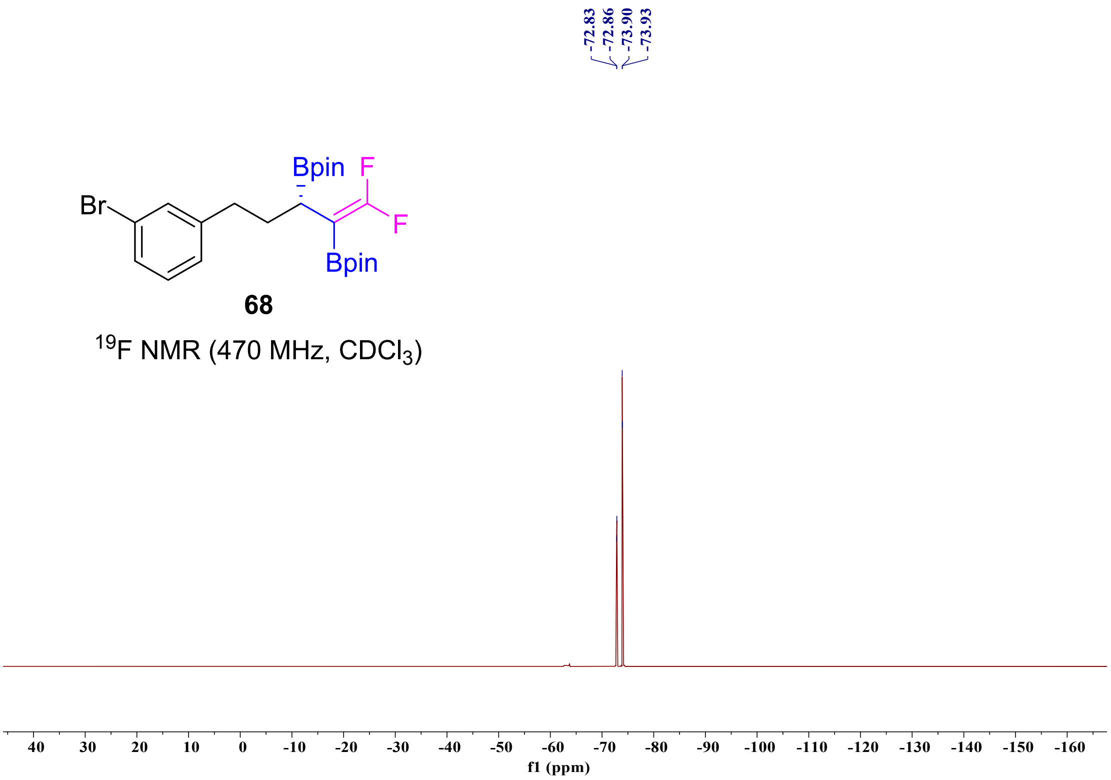

(S)-2,2'-(5-(3-bromophenyl)-1,1-difluoropent-1-ene-2,3-diyl)bis(4,4,5,5-tetramethyl-1,3,2-dioxaborolane) (68)

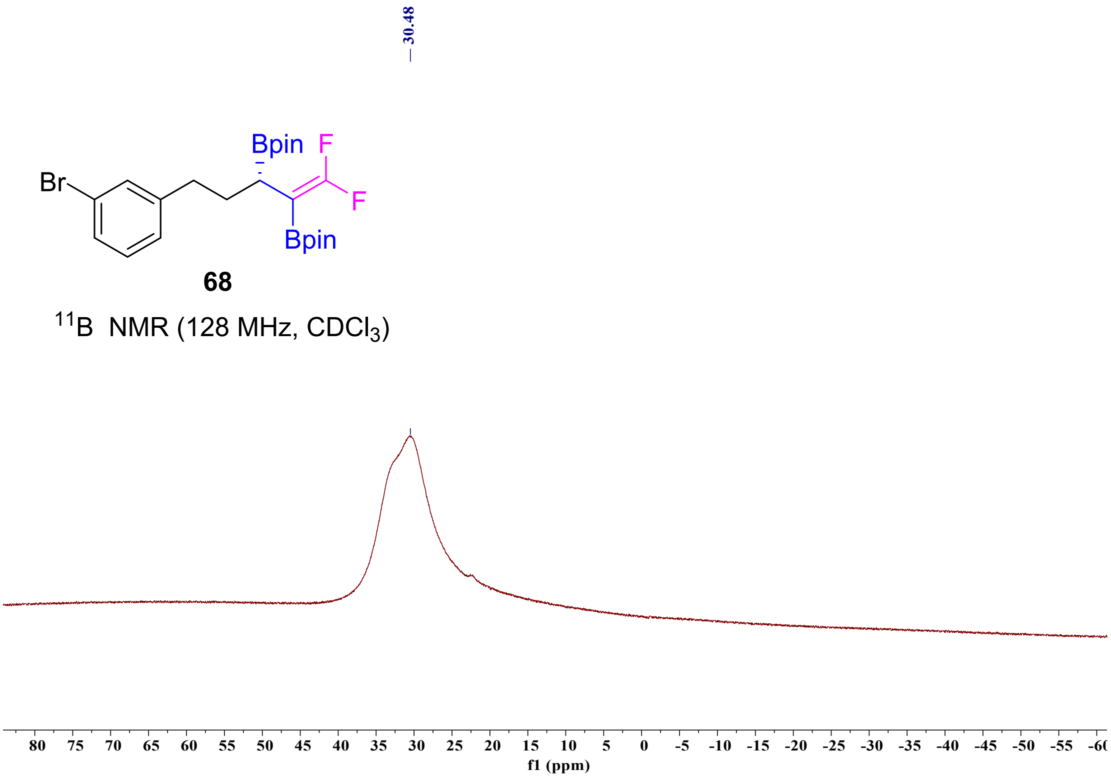

(S)-2,2'-(5-(2-bromophenyl)-1,1-difluoropent-1-ene-2,3-diyl)bis(4,4,5,5-tetramethyl-1,3,2-dioxaborolane) (69)

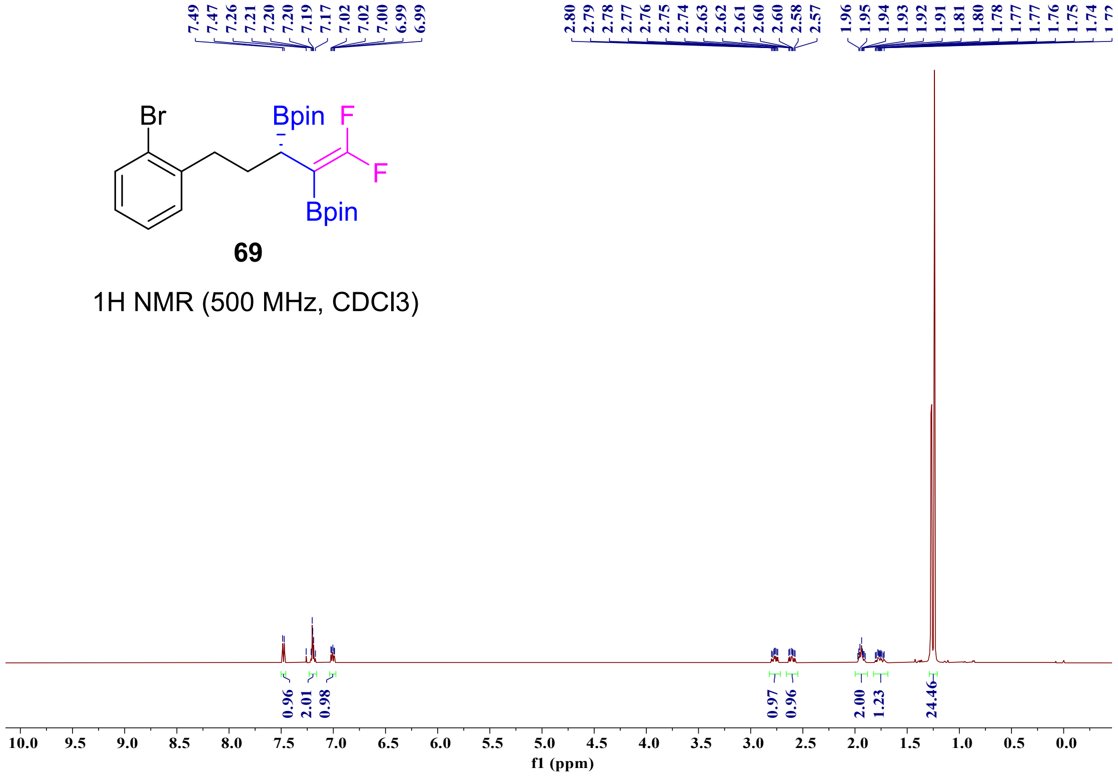

(S)-2,2'-(5-(2-bromophenyl)-1,1-difluoropent-1-ene-2,3-diyl)bis(4,4,5,5-tetramethyl-1,3,2-dioxaborolane) (69)

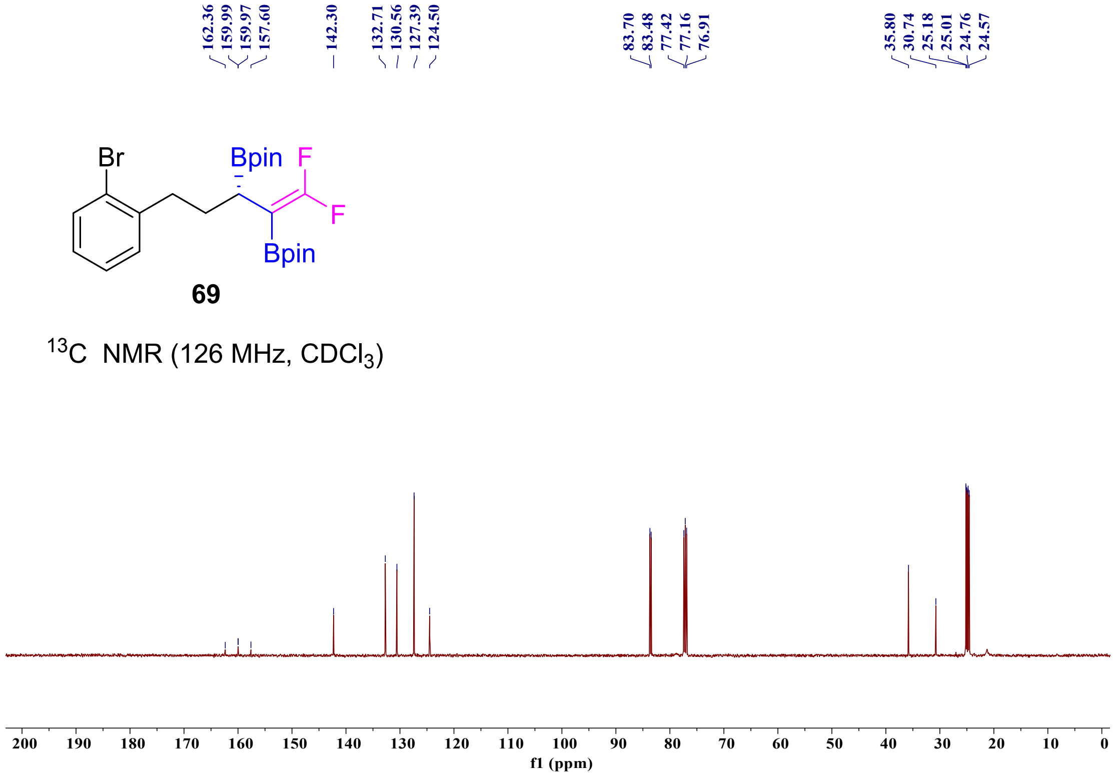

(S)-2,2'-(5-(2-bromophenyl)-1,1-difluoropent-1-ene-2,3-diyl)bis(4,4,5,5-tetramethyl-1,3,2-dioxaborolane) (69)

-71.38
-71.41
-72.51
-72.54

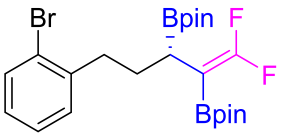

69

^{19}F NMR (470 MHz, $CDCl_3$)

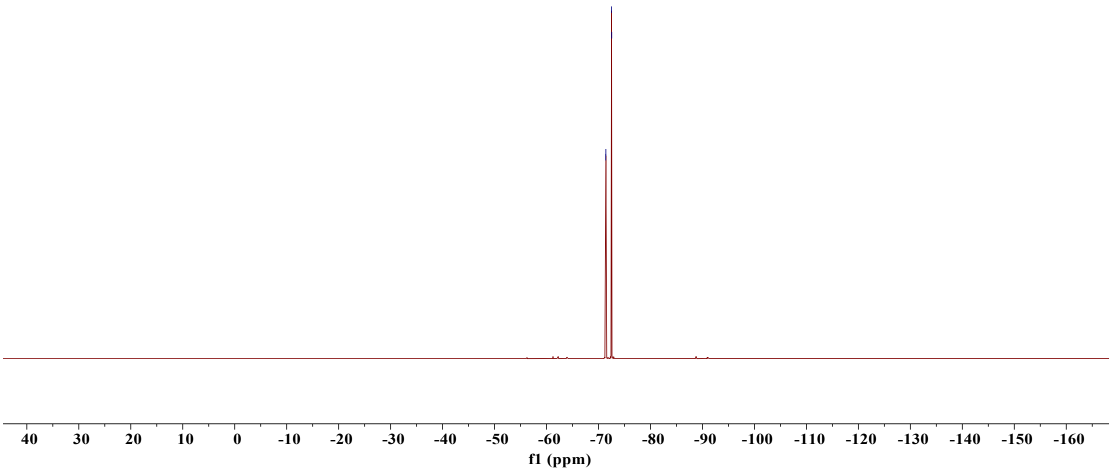

(S)-2,2'-(5-(2-bromophenyl)-1,1-difluoropent-1-ene-2,3-diyl)bis(4,4,5,5-tetramethyl-1,3,2-dioxaborolane) (69)

30.80

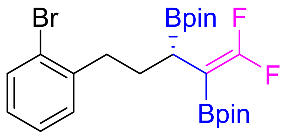

69

^{11}B NMR (128 MHz, $CDCl_3$)

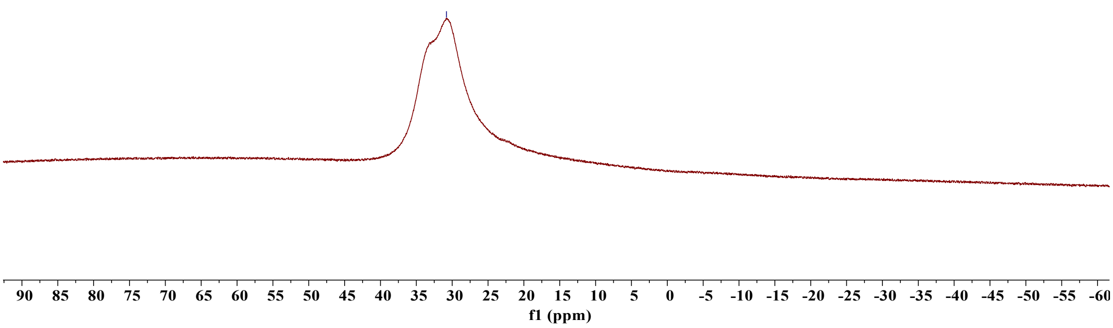

(S)-2,2'-(1,1-difluoro-5,5-diphenylpent-1-ene-2,3-diyl)bis(4,4,5,5-tetramethyl-1,3,2-dioxaborolane) (70)

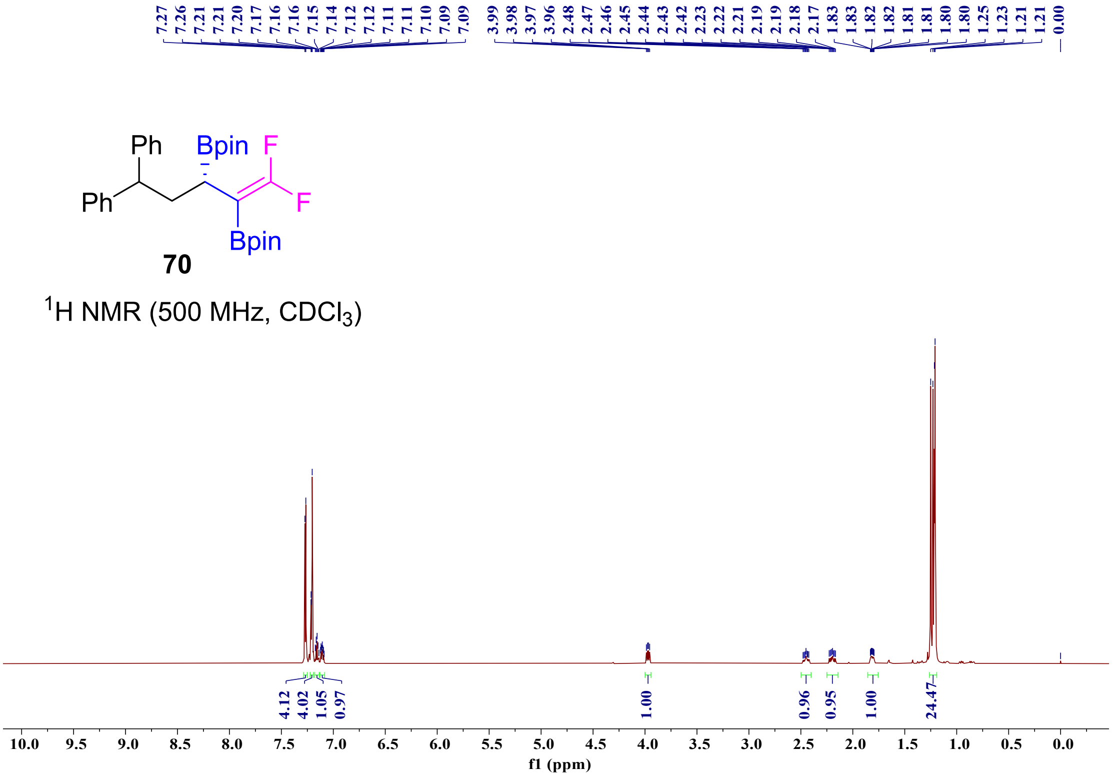

(S)-2,2'-(1,1-difluoro-5,5-diphenylpent-1-ene-2,3-diyl)bis(4,4,5,5-tetramethyl-1,3,2-dioxaborolane) (70)

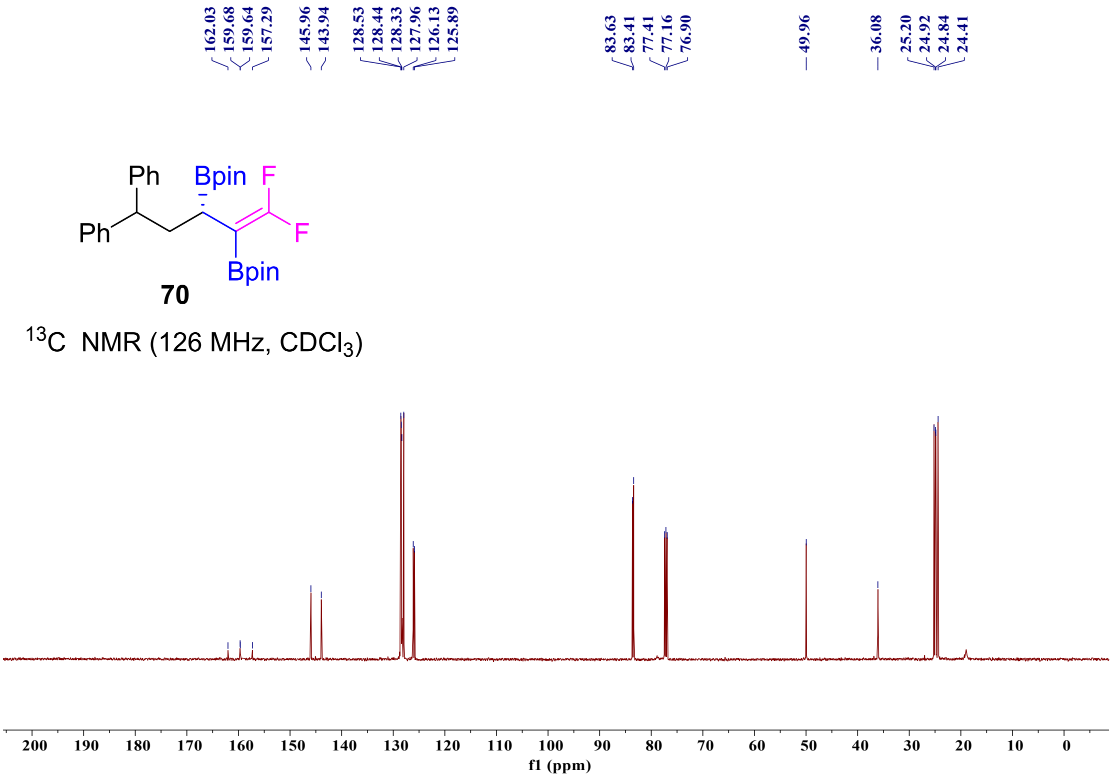

(S)-2,2'-(1,1-difluoro-5,5-diphenylpent-1-ene-2,3-diyl)bis(4,4,5,5-tetramethyl-1,3,2-dioxaborolane) (70)

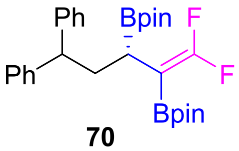

70

^{19}F NMR (470 MHz, $CDCl_3$)

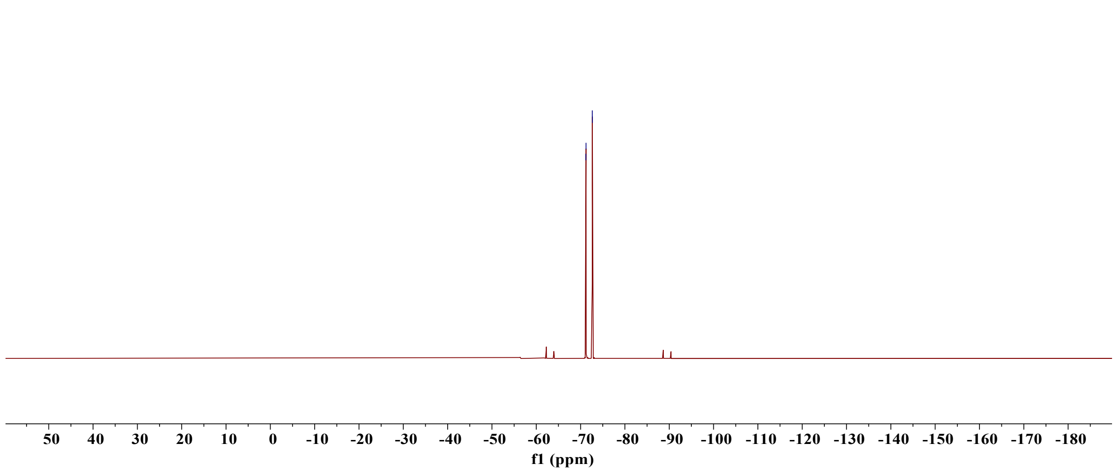

(S)-2,2'-(1,1-difluoro-5,5-diphenylpent-1-ene-2,3-diyl)bis(4,4,5,5-tetramethyl-1,3,2-dioxaborolane) (70)

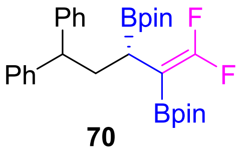

70

^{11}B NMR (128 MHz, $CDCl_3$)

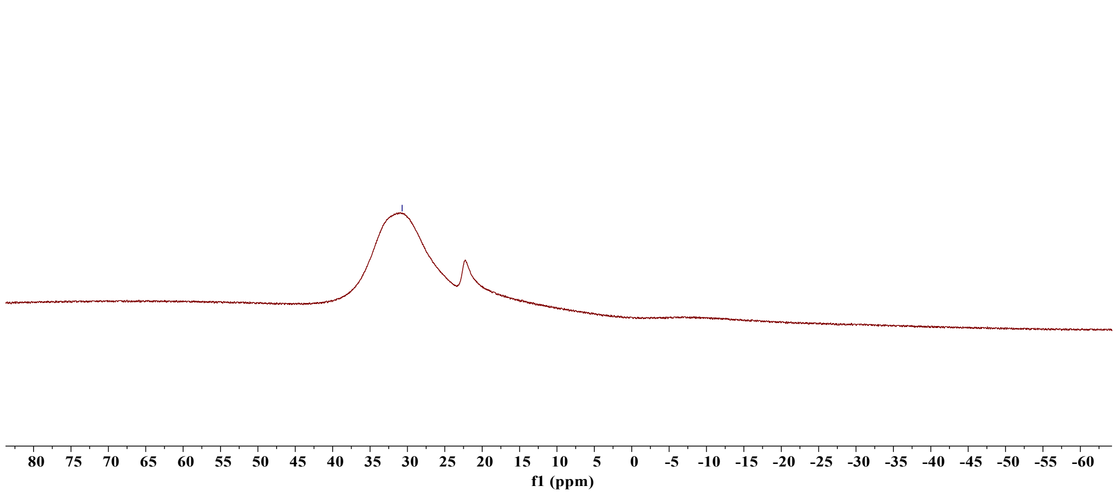

(S)2,2'-(1,1-difluoro-6-phenylhex-1-ene-2,3-diyl)bis(4,4,5,5-tetramethyl-1,3,2-dioxaborolane) (71)

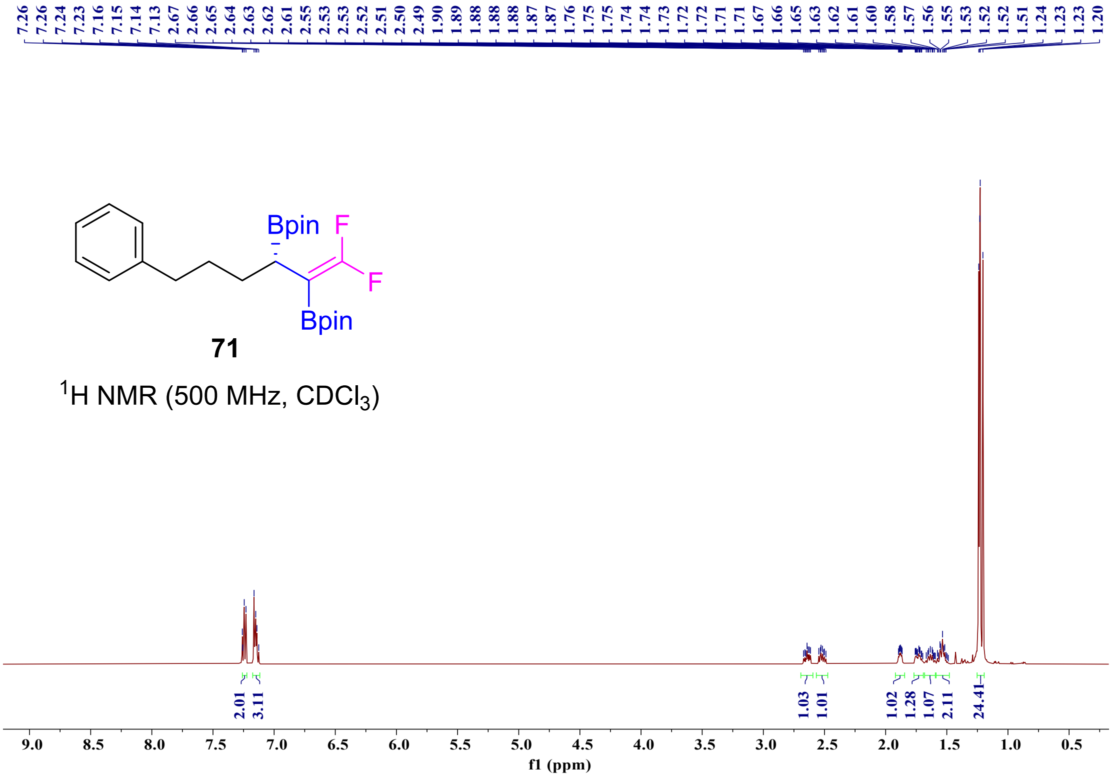

(S)2,2'-(1,1-difluoro-6-phenylhex-1-ene-2,3-diyl)bis(4,4,5,5-tetramethyl-1,3,2-dioxaborolane) (71)

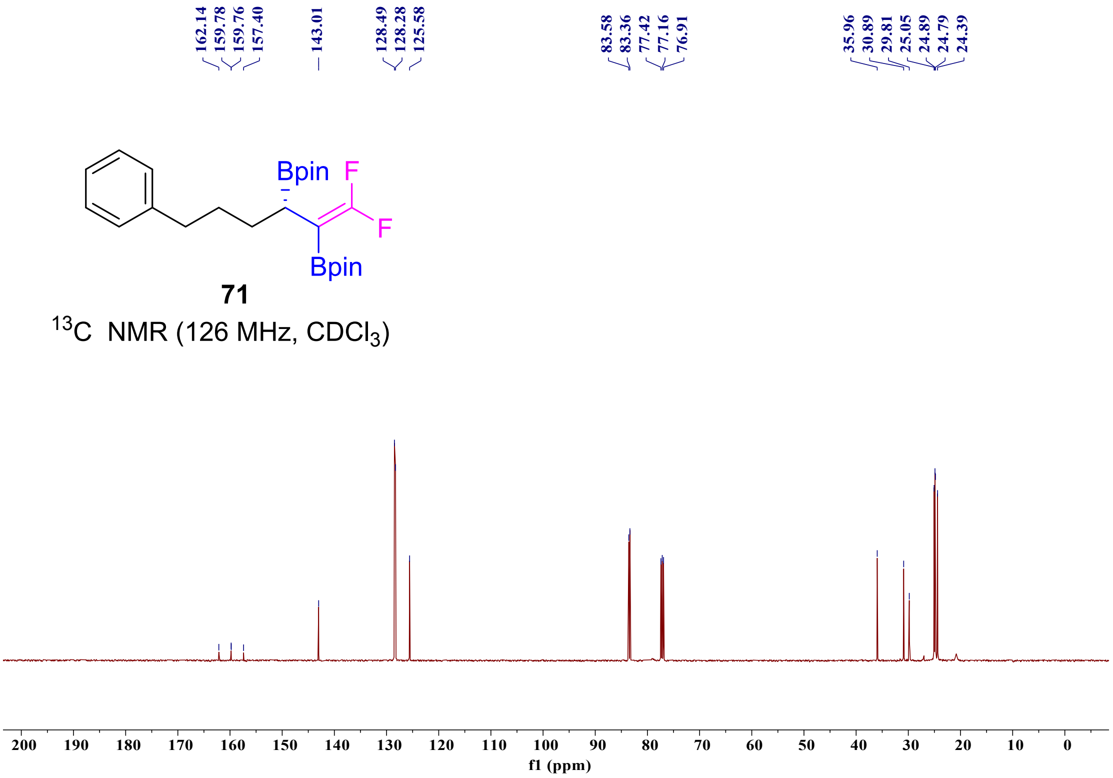

(S)2,2'-(1,1-difluoro-6-phenylhex-1-ene-2,3-diyl)bis(4,4,5,5-tetramethyl-1,3,2-dioxaborolane) (71)

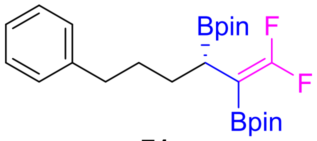

71

^{19}F NMR (470 MHz, $CDCl_3$)

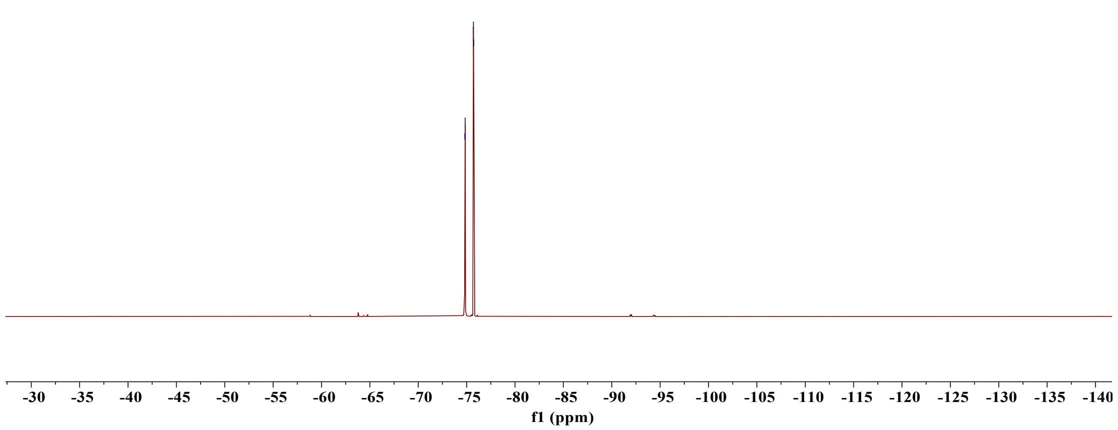

(S)2,2'-(1,1-difluoro-6-phenylhex-1-ene-2,3-diyl)bis(4,4,5,5-tetramethyl-1,3,2-dioxaborolane) (71)

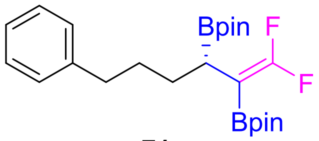

71

^{11}B NMR (128 MHz, $CDCl_3$)

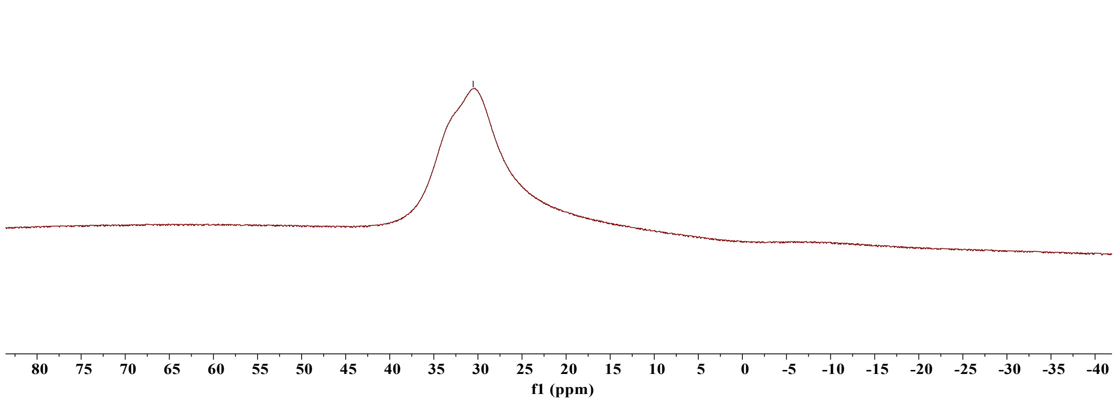

(S)-2,2'-(1,1-difluoro-4-phenylbut-1-ene-2,3-diyl)bis(4,4,5,5-tetramethyl-1,3,2-dioxaborolane) (72)

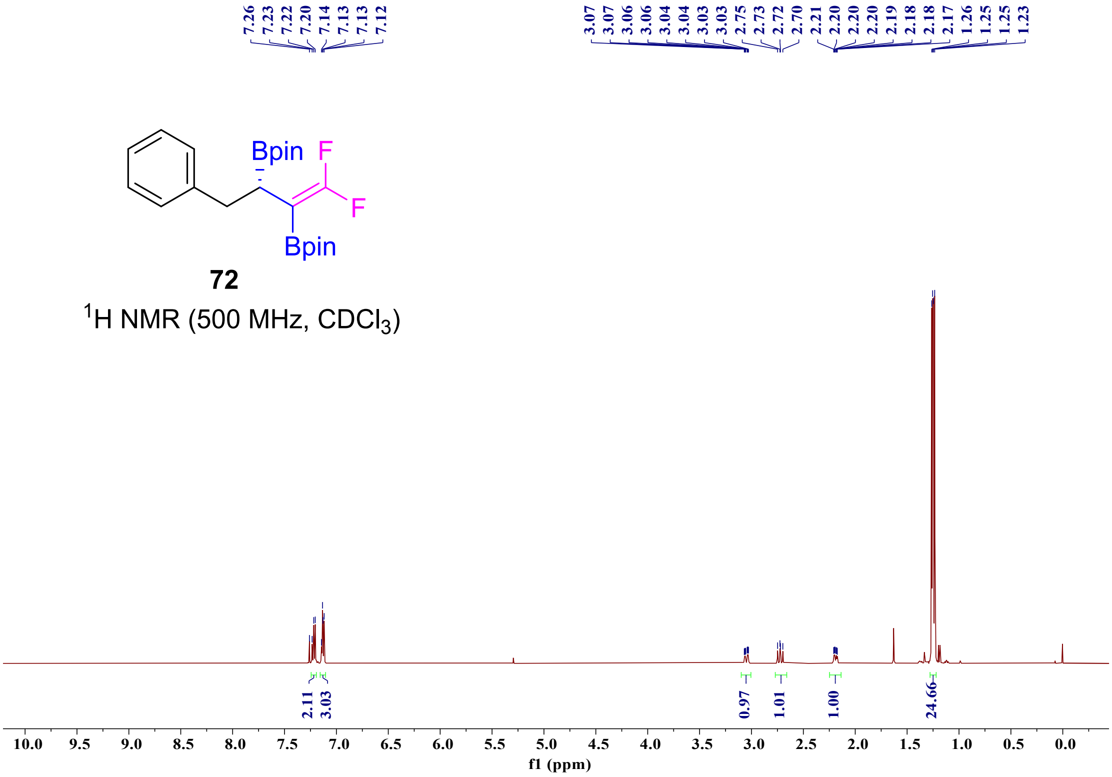

(S)-2,2'-(1,1-difluoro-4-phenylbut-1-ene-2,3-diyl)bis(4,4,5,5-tetramethyl-1,3,2-dioxaborolane) (72)

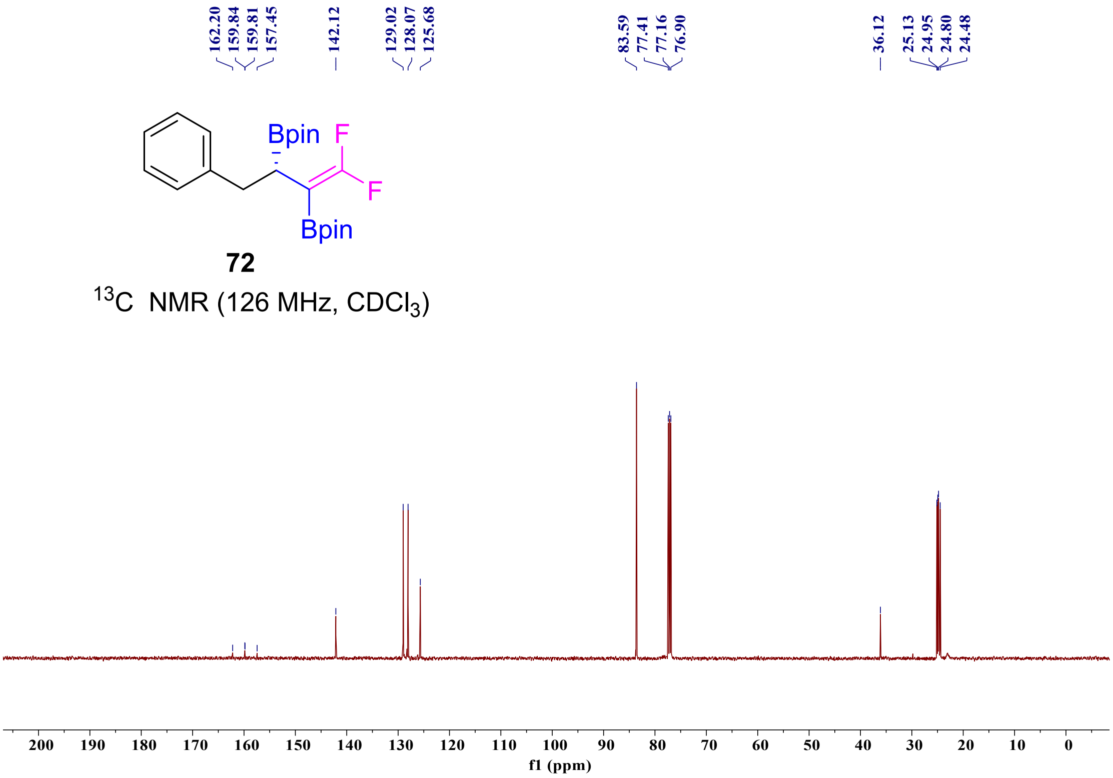

(S)-2,2'-(1,1-difluoro-4-phenylbut-1-ene-2,3-diyl)bis(4,4,5,5-tetramethyl-1,3,2-dioxaborolane) (72)

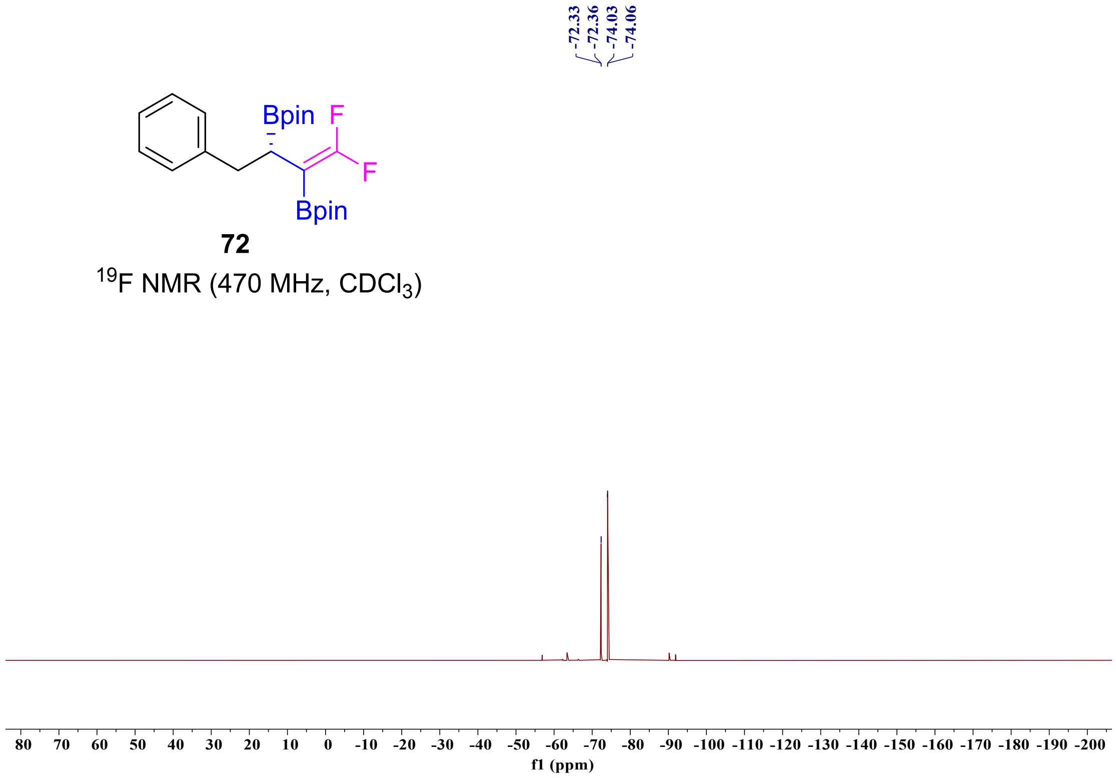

(S)-2,2'-(1,1-difluoro-4-phenylbut-1-ene-2,3-diyl)bis(4,4,5,5-tetramethyl-1,3,2-dioxaborolane) (72)

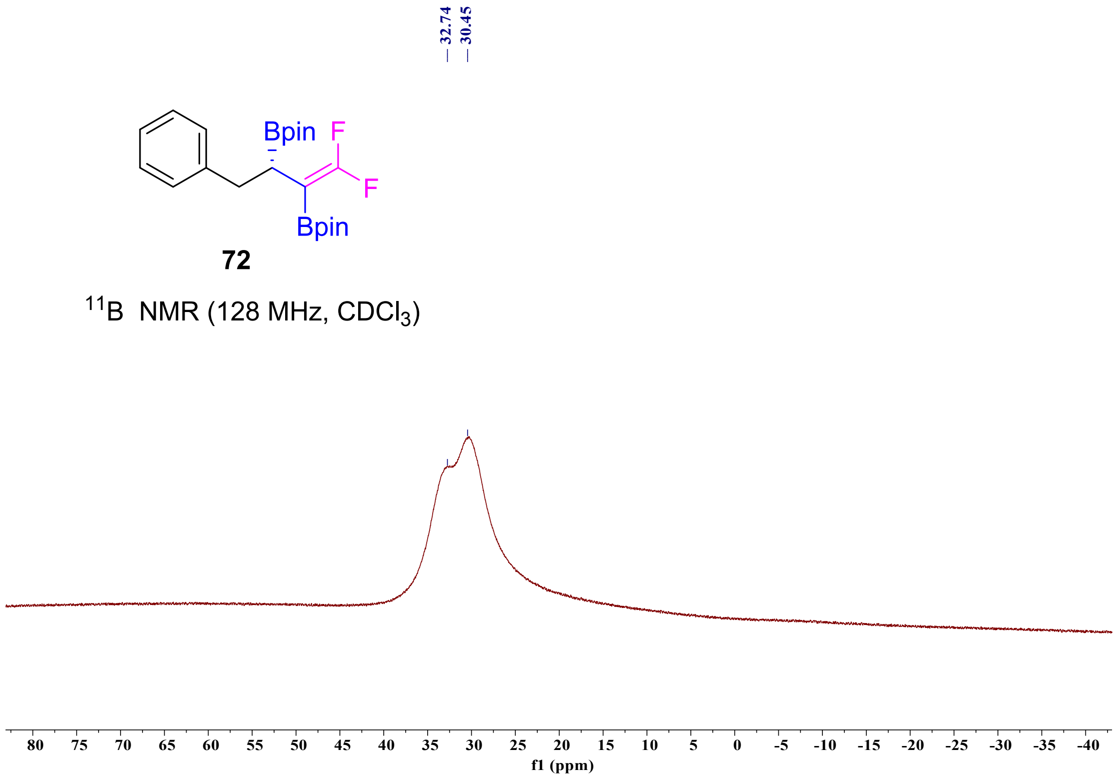

(S)-2,2'-(1,1-difluoro-4-(naphthalen-1-yl)but-1-ene-2,3-diyl)bis(4,4,5,5-tetramethyl-1,3,2-dioxaborolane) (73)

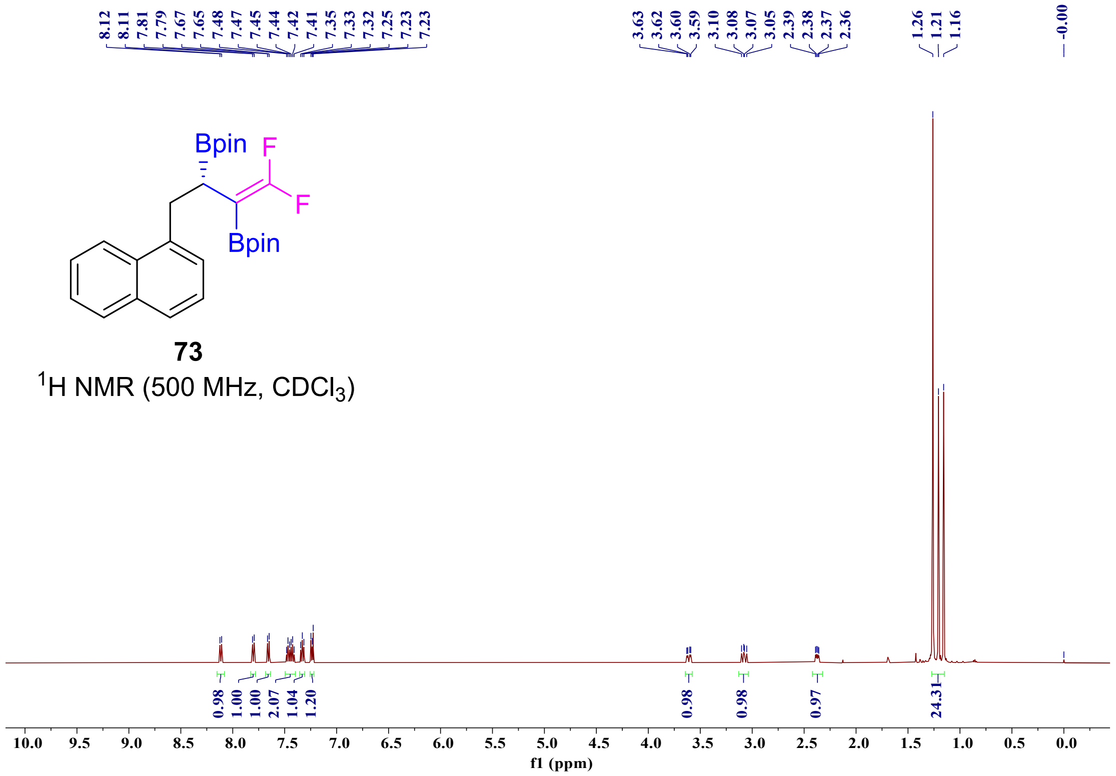

(S)-2,2'-(1,1-difluoro-4-(naphthalen-1-yl)but-1-ene-2,3-diyl)bis(4,4,5,5-tetramethyl-1,3,2-dioxaborolane) (73)

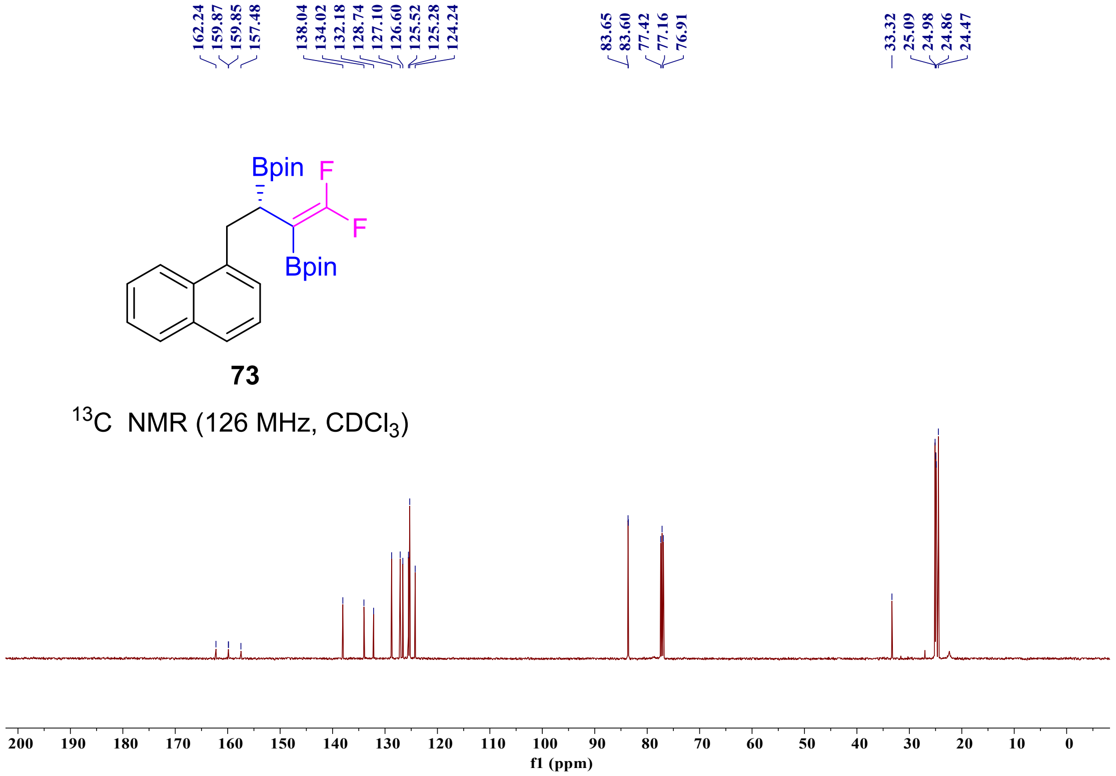

(S)-2,2'-(1,1-difluoro-4-(naphthalen-1-yl)but-1-ene-2,3-diyl)bis(4,4,5,5-tetramethyl-1,3,2-dioxaborolane) (73)

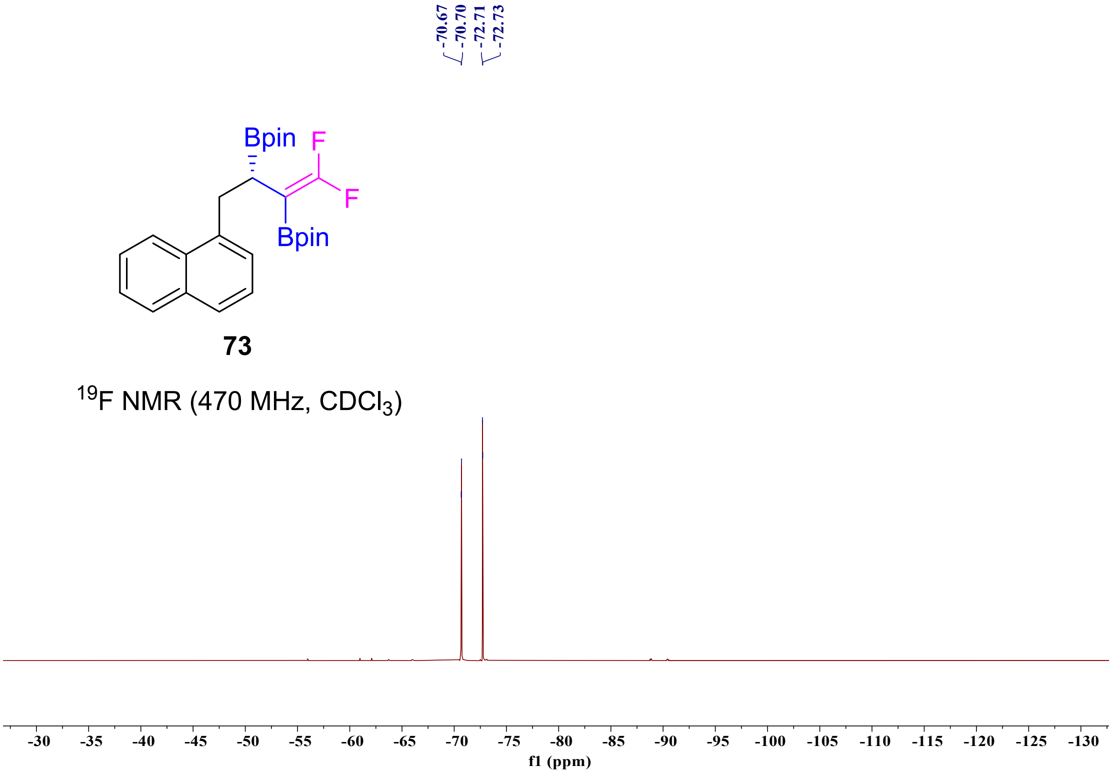

(S)-2,2'-(1,1-difluoro-4-(naphthalen-1-yl)but-1-ene-2,3-diyl)bis(4,4,5,5-tetramethyl-1,3,2-dioxaborolane) (73)

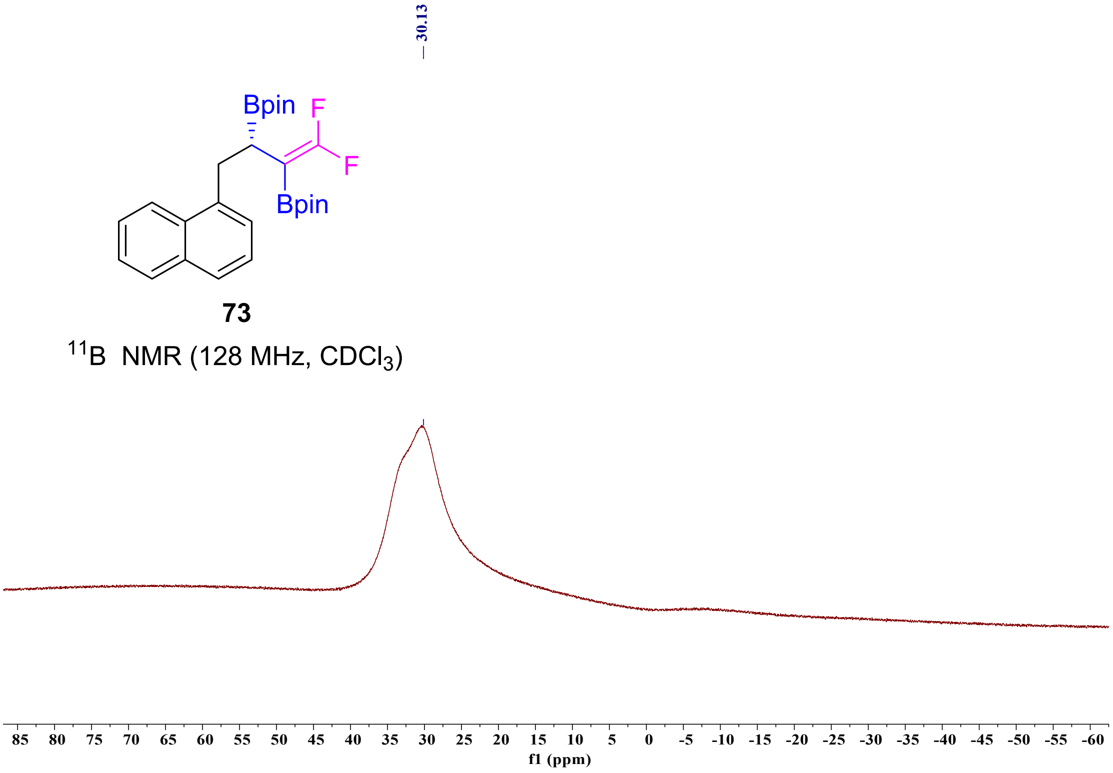

(S)2,2'-(4-([1,1'-biphenyl]-4-yl)-1,1-difluorobut-1-ene-2,3-diyl)bis(4,4,5,5-tetramethyl-1,3,2-dioxaborolane) (74)

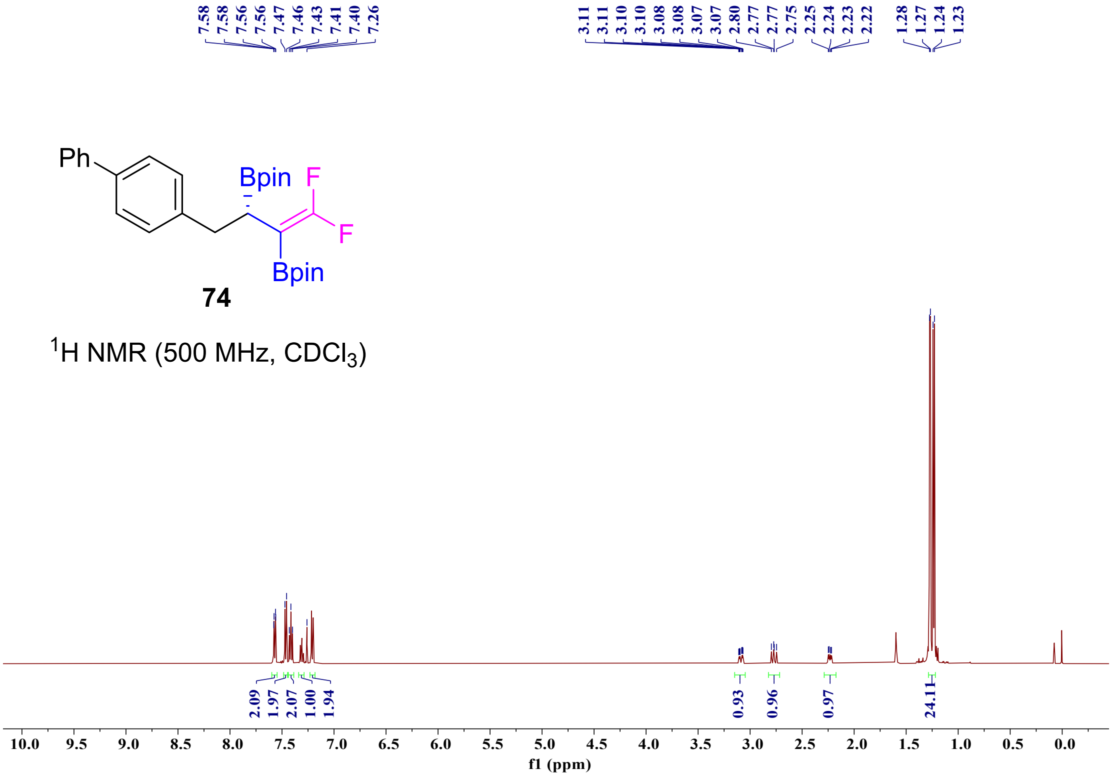

(S)2,2'-(4-([1,1'-biphenyl]-4-yl)-1,1-difluorobut-1-ene-2,3-diyl)bis(4,4,5,5-tetramethyl-1,3,2-dioxaborolane) (74)

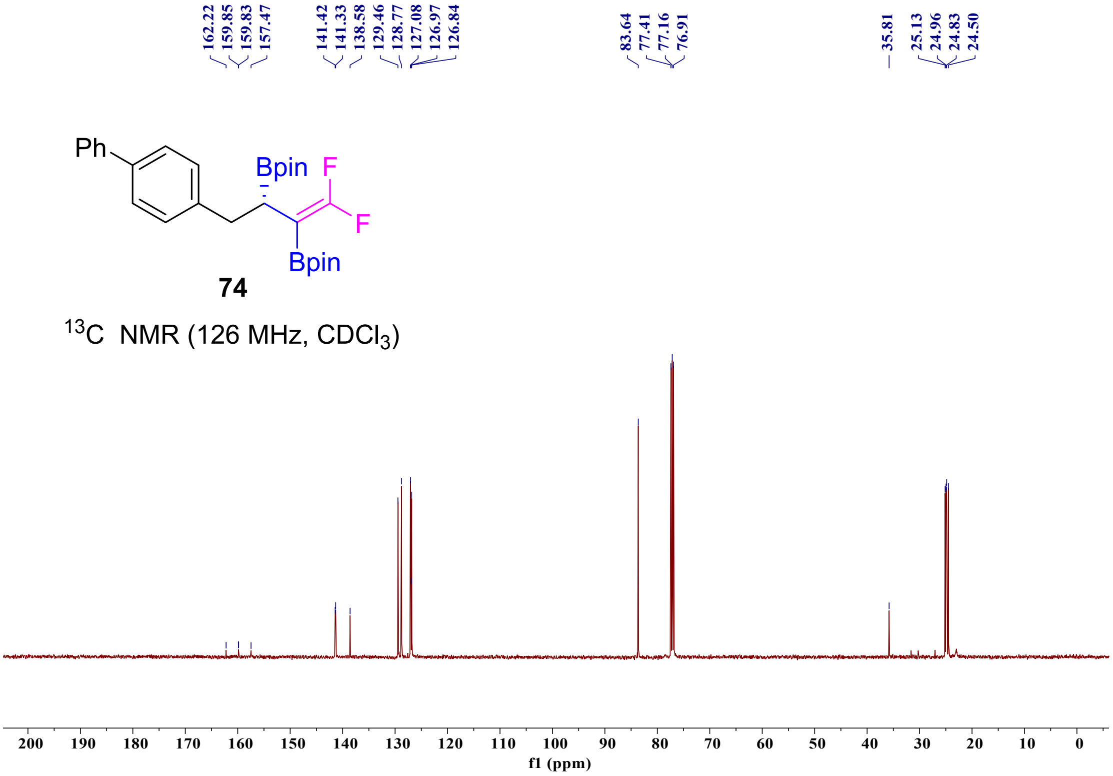

(S)2,2'-(4-([1,1'-biphenyl]-4-yl)-1,1-difluorobut-1-ene-2,3-diyl)bis(4,4,5,5-tetramethyl-1,3,2-dioxaborolane) (74)

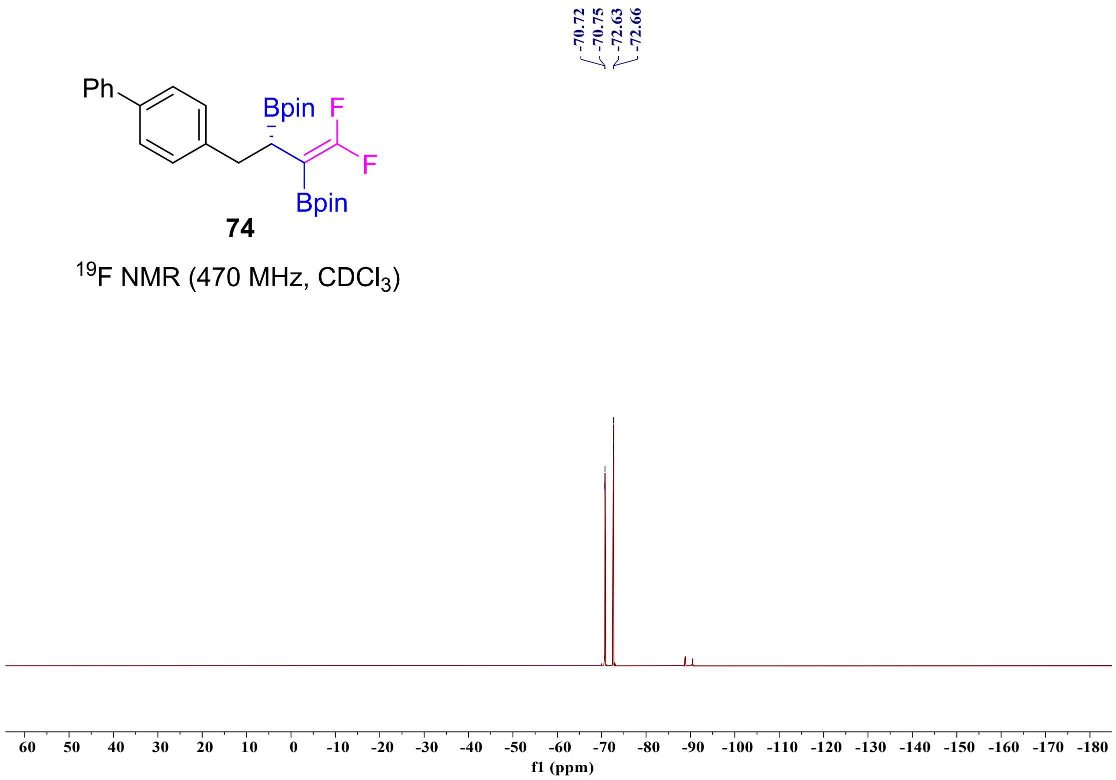

(S)2,2'-(4-([1,1'-biphenyl]-4-yl)-1,1-difluorobut-1-ene-2,3-diyl)bis(4,4,5,5-tetramethyl-1,3,2-dioxaborolane) (74)

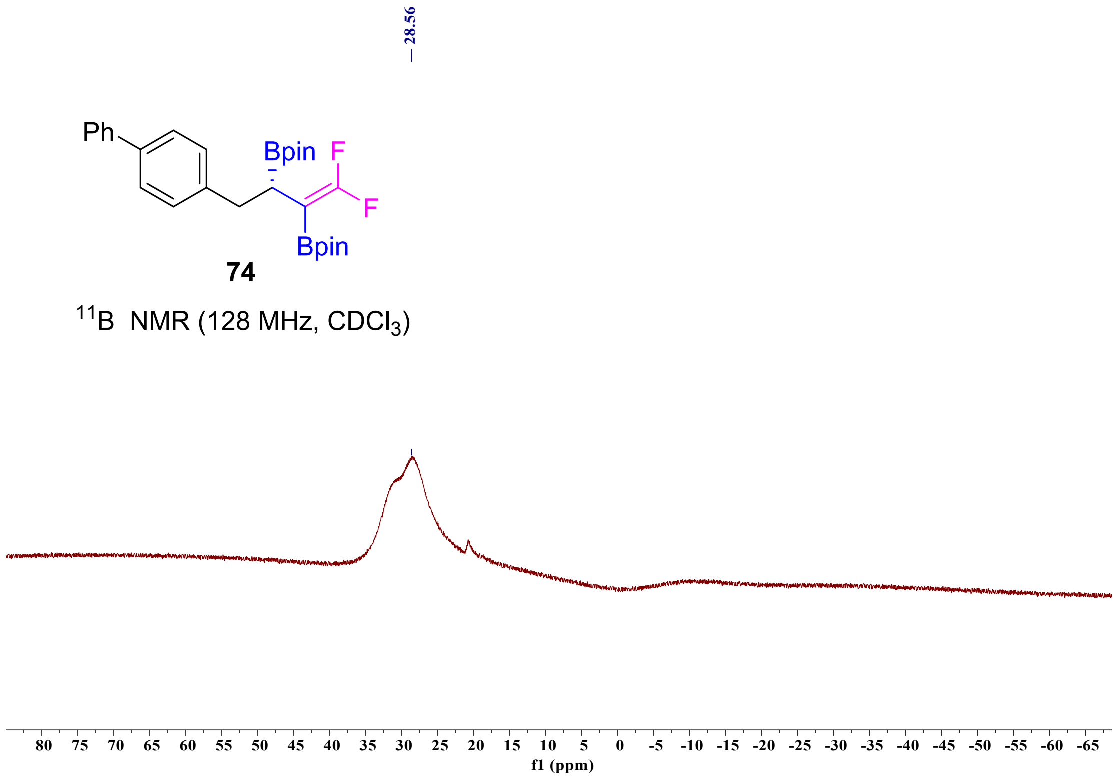

(S)-2,2'-(4-(benzo[d][1,3]dioxol-5-yl)-1,1-difluorobut-1-ene-2,3-diyl)bis(4,4,5,5-tetramethyl-1,3,2-dioxaborolane) (75)

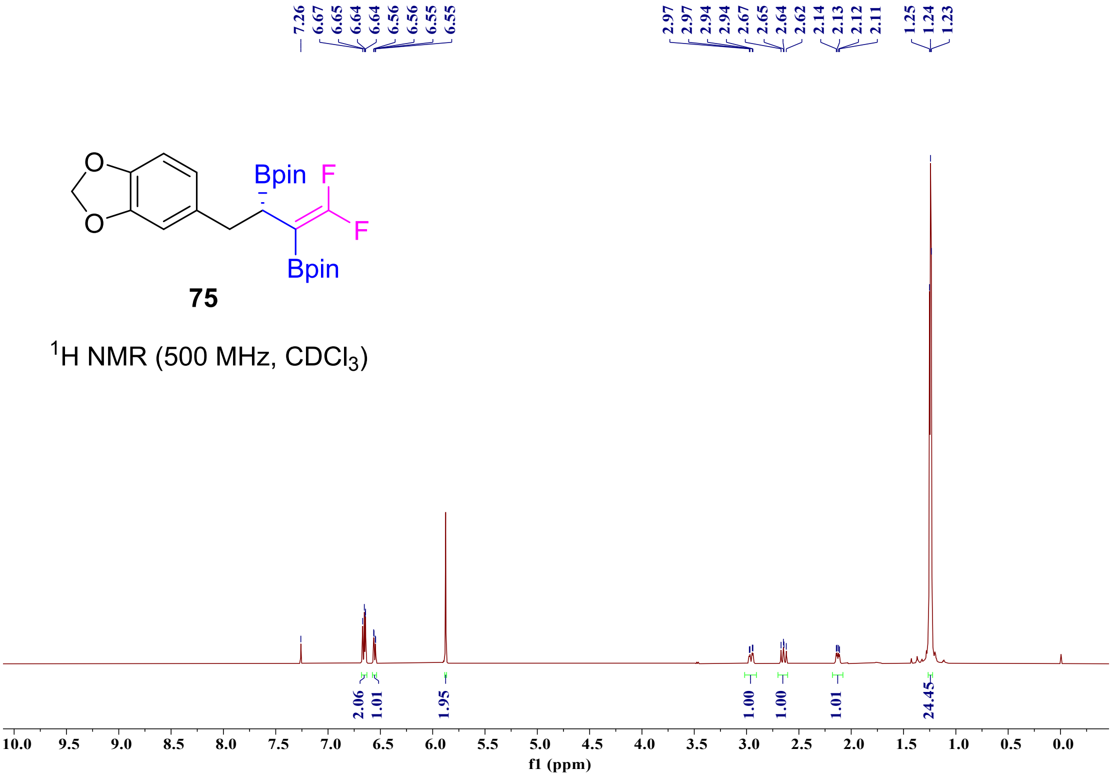

(S)-2,2'-(4-(benzo[d][1,3]dioxol-5-yl)-1,1-difluorobut-1-ene-2,3-diyl)bis(4,4,5,5-tetramethyl-1,3,2-dioxaborolane) (75)

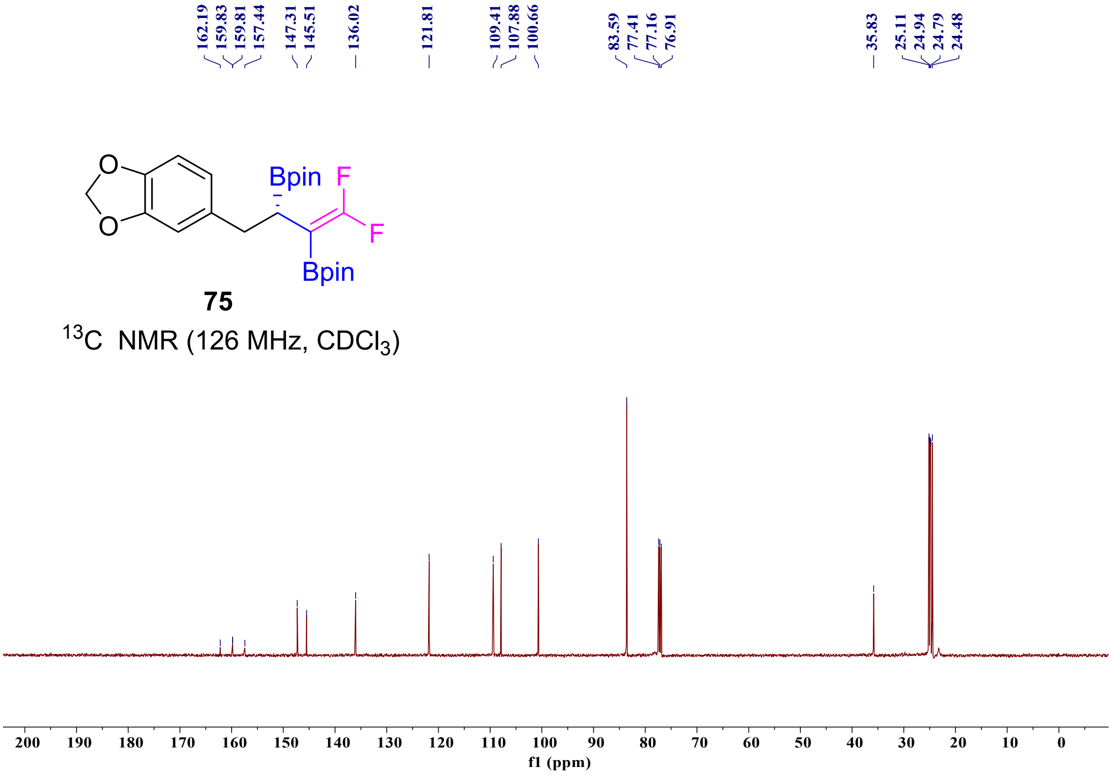

(S)-2,2'-(4-(benzo[d][1,3]dioxol-5-yl)-1,1-difluorobut-1-ene-2,3-diyl)bis(4,4,5,5-tetramethyl-1,3,2-dioxaborolane) (75)

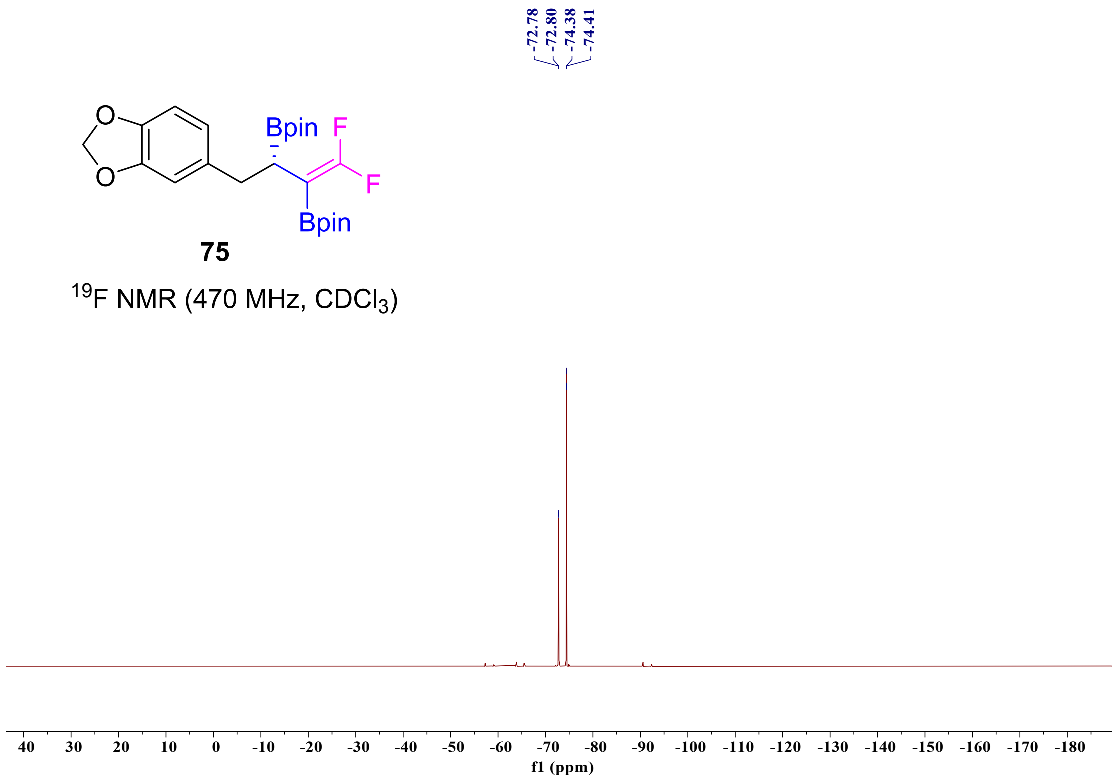

(S)-2,2'-(4-(benzo[d][1,3]dioxol-5-yl)-1,1-difluorobut-1-ene-2,3-diyl)bis(4,4,5,5-tetramethyl-1,3,2-dioxaborolane) (75)

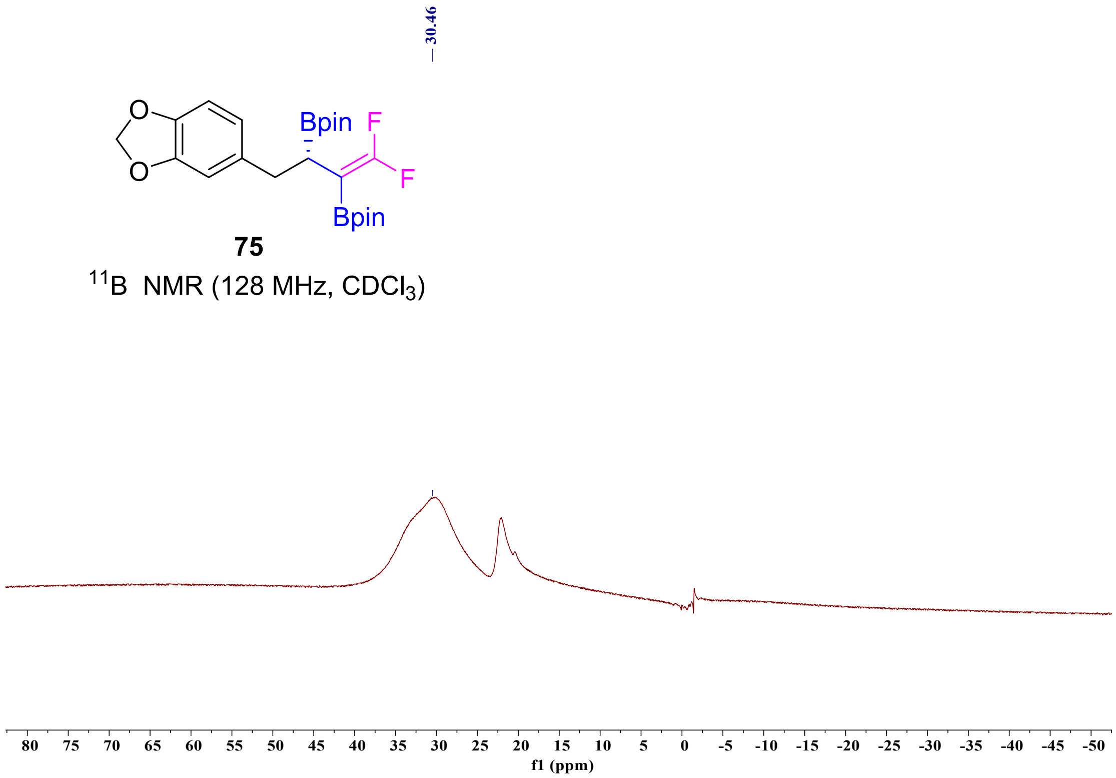

(S)-2,2'-(1,1-difluoro-5-(5-methylfuran-2-yl)pent-1-ene-2,3-diyl)bis(4,4,5,5-tetramethyl-1,3,2-dioxaborolane) (76)

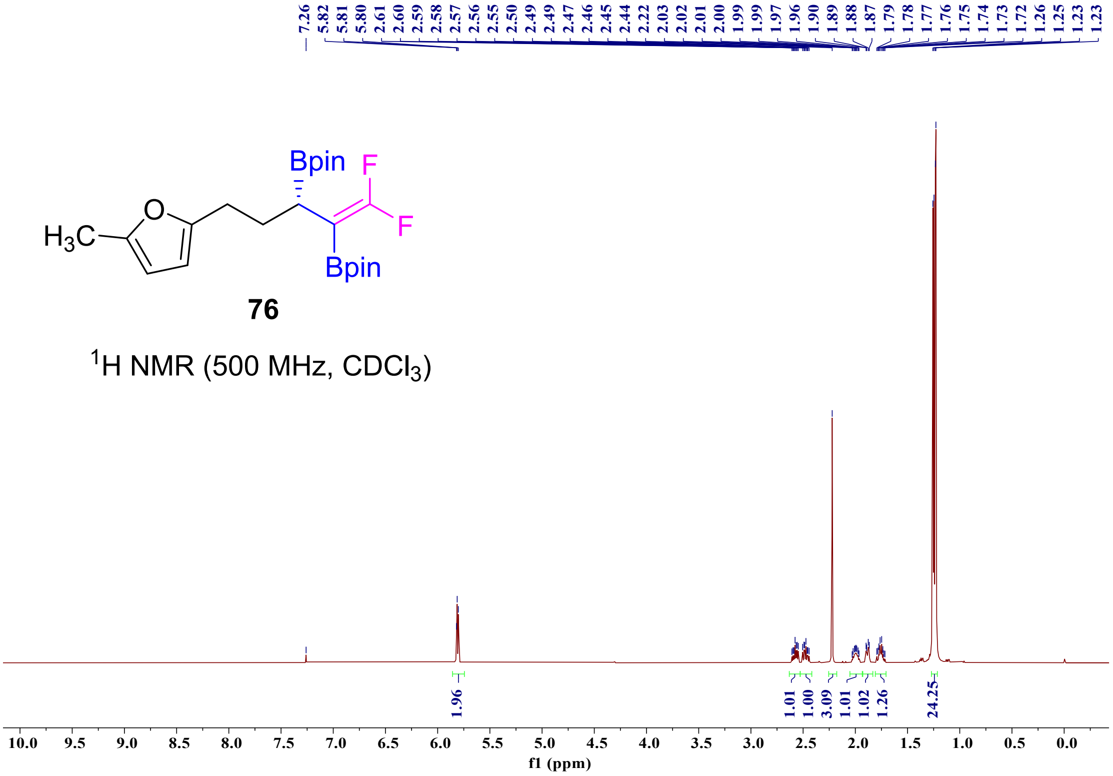

(S)-2,2'-(1,1-difluoro-5-(5-methylfuran-2-yl)pent-1-ene-2,3-diyl)bis(4,4,5,5-tetramethyl-1,3,2-dioxaborolane) (76)

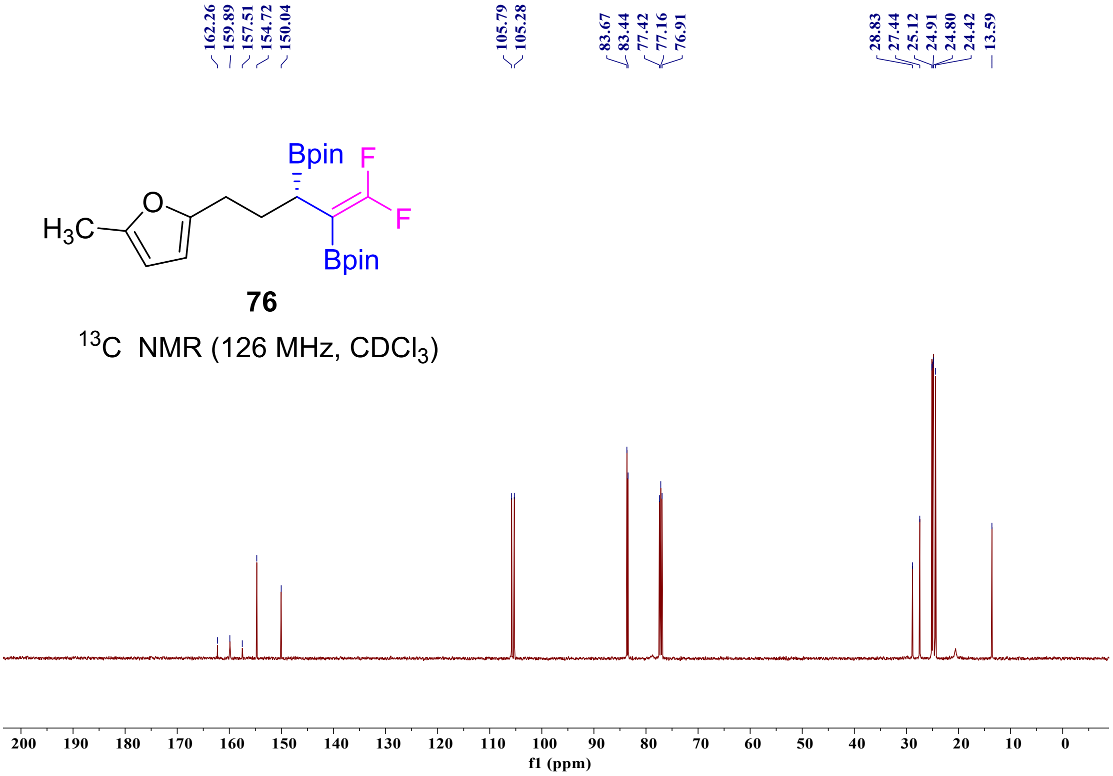

(S)-2,2'-(1,1-difluoro-5-(5-methylfuran-2-yl)pent-1-ene-2,3-diyl)bis(4,4,5,5-tetramethyl-1,3,2-dioxaborolane) (76)

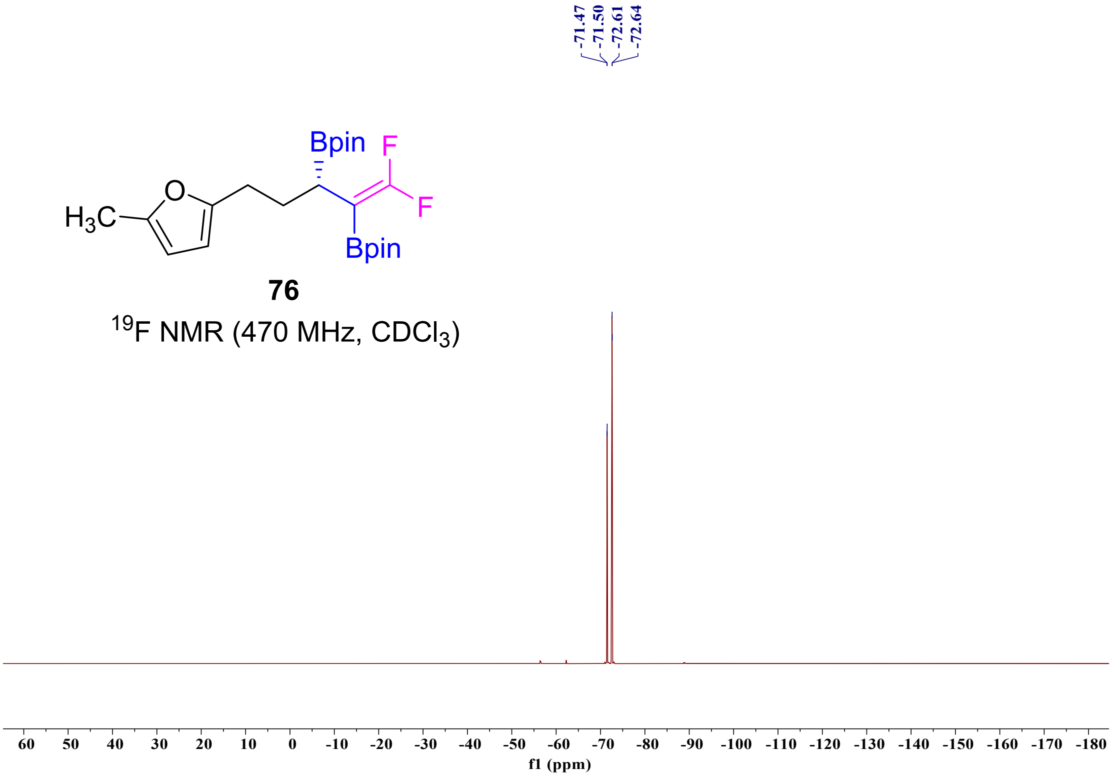

(S)-2,2'-(1,1-difluoro-5-(5-methylfuran-2-yl)pent-1-ene-2,3-diyl)bis(4,4,5,5-tetramethyl-1,3,2-dioxaborolane) (76)

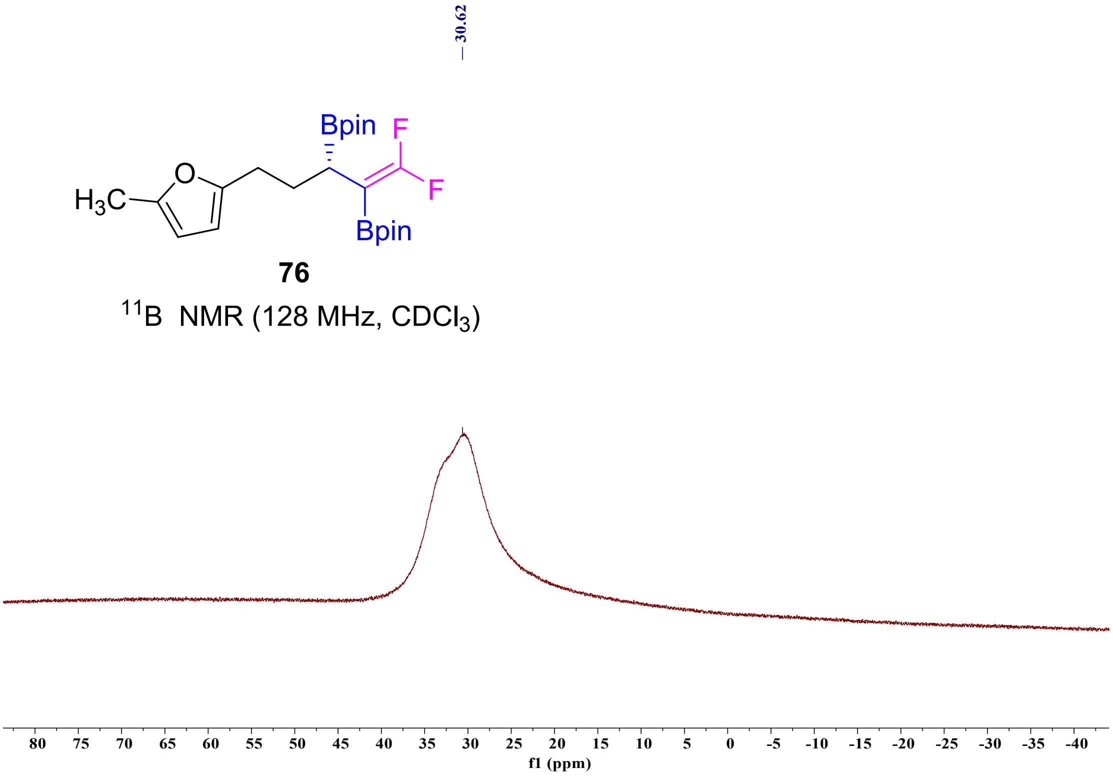

(S)-2,2'-(1,1-difluoro-6-(thiophen-2-yl)hex-1-ene-2,3-diyl)bis(4,4,5,5-tetramethyl-1,3,2-dioxaborolane) (77)

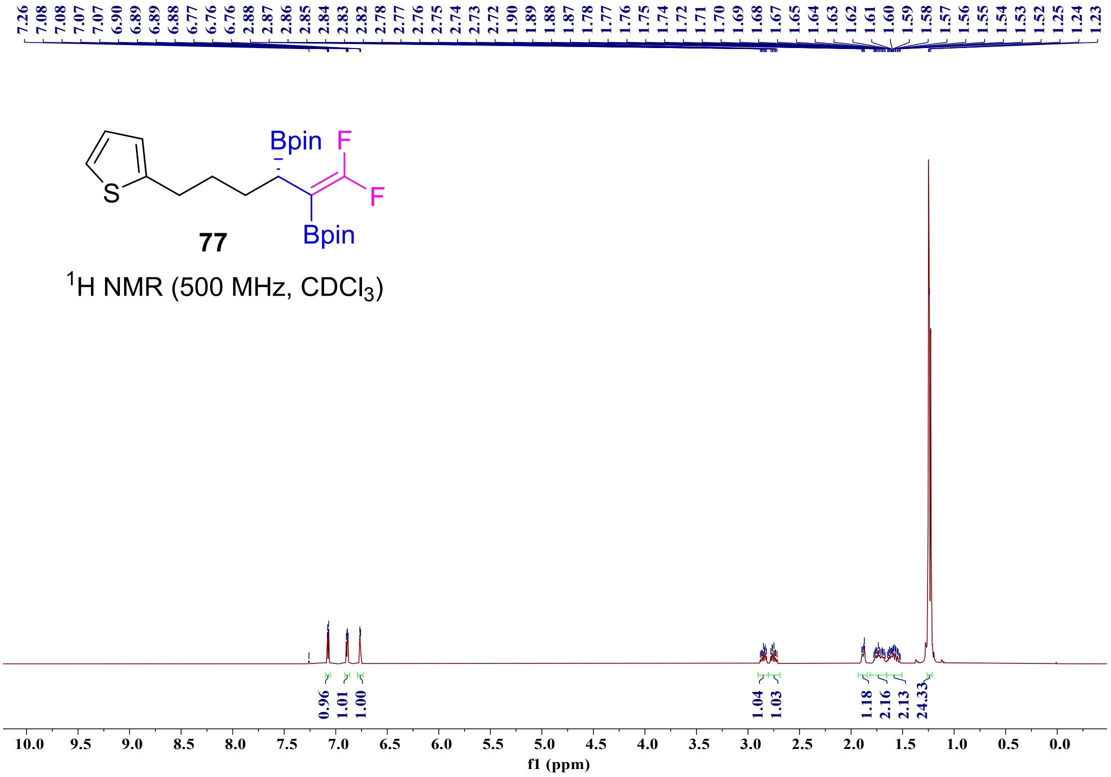

(S)-2,2'-(1,1-difluoro-6-(thiophen-2-yl)hex-1-ene-2,3-diyl)bis(4,4,5,5-tetramethyl-1,3,2-dioxaborolane) (77)

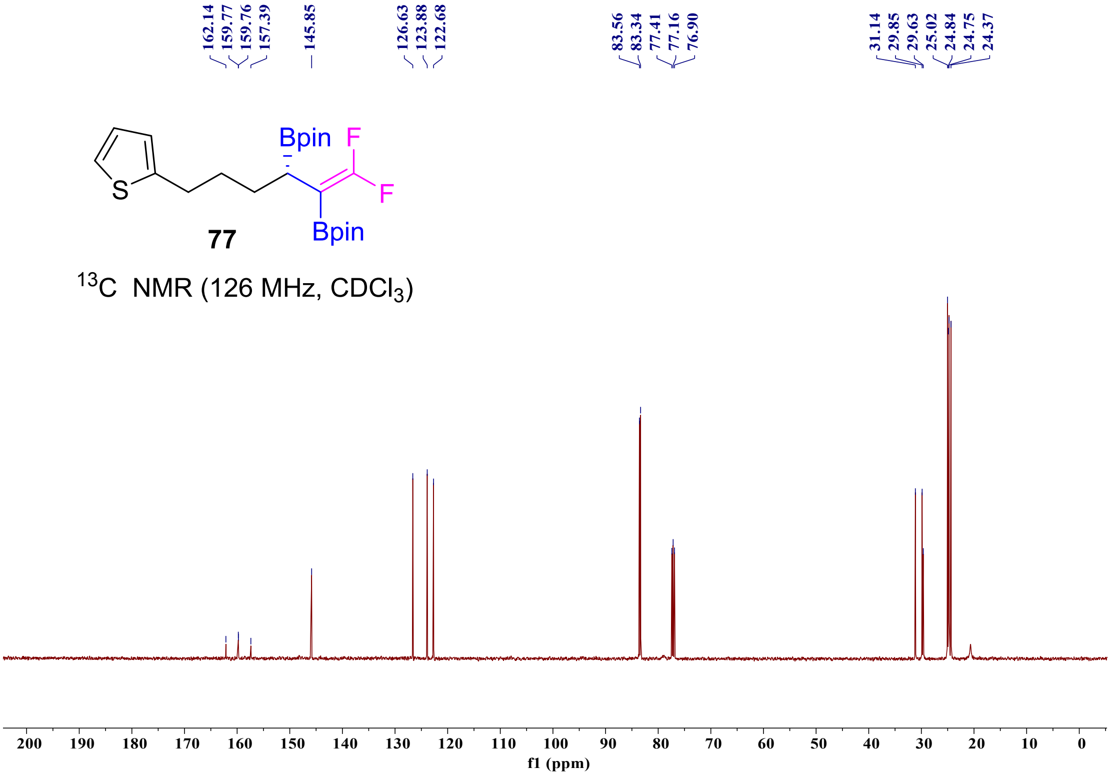

(S)-2,2'-(1,1-difluoro-6-(thiophen-2-yl)hex-1-ene-2,3-diyl)bis(4,4,5,5-tetramethyl-1,3,2-dioxaborolane) (77)

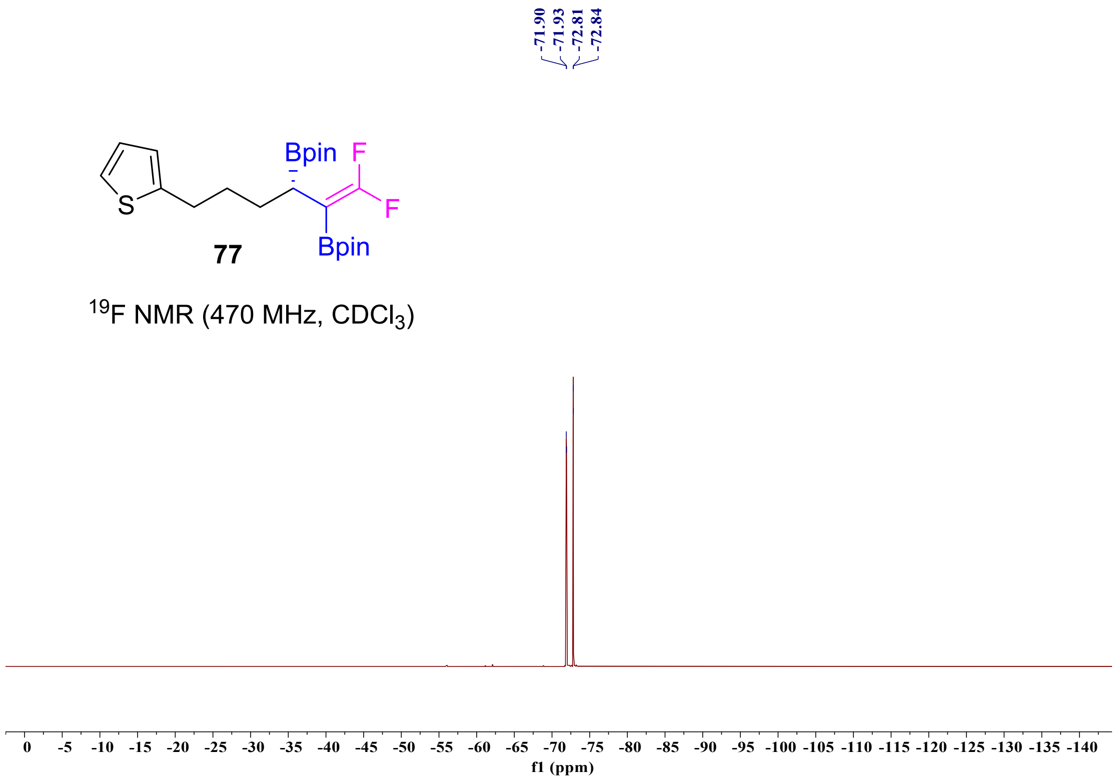

(S)-2,2'-(1,1-difluoro-6-(thiophen-2-yl)hex-1-ene-2,3-diyl)bis(4,4,5,5-tetramethyl-1,3,2-dioxaborolane) (77)

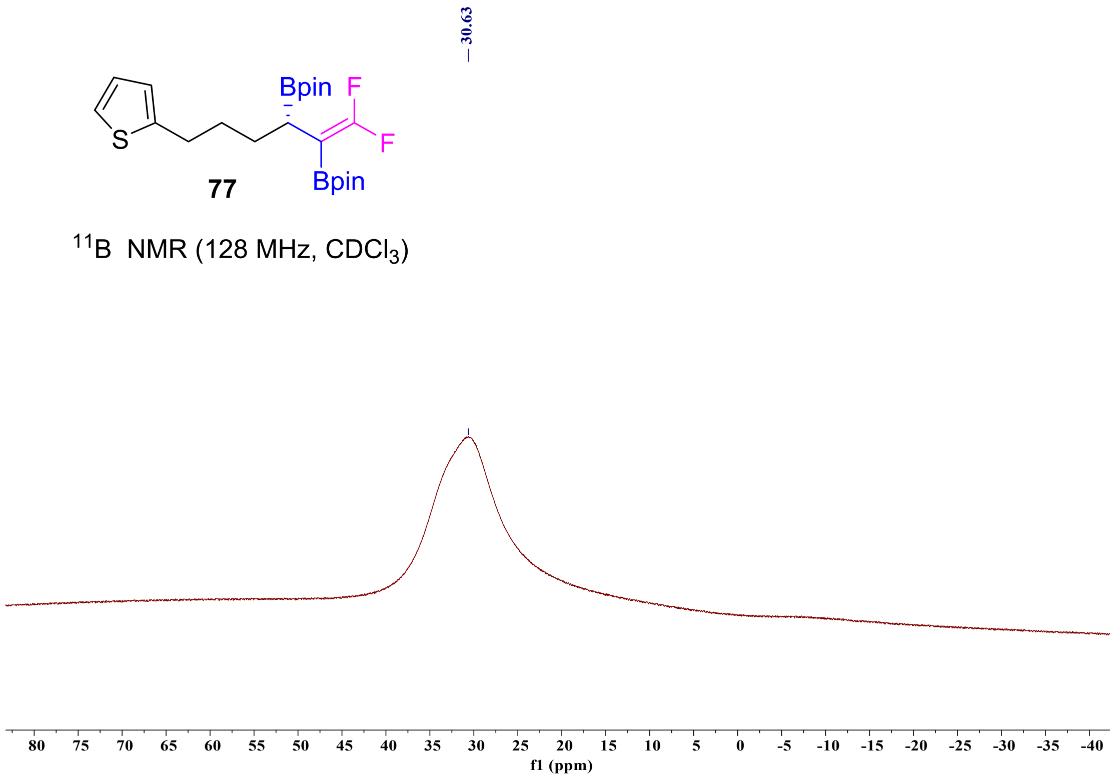

(S)-2,2'-(1,1-difluoro-4-(tetrahydro-2H-pyran-4-yl)but-1-ene-2,3-diyl)bis(4,4,5,5-tetramethyl-1,3,2-dioxaborolane) (78)

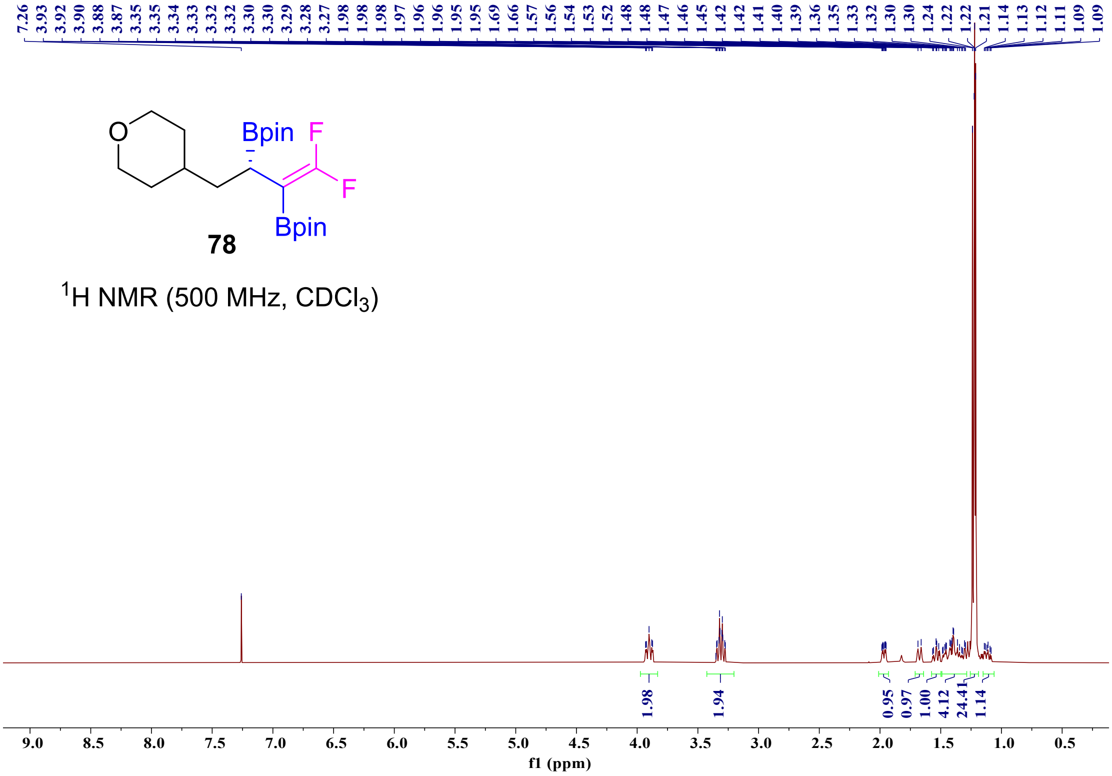

(S)-2,2'-(1,1-difluoro-4-(tetrahydro-2H-pyran-4-yl)but-1-ene-2,3-diyl)bis(4,4,5,5-tetramethyl-1,3,2-dioxaborolane) (78)

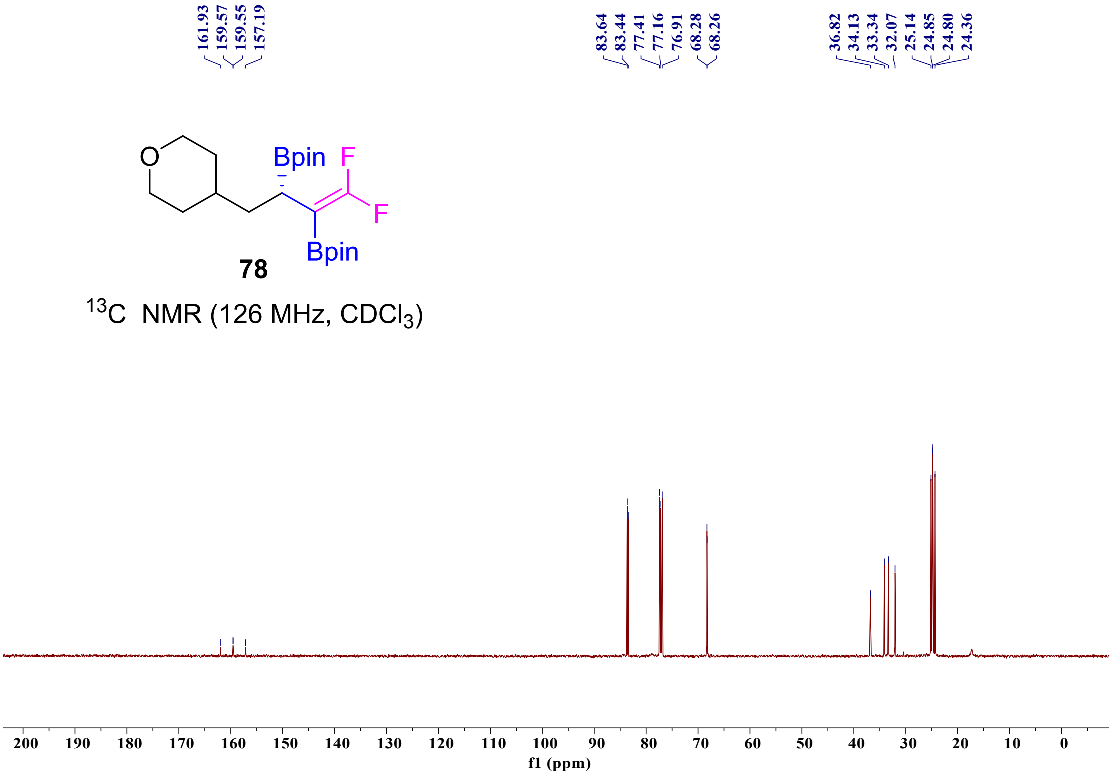

(S)-2,2'-(1,1-difluoro-4-(tetrahydro-2H-pyran-4-yl)but-1-ene-2,3-diyl)bis(4,4,5,5-tetramethyl-1,3,2-dioxaborolane) (78)

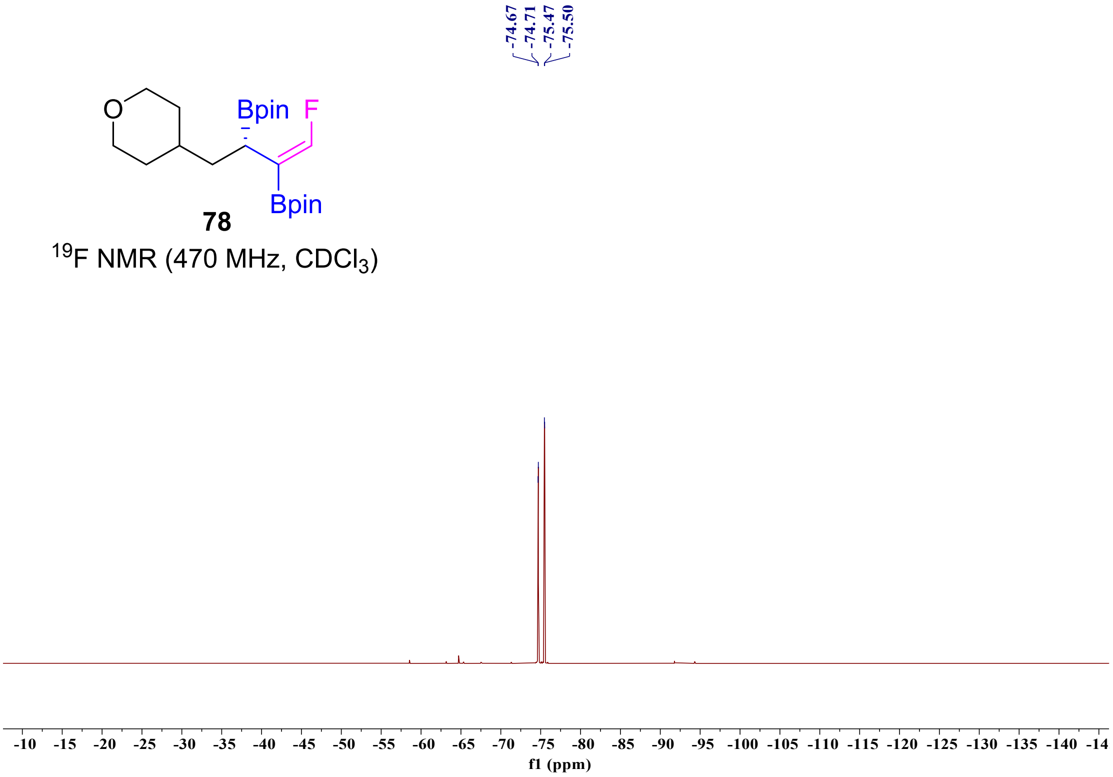

(S)-2,2'-(1,1-difluoro-4-(tetrahydro-2H-pyran-4-yl)but-1-ene-2,3-diyl)bis(4,4,5,5-tetramethyl-1,3,2-dioxaborolane) (78)

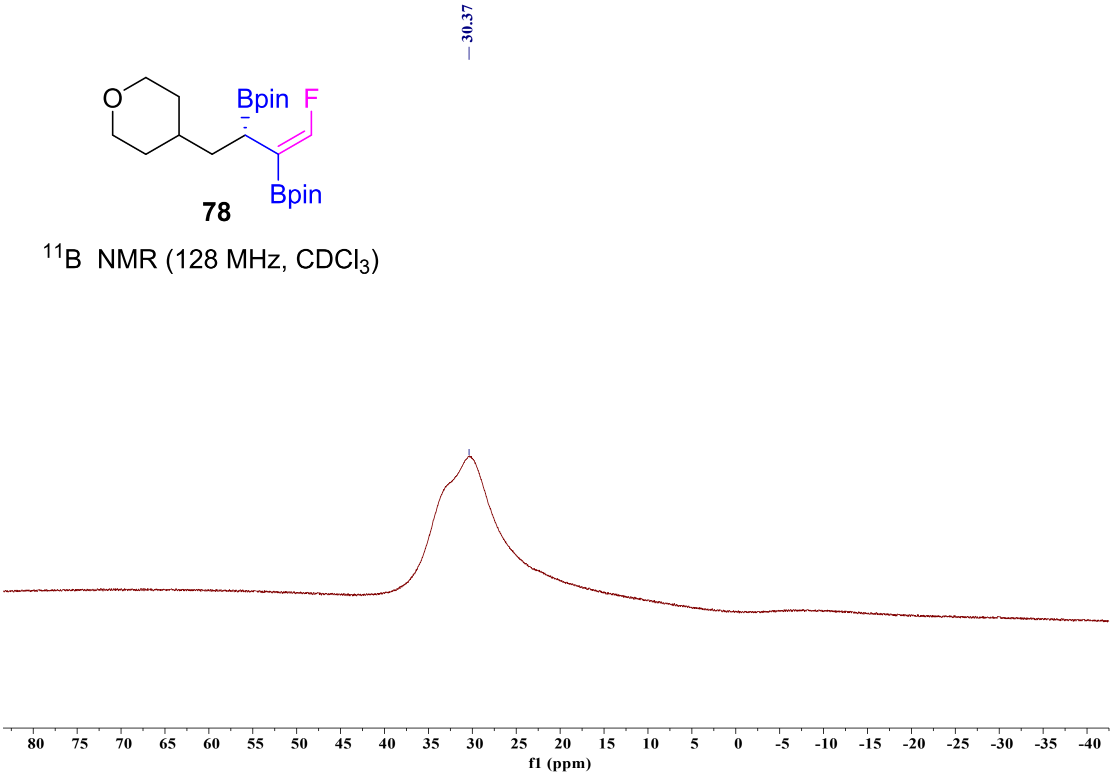

(S)-benzyl4-(3,3-difluoro-1,2-bis(4,4,5,5-tetramethyl-1,3,2-dioxaborolan-2-yl)allyl)piperidine-1-carboxylate (79)

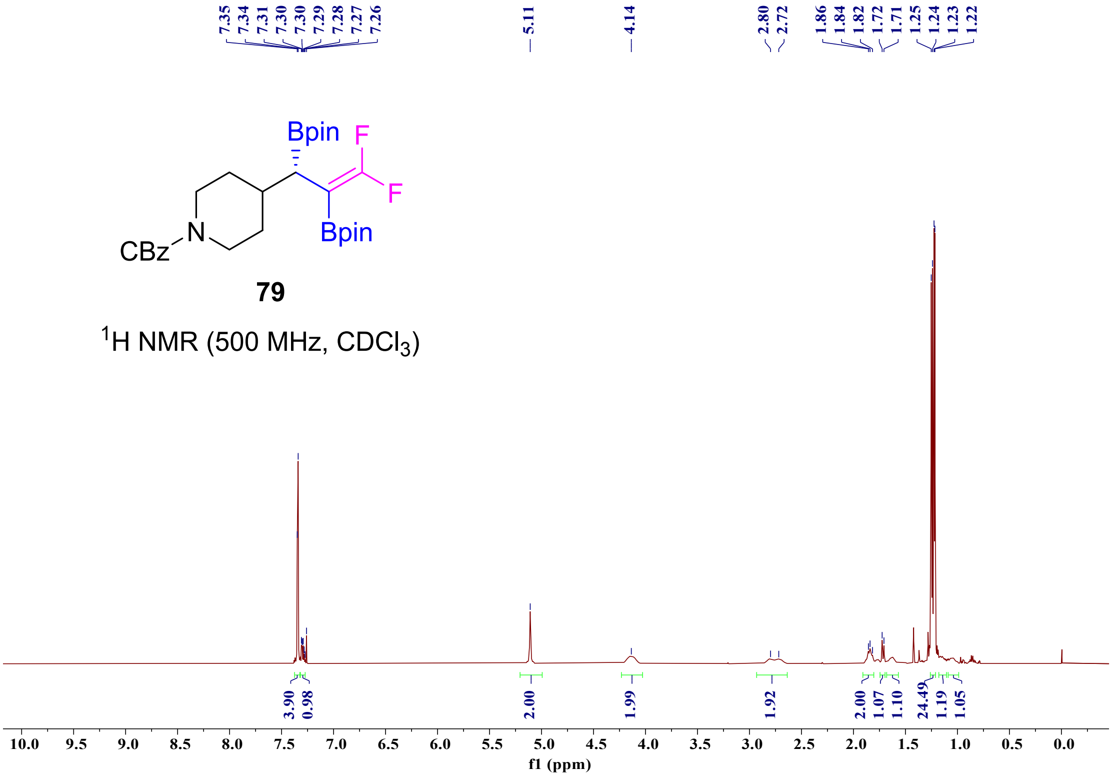

benzyl4-(3,3-difluoro-1,2-bis(4,4,5,5-tetramethyl-1,3,2-dioxaborolan-2-yl)allyl)piperidine-1-carboxylate(26)

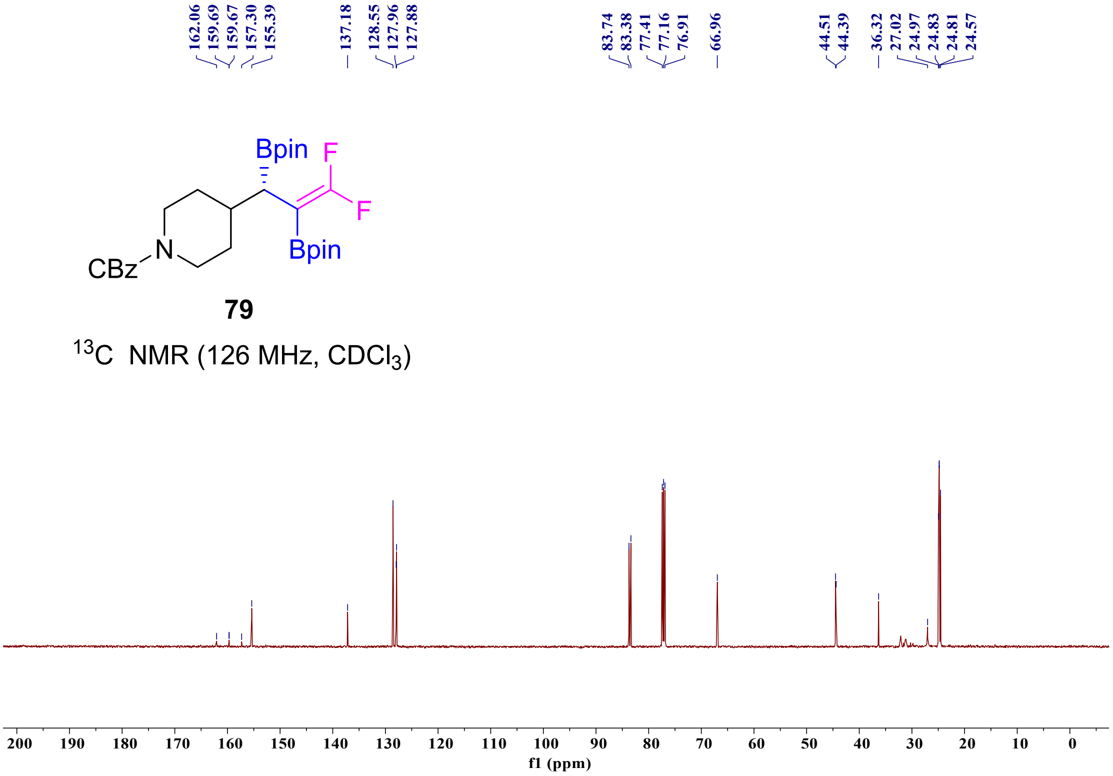

benzyl4-(3,3-difluoro-1,2-bis(4,4,5,5-tetramethyl-1,3,2-dioxaborolan-2-yl)allyl)piperidine-1-carboxylate(26)

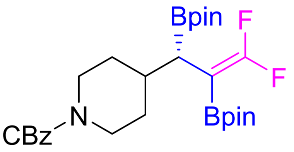

79

^{19}F NMR (470 MHz, $CDCl_3$)

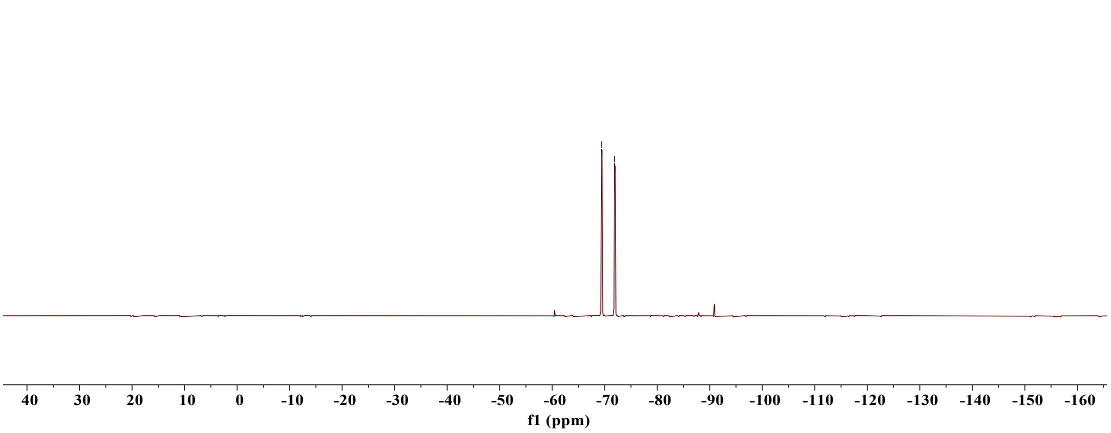

benzyl4-(3,3-difluoro-1,2-bis(4,4,5,5-tetramethyl-1,3,2-dioxaborolan-2-yl)allyl)piperidine-1-carboxylate(26)

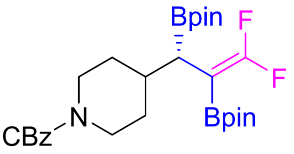

79

^{11}B NMR (128 MHz, $CDCl_3$)

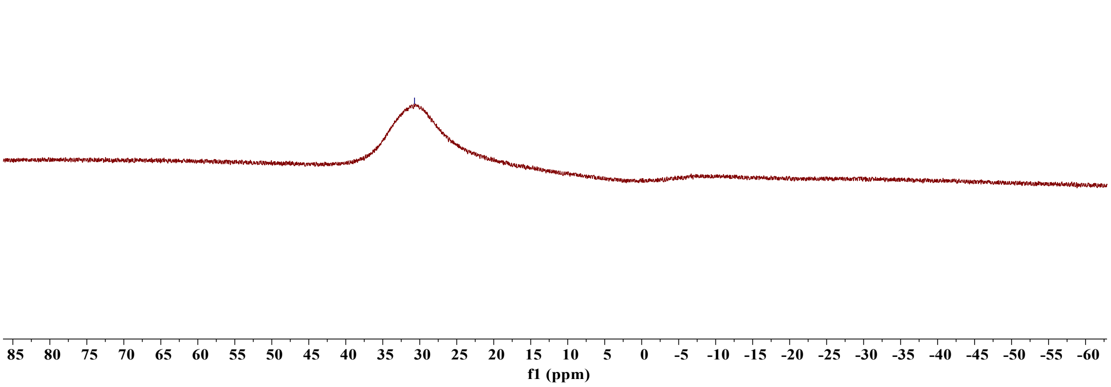

(S)tert-butyl4-(3,3-difluoro-1,2-bis(4,4,5,5-tetramethyl-1,3,2-dioxaborolan-2-yl)allyl)piperidine-1-carboxylate (80)

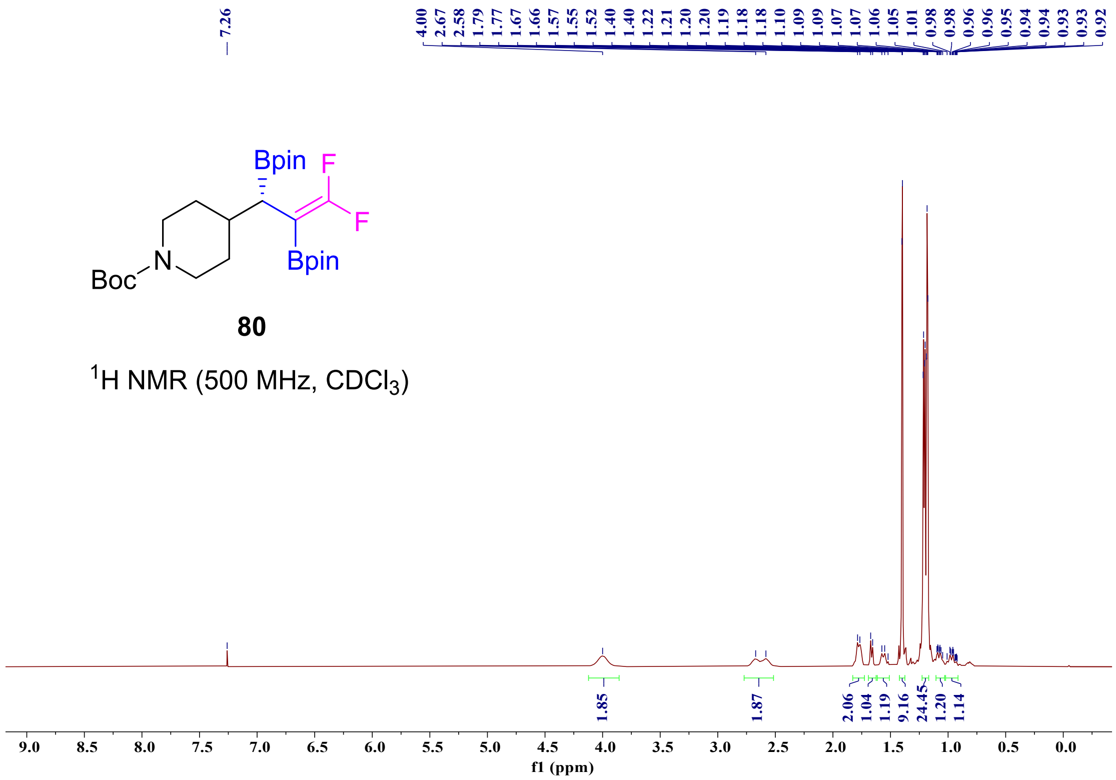

(S)tert-butyl4-(3,3-difluoro-1,2-bis(4,4,5,5-tetramethyl-1,3,2-dioxaborolan-2-yl)allyl)piperidine-1-carboxylate (80)

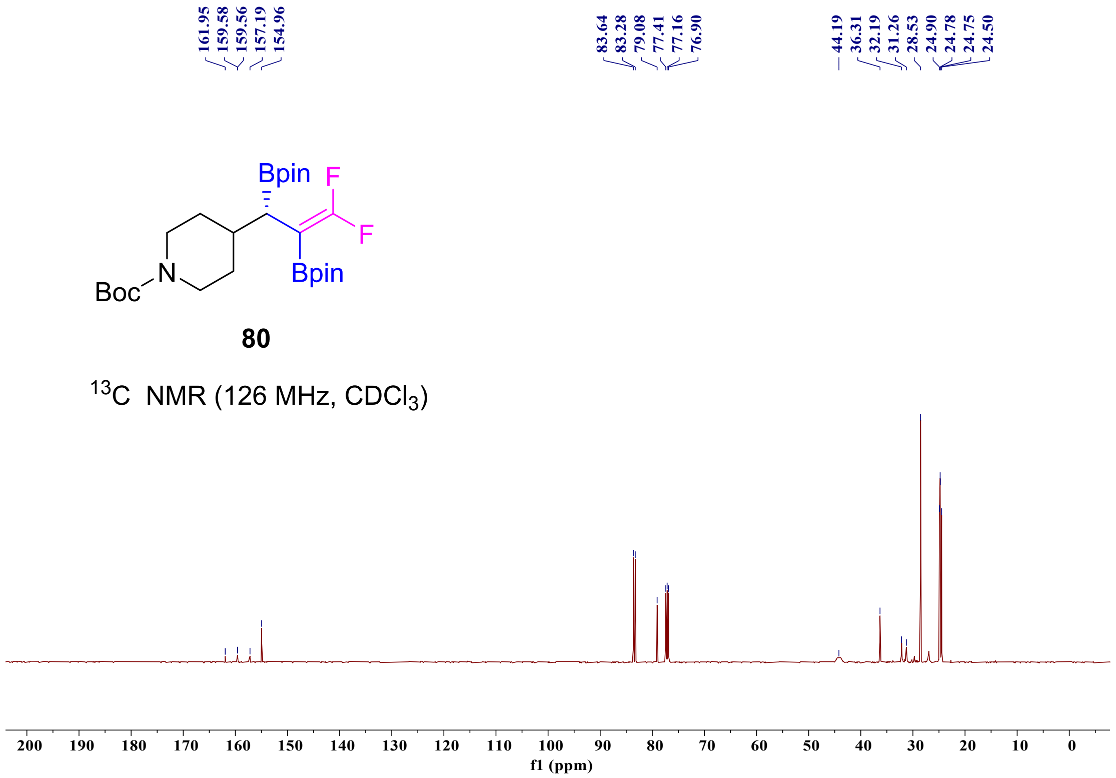

(S)tert-butyl4-(3,3-difluoro-1,2-bis(4,4,5,5-tetramethyl-1,3,2-dioxaborolan-2-yl)allyl)piperidine-1-carboxylate (80)

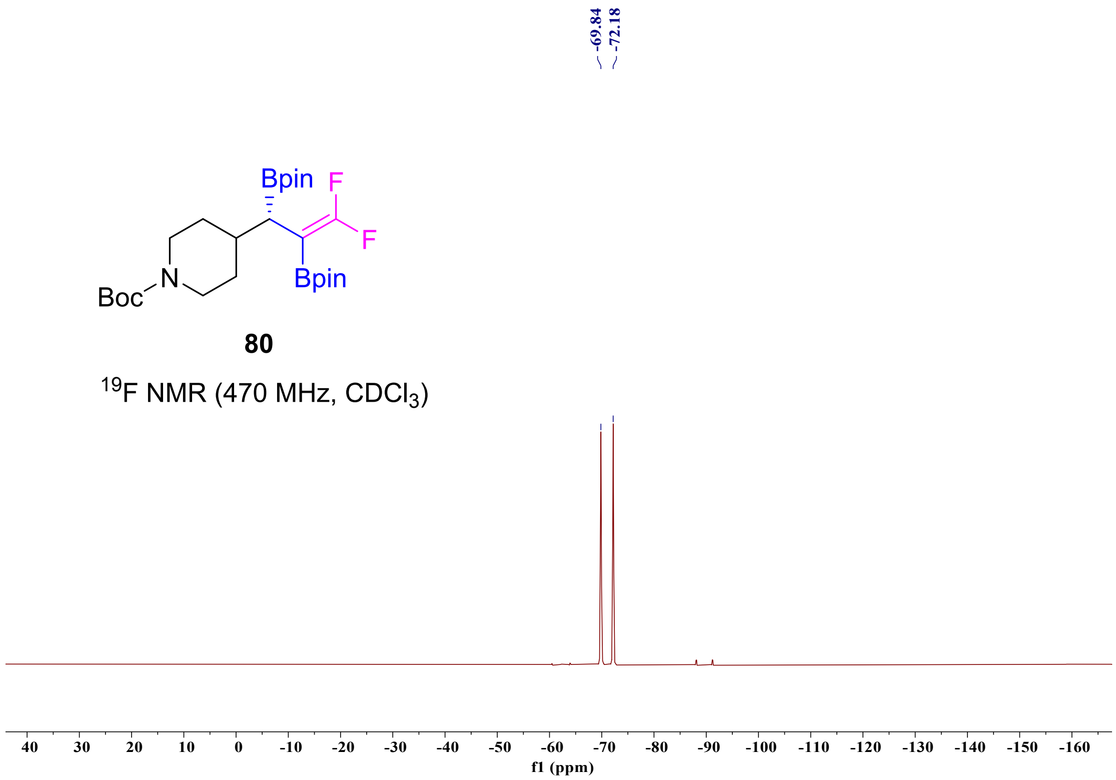

(S)tert-butyl4-(3,3-difluoro-1,2-bis(4,4,5,5-tetramethyl-1,3,2-dioxaborolan-2-yl)allyl)piperidine-1-carboxylate (80)

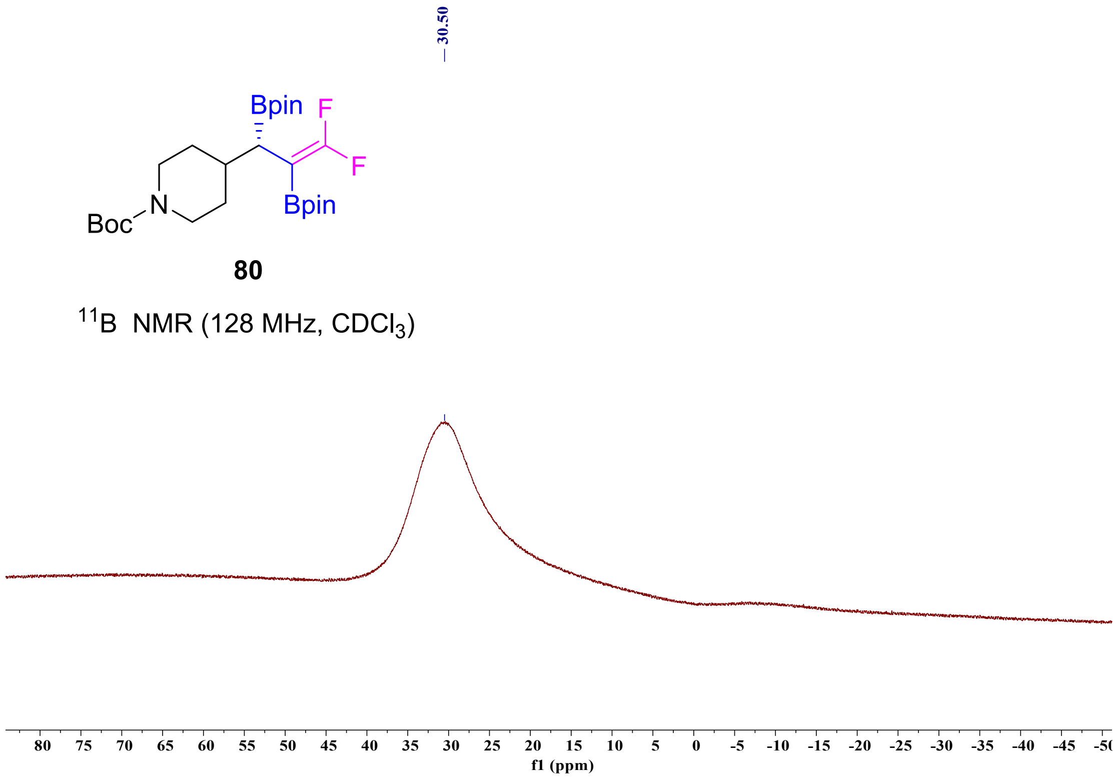

(S)-4-(4,4-difluoro-2,3-bis(4,4,5,5-tetramethyl-1,3,2-dioxaborolan-2-yl)but-3-en-1-yl)-1-tosylpiperidine (81)

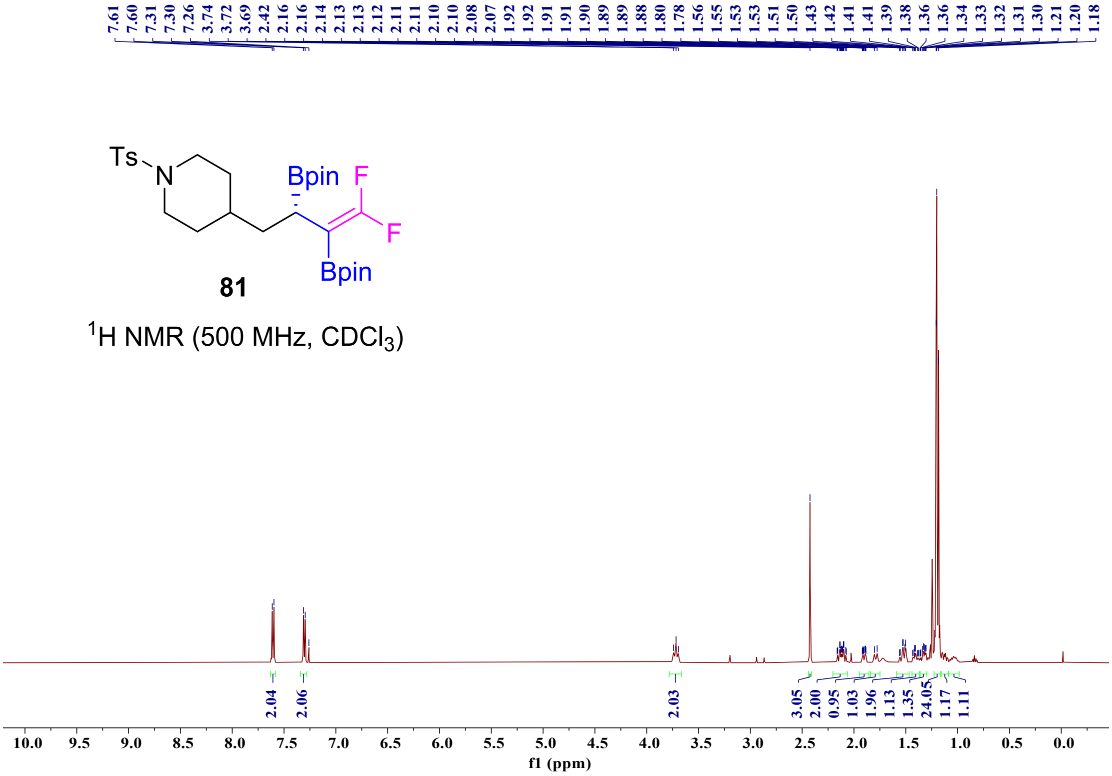

(S)-4-(4,4-difluoro-2,3-bis(4,4,5,5-tetramethyl-1,3,2-dioxaborolan-2-yl)but-3-en-1-yl)-1-tosylpiperidine (81)

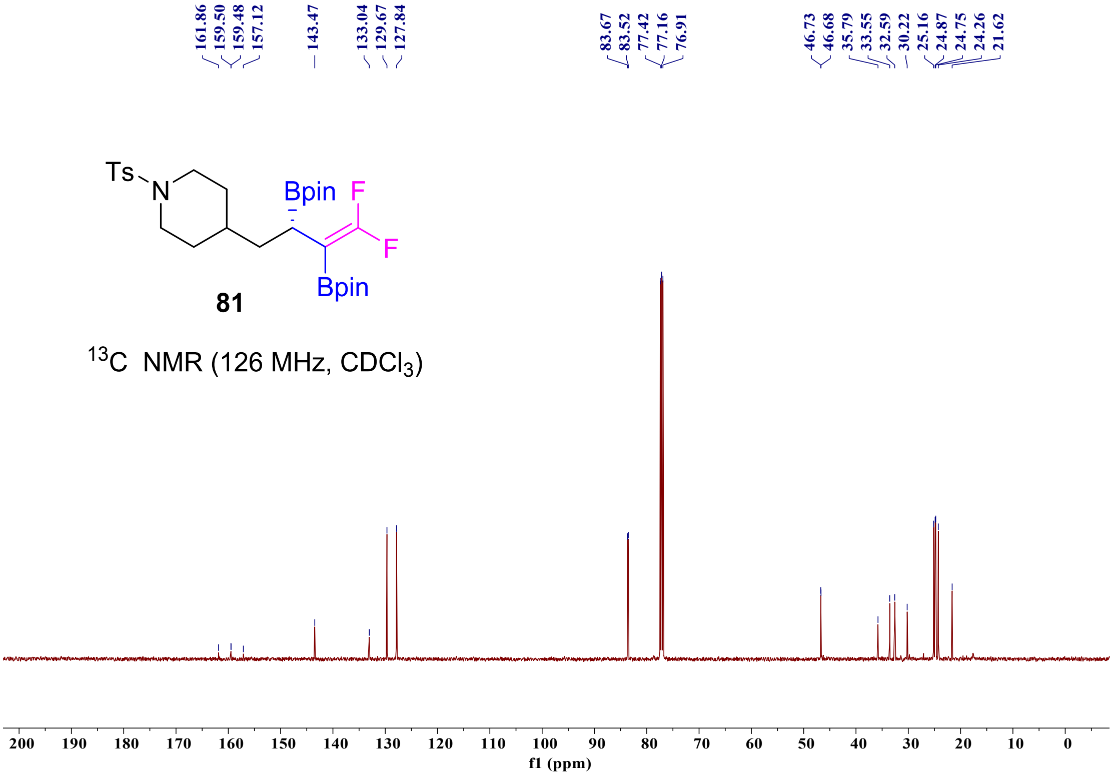

(S)-4-(4,4-difluoro-2,3-bis(4,4,5,5-tetramethyl-1,3,2-dioxaborolan-2-yl)but-3-en-1-yl)-1-tosylpiperidine (81)

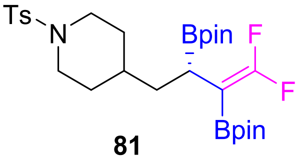

81

^{19}F NMR (470 MHz, $CDCl_3$)

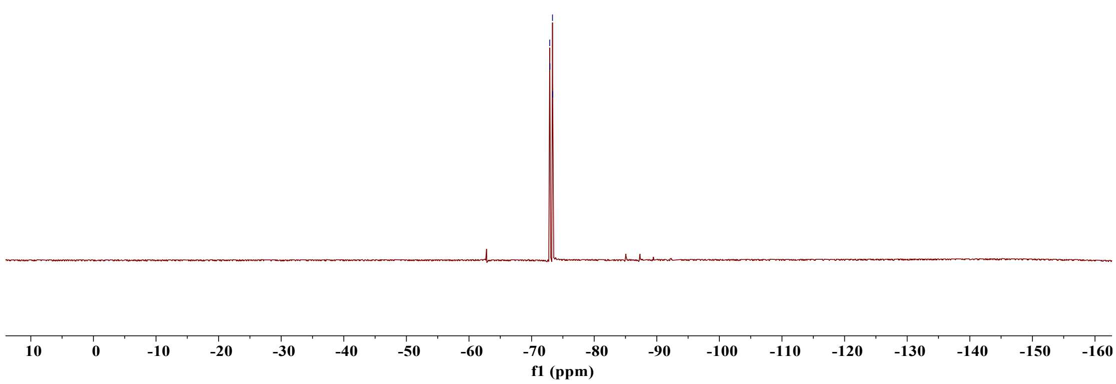

(S)-4-(4,4-difluoro-2,3-bis(4,4,5,5-tetramethyl-1,3,2-dioxaborolan-2-yl)but-3-en-1-yl)-1-tosylpiperidine (81)

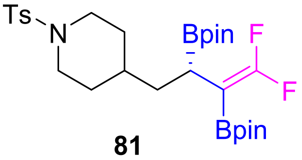

81

^{11}B NMR (128 MHz, $CDCl_3$)

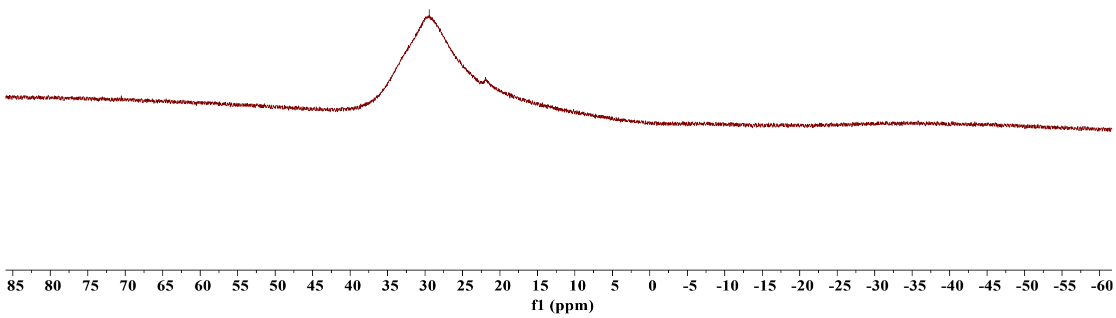

(S)-2,2'-(1,1-difluoro-5-phenoxypent-1-ene-2,3-diyl)bis(4,4,5,5-tetramethyl-1,3,2-dioxaborolane) (82)

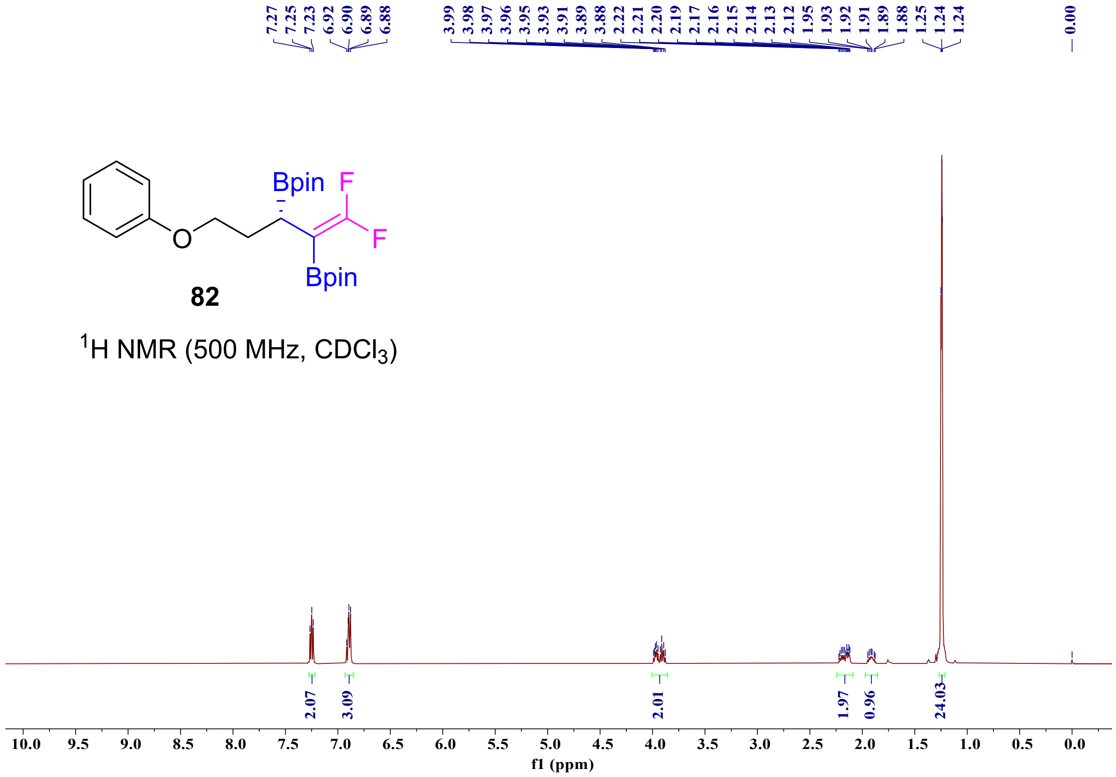

(S)-2,2'-(1,1-difluoro-5-phenoxypent-1-ene-2,3-diyl)bis(4,4,5,5-tetramethyl-1,3,2-dioxaborolane) (82)

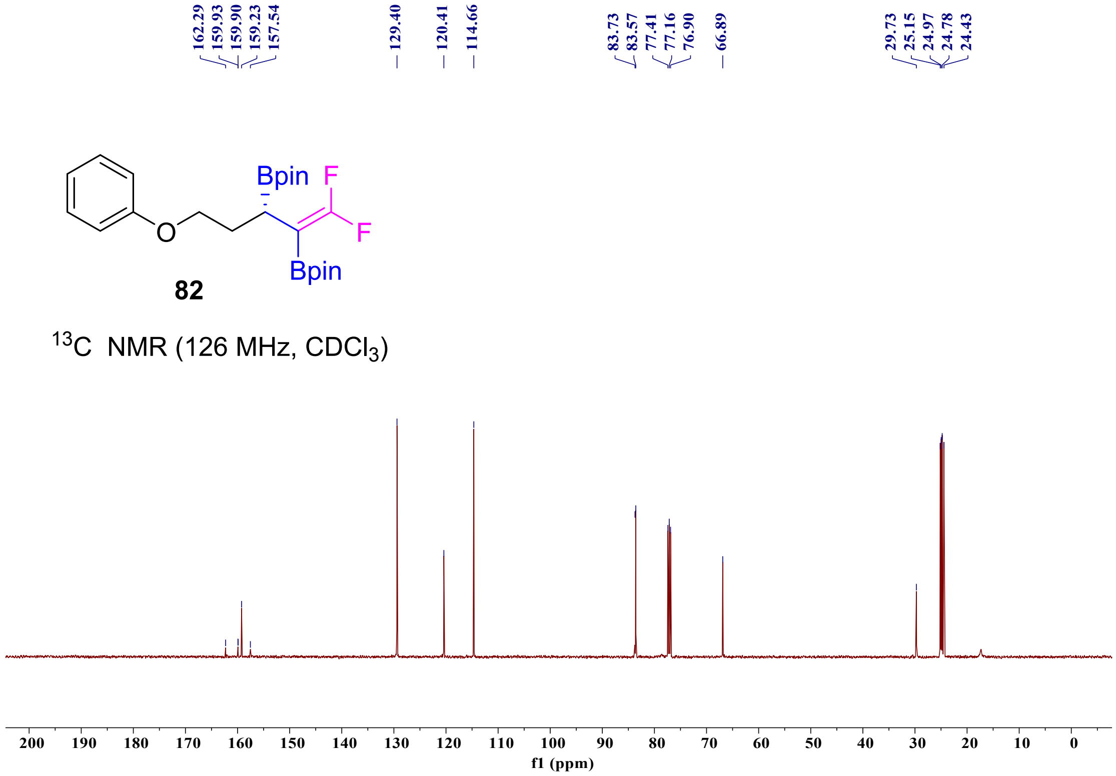

(S)-2,2'-(1,1-difluoro-5-phenoxypent-1-ene-2,3-diyl)bis(4,4,5,5-tetramethyl-1,3,2-dioxaborolane) (82)

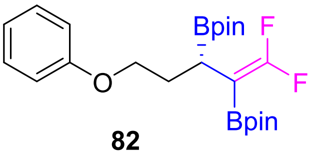

82

^{19}F NMR (470 MHz, $CDCl_3$)

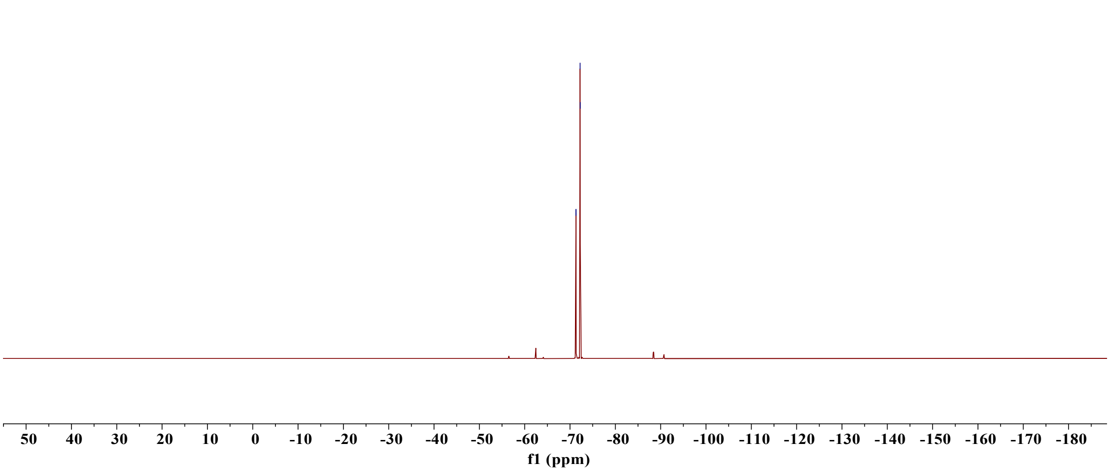

(S)-2,2'-(1,1-difluoro-5-phenoxypent-1-ene-2,3-diyl)bis(4,4,5,5-tetramethyl-1,3,2-dioxaborolane) (82)

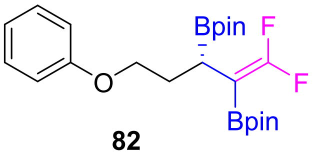

82

^{11}B NMR (128 MHz, $CDCl_3$)

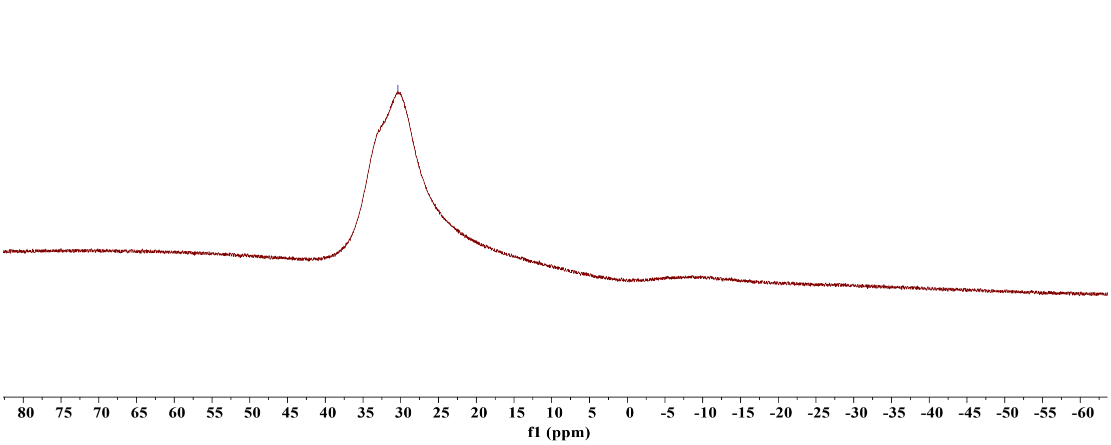

ethyl (S)-8,8-difluoro-6,7-bis(4,4,5,5-tetramethyl-1,3,2-dioxaborolan-2-yl)oct-7-enoate (83)

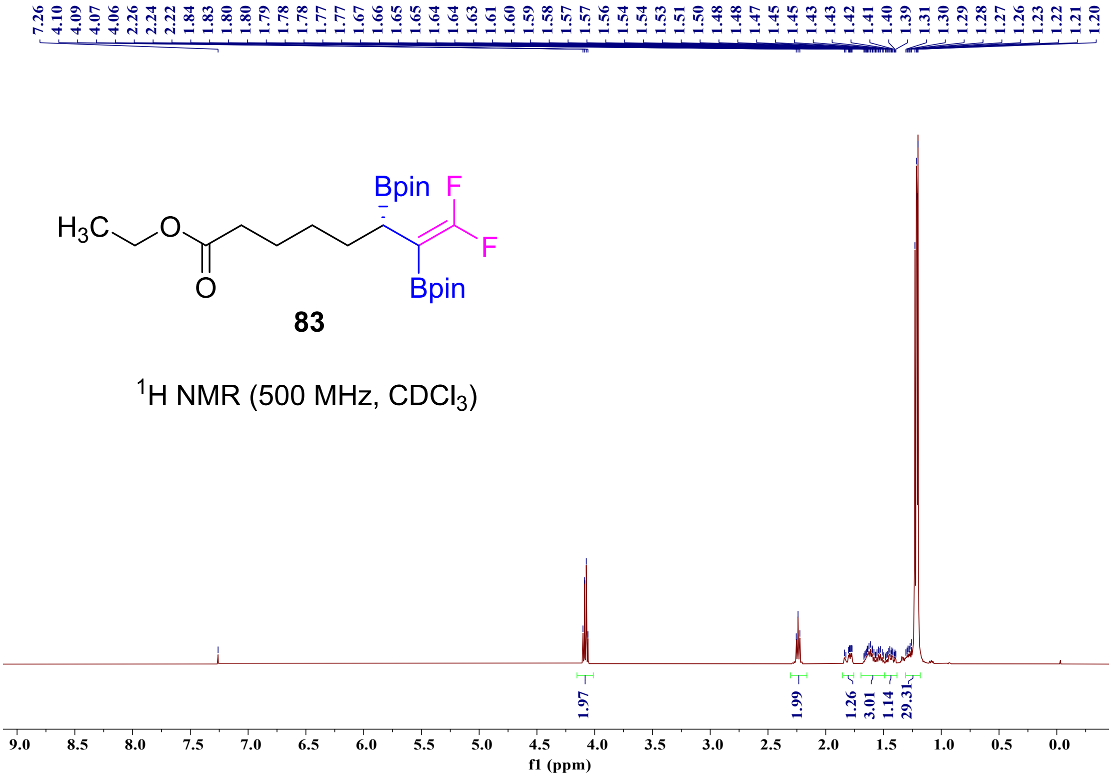

ethyl (S)-8,8-difluoro-6,7-bis(4,4,5,5-tetramethyl-1,3,2-dioxaborolan-2-yl)oct-7-enoate (83)

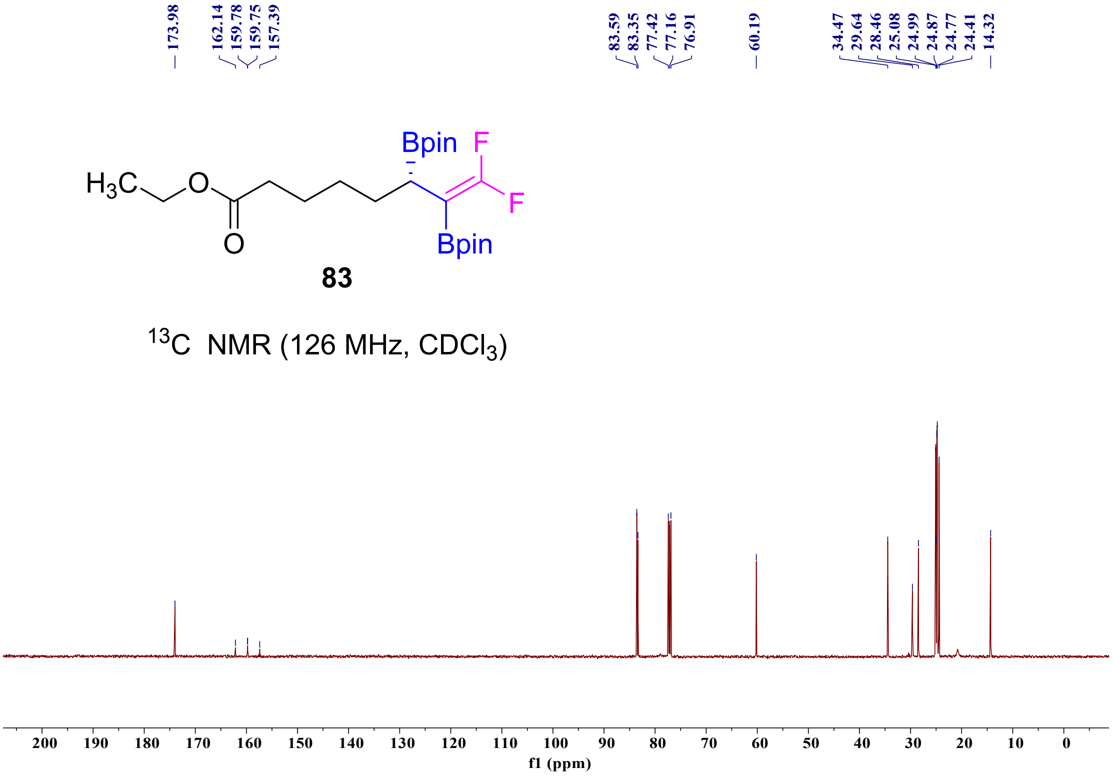

ethyl (S)-8,8-difluoro-6,7-bis(4,4,5,5-tetramethyl-1,3,2-dioxaborolan-2-yl)oct-7-enoate (83)

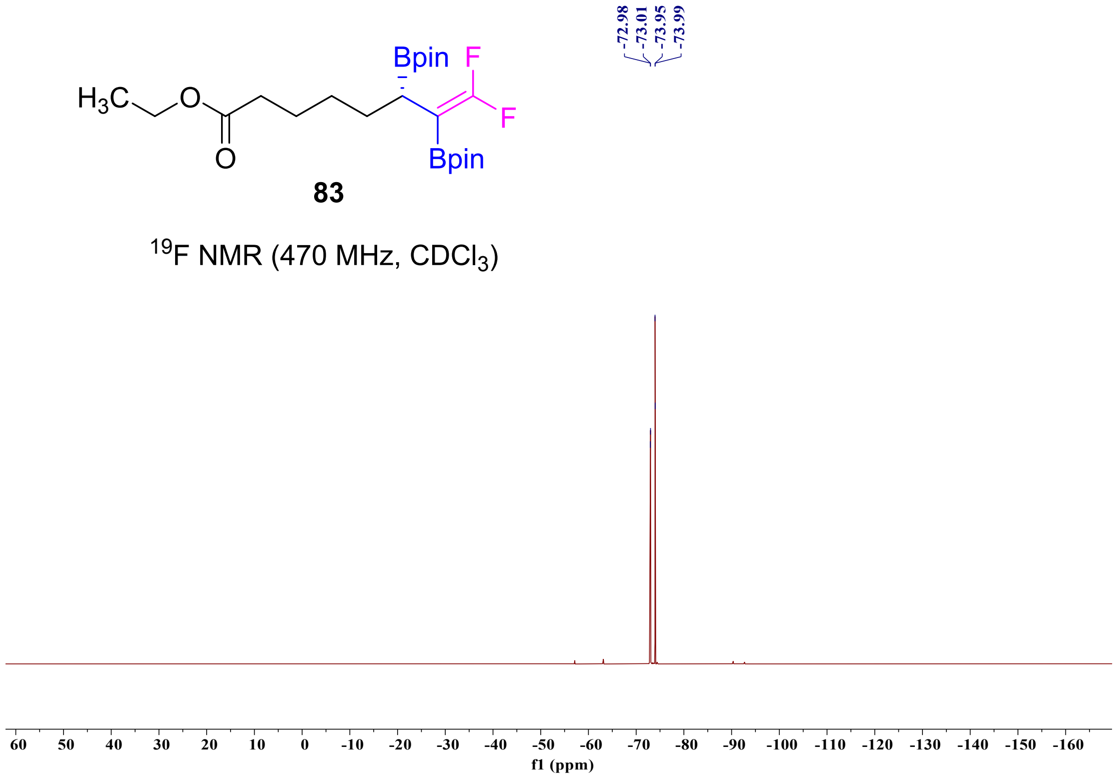

ethyl (S)-8,8-difluoro-6,7-bis(4,4,5,5-tetramethyl-1,3,2-dioxaborolan-2-yl)oct-7-enoate (83)

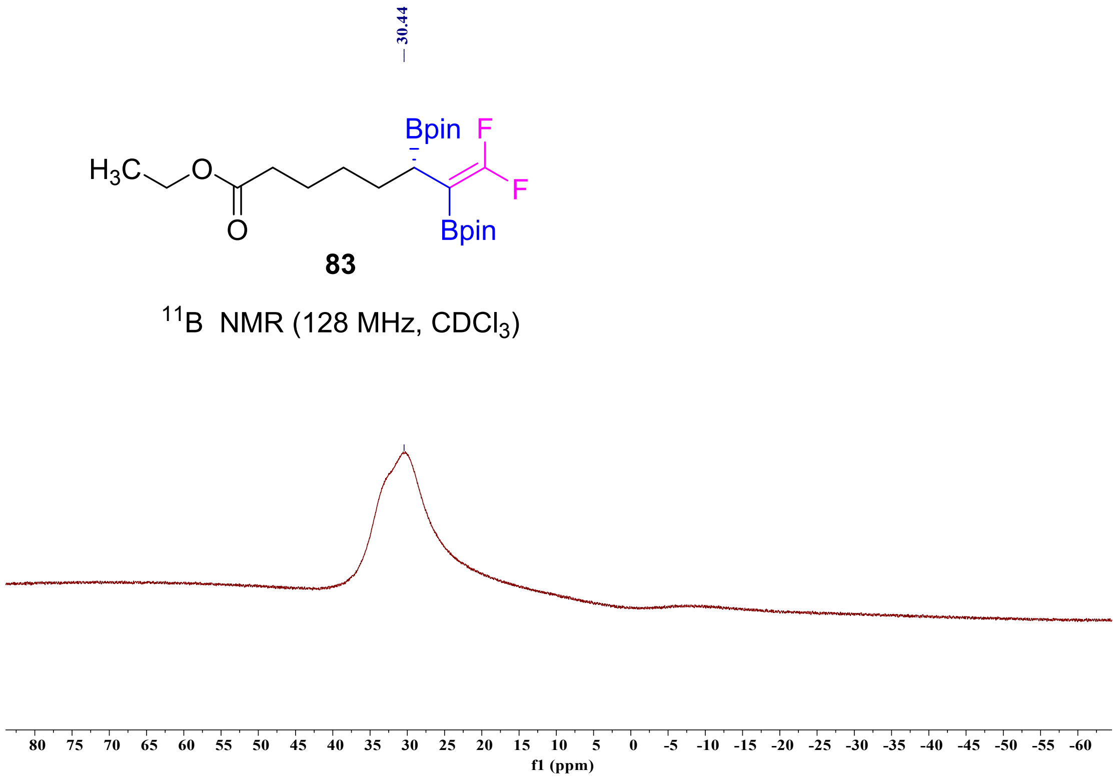

(S)-2,2'-(1-(4,4-difluorocyclohexyl)-3,3-difluoroprop-2-ene-1,2-diyl)bis(4,4,5,5-tetramethyl-1,3,2-dioxaborolane) (84)

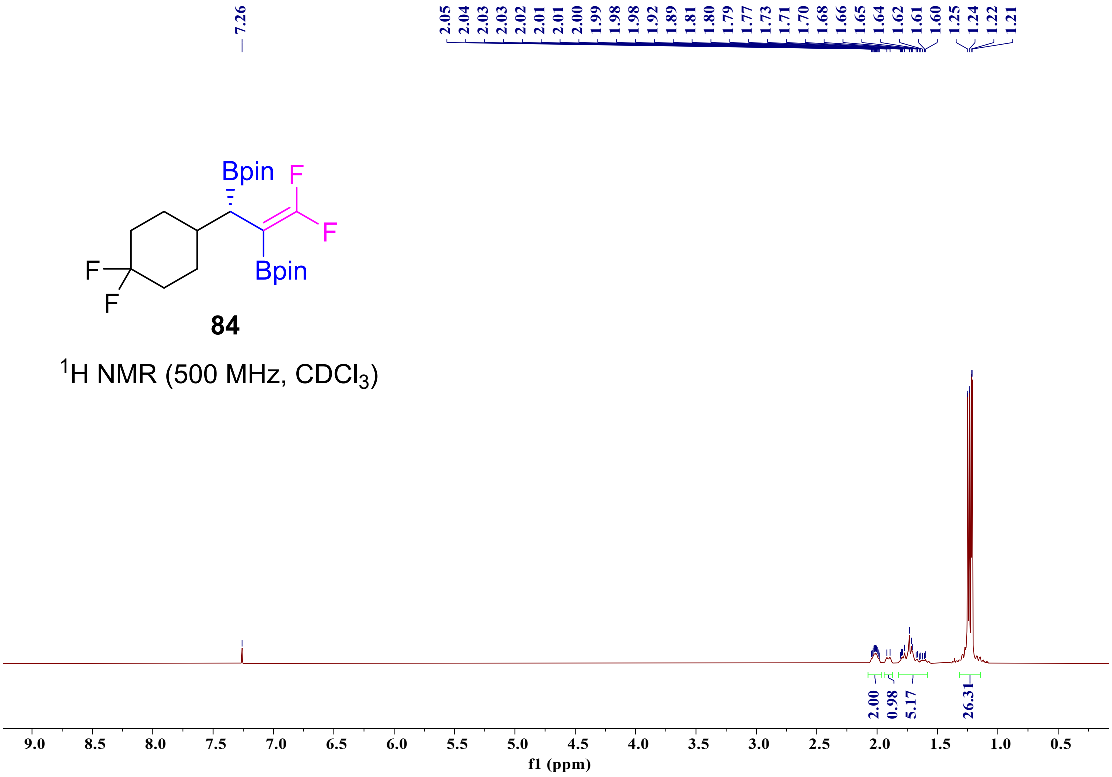

(S)-2,2'-(1-(4,4-difluorocyclohexyl)-3,3-difluoroprop-2-ene-1,2-diyl)bis(4,4,5,5-tetramethyl-1,3,2-dioxaborolane) (84)

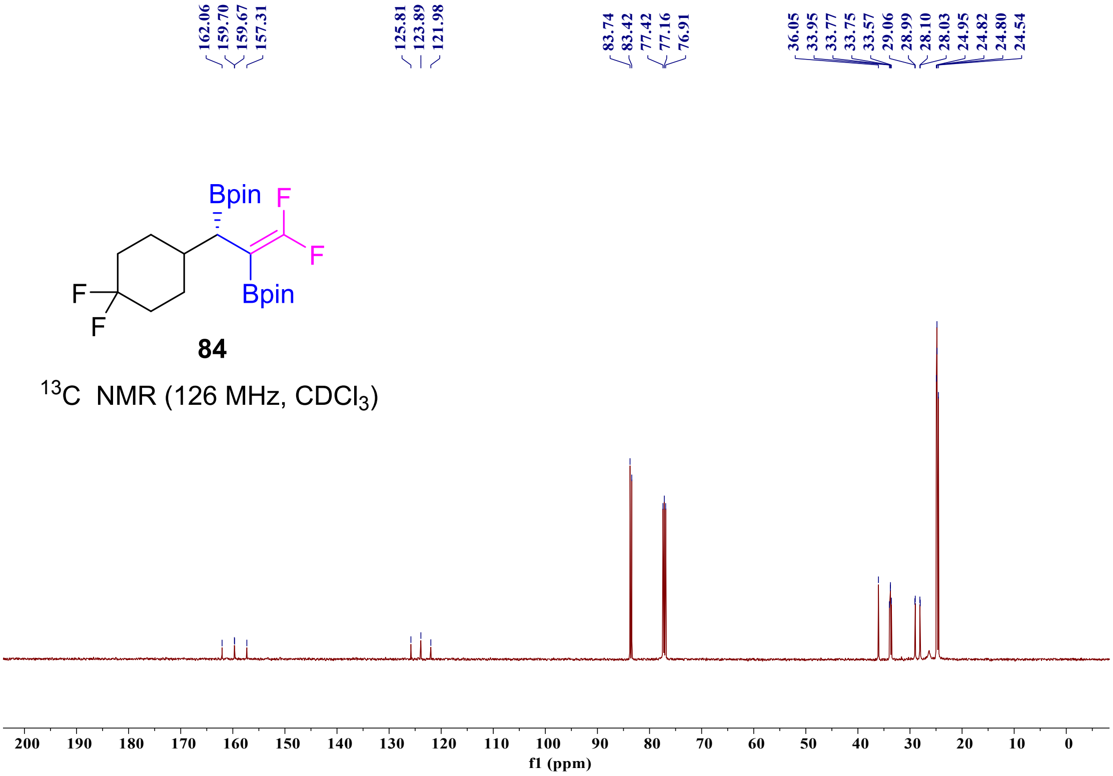

(S)-2,2'-(1-(4,4-difluorocyclohexyl)-3,3-difluoroprop-2-ene-1,2-diyl)bis(4,4,5,5-tetramethyl-1,3,2-dioxaborolane) (84)

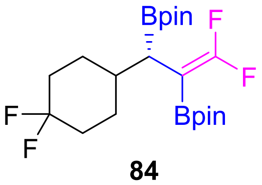

84

^{19}F NMR (470 MHz, $CDCl_3$)

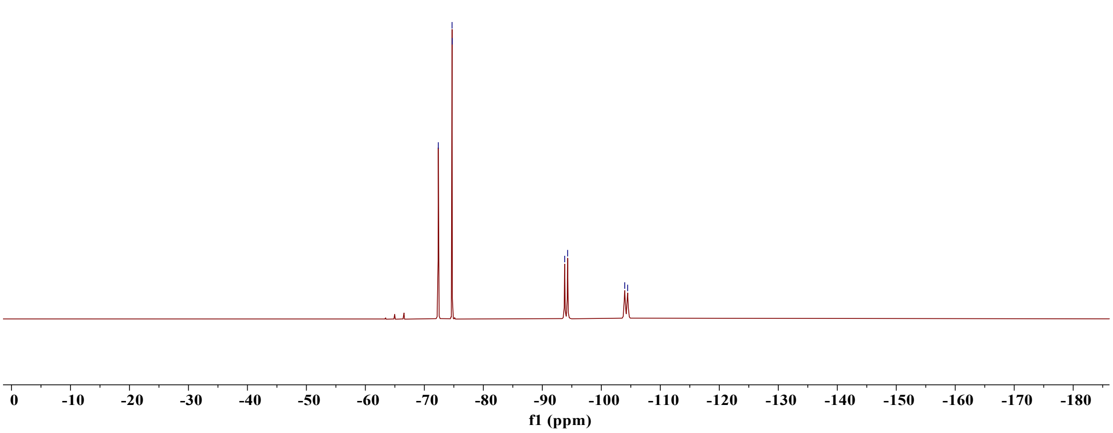

(S)-2,2'-(1-(4,4-difluorocyclohexyl)-3,3-difluoroprop-2-ene-1,2-diyl)bis(4,4,5,5-tetramethyl-1,3,2-dioxaborolane) (84)

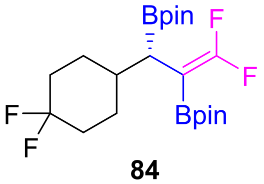

84

^{11}B NMR (128 MHz, $CDCl_3$)

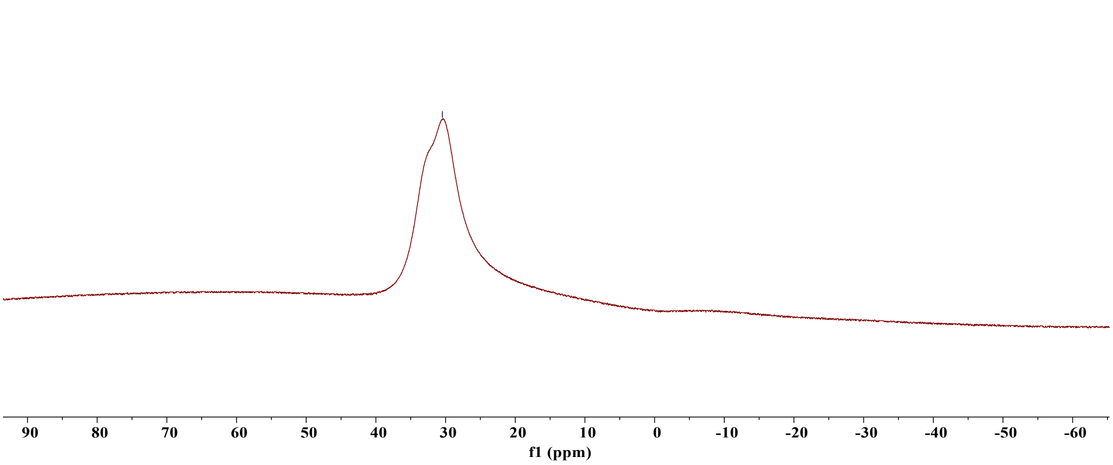

(S)-2,2'-(1-(2,3-dihydro-1H-inden-2-yl)-3,3-difluoroprop-2-ene-1,2-diyl)bis(4,4,5,5-tetramethyl-1,3,2-dioxaborolane) (85)

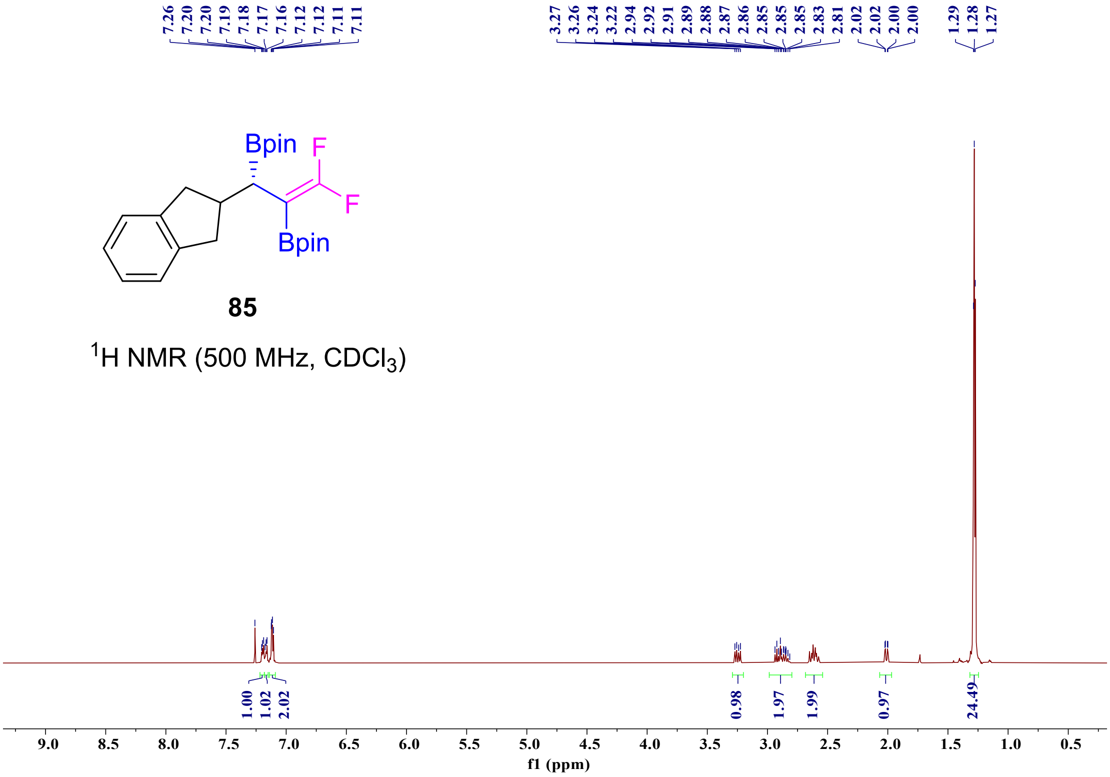

(S)-2,2'-(1-(2,3-dihydro-1H-inden-2-yl)-3,3-difluoroprop-2-ene-1,2-diyl)bis(4,4,5,5-tetramethyl-1,3,2-dioxaborolane) (85)

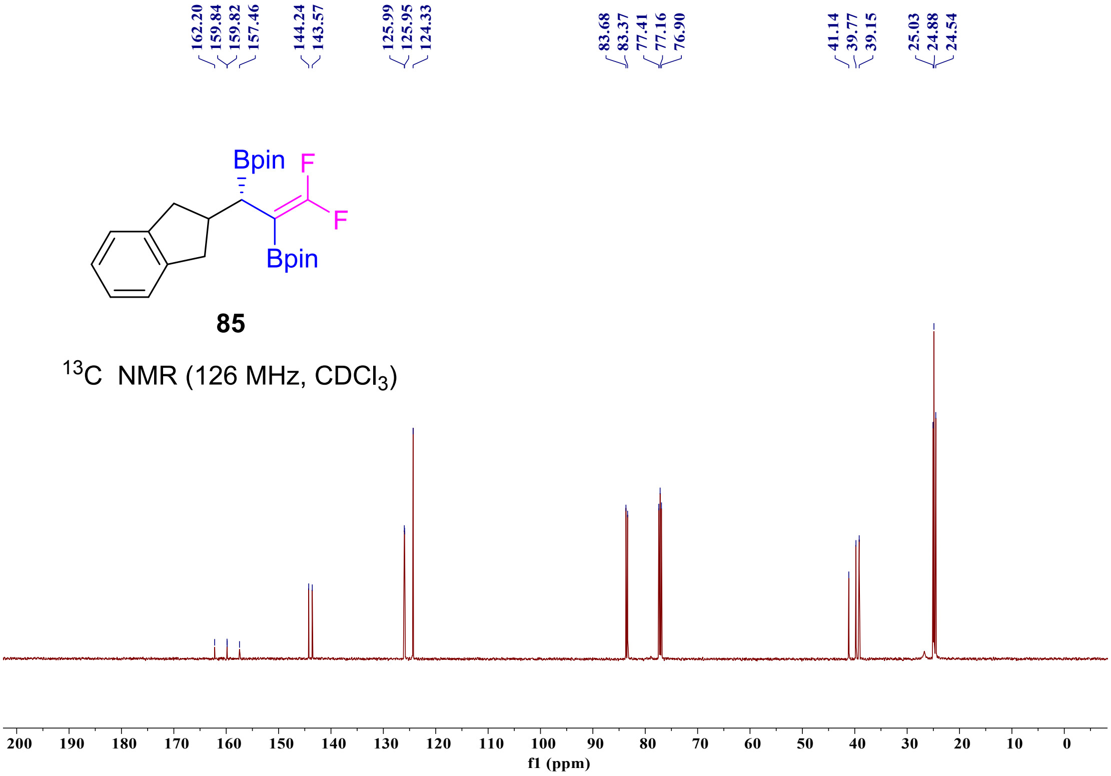

(S)-2,2'-(1-(2,3-dihydro-1H-inden-2-yl)-3,3-difluoroprop-2-ene-1,2-diyl)bis(4,4,5,5-tetramethyl-1,3,2-dioxaborolane) (85)

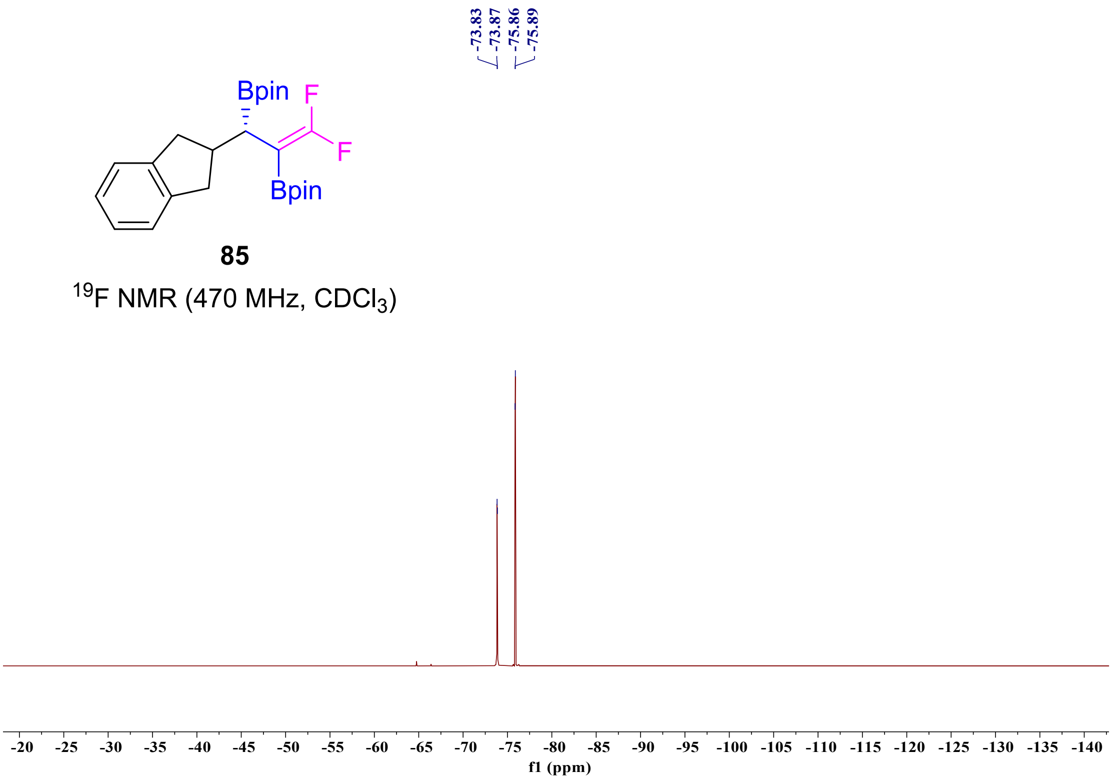

(S)-2,2'-(1-(2,3-dihydro-1H-inden-2-yl)-3,3-difluoroprop-2-ene-1,2-diyl)bis(4,4,5,5-tetramethyl-1,3,2-dioxaborolane) (85)

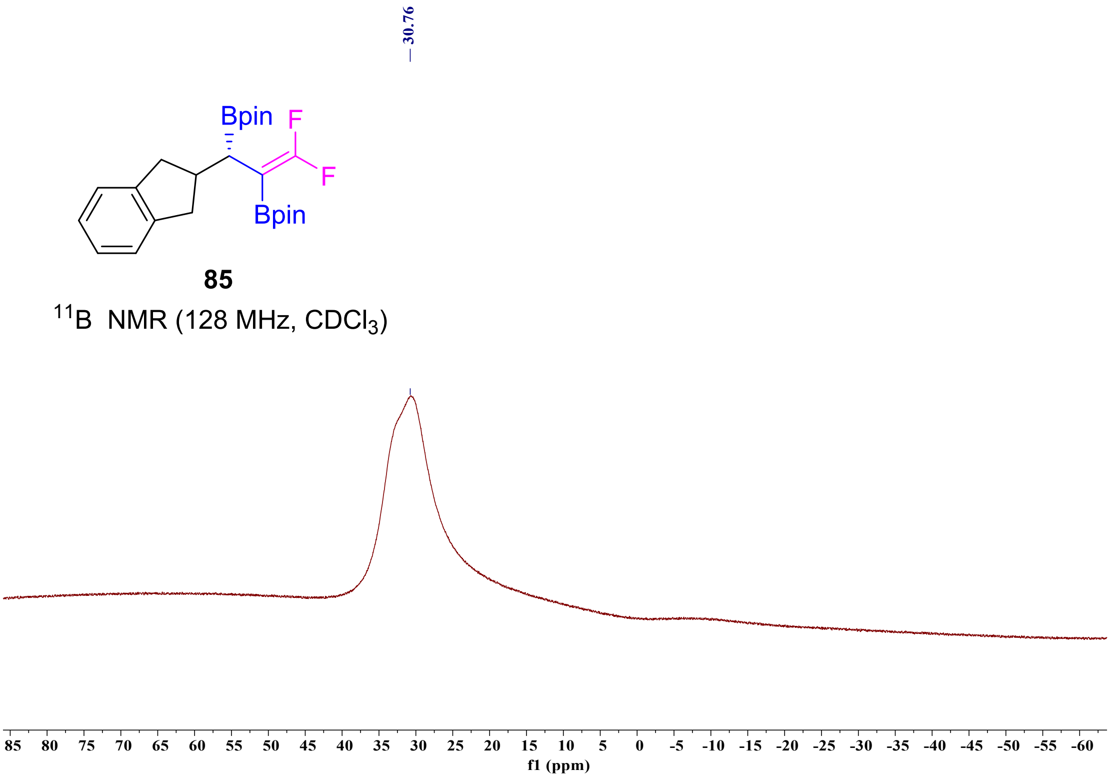

(S)-2,2'-(5-cyclopentyl-1,1-difluoropent-1-ene-2,3-diyl)bis(4,4,5,5-tetramethyl-1,3,2-dioxaborolane) (86)

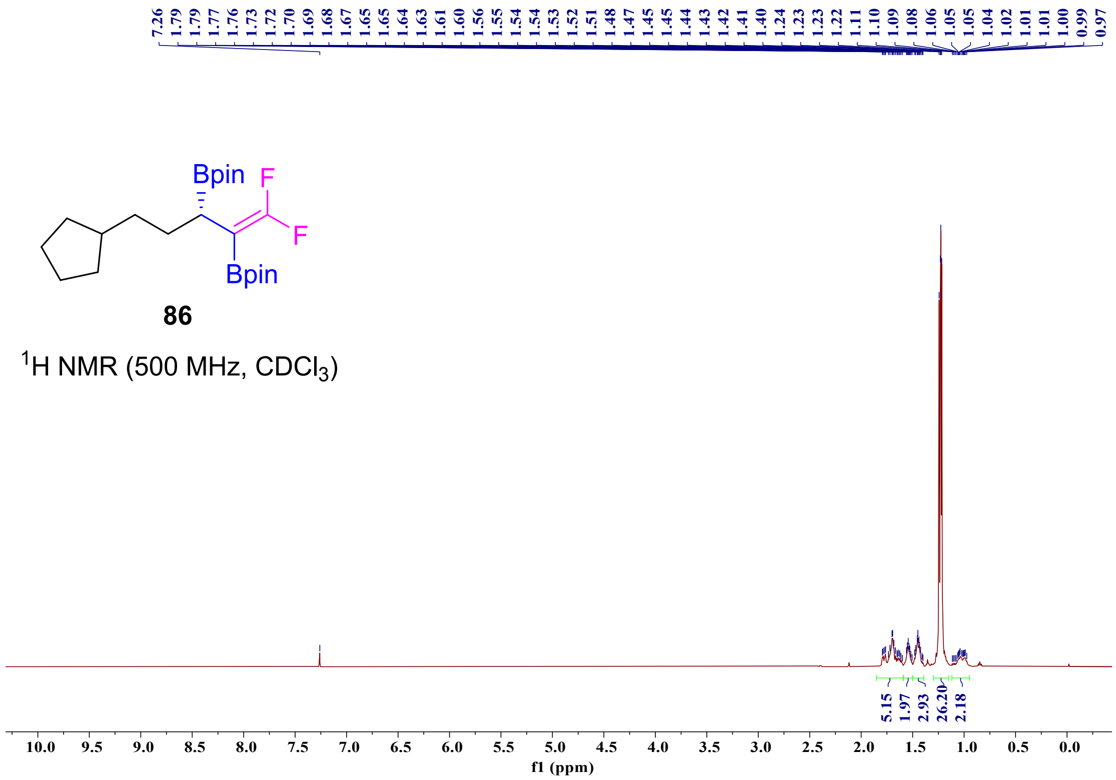

(S)-2,2'-(5-cyclopentyl-1,1-difluoropent-1-ene-2,3-diyl)bis(4,4,5,5-tetramethyl-1,3,2-dioxaborolane) (86)

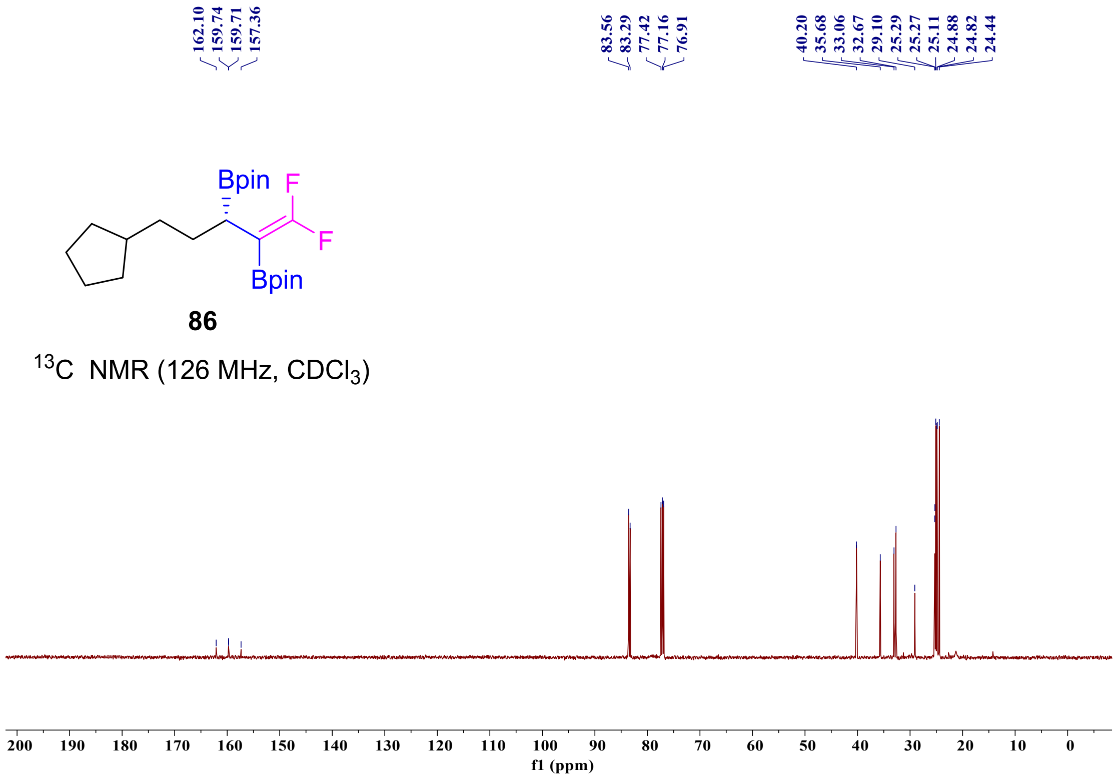

(S)-2,2'-(5-cyclopentyl-1,1-difluoropent-1-ene-2,3-diyl)bis(4,4,5,5-tetramethyl-1,3,2-dioxaborolane) (86)

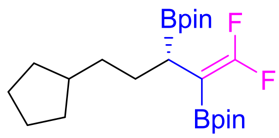

86

19F NMR (470 MHz, CDCl3)

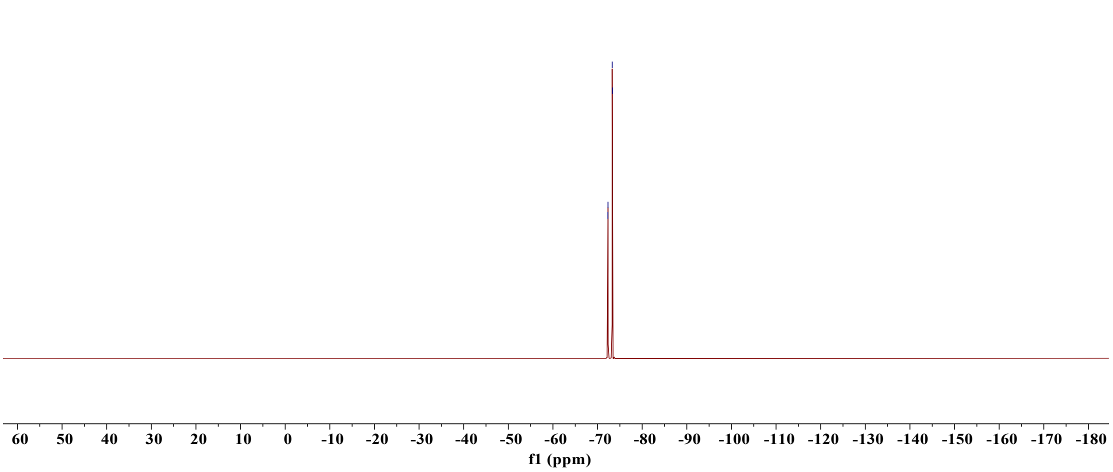

(S)-2,2'-(5-cyclopentyl-1,1-difluoropent-1-ene-2,3-diyl)bis(4,4,5,5-tetramethyl-1,3,2-dioxaborolane) (86)

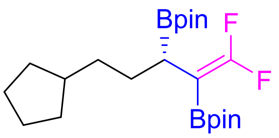

86

^{11}B NMR (128 MHz, $CDCl_3$)

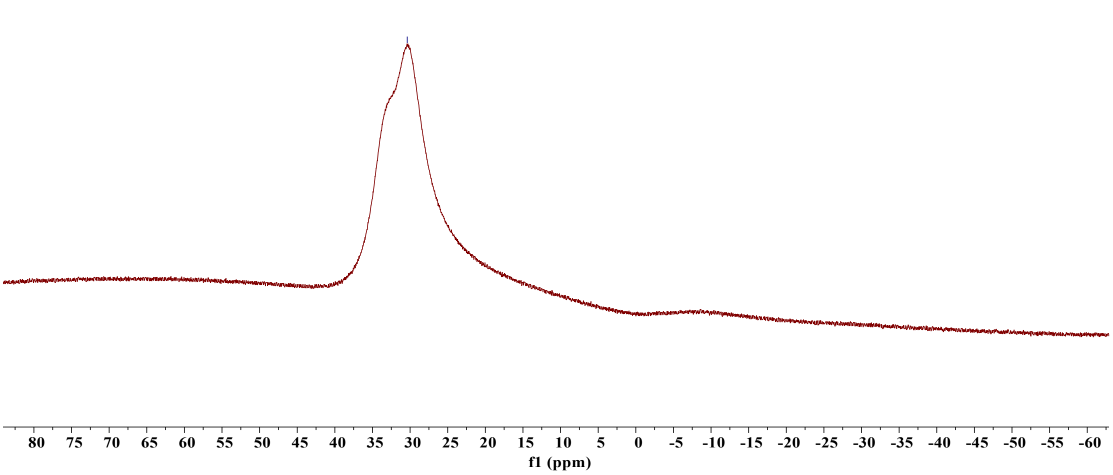

(S)-2,2'-(5-cyclohexyl-1,1-difluoropent-1-ene-2,3-diyl)bis(4,4,5,5-tetramethyl-1,3,2-dioxaborolane) (87)

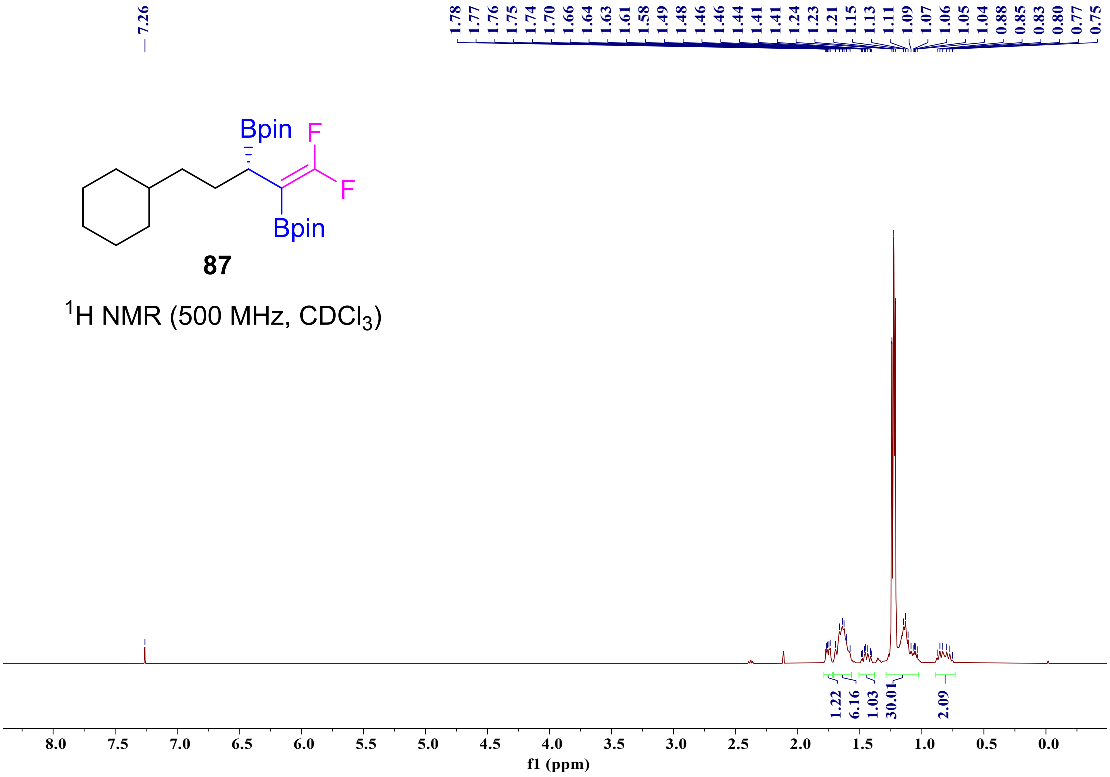

(S)-2,2'-(5-cyclohexyl-1,1-difluoropent-1-ene-2,3-diyl)bis(4,4,5,5-tetramethyl-1,3,2-dioxaborolane) (87)

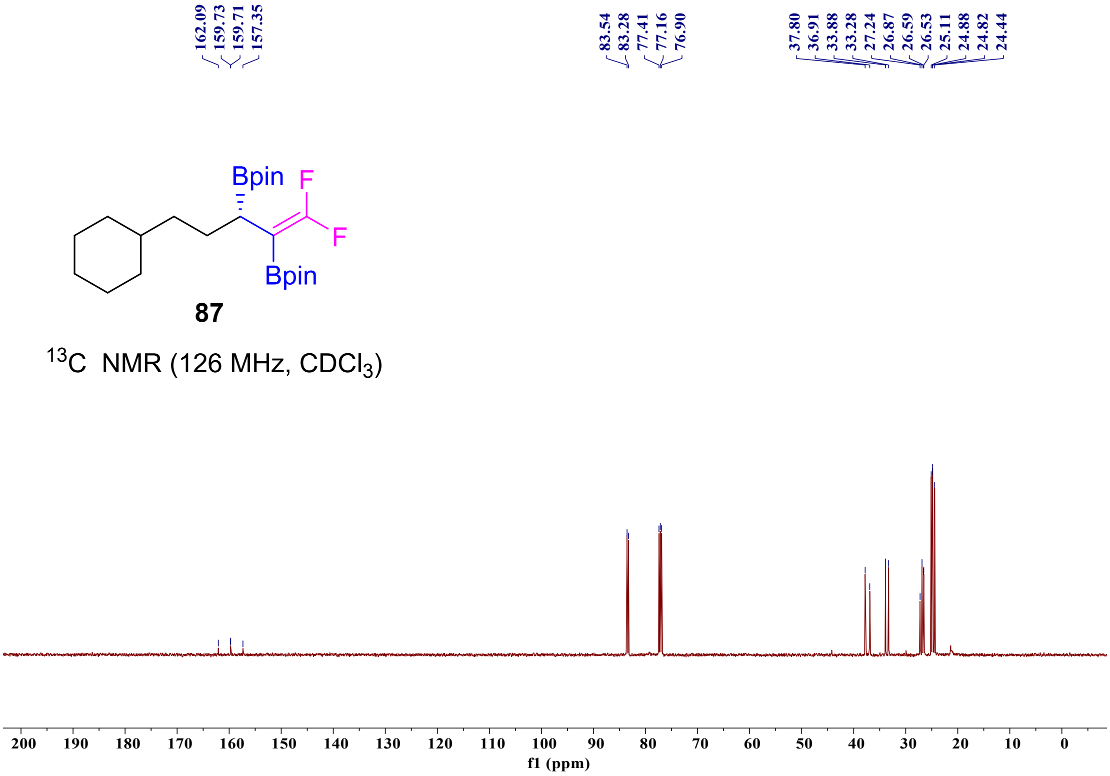

(S)-2,2'-(5-cyclohexyl-1,1-difluoropent-1-ene-2,3-diyl)bis(4,4,5,5-tetramethyl-1,3,2-dioxaborolane) (87)

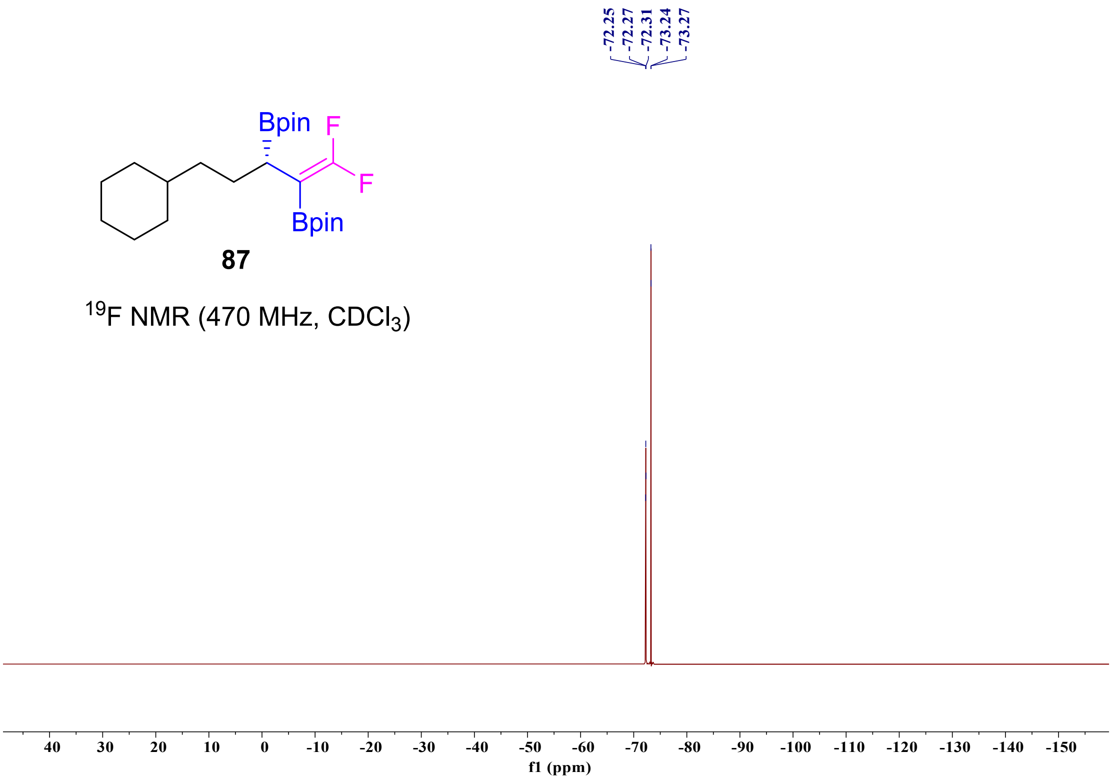

(S)-2,2'-(5-cyclohexyl-1,1-difluoropent-1-ene-2,3-diyl)bis(4,4,5,5-tetramethyl-1,3,2-dioxaborolane) (87)

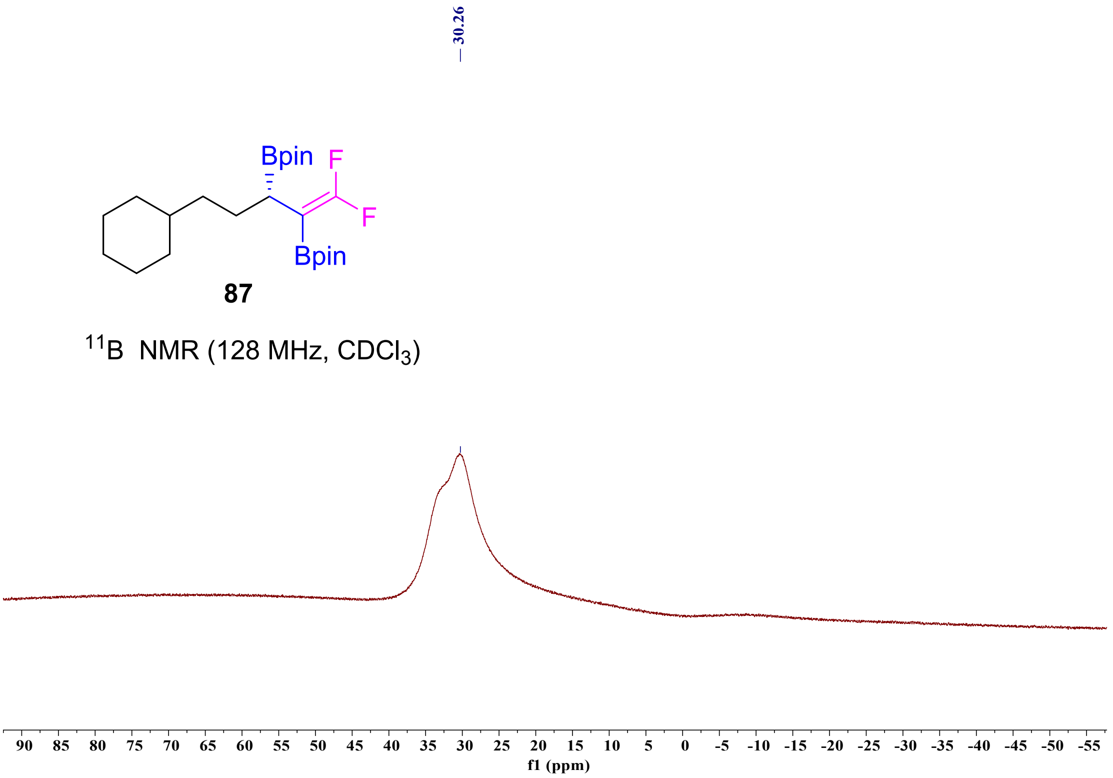

2,2'-((S)-4-((3S,5S,7S)-adamantan-1-yl)-1,1-difluorobut-1-ene-2,3-diyl)bis(4,4,5,5-tetramethyl-1,3,2-dioxaborolane) (88)

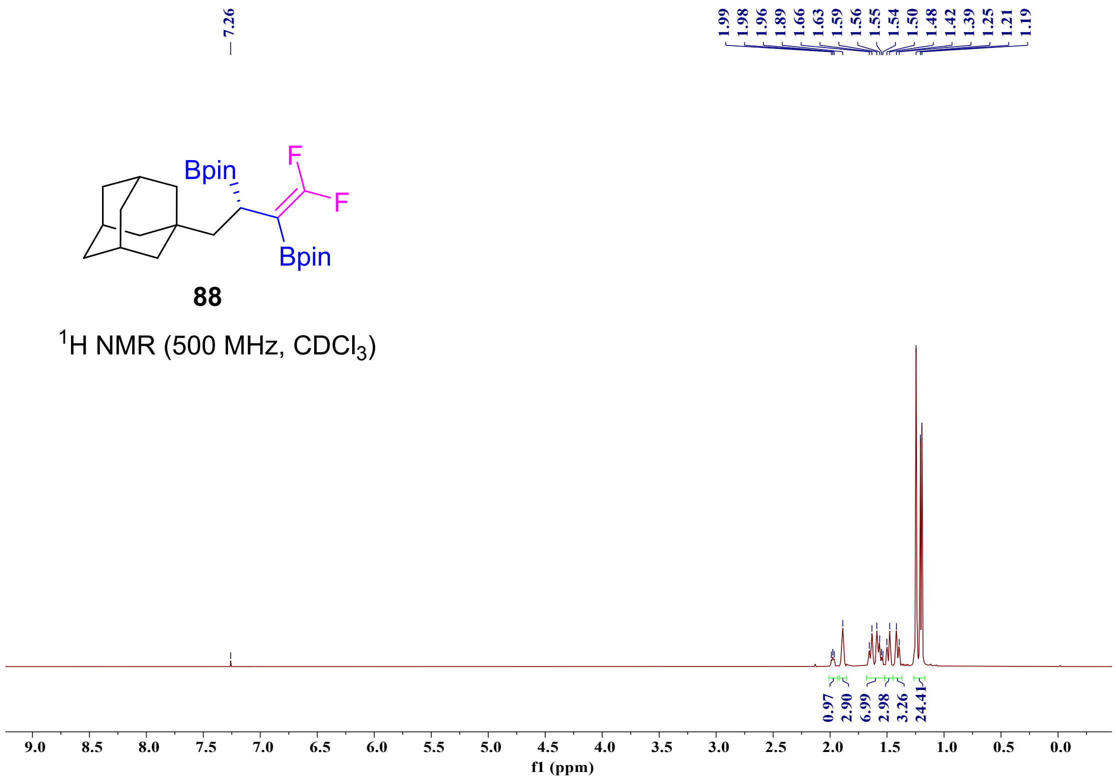

2,2'-((S)-4-((3S,5S,7S)-adamantan-1-yl)-1,1-difluorobut-1-ene-2,3-diyl)bis(4,4,5,5-tetramethyl-1,3,2-dioxaborolane) (88)

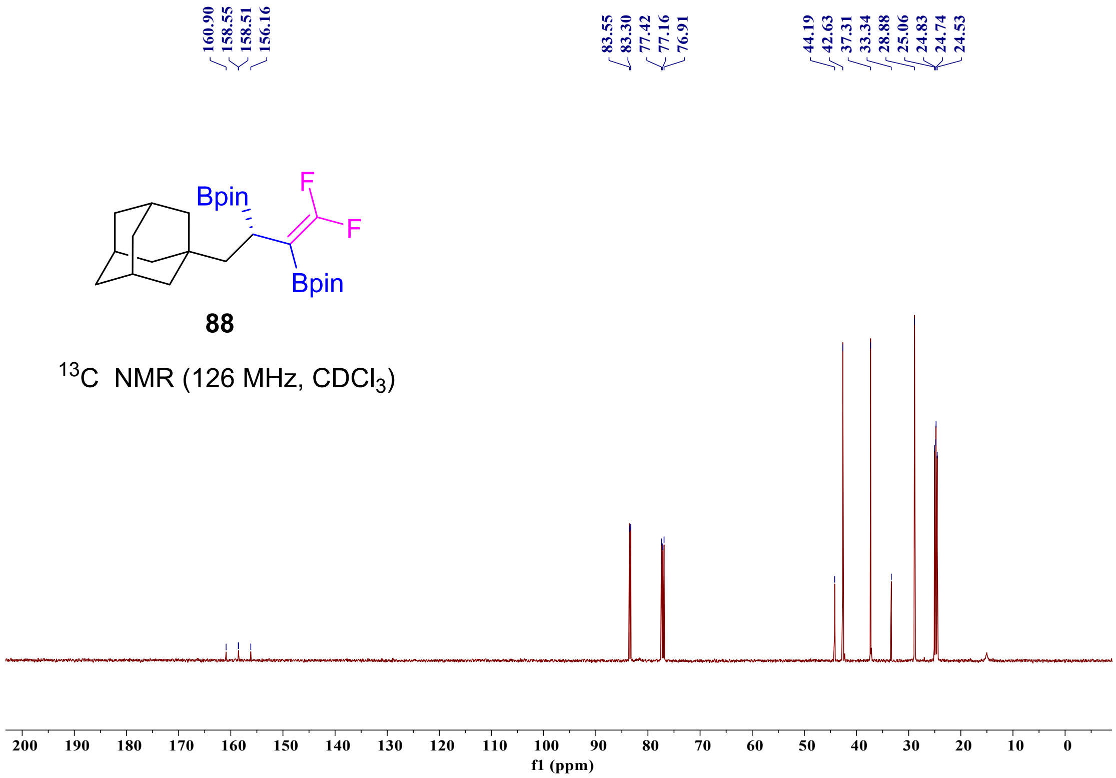

2,2'-((S)-4-((3S,5S,7S)-adamantan-1-yl)-1,1-difluorobut-1-ene-2,3-diyl)bis(4,4,5,5-tetramethyl-1,3,2-dioxaborolane) (88)

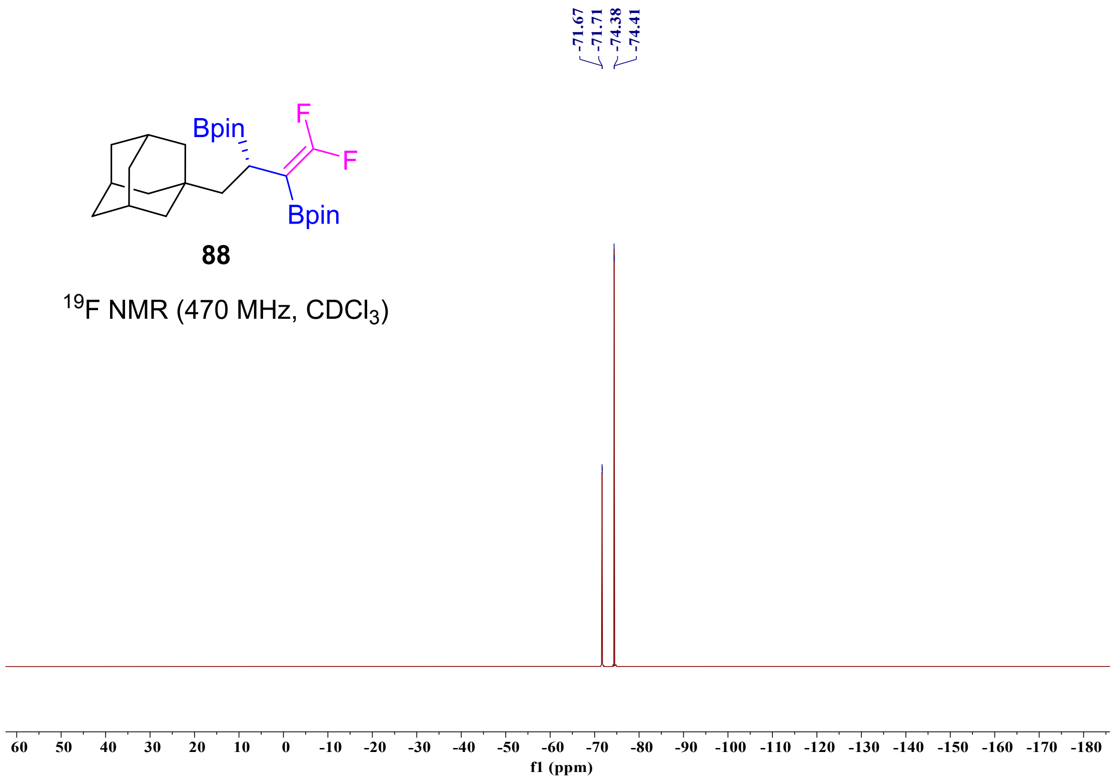

2,2'-((S)-4-((3S,5S,7S)-adamantan-1-yl)-1,1-difluorobut-1-ene-2,3-diyl)bis(4,4,5,5-tetramethyl-1,3,2-dioxaborolane) (88)

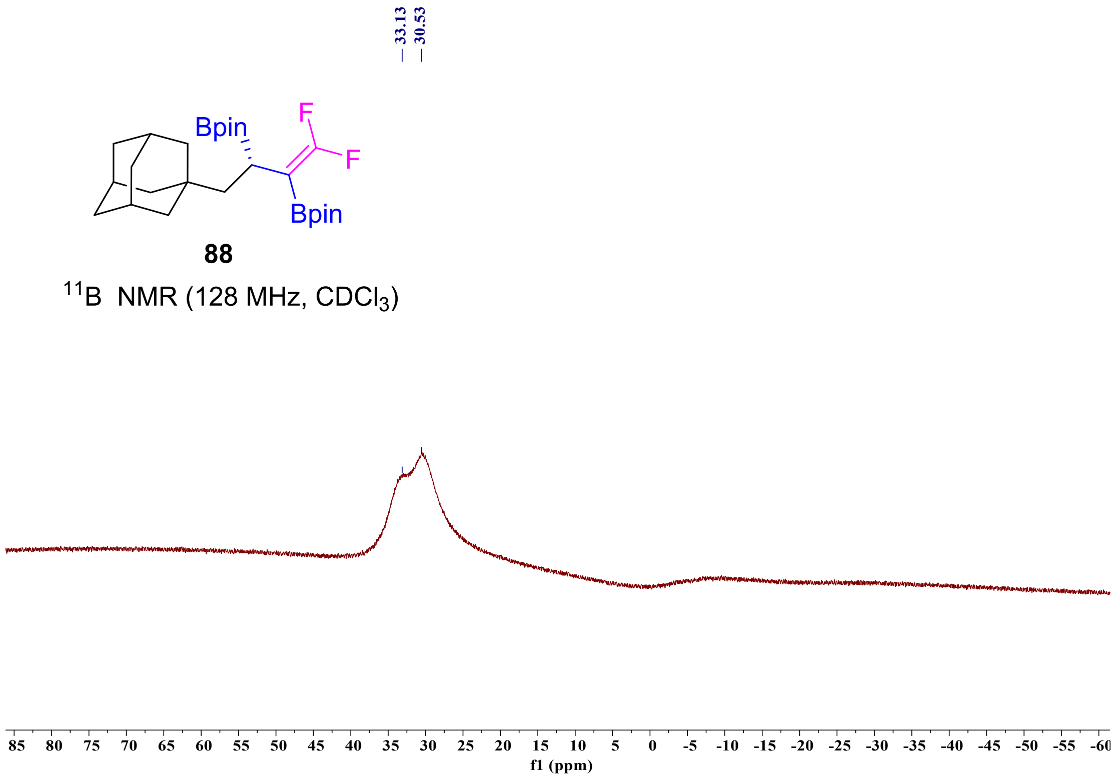

(S)2,2'-(3,3-difluoro-1-(4-(methylthio)phenyl)prop-2-ene-1,2-diyl)bis(4,4,5,5-tetramethyl-1,3,2-dioxaborolane) (89)

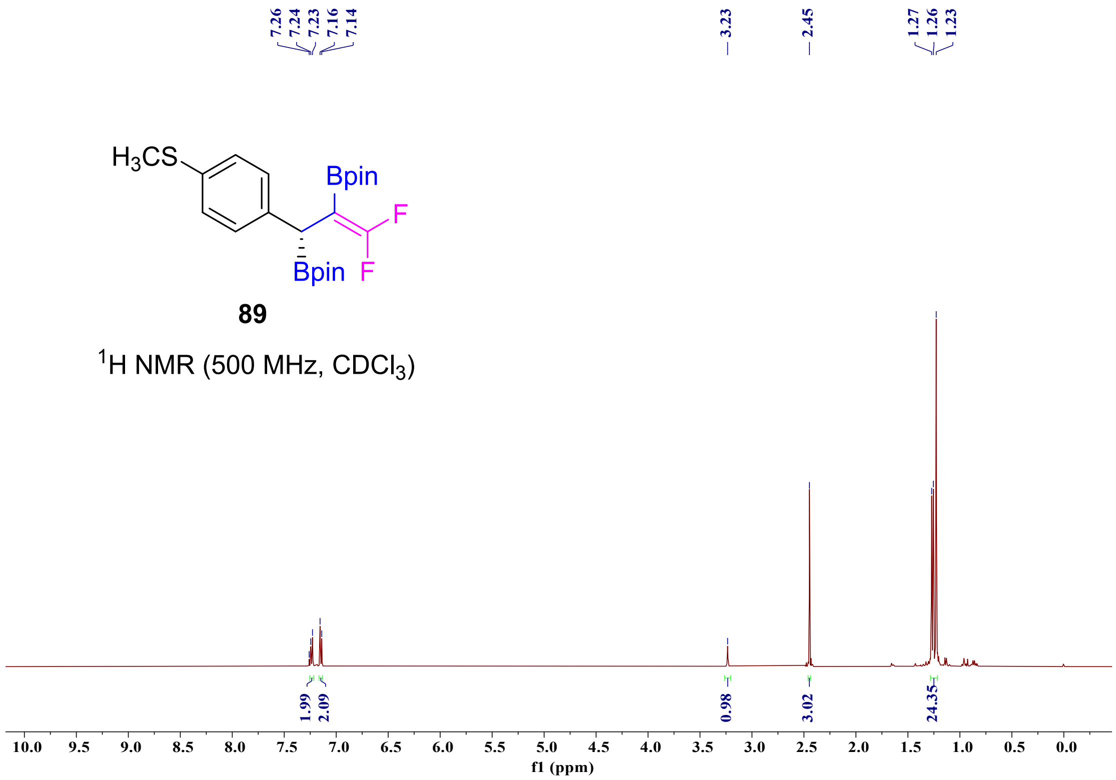

(S)2,2'-(3,3-difluoro-1-(4-(methylthio)phenyl)prop-2-ene-1,2-diyl)bis(4,4,5,5-tetramethyl-1,3,2-dioxaborolane) (89)

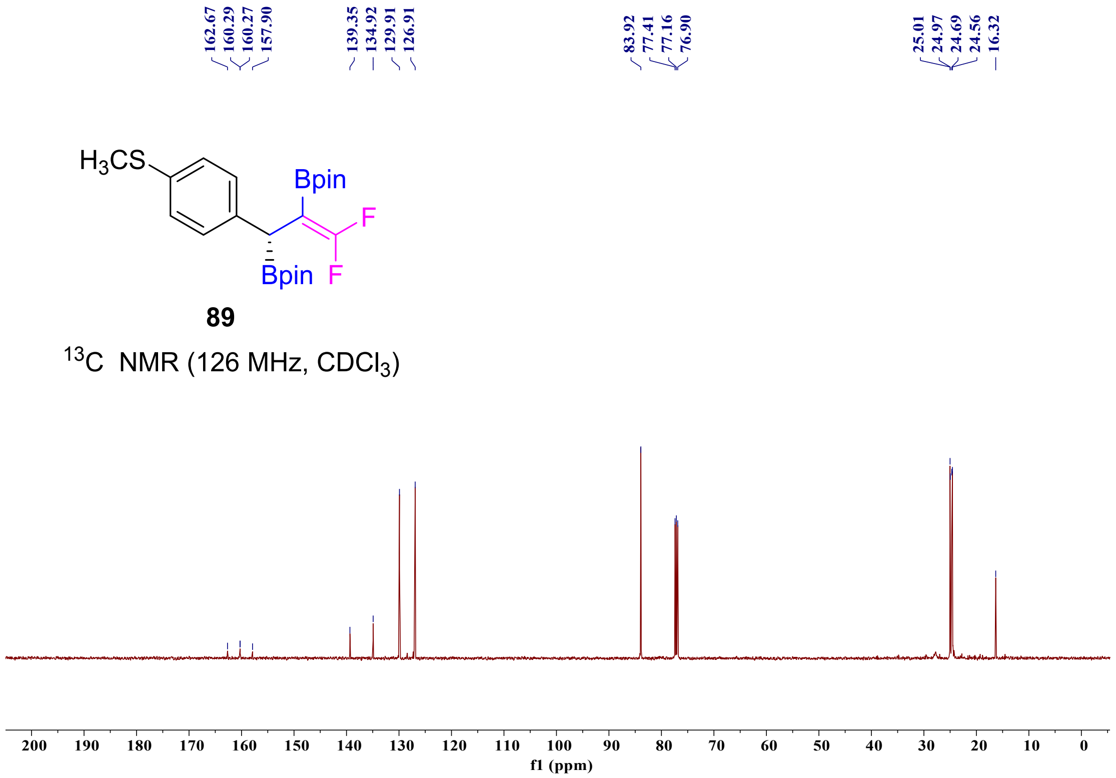

(S)2,2'-(3,3-difluoro-1-(4-(methylthio)phenyl)prop-2-ene-1,2-diyl)bis(4,4,5,5-tetramethyl-1,3,2-dioxaborolane) (89)

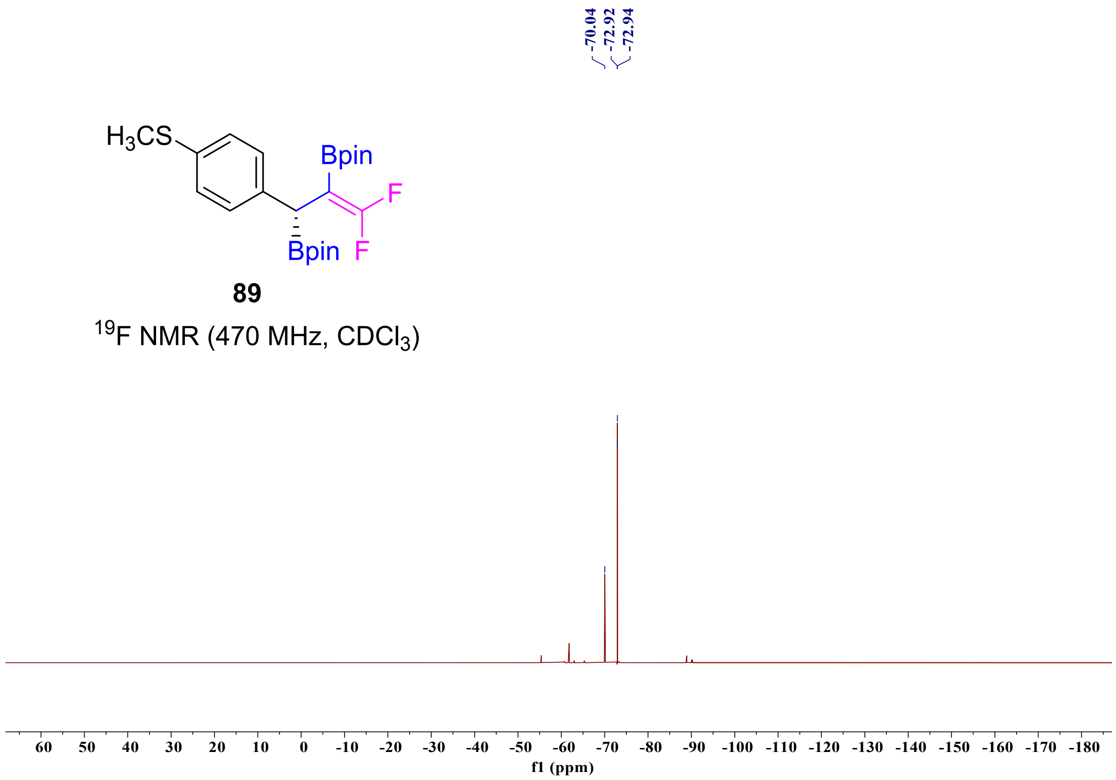

(S)2,2'-(3,3-difluoro-1-(4-(methylthio)phenyl)prop-2-ene-1,2-diyl)bis(4,4,5,5-tetramethyl-1,3,2-dioxaborolane) (89)

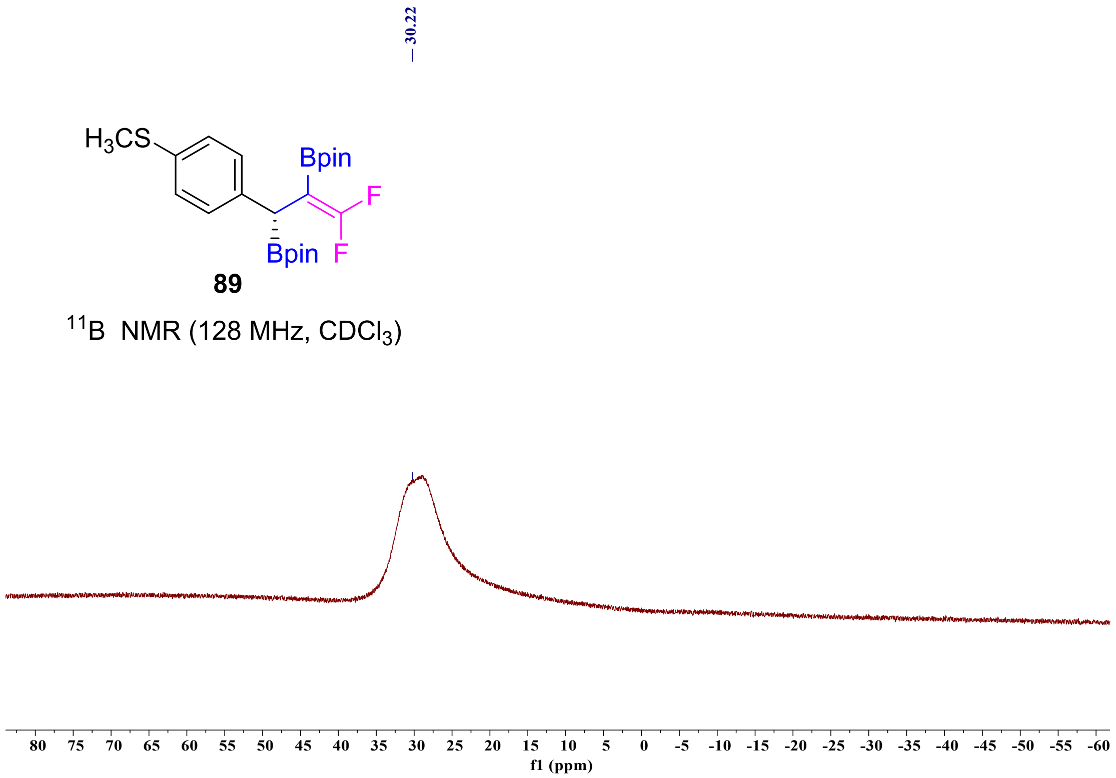

(S)-2,2'-(1-(4-(tert-butyl)phenyl)-3,3-difluoroprop-2-ene-1,2-diyl)bis(4,4,5,5-tetramethyl-1,3,2-dioxaborolane) (90)

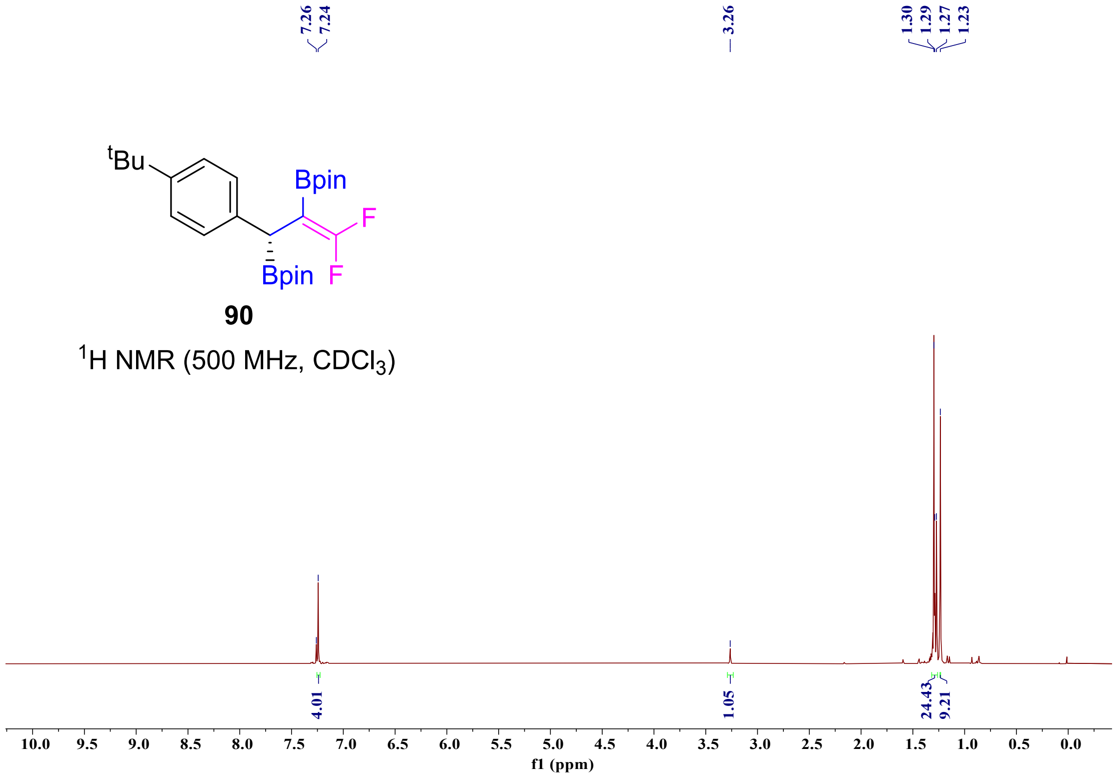

(S)-2,2'-(1-(4-(tert-butyl)phenyl)-3,3-difluoroprop-2-ene-1,2-diyl)bis(4,4,5,5-tetramethyl-1,3,2-dioxaborolane) (90)

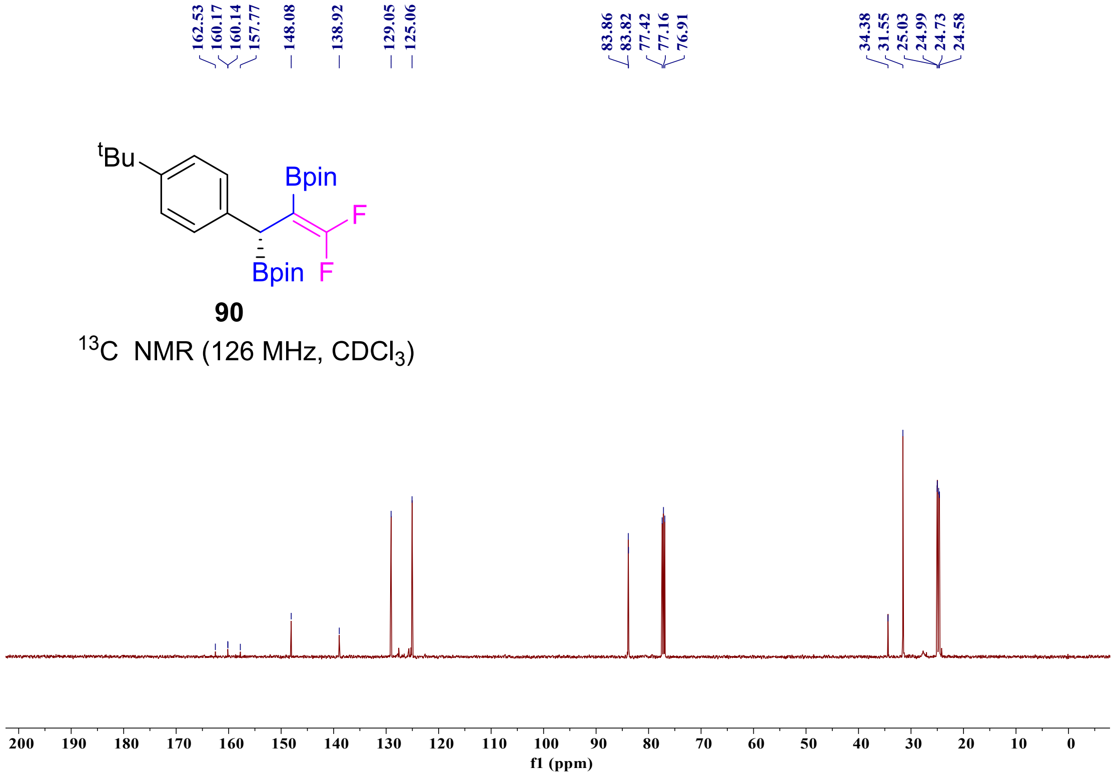

(S)-2,2'-(1-(4-(tert-butyl)phenyl)-3,3-difluoroprop-2-ene-1,2-diyl)bis(4,4,5,5-tetramethyl-1,3,2-dioxaborolane) (90)

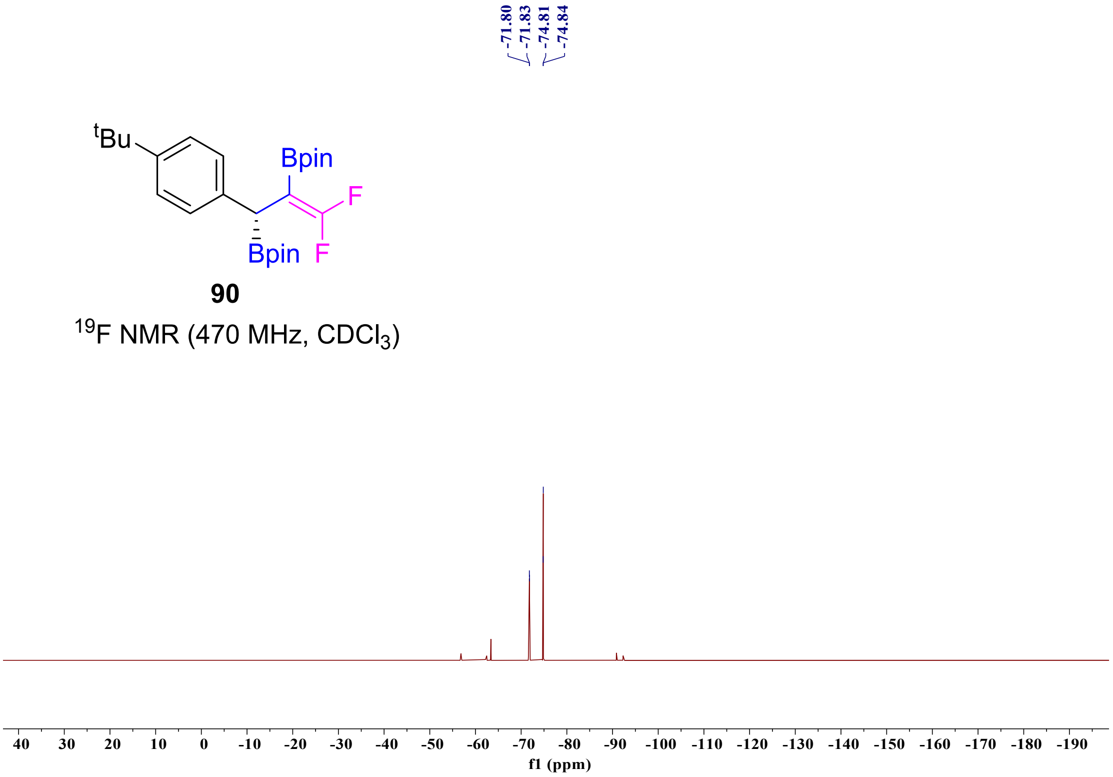

(S)-2,2'-(1-(4-(tert-butyl)phenyl)-3,3-difluoroprop-2-ene-1,2-diyl)bis(4,4,5,5-tetramethyl-1,3,2-dioxaborolane) (90)

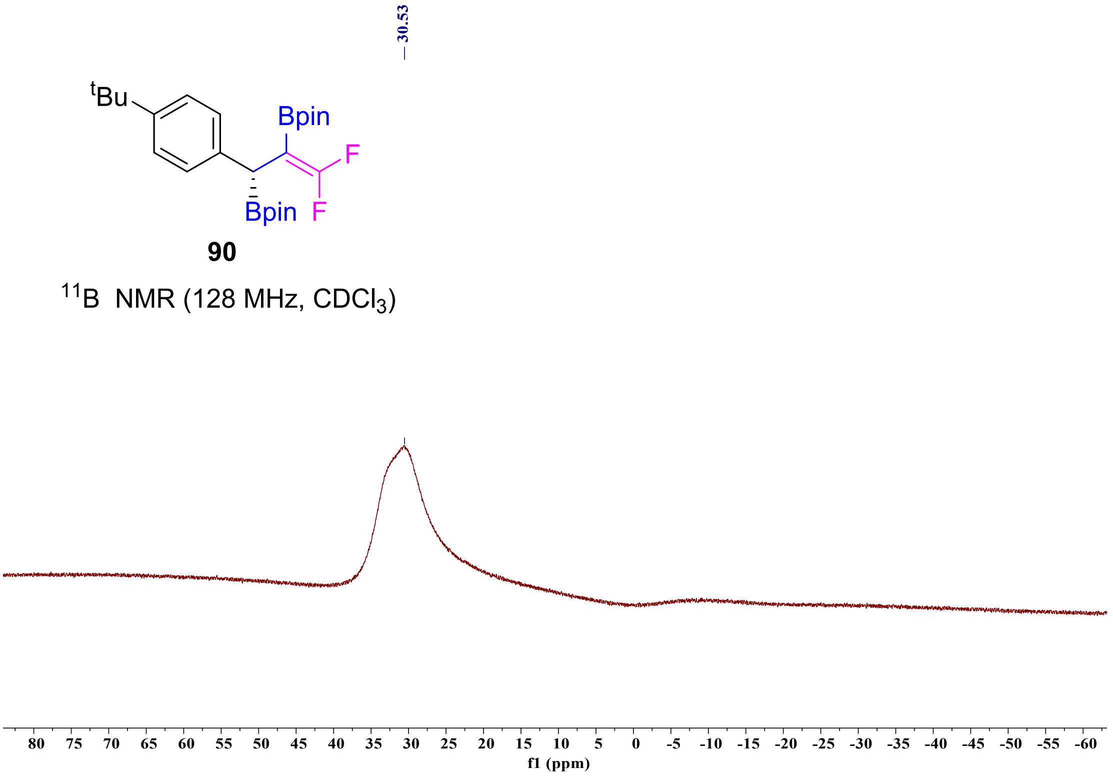

(S)-2,2'-(3,3-difluoro-1-(4-methoxyphenyl)prop-2-ene-1,2-diyl)bis(4,4,5,5-tetramethyl-1,3,2-dioxaborolane) (91)

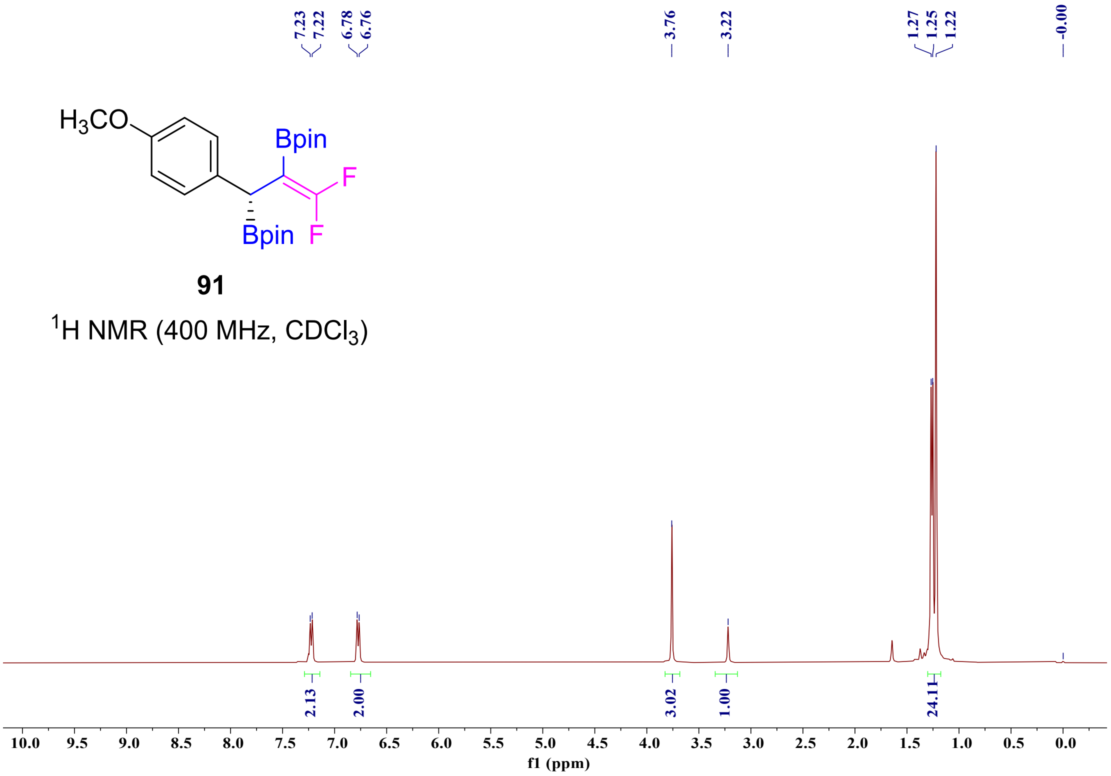

(S)-2,2'-(3,3-difluoro-1-(4-methoxyphenyl)prop-2-ene-1,2-diyl)bis(4,4,5,5-tetramethyl-1,3,2-dioxaborolane) (91)

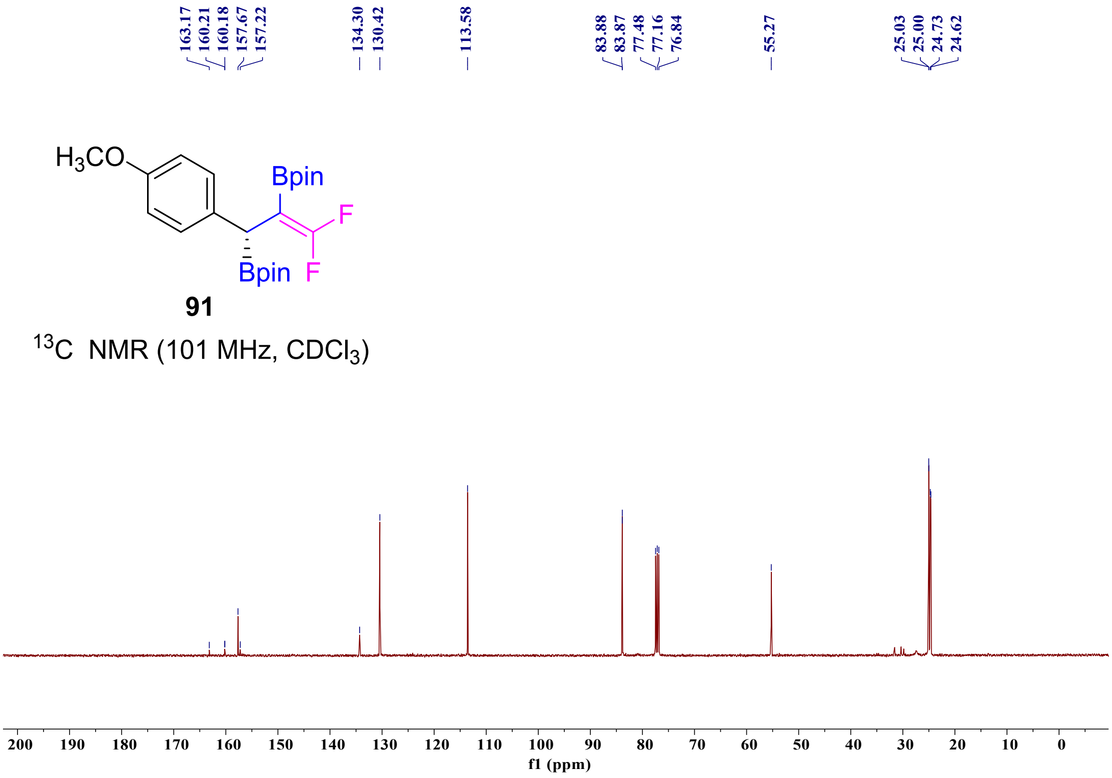

(S)-2,2'-(3,3-difluoro-1-(4-methoxyphenyl)prop-2-ene-1,2-diyl)bis(4,4,5,5-tetramethyl-1,3,2-dioxaborolane) (91)

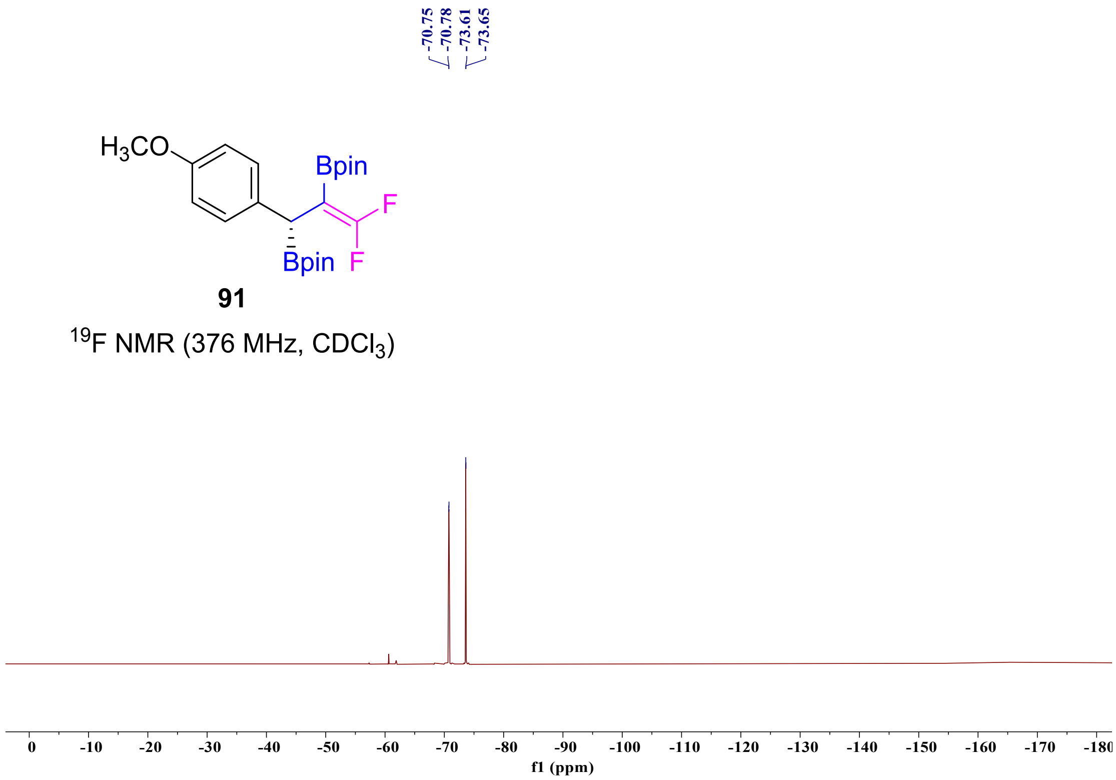

(S)-2,2'-(3,3-difluoro-1-(4-methoxyphenyl)prop-2-ene-1,2-diyl)bis(4,4,5,5-tetramethyl-1,3,2-dioxaborolane) (91)

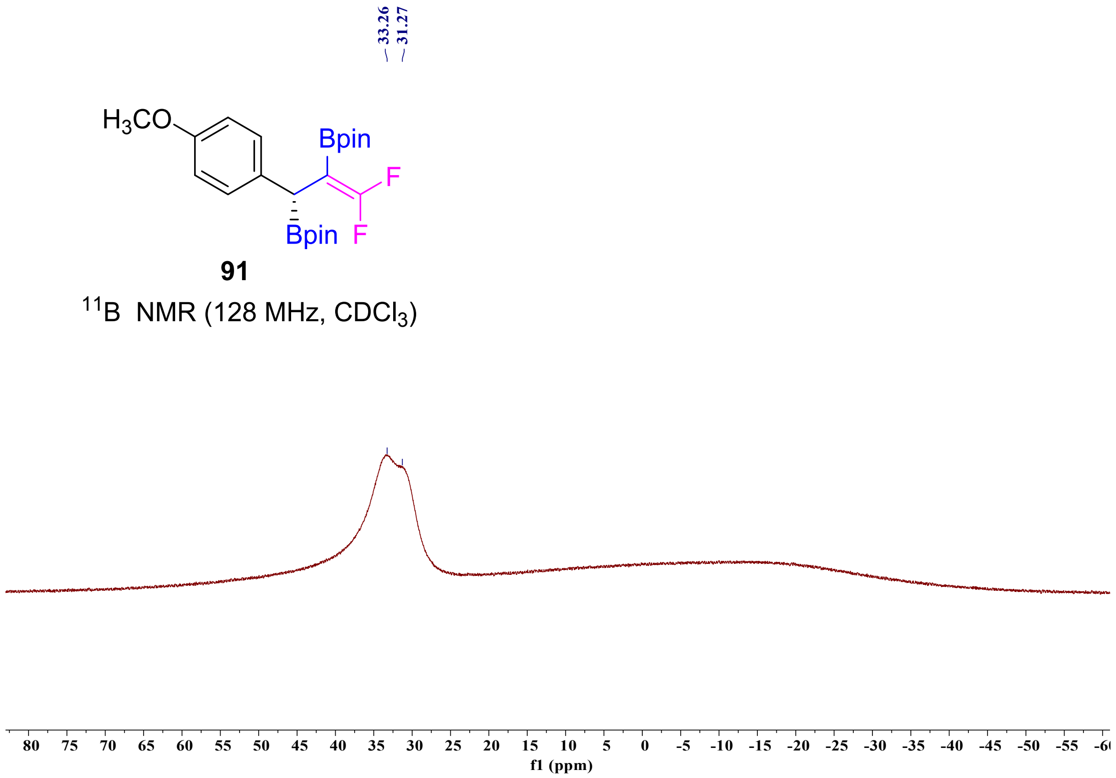

(S)-2,2'-(3,3-difluoro-1-(4-(trifluoromethoxy)phenyl)prop-2-ene-1,2-diyl)bis(4,4,5,5-tetramethyl-1,3,2-dioxaborolane) (92)

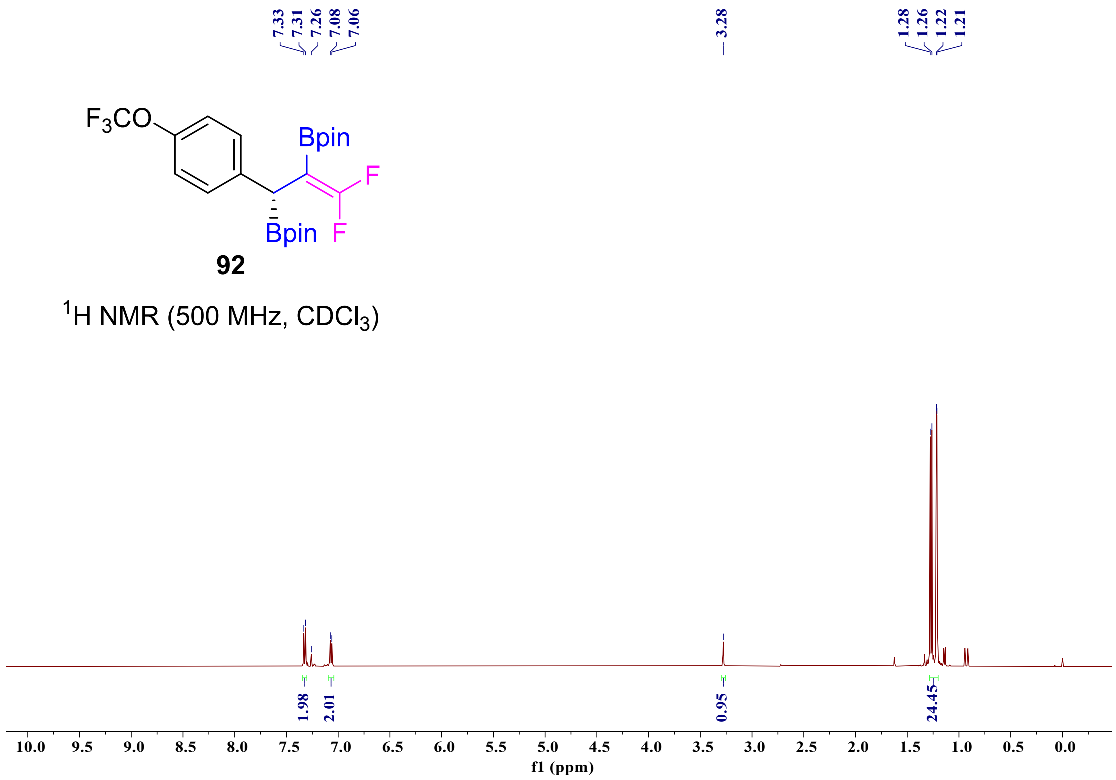

(S)-2,2'-(3,3-difluoro-1-(4-(trifluoromethoxy)phenyl)prop-2-ene-1,2-diyl)bis(4,4,5,5-tetramethyl-1,3,2-dioxaborolane) (92)

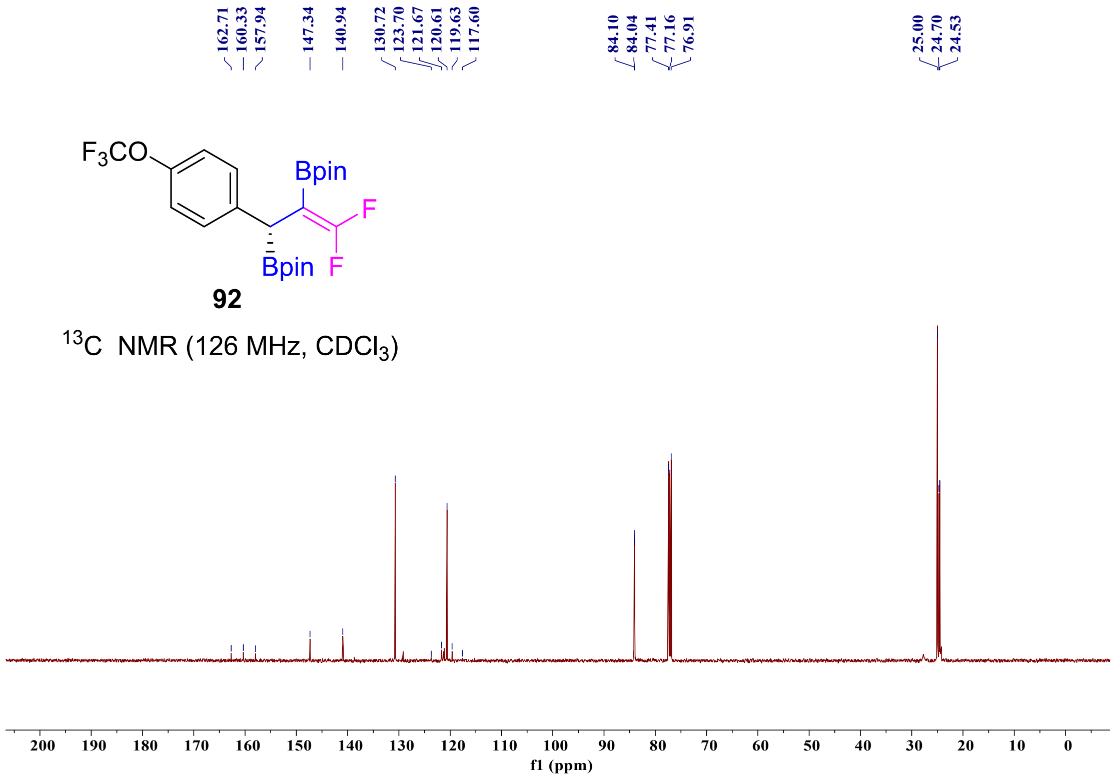

(S)-2,2'-(3,3-difluoro-1-(4-(trifluoromethoxy)phenyl)prop-2-ene-1,2-diyl)bis(4,4,5,5-tetramethyl-1,3,2-dioxaborolane) (92)

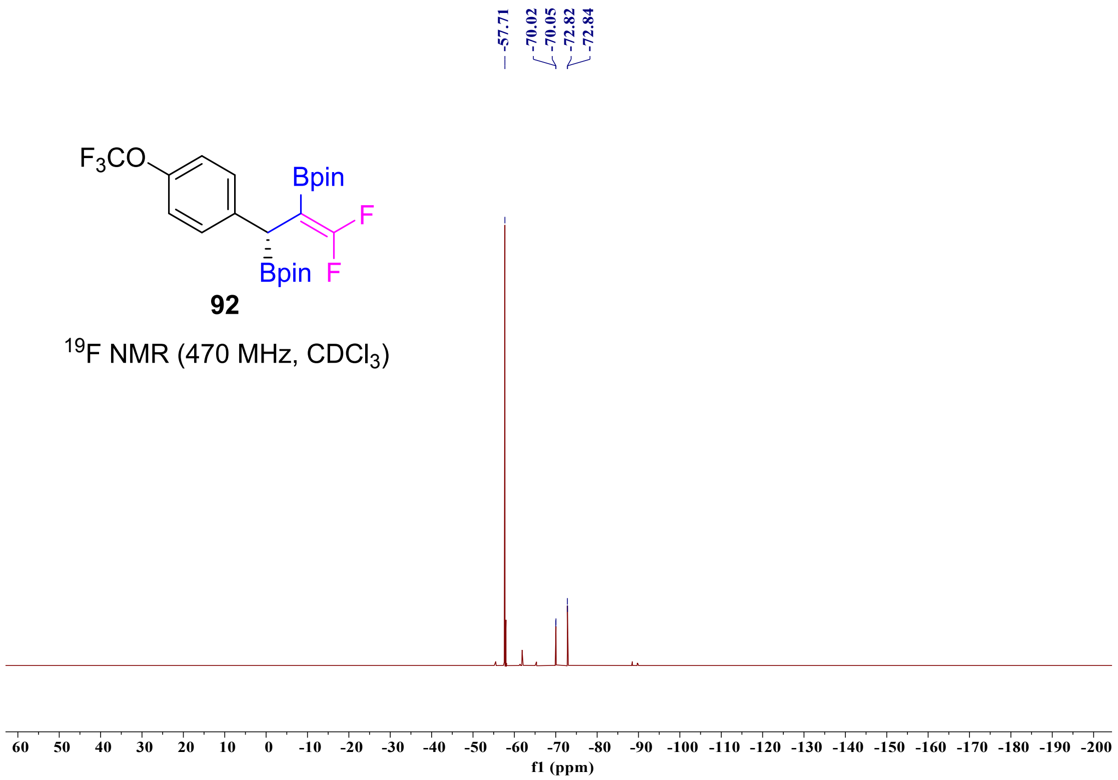

(S)-2,2'-(3,3-difluoro-1-(4-(trifluoromethoxy)phenyl)prop-2-ene-1,2-diyl)bis(4,4,5,5-tetramethyl-1,3,2-dioxaborolane) (92)

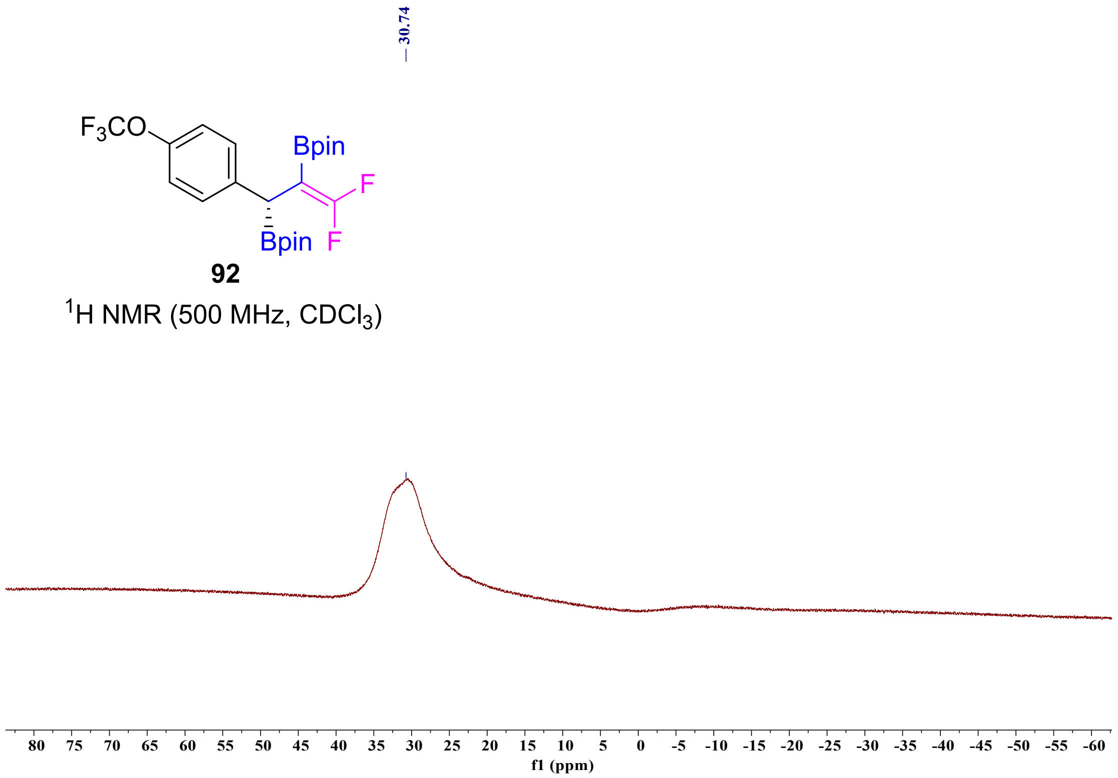

(S)-2,2'-(4-(3-chlorophenyl)-1,1-difluorobut-1-ene-2,3-diyl)bis(4,4,5,5-tetramethyl-1,3,2-dioxaborolane) (93)

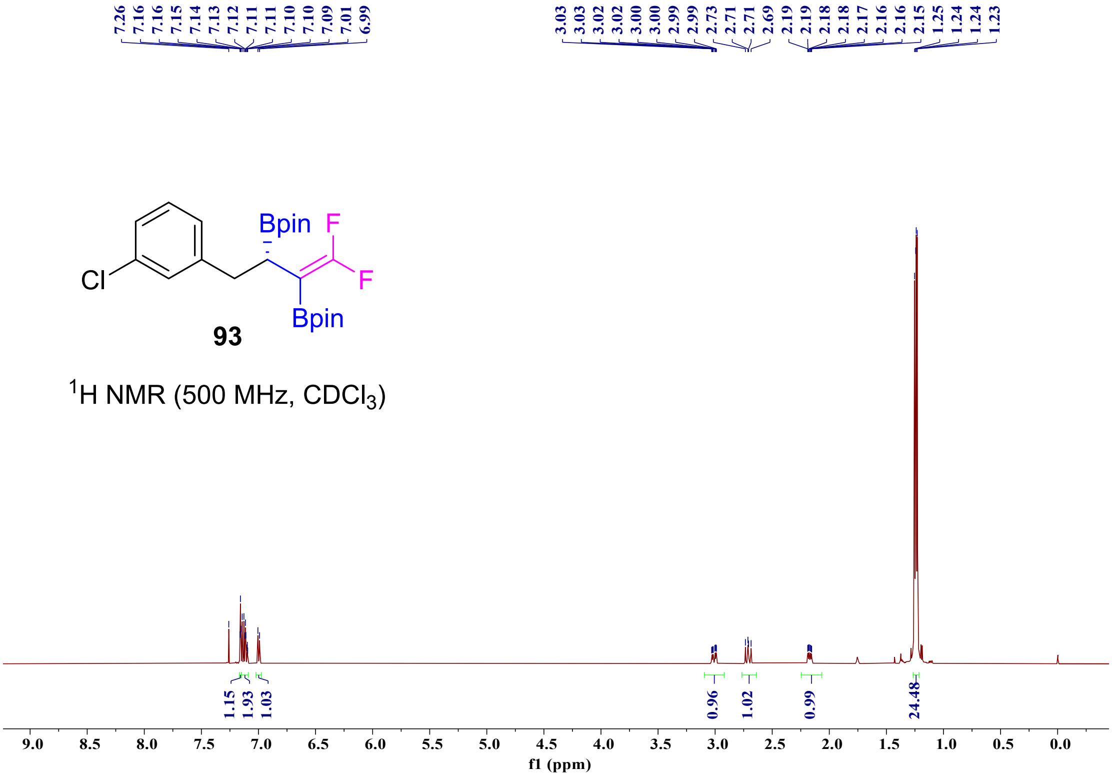

(S)-2,2'-(4-(3-chlorophenyl)-1,1-difluorobut-1-ene-2,3-diyl)bis(4,4,5,5-tetramethyl-1,3,2-dioxaborolane) (93)

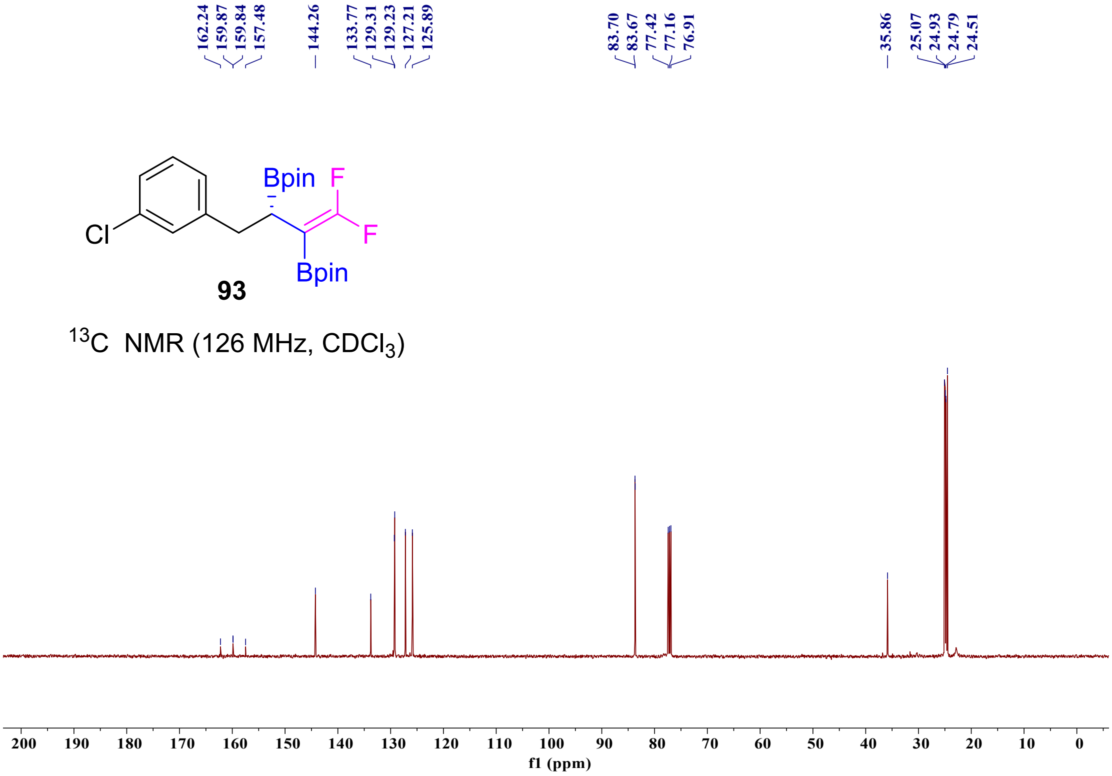

(S)-2,2'-(4-(3-chlorophenyl)-1,1-difluorobut-1-ene-2,3-diyl)bis(4,4,5,5-tetramethyl-1,3,2-dioxaborolane) (93)

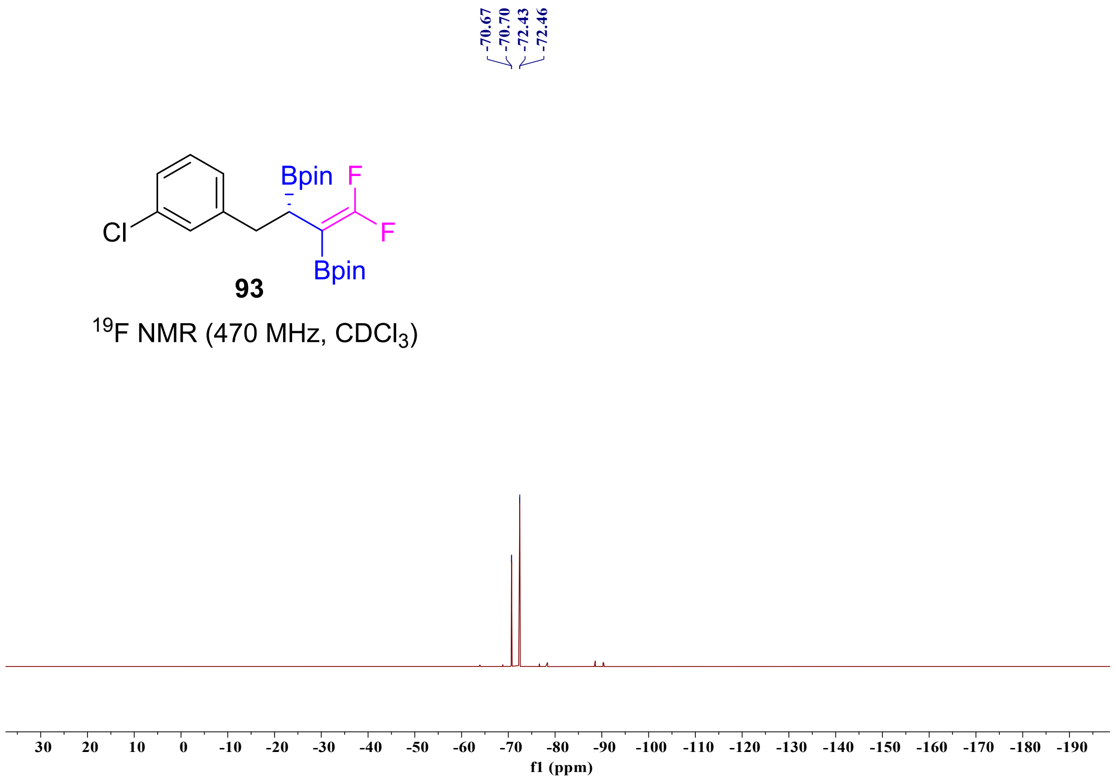

(S)-2,2'-(4-(3-chlorophenyl)-1,1-difluorobut-1-ene-2,3-diyl)bis(4,4,5,5-tetramethyl-1,3,2-dioxaborolane) (93)

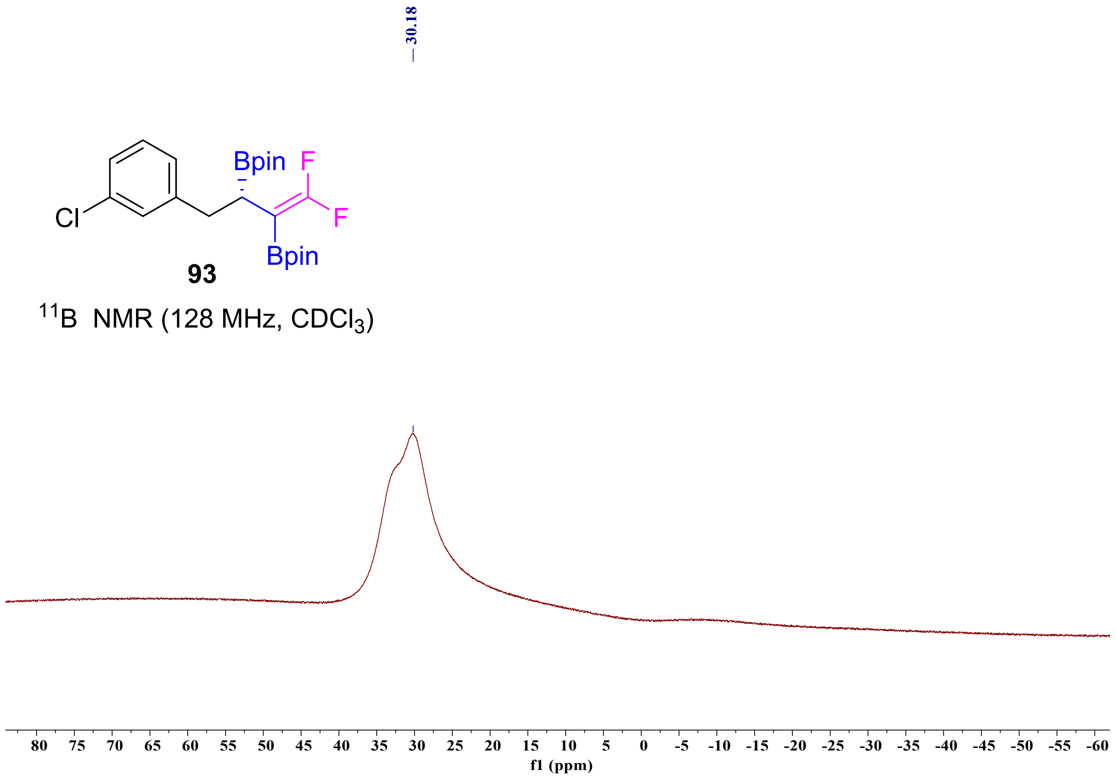

(S)-4-(4,4-difluoro-2,3-bis(4,4,5,5-tetramethyl-1,3,2-dioxaborolan-2-yl)but-3-en-1-yl)benzonitrile (94)

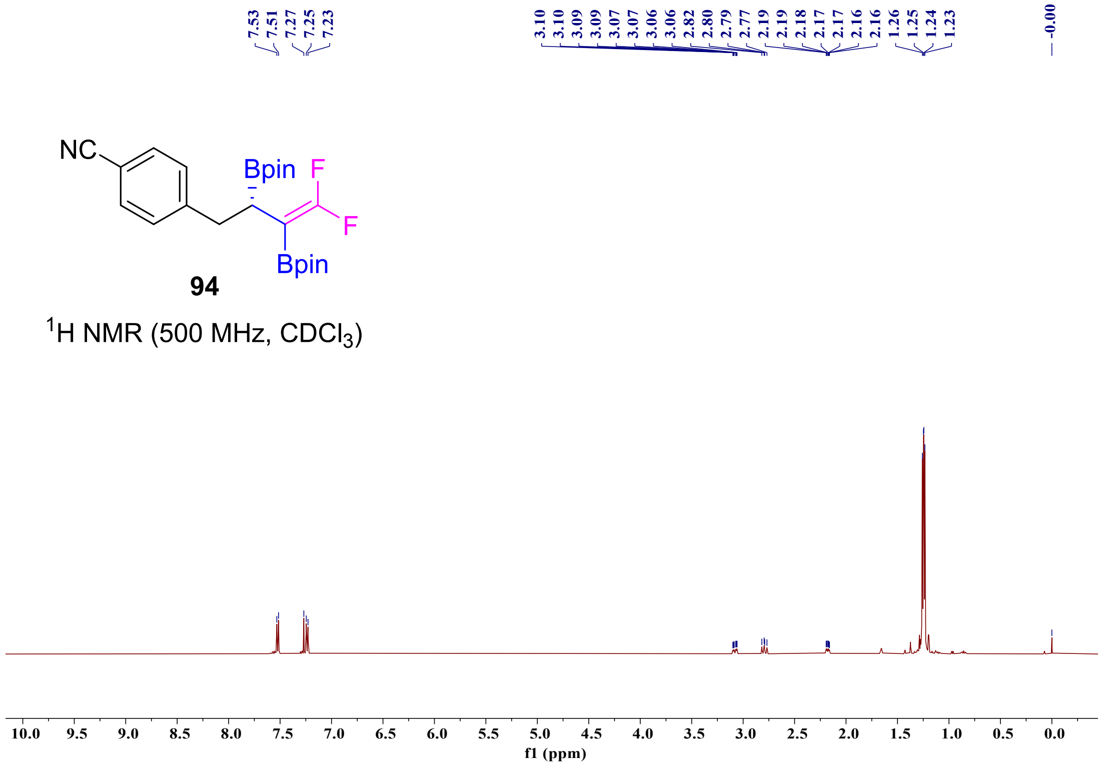

(S)-4-(4,4-difluoro-2,3-bis(4,4,5,5-tetramethyl-1,3,2-dioxaborolan-2-yl)but-3-en-1-yl)benzonitrile (94)

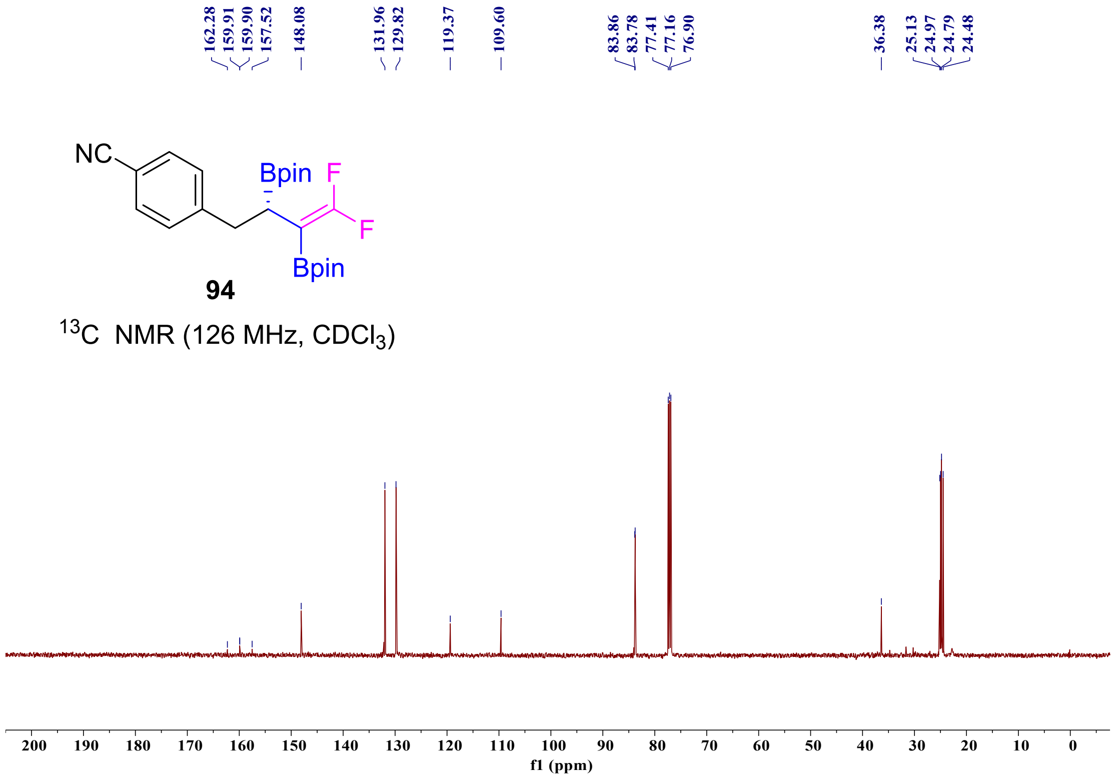

(S)-4-(4,4-difluoro-2,3-bis(4,4,5,5-tetramethyl-1,3,2-dioxaborolan-2-yl)but-3-en-1-yl)benzonitrile (94)

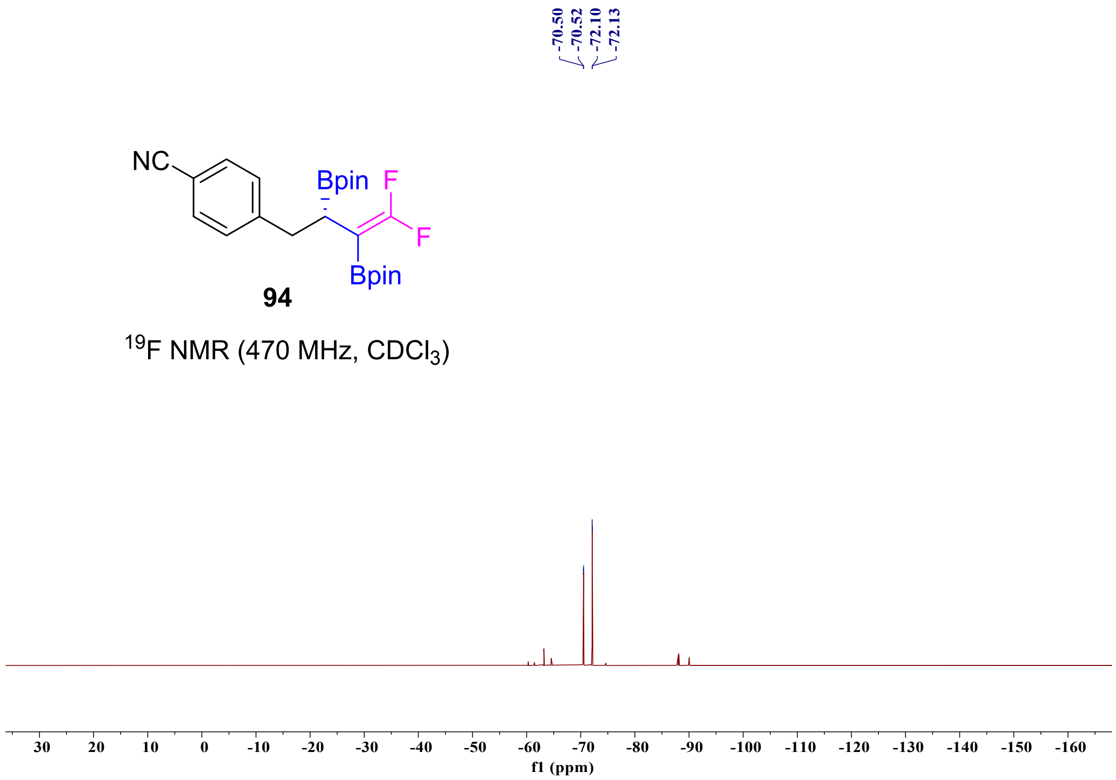

(S)-4-(4,4-difluoro-2,3-bis(4,4,5,5-tetramethyl-1,3,2-dioxaborolan-2-yl)but-3-en-1-yl)benzonitrile (94)

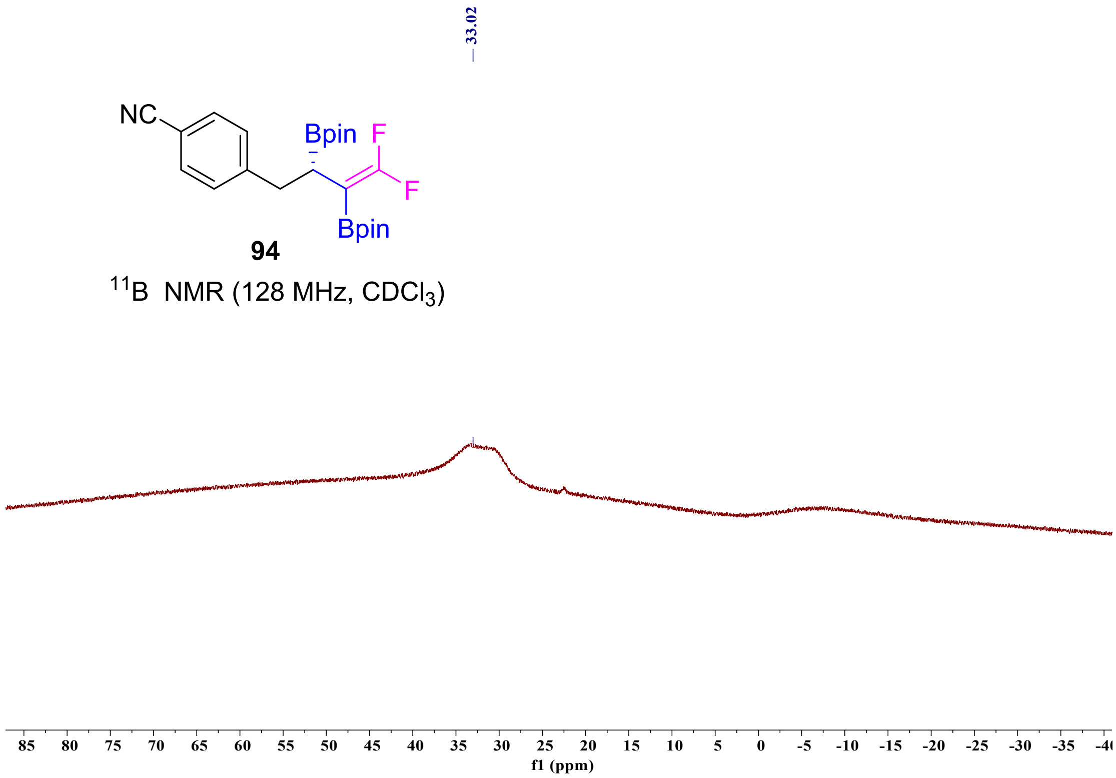

(S)-(4-chlorophenyl)(3-(4,4-difluoro-2,3-bis(4,4,5,5-tetramethyl-1,3,2-dioxaborolan-2-yl)but-3-en-1-yl)-2-methyl-1H-indol-1-yl)methanone (95)

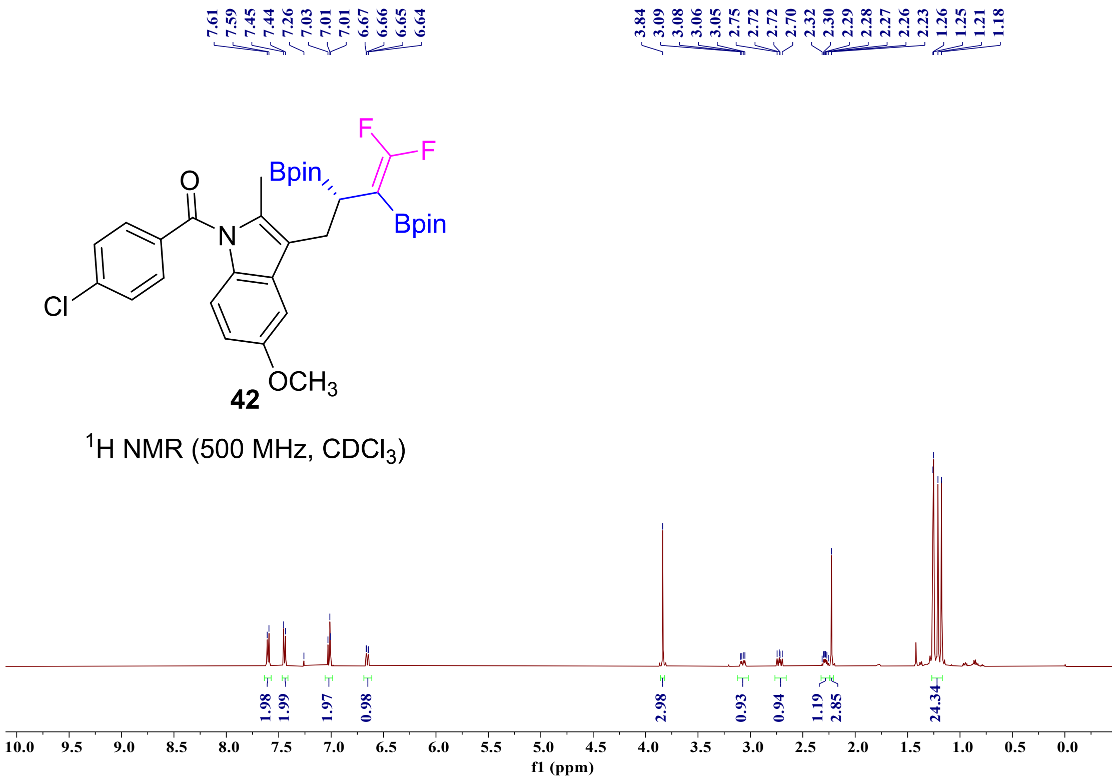

(S)-(4-chlorophenyl)(3-(4,4-difluoro-2,3-bis(4,4,5,5-tetramethyl-1,3,2-dioxaborolan-2-yl)but-3-en-1-yl)-2-methyl-1H-indol-1-yl)methanone (95)

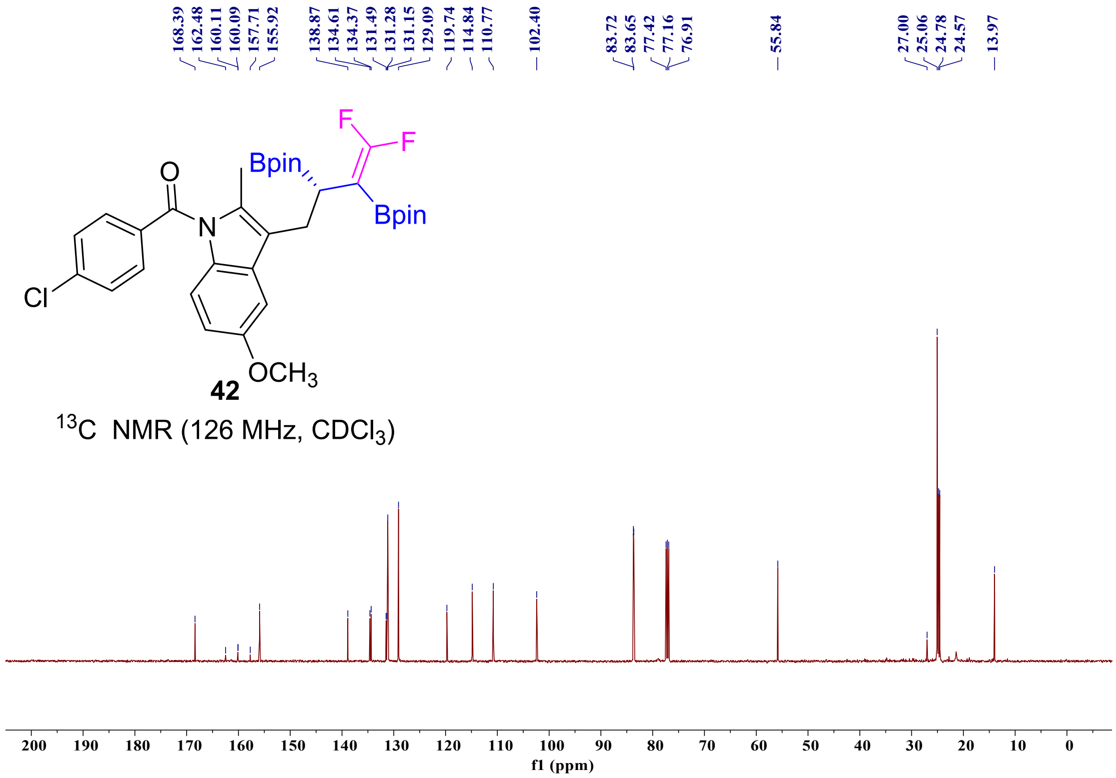

(S)-(4-chlorophenyl)(3-(4,4-difluoro-2,3-bis(4,4,5,5-tetramethyl-1,3,2-dioxaborolan-2-yl)but-3-en-1-yl)-2-methyl-1H-indol-1-yl)methanone (95)

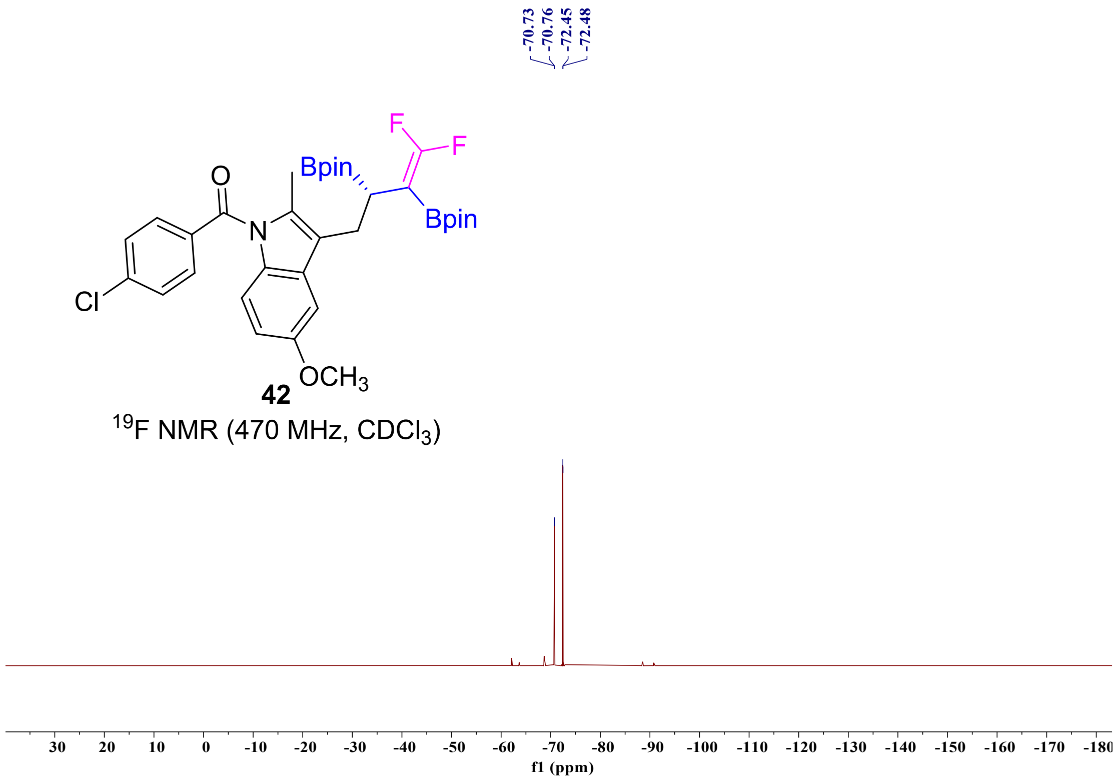

(S)-(4-chlorophenyl)(3-(4,4-difluoro-2,3-bis(4,4,5,5-tetramethyl-1,3,2-dioxaborolan-2-yl)but-3-en-1-yl)-2-methyl-1H-indol-1-yl)methanone (95)

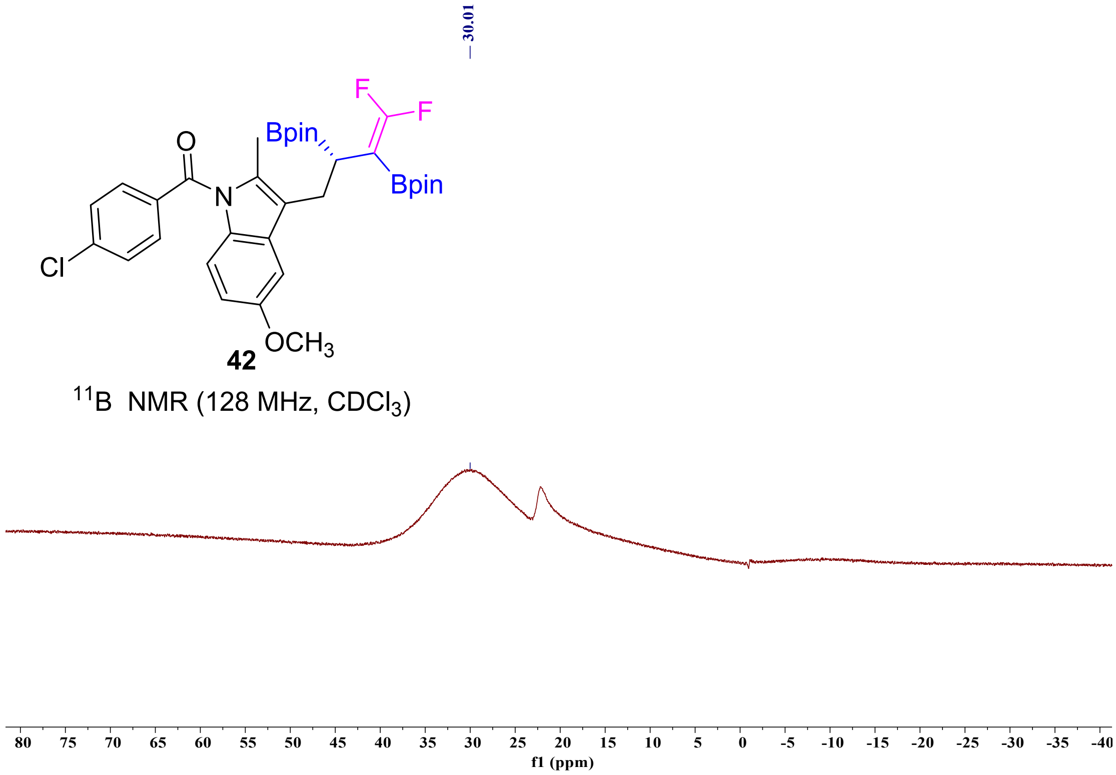

tert-butyl(((3R,8R,9S,10S,13R,14S,17R)-17-((2R,5S)-7,7-difluoro-5,6-bis(4,4,5,5-tetramethyl-1,3,2-dioxaborolan-2-yl)hept-6-en-2-yl)-10,13-dimethylhexadecahydro-1H-cyclopenta[a]phenanthren-3-yl)oxy)dimethylsilane(96)

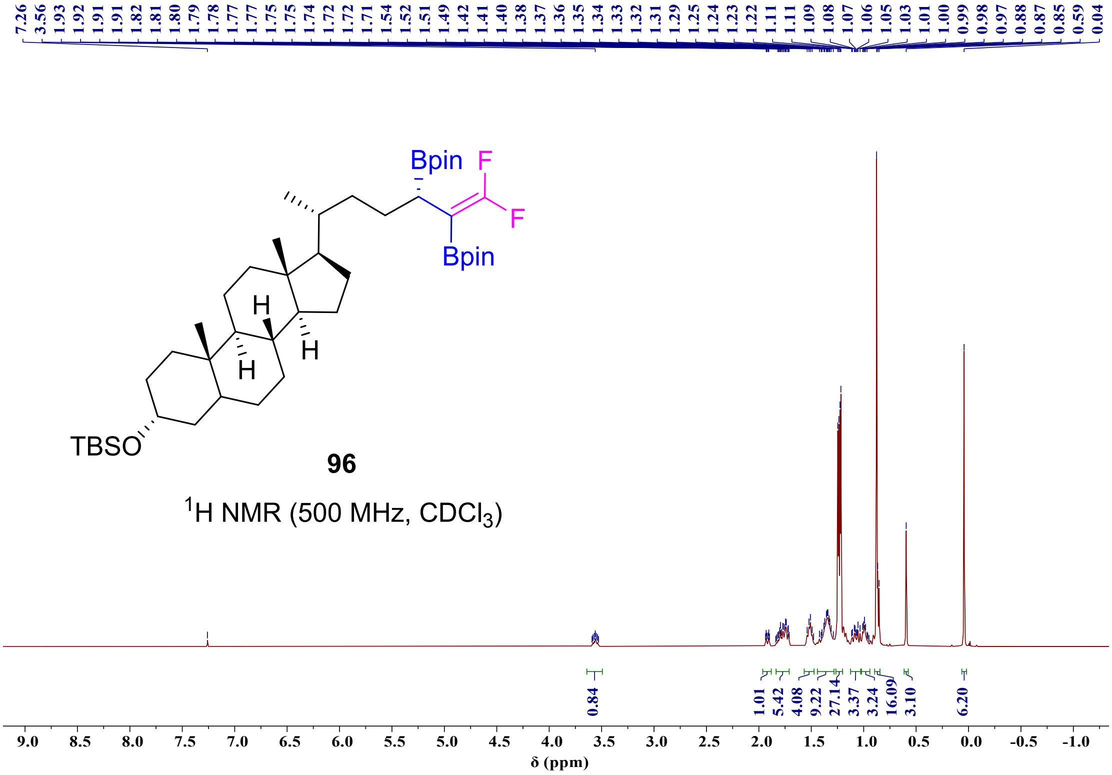

tert-butyl(((3R,8R,9S,10S,13R,14S,17R)-17-((2R,5S)-7,7-difluoro-5,6-bis(4,4,5,5-tetramethyl-1,3,2-dioxaborolan-2-yl)hept-6-en-2-yl)-10,13-dimethylhexadecahydro-1H-cyclopenta[a]phenanthren-3-yl)oxy)dimethylsilane(96)

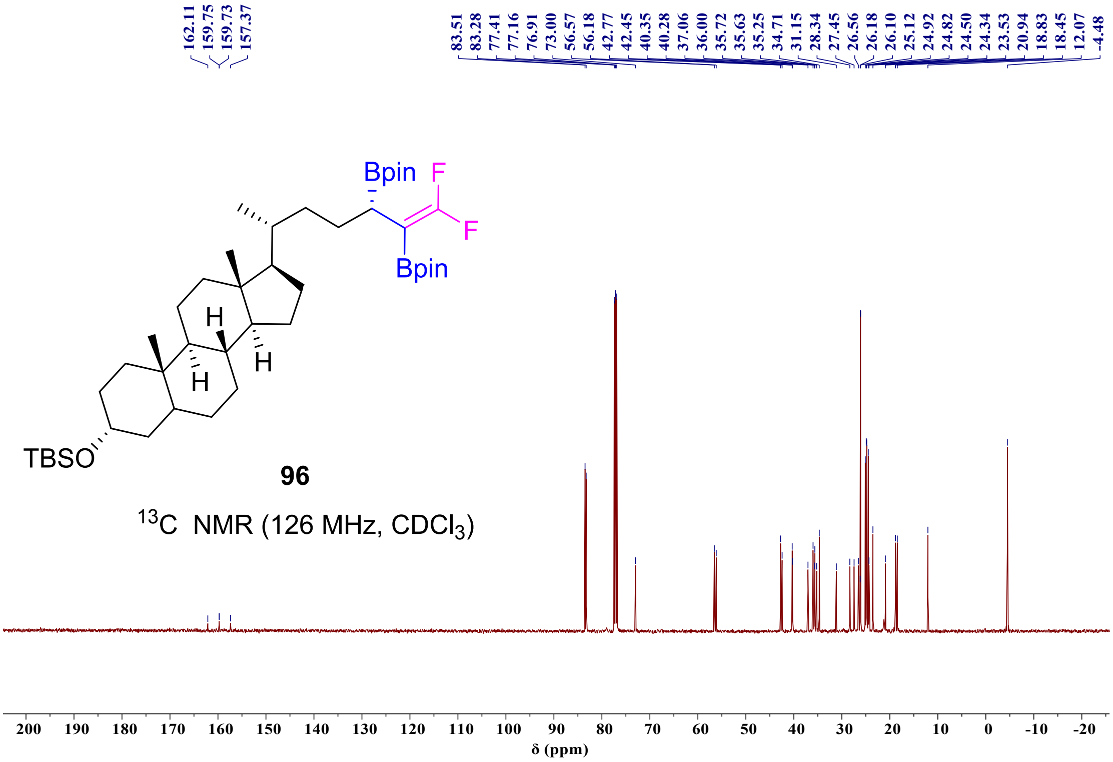

tert-butyl(((3R,8R,9S,10S,13R,14S,17R)-17-((2R,5S)-7,7-difluoro-5,6-bis(4,4,5,5-tetramethyl-1,3,2-dioxaborolan-2-yl)hept-6-en-2-yl)-10,13-dimethylhexadecahydro-1H-cyclopenta[a]phenanthren-3-yl)oxy)dimethylsilane(96)

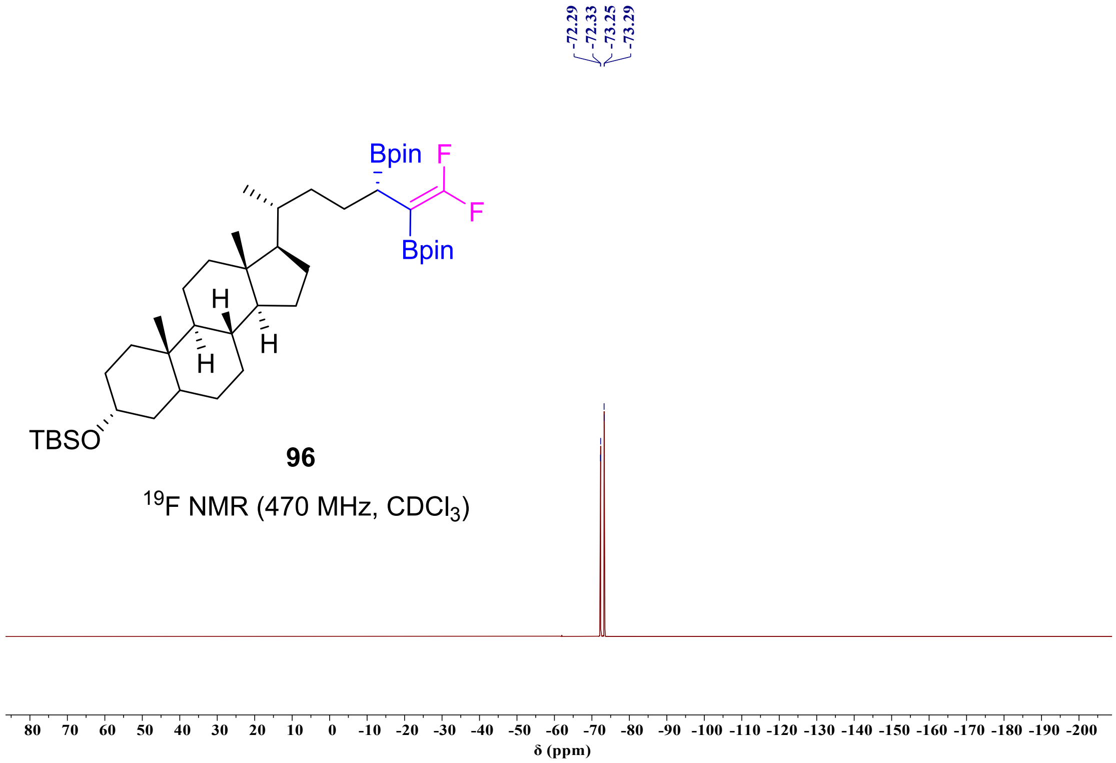

tert-butyl(((3R,8R,9S,10S,13R,14S,17R)-17-((2R,5S)-7,7-difluoro-5,6-bis(4,4,5,5-tetramethyl-1,3,2-dioxaborolan-2-yl)hept-6-en-2-yl)-10,13-dimethylhexadecahydro-1H-cyclopenta[a]phenanthren-3-yl)oxy)dimethylsilane(96)

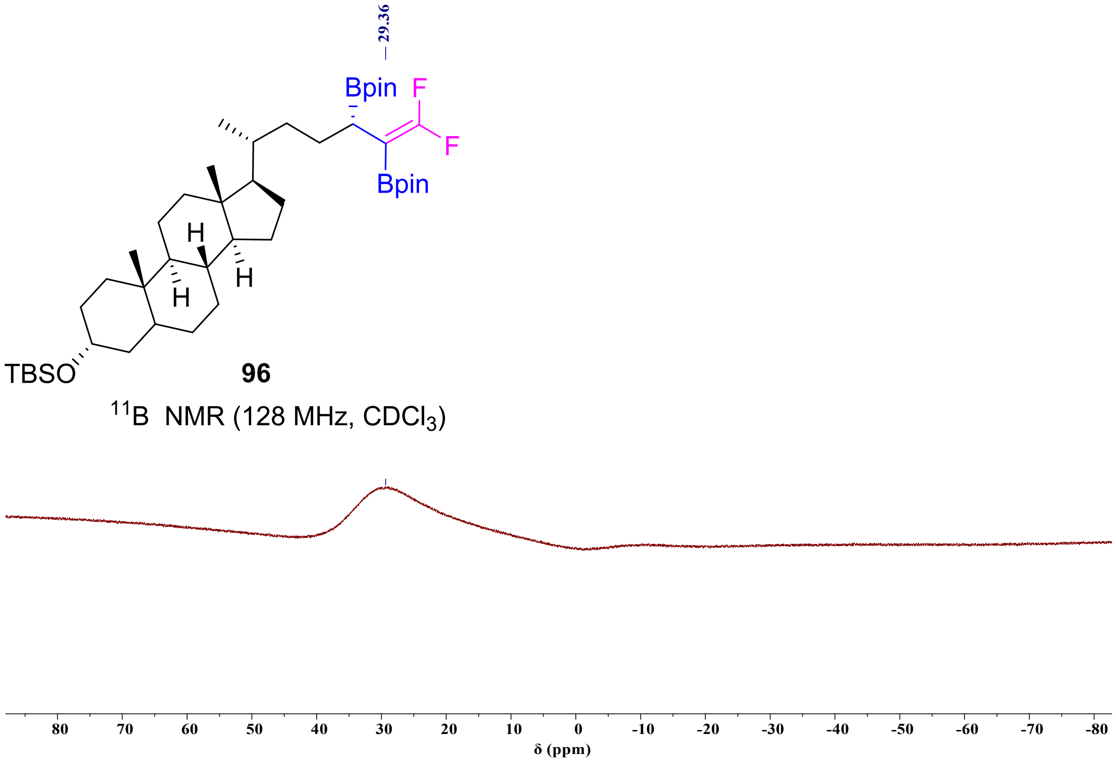

(8R,9S,13S,14S,17S)-3-(((S)-10,10-difluoro-8,9-bis(4,4,5,5-tetramethyl-1,3,2-dioxaborolan-2-yl)dec-9-en-1-yl)oxy)-13-methyl-7,8,9,11,12,13,14,15,16,17-decahydro-6H-cyclopenta[a]phenanthren-17-yl heptanoate (97)

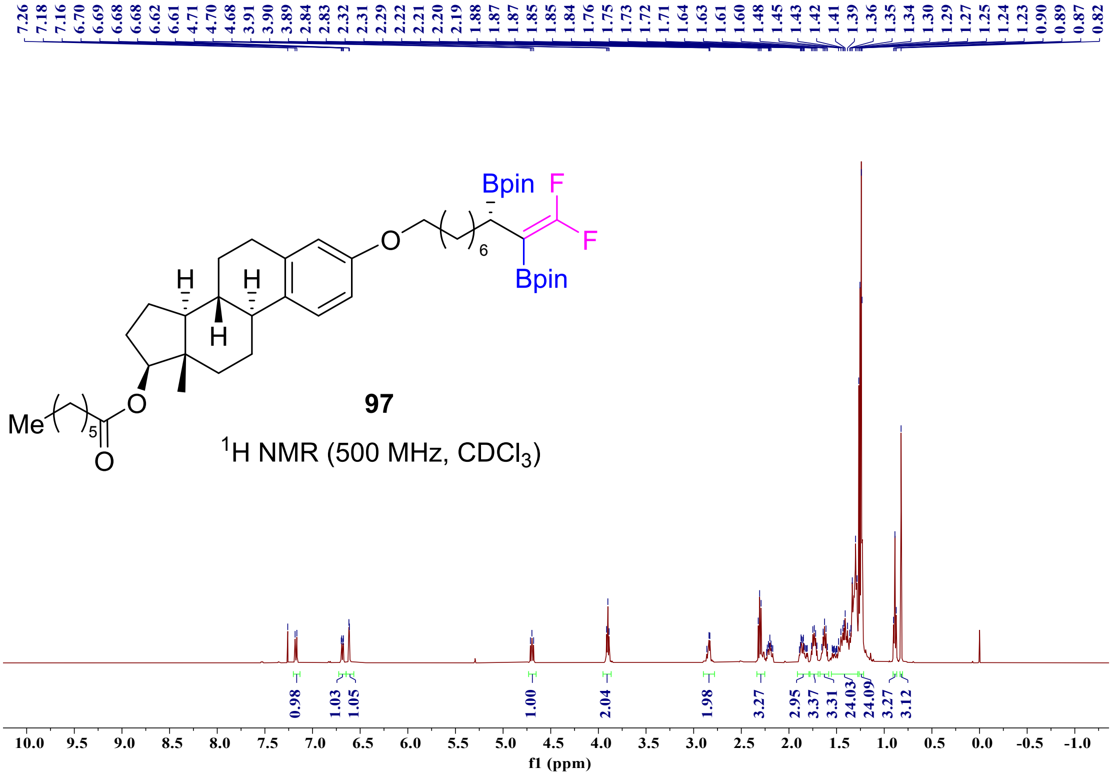

(8R,9S,13S,14S,17S)-3-(((S)-10,10-difluoro-8,9-bis(4,4,5,5-tetramethyl-1,3,2-dioxaborolan-2-yl)dec-9-en-1-yl)oxy)-13-methyl-7,8,9,11,12,13,14,15,16,17-decahydro-6H-cyclopenta[a]phenanthren-17-yl heptanoate (97)

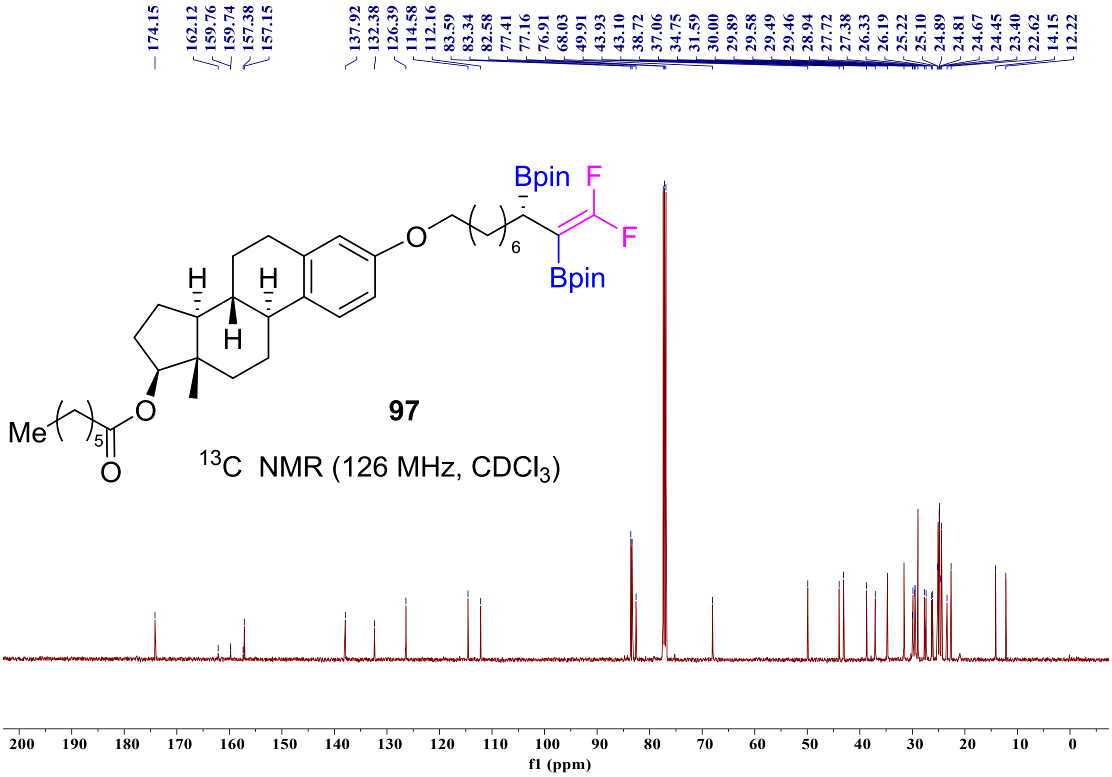

(8R,9S,13S,14S,17S)-3-(((S)-10,10-difluoro-8,9-bis(4,4,5,5-tetramethyl-1,3,2-dioxaborolan-2-yl)dec-9-en-1-yl)oxy)-13-methyl-7,8,9,11,12,13,14,15,16,17-decahydro-6H-cyclopenta[a]phenanthren-17-yl heptanoate (97)

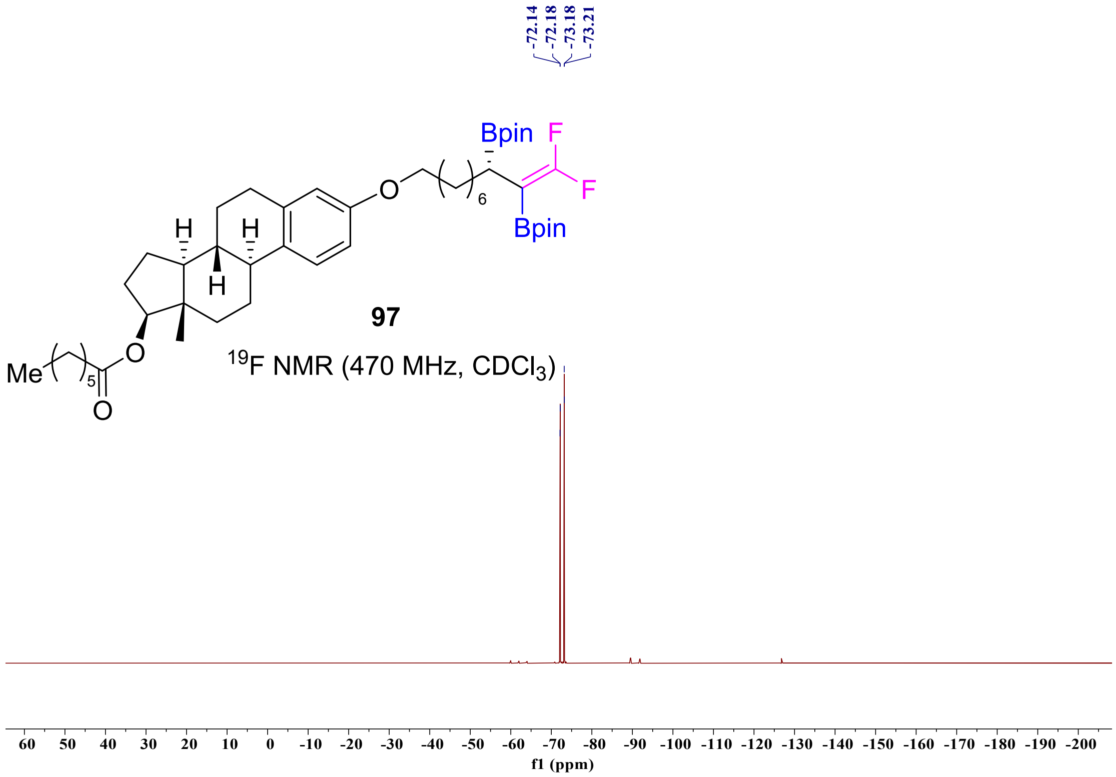

(8R,9S,13S,14S,17S)-3-(((S)-10,10-difluoro-8,9-bis(4,4,5,5-tetramethyl-1,3,2-dioxaborolan-2-yl)dec-9-en-1-yl)oxy)-13-methyl-7,8,9,11,12,13,14,15,16,17-decahydro-6H-cyclopenta[a]phenanthren-17-yl heptanoate (97)

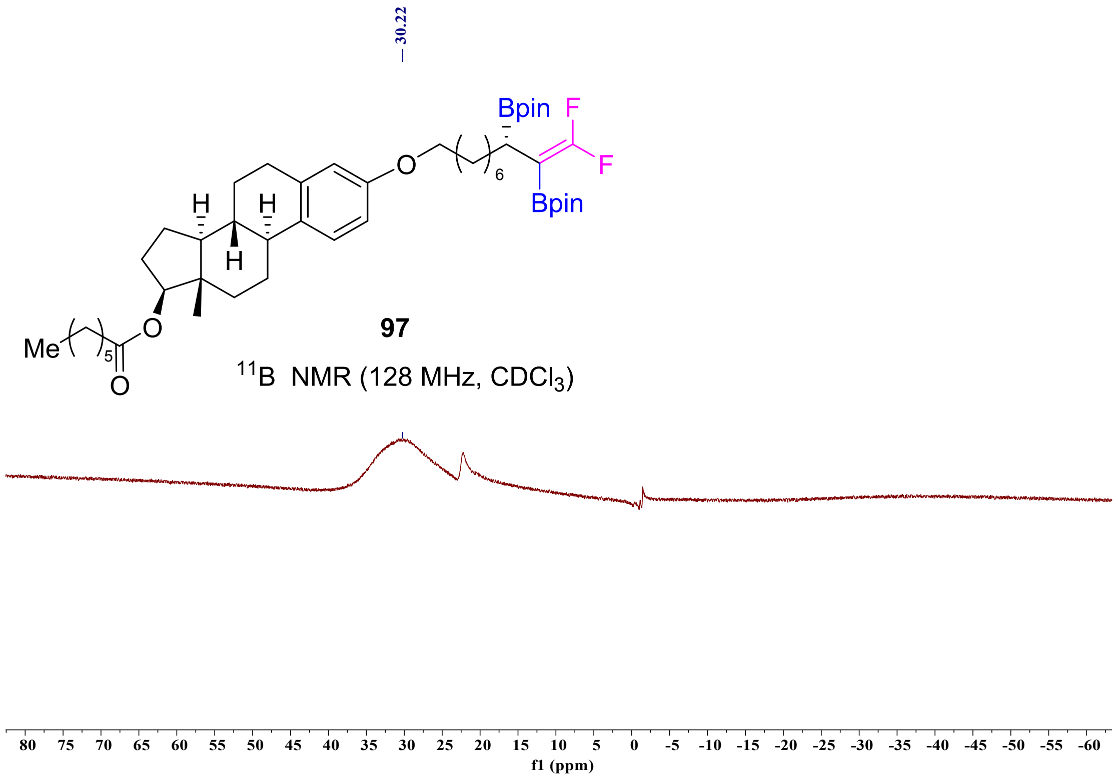

methyl (S)-4-(1,1-difluoro-3-hydroxy-5-phenylpent-1-en-2-yl)benzoate (98)

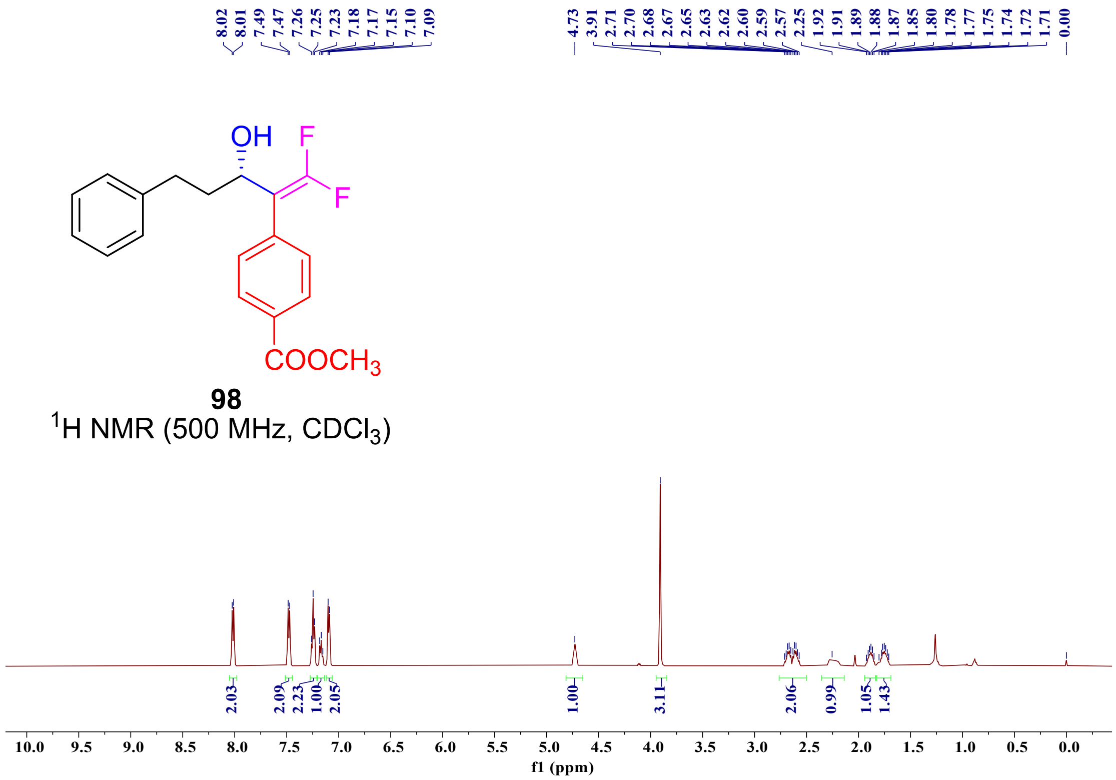

methyl (S)-4-(1,1-difluoro-3-hydroxy-5-phenylpent-1-en-2-yl)benzoate (98)

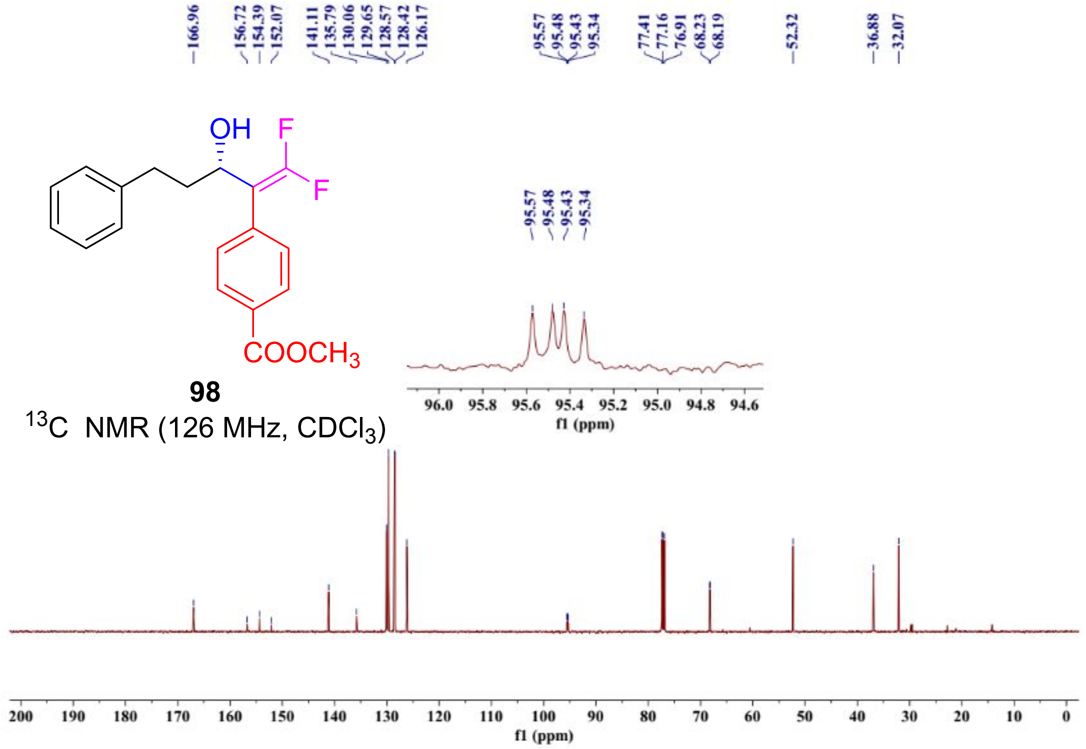

methyl (S)-4-(1,1-difluoro-3-hydroxy-5-phenylpent-1-en-2-yl)benzoate (98)

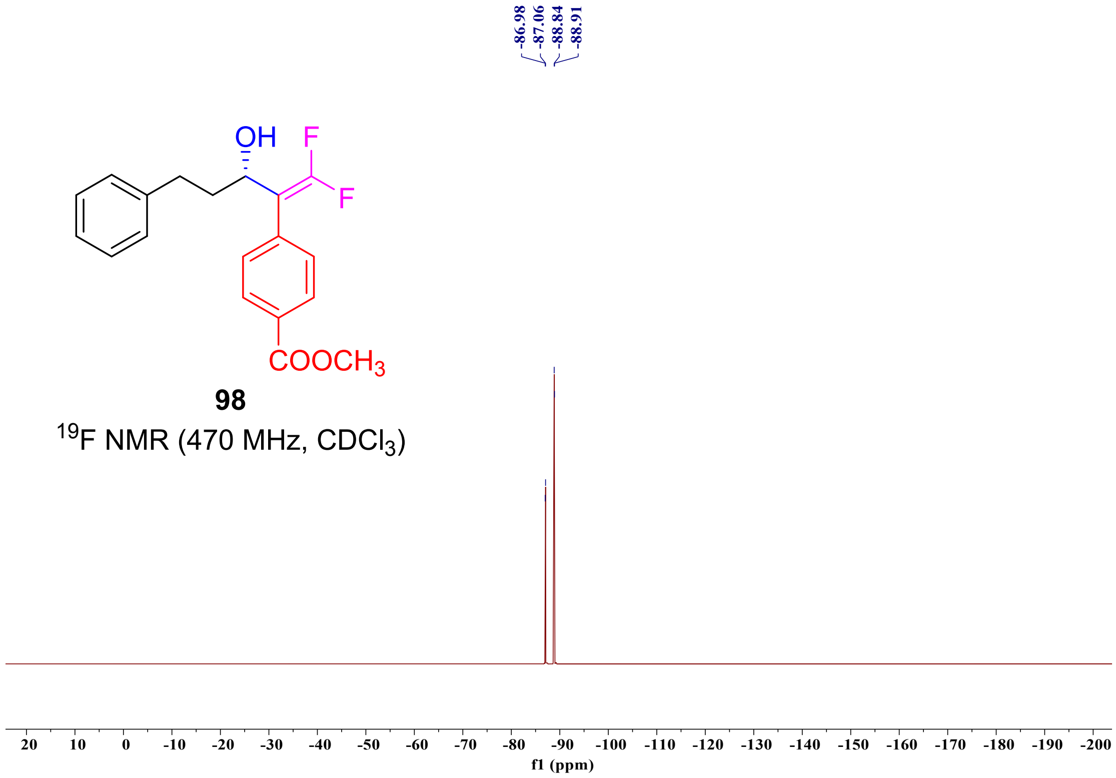

methyl (S)-4-(1,1-difluoro-3-hydroxy-5-(4-(trifluoromethyl)phenyl)pent-1-en-2-yl)benzoate (99)

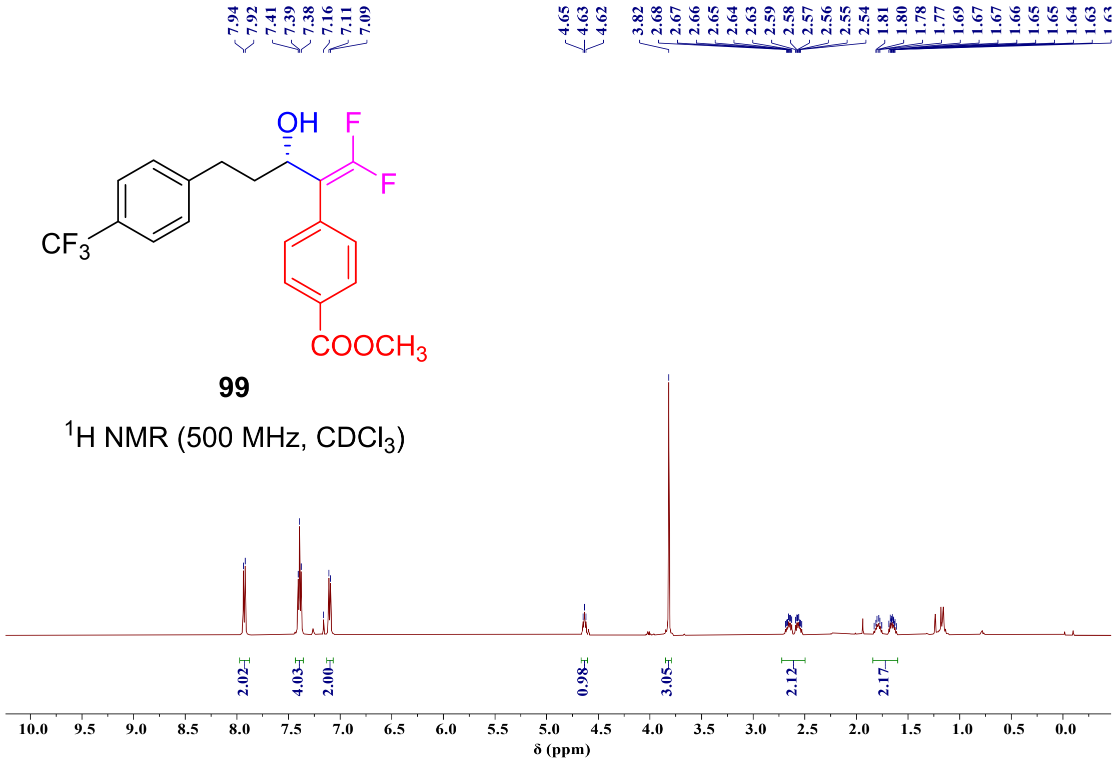

methyl (S)-4-(1,1-difluoro-3-hydroxy-5-(4-(trifluoromethyl)phenyl)pent-1-en-2-yl)benzoate (99)

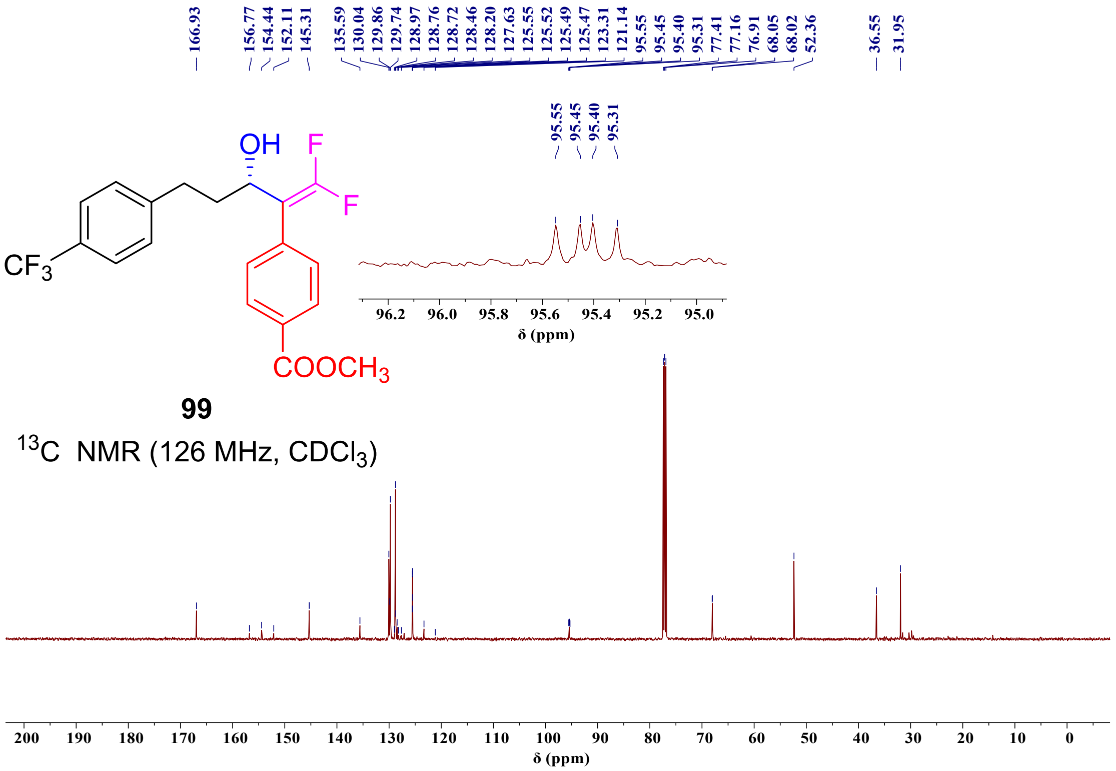

methyl (S)-4-(1,1-difluoro-3-hydroxy-5-(4-(trifluoromethyl)phenyl)pent-1-en-2-yl)benzoate (99)

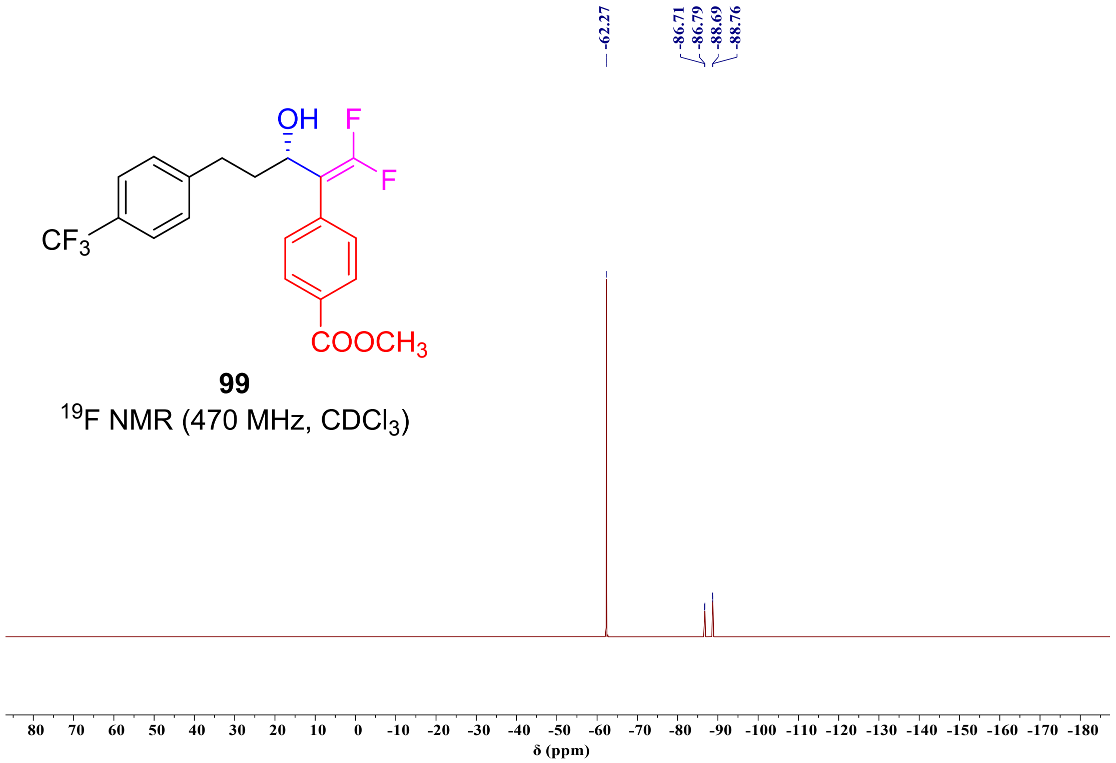

methyl (S)-4-(1,1-difluoro-3-hydroxydodec-1-en-2-yl)benzoate (100)

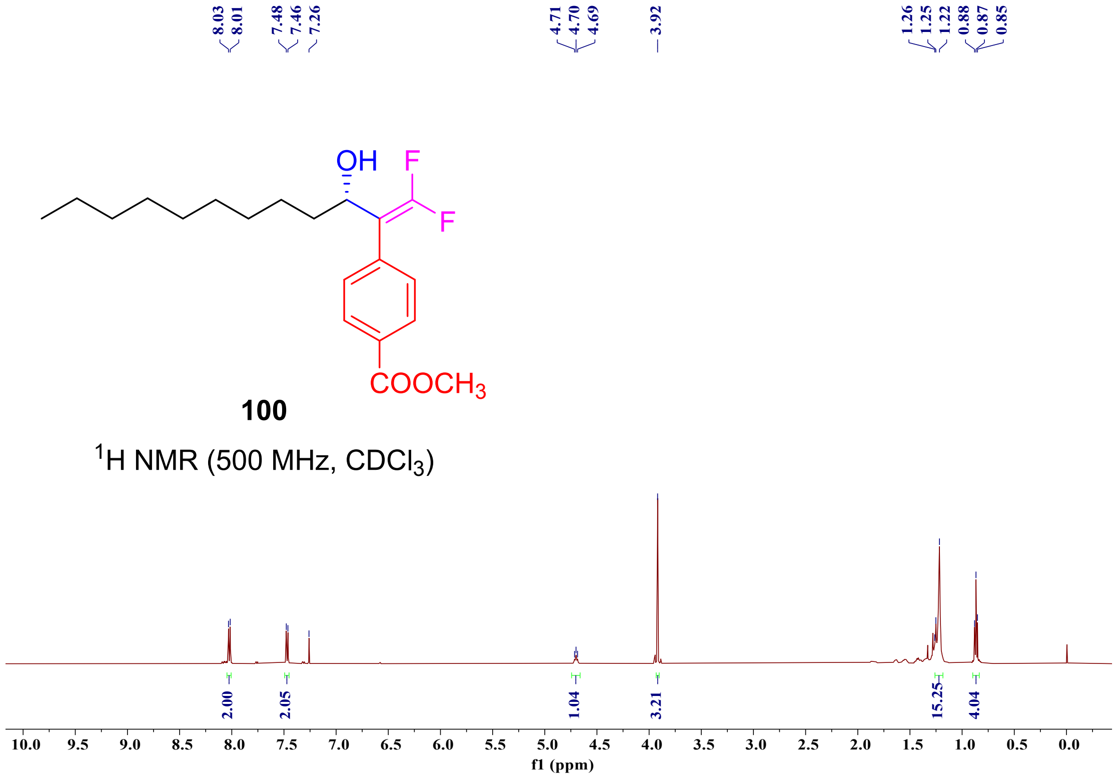

methyl (S)-4-(1,1-difluoro-3-hydroxydodec-1-en-2-yl)benzoate (100)

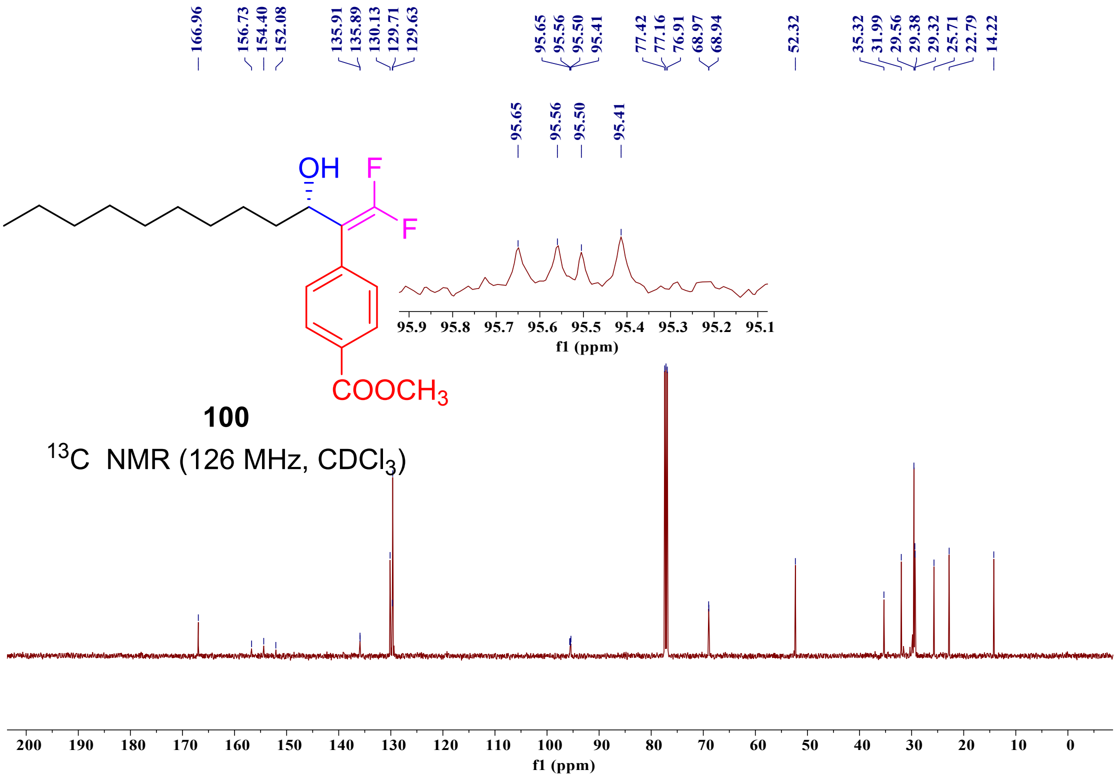

methyl (S)-4-(1,1-difluoro-3-hydroxydodec-1-en-2-yl)benzoate (100)

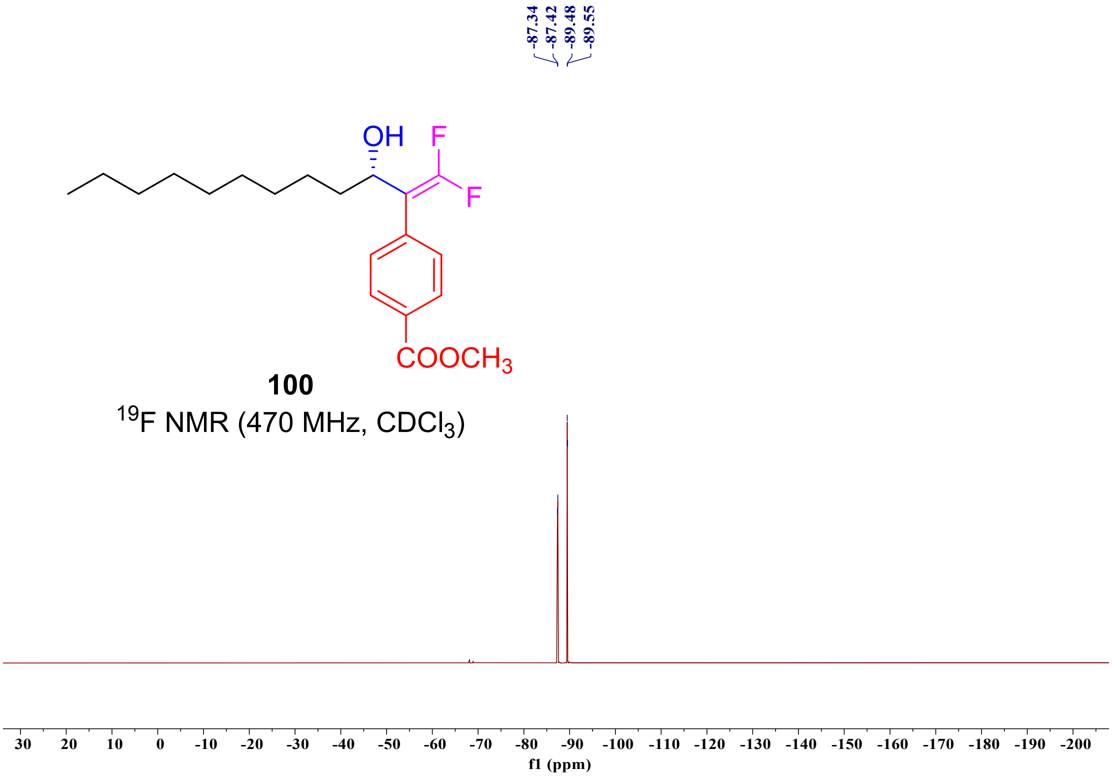

methyl (S)-4-(11-bromo-1,1-difluoro-3-hydroxyundec-1-en-2-yl)benzoate (101)

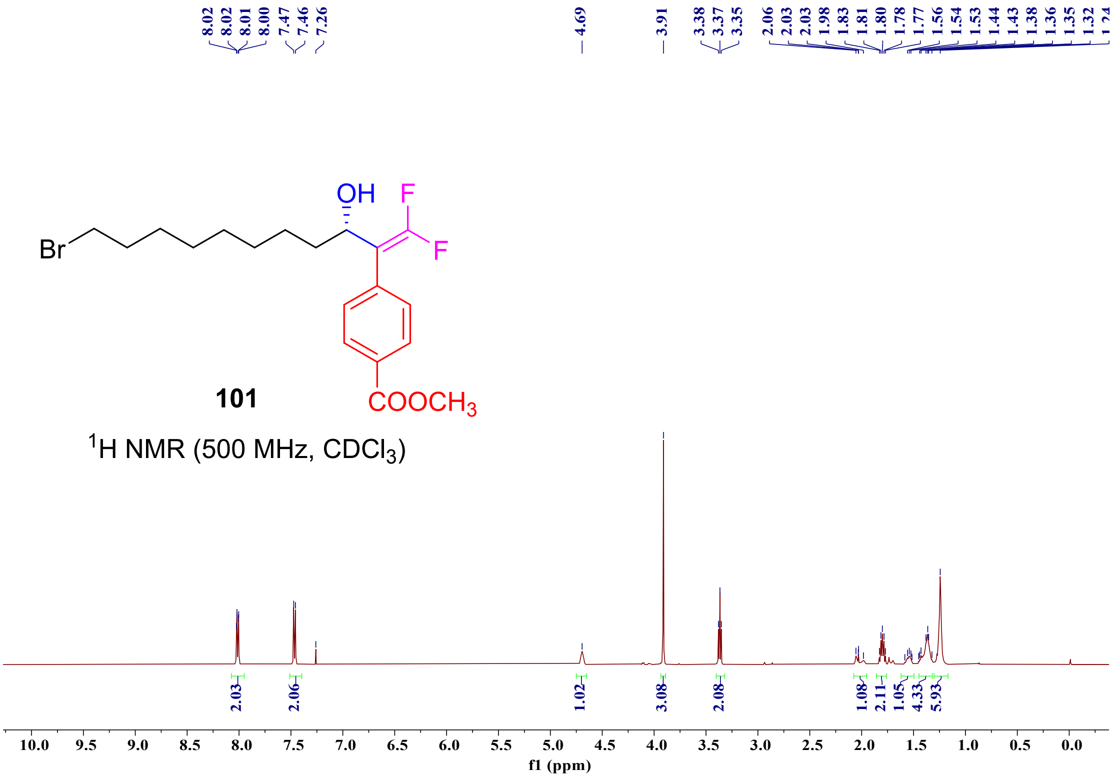

methyl (S)-4-(11-bromo-1,1-difluoro-3-hydroxyundec-1-en-2-yl)benzoate (101)

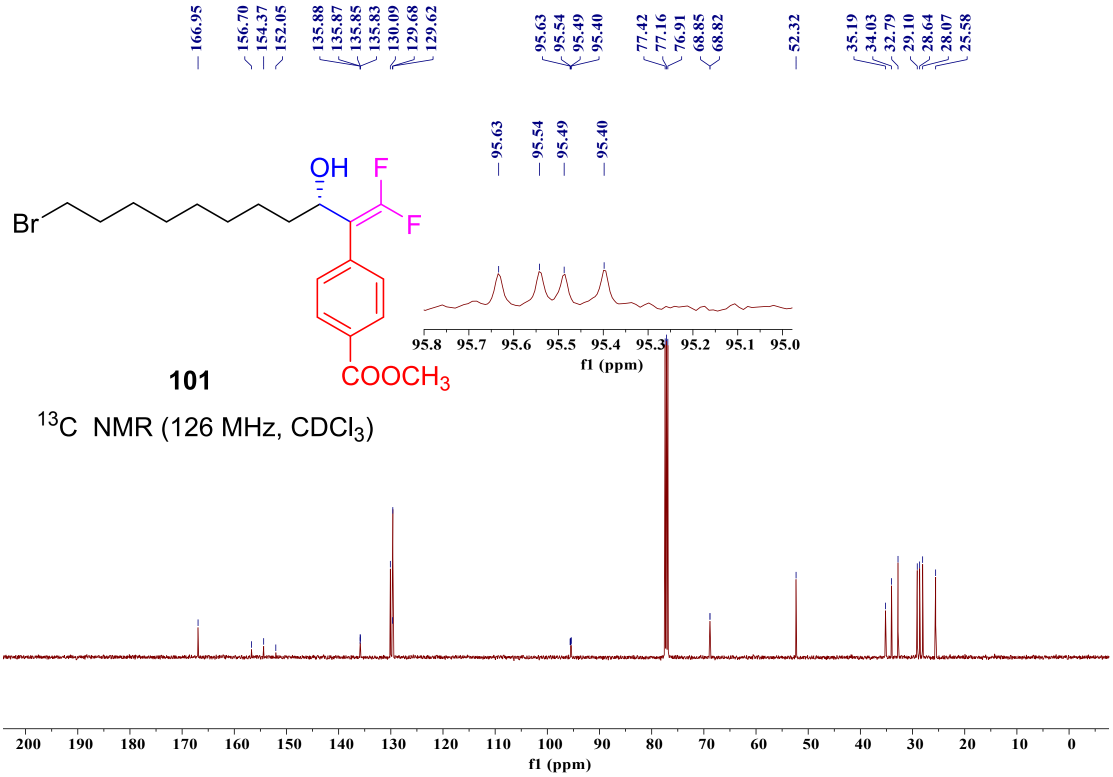

methyl (S)-4-(11-bromo-1,1-difluoro-3-hydroxyundec-1-en-2-yl)benzoate (101)

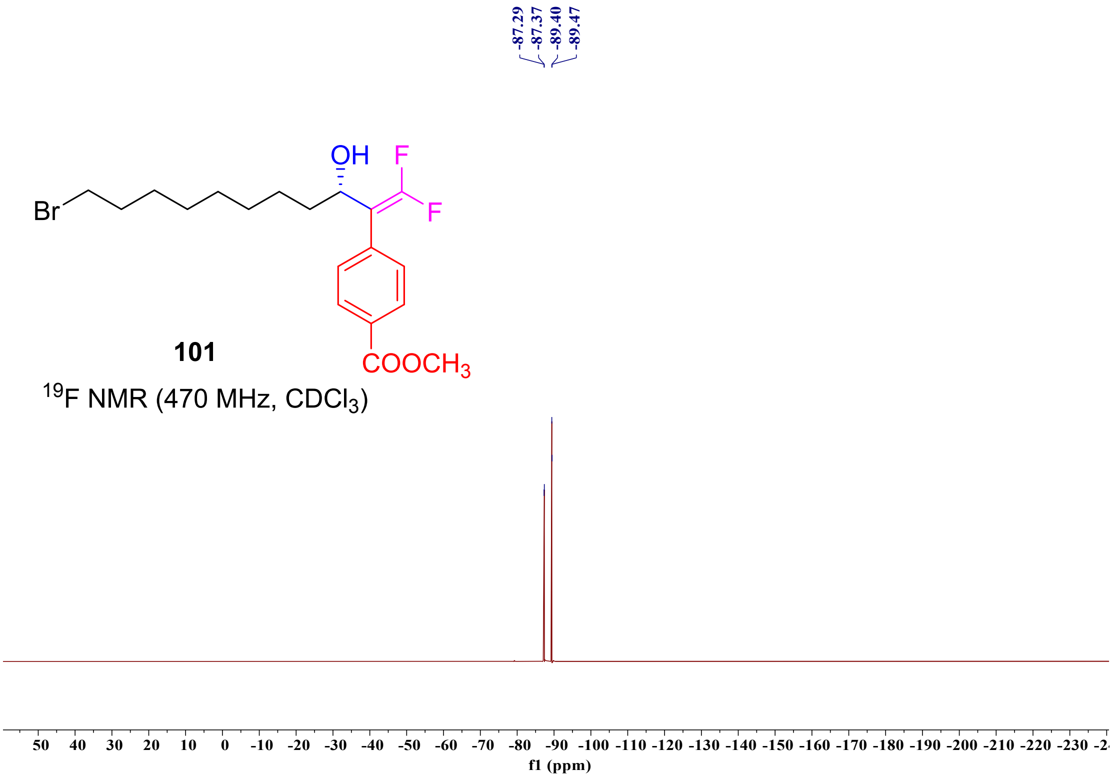

(S)-2-(2-bromo-1,1-difluoro-5-phenylpent-1-en-3-yl)-4,4,5,5-tetramethyl-1,3,2-dioxaborolane(102)

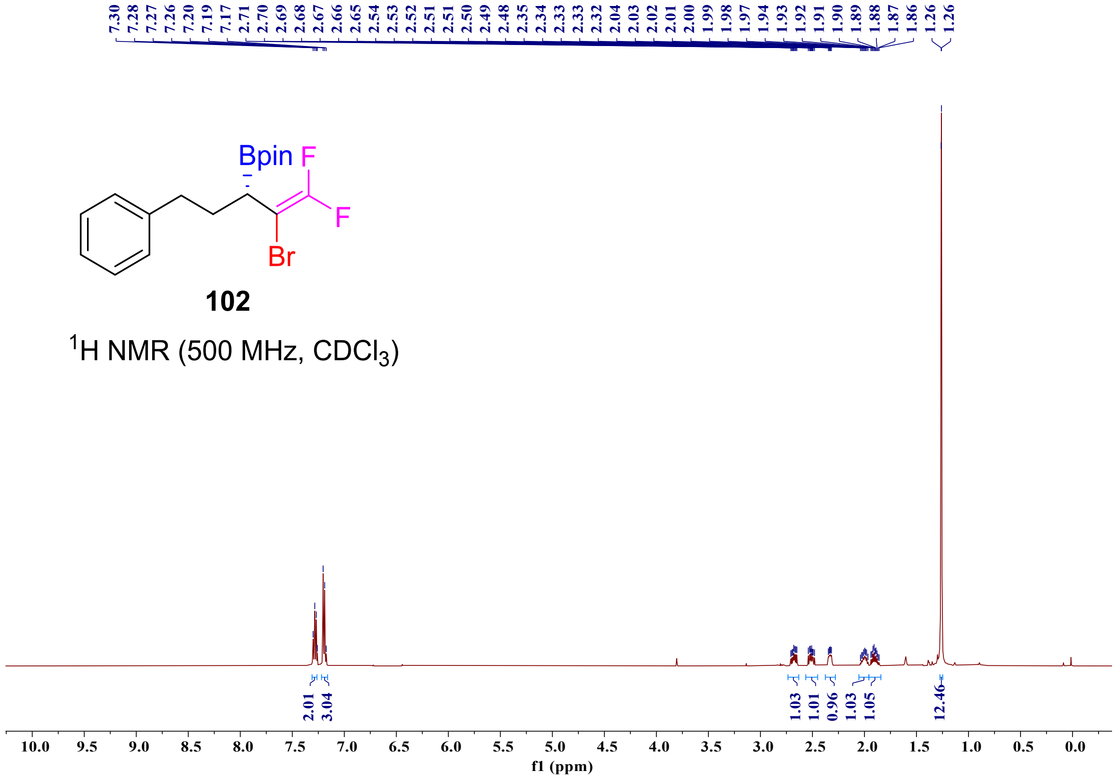

(S)-2-(2-bromo-1,1-difluoro-5-phenylpent-1-en-3-yl)-4,4,5,5-tetramethyl-1,3,2-dioxaborolane(102)

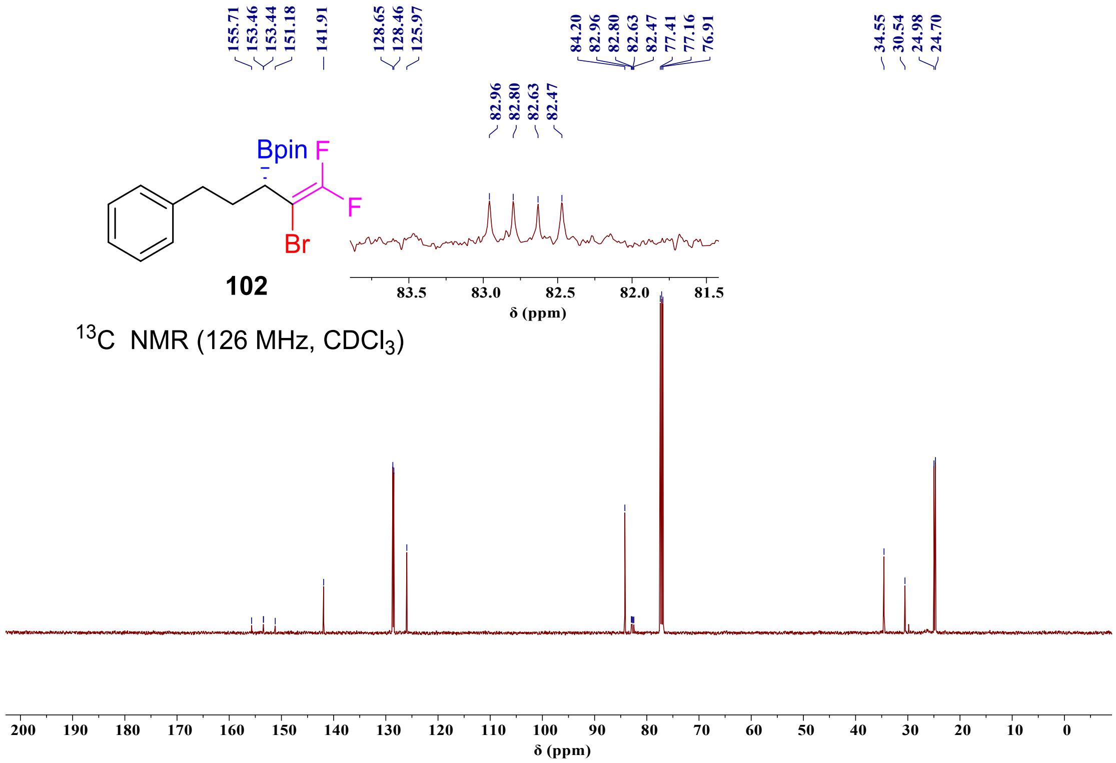

(S)-2-(2-bromo-1,1-difluoro-5-phenylpent-1-en-3-yl)-4,4,5,5-tetramethyl-1,3,2-dioxaborolane(102)

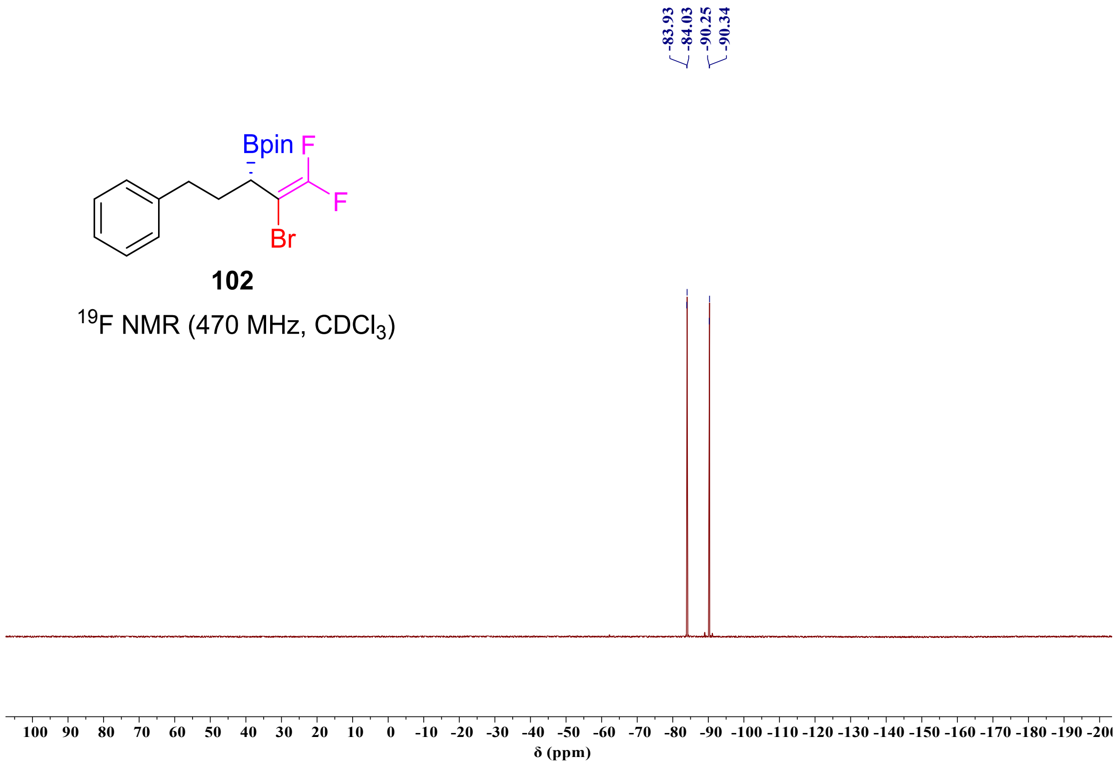

(S)-2-(2-bromo-1,1-difluoro-5-phenylpent-1-en-3-yl)-4,4,5,5-tetramethyl-1,3,2-dioxaborolane(102)

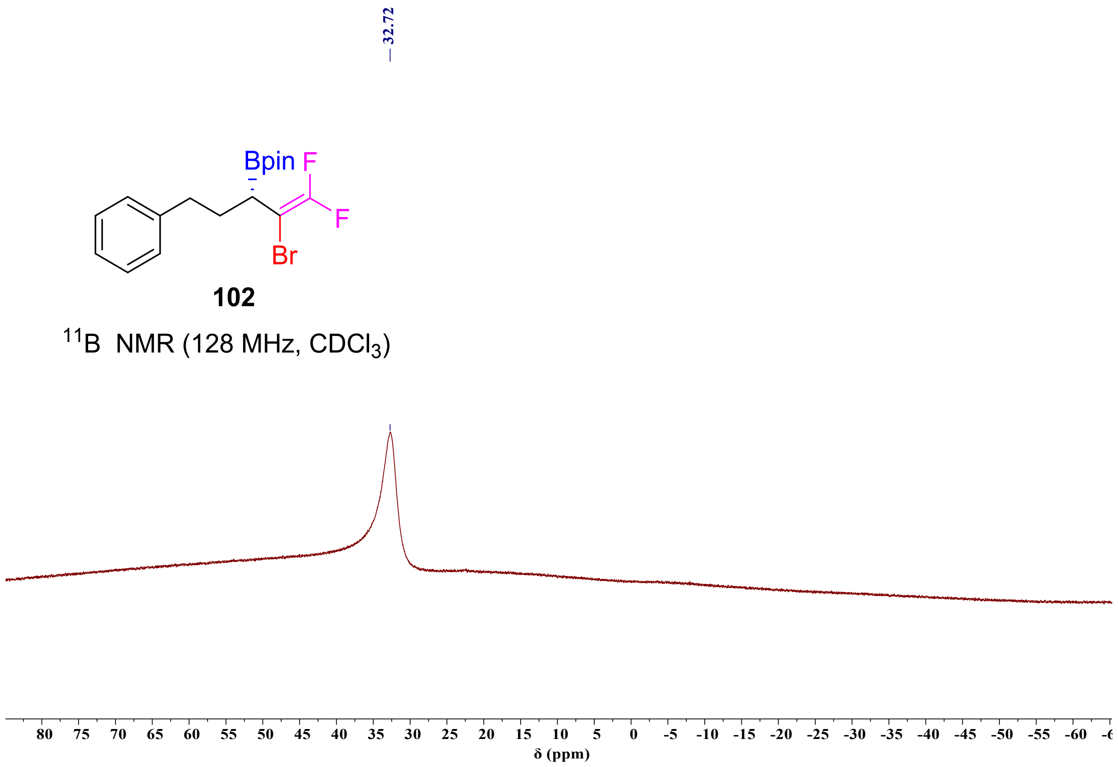

(S)-2-(1,1-difluoro-2-methyl-5-phenylpent-1-en-3-yl)-4,4,5,5-tetramethyl-1,3,2-dioxaborolane (103)

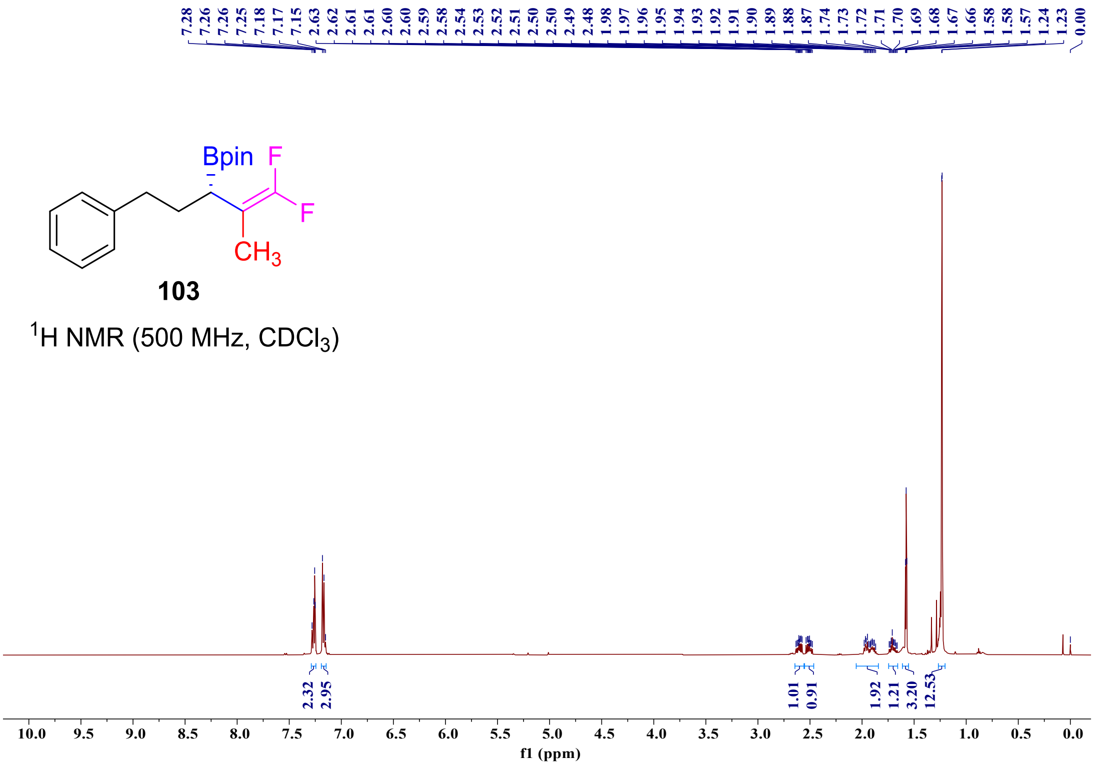

(S)-2-(1,1-difluoro-2-methyl-5-phenylpent-1-en-3-yl)-4,4,5,5-tetramethyl-1,3,2-dioxaborolane (103)

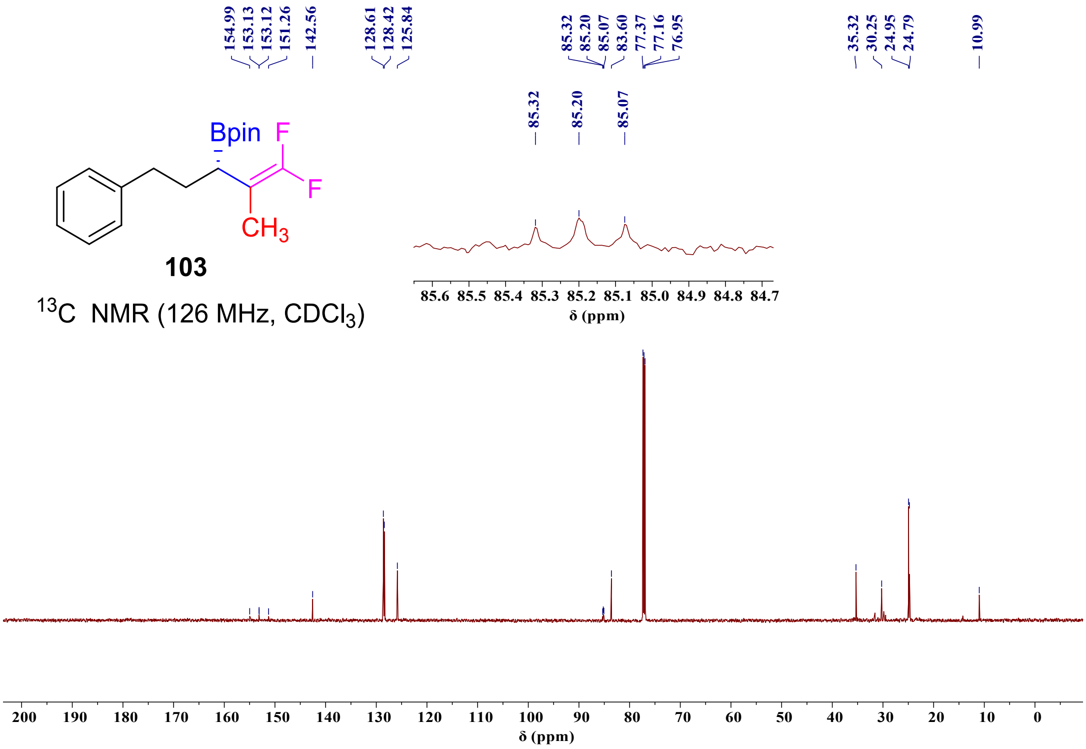

(S)-2-(1,1-difluoro-2-methyl-5-phenylpent-1-en-3-yl)-4,4,5,5-tetramethyl-1,3,2-dioxaborolane (103)

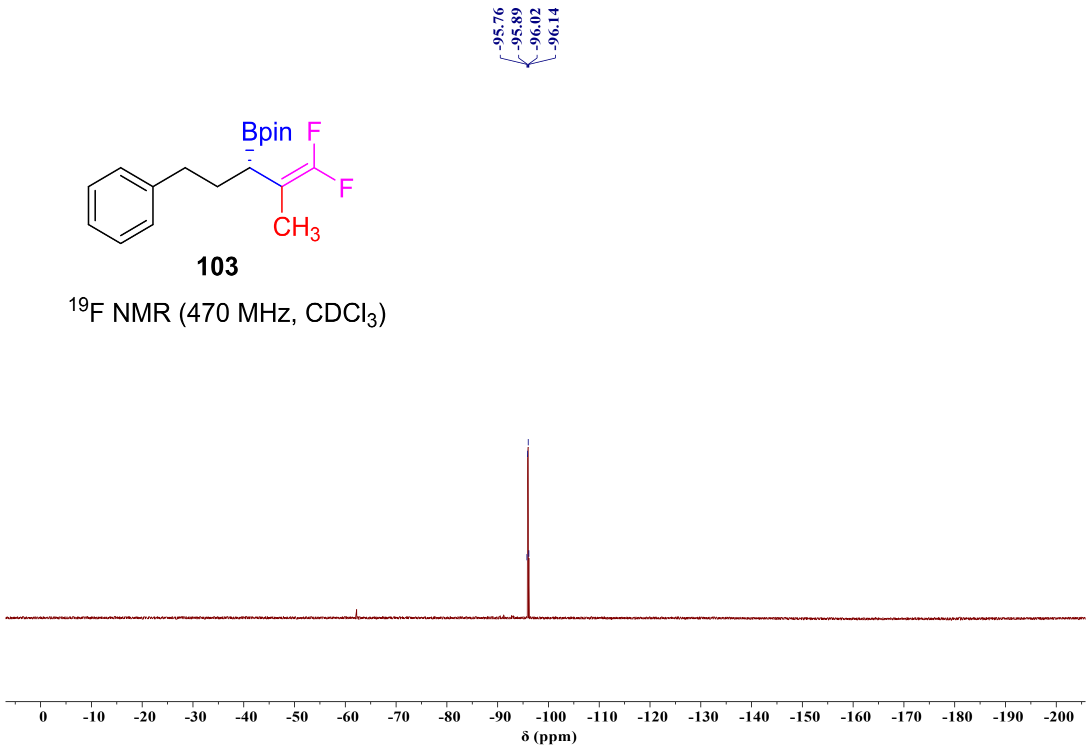

(S)-2-(1,1-difluoro-2-methyl-5-phenylpent-1-en-3-yl)-4,4,5,5-tetramethyl-1,3,2-dioxaborolane (103)

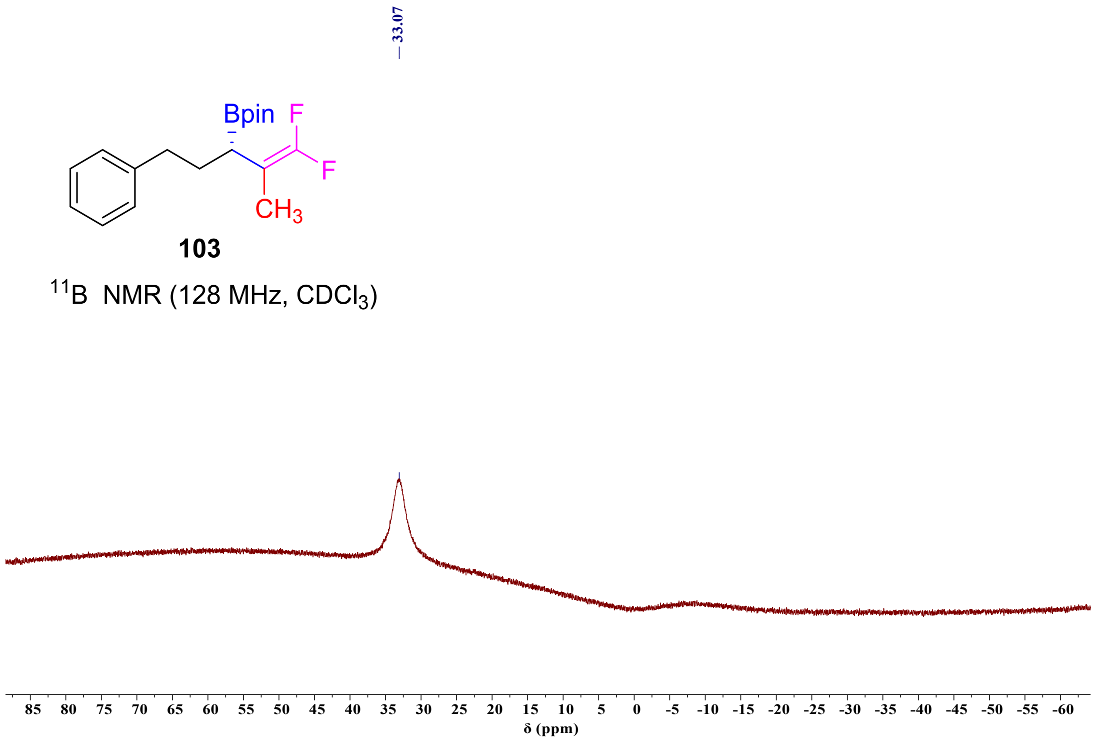

(S)-2-(1,1-difluoro-5-phenyl-2-(thiophen-2-yl)pent-1-en-3-yl)-4,4,5,5-tetramethyl-1,3,2-dioxaborolane (104)

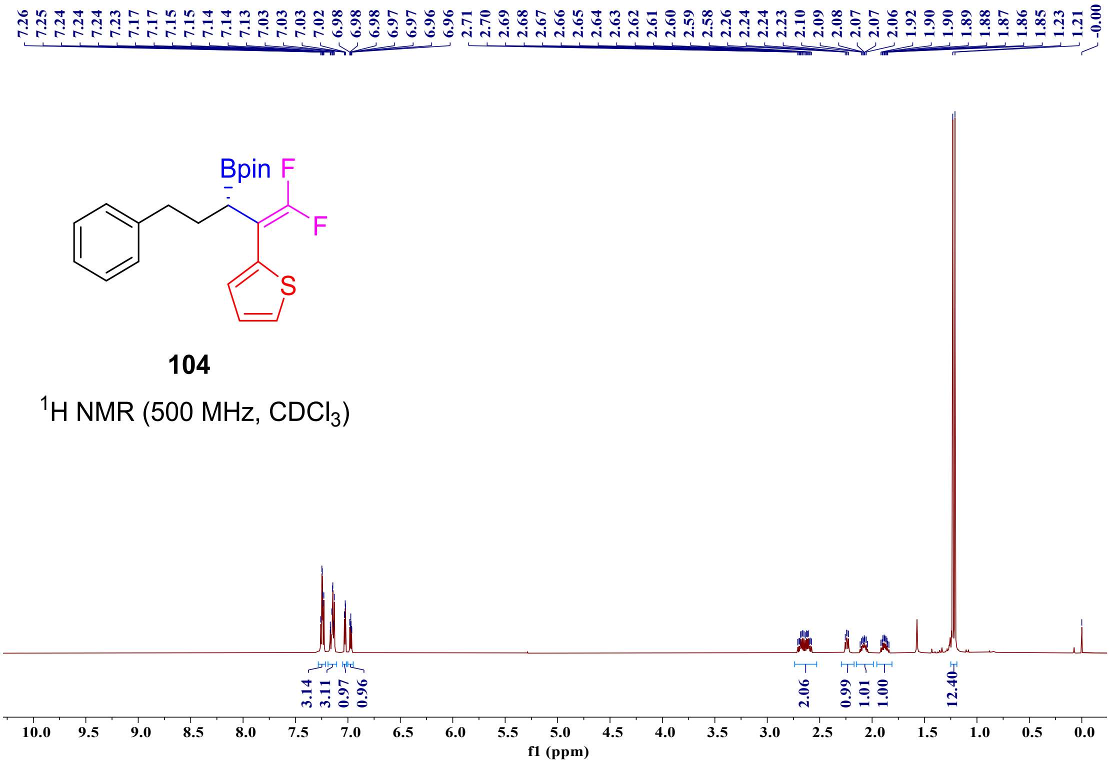

(S)-2-(1,1-difluoro-5-phenyl-2-(thiophen-2-yl)pent-1-en-3-yl)-4,4,5,5-tetramethyl-1,3,2-dioxaborolane(104)

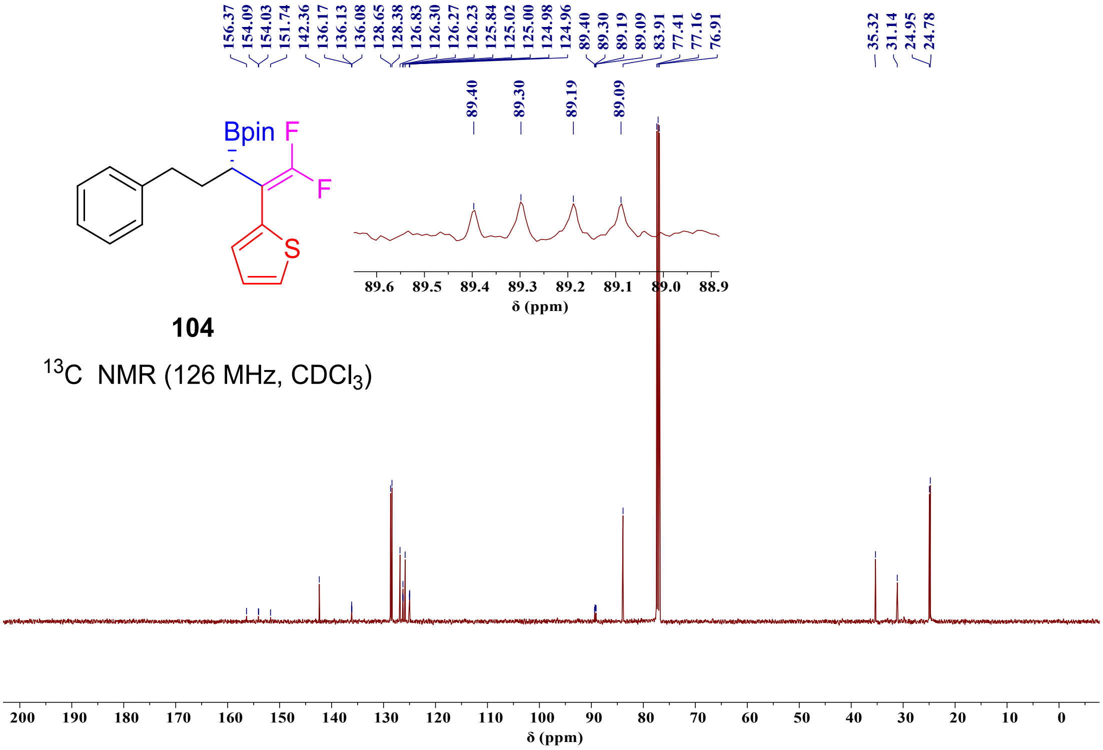

(S)-2-(1,1-difluoro-5-phenyl-2-(thiophen-2-yl)pent-1-en-3-yl)-4,4,5,5-tetramethyl-1,3,2-dioxaborolane (104)

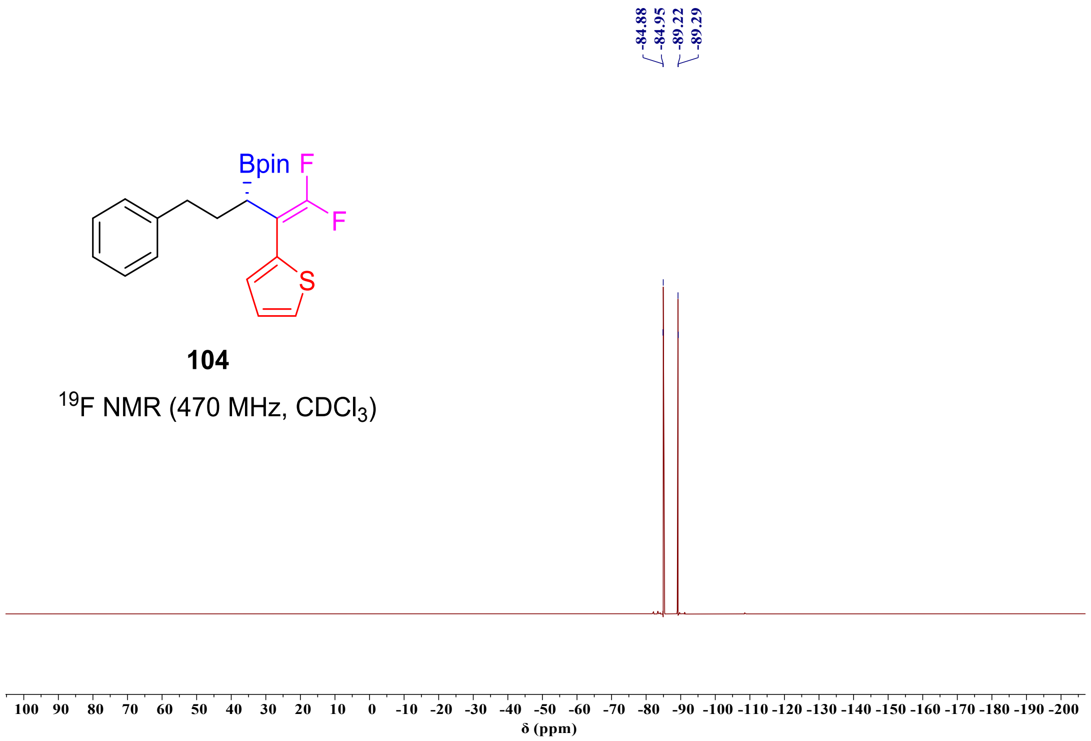

(S)-2-(1,1-difluoro-5-phenyl-2-(thiophen-2-yl)pent-1-en-3-yl)-4,4,5,5-tetramethyl-1,3,2-dioxaborolane(104)

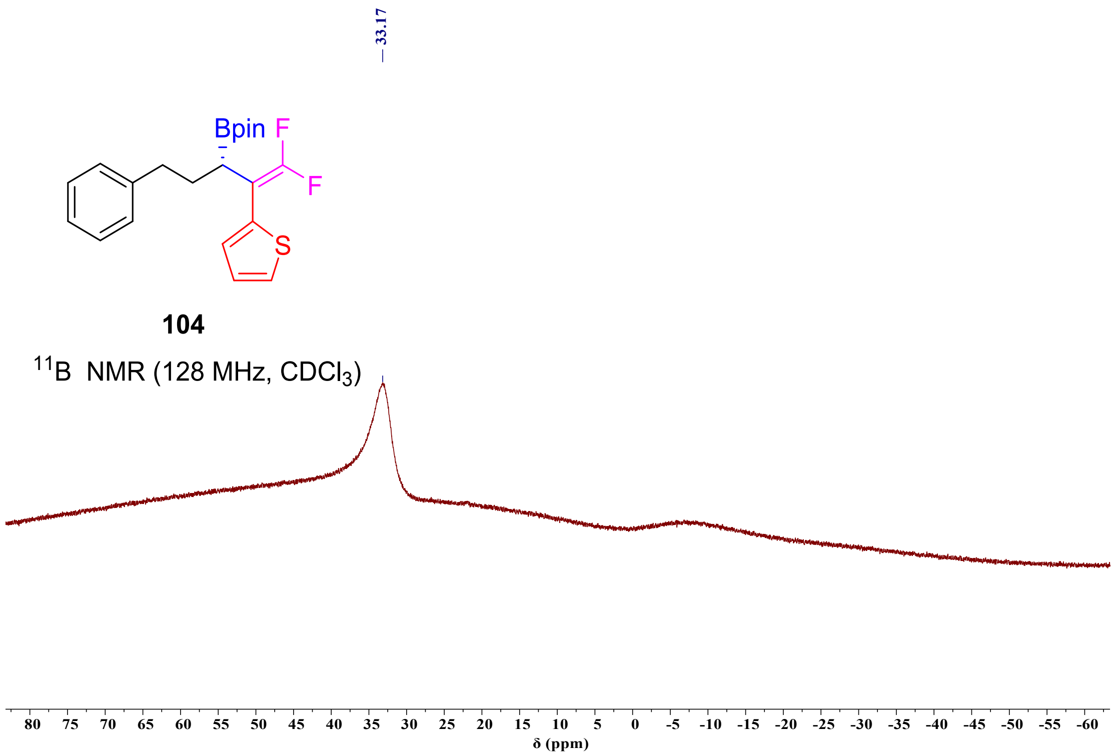

(S,E)-2-(4-(difluoromethylene)-1,6-diphenylhex-5-en-3-yl)-4,4,5,5-tetramethyl-1,3,2-dioxaborolane(105)

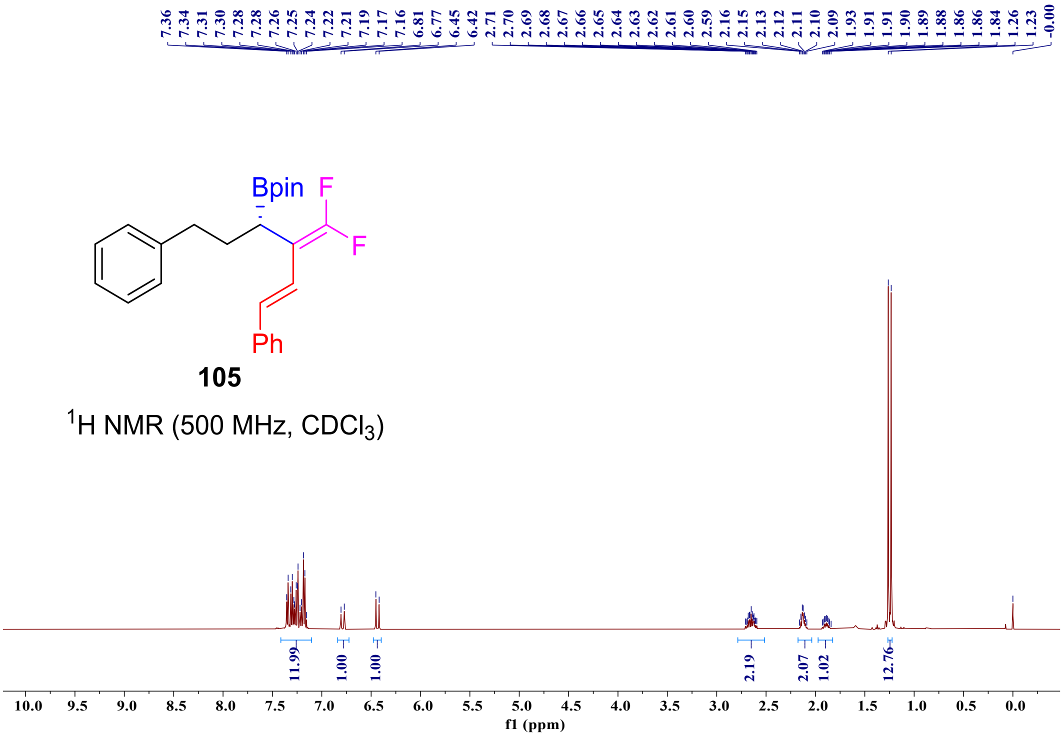

(S,E)-2-(4-(difluoromethylene)-1,6-diphenylhex-5-en-3-yl)-4,4,5,5-tetramethyl-1,3,2-dioxaborolane (105)

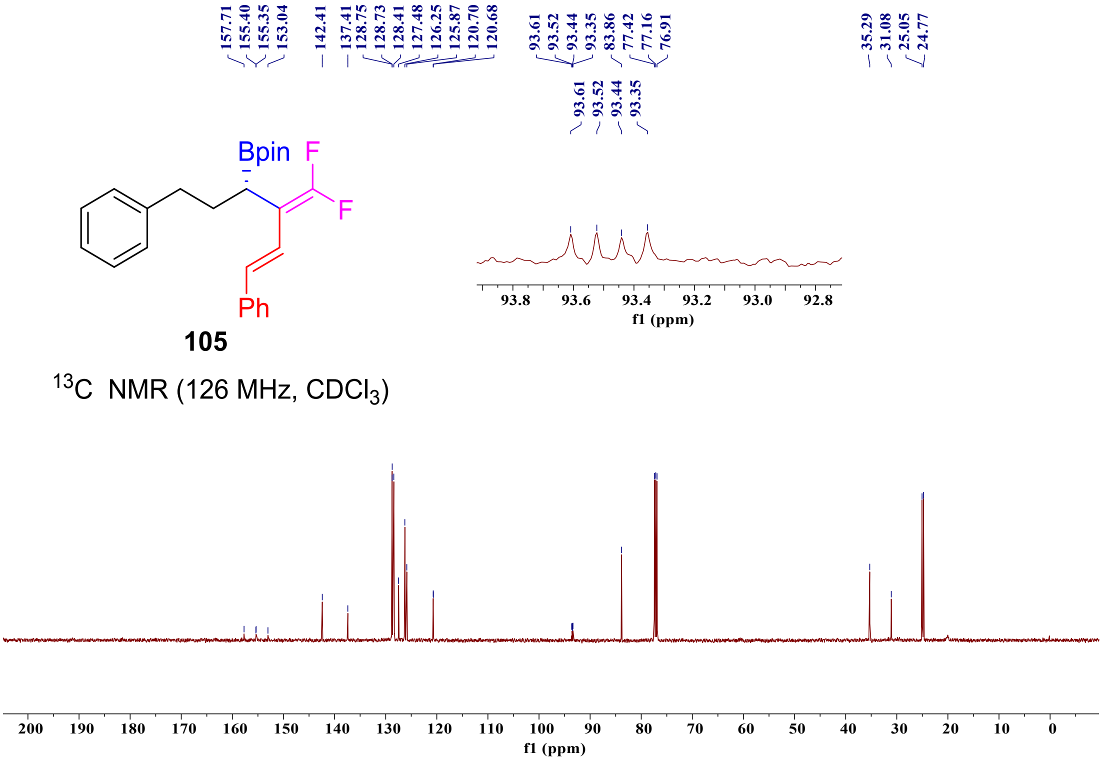

(S,E)-2-(4-(difluoromethylene)-1,6-diphenylhex-5-en-3-yl)-4,4,5,5-tetramethyl-1,3,2-dioxaborolane (105)

-88.16
-88.22
-88.86
-88.93

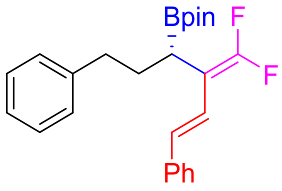

105

^{19}F NMR (470 MHz, $CDCl_3$)

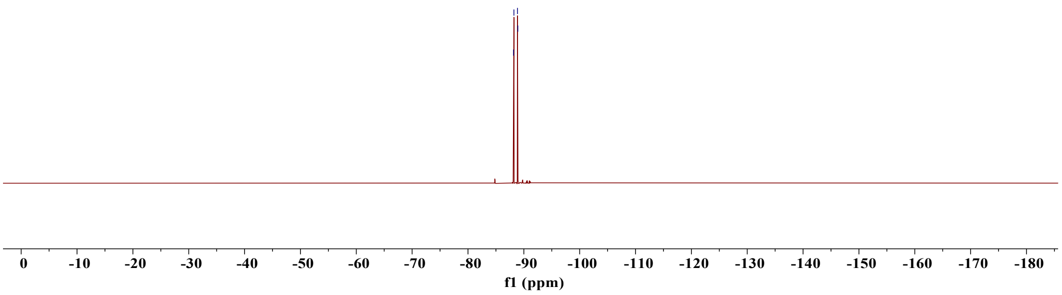

(S,E)-2-(4-(difluoromethylene)-1,6-diphenylhex-5-en-3-yl)-4,4,5,5-tetramethyl-1,3,2-dioxaborolane (105)

34.28

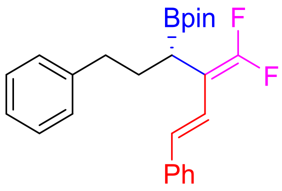

105

^{11}B NMR (128 MHz, $CDCl_3$)

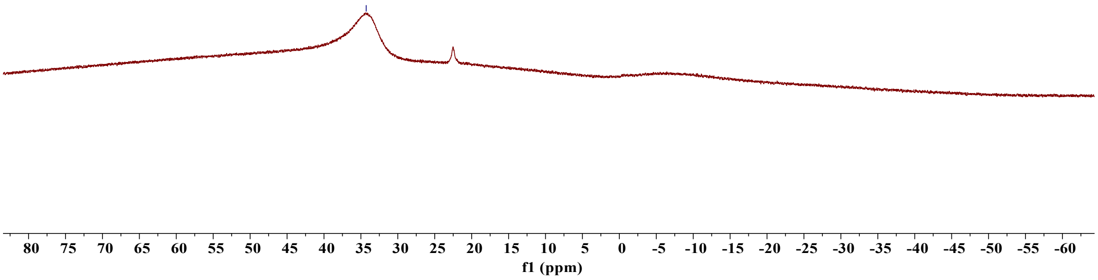

(S)-2-(2-benzyl-1,1-difluoro-5-phenylpent-1-en-3-yl)-4,4,5,5-tetramethyl-1,3,2-dioxaborolane (106)

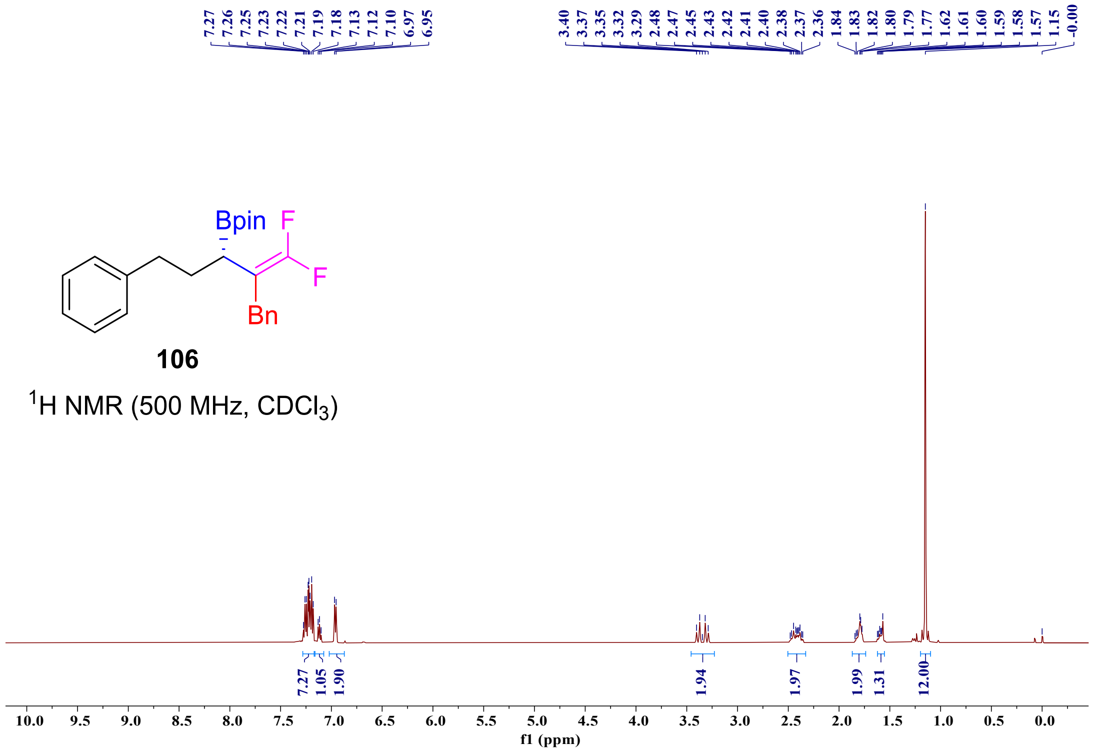

(S)-2-(2-benzyl-1,1-difluoro-5-phenylpent-1-en-3-yl)-4,4,5,5-tetramethyl-1,3,2-dioxaborolane(106)

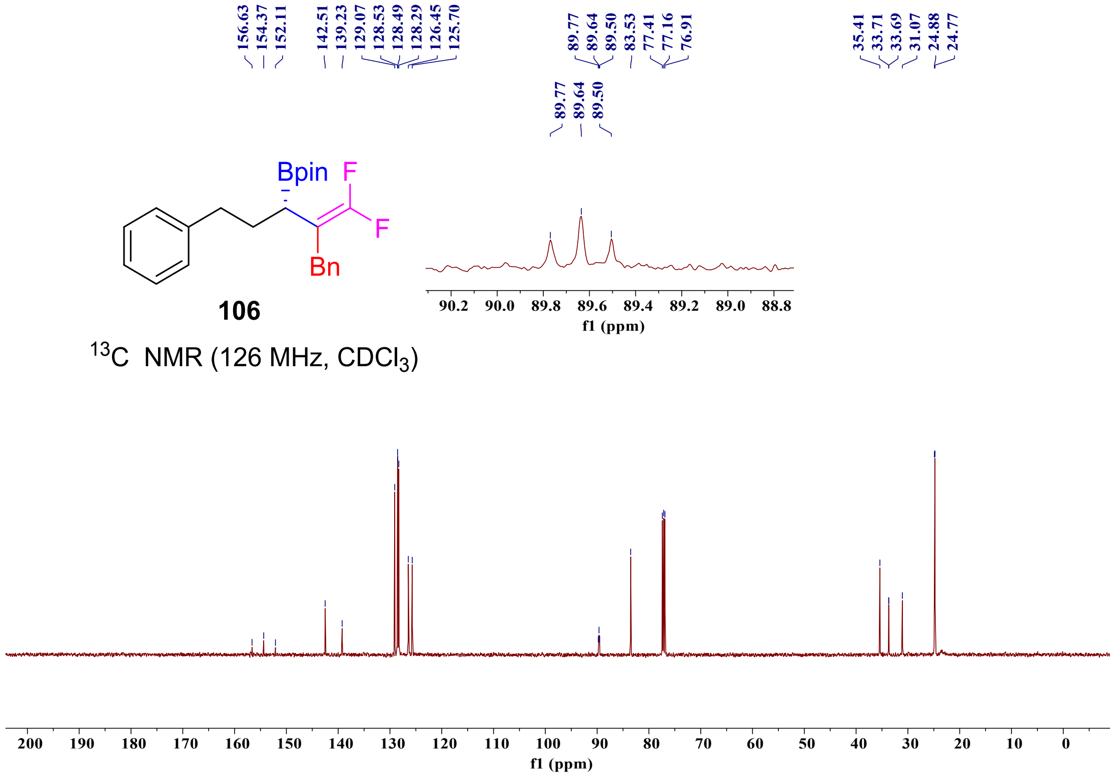

(S)-2-(2-benzyl-1,1-difluoro-5-phenylpent-1-en-3-yl)-4,4,5,5-tetramethyl-1,3,2-dioxaborolane (106)

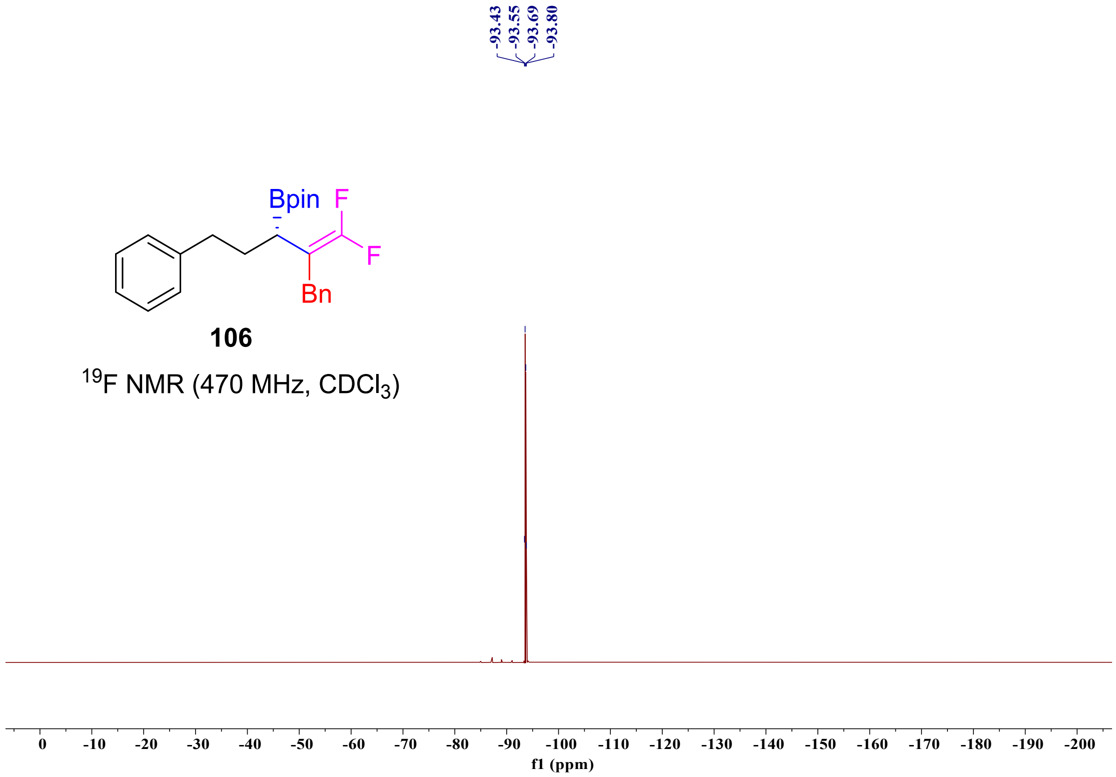

(S)-2-(2-benzyl-1,1-difluoro-5-phenylpent-1-en-3-yl)-4,4,5,5-tetramethyl-1,3,2-dioxaborolane (106)

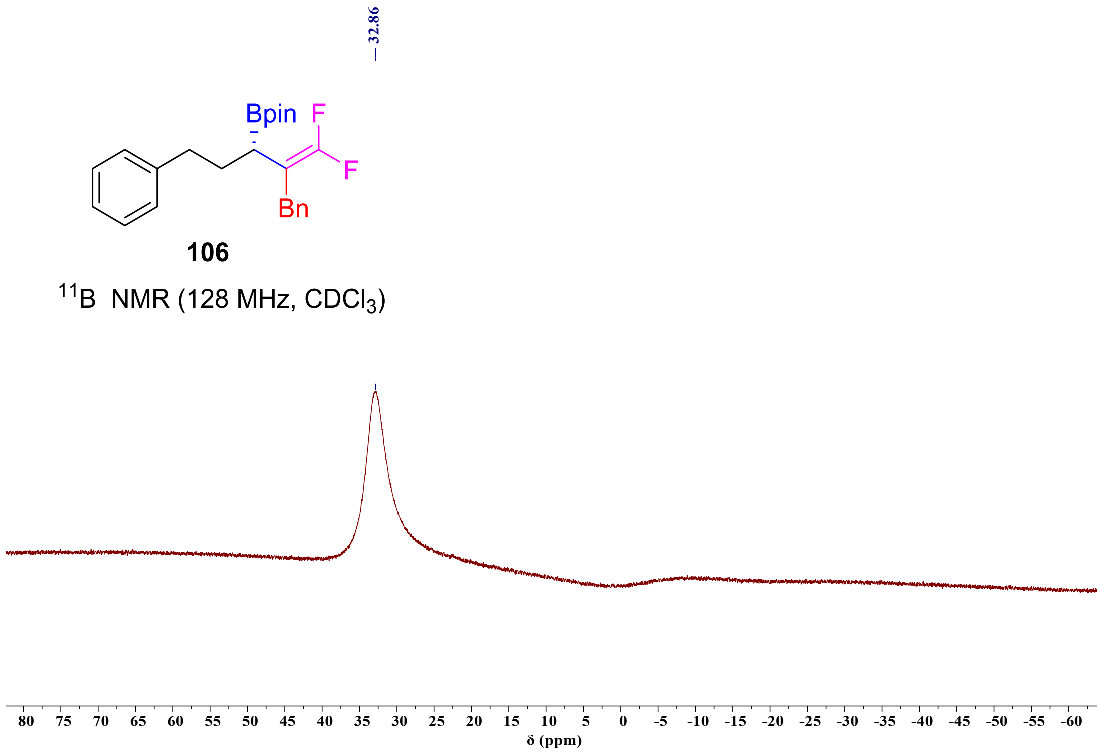

(S)-4-(1,1-difluoro-5-phenyl-3-(4,4,5,5-tetramethyl-1,3,2-dioxaborolan-2-yl)pent-1-en-2-yl)benzoate (107)

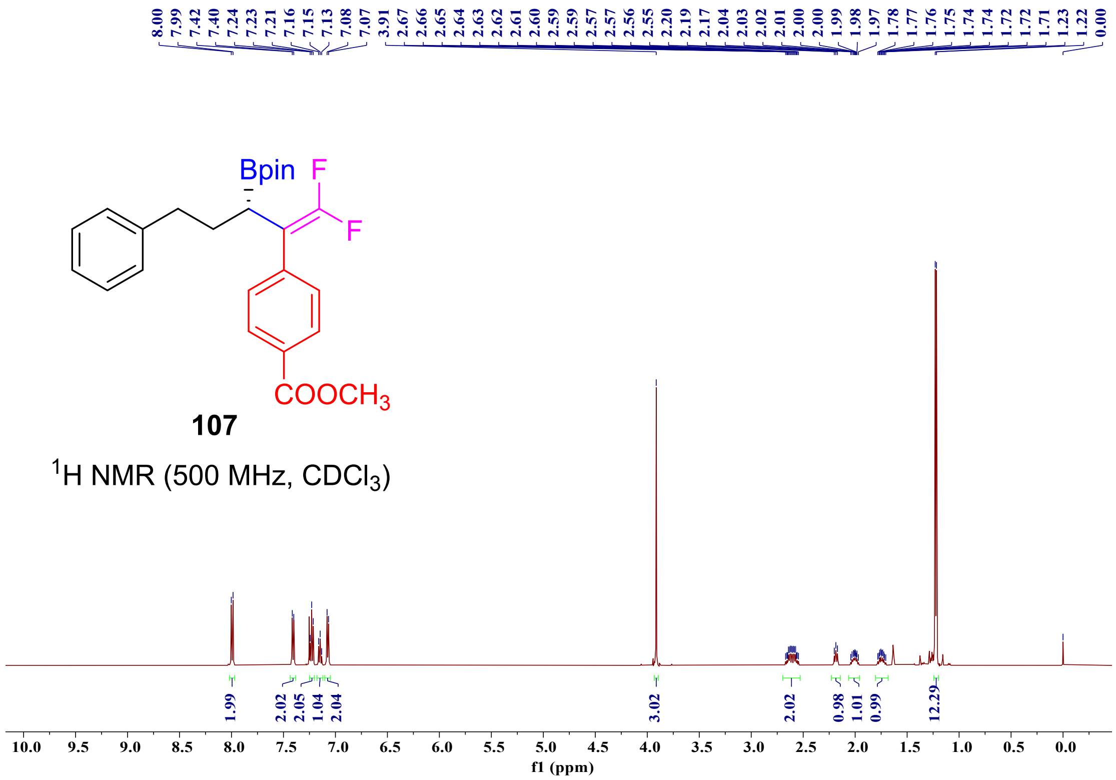

(S)-4-(1,1-difluoro-5-phenyl-3-(4,4,5,5-tetramethyl-1,3,2-dioxaborolan-2-yl)pent-1-en-2-yl)benzoate (107)

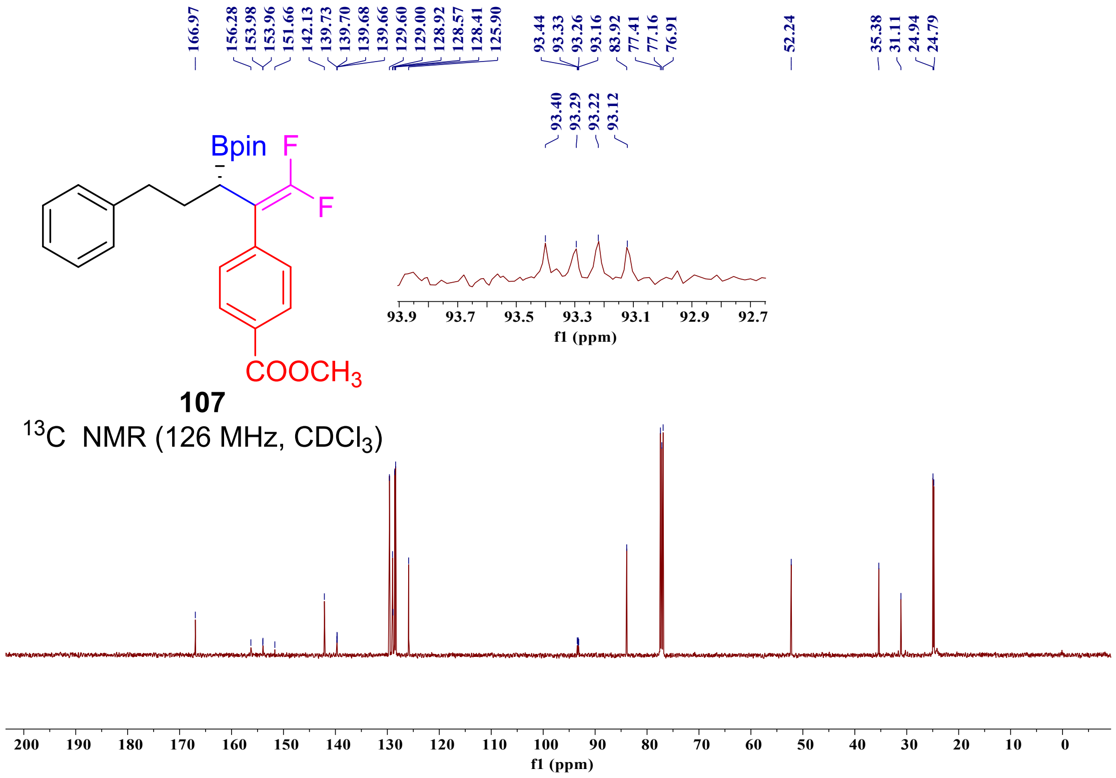

(S)-4-(1,1-difluoro-5-phenyl-3-(4,4,5,5-tetramethyl-1,3,2-dioxaborolan-2-yl)pent-1-en-2-yl) benzoate (107)

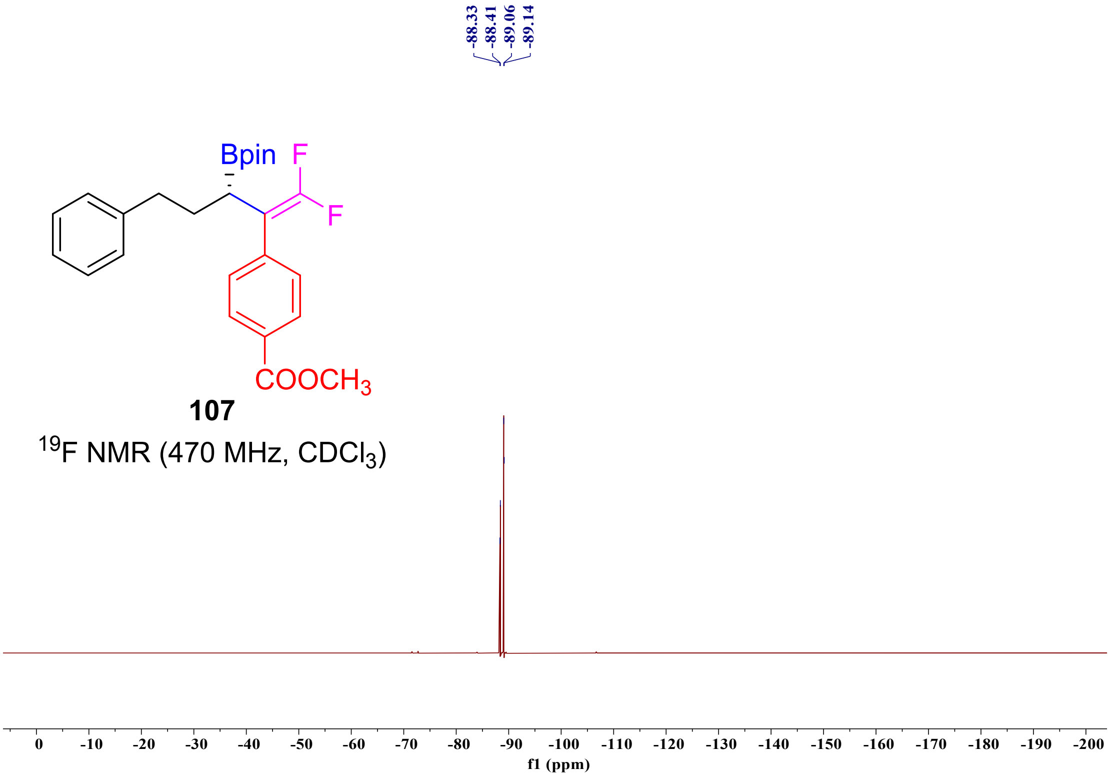

(S)-4-(1,1-difluoro-5-phenyl-3-(4,4,5,5-tetramethyl-1,3,2-dioxaborolan-2-yl)pent-1-en-2-yl)benzoate (107)

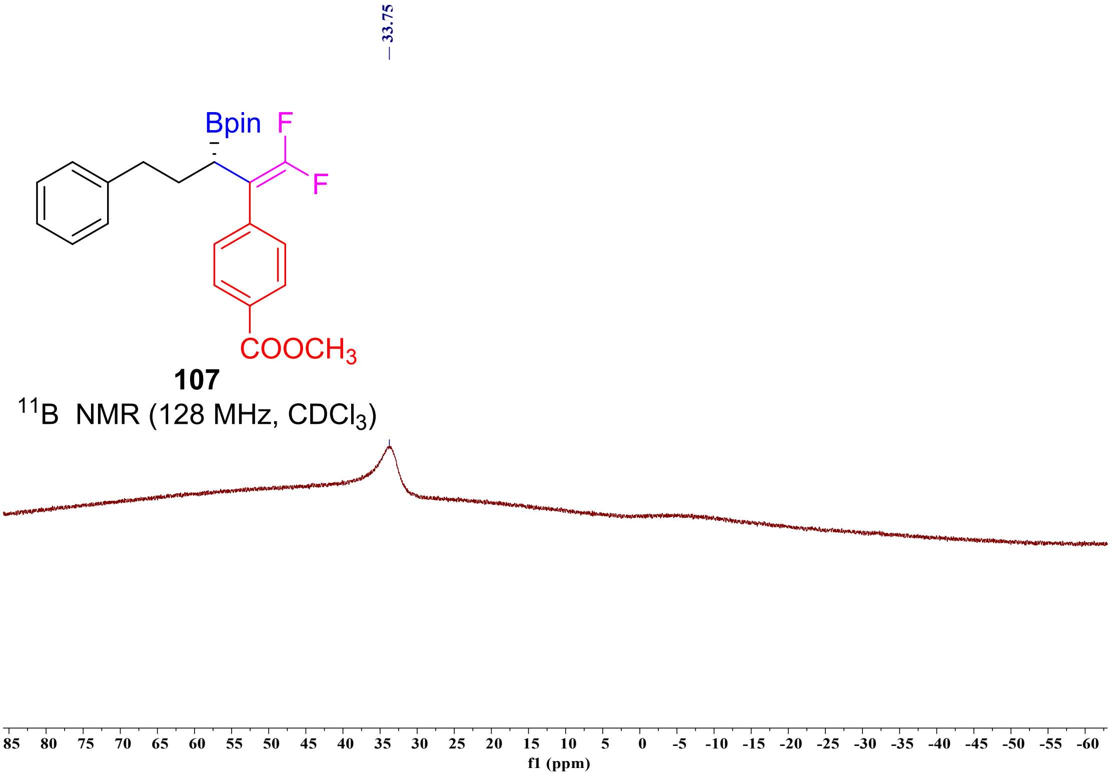

methyl(S)-4-(1,1-difluoro-5-phenyl-3-(trifluoro-l4-boraneyl)pent-1-en-2-yl)benzoate,potassium salt (108)

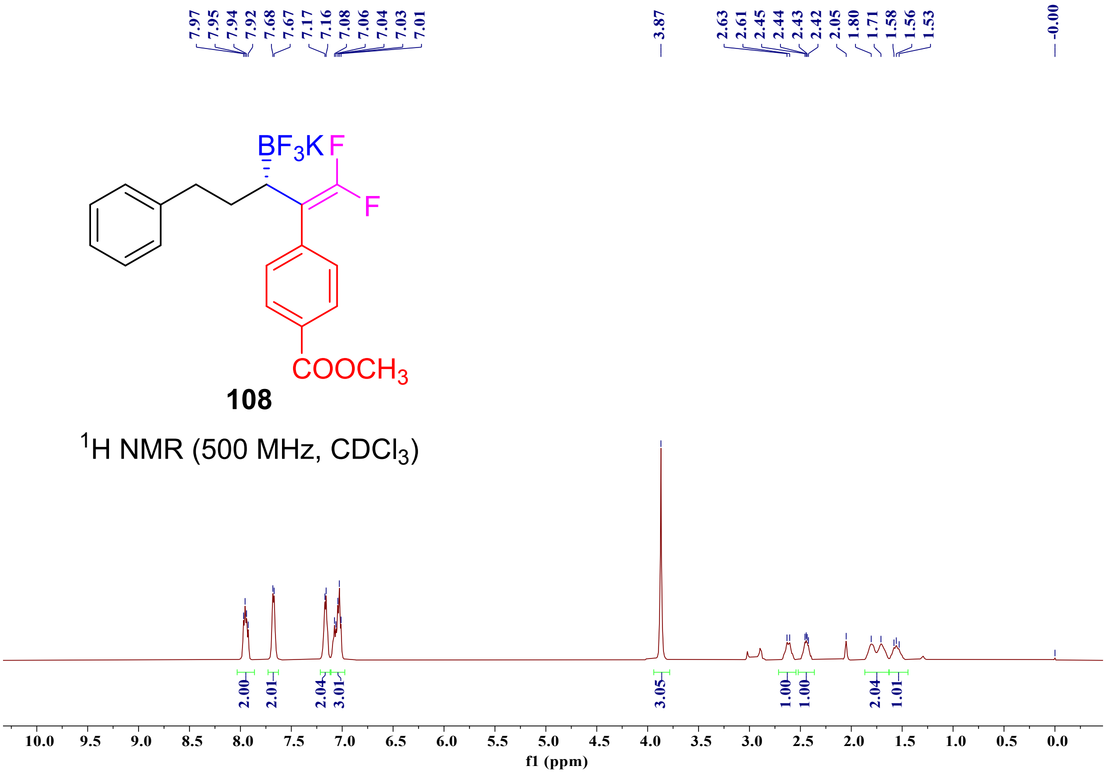

methyl(S)-4-(1,1-difluoro-5-phenyl-3-(trifluoro-l4-boraneyl)pent-1-en-2-yl)benzoate,potassium salt (108)

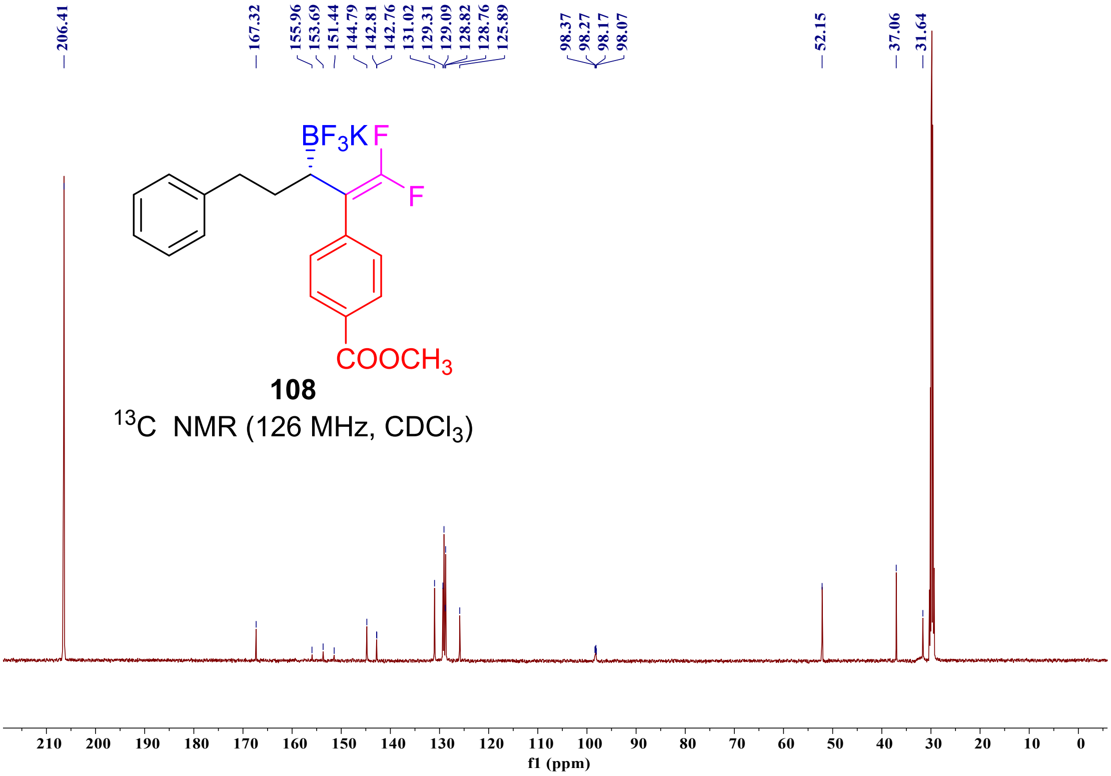

methyl(S)-4-(1,1-difluoro-5-phenyl-3-(trifluoro-l4-boraneyl)pent-1-en-2-yl)benzoate,potassium salt (108)

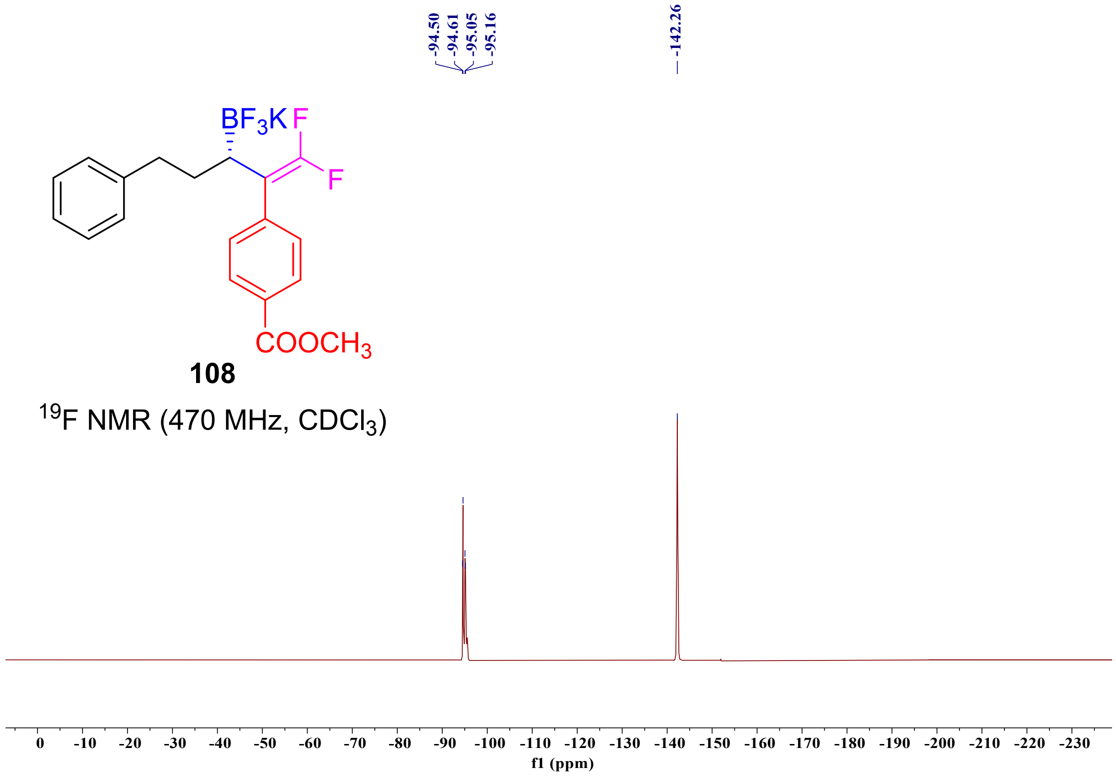

methyl(S)-4-(1,1-difluoro-5-phenyl-3-(trifluoro-l4-boraneyl)pent-1-en-2-yl)benzoate,potassium salt (108)

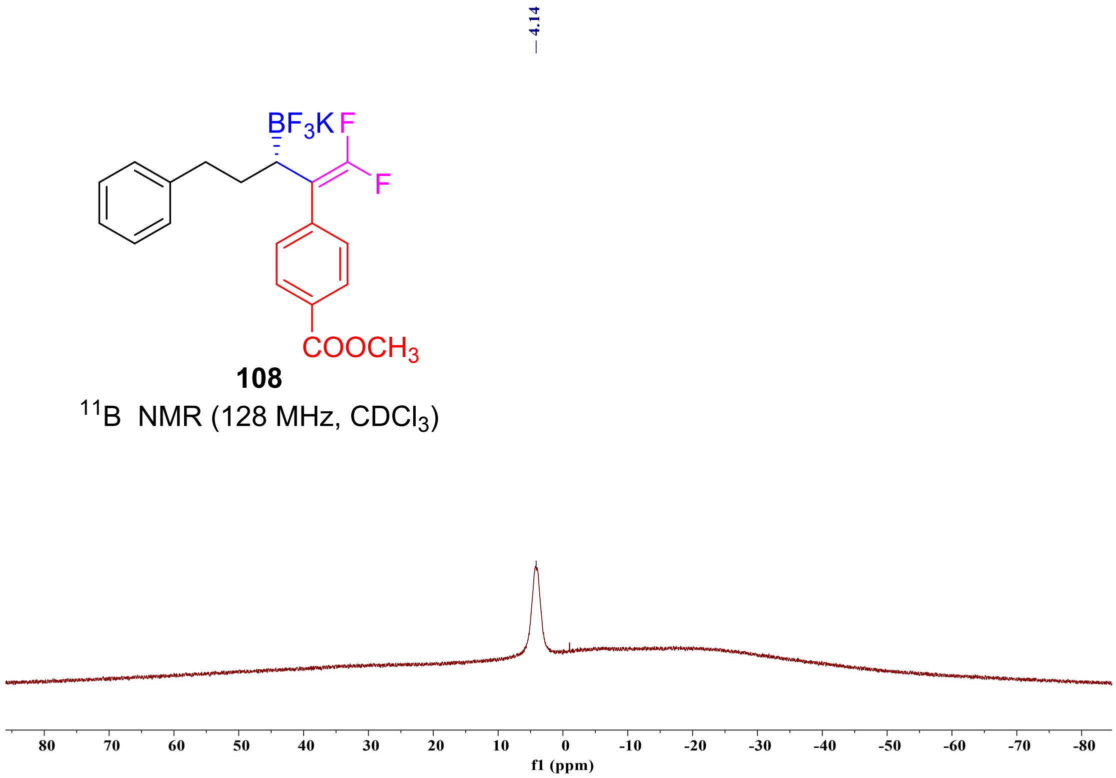

Methyl(S)-4-(1,1-difluoro-5-phenyl-3-((4,4,5,5-tetramethyl-1,3,2-dioxaborolan-2-yl)methyl)pent-1-en-2-yl)benzoate (109)

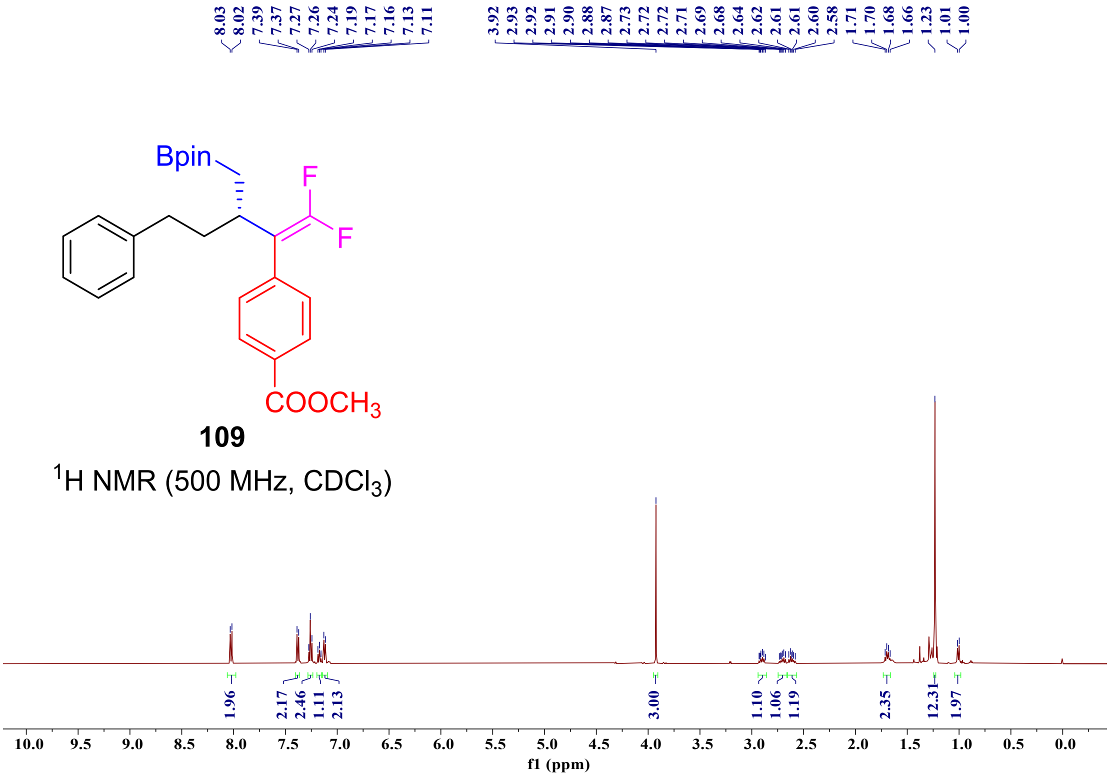

Methyl(S)-4-(1,1-difluoro-5-phenyl-3-((4,4,5,5-tetramethyl-1,3,2-dioxaborolan-2-yl)methyl)pent-1-en-2-yl)benzoate (109)

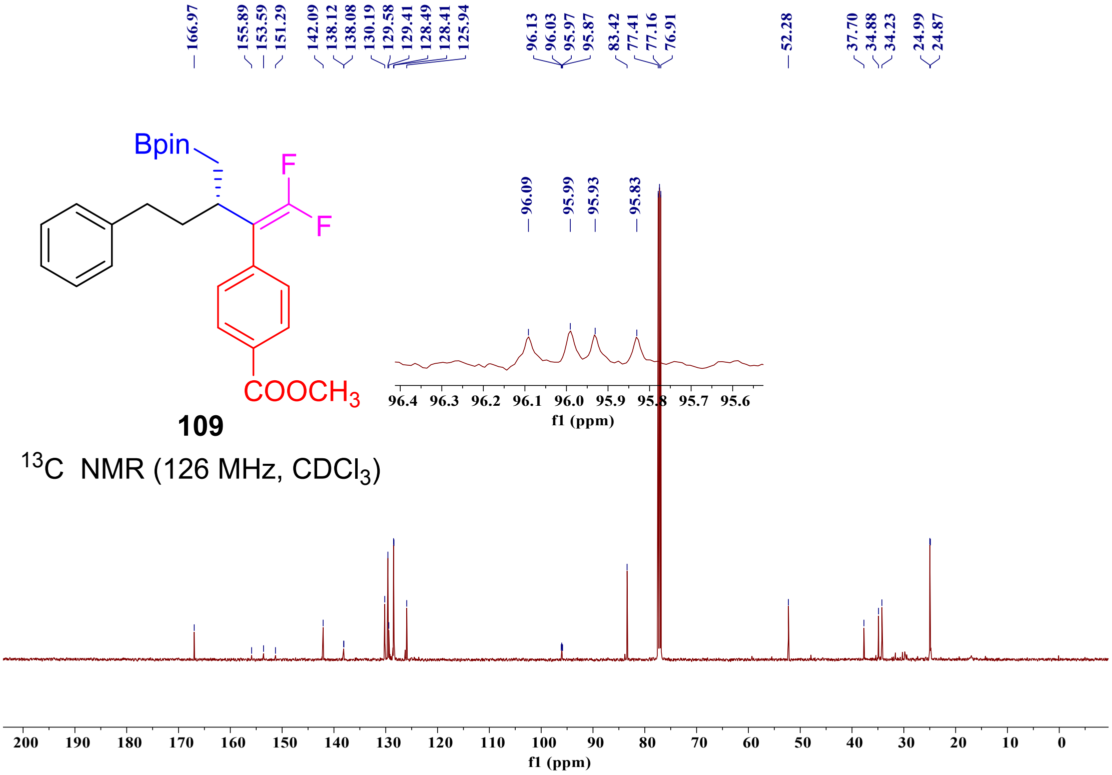

Methyl(S)-4-(1,1-difluoro-5-phenyl-3-((4,4,5,5-tetramethyl-1,3,2-dioxaborolan-2-yl)methyl)pent-1-en-2-yl)benzoate (109)

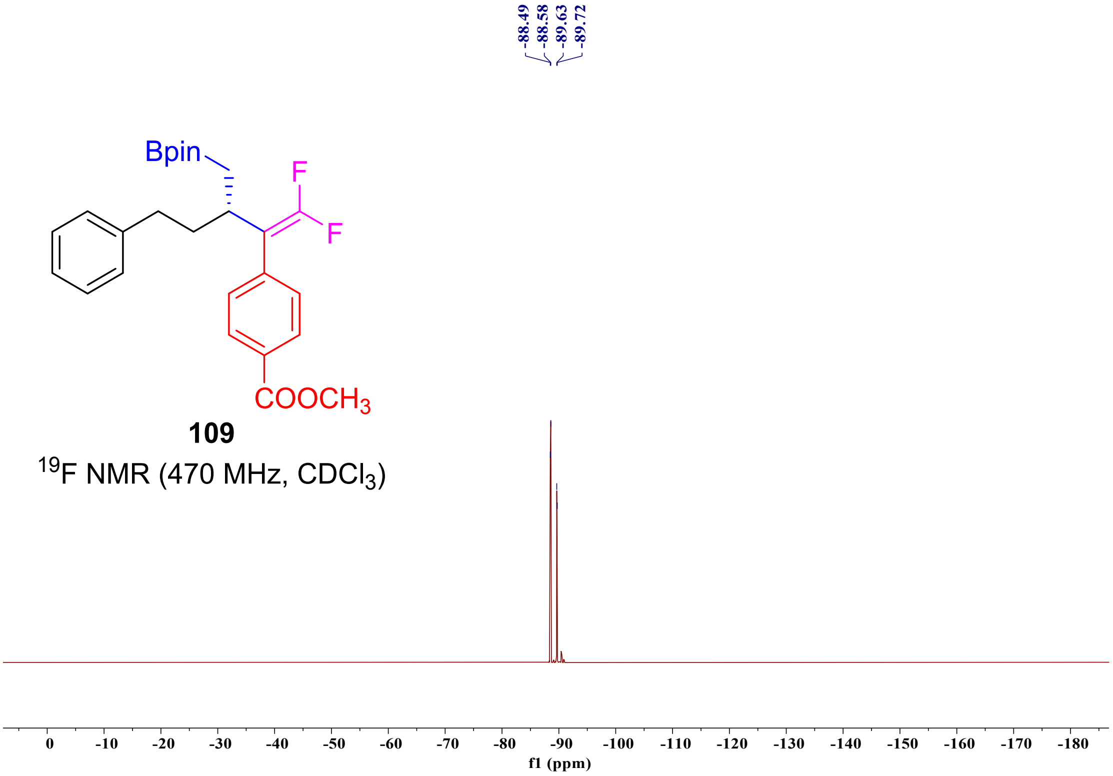

Methyl(S)-4-(1,1-difluoro-5-phenyl-3-((4,4,5,5-tetramethyl-1,3,2-dioxaborolan-2-yl)methyl)pent-1-en-2-yl)benzoate (109)

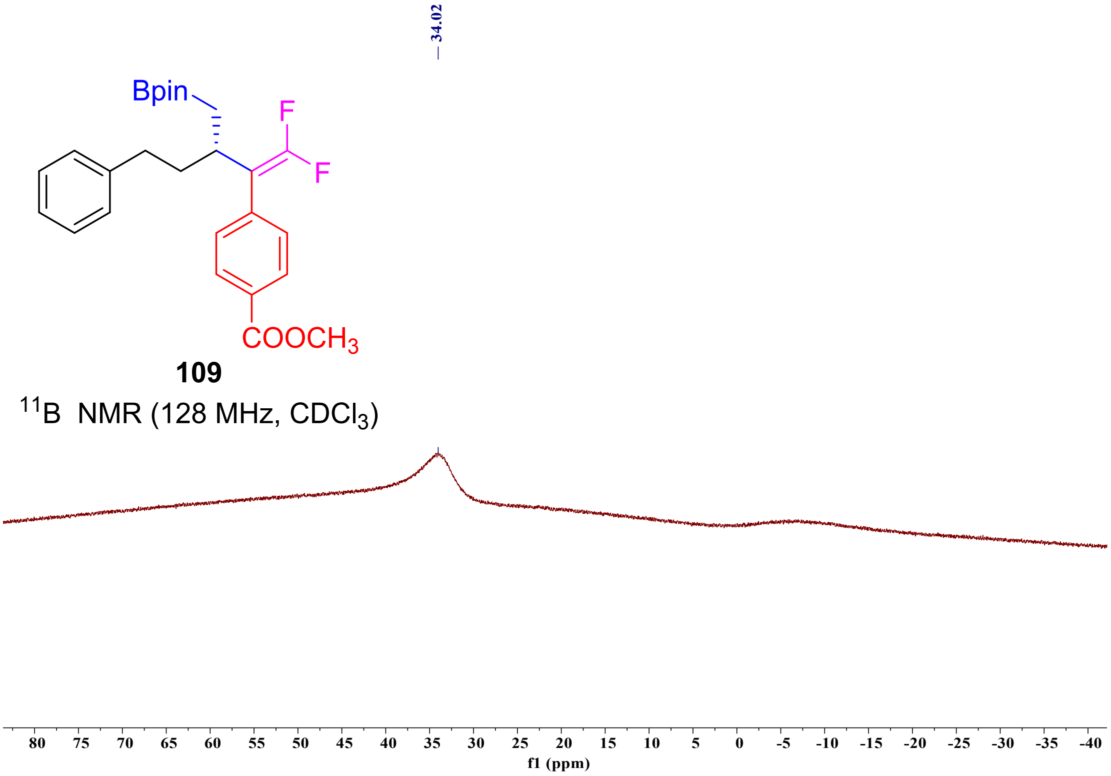

methyl4-((3S)-1,1-difluoro-5-phenyl-3-(4,4,5,5-tetramethyl-1,3,2-dioxaborolan-2-yl)pentan-2-yl)benzoate(110)

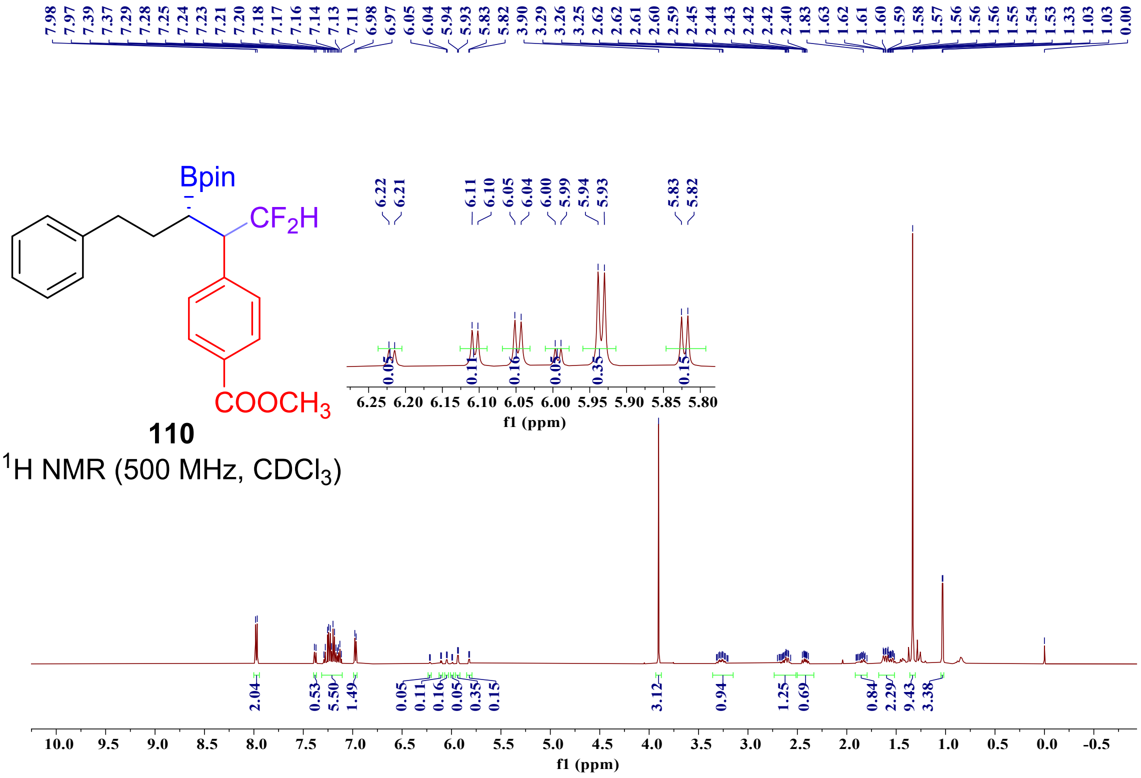

methyl4-((3S)-1,1-difluoro-5-phenyl-3-(4,4,5,5-tetramethyl-1,3,2-dioxaborolan-2-yl)pentan-2-yl)benzoate(110)

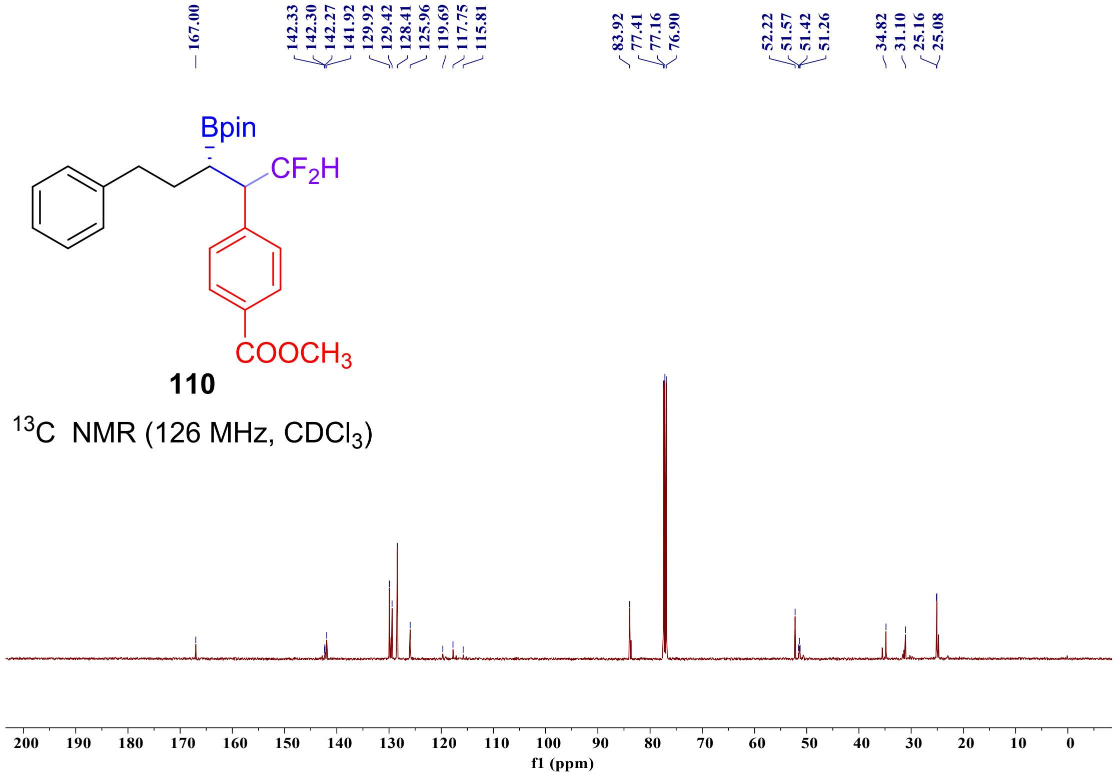

methyl4-((3S)-1,1-difluoro-5-phenyl-3-(4,4,5,5-tetramethyl-1,3,2-dioxaborolan-2-yl)pentan-2-yl)benzoate(110)

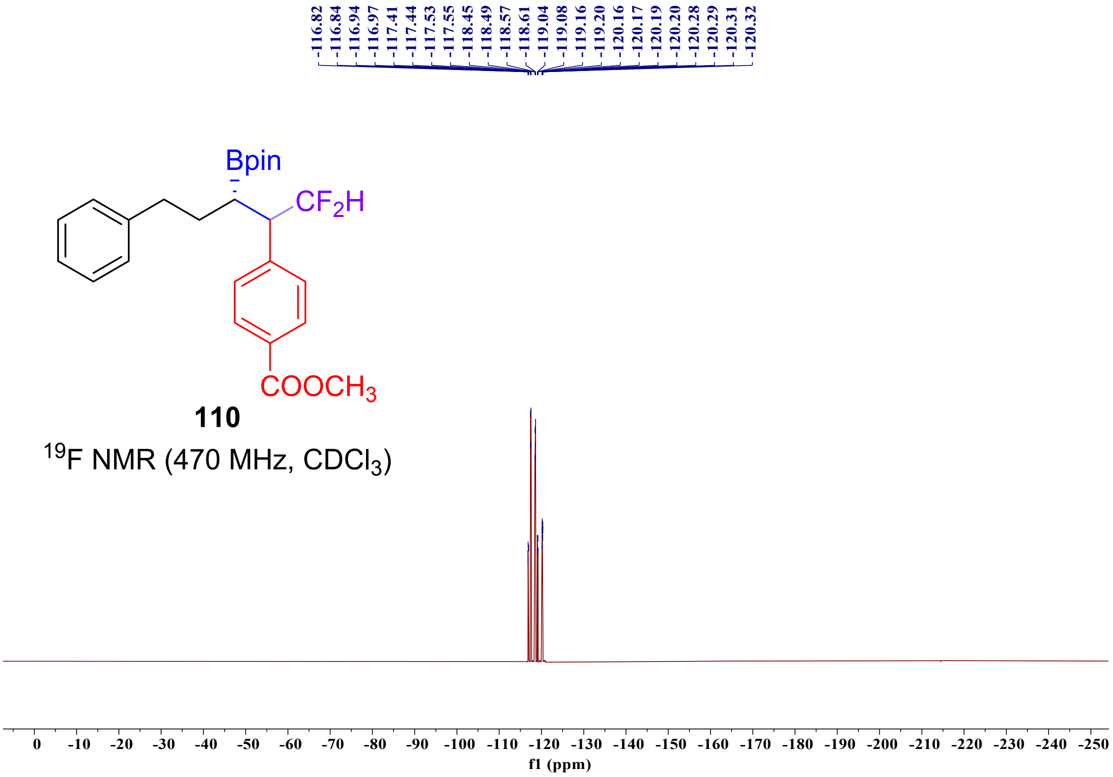

methyl4-((3S)-1,1-difluoro-5-phenyl-3-(4,4,5,5-tetramethyl-1,3,2-dioxaborolan-2-yl)pentan-2-yl)benzoate(110)

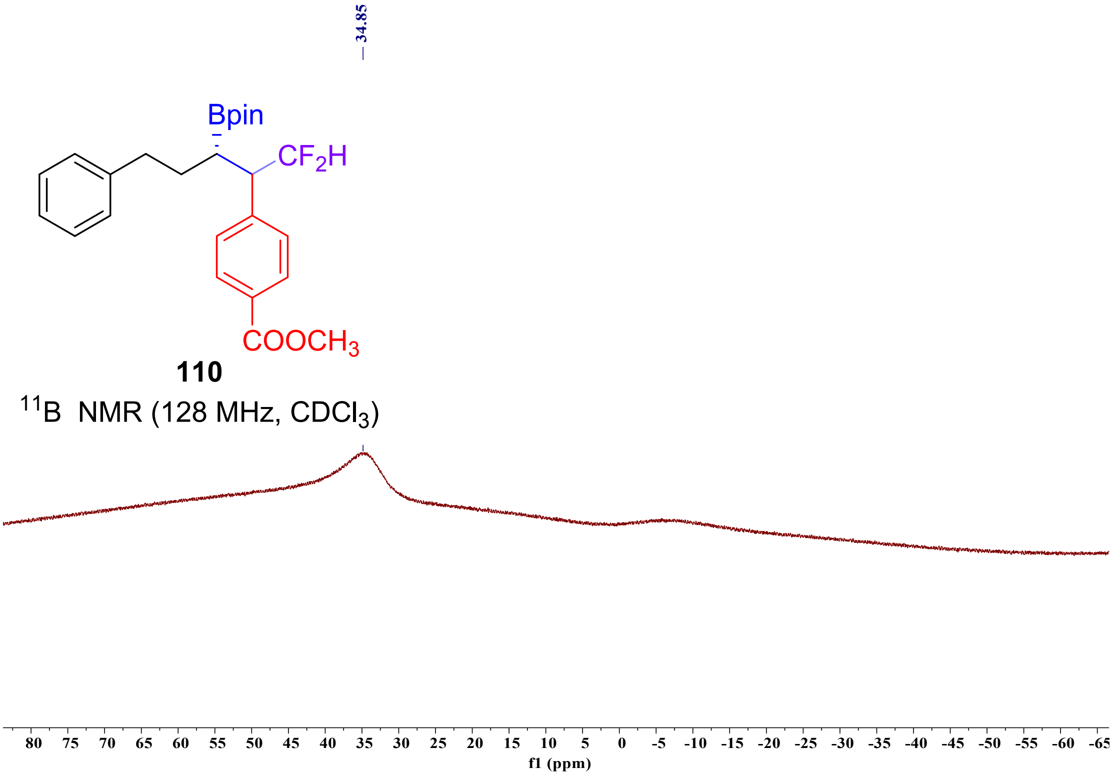

methyl (S)-4-(1,1-difluoro-3-(furan-2-yl)-5-phenylpent-1-en-2-yl)benzoate (111)

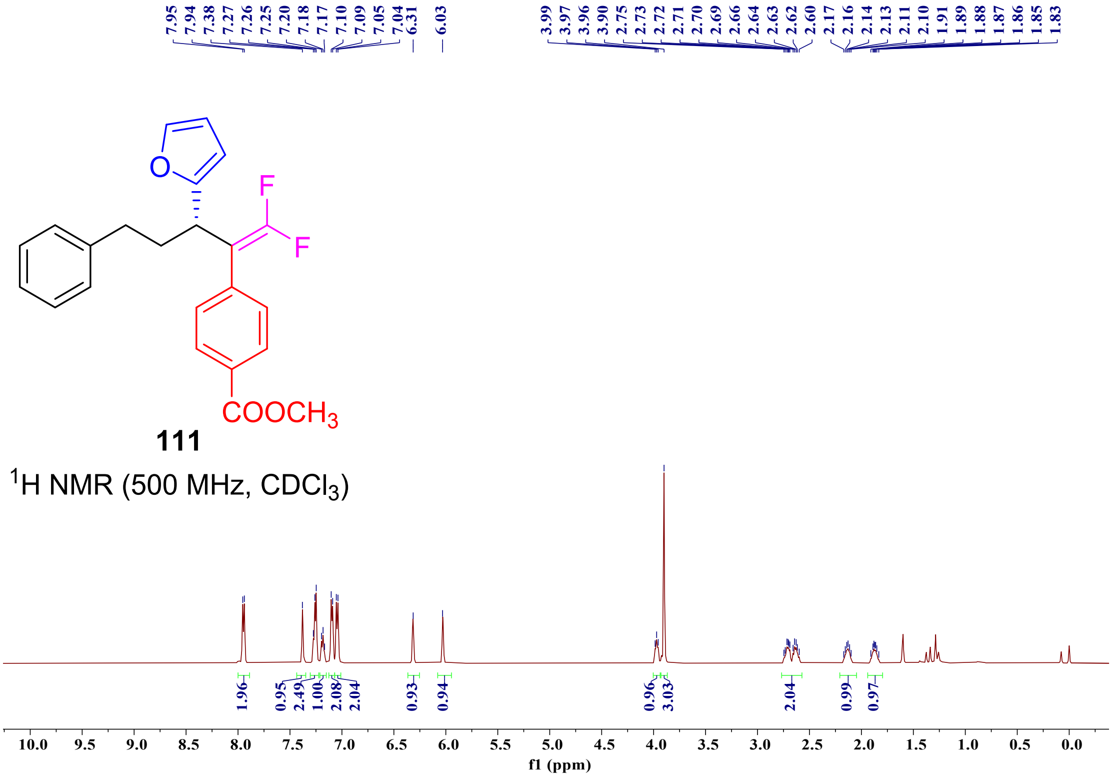

methyl (S)-4-(1,1-difluoro-3-(furan-2-yl)-5-phenylpent-1-en-2-yl)benzoate (111)

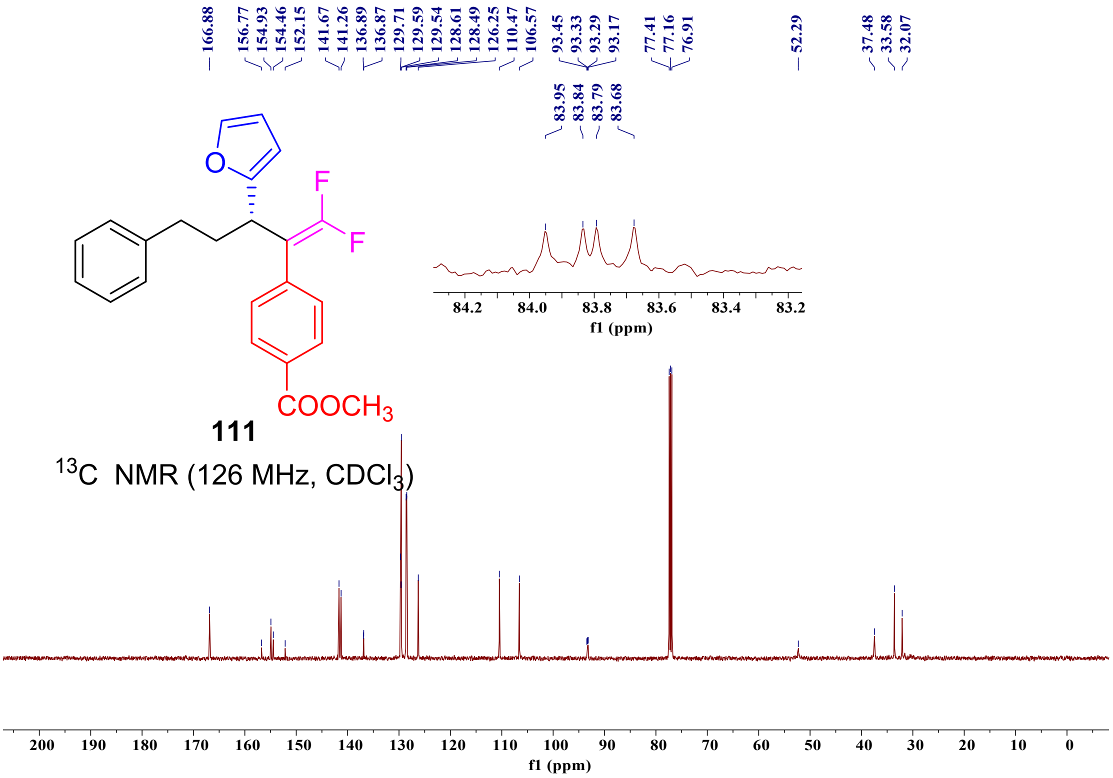

methyl (S)-4-(1,1-difluoro-3-(furan-2-yl)-5-phenylpent-1-en-2-yl)benzoate (111)

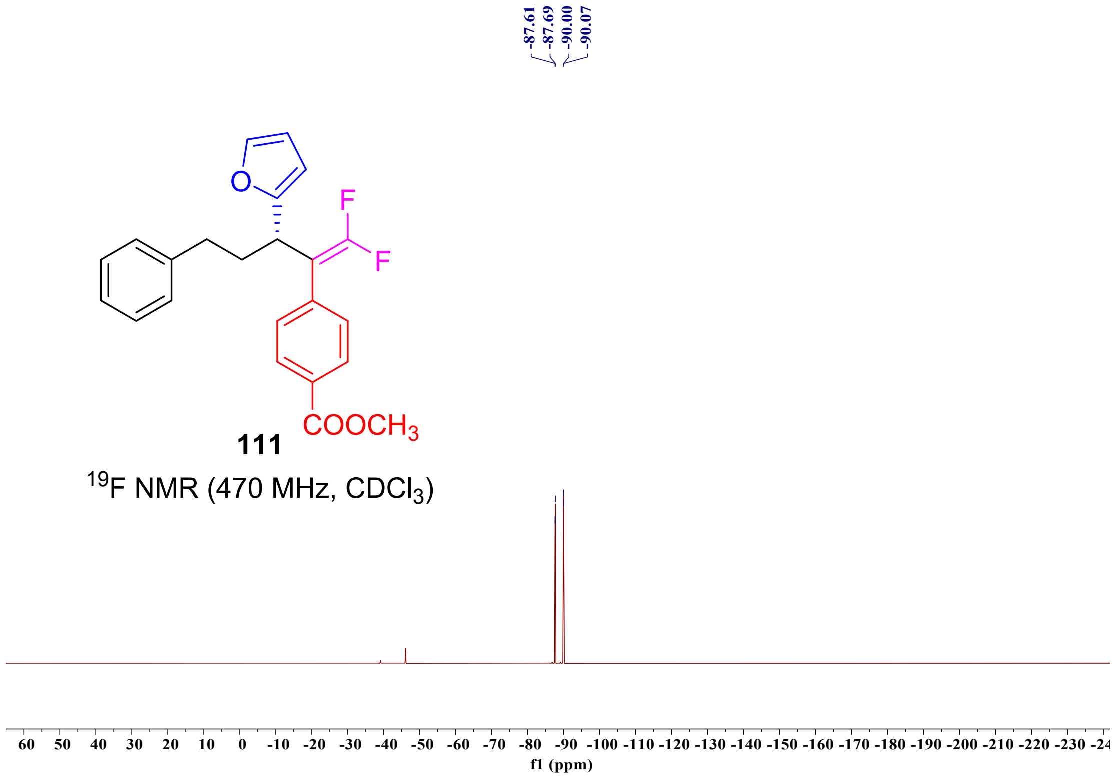

methyl (S)-4-(1,1-difluoro-3-phenethylpenta-1,4-dien-2-yl)benzoate (112)

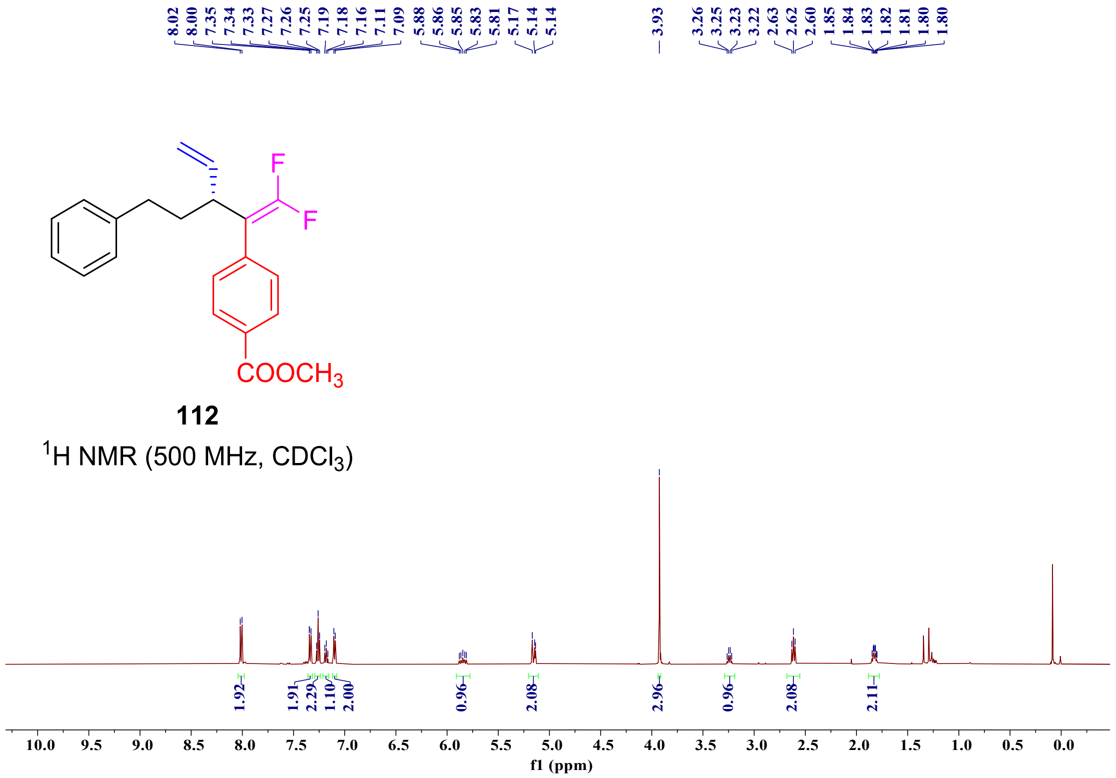

methyl (S)-4-(1,1-difluoro-3-phenethylpenta-1,4-dien-2-yl)benzoate (112)

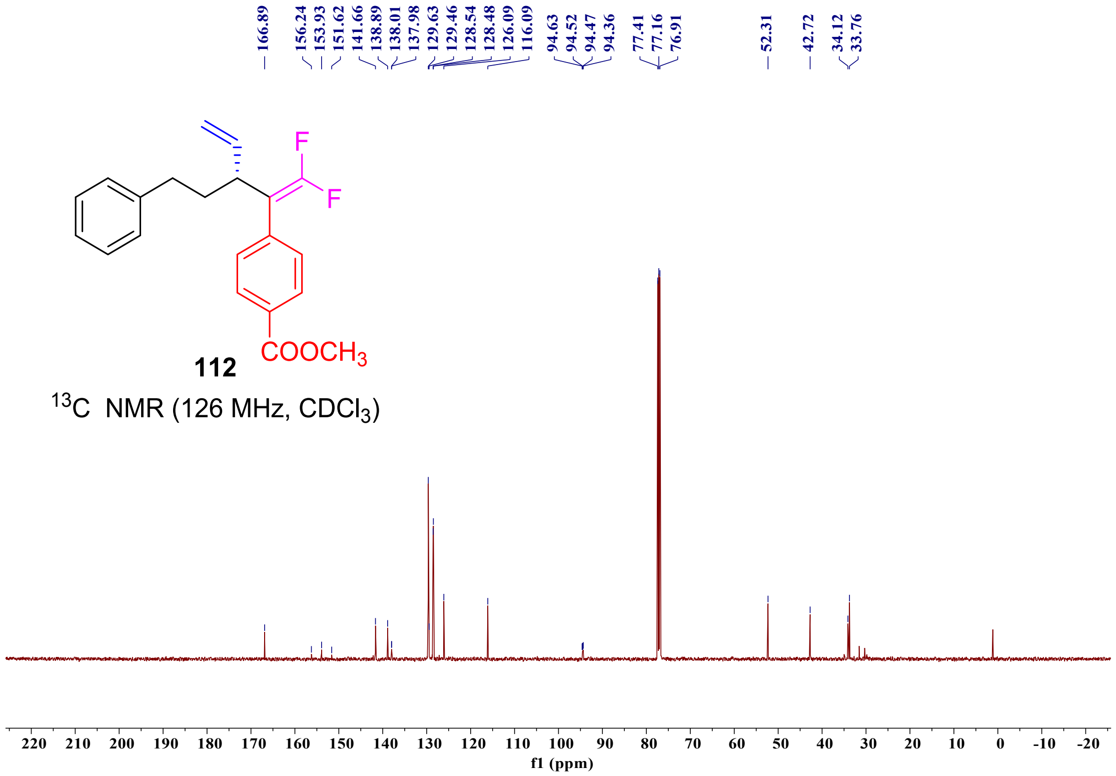

methyl (S)-4-(1,1-difluoro-3-phenethylpenta-1,4-dien-2-yl)benzoate (112)

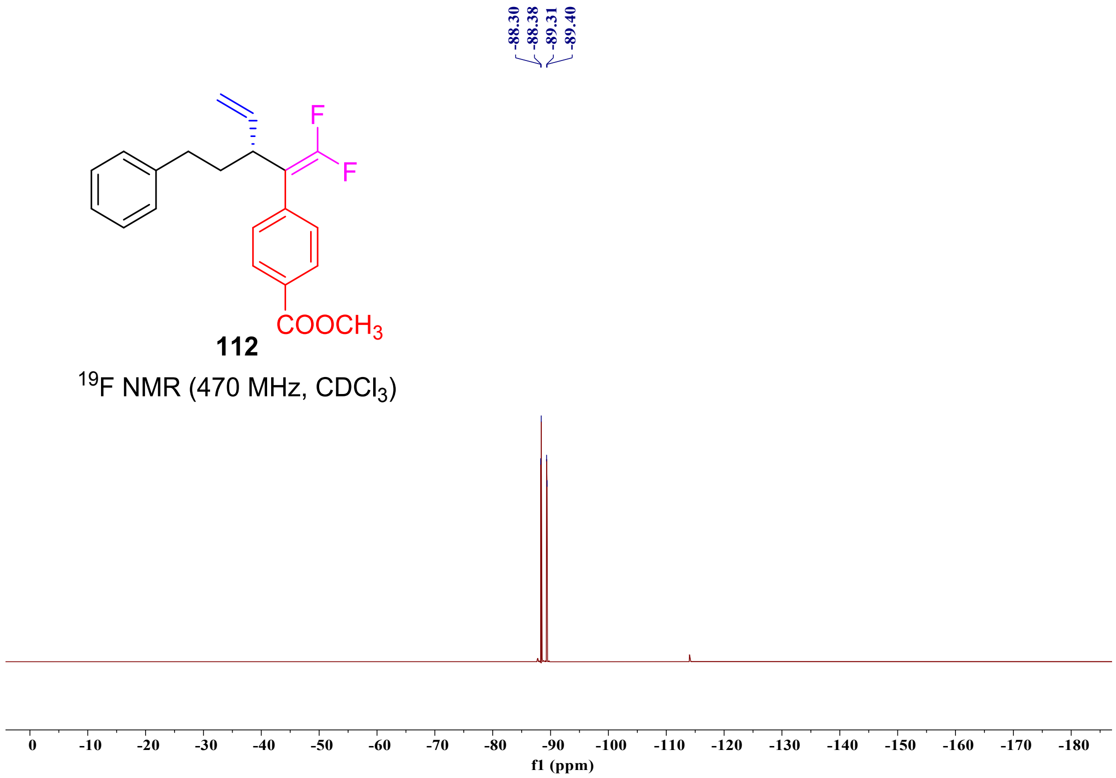

(E)-4,4,5,5-tetramethyl-2-(1,1,1-trifluoro-5-phenylpent-2-en-2-yl)-1,3,2-dioxaborolane (113)

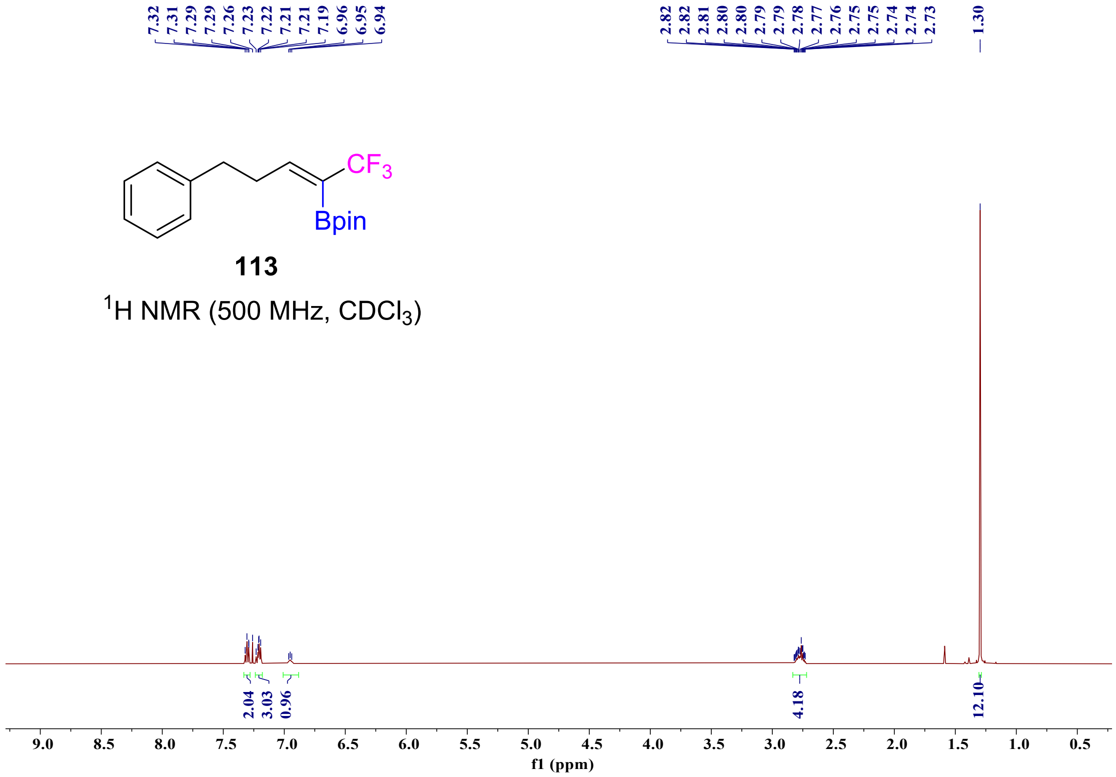

(E)-4,4,5,5-tetramethyl-2-(1,1,1-trifluoro-5-phenylpent-2-en-2-yl)-1,3,2-dioxaborolane (113)

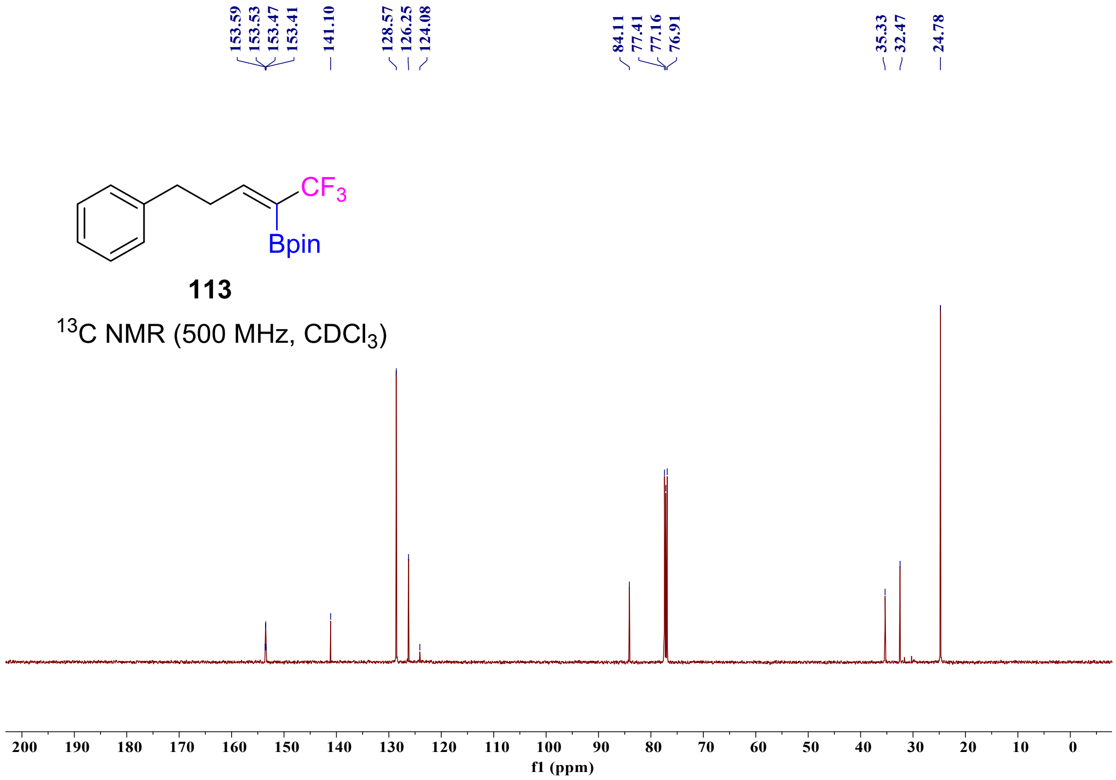

(E)-4,4,5,5-tetramethyl-2-(1,1,1-trifluoro-5-phenylpent-2-en-2-yl)-1,3,2-dioxaborolane (113)

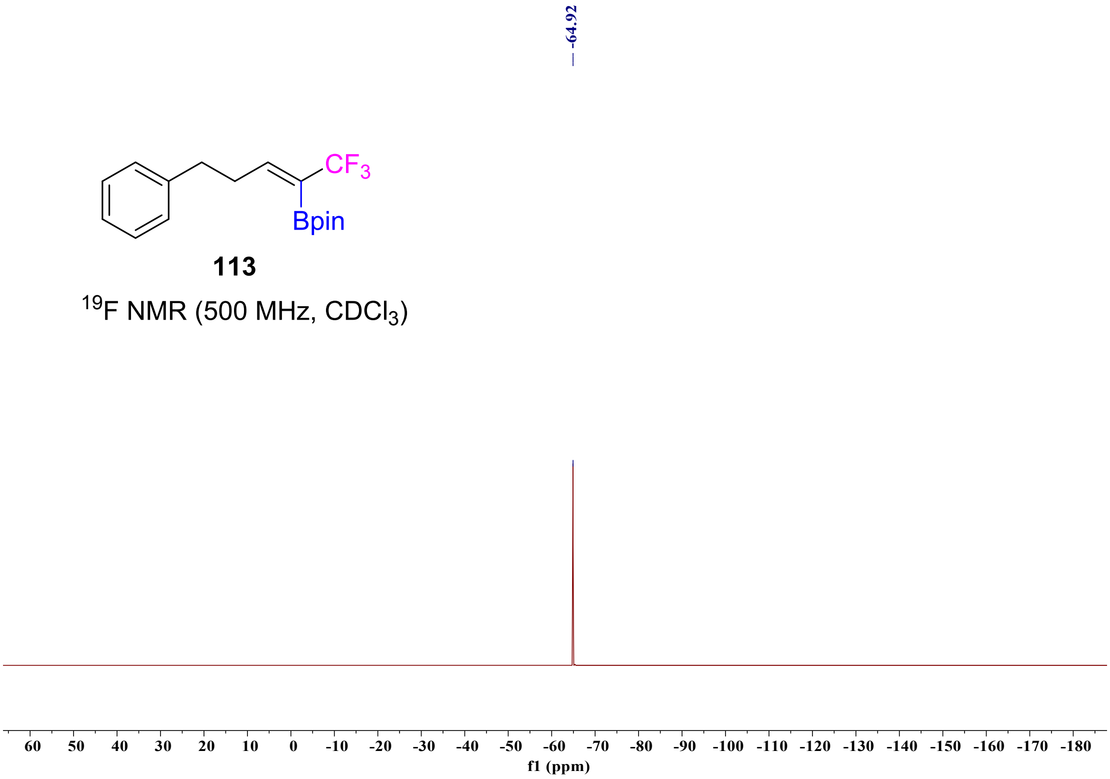

(E)-4,4,5,5-tetramethyl-2-(1,1,1-trifluoro-5-phenylpent-2-en-2-yl)-1,3,2-dioxaborolane (113)

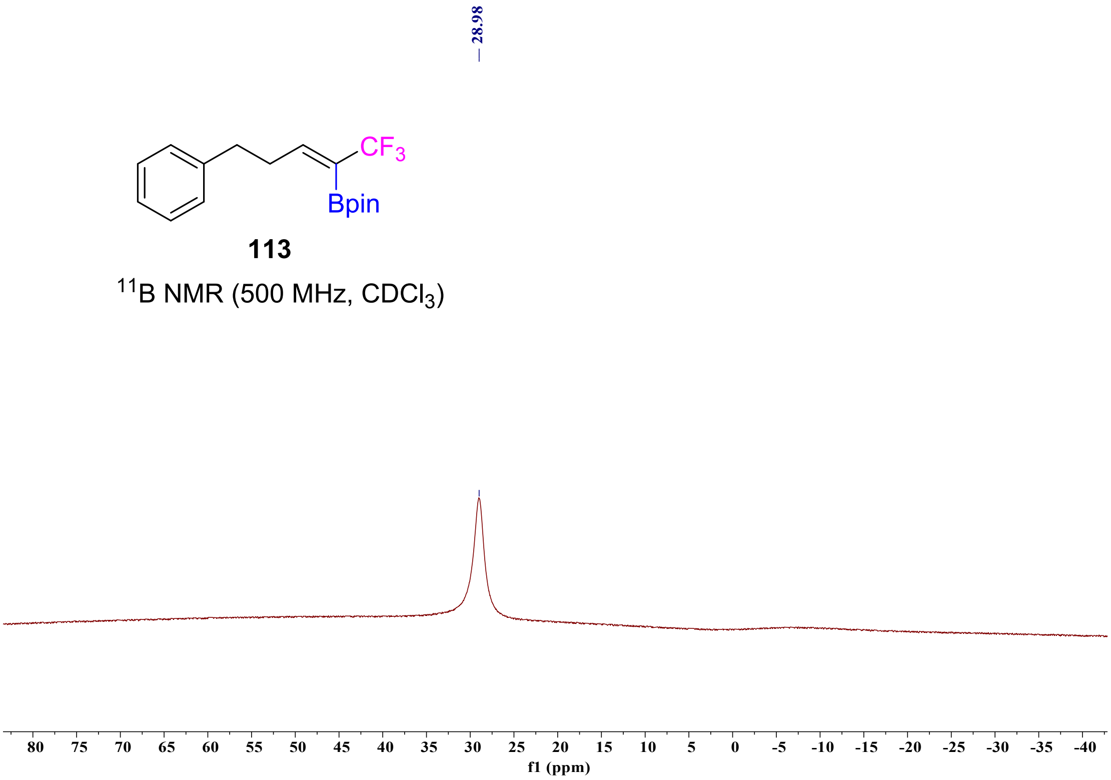

11. Reference:

1. L, Wang.; J. M. Lear.; S. M. Rafferty.; S. C. Fosu.; D. A. Nagib., Ketyl radical reactivity via atom transfer catalysis. *Science*. **2018**, *362*, 225–229

2. M, Fujita.; T, Hiyama.; and K, Kondo., Pracital and stereocontrolled syntheses of roth (lR,3S)- and (lR,3R)- 3-(2-Chloro-3,3,3-trifluoro-I-Propenyl)-2,2-dimethykclopropaecarboxylates. *Tetra. Lett*. **1986**, *19*, 2139-2142.

3. Zhao, Y.; Zhou, Y.; Liu, J.; Yang, D.; Tao, L.; Liu, Y.; Dong, X.; Liu, J.; Qu, J., Synthesis of (Z)-alpha-Trifluoromethyl Alkenyl Triflate: A Scaffold for Diverse Trifluoromethylated Species. *J. Org. Chem*. **2016**, *81*, 4797-4806.

4. Jayaram, V.; Sridhar, T.; Sharma, G. V.; Berree, F.; Carboni, B., Synthesis of 1-Amino-1H-Indenes via a Sequential Suzuki-Miyaura Coupling/Petasis Condensation Sequence. *J. Org. Chem*. *2017*, *82*, 1803-1811.

5. Gao, P.; Yuan, C.; Zhao, Y.; Shi, Z., Copper-Catalyzed Asymmetric Defluoroborylation of 1-(Trifluoromethyl)Alkenes. *Chem*. **2018**, *4*, 2201-2211.

6. Tao, L.; Guo, X.; Li, J.; Li, R.; Lin, Z.; Zhao, W., Rhodium-Catalyzed Deoxygenation and Borylation of Ketones: A Combined Experimental and Theoretical Investigation. *J. Am. Chem. Soc*. **2020**, *142*, 18118-18127.

7. Wang, S.; Zhang, J.; Kong, L.; Tan, Z.; Bai, Y.; Zhu, G., Palladium-Catalyzed anti-Selective Fluoroalkylboration of Internal and Terminal Alkynes. *Org. Lett*. **2018**, *20*, 5631-5635.

8. Lee, H. J.; Yonekura, Y.; Kim, N.; Yoshida, J. I.; Kim, H., Regioselective Synthesis of alpha-Functional Stilbenes via Precise Control of Rapid cis-trans Isomerization in Flow. *Org. Lett*. **2021**, *23*, 2904-2910.

9. Cao, Z.-C.; Luo, F.-X.; Shi, W.-J.; Shi, Z.-J., Direct borylation of benzyl alcohol and its analogues in the absence of bases. *Org. Chem. Front*. **2015**, *2*, 1505-1510.

10. Bonet, A.; Odachowski, M.; Leonori, D.; Essafi, S.; Aggarwal, V. K., Enantiospecific sp(2)-sp(3) coupling of secondary and tertiary boronic esters. *Nat. Chem*. **2014**, *6*, 584-9.

11. Wang, D.; Xu, T., A Pivotal Role of Chloride Ion on Nickel-Catalyzed Enantioselective Reductive Cross-Coupling to Perfluoroalkylated Boronate Esters. *ACS Catal*. **2021**, *11*, 12469-12475
